# Supplementary material for: Development and implementation of a scalable and versatile test for COVID-19 diagnostics in rural communities
Source: Nat Commun. 2021 Jul 20;12:4400. doi: 10.1038/s41467-021-24552-4 (PMC8292415; doi:10.1038/s41467-021-24552-4)
Supplement: Supplementary file 1 — Supplementary Information [file 41467_2021_24552_MOESM1_ESM.pdf]

Supplementary Information

Development and Implementation of a scalable and versatile test for COVID-19 diagnostics in rural communities

Ceci A.[1,#], Muñoz-Ballester C.[2,#], Tegge A.[3], Brown K.L.[4], Umans R.A.[2], Michel F.M.[5], Patel D.[2], Tewari B.[2], Martin J.[2,6], Alcoreza O.[2,4,6], Maynard T.[7], Martinez-Martinez D.[8,9], Bordwine P.[10], Bissell N.[11], Friedlander M.J.[7], Sontheimer H.[2], Finkielstein C.V.[1,12,13,*]

[1] Molecular Diagnostics Laboratory, Fralin Biomedical Research Institute, Virginia Tech, Roanoke, VA 24016, United States

[2] Center for Glial Biology in Health, Disease, and Cancer, Fralin Biomedical Research Institute at VTC, Roanoke, Virginia 24016, United States

[3] Department of Statistics, Virginia Tech, Blacksburg, VA 24060, United States

[4] Virginia Tech Carilion School of Medicine, Roanoke, VA 24016, United States

[5] Department of Geosciences, Virginia Tech, Blacksburg, VA 24060, United States

[6] Translational Biology, Medicine, and Health Graduate Program, Virginia Tech, Roanoke, VA 24016, United States

[7] Fralin Biomedical Research Institute at VTC, Roanoke, Virginia 24016, United States

[8] MRC London Institute of Medical Sciences, London, W12 ONN, UK

[9] Institute of Clinical Sciences, Imperial College London, London, W12 ONN, UK

[10] Division of Surveillance and Investigation, Office of Epidemiology, Virginia Department of Health

[11] New River Valley Health District, Virginia Department of Health

[12] Integrated Cellular Responses Laboratory, Fralin Biomedical Research Institute at VTC, Roanoke, Virginia 24016, United States,

[13] Department of Biological Sciences, Virginia Tech, Blacksburg, VA 24060

Supplementary Figures

Supplementary Figure 1. Layout of the Molecular Diagnostics testing facility.

Supplementary Figure 2. Real time RT-qPCR efficiencies for SARS-CoV-2 viral genes.

Supplementary Tables

Supplementary Table 1. Experimental setup.

Supplementary Table 2. Interpretation of results.

Supplementary Table 3. Validation of the LOD using 300 copies of viral particles per reaction.

Supplementary Table 4. Validation of the LOD using 20 copies of viral particles per reaction.

Supplementary Table 5. Validation of the LOD using 1.3 copies of viral particles per reaction.

Supplementary Table 6. Analyses of contrived samples.

Supplementary Table 7. Analyses of participant samples.

Supplementary Table 8. Impact of pooling on individually tested samples.

Supplementary Table 9. Summary of demographic and clinical data from three health districts in Virginia.

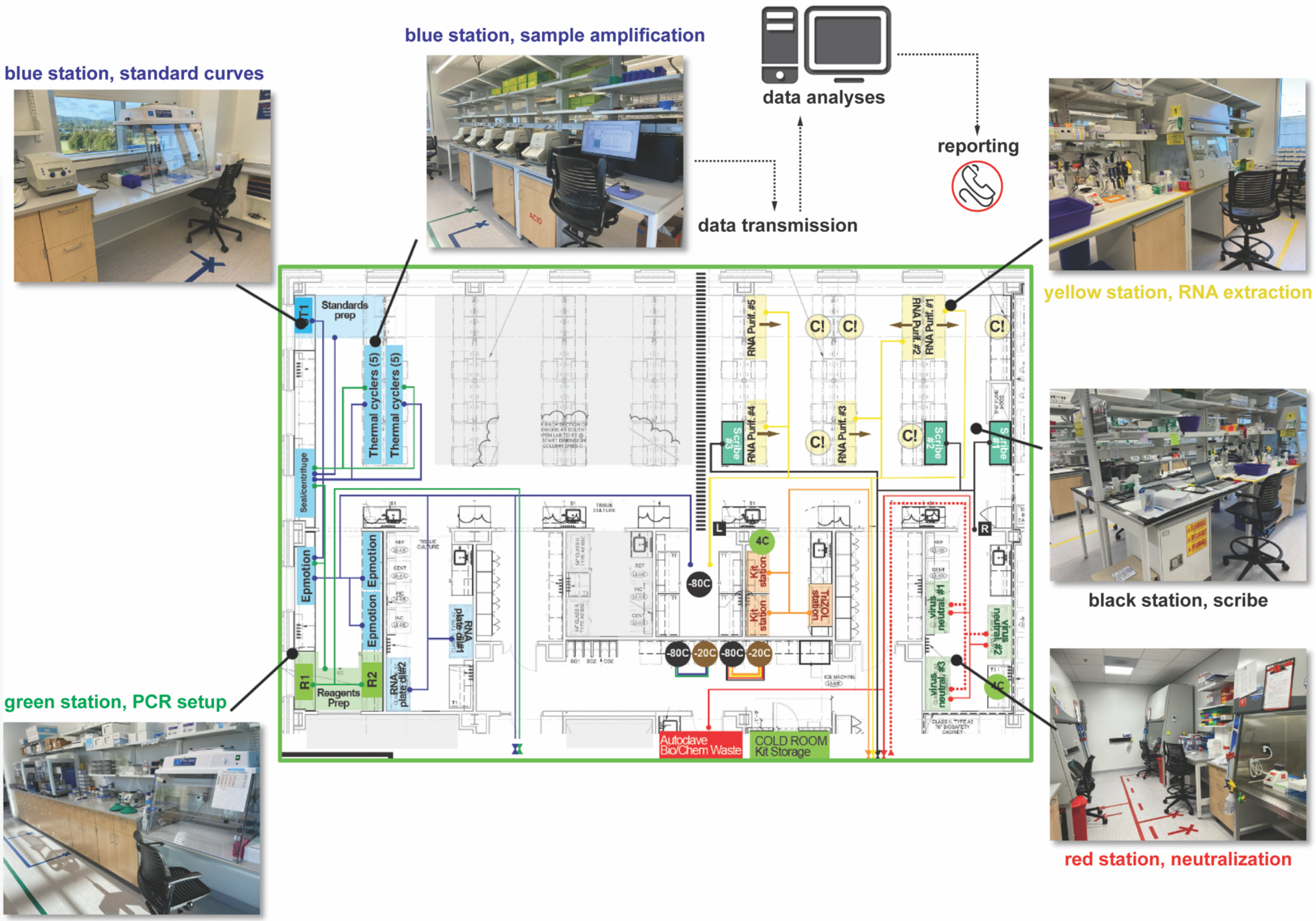
blue station, standard curves
blue station, sample amplification
data analyses
data transmission
reporting
yellow station, RNA extraction
black station, scribe
red station, neutralization
green station, PCR setup
T1
Standards prep
Thermal cyclers (5)
Thermal cyclers (5)
Seal/centrifuge
Epmotion
Epmotion
Epmotion
R1
Reagents Prep
R2
RNA plate dil#2
RNA plate dil#1
RNA Purif. #5
RNA Purif. #4
Scribe #3
RNA Purif. #3
RNA Purif. #2
RNA Purif. #1
Scribe #2
Scribe #1
C!
-80C
-20C
4C
Kit station
Kit station
TriZOL station
L
R
virus neutral. #1
virus neutral. #2
virus neutral. #3
Autoclave Bio/Chem Waste
COLD ROOM Kit Storage

Supplementary Figure 1. Layout of the Molecular Diagnostics testing facility. Diagram of the COVID-19 testing laboratory indicating the various work stations and locations of major equipment with color-coded markings along the floor indicating paths for specifically assigned laboratory personnel. Neutralization room (red station): Clinical samples are received, checked for compliance, neutralized, and aliquots separated for freezing in this location. Scribe section (black station): Neutralized samples are checked into the system and keys are assembled to follow samples throughout the pipeline. RNA extraction (yellow station): Technicians process plate samples in dedicated workstations to avoid contamination. Once the procedure is completed and samples eluted, plates are sealed and stored at -80°C for RT-qPCR amplification. PCR setup (Green station): Reaction mixture for RT-qPCR amplifications are set up in a PCR-clean workstation located opposite from where RNA samples were processed and in a dedicated alcove. The assembled PCR master mix is loaded into an automated epMotion liquid handler and dispensed in a 384-well plate format. Standard curves and sample amplification (blue station): A separate station is allocated to the manual loading of the standard curves for the *N* and *RPP30* genes as well as the positive (inactive virus) and negative (no template reaction) controls that are added to each plate. Amplification is carried out using 18 Bio-Rad CFX384 Touch Real-Time PCR detection systems placed in parallel sides of an island, connected to power backup systems, and remotely monitored during the run and for data retrieval.

Copy # (10^X)	N	E	S
0	37.93333	36.56667	-
1	34.34667	34.04000	33.48333
2	30.56667	30.44667	29.99667
3	27.08333	26.83667	26.59000
4	23.63667	23.26667	23.16000

slope	-3.58570	-3.38030	-3.43770
efficiency	1.90058	1.97620	1.95385
percentage	90.05775	97.62038	95.3854

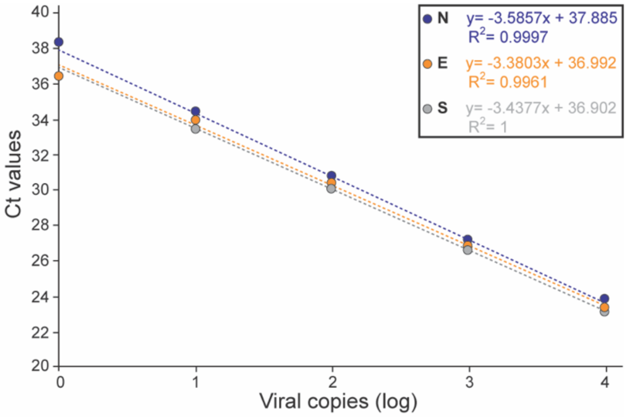

Supplementary Figure 2. Real time RT-qPCR efficiencies for SARS-CoV-2 viral genes. For N and E, n=5; for S, n=4. For each gene, a model fit was performed, and the quality of the model fit was summarized using R^2.

a.

PCR Master Mix:

Master mix components	Volume per reaction (µl)/well
RT-PCR enzyme mix (125x)	0.08
SYBR™ master mix (2x)	5
4µM Forward Primer	0.5
4µM Reverse Primer	0.5
DNase/RNase free water	1.92
Total	8

+ 2 µl (1:20 dilution) of RNA template, positive control plasmid, mon-template control water.
Cyclin parameters:

Cycling Stage	Step	Temperature	Time
Holding	1	48°C	30:00
	2	95°C	10:00
Cycling	3	95°C	00:15
	4	60°C	01:00
	5	Go to Step 3, 44 more times	
Melt Curve, increment 0.5°C	6	95°C	00:15
		65°C	00:15

b.

Primer name	Forward sequence (5' to 3')	Reverse sequence (5' to 3')	Target gene	Gene product
*N**	GCTGCAATCGTGCTACAACT	TGAACTGTTGCGACTACGTG	*N* gene	SARS-CoV-2 nucleocapsid protein
*E**	TTCGGAAGAGACAGGTACGTT	CACACAATCGATGCGCAGTA	*E* gene	SARS-CoV-2 Small envelope protein
S	GCTGGTGCTGCAGCTTATTA	AGGGTCAAGTGCACAGTCTA	*S* gene	SARS-CoV-2 Spike surface glycoprotein
CDC_RNAseP (from CDC) *Hs_RPP30* (this paper)	AGATTTGGACCTGCGAGCG	GAGCGGCTGTCTCCACAAGT	*RPP30* gene	Human Ribonuclease P (RNase P) protein

Supplementary Table 1. Experimental setup. a. Description of the RT-qPCR experimental conditions. b. (*) Primers were published by Won et al. (2020) Exp. Neurobiol. April 30; 29(2):107-119. Doi: 10.5607/en20009 and were based on information deposited in the NCBI site and i) viral sequence access number: GenBank: MT039890.1, ii) *S* gene access number: ACCESSION MT039890 REGION: 21563..25384, https://www.ncbi.nlm.nih.gov/nuccore/MT039890.1?from=21563&to=25384, iii) *E* gene access number: ACCESSION MT039890 REGION: 26245..26472, https://www.ncbi.nlm.nih.gov/nuccore/MT039890.1?from=26245&to=26472, iv) *N* gene access number: MT039890 REGION: 28274..29533, v) https://www.ncbi.nlm.nih.gov/nuccore/MT039890.1?from=28274&to=29533. The sequence of the human RNA specific primer sets that serve as internal control were retrieved from the bulletin published by the Department of Health & Human Services, Centers for Disease Control and Prevention (CDC), Atlanta, GA on 24 Ja. 2020 in the "2019-Novel Coronavirus (2019-nCoV) Real-time rRT-PCR Panel Primers and Probes" document from the Division of Viral Diseases (https://www.cdc.gov/coronavirus/2019-ncov/lab/rt-pcr-panel-primer-probes.html).

N gene	*S* gene	*E* gene	*RPP30* gene	Status	Result	Action
NEG	NEG	NEG	NEG	Invalid	N/A	Repeat test. If the repeat results remain invalid, a new sample would need to be collected.
NEG	NEG	NEG	POS	Valid	SARS-CoV-2 no detected	Report results.
Only one SARS-CoV-2 target gene is POS			POS	Valid	SARS-CoV-2 inconclusive	Repeat test. If the repeat result remains inconclusive, additional confirmation testing should be conducted if clinically indicated.
Two or more SARS-CoV-2 target gene are POS			POS	Valid	SARS-CoV-2 positive	Report results.

Supplementary Table 2. Interpretation of results. Summary of possible result outcomes and their reporting. NEG: Negative (no amplification), POS: Positive (amplification below the threshold).

cut-off value for N: 37.29, 95% CI: [36.91, 37.67]							
**Viral RNA copies/reaction**	**Replicate**	**C_T_N**	**N Conclusion**	**C_T_E**	**E Conclusion**	**C_T_S**	**S Conclusion**
300 copies	1	31.36	Positive	31.45	Positive	31.39	Positive
300 copies	2	31.25	Positive	31.15	Positive	31.79	Positive
300 copies	3	31.02	Positive	31.23	Positive	31.59	Positive
300 copies	4	31.32	Positive	31.55	Positive	31.55	Positive
300 copies	5	31.53	Positive	31.25	Positive	31.41	Positive
300 copies	6	31.28	Positive	30.74	Positive	31.34	Positive
300 copies	7	31.13	Positive	30.97	Positive	31.35	Positive
300 copies	8	31.04	Positive	31.03	Positive	31.31	Positive
300 copies	9	31.19	Positive	31.31	Positive	31.43	Positive
300 copies	10	31.31	Positive	31.45	Positive	31.63	Positive
300 copies	11	31.44	Positive	31.09	Positive	30.98	Positive
300 copies	12	31	Positive	30.73	Positive	30.46	Positive
300 copies	13	30.92	Positive	30.75	Positive	31.1	Positive
300 copies	14	31.09	Positive	31.04	Positive	30.74	Positive
300 copies	15	31.38	Positive	30.94	Positive	31.09	Positive
300 copies	16	31.2	Positive	30.57	Positive	30.53	Positive
300 copies	17	31.15	Positive	30.94	Positive	31.03	Positive
300 copies	18	31.1	Positive	30.79	Positive	30.97	Positive
300 copies	19	31.47	Positive	31.04	Positive	31.06	Positive
300 copies	20	31.36	Positive	31.26	Positive	30.97	Positive
	**SD**	**0.17**		**0.27**		**0.36**	
	**% Positive**		**20/20**		**20/20**		**20/20**

Supplementary Table 3. Validation of the LOD using 300 copies of viral particles per reaction. The cut-off value for the SARS-CoV-2 *N* gene was 37.29, 95% CI: [36.91, 37.67]. Each biological sample (n=20) was analyzed in duplicate for the expression of each gene and the “mean” value is listed.

cut-off value for N: 37.7747, 95% CI: [37.04, 38.51]							
**Viral RNA copies/reaction**	**Replicate**	**C_T_N**	**N Conclusion**	**C_T_E**	**E Conclusion**	**C_T_S**	**S Conclusion**
20 copies	1	35	Positive	33.6	Positive	33.11	Positive
20 copies	2	33.39	Positive	33.72	Positive	33.42	Positive
20 copies	3	33.92	Positive	33.4	Positive	33.07	Positive
20 copies	4	33.38	Positive	33.09	Positive	32.59	Positive
20 copies	5	33.87	Positive	33.6	Positive	34.2	Positive
20 copies	6	33.2	Positive	33.18	Positive	33.43	Positive
20 copies	7	33.21	Positive	33.47	Positive	32.95	Positive
20 copies	8	33.81	Positive	33.8	Positive	34.51	Positive
20 copies	9	34.14	Positive	33.69	Positive	33.28	Positive
20 copies	10	34.04	Positive	34.41	Positive	33.67	Positive
20 copies	11	33.75	Positive	32.96	Positive	33.25	Positive
20 copies	12	34.06	Positive	33.83	Positive	33.2	Positive
20 copies	13	33.67	Positive	33.63	Positive	33.18	Positive
20 copies	14	33.68	Positive	33	Positive	33.27	Positive
20 copies	15	34.35	Positive	33.74	Positive	33.69	Positive
20 copies	16	33.67	Positive	33.35	Positive	33.32	Positive
20 copies	17	33.85	Positive	33.85	Positive	33.02	Positive
20 copies	18	33.71	Positive	33.61	Positive	32.5	Positive
20 copies	19	35.33	Positive	33.44	Positive	32.94	Positive
20 copies	20	33.66	Positive	34.27	Positive	32.82	Positive
	**SD**	**0.5298607**		**0.3733293**		**0.4812363**	
	**% Positive**		**20/20**		**20/20**		**20/20**

Supplementary Table 4. Validation of the LOD using 20 copies of viral particles per reaction. The cut-off value for the SARS-CoV-2 *N* gene was 37.7747, 95% CI: [37.04, 38.51]. Each biological sample (n=20) was analyzed in duplicate for the expression of each gene and the “mean” value is listed.

cut-off value for N: 37.29, 95% CI: [36.91, 37.67]							
Viral RNA copies/reaction	Replicate	C_T_N	N Conclusion	C_T_E	E Conclusion	C_T_S	S Conclusion
1.3 copies	1	37.17	Negative	N/A	Negative	N/A	Negative
1.3 copies	2	N/A	Negative	37.14	Negative	N/A	Negative
1.3 copies	3	N/A	Negative	N/A	Negative	37.49	Negative
1.3 copies	4	N/A	Negative	N/A	Negative	40.63	Negative
1.3 copies	5	N/A	Negative	N/A	Negative	N/A	Negative
1.3 copies	6	37.64	Negative	37.14	Negative	N/A	Negative
1.3 copies	7	N/A	Negative	37.23	Negative	N/A	Negative
1.3 copies	8	N/A	Negative	N/A	Negative	N/A	Negative
1.3 copies	9	N/A	Negative	N/A	Negative	37.5	Negative
1.3 copies	10	N/A	Negative	37.29	Negative	N/A	Negative
1.3 copies	11	N/A	Negative	N/A	Negative	N/A	Negative
1.3 copies	12	37.07	Negative	N/A	Negative	N/A	Negative
1.3 copies	13	N/A	Negative	N/A	Negative	36.6	Negative
1.3 copies	14	N/A	Negative	N/A	Negative	38.38	Negative
1.3 copies	15	N/A	Negative	37.42	Negative	N/A	Negative
1.3 copies	16	N/A	Negative	N/A	Negative	N/A	Negative
1.3 copies	17	37.35	Negative	37.25	Negative	37.55	Negative
1.3 copies	18	N/A	Negative	N/A	Negative	N/A	Negative
1.3 copies	19	N/A	Negative	N/A	Negative	44.65	Negative
1.3 copies	20	N/A	Negative	42.59	Negative	N/A	Negative
	**SD**	0.25		2.02		2.8	
	**% Positive**		**0/20**		**0/20**		**0/20**

Supplementary Table 5. Validation of the LOD using 1.3 copies of viral particles per reaction. The cut-off value for the SARS-CoV-2 *N* gene was 37.29, 95% CI: [36.91, 37.67]. Each biological sample (n=20) was analyzed in duplicate for the expression of each gene and the “mean” value is listed.

Positive samples

**Spiked Samples**	***N* primer** mean **Ct**	***S* primer** mean **Ct**	***E* primer** mean **Ct**	**Interpretation**
1	34.38	32.6	33.13	Positive
2	33.585	32.48	33.075	Positive
3	32.88	32.33	32.745	Positive
4	32.9	32.705	32.655	Positive
5	32.86	32.675	32.21	Positive
6	32.535	32.43	32.83	Positive
7	32.905	32.675	32.56	Positive
8	32.93	32.28	32.66	Positive
9	33.845	32.64	32.935	Positive
10	33.225	33.08	32.87	Positive
11	33.025	32.82	32.64	Positive
12	32.535	32.43	32.325	Positive
13	32.735	33.13	32.63	Positive
14	33.025	32.51	32.945	Positive
15	32.905	32.625	32.875	Positive
16	33.04	32.29	32.52	Positive
17	34.08	32.94	32.67	Positive
18	33.23	33.05	32.51	Positive
19	33.02	33.09	32.705	Positive
20	32.74	32.97	32.685	Positive
21	32.67	32.795	32.91	Positive
22	32.92	32.63	33.05	Positive
23	33.055	32.57	33.07	Positive
24	33.195	32.44	32.185	Positive
25	34.16	32.86	32.56	Positive
26	34.315	33.255	33.115	Positive
27	33.86	33.035	33.085	Positive
**Mean CT**	**33.21**	**32.72**	**32.75**	
**S.D.**	**0.54**	**0.28**	**0.27**	

**Swab Samples**	***N* primer** mean **Ct**	***S* primer** mean **Ct**	***E* primer** mean **Ct**	**Interpretation**
28	20.96	21.72	21.42	Positive
29	22.695	23.52	23.37	Positive
30	34.665	32.5	32.845	Positive
31	24.82	24.85	24.925	Positive
32	24.25	25.37	24.405	Positive
33	23.79	24.445	24.85	Positive
**Mean CT**	**25.20**	**25.40**	**25.30**	
**S.D.**	**4.84**	**3.71**	**3.92**	

(cont.)

Negative samples

**Swab Samples**	***N* primer** mean **Ct**	***S* primer** mean **Ct**	***E* primer** mean **Ct**	**Interpretation**
1	undetermined	undetermined	undetermined	Negative

2	43.08	43.3	38.77	Negative
3	42.12	43.37	44.78	Negative
4	undetermined	undetermined	40.165	Negative
5	undetermined	undetermined	undetermined	Negative
6	undetermined	41.57	40.76	Negative
7	undetermined	36.785	undetermined	Negative
8	undetermined	37.395	undetermined	Negative
9	39.36	41.41	undetermined	Negative
10	undetermined	40.015	44.69	Negative
11	undetermined	undetermined	undetermined	Negative
12	undetermined	undetermined	undetermined	Negative
13	undetermined	undetermined	undetermined	Negative
14	undetermined	undetermined	undetermined	Negative
15	undetermined	undetermined	41.93	Negative
16	undetermined	38.61	43.54	Negative
17	undetermined	undetermined	undetermined	Negative
18	undetermined	undetermined	undetermined	Negative
19	undetermined	undetermined	undetermined	Negative
20	undetermined	undetermined	undetermined	Negative
21	undetermined	undetermined	undetermined	Negative
22	undetermined	undetermined	undetermined	Negative
23	undetermined	undetermined	undetermined	Negative
24	undetermined	undetermined	undetermined	Negative
25	undetermined	undetermined	undetermined	Negative
26	undetermined	undetermined	undetermined	Negative
27	undetermined	undetermined	undetermined	Negative
28	37.56	undetermined	undetermined	Negative
29	38.85	undetermined	undetermined	Negative
30	undetermined	38.65	undetermined	Negative
31	undetermined	40.79	undetermined	Negative
32	undetermined	undetermined	undetermined	Negative
33	undetermined	undetermined	undetermined	Negative

Supplementary Table 6. Analyses of contrived samples. Previously reported negative samples were contrived with either inactive SARS-CoV-2 or BCoVs virus (33 samples each) at concentrations of more than two-times the LOD as determined in Table I. Following, RNA was purified from all samples and amplified using primers to detect *N*, *E*, and *S* genes. Cut-off: Ct: 36.25, 95%CI [35.60, 36.90]. Each biological sample (n=33 positive and n=33 negative) was analyzed in duplicate for the expression of each gene and the “mean” value is listed.

Sample ID	Ct_N	Ct_E	Ct_S	Ct_{RPP30}	Cut-off N	Cut-off RPP30	CDC N1 Ct	CDC N2 Ct	Agreement?
V0001455	18.82	20.85	21.45	28.45	36.51	38.26	17.3	17.2	YES
V0001531	42.23	--	38.93	27.64	37.07	38.34	--	--	YES
V0001533	--	40.97	40.09	27.44	37.07	38.34	--	--	YES
V0001537	37.86	--	39.73	27.11	37.07	38.34	--	--	YES
V0001547	44.16	--	40.36	26.01	37.07	38.34	--	--	YES
V0001550	--	44.64	41.39	27.80	37.07	38.34	--	--	YES
V0001730	--	--	--	29.32	35.60	35.42	--	--	YES
V0001792	24.59	25.37	25.74	32.37	33.94	36.13	18.5	18.5	YES
V0001939	--	--	--	26.94	37.07	38.34	--	--	YES
V0001947	34.55	34.63	33.77	29.87	36.61	34.36	30.8	31.2	YES
V0002420	--	--	36.12	27.13	37.07	38.34	--	--	YES
V0002422	--	--	--	29.47	37.07	38.34	--	--	YES
V0002449	38.09	42.18	43.74	27.50	37.07	38.34	--	--	YES
V0002450	43.12	--	38.56	26.24	37.07	38.34	--	--	YES
V0002455	39.45	--	44.77	27.56	37.07	38.34	--	--	YES
V0002456	--	43.03	39.87	27.0	37.07	38.34	--	--	YES
V0002457	--	--	--	29.77	37.07	38.34	--	--	YES
V0002458	--	42.44	39.81	28.20	37.07	38.34	--	--	YES
V0002459	43.94	--	41.70	27.75	37.07	38.34	--	--	YES
V0002460	42.24	--	--	28.31	37.07	38.34	--	--	YES
V0002463	40.38	--	38.49	27.16	37.07	38.34	--	--	YES
V0002466	43.73	--	43.25	27.04	37.07	38.34	--	--	YES
V0002467	43.59	42.54	39.57	26.37	37.07	38.34	--	--	YES
V0002470	--	41.07	39.11	28.07	37.07	38.34	--	--	YES
V0002471	--	--	--	27.54	37.07	38.34	--	--	YES
V0002472	--	--	44.41	27.97	37.07	38.34	--	--	YES
V0002474	37.34	--	40.06	28.02	37.07	38.34	--	--	YES
V0002475	--	--	42.54	28.14	37.07	38.34	--	--	YES
V0002478	41.09	--	--	27.57	37.07	38.34	--	40.6	YES
V0002479	41.29	--	40.19	26.76	37.07	38.34	--	--	YES
V0002481	--	--	43.63	28.02	37.07	38.34	--	--	YES
V0002482	--	--	43.37	28.40	37.07	38.34	--	--	YES
V0002483	--	--	37.84	27.52	37.07	38.34	--	--	YES
V0002487	--	--	40.31	27.73	37.07	38.34	--	--	YES
V0002489	--	--	39.34	27.80	37.07	38.34	--	--	YES
V0002510	--	37.3	--	29.31	35.60	35.42	--	--	YES
V0002520	--	36.45	--	27.37	35.60	35.42	--	--	YES
V0003324	41.12	--	--	28.02	35.60	35.42	--	--	YES
V0003341	--	--	--	29.35	35.60	35.42	--	--	YES
V0003371	31.62	31.51	31.89	31.11	33.65	35.31	27.6	27.6	YES
V0003376	27.54	27.62	27.84	29.89	33.65	35.31	23.5	23.5	YES
V0004170	24.56	24.79	24.88	32.41	34.36	34.94	15.6	16.0	YES
V0004173	20.57	21.52	21.40	29.37	34.34	35.47	16.6	16.6	YES
V0004179	25.08	25.83	25.28	29.48	34.34	35.47	20.1	20.2	YES
V0004182	24.92	27.03	26.47	33.53	36.77	37.91	17.2	17.2	YES
V0004184	30.47	34.66	33.28	34.83	36.77	37.91	21.5	21.5	YES
V0004191	26.14	27.90	27.32	35.26	36.77	37.91	17.1	16.8	YES
V0004195	31.10	32.23	31.44	34.49	36.77	37.91	22.3	21.8	YES
V0004196	27.93	28.04	27.70	33.62	34.34	35.47	21.9	21.5	YES
V0004197	32.24	32.65	32.40	28.32	34.34	35.47	27.2	27.7	YES

(cont.)

V0004198	34.38	36.20	35.54	35.49	36.77	37.91	23.5	23.4	YES
V0004199	31.72	31.90	32.85	38.66	34.36	34.94	16.4	16.7	YES
V0004200	28.88	31.17	30.35	36.05	36.77	37.91	18.3	18.3	YES
V0004201	26.63	28.49	28.30	35.37	36.77	37.91	15.8	16.3	YES
V0004202	28.61	30.30	29.32	34.53	36.77	37.91	20.2	19.9	YES
V0004205	23.02	25.07	23.98	35.68	36.77	37.91	15.7	16.1	YES
V0004222	25.38	25.19	24.93	30.17	34.34	35.47	19.7	19.4	YES
V0004230	30.65	30.97	30.80	36.19	34.36	34.94	19.9	19.0	YES
V0004251	24.59	25.98	25.88	30.82	34.34	35.47	19.6	19.5	YES
V0004258	27.57	28.01	27.68	29.81	34.34	35.47	19.8	21.0	YES
V0004309	27.01	29.15	29.49	31.88	34.36	34.94	18.7	18.9	YES
V0004312	32.22	32.34	32.40	36.22	34.36	34.94	22.3	22.0	YES
V0004313	30.03	30.15	29.97	26.41	34.34	35.47	26.2	25.9	YES
V0004318	21.42	24.36	23.99	35.36	36.77	37.91	15.5	16.3	YES
V0004319	20.52	22.66	21.72	26.81	34.36	34.94	16.6	17.4	YES
V0004323	24.92	25.96	26.55	34.11	34.36	34.94	16.1	16.3	YES
V0004324	26.08	28.82	28.41	34.26	36.77	37.91	16.3	17.0	YES
V0004329	30.18	30.68	30.17	30.80	34.34	35.47	35.1	38.1	YES
V0004338	29.38	31.72	31.61	34.37	36.77	37.91	20.1	20.2	YES
V0004342	21.86	24.98	24.99	35.27	36.77	37.91	15.6	16.4	YES
V0004344	27.40	29.58	28.63	35.93	36.77	37.91	16.7	17.5	YES
V0004349	27.18	30.11	29.70	36.25	36.77	37.91	17.4	17.6	YES
V0004352	25.46	26.81	27.03	35.10	34.36	34.94	15.9	15.9	YES
V0004360	32.69	34.27	35.14	34.44	34.36	34.94	23.0	22.8	YES
V0004364	26.04	28.54	28.02	34.92	36.77	37.91	16.4	16.7	YES
V0004365	28.43	29.83	29.05	36.5	36.77	37.91	18.9	18.6	YES
V0004366	32.46	31.49	31.89	36.64	34.36	34.94	16.4	16.9	YES
V0004368	23.71	24.54	24.66	30.76	34.34	35.47	17.4	17.3	YES
V0004380	26.66	27.33	27.07	37.34	34.36	34.94	15.8	16.0	YES
V0004565	29.14	31.81	31.29	35.13	36.77	37.91	17.7	17.9	YES
V0004567	22.30	22.64	22.43	28.68	34.34	35.47	18.2	18.2	YES

Supplementary Table 7. Analyses of participant samples. Samples from participants were separated in two aliquots. One aliquot was analyzed using our test and its duplicate was submitted for confirmation to the Virginia Division of Consolidated Lab Services (DCLS) in Virginia. For each sample, Ct_N, Ct_E, Ct_S, and Ct_{RPP30} indicate Ct values when obtained using our test. CDC *N1* and *N2* indicate Ct values obtained by DCLS. Under “Agreement”, “Yes” indicates consensus between our test and the DCLS result. Each biological sample (n=81) was analyzed in duplicate for the expression of each gene and the “mean” value is listed.

a.

		Pooled Result	
		-	+
Individual Result	-	20	0
	+	0	21

b.

Gene	Slope	Lower 95% CI	Upper 95% CI
N	0.94	0.86	1.11
E	0.96	0.83	1.10
S	0.98	0.84	1.17

Supplementary Table 8. Impact of pooling on individually tested samples. a. Two-by-two report of positive percent agreement (PPA) and negative percent agreement (NPA). b. Passing-Bablok regression analysis for the *N*, *E*, and *S* genes.

		MRHD	NRVHD	FHD
Demographics*	Population	187,324	182,147	1,147,532
	Median age	47.4	34.4	38.4
	Population under 18	19%	16%	23%
	Population 18-64	58%	67%	63%
	Population +65	23%	16%	14%
	Per capita income	25073	28,210	57,492
	Median household income	44600	55825	128374
	Persons below poverty line	14.80%	21.1	6%
	High school or higher	84.6%	91.90%	92.70%
	Bachelor's degree or higher	21%	34.80%	62.40%
	Veteran status	8.80%	5.90%	8.50%
Clinical data				
	Uninsured*	10.50%	6.97%	8.18%
	Medicaid*	15.60%	9.08%	6.03%
	Medicare*	18%	12.20%	8.36%
	Employer coverage*	40.9	54.60%	58.40%
	Military or VA*	2.09%	2.07%	3.81%
	ICU beds**	16	35	333
	ICU bed per capita***	11,708 people to 1 bed	5,204 people to 1 bed	3,446 people to 1 bed

Supplementary Table 9. Summary of demographic and clinical data from three health districts in Virginia. MRHD: Mt. Rogers Health District, NRVHD: New River Valley Health District, FHD: Fairfax Health District. Data was obtained from * https://datausa.io/ , ** U.S. Department of Health and Human Services (https://healthdata.gov/dataset/covid-19-reported-patient-impact-and-hospital-capacity-facility) and https://www.vdh.virginia.gov/ , *** population in health district divided by the number of ICU beds.
