# Supplementary material for: Development and implementation of a scalable and versatile test for COVID-19 diagnostics in rural communities
Source: Nat Commun. 2021 Jul 20;12:4400. doi: 10.1038/s41467-021-24552-4 (PMC8292415; doi:10.1038/s41467-021-24552-4)
Supplement: Supplementary file 2 — Description of Additional Supplementary Files [file 41467_2021_24552_MOESM2_ESM.pdf]

Supplementary Data 1. Predicted analytical sensitivity of the COVID-19 assay primers using SARS-CoV-2 sequences downloaded from NCBI. Virus sequences were sorted by their DECIPHER database number. Sequences are shown 5' to 3'. For each virus sequence, a dot is shown if the same nucleotide is found in the COVID-19 primer at that position. The IUPAC nucleotide code is given when there is a difference at that position compared to the assay primer. Virus sequences with mismatches that may impact assay performance are identified by gray shading and are tabulated in the appropriate column. Poor quality virus sequences were separated into their own spreadsheet and the reason for their exclusion is tabulated.

Supplementary Data 2. Predicted analytical sensitivity of the COVID-19 assay primers using SARS-CoV-2 sequences downloaded from GISAID. Virus sequences were divided into seven groups based on submission date and placed into seven different spreadsheets. Each group was further divided into four DECIPHER databases. Virus sequences are sorted by their DECIPHER database and database number. Sequences are shown 5' to 3'. For each virus sequence, a dot is shown if the same nucleotide is found in the COVID-19 primer at that position. The IUPAC nucleotide code is given when there is a difference at that position compared to the assay primer. Virus sequences with mismatches that may impact assay performance are identified by gray shading and are tabulated in the appropriate column. Poor quality virus sequences were separated into their own spreadsheet and the reason for their exclusion is tabulated. An accounting of the GISAID sequences is given in the last spreadsheet.

Supplementary Data 3. SARS-Coronavirus (Tax ID: 694009) alignments using BLAST for customized *N* and *E* sets of primers. Alignment of *S* primers did not result in any significant homology.

Supplementary Data 4. Analysis of pooled (n=2) samples. Pool contained n=2 samples, i.e., two negative (named "N") or one positive + one negative (named "P"). Tab "Pool_Raw_Data:" Ct values for *N*, *E*, *S*, and *RPP30* genes are indicated for each sample (columns F-L and P-V) and the pool (column Z-AF). Tab "Positives:" Indicated the Ct difference for all genes of each positive sample compared to the pool value for that sample. Tab "Regression:" Regression analysis Pool (Ct) values versus single Ct values for *N*, *E*, and *S* genes. N/A: No amplification.

Supplementary Data 5. Analysis of pooled (n=3) samples. Pool contained n=3 samples, i.e., three negative (named "N") or one positive + two negative (named "P"). Tab "Pool_Raw_Data:" Ct values for *N*, *E*, *S*, and *RPP30* genes are indicated for each sample (columns F-L, P-V, and Z-AF) and the pool (column AJ-AP). Tab "Positives:" Indicated the Ct difference for all genes of each positive sample compared to the pool value for that sample. Tab "Regression:" Regression analysis Pool (Ct) values versus single Ct values for *N*, *E*, and *S* genes. N/A: No amplification.

Supplementary Data 6. Analysis of pooled (n=4) samples. Pool contained n=4 samples, i.e., four negative (named "N") or one positive + three negative (named "P"). Tab "Pool_Raw_Data:" Ct values for *N*, *E*, *S*, and *RPP30* genes are indicated for each sample (columns C-H, J-O, Q-V, and X-AC) and the pool (column AE-AJ). Tab "Positives:" Indicated the Ct difference for all genes of each positive sample compared to the pool value for that sample. Tab "Regression:" Regression analysis Pool (Ct) values versus single Ct values for *N*, *E*, and *S* genes. N/A: No amplification.

Supplementary Data 7. Theoretical results from pooling experiments using 100 positive samples diluted into pools of n=2, n=3, and n=4. Each worksheet includes the observed Ct values for the *N*, *E*, *S*, and *RPP30* genes (columns E-H), the observed (column I), theoretical (column K), and estimated (column L) plate specific Ct_N thresholds used for determining positivity, and the theoretical (columns M-O) and estimated (columns P-R) results for the *N*, *E*, and *S* genes. Theoretical Ct values were defined using a log2(n) shift in the Ct value of the threshold. Estimated Ct values were defined empirically, as described in the text.

Supplementary Data 8. Matching saliva and swab samples were collected from 122 participants and analyzed by RT-qPCR. Of these, 92 and 30 were reported negative and positive, respectively. Columns E-H show Ct values for the *RPP30*, *N*, *E*, and *S* genes obtained from swab samples. Columns I-J show cut-off values for the *RPP30* and *N* genes for each swab sample. Columns O-R show Ct values for the *RPP30*, *N*, *E*, and *S* genes obtained from saliva samples. Columns S-T show cut-off values for the *RPP30* and *N* genes for each saliva sample. Columns K and U have the test results and the column V states "True" when the results from both sample collection methods for each patient match.
