# Supplementary material for: Development and implementation of a scalable and versatile test for COVID-19 diagnostics in rural communities
Source: Nat Commun. 2021 Jul 20;12:4400. doi: 10.1038/s41467-021-24552-4 (PMC8292415; doi:10.1038/s41467-021-24552-4)
Supplement: Supplementary file 5 — Supplementary Data 3 [file 41467_2021_24552_MOESM5_ESM.pdf]

The following alignments refer to sequences that have filtered results of ≥80% Identity, ≥80% query coverage, and an E value ≤10 for the N forward primer; Total of 3 hits.

Bat coronavirus RaTG13, complete genome

Sequence ID: MN996532.1 **Length:** 29855 **Number of Matches:** 1

Range 1: 28702 to 28721 GenBank Graphics ▼ Next Match ▲ Previous Match

Score	Expect	Identities	Gaps	Strand
40.1 bits(20)	0.47	20/20(100%)	0/20(0%)	Plus/Plus

```
Query  1      GCTGCAATCGTGCTACAACT  20
              ||||||||||||||||||||
Sbjct  28702  GCTGCAATCGTGCTACAACT  28721
```

Bat SARS-like coronavirus isolate bat-SL-CoVZXC21, complete genome

Sequence ID: MG772934.1 **Length:** 29732 **Number of Matches:** 1

Range 1: 28572 to 28591 GenBank Graphics ▼ Next Match ▲ Previous Match

Score	Expect	Identities	Gaps	Strand
40.1 bits(20)	0.47	20/20(100%)	0/20(0%)	Plus/Plus

```
Query  1      GCTGCAATCGTGCTACAACT  20
              ||||||||||||||||||||
Sbjct  28572  GCTGCAATCGTGCTACAACT  28591
```

Bat SARS-like coronavirus isolate bat-SL-CoVZC45, complete genome

Sequence ID: MG772933.1 **Length:** 29802 **Number of Matches:** 1

Range 1: 28641 to 28660 GenBank Graphics ▼ Next Match ▲ Previous Match

Score	Expect	Identities	Gaps	Strand
40.1 bits(20)	0.47	20/20(100%)	0/20(0%)	Plus/Plus

```
Query  1      GCTGCAATCGTGCTACAACT  20
              ||||||||||||||||||||
Sbjct  28641  GCTGCAATCGTGCTACAACT  28660
```

The following alignments refer to sequences that have filtered results of ≥80% Identity, ≥80% query coverage, and an E value ≤10 for the N reverse primer; Total of 3 hits.

Bat coronavirus RaTG13, complete genome

Sequence ID: MN996532.1 **Length:** 29855 **Number of Matches:** 1

Range 1: 28802 to 28821 GenBank Graphics ▼ Next Match ▲ Previous Match

Score	Expect	Identities	Gaps	Strand
40.1 bits(20)	0.47	20/20(100%)	0/20(0%)	Plus/Minus

```
Query  1      TGAACTGTTGCGACTACGTG  20
              ||||||||||||||||||||
Sbjct  28821  TGAACTGTTGCGACTACGTG  28802
```

Bat SARS-like coronavirus isolate bat-SL-CoVZXC21, complete genome

Sequence ID: MG772934.1 **Length:** 29732 **Number of Matches:** 1

Range 1: 28672 to 28691 GenBank Graphics ▼ Next Match ▲ Previous Match

Score	Expect	Identities	Gaps	Strand
40.1 bits(20)	0.47	20/20(100%)	0/20(0%)	Plus/Minus

```
Query  1      TGAACTGTTGCGACTACGTG  20
              ||||||||||||||||||||
Sbjct  28691  TGAACTGTTGCGACTACGTG  28672
```

Bat SARS-like coronavirus isolate bat-SL-CoVZC45, complete genome

Sequence ID: MG772933.1 **Length:** 29802 **Number of Matches:** 1

Range 1: 28741 to 28760 GenBank Graphics ▼ Next Match ▲ Previous Match

Score	Expect	Identities	Gaps	Strand
40.1 bits(20)	0.47	20/20(100%)	0/20(0%)	Plus/Minus

```
Query  1      TGAACTGTTGCGACTACGTG  20
              ||||||||||||||||||||
Sbjct  28760  TGAACTGTTGCGACTACGTG  28741
```

The following alignments refer to sequences that have filtered results of ≥80% Identity, ≥80% query coverage, and an E value ≤10 for the E forward primer; Total of 21 hits.

Bat coronavirus RaTG13, complete genome

Sequence ID: MN996532.1 **Length:** 29855 **Number of Matches:** 1

Range 1: 26229 to 26249 GenBank Graphics ▼ Next Match ▲ Previous Match

Score	Expect	Identities	Gaps	Strand
42.1 bits(21)	0.12	21/21(100%)	0/21(0%)	Plus/Plus

```
Query  1      TTCGGAAGAGACAGGTACGTT  21
              |||||||||||||||||||||
Sbjct  26229  TTCGGAAGAGACAGGTACGTT  26249
```

Bat SARS-like coronavirus isolate bat-SL-CoVZXC21, complete genome

Sequence ID: MG772934.1 **Length:** 29732 **Number of Matches:** 1

Range 1: 26095 to 26115 GenBank Graphics ▼ Next Match ▲ Previous Match

Score	Expect	Identities	Gaps	Strand
42.1 bits(21)	0.12	21/21(100%)	0/21(0%)	Plus/Plus

```
Query  1      TTCGGAAGAGACAGGTACGTT  21
              |||||||||||||||||||||
Sbjct  26095  TTCGGAAGAGACAGGTACGTT  26115
```

Bat SARS-like coronavirus isolate bat-SL-CoVZC45, complete genome

Sequence ID: MG772933.1 **Length:** 29802 **Number of Matches:** 1

Range 1: 26164 to 26184 GenBank Graphics ▼ Next Match ▲ Previous Match

Score	Expect	Identities	Gaps	Strand
42.1 bits(21)	0.12	21/21(100%)	0/21(0%)	Plus/Plus

```
Query  1      TTCGGAAGAGACAGGTACGTT  21
              |||||||||||||||||||||
Sbjct  26164  TTCGGAAGAGACAGGTACGTT  26184
```

BtRs-BetaCoV/YN2013, complete genome

Sequence ID: KJ473816.1 **Length:** 29142 **Number of Matches:** 1

Range 1: 25831 to 25851 GenBank Graphics ▼ Next Match ▲ Previous Match

Score	Expect	Identities	Gaps	Strand
42.1 bits(21)	0.12	21/21(100%)	0/21(0%)	Plus/Plus

```
Query  1      TTCGGAAGAGACAGGTACGTT  21
              |||||||||||||||||||||
Sbjct  25831  TTCGGAAGAGACAGGTACGTT  25851
```

BtRs-BetaCoV/GX2013, complete genome

Sequence ID: KJ473815.1 **Length:** 29161 **Number of Matches:** 1

Range 1: 25847 to 25867 GenBank Graphics ▼ Next Match ▲ Previous Match

Score	Expect	Identities	Gaps	Strand
42.1 bits(21)	0.12	21/21(100%)	0/21(0%)	Plus/Plus

```
Query  1      TTCGGAAGAGACAGGTACGTT  21
              |||||||||||||||||||||
Sbjct  25847  TTCGGAAGAGACAGGTACGTT  25867
```

BtRs-BetaCoV/HuB2013, complete genome

Sequence ID: KJ473814.1 **Length:** 29658 **Number of Matches:** 1

Range 1: 26021 to 26041 GenBank Graphics ▼ Next Match ▲ Previous Match

Score	Expect	Identities	Gaps	Strand
42.1 bits(21)	0.12	21/21(100%)	0/21(0%)	Plus/Plus

```
Query  1      TTCGGAAGAGACAGGTACGTT  21
              |||||||||||||||||||||
Sbjct  26021  TTCGGAAGAGACAGGTACGTT  26041
```

SARS-related bat coronavirus isolate Longquan-140 orf1ab polyprotein, spike glycoprotein, envelope protein, membrane protein, and nucleocapsid protein genes, complete cds

Sequence ID: KF294457.1 **Length:** 29676 **Number of Matches:** 1

Range 1: 26061 to 26081 GenBank Graphics ▼ Next Match ▲ Previous Match

Score	Expect	Identities	Gaps	Strand
42.1 bits(21)	0.12	21/21(100%)	0/21(0%)	Plus/Plus

```
Query  1      TTCGGAAGAGACAGGTACGTT  21
              |||||||||||||||||||||
Sbjct  26061  TTCGGAAGAGACAGGTACGTT  26081
```

Bat coronavirus Cp/Yunnan2011, complete genome

Sequence ID: JX993988.1 **Length:** 29452 **Number of Matches:** 1

Range 1: 25989 to 26009 GenBank Graphics ▼ Next Match ▲ Previous Match

Score	Expect	Identities	Gaps	Strand
42.1 bits(21)	0.12	21/21(100%)	0/21(0%)	Plus/Plus

```
Query  1      TTCGGAAGAGACAGGTACGTT  21
              |||||||||||||||||||||
Sbjct  25989  TTCGGAAGAGACAGGTACGTT  26009
```

Bat SARS coronavirus HKU3-13, complete genome

Sequence ID: GQ153548.1 **Length:** 29677 **Number of Matches:** 1

Range 1: 26045 to 26065 GenBank Graphics ▼ Next Match ▲ Previous Match

Score	Expect	Identities	Gaps	Strand
42.1 bits(21)	0.12	21/21(100%)	0/21(0%)	Plus/Plus

```
Query  1      TTCGGAAGAGACAGGTACGTT  21
              |||||||||||||||||||||
Sbjct  26045  TTCGGAAGAGACAGGTACGTT  26065
```

Bat SARS coronavirus HKU3-12, complete genome

Sequence ID: GQ153547.1 **Length:** 29704 **Number of Matches:** 1

Range 1: 26072 to 26092 GenBank Graphics ▼ Next Match ▲ Previous Match

Score	Expect	Identities	Gaps	Strand
42.1 bits(21)	0.12	21/21(100%)	0/21(0%)	Plus/Plus

```
Query  1      TTCGGAAGAGACAGGTACGTT  21
              |||||||||||||||||||||
Sbjct  26072  TTCGGAAGAGACAGGTACGTT  26092
```

Bat SARS coronavirus HKU3-11, complete genome

Sequence ID: GQ153546.1 **Length:** 29695 **Number of Matches:** 1

Range 1: 26063 to 26083 GenBank Graphics ▼ Next Match ▲ Previous Match

Score	Expect	Identities	Gaps	Strand
42.1 bits(21)	0.12	21/21(100%)	0/21(0%)	Plus/Plus

```
Query  1      TTCGGAAGAGACAGGTACGTT  21
              |||||||||||||||||||||
Sbjct  26063  TTCGGAAGAGACAGGTACGTT  26083
```

Bat SARS coronavirus HKU3-10, complete genome

Sequence ID: GQ153545.1 **Length:** 29695 **Number of Matches:** 1

Range 1: 26063 to 26083 GenBank Graphics ▼ Next Match ▲ Previous Match

Score	Expect	Identities	Gaps	Strand
42.1 bits(21)	0.12	21/21(100%)	0/21(0%)	Plus/Plus

```
Query  1      TTCGGAAGAGACAGGTACGTT  21
              |||||||||||||||||||||
Sbjct  26063  TTCGGAAGAGACAGGTACGTT  26083
```

Bat SARS coronavirus HKU3-9, complete genome

Sequence ID: GQ153544.1 **Length:** 29695 **Number of Matches:** 1

Range 1: 26063 to 26083 GenBank Graphics ▼ Next Match ▲ Previous Match

Score	Expect	Identities	Gaps	Strand
42.1 bits(21)	0.12	21/21(100%)	0/21(0%)	Plus/Plus

```
Query  1      TTCGGAAGAGACAGGTACGTT  21
              |||||||||||||||||||||
Sbjct  26063  TTCGGAAGAGACAGGTACGTT  26083
```

Bat SARS coronavirus HKU3-8, complete genome

Sequence ID: GQ153543.1 **Length:** 29681 **Number of Matches:** 1

Range 1: 26075 to 26095 GenBank Graphics ▼ Next Match ▲ Previous Match

Score	Expect	Identities	Gaps	Strand
42.1 bits(21)	0.12	21/21(100%)	0/21(0%)	Plus/Plus

```
Query  1      TTCGGAAGAGACAGGTACGTT  21
              |||||||||||||||||||||
Sbjct  26075  TTCGGAAGAGACAGGTACGTT  26095
```

Bat SARS coronavirus HKU3-7, complete genome

Sequence ID: GQ153542.1 **Length:** 29716 **Number of Matches:** 1

Range 1: 26084 to 26104 GenBank Graphics ▼ Next Match ▲ Previous Match

Score	Expect	Identities	Gaps	Strand
42.1 bits(21)	0.12	21/21(100%)	0/21(0%)	Plus/Plus

```
Query  1      TTCGGAAGAGACAGGTACGTT  21
              |||||||||||||||||||||
Sbjct  26084  TTCGGAAGAGACAGGTACGTT  26104
```

Bat SARS coronavirus HKU3-6, complete genome

Sequence ID: GQ153541.1 **Length:** 29704 **Number of Matches:** 1

Range 1: 26072 to 26092 GenBank Graphics ▼ Next Match ▲ Previous Match

Score	Expect	Identities	Gaps	Strand
42.1 bits(21)	0.12	21/21(100%)	0/21(0%)	Plus/Plus

```
Query  1      TTCGGAAGAGACAGGTACGTT  21
              |||||||||||||||||||||
Sbjct  26072  TTCGGAAGAGACAGGTACGTT  26092
```

Bat SARS coronavirus HKU3-5, complete genome

Sequence ID: GQ153540.1 **Length:** 29704 **Number of Matches:** 1

Range 1: 26072 to 26092 GenBank Graphics ▼ Next Match ▲ Previous Match

Score	Expect	Identities	Gaps	Strand
42.1 bits(21)	0.12	21/21(100%)	0/21(0%)	Plus/Plus

```
Query  1      TTCGGAAGAGACAGGTACGTT  21
              |||||||||||||||||||||
Sbjct  26072  TTCGGAAGAGACAGGTACGTT  26092
```

Bat SARS coronavirus HKU3-4, complete genome

Sequence ID: GQ153539.1 **Length:** 29704 **Number of Matches:** 1

Range 1: 26072 to 26092 GenBank Graphics ▼ Next Match ▲ Previous Match

Score	Expect	Identities	Gaps	Strand
42.1 bits(21)	0.12	21/21(100%)	0/21(0%)	Plus/Plus

```
Query  1      TTCGGAAGAGACAGGTACGTT  21
              |||||||||||||||||||||
Sbjct  26072  TTCGGAAGAGACAGGTACGTT  26092
```

bat SARS coronavirus HKU3-3, complete genome

Sequence ID: DQ084200.1 **Length:** 29711 **Number of Matches:** 1

Range 1: 26055 to 26075 GenBank Graphics ▼ Next Match ▲ Previous Match

Score	Expect	Identities	Gaps	Strand
42.1 bits(21)	0.12	21/21(100%)	0/21(0%)	Plus/Plus

```
Query  1      TTCGGAAGAGACAGGTACGTT  21
              |||||||||||||||||||||
Sbjct  26055  TTCGGAAGAGACAGGTACGTT  26075
```

Bat SARS coronavirus HKU3-1, complete genome

Sequence ID: DQ022305.2 **Length:** 29728 **Number of Matches:** 1

Range 1: 26072 to 26092 GenBank Graphics ▼ Next Match ▲ Previous Match

Score	Expect	Identities	Gaps	Strand
42.1 bits(21)	0.12	21/21(100%)	0/21(0%)	Plus/Plus

```
Query  1      TTCGGAAGAGACAGGTACGTT  21
              |||||||||||||||||||||
Sbjct  26072  TTCGGAAGAGACAGGTACGTT  26092
```

bat SARS coronavirus HKU3-2, complete genome

Sequence ID: DQ084199.1 **Length:** 29687 **Number of Matches:** 1

Range 1: 26055 to 26075 GenBank Graphics ▼ Next Match ▲ Previous Match

Score	Expect	Identities	Gaps	Strand
42.1 bits(21)	0.12	21/21(100%)	0/21(0%)	Plus/Plus

```
Query  1      TTCGGAAGAGACAGGTACGTT  21
              |||||||||||||||||||||
Sbjct  26055  TTCGGAAGAGACAGGTACGTT  26075
```

The following alignments refer to sequences that have filtered results of ≥80% Identity, ≥80% query coverage, and an E value ≤10 for the E reverse primer; Total of 3 hits.

SARS coronavirus ExoN1 strain SARS/VeroE6_lab/USA/ExoN1_c5.7P20/2010, complete genome

Sequence ID: KF514407.1 **Length:** 29689 **Number of Matches:** 1

Range 1: 26198 to 26217 GenBank Graphics ▼ Next Match ▲ Previous Match

Score	Expect	Identities	Gaps	Strand
40.1 bits(20)	0.47	20/20(100%)	0/20(0%)	Plus/Minus

```
Query  1      CACACAATCGATGCGCAGTA  20
              ||||||||||||||||||||
Sbjct  26217  CACACAATCGATGCGCAGTA  26198
```

SARS coronavirus ExoN1 isolate c5P10, complete genome

Sequence ID: JX162087.1 **Length:** 29688 **Number of Matches:** 1

Range 1: 26198 to 26217 GenBank Graphics ▼ Next Match ▲ Previous Match

Score	Expect	Identities	Gaps	Strand
40.1 bits(20)	0.47	20/20(100%)	0/20(0%)	Plus/Minus

```
Query  1      CACACAATCGATGCGCAGTA  20
              ||||||||||||||||||||
Sbjct  26217  CACACAATCGATGCGCAGTA  26198
```

SARS coronavirus ExoN1 isolate P3pp53, complete genome

Sequence ID: FJ882956.1 **Length:** 29644 **Number of Matches:** 1

Range 1: 26178 to 26197 GenBank Graphics ▼ Next Match ▲ Previous Match

Score	Expect	Identities	Gaps	Strand
40.1 bits(20)	0.47	20/20(100%)	0/20(0%)	Plus/Minus

```
Query  1      CACACAATCGATGCGCAGTA  20
              ||||||||||||||||||||
Sbjct  26197  CACACAATCGATGCGCAGTA  26178
```
