# Supplementary material for: Development and implementation of a scalable and versatile test for COVID-19 diagnostics in rural communities
Source: Nat Commun. 2021 Jul 20;12:4400. doi: 10.1038/s41467-021-24552-4 (PMC8292415; doi:10.1038/s41467-021-24552-4)
Supplement: Supplementary file 6 — Supplementary Data 4 [file 41467_2021_24552_MOESM6_ESM.pdf]

Pool raw data

A	C	D	E	F	G	H	I	J	K	L	M	N	O	P	Q	R	S	T	U	V	W	X	Y	Z	AA	AB	AC	AD	AE	AF
	Sample 1										Sample 2										Pool									
Pool name	Original test date	Plate number	Sample	Ct_{RPP30}	Ct_N	Ct_E	Ct_S	Result	cut-off N	cut-off RPP30	Original test date	Plate number	Sample	Ct_{RPP}	Ct_N	Ct_E	Ct_S	Result	cut-off N	cut-off RPP30	Test date	Plate number	Sample	Ct_{RPP}	Ct_N	Ct_E	Ct_S	Result	cut-off N	cut-off RPP30
1N				29.19	N/A	N/A	44.51	Negative	35.59	34.67				28.94	N/A	N/A	N/A	Negative	35.59	34.67			1N	29.72	N/A	N/A	41.24	Negative	35.54	36.06
2N				29.20	N/A	N/A	N/A	Negative	35.59	34.67				28.87	N/A	N/A	N/A	Negative	35.59	34.67			2N	29.97	38.77	N/A	N/A	Negative	35.54	36.06
3N				29.73	N/A	N/A	N/A	Negative	35.59	34.67				29.48	N/A	N/A	N/A	Negative	35.59	34.67			3N	31.05	N/A	N/A	N/A	Negative	35.54	36.06
4N				29.80	N/A	N/A	N/A	Negative	35.59	34.67				29.48	N/A	N/A	N/A	Negative	35.59	34.67			4N	30.23	N/A	N/A	N/A	Negative	35.54	36.06
5N				29.73	N/A	N/A	N/A	Negative	35.59	34.67				30.36	N/A	N/A	N/A	Negative	35.59	34.67			5N	30.77	N/A	N/A	N/A	Negative	35.97	34.87
6N				29.98	N/A	N/A	N/A	Negative	35.59	34.67				30.14	N/A	N/A	N/A	Negative	35.59	34.67			6N	30.51	37.11	N/A	N/A	Negative	35.97	34.87
7N				29.13	N/A	N/A	N/A	Negative	35.59	34.67				28.96	N/A	N/A	N/A	Negative	35.59	34.67			7N	29.18	N/A	N/A	42.43	Negative	36.42	36.49
8N				28.92	N/A	N/A	39.89	Negative	35.55	35.75				31.16	43.59	N/A	N/A	Negative	35.59	34.67			8N	30.27	42.24	N/A	41.85	Negative	36.42	36.49
9N				31.06	N/A	N/A	N/A	Negative	35.59	34.67				29.30	N/A	N/A	N/A	Negative	35.59	34.67			9N	30.22	37.15	N/A	N/A	Negative	36.42	36.49
10N				29.97	N/A	N/A	N/A	Negative	35.59	34.67				30.28	38.02	N/A	N/A	Negative	35.55	35.75			10N	29.25	N/A	N/A	N/A	Negative	36.10	34.84
11N				29.37	N/A	N/A	43.98	Negative	35.60	35.06				30.20	N/A	N/A	N/A	Negative	35.60	35.06			11N	29.89	N/A	N/A	N/A	Negative	36.15	35.56
12N				29.96	N/A	N/A	N/A	Negative	35.60	35.06				30.01	40.05	N/A	N/A	Negative	35.60	35.06			12N	29.62	N/A	N/A	N/A	Negative	36.15	35.56
13N				31.63	N/A	N/A	N/A	Negative	35.60	35.06				30.16	N/A	N/A	N/A	Negative	35.60	35.06			13N	30.01	N/A	N/A	N/A	Negative	36.15	35.56
14N				29.81	N/A	N/A	N/A	Negative	35.60	35.06				30.22	N/A	N/A	N/A	Negative	35.60	35.06			14N	29.62	N/A	42.02	N/A	Negative	36.15	35.56
15N				28.87	N/A	N/A	N/A	Negative	35.60	35.06				29.46	N/A	N/A	37.69	Negative	35.60	35.06			15N	28.94	N/A	N/A	N/A	Negative	36.15	35.56
16N				29.23	N/A	N/A	N/A	Negative	35.60	35.06				26.20	N/A	N/A	43.98	Negative	35.60	35.06			16N	29.27	40.62	N/A	N/A	Negative	36.15	35.56
17N				29.97	N/A	N/A	N/A	Negative	35.60	35.06				29.48	36.31	N/A	N/A	Negative	35.60	35.06			17N	30.19	N/A	N/A	N/A	Negative	36.15	35.56
18N				30.02	N/A	N/A	N/A	Negative	35.60	35.06				28.88	N/A	N/A	N/A	Negative	35.60	35.06			18N	29.86	N/A	N/A	N/A	Negative	36.15	35.56
19N				29.96	N/A	N/A	N/A	Negative	35.60	35.06				29.63	N/A	N/A	N/A	Negative	35.60	35.06			19N	29.81	N/A	N/A	N/A	Negative	36.15	35.56
20N				29.95	N/A	N/A	N/A	Negative	35.60	35.06				30.24	38.69	N/A	N/A	Negative	35.60	35.06			20N	30.13	N/A	N/A	N/A	Negative	36.15	35.56
1P				29.62	13.39	14.94	15.31	Positive	35.87	36.02				28.94	N/A	N/A	N/A	Negative	35.59	34.67			1P	29.94	14.52	16.19	16.66	Positive	36.42	36.49
2P				30.67	17.24	17.99	17.77	Positive	35.87	36.02				28.87	N/A	N/A	N/A	Negative	35.59	34.67			2P	30.12	18.21	19.05	18.82	Positive	36.42	36.49
3P				30.61	18.55	20.04	20.17	Positive	35.87	36.02				29.48	N/A	N/A	N/A	Negative	35.59	34.67			3P	30.86	21.31	23.07	23.23	Positive	36.42	36.49
4P				31.02	22.28	22.27	22.07	Positive	36.09	35.73				29.48	N/A	N/A	N/A	Negative	35.59	34.67			4P	30.11	22.92	23.08	22.91	Positive	36.42	36.49
5P				31.22	17.15	18.22	18.54	Positive	36.09	35.73				30.35	N/A	N/A	N/A	Negative	35.59	34.67			5P	31.23	18.43	19.50	19.91	Positive	36.42	36.49
6P				29.15	17.18	18.54	18.88	Positive	36.09	35.73				31.05	N/A	N/A	N/A	Negative	35.59	34.67			6P	29.85	18.07	19.24	19.56	Positive	36.42	36.49
7P				29.99	17.05	19.17	19.53	Positive	35.97	36.32				30.36	N/A	N/A	N/A	Negative	35.59	34.67			7P	31.25	18.92	20.93	21.63	Positive	36.42	36.49
8P				32.00	22.00	23.00	23.30	Positive	35.47	36.52				30.14	N/A	N/A	N/A	Negative	35.59	34.67			8P	31.16	22.81	24.44	24.68	Positive	36.42	36.49
9P				29.13	16.78	18.44	19.05	Positive	36.14	36.22				29.94	N/A	N/A	N/A	Negative	35.59	34.67			9P	29.81	17.82	19.52	20.00	Positive	36.42	36.49
10P				29.02	24.75	25.29	25.51	Positive	35.53	35.84				30.45	N/A	N/A	N/A	Negative	35.59	34.67			10P	28.40	23.86	24.85	25.31	Positive	36.42	36.49
11P				30.89	15.28	17.27	17.78	Positive	35.88	34.58				28.96	N/A	N/A	N/A	Negative	35.59	34.67			11P	29.84	15.23	17.08	17.49	Positive	36.42	36.49
12P				30.88	17.33	18.76	19.30	Positive	35.40	35.38				31.16	43.59	N/A	N/A	Negative	35.59	34.67			12P	30.53	16.46	17.89	18.58	Positive	36.42	36.49
13P				27.15	22.54	22.26	21.56	Positive	35.40	35.38				29.30	N/A	N/A	N/A	Negative	35.59	34.67			13P	27.19	22.65	22.55	22.27	Positive	36.42	36.49
14P				31.85	15.96	17.44	17.86	Positive	35.40	35.38				30.20	N/A	N/A	N/A	Negative	35.59	34.67			14P	31.05	16.85	18.43	19.18	Positive	36.42	36.49
15P				30.25	15.27	16.31	16.54	Positive	35.40	35.38				30.71	N/A	N/A	N/A	Negative	35.59	34.67			15P	30.34	16.42	18.03	18.30	Positive	36.42	36.49
16P				31.57	20.15	21.46	22.22	Positive	35.40	35.38				30.07	N/A	N/A	N/A	Negative	35.55	35.75			16P	30.95	19.36	20.91	21.34	Positive	35.97	34.87
17P				29.94	20.48	21.76	22.11	Positive	35.40	35.38				28.88	39.95	N/A	37.85	Negative	35.55	35.75			17P	29.95	20.58	22.05	22.22	Positive	35.97	34.87
18P				30.03	17.27	18.81	19.38	Positive	35.40	35.38				29.60	N/A	N/A	N/A	Negative	35.55	35.75			18P	30.35	18.37	20.15	20.58	Positive	35.97	34.87
19P				29.23	16.83	17.94	17.89	Positive	35.40	35.38				29.27	39.31	42.68	N/A	Negative	35.55	35.75			19P	30.37	17.99	19.47	19.71	Positive	35.97	34.87
20P				30.28	19.54	20.68	20.97	Positive	35.47	35.39				29.67	N/A	N/A	36.29	Negative	35.55	35.75			20P	30.39	20.47	21.37	21.57	Positive	35.97	34.87
21P				31.28	16.16	17.94	18.29	Positive	35.47	35.39				30.28	38.02	N/A	N/A	Negative	35.55	35.75			21P	29.13	17.74	19.55	19.75	Positive	35.97	34.87
22P				30.00	29.20	28.80	29.00	Positive	35.89	36.32				29.85	N/A	N/A	38.69	Negative	35.55	35.75			22P	30.81	29.40	29.74	29.99	Positive	35.97	34.87

Positives

A	B	C	D	E	F	G	H	I	J	K	L	M
	Ct_{RPP}			Ct_N			Ct_E			Ct_S		
**Pool name**	**Individual**	**Pool**	**Ct diffrence**	**Individual**	**Pool**	**Ct diffrence**	**Individual**	**Pool**	**Ct diffrence**	**Individual**	**Pool**	**Ct diffrence**
1P	29.62	29.94	0.32	13.39	14.52	1.13	14.94	16.19	1.24	15.31	16.66	1.34
2P	30.67	30.12	-0.55	17.24	18.21	0.97	17.99	19.05	1.06	17.77	18.82	1.05
3P	30.61	30.86	0.25	18.55	21.31	2.76	20.04	23.07	3.03	20.17	23.23	3.06
4P	31.02	30.11	-0.90	22.28	22.92	0.65	22.27	23.08	0.81	22.07	22.91	0.84
5P	31.22	31.23	0.01	17.15	18.43	1.29	18.22	19.50	1.27	18.54	19.91	1.37
6P	29.15	29.85	0.70	17.18	18.07	0.89	18.54	19.24	0.70	18.88	19.56	0.68
7P	29.99	31.25	1.26	17.05	18.92	1.87	19.17	20.93	1.76	19.53	21.63	2.10
8P	32.00	31.16	-0.84	22.00	22.81	0.81	23.00	24.44	1.44	23.30	24.68	1.38
9P	29.13	29.81	0.68	16.78	17.82	1.04	18.44	19.52	1.08	19.05	20.00	0.96
10P	29.02	28.40	-0.63	24.75	23.86	-0.89	25.29	24.85	-0.44	25.51	25.31	-0.20
11P	30.89	29.84	-1.05	15.28	15.23	-0.06	17.27	17.08	-0.20	17.78	17.49	-0.30
12P	30.88	30.53	-0.35	17.33	16.46	-0.87	18.76	17.89	-0.88	19.30	18.58	-0.71
13P	27.15	27.19	0.04	22.54	22.65	0.11	22.26	22.55	0.28	21.56	22.27	0.71
14P	31.85	31.05	-0.80	15.96	16.85	0.90	17.44	18.43	1.00	17.86	19.18	1.32
15P	30.25	30.34	0.08	15.27	16.42	1.16	16.31	18.03	1.72	16.54	18.30	1.76
16P	31.57	30.95	-0.62	20.15	19.36	-0.79	21.46	20.91	-0.55	22.22	21.34	-0.88
17P	29.94	29.95	0.02	20.48	20.58	0.10	21.76	22.05	0.29	22.11	22.22	0.11
18P	30.03	30.35	0.33	17.27	18.37	1.10	18.81	20.15	1.34	19.38	20.58	1.20
19P	29.23	30.37	1.14	16.83	17.99	1.15	17.94	19.47	1.54	17.89	19.71	1.82
20P	30.28	30.39	0.10	19.54	20.47	0.93	20.68	21.37	0.69	20.97	21.57	0.60
21P	31.28	29.13	-2.16	16.16	17.74	1.58	17.94	19.55	1.61	18.29	19.75	1.47
22P	30.00	30.81	0.81	29.20	29.40	0.20	28.80	29.74	0.94	29.00	29.99	0.99

Regression

A	B	C	D	E	F	G
	Ct_N		Ct_E		Ct_S	
**Pool name**	**Individual**	**Pool**	**Individual**	**Pool**	**Individual**	**Pool**
1P	13.39	14.52	14.94	16.19	15.31	16.66
2P	17.24	18.21	17.99	19.05	17.77	18.82
3P	18.55	21.31	20.04	23.07	20.17	23.23
4P	22.28	22.92	22.27	23.08	22.07	22.91
5P	17.15	18.43	18.22	19.50	18.54	19.91
6P	17.18	18.07	18.54	19.24	18.88	19.56
7P	17.05	18.92	19.17	20.93	19.53	21.63
8P	22.00	22.81	23.00	24.44	23.30	24.68
9P	16.78	17.82	18.44	19.52	19.05	20.00
10P	24.75	23.86	25.29	24.85	25.51	25.31
11P	15.28	15.23	17.27	17.08	17.78	17.49
12P	17.33	16.46	18.76	17.89	19.30	18.58
13P	22.54	22.65	22.26	22.55	21.56	22.27
14P	15.96	16.85	17.44	18.43	17.86	19.18
15P	15.27	16.42	16.31	18.03	16.54	18.30
16P	20.15	19.36	21.46	20.91	22.22	21.34
17P	20.48	20.58	21.76	22.05	22.11	22.22
18P	17.27	18.37	18.81	20.15	19.38	20.58
19P	16.83	17.99	17.94	19.47	17.89	19.71
20P	19.54	20.47	20.68	21.37	20.97	21.57
21P	16.16	17.74	17.94	19.55	18.29	19.75
22P	29.20	29.40	28.80	29.74	29.00	29.99

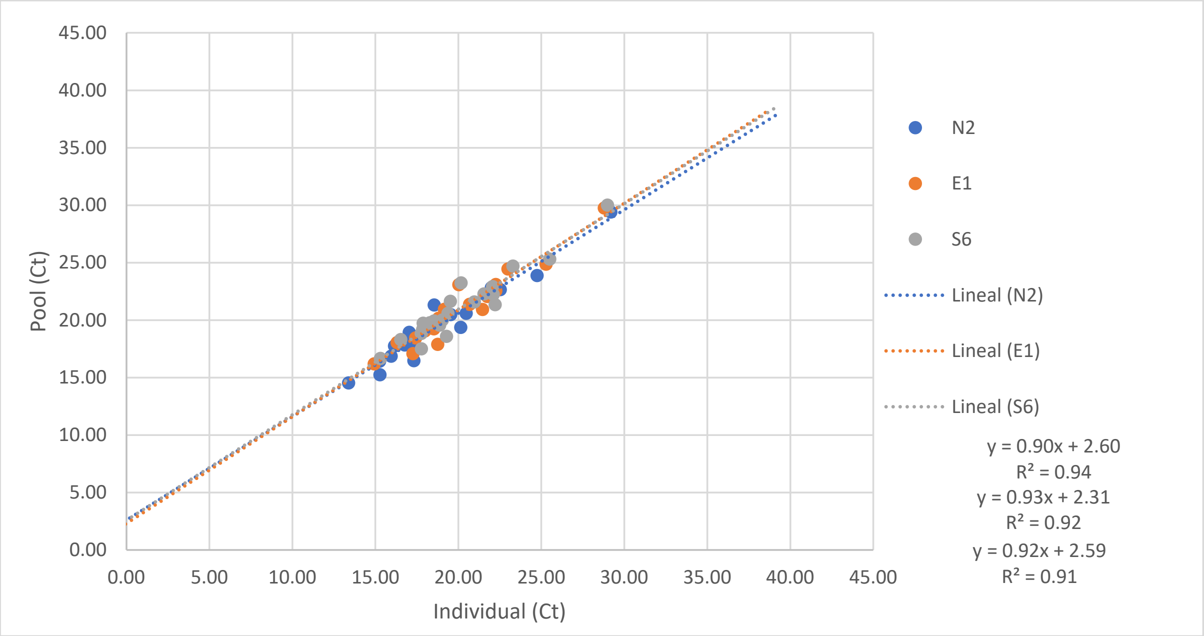

	Pool			Individual		
	LOD	95% CI low	95% CI up	LOD	95% CI low	95% CI upper
N	37.29	36.91	37.67	38.54	38.12	38.97
E	37.29	36.91	37.67	37.61	37.20	38.02
S	37.29	36.91	37.67	37.72	37.30	38.13

For a pool which is at the limit of detection, the individual sample would have had a Ct around 38.54 for N2 based on the regression analysis. Near the limit of detection, the regression analysis indicated this slight increase in Ct due to pooling when

n=2, this is likely due to little change in Cts due to pooling two samples.
