# Supplementary material for: Development and implementation of a scalable and versatile test for COVID-19 diagnostics in rural communities
Source: Nat Commun. 2021 Jul 20;12:4400. doi: 10.1038/s41467-021-24552-4 (PMC8292415; doi:10.1038/s41467-021-24552-4)
Supplement: Supplementary file 7 — Supplementary Data 5 [file 41467_2021_24552_MOESM7_ESM.pdf]

Pool raw data

B	C	D	E	F	G	H	I	J	K	L
	Sample 1									
Pool name	Original test date	Plate number	Sample	Ct_{RPP}	Ct_N	Ct_E	Ct_S	Result	cut-off N	cut-off RPP30
1N				29.49	40.66	N/A	N/A	Negative	35.59	34.67
2N				29.02	40.24	37.58	N/A	Negative	35.59	34.67
3N				29.13	N/A	N/A	N/A	Negative	35.59	34.67
4N				31.06	N/A	N/A	N/A	Negative	35.59	34.67
5N				30.59	N/A	N/A	N/A	Negative	35.59	34.67
6N				28.84	N/A	N/A	N/A	Negative	35.59	34.67
7N				30.09	N/A	N/A	N/A	Negative	35.59	34.67
8N				29.50	N/A	N/A	N/A	Negative	35.59	34.67
9N				30.05	N/A	N/A	N/A	Negative	35.59	34.67
10N				29.97	N/A	N/A	N/A	Negative	35.59	34.67
11N				29.37	N/A	N/A	43.98	Negative	35.60	35.06
12N				29.96	N/A	N/A	N/A	Negative	35.60	35.06
13N				31.63	N/A	N/A	N/A	Negative	35.60	35.06
14N				29.81	N/A	N/A	N/A	Negative	35.60	35.06
15N				28.87	N/A	N/A	N/A	Negative	35.60	35.06
16N				29.23	N/A	N/A	N/A	Negative	35.60	35.06
17N				29.97	N/A	N/A	N/A	Negative	35.60	35.06
18N				30.02	N/A	N/A	N/A	Negative	35.60	35.06
19N				29.96	N/A	N/A	N/A	Negative	35.60	35.06
20N				29.95	N/A	N/A	N/A	Negative	35.60	35.06
1P				29.62	13.39	14.94	15.31	Positive	35.87	36.02
2P				30.67	17.24	17.99	17.77	Positive	35.87	36.02
3P				30.61	18.55	20.04	20.17	Positive	35.87	36.02
4P				31.02	22.28	22.27	22.07	Positive	36.09	35.73
5P				31.22	17.15	18.22	18.54	Positive	36.09	35.73
6P				29.15	17.18	18.54	18.88	Positive	36.09	35.73
7P				29.99	17.05	19.17	19.53	Positive	35.97	36.32
8P				32.00	22.00	23.00	23.30	Positive	35.47	36.52
9P				29.13	16.78	18.44	19.05	Positive	36.14	36.22
10P				29.02	24.75	25.29	25.51	Positive	35.53	35.84
11P				30.89	15.28	17.27	17.78	Positive	35.88	34.58
12P				30.88	17.33	18.76	19.30	Positive	35.40	35.38
13P				27.15	22.54	22.26	21.56	Positive	35.40	35.38
14P				31.85	15.96	17.44	17.86	Positive	35.40	35.38
15P				30.25	15.27	16.31	16.54	Positive	35.40	35.38
16P				31.57	20.15	21.46	22.22	Positive	35.40	35.38
17P				29.94	20.48	21.76	22.11	Positive	35.40	35.38
18P				30.03	17.27	18.81	19.38	Positive	35.40	35.38
19P				29.23	16.83	17.94	17.89	Positive	35.40	35.38
20P				30.28	19.54	20.68	20.97	Positive	35.47	35.39
21P				31.28	16.16	17.94	18.29	Positive	35.47	35.39
22P				30.00	29.20	28.80	29.00	Positive	35.89	36.32

B	M	N	O	P	Q	R	S	T	U	V
	Sample 2									
Pool name	Original test date	Plate number	Sample	Ct_{RPP}	Ct_N	Ct_E	Ct_S	Result	cut-off N	cut-off RPP30
1N				28.76	37.60	N/A	N/A	Negative	35.81	35.08
2N				29.04	38.01	42.29	39.06	Negative	35.81	35.08
3N				31.13	N/A	N/A	N/A	Negative	35.81	35.08
4N				30.86	N/A	42.50	N/A	Negative	35.81	35.08
5N				30.76	N/A	N/A	38.68	Negative	35.81	35.08
6N				31.01	N/A	N/A	N/A	Negative	35.81	35.08
7N				31.47	N/A	N/A	N/A	Negative	35.81	35.08
8N				30.12	N/A	N/A	N/A	Negative	35.81	35.08
9N				31.92	N/A	N/A	N/A	Negative	35.81	35.08
10N				31.13	N/A	N/A	N/A	Negative	35.81	35.08
11N				30.20	N/A	N/A	N/A	Negative	35.60	35.06
12N				30.01	40.05	N/A	N/A	Negative	35.60	35.06
13N				30.16	N/A	N/A	N/A	Negative	35.60	35.06
14N				30.22	N/A	N/A	N/A	Negative	35.60	35.06
15N				29.46	N/A	N/A	37.69	Negative	35.60	35.06
16N				26.20	N/A	N/A	43.98	Negative	35.60	35.06
17N				29.48	36.31	N/A	N/A	Negative	35.60	35.06
18N				28.88	N/A	N/A	N/A	Negative	35.60	35.06
19N				29.63	N/A	N/A	N/A	Negative	35.60	35.06
20N				30.24	38.69	N/A	N/A	Negative	35.60	35.06
1P				29.20	N/A	N/A	N/A	Negative	35.81	35.08
2P				28.06	N/A	N/A	N/A	Negative	35.81	35.08
3P				29.21	N/A	N/A	44.93	Negative	35.81	35.08
4P				29.94	N/A	N/A	N/A	Negative	35.81	35.08
5P				28.76	37.60	N/A	N/A	Negative	35.81	35.08
6P				29.04	38.01	42.29	39.06	Negative	35.81	35.08
7P				30.11	N/A	N/A	N/A	Negative	35.81	35.08
8P				29.75	N/A	N/A	41.95	Negative	35.81	35.08
9P				29.36	N/A	N/A	N/A	Negative	35.81	35.08
10P				30.27	N/A	N/A	N/A	Negative	35.81	35.08
11P				31.13	N/A	N/A	N/A	Negative	35.81	35.08
12P				31.11	N/A	N/A	41.61	Negative	35.81	35.08
13P				30.86	N/A	42.50	N/A	Negative	35.81	35.08
14P				30.76	N/A	N/A	38.68	Negative	35.81	35.08
15P				31.01	N/A	N/A	N/A	Negative	35.81	35.08
16P				31.47	N/A	N/A	N/A	Negative	35.81	35.08
17P				30.12	N/A	N/A	N/A	Negative	35.81	35.08
18P				31.92	N/A	N/A	N/A	Negative	35.81	35.08
19P				31.07	N/A	N/A	N/A	Negative	35.81	35.08
20P				30.21	N/A	N/A	37.06	Negative	35.81	35.08
21P				30.75	N/A	N/A	N/A	Negative	35.81	35.08
22P				30.11	N/A	44.99	N/A	Negative	35.81	35.08

B	W	X	Y	Z	AA	AB	AC	AD	AE	AF
	Sample 3									
Pool name	Original test date	Plate number	Sample	Ct_{RPP}	Ct_N	Ct_E	Ct_S	Result	cut-off N	cut-off RPP30
1N				28.98	N/A	N/A	N/A	Negative	35.81	35.08
2N				30.07	N/A	43.27	44.51	Negative	35.81	35.08
3N				29.72	N/A	N/A	N/A	Negative	35.81	35.08
4N				29.47	41.01	N/A	N/A	Negative	35.81	35.08
5N				29.38	N/A	N/A	N/A	Negative	35.81	35.08
6N				28.99	41.86	N/A	N/A	Negative	35.81	35.08
7N				29.25	N/A	N/A	N/A	Negative	35.81	35.08
8N				29.48	N/A	N/A	N/A	Negative	35.81	35.08
9N				29.22	N/A	N/A	N/A	Negative	35.81	35.08
10N				29.72	N/A	N/A	N/A	Negative	35.59	34.67
11N				31.13	N/A	N/A	N/A	Negative	35.60	35.06
12N				29.75	N/A	N/A	N/A	Negative	35.60	35.06
13N				30.14	N/A	N/A	N/A	Negative	35.60	35.06
14N				30.18	44.59	N/A	N/A	Negative	35.60	35.06
15N				28.86	N/A	N/A	40.91	Negative	35.60	35.06
16N				30.26	N/A	N/A	N/A	Negative	35.60	35.06
17N				30.37	N/A	N/A	40.47	Negative	35.60	35.06
18N				29.55	N/A	N/A	N/A	Negative	35.60	35.06
19N				30.03	N/A	N/A	N/A	Negative	35.52	34.96
20N				32.18	N/A	N/A	N/A	Negative	35.52	34.96
1P				34.10	N/A	N/A	N/A	Negative	35.81	35.08
2P				33.04	N/A	N/A	N/A	Negative	35.81	35.08
3P				29.10	N/A	N/A	N/A	Negative	35.81	35.08
4P				29.38	N/A	N/A	N/A	Negative	35.81	35.08
5P				28.98	N/A	N/A	N/A	Negative	35.81	35.08
6P				30.07	N/A	43.27	44.51	Negative	35.81	35.08
7P				28.95	N/A	N/A	N/A	Negative	35.81	35.08
8P				28.93	N/A	N/A	44.70	Negative	35.81	35.08
9P				30.07	43.32	N/A	N/A	Negative	35.81	35.08
10P				29.38	N/A	N/A	38.52	Negative	35.81	35.08
11P				29.72	N/A	N/A	N/A	Negative	35.81	35.08
12P				28.55	N/A	40.18	41.41	Negative	35.81	35.08
13P				29.47	41.01	N/A	N/A	Negative	35.81	35.08
14P				29.38	N/A	N/A	N/A	Negative	35.81	35.08
15P				28.99	41.86	N/A	N/A	Negative	35.81	35.08
16P				29.25	N/A	N/A	N/A	Negative	35.81	35.08
17P				29.48	N/A	N/A	N/A	Negative	35.81	35.08
18P				29.22	N/A	N/A	N/A	Negative	35.81	35.08
19P				29.46	N/A	N/A	N/A	Negative	35.59	34.67
20P				30.79	N/A	N/A	N/A	Negative	35.59	34.67
21P				29.53	N/A	N/A	N/A	Negative	35.59	34.67
22P				29.96	N/A	N/A	42.90	Negative	35.59	34.67

B	AG	AH	AI	AJ	AK	AL	AM	AN	AO	AP
	Pool									
Pool name	Test date	Plate number	Sample	Ct_{RPP}	Ct_N	Ct_E	Ct_S	Result	cut-off N	cut-off RPP30
1N				29.40	37.25	N/A	N/A	Negative	36.08	35.95
2N				29.66	N/A	42.87	42.20	Negative	36.08	35.95
3N				29.64	37.13	N/A	36.76	Negative	36.08	35.95
4N				30.05	N/A	44.93	44.80	Negative	36.08	35.95
5N				29.65	37.90	N/A	N/A	Negative	36.08	35.95
6N				30.67	N/A	N/A	N/A	Negative	36.08	35.95
7N				31.04	37.52	N/A	N/A	Negative	36.08	35.95
8N				29.84	36.22	N/A	38.27	Negative	36.08	35.95
9N				29.79	N/A	N/A	N/A	Negative	36.10	34.84
10N				31.14	N/A	N/A	N/A	Negative	36.10	34.84
11N				29.83	N/A	N/A	40.18	Negative	36.15	35.56
12N				29.48	N/A	N/A	N/A	Negative	36.15	35.56
13N				30.46	N/A	N/A	N/A	Negative	36.15	35.56
14N				30.11	N/A	N/A	N/A	Negative	36.15	35.56
15N				29.32	N/A	N/A	N/A	Negative	36.15	35.56
16N				29.88	N/A	N/A	N/A	Negative	36.15	35.56
17N				29.78	N/A	N/A	N/A	Negative	36.15	35.56
18N				29.60	N/A	N/A	N/A	Negative	36.15	35.56
19N				30.03	N/A	N/A	N/A	Negative	36.15	35.56
20N				30.12	N/A	N/A	N/A	Negative	36.15	35.56
1P				29.66	15.21	16.09	16.89	Positive	36.08	35.95
2P				29.83	18.22	18.52	18.65	Positive	36.08	35.95
3P				31.34	21.62	22.81	23.21	Positive	36.08	35.95
4P				31.78	23.63	23.67	23.57	Positive	36.08	35.95
5P				30.01	18.84	19.88	20.23	Positive	36.08	35.95
6P				29.64	18.71	19.63	20.10	Positive	36.08	35.95
7P				30.74	18.52	20.06	20.94	Positive	36.08	35.95
8P				30.63	23.69	24.80	25.28	Positive	36.08	35.95
9P				29.30	18.04	19.19	20.01	Positive	36.08	35.95
10P				29.36	24.88	25.64	26.38	Positive	36.08	35.95
11P				30.88	16.00	17.49	18.67	Positive	36.08	35.95
12P				31.38	17.91	19.03	19.89	Positive	36.08	35.95
13P				28.31	23.54	23.50	23.32	Positive	36.08	35.95
14P				30.42	16.05	17.09	17.86	Positive	36.08	35.95
15P				30.23	17.07	18.10	18.75	Positive	36.08	35.95
16P				31.58	20.74	22.05	22.97	Positive	36.08	35.95
17P				30.17	21.70	22.80	23.48	Positive	36.08	35.95
18P				30.09	17.77	19.30	20.21	Positive	36.08	35.95
19P				30.47	17.97	19.10	19.44	Positive	36.08	35.95
20P				30.77	21.28	22.08	22.53	Positive	36.08	35.95
21P				31.06	18.31	20.25	20.70	Positive	35.19	34.96
22P				31.15	30.92	30.70	30.94	Positive	35.19	34.96

Positives

A	B	C	D	E	F	G	H	I	J	K	L	M
	Ct_{RPP}			Ct_N			Ct_E			Ct_S		
**Pool name**	**Individual**	**Pool**	**Ct diffrence**	**Individual**	**Pool**	**Ct diffrence**	**Individual**	**Pool**	**Ct diffrence**	**Individual**	**Pool**	**Ct diffrence**
1P	29.62	29.66	0.04	13.39	15.21	1.81	14.94	16.09	1.14	15.31	16.89	1.58
2P	30.67	29.83	-0.83	17.24	18.22	0.98	17.99	18.52	0.53	17.77	18.65	0.88
3P	30.61	31.34	0.73	18.55	21.62	3.07	20.04	22.81	2.77	20.17	23.21	3.04
4P	31.02	31.78	0.76	22.28	23.63	1.35	22.27	23.67	1.40	22.07	23.57	1.50
5P	31.22	30.01	-1.21	17.15	18.84	1.69	18.22	19.88	1.66	18.54	20.23	1.69
6P	29.15	29.64	0.49	17.18	18.71	1.53	18.54	19.63	1.09	18.88	20.10	1.22
7P	29.99	30.74	0.75	17.05	18.52	1.47	19.17	20.06	0.89	19.53	20.94	1.42
8P	32.00	30.63	-1.37	22.00	23.69	1.69	23.00	24.80	1.80	23.30	25.28	1.98
9P	29.13	29.30	0.17	16.78	18.04	1.26	18.44	19.19	0.75	19.05	20.01	0.96
10P	29.02	29.36	0.34	24.75	24.88	0.13	25.29	25.64	0.35	25.51	26.38	0.87
11P	30.89	30.88	-0.01	15.28	16.00	0.71	17.27	17.49	0.22	17.78	18.67	0.89
12P	30.88	31.38	0.50	17.33	17.91	0.58	18.76	19.03	0.27	19.30	19.89	0.59
13P	27.15	28.31	1.16	22.54	23.54	1.00	22.26	23.50	1.24	21.56	23.32	1.76
14P	31.85	30.42	-1.43	15.96	16.05	0.09	17.44	17.09	-0.34	17.86	17.86	0.00
15P	30.25	30.23	-0.02	15.27	17.07	1.80	16.31	18.10	1.79	16.54	18.75	2.21
16P	31.57	31.58	0.01	20.15	20.74	0.59	21.46	22.05	0.59	22.22	22.97	0.75
17P	29.94	30.17	0.24	20.48	21.70	1.21	21.76	22.80	1.04	22.11	23.48	1.37
18P	30.03	30.09	0.06	17.27	17.77	0.50	18.81	19.30	0.49	19.38	20.21	0.83
19P	29.23	30.47	1.24	16.83	17.97	1.13	17.94	19.10	1.16	17.89	19.44	1.55
20P	30.28	30.77	0.49	19.54	21.28	1.73	20.68	22.08	1.40	20.97	22.53	1.56
21P	31.28	31.06	-0.23	16.16	18.31	2.15	17.94	20.25	2.31	18.29	20.70	2.42
22P	30.00	31.15	1.15	29.20	30.92	1.72	28.80	30.70	1.90	29.00	30.94	1.94

Regression

A	B	C	D	E	F	G
	Ct_N		Ct_E		Ct_S	
**Pool name**	**Individual**	**Pool**	**Individual**	**Pool**	**Individual**	**Pool**
1P	13.39	15.21	14.94	16.09	15.31	16.89
2P	17.24	18.22	17.99	18.52	17.77	18.65
3P	18.55	21.62	20.04	22.81	20.17	23.21
4P	22.28	23.63	22.27	23.67	22.07	23.57
5P	17.15	18.84	18.22	19.88	18.54	20.23
6P	17.18	18.71	18.54	19.63	18.88	20.10
7P	17.05	18.52	19.17	20.06	19.53	20.94
8P	22.00	23.69	23.00	24.80	23.30	25.28
9P	16.78	18.04	18.44	19.19	19.05	20.01
10P	24.75	24.88	25.29	25.64	25.51	26.38
11P	15.28	16.00	17.27	17.49	17.78	18.67
12P	17.33	17.91	18.76	19.03	19.30	19.89
13P	22.54	23.54	22.26	23.50	21.56	23.32
14P	15.96	16.05	17.44	17.09	17.86	17.86
15P	15.27	17.07	16.31	18.10	16.54	18.75
16P	20.15	20.74	21.46	22.05	22.22	22.97
17P	20.48	21.70	21.76	22.80	22.11	23.48
18P	17.27	17.77	18.81	19.30	19.38	20.21
19P	16.83	17.97	17.94	19.10	17.89	19.44
20P	19.54	21.28	20.68	22.08	20.97	22.53
21P	16.16	18.31	17.94	20.25	18.29	20.70
22P	29.20	30.92	28.80	30.70	29.00	30.94

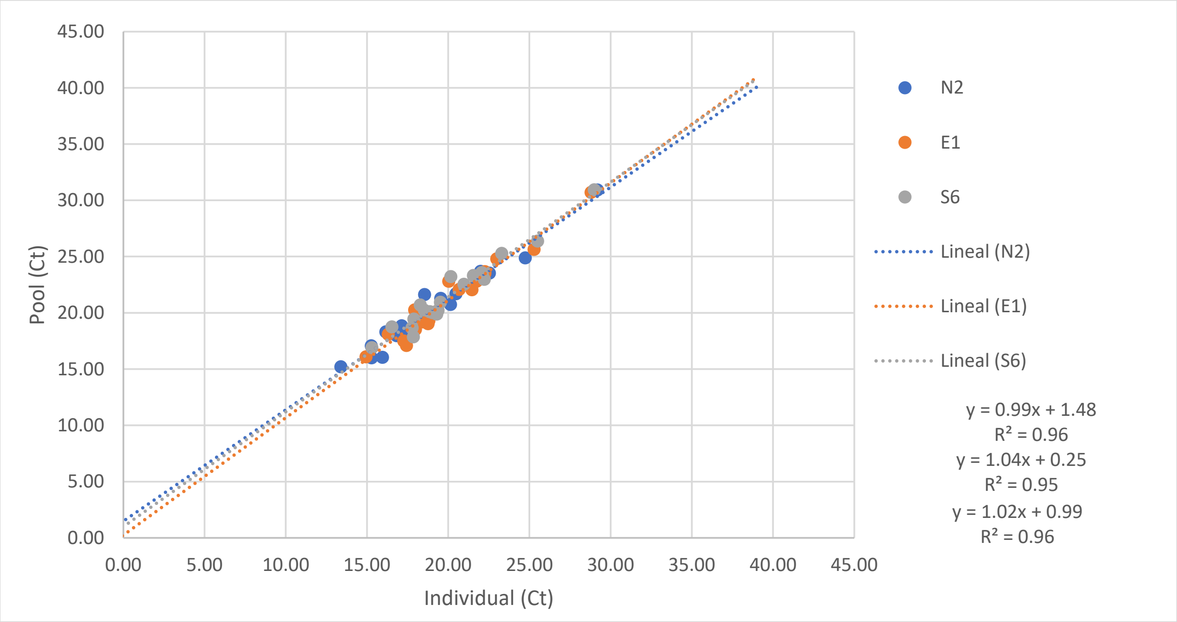

	Pool			Individual		
	LOD	95% CI low	95% CI upp	LOD	95% CI low	95% CI upper
N	37.29	36.91	37.67	36.17	35.79	36.56
E	37.29	36.91	37.67	35.62	35.25	35.98
S	37.29	36.91	37.67	35.59	35.22	35.96

For a pool which is at the limit of detection, the individual sample would have had a Ct around 36.17 for N2. This means that during the pooling procedure samples with Cts between 36.17 and 37.29 would have been detected using individual testing but will not be due to pooling.
