# Supplementary material for: Development and implementation of a scalable and versatile test for COVID-19 diagnostics in rural communities
Source: Nat Commun. 2021 Jul 20;12:4400. doi: 10.1038/s41467-021-24552-4 (PMC8292415; doi:10.1038/s41467-021-24552-4)
Supplement: Supplementary file 8 — Supplementary Data 6 [file 41467_2021_24552_MOESM8_ESM.pdf]

Pool_raw_data

A	B	CtRPP	D	E	F	G	H
	Sample 1						
**Pool name**	**Original test date**	**Ct_{RPP}**	**Ct_{N2}**	**Ct_{E1}**	**Ct_{S6}**	**cut-off N2**	**cut-off RPP30**
1N		29.65	N/A	N/A	N/A	34.78	35.37
2N		32.04	N/A	N/A	N/A	34.78	35.37
3N		29.14	N/A	N/A	N/A	34.78	35.37
4N		29.08	43.23	N/A	N/A	34.78	35.37
5N		28.40	N/A	43.83	43.58	34.78	35.37
6N		32.08	N/A	N/A	N/A	34.78	35.37
7N		29.17	N/A	N/A	N/A	34.86	34.75
8N		27.52	N/A	43.27	N/A	34.86	34.75
9N		30.52	N/A	N/A	N/A	34.86	34.75
10N		28.02	N/A	N/A	N/A	34.86	34.75
11N		27.38	N/A	N/A	N/A	34.78	35.37
12N		31.82	N/A	37.02	N/A	34.86	34.75
13N		30.12	N/A	N/A	41.78	34.78	35.37
14N		30.14	N/A	N/A	N/A	34.78	35.37
15N		30.59	N/A	N/A	N/A	34.86	34.75
16N		34.93	N/A	N/A	N/A	34.78	35.37
17N		28.97	N/A	N/A	N/A	34.86	34.75
18N		30.30	41.06	N/A	N/A	34.62	35.53
19N		28.61	N/A	N/A	40.87	34.83	36.97
20N		29.54	N/A	N/A	38.64	34.83	36.97
1P		27.99	25.93	28.19	28.31	34.85	36.50
2P		30.23	19.81	20.86	21.52	35.05	36.98
3P		30.50	20.67	20.84	20.54	35.45	36.50
4P		27.63	32.52	33.79	34.24	34.75	34.73
5P		28.43	15.32	16.49	16.58	34.82	38.17
6P		29.03	26.65	28.78	29.38	34.82	38.17
7P		29.10	21.48	23.29	23.16	35.36	37.96
8P		29.60	32.91	33.68	33.48	35.36	37.96
9P		28.41	21.68	22.80	22.99	35.36	37.96
10P		28.27	20.07	21.38	21.71	35.14	38.32
11P		29.45	21.49	23.53	24.17	35.49	38.34
12P		28.25	17.45	19.10	19.55	35.36	37.96
13P		28.56	20.85	21.81	21.73	34.72	37.90
14P		29.29	17.10	19.07	19.20	34.72	37.90
15P		28.49	23.06	24.23	24.17	34.72	37.90
16P		28.01	21.08	22.30	22.20	34.72	37.90
17P		27.86	22.61	24.66	24.47	34.72	37.90
18P		27.79	31.22	31.20	30.91	34.72	37.90
19P		27.81	33.73	32.82	33.39	33.76	38.43
20P		28.46	21.16	22.26	22.16	34.89	37.21
21P		27.06	33.26	33.12	32.61	37.16	38.01

A	I	J	K	L	M	N	O
	Sample 2						
**Pool name**	**Original test date**	**Ct_{RPP}**	**Ct_{N2}**	**Ct_{E1}**	**Ct_{S6}**	**cut-off N2**	**cut-off RPP30**
1N		30.66	N/A	N/A	N/A	34.78	35.37
2N		29.48	38.87	N/A	N/A	34.78	35.37
3N		30.17	44.61	N/A	N/A	34.78	35.37
4N		28.23	N/A	N/A	40.97	34.78	35.37
5N		29.67	N/A	N/A	40.24	34.78	35.37
6N		30.35	N/A	N/A	40.00	34.78	35.37
7N		30.81	N/A	N/A	N/A	34.86	34.75
8N		30.64	N/A	N/A	N/A	34.86	34.75
9N		30.88	N/A	N/A	N/A	34.86	34.75
10N		28.38	N/A	N/A	43.41	34.86	34.75
11N		31.39	N/A	N/A	N/A	34.86	34.75
12N		31.16	N/A	N/A	N/A	34.86	34.75
13N		28.47	39.42	N/A	N/A	34.78	35.37
14N		29.53	42.71	N/A	N/A	34.78	35.37
15N		28.02	N/A	N/A	42.06	34.57	35.53
16N		29.22	N/A	36.63	N/A	34.78	35.37
17N		28.22	N/A	N/A	41.10	34.86	34.75
18N		31.55	N/A	N/A	N/A	34.86	34.75
19N		29.38	41.11	N/A	N/A	34.83	36.97
20N		28.95	N/A	N/A	43.42	34.83	36.97
1P		30.66	N/A	N/A	N/A	34.83	36.97
2P		30.64	N/A	N/A	N/A	34.83	36.97
3P		28.38	N/A	N/A	43.41	34.83	36.97
4P		25.68	36.67	N/A	36.33	34.75	34.73
5P		26.46	41.00	41.41	40.55	34.82	38.17
6P		27.17	36.47	36.01	42.53	34.82	38.17
7P		29.99	N/A	N/A	N/A	35.36	37.96
8P		29.50	N/A	N/A	39.25	35.36	37.96
9P		28.99	N/A	N/A	37.58	35.36	37.96
10P		29.83	N/A	N/A	N/A	35.14	38.32
11P		27.67	43.23	N/A	N/A	35.49	38.34
12P		32.04	N/A	N/A	N/A	35.36	37.96
13P		28.70	37.18	N/A	N/A	34.72	37.90
14P		29.42	N/A	N/A	N/A	33.76	38.43
15P		27.28	38.62	N/A	37.70	34.89	37.21
16P		27.91	43.50	N/A	44.66	34.72	37.90
17P		27.56	N/A	42.57	41.58	34.72	37.90
18P		29.22	N/A	36.63	N/A	34.72	37.90
19P		28.22	N/A	N/A	41.10	33.76	38.43
20P		31.55	N/A	N/A	N/A	34.89	37.21
21P		27.56	N/A	N/A	41.85	37.16	38.01

A	P	Q	R	S	T	U	V
	Sample 3						
**Pool name**	**Original test date**	**Ct_{RPP}**	**Ct_{N2}**	**Ct_{E1}**	**Ct_{S6}**	**cut-off N2**	**cut-off RPP30**
1N		26.43	N/A	N/A	44.16	34.78	35.37
2N		27.93	39.49	N/A	42.52	34.78	35.37
3N		29.06	N/A	N/A	N/A	34.78	35.37
4N		31.01	N/A	N/A	N/A	34.78	35.37
5N		29.14	N/A	N/A	N/A	34.78	35.37
6N		29.44	38.40	40.34	N/A	34.78	35.37
7N		29.33	N/A	N/A	N/A	34.86	34.75
8N		27.81	N/A	N/A	40.70	34.86	34.75
9N		30.29	37.16	N/A	N/A	34.86	34.75
10N		29.17	N/A	N/A	N/A	34.86	34.75
11N		29.46	N/A	N/A	N/A	34.86	34.75
12N		30.27	N/A	N/A	39.80	34.86	34.75
13N		27.05	N/A	N/A	38.24	34.78	35.37
14N		28.44	N/A	N/A	44.75	34.78	35.37
15N		27.35	N/A	N/A	40.98	34.57	35.53
16N		27.68	N/A	N/A	43.37	34.78	35.37
17N		28.56	N/A	N/A	39.98	34.86	34.75
18N		29.71	36.96	N/A	N/A	34.86	34.75
19N		28.51	N/A	N/A	N/A	34.83	36.97
20N		31.05	N/A	39.29	N/A	34.83	36.97
1P		26.43	N/A	N/A	44.16	34.83	36.97
2P		27.81	N/A	N/A	40.70	34.83	36.97
3P		29.17	N/A	N/A	N/A	34.83	36.97
4P		27.20	36.60	44.43	44.24	34.75	34.73
5P		29.24	N/A	N/A	N/A	34.82	38.17
6P		27.35	N/A	N/A	38.54	34.82	38.17
7P		28.23	42.18	N/A	N/A	35.36	37.96
8P		28.65	35.15	37.22	44.27	35.36	37.96
9P		28.80	N/A	N/A	37.14	35.36	37.96
10P		28.76	N/A	N/A	N/A	35.14	38.32
11P		28.29	N/A	N/A	N/A	35.49	38.34
12P		27.98	N/A	N/A	40.87	35.36	37.96
13P		27.68	37.09	N/A	38.44	34.72	37.90
14P		28.22	N/A	N/A	41.53	33.76	38.43
15P		28.20	44.63	37.09	36.69	34.89	37.21
16P		27.89	N/A	N/A	42.15	34.72	37.90
17P		28.17	N/A	N/A	39.26	34.72	37.90
18P		27.68	N/A	N/A	43.37	34.72	37.90
19P		28.56	N/A	N/A	39.98	33.76	38.43
20P		29.71	36.96	N/A	N/A	34.89	37.21
21P		29.65	39.62	44.09	N/A	37.16	38.01

A	W	X	Y	Z	AA	AB	AC
	Sample 4						
**Pool name**	**Original test date**	**Ct_{RPP}**	**Ct_{N2}**	**Ct_{E1}**	**Ct_{S6}**	**cut-off N2**	**cut-off RPP30**
1N		23.62	N/A	N/A	41.22	34.78	35.37
2N		28.55	N/A	N/A	N/A	34.78	35.37
3N		29.13	N/A	N/A	37.27	34.78	35.37
4N		29.73	N/A	N/A	41.21	34.78	35.37
5N		29.15	N/A	N/A	38.58	34.78	35.37
6N		29.45	N/A	N/A	41.71	34.86	34.75
7N		30.38	N/A	37.10	39.72	34.86	34.75
8N		28.70	N/A	N/A	40.85	34.86	34.75
9N		31.64	N/A	N/A	N/A	34.86	34.75
10N		31.14	N/A	N/A	40.94	34.86	34.75
11N		29.62	36.24	37.70	41.67	34.86	34.75
12N		30.44	N/A	N/A	N/A	34.86	34.75
13N		28.80	N/A	N/A	44.72	34.78	35.37
14N		27.16	N/A	N/A	42.08	34.78	35.37
15N		28.56	44.14	N/A	N/A	34.57	35.53
16N		28.81	N/A	N/A	N/A	34.78	35.37
17N		28.42	N/A	N/A	N/A	34.86	34.75
18N		29.12	N/A	N/A	N/A	34.86	34.75
19N		29.18	N/A	N/A	44.22	34.83	36.97
20N		29.57	N/A	N/A	N/A	34.83	36.97
1P		23.62	N/A	N/A	41.22	34.83	36.97
2P		28.70	N/A	N/A	40.85	34.83	36.97
3P		31.14	N/A	N/A	40.94	34.83	36.97
4P		27.27	44.23	N/A	41.82	34.75	34.73
5P		27.30	42.96	44.99	41.84	34.82	38.17
6P		29.59	N/A	N/A	N/A	34.82	38.17
7P		27.83	N/A	42.17	37.90	35.36	37.96
8P		29.08	N/A	N/A	42.11	35.36	37.96
9P		30.19	N/A	N/A	43.22	35.36	37.96
10P		28.11	36.28	36.04	37.87	35.14	38.32
11P		29.39	37.08	N/A	N/A	35.49	38.34
12P		28.57	N/A	N/A	42.20	35.36	37.96
13P		28.85	N/A	N/A	N/A	34.72	37.90
14P		26.30	N/A	N/A	N/A	33.76	38.43
15P		28.32	41.72	N/A	42.70	34.89	37.21
16P		28.25	35.86	36.22	36.62	34.72	37.90
17P		27.19	N/A	N/A	39.64	34.72	37.90
18P		28.81	N/A	N/A	N/A	34.72	37.90
19P		28.42	N/A	N/A	N/A	33.76	38.43
20P		29.12	N/A	N/A	N/A	34.89	37.21
21P		29.24	N/A	40.00	44.69	37.16	38.01

A	AD	AE	AF	AG	AH	AI	AJ
	Pool						
**Pool name**	**Original test date**	**Ct_{RPP}**	**Ct_{N2}**	**Ct_{E1}**	**Ct_{S6}**	**cut-off N2**	**cut-off RPP30**
1N		27.49	27.49	N/A	N/A	35.45	36.50
2N		28.20	43.22	N/A	38.33	35.45	36.50
3N		28.99	N/A	N/A	N/A	35.45	36.50
4N		29.10	N/A	N/A	44.81	35.45	36.50
5N		28.54	N/A	N/A	37.38	35.45	36.50
6N		29.97	N/A	N/A	N/A	35.45	36.50
7N		29.28	38.82	N/A	N/A	35.45	36.50
8N		29.26	N/A	N/A	N/A	35.45	36.50
9N		29.84	N/A	N/A	40.30	35.45	36.50
10N		28.10	N/A	N/A	37.62	35.45	36.50
11N		28.37	N/A	N/A	43.20	35.45	36.50
12N		30.37	42.20	N/A	37.71	35.45	36.50
13N		28.10	N/A	N/A	N/A	35.45	36.50
14N		28.14	N/A	N/A	41.68	35.45	36.50
15N		27.48	N/A	N/A	38.61	35.45	36.50
16N		28.43	N/A	41.70	N/A	34.84	37.00
17N		28.13	N/A	N/A	N/A	34.84	37.00
18N		29.32	N/A	N/A	N/A	34.84	37.00
19N		28.64	N/A	N/A	42.16	34.84	37.00
20N		30.06	N/A	N/A	N/A	34.84	37.00
1P		27.66	28.73	30.42	30.70	35.45	36.50
2P		28.87	23.25	24.25	25.45	35.45	36.50
3P		29.01	20.31	20.61	20.46	35.45	36.50
4P		27.08	33.09	34.22	34.70	35.82	34.13
5P		28.82	18.01	18.74	18.38	35.82	34.13
6P		28.07	29.18	30.90	31.17	35.82	34.13
7P		29.04	23.68	25.06	24.96	35.82	34.13
8P		28.73	33.72	34.38	34.91	35.82	34.13
9P		28.09	23.05	24.41	24.19	34.22	34.24
10P		30.80	25.15	26.27	26.49	35.14	37.18
11P		30.11	24.67	26.74	27.12	35.14	37.18
12P		31.54	21.92	23.31	23.86	35.14	37.18
13P		29.62	24.14	24.72	24.63	35.14	37.18
14P		28.85	19.40	20.95	21.14	35.14	37.18
15P		30.08	26.01	27.06	27.09	35.14	37.18
16P		29.42	23.36	24.73	24.56	35.14	37.18
17P		29.61	28.27	30.14	30.08	35.14	37.18
18P		28.34	32.06	32.87	32.60	34.84	37.00
19P		28.20	35.50	35.10	36.11	34.84	37.00
20P		28.60	22.62	24.04	24.09	34.84	37.00
21P		28.06	35.91	35.93	34.74	34.84	37.00

Positives

A	B	C	D	E	F	G	H	I	J	K	L	M
	Ct_{RPP}			Ct_{N2}			Ct_{E1}			Ct_{S6}		
**Pool name**	**Individual**	**Pool**	**Ct diffrence**	**Individual**	**Pool**	**Ct diffrence**	**Individual**	**Pool**	**Ct diffrence**	**Individual**	**Pool**	**Ct diffrence**
1P	27.99	27.66	-0.33	25.93	28.73	2.80	28.19	30.42	2.23	28.31	30.70	2.39
2P	30.23	28.87	-1.36	19.81	23.25	3.43	20.86	24.25	3.39	21.52	25.45	3.93
3P	30.50	29.01	-1.50	20.67	20.31	-0.36	20.84	20.61	-0.22	20.54	20.46	-0.08
4P	27.63	27.08	-0.55	32.52	33.09	0.57	33.79	34.22	0.43	34.24	34.70	0.45
5P	28.43	28.82	0.39	15.32	18.01	2.69	16.49	18.74	2.25	16.58	18.38	1.79
6P	29.03	28.07	-0.95	26.65	29.18	2.52	28.78	30.90	2.12	29.38	31.17	1.79
7P	29.10	29.04	-0.05	21.48	23.68	2.20	23.29	25.06	1.77	23.16	24.96	1.80
8P	29.60	28.73	-0.88	32.91	33.72	0.81	33.68	34.38	0.70	33.48	34.91	1.43
9P	28.41	28.09	-0.31	21.68	23.05	1.37	22.80	24.41	1.61	22.99	24.19	1.20
10P	28.27	30.80	2.53	20.07	25.15	5.08	21.38	26.27	4.89	21.71	26.49	4.78
11P	29.45	30.11	0.66	21.49	24.67	3.17	23.53	26.74	3.21	24.17	27.12	2.95
12P	28.25	31.54	3.28	17.45	21.92	4.48	19.10	23.31	4.21	19.55	23.86	4.31
13P	28.56	29.62	1.06	20.85	24.14	3.29	21.81	24.72	2.90	21.73	24.63	2.90
14P	29.29	28.85	-0.44	17.10	19.40	2.31	19.07	20.95	1.88	19.20	21.14	1.95
15P	28.49	30.08	1.59	23.06	26.01	2.95	24.23	27.06	2.83	24.17	27.09	2.92
16P	28.01	29.42	1.41	21.08	23.36	2.28	22.30	24.73	2.43	22.20	24.56	2.35
17P	27.86	29.61	1.75	22.61	28.27	5.66	24.66	30.14	5.48	24.47	30.08	5.61
18P	27.79	28.34	0.54	31.22	32.06	0.84	31.20	32.87	1.67	30.91	32.60	1.70
19P	27.81	28.20	0.39	33.73	35.50	1.78	32.82	35.10	2.28	33.39	36.11	2.72
20P	28.46	28.60	0.13	21.16	22.62	1.46	22.26	24.04	1.78	22.16	24.09	1.93
21P	27.06	28.06	1.00	33.26	35.91	2.65	33.12	35.93	2.81	32.61	34.74	2.13

Regression

	Ct_N		Ct_E		Ct_S	
Pool name	Individual	Pool	Individual	Pool	Individual	Pool
1P	25.93	28.73	28.19	30.42	28.31	30.70
2P	19.81	23.25	20.86	24.25	21.52	25.45
3P	20.67	20.31	20.84	20.61	20.54	20.46
4P	32.52	33.09	33.79	34.22	34.24	34.70
5P	15.32	18.01	16.49	18.74	16.58	18.38
6P	26.65	29.18	28.78	30.90	29.38	31.17
7P	21.48	23.68	23.29	25.06	23.16	24.96
8P	32.91	33.72	33.68	34.38	33.48	34.91
9P	21.68	23.05	22.80	24.41	22.99	24.19
10P	20.07	25.15	21.38	26.27	21.71	26.49
11P	21.49	24.67	23.53	26.74	24.17	27.12
12P	17.45	21.92	19.10	23.31	19.55	23.86
13P	20.85	24.14	21.81	24.72	21.73	24.63
14P	17.10	19.40	19.07	20.95	19.20	21.14
15P	23.06	26.01	24.23	27.06	24.17	27.09
16P	21.08	23.36	22.30	24.73	22.20	24.56
17P	22.61	28.27	24.66	30.14	24.47	30.08
18P	31.22	32.06	31.20	32.87	30.91	32.60
19P	33.73	35.50	32.82	35.10	33.39	36.11
20P	21.16	22.62	22.26	24.04	22.16	24.09
21P	33.26	35.91	33.12	35.93	32.61	34.74

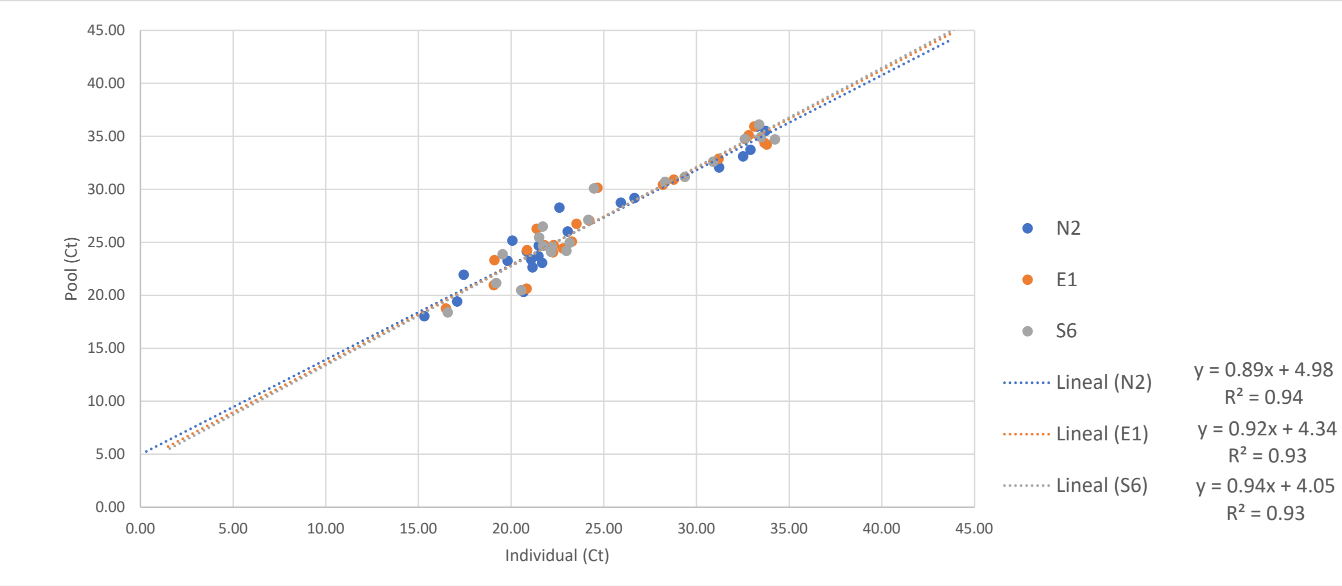

	Pool			Individual		
Gene	LOD	95% CI lower	95% CI upper	LOD	95% CI lower	95% CI upper
N	37.29	36.91	37.67	36.30	35.88	36.73
E	37.29	36.91	37.67	35.82	35.40	36.23
S	37.29	36.91	37.67	35.36	34.96	35.77

For a pool which is at the limit of detection, the individual sample would have had a Ct around 36.30 for N2. This means that during the pooling procedure samples with Cts between 36.30 and 37.29 would have been detected using individual testing but will not be due to pooling.
