# Supplementary material for: Development and implementation of a scalable and versatile test for COVID-19 diagnostics in rural communities
Source: Nat Commun. 2021 Jul 20;12:4400. doi: 10.1038/s41467-021-24552-4 (PMC8292415; doi:10.1038/s41467-021-24552-4)
Supplement: Supplementary file 9 — Supplementary Data 7 [file 41467_2021_24552_MOESM9_ESM.pdf]

Cts for FDA (n=4)

A	B	C	D	E	F	G	H	I	J	K	L	M	N	O	P	Q	R
Positive	Specimen code	Date qPCR run	Plate number	Ct_{RPP}	Ct_{N2}	Ct_{E1}	Ct_{S6}	cut-off N	cut-off RPP30	Theoretical Ct cutoff	Estimated CT cutoff	Theoretical Result N	Theoretical Result E	Theoretical Result S	Estimated Result N	Estimated Result E	Estimated Result S
1	V0010590			28.22	15.73	17.65	18.22	35.95	35.46	33.95	34.81	Positive	Positive	Positive	Positive	Positive	Positive
2	V0012870			29.06	17.38	18.63	19.21	35.95	35.46	33.95	34.81	Positive	Positive	Positive	Positive	Positive	Positive
3	V0014312			27.97	14.82	16.26	16.66	35.95	35.46	33.95	34.81	Positive	Positive	Positive	Positive	Positive	Positive
4	V0015270			28.23	23.00	23.36	23.08	35.95	35.46	33.95	34.81	Positive	Positive	Positive	Positive	Positive	Positive
5	V0015941			29.27	16.67	18.43	18.47	35.95	35.46	33.95	34.81	Positive	Positive	Positive	Positive	Positive	Positive
6	V0016364			27.74	36.70	33.60	35.19	35.45	35.37	33.45	34.24	Negative	Negative	Negative	Negative	Positive	Negative
7	258011			35.23	23.38	23.41	22.96	34.75	35.43	32.75	33.47	Positive	Positive	Positive	Positive	Positive	Positive
8	266249			29.77	24.76	25.29	24.76	34.75	35.43	32.75	33.47	Positive	Positive	Positive	Positive	Positive	Positive
9	V0016301			29.11	34.33	33.48	35.10	34.75	35.43	32.75	33.47	Negative	Negative	Negative	Negative	Negative	Negative
10	V0010606			28.22	30.24	30.20	30.03	35.48	35.63	33.48	34.28	Positive	Positive	Positive	Positive	Positive	Positive
11	V0015699			29.76	18.06	19.57	20.18	35.48	35.63	33.48	34.28	Positive	Positive	Positive	Positive	Positive	Positive
12	V0014597			30.07	18.06	19.20	20.00	35.26	36.36	33.26	34.03	Positive	Positive	Positive	Positive	Positive	Positive
13	V0015158			28.52	32.77	32.66	34.00	35.31	36.34	33.31	34.09	Positive	Positive	Negative	Positive	Positive	Positive
14	V0015163			29.45	31.35	30.63	31.00	35.31	36.34	33.31	34.09	Positive	Positive	Positive	Positive	Positive	Positive
15	V0010589			29.76	33.66	33.57	33.70	36.21	36.58	34.21	35.10	Positive	Positive	Positive	Positive	Positive	Positive
16	V0016168			29.38	34.07	33.15	33.75	35.15	35.61	33.15	33.91	Negative	Positive	Negative	Negative	Positive	Positive
17	V0014934			28.25	27.92	28.42	28.62	35.12	35.85	33.12	33.88	Positive	Positive	Positive	Positive	Positive	Positive
18	V0015461			29.25	33.51	32.90	33.58	35.12	35.85	33.12	33.88	Negative	Positive	Negative	Positive	Positive	Positive
19	V0014582			28.98	30.32	30.12	30.35	35.21	36.42	33.21	33.98	Positive	Positive	Positive	Positive	Positive	Positive
20	V0014587			27.31	34.65	34.36	33.93	35.21	36.42	33.21	33.98	Negative	Negative	Negative	Negative	Negative	Positive
21	V0015145			28.12	31.64	31.54	32.14	35.21	36.42	33.21	33.98	Positive	Positive	Positive	Positive	Positive	Positive
22	V0014584			28.19	21.33	23.24	24.14	36.07	35.23	34.07	34.94	Positive	Positive	Positive	Positive	Positive	Positive
23	V0014589			28.86	20.21	21.28	21.60	36.07	35.23	34.07	34.94	Positive	Positive	Positive	Positive	Positive	Positive
24	V0014595			29.05	18.88	20.66	21.64	36.07	35.23	34.07	34.94	Positive	Positive	Positive	Positive	Positive	Positive
25	V0014616			28.45	31.51	31.73	32.37	35.80	35.45	33.80	34.65	Positive	Positive	Positive	Positive	Positive	Positive
26	V0014903			28.57	32.77	33.42	34.04	35.80	35.45	33.80	34.65	Positive	Positive	Negative	Positive	Positive	Positive
27	V0014995			28.96	35.25	33.92	34.60	35.80	35.45	33.80	34.65	Negative	Negative	Negative	Negative	Positive	Positive
28	249008			29.30	21.14	22.74	23.00	34.38	36.43	32.38	33.04	Positive	Positive	Positive	Positive	Positive	Positive
29	V0014349			27.23	33.86	33.41	35.71	35.26	35.46	33.26	34.04	Negative	Negative	Negative	Positive	Positive	Negative
30	239215			29.84	18.68	19.70	20.25	36.19	36.27	34.19	35.08	Positive	Positive	Positive	Positive	Positive	Positive
31	V0014367			29.57	19.07	20.62	21.66	36.19	36.27	34.19	35.08	Positive	Positive	Positive	Positive	Positive	Positive
32	V0013598			30.79	20.50	22.21	22.95	36.16	36.06	34.16	35.04	Positive	Positive	Positive	Positive	Positive	Positive
33	V0013672			29.30	27.35	28.91	29.54	36.16	36.06	34.16	35.04	Positive	Positive	Positive	Positive	Positive	Positive
34	V0244808			32.05	31.01	32.18	33.34	35.95	35.40	33.95	34.81	Positive	Positive	Positive	Positive	Positive	Positive
35	V0249008			30.80	22.04	23.67	24.34	35.95	35.40	33.95	34.81	Positive	Positive	Positive	Positive	Positive	Positive
36	V0267038			30.37	16.99	19.28	19.82	35.95	35.40	33.95	34.81	Positive	Positive	Positive	Positive	Positive	Positive
37	V0014502			29.25	19.77	19.55	20.06	35.68	35.84	33.68	34.51	Positive	Positive	Positive	Positive	Positive	Positive
38	V0014521			30.47	25.08	24.55	24.81	35.68	35.84	33.68	34.51	Positive	Positive	Positive	Positive	Positive	Positive
39	V0013602			29.41	17.20	18.65	19.01	35.57	36.89	33.57	34.38	Positive	Positive	Positive	Positive	Positive	Positive
40	V0013659			29.99	13.49	16.05	16.61	35.80	36.81	33.80	34.64	Positive	Positive	Positive	Positive	Positive	Positive
41	V0013660			28.91	19.76	20.31	20.84	35.80	36.81	33.80	34.64	Positive	Positive	Positive	Positive	Positive	Positive
42	V0013691			28.96	24.06	24.09	24.1	35.14	36.38	33.14	33.90	Positive	Positive	Positive	Positive	Positive	Positive
43	V0013695			29.90	26.89	26.79	26.9	35.14	36.38	33.14	33.90	Positive	Positive	Positive	Positive	Positive	Positive
44	V0014147			27.70	24.30	24.99	25.18	36.02	36.37	34.02	34.89	Positive	Positive	Positive	Positive	Positive	Positive
45	V0011683			29.73	17.58	19.49	19.37	36.63	36.94	34.63	35.57	Positive	Positive	Positive	Positive	Positive	Positive
46	V0013950			27.19	19.63	20.55	20.56	36.63	36.94	34.63	35.57	Positive	Positive	Positive	Positive	Positive	Positive
47	V0014047			27.99	24.22	25.13	25.22	36.63	36.94	34.63	35.57	Positive	Positive	Positive	Positive	Positive	Positive
48	V0013209			28.73	35.81	35.48	35.51	36.18	35.46	34.18	35.07	Negative	Negative	Negative	Negative	Negative	Negative
49	V0014172			28.39	26.94	27.60	27.08	36.18	35.46	34.18	35.07	Positive	Positive	Positive	Positive	Positive	Positive
50	V0014208			28.28	22.75	23.47	23.50	36.18	35.46	34.18	35.07	Positive	Positive	Positive	Positive	Positive	Positive
51	V0011423			27.97	26.64	28.07	28.40	36.53	35.77	34.53	35.46	Positive	Positive	Positive	Positive	Positive	Positive
52	V0014057			28.89	21.48	22.35	22.57	36.53	35.77	34.53	35.46	Positive	Positive	Positive	Positive	Positive	Positive

53	V0011438	28.28	19.32	21.47	22.33	36.94	36.95	34.94	35.92	Positive	Positive	Positive	Positive	Positive	Positive
54	V0012919	27.95	22.28	22.81	22.95	36.94	36.95	34.94	35.92	Positive	Positive	Positive	Positive	Positive	Positive
55	V0014055	29.30	27.99	28.54	28.45	36.94	36.95	34.94	35.92	Positive	Positive	Positive	Positive	Positive	Positive
56	V0014061	28.23	26.56	27.50	27.86	36.94	36.95	34.94	35.92	Positive	Positive	Positive	Positive	Positive	Positive
57	V0012907	29.70	15.19	16.42	17.14	34.83	36.30	32.83	33.55	Positive	Positive	Positive	Positive	Positive	Positive
58	V0003662	29.15	33.54	34.72	35.70	34.83	36.30	32.83	33.55	Negative	Negative	Negative	Positive	Negative	Negative
59	V0012806	29.20	27.11	26.94	26.93	37.03	35.40	35.03	36.02	Positive	Positive	Positive	Positive	Positive	Positive
60	V0012116	29.97	35.21	36.56	36.21	37.03	35.40	35.03	36.02	Negative	Negative	Negative	Positive	Negative	Negative
61	V0012153	29.33	34.55	37.44	35.54	37.03	35.40	35.03	36.02	Positive	Negative	Negative	Positive	Negative	Positive
62	V0012513	32.64	35.03	35.09	36.12	36.60	36.96	34.60	35.54	Negative	Negative	Negative	Positive	Positive	Negative
63	V0014030	29.40	36.55	36.39	36.83	36.60	36.96	34.60	35.54	Negative	Negative	Negative	Negative	Negative	Negative
64	V0011709	28.99	34.29	34.75	36.70	36.60	36.96	34.60	35.54	Positive	Negative	Negative	Positive	Positive	Negative
65	V0012038	27.46	27.07	27.58	27.68	36.46	35.56	34.46	35.39	Positive	Positive	Positive	Positive	Positive	Positive
66	V0012076	30.36	20.23	21.60	21.85	36.46	35.56	34.46	35.39	Positive	Positive	Positive	Positive	Positive	Positive
67	V0013487	30.13	34.67	34.95	35.82	36.46	35.56	34.46	35.39	Negative	Negative	Negative	Positive	Positive	Negative
68	V0012539	32.13	36.04	36.52	36.30	36.37	36.90	34.37	35.28	Negative	Negative	Negative	Negative	Negative	Negative
69	V0013218	29.54	20.20	20.24	20.29	36.37	36.90	34.37	35.28	Positive	Positive	Positive	Positive	Positive	Positive
70	V0013385	28.72	27.71	27.52	27.85	36.37	36.90	34.37	35.28	Positive	Positive	Positive	Positive	Positive	Positive
71	V0013210	28.33	29.53	29.88	29.79	36.09	35.41	34.09	34.97	Positive	Positive	Positive	Positive	Positive	Positive
72	V0012016	28.18	25.03	23.84	23.99	36.04	36.13	34.04	34.91	Positive	Positive	Positive	Positive	Positive	Positive
73	V0012096	27.94	29.64	30.30	30.29	36.04	36.13	34.04	34.91	Positive	Positive	Positive	Positive	Positive	Positive
74	V0012100	28.21	25.32	25.51	25.89	36.04	36.13	34.04	34.91	Positive	Positive	Positive	Positive	Positive	Positive
75	V0012315	30.14	36.14	34.94	35.90	35.98	35.56	33.98	34.84	Negative	Negative	Negative	Negative	Negative	Negative
76	V0010757	28.60	16.49	17.23	17.09	36.11	36.43	34.11	34.99	Positive	Positive	Positive	Positive	Positive	Positive
77	V0010968	28.45	34.05	35.83	35.88	36.24	36.28	34.24	35.14	Positive	Negative	Negative	Positive	Negative	Negative
78	V0012065	29.89	35.11	34.55	36.57	36.24	36.28	34.24	35.14	Negative	Negative	Negative	Positive	Positive	Negative
79	V0012208	28.20	32.33	32.87	33.42	36.24	36.28	34.24	35.14	Positive	Positive	Positive	Positive	Positive	Positive
80	V0012303	30.40	18.75	20.23	20.66	34.72	36.43	32.72	33.43	Positive	Positive	Positive	Positive	Positive	Positive
81	V0012307	31.77	21.62	23.33	24.10	34.72	36.43	32.72	33.43	Positive	Positive	Positive	Positive	Positive	Positive
82	V0011361	30.28	34.42	36.63	35.55	35.76	35.17	33.76	34.59	Negative	Negative	Negative	Positive	Negative	Negative
83	V0012002	29.06	17.45	17.82	18.21	35.70	35.19	33.70	34.53	Positive	Positive	Positive	Positive	Positive	Positive
84	V0012305	29.46	23.81	25.37	26.50	35.70	35.19	33.70	34.53	Positive	Positive	Positive	Positive	Positive	Positive
85	V0012311	25.18	36.73	34.84	34.49	35.70	35.19	33.70	34.53	Negative	Negative	Negative	Negative	Negative	Positive
86	V0009459	28.97	35.91	35.35	35.86	35.89	35.44	33.89	34.75	Negative	Negative	Negative	Negative	Negative	Negative
87	V0010733	29.56	16.35	18.29	18.17	36.45	34.90	34.45	35.37	Positive	Positive	Positive	Positive	Positive	Positive
88	V0010756	29.71	34.39	37.02	36.20	36.45	34.90	34.45	35.37	Positive	Negative	Negative	Positive	Negative	Negative
89	V0011595	28.00	21.16	23.21	23.50	36.70	34.78	34.70	35.65	Positive	Positive	Positive	Positive	Positive	Positive
90	V0011727	28.48	27.96	29.20	29.03	36.70	34.78	34.70	35.65	Positive	Positive	Positive	Positive	Positive	Positive
91	V0011736	28.71	34.72	34.87	35.54	36.70	34.78	34.70	35.65	Negative	Negative	Negative	Positive	Positive	Positive
92	V0011057	27.14	34.89	35.73	35.67	36.12	36.22	34.12	35.00	Negative	Negative	Negative	Positive	Negative	Negative
93	V0011710	28.08	35.74	34.20	36.01	36.12	36.22	34.12	35.00	Negative	Negative	Negative	Negative	Positive	Negative
94	V0010958	27.83	37.29	37.23	45.00	37.33	36.09	35.33	36.36	Negative	Negative	Negative	Negative	Negative	Negative
95	V0011446	27.37	23.50	25.28	25.97	37.33	36.09	35.33	36.36	Positive	Positive	Positive	Positive	Positive	Positive
96	V0009529	28.67	33.80	36.04	36.63	37.33	36.09	35.33	36.36	Positive	Negative	Negative	Positive	Positive	Negative
97	V0009535	29.38	15.97	17.74	18.86	37.33	36.09	35.33	36.36	Positive	Positive	Positive	Positive	Positive	Positive
98	V0009548	28.25	36.10	37.26	37.60	37.33	36.09	35.33	36.36	Negative	Negative	Negative	Positive	Negative	Negative
99	V0012405	28.69	33.34	32.72	33.96	36.87	35.30	34.87	35.84	Positive	Positive	Positive	Positive	Positive	Positive
100	V0010641	26.97	33.80	34.16	33.61	35.84	35.60	33.84	34.68	Positive	Negative	Positive	Positive	Positive	Positive

Cts for FDA (n=3)

A	B	C	D	E	F	G	H	I	J	K	L	M	N	O	P	Q	R
Positive	Specimen code	Date qPCR run	Plate number	Ct_{RPP}	Ct_{N2}	Ct_{E1}	Ct_{S6}	cut-off N	cut-off RPP30	Theoretical Ct cutoff	Estimated CT cutoff	Theoretical Result N	Theoretical Result E	Theoretical Result S	Estimated Result N	Estimated Result E	Estimated Result S
1	V0010590			28.22	15.73	17.65	18.22	35.95	35.46	34.36	34.82	Positive	Positive	Positive	Positive	Positive	Positive
2	V0012870			29.06	17.38	18.63	19.21	35.95	35.46	34.36	34.82	Positive	Positive	Positive	Positive	Positive	Positive
3	V0014312			27.97	14.82	16.26	16.66	35.95	35.46	34.36	34.82	Positive	Positive	Positive	Positive	Positive	Positive
4	V0015270			28.23	23.00	23.36	23.08	35.95	35.46	34.36	34.82	Positive	Positive	Positive	Positive	Positive	Positive
5	V0015941			29.27	16.67	18.43	18.47	35.95	35.46	34.36	34.82	Positive	Positive	Positive	Positive	Positive	Positive
6	V0016364			27.74	36.70	33.60	35.19	35.45	35.37	33.86	34.31	Negative	Positive	Negative	Negative	Positive	Negative
7	258011			35.23	23.38	23.41	22.96	34.75	35.43	33.17	33.61	Positive	Positive	Positive	Positive	Positive	Positive
8	266249			29.77	24.76	25.29	24.76	34.75	35.43	33.17	33.61	Positive	Positive	Positive	Positive	Positive	Positive
9	V0016301			29.11	34.33	33.48	35.10	34.75	35.43	33.17	33.61	Negative	Negative	Negative	Negative	Positive	Negative
10	V0010606			28.22	30.24	30.20	30.03	35.48	35.63	33.90	34.35	Positive	Positive	Positive	Positive	Positive	Positive
11	V0015699			29.76	18.06	19.57	20.18	35.48	35.63	33.90	34.35	Positive	Positive	Positive	Positive	Positive	Positive
12	V0014597			30.07	18.06	19.20	20.00	35.26	36.36	33.67	34.12	Positive	Positive	Positive	Positive	Positive	Positive
13	V0015158			28.52	32.77	32.66	34.00	35.31	36.34	33.73	34.18	Positive	Positive	Negative	Positive	Positive	Positive
14	V0015163			29.45	31.35	30.63	31.00	35.31	36.34	33.73	34.18	Positive	Positive	Positive	Positive	Positive	Positive
15	V0010589			29.76	33.66	33.57	33.70	36.21	36.58	34.62	35.08	Positive	Positive	Positive	Positive	Positive	Positive
16	V0016168			29.38	34.07	33.15	33.75	35.15	35.61	33.56	34.01	Negative	Positive	Negative	Negative	Positive	Positive
17	V0014934			28.25	27.92	28.42	28.62	35.12	35.85	33.54	33.98	Positive	Positive	Positive	Positive	Positive	Positive
18	V0015461			29.25	33.51	32.90	33.58	35.12	35.85	33.54	33.98	Positive	Positive	Negative	Positive	Positive	Positive
19	V0014582			28.98	30.32	30.12	30.35	35.21	36.42	33.62	34.07	Positive	Positive	Positive	Positive	Positive	Positive
20	V0014587			27.31	34.65	34.36	33.93	35.21	36.42	33.62	34.07	Negative	Negative	Negative	Negative	Negative	Positive
21	V0015145			28.12	31.64	31.54	32.14	35.21	36.42	33.62	34.07	Positive	Positive	Positive	Positive	Positive	Positive
22	V0014584			28.19	21.33	23.24	24.14	36.07	35.23	34.48	34.94	Positive	Positive	Positive	Positive	Positive	Positive
23	V0014589			28.86	20.21	21.28	21.60	36.07	35.23	34.48	34.94	Positive	Positive	Positive	Positive	Positive	Positive
24	V0014595			29.05	18.88	20.66	21.64	36.07	35.23	34.48	34.94	Positive	Positive	Positive	Positive	Positive	Positive
25	V0014616			28.45	31.51	31.73	32.37	35.80	35.45	34.22	34.67	Positive	Positive	Positive	Positive	Positive	Positive
26	V0014903			28.57	32.77	33.42	34.04	35.80	35.45	34.22	34.67	Positive	Positive	Positive	Positive	Positive	Positive
27	V0014995			28.96	35.25	33.92	34.60	35.80	35.45	34.22	34.67	Negative	Positive	Negative	Negative	Positive	Positive
28	249008			29.30	21.14	22.74	23.00	34.38	36.43	32.79	33.23	Positive	Positive	Positive	Positive	Positive	Positive
29	V0014349			27.23	33.86	33.41	35.71	35.26	35.46	33.68	34.12	Negative	Positive	Negative	Positive	Positive	Negative
30	239215			29.84	18.68	19.70	20.25	36.19	36.27	34.60	35.06	Positive	Positive	Positive	Positive	Positive	Positive
31	V0014367			29.57	19.07	20.62	21.66	36.19	36.27	34.60	35.06	Positive	Positive	Positive	Positive	Positive	Positive
32	V0013598			30.79	20.50	22.21	22.95	36.16	36.06	34.57	35.03	Positive	Positive	Positive	Positive	Positive	Positive
33	V0013672			29.30	27.35	28.91	29.54	36.16	36.06	34.57	35.03	Positive	Positive	Positive	Positive	Positive	Positive
34	V0244808			32.05	31.01	32.18	33.34	35.95	35.40	34.37	34.82	Positive	Positive	Positive	Positive	Positive	Positive
35	V0249008			30.80	22.04	23.67	24.34	35.95	35.40	34.37	34.82	Positive	Positive	Positive	Positive	Positive	Positive
36	V0267038			30.37	16.99	19.28	19.82	35.95	35.40	34.37	34.82	Positive	Positive	Positive	Positive	Positive	Positive
37	V0014502			29.25	19.77	19.55	20.06	35.68	35.84	34.09	34.55	Positive	Positive	Positive	Positive	Positive	Positive
38	V0014521			30.47	25.08	24.55	24.81	35.68	35.84	34.09	34.55	Positive	Positive	Positive	Positive	Positive	Positive
39	V0013602			29.41	17.20	18.65	19.01	35.57	36.89	33.99	34.43	Positive	Positive	Positive	Positive	Positive	Positive
40	V0013659			29.99	13.49	16.05	16.61	35.80	36.81	34.21	34.66	Positive	Positive	Positive	Positive	Positive	Positive
41	V0013660			28.91	19.76	20.31	20.84	35.80	36.81	34.21	34.66	Positive	Positive	Positive	Positive	Positive	Positive
42	V0013691			28.96	24.06	24.09	24.1	35.14	36.38	33.55	34.00	Positive	Positive	Positive	Positive	Positive	Positive
43	V0013695			29.90	26.89	26.79	26.9	35.14	36.38	33.55	34.00	Positive	Positive	Positive	Positive	Positive	Positive
44	V0014147			27.70	24.30	24.99	25.18	36.02	36.37	34.44	34.89	Positive	Positive	Positive	Positive	Positive	Positive
45	V0011683			29.73	17.58	19.49	19.37	36.63	36.94	35.04	35.50	Positive	Positive	Positive	Positive	Positive	Positive
46	V0013950			27.19	19.63	20.55	20.56	36.63	36.94	35.04	35.50	Positive	Positive	Positive	Positive	Positive	Positive
47	V0014047			27.99	24.22	25.13	25.22	36.63	36.94	35.04	35.50	Positive	Positive	Positive	Positive	Positive	Positive
48	V0013209			28.73	35.81	35.48	35.51	36.18	35.46	34.60	35.05	Negative	Negative	Negative	Negative	Negative	Negative
49	V0014172			28.39	26.94	27.60	27.08	36.18	35.46	34.60	35.05	Positive	Positive	Positive	Positive	Positive	Positive
50	V0014208			28.28	22.75	23.47	23.50	36.18	35.46	34.60	35.05	Positive	Positive	Positive	Positive	Positive	Positive
51	V0011423			27.97	26.64	28.07	28.40	36.53	35.77	34.95	35.41	Positive	Positive	Positive	Positive	Positive	Positive
52	V0014057			28.89	21.48	22.35	22.57	36.53	35.77	34.95	35.41	Positive	Positive	Positive	Positive	Positive	Positive

53	V0011438	28.28	19.32	21.47	22.33	36.94	36.95	35.35	35.81	Positive	Positive	Positive	Positive	Positive	Positive
54	V0012919	27.95	22.28	22.81	22.95	36.94	36.95	35.35	35.81	Positive	Positive	Positive	Positive	Positive	Positive
55	V0014055	29.30	27.99	28.54	28.45	36.94	36.95	35.35	35.81	Positive	Positive	Positive	Positive	Positive	Positive
56	V0014061	28.23	26.56	27.50	27.86	36.94	36.95	35.35	35.81	Positive	Positive	Positive	Positive	Positive	Positive
57	V0012907	29.70	15.19	16.42	17.14	34.83	36.30	33.24	33.69	Positive	Positive	Positive	Positive	Positive	Positive
58	V0003662	29.15	33.54	34.72	35.70	34.83	36.30	33.24	33.69	Negative	Negative	Negative	Positive	Negative	Negative
59	V0012806	29.20	27.11	26.94	26.93	37.03	35.40	35.44	35.90	Positive	Positive	Positive	Positive	Positive	Positive
60	V0012116	29.97	35.21	36.56	36.21	37.03	35.40	35.44	35.90	Positive	Negative	Negative	Positive	Negative	Negative
61	V0012153	29.33	34.55	37.44	35.54	37.03	35.40	35.44	35.90	Positive	Negative	Negative	Positive	Negative	Positive
62	V0012513	32.64	35.03	35.09	36.12	36.60	36.96	35.02	35.48	Negative	Negative	Negative	Positive	Positive	Negative
63	V0014030	29.40	36.55	36.39	36.83	36.60	36.96	35.02	35.48	Negative	Negative	Negative	Negative	Negative	Negative
64	V0011709	28.99	34.29	34.75	36.70	36.60	36.96	35.02	35.48	Positive	Positive	Negative	Positive	Positive	Negative
65	V0012038	27.46	27.07	27.58	27.68	36.46	35.56	34.88	35.34	Positive	Positive	Positive	Positive	Positive	Positive
66	V0012076	30.36	20.23	21.60	21.85	36.46	35.56	34.88	35.34	Positive	Positive	Positive	Positive	Positive	Positive
67	V0013487	30.13	34.67	34.95	35.82	36.46	35.56	34.88	35.34	Positive	Negative	Negative	Positive	Positive	Negative
68	V0012539	32.13	36.04	36.52	36.30	36.37	36.90	34.78	35.24	Negative	Negative	Negative	Negative	Negative	Negative
69	V0013218	29.54	20.20	20.24	20.29	36.37	36.90	34.78	35.24	Positive	Positive	Positive	Positive	Positive	Positive
70	V0013385	28.72	27.71	27.52	27.85	36.37	36.90	34.78	35.24	Positive	Positive	Positive	Positive	Positive	Positive
71	V0013210	28.33	29.53	29.88	29.79	36.09	35.41	34.51	34.96	Positive	Positive	Positive	Positive	Positive	Positive
72	V0012016	28.18	25.03	23.84	23.99	36.04	36.13	34.46	34.91	Positive	Positive	Positive	Positive	Positive	Positive
73	V0012096	27.94	29.64	30.30	30.29	36.04	36.13	34.46	34.91	Positive	Positive	Positive	Positive	Positive	Positive
74	V0012100	28.21	25.32	25.51	25.89	36.04	36.13	34.46	34.91	Positive	Positive	Positive	Positive	Positive	Positive
75	V0012315	30.14	36.14	34.94	35.90	35.98	35.56	34.39	34.84	Negative	Negative	Negative	Negative	Negative	Negative
76	V0010757	28.60	16.49	17.23	17.09	36.11	36.43	34.52	34.98	Positive	Positive	Positive	Positive	Positive	Positive
77	V0010968	28.45	34.05	35.83	35.88	36.24	36.28	34.66	35.11	Positive	Negative	Negative	Positive	Negative	Negative
78	V0012065	29.89	35.11	34.55	36.57	36.24	36.28	34.66	35.11	Negative	Positive	Negative	Positive	Positive	Negative
79	V0012208	28.20	32.33	32.87	33.42	36.24	36.28	34.66	35.11	Positive	Positive	Positive	Positive	Positive	Positive
80	V0012303	30.40	18.75	20.23	20.66	34.72	36.43	33.14	33.58	Positive	Positive	Positive	Positive	Positive	Positive
81	V0012307	31.77	21.62	23.33	24.10	34.72	36.43	33.14	33.58	Positive	Positive	Positive	Positive	Positive	Positive
82	V0011361	30.28	34.42	36.63	35.55	35.76	35.17	34.17	34.63	Negative	Negative	Negative	Positive	Negative	Negative
83	V0012002	29.06	17.45	17.82	18.21	35.70	35.19	34.11	34.56	Positive	Positive	Positive	Positive	Positive	Positive
84	V0012305	29.46	23.81	25.37	26.50	35.70	35.19	34.11	34.56	Positive	Positive	Positive	Positive	Positive	Positive
85	V0012311	25.18	36.73	34.84	34.49	35.70	35.19	34.11	34.56	Negative	Negative	Negative	Negative	Negative	Positive
86	V0009459	28.97	35.91	35.35	35.86	35.89	35.44	34.31	34.76	Negative	Negative	Negative	Negative	Negative	Negative
87	V0010733	29.56	16.35	18.29	18.17	36.45	34.90	34.86	35.32	Positive	Positive	Positive	Positive	Positive	Positive
88	V0010756	29.71	34.39	37.02	36.20	36.45	34.90	34.86	35.32	Positive	Negative	Negative	Positive	Negative	Negative
89	V0011595	28.00	21.16	23.21	23.50	36.70	34.78	35.11	35.57	Positive	Positive	Positive	Positive	Positive	Positive
90	V0011727	28.48	27.96	29.20	29.03	36.70	34.78	35.11	35.57	Positive	Positive	Positive	Positive	Positive	Positive
91	V0011736	28.71	34.72	34.87	35.54	36.70	34.78	35.11	35.57	Positive	Positive	Negative	Positive	Positive	Positive
92	V0011057	27.14	34.89	35.73	35.67	36.12	36.22	34.53	34.99	Negative	Negative	Negative	Positive	Negative	Negative
93	V0011710	28.08	35.74	34.20	36.01	36.12	36.22	34.53	34.99	Negative	Positive	Negative	Negative	Positive	Negative
94	V0010958	27.83	37.29	37.23	45.00	37.33	36.09	35.75	36.21	Negative	Negative	Negative	Negative	Negative	Negative
95	V0011446	27.37	23.50	25.28	25.97	37.33	36.09	35.75	36.21	Positive	Positive	Positive	Positive	Positive	Positive
96	V0009529	28.67	33.80	36.04	36.63	37.33	36.09	35.75	36.21	Positive	Negative	Negative	Positive	Positive	Negative
97	V0009535	29.38	15.97	17.74	18.86	37.33	36.09	35.75	36.21	Positive	Positive	Positive	Positive	Positive	Positive
98	V0009548	28.25	36.10	37.26	37.60	37.33	36.09	35.75	36.21	Negative	Negative	Negative	Positive	Negative	Negative
99	V0012405	28.69	33.34	32.72	33.96	36.87	35.30	35.28	35.75	Positive	Positive	Positive	Positive	Positive	Positive
100	V0010641	26.97	33.80	34.16	33.61	35.84	35.60	34.25	34.71	Positive	Positive	Positive	Positive	Positive	Positive

Cts for FDA (n=2)

A	B	C	D	E	F	G	H	I	J	K	L	M	N	O	P	Q	R
**Positive**	**Specimen code**	**Date qPCR run**	**Plate number**	**Ct_{RPP}**	**Ct_{N2}**	**Ct_{E1}**	**Ct_{S6}**	**cut-off N**	**cut-off RPP30**	**Theoretical Ct cutoff**	**Estimated CT cutoff**	**Theoretical Result N**	**Theoretical Result E**	**Theoretical Result S**	**Estimated Result N**	**Estimated Result E**	**Estimated Result S**
1	V0010590			28.22	15.73	17.65	18.22	35.95	35.46	34.95	37.05	Positive	Positive	Positive	Positive	Positive	Positive
2	V0012870			29.06	17.38	18.63	19.21	35.95	35.46	34.95	37.05	Positive	Positive	Positive	Positive	Positive	Positive
3	V0014312			27.97	14.82	16.26	16.66	35.95	35.46	34.95	37.05	Positive	Positive	Positive	Positive	Positive	Positive
4	V0015270			28.23	23.00	23.36	23.08	35.95	35.46	34.95	37.05	Positive	Positive	Positive	Positive	Positive	Positive
5	V0015941			29.27	16.67	18.43	18.47	35.95	35.46	34.95	37.05	Positive	Positive	Positive	Positive	Positive	Positive
6	V0016364			27.74	36.70	33.60	35.19	35.45	35.37	34.45	36.50	Negative	Positive	Negative	Negative	Positive	Positive
7	###011			35.23	23.38	23.41	22.96	34.75	35.43	33.75	35.73	Positive	Positive	Positive	Positive	Positive	Positive
8	###249			29.77	24.76	25.29	24.76	34.75	35.43	33.75	35.73	Positive	Positive	Positive	Positive	Positive	Positive
9	V0016301			29.11	34.33	33.48	35.10	34.75	35.43	33.75	35.73	Negative	Positive	Negative	Positive	Positive	Positive
10	V0010606			28.22	30.24	30.20	30.03	35.48	35.63	34.48	36.54	Positive	Positive	Positive	Positive	Positive	Positive
11	V0015699			29.76	18.06	19.57	20.18	35.48	35.63	34.48	36.54	Positive	Positive	Positive	Positive	Positive	Positive
12	V0014597			30.07	18.06	19.20	20.00	35.26	36.36	34.26	36.29	Positive	Positive	Positive	Positive	Positive	Positive
13	V0015158			28.52	32.77	32.66	34.00	35.31	36.34	34.31	36.35	Positive	Positive	Positive	Positive	Positive	Positive
14	V0015163			29.45	31.35	30.63	31.00	35.31	36.34	34.31	36.35	Positive	Positive	Positive	Positive	Positive	Positive
15	V0010589			29.76	33.66	33.57	33.70	36.21	36.58	35.21	37.34	Positive	Positive	Positive	Positive	Positive	Positive
16	V0016168			29.38	34.07	33.15	33.75	35.15	35.61	34.15	36.16	Positive	Positive	Positive	Positive	Positive	Positive
17	V0014934			28.25	27.92	28.42	28.62	35.12	35.85	34.12	36.14	Positive	Positive	Positive	Positive	Positive	Positive
18	V0015461			29.25	33.51	32.90	33.58	35.12	35.85	34.12	36.14	Positive	Positive	Positive	Positive	Positive	Positive
19	V0014582			28.98	30.32	30.12	30.35	35.21	36.42	34.21	36.23	Positive	Positive	Positive	Positive	Positive	Positive
20	V0014587			27.31	34.65	34.36	33.93	35.21	36.42	34.21	36.23	Negative	Negative	Positive	Positive	Positive	Positive
21	V0015145			28.12	31.64	31.54	32.14	35.21	36.42	34.21	36.23	Positive	Positive	Positive	Positive	Positive	Positive
22	V0014584			28.19	21.33	23.24	24.14	36.07	35.23	35.07	37.19	Positive	Positive	Positive	Positive	Positive	Positive
23	V0014589			28.86	20.21	21.28	21.60	36.07	35.23	35.07	37.19	Positive	Positive	Positive	Positive	Positive	Positive
24	V0014595			29.05	18.88	20.66	21.64	36.07	35.23	35.07	37.19	Positive	Positive	Positive	Positive	Positive	Positive
25	V0014616			28.45	31.51	31.73	32.37	35.80	35.45	34.80	36.89	Positive	Positive	Positive	Positive	Positive	Positive
26	V0014903			28.57	32.77	33.42	34.04	35.80	35.45	34.80	36.89	Positive	Positive	Positive	Positive	Positive	Positive
27	V0014995			28.96	35.25	33.92	34.60	35.80	35.45	34.80	36.89	Negative	Positive	Positive	Positive	Positive	Positive
28	###008			29.30	21.14	22.74	23.00	34.38	36.43	33.38	35.31	Positive	Positive	Positive	Positive	Positive	Positive
29	V0014349			27.23	33.86	33.41	35.71	35.26	35.46	34.26	36.29	Positive	Positive	Negative	Positive	Positive	Positive
30	###215			29.84	18.68	19.70	20.25	36.19	36.27	35.19	37.32	Positive	Positive	Positive	Positive	Positive	Positive
31	V0014367			29.57	19.07	20.62	21.66	36.19	36.27	35.19	37.32	Positive	Positive	Positive	Positive	Positive	Positive
32	V0013598			30.79	20.50	22.21	22.95	36.16	36.06	35.16	37.29	Positive	Positive	Positive	Positive	Positive	Positive
33	V0013672			29.30	27.35	28.91	29.54	36.16	36.06	35.16	37.29	Positive	Positive	Positive	Positive	Positive	Positive
34	V0244808			32.05	31.01	32.18	33.34	35.95	35.40	34.95	37.06	Positive	Positive	Positive	Positive	Positive	Positive
35	V0249008			30.80	22.04	23.67	24.34	35.95	35.40	34.95	37.06	Positive	Positive	Positive	Positive	Positive	Positive
36	V0267038			30.37	16.99	19.28	19.82	35.95	35.40	34.95	37.06	Positive	Positive	Positive	Positive	Positive	Positive
37	V0014502			29.25	19.77	19.55	20.06	35.68	35.84	34.68	36.76	Positive	Positive	Positive	Positive	Positive	Positive
38	V0014521			30.47	25.08	24.55	24.81	35.68	35.84	34.68	36.76	Positive	Positive	Positive	Positive	Positive	Positive
39	V0013602			29.41	17.20	18.65	19.01	35.57	36.89	34.57	36.63	Positive	Positive	Positive	Positive	Positive	Positive
40	V0013659			29.99	13.49	16.05	16.61	35.80	36.81	34.80	36.89	Positive	Positive	Positive	Positive	Positive	Positive
41	V0013660			28.91	19.76	20.31	20.84	35.80	36.81	34.80	36.89	Positive	Positive	Positive	Positive	Positive	Positive
42	V0013691			28.96	24.06	24.09	24.1	35.14	36.38	34.14	36.15	Positive	Positive	Positive	Positive	Positive	Positive
43	V0013695			29.90	26.89	26.79	26.9	35.14	36.38	34.14	36.15	Positive	Positive	Positive	Positive	Positive	Positive
44	V0014147			27.70	24.30	24.99	25.18	36.02	36.37	35.02	37.14	Positive	Positive	Positive	Positive	Positive	Positive
45	V0011683			29.73	17.58	19.49	19.37	36.63	36.94	35.63	37.81	Positive	Positive	Positive	Positive	Positive	Positive
46	V0013950			27.19	19.63	20.55	20.56	36.63	36.94	35.63	37.81	Positive	Positive	Positive	Positive	Positive	Positive
47	V0014047			27.99	24.22	25.13	25.22	36.63	36.94	35.63	37.81	Positive	Positive	Positive	Positive	Positive	Positive
48	V0013209			28.73	35.81	35.48	35.51	36.18	35.46	35.18	37.32	Negative	Negative	Negative	Positive	Positive	Positive
49	V0014172			28.39	26.94	27.60	27.08	36.18	35.46	35.18	37.32	Positive	Positive	Positive	Positive	Positive	Positive
50	V0014208			28.28	22.75	23.47	23.50	36.18	35.46	35.18	37.32	Positive	Positive	Positive	Positive	Positive	Positive

51	V0011423	27.97	26.64	28.07	28.40	36.53	35.77	35.53	37.70	Positive	Positive	Positive	Positive	Positive	Positive
52	V0014057	28.89	21.48	22.35	22.57	36.53	35.77	35.53	37.70	Positive	Positive	Positive	Positive	Positive	Positive
53	V0011438	28.28	19.32	21.47	22.33	36.94	36.95	35.94	38.15	Positive	Positive	Positive	Positive	Positive	Positive
54	V0012919	27.95	22.28	22.81	22.95	36.94	36.95	35.94	38.15	Positive	Positive	Positive	Positive	Positive	Positive
55	V0014055	29.30	27.99	28.54	28.45	36.94	36.95	35.94	38.15	Positive	Positive	Positive	Positive	Positive	Positive
56	V0014061	28.23	26.56	27.50	27.86	36.94	36.95	35.94	38.15	Positive	Positive	Positive	Positive	Positive	Positive
57	V0012907	29.70	15.19	16.42	17.14	34.83	36.30	33.83	35.81	Positive	Positive	Positive	Positive	Positive	Positive
58	V0003662	29.15	33.54	34.72	35.70	34.83	36.30	33.83	35.81	Positive	Negative	Negative	Positive	Positive	Positive
59	V0012806	29.20	27.11	26.94	26.93	37.03	35.40	36.03	38.25	Positive	Positive	Positive	Positive	Positive	Positive
60	V0012116	29.97	35.21	36.56	36.21	37.03	35.40	36.03	38.25	Positive	Negative	Negative	Positive	Positive	Positive
61	V0012153	29.33	34.55	37.44	35.54	37.03	35.40	36.03	38.25	Positive	Negative	Positive	Positive	Positive	Positive
62	V0012513	32.64	35.03	35.09	36.12	36.60	36.96	35.60	37.78	Positive	Positive	Negative	Positive	Positive	Positive
63	V0014030	29.40	36.55	36.39	36.83	36.60	36.96	35.60	37.78	Negative	Negative	Negative	Positive	Positive	Positive
64	V0011709	28.99	34.29	34.75	36.70	36.60	36.96	35.60	37.78	Positive	Positive	Negative	Positive	Positive	Positive
65	V0012038	27.46	27.07	27.58	27.68	36.46	35.56	35.46	37.63	Positive	Positive	Positive	Positive	Positive	Positive
66	V0012076	30.36	20.23	21.60	21.85	36.46	35.56	35.46	37.63	Positive	Positive	Positive	Positive	Positive	Positive
67	V0013487	30.13	34.67	34.95	35.82	36.46	35.56	35.46	37.63	Positive	Positive	Negative	Positive	Positive	Positive
68	V0012539	32.13	36.04	36.52	36.30	36.37	36.90	35.37	37.52	Negative	Negative	Negative	Positive	Positive	Positive
69	V0013218	29.54	20.20	20.24	20.29	36.37	36.90	35.37	37.52	Positive	Positive	Positive	Positive	Positive	Positive
70	V0013385	28.72	27.71	27.52	27.85	36.37	36.90	35.37	37.52	Positive	Positive	Positive	Positive	Positive	Positive
71	V0013210	28.33	29.53	29.88	29.79	36.09	35.41	35.09	37.21	Positive	Positive	Positive	Positive	Positive	Positive
72	V0012016	28.18	25.03	23.84	23.99	36.04	36.13	35.04	37.16	Positive	Positive	Positive	Positive	Positive	Positive
73	V0012096	27.94	29.64	30.30	30.29	36.04	36.13	35.04	37.16	Positive	Positive	Positive	Positive	Positive	Positive
74	V0012100	28.21	25.32	25.51	25.89	36.04	36.13	35.04	37.16	Positive	Positive	Positive	Positive	Positive	Positive
75	V0012315	30.14	36.14	34.94	35.90	35.98	35.56	34.98	37.08	Negative	Positive	Negative	Positive	Positive	Positive
76	V0010757	28.60	16.49	17.23	17.09	36.11	36.43	35.11	37.23	Positive	Positive	Positive	Positive	Positive	Positive
77	V0010968	28.45	34.05	35.83	35.88	36.24	36.28	35.24	37.38	Positive	Negative	Negative	Positive	Positive	Positive
78	V0012065	29.89	35.11	34.55	36.57	36.24	36.28	35.24	37.38	Positive	Positive	Negative	Positive	Positive	Positive
79	V0012208	28.20	32.33	32.87	33.42	36.24	36.28	35.24	37.38	Positive	Positive	Positive	Positive	Positive	Positive
80	V0012303	30.40	18.75	20.23	20.66	34.72	36.43	33.72	35.69	Positive	Positive	Positive	Positive	Positive	Positive
81	V0012307	31.77	21.62	23.33	24.10	34.72	36.43	33.72	35.69	Positive	Positive	Positive	Positive	Positive	Positive
82	V0011361	30.28	34.42	36.63	35.55	35.76	35.17	34.76	36.84	Positive	Negative	Negative	Positive	Positive	Positive
83	V0012002	29.06	17.45	17.82	18.21	35.70	35.19	34.70	36.78	Positive	Positive	Positive	Positive	Positive	Positive
84	V0012305	29.46	23.81	25.37	26.50	35.70	35.19	34.70	36.78	Positive	Positive	Positive	Positive	Positive	Positive
85	V0012311	25.18	36.73	34.84	34.49	35.70	35.19	34.70	36.78	Negative	Negative	Positive	Positive	Positive	Positive
86	V0009459	28.97	35.91	35.35	35.86	35.89	35.44	34.89	36.99	Negative	Negative	Negative	Positive	Positive	Positive
87	V0010733	29.56	16.35	18.29	18.17	36.45	34.90	35.45	37.61	Positive	Positive	Positive	Positive	Positive	Positive
88	V0010756	29.71	34.39	37.02	36.20	36.45	34.90	35.45	37.61	Positive	Negative	Negative	Positive	Positive	Positive
89	V0011595	28.00	21.16	23.21	23.50	36.70	34.78	35.70	37.89	Positive	Positive	Positive	Positive	Positive	Positive
90	V0011727	28.48	27.96	29.20	29.03	36.70	34.78	35.70	37.89	Positive	Positive	Positive	Positive	Positive	Positive
91	V0011736	28.71	34.72	34.87	35.54	36.70	34.78	35.70	37.89	Positive	Positive	Positive	Positive	Positive	Positive
92	V0011057	27.14	34.89	35.73	35.67	36.12	36.22	35.12	37.24	Positive	Negative	Negative	Positive	Positive	Positive
93	V0011710	28.08	35.74	34.20	36.01	36.12	36.22	35.12	37.24	Negative	Positive	Negative	Positive	Positive	Positive
94	V0010958	27.83	37.29	37.23	45.00	37.33	36.09	36.33	38.59	Negative	Negative	Negative	Positive	Positive	Negative
95	V0011446	27.37	23.50	25.28	25.97	37.33	36.09	36.33	38.59	Positive	Positive	Positive	Positive	Positive	Positive
96	V0009529	28.67	33.80	36.04	36.63	37.33	36.09	36.33	38.59	Positive	Positive	Negative	Positive	Positive	Positive
97	V0009535	29.38	15.97	17.74	18.86	37.33	36.09	36.33	38.59	Positive	Positive	Positive	Positive	Positive	Positive
98	V0009548	28.25	36.10	37.26	37.60	37.33	36.09	36.33	38.59	Positive	Negative	Negative	Positive	Positive	Positive
99	V0012405	28.69	33.34	32.72	33.96	36.87	35.30	35.87	38.08	Positive	Positive	Positive	Positive	Positive	Positive
100	V0010641	26.97	33.80	34.16	33.61	35.84	35.60	34.84	36.93	Positive	Positive	Positive	Positive	Positive	Positive
