# Supplementary material for: Development and implementation of a scalable and versatile test for COVID-19 diagnostics in rural communities
Source: Nat Commun. 2021 Jul 20;12:4400. doi: 10.1038/s41467-021-24552-4 (PMC8292415; doi:10.1038/s41467-021-24552-4)
Supplement: Supplementary file 10 — Supplementary Data 8 [file 41467_2021_24552_MOESM10_ESM.pdf]

Saliva samples

A	B	C	D	E	F	G	H	I	J	K	L	M	N	O	P	Q	R	S	T	U	V	W
**Patient number**	**Swab VT ID**	**Swab date received**	**Swab plate number**	**Sw Ct_{RPP}**	**Sw Ct_N**	**Sw Ct_E**	**Sw Ct_S**	**Sw-RPP cutoff**	**Sw-N cutoff**	**Swab result**	**Saliva VT ID**	**Saliva date received**	**Saliva plate number**	**Sl Ct_{RPP}**	**Sl Ct_N**	**Sl Ct_E**	**Sl Ct_S**	**Sl-RPP cutoff**	**Sl-N cuttoff**	**Saliva result**	**Swab and Saliva match**	**Final call**
	V0008867			29.62	N/A	N/A	N/A	36.94	35.36	Negative	V0012691			29.93	N/A	N/A	44.43	36.94	35.36	Negative	true	True negative
	V0009268			28.60	41.33	N/A	41.85	36.94	35.36	Negative	V0012713			35.69	N/A	N/A	N/A	36.94	35.36	Negative	true	True negative
	V0009379			30.53	N/A	N/A	N/A	36.94	35.36	Negative	V0012726			34.98	N/A	N/A	N/A	36.94	35.36	Negative	true	True negative
	V0010862			28.46	N/A	N/A	38.63	36.94	35.36	Negative	V0012701			30.38	N/A	N/A	N/A	36.94	35.36	Negative	true	True negative
	V0010888			28.49	N/A	N/A	38.94	36.94	35.36	Negative	V0012707			30.64	37.43	N/A	41.53	36.94	35.36	Negative	true	True negative
	V0010750			30.04	36.63	36.73	36.17	38.28	35.38	Negative	V0012728			30.76	N/A	43.92	N/A	38.28	35.38	Negative	true	True negative
	V0011036			28.97	44.53	39.92	43.86	38.28	35.38	Negative	V0012689			34.96	N/A	N/A	N/A	37.60	35.92	Negative	true	True negative
	V0010899			29.34	N/A	N/A	N/A	37.60	35.92	Negative	V0012492			33.14	N/A	N/A	N/A	37.60	35.92	Negative	true	True negative
	V0011029			28.76	N/A	N/A	N/A	37.60	35.92	Negative	V0012685			36.11	N/A	N/A	N/A	37.60	35.92	Negative	true	True negative
	V0011039			28.91	N/A	N/A	38.45	37.60	35.92	Negative	V0012682			33.15	N/A	N/A	N/A	37.60	35.92	Negative	true	True negative
	V0011045			28.74	N/A	N/A	N/A	37.60	35.92	Negative	V0012725			32.57	43.35	N/A	42.64	37.60	35.92	Negative	true	True negative
	V0011054			27.90	N/A	42.16	39.60	37.60	35.92	Negative	V0012719			34.13	N/A	N/A	N/A	37.60	35.92	Negative	true	True negative
	V0011767			29.27	N/A	N/A	N/A	37.60	35.92	Negative	V0012489			32.37	N/A	N/A	N/A	37.60	35.92	Negative	true	True negative
	V0012985			29.12	37.91	N/A	N/A	36.22	36.12	Negative	V0012720			32.44	N/A	N/A	N/A	36.13	36.95	Negative	true	True negative
	V0011484			28.79	N/A	N/A	N/A	39.12	37.98	Negative	V0012490			32.08	N/A	N/A	N/A	39.12	37.98	Negative	true	True negative
	V0010674			28.13	43.35	N/A	43.10	37.30	37.12	Negative	V0012487			38.06	N/A	N/A	N/A	37.30	37.12	Negative	true	True negative
	V0011742			28.20	44.75	N/A	N/A	37.30	37.12	Negative	V0012493			32.66	42.60	41.67	N/A	37.30	37.12	Negative	true	True negative
	V0010710			26.69	N/A	N/A	N/A	37.30	37.12	Negative	V0012496			35.36	N/A	N/A	42.02	37.30	37.12	Negative	true	True negative
	V0010702			28.03	N/A	N/A	41.83	37.30	37.12	Negative	V0012575			31.87	41.56	41.20	N/A	37.30	37.12	Negative	true	True negative
	V0011658			28.37	N/A	38.07	N/A	37.30	37.12	Negative	V0012683			32.42	N/A	N/A	N/A	37.30	37.12	Negative	true	True negative
	V0010644			29.18	N/A	N/A	43.52	37.30	37.12	Negative	V0012690			30.53	N/A	N/A	43.73	37.30	37.12	Negative	true	True negative
	V0013010			28.21	43.26	N/A	37.98	34.78	36.70	Negative	V0012579			34.78	N/A	N/A	N/A	35.17	35.76	Negative	true	True negative
	V0010762			29.17	N/A	43.66	N/A	36.09	37.33	Negative	V0012574			29.33	N/A	39.45	38.15	35.17	35.76	Negative	true	True negative
	V0011433			28.97	40.69	39.36	42.44	36.09	37.33	Negative	V0012518			31.47	N/A	N/A	43.68	35.17	35.76	Negative	true	True negative
	V0011650			31.02	N/A	N/A	N/A	35.44	35.89	Negative	V0012521			32.85	N/A	N/A	N/A	35.17	35.76	Negative	true	True negative
	V0011678			29.09	42.88	42.01	N/A	36.22	36.12	Negative	V0012567			34.98	N/A	N/A	N/A	35.17	35.76	Negative	true	True negative
	V0013241			28.14	39.25	N/A	40.34	34.89	35.60	Negative	V0012532			34.64	N/A	N/A	N/A	34.89	35.60	Negative	true	True negative
	V0012786			28.25	N/A	N/A	42.23	36.30	34.83	Negative	V0012540			34.40	N/A	N/A	N/A	36.91	35.28	Negative	true	True negative
	V0012926			29.32	N/A	N/A	N/A	36.30	34.83	Negative	V0012537			36.21	N/A	N/A	N/A	36.91	35.28	Negative	true	True negative
	V0012921			28.25	N/A	N/A	44.53	36.30	34.83	Negative	V0012511			32.21	N/A	N/A	N/A	36.91	35.28	Negative	true	True negative
	V0013398			28.26	N/A	N/A	41.01	36.90	36.37	Negative	V0012500			29.79	N/A	N/A	N/A	36.91	35.28	Negative	true	True negative
	V0013014			28.99	N/A	N/A	N/A	36.95	36.94	Negative	V0012556			34.46	N/A	N/A	N/A	35.83	36.17	Negative	true	True negative
	V0014305			29.01	N/A	N/A	N/A	36.95	36.94	Negative	V0012555			34.12	N/A	N/A	N/A	35.83	36.17	Negative	true	True negative
	V0013231			27.38	39.57	38.67	39.17	36.95	36.94	Negative	V0010599			33.16	N/A	N/A	N/A	35.83	36.17	Negative	true	True negative
	V0013204			29.18	N/A	N/A	N/A	35.77	36.53	Negative	V0012550			34.94	N/A	N/A	N/A	35.83	36.17	Negative	true	True negative
	V0013948			27.53	N/A	N/A	N/A	36.95	36.94	Negative	V0012531			31.28	N/A	37.99	N/A	37.06	35.68	Negative	true	True negative
	V0014222			27.04	37.62	N/A	41.66	36.95	36.94	Negative	V0012495			31.73	N/A	44.85	41.52	37.06	35.68	Negative	true	True negative
	V0014249			28.19	39.93	N/A	43.67	35.77	36.53	Negative	V0012508			33.84	N/A	N/A	N/A	37.06	35.68	Negative	true	True negative
	V0014118			27.31	N/A	N/A	N/A	35.77	36.53	Negative	V0012524			31.10	N/A	N/A	N/A	37.06	35.68	Negative	true	True negative
	V0014227			27.88	43.83	N/A	N/A	35.77	36.53	Negative	V0012560			35.77	37.27	N/A	N/A	37.06	35.68	Negative	true	True negative
	V0013205			29.39	N/A	N/A	N/A	35.77	36.53	Negative	V0012565			31.81	N/A	39.16	N/A	37.06	35.68	Negative	true	True negative
	V0014243			27.88	43.07	N/A	43.56	36.95	36.94	Negative	V0012534			28.86	N/A	N/A	N/A	37.06	35.68	Negative	true	True negative
	V0014177			29.15	N/A	N/A	42.86	36.95	36.94	Negative	V0012563			29.85	N/A	N/A	N/A	37.06	35.68	Negative	true	True negative
	V0014221			29.22	N/A	N/A	40.19	36.95	36.94	Negative	V0012519			31.50	N/A	38.10	N/A	37.06	35.68	Negative	true	True negative
	V0014341			28.22	N/A	N/A	38.89	36.95	36.94	Negative	V0012549			31.26	N/A	N/A	N/A	37.06	35.68	Negative	true	True negative
	V0013980			28.42	43.10	44.80	N/A	35.46	36.18	Negative	V0012498			34.32	N/A	N/A	38.65	37.06	35.68	Negative	true	True negative
	V0014256			27.56	42.38	N/A	42.74	36.95	36.94	Negative	V0012559			33.44	N/A	N/A	N/A	37.06	35.68	Negative	true	True negative
	V0010569			28.56	N/A	41.00	43.48	36.95	36.94	Negative	V0010597			31.21	N/A	N/A	N/A	37.06	35.68	Negative	true	True negative
	V0014168			26.07	44.53	N/A	N/A	35.77	36.53	Negative	V0012497			30.33	N/A	N/A	N/A	37.06	35.68	Negative	true	True negative
	V0014258			29.34	43.46	N/A	38.76	35.77	36.53	Negative	V0012527			30.15	N/A	N/A	N/A	37.06	35.68	Negative	true	True negative
	V0014022			30.42	41.57	N/A	N/A	35.77	36.53	Negative	V0012514			35.05	N/A	N/A	37.47	37.06	35.68	Negative	true	True negative
	V0014174			28.14	N/A	N/A	N/A	35.77	36.53	Negative	V0012516			32.72	N/A	N/A	39.05	37.06	35.68	Negative	true	True negative
	V0012894			27.49	N/A	42.45	N/A	35.37	35.45	Negative	V0012552			32.39	N/A	N/A	N/A	35.37	35.45	Negative	true	True negative
	V0015894			27.65	N/A	N/A	N/A	36.42	34.78	Negative	V0012485			34.40	N/A	N/A	N/A	36.42	34.78	Negative	true	True negative
	V0015689			29.92	N/A	N/A	N/A	36.42	34.78	Negative	V0012545			31.33	N/A	N/A	N/A	36.42	34.78	Negative	true	True negative
	V0015939			29.46	N/A	39.31	N/A	36.42	34.78	Negative	V0012551			38.14	N/A	N/A	N/A	36.42	34.78	Negative	true	True negative
	V0015700			28.72	37.21	N/A	N/A	35.46	35.95	Negative	V0012568			31.09	N/A	N/A	N/A	36.42	34.78	Negative	true	True negative
	V0015931			29.33	37.19	N/A	N/A	35.46	35.95	Negative	V0012488			34.00	N/A	N/A	N/A	36.10	36.36	Negative	true	True negative
	V0015917			28.31	N/A	N/A	N/A	36.42	34.78	Negative	V0012491			33.63	N/A	N/A	N/A	36.10	36.36	Negative	true	True negative

	V0015298			28.77	37.21	36.07	N/A	35.46	35.95	Negative	V0012501			32.64	N/A	N/A	N/A	36.10	36.36	Negative	true	True negative
	V0015687			32.34	N/A	N/A	N/A	35.46	35.95	Negative	V0012572			30.97	N/A	N/A	N/A	36.10	36.36	Negative	true	True negative
	V0010574			26.68	40.65	N/A	42.30	36.42	34.78	Negative	V0012573			35.09	N/A	N/A	N/A	36.10	36.36	Negative	true	True negative
	V0015982			29.10	N/A	N/A	41.40	36.20	36.35	Negative	V0012576			33.84	N/A	N/A	N/A	36.10	36.36	Negative	true	True negative
	V0016413			29.20	N/A	N/A	38.00	37.03	36.52	Negative	V0016982			32.90	N/A	N/A	N/A	36.39	37.05	Negative	true	True negative
	V0016407			29.90	N/A	37.10	37.70	37.03	36.52	Negative	V0016984			35.00	N/A	N/A	N/A	36.39	37.05	Negative	true	True negative
	V0016754			29.01	N/A	N/A	37.68	36.95	35.95	Negative	V0016986			34.70	N/A	N/A	N/A	36.39	37.05	Negative	true	True negative
	V0016764			29.07	N/A	N/A	N/A	36.39	37.05	Negative	V0016991			32.10	N/A	N/A	N/A	36.39	37.05	Negative	true	True negative
	V0015373			28.90	N/A	N/A	N/A	37.03	36.52	Negative	V0017001			31.30	N/A	N/A	N/A	36.39	37.05	Negative	true	True negative
	V0016400			29.30	N/A	N/A	N/A	37.03	36.52	Negative	V0017011			30.10	N/A	N/A	N/A	36.39	37.05	Negative	true	True negative
	V0015694			28.80	N/A	N/A	N/A	37.03	36.52	Negative	V0017014			31.90	N/A	N/A	N/A	36.39	37.05	Negative	true	True negative
	V0015820			28.82	36.22	36.51	N/A	34.85	35.12	Negative	V0016792			34.04	N/A	N/A	N/A	37.78	36.02	Negative	true	True negative
	V0015407			28.39	N/A	N/A	N/A	35.41	36.85	Negative	V0016806			37.55	N/A	N/A	N/A	37.78	36.02	Negative	true	True negative
	V0009668			30.19	N/A	N/A	N/A	37.03	36.11	Negative	V0016820			33.40	N/A	N/A	N/A	37.78	36.02	Negative	true	True negative
	V0015406			29.07	N/A	N/A	38.14	35.70	36.11	Negative	V0016782			33.98	36.52	37.58	36.25	35.54	35.88	Negative	true	True negative
	V0016864			29.26	N/A	N/A	N/A	35.91	36.21	Negative	V0016987			32.20	45.00	44.60	45.00	36.46	36.01	Negative	true	True negative
	V0015380			27.13	N/A	N/A	N/A	36.76	35.76	Negative	V0016988			32.70	45.00	45.00	45.00	36.46	36.01	Negative	true	true negative
	V0015378			29.19	N/A	N/A	N/A	37.51	36.06	Negative	V0016993			34.80	45.00	45.00	45.00	36.46	36.01	Negative	true	True negative
	V0017052			28.90	N/A	N/A	37.42	36.76	35.76	Negative	V0016994			31.10	45.00	45.00	45.00	36.46	36.01	Negative	true	True negative
	V0015374			29.14	N/A	N/A	N/A	36.76	35.76	Negative	V0016998			32.50	45.00	45.00	45.00	36.46	36.01	Negative	true	True negative
	V0016877			30.27	N/A	N/A	N/A	36.76	35.76	Negative	V0017000			32.30	45.00	41.20	45.00	36.46	36.01	Negative	true	True negative
	V0017193			28.28	N/A	N/A	N/A	36.76	35.76	Negative	V0017009			32.40	45.00	45.00	45.00	36.46	36.01	Negative	true	True negative
	V0017194			27.28	N/A	N/A	38.31	36.76	35.76	Negative	V0017012			32.80	45.00	45.00	40.30	36.46	36.01	Negative	true	True negative
	V0016791			28.05	N/A	N/A	44.88	36.76	35.76	Negative	V0017017			33.00	45.00	45.00	45.00	36.46	36.01	Negative	true	True negative
	V0017262			27.02	N/A	N/A	38.76	36.76	35.76	Negative	V0017018			28.70	45.00	45.00	45.00	36.46	36.01	Negative	true	True negative
	V0016803			27.13	N/A	N/A	38.01	36.76	35.76	Negative	V0017021			33.70	45.00	45.00	45.00	36.46	36.01	Negative	true	true negative
	V0017354			31.31	N/A	N/A	N/A	35.78	36.30	Negative	V0015057			34.93	N/A	N/A	N/A	35.16	36.00	Negative	true	True negative
	V0016985			33.43	N/A	N/A	N/A	35.78	36.30	Negative	V0018455			34.42	N/A	N/A	N/A	35.67	36.11	Negative	true	True negative
	V0018463			29.11	37.02	37.13	38.30	35.78	36.30	Negative	V0018462			32.88	N/A	N/A	N/A	35.67	36.11	Negative	true	True negative
	V0018465			30.14	37.06	N/A	38.03	35.78	36.30	Negative	V0018439			34.08	N/A	N/A	N/A	35.67	36.11	Negative	true	True negative
	V0018411			28.94	N/A	N/A	N/A	36.02	35.87	Negative	V0019258			33.90	N/A	N/A	N/A	35.10	35.65	Negative	true	True negative
	V0018364			27.85	36.92	N/A	38.24	36.13	35.17	Negative	V0019262			34.00	36.70	N/A	N/A	35.10	35.65	Negative	true	True negative
	V0015036			29.49	N/A	N/A	N/A	36.13	35.17	Negative	V0019268			32.80	N/A	N/A	N/A	35.10	35.65	Negative	true	True negative
	V0017794			27.27	23.65	24.36	24.57	34.93	36.37	Positive	V0017791			34.40	34.80	35.00	34.90	34.82	36.02	Positive	true	True positive
	V0017841			28.42	24.36	23.92	24.09	34.93	36.37	Positive	V0017792			30.40	31.30	32.80	31.20	34.82	36.02	Positive	true	True positive
	V0017807			28.94	18.49	19.18	19.46	34.93	36.37	Positive	V0017798			33.80	23.70	24.20	23.00	34.82	36.02	Positive	true	True positive
	V0017813			28.12	25.18	24.60	24.71	34.93	36.37	Positive	V0017800			29.80	24.70	24.90	24.50	34.82	36.02	Positive	true	True positive
	V0017831			28.62	16.86	18.08	18.51	34.93	36.37	Positive	V0017804			29.30	27.20	27.90	27.00	34.82	36.02	Positive	true	True positive
	V0017789			27.95	28.21	28.48	28.52	34.93	36.37	Positive	V0017819			34.50	32.10	33.50	34.10	34.82	36.02	Positive	true	True positive
	V0017796			28.63	18.81	18.84	18.91	34.93	36.37	Positive	V0017825			33.10	25.70	26.90	26.90	34.82	36.02	Positive	true	True positive
	V0017817			27.95	28.80	29.11	29.24	34.93	36.37	Positive	V0017835			30.50	32.20	32.10	31.60	34.82	36.02	Positive	true	True positive
	V0017821			28.15	24.05	24.25	24.43	34.93	36.37	Positive	V0017836			33.80	30.20	30.40	30.20	34.82	36.02	Positive	true	True positive
	V0017832			29.20	17.38	17.52	17.70	34.93	36.37	Positive	V0017837			32.30	24.00	24.30	24.00	34.82	36.02	Positive	true	True positive
	V0017801			25.49	16.73	16.39	15.97	34.93	36.37	Positive	V0017839			34.10	31.70	31.50	31.90	34.82	36.02	Positive	true	True positive
	V0017827			28.70	25.40	25.59	25.69	35.85	37.10	Positive	V0017828			31.00	26.10	26.70	26.80	34.82	36.02	Positive	true	True positive
	V0018461			29.18	30.68	32.15	32.93	35.78	36.30	Positive	V0018451			32.44	26.27	25.99	25.69	35.67	36.11	Positive	true	True positive
	V0018464			29.38	31.98	32.29	32.47	35.78	36.30	Positive	V0018477			33.54	32.30	32.21	32.12	35.67	36.11	Positive	true	True positive
	V0018374			29.62	13.39	14.94	15.31	36.02	35.87	Positive	V0019280			32.50	27.90	28.90	29.30	35.10	35.65	Positive	true	True positive
	V0017196			29.11	16.55	17.82	18.32	36.76	35.76	Positive	V0016989			31.20	28.50	28.50	29.30	36.46	36.01	Positive	true	True positive
	V0016771			26.50	23.10	19.20	19.10	37.03	36.52	Positive	V0017016			33.00	34.30	29.10	29.00	36.39	37.05	Positive	true	True positive
	V0015396			28.60	35.05	36.18	37.94	35.70	36.11	Positive	V0016786			34.38	34.20	34.41	36.71	37.78	36.02	Positive	true	True positive
	V0015398			26.60	14.19	15.77	15.94	36.90	35.71	Positive	V0016811			33.70	34.00	35.15	34.85	37.78	36.02	Positive	true	True positive
	V0007630			29.10	21.48	23.29	23.16	33.84	33.95	Positive	V0007330			30.15	28.36	28.45	28.28	34.28	34.55	Positive	true	True positive
	V0007197			28.49	23.06	24.23	24.17	34.92	34.18	Positive	V0005904			35.78	31.98	31.96	32.17	34.75	34.35	Positive	true	True positive
	V0008357			29.45	21.49	23.53	24.17	34.35	34.00	Positive	V0003705			34.64	34.08	34.65	34.02	34.75	34.35	Positive	true	True positive
	V0008371			29.29	17.10	19.07	19.20	34.89	34.49	Positive	V0003697			35.26	27.79	27.85	27.71	34.75	34.35	Positive	true	True positive
	V0010895			29.33	26.08	26.80	27.19	38.28	35.38	Positive	V0012730			30.66	32.50	32.34	32.65	38.28	35.38	Positive	true	True positive
	V0011736			28.71	34.73	34.87	35.54	34.78	36.70	Positive	V0012535			31.80	30.11	30.51	30.59	35.17	35.76	Positive	true	True positive
	V0011727			28.49	27.96	29.20	29.04	34.78	36.70	Positive	V0012580			30.24	33.12	33.35	33.78	35.17	35.76	Positive	true	True positive
	V0010757			28.60	16.49	17.23	17.09	36.43	36.11	Positive	V0011478			34.59	30.42	31.01	31.38	36.43	36.11	Positive	true	True positive
	V0015941			29.27	16.67	18.43	18.47	35.46	35.95	Positive	V0012566			30.31	31.79	31.69	32.29	36.42	34.78	Positive	true	True positive
	V0014312			27.97	14.82	16.26	16.66	35.46	35.95	Positive	V0012571			32.21	26.46	27.46	27.35	36.10	36.36	Positive	true	True positive
	V0012907			29.70	15.19	16.42	17.14	36.30	34.83	Positive	V0012510			31.88	26.80	26.91	27.05	36.91	35.28	Positive	true	True positive
