# Supplementary material for: Development and implementation of a scalable and versatile test for COVID-19 diagnostics in rural communities
Source: Nat Commun. 2021 Jul 20;12:4400. doi: 10.1038/s41467-021-24552-4 (PMC8292415; doi:10.1038/s41467-021-24552-4)
Supplement: Supplementary file 3 — Supplementary Data 1 [file 41467_2021_24552_MOESM3_ESM.pdf]

NCBI

A	B	C	D	E	F	G	H	I	J	K
Database ID	NCBI Description	SK_N forward GCTGCAATCGTGCTACAACT	SK_N reverse TGAACTGTTGCGACTACGTG	SK_E forward TTCGGAAGAGACAGGTACGTT	SK_E reverse CACACAATCGAAGCGCAGTA	SK_S forward GCTGGTGCTGCAGCTTATTA	SK_S reverse AGGGTCAAGTGCACAGTCTA	3' mismatches?	Multiple mismatches?	More than 1 primer set?
1	NC_045512.2 |Severe acute respiratory syndrome cor	····················	····················	····················	····················	····················	····················			
2	MW466791.1 |Severe acute respiratory syndrome coro	····················	····················	····················	····················	····················	····················			
3	MW466792.1 |Severe acute respiratory syndrome coro	····················	····················	····················	····················	····················	····················			
4	MW466793.1 |Severe acute respiratory syndrome coro	····················	····················	····················	····················	····················	····················			
5	MW466794.1 |Severe acute respiratory syndrome coro	····················	····················	····················	····················	····················	····················			
6	MW466795.1 |Severe acute respiratory syndrome coro	····················	····················	····················	····················	····················	····················			
7	MW466796.1 |Severe acute respiratory syndrome coro	····················	····················	····················	····················	····················	····················			
8	MW466797.1 |Severe acute respiratory syndrome coro	····················	····················	····················	····················	····················	····················			
9	MW466798.1 |Severe acute respiratory syndrome coro	····················	····················	····················	····················	····················	····················			
10	MW466799.1 |Severe acute respiratory syndrome coro	····················	····················	····················	····················	····················	····················			
11	MW466800.1 |Severe acute respiratory syndrome coro	····················	····················	····················	····················	····················	····················			
12	MW467418.1 |Severe acute respiratory syndrome coro	····················	····················	····················	····················	····················	····················			
13	MW467419.1 |Severe acute respiratory syndrome coro	····················	·A··················	····················	····················	····················	····················			
14	MW467420.1 |Severe acute respiratory syndrome coro	····················	····················	····················	····················	····················	····················			
15	MW467421.1 |Severe acute respiratory syndrome coro	····················	····················	····················	····················	····················	····················			
16	MW467422.1 |Severe acute respiratory syndrome coro	····················	····················	····················	····················	····················	····················			
17	MW467423.1 |Severe acute respiratory syndrome coro	····················	····················	····················	····················	····················	····················			
18	MW467424.1 |Severe acute respiratory syndrome coro	····················	·····A··············	····················	····················	····················	····················			
19	MW467425.1 |Severe acute respiratory syndrome coro	····················	·····A··············	····················	····················	····················	····················			
20	MW467426.1 |Severe acute respiratory syndrome coro	····················	····················	····················	····················	····················	····················			
21	MW467427.1 |Severe acute respiratory syndrome coro	····················	····················	····················	····················	····················	····················			
22	MW467428.1 |Severe acute respiratory syndrome coro	····················	····················	····················	····················	····················	····················			
23	MW467429.1 |Severe acute respiratory syndrome coro	····················	····················	····················	····················	····················	····················			
24	MW467430.1 |Severe acute respiratory syndrome coro	····················	····················	····················	····················	····················	····················			
25	MW467431.1 |Severe acute respiratory syndrome coro	····················	········A···········	····················	····················	····················	····················			
26	MW467432.1 |Severe acute respiratory syndrome coro	····················	····················	····················	····················	····················	····················			
27	MW467433.1 |Severe acute respiratory syndrome coro	····················	····················	····················	····················	····················	····················			
28	MW467434.1 |Severe acute respiratory syndrome coro	····················	····················	····················	····················	····················	····················			
29	MW467435.1 |Severe acute respiratory syndrome coro	····················	····················	····················	····················	····················	····················			
30	MW467436.1 |Severe acute respiratory syndrome coro	····················	····················	····················	····················	····················	····················			
31	MW467437.1 |Severe acute respiratory syndrome coro	····················	····················	····················	····················	····················	····················			
32	MW467438.1 |Severe acute respiratory syndrome coro	····················	····················	····················	····················	····················	····················			
33	MW467439.1 |Severe acute respiratory syndrome coro	····················	····················	····················	····················	····················	····················			
34	MW467440.1 |Severe acute respiratory syndrome coro	····················	····················	····················	····················	····················	····················			
35	MW467441.1 |Severe acute respiratory syndrome coro	····················	····················	····················	····················	····················	····················			
36	MW467442.1 |Severe acute respiratory syndrome coro	····················	····················	····················	····················	····················	····················			
37	MW467443.1 |Severe acute respiratory syndrome coro	····················	····················	····················	····················	····················	····················			
38	MW467444.1 |Severe acute respiratory syndrome coro	····················	····················	····················	····················	····················	····················			
39	MW467445.1 |Severe acute respiratory syndrome coro	····················	····················	····················	····················	····················	····················			
40	MW467446.1 |Severe acute respiratory syndrome coro	····················	····················	····················	····················	····················	····················			
41	MW467447.1 |Severe acute respiratory syndrome coro	····················	····················	····················	····················	····················	····················			
42	MW467448.1 |Severe acute respiratory syndrome coro	····················	····················	····················	····················	····················	····················			
43	MW467449.1 |Severe acute respiratory syndrome coro	····················	····················	····················	····················	····················	····················			
44	MW467450.1 |Severe acute respiratory syndrome coro	····················	····················	····················	····················	····················	····················			
45	MW467451.1 |Severe acute respiratory syndrome coro	····················	········A···········	····················	····················	····················	····················			
46	MW467452.1 |Severe acute respiratory syndrome coro	····················	····················	····················	····················	····················	····················			
47	MW467453.1 |Severe acute respiratory syndrome coro	····················	····················	····················	····················	····················	····················			
48	MW467454.1 |Severe acute respiratory syndrome coro	····················	·A··················	····················	····················	····················	····················			
49	MW467455.1 |Severe acute respiratory syndrome coro	····················	····················	····················	····················	····················	····················			
50	MW467456.1 |Severe acute respiratory syndrome coro	····················	····················	····················	····················	····················	····················			
51	MW467457.1 |Severe acute respiratory syndrome coro	····················	········A···········	····················	····················	····················	····················			
52	MW467458.1 |Severe acute respiratory syndrome coro	····················	····················	····················	····················	····················	····················			
53	MW467459.1 |Severe acute respiratory syndrome coro	····················	········A···········	····················	····················	····················	····················			
54	MW467460.1 |Severe acute respiratory syndrome coro	····················	····················	····················	····················	····················	····················			
55	MW467461.1 |Severe acute respiratory syndrome coro	····················	····················	····················	····················	····················	····················			
56	MW467462.1 |Severe acute respiratory syndrome coro	····················	····················	····················	····················	····················	····················			
57	MW467463.1 |Severe acute respiratory syndrome coro	····················	····················	····················	····················	····················	····················			
58	MW467464.1 |Severe acute respiratory syndrome coro	····················	····················	····················	····················	····················	····················			
59	MW467465.1 |Severe acute respiratory syndrome coro	····················	········A···········	····················	····················	····················	····················			
60	MW467466.1 |Severe acute respiratory syndrome coro	····················	····················	····················	····················	····················	····················			
61	MW467467.1 |Severe acute respiratory syndrome coro	····················	····················	····················	··················C·	····················	····················	1		
62	MW467468.1 |Severe acute respiratory syndrome coro	····················	····················	····················	····················	····················	··A·················			
63	MW467469.1 |Severe acute respiratory syndrome coro	····················	····················	····················	····················	····················	····················			
64	MW467470.1 |Severe acute respiratory syndrome coro	····················	····················	····················	····················	····················	····················			
65	MW467471.1 |Severe acute respiratory syndrome coro	····················	····················	····················	····················	····················	····················			
66	MW467472.1 |Severe acute respiratory syndrome coro	····················	····················	····················	····················	····················	····················			
67	MW467473.1 |Severe acute respiratory syndrome coro	····················	····················	····················	····················	····················	····················			

68	MW467474.1	|Severe acute respiratory syndrome coro	····················	····················	····················	····················	····················	····················	
69	MW467475.1	|Severe acute respiratory syndrome coro	····················	····················	····················	····················	····················	····················	
70	MW467476.1	|Severe acute respiratory syndrome coro	····················	····················	····················	····················	····················	····················	
71	MW467477.1	|Severe acute respiratory syndrome coro	····················	····················	····················	····················	····················	····················	
72	MW467478.1	|Severe acute respiratory syndrome coro	····················	······A·············	····················	····················	····················	····················	
73	MW467479.1	|Severe acute respiratory syndrome coro	····················	····················	····················	····················	····················	····················	
74	MW467480.1	|Severe acute respiratory syndrome coro	····················	····················	····················	····················	····················	····················	
75	MW467481.1	|Severe acute respiratory syndrome coro	····················	····················	····················	····················	····················	····················	
76	MW467482.1	|Severe acute respiratory syndrome coro	····················	····················	····················	····················	····················	····················	
77	MW467483.1	|Severe acute respiratory syndrome coro	····················	·········A··········	····················	····················	····················	····················	
78	MW467484.1	|Severe acute respiratory syndrome coro	····················	······A·············	····················	····················	····················	····················	
79	MW467485.1	|Severe acute respiratory syndrome coro	····················	······A·············	····················	····················	····················	····················	
80	MW467486.1	|Severe acute respiratory syndrome coro	····················	·A··················	····················	····················	····················	···A················	
81	MW467487.1	|Severe acute respiratory syndrome coro	····················	····················	····················	····················	····················	····················	
82	MW467488.1	|Severe acute respiratory syndrome coro	····················	····················	····················	····················	····················	····················	
83	MW467489.1	|Severe acute respiratory syndrome coro	····················	·A··················	····················	····················	····················	···A················	
84	MW467490.1	|Severe acute respiratory syndrome coro	····················	·A··················	····················	····················	······A·············	····················	
85	MW467491.1	|Severe acute respiratory syndrome coro	····················	······A·············	····················	····················	····················	····················	
86	MW467492.1	|Severe acute respiratory syndrome coro	····················	····················	····················	····················	····················	····················	
87	MW467493.1	|Severe acute respiratory syndrome coro	····················	····················	····················	····················	····················	····················	
88	MW467494.1	|Severe acute respiratory syndrome coro	····················	····················	····················	····················	····················	····················	
89	MW467495.1	|Severe acute respiratory syndrome coro	····················	····················	····················	····················	····················	····················	
90	MW467496.1	|Severe acute respiratory syndrome coro	····················	····················	····················	····················	····················	····················	
91	MW467497.1	|Severe acute respiratory syndrome coro	····················	····················	····················	····················	····A···············	····················	
92	MW467498.1	|Severe acute respiratory syndrome coro	····················	····················	····················	····················	····················	····················	
93	MW467499.1	|Severe acute respiratory syndrome coro	····················	····················	····················	····················	····················	····················	
94	MW467500.1	|Severe acute respiratory syndrome coro	····················	····················	····················	····················	····················	····················	
95	MW467501.1	|Severe acute respiratory syndrome coro	····················	·········A··········	····················	····················	····················	·················G··	1
96	MW467502.1	|Severe acute respiratory syndrome coro	····················	····················	····················	····················	····················	····················	
97	MW467503.1	|Severe acute respiratory syndrome coro	····················	····················	····················	····················	····················	····················	
98	MW467504.1	|Severe acute respiratory syndrome coro	····················	····················	····················	····················	····················	····················	
99	MW467505.1	|Severe acute respiratory syndrome coro	····················	····················	····················	····················	····················	····················	
100	MW467506.1	|Severe acute respiratory syndrome coro	····················	····················	····················	····················	····················	····················	
101	MW467507.1	|Severe acute respiratory syndrome coro	····················	······A·············	····················	····················	····················	····················	
102	MW468415.1	|Severe acute respiratory syndrome coro	····················	····················	····················	····················	····················	····················	
103	MW471388.1	|Severe acute respiratory syndrome coro	····················	····················	····················	····················	····················	····················	
104	MW471389.1	|Severe acute respiratory syndrome coro	····················	····················	····················	····················	····················	····················	
105	MW471390.1	|Severe acute respiratory syndrome coro	····················	····················	····················	····················	····················	····················	
106	MW471391.1	|Severe acute respiratory syndrome coro	····················	····················	····················	····················	····················	····················	
107	MW471392.1	|Severe acute respiratory syndrome coro	····················	····················	····················	····················	····················	····················	
108	MW471393.1	|Severe acute respiratory syndrome coro	····················	····················	····················	····················	····················	····················	
109	MW471398.1	|Severe acute respiratory syndrome coro	····················	····················	····················	····················	····················	····················	
110	MW471399.1	|Severe acute respiratory syndrome coro	····················	····················	····················	····················	····················	····················	
111	MW471400.1	|Severe acute respiratory syndrome coro	····················	····················	····················	····················	····················	····················	
112	MW471401.1	|Severe acute respiratory syndrome coro	····················	····················	····················	····················	····················	····················	
113	MW473685.1	|Severe acute respiratory syndrome coro	····················	····················	····················	····················	····················	····················	
114	MW473686.1	|Severe acute respiratory syndrome coro	····················	····················	····················	····················	····················	····················	
115	MW473687.1	|Severe acute respiratory syndrome coro	····················	····················	····················	····················	····················	····················	
116	MW473688.1	|Severe acute respiratory syndrome coro	····················	····················	····················	····················	····················	····················	
117	MW473689.1	|Severe acute respiratory syndrome coro	····················	····················	····················	····················	····················	····················	
118	MW473690.1	|Severe acute respiratory syndrome coro	····················	····················	····················	····················	····················	····················	
119	MW473692.1	|Severe acute respiratory syndrome coro	····················	····················	····················	····················	····················	····················	
120	MW473694.1	|Severe acute respiratory syndrome coro	····················	····················	····················	····················	····················	····················	
121	MW473696.1	|Severe acute respiratory syndrome coro	····················	····················	····················	····················	····················	····················	
122	MW473697.1	|Severe acute respiratory syndrome coro	····················	····················	····················	····················	····················	····················	
123	MW473699.1	|Severe acute respiratory syndrome coro	····················	····················	····················	····················	····················	····················	
124	MW473700.1	|Severe acute respiratory syndrome coro	····················	····················	····················	····················	····················	····················	
125	MW474104.1	|Severe acute respiratory syndrome coro	····················	····················	····················	····················	····················	····················	
126	MW474105.1	|Severe acute respiratory syndrome coro	····················	·A··················	····················	····················	····················	····················	
127	MW474106.1	|Severe acute respiratory syndrome coro	····················	····················	····················	····················	····················	····················	
128	MW474107.1	|Severe acute respiratory syndrome coro	····················	····················	····················	····················	····················	····················	
129	MW474108.1	|Severe acute respiratory syndrome coro	····················	····················	····················	····················	····················	····················	
130	MW474109.1	|Severe acute respiratory syndrome coro	····················	·A··················	····················	····················	····················	····················	
131	MW474110.1	|Severe acute respiratory syndrome coro	····················	····················	····················	····················	····················	····················	
132	MW474111.1	|Severe acute respiratory syndrome coro	····················	····················	····················	····················	····················	····················	
133	MW474112.1	|Severe acute respiratory syndrome coro	····················	····················	····················	····················	····················	····················	
134	MW474113.1	|Severe acute respiratory syndrome coro	····················	·A··················	····················	····················	····················	····················	
135	MW474114.1	|Severe acute respiratory syndrome coro	····················	····················	····················	····················	····················	····················	
136	MW474115.1	|Severe acute respiratory syndrome coro	····················	····················	····················	····················	····················	····················	
137	MW474116.1	|Severe acute respiratory syndrome coro	····················	····················	····················	····················	····················	····················	
138	MW474117.1	|Severe acute respiratory syndrome coro	····················	····················	····················	····················	····················	····················	
139	MW474118.1	|Severe acute respiratory syndrome coro	····················	····················	····················	····················	····················	····················	
140	MW474119.1	|Severe acute respiratory syndrome coro	····················	·A··················	····················	····················	····················	····················	
141	MW474120.1	|Severe acute respiratory syndrome coro	····················	·A··················	····················	····················	····················	····················	
142	MW474121.1	|Severe acute respiratory syndrome coro	····················	····················	····················	····················	····················	····················	
143	MW474122.1	|Severe acute respiratory syndrome coro	····················	····················	····················	····················	····················	····················	
144	MW474123.1	|Severe acute respiratory syndrome coro	····················	····················	····················	····················	····················	····················	
145	MW474124.1	|Severe acute respiratory syndrome coro	····················	····················	····················	····················	····················	····················	

146 MW474125.1 |Severe acute respiratory syndrome coro ···················· ···················· ···················· ···················· ···················· ····················
147 MW474126.1 |Severe acute respiratory syndrome coro ···················· ···················· ···················· ···················· ···················· ····················
148 MW474127.1 |Severe acute respiratory syndrome coro ···················· ···················· ···················· ···················· ···················· ····················
149 MW474128.1 |Severe acute respiratory syndrome coro ···················· ···················· ···················· ···················· ···················· ····················
150 MW474129.1 |Severe acute respiratory syndrome coro ···················· ···················· ···················· ···················· ···················· ····················
151 MW474130.1 |Severe acute respiratory syndrome coro ···················· ···················· ···················· ···················· ···················· ····················
152 MW474131.1 |Severe acute respiratory syndrome coro ···················· ···················· ···················· ···················· ···················· ····················
153 MW474132.1 |Severe acute respiratory syndrome coro ···················· ···················· ···················· ···················· ···················· ····················
154 MW474133.1 |Severe acute respiratory syndrome coro ···················· ···················· ···················· ···················· ···················· ····················
155 MW474134.1 |Severe acute respiratory syndrome coro ···················· ···················· ···················· ···················· ···················· ····················
156 MW474135.1 |Severe acute respiratory syndrome coro ···················· ···················· ···················· ···················· ···················· ····················
157 MW474136.1 |Severe acute respiratory syndrome coro ···················· ···················· ···················· ···················· ···················· ····················
158 MW474137.1 |Severe acute respiratory syndrome coro ···················· ···················· ···················· ···················· ···················· ····················
159 MW474138.1 |Severe acute respiratory syndrome coro ···················· ···················· ···················· ···················· ···················· ····················
160 MW474139.1 |Severe acute respiratory syndrome coro ···················· ···················· ···················· ···················· ···················· ····················
161 MW474140.1 |Severe acute respiratory syndrome coro ···················· ···················· ···················· ···················· ···················· ····················
162 MW474141.1 |Severe acute respiratory syndrome coro ···················· ···················· ···················· ···················· ···················· ····················
163 MW474142.1 |Severe acute respiratory syndrome coro ···················· ···················· ···················· ···················· ···················· ····················
164 MW474143.1 |Severe acute respiratory syndrome coro ···················· ···················· ···················· ···················· ···················· ····················
165 MW474144.1 |Severe acute respiratory syndrome coro ···················· ···················· ···················· ···················· ···················· ····················
166 MW474145.1 |Severe acute respiratory syndrome coro ···················· ···················· ···················· ···················· ···················· ····················
167 MW474146.1 |Severe acute respiratory syndrome coro ···················· ·A·················· ···················· ···················· ···················· ····················
168 MW474147.1 |Severe acute respiratory syndrome coro ···················· ·A·················· ···················· ···················· ···················· ····················
169 MW474148.1 |Severe acute respiratory syndrome coro ···················· ···················· ···················· ···················· ···················· ····················
170 MW474149.1 |Severe acute respiratory syndrome coro ···················· ···················· ···················· ···················· ···················· ····················
171 MW474150.1 |Severe acute respiratory syndrome coro ···················· ···················· ···················· ···················· ···················· ····················
172 MW474151.1 |Severe acute respiratory syndrome coro ···················· ···················· ···················· ···················· ···················· ····················
173 MW474152.1 |Severe acute respiratory syndrome coro ···················· ···················· ···················· ···················· ···················· ····················
174 MW474153.1 |Severe acute respiratory syndrome coro ···················· ···················· ···················· ···················· ···················· ····················
175 MW474154.1 |Severe acute respiratory syndrome coro ···················· ···················· ···················· ···················· ···················· ····················
176 MW474155.1 |Severe acute respiratory syndrome coro ···················· ···················· ···················· ···················· ···················· ····················
177 MW474156.1 |Severe acute respiratory syndrome coro ···················· ···················· ···················· ···················· ···················· ····················
178 MW474157.1 |Severe acute respiratory syndrome coro ···················· ·A·················· ···················· ···················· ···················· ····················
179 MW474158.1 |Severe acute respiratory syndrome coro ···················· ···················· ···················· ···················· ···················· ····················
180 MW474159.1 |Severe acute respiratory syndrome coro ···················· ···················· ···················· ···················· ···················· ····················
181 MW474160.1 |Severe acute respiratory syndrome coro ···················· ·A·················· ···················· ···················· ···················· ····················
182 MW474161.1 |Severe acute respiratory syndrome coro ···················· ·A·················· ···················· ···················· ···················· ····················
183 MW474162.1 |Severe acute respiratory syndrome coro ···················· ···················· ···················· ···················· ···················· ····················
184 MW474163.1 |Severe acute respiratory syndrome coro ···················· ···················· ···················· ···················· ···················· ····················
185 MW474164.1 |Severe acute respiratory syndrome coro ···················· ···················· ···················· ···················· ···················· ····················
186 MW474165.1 |Severe acute respiratory syndrome coro ···················· ···················· ···················· ···················· ···················· ····················
187 MW474166.1 |Severe acute respiratory syndrome coro ···················· ···················· ··A················· ···················· ···················· ····················
188 MW474167.1 |Severe acute respiratory syndrome coro ···················· ···················· ···················· ···················· ···················· ····················
189 MW474168.1 |Severe acute respiratory syndrome coro ···················· ···················· ···················· ···················· ···················· ····················
190 MW474169.1 |Severe acute respiratory syndrome coro ···················· ···················· ···················· ···················· ···················· ····················
191 MW474170.1 |Severe acute respiratory syndrome coro ···················· ···················· ···················· ···················· ···················· ····················
192 MW474171.1 |Severe acute respiratory syndrome coro ···················· ···················· ···················· ···················· ···················· ····················
193 MW474173.1 |Severe acute respiratory syndrome coro ···················· ·A·················· ···················· ···················· ···················· ····················
194 MW474175.1 |Severe acute respiratory syndrome coro ···················· ·A·················· ···················· ···················· ···················· ····················
195 MW474176.1 |Severe acute respiratory syndrome coro ···················· ···················· ···················· ···················· ···················· ····················
196 MW474177.1 |Severe acute respiratory syndrome coro ···················· ···················· ···················· ···················· ···················· ····················
197 MW474178.1 |Severe acute respiratory syndrome coro ···················· ···················· ···················· ···················· ···················· ····················
198 MW474179.1 |Severe acute respiratory syndrome coro ···················· ·A·················· ···················· ···················· ···················· ····················
199 MW474180.1 |Severe acute respiratory syndrome coro ···················· ···················· ···················· ···················· ···················· ····················
200 MW474181.1 |Severe acute respiratory syndrome coro ···················· ···················· ···················· ···················· ···················· ····················
201 MW474182.1 |Severe acute respiratory syndrome coro ···················· ···················· ···················· ···················· ···················· ····················
202 MW474183.1 |Severe acute respiratory syndrome coro ···················· ···················· ···················· ···················· ···················· ····················
203 MW474184.1 |Severe acute respiratory syndrome coro ···················· ···················· ···················· ···················· ···················· ····················
204 MW474185.1 |Severe acute respiratory syndrome coro ···················· ···················· ···················· ···················· ···················· ····················
205 MW474186.1 |Severe acute respiratory syndrome coro ···················· ···················· ···················· ···················· ···················· ····················
206 MW474187.1 |Severe acute respiratory syndrome coro ···················· ···················· ···················· ···················· ···················· ····················
207 MW474188.1 |Severe acute respiratory syndrome coro ···················· ·A·················· ···················· ···················· ···················· ····················
208 MW474189.1 |Severe acute respiratory syndrome coro ···················· ···················· ··A················· ···················· ···················· ····················
209 MW474190.1 |Severe acute respiratory syndrome coro ···················· ···················· ···················· ···················· ···················· ····················
210 MW474191.1 |Severe acute respiratory syndrome coro ···················· ···················· ···················· ···················· ···················· ····················
211 MW474192.1 |Severe acute respiratory syndrome coro ···················· ·A·················· ···················· ···················· ···················· ····················
212 MW474193.1 |Severe acute respiratory syndrome coro ···················· ···················· ···················· ···················· ···················· ····················
213 MW474194.1 |Severe acute respiratory syndrome coro ···················· ···················· ···················· ···················· ···················· ····················
214 MW474195.1 |Severe acute respiratory syndrome coro ···················· ···················· ···················· ···················· ···················· ····················
215 MW474196.1 |Severe acute respiratory syndrome coro ···················· ·A·················· ···················· ···················· ···················· ····················
216 MW474197.1 |Severe acute respiratory syndrome coro ···················· ···················· ···················· ···················· ···················· ····················
217 MW474198.1 |Severe acute respiratory syndrome coro ···················· ···················· ···················· ···················· ···················· ····················
218 MW474199.1 |Severe acute respiratory syndrome coro ···················· ···················· ···················· ···················· ···················· ····················
219 MW474200.1 |Severe acute respiratory syndrome coro ···················· ···················· ···················· ···················· ···················· ····················
220 MW474201.1 |Severe acute respiratory syndrome coro ···················· ···················· ···················· ···················· ···················· ····················
221 MW474202.1 |Severe acute respiratory syndrome coro ···················· ···················· ···················· ···················· ···················· ····················
222 MW474203.1 |Severe acute respiratory syndrome coro ···················· ···················· ···················· ···················· ···················· ····················
223 MW474204.1 |Severe acute respiratory syndrome coro ···················· ···················· ···················· ···················· ···················· ····················

```
224 MW474205.1 |Severe acute respiratory syndrome coro    ....................  ....................  ....................  ....................  ....................  ....................
225 MW474206.1 |Severe acute respiratory syndrome coro    ....................  ....................  ....................  ....................  ....................  ....................
226 MW474207.1 |Severe acute respiratory syndrome coro    ....................  ....................  ....................  ....................  ....................  ....................
227 MW474209.1 |Severe acute respiratory syndrome coro    ....................  .A..................  ....................  ....................  ....................  ....................
228 MW474210.1 |Severe acute respiratory syndrome coro    ....................  ....................  ....................  ....................  ....................  ....................
229 MW474211.1 |Severe acute respiratory syndrome coro    ....................  ....................  ....................  ....................  ....................  ....................
230 MW474212.1 |Severe acute respiratory syndrome coro    ....................  ....................  ....................  ....................  ....................  ....................
231 MW474213.1 |Severe acute respiratory syndrome coro    ....................  ....................  ....................  ....................  ....................  ....................
232 MW474214.1 |Severe acute respiratory syndrome coro    ....................  ....................  ....................  ....................  ....................  ....................
233 MW474215.1 |Severe acute respiratory syndrome coro    ....................  ....................  ....................  ....................  ....................  ....................
234 MW474216.1 |Severe acute respiratory syndrome coro    ....................  ....................  ....................  ....................  ....................  ....................
235 MW474217.1 |Severe acute respiratory syndrome coro    ....................  ....................  ....................  ....................  ....................  ....................
236 MW474219.1 |Severe acute respiratory syndrome coro    ....................  ....................  ....................  ....................  ....................  ....................
237 MW474220.1 |Severe acute respiratory syndrome coro    ....................  ....................  ....................  ....................  ....................  ....................
238 MW474222.1 |Severe acute respiratory syndrome coro    ....................  ....................  ....................  ....................  ....................  ....................
239 MW474223.1 |Severe acute respiratory syndrome coro    ....................  ....................  ....................  ....................  ....................  ....................
240 MW474224.1 |Severe acute respiratory syndrome coro    ....................  ....................  ....................  ....................  ....................  ....................
241 MW474225.1 |Severe acute respiratory syndrome coro    ....................  ....................  ....................  ....................  ....................  ....................
242 MW474226.1 |Severe acute respiratory syndrome coro    ....................  ....................  ....................  ....................  ....................  ....................
243 MW474227.1 |Severe acute respiratory syndrome coro    ....................  ....................  ....................  ....................  ....................  ....................
244 MW474228.1 |Severe acute respiratory syndrome coro    ....................  ....................  ....................  ....................  ....................  ....................
245 MW474247.1 |Severe acute respiratory syndrome coro    ....................  ....................  ....................  ....................  ....................  ....................
246 MW474249.1 |Severe acute respiratory syndrome coro    ....................  .A..................  ....................  ....................  ....................  ....................
247 MW474278.1 |Severe acute respiratory syndrome coro    ....................  ....................  ....................  ....................  ....................  ....................
248 MW474304.1 |Severe acute respiratory syndrome coro    ....................  ....................  ....................  ....................  ....................  ....................
249 MW474307.1 |Severe acute respiratory syndrome coro    ....................  .A..................  ....................  ....................  ....................  ....................
250 MW474328.1 |Severe acute respiratory syndrome coro    ....................  ....................  ....................  ....................  ....................  ....................
251 MW474332.1 |Severe acute respiratory syndrome coro    ....................  ....................  ....................  ....................  ....................  ....................
252 MW474335.1 |Severe acute respiratory syndrome coro    ....................  ....................  ....................  ....................  ....................  ....................
253 MW474339.1 |Severe acute respiratory syndrome coro    ....................  ....................  ....................  ....................  ....................  ....................
254 MW474341.1 |Severe acute respiratory syndrome coro    ....................  ....................  ....................  ....................  ....................  ....................
255 MW474346.1 |Severe acute respiratory syndrome coro    ....................  ....................  ....................  ....................  ....................  ....................
256 MW474350.1 |Severe acute respiratory syndrome coro    ....................  .A..................  ....................  ....................  ....................  ....................
257 MW474354.1 |Severe acute respiratory syndrome coro    ....................  ....................  ....................  ....................  ....................  ....................
258 MW474367.1 |Severe acute respiratory syndrome coro    ....................  ....................  ....................  ....................  ....................  ....................
259 MW474739.1 |Severe acute respiratory syndrome coro    ....................  ....................  ....................  ....................  ....................  ....................
260 MW474741.1 |Severe acute respiratory syndrome coro    ....................  ....................  ....................  ....................  ....................  ....................
261 MW474742.1 |Severe acute respiratory syndrome coro    ....................  ....................  ....................  ....................  ....................  ....................
262 MW463452.1 |Severe acute respiratory syndrome coro    ....................  ....................  ....................  ....................  ....................  ....................
263 MW463453.1 |Severe acute respiratory syndrome coro    ....................  ....................  ....................  ....................  ....................  ....................
264 MW463456.1 |Severe acute respiratory syndrome coro    ....................  ....................  ....................  ....................  ....................  ....................
265 MW463458.1 |Severe acute respiratory syndrome coro    ....................  ....................  ....................  ....................  ....................  ....................
266 MW466639.1 |Severe acute respiratory syndrome coro    ....................  ....................  ....................  ....................  ....................  ....................
267 MW454126.1 |Severe acute respiratory syndrome coro    ....................  ....................  ....................  ....................  ....................  ....................
268 MW454127.1 |Severe acute respiratory syndrome coro    ....................  ....................  ....................  ....................  ..A.................  ....................
269 MW454130.1 |Severe acute respiratory syndrome coro    ....................  ....................  ....................  ....................  ....................  ....................
270 MW454390.1 |Severe acute respiratory syndrome coro    ....................  ....................  ....................  ....................  ....................  ....................
271 MW454391.1 |Severe acute respiratory syndrome coro    ....................  ....................  ....................  ....................  ....................  ....................
272 MW454392.1 |Severe acute respiratory syndrome coro    ....................  ....................  ....................  ....................  ....................  ....................
273 MW454393.1 |Severe acute respiratory syndrome coro    ....................  ....................  ....................  ....................  ....................  ....................
274 MW454394.1 |Severe acute respiratory syndrome coro    ....................  ....................  ....................  ....................  ....................  ....................
275 MW454395.1 |Severe acute respiratory syndrome coro    ....................  ....................  ....................  ....................  ....................  ....................
276 MW454396.1 |Severe acute respiratory syndrome coro    ....................  ....................  ....................  ....................  ....................  ....................
277 MW454397.1 |Severe acute respiratory syndrome coro    ....................  .A..................  ....................  ....................  ....................  ....................
278 MW454398.1 |Severe acute respiratory syndrome coro    ....................  .A..................  ....................  ....................  ....................  ....................
279 MW454399.1 |Severe acute respiratory syndrome coro    ....................  ....................  ....................  ....................  ....................  ....................
280 MW454400.1 |Severe acute respiratory syndrome coro    ....................  ....................  ....................  ....................  ....................  ....................
281 MW454401.1 |Severe acute respiratory syndrome coro    ....................  ....................  ....................  ....................  ....................  ....................
282 MW454402.1 |Severe acute respiratory syndrome coro    ....................  .A..................  ....................  ....................  ....................  ....................
283 MW454403.1 |Severe acute respiratory syndrome coro    ....................  ....................  ....................  ....................  ....................  ....................
284 MW454404.1 |Severe acute respiratory syndrome coro    ....................  ....................  ....................  ....................  ....................  ....................
285 MW454406.1 |Severe acute respiratory syndrome coro    ....................  ....................  ....................  ....................  ....................  ....................
286 MW454407.1 |Severe acute respiratory syndrome coro    ....................  ....................  ....................  ....................  ....................  ....................
287 MW454408.1 |Severe acute respiratory syndrome coro    ....................  ....................  ....................  ....................  ....................  ....................
288 MW454410.1 |Severe acute respiratory syndrome coro    ....................  ....................  ....................  ....................  ....................  ....................
289 MW454411.1 |Severe acute respiratory syndrome coro    ....................  .A..................  ....................  ....................  ....................  ....................
290 MW454412.1 |Severe acute respiratory syndrome coro    ....................  .A..................  ....................  ....................  ....................  ....................
291 MW454414.1 |Severe acute respiratory syndrome coro    ....................  ....................  ....................  ....................  ....................  ....................
292 MW454415.1 |Severe acute respiratory syndrome coro    ....................  ....................  ....................  ....................  ....................  ....................
293 MW454416.1 |Severe acute respiratory syndrome coro    ....................  ....................  ....................  ....................  ....................  ....................
294 MW454417.1 |Severe acute respiratory syndrome coro    ....................  .A..................  ....................  ....................  ....................  ....................
295 MW454418.1 |Severe acute respiratory syndrome coro    ....................  .A..................  ....................  ....................  ....................  ....................
296 MW454419.1 |Severe acute respiratory syndrome coro    ....................  ....................  ....................  ....................  ....................  ....................
297 MW454420.1 |Severe acute respiratory syndrome coro    ....................  ....................  ....................  ....................  ....................  ....................
298 MW454421.1 |Severe acute respiratory syndrome coro    ....................  .A..................  ....................  ....................  ....................  ....................
299 MW454422.1 |Severe acute respiratory syndrome coro    ....................  ....................  ....................  ....................  ....................  ....................
300 MW454423.1 |Severe acute respiratory syndrome coro    ....................  ....................  ....................  ....................  ....................  ....................
301 MW454424.1 |Severe acute respiratory syndrome coro    ....................  .A..................  ....................  ....................  ....................  ....................
```

```
302 MW454425.1 |Severe acute respiratory syndrome coro ···················· ·A·················· ····················· ···················· ···················· ····················
303 MW454426.1 |Severe acute respiratory syndrome coro ···················· ·A·················· ····················· ···················· ···················· ····················
304 MW454427.1 |Severe acute respiratory syndrome coro ···················· ·A·················· ····················· ···················· ···················· ····················
305 MW454428.1 |Severe acute respiratory syndrome coro ···················· ···················· ····················· ···················· ···················· ····················
306 MW454429.1 |Severe acute respiratory syndrome coro ···················· ·A·················· ····················· ···················· ···················· ····················
307 MW454431.1 |Severe acute respiratory syndrome coro ···················· ···················· ····················· ···················· ···················· ····················
308 MW454433.1 |Severe acute respiratory syndrome coro ···················· ···················· ··T·················· ···················· ···················· ····················
309 MW454434.1 |Severe acute respiratory syndrome coro ···················· ···················· ····················· ···················· ···················· ····················
310 MW454435.1 |Severe acute respiratory syndrome coro ···················· ···················· ····················· ···················· ···················· ····················
311 MW454436.1 |Severe acute respiratory syndrome coro ···················· ···················· ····················· ···················· ···················· ····················
312 MW454438.1 |Severe acute respiratory syndrome coro ···················· ·A·················· ····················· ···················· ···················· ····················
313 MW454440.1 |Severe acute respiratory syndrome coro ···················· ·A·················· ····················· ···················· ···················· ····················
314 MW454441.1 |Severe acute respiratory syndrome coro ···················· ···················· ····················· ···················· ···················· ····················
315 MW454442.1 |Severe acute respiratory syndrome coro ···················· ·A·················· ····················· ···················· ···················· ····················
316 MW454446.1 |Severe acute respiratory syndrome coro ···················· ·A·················· ····················· ···················· ···················· ····················
317 MW454447.1 |Severe acute respiratory syndrome coro ···················· ·A·················· ····················· ···················· ···················· ····················
318 MW454449.1 |Severe acute respiratory syndrome coro ···················· ···················· ····················· ···················· ···················· ····················
319 MW454450.1 |Severe acute respiratory syndrome coro ···················· ···················· ····················· ···················· ···················· ····················
320 MW454451.1 |Severe acute respiratory syndrome coro ···················· ···················· ····················· ···················· ···················· ····················
321 MW454452.1 |Severe acute respiratory syndrome coro ···················· ···················· ····················· ···················· ···················· ····················
322 MW454453.1 |Severe acute respiratory syndrome coro ···················· ···················· ····················· ···················· ···················· ····················
323 MW454455.1 |Severe acute respiratory syndrome coro ···················· ···················· ····················· ···················· ···················· ····················
324 MW454456.1 |Severe acute respiratory syndrome coro ···················· ···················· ····················· ···················· ···················· ····················
325 MW454457.1 |Severe acute respiratory syndrome coro ···················· ···················· ····················· ···················· ···················· ····················
326 MW454459.1 |Severe acute respiratory syndrome coro ···················· ···················· ····················· ···················· ···················· ····················
327 MW454460.1 |Severe acute respiratory syndrome coro ···················· ···················· ····················· ···················· ···················· ····················
328 MW454461.1 |Severe acute respiratory syndrome coro ···················· ·A·················· ····················· ···················· ···················· ····················
329 MW454462.1 |Severe acute respiratory syndrome coro ···················· ·A·················· ····················· ···················· ···················· ····················
330 MW454463.1 |Severe acute respiratory syndrome coro ···················· ···················· ····················· ···················· ···················· ····················
331 MW454464.1 |Severe acute respiratory syndrome coro ···················· ···················· ····················· ···················· ···················· ····················
332 MW454467.1 |Severe acute respiratory syndrome coro ···················· ···················· ····················· ···················· ···················· ····················
333 MW454468.1 |Severe acute respiratory syndrome coro ···················· ···················· ····················· ···················· ···················· ····················
334 MW454469.1 |Severe acute respiratory syndrome coro ···················· ···················· ····················· ···················· ···················· ····················
335 MW454470.1 |Severe acute respiratory syndrome coro ···················· ···················· ····················· ···················· ···················· ····················
336 MW454471.1 |Severe acute respiratory syndrome coro ···················· ···················· ····················· ···················· ···················· ····················
337 MW454472.1 |Severe acute respiratory syndrome coro ···················· ···················· ····················· ···················· ···················· ····················
338 MW454474.1 |Severe acute respiratory syndrome coro ···················· ···················· ····················· ···················· ···················· ····················
339 MW454475.1 |Severe acute respiratory syndrome coro ···················· ···················· ····················· ···················· ···················· ····················
340 MW454476.1 |Severe acute respiratory syndrome coro ···················· ···················· ····················· ···················· ···················· ····················
341 MW454477.1 |Severe acute respiratory syndrome coro ···················· ···················· ····················· ···················· ···················· ····················
342 MW454478.1 |Severe acute respiratory syndrome coro ···················· ···················· ····················· ···················· ···················· ····················
343 MW454479.1 |Severe acute respiratory syndrome coro ···················· ···················· ····················· ···················· ···················· ····················
344 MW454480.1 |Severe acute respiratory syndrome coro ···················· ···················· ····················· ···················· ···················· ····················
345 MW454481.1 |Severe acute respiratory syndrome coro ···················· ···················· ····················· ···················· ···················· ····················
346 MW454482.1 |Severe acute respiratory syndrome coro ···················· ···················· ····················· ···················· ···················· ····················
347 MW454483.1 |Severe acute respiratory syndrome coro ···················· ···················· ····················· ···················· ···················· ····················
348 MW454486.1 |Severe acute respiratory syndrome coro ···················· ···················· ····················· ···················· ···················· ····················
349 MW454487.1 |Severe acute respiratory syndrome coro ···················· ···················· ····················· ···················· ···················· ····················
351 MW454490.1 |Severe acute respiratory syndrome coro ···················· ···················· ····················· ···················· ···················· ····················
352 MW454492.1 |Severe acute respiratory syndrome coro ···················· ···················· ····················· ···················· ···················· ····················
353 MW454493.1 |Severe acute respiratory syndrome coro ···················· ···················· ····················· ···················· ···················· ····················
354 MW454494.1 |Severe acute respiratory syndrome coro ···················· ···················· ····················· ···················· ···················· ····················
355 MW454495.1 |Severe acute respiratory syndrome coro ···················· ···················· ····················· ···················· ···················· ····················
356 MW454496.1 |Severe acute respiratory syndrome coro ···················· ···················· ····················· ···················· ···················· ····················
357 MW454498.1 |Severe acute respiratory syndrome coro ···················· ···················· ····················· ···················· ···················· ····················
358 MW454499.1 |Severe acute respiratory syndrome coro ···················· ···················· ····················· ···················· ···················· ····················
359 MW454500.1 |Severe acute respiratory syndrome coro ···················· ···················· ····················· ···················· ···················· ····················
360 MW454502.1 |Severe acute respiratory syndrome coro ···················· ···················· ····················· ···················· ···················· ····················
361 MW454503.1 |Severe acute respiratory syndrome coro ···················· ···················· ····················· ···················· ···················· ····················
362 MW454504.1 |Severe acute respiratory syndrome coro ···················· ···················· ····················· ···················· ···················· ····················
363 MW454505.1 |Severe acute respiratory syndrome coro ···················· ···················· ····················· ···················· ···················· ····················
364 MW454506.1 |Severe acute respiratory syndrome coro ···················· ···················· ····················· ···················· ···················· ····················
365 MW454507.1 |Severe acute respiratory syndrome coro ···················· ···················· ····················· ···················· ···················· ····················
366 MW454508.1 |Severe acute respiratory syndrome coro ···················· ···················· ····················· ···················· ······T············· ····················
367 MW454509.1 |Severe acute respiratory syndrome coro ···················· ···················· ····················· ···················· ···················· ····················
368 MW454510.1 |Severe acute respiratory syndrome coro ···················· ···················· ····················· ···················· ···················· ····················
369 MW454511.1 |Severe acute respiratory syndrome coro ···················· ···················· ····················· ···················· ···················· ····················
370 MW454512.1 |Severe acute respiratory syndrome coro ···················· ·A·················· ····················· ···················· ···················· ····················
371 MW454513.1 |Severe acute respiratory syndrome coro ···················· ···················· ····················· ···················· ···················· ····················
372 MW454514.1 |Severe acute respiratory syndrome coro ···················· ·A·················· ····················· ···················· ···················· ····················
373 MW454515.1 |Severe acute respiratory syndrome coro ···················· ···················· ····················· ···················· ···················· ····················
374 MW454516.1 |Severe acute respiratory syndrome coro ···················· ·A·················· ····················· ···················· ···················· ····················
375 MW454517.1 |Severe acute respiratory syndrome coro ···················· ···················· ····················· ···················· ···················· ····················
376 MW454518.1 |Severe acute respiratory syndrome coro ···················· ···················· ····················· ···················· ···················· ····················
377 MW454519.1 |Severe acute respiratory syndrome coro ···················· ·A·················· ····················· ···················· ···················· ····················
378 MW454520.1 |Severe acute respiratory syndrome coro ···················· ···················· ····················· ···················· ···················· ····················
379 MW454521.1 |Severe acute respiratory syndrome coro ···················· ···················· ····················· ···················· ···················· ····················
380 MW454522.1 |Severe acute respiratory syndrome coro ···················· ···················· ····················· ···················· ···················· ····················
```

```
381 MW454523.1 |Severe acute respiratory syndrome coro    ....................  ....................  ....................  ....................  ....................  ....................
382 MW454524.1 |Severe acute respiratory syndrome coro    ....................  ....................  ....................  ....................  ....................  ....................
383 MW454525.1 |Severe acute respiratory syndrome coro    ....................  ....................  ....................  ....................  ....................  ....................
384 MW454526.1 |Severe acute respiratory syndrome coro    ....................  ....................  ....................  ....................  ....................  ....................
385 MW454527.1 |Severe acute respiratory syndrome coro    ....................  ....................  ....................  ....................  ....................  ....................
386 MW454528.1 |Severe acute respiratory syndrome coro    ....................  ....................  ....................  ....................  ....................  ....................
387 MW454529.1 |Severe acute respiratory syndrome coro    ....................  ....................  ....................  ....................  ....................  ....................
388 MW454530.1 |Severe acute respiratory syndrome coro    ....................  ....................  ....................  ....................  ....................  ....................
389 MW454531.1 |Severe acute respiratory syndrome coro    ....................  ....................  ....................  ....................  ....................  ....................
390 MW454532.1 |Severe acute respiratory syndrome coro    ....................  ....................  ....................  ....................  ....................  ....................
391 MW454533.1 |Severe acute respiratory syndrome coro    ....................  ....................  ....................  ....................  ....................  ....................
392 MW454534.1 |Severe acute respiratory syndrome coro    ....................  ....................  ....................  ....................  ....................  ....................
393 MW454535.1 |Severe acute respiratory syndrome coro    ....................  .A..................  ....................  ....................  ....................  ....................
394 MW454536.1 |Severe acute respiratory syndrome coro    ....................  ....................  ....................  ....................  ....................  ....................
395 MW454537.1 |Severe acute respiratory syndrome coro    ....................  ....................  ....................  ....................  ....................  ....................
396 MW454538.1 |Severe acute respiratory syndrome coro    ....................  ....................  ....................  ....................  ....................  ....................
397 MW454539.1 |Severe acute respiratory syndrome coro    ....................  ....................  ....................  ....................  ....................  ....................
398 MW454541.1 |Severe acute respiratory syndrome coro    ....................  .A..................  ....................  ....................  ....................  ....................
399 MW454542.1 |Severe acute respiratory syndrome coro    ....................  ....................  ....................  ....................  ....................  ....................
400 MW454543.1 |Severe acute respiratory syndrome coro    ....................  ....................  ....................  ....................  ....................  ....................
401 MW454544.1 |Severe acute respiratory syndrome coro    ....................  ....................  ....................  ....................  ....................  ....................
402 MW454545.1 |Severe acute respiratory syndrome coro    ....................  ....................  ....................  ....................  ....................  ....................
403 MW454546.1 |Severe acute respiratory syndrome coro    ....................  .A..................  ....................  ....................  ....................  ....................
404 MW454547.1 |Severe acute respiratory syndrome coro    ....................  ....................  ....................  ....................  ....................  ....................
405 MW454548.1 |Severe acute respiratory syndrome coro    ....................  ....................  ....................  ....................  ....................  ....................
406 MW454549.1 |Severe acute respiratory syndrome coro    ....................  ....................  ....................  ....................  ....................  ....................
407 MW454550.1 |Severe acute respiratory syndrome coro    ....................  ....................  ....................  ....................  ....................  ....................
408 MW454551.1 |Severe acute respiratory syndrome coro    ....................  ....................  ....................  ....................  ....................  ....................
409 MW454552.1 |Severe acute respiratory syndrome coro    ....................  ....................  ....................  ....................  ....................  ....................
410 MW454553.1 |Severe acute respiratory syndrome coro    ....................  ....................  ....................  ....................  ....................  ....................
411 MW454554.1 |Severe acute respiratory syndrome coro    ....................  ....................  ....................  ....................  ....................  ....................
412 MW454555.1 |Severe acute respiratory syndrome coro    ....................  ....................  ....................  ....................  ....................  ....................
413 MW454556.1 |Severe acute respiratory syndrome coro    ....................  ....................  ....................  ....................  ....................  ....................
414 MW454557.1 |Severe acute respiratory syndrome coro    ....................  ....................  ....................  ....................  ....................  ....................
415 MW454558.1 |Severe acute respiratory syndrome coro    ....................  ....................  ....................  ....................  ....................  ....................
416 MW454559.1 |Severe acute respiratory syndrome coro    ....................  ....................  ....................  ....................  ....................  ....................
417 MW454560.1 |Severe acute respiratory syndrome coro    ....................  ....................  ....................  ....................  ....................  ....................
418 MW454561.1 |Severe acute respiratory syndrome coro    ....................  ....................  ....................  ....................  ....................  ....................
419 MW454562.1 |Severe acute respiratory syndrome coro    ....................  ....................  ....................  ....................  ....................  ....................
420 MW454563.1 |Severe acute respiratory syndrome coro    ....................  ....................  ....................  ....................  ....................  ....................
421 MW454564.1 |Severe acute respiratory syndrome coro    ....................  ....................  ....................  ....................  ....................  ....................
422 MW454565.1 |Severe acute respiratory syndrome coro    ....................  ....................  ....................  ....................  ....................  ....................
423 MW454566.1 |Severe acute respiratory syndrome coro    ....................  .........A..........  ....................  ....................  ....................  ....................
424 MW454567.1 |Severe acute respiratory syndrome coro    ....................  ....................  ....................  ....................  ....................  ....................
425 MW454568.1 |Severe acute respiratory syndrome coro    ....................  ....................  ....................  ....................  ....................  ....................
426 MW454569.1 |Severe acute respiratory syndrome coro    ....................  ....................  ....................  ....................  ....................  ....................
427 MW454570.1 |Severe acute respiratory syndrome coro    ....................  ....................  ....................  ....................  ....................  ....................
428 MW454571.1 |Severe acute respiratory syndrome coro    ....................  ....................  ....................  ....................  ....................  ....................
429 MW454572.1 |Severe acute respiratory syndrome coro    ....................  ....................  ....................  ....................  ....................  ....................
430 MW454573.1 |Severe acute respiratory syndrome coro    ....................  ....................  ....................  ....................  ....................  ....................
431 MW454574.1 |Severe acute respiratory syndrome coro    ....................  ....................  ....................  ....................  ....................  ....................
432 MW454575.1 |Severe acute respiratory syndrome coro    ....................  ....................  ....................  ....................  ....................  ....................
433 MW454576.1 |Severe acute respiratory syndrome coro    ....................  .A..................  ....................  ....................  ....................  ....................
434 MW454577.1 |Severe acute respiratory syndrome coro    ....................  ....................  ....................  ....................  ....................  ....................
435 MW454578.1 |Severe acute respiratory syndrome coro    ....................  ....................  ....................  ....................  ....................  ....................
436 MW454579.1 |Severe acute respiratory syndrome coro    ....................  ....................  ....................  ....................  ....................  ....................
437 MW454580.1 |Severe acute respiratory syndrome coro    ....................  ....................  ....................  ....................  ....................  ....................
438 MW454581.1 |Severe acute respiratory syndrome coro    ....................  ....................  ....................  ....................  ....................  ....................
439 MW454582.1 |Severe acute respiratory syndrome coro    ....................  ....................  ..........T.........  ....................  ....................  ....................
440 MW454583.1 |Severe acute respiratory syndrome coro    ....................  ....................  ....................  ....................  ....................  ....................
441 MW454584.1 |Severe acute respiratory syndrome coro    ....................  ....................  ....................  ....................  ....................  ....................
442 MW454585.1 |Severe acute respiratory syndrome coro    ....................  ....................  ....................  ....................  ....................  ....................
443 MW454586.1 |Severe acute respiratory syndrome coro    ....................  ....................  ....................  ....................  ....................  ....................
444 MW454587.1 |Severe acute respiratory syndrome coro    ....................  ....................  ....................  ....................  ....................  ....................
445 MW454588.1 |Severe acute respiratory syndrome coro    ....................  ....................  ....................  ....................  ....................  ....................
446 MW454589.1 |Severe acute respiratory syndrome coro    ....................  ....................  ....................  ....................  ....................  ....................
447 MW454590.1 |Severe acute respiratory syndrome coro    ....................  ....................  ....................  ....................  ....................  ....................
448 MW454591.1 |Severe acute respiratory syndrome coro    ....................  ....................  ....................  ....................  ....................  ....................
449 MW454592.1 |Severe acute respiratory syndrome coro    ....................  ....................  ....................  ....................  ....................  ....................
450 MW454593.1 |Severe acute respiratory syndrome coro    ....................  ....................  ....................  ....................  ....................  ....................
451 MW454594.1 |Severe acute respiratory syndrome coro    ....................  ....................  ....................  ....................  ....................  ....................
452 MW454595.1 |Severe acute respiratory syndrome coro    ....................  ....................  ....................  ....................  ....................  ....................
453 MW454596.1 |Severe acute respiratory syndrome coro    ....................  ....................  ....................  .............A......  ....................  ....................
454 MW454597.1 |Severe acute respiratory syndrome coro    ....................  ....................  ....................  ....................  ....................  ....................
455 MW454598.1 |Severe acute respiratory syndrome coro    ....................  ....................  ....................  ....................  ....................  ....................
456 MW454599.1 |Severe acute respiratory syndrome coro    ....................  ....................  ....................  ....................  ....................  ....................
457 MW454600.1 |Severe acute respiratory syndrome coro    ....................  ....................  ....................  ....................  ....................  ....................
458 MW454601.1 |Severe acute respiratory syndrome coro    ....................  ....................  ....................  ....................  ....................  ....................
```

459	MW454602.1	|Severe acute respiratory syndrome coro	....	....	....	....	....	....
460	MW454603.1	|Severe acute respiratory syndrome coro	....	....	....	....	....	....
461	MW454604.1	|Severe acute respiratory syndrome coro	....	....	....	....	....	....
462	MW454605.1	|Severe acute respiratory syndrome coro	....	....	....	....	....	....
463	MW454606.1	|Severe acute respiratory syndrome coro	....	....	....	....	....	....
464	MW454607.1	|Severe acute respiratory syndrome coro	....	....	....	....	....	....
465	MW454608.1	|Severe acute respiratory syndrome coro	....	.A..................	....	....	....	....
466	MW454609.1	|Severe acute respiratory syndrome coro	....	....	....	....	....	....
467	MW454610.1	|Severe acute respiratory syndrome coro	....	....	....	....	....	....
468	MW454611.1	|Severe acute respiratory syndrome coro	....	....	....	....	....	....
469	MW454612.1	|Severe acute respiratory syndrome coro	....	....	....	....	....	....
470	MW454613.1	|Severe acute respiratory syndrome coro	....	....	....	....	....	....
471	MW454614.1	|Severe acute respiratory syndrome coro	....	....	....	....	....	....
472	MW454615.1	|Severe acute respiratory syndrome coro	....	....	....	....	....	....
473	MW454617.1	|Severe acute respiratory syndrome coro	....	....	....	....	....	....
474	MW454618.1	|Severe acute respiratory syndrome coro	....	....	....	....	....	....
475	MW454619.1	|Severe acute respiratory syndrome coro	....	....	....	....	....	....
476	MW454620.1	|Severe acute respiratory syndrome coro	....	....	....	....	....	....
477	MW454621.1	|Severe acute respiratory syndrome coro	....	....	....	....	....	....
478	MW454622.1	|Severe acute respiratory syndrome coro	....	....A...............	....	....	....	....
479	MW454623.1	|Severe acute respiratory syndrome coro	....	....	....	....	....	....
480	MW454624.1	|Severe acute respiratory syndrome coro	....	....	....	....	....	....
481	MW454625.1	|Severe acute respiratory syndrome coro	....	....	....	....	....	....
482	MW454626.1	|Severe acute respiratory syndrome coro	....	....	....	....	....	....
483	MW454628.1	|Severe acute respiratory syndrome coro	....	....	....	....	....	....
484	MW454629.1	|Severe acute respiratory syndrome coro	....	....	....	....	....	....
485	MW454630.1	|Severe acute respiratory syndrome coro	....	....	....	....	....	....
486	MW454631.1	|Severe acute respiratory syndrome coro	....	.A..................	....	....	....	....
487	MW454632.1	|Severe acute respiratory syndrome coro	....	....	....	....	....	....
488	MW454633.1	|Severe acute respiratory syndrome coro	....	....	....	....	....	....
489	MW454634.1	|Severe acute respiratory syndrome coro	....	....	....	....	....	....
490	MW454635.1	|Severe acute respiratory syndrome coro	....	....	....	....	....	....
491	MW454636.1	|Severe acute respiratory syndrome coro	....	....	....	....	....	....
492	MW454637.1	|Severe acute respiratory syndrome coro	....	....	....	....	....	....
493	MW454638.1	|Severe acute respiratory syndrome coro	....	....	....	....	....	....
494	MW454639.1	|Severe acute respiratory syndrome coro	....	....	....	....	....	....
495	MW454640.1	|Severe acute respiratory syndrome coro	....	....	....	....	....	....
496	MW454641.1	|Severe acute respiratory syndrome coro	....	....	....	....	....	....
497	MW454642.1	|Severe acute respiratory syndrome coro	....	....	....	....	....	....
498	MW454643.1	|Severe acute respiratory syndrome coro	....	....	....	....	....	....
499	MW454644.1	|Severe acute respiratory syndrome coro	....	....	....	....	....	....
500	MW454645.1	|Severe acute respiratory syndrome coro	....	....	....	....	....	....
501	MW454646.1	|Severe acute respiratory syndrome coro	....	....	....	....	....	....
502	MW454647.1	|Severe acute respiratory syndrome coro	....	....	....	....	....	....
503	MW454648.1	|Severe acute respiratory syndrome coro	....	....	....	....	....	....
504	MW454649.1	|Severe acute respiratory syndrome coro	....	.A..................	....	....	....	....
505	MW454650.1	|Severe acute respiratory syndrome coro	....	....	....	....	....	....
506	MW454651.1	|Severe acute respiratory syndrome coro	....	....	....	....	....	....
507	MW454652.1	|Severe acute respiratory syndrome coro	....	....	....	....	......T.............	....
508	MW454653.1	|Severe acute respiratory syndrome coro	....	....	....	....	....	....
509	MW454654.1	|Severe acute respiratory syndrome coro	....	....	....	....	....	....
510	MW454655.1	|Severe acute respiratory syndrome coro	....	....	....	....	....	....
511	MW454656.1	|Severe acute respiratory syndrome coro	....	....	....	....	....	....
512	MW454657.1	|Severe acute respiratory syndrome coro	....	....	....	....	....	....
513	MW454658.1	|Severe acute respiratory syndrome coro	....	....	....	....	....	....
514	MW454659.1	|Severe acute respiratory syndrome coro	....	....	....	....	....	....
515	MW454660.1	|Severe acute respiratory syndrome coro	....	.A..................	....	....	....	....
516	MW454661.1	|Severe acute respiratory syndrome coro	....	....	....	....	....	....
517	MW454662.1	|Severe acute respiratory syndrome coro	....	....	....	....	....	....
518	MW454663.1	|Severe acute respiratory syndrome coro	....	.A..................	....	....	....	....
519	MW454664.1	|Severe acute respiratory syndrome coro	....	....	....	....	....	....
520	MW454665.1	|Severe acute respiratory syndrome coro	....	....	....	....	....	....
521	MW454666.1	|Severe acute respiratory syndrome coro	....	....	....	....	....	....
522	MW454667.1	|Severe acute respiratory syndrome coro	....	....	....	....	....	....
524	MW454669.1	|Severe acute respiratory syndrome coro	....	....	....	....	....	....
525	MW454670.1	|Severe acute respiratory syndrome coro	....	....	....	....	....	....
526	MW454671.1	|Severe acute respiratory syndrome coro	....	.A..................	....	....	....	....
527	MW454672.1	|Severe acute respiratory syndrome coro	....	....	....	....	....	....
528	MW454674.1	|Severe acute respiratory syndrome coro	....	....	....	....	....	....
529	MW454675.1	|Severe acute respiratory syndrome coro	....	....	....	....	....	....
530	MW454676.1	|Severe acute respiratory syndrome coro	....	....	....	....	....	....
531	MW454677.1	|Severe acute respiratory syndrome coro	....	....	....	....	....	....
532	MW454678.1	|Severe acute respiratory syndrome coro	....	....	....	....	....	....
533	MW454679.1	|Severe acute respiratory syndrome coro	....	....	....	....	....	....
534	MW454680.1	|Severe acute respiratory syndrome coro	....	....	....	....	....	....
535	MW454681.1	|Severe acute respiratory syndrome coro	....	....	....	....	....	....
536	MW454682.1	|Severe acute respiratory syndrome coro	....	....	....	....	....	....
537	MW454683.1	|Severe acute respiratory syndrome coro	....	....	....	....	....	....

```
538 MW454684.1 |Severe acute respiratory syndrome coro    ....................  ....................  ....................  ....................  ....................  ....................
539 MW454685.1 |Severe acute respiratory syndrome coro    ....................  ....................  ....................  ....................  ....................  ....................
540 MW454686.1 |Severe acute respiratory syndrome coro    ....................  ....................  ....................  ....................  ....................  ....................
541 MW454687.1 |Severe acute respiratory syndrome coro    ....................  ....................  ....................  ....................  ....................  ....................
542 MW454688.1 |Severe acute respiratory syndrome coro    ....................  ....................  ....................  ....................  ....................  ....................
544 MW454690.1 |Severe acute respiratory syndrome coro    ....................  ....................  ....................  ....................  ....................  ....................
545 MW454691.1 |Severe acute respiratory syndrome coro    ....................  ....................  ....................  ....................  ....................  ....................
546 MW454692.1 |Severe acute respiratory syndrome coro    ....................  .A..................  ....................  ....................  ....................  ....................
547 MW454693.1 |Severe acute respiratory syndrome coro    ....................  ....................  ....................  ....................  ....................  ....................
548 MW454694.1 |Severe acute respiratory syndrome coro    ....................  ....................  ....................  ....................  ....................  ....................
549 MW454695.1 |Severe acute respiratory syndrome coro    ....................  ....................  ....................  ....................  ....................  ....................
550 MW454696.1 |Severe acute respiratory syndrome coro    ....................  .A..................  ....................  ....................  ....................  ....................
551 MW454697.1 |Severe acute respiratory syndrome coro    ....................  ....................  ....................  ....................  ....................  ....................
552 MW454698.1 |Severe acute respiratory syndrome coro    ....................  ....................  ....................  ....................  ....................  ....................
553 MW454699.1 |Severe acute respiratory syndrome coro    ....................  ....................  ....................  ....................  ....................  ....................
554 MW454700.1 |Severe acute respiratory syndrome coro    ....................  ....................  ....................  ....................  ....................  ....................
555 MW454701.1 |Severe acute respiratory syndrome coro    ....................  ....................  ....................  ....................  ....................  ....................
556 MW454702.1 |Severe acute respiratory syndrome coro    ....................  ....................  ....................  ....................  ....................  ....................
557 MW454704.1 |Severe acute respiratory syndrome coro    ....................  ....................  ....................  ....................  ....................  ....................
558 MW454707.1 |Severe acute respiratory syndrome coro    ....................  ....................  ....................  ....................  ....................  ....................
559 MW454708.1 |Severe acute respiratory syndrome coro    ....................  ....................  ....................  ....................  ....................  ....................
560 MW454709.1 |Severe acute respiratory syndrome coro    ....................  ....................  ....................  ....................  ....................  ....................
561 MW454710.1 |Severe acute respiratory syndrome coro    ....................  ....................  ....................  ....................  ....................  ....................
562 MW454711.1 |Severe acute respiratory syndrome coro    ....................  ....................  ....................  ....................  ....................  ....................
563 MW454712.1 |Severe acute respiratory syndrome coro    ....................  ....................  ....................  ....................  ....................  ....................
564 MW454713.1 |Severe acute respiratory syndrome coro    ....................  ....................  ....................  ....................  ....................  ....................
565 MW454714.1 |Severe acute respiratory syndrome coro    ....................  ....................  ....................  ....................  ....................  ....................
566 MW454715.1 |Severe acute respiratory syndrome coro    ....................  ....................  ....................  ....................  ....................  ....................
567 MW454716.1 |Severe acute respiratory syndrome coro    ....................  ....................  ....................  ....................  ....................  ....................
568 MW454717.1 |Severe acute respiratory syndrome coro    ....................  ....................  ....................  ....................  ....................  ....................
569 MW454718.1 |Severe acute respiratory syndrome coro    ....................  ....................  ....................  ....................  ....................  ....................
570 MW454719.1 |Severe acute respiratory syndrome coro    ....................  .A..................  ....................  ....................  ....................  ....................
571 MW454720.1 |Severe acute respiratory syndrome coro    ....................  ....................  ....................  ....................  ....................  ....................
572 MW454721.1 |Severe acute respiratory syndrome coro    ....................  ....................  ....................  ....................  ....................  ....................
573 MW454722.1 |Severe acute respiratory syndrome coro    ....................  ....................  ....................  ....................  ....................  ....................
574 MW454723.1 |Severe acute respiratory syndrome coro    ....................  ....................  ....................  ....................  ....................  ....................
575 MW454724.1 |Severe acute respiratory syndrome coro    ....................  ....................  ....................  ....................  ....................  ....................
576 MW454725.1 |Severe acute respiratory syndrome coro    ....................  ....................  ....................  ....................  ....................  ....................
577 MW454726.1 |Severe acute respiratory syndrome coro    ....................  ....................  ....................  ....................  ....................  ....................
578 MW454727.1 |Severe acute respiratory syndrome coro    ....................  .A..................  ....................  ....................  ....................  ....................
579 MW454728.1 |Severe acute respiratory syndrome coro    ....................  ....................  ....................  ....................  ....................  ....................
580 MW454729.1 |Severe acute respiratory syndrome coro    ....................  ....................  ....................  ....................  ....................  ....................
581 MW454730.1 |Severe acute respiratory syndrome coro    ....................  ....................  ....................  ....................  ....................  ....................
582 MW454731.1 |Severe acute respiratory syndrome coro    ....................  ....................  ....................  ....................  ....................  ....................
583 MW454732.1 |Severe acute respiratory syndrome coro    ....................  ....................  ....................  ....................  ....................  ....................
584 MW454733.1 |Severe acute respiratory syndrome coro    ....................  ....................  ....................  ....................  ....................  ....................
585 MW454734.1 |Severe acute respiratory syndrome coro    ....................  .A..................  ....................  ....................  ....................  ....................
586 MW454735.1 |Severe acute respiratory syndrome coro    ....................  ....................  ....................  ....................  ....................  ....................
587 MW454736.1 |Severe acute respiratory syndrome coro    ....................  ....................  ....................  ....................  ....................  ....................
588 MW454737.1 |Severe acute respiratory syndrome coro    ....................  .A..................  ....................  ....................  ....................  ....................
589 MW454738.1 |Severe acute respiratory syndrome coro    ....................  .A..................  ....................  ....................  ....................  ....................
590 MW454739.1 |Severe acute respiratory syndrome coro    ....................  ....................  ....................  ....................  ....................  ....................
591 MW454740.1 |Severe acute respiratory syndrome coro    ....................  ....................  ....................  ....................  ....................  ....................
592 MW454741.1 |Severe acute respiratory syndrome coro    ....................  ....................  ....................  ....................  ....................  ....................
593 MW454742.1 |Severe acute respiratory syndrome coro    ....................  .............A......  ....................  ....................  ....................  ....................
594 MW454743.1 |Severe acute respiratory syndrome coro    ....................  ....................  ....................  ....................  ....................  ....................
595 MW454744.1 |Severe acute respiratory syndrome coro    ....................  .A..................  ....................  ....................  ....................  ....................
596 MW454745.1 |Severe acute respiratory syndrome coro    ....................  ....................  ....................  ....................  ....................  ....................
597 MW454746.1 |Severe acute respiratory syndrome coro    ....................  ....................  ....................  ....................  ....................  ....................
598 MW454747.1 |Severe acute respiratory syndrome coro    ....................  ....................  ....................  ....................  ....................  ....................
599 MW454748.1 |Severe acute respiratory syndrome coro    ....................  ....................  ....................  ....................  ....................  ....................
600 MW454749.1 |Severe acute respiratory syndrome coro    ....................  ....................  ....................  ....................  ....................  ....................
601 MW454750.1 |Severe acute respiratory syndrome coro    ....................  ....................  ....................  ....................  ....................  ....................
602 MW454751.1 |Severe acute respiratory syndrome coro    ....................  ....................  ....................  ....................  ....................  ....................
603 MW454752.1 |Severe acute respiratory syndrome coro    ....................  ....................  ....................  ....................  ....................  ....................
604 MW454753.1 |Severe acute respiratory syndrome coro    ....................  ....................  ....................  ....................  ....................  ....................
605 MW454754.1 |Severe acute respiratory syndrome coro    ....................  ....................  ....................  ....................  ....................  ....................
606 MW454755.1 |Severe acute respiratory syndrome coro    ....................  ....................  ....................  ....................  ....................  ....................
607 MW454756.1 |Severe acute respiratory syndrome coro    ....................  ....................  ....................  ....................  ....................  ....................
608 MW454757.1 |Severe acute respiratory syndrome coro    ....................  ....................  ....................  ....................  ....................  ....................
609 MW454758.1 |Severe acute respiratory syndrome coro    ....................  ....................  ....................  ....................  ....................  ....................
610 MW454759.1 |Severe acute respiratory syndrome coro    ....................  ....................  ....................  ....................  ....................  ....................
611 MW454760.1 |Severe acute respiratory syndrome coro    ....................  ....................  ....................  ....................  ....................  ....................
612 MW454761.1 |Severe acute respiratory syndrome coro    ....................  .A..................  ....................  ....................  ....................  ....................
613 MW454762.1 |Severe acute respiratory syndrome coro    ....................  ....................  ....................  ....................  ....................  ....................
614 MW454763.1 |Severe acute respiratory syndrome coro    ....................  ....................  ....................  ....................  ....................  ....................
615 MW454764.1 |Severe acute respiratory syndrome coro    ....................  ....................  ....................  ....................  ....................  ....................
616 MW454765.1 |Severe acute respiratory syndrome coro    ....................  ....................  ....................  ....................  ....................  ....................
```

```
617 MW454766.1 |Severe acute respiratory syndrome coro    ····················  ····················  ····················  ····················  ····················  ····················
618 MW454767.1 |Severe acute respiratory syndrome coro    ····················  ·A··················  ····················  ····················  ····················  ····················
619 MW454768.1 |Severe acute respiratory syndrome coro    ····················  ····················  ····················  ····················  ····················  ····················
620 MW454770.1 |Severe acute respiratory syndrome coro    ····················  ····················  ····················  ····················  ····················  ····················
621 MW454771.1 |Severe acute respiratory syndrome coro    ····················  ····················  ····················  ····················  ····················  ····················
622 MW456917.1 |Severe acute respiratory syndrome coro    ····················  ····················  ····················  ····················  ····················  ····················
623 MW460569.1 |Severe acute respiratory syndrome coro    ····················  ····················  ····················  ····················  ····················  ····················
624 MW460570.1 |Severe acute respiratory syndrome coro    ····················  ····················  ····················  ····················  ····················  ····················
625 MW460572.1 |Severe acute respiratory syndrome coro    ····················  ····················  ····················  ····················  ····················  ····················
626 MW460573.1 |Severe acute respiratory syndrome coro    ····················  ····················  ····················  ····················  ···········C········  ····················
627 MW460574.1 |Severe acute respiratory syndrome coro    ····················  ····················  ····················  ····················  ····················  ····················
628 MW460575.1 |Severe acute respiratory syndrome coro    ····················  ····················  ····················  ····················  ····················  ····················
629 MW460576.1 |Severe acute respiratory syndrome coro    ····················  ····················  ····················  ····················  ····················  ····················
630 MW460578.1 |Severe acute respiratory syndrome coro    ····················  ····················  ····················  ····················  ····················  ····················
631 MW460580.1 |Severe acute respiratory syndrome coro    ····················  ·A··················  ····················  ····················  ····················  ····················
632 MW460582.1 |Severe acute respiratory syndrome coro    ····················  ····················  ····················  ····················  ····················  ····················
633 MW460583.1 |Severe acute respiratory syndrome coro    ····················  ····················  ····················  ····················  ····················  ····················
634 MW460586.1 |Severe acute respiratory syndrome coro    ····················  ····················  ····················  ····················  ····················  ····················
635 MW460587.1 |Severe acute respiratory syndrome coro    ····················  ····················  ····················  ····················  ····················  ····················
636 MW460589.1 |Severe acute respiratory syndrome coro    ····················  ·A··················  ····················  ····················  ····················  ····················
637 MW460593.1 |Severe acute respiratory syndrome coro    ····················  ·A··················  ····················  ····················  ····················  ····················
638 MW460594.1 |Severe acute respiratory syndrome coro    ····················  ·A··················  ····················  ····················  ····················  ····················
639 MW460595.1 |Severe acute respiratory syndrome coro    ····················  ·A··················  ····················  ····················  ····················  ····················
640 MW460596.1 |Severe acute respiratory syndrome coro    ····················  ·A··················  ····················  ····················  ····················  ····················
641 MW460597.1 |Severe acute respiratory syndrome coro    ····················  ····················  ····················  ····················  ····················  ···········T········
642 MW460598.1 |Severe acute respiratory syndrome coro    ····················  ·A··················  ····················  ····················  ····················  ····················
643 MW460601.1 |Severe acute respiratory syndrome coro    ····················  ····················  ····················  ····················  ····················  ····················
644 MW460604.1 |Severe acute respiratory syndrome coro    ····················  ····················  ····················  ····················  ····················  ····················
645 MW460605.1 |Severe acute respiratory syndrome coro    ····················  ····················  ····················  ····················  ····················  ····················
646 MW460611.1 |Severe acute respiratory syndrome coro    ····················  ·A··················  ····················  ····················  ····················  ····················
647 MW460612.1 |Severe acute respiratory syndrome coro    ····················  ·A··················  ····················  ····················  ····················  ····················
648 MW460613.1 |Severe acute respiratory syndrome coro    ····················  ····················  ····················  ····················  ····················  ····················
649 MW460617.1 |Severe acute respiratory syndrome coro    ····················  ····················  ····················  ····················  ····················  ····················
650 MW460618.1 |Severe acute respiratory syndrome coro    ····················  ····················  ····················  ····················  ····················  ····················
651 MW460619.1 |Severe acute respiratory syndrome coro    ····················  ·A··················  ····················  ····················  ····················  ····················
652 MW460620.1 |Severe acute respiratory syndrome coro    ····················  ····················  ····················  ····················  ····················  ····················
653 MW460621.1 |Severe acute respiratory syndrome coro    ····················  ····················  ····················  ····················  ····················  ····················
654 MW460622.1 |Severe acute respiratory syndrome coro    ····················  ····················  ····················  ····················  ····················  ····················
655 MW460623.1 |Severe acute respiratory syndrome coro    ····················  ····················  ····················  ····················  ···········C········  ····················
656 MW460624.1 |Severe acute respiratory syndrome coro    ····················  ····················  ····················  ····················  ····················  ····················
657 MW460627.1 |Severe acute respiratory syndrome coro    ····················  ····················  ····················  ····················  ····················  ····················
658 MW460629.1 |Severe acute respiratory syndrome coro    ····················  ····················  ····················  ····················  ····················  ····················
659 MW460630.1 |Severe acute respiratory syndrome coro    ····················  ····················  ····················  ····················  ····················  ····················
660 MW460631.1 |Severe acute respiratory syndrome coro    ····················  ····················  ····················  ····················  ····················  ····················
661 MW460632.1 |Severe acute respiratory syndrome coro    ····················  ····················  ····················  ····················  ····················  ····················
662 MW460634.1 |Severe acute respiratory syndrome coro    ····················  ····················  ····················  ····················  ····················  ····················
663 MW460636.1 |Severe acute respiratory syndrome coro    ····················  ····················  ····················  ····················  ····················  ····················
664 MW460638.1 |Severe acute respiratory syndrome coro    ····················  ····················  ····················  ····················  ····················  ····················
665 MW460640.1 |Severe acute respiratory syndrome coro    ····················  ····················  ····················  ····················  ····················  ····················
666 MW460641.1 |Severe acute respiratory syndrome coro    ····················  ····················  ····················  ····················  ····················  ····················
667 MW460643.1 |Severe acute respiratory syndrome coro    ····················  ····················  ····················  ····················  ····················  ····················
668 MW460644.1 |Severe acute respiratory syndrome coro    ····················  ····················  ····················  ····················  ····················  ····················
669 MW460647.1 |Severe acute respiratory syndrome coro    ····················  ····················  ····················  ····················  ····················  ····················
670 MW460648.1 |Severe acute respiratory syndrome coro    ····················  ····················  ····················  ····················  ····················  ····················
671 MW460650.1 |Severe acute respiratory syndrome coro    ····················  ····················  ····················  ····················  ··········T·········  ····················
672 MW460654.1 |Severe acute respiratory syndrome coro    ····················  ····················  ····················  ····················  ····················  ····················
673 MW460658.1 |Severe acute respiratory syndrome coro    ····················  ····················  ····················  ····················  ····················  ····················
674 MW460660.1 |Severe acute respiratory syndrome coro    ····················  ····················  ····················  ····················  ····················  ····················
675 MW460663.1 |Severe acute respiratory syndrome coro    ····················  ·A··················  ····················  ····················  ····················  ····················
676 MW460665.1 |Severe acute respiratory syndrome coro    ····················  ····················  ····················  ····················  ····················  ····················
677 MW460666.1 |Severe acute respiratory syndrome coro    ····················  ····················  ····················  ····················  ····················  ····················
678 MW460667.1 |Severe acute respiratory syndrome coro    ····················  ····················  ····················  ····················  ····················  ····················
679 MW460668.1 |Severe acute respiratory syndrome coro    ····················  ·A··················  ····················  ····················  ····················  ····················
680 MW460669.1 |Severe acute respiratory syndrome coro    ····················  ·A··················  ····················  ····················  ····················  ····················
681 MW460670.1 |Severe acute respiratory syndrome coro    ····················  ····················  ····················  ····················  ····················  ····················
682 MW460876.1 |Severe acute respiratory syndrome coro    ····················  ····················  ····················  ····················  ····················  ····················
683 MW460878.1 |Severe acute respiratory syndrome coro    ········T···········  ·A··················  ····················  ····················  ····················  ····················
684 MW460879.1 |Severe acute respiratory syndrome coro    ····················  ·A··················  ····················  ····················  ····················  ····················
685 MW460883.1 |Severe acute respiratory syndrome coro    ····················  ····················  ····················  ····················  ····················  ····················
686 MW460884.1 |Severe acute respiratory syndrome coro    ····················  ····················  ····················  ····················  ····················  ····················
687 MW460885.1 |Severe acute respiratory syndrome coro    ····················  ····················  ····················  ····················  ····················  ····················
688 MW462644.1 |Severe acute respiratory syndrome coro    ····················  ·A··················  ····················  ····················  ····················  ····················
689 MW462645.1 |Severe acute respiratory syndrome coro    ····················  ·A··················  ····················  ····················  ····················  ····················
690 MW462646.1 |Severe acute respiratory syndrome coro    ····················  ·A··················  ····················  ····················  ····················  ····················
691 MW462647.1 |Severe acute respiratory syndrome coro    ····················  ····················  ····················  ····················  ····················  ····················
692 MW462648.1 |Severe acute respiratory syndrome coro    ····················  ·A··················  ····················  ····················  ····················  ····················
693 MW462649.1 |Severe acute respiratory syndrome coro    ····················  ····················  ····················  ····················  ····················  ····················
694 MW462650.1 |Severe acute respiratory syndrome coro    ····················  ····················  ····················  ····················  ····················  ····················
```

```
695 MW462651.1 |Severe acute respiratory syndrome coro   .................... .................... .................... .................... .................... ....................
696 MW462652.1 |Severe acute respiratory syndrome coro   .................... .................... .................... .................... .................... ..R.................
697 MW462654.1 |Severe acute respiratory syndrome coro   .................... .................... .................... .................... .................... ....................
698 MW462655.1 |Severe acute respiratory syndrome coro   .................... .................... .................... .................... .................... ....................
699 MW462656.1 |Severe acute respiratory syndrome coro   .................... .................... .................... .................... .................... ....................
700 MW462657.1 |Severe acute respiratory syndrome coro   .................... .................... .................... .................... .................... ....................
701 MW462658.1 |Severe acute respiratory syndrome coro   .................... .................... .................... .................... .................... ....................
702 MW462659.1 |Severe acute respiratory syndrome coro   .................... .................... .................... .................... .................... ....................
703 MW462660.1 |Severe acute respiratory syndrome coro   .................... .................... .................... .................... .................... ....................
704 MW462661.1 |Severe acute respiratory syndrome coro   .................... .................... .................... .................... .................... ....................
705 MW462662.1 |Severe acute respiratory syndrome coro   .................... .................... .................... .................... .................... ....................
706 MW462663.1 |Severe acute respiratory syndrome coro   .................... .................... .................... .................... .................... ....................
707 MW462664.1 |Severe acute respiratory syndrome coro   .................... .................... .................... .................... .................... ....................
708 MW462665.1 |Severe acute respiratory syndrome coro   .................... .................... .................... .................... .................... ....................
709 MW462666.1 |Severe acute respiratory syndrome coro   .................... .A.................. .................... .................... .................... ....................
710 MW462667.1 |Severe acute respiratory syndrome coro   .................... .................... .................... .................... .................... ....................
711 MW462668.1 |Severe acute respiratory syndrome coro   .................... .................... .................... .................... .................... ....................
712 MW462669.1 |Severe acute respiratory syndrome coro   .................... .................... .................... .................... .................... ....................
713 MW462670.1 |Severe acute respiratory syndrome coro   .................... .A.................. .................... .................... .................... ....................
714 MW462671.1 |Severe acute respiratory syndrome coro   .................... .................... .................... .................... .................... ....................
715 MW462672.1 |Severe acute respiratory syndrome coro   .................... .................... .................... .................... .................... ....................
716 MW462673.1 |Severe acute respiratory syndrome coro   .................... .................... .................... .................... .................... ....................
717 MW462674.1 |Severe acute respiratory syndrome coro   .................... ............A....... .................... .................... .................... ....................
718 MW462675.1 |Severe acute respiratory syndrome coro   .................... .................... .................... .................... .................... ....................
719 MW463056.1 |Severe acute respiratory syndrome coro   .................... .................... .................... .................... .................... ....................
720 MW453103.1 |Severe acute respiratory syndrome coro   .................... .................... .................... .................... .................... ....................
721 MW453106.1 |Severe acute respiratory syndrome coro   .................... .................... .................... .................... .................... ....................
722 MW453107.1 |Severe acute respiratory syndrome coro   .................... .................... .................... .................... .................... ....................
723 MW453108.1 |Severe acute respiratory syndrome coro   .................... .................... .................... .................... .................... ....................
724 MW453109.1 |Severe acute respiratory syndrome coro   .................... .................... .................... .................... .................... ....................
725 MW453110.1 |Severe acute respiratory syndrome coro   .................... .................... .................... .................... .................... ....................
726 MW453111.1 |Severe acute respiratory syndrome coro   .................... .A.................. .................... .................... .................... ....................
727 MW453112.1 |Severe acute respiratory syndrome coro   .................... .................... .................... .................... .................... ....................
728 MW453114.1 |Severe acute respiratory syndrome coro   .................... .................... .................... .................... .................... ....................
729 MW453115.1 |Severe acute respiratory syndrome coro   .................... .A.................. .................... .................... .................... ....................
730 MW453116.1 |Severe acute respiratory syndrome coro   .................... .................... .................... .................... .................... ....................
731 MW453117.1 |Severe acute respiratory syndrome coro   .................... .A.................. .................... .................... .................... ....................
732 MW453118.1 |Severe acute respiratory syndrome coro   .................... .A.................. .................... .................... .................... ....................
733 MW453119.1 |Severe acute respiratory syndrome coro   .................... .................... .................... .................... .................... ....................
734 MW453120.1 |Severe acute respiratory syndrome coro   .................... .................... .................... .................... .................... ....................
735 MW453121.1 |Severe acute respiratory syndrome coro   .................... .A.................. .................... .................... .................... ....................
736 MW453122.1 |Severe acute respiratory syndrome coro   .................... .................... .................... .................... .................... ....................
737 MW453123.1 |Severe acute respiratory syndrome coro   .................... .................... .................... .................... .................... ....................
738 MW453124.1 |Severe acute respiratory syndrome coro   .................... .A.................. .................... .................... .................... ....................
739 MW453126.1 |Severe acute respiratory syndrome coro   .................... .A.................. .................... .................... .................... ....................
740 MW453084.1 |Severe acute respiratory syndrome coro   .................... .................... .................... .................... .................... ....................
741 MW453085.1 |Severe acute respiratory syndrome coro   .................... .................... .................... .................... .................... ....................
742 LR991698.2 |Severe acute respiratory syndrome coro   .................... .................... .................... .................... .................... ....................
743 LR991699.2 |Severe acute respiratory syndrome coro   .................... .................... .................... .................... .................... ....................
744 MW450666.1 |Severe acute respiratory syndrome coro   .................... .................... .................... .................... .................... ....................
745 MW451205.1 |Severe acute respiratory syndrome coro   .................... .................... .................... .................... .................... ....................
746 MW451601.1 |Severe acute respiratory syndrome coro   .................... .................... .................... .................... .................... ....................
747 MW451602.1 |Severe acute respiratory syndrome coro   .................... .................... .................... .................... .................... ....................
748 MW452531.1 |Severe acute respiratory syndrome coro   .................... .................... .................... .................... .................... ....................
749 MW452532.1 |Severe acute respiratory syndrome coro   .................... .................... .................... .................... ..........T......... ....................
750 MW452534.1 |Severe acute respiratory syndrome coro   .................... .................... .................... .................... .................... ....................
751 MW452536.1 |Severe acute respiratory syndrome coro   .................... .................... .................... .................... .................... ....................
752 MW452537.1 |Severe acute respiratory syndrome coro   .................... .................... .................... .................... .................... ....................
753 MW452539.1 |Severe acute respiratory syndrome coro   .................... .................... .................... .................... .................... ....................
754 MW452542.1 |Severe acute respiratory syndrome coro   .................... .................... .................... .................... .................... ....................
755 MW452543.1 |Severe acute respiratory syndrome coro   .................... .................... .................... .................... .................... ....................
756 MW452544.1 |Severe acute respiratory syndrome coro   .................... .................... .................... .................... .................... ....................
757 MW446194.1 |Severe acute respiratory syndrome coro   .................... .................... .................... .................... .................... ....................
758 MW446195.1 |Severe acute respiratory syndrome coro   .................... .................... .................... .................... .................... ....................
759 MW446196.1 |Severe acute respiratory syndrome coro   .................... .................... .................... .................... .................... ....................
760 MW446197.1 |Severe acute respiratory syndrome coro   .................... .................... .................... .................... .................... ....................
761 MW446198.1 |Severe acute respiratory syndrome coro   .................... .................... .................... .................... .................... ....................
762 MW446199.1 |Severe acute respiratory syndrome coro   .................... .................... .................... .................... .................... ....................
763 MW446200.1 |Severe acute respiratory syndrome coro   .................... .................... .................... .................... .................... ....................
764 MW446201.1 |Severe acute respiratory syndrome coro   .................... .................... .................... .................... .................... ....................
765 MW446202.1 |Severe acute respiratory syndrome coro   .................... .................... .................... .................... .................... ....................
766 MW446203.1 |Severe acute respiratory syndrome coro   .................... .................... .................... .................... .................... ....................
767 MW446204.1 |Severe acute respiratory syndrome coro   .................... .................... .................... .................... .................... ....................
768 MW446205.1 |Severe acute respiratory syndrome coro   .................... .................... .................... .................... .................... ....................
769 MW446206.1 |Severe acute respiratory syndrome coro   .................... .................... .................... .................... .................... ....................
770 MW446207.1 |Severe acute respiratory syndrome coro   .................... .................... .................... .................... .................... ....................
771 MW446208.1 |Severe acute respiratory syndrome coro   .................... .................... .................... .................... .................... ....................
772 MW446209.1 |Severe acute respiratory syndrome coro   .................... .A.................. .................... .................... .NN................. ....................
```

773	MW446210.1	Severe acute respiratory syndrome coro	····················	·A··················	····················	····················	····················	····················
774	MW446211.1	Severe acute respiratory syndrome coro	····················	····················	····················	····················	····················	····················
775	MW446212.1	Severe acute respiratory syndrome coro	····················	····················	····················	····················	····················	····················
776	MW446213.1	Severe acute respiratory syndrome coro	····················	·A··················	····················	····················	····················	····················
777	MW446214.1	Severe acute respiratory syndrome coro	····················	····················	····················	····················	····················	····················
778	MW446215.1	Severe acute respiratory syndrome coro	····················	····················	····················	····················	····················	····················
779	MW446216.1	Severe acute respiratory syndrome coro	····················	····················	····················	····················	····················	····················
780	MW446217.1	Severe acute respiratory syndrome coro	····················	····················	····················	····················	····················	····················
781	MW446218.1	Severe acute respiratory syndrome coro	····················	····················	····················	····················	····················	····················
783	MW446220.1	Severe acute respiratory syndrome coro	····················	····················	····················	····················	····················	····················
784	MW446221.1	Severe acute respiratory syndrome coro	····················	·A··················	····················	····················	····················	····················
785	MW446222.1	Severe acute respiratory syndrome coro	····················	····················	····················	····················	····················	····················
786	MW446223.1	Severe acute respiratory syndrome coro	····················	····················	····················	····················	····················	····················
787	MW446224.1	Severe acute respiratory syndrome coro	····················	·A··················	····················	····················	····················	····················
788	MW446225.1	Severe acute respiratory syndrome coro	····················	·A··················	····················	····················	····················	····················
789	MW446226.1	Severe acute respiratory syndrome coro	····················	·A··················	····················	····················	····················	····················
790	MW446227.1	Severe acute respiratory syndrome coro	····················	····················	····················	····················	····················	····················
791	MW446228.1	Severe acute respiratory syndrome coro	····················	·A··················	····················	····················	····················	····················
792	MW446229.1	Severe acute respiratory syndrome coro	····················	····················	····················	····················	····················	····················
793	MW446230.1	Severe acute respiratory syndrome coro	····················	····················	····················	····················	····················	····················
794	MW446231.1	Severe acute respiratory syndrome coro	····················	····················	····················	····················	····················	····················
795	MW446232.1	Severe acute respiratory syndrome coro	····················	····················	····················	····················	····················	····················
796	MW446233.1	Severe acute respiratory syndrome coro	····················	····················	····················	····················	····················	····················
797	MW446234.1	Severe acute respiratory syndrome coro	····················	····················	····················	····················	····················	····················
798	MW446235.1	Severe acute respiratory syndrome coro	····················	····················	····················	····················	····················	····················
799	MW446821.1	Severe acute respiratory syndrome coro	····················	····················	····················	····················	····················	····················
800	MW446822.1	Severe acute respiratory syndrome coro	····················	·A··················	····················	····················	····················	····················
801	MW446823.1	Severe acute respiratory syndrome coro	····················	····················	····················	····················	····················	····················
802	MW446824.1	Severe acute respiratory syndrome coro	····················	····················	····················	····················	····················	····················
803	MW446825.1	Severe acute respiratory syndrome coro	····················	····················	····················	····················	····················	····················
804	MW446826.1	Severe acute respiratory syndrome coro	····················	····················	····················	····················	····················	····················
805	MW446827.1	Severe acute respiratory syndrome coro	····················	····················	····················	····················	····················	····················
806	MW446828.1	Severe acute respiratory syndrome coro	····················	····················	····················	····················	····················	····················
807	MW446829.1	Severe acute respiratory syndrome coro	····················	····················	····················	····················	····················	····················
808	MW446830.1	Severe acute respiratory syndrome coro	····················	····················	····················	····················	····················	····················
809	MW446831.1	Severe acute respiratory syndrome coro	····················	····················	····················	····················	····················	····················
810	MW446832.1	Severe acute respiratory syndrome coro	····················	·A··················	····················	····················	····················	····················
811	MW446833.1	Severe acute respiratory syndrome coro	····················	····················	····················	····················	····················	····················
812	MW446834.1	Severe acute respiratory syndrome coro	····················	····················	····················	····················	····················	····················
813	MW446835.1	Severe acute respiratory syndrome coro	····················	····················	····················	····················	····················	····················
814	MW446836.1	Severe acute respiratory syndrome coro	····················	····················	····················	····················	····················	····················
815	MW446837.1	Severe acute respiratory syndrome coro	····················	·A··················	····················	····················	····················	····················
816	MW446838.1	Severe acute respiratory syndrome coro	····················	····················	····················	····················	····················	····················
817	MW446839.1	Severe acute respiratory syndrome coro	····················	····················	····················	····················	····················	····················
818	MW446840.1	Severe acute respiratory syndrome coro	····················	····················	····················	····················	····················	····················
819	MW446843.1	Severe acute respiratory syndrome coro	····················	····················	····················	····················	····················	····················
820	MW446844.1	Severe acute respiratory syndrome coro	····················	····················	····················	····················	····················	····················
821	MW446846.1	Severe acute respiratory syndrome coro	····················	····················	····················	····················	····················	····················
822	MW446847.1	Severe acute respiratory syndrome coro	····················	···········C········	····················	····················	····················	····················
823	MW446848.1	Severe acute respiratory syndrome coro	····················	···········C········	····················	····················	····················	····················
824	MW446849.1	Severe acute respiratory syndrome coro	····················	····················	····················	····················	····················	····················
825	MW446850.1	Severe acute respiratory syndrome coro	····················	····················	····················	····················	····················	····················
826	MW446851.1	Severe acute respiratory syndrome coro	····················	·A··················	····················	····················	····················	····················
827	MW446852.1	Severe acute respiratory syndrome coro	····················	····················	····················	····················	····················	····················
828	MW446854.1	Severe acute respiratory syndrome coro	····················	···········C········	····················	····················	····················	····················
829	MW446855.1	Severe acute respiratory syndrome coro	····················	···········C········	····················	····················	····················	····················
830	MW446856.1	Severe acute respiratory syndrome coro	····················	····················	····················	····················	····················	····················
831	MW446857.1	Severe acute respiratory syndrome coro	····················	····················	····················	····················	····················	····················
832	MW446858.1	Severe acute respiratory syndrome coro	····················	···········C········	····················	····················	····················	····················
833	MW446859.1	Severe acute respiratory syndrome coro	····················	····················	····················	····················	····················	····················
834	MW446860.1	Severe acute respiratory syndrome coro	····················	·A··················	····················	····················	····················	····················
835	MW446861.1	Severe acute respiratory syndrome coro	····················	·A··················	····················	····················	····················	····················
836	MW446862.1	Severe acute respiratory syndrome coro	····················	····················	····················	····················	····················	····················
837	MW446863.1	Severe acute respiratory syndrome coro	····················	····················	····················	····················	····················	····················
838	MW446864.1	Severe acute respiratory syndrome coro	····················	····················	····················	····················	····················	····················
839	MW446865.1	Severe acute respiratory syndrome coro	····················	····················	····················	····················	····················	····················
840	MW446866.1	Severe acute respiratory syndrome coro	····················	·A··················	····················	····················	····················	····················
841	MW446867.1	Severe acute respiratory syndrome coro	····················	····················	····················	····················	····················	····················
842	MW446868.1	Severe acute respiratory syndrome coro	····················	·A··················	····················	····················	····················	····················
843	MW446869.1	Severe acute respiratory syndrome coro	····················	····················	····················	····················	····················	····················
844	MW446870.1	Severe acute respiratory syndrome coro	····················	····················	····················	····················	····················	····················
845	MW446871.1	Severe acute respiratory syndrome coro	····················	····················	····················	····················	····················	····················
846	MW446872.1	Severe acute respiratory syndrome coro	····················	···········C········	····················	····················	····················	····················
847	MW446873.1	Severe acute respiratory syndrome coro	····················	····················	····················	····················	····················	····················
848	MW446874.1	Severe acute respiratory syndrome coro	····················	····················	····················	····················	····················	····················
849	MW446875.1	Severe acute respiratory syndrome coro	····················	····················	····················	····················	····················	····················
850	MW446876.1	Severe acute respiratory syndrome coro	····················	····················	····················	····················	····················	····················
851	MW446877.1	Severe acute respiratory syndrome coro	····················	····················	····················	····················	····················	····················

852	MW447609.1	|Severe acute respiratory syndrome coro	····················	····················	····················	····················	····················	····················
853	MW447610.1	|Severe acute respiratory syndrome coro	····················	····················	····················	····················	····················	····················
854	MW447611.1	|Severe acute respiratory syndrome coro	····················	····················	····················	····················	····················	····················
855	MW447612.1	|Severe acute respiratory syndrome coro	····················	····················	····················	····················	····················	····················
856	MW447614.1	|Severe acute respiratory syndrome coro	····················	·A··················	····················	····················	····················	··A·················
857	MW447616.1	|Severe acute respiratory syndrome coro	····················	·A··················	····················	····················	····················	··A·················
858	MW447618.1	|Severe acute respiratory syndrome coro	····················	····················	····················	····················	····················	····················
859	MW447619.1	|Severe acute respiratory syndrome coro	····················	·A··················	····················	····················	····················	··A·················
860	MW447620.1	|Severe acute respiratory syndrome coro	····················	····················	····················	····················	····················	····················
861	MW447621.1	|Severe acute respiratory syndrome coro	····················	····················	····················	····················	····················	····················
862	MW447622.1	|Severe acute respiratory syndrome coro	····················	····················	····················	····················	····················	····················
863	MW447623.1	|Severe acute respiratory syndrome coro	····················	····················	····················	····················	····················	····················
864	MW447624.1	|Severe acute respiratory syndrome coro	····················	·A··················	····················	····················	····················	··A·················
865	MW447626.1	|Severe acute respiratory syndrome coro	····················	····················	····················	····················	····················	····················
866	MW447627.1	|Severe acute respiratory syndrome coro	····················	····················	····················	····················	····················	····················
867	MW447630.1	|Severe acute respiratory syndrome coro	····················	····················	····················	····················	····················	····················
868	MW447632.1	|Severe acute respiratory syndrome coro	····················	·A··················	····················	····················	····················	··A·················
869	MW447634.1	|Severe acute respiratory syndrome coro	····················	····················	····················	····················	····················	····················
870	MW447635.1	|Severe acute respiratory syndrome coro	····················	····················	····················	····················	····················	····················
871	MW447636.1	|Severe acute respiratory syndrome coro	····················	····················	····················	····················	····················	····················
872	MW447637.1	|Severe acute respiratory syndrome coro	····················	····················	····················	····················	····················	····················
873	MW447638.1	|Severe acute respiratory syndrome coro	····················	·A··················	····················	····················	····················	··A·················
874	MW447639.1	|Severe acute respiratory syndrome coro	····················	····················	····················	····················	····················	····················
875	MW447640.1	|Severe acute respiratory syndrome coro	····················	·A··················	····················	····················	····················	··A·················
876	MW447641.1	|Severe acute respiratory syndrome coro	····················	····················	····················	····················	····················	····················
877	MW447642.1	|Severe acute respiratory syndrome coro	····················	····················	····················	····················	····················	····················
878	MW447643.1	|Severe acute respiratory syndrome coro	····················	····················	····················	····················	····················	····················
879	MW447644.1	|Severe acute respiratory syndrome coro	····················	····················	····················	····················	····················	····················
880	MW447645.1	|Severe acute respiratory syndrome coro	····················	·A··················	····················	····················	····················	··A·················
881	MW449268.1	|Severe acute respiratory syndrome coro	····················	····················	····················	····················	····················	····················
882	MW449277.1	|Severe acute respiratory syndrome coro	····················	·A··················	····················	····················	····················	····················
883	MW449278.1	|Severe acute respiratory syndrome coro	····················	····················	····················	····················	····················	····················
884	MW449279.1	|Severe acute respiratory syndrome coro	····················	····················	····················	····················	····················	····················
885	MW449281.1	|Severe acute respiratory syndrome coro	····················	·A··················	····················	····················	····················	····················
886	MW449283.1	|Severe acute respiratory syndrome coro	····················	·A··················	····················	····················	····················	····················
887	MW449287.1	|Severe acute respiratory syndrome coro	····················	····················	····················	····················	····················	····················
888	MW449289.1	|Severe acute respiratory syndrome coro	····················	····················	····················	····················	····················	····················
889	MW449290.1	|Severe acute respiratory syndrome coro	····················	·A··················	····················	····················	····················	····················
890	MW449292.1	|Severe acute respiratory syndrome coro	····················	·A··················	····················	····················	····················	····················
891	MW449293.1	|Severe acute respiratory syndrome coro	····················	····················	····················	····················	····················	····················
892	MW449298.1	|Severe acute respiratory syndrome coro	····················	·A··················	····················	····················	····················	····················
893	MW449300.1	|Severe acute respiratory syndrome coro	····················	·A··················	····················	····················	····················	····················
894	MW449301.1	|Severe acute respiratory syndrome coro	····················	·A··················	····················	····················	····················	··A·················
895	MW449303.1	|Severe acute respiratory syndrome coro	····················	····················	··T·················	····················	····················	····················
896	MW449305.1	|Severe acute respiratory syndrome coro	····················	·A··················	····················	····················	····················	····················
897	MW449310.1	|Severe acute respiratory syndrome coro	····················	·A··················	····················	····················	····················	····················
898	MW449312.1	|Severe acute respiratory syndrome coro	····················	····················	····················	····················	····················	····················
899	MW449317.1	|Severe acute respiratory syndrome coro	····················	····················	····················	····················	····················	····················
900	MW449319.1	|Severe acute respiratory syndrome coro	····················	·A··················	····················	····················	····················	····················
901	MW449321.1	|Severe acute respiratory syndrome coro	····················	····················	····················	····················	····················	····················
902	MW449323.1	|Severe acute respiratory syndrome coro	····················	·A··················	····················	····················	····················	····················
903	MW449325.1	|Severe acute respiratory syndrome coro	····················	·A··················	····················	····················	····················	····················
904	MW449326.1	|Severe acute respiratory syndrome coro	····················	····················	····················	····················	····················	····················
905	MW449329.1	|Severe acute respiratory syndrome coro	····················	·A··················	····················	····················	····················	····················
906	MW449331.1	|Severe acute respiratory syndrome coro	····················	····················	····················	····················	····················	····················
907	MW449339.1	|Severe acute respiratory syndrome coro	····················	····················	····················	····················	····················	····················
908	MW449343.1	|Severe acute respiratory syndrome coro	····················	····················	····················	····················	····················	····················
909	MW449346.1	|Severe acute respiratory syndrome coro	····················	·A··················	····················	····················	····················	····················
910	MW449349.1	|Severe acute respiratory syndrome coro	····················	·A··················	····················	····················	····················	····················
911	MW449351.1	|Severe acute respiratory syndrome coro	····················	····················	····················	····················	····················	····················
912	MW449355.1	|Severe acute respiratory syndrome coro	····················	····················	····················	····················	····················	····················
913	MW449356.1	|Severe acute respiratory syndrome coro	····················	·A··················	····················	····················	····················	····················
914	MW449357.1	|Severe acute respiratory syndrome coro	····················	·A··················	····················	····················	····················	····················
915	MW449360.1	|Severe acute respiratory syndrome coro	····················	····················	····················	····················	····················	····················
916	MW449368.1	|Severe acute respiratory syndrome coro	····················	····················	····················	····················	····················	····················
917	MW449369.1	|Severe acute respiratory syndrome coro	····················	····················	····················	····················	····················	····················
918	MW449370.1	|Severe acute respiratory syndrome coro	····················	·A··················	····················	····················	····················	····················
919	MW449372.1	|Severe acute respiratory syndrome coro	····················	····················	····················	····················	····················	····················
920	MW449373.1	|Severe acute respiratory syndrome coro	····················	····················	····················	····················	····················	····················
921	MW449374.1	|Severe acute respiratory syndrome coro	····················	····················	····················	····················	····················	····················
922	MW449376.1	|Severe acute respiratory syndrome coro	····················	····················	····················	····················	····················	····················
923	MW449377.1	|Severe acute respiratory syndrome coro	····················	····················	····················	····················	····················	····················
924	MW449383.1	|Severe acute respiratory syndrome coro	····················	····················	····················	····················	····················	····················
925	MW449386.1	|Severe acute respiratory syndrome coro	····················	····················	····················	····················	····················	····················
926	MW449387.1	|Severe acute respiratory syndrome coro	····················	····················	····················	····················	····················	····················
927	MW449389.1	|Severe acute respiratory syndrome coro	····················	····················	····················	····················	····················	····················
928	MW449392.1	|Severe acute respiratory syndrome coro	····················	·A··················	····················	····················	····················	····················
929	MW449399.1	|Severe acute respiratory syndrome coro	····················	·A··················	····················	····················	····················	····················

930	MW449403.1 |Severe acute respiratory syndrome coro	....	.A..................	....	....	....	....
931	MW449406.1 |Severe acute respiratory syndrome coro	....	.A..................	....	....	....	....
932	MW449407.1 |Severe acute respiratory syndrome coro	....	.A..................	....	....	....	....
933	MW449408.1 |Severe acute respiratory syndrome coro	....	....	....	....	....	....
934	MW449412.1 |Severe acute respiratory syndrome coro	....	.A..................	....	....	....	....
935	MW449414.1 |Severe acute respiratory syndrome coro	....	....	....	....	....	....
936	MW449416.1 |Severe acute respiratory syndrome coro	....	....	....	....	....	....
937	MW449419.1 |Severe acute respiratory syndrome coro	....	....	....	....	....	....
938	MW449421.1 |Severe acute respiratory syndrome coro	....	....	....	....	....	....
939	MW449422.1 |Severe acute respiratory syndrome coro	....	....	....	....	....	....
940	MW449428.1 |Severe acute respiratory syndrome coro	....	.A..................	....	....	....	....
941	MW449429.1 |Severe acute respiratory syndrome coro	....	.A..................	....	....	....	....
942	MW449431.1 |Severe acute respiratory syndrome coro	.......T............	.A..................	....	....	....	....
943	MW449432.1 |Severe acute respiratory syndrome coro	....	....	....	....	....	....
944	MW449434.1 |Severe acute respiratory syndrome coro	....	.A..................	....	....	....	....
945	MW449438.1 |Severe acute respiratory syndrome coro	....	....	....	....	....	....
946	MW449440.1 |Severe acute respiratory syndrome coro	....	.A..................	....	....	....	....
947	MW449448.1 |Severe acute respiratory syndrome coro	....	.A..................	....	....	....	....
948	MW449449.1 |Severe acute respiratory syndrome coro	....	....	....	....	....	....
949	MW449452.1 |Severe acute respiratory syndrome coro	....	....	....	....	....	....
950	MW449453.1 |Severe acute respiratory syndrome coro	....	.A..................	....	....	....	....
951	MW449454.1 |Severe acute respiratory syndrome coro	....	....	....	....	....	....
952	MW449455.1 |Severe acute respiratory syndrome coro	....	.A..................	....	....	....	....
953	MW449457.1 |Severe acute respiratory syndrome coro	....	.A..................	....	....	....	....
954	MW449459.1 |Severe acute respiratory syndrome coro	....	.A..................	....	....	....	....
955	MW449466.1 |Severe acute respiratory syndrome coro	....	....	....	....	....	....
956	MW449467.1 |Severe acute respiratory syndrome coro	....	.A..................	....	....	....	....
957	MW449468.1 |Severe acute respiratory syndrome coro	....	....	....	....	....	....
958	MW449470.1 |Severe acute respiratory syndrome coro	....	....	....	....	....	....
959	MW449477.1 |Severe acute respiratory syndrome coro	....	....	....	....	....	....
960	MW449478.1 |Severe acute respiratory syndrome coro	....	....	....	....	....	....
961	MW449482.1 |Severe acute respiratory syndrome coro	....	....	....	....	....	....
962	MW449494.1 |Severe acute respiratory syndrome coro	.......T............	.A..................	....	....	....	....
963	MW449499.1 |Severe acute respiratory syndrome coro	....	.A..................	....	....	....	....
964	MW449514.1 |Severe acute respiratory syndrome coro	....	.............A......	....	....	....	....
965	MW322968.1 |Severe acute respiratory syndrome coro	....	....	....	....	....	....
966	MW441903.1 |Severe acute respiratory syndrome coro	....	....	....	....	....	....
967	MW441904.1 |Severe acute respiratory syndrome coro	....	....	....	....	....	....
968	MW441905.1 |Severe acute respiratory syndrome coro	....	....	....	....	....	....
969	MW441907.1 |Severe acute respiratory syndrome coro	....	....	....	....	....	....
970	MW441908.1 |Severe acute respiratory syndrome coro	....	....	....	....	....	....
971	MW441909.1 |Severe acute respiratory syndrome coro	....	....	....	....	....	....
972	MW441910.1 |Severe acute respiratory syndrome coro	....	....	....	....	....	....
973	MW441911.1 |Severe acute respiratory syndrome coro	....	....	....	....	....	....
974	MW441912.1 |Severe acute respiratory syndrome coro	....	....	....	....	....	....
975	MW441913.1 |Severe acute respiratory syndrome coro	....	....	....	....	....	....
976	MW441914.1 |Severe acute respiratory syndrome coro	....	....	....	....	....	....
977	MW441915.1 |Severe acute respiratory syndrome coro	....	....	....	....	....	....
978	MW441916.1 |Severe acute respiratory syndrome coro	....	....	....	....	....	....
979	MW441917.1 |Severe acute respiratory syndrome coro	....	....	....	....	....	....
980	MW441918.1 |Severe acute respiratory syndrome coro	....	....	....	....	....	....
981	MW441919.1 |Severe acute respiratory syndrome coro	....	....	....	....	....	....
982	MW441920.1 |Severe acute respiratory syndrome coro	....	....	....	....	....	....
983	MW433744.1 |Severe acute respiratory syndrome coro	....	....	....	....	....	....
984	MW433746.1 |Severe acute respiratory syndrome coro	....	....	....	....	....	....
985	MW433749.1 |Severe acute respiratory syndrome coro	....	....	....	....	....	....
986	MW433750.1 |Severe acute respiratory syndrome coro	....	....	....	....	....	....
987	MW433752.1 |Severe acute respiratory syndrome coro	....	....	....	....	....	....
988	MW433757.1 |Severe acute respiratory syndrome coro	....	....	....	....	....	....
989	MW433758.1 |Severe acute respiratory syndrome coro	....	....	....	....	....	....
990	MW433759.1 |Severe acute respiratory syndrome coro	....	....	....	....	....	....
991	MW433762.1 |Severe acute respiratory syndrome coro	....	.A..................	....	....	....	....
992	MW433763.1 |Severe acute respiratory syndrome coro	....	....	....	....	....	....
993	MW433764.1 |Severe acute respiratory syndrome coro	....	.A..................	....	....	....	....
995	MW433769.1 |Severe acute respiratory syndrome coro	....	....	....	....	....	....
996	MW433771.1 |Severe acute respiratory syndrome coro	....	....	....	....	....	....
997	MW433772.1 |Severe acute respiratory syndrome coro	....	....	....	....	....	....
998	MW435626.1 |Severe acute respiratory syndrome coro	....	.A..................	....	....	....	....
999	MW435630.1 |Severe acute respiratory syndrome coro	....	....	....	....	....	....
1000	MW435638.1 |Severe acute respiratory syndrome coro	....	....	....	....	....	....
1001	MW435642.1 |Severe acute respiratory syndrome coro	....	....	....	....	....	....
1002	MW435649.1 |Severe acute respiratory syndrome coro	....	.A..................	....	....	....	....
1003	MW435658.1 |Severe acute respiratory syndrome coro	....	.A..................	....	....	....	....
1004	MW435661.1 |Severe acute respiratory syndrome coro	....	....	....	....	....	....
1005	MW435666.1 |Severe acute respiratory syndrome coro	....	....	....	....	....	....
1006	MW439257.1 |Severe acute respiratory syndrome coro	....	....	....	....	....	....
1007	MW440397.1 |Severe acute respiratory syndrome coro	....	.A..................	....	....	....	....
1008	MW440410.1 |Severe acute respiratory syndrome coro	....	.A..................	....	....	....	....

1009 MW440433.1 |Severe acute respiratory syndrome coro ....
1010 MW426406.1 |Severe acute respiratory syndrome coro ....
1011 MW428253.1 |Severe acute respiratory syndrome coro ....
1012 MW430964.1 |Severe acute respiratory syndrome coro ....
1013 MW430965.1 |Severe acute respiratory syndrome coro ....
1014 MW430966.1 |Severe acute respiratory syndrome coro ....
1015 MW430970.1 |Severe acute respiratory syndrome coro ....
1016 MW430971.1 |Severe acute respiratory syndrome coro ....
1017 MW430972.1 |Severe acute respiratory syndrome coro ....
1018 MW430973.1 |Severe acute respiratory syndrome coro ....
1019 MW430974.1 |Severe acute respiratory syndrome coro ....
1020 MW430975.1 |Severe acute respiratory syndrome coro ....
1021 MW430976.1 |Severe acute respiratory syndrome coro ....
1022 MW430977.1 |Severe acute respiratory syndrome coro ....
1023 MW430981.1 |Severe acute respiratory syndrome coro ....
1024 MW430983.1 |Severe acute respiratory syndrome coro ....
1025 MW430984.1 |Severe acute respiratory syndrome coro ....
1026 MW430985.1 |Severe acute respiratory syndrome coro ....
1027 MW430988.1 |Severe acute respiratory syndrome coro ....
1028 MW430989.1 |Severe acute respiratory syndrome coro ....
1029 MW430990.1 |Severe acute respiratory syndrome coro ....
1030 MW430991.1 |Severe acute respiratory syndrome coro ....
1031 MT628700.1 |Severe acute respiratory syndrome coro ....
1032 MT628701.1 |Severe acute respiratory syndrome coro ....
1033 MW305250.1 |Severe acute respiratory syndrome coro ....
1034 MW305251.1 |Severe acute respiratory syndrome coro ....
1035 MW425563.1 |Severe acute respiratory syndrome coro ....
1036 MW425708.1 |Severe acute respiratory syndrome coro .... .A.................. ....
1037 MW425715.1 |Severe acute respiratory syndrome coro ....
1038 MW425716.1 |Severe acute respiratory syndrome coro ....A...... ....
1039 MW425719.1 |Severe acute respiratory syndrome coro ....
1040 MW425720.1 |Severe acute respiratory syndrome coro ....T............... ....
1041 MW425722.1 |Severe acute respiratory syndrome coro ....
1042 MW425727.1 |Severe acute respiratory syndrome coro ....
1043 MW425735.1 |Severe acute respiratory syndrome coro ....
1044 MW425741.1 |Severe acute respiratory syndrome coro .... .A.................. ....
1045 MW425743.1 |Severe acute respiratory syndrome coro ....
1046 MW425747.1 |Severe acute respiratory syndrome coro ....
1047 MW425749.1 |Severe acute respiratory syndrome coro ....
1048 MW425750.1 |Severe acute respiratory syndrome coro ....
1049 MW425751.1 |Severe acute respiratory syndrome coro ....
1050 MW425837.1 |Severe acute respiratory syndrome coro ....
1051 MW425851.1 |Severe acute respiratory syndrome coro ....
1052 MW425852.1 |Severe acute respiratory syndrome coro ....
1053 MW425853.1 |Severe acute respiratory syndrome coro ....
1054 MW425854.1 |Severe acute respiratory syndrome coro ....
1055 MW425855.1 |Severe acute respiratory syndrome coro ....
1056 MW425856.1 |Severe acute respiratory syndrome coro ....
1057 MW422884.1 |Severe acute respiratory syndrome coro ....
1058 MW423684.1 |Severe acute respiratory syndrome coro ....
1059 MW423685.1 |Severe acute respiratory syndrome coro ....
1060 MW423686.1 |Severe acute respiratory syndrome coro ....
1061 MW424824.1 |Severe acute respiratory syndrome coro ....
1062 MW424827.1 |Severe acute respiratory syndrome coro ....
1063 MW424832.1 |Severe acute respiratory syndrome coro ....
1064 MW424835.1 |Severe acute respiratory syndrome coro ....
1065 MW424836.1 |Severe acute respiratory syndrome coro ....
1066 MW424837.1 |Severe acute respiratory syndrome coro ....
1067 MW424842.1 |Severe acute respiratory syndrome coro ....
1068 MW424854.1 |Severe acute respiratory syndrome coro ....
1069 MW424857.1 |Severe acute respiratory syndrome coro ....
1070 MW424860.1 |Severe acute respiratory syndrome coro ....
1071 MW424861.1 |Severe acute respiratory syndrome coro .... .A.................. ....
1072 MW424862.1 |Severe acute respiratory syndrome coro ....
1073 MW424863.1 |Severe acute respiratory syndrome coro ....
1074 MW424864.1 |Severe acute respiratory syndrome coro ....
1075 MW424865.1 |Severe acute respiratory syndrome coro ....
1076 MW424867.1 |Severe acute respiratory syndrome coro ....
1077 MW424868.1 |Severe acute respiratory syndrome coro ....
1078 MW424869.1 |Severe acute respiratory syndrome coro ....
1079 MW424871.1 |Severe acute respiratory syndrome coro ....
1080 MW424872.1 |Severe acute respiratory syndrome coro .... .A.................. ....
1081 MW424873.1 |Severe acute respiratory syndrome coro .... .A.................. ....
1082 MW424874.1 |Severe acute respiratory syndrome coro ..A................. ....
1083 MW424875.1 |Severe acute respiratory syndrome coro ....
1084 MW424876.1 |Severe acute respiratory syndrome coro ....
1085 MW424877.1 |Severe acute respiratory syndrome coro ....
1086 MW424878.1 |Severe acute respiratory syndrome coro ....

1087 MW424879.1 |Severe acute respiratory syndrome coro ···················· ···················· ···················· ···················· ···················· ····················
1088 MW424882.1 |Severe acute respiratory syndrome coro ···················· ···················· ···················· ···················· ···················· ····················
1089 MW424883.1 |Severe acute respiratory syndrome coro ···················· ···················· ···················· ···················· ···················· ····················
1090 MW424884.1 |Severe acute respiratory syndrome coro ···················· ···················· ···················· ···················· ···················· ····················
1091 MW424886.1 |Severe acute respiratory syndrome coro ···················· ···················· ···················· ···················· ···················· ····················
1092 MW424892.1 |Severe acute respiratory syndrome coro ···················· ···················· ···················· ···················· ···················· ····················
1093 MW424895.1 |Severe acute respiratory syndrome coro ···················· ·A·················· ···················· ···················· ···················· ····················
1094 MW424897.1 |Severe acute respiratory syndrome coro ···················· ···················· ···················· ···················· ···················· ····················
1095 MW424898.1 |Severe acute respiratory syndrome coro ···················· ···················· ···················· ···················· ···················· ····················
1096 MW424900.1 |Severe acute respiratory syndrome coro ···················· ···················· ···················· ···················· ···················· ····················
1097 MW424901.1 |Severe acute respiratory syndrome coro ···················· ···················· ···················· ···················· ···················· ····················
1098 MW424903.1 |Severe acute respiratory syndrome coro ···················· ···················· ···················· ···················· ···················· ····················
1099 MW424907.1 |Severe acute respiratory syndrome coro ···················· ·A·················· ···················· ···················· ···················· ····················
1100 MW424908.1 |Severe acute respiratory syndrome coro ···················· ·A·················· ···················· ···················· ···················· ····················
1101 MW424909.1 |Severe acute respiratory syndrome coro ···················· ···················· ···················· ···················· ···················· ····················
1102 MW424912.1 |Severe acute respiratory syndrome coro ···················· ···················· ···················· ···················· ···················· ····················
1103 MW424915.1 |Severe acute respiratory syndrome coro ···················· ·A·················· ···················· ···················· ···················· ····················
1104 MW424916.1 |Severe acute respiratory syndrome coro ···················· ···················· ···················· ···················· ···················· ····················
1105 MW424917.1 |Severe acute respiratory syndrome coro ···················· ···················· ···················· ···················· ···················· ····················
1106 MW424923.1 |Severe acute respiratory syndrome coro ···················· ···················· ···················· ···················· ···················· ····················
1107 MW424924.1 |Severe acute respiratory syndrome coro ···················· ···················· ···················· ···················· ···················· ····················
1108 MW424926.1 |Severe acute respiratory syndrome coro ···················· ·····A·············· ···················· ···················· ···················· ····················
1109 MW424929.1 |Severe acute respiratory syndrome coro ···················· ···················· ···················· ···················· ···················· ····················
1110 MW424936.1 |Severe acute respiratory syndrome coro ···················· ·A·················· ···················· ···················· ···················· ····················
1111 MW424937.1 |Severe acute respiratory syndrome coro ···················· ···················· ···················· ···················· ···················· ····················
1112 MW424939.1 |Severe acute respiratory syndrome coro ···················· ·A·················· ···················· ···················· ···················· ····················
1113 MW424940.1 |Severe acute respiratory syndrome coro ···················· ···················· ···················· ···················· ···················· ····················
1114 MW424941.1 |Severe acute respiratory syndrome coro ···················· ···················· ···················· ···················· ···················· ····················
1115 MW424945.1 |Severe acute respiratory syndrome coro ···················· ···················· ···················· ···················· ···················· ····················
1116 MW424946.1 |Severe acute respiratory syndrome coro ···················· ···················· ···················· ···················· ···················· ····················
1117 MW424947.1 |Severe acute respiratory syndrome coro ···················· ·A·················· ···················· ···················· ···················· ····················
1118 MW424948.1 |Severe acute respiratory syndrome coro ···················· ···················· ···················· ···················· ···················· ····················
1119 MW424949.1 |Severe acute respiratory syndrome coro ···················· ···················· ···················· ···················· ···················· ····················
1120 MW424951.1 |Severe acute respiratory syndrome coro ···················· ···················· ···················· ···················· ···················· ····················
1121 MW424953.1 |Severe acute respiratory syndrome coro ···················· ···················· ···················· ···················· ···················· ····················
1122 MW424954.1 |Severe acute respiratory syndrome coro ···················· ···················· ···················· ···················· ···················· ····················
1123 MW424955.1 |Severe acute respiratory syndrome coro ···················· ·A·················· ···················· ···················· ···················· ····················
1124 MW424957.1 |Severe acute respiratory syndrome coro ···················· ···················· ···················· ···················· ···················· ····················
1125 MW424958.1 |Severe acute respiratory syndrome coro ···················· ·A·················· ···················· ···················· ···················· ····················
1126 MW424959.1 |Severe acute respiratory syndrome coro ···················· ···················· ···················· ···················· ···················· ····················
1127 MW424963.1 |Severe acute respiratory syndrome coro ···················· ···················· ···················· ···················· ···················· ····················
1128 MW424967.1 |Severe acute respiratory syndrome coro ···················· ···················· ···················· ···················· ···················· ····················
1129 MW424968.1 |Severe acute respiratory syndrome coro ···················· ···················· ···················· ···················· ···················· ····················
1130 MW424969.1 |Severe acute respiratory syndrome coro ···················· ·A·················· ···················· ···················· ···················· ····················
1131 MW424971.1 |Severe acute respiratory syndrome coro ···················· ···················· ···················· ···················· ···················· ····················
1132 MW424973.1 |Severe acute respiratory syndrome coro ···················· ···················· ···················· ···················· ···················· ····················
1133 MW424977.1 |Severe acute respiratory syndrome coro ···················· ···················· ···················· ···················· ···················· ····················
1134 MW424978.1 |Severe acute respiratory syndrome coro ···················· ···················· ···················· ···················· ···················· ····················
1135 MW424980.1 |Severe acute respiratory syndrome coro ···················· ···················· ···················· ···················· ···················· ····················
1136 MW424981.1 |Severe acute respiratory syndrome coro ···················· ···················· ···················· ···················· ···················· ····················
1137 MW424983.1 |Severe acute respiratory syndrome coro ···················· ···················· ···················· ···················· ···················· ····················
1138 MW424984.1 |Severe acute respiratory syndrome coro ···················· ···················· ···················· ···················· ···················· ····················
1139 MW419981.1 |Severe acute respiratory syndrome coro ···················· ···················· ···················· ···················· ···················· ····················
1140 MW419982.1 |Severe acute respiratory syndrome coro ···················· ···················· ···················· ···················· ···················· ····················
1141 MW419983.1 |Severe acute respiratory syndrome coro ···················· ···················· ···················· ···················· ···················· ····················
1142 MW419984.1 |Severe acute respiratory syndrome coro ···················· ···················· ···················· ···················· ···················· ····················
1143 MW419985.1 |Severe acute respiratory syndrome coro ···················· ···················· ···················· ···················· ···················· ····················
1144 MW419986.1 |Severe acute respiratory syndrome coro ···················· ···················· ···················· ···················· ···················· ····················
1145 MW419987.1 |Severe acute respiratory syndrome coro ···················· ···················· ···················· ···················· ···················· ····················
1146 MW419988.1 |Severe acute respiratory syndrome coro ···················· ···················· ···················· ···················· ···················· ····················
1147 MW419997.1 |Severe acute respiratory syndrome coro ···················· ···················· ···················· ···················· ···················· ····················
1148 MW419999.1 |Severe acute respiratory syndrome coro ···················· ···················· ···················· ···················· ···················· ····················
1149 MW420006.1 |Severe acute respiratory syndrome coro ···················· ···················· ···················· ···················· ···················· ····················
1150 MW420012.1 |Severe acute respiratory syndrome coro ···················· ···················· ···················· ···················· ···················· ····················
1151 MW420023.1 |Severe acute respiratory syndrome coro ···················· ···················· ···················· ···················· ···················· ····················
1152 MW420026.1 |Severe acute respiratory syndrome coro ···················· ···················· ···················· ···················· ···················· ····················
1153 MW420028.1 |Severe acute respiratory syndrome coro ···················· ···················· ···················· ···················· ···················· ····················
1154 MW420032.1 |Severe acute respiratory syndrome coro ···················· ···················· ···················· ···················· ···················· ····················
1155 MW420033.1 |Severe acute respiratory syndrome coro ···················· ···················· ···················· ···················· ···················· ····················
1156 MW420035.1 |Severe acute respiratory syndrome coro ···················· ···················· ···················· ···················· ···················· ····················
1157 MW420038.1 |Severe acute respiratory syndrome coro ···················· ·A·················· ···················· ···················· ···················· ····················
1158 MW420039.1 |Severe acute respiratory syndrome coro ···················· ·A·················· ···················· ···················· ···················· ····················
1159 MW420041.1 |Severe acute respiratory syndrome coro ···················· ···················· ···················· ···················· ···················· ····················
1160 MW420043.1 |Severe acute respiratory syndrome coro ···················· ···················· ···················· ···················· ···················· ····················
1161 MW420047.1 |Severe acute respiratory syndrome coro ···················· ·A·················· ···················· ···················· ···················· ····················
1162 MW420048.1 |Severe acute respiratory syndrome coro ···················· ·A·················· ···················· ···················· ···················· ····················
1163 MW420049.1 |Severe acute respiratory syndrome coro ···················· ···················· ···················· ···················· ···················· ····················
1164 MW420050.1 |Severe acute respiratory syndrome coro ···················· ···················· ···················· ···················· ···················· ····················

1165 MW420051.1 |Severe acute respiratory syndrome coro ···················· ···················· ···················· ···················· ···················· ····················
1166 MW420054.1 |Severe acute respiratory syndrome coro ···················· ···················· ···················· ···················· ···················· ····················
1167 MW420055.1 |Severe acute respiratory syndrome coro ···················· ···················· ···················· ···················· ···················· ····················
1168 MW420060.1 |Severe acute respiratory syndrome coro ···················· ···················· ···················· ···················· ···················· ····················
1169 MW420061.1 |Severe acute respiratory syndrome coro ···················· ·A·················· ···················· ···················· ···················· ····················
1170 MW420065.1 |Severe acute respiratory syndrome coro ···················· ···················· ···················· ···················· ···················· ····················
1171 MW420066.1 |Severe acute respiratory syndrome coro ···················· ···················· ···················· ···················· ···················· ····················
1172 MW420068.1 |Severe acute respiratory syndrome coro ···················· ···················· ···················· ···················· ···················· ····················
1173 MW420069.1 |Severe acute respiratory syndrome coro ···················· ·A·················· ···················· ···················· ···················· ····················
1174 MW420071.1 |Severe acute respiratory syndrome coro ···················· ···················· ···················· ···················· ·········A·········· ····················
1175 MW420072.1 |Severe acute respiratory syndrome coro ···················· ···················· ···················· ···················· ···················· ····················
1176 MW420073.1 |Severe acute respiratory syndrome coro ···················· ···················· ···················· ···················· ···················· ····················
1177 MW420074.1 |Severe acute respiratory syndrome coro ···················· ···················· ···················· ···················· ···················· ····················
1178 MW420076.1 |Severe acute respiratory syndrome coro ···················· ···················· ···················· ···················· ···················· ····················
1179 MW420077.1 |Severe acute respiratory syndrome coro ···················· ···················· ···················· ···················· ···················· ····················
1180 MW420079.1 |Severe acute respiratory syndrome coro ···················· ···················· ···················· ···················· ···················· ····················
1181 MW420080.1 |Severe acute respiratory syndrome coro ···················· ···················· ···················· ···················· ···················· ····················
1182 MW420081.1 |Severe acute respiratory syndrome coro ···················· ···················· ···················· ···················· ···················· ····················
1183 MW420082.1 |Severe acute respiratory syndrome coro ···················· ···················· ···················· ···················· ···················· ····················
1184 MW420084.1 |Severe acute respiratory syndrome coro ···················· ···················· ···················· ···················· ···················· ····················
1185 MW420085.1 |Severe acute respiratory syndrome coro ···················· ···················· ···················· ···················· ···················· ····················
1186 MW420086.1 |Severe acute respiratory syndrome coro ···················· ·A·················· ···················· ···················· ···················· ····················
1187 MW420088.1 |Severe acute respiratory syndrome coro ···················· ···················· ···················· ···················· ···················· ····················
1188 MW420091.1 |Severe acute respiratory syndrome coro ···················· ·A·················· ···················· ···················· ···················· ····················
1189 MW420092.1 |Severe acute respiratory syndrome coro ···················· ···················· ···················· ···················· ···················· ····················
1190 MW420095.1 |Severe acute respiratory syndrome coro ···················· ···················· ···················· ···················· ···················· ····················
1191 MW420096.1 |Severe acute respiratory syndrome coro ···················· ···················· ···················· ···················· ···················· ····················
1192 MW420097.1 |Severe acute respiratory syndrome coro ···················· ···················· ···················· ···················· ···················· ····················
1193 MW420098.1 |Severe acute respiratory syndrome coro ···················· ·A·················· ···················· ···················· ···················· ····················
1194 MW420099.1 |Severe acute respiratory syndrome coro ············T······· ···················· ···················· ···················· ···················· ····················
1195 MW420100.1 |Severe acute respiratory syndrome coro ···················· ···················· ···················· ···················· ···················· ····················
1196 MW420103.1 |Severe acute respiratory syndrome coro ···················· ···················· ···················· ···················· ···················· ····················
1197 MW420104.1 |Severe acute respiratory syndrome coro ···················· ·A·················· ···················· ···················· ···················· ····················
1198 MW420105.1 |Severe acute respiratory syndrome coro ···················· ···················· ···················· ···················· ···················· ····················
1199 MW420109.1 |Severe acute respiratory syndrome coro ···················· ·A·················· ···················· ···················· ···················· ····················
1200 MW420110.1 |Severe acute respiratory syndrome coro ···················· ···················· ···················· ···················· ···················· ····················
1201 MW420111.1 |Severe acute respiratory syndrome coro ···················· ·A·················· ···················· ···················· ···················· ····················
1202 MW420115.1 |Severe acute respiratory syndrome coro ···················· ···················· ···················· ···················· ···················· ····················
1203 MW420121.1 |Severe acute respiratory syndrome coro ···················· ·A·················· ···················· ···················· ···················· ····················
1204 MW420122.1 |Severe acute respiratory syndrome coro ···················· ·A·················· ···················· ···················· ···················· ····················
1205 MW420123.1 |Severe acute respiratory syndrome coro ···················· ···················· ···················· ···················· ···················· ····················
1206 MW420127.1 |Severe acute respiratory syndrome coro ···················· ···················· ···················· ···················· ···················· ····················
1207 MW420128.1 |Severe acute respiratory syndrome coro ···················· ···················· ···················· ···················· ···················· ····················
1208 MW420130.1 |Severe acute respiratory syndrome coro ···················· ···················· ···················· ···················· ···················· ····················
1209 MW420131.1 |Severe acute respiratory syndrome coro ···················· ·A·················· ···················· ···················· ···················· ····················
1210 MW420137.1 |Severe acute respiratory syndrome coro ···················· ·A·················· ···················· ···················· ···················· ····················
1211 MW420138.1 |Severe acute respiratory syndrome coro ···················· ···················· ···················· ···················· ···················· ····················
1212 MW420140.1 |Severe acute respiratory syndrome coro ···················· ···················· ···················· ···················· ···················· ····················
1213 MW420141.1 |Severe acute respiratory syndrome coro ···················· ·A·················· ···················· ···················· ···················· ····················
1214 MW420143.1 |Severe acute respiratory syndrome coro ···················· ···················· ···················· ···················· ···················· ····················
1215 MW420145.1 |Severe acute respiratory syndrome coro ···················· ···················· ···················· ···················· ···················· ····················
1216 MW420146.1 |Severe acute respiratory syndrome coro ···················· ···················· ···················· ···················· ···················· ····················
1217 MW420147.1 |Severe acute respiratory syndrome coro ···················· ···················· ···················· ···················· ···················· ····················
1218 MW420148.1 |Severe acute respiratory syndrome coro ···················· ·A·················· ···················· ···················· ···················· ····················
1219 MW420149.1 |Severe acute respiratory syndrome coro ···················· ···················· ···················· ···················· ···················· ···············A····
1220 MW420150.1 |Severe acute respiratory syndrome coro ···················· ···················· ···················· ···················· ···················· ····················
1221 MW420151.1 |Severe acute respiratory syndrome coro ···················· ·A·················· ···················· ···················· ···················· ····················
1222 MW420154.1 |Severe acute respiratory syndrome coro ···················· ·A····A············· ···················· ···················· ···················· ····················
1223 MW420155.1 |Severe acute respiratory syndrome coro ···················· ···················· ···················· ···················· ···················· ····················
1224 MW420156.1 |Severe acute respiratory syndrome coro ···················· ···················· ···················· ···················· ···················· ····················
1225 MW420157.1 |Severe acute respiratory syndrome coro ···················· ·A·················· ···················· ···················· ···················· ····················
1226 MW420158.1 |Severe acute respiratory syndrome coro ···················· ···················· ···················· ···················· ···················· ····················
1227 MW420160.1 |Severe acute respiratory syndrome coro ···················· ···················· ···················· ···················· ···················· ····················
1228 MW420161.1 |Severe acute respiratory syndrome coro ···················· ···················· ···················· ···················· ···················· ····················
1229 MW420163.1 |Severe acute respiratory syndrome coro ···················· ·A·················· ···················· ···················· ···················· ····················
1230 MW420164.1 |Severe acute respiratory syndrome coro ···················· ·A·················· ···················· ···················· ···················· ····················
1231 MW420165.1 |Severe acute respiratory syndrome coro ···················· ···················· ···················· ···················· ···················· ····················
1232 MW420167.1 |Severe acute respiratory syndrome coro ···················· ···················· ···················· ···················· ···················· ····················
1233 MW420168.1 |Severe acute respiratory syndrome coro ···················· ·A·················· ···················· ···················· ···················· ····················
1234 MW420172.1 |Severe acute respiratory syndrome coro ···················· ···················· ···················· ···················· ···················· ····················
1235 MW420175.1 |Severe acute respiratory syndrome coro ···················· ···················· ···················· ···················· ···················· ····················
1236 MW420179.1 |Severe acute respiratory syndrome coro ···················· ·A·················· ···················· ···················· ···················· ····················
1237 MW420180.1 |Severe acute respiratory syndrome coro ···················· ···················· ···················· ···················· ···················· ····················
1238 MW420181.1 |Severe acute respiratory syndrome coro ···················· ···················· ···················· ···················· ···················· ····················
1239 MW420182.1 |Severe acute respiratory syndrome coro ···················· ···················· ···················· ···················· ···················· ····················
1240 MW420184.1 |Severe acute respiratory syndrome coro ···················· ···················· ···················· ···················· ···················· ····················
1241 MW420187.1 |Severe acute respiratory syndrome coro ···················· ···················· ···················· ···················· ···················· ····················
1242 MW420189.1 |Severe acute respiratory syndrome coro ···················· ···················· ···················· ···················· ···················· ···············A····

1243	MW420190.1	|Severe acute respiratory syndrome coro	····················	····················	····················	····················	····················	····················
1244	MW420192.1	|Severe acute respiratory syndrome coro	····················	·A··················	····················	····················	····················	····················
1245	MW420194.1	|Severe acute respiratory syndrome coro	····················	····················	····················	····················	····················	····················
1246	MW420195.1	|Severe acute respiratory syndrome coro	····················	····················	····················	····················	····················	····················
1247	MW420196.1	|Severe acute respiratory syndrome coro	····················	····················	····················	····················	····················	····················
1248	MW420198.1	|Severe acute respiratory syndrome coro	····················	····················	····················	····················	····················	····················
1249	MW420200.1	|Severe acute respiratory syndrome coro	····················	····················	····················	····················	····················	····················
1250	MW420201.1	|Severe acute respiratory syndrome coro	····················	····················	····················	····················	····················	····················
1251	MW420202.1	|Severe acute respiratory syndrome coro	····················	····················	····················	····················	····················	····················
1252	MW420204.1	|Severe acute respiratory syndrome coro	····················	····················	····················	····················	····················	····················
1253	MW420211.1	|Severe acute respiratory syndrome coro	····················	····················	····················	····················	····················	····················
1254	MW420214.1	|Severe acute respiratory syndrome coro	····················	·A··················	····················	····················	····················	····················
1255	MW420215.1	|Severe acute respiratory syndrome coro	····················	····················	····················	····················	····················	····················
1256	MW420217.1	|Severe acute respiratory syndrome coro	····················	····················	····················	····················	····················	····················
1257	MW420218.1	|Severe acute respiratory syndrome coro	····················	·A··················	····················	····················	····················	····················
1258	MW420219.1	|Severe acute respiratory syndrome coro	····················	····················	····················	····················	····················	····················
1259	MW420220.1	|Severe acute respiratory syndrome coro	····················	····················	····················	····················	····················	····················
1260	MW420221.1	|Severe acute respiratory syndrome coro	····················	····················	····················	····················	····················	····················
1261	MW420223.1	|Severe acute respiratory syndrome coro	····················	·A··················	····················	····················	····················	····················
1262	MW420226.1	|Severe acute respiratory syndrome coro	····················	····················	····················	····················	····················	····················
1263	MW420227.1	|Severe acute respiratory syndrome coro	····················	····················	····················	····················	·····T··············	····················
1264	MW420228.1	|Severe acute respiratory syndrome coro	····················	·A··················	····················	····················	····················	····················
1265	MW420229.1	|Severe acute respiratory syndrome coro	····················	····················	····················	····················	····················	····················
1266	MW420231.1	|Severe acute respiratory syndrome coro	····················	····················	····················	····················	····················	····················
1267	MW420232.1	|Severe acute respiratory syndrome coro	····················	·A··················	····················	····················	····················	····················
1268	MW420234.1	|Severe acute respiratory syndrome coro	····················	····················	····················	····················	····················	····················
1269	MW420235.1	|Severe acute respiratory syndrome coro	····················	·A··················	····················	····················	····················	····················
1270	MW420237.1	|Severe acute respiratory syndrome coro	····················	····················	····················	····················	····················	····················
1271	MW420238.1	|Severe acute respiratory syndrome coro	····················	····················	····················	····················	····················	····················
1272	MW420240.1	|Severe acute respiratory syndrome coro	····················	····················	····················	····················	····················	····················
1273	MW420241.1	|Severe acute respiratory syndrome coro	····················	····················	····················	····················	····················	····················
1274	MW420242.1	|Severe acute respiratory syndrome coro	····················	····················	····················	····················	····················	····················
1275	MW420243.1	|Severe acute respiratory syndrome coro	····················	····················	····················	····················	····················	····················
1276	MW420244.1	|Severe acute respiratory syndrome coro	····················	····················	····················	····················	····················	····················
1277	MW420245.1	|Severe acute respiratory syndrome coro	····················	·A··················	····················	····················	····················	····················
1278	MW420246.1	|Severe acute respiratory syndrome coro	····················	····················	····················	····················	····················	····················
1279	MW420247.1	|Severe acute respiratory syndrome coro	····················	····················	····················	····················	····················	····················
1280	MW420248.1	|Severe acute respiratory syndrome coro	····················	····················	····················	····················	····················	····················
1281	MW420249.1	|Severe acute respiratory syndrome coro	····················	····················	····················	····················	····················	····················
1282	MW420251.1	|Severe acute respiratory syndrome coro	····················	····················	····················	····················	····················	····················
1283	MW420252.1	|Severe acute respiratory syndrome coro	····················	····················	····················	····················	····················	····················
1284	MW420254.1	|Severe acute respiratory syndrome coro	····················	····················	····················	····················	····················	····················
1285	MW420256.1	|Severe acute respiratory syndrome coro	····················	·A··················	····················	····················	····················	····················
1286	MW420257.1	|Severe acute respiratory syndrome coro	····················	····················	····················	····················	····················	····················
1287	MW420258.1	|Severe acute respiratory syndrome coro	····················	····················	····················	····················	····················	····················
1288	MW420261.1	|Severe acute respiratory syndrome coro	····················	····················	····················	····················	····················	····················
1289	MW420262.1	|Severe acute respiratory syndrome coro	····················	····················	····················	····················	····················	····················
1290	MW420264.1	|Severe acute respiratory syndrome coro	····················	····················	····················	····················	····················	····················
1291	MW420266.1	|Severe acute respiratory syndrome coro	····················	····················	····················	····················	····················	····················
1292	MW420267.1	|Severe acute respiratory syndrome coro	····················	····················	····················	····················	····················	····················
1293	MW420270.1	|Severe acute respiratory syndrome coro	····················	·A··················	····················	····················	····················	····················
1294	MW420271.1	|Severe acute respiratory syndrome coro	····················	····················	····················	····················	····················	····················
1295	MW420272.1	|Severe acute respiratory syndrome coro	····················	····················	····················	····················	····················	····················
1296	MW420273.1	|Severe acute respiratory syndrome coro	····················	····················	····················	····················	····················	····················
1297	MW420274.1	|Severe acute respiratory syndrome coro	····················	·A··················	····················	····················	····················	····················
1298	MW420275.1	|Severe acute respiratory syndrome coro	····················	·A··················	····················	····················	····················	····················
1299	MW420276.1	|Severe acute respiratory syndrome coro	····················	····················	····················	····················	····················	····················
1300	MW420278.1	|Severe acute respiratory syndrome coro	····················	·A··················	····················	····················	····················	····················
1301	MW420279.1	|Severe acute respiratory syndrome coro	····················	····················	····················	····················	····················	····················
1302	MW420280.1	|Severe acute respiratory syndrome coro	····················	·A··················	····················	····················	····················	····················
1303	MW420282.1	|Severe acute respiratory syndrome coro	····················	····················	····················	····················	····················	····················
1304	MW420283.1	|Severe acute respiratory syndrome coro	····················	····················	····················	····················	····················	····················
1305	MW420284.1	|Severe acute respiratory syndrome coro	····················	····················	····················	····················	····················	····················
1306	MW420285.1	|Severe acute respiratory syndrome coro	····················	····················	····················	····················	····················	····················
1307	MW420286.1	|Severe acute respiratory syndrome coro	····················	·A··················	····················	····················	····················	····················
1308	MW420288.1	|Severe acute respiratory syndrome coro	····················	····················	····················	····················	····················	····················
1309	MW420291.1	|Severe acute respiratory syndrome coro	····················	····················	····················	····················	····················	····················
1310	MW420294.1	|Severe acute respiratory syndrome coro	····················	····················	····················	····················	····················	····················
1311	MW420295.1	|Severe acute respiratory syndrome coro	····················	····················	····················	····················	····················	····················
1312	MW420296.1	|Severe acute respiratory syndrome coro	····················	·A··················	····················	····················	····················	····················
1313	MW420297.1	|Severe acute respiratory syndrome coro	····················	····················	····················	····················	····················	····················
1314	MW420298.1	|Severe acute respiratory syndrome coro	····················	····················	····················	····················	····················	····················
1315	MW420300.1	|Severe acute respiratory syndrome coro	····················	····················	····················	····················	····················	····················
1316	MW420304.1	|Severe acute respiratory syndrome coro	····················	····················	····················	····················	····················	····················
1317	MW420309.1	|Severe acute respiratory syndrome coro	····················	····················	····················	····················	····················	····················
1318	MW420310.1	|Severe acute respiratory syndrome coro	····················	····················	····················	····················	····················	····················
1319	MW420312.1	|Severe acute respiratory syndrome coro	····················	····················	····················	····················	····················	····················
1320	MW420314.1	|Severe acute respiratory syndrome coro	····················	····················	····················	····················	····················	····················

1321	MW420316.1	|Severe acute respiratory syndrome coro	····················	····················	····················	····················	····················	····················
1322	MW420319.1	|Severe acute respiratory syndrome coro	····················	····················	····················	····················	····················	····················
1323	MW420325.1	|Severe acute respiratory syndrome coro	····················	·A··················	····················	····················	····················	····················
1324	MW420327.1	|Severe acute respiratory syndrome coro	····················	····················	····················	····················	····················	····················
1325	MW420328.1	|Severe acute respiratory syndrome coro	····················	·A··················	····················	····················	····················	····················
1326	MW420329.1	|Severe acute respiratory syndrome coro	····················	····················	····················	····················	····················	····················
1327	MW420330.1	|Severe acute respiratory syndrome coro	····················	····················	····················	····················	····················	····················
1328	MW420334.1	|Severe acute respiratory syndrome coro	····················	····················	····················	····················	····················	····················
1329	MW420336.1	|Severe acute respiratory syndrome coro	····················	····················	····················	····················	····················	····················
1330	MW420337.1	|Severe acute respiratory syndrome coro	····················	····················	····················	····················	····················	····················
1331	MW420339.1	|Severe acute respiratory syndrome coro	····················	·A··················	····················	····················	····················	····················
1332	MW420342.1	|Severe acute respiratory syndrome coro	····················	····················	····················	····················	····················	····················
1333	MW420343.1	|Severe acute respiratory syndrome coro	····················	····················	····················	····················	····················	····················
1334	MW420346.1	|Severe acute respiratory syndrome coro	····················	·A··················	····················	····················	····················	····················
1335	MW420347.1	|Severe acute respiratory syndrome coro	····················	····················	····················	····················	····················	····················
1336	MW420349.1	|Severe acute respiratory syndrome coro	····················	····················	····················	····················	····················	····················
1337	MW420350.1	|Severe acute respiratory syndrome coro	····················	····················	····················	····················	····················	····················
1338	MW420355.1	|Severe acute respiratory syndrome coro	····················	····················	····················	····················	····················	····················
1339	MW420356.1	|Severe acute respiratory syndrome coro	····················	····················	····················	····················	····················	····················
1340	MW420357.1	|Severe acute respiratory syndrome coro	····················	····················	····················	····················	····················	····················
1341	MW420361.1	|Severe acute respiratory syndrome coro	····················	·A··················	····················	····················	····················	····················
1342	MW420362.1	|Severe acute respiratory syndrome coro	····················	····················	····················	····················	····················	····················
1343	MW420366.1	|Severe acute respiratory syndrome coro	····················	····················	····················	····················	····················	····················
1344	MW420367.1	|Severe acute respiratory syndrome coro	····················	····················	····················	····················	····················	····················
1345	MW420368.1	|Severe acute respiratory syndrome coro	····················	····················	····················	····················	····················	····················
1346	MW420370.1	|Severe acute respiratory syndrome coro	····················	····················	····················	····················	····················	····················
1347	MW420371.1	|Severe acute respiratory syndrome coro	····················	····················	····················	····················	····················	····················
1348	MW420372.1	|Severe acute respiratory syndrome coro	····················	····················	····················	····················	····················	····················
1349	MW420373.1	|Severe acute respiratory syndrome coro	····················	····················	····················	····················	····················	····················
1350	MW420374.1	|Severe acute respiratory syndrome coro	····················	····················	····················	····················	····················	····················
1351	MW420375.1	|Severe acute respiratory syndrome coro	····················	····················	····················	····················	····················	····················
1352	MW420377.1	|Severe acute respiratory syndrome coro	····················	····················	····················	····················	····················	····················
1353	MW420378.1	|Severe acute respiratory syndrome coro	····················	····················	····················	····················	····················	····················
1354	MW420379.1	|Severe acute respiratory syndrome coro	····················	·A··················	····················	····················	····················	····················
1355	MW420381.1	|Severe acute respiratory syndrome coro	····················	····················	····················	····················	····················	····················
1356	MW420382.1	|Severe acute respiratory syndrome coro	····················	·A··················	····················	····················	····················	····················
1357	MW420383.1	|Severe acute respiratory syndrome coro	····················	····················	····················	····················	····················	····················
1358	MW420385.1	|Severe acute respiratory syndrome coro	····················	····················	····················	····················	····················	····················
1359	MW420386.1	|Severe acute respiratory syndrome coro	····················	····················	····················	····················	····················	····················
1360	MW420387.1	|Severe acute respiratory syndrome coro	····················	·A··················	····················	····················	····················	····················
1361	MW420388.1	|Severe acute respiratory syndrome coro	····················	·A··················	····················	····················	····················	····················
1362	MW420390.1	|Severe acute respiratory syndrome coro	····················	····················	····················	····················	····················	····················
1363	MW420392.1	|Severe acute respiratory syndrome coro	····················	····················	····················	····················	····················	····················
1364	MW420393.1	|Severe acute respiratory syndrome coro	····················	····················	····················	····················	····················	····················
1365	MW420395.1	|Severe acute respiratory syndrome coro	····················	····················	····················	····················	····················	····················
1366	MW420396.1	|Severe acute respiratory syndrome coro	····················	····················	····················	····················	····················	····················
1367	MW420398.1	|Severe acute respiratory syndrome coro	····················	····················	····················	····················	····················	····················
1368	MW420399.1	|Severe acute respiratory syndrome coro	····················	····················	····················	····················	····················	····················
1369	MW420401.1	|Severe acute respiratory syndrome coro	····················	·A··················	····················	····················	····················	····················
1370	MW420402.1	|Severe acute respiratory syndrome coro	····················	····················	····················	····················	····················	····················
1371	MW420403.1	|Severe acute respiratory syndrome coro	····················	····················	····················	····················	····················	····················
1372	MW420404.1	|Severe acute respiratory syndrome coro	····················	····················	····················	····················	····················	····················
1373	MW420405.1	|Severe acute respiratory syndrome coro	····················	····················	····················	····················	····················	····················
1374	MW420406.1	|Severe acute respiratory syndrome coro	····················	····················	····················	····················	····················	····················
1375	MW420407.1	|Severe acute respiratory syndrome coro	····················	····················	····················	····················	····················	····················
1376	MW420408.1	|Severe acute respiratory syndrome coro	····················	····················	····················	····················	····················	····················
1377	MW420409.1	|Severe acute respiratory syndrome coro	····················	····················	····················	····················	····················	····················
1378	MW420410.1	|Severe acute respiratory syndrome coro	····················	····················	····················	····················	····················	····················
1379	MW420411.1	|Severe acute respiratory syndrome coro	····················	····················	····················	············A·······	····················	····················
1380	MW420414.1	|Severe acute respiratory syndrome coro	····················	····················	····················	····················	····················	····················
1381	MW420415.1	|Severe acute respiratory syndrome coro	····················	·A··················	····················	····················	····················	····················
1382	MW420416.1	|Severe acute respiratory syndrome coro	····················	·A··················	····················	····················	····················	····················
1383	MW420417.1	|Severe acute respiratory syndrome coro	····················	·A··················	····················	····················	····················	····················
1384	MW420418.1	|Severe acute respiratory syndrome coro	····················	····················	····················	····················	····················	····················
1385	MW420419.1	|Severe acute respiratory syndrome coro	····················	·A··················	····················	····················	····················	····················
1386	MW420422.1	|Severe acute respiratory syndrome coro	····················	····················	····················	····················	····················	····················
1387	MW420425.1	|Severe acute respiratory syndrome coro	····················	····················	····················	····················	····················	····················
1388	MW420427.1	|Severe acute respiratory syndrome coro	····················	····················	····················	····················	····················	····················
1389	MW420428.1	|Severe acute respiratory syndrome coro	····················	····················	····················	····················	····················	····················
1390	MW420429.1	|Severe acute respiratory syndrome coro	····················	····················	····················	····················	····················	····················
1391	MW420431.1	|Severe acute respiratory syndrome coro	····················	····················	····················	····················	····················	····················
1392	MW420433.1	|Severe acute respiratory syndrome coro	····················	·A··················	····················	····················	····················	····················
1393	MW420434.1	|Severe acute respiratory syndrome coro	····················	····················	····················	····················	····················	····················
1394	MW420435.1	|Severe acute respiratory syndrome coro	····················	····················	····················	····················	····················	····················
1395	MW420441.1	|Severe acute respiratory syndrome coro	····················	····················	····················	····················	····················	····················
1396	MW420442.1	|Severe acute respiratory syndrome coro	····················	·A···A··············	····················	····················	····················	····················
1397	MW420443.1	|Severe acute respiratory syndrome coro	····················	····················	····················	····················	····················	····················
1398	MW420444.1	|Severe acute respiratory syndrome coro	····················	····················	····················	····················	····················	····················

1399 MW420445.1 |Severe acute respiratory syndrome coro .... .A.................. ....
1400 MW420447.1 |Severe acute respiratory syndrome coro .... .A.................. ....
1401 MW420449.1 |Severe acute respiratory syndrome coro .... .A.................. ....
1402 MW420451.1 |Severe acute respiratory syndrome coro ....
1403 MW420452.1 |Severe acute respiratory syndrome coro ....
1404 MW420457.1 |Severe acute respiratory syndrome coro ....
1405 MW420458.1 |Severe acute respiratory syndrome coro ....
1406 MW420459.1 |Severe acute respiratory syndrome coro .... .A.................. ....
1407 MW420460.1 |Severe acute respiratory syndrome coro .... .A.................. ....
1408 MW420462.1 |Severe acute respiratory syndrome coro .... .A.................. ....
1409 MW420463.1 |Severe acute respiratory syndrome coro ....
1410 MW420464.1 |Severe acute respiratory syndrome coro ....
1411 MW420465.1 |Severe acute respiratory syndrome coro .... .A.................. ....
1412 MW420467.1 |Severe acute respiratory syndrome coro ....
1413 MW420468.1 |Severe acute respiratory syndrome coro ....
1414 MW420470.1 |Severe acute respiratory syndrome coro ....
1415 MW420471.1 |Severe acute respiratory syndrome coro ....
1416 MW420473.1 |Severe acute respiratory syndrome coro ....
1417 MW420477.1 |Severe acute respiratory syndrome coro ....
1418 MW420479.1 |Severe acute respiratory syndrome coro .... .A.................. ....
1419 MW420480.1 |Severe acute respiratory syndrome coro ....
1420 MW420481.1 |Severe acute respiratory syndrome coro ....
1421 MW420482.1 |Severe acute respiratory syndrome coro .... .A.................. ....
1422 MW420483.1 |Severe acute respiratory syndrome coro ....
1423 MW420487.1 |Severe acute respiratory syndrome coro .... .A.................. ....
1424 MW420488.1 |Severe acute respiratory syndrome coro ....
1425 MW420491.1 |Severe acute respiratory syndrome coro ....
1426 MW420492.1 |Severe acute respiratory syndrome coro ....
1427 MW420494.1 |Severe acute respiratory syndrome coro ....
1428 MW420495.1 |Severe acute respiratory syndrome coro ....
1429 MW420497.1 |Severe acute respiratory syndrome coro ....
1430 MW420498.1 |Severe acute respiratory syndrome coro ....
1431 MW420501.1 |Severe acute respiratory syndrome coro ....
1432 MW420503.1 |Severe acute respiratory syndrome coro ....
1433 MW420505.1 |Severe acute respiratory syndrome coro ....
1434 MW420506.1 |Severe acute respiratory syndrome coro ....
1435 MW420507.1 |Severe acute respiratory syndrome coro ....
1436 MW420512.1 |Severe acute respiratory syndrome coro .... .A...A.............. ....
1437 MW420513.1 |Severe acute respiratory syndrome coro .... .A.................. ....
1438 MW420514.1 |Severe acute respiratory syndrome coro ....
1439 MW420517.1 |Severe acute respiratory syndrome coro ....
1440 MW420521.1 |Severe acute respiratory syndrome coro .... .A.................. ....
1441 MW420526.1 |Severe acute respiratory syndrome coro .... .A.................. ....
1442 MW420530.1 |Severe acute respiratory syndrome coro ....
1443 MW420531.1 |Severe acute respiratory syndrome coro .... .A.................. ....
1444 MW420532.1 |Severe acute respiratory syndrome coro ....
1445 MW420533.1 |Severe acute respiratory syndrome coro .... .A.................. ....
1446 MW420535.1 |Severe acute respiratory syndrome coro ....
1447 MW420537.1 |Severe acute respiratory syndrome coro ....
1448 MW420539.1 |Severe acute respiratory syndrome coro .... .A.................. ....
1449 MW420540.1 |Severe acute respiratory syndrome coro ....
1450 MW420541.1 |Severe acute respiratory syndrome coro ....
1451 MW420542.1 |Severe acute respiratory syndrome coro .... .A.................. ....
1452 MW420544.1 |Severe acute respiratory syndrome coro ....
1453 MW420546.1 |Severe acute respiratory syndrome coro ....
1454 MW420547.1 |Severe acute respiratory syndrome coro ....
1455 MW420548.1 |Severe acute respiratory syndrome coro ....
1456 MW420549.1 |Severe acute respiratory syndrome coro ....
1457 MW420550.1 |Severe acute respiratory syndrome coro .... .A.................. ....
1458 MW420553.1 |Severe acute respiratory syndrome coro ....
1459 MW420554.1 |Severe acute respiratory syndrome coro ....
1460 MW420557.1 |Severe acute respiratory syndrome coro .... .A.................. ....
1461 MW420559.1 |Severe acute respiratory syndrome coro .... .A.................. ....
1462 MW420560.1 |Severe acute respiratory syndrome coro .... .A.................. ....
1463 MW420562.1 |Severe acute respiratory syndrome coro ....
1464 MW420565.1 |Severe acute respiratory syndrome coro ....
1465 MW420568.1 |Severe acute respiratory syndrome coro .... .A.................. ....
1466 MW420570.1 |Severe acute respiratory syndrome coro ....
1467 MW420571.1 |Severe acute respiratory syndrome coro ....
1468 MW420572.1 |Severe acute respiratory syndrome coro ....
1469 MW420573.1 |Severe acute respiratory syndrome coro ....
1470 MW420574.1 |Severe acute respiratory syndrome coro ....
1471 MW420575.1 |Severe acute respiratory syndrome coro ....
1472 MW420576.1 |Severe acute respiratory syndrome coro ....
1473 MW420579.1 |Severe acute respiratory syndrome coro ....
1474 MW420582.1 |Severe acute respiratory syndrome coro ....
1475 MW420588.1 |Severe acute respiratory syndrome coro .... .A.................. ....
1476 MW420593.1 |Severe acute respiratory syndrome coro .... .A.................. ....

1477	MW420594.1	Severe acute respiratory syndrome coro	....	....	....	....	....	....
1478	MW420595.1	Severe acute respiratory syndrome coro	....	.A..................	....	....	....	....
1479	MW420599.1	Severe acute respiratory syndrome coro	....	....	....	....	....	....
1480	MW420601.1	Severe acute respiratory syndrome coro	....	.A..................	....	....	....	....
1481	MW420607.1	Severe acute respiratory syndrome coro	....	....	....	....	....	....
1482	MW420609.1	Severe acute respiratory syndrome coro	....	.A..................	....	....	....	....
1483	MW420612.1	Severe acute respiratory syndrome coro	....	.A..................	....	....	....	....
1484	MW420617.1	Severe acute respiratory syndrome coro	....	....	....	....	....	....
1485	MW420621.1	Severe acute respiratory syndrome coro	....	....	....	....	....	....
1486	MW420624.1	Severe acute respiratory syndrome coro	....	....	....	....	....	....
1487	MW420626.1	Severe acute respiratory syndrome coro	....	....	....	....	....	....
1488	MW420628.1	Severe acute respiratory syndrome coro	....	....	....	....	....	....
1489	MW420631.1	Severe acute respiratory syndrome coro	....	.A..................	....	....	....	....
1490	MW420634.1	Severe acute respiratory syndrome coro	....	....	....	....	....	....
1491	MW420638.1	Severe acute respiratory syndrome coro	....	....	....	....	....	....
1492	MW420640.1	Severe acute respiratory syndrome coro	....	....	....	....	....	....
1493	MW420644.1	Severe acute respiratory syndrome coro	....	....	....	....	....	....
1494	MW420645.1	Severe acute respiratory syndrome coro	....	....	....	....	....	....
1495	MW420646.1	Severe acute respiratory syndrome coro	....	....	....	....	....	....
1496	MW420651.1	Severe acute respiratory syndrome coro	....	.A..................	....	....	....	....
1497	MW420653.1	Severe acute respiratory syndrome coro	....	.A..................	....	....	....	....
1498	MW420654.1	Severe acute respiratory syndrome coro	....	....	....	....	....	....
1499	MW420655.1	Severe acute respiratory syndrome coro	....	.A..................	....	....	....	....
1500	MW420656.1	Severe acute respiratory syndrome coro	....	....	....	....	....	....
1501	MW420659.1	Severe acute respiratory syndrome coro	....	....	....	....	....	....
1502	MW420663.1	Severe acute respiratory syndrome coro	....	....	....	....	....	....
1503	MW420664.1	Severe acute respiratory syndrome coro	....	....	....	....	....	....
1504	MW420666.1	Severe acute respiratory syndrome coro	....	....	....	....	....	....
1505	MW420669.1	Severe acute respiratory syndrome coro	....	....	....	....	....	....
1506	MW420671.1	Severe acute respiratory syndrome coro	....	.A..................	....	....	....	....
1507	MW420673.1	Severe acute respiratory syndrome coro	....	.A..................	....	....	....	....
1508	MW420675.1	Severe acute respiratory syndrome coro	....	....	....	....	....	....
1509	MW420679.1	Severe acute respiratory syndrome coro	....	....	....	....	....	....
1510	MW420681.1	Severe acute respiratory syndrome coro	....	....	....	....	....	....
1511	MW420683.1	Severe acute respiratory syndrome coro	....	....	....	....	....	....
1512	MW420684.1	Severe acute respiratory syndrome coro	....	....	....	....	....	....
1513	MW420685.1	Severe acute respiratory syndrome coro	....	....	....	....	....	....
1514	MW420686.1	Severe acute respiratory syndrome coro	....	....	....	....	....	....
1515	MW420687.1	Severe acute respiratory syndrome coro	....	....	....	....	....	....
1516	MW420689.1	Severe acute respiratory syndrome coro	....	....	....	....	....	....
1517	MW420690.1	Severe acute respiratory syndrome coro	....	....	....	....	....	....
1518	MW420691.1	Severe acute respiratory syndrome coro	....	....	....	....	....	....
1519	MW420693.1	Severe acute respiratory syndrome coro	....	....	....	....	....	....
1520	MW420694.1	Severe acute respiratory syndrome coro	....	....	....	....	....	....
1521	MW420695.1	Severe acute respiratory syndrome coro	....	....	....	....	....	....
1522	MW420696.1	Severe acute respiratory syndrome coro	....	....	....	....	....	....
1523	MW420698.1	Severe acute respiratory syndrome coro	....	....	....	....	....	....
1524	MW420701.1	Severe acute respiratory syndrome coro	....	....	....	....	....	....
1525	MW420702.1	Severe acute respiratory syndrome coro	....	....	....	....	....	....
1526	MW420704.1	Severe acute respiratory syndrome coro	....	....	....	....	....	....
1527	MW420705.1	Severe acute respiratory syndrome coro	....	.A..................	....	....	....	....
1528	MW420707.1	Severe acute respiratory syndrome coro	....	....	....	....	....	....
1529	MW420708.1	Severe acute respiratory syndrome coro	....	.A..................	....	....	....	....
1530	MW420709.1	Severe acute respiratory syndrome coro	....	.A..................	....	....	....	....
1531	MW420710.1	Severe acute respiratory syndrome coro	....	....	....	....	....	....
1532	MW420712.1	Severe acute respiratory syndrome coro	....	....	....	....	....	....
1533	MW420716.1	Severe acute respiratory syndrome coro	....	....	....	....	....	....
1534	MW420719.1	Severe acute respiratory syndrome coro	....	....	....	....	....	....
1535	MW420721.1	Severe acute respiratory syndrome coro	....	....	....	....	....	....
1536	MW420722.1	Severe acute respiratory syndrome coro	....	....	....	....	....	....
1537	MW420723.1	Severe acute respiratory syndrome coro	....	.A..................	....	....	....	....
1538	MW420727.1	Severe acute respiratory syndrome coro	....	....	....	....	....	....
1539	MW420728.1	Severe acute respiratory syndrome coro	....	....	....	....	....	....
1540	MW420729.1	Severe acute respiratory syndrome coro	....	....	....	....	....	....
1541	MW420730.1	Severe acute respiratory syndrome coro	....	....	....	....	....	....
1542	MW420731.1	Severe acute respiratory syndrome coro	....	....	....	....	....	....
1543	MW420732.1	Severe acute respiratory syndrome coro	....	.A..................	....	....	....	....
1544	MW420734.1	Severe acute respiratory syndrome coro	....	....	....	....	....	....
1545	MW420735.1	Severe acute respiratory syndrome coro	....	....	....	....	....	....
1546	MW420737.1	Severe acute respiratory syndrome coro	....	.A..................	....	....	....	....
1547	MW420740.1	Severe acute respiratory syndrome coro	....	.A..................	....	....	....	....
1548	MW420741.1	Severe acute respiratory syndrome coro	....	.A..................	....	....	....	....
1549	MW420742.1	Severe acute respiratory syndrome coro	....	....	....	....	....	....
1550	MW420746.1	Severe acute respiratory syndrome coro	....	.A..................	....	....	....	....
1551	MW420747.1	Severe acute respiratory syndrome coro	....	....	....	....	....	....
1552	MW420748.1	Severe acute respiratory syndrome coro	....	....	....	....	....	....
1553	MW420749.1	Severe acute respiratory syndrome coro	....	....	....	....	....	....
1554	MW420751.1	Severe acute respiratory syndrome coro	....	....	....	....	....	....

1555	MW420756.1	|Severe acute respiratory syndrome coro	····················	····················	····················	····················	····················	····················
1556	MW420759.1	|Severe acute respiratory syndrome coro	····················	····················	····················	····················	····················	····················
1557	MW420761.1	|Severe acute respiratory syndrome coro	····················	·A··················	····················	····················	····················	····················
1558	MW420762.1	|Severe acute respiratory syndrome coro	····················	····················	····················	····················	····················	····················
1559	MW420765.1	|Severe acute respiratory syndrome coro	····················	····················	····················	····················	····················	····················
1560	MW420766.1	|Severe acute respiratory syndrome coro	····················	·A··················	····················	····················	····················	····················
1561	MW420769.1	|Severe acute respiratory syndrome coro	····················	····················	····················	····················	····················	····················
1562	MW420772.1	|Severe acute respiratory syndrome coro	····················	····················	····················	····················	····················	····················
1563	MW420774.1	|Severe acute respiratory syndrome coro	····················	····················	····················	····················	····················	····················
1564	MW420776.1	|Severe acute respiratory syndrome coro	····················	····················	····················	····················	····················	····················
1565	MW420777.1	|Severe acute respiratory syndrome coro	····················	·A··················	····················	····················	····················	····················
1566	MW420778.1	|Severe acute respiratory syndrome coro	····················	····················	····················	····················	····················	····················
1567	MW420780.1	|Severe acute respiratory syndrome coro	····················	····················	····················	····················	····················	····················
1568	MW420781.1	|Severe acute respiratory syndrome coro	····················	····················	····················	····················	····················	····················
1569	MW420782.1	|Severe acute respiratory syndrome coro	····················	····················	····················	····················	····················	····················
1570	MW420783.1	|Severe acute respiratory syndrome coro	····················	····················	····················	····················	····················	····················
1571	MW420787.1	|Severe acute respiratory syndrome coro	····················	····················	····················	····················	····················	····················
1572	MW420789.1	|Severe acute respiratory syndrome coro	····················	·A··················	····················	····················	····················	····················
1573	MW420791.1	|Severe acute respiratory syndrome coro	····················	····················	····················	····················	····················	····················
1574	MW420794.1	|Severe acute respiratory syndrome coro	····················	·A··················	····················	····················	····················	····················
1575	MW420795.1	|Severe acute respiratory syndrome coro	····················	····················	····················	····················	····················	····················
1576	MW420796.1	|Severe acute respiratory syndrome coro	····················	····················	····················	····················	····················	····················
1577	MW420797.1	|Severe acute respiratory syndrome coro	····················	····················	····················	····················	····················	····················
1578	MW420798.1	|Severe acute respiratory syndrome coro	····················	····················	····················	····················	····················	····················
1579	MW420801.1	|Severe acute respiratory syndrome coro	····················	····················	····················	····················	····················	····················
1580	MW420802.1	|Severe acute respiratory syndrome coro	····················	····················	····················	····················	····················	····················
1581	MW420804.1	|Severe acute respiratory syndrome coro	····················	····················	····················	····················	····················	····················
1582	MW420805.1	|Severe acute respiratory syndrome coro	····················	····················	····················	····················	····················	····················
1583	MW420809.1	|Severe acute respiratory syndrome coro	····················	····················	····················	····················	····················	····················
1584	MW420814.1	|Severe acute respiratory syndrome coro	····················	····················	····················	····················	····················	····················
1585	MW420816.1	|Severe acute respiratory syndrome coro	····················	····················	····················	····················	····················	····················
1586	MW420818.1	|Severe acute respiratory syndrome coro	····················	····················	····················	····················	····················	····················
1587	MW420819.1	|Severe acute respiratory syndrome coro	····················	····················	····················	····················	····················	····················
1588	MW420826.1	|Severe acute respiratory syndrome coro	····················	····················	····················	····················	····················	····················
1589	MW420827.1	|Severe acute respiratory syndrome coro	····················	·A··················	····················	····················	····················	····················
1590	MW420828.1	|Severe acute respiratory syndrome coro	····················	····················	····················	····················	····················	····················
1591	MW420829.1	|Severe acute respiratory syndrome coro	····················	····················	····················	····················	····················	····················
1592	MW420831.1	|Severe acute respiratory syndrome coro	····················	····················	····················	····················	····················	····················
1593	MW420832.1	|Severe acute respiratory syndrome coro	····················	····················	····················	····················	····················	····················
1594	MW420833.1	|Severe acute respiratory syndrome coro	····················	····················	····················	····················	····················	····················
1595	MW420835.1	|Severe acute respiratory syndrome coro	····················	····················	····················	····················	····················	····················
1596	MW420836.1	|Severe acute respiratory syndrome coro	····················	····················	····················	····················	····················	····················
1597	MW420838.1	|Severe acute respiratory syndrome coro	····················	····················	····················	····················	····················	····················
1598	MW420839.1	|Severe acute respiratory syndrome coro	····················	····················	····················	····················	····················	····················
1599	MW420842.1	|Severe acute respiratory syndrome coro	····················	····················	····················	····················	····················	····················
1600	MW420844.1	|Severe acute respiratory syndrome coro	····················	····················	····················	····················	····················	····················
1601	MW420846.1	|Severe acute respiratory syndrome coro	····················	·A··················	····················	····················	····················	····················
1602	MW420849.1	|Severe acute respiratory syndrome coro	····················	·A··················	····················	····················	····················	····················
1603	MW420851.1	|Severe acute respiratory syndrome coro	····················	····················	····················	····················	····················	····················
1604	MW420852.1	|Severe acute respiratory syndrome coro	····················	····················	····················	····················	····················	····················
1605	MW420854.1	|Severe acute respiratory syndrome coro	····················	·A··················	····················	····················	····················	····················
1606	MW420856.1	|Severe acute respiratory syndrome coro	····················	····················	····················	····················	····················	····················
1607	MW420857.1	|Severe acute respiratory syndrome coro	····················	····················	····················	····················	····················	····················
1608	MW420858.1	|Severe acute respiratory syndrome coro	····················	····················	····················	····················	····················	····················
1609	MW420859.1	|Severe acute respiratory syndrome coro	····················	····················	····················	····················	····················	····················
1610	MW420860.1	|Severe acute respiratory syndrome coro	····················	····················	····················	····················	····················	····················
1611	MW420861.1	|Severe acute respiratory syndrome coro	····················	·A··················	····················	····················	····················	····················
1612	MW420862.1	|Severe acute respiratory syndrome coro	····················	····················	····················	····················	····················	····················
1613	MW420864.1	|Severe acute respiratory syndrome coro	····················	·A··················	····················	····················	····················	····················
1614	MW420865.1	|Severe acute respiratory syndrome coro	····················	····················	····················	····················	····················	····················
1615	MW420866.1	|Severe acute respiratory syndrome coro	····················	·A··················	····················	····················	····················	····················
1616	MW420867.1	|Severe acute respiratory syndrome coro	····················	····················	····················	····················	····················	····················
1617	MW420868.1	|Severe acute respiratory syndrome coro	····················	····················	····················	····················	····················	····················
1618	MW420869.1	|Severe acute respiratory syndrome coro	····················	····················	····················	····················	····················	····················
1619	MW420870.1	|Severe acute respiratory syndrome coro	····················	····················	····················	····················	····················	····················
1620	MW420871.1	|Severe acute respiratory syndrome coro	····················	····················	····················	····················	····················	····················
1621	MW420874.1	|Severe acute respiratory syndrome coro	····················	····················	····················	····················	····················	····················
1622	MW420875.1	|Severe acute respiratory syndrome coro	····················	····················	····················	····················	····················	····················
1623	MW420876.1	|Severe acute respiratory syndrome coro	····················	·A··················	····················	····················	····················	····················
1624	MW420877.1	|Severe acute respiratory syndrome coro	····················	····················	····················	····················	····················	····················
1625	MW420878.1	|Severe acute respiratory syndrome coro	····················	····················	····················	····················	····················	····················
1626	MW420880.1	|Severe acute respiratory syndrome coro	····················	····················	····················	····················	····················	····················
1627	MW420881.1	|Severe acute respiratory syndrome coro	····················	····················	····················	····················	····················	····················
1628	MW420882.1	|Severe acute respiratory syndrome coro	····················	····················	····················	····················	····················	····················
1629	MW420884.1	|Severe acute respiratory syndrome coro	····················	·A··················	····················	····················	····················	····················
1630	MW420885.1	|Severe acute respiratory syndrome coro	····················	·A··················	····················	····················	····················	····················
1631	MW420886.1	|Severe acute respiratory syndrome coro	····················	····················	····················	····················	····················	····················
1632	MW420888.1	|Severe acute respiratory syndrome coro	····················	····················	····················	····················	····················	····················

```
1633 MW420889.1 |Severe acute respiratory syndrome coro   ····················  ····················  ·····················  ····················  ····················  ····················
1634 MW420890.1 |Severe acute respiratory syndrome coro   ····················  ····················  ·····················  ····················  ····················  ····················
1635 MW420891.1 |Severe acute respiratory syndrome coro   ····················  ····················  ·····················  ····················  ····················  ····················
1636 MW420892.1 |Severe acute respiratory syndrome coro   ····················  ····················  ·····················  ····················  ····················  ····················
1637 MW420893.1 |Severe acute respiratory syndrome coro   ····················  ····················  ·····················  ····················  ····················  ····················
1638 MW420894.1 |Severe acute respiratory syndrome coro   ····················  ····················  ·····················  ····················  ····················  ····················
1639 MW420895.1 |Severe acute respiratory syndrome coro   ····················  ····················  ·····················  ····················  ····················  ····················
1640 MW420896.1 |Severe acute respiratory syndrome coro   ····················  ·A··················  ·····················  ····················  ····················  ····················
1641 MW420897.1 |Severe acute respiratory syndrome coro   ····················  ····················  ·····················  ····················  ····················  ····················
1642 MW420898.1 |Severe acute respiratory syndrome coro   ····················  ····················  ·····················  ····················  ····················  ····················
1643 MW420899.1 |Severe acute respiratory syndrome coro   ····················  ·A··················  ·····················  ····················  ····················  ····················
1644 MW420900.1 |Severe acute respiratory syndrome coro   ····················  ····················  ·····················  ····················  ····················  ····················
1645 MW420901.1 |Severe acute respiratory syndrome coro   ····················  ···A················  ·····················  ····················  ····················  ····················
1646 MW420902.1 |Severe acute respiratory syndrome coro   ····················  ····················  ·····················  ····················  ····················  ····················
1647 MW420903.1 |Severe acute respiratory syndrome coro   ····················  ····················  ·····················  ····················  ····················  ····················
1648 MW420904.1 |Severe acute respiratory syndrome coro   ····················  ·A··················  ·····················  ····················  ····················  ····················
1649 MW420905.1 |Severe acute respiratory syndrome coro   ····················  ····················  ·····················  ····················  ····················  ····················
1650 MW420906.1 |Severe acute respiratory syndrome coro   ····················  ····················  ·····················  ····················  ····················  ····················
1651 MW420907.1 |Severe acute respiratory syndrome coro   ····················  ·A··················  ·····················  ····················  ····················  ····················
1652 MW420909.1 |Severe acute respiratory syndrome coro   ····················  ····················  ·····················  ····················  ····················  ····················
1653 MW420910.1 |Severe acute respiratory syndrome coro   ····················  ····················  ·····················  ····················  ····················  ····················
1654 MW420912.1 |Severe acute respiratory syndrome coro   ····················  ·A··················  ·····················  ····················  ····················  ····················
1655 MW420913.1 |Severe acute respiratory syndrome coro   ····················  ····················  ·····················  ····················  ····················  ····················
1656 MW420914.1 |Severe acute respiratory syndrome coro   ····················  ····················  ·····················  ····················  ····················  ····················
1657 MW420916.1 |Severe acute respiratory syndrome coro   ····················  ····················  ·····················  ····················  ····················  ····················
1658 MW420917.1 |Severe acute respiratory syndrome coro   ····················  ····················  ·····················  ····················  ····················  ····················
1659 MW421921.1 |Severe acute respiratory syndrome coro   ····················  ····················  ·····················  ····················  ····················  ····················
1660 MW421927.1 |Severe acute respiratory syndrome coro   ····················  ·A··················  ·····················  ····················  ····················  ····················
1661 MW421928.1 |Severe acute respiratory syndrome coro   ····················  ····················  ·····················  ····················  ····················  ····················
1662 MW421930.1 |Severe acute respiratory syndrome coro   ····················  ····················  ·····················  ····················  ····················  ····················
1663 MW421939.1 |Severe acute respiratory syndrome coro   ····················  ····················  ·····················  ····················  ····················  ····················
1664 MW421942.1 |Severe acute respiratory syndrome coro   ····················  ·A··················  ·····················  ····················  ····················  ····················
1665 MW421949.1 |Severe acute respiratory syndrome coro   ····················  ····················  ·····················  ····················  ····················  ····················
1666 MW421952.1 |Severe acute respiratory syndrome coro   ····················  ····················  ·····················  ····················  ····················  ····················
1667 MW421954.1 |Severe acute respiratory syndrome coro   ····················  ····················  ·····················  ····················  ····················  ····················
1668 MW421957.1 |Severe acute respiratory syndrome coro   ····················  ····················  ·····················  ····················  ····················  ····················
1669 MW421959.1 |Severe acute respiratory syndrome coro   ····················  ····················  ·····················  ····················  ····················  ····················
1670 MW421962.1 |Severe acute respiratory syndrome coro   ····················  ····················  ·····················  ····················  ····················  ····················
1671 MW421966.1 |Severe acute respiratory syndrome coro   ····················  ·A··················  ·····················  ····················  ····················  ····················
1672 MW421973.1 |Severe acute respiratory syndrome coro   ····················  ····················  ·····················  ····················  ····················  ····················
1673 MW421975.1 |Severe acute respiratory syndrome coro   ····················  ····················  ·····················  ····················  ····················  ····················
1674 MW421976.1 |Severe acute respiratory syndrome coro   ····················  ····················  ·····················  ····················  ····················  ····················
1675 MW421977.1 |Severe acute respiratory syndrome coro   ····················  ····················  ·····················  ····················  ····················  ····················
1676 MW422255.1 |Severe acute respiratory syndrome coro   ····················  ····················  ·····················  ····················  ····················  ····················
1677 MW422256.1 |Severe acute respiratory syndrome coro   ····················  ····················  ·····················  ····················  ····················  ····················
1678 MW406485.1 |Severe acute respiratory syndrome coro   ····················  ····················  ·····················  ····················  ····················  ····················
1679 MW406487.1 |Severe acute respiratory syndrome coro   ····················  ·A··················  ·····················  ····················  ····················  ····················
1680 MW406488.1 |Severe acute respiratory syndrome coro   ····················  ·A··················  ·····················  ····················  ····················  ····················
1681 MW406490.1 |Severe acute respiratory syndrome coro   ····················  ·A··················  ·····················  ····················  ····················  ····················
1682 MW406491.1 |Severe acute respiratory syndrome coro   ····················  ····················  ·····················  ····················  ····················  ····················
1683 MW406492.1 |Severe acute respiratory syndrome coro   ····················  ····················  ·····················  ····················  ····················  ····················
1684 MW406493.1 |Severe acute respiratory syndrome coro   ····················  ·A··················  ·····················  ····················  ····················  ····················
1685 MW406494.1 |Severe acute respiratory syndrome coro   ····················  ····················  ·····················  ····················  ····················  ····················
1686 MW406495.1 |Severe acute respiratory syndrome coro   ····················  ·A··················  ·····················  ····················  ····················  ····················
1687 MW406496.1 |Severe acute respiratory syndrome coro   ····················  ····················  ·····················  ····················  ····················  ····················
1688 MW406497.1 |Severe acute respiratory syndrome coro   ····················  ····················  ·····················  ····················  ····················  ····················
1689 MW406498.1 |Severe acute respiratory syndrome coro   ····················  ····················  ·····················  ····················  ····················  ····················
1690 MW406499.1 |Severe acute respiratory syndrome coro   ····················  ····················  ·····················  ····················  ····················  ····················
1691 MW406500.1 |Severe acute respiratory syndrome coro   ····················  ····················  ·····················  ····················  ····················  ····················
1692 MW406501.1 |Severe acute respiratory syndrome coro   ····················  ····················  ·····················  ····················  ····················  ····················
1693 MW406502.1 |Severe acute respiratory syndrome coro   ····················  ·A··················  ·····················  ····················  ····················  ····················
1694 MW406503.1 |Severe acute respiratory syndrome coro   ····················  ····················  ·····················  ····················  ····················  ····················
1695 MW406504.1 |Severe acute respiratory syndrome coro   ····················  ····················  ·····················  ····················  ····················  ····················
1696 MW406505.1 |Severe acute respiratory syndrome coro   ····················  ····················  ·····················  ····················  ····················  ····················
1697 MW406507.1 |Severe acute respiratory syndrome coro   ····················  ····················  ·····················  ····················  ····················  ····················
1698 MW406509.1 |Severe acute respiratory syndrome coro   ····················  ····················  ·····················  ····················  ····················  ····················
1699 MW406511.1 |Severe acute respiratory syndrome coro   ····················  ·············A······  ·····················  ····················  ····················  ····················
1700 MW406512.1 |Severe acute respiratory syndrome coro   ····················  ····················  ·····················  ····················  ····················  ····················
1701 MW406513.1 |Severe acute respiratory syndrome coro   ····················  ····················  ·····················  ····················  ····················  ····················
1702 MW406514.1 |Severe acute respiratory syndrome coro   ····················  ····················  ·····················  ····················  ····················  ····················
1703 MW406515.1 |Severe acute respiratory syndrome coro   ····················  ····················  ·····················  ····················  ····················  ····················
1704 MW406516.1 |Severe acute respiratory syndrome coro   ····················  ····················  ·····················  ····················  ····················  ····················
1705 MW406517.1 |Severe acute respiratory syndrome coro   ····················  ····················  ·····················  ····················  ····················  ····················
1706 MW406518.1 |Severe acute respiratory syndrome coro   ····················  ····················  ·····················  ····················  ····················  ····················
1707 MW406519.1 |Severe acute respiratory syndrome coro   ····················  ·A··················  ·····················  ····················  ····················  ····················
1708 MW406520.1 |Severe acute respiratory syndrome coro   ····················  ····················  ·····················  ····················  ····················  ····················
1709 MW406521.1 |Severe acute respiratory syndrome coro   ····················  ····················  ·····················  ····················  ····················  ····················
1710 MW406522.1 |Severe acute respiratory syndrome coro   ····················  ····················  ·····················  ····················  ····················  ····················
```

1711	MW406523.1	|Severe acute respiratory syndrome coro	····················	····················	····················	····················	····················	····················
1712	MW406524.1	|Severe acute respiratory syndrome coro	····················	·A··················	····················	····················	····················	····················
1713	MW406525.1	|Severe acute respiratory syndrome coro	····················	·A··················	····················	····················	····················	····················
1714	MW406526.1	|Severe acute respiratory syndrome coro	····················	····················	····················	····················	····················	····················
1715	MW406527.1	|Severe acute respiratory syndrome coro	····················	····················	····················	····················	····················	····················
1716	MW406528.1	|Severe acute respiratory syndrome coro	····················	····················	····················	····················	····················	····················
1717	MW406529.1	|Severe acute respiratory syndrome coro	····················	····················	····················	····················	····················	····················
1718	MW406531.1	|Severe acute respiratory syndrome coro	····················	····················	····················	····················	····················	····················
1719	MW406532.1	|Severe acute respiratory syndrome coro	····················	····················	····················	····················	····················	····················
1720	MW406533.1	|Severe acute respiratory syndrome coro	····················	····················	····················	····················	····················	····················
1721	MW406534.1	|Severe acute respiratory syndrome coro	····················	····················	····················	····················	····················	····················
1722	MW406535.1	|Severe acute respiratory syndrome coro	····················	····················	····················	····················	T···················	····················
1723	MW406536.1	|Severe acute respiratory syndrome coro	····················	····················	····················	····················	····················	····················
1724	MW406537.1	|Severe acute respiratory syndrome coro	····················	····················	····················	····················	····················	····················
1725	MW406538.1	|Severe acute respiratory syndrome coro	····················	····················	····················	····················	····················	····················
1726	MW406539.1	|Severe acute respiratory syndrome coro	····················	····················	····················	····················	····················	····················
1727	MW406540.1	|Severe acute respiratory syndrome coro	····················	····················	····················	····················	····················	····················
1728	MW406542.1	|Severe acute respiratory syndrome coro	····················	·A··················	····················	····················	····················	····················
1729	MW406543.1	|Severe acute respiratory syndrome coro	····················	····················	····················	····················	····················	····················
1730	MW406544.1	|Severe acute respiratory syndrome coro	····················	····················	····················	····················	····················	····················
1731	MW406545.1	|Severe acute respiratory syndrome coro	····················	····················	····················	····················	····················	····················
1732	MW406546.1	|Severe acute respiratory syndrome coro	····················	····················	····················	····················	····················	····················
1733	MW406547.1	|Severe acute respiratory syndrome coro	····················	·A··················	····················	····················	····················	····················
1734	MW406548.1	|Severe acute respiratory syndrome coro	····················	·A··················	····················	····················	····················	····················
1735	MW406549.1	|Severe acute respiratory syndrome coro	····················	····················	····················	····················	····················	····················
1736	MW406550.1	|Severe acute respiratory syndrome coro	····················	·A··················	····················	····················	····················	····················
1737	MW406551.1	|Severe acute respiratory syndrome coro	····················	·············A······	····················	····················	····················	····················
1738	MW406552.1	|Severe acute respiratory syndrome coro	····················	····················	····················	····················	····················	····················
1739	MW406553.1	|Severe acute respiratory syndrome coro	····················	·············A······	····················	····················	····················	····················
1740	MW406554.1	|Severe acute respiratory syndrome coro	····················	····················	····················	····················	····················	····················
1741	MW406555.1	|Severe acute respiratory syndrome coro	····················	····················	····················	····················	····················	····················
1742	MW406556.1	|Severe acute respiratory syndrome coro	····················	····················	····················	····················	····················	····················
1743	MW406557.1	|Severe acute respiratory syndrome coro	····················	····················	····················	····················	····················	····················
1744	MW406558.1	|Severe acute respiratory syndrome coro	····················	····················	····················	····················	·T··················	····················
1745	MW406559.1	|Severe acute respiratory syndrome coro	····················	····················	····················	····················	····················	····················
1746	MW406560.1	|Severe acute respiratory syndrome coro	····················	····················	····················	····················	····················	····················
1747	MW406564.1	|Severe acute respiratory syndrome coro	····················	····················	····················	····················	····················	····················
1748	MW406565.1	|Severe acute respiratory syndrome coro	····················	·············A······	····················	····················	····················	····················
1749	MW406566.1	|Severe acute respiratory syndrome coro	····················	····················	····················	····················	····················	····················
1750	MW406568.1	|Severe acute respiratory syndrome coro	····················	·············M······	····················	····················	····················	····················
1751	MW406569.1	|Severe acute respiratory syndrome coro	····················	····················	····················	····················	····················	····················
1752	MW406570.1	|Severe acute respiratory syndrome coro	····················	·A··················	····················	····················	····················	····················
1753	MW406571.1	|Severe acute respiratory syndrome coro	····················	····················	····················	····················	····················	····················
1754	MW406572.1	|Severe acute respiratory syndrome coro	····················	····················	····················	····················	····················	····················
1755	MW406573.1	|Severe acute respiratory syndrome coro	····················	·A··················	····················	····················	····················	····················
1756	MW406574.1	|Severe acute respiratory syndrome coro	····················	····················	····················	····················	····················	····················
1757	MW406577.1	|Severe acute respiratory syndrome coro	····················	····················	····················	····················	····················	····················
1758	MW406580.1	|Severe acute respiratory syndrome coro	····················	····················	····················	····················	····················	····················
1759	MW406581.1	|Severe acute respiratory syndrome coro	····················	····················	····················	····················	····················	····················
1760	MW406584.1	|Severe acute respiratory syndrome coro	····················	····················	····················	····················	····················	····················
1761	MW406586.1	|Severe acute respiratory syndrome coro	····················	····················	····················	····················	····················	····················
1762	MW406587.1	|Severe acute respiratory syndrome coro	····················	····················	····················	····················	····················	····················
1763	MW406588.1	|Severe acute respiratory syndrome coro	····················	····················	····················	····················	····················	····················
1764	MW406590.1	|Severe acute respiratory syndrome coro	····················	····················	····················	····················	····················	····················
1765	MW406591.1	|Severe acute respiratory syndrome coro	····················	····················	····················	····················	····················	····················
1766	MW406594.1	|Severe acute respiratory syndrome coro	····················	····················	····················	····················	····················	····················
1767	MW406595.1	|Severe acute respiratory syndrome coro	····················	····················	····················	····················	····················	····················
1768	MW406597.1	|Severe acute respiratory syndrome coro	····················	····················	····················	····················	····················	····················
1769	MW406604.1	|Severe acute respiratory syndrome coro	····················	····················	····················	····················	····················	····················
1770	MW406608.1	|Severe acute respiratory syndrome coro	····················	·R··················	····················	····················	····················	····················
1771	MW406610.1	|Severe acute respiratory syndrome coro	····················	·R··················	····················	····················	····················	····················
1772	MW406613.1	|Severe acute respiratory syndrome coro	····················	····················	····················	····················	····················	····················
1773	MW406618.1	|Severe acute respiratory syndrome coro	····················	·A··················	····················	····················	····················	····················
1774	MW406619.1	|Severe acute respiratory syndrome coro	····················	····················	····················	····················	····················	····················
1775	MW406620.1	|Severe acute respiratory syndrome coro	····················	····················	····················	····················	····················	····················
1776	MW406627.1	|Severe acute respiratory syndrome coro	····················	····················	····················	····················	····················	····················
1777	MW406628.1	|Severe acute respiratory syndrome coro	····················	····················	····················	····················	····················	····················
1778	MW406629.1	|Severe acute respiratory syndrome coro	····················	····················	····················	····················	····················	····················
1779	MW406631.1	|Severe acute respiratory syndrome coro	····················	····················	····················	····················	····················	····················
1780	MW406633.1	|Severe acute respiratory syndrome coro	····················	····················	····················	····················	····················	····················
1781	MW406639.1	|Severe acute respiratory syndrome coro	····················	····················	····················	····················	····················	····················
1782	MW406642.1	|Severe acute respiratory syndrome coro	····················	·R··················	····················	····················	····················	····················
1783	MW406643.1	|Severe acute respiratory syndrome coro	····················	····················	····················	····················	····················	····················
1784	MW406644.1	|Severe acute respiratory syndrome coro	····················	·R··················	····················	····················	····················	····················
1785	MW411627.1	|Severe acute respiratory syndrome coro	····················	····················	····················	····················	····················	····················
1786	MW411628.1	|Severe acute respiratory syndrome coro	····················	····················	····················	····················	····················	····················
1787	MW411629.1	|Severe acute respiratory syndrome coro	····················	····················	····················	····················	····················	····················
1788	MW411630.1	|Severe acute respiratory syndrome coro	····················	····················	····················	····················	····················	····················

1789 MW411631.1 |Severe acute respiratory syndrome coro ···················· ···················· ···················· ···················· ···················· ····················
1790 MW411632.1 |Severe acute respiratory syndrome coro ···················· ···················· ···················· ···················· ···················· ····················
1791 MW411633.1 |Severe acute respiratory syndrome coro ···················· ···················· ···················· ···················· ···················· ····················
1792 MW411634.1 |Severe acute respiratory syndrome coro ···················· ···················· ···················· ···················· ···················· ····················
1793 MW411635.1 |Severe acute respiratory syndrome coro ···················· ·A·················· ···················· ···················· ···················· ····················
1794 MW411636.1 |Severe acute respiratory syndrome coro ···················· ···················· ···················· ···················· ···················· ····················
1795 MW411637.1 |Severe acute respiratory syndrome coro ···················· ·A·················· ···················· ···················· ···················· ····················
1796 MW411638.1 |Severe acute respiratory syndrome coro ···················· ···················· ···················· ···················· ···················· ····················
1797 MW411639.1 |Severe acute respiratory syndrome coro ···················· ···················· ···················· ···················· ···················· ····················
1798 MW411640.1 |Severe acute respiratory syndrome coro ···················· ·A·················· ···················· ···················· ···················· ····················
1799 MW411641.1 |Severe acute respiratory syndrome coro ···················· ···················· ···················· ···················· ·········T·········· ····················
1800 MW411642.1 |Severe acute respiratory syndrome coro ···················· ·A·················· ···················· ···················· ···················· ····················
1801 MW411643.1 |Severe acute respiratory syndrome coro ···················· ·A·················· ···················· ···················· ···················· ····················
1802 MW411644.1 |Severe acute respiratory syndrome coro ···················· ·A·················· ···················· ···················· ···················· ····················
1803 MW411645.1 |Severe acute respiratory syndrome coro ···················· ·A·················· ···················· ···················· ···················· ····················
1804 MW411646.1 |Severe acute respiratory syndrome coro ···················· ···················· ···················· ···················· ···················· ····················
1805 MW411647.1 |Severe acute respiratory syndrome coro ···················· ···················· ···················· ···················· ···················· ····················
1806 MW411648.1 |Severe acute respiratory syndrome coro ···················· ·A·················· ···················· ···················· ···················· ····················
1807 MW411649.1 |Severe acute respiratory syndrome coro ···················· ····A··············· ···················· ···················· ···················· ····················
1808 MW411650.1 |Severe acute respiratory syndrome coro ···················· ···················· ···················· ···················· ···················· ····················
1809 MW411651.1 |Severe acute respiratory syndrome coro ···················· ···················· ···················· ···················· ···················· ····················
1810 MW411652.1 |Severe acute respiratory syndrome coro ···················· ···················· ···················· ···················· ···················· ····················
1811 MW411653.1 |Severe acute respiratory syndrome coro ···················· ···················· ···················· ···················· ···················· ····················
1812 MW411654.1 |Severe acute respiratory syndrome coro ···················· ···················· ···················· ···················· ···················· ····················
1813 MW411655.1 |Severe acute respiratory syndrome coro ···················· ···················· ···················· ···················· ···················· ····················
1814 MW411656.1 |Severe acute respiratory syndrome coro ···················· ···················· ···················· ···················· ···················· ····················
1815 MW411871.1 |Severe acute respiratory syndrome coro ···················· ···················· ···················· ···················· ···················· ····················
1816 MW411872.1 |Severe acute respiratory syndrome coro ···················· ·A·················· ···················· ···················· ···················· ····················
1817 MW411873.1 |Severe acute respiratory syndrome coro ···················· ·A·················· ···················· ···················· ···················· ····················
1818 MW411874.1 |Severe acute respiratory syndrome coro ···················· ···················· ···················· ···················· ···················· ····················
1819 MW411875.1 |Severe acute respiratory syndrome coro ···················· ·A·················· ···················· ···················· ···················· ····················
1820 MW411876.1 |Severe acute respiratory syndrome coro ···················· ·A·················· ···················· ···················· ···················· ···A················
1821 MW411877.1 |Severe acute respiratory syndrome coro ···················· ·A·················· ···················· ···················· ···················· ···A················
1822 MW411878.1 |Severe acute respiratory syndrome coro ···················· ·A·················· ···················· ···················· ···················· ····················
1823 MW411879.1 |Severe acute respiratory syndrome coro ···················· ···················· ···················· ···················· ···················· ····················
1824 MW411880.1 |Severe acute respiratory syndrome coro ···················· ···················· ···················· ···················· ···················· ····················
1825 MW411881.1 |Severe acute respiratory syndrome coro ···················· ·A·················· ···················· ···················· ···················· ····················
1826 MW411882.1 |Severe acute respiratory syndrome coro ···················· ·A·················· ···················· ···················· ···················· ···A················
1827 MW411883.1 |Severe acute respiratory syndrome coro ···················· ···················· ···················· ···················· ···················· ····················
1828 MW411884.1 |Severe acute respiratory syndrome coro ···················· ·A·················· ···················· ···················· ···················· ····················
1829 MW411885.1 |Severe acute respiratory syndrome coro ···················· ···················· ···················· ···················· ···················· ····················
1830 MW411886.1 |Severe acute respiratory syndrome coro ···················· ···················· ···················· ···················· ···················· ····················
1831 MW411887.1 |Severe acute respiratory syndrome coro ··················A· ···················· ···················· ···················· ···················· ···················· 1
1832 MW411888.1 |Severe acute respiratory syndrome coro ···················· ···················· ···················· ···················· ···················· ····················
1833 MW411889.1 |Severe acute respiratory syndrome coro ···················· ···················· ···················· ···················· ···················· ····················
1834 MW411890.1 |Severe acute respiratory syndrome coro ···················· ···················· ···················· ···················· ···················· ····················
1835 MW411891.1 |Severe acute respiratory syndrome coro ···················· ·A·················· ···················· ···················· ···················· ····················
1836 MW411892.1 |Severe acute respiratory syndrome coro ···················· ···················· ···················· ···················· ···················· ····················
1837 MW411893.1 |Severe acute respiratory syndrome coro ···················· ···················· ···················· ···················· ···················· ····················
1838 MW411894.1 |Severe acute respiratory syndrome coro ···················· ·A·················· ···················· ···················· ···················· ····················
1839 MW411895.1 |Severe acute respiratory syndrome coro ···················· ···················· ···················· ···················· ···················· ····················
1840 MW411896.1 |Severe acute respiratory syndrome coro ···················· ···················· ···················· ···················· ···················· ····················
1841 MW411897.1 |Severe acute respiratory syndrome coro ···················· ·A·················· ···················· ···················· ···················· ····················
1842 MW411898.1 |Severe acute respiratory syndrome coro ···················· ···················· ···················· ···················· ···················· ····················
1843 MW411899.1 |Severe acute respiratory syndrome coro ···················· ···················· ···················· ···················· ···················· ····················
1844 MW411900.1 |Severe acute respiratory syndrome coro ···················· ·A·················· ···················· ···················· ···················· ····················
1845 MW411901.1 |Severe acute respiratory syndrome coro ···················· ···················· ···················· ···················· ···················· ····················
1846 MW411903.1 |Severe acute respiratory syndrome coro ···················· ···················· ···················· ···················· ···················· ····················
1847 MW411904.1 |Severe acute respiratory syndrome coro ···················· ···················· ···················· ···················· ···················· ····················
1848 MW411905.1 |Severe acute respiratory syndrome coro ···················· ···················· ···················· ···················· ···················· ····················
1849 MW411906.1 |Severe acute respiratory syndrome coro ···················· ···················· ···················· ···················· ···················· ····················
1850 MW411907.1 |Severe acute respiratory syndrome coro ···················· ···················· ···················· ···················· ···················· ····················
1851 MW411908.1 |Severe acute respiratory syndrome coro ···················· ···················· ···················· ···················· ···················· ····················
1852 MW411909.1 |Severe acute respiratory syndrome coro ···················· ···················· ···················· ···················· ···················· ····················
1853 MW411910.1 |Severe acute respiratory syndrome coro ···················· ···················· ···················· ···················· ···················· ····················
1854 MW411911.1 |Severe acute respiratory syndrome coro ···················· ···················· ···················· ···················· ···················· ····················
1855 MW411912.1 |Severe acute respiratory syndrome coro ···················· ·A·················· ···················· ···················· ···················· ····················
1856 MW411913.1 |Severe acute respiratory syndrome coro ···················· ·A·················· ···················· ···················· ···················· ····················
1857 MW411914.1 |Severe acute respiratory syndrome coro ···················· ·A·················· ···················· ···················· ···················· ····················
1858 MW411915.1 |Severe acute respiratory syndrome coro ···················· ·A·················· ···················· ···················· ···················· ····················
1859 MW411916.1 |Severe acute respiratory syndrome coro ···················· ·A·················· ···················· ···················· ···················· ····················
1860 MW411917.1 |Severe acute respiratory syndrome coro ···················· ·A·················· ···················· ···················· ···················· ····················
1861 MW411918.1 |Severe acute respiratory syndrome coro ···················· ·A·················· ···················· ···················· ···················· ····················
1862 MW411919.1 |Severe acute respiratory syndrome coro ···················· ···················· ···················· ···················· ·········T·········· ····················
1863 MW411920.1 |Severe acute respiratory syndrome coro ···················· ···················· ···················· ···················· ···················· ····················
1864 MW411921.1 |Severe acute respiratory syndrome coro ···················· ···················· ···················· ···················· ···················· ····················
1865 MW411922.1 |Severe acute respiratory syndrome coro ···················· ···················· ···················· ···················· ···················· ····················
1866 MW411923.1 |Severe acute respiratory syndrome coro ···················· ···················· ···················· ···················· ···················· ····················

1867 MW411924.1 |Severe acute respiratory syndrome coro ···················· ·A····A············· ···················· ···················· ···················· ····················
1868 MW411926.1 |Severe acute respiratory syndrome coro ···················· ···················· ···················· ···················· ···················· ····················
1869 MW411928.1 |Severe acute respiratory syndrome coro ···················· ·A·················· ···················· ···················· ···················· ····················
1870 MW411929.1 |Severe acute respiratory syndrome coro ···················· ·A·················· ···················· ···················· ···················· ····················
1871 MW411931.1 |Severe acute respiratory syndrome coro ···················· ···················· ···················· ···················· ···················· ····················
1872 MW411932.1 |Severe acute respiratory syndrome coro ···················· ···················· ···················· ···················· ···················· ····················
1873 MW411933.1 |Severe acute respiratory syndrome coro ···················· ···················· ···················· ···················· ···················· ····················
1874 MW411934.1 |Severe acute respiratory syndrome coro ···················· ···················· ···················· ···················· ···················· ····················
1875 MW411937.1 |Severe acute respiratory syndrome coro ···················· ·············A······ ···················· ···················· ···················· ····················
1876 MW411938.1 |Severe acute respiratory syndrome coro ···················· ···················· ···················· ···················· ···················· ····················
1877 MW411939.1 |Severe acute respiratory syndrome coro ···················· ···················· ···················· ···················· ···················· ····················
1878 MW411940.1 |Severe acute respiratory syndrome coro ···················· ···················· ···················· ···················· ···················· ····················
1879 MW411941.1 |Severe acute respiratory syndrome coro ···················· ···················· ···················· ···················· ···················· ····················
1880 MW411943.1 |Severe acute respiratory syndrome coro ···················· ···················· ···················· ···················· ···················· ····················
1881 MW411944.1 |Severe acute respiratory syndrome coro ···················· ···················· ···················· ···················· ···················· ····················
1882 MW411945.1 |Severe acute respiratory syndrome coro ···················· ···················· ···················· ···················· ···················· ····················
1883 MW411947.1 |Severe acute respiratory syndrome coro ···················· ···················· ···················· ···················· ···················· ····················
1884 MW411948.1 |Severe acute respiratory syndrome coro ···················· ···················· ···················· ···················· ···················· ····················
1885 MW411949.1 |Severe acute respiratory syndrome coro ···················· ···················· ···················· ···················· ···················· ····················
1886 MT232662.1 |Severe acute respiratory syndrome coro ···················· ···················· ···················· ···················· ···················· ····················
1887 MT232664.1 |Severe acute respiratory syndrome coro ···················· ···················· ···················· ···················· ···················· ····················
1888 MT232667.1 |Severe acute respiratory syndrome coro ···················· ···················· ···················· ···················· ···················· ····················
1889 MT232671.1 |Severe acute respiratory syndrome coro ···················· ···················· ···················· ···················· ···················· ····················
1890 MT232672.1 |Severe acute respiratory syndrome coro ···················· ···················· ···················· ···················· ···················· ····················
1891 MT232673.1 |Severe acute respiratory syndrome coro ···················· ···················· ···················· ···················· ···················· ····················
1892 MT232675.1 |Severe acute respiratory syndrome coro ···················· ···················· ···················· ···················· ···················· ····················
1893 MT232677.1 |Severe acute respiratory syndrome coro ···················· ···················· ···················· ···················· ···················· ····················
1894 MT232679.1 |Severe acute respiratory syndrome coro ···················· ···················· ···················· ···················· ···················· ····················
1895 MT232681.1 |Severe acute respiratory syndrome coro ···················· ···················· ···················· ···················· ···················· ····················
1896 MT232682.1 |Severe acute respiratory syndrome coro ···················· ···················· ···················· ···················· ···················· ····················
1897 MT232683.1 |Severe acute respiratory syndrome coro ···················· ···················· ···················· ···················· ···················· ····················
1898 MT232684.1 |Severe acute respiratory syndrome coro ···················· ···················· ···················· ···················· ···················· ····················
1899 MT232686.1 |Severe acute respiratory syndrome coro ···················· ···················· ···················· ···················· ···················· ····················
1900 MT232687.1 |Severe acute respiratory syndrome coro ···················· ···················· ···················· ···················· ···················· ····················
1901 MT232688.1 |Severe acute respiratory syndrome coro ···················· ···················· ···················· ···················· ···················· ····················
1902 MT232690.1 |Severe acute respiratory syndrome coro ···················· ···················· ···················· ···················· ···················· ····················
1903 MT232692.1 |Severe acute respiratory syndrome coro ···················· ···················· ···················· ···················· ···················· ··················C· 1
1904 MT232693.1 |Severe acute respiratory syndrome coro ···················· ···················· ···················· ···················· ···················· ····················
1905 MT232694.1 |Severe acute respiratory syndrome coro ···················· ···················· ···················· ···················· ···················· ····················
1906 MT232696.1 |Severe acute respiratory syndrome coro ···················· ···················· ···················· ···················· ···················· ····················
1907 MT232697.1 |Severe acute respiratory syndrome coro ···················· ···················· ···················· ···················· ···················· ····················
1908 MT232702.1 |Severe acute respiratory syndrome coro ···················· ···················· ···················· ···················· ···················· ····················
1909 MT232704.1 |Severe acute respiratory syndrome coro ···················· ···················· ···················· ···················· ···················· ····················
1910 MT232707.1 |Severe acute respiratory syndrome coro ···················· ···················· ···················· ···················· ···················· ····················
1911 MT232709.1 |Severe acute respiratory syndrome coro ···················· ···················· ···················· ···················· ···················· ····················
1912 MW406646.1 |Severe acute respiratory syndrome coro ···················· ···················· ···················· ···················· ·········T·········· ····················
1913 MW406647.1 |Severe acute respiratory syndrome coro ···················· ···················· ···················· ···················· ·········T·········· ····················
1914 MW406648.1 |Severe acute respiratory syndrome coro ···················· ···················· ···················· ···················· ·········T·········· ····················
1915 MW406649.1 |Severe acute respiratory syndrome coro ···················· ···················· ···················· ···················· ·········T·········· ····················
1916 MW406650.1 |Severe acute respiratory syndrome coro ···················· ···················· ···················· ···················· ·········T·········· ····················
1917 MW406651.1 |Severe acute respiratory syndrome coro ···················· ···················· ···················· ···················· ·········T·········· ····················
1918 MW406652.1 |Severe acute respiratory syndrome coro ···················· ···················· ···················· ···················· ·········T·········· ····················
1919 MW406653.1 |Severe acute respiratory syndrome coro ···················· ···················· ···················· ···················· ·········T·········· ····················
1920 MW406654.1 |Severe acute respiratory syndrome coro ···················· ···················· ···················· ···················· ·········T·········· ····················
1921 MW406655.1 |Severe acute respiratory syndrome coro ···················· ···················· ···················· ···················· ·········T·········· ····················
1922 MW406656.1 |Severe acute respiratory syndrome coro ···················· ···················· ···················· ···················· ···················· ····················
1923 MW406657.1 |Severe acute respiratory syndrome coro ···················· ···················· ···················· ···················· ···················· ····················
1924 MW406658.1 |Severe acute respiratory syndrome coro ···················· ···················· ···················· ···················· ···················· ····················
1925 MW406659.1 |Severe acute respiratory syndrome coro ···················· ···················· ···················· ···················· ···················· ····················
1926 MW406660.1 |Severe acute respiratory syndrome coro ···················· ···················· ···················· ···················· ···················· ····················
1927 MW406661.1 |Severe acute respiratory syndrome coro ···················· ···················· ···················· ···················· ···················· ····················
1928 MW406662.1 |Severe acute respiratory syndrome coro ···················· ······A············· ···················· ···················· ···················· ····················
1929 MW406663.1 |Severe acute respiratory syndrome coro ···················· ······A············· ···················· ···················· ···················· ····················
1930 MW406664.1 |Severe acute respiratory syndrome coro ···················· ······A············· ···················· ···················· ···················· ····················
1931 MW406665.1 |Severe acute respiratory syndrome coro ···················· ······A············· ···················· ···················· ···················· ····················
1932 MW406666.1 |Severe acute respiratory syndrome coro ···················· ······A············· ···················· ···················· ···················· ····················
1933 MW406667.1 |Severe acute respiratory syndrome coro ···················· ···················· ···················· ···················· ···················· ····················
1934 MW406668.1 |Severe acute respiratory syndrome coro ···················· ······A············· ···················· ···················· ···················· ····················
1935 MW406669.1 |Severe acute respiratory syndrome coro ···················· ···················· ···················· ···················· ···················· ····················
1936 MW406670.1 |Severe acute respiratory syndrome coro ···················· ···················· ···················· ···················· ···················· ····················
1937 MW406671.1 |Severe acute respiratory syndrome coro ···················· ···················· ···················· ···················· ···················· ····················
1938 MW406672.1 |Severe acute respiratory syndrome coro ···················· ···················· ···················· ···················· ···················· ····················
1939 MW406673.1 |Severe acute respiratory syndrome coro ···················· ···················· ···················· ···················· ···················· ····················
1940 MW406674.1 |Severe acute respiratory syndrome coro ···················· ···················· ···················· ···················· ···················· ····················
1941 MW406675.1 |Severe acute respiratory syndrome coro ···················· ···················· ···················· ···················· ···················· ····················
1942 MW406676.1 |Severe acute respiratory syndrome coro ···················· ···················· ···················· ···················· ···················· ····················
1943 MW406677.1 |Severe acute respiratory syndrome coro ···················· ···················· ···················· ···················· ···················· ····················
1944 MW406678.1 |Severe acute respiratory syndrome coro ···················· ···················· ···················· ···················· ···················· ····················

```
1945 MW406679.1 |Severe acute respiratory syndrome coro   ····················  ····················  ····················  ····················  ····················  ····················
1946 MW406680.1 |Severe acute respiratory syndrome coro   ····················  ····················  ····················  ····················  ····················  ····················
1947 MW406681.1 |Severe acute respiratory syndrome coro   ····················  ····················  ····················  ····················  ····················  ····················
1948 MW406682.1 |Severe acute respiratory syndrome coro   ····················  ····················  ····················  ····················  ····················  ····················
1949 MW406731.1 |Severe acute respiratory syndrome coro   ····················  ····T···············  ····················  ····················  ····················  ····················
1950 MW406732.1 |Severe acute respiratory syndrome coro   ····················  ····T···············  ····················  ····················  ····················  ····················
1951 MW406733.1 |Severe acute respiratory syndrome coro   ····················  ····T···············  ····················  ····················  ····················  ····················
1952 MW406735.1 |Severe acute respiratory syndrome coro   ····················  ····················  ····················  ····················  ····················  ····················
1953 MW406736.1 |Severe acute respiratory syndrome coro   ····················  ····T···············  ····················  ····················  ····················  ····················
1954 MW406737.1 |Severe acute respiratory syndrome coro   ····················  ·A··················  ····················  ····················  ····················  ····················
1955 MW406738.1 |Severe acute respiratory syndrome coro   ····················  ····················  ····················  ····················  ····················  ····················
1956 MW406739.1 |Severe acute respiratory syndrome coro   ····················  ····T···············  ····················  ····················  ····················  ····················
1957 MW406740.1 |Severe acute respiratory syndrome coro   ····················  ····················  ····················  ····················  ····················  ····················
1958 MW406781.1 |Severe acute respiratory syndrome coro   ····················  ····················  ····················  ····················  ····················  ····················
1959 MW406794.1 |Severe acute respiratory syndrome coro   ····················  ····················  ····················  ····················  ····················  ····················
1960 MW404672.1 |Severe acute respiratory syndrome coro   ····················  ····················  ····················  ····················  ····················  ····················
1961 MW404673.1 |Severe acute respiratory syndrome coro   ····················  ····················  ····················  ····················  ····················  ····················
1962 MW405462.1 |Severe acute respiratory syndrome coro   ····················  ····················  ····················  ····················  ····················  ····················
1963 MW405463.1 |Severe acute respiratory syndrome coro   ····················  ····················  ····················  ····················  ····················  ····················
1964 MW405464.1 |Severe acute respiratory syndrome coro   ····················  ····················  ····················  ····················  ····················  ····················
1965 MW405465.1 |Severe acute respiratory syndrome coro   ····················  ····················  ····················  ····················  ····················  ····················
1966 MW405466.1 |Severe acute respiratory syndrome coro   ····················  ····················  ····················  ····················  ····················  ····················
1967 MW405467.1 |Severe acute respiratory syndrome coro   ····················  ····················  ····················  ····················  ····················  ····················
1968 MW405468.1 |Severe acute respiratory syndrome coro   ····················  ····················  ····················  ····················  ····················  ····················
1969 MW405469.1 |Severe acute respiratory syndrome coro   ····················  ·A··················  ····················  ····················  ····················  ····················
1970 MW405470.1 |Severe acute respiratory syndrome coro   ····················  ····················  ····················  ····················  ····················  ····················
1971 MW405471.1 |Severe acute respiratory syndrome coro   ····················  ····················  ····················  ····················  ····················  ····················
1972 MW405472.1 |Severe acute respiratory syndrome coro   ····················  ····················  ····················  ····················  ····················  ····················
1973 MW405473.1 |Severe acute respiratory syndrome coro   ········T···········  ·A··················  ····················  ····················  ····················  ····················
1974 MW405474.1 |Severe acute respiratory syndrome coro   ····················  ····················  ····················  ····················  ····················  ····················
1975 MW405475.1 |Severe acute respiratory syndrome coro   ····················  ····················  ····················  ····················  ····················  ····················
1976 MW405476.1 |Severe acute respiratory syndrome coro   ····················  ····················  ····················  ····················  ····················  ····················
1977 MW405477.1 |Severe acute respiratory syndrome coro   ····················  ·A··················  ····················  ····················  ····················  ····················
1978 MW405478.1 |Severe acute respiratory syndrome coro   ····················  ······A·············  ····················  ····················  ····················  ····················
1979 MW405479.1 |Severe acute respiratory syndrome coro   ····················  ····················  ····················  ····················  ····················  ····················
1980 MW405480.1 |Severe acute respiratory syndrome coro   ····················  ····················  ····················  ····················  ····················  ····················
1981 MW405481.1 |Severe acute respiratory syndrome coro   ····················  ····················  ····················  ····················  ····················  ····················
1982 MW405482.1 |Severe acute respiratory syndrome coro   ····················  ····A···············  ····················  ····················  ····················  ····················
1983 MW405483.1 |Severe acute respiratory syndrome coro   ····················  ····A···············  ····················  ····················  ····················  ····················
1984 MW405484.1 |Severe acute respiratory syndrome coro   ····················  ····················  ····················  ····················  ····················  ····················
1985 MW405485.1 |Severe acute respiratory syndrome coro   ····················  ····················  ····················  ····················  ····················  ····················
1986 MW405486.1 |Severe acute respiratory syndrome coro   ····················  ····················  ····················  ····················  ····················  ····················
1987 MW403520.1 |Severe acute respiratory syndrome coro   ····················  ····················  ····················  ····················  ····················  ····················
1988 MW403523.1 |Severe acute respiratory syndrome coro   ····················  ····················  ····················  ····················  ····················  ····················
1989 MW403524.1 |Severe acute respiratory syndrome coro   ····················  ····················  ····················  ····················  ····················  ····················
1990 MW403525.1 |Severe acute respiratory syndrome coro   ····················  ····················  ····················  ····················  ····················  ····················
1991 MW403526.1 |Severe acute respiratory syndrome coro   ····················  ····················  ····················  ····················  ····················  ····················
1992 MW403527.1 |Severe acute respiratory syndrome coro   ····················  ····················  ····················  ····················  ····················  ····················
1993 MW403529.1 |Severe acute respiratory syndrome coro   ····················  ····················  ····················  ····················  ····················  ····················
1994 MW403530.1 |Severe acute respiratory syndrome coro   ····················  ····················  ····················  ····················  ····················  ····················
1995 MW403532.1 |Severe acute respiratory syndrome coro   ····················  ····················  ····················  ····················  ····················  ····················
1996 MW403533.1 |Severe acute respiratory syndrome coro   ····················  ····················  ····················  ····················  ····················  ····················
1997 MW403534.1 |Severe acute respiratory syndrome coro   ····················  ····················  ····················  ····················  ····················  ····················
1998 MW403535.1 |Severe acute respiratory syndrome coro   ····················  ····················  ····················  ····················  ····················  ····················
1999 MW403538.1 |Severe acute respiratory syndrome coro   ····················  ····················  ····················  ····················  ····················  ····················
2000 MW403540.1 |Severe acute respiratory syndrome coro   ····················  ····················  ····················  ····················  ····················  ····················
2001 MW403541.1 |Severe acute respiratory syndrome coro   ····················  ····················  ····················  ····················  ····················  ····················
2002 MW403542.1 |Severe acute respiratory syndrome coro   ····················  ····················  ····················  ····················  ····················  ····················
2003 MW403544.1 |Severe acute respiratory syndrome coro   ····················  ····················  ····················  ····················  ····················  ····················
2004 MW403545.1 |Severe acute respiratory syndrome coro   ····················  ····················  ····················  ····················  ····················  ····················
2005 MW403546.1 |Severe acute respiratory syndrome coro   ····················  ····················  ····················  ····················  ····················  ····················
2006 MW403548.1 |Severe acute respiratory syndrome coro   ····················  ····················  ····················  ····················  ····················  ····················
2007 MW403549.1 |Severe acute respiratory syndrome coro   ····················  ····················  ····················  ····················  ····················  ····················
2008 MW403550.1 |Severe acute respiratory syndrome coro   ····················  ····················  ····················  ····················  ····················  ····················
2009 MW403551.1 |Severe acute respiratory syndrome coro   ····················  ····················  ····················  ····················  ····················  ····················
2010 MW403552.1 |Severe acute respiratory syndrome coro   ····················  ····················  ····················  ····················  ····················  ····················
2011 MW403553.1 |Severe acute respiratory syndrome coro   ····················  ····················  ····················  ····················  ····················  ····················
2012 MW403557.1 |Severe acute respiratory syndrome coro   ····················  ····················  ····················  ····················  ····················  ····················
2013 MW403559.1 |Severe acute respiratory syndrome coro   ····················  ····················  ····················  ····················  ····················  ····················
2014 MW403562.1 |Severe acute respiratory syndrome coro   ····················  ····················  ····················  ····················  ····················  ····················
2015 MW403564.1 |Severe acute respiratory syndrome coro   ····················  ····················  ····················  ····················  ····················  ····················
2016 MW403573.1 |Severe acute respiratory syndrome coro   ····················  ····················  ····················  ····················  ····················  ····················
2017 MW403589.1 |Severe acute respiratory syndrome coro   ····················  ····················  ····················  ····················  ····················  ····················
2018 MW403598.1 |Severe acute respiratory syndrome coro   ····················  ····················  ····················  ····················  ····················  ····················
2019 MW403600.1 |Severe acute respiratory syndrome coro   ····················  ·A··················  ····················  ····················  ····················  ····················
2020 MW403604.1 |Severe acute respiratory syndrome coro   ····················  ····················  ····················  ····················  ····················  ····················
2021 MW403605.1 |Severe acute respiratory syndrome coro   ····················  ····················  ····················  ····················  ····················  ····················
2022 MW403606.1 |Severe acute respiratory syndrome coro   ····················  ····················  ····················  ····················  ····················  ····················
```

```
2023 MW403607.1 |Severe acute respiratory syndrome coro   ····················  ····················  ····················  ····················  ····················  ····················
2024 MW403608.1 |Severe acute respiratory syndrome coro   ····················  ····················  ····················  ····················  ····················  ····················
2025 MW403609.1 |Severe acute respiratory syndrome coro   ····················  ····················  ····················  ····················  ····················  ····················
2026 MW403610.1 |Severe acute respiratory syndrome coro   ····················  ····················  ····················  ····················  ····················  ····················
2027 MW403611.1 |Severe acute respiratory syndrome coro   ····················  ·A··················  ····················  ····················  ····················  ····················
2028 MW403612.1 |Severe acute respiratory syndrome coro   ····················  ····················  ····················  ····················  ····················  ····················
2029 MW403613.1 |Severe acute respiratory syndrome coro   ····················  ····················  ····················  ····················  ····················  ····················
2030 MW403615.1 |Severe acute respiratory syndrome coro   ····················  ····················  ····················  ····················  ····················  ····················
2031 MW403616.1 |Severe acute respiratory syndrome coro   ····················  ····················  ····················  ····················  ····················  ····················
2032 MW403617.1 |Severe acute respiratory syndrome coro   ····················  ····················  ····················  ····················  ····················  ····················
2033 MW403618.1 |Severe acute respiratory syndrome coro   ····················  ·A··················  ····················  ····················  ····················  ····················
2034 MW403625.1 |Severe acute respiratory syndrome coro   ····················  ····················  ····················  ····················  ····················  ····················
2035 MW403627.1 |Severe acute respiratory syndrome coro   ····················  ····················  ····················  ····················  ····················  ····················
2036 MW403628.1 |Severe acute respiratory syndrome coro   ····················  ····················  ····················  ····················  ····················  ····················
2037 MW403629.1 |Severe acute respiratory syndrome coro   ····················  ····················  ····················  ····················  ····················  ····················
2038 MW403631.1 |Severe acute respiratory syndrome coro   ····················  ····················  ····················  ····················  ····················  ····················
2039 MW403632.1 |Severe acute respiratory syndrome coro   ····················  ····················  ····················  ····················  ····················  ····················
2040 MW403636.1 |Severe acute respiratory syndrome coro   ····················  ····················  ····················  ····················  ····················  ····················
2041 MW403641.1 |Severe acute respiratory syndrome coro   ····················  ·A··················  ····················  ····················  ····················  ····················
2042 MW403642.1 |Severe acute respiratory syndrome coro   ····················  ····················  ····················  ····················  ····················  ····················
2043 MW403644.1 |Severe acute respiratory syndrome coro   ····················  ····················  ····················  ····················  ····················  ····················
2044 MW403646.1 |Severe acute respiratory syndrome coro   ····················  ····················  ····················  ····················  ····················  ····················
2045 MW403648.1 |Severe acute respiratory syndrome coro   ····················  ····················  ····················  ····················  ····················  ····················
2046 MW403649.1 |Severe acute respiratory syndrome coro   ····················  ····················  ····················  ····················  ····················  ····················
2047 MW403651.1 |Severe acute respiratory syndrome coro   ·····T··············  ····················  ····················  ····················  ····················  ····················
2048 MW403652.1 |Severe acute respiratory syndrome coro   ····················  ····················  ····················  ····················  ····················  ····················
2049 MW403653.1 |Severe acute respiratory syndrome coro   ····················  ····················  ····················  ····················  ····················  ····················
2050 MW403655.1 |Severe acute respiratory syndrome coro   ····················  ····················  ····················  ····················  ····················  ····················
2051 MW403656.1 |Severe acute respiratory syndrome coro   ····················  ····················  ····················  ····················  ····················  ····················
2052 MW403661.1 |Severe acute respiratory syndrome coro   ····················  ····················  ····················  ····················  ····················  ····················
2053 MW403662.1 |Severe acute respiratory syndrome coro   ····················  ····················  ····················  ····················  ····················  ····················
2054 MW403663.1 |Severe acute respiratory syndrome coro   ····················  ····G···············  ····················  ····················  ····················  ····················
2055 MW403664.1 |Severe acute respiratory syndrome coro   ····················  ····················  ····················  ····················  ····················  ····················
2056 MW403665.1 |Severe acute respiratory syndrome coro   ····················  ····················  ····················  ····················  ····················  ····················
2057 MW403668.1 |Severe acute respiratory syndrome coro   ····················  ····················  ····················  ····················  ····················  ····················
2058 MW403670.1 |Severe acute respiratory syndrome coro   ····················  ····················  ····················  ····················  ····················  ····················
2059 MW403671.1 |Severe acute respiratory syndrome coro   ····················  ····················  ····················  ····················  ····················  ····················
2060 MW403672.1 |Severe acute respiratory syndrome coro   ····················  ····················  ····················  ····················  ····················  ····················
2061 MW403673.1 |Severe acute respiratory syndrome coro   ····················  ····················  ····················  ····················  ·········T··········  ····················
2062 MW403674.1 |Severe acute respiratory syndrome coro   ····················  ····················  ····················  ····················  ·········T··········  ····················
2063 MW403675.1 |Severe acute respiratory syndrome coro   ····················  ····················  ····················  ····················  ·········T··········  ····················
2064 MW403676.1 |Severe acute respiratory syndrome coro   ····················  ····················  ····················  ····················  ·········T··········  ····················
2065 MW403677.1 |Severe acute respiratory syndrome coro   ····················  ····················  ····················  ····················  ·········T··········  ····················
2066 MW403678.1 |Severe acute respiratory syndrome coro   ····················  ····················  ····················  ····················  ·········T··········  ····················
2067 MW403679.1 |Severe acute respiratory syndrome coro   ····················  ····················  ····················  ····················  ·········T··········  ····················
2068 MW403681.1 |Severe acute respiratory syndrome coro   ····················  ····················  ····················  ····················  ·········T··········  ····················
2069 MW403682.1 |Severe acute respiratory syndrome coro   ····················  ····················  ····················  ····················  ·········T··········  ····················
2070 MW403683.1 |Severe acute respiratory syndrome coro   ····················  ····················  ····················  ····················  ·········T··········  ····················
2071 MW403684.1 |Severe acute respiratory syndrome coro   ····················  ····················  ····················  ····················  ·········T··········  ····················
2072 MW403685.1 |Severe acute respiratory syndrome coro   ····················  ····················  ····················  ····················  ·········T··········  ····················
2073 MW403686.1 |Severe acute respiratory syndrome coro   ····················  ····················  ····················  ····················  ·········T··········  ····················
2074 MW403687.1 |Severe acute respiratory syndrome coro   ····················  ····················  ····················  ····················  ·········T··········  ····················
2075 MW403688.1 |Severe acute respiratory syndrome coro   ····················  ····················  ····················  ····················  ·········T··········  ····················
2076 MW403689.1 |Severe acute respiratory syndrome coro   ····················  ····················  ····················  ····················  ·········T··········  ····················
2077 MW403690.1 |Severe acute respiratory syndrome coro   ····················  ····················  ····················  ····················  ·········T··········  ····················
2078 MW403691.1 |Severe acute respiratory syndrome coro   ····················  ····················  ····················  ····················  ·········T··········  ····················
2079 MW403692.1 |Severe acute respiratory syndrome coro   ····················  ····················  ····················  ····················  ····················  ····················
2080 MW403693.1 |Severe acute respiratory syndrome coro   ····················  ····················  ····················  ····················  ····················  ····················
2081 MW403694.1 |Severe acute respiratory syndrome coro   ····················  ····················  ····················  ····················  ····················  ····················
2082 MW403695.1 |Severe acute respiratory syndrome coro   ····················  ····················  ····················  ····················  ····················  ····················
2083 MW403696.1 |Severe acute respiratory syndrome coro   ····················  ····················  ····················  ····················  ····················  ····················
2084 MW403697.1 |Severe acute respiratory syndrome coro   ····················  ····················  ····················  ····················  ····················  ····················
2085 MW403698.1 |Severe acute respiratory syndrome coro   ····················  ····················  ····················  ····················  ····················  ····················
2086 MW403699.1 |Severe acute respiratory syndrome coro   ····················  ····················  ····················  ····················  ····················  ····················
2087 MW403700.1 |Severe acute respiratory syndrome coro   ····················  ····················  ····················  ····················  ····················  ····················
2089 MW403702.1 |Severe acute respiratory syndrome coro   ····················  ····················  ····················  ····················  ····················  ····················
2090 MW403703.1 |Severe acute respiratory syndrome coro   ····················  ····················  ····················  ····················  ····················  ····················
2091 MW403763.1 |Severe acute respiratory syndrome coro   ····················  ····················  ····················  ····················  ····················  ····················
2092 MW403764.1 |Severe acute respiratory syndrome coro   ····················  ····················  ····················  ····················  ····················  ····················
2093 MW403765.1 |Severe acute respiratory syndrome coro   ····················  ····················  ····················  ····················  ····················  ····················
2094 MW403766.1 |Severe acute respiratory syndrome coro   ····················  ····················  ····················  ····················  ····················  ····················
2095 MW403767.1 |Severe acute respiratory syndrome coro   ····················  ····················  ····················  ····················  ····················  ····················
2096 MW403768.1 |Severe acute respiratory syndrome coro   ····················  ····················  ····················  ····················  ····················  ····················
2097 MW403769.1 |Severe acute respiratory syndrome coro   ····················  ····················  ····················  ····················  ····················  ····················
2098 MW064259.1 |Severe acute respiratory syndrome coro   ····················  ····················  ····················  ····················  ····················  ····················
2099 MW064260.1 |Severe acute respiratory syndrome coro   ····················  ····················  ····················  ····················  ····················  ····················
2100 MW064263.1 |Severe acute respiratory syndrome coro   ····················  ····················  ····················  ····················  ····················  ····················
2101 MW064264.1 |Severe acute respiratory syndrome coro   ····················  ····················  ····················  ····················  ····················  ····················
```

```
2102 MW389566.1 |Severe acute respiratory syndrome coro   ....................  ....................  ....................  ....................  ....................  ....................
2103 MW389567.1 |Severe acute respiratory syndrome coro   ....................  ....T...............  ....................  ....................  ....................  ....................
2104 MW389569.1 |Severe acute respiratory syndrome coro   ....................  ....T...............  ....................  ....................  ....................  ....................
2105 MW389570.1 |Severe acute respiratory syndrome coro   ....................  ....T...............  ....................  ....................  ....................  ....................
2106 MW389571.1 |Severe acute respiratory syndrome coro   ....................  ....................  ....................  ....................  ....................  ....................
2107 MW389572.1 |Severe acute respiratory syndrome coro   ....................  ....T...............  ....................  ....................  ....................  ....................
2108 MW389573.1 |Severe acute respiratory syndrome coro   ....................  ....................  ....................  ....................  ....................  ....................
2109 MW389574.1 |Severe acute respiratory syndrome coro   ....................  ....T...............  ....................  ....................  ....................  ....................
2110 MW389575.1 |Severe acute respiratory syndrome coro   ....................  ....T...............  ....................  ....................  ....................  ....................
2111 MW389576.1 |Severe acute respiratory syndrome coro   ....................  ....................  ....................  ....................  ....................  ....................
2112 MW389579.1 |Severe acute respiratory syndrome coro   ....................  ....................  ....................  ....................  ....................  ....................
2113 MW389583.1 |Severe acute respiratory syndrome coro   ....................  ....................  ....................  ....................  ....................  ....................
2114 MW389598.1 |Severe acute respiratory syndrome coro   ....................  ....................  ....................  ....................  ....................  ....................
2115 MW389599.1 |Severe acute respiratory syndrome coro   ....................  ....................  ....................  ....................  ....................  ....................
2116 MW389600.1 |Severe acute respiratory syndrome coro   ....................  ....................  ....................  ....................  ....................  ....................
2117 MW389602.1 |Severe acute respiratory syndrome coro   ....................  ....................  ....................  ....................  ....................  ....................
2118 MW389603.1 |Severe acute respiratory syndrome coro   ....................  ....................  ....................  ....................  ....................  ....................
2119 MW389604.1 |Severe acute respiratory syndrome coro   ....................  ....................  ....................  ....................  ....................  ....................
2120 MW389605.1 |Severe acute respiratory syndrome coro   ....................  ....................  ....................  ....................  ....................  ....................
2121 MW389606.1 |Severe acute respiratory syndrome coro   ....................  ....................  ....................  ....................  ....................  ....................
2122 MW389607.1 |Severe acute respiratory syndrome coro   ....................  ....................  ....................  ....................  ....................  ....................
2123 MW389608.1 |Severe acute respiratory syndrome coro   ....................  ....T...............  ....................  ....................  ....................  ....................
2124 MW389609.1 |Severe acute respiratory syndrome coro   ....................  ....................  ....................  ....................  ....................  ....................
2125 MW389611.1 |Severe acute respiratory syndrome coro   ....................  ....................  ....................  ....................  ....................  ....................
2126 MW389612.1 |Severe acute respiratory syndrome coro   ....................  ....................  ....................  ....................  ....................  ....................
2127 MW389613.1 |Severe acute respiratory syndrome coro   ....................  .A..................  ....................  ....................  ....................  ....................
2128 MW389614.1 |Severe acute respiratory syndrome coro   ....................  .A..................  ....................  ....................  ....................  ....................
2129 MW389615.1 |Severe acute respiratory syndrome coro   ....................  .A..................  ....................  ....................  ....................  ....................
2130 MW389616.1 |Severe acute respiratory syndrome coro   ....................  ....................  ....................  ....................  ....................  ....................
2131 MW389618.1 |Severe acute respiratory syndrome coro   ....................  ....................  ....................  ....................  ....................  ....................
2132 MW389619.1 |Severe acute respiratory syndrome coro   ....................  .A..................  ....................  ....................  ....................  ....................
2133 MW389621.1 |Severe acute respiratory syndrome coro   ....................  .A..................  ....................  ....................  ....................  ....................
2134 MW389622.1 |Severe acute respiratory syndrome coro   ....................  ....................  ....................  ....................  ....................  ....................
2135 MW389623.1 |Severe acute respiratory syndrome coro   ....................  ....................  ....................  ....................  ....................  ....................
2136 MW389624.1 |Severe acute respiratory syndrome coro   ....................  .A..................  ....................  ....................  ....................  ....................
2137 MW389625.1 |Severe acute respiratory syndrome coro   ....................  ....................  ....................  ....................  ....................  ....................
2138 MW389626.1 |Severe acute respiratory syndrome coro   ....................  ....T...............  ....................  ....................  ....................  ....................
2139 MW389627.1 |Severe acute respiratory syndrome coro   ....................  .A..................  ....................  ....................  ....................  ....................
2140 MW389628.1 |Severe acute respiratory syndrome coro   ....................  ....................  ....................  ....................  ....................  ....................
2141 MW389630.1 |Severe acute respiratory syndrome coro   ....................  ....T...............  ....................  ....................  ....................  ....................
2142 MW389631.1 |Severe acute respiratory syndrome coro   ....................  ....................  ....................  ....................  ....................  ....................
2143 MW389635.1 |Severe acute respiratory syndrome coro   ....................  .A..................  ....................  ....................  ....................  ....................
2144 MW389636.1 |Severe acute respiratory syndrome coro   ....................  ....................  ....................  ....................  ....................  ....................
2145 MW389638.1 |Severe acute respiratory syndrome coro   ....................  ....................  ....................  ....................  ....................  ....................
2146 MW389639.1 |Severe acute respiratory syndrome coro   ....................  ....................  ....................  ....................  ....................  ....................
2147 MW389640.1 |Severe acute respiratory syndrome coro   ....................  ....................  ....................  ....................  ....................  ....................
2148 MW389641.1 |Severe acute respiratory syndrome coro   ....................  .A..................  ....................  ....................  ....................  ....................
2149 MW389644.1 |Severe acute respiratory syndrome coro   ....................  ....................  ....................  ....................  ....................  ....................
2150 MW389646.1 |Severe acute respiratory syndrome coro   ....................  .A..................  ....................  ....................  ....................  ....................
2151 MW389649.1 |Severe acute respiratory syndrome coro   ....................  .A..................  ....................  ....................  ....................  ....................
2152 MW390832.1 |Severe acute respiratory syndrome coro   ....................  ....................  ....................  ....................  ....................  ....................
2153 MW390833.1 |Severe acute respiratory syndrome coro   ....................  ....................  ....................  ....................  ....................  ....................
2154 MW390834.1 |Severe acute respiratory syndrome coro   ....................  ....................  ....................  ....................  ....................  ....................
2155 MW390835.1 |Severe acute respiratory syndrome coro   ....................  ....................  ....................  ....................  ....................  ....................
2156 MW390836.1 |Severe acute respiratory syndrome coro   ....................  .A..................  ....................  ....................  ....................  ....................
2157 MW390837.1 |Severe acute respiratory syndrome coro   ....................  ....................  ....................  ....................  ....................  ....................
2158 MW390838.1 |Severe acute respiratory syndrome coro   ....................  ....................  ....................  ....................  ....................  ....................
2159 MW390839.1 |Severe acute respiratory syndrome coro   ....................  ....................  ....................  ....................  ....................  ....................
2160 MW390840.1 |Severe acute respiratory syndrome coro   ....................  ....................  ....................  ....................  ....................  ....................
2161 MW390841.1 |Severe acute respiratory syndrome coro   ....................  ....................  ....................  ....................  ....................  ....................
2162 MW390842.1 |Severe acute respiratory syndrome coro   ....................  ....................  ....................  ....................  ....................  ....................
2163 MW390843.1 |Severe acute respiratory syndrome coro   ....................  ....................  ....................  ....................  ....................  ....................
2164 MW390844.1 |Severe acute respiratory syndrome coro   ....................  ....................  ....................  ....................  ....................  ....................
2165 MW390845.1 |Severe acute respiratory syndrome coro   ....................  ....................  ....................  ....................  ....................  ....................
2166 MW390846.1 |Severe acute respiratory syndrome coro   ....................  ....................  ....................  ....................  ....................  ....................
2167 MW390847.1 |Severe acute respiratory syndrome coro   ....................  ....................  ....................  ....................  ....................  ....................
2168 MW390848.1 |Severe acute respiratory syndrome coro   ....................  ....................  ....................  ....................  ....................  ....................
2169 MW390849.1 |Severe acute respiratory syndrome coro   ....................  ....................  ....................  ....................  ....................  ....................
2170 MW390850.1 |Severe acute respiratory syndrome coro   ....................  ....................  ....................  ....................  ....................  ....................
2171 MW390851.1 |Severe acute respiratory syndrome coro   ....................  ....................  ....................  ....................  ....................  ....................
2172 MW390852.1 |Severe acute respiratory syndrome coro   ....................  ....................  ....................  ....................  ....................  ....................
2173 MW390853.1 |Severe acute respiratory syndrome coro   ....................  ....................  ....................  ....................  ....................  ....................
2174 MW390854.1 |Severe acute respiratory syndrome coro   ....................  ....................  ....................  ....................  ....................  ....................
2175 MW390855.1 |Severe acute respiratory syndrome coro   ....................  ....................  ....................  ....................  ....................  ....................
2176 MW390856.1 |Severe acute respiratory syndrome coro   ....................  ....................  ....................  ....................  ....................  ....................
2177 MW390857.1 |Severe acute respiratory syndrome coro   ....................  ....................  ....................  ....................  ....................  ....................
2178 MW390858.1 |Severe acute respiratory syndrome coro   ....................  ....................  ....................  ....................  ....................  ....................
2179 MW390859.1 |Severe acute respiratory syndrome coro   ....................  ....................  ....................  ....................  ....................  ....................
```

2180	MW390860.1	|Severe acute respiratory syndrome coro	····················	····················	····················	····················	····················	····················
2181	MW390861.1	|Severe acute respiratory syndrome coro	····················	····················	····················	····················	····················	····················
2182	MW390862.1	|Severe acute respiratory syndrome coro	····················	····················	····················	····················	····················	····················
2183	MW390863.1	|Severe acute respiratory syndrome coro	····················	····················	····················	····················	····················	····················
2184	MW390864.1	|Severe acute respiratory syndrome coro	····················	····················	····················	····················	····················	····················
2185	MW390865.1	|Severe acute respiratory syndrome coro	····················	····················	····················	····················	····················	····················
2187	MW390867.1	|Severe acute respiratory syndrome coro	····················	····················	····················	····················	····················	····················
2188	MW390868.1	|Severe acute respiratory syndrome coro	····················	····················	····················	····················	····················	····················
2189	MW384414.1	|Severe acute respiratory syndrome coro	····················	····················	····················	····················	····················	····················
2190	MW384415.1	|Severe acute respiratory syndrome coro	····················	····················	····················	····················	····················	····················
2191	MW384417.1	|Severe acute respiratory syndrome coro	····················	····················	····················	····················	····················	····················
2192	MW384418.1	|Severe acute respiratory syndrome coro	····················	····················	····················	····················	····················	····················
2193	MW384419.1	|Severe acute respiratory syndrome coro	····················	····················	····················	····················	····················	····················
2194	MW384420.1	|Severe acute respiratory syndrome coro	····················	····················	····················	····················	····················	····················
2195	MW384421.1	|Severe acute respiratory syndrome coro	····················	····················	····················	····················	····················	····················
2196	MW384422.1	|Severe acute respiratory syndrome coro	····················	····················	····················	····················	····················	····················
2197	MW384423.1	|Severe acute respiratory syndrome coro	····················	····················	····················	····················	····················	····················
2198	MW384424.1	|Severe acute respiratory syndrome coro	····················	····················	····················	····················	····················	····················
2199	MW384425.1	|Severe acute respiratory syndrome coro	····················	····················	····················	····················	····················	····················
2200	MW384426.1	|Severe acute respiratory syndrome coro	····················	····················	····················	····················	····················	····················
2201	MW384427.1	|Severe acute respiratory syndrome coro	····················	·A··················	····················	····················	····················	····················
2202	MW386175.1	|Severe acute respiratory syndrome coro	····················	····················	····················	····················	····················	····················
2203	MW386176.1	|Severe acute respiratory syndrome coro	····················	····················	····················	····················	····················	····················
2204	MW386177.1	|Severe acute respiratory syndrome coro	····················	····················	····················	····················	····················	····················
2205	MW375726.1	|Severe acute respiratory syndrome coro	····················	····················	····················	····················	····················	····················
2206	MW375727.1	|Severe acute respiratory syndrome coro	····················	····················	····················	····················	····················	····················
2207	MW375728.1	|Severe acute respiratory syndrome coro	····················	····················	····················	····················	····················	····················
2208	MW375729.1	|Severe acute respiratory syndrome coro	····················	····················	····················	····················	····················	····················
2209	MW375730.1	|Severe acute respiratory syndrome coro	····················	····················	····················	····················	····················	····················
2210	MW375731.1	|Severe acute respiratory syndrome coro	····················	····················	····················	····················	····················	····················
2211	MW375993.1	|Severe acute respiratory syndrome coro	····················	····················	····················	····················	····················	····················
2212	MW375994.1	|Severe acute respiratory syndrome coro	····················	····················	····················	····················	····················	····················
2213	MW368700.1	|Severe acute respiratory syndrome coro	····················	·A··················	····················	····················	····················	···A················
2214	MW374904.1	|Severe acute respiratory syndrome coro	····················	····················	····················	····················	····················	····················
2215	MW374905.1	|Severe acute respiratory syndrome coro	····················	····················	····················	····················	····················	····················
2216	MW374906.1	|Severe acute respiratory syndrome coro	····················	····················	····················	····················	····················	····················
2217	MW374907.1	|Severe acute respiratory syndrome coro	····················	·A··················	····················	····················	····················	····················
2218	MW374908.1	|Severe acute respiratory syndrome coro	····················	·A··················	····················	····················	····················	····················
2219	MW374909.1	|Severe acute respiratory syndrome coro	····················	····················	····················	····················	····················	····················
2220	MW374910.1	|Severe acute respiratory syndrome coro	····················	····················	····················	····················	····················	····················
2221	MW374911.1	|Severe acute respiratory syndrome coro	····················	····················	····················	····················	····················	····················
2222	MW374912.1	|Severe acute respiratory syndrome coro	····················	····················	····················	····················	····················	····················
2223	MW374913.1	|Severe acute respiratory syndrome coro	····················	····················	····················	····················	····················	····················
2224	MW374914.1	|Severe acute respiratory syndrome coro	····················	····················	····················	····················	····················	····················
2225	MW374915.1	|Severe acute respiratory syndrome coro	····················	····················	····················	····················	····················	····················
2226	MW374916.1	|Severe acute respiratory syndrome coro	····················	····················	····················	····················	····················	····················
2227	MW374917.1	|Severe acute respiratory syndrome coro	····················	··C·················	····················	····················	····················	····················
2228	MW374918.1	|Severe acute respiratory syndrome coro	····················	····················	····················	····················	····················	····················
2229	MW374919.1	|Severe acute respiratory syndrome coro	····················	····················	····················	····················	····················	····················
2230	MW374920.1	|Severe acute respiratory syndrome coro	····················	····················	····················	····················	····················	····················
2231	MW374921.1	|Severe acute respiratory syndrome coro	····················	·A··················	····················	····················	····················	····················
2232	MW374922.1	|Severe acute respiratory syndrome coro	····················	····················	····················	····················	····················	····················
2233	MW374923.1	|Severe acute respiratory syndrome coro	····················	····················	····················	····················	····················	····················
2234	MW374924.1	|Severe acute respiratory syndrome coro	····················	·A··················	····················	····················	····················	····················
2235	MW374925.1	|Severe acute respiratory syndrome coro	····················	····················	····················	····················	····················	····················
2236	MW374926.1	|Severe acute respiratory syndrome coro	····················	·A··················	····················	····················	····················	····················
2237	MW374927.1	|Severe acute respiratory syndrome coro	····················	····················	····················	····················	····················	····················
2238	MW374928.1	|Severe acute respiratory syndrome coro	····················	·A··················	····················	····················	····················	····················
2239	MW374929.1	|Severe acute respiratory syndrome coro	····················	····················	····················	····················	····················	····················
2240	MW374930.1	|Severe acute respiratory syndrome coro	····················	····················	····················	····················	····················	····················
2241	MW374931.1	|Severe acute respiratory syndrome coro	····················	····················	····················	····················	····················	····················
2242	MW374932.1	|Severe acute respiratory syndrome coro	····················	····················	····················	····················	····················	····················
2243	MW374933.1	|Severe acute respiratory syndrome coro	····················	····················	····················	····················	····················	····················
2244	MW374934.1	|Severe acute respiratory syndrome coro	····················	····················	····················	····················	····················	····················
2245	MW374935.1	|Severe acute respiratory syndrome coro	····················	····················	····················	····················	····················	····················
2246	MW374936.1	|Severe acute respiratory syndrome coro	····················	····················	····················	····················	····················	····················
2247	MW374937.1	|Severe acute respiratory syndrome coro	····················	····················	····················	····················	····················	····················
2248	MW374938.1	|Severe acute respiratory syndrome coro	····················	····················	····················	····················	····················	····················
2249	MW374939.1	|Severe acute respiratory syndrome coro	····················	····················	····················	····················	····················	····················
2250	MW374940.1	|Severe acute respiratory syndrome coro	····················	····················	····················	····················	····················	····················
2251	MW374941.1	|Severe acute respiratory syndrome coro	····················	····················	····················	····················	····················	····················
2252	MW374942.1	|Severe acute respiratory syndrome coro	····················	····················	····················	····················	····················	····················
2253	MW374943.1	|Severe acute respiratory syndrome coro	····················	····················	····················	····················	····················	····················
2254	MW374944.1	|Severe acute respiratory syndrome coro	····················	····················	····················	····················	····················	····················
2255	MW374945.1	|Severe acute respiratory syndrome coro	····················	····················	····················	····················	····················	····················
2256	MW374946.1	|Severe acute respiratory syndrome coro	········T···········	·A··················	····················	····················	····················	····················
2257	MW374947.1	|Severe acute respiratory syndrome coro	····················	····················	····················	····················	····················	····················
2258	MW374948.1	|Severe acute respiratory syndrome coro	····················	····················	····················	····················	····················	····················

2259	MW365356.1	|Severe acute respiratory syndrome coro	....	....	....	....	....	....
2260	MW365357.1	|Severe acute respiratory syndrome coro	....	....	....	....	....	....
2261	MW365358.1	|Severe acute respiratory syndrome coro	....	....	....	....	....	....
2262	MW365359.1	|Severe acute respiratory syndrome coro	....	....	....	....	....	....
2263	MW366724.1	|Severe acute respiratory syndrome coro	....	....	....	....	....	....
2264	MW366750.1	|Severe acute respiratory syndrome coro	....	....	....	....	....	....
2265	MW368439.1	|Severe acute respiratory syndrome coro	....	....	....	....	....	....
2266	MW368440.1	|Severe acute respiratory syndrome coro	....	....	....	....	....	....
2267	MW362553.1	|Severe acute respiratory syndrome coro	....	....	....	....	....	....
2268	MW362554.1	|Severe acute respiratory syndrome coro	....	....	....	....	....	....
2269	MW263334.1	|Severe acute respiratory syndrome coro	....	.A..................	....	....	....	....
2270	MW263335.1	|Severe acute respiratory syndrome coro	....	....	....	....	....	....
2271	MW263336.1	|Severe acute respiratory syndrome coro	....	....	....	....	....	....
2272	MW263337.1	|Severe acute respiratory syndrome coro	....	....	....	....	....	....
2273	MW356670.1	|Severe acute respiratory syndrome coro	....	....	....	....	....	....
2274	MW356671.1	|Severe acute respiratory syndrome coro	....	....	....	....	....	....
2275	MW356672.1	|Severe acute respiratory syndrome coro	....	.A..................	....	....	....	...A................
2276	MW356784.1	|Severe acute respiratory syndrome coro	....	....	....	....	....	....
2277	MW356785.1	|Severe acute respiratory syndrome coro	....	....	....	....	......T.............	....
2278	MW356786.1	|Severe acute respiratory syndrome coro	....	....	....	....	....	....
2279	MW345922.1	|Severe acute respiratory syndrome coro	....	....	....	....	....	....
2280	MW345923.1	|Severe acute respiratory syndrome coro	.T..................	....	....	....	....	....
2281	MW345924.1	|Severe acute respiratory syndrome coro	....	.A..................	....	....	....	...A................
2282	MW345925.1	|Severe acute respiratory syndrome coro	....	....	....	....	....	....
2283	MW345926.1	|Severe acute respiratory syndrome coro	....	....A...............	....	....	....	....
2284	MW345927.1	|Severe acute respiratory syndrome coro	....	....A...............	....	....	....	....
2285	MW345928.1	|Severe acute respiratory syndrome coro	....	....	....	....	....	....
2286	MW345929.1	|Severe acute respiratory syndrome coro	....	....	....	....	.......N............	....
2287	MW345930.1	|Severe acute respiratory syndrome coro	....	....	....	....	....	....
2288	MW346179.1	|Severe acute respiratory syndrome coro	....	....	....	....	....	....
2289	MW346315.1	|Severe acute respiratory syndrome coro	....	....	....	....	....	....
2290	MW346329.1	|Severe acute respiratory syndrome coro	....	....	....	....	....	....
2291	MW346331.1	|Severe acute respiratory syndrome coro	....	....	....	....	....	....
2292	MW346340.1	|Severe acute respiratory syndrome coro	....	....	....	....	....	....
2293	MW346345.1	|Severe acute respiratory syndrome coro	....	....	....	....	....	....
2294	MW346348.1	|Severe acute respiratory syndrome coro	....	....	....	....	......T.............	....
2295	MW346358.1	|Severe acute respiratory syndrome coro	....	....	....	....	....	....
2296	MW346365.1	|Severe acute respiratory syndrome coro	....	....	....	....	....	....
2297	MW346366.1	|Severe acute respiratory syndrome coro	....	....	....	....	....	....
2298	MW346369.1	|Severe acute respiratory syndrome coro	....	....	....	....	....	....
2299	MW346370.1	|Severe acute respiratory syndrome coro	....	....	....	....	....	....
2300	MW346371.1	|Severe acute respiratory syndrome coro	....	....	....	....	....	....
2301	MW349032.1	|Severe acute respiratory syndrome coro	....	....	....	....	....	....
2302	MW349033.1	|Severe acute respiratory syndrome coro	....	....	....	....	....	....
2303	MW349034.1	|Severe acute respiratory syndrome coro	....	....	....	....	....	....
2304	MW349035.1	|Severe acute respiratory syndrome coro	....	....	....	....	....	....
2305	MW349036.1	|Severe acute respiratory syndrome coro	....	....	....	....	....	....
2306	MW349037.1	|Severe acute respiratory syndrome coro	....	....	....	....	....	....
2307	MW349038.1	|Severe acute respiratory syndrome coro	....	....	....	....	....	....
2308	MW349039.1	|Severe acute respiratory syndrome coro	....	....	....	....	....	....
2309	MW349040.1	|Severe acute respiratory syndrome coro	....	....	....	....	....	....
2310	MW349041.1	|Severe acute respiratory syndrome coro	....	....	....	....	....	....
2311	MW349042.1	|Severe acute respiratory syndrome coro	....	....	....	....	....	....
2312	MW349043.1	|Severe acute respiratory syndrome coro	....	....	....	....	....	....
2313	MW349044.1	|Severe acute respiratory syndrome coro	....	....	....	....	....	....
2314	MW349045.1	|Severe acute respiratory syndrome coro	....	....	....	....	....	....
2315	MW349046.1	|Severe acute respiratory syndrome coro	....	....	....	....	....	....
2316	MW349047.1	|Severe acute respiratory syndrome coro	....	....	....	....	....	....
2317	MW349048.1	|Severe acute respiratory syndrome coro	....	....	....	....	....	....
2318	MW349049.1	|Severe acute respiratory syndrome coro	....	....	....	....	....	....
2319	MW349050.1	|Severe acute respiratory syndrome coro	....	....	....	....	....	....
2320	MW349051.1	|Severe acute respiratory syndrome coro	....	....	....	....	..NN................	....
2321	MW349052.1	|Severe acute respiratory syndrome coro	....	....	....	....	....	....
2322	MW349053.1	|Severe acute respiratory syndrome coro	....	....	....	....	....	....
2323	MW349054.1	|Severe acute respiratory syndrome coro	....	....	....	....	....	....
2324	MW349055.1	|Severe acute respiratory syndrome coro	....	....	....	....	....	....
2325	MW349056.1	|Severe acute respiratory syndrome coro	....	....	....	....	....	....
2326	MW349057.1	|Severe acute respiratory syndrome coro	....	....	....	....	....	....
2327	MW349058.1	|Severe acute respiratory syndrome coro	....	....	....	....	....	....
2328	MW349059.1	|Severe acute respiratory syndrome coro	....	....	....	....	....	....
2329	MW349060.1	|Severe acute respiratory syndrome coro	....	....	....	....	....	....
2330	MW349061.1	|Severe acute respiratory syndrome coro	....	....	....	....	....	....
2331	MW349062.1	|Severe acute respiratory syndrome coro	....	.A..................	....	....	....	....
2332	MW349063.1	|Severe acute respiratory syndrome coro	....	....	....	....	....	....
2333	MW349064.1	|Severe acute respiratory syndrome coro	....	....	....	....	....	....
2334	MW349065.1	|Severe acute respiratory syndrome coro	....	....	....	....	....	....
2335	MW349066.1	|Severe acute respiratory syndrome coro	....	....	....	....	....	....
2336	MW349067.1	|Severe acute respiratory syndrome coro	....	....	....	....	....	....

```
2337 MW349068.1 |Severe acute respiratory syndrome coro  ····················  ····················  ····················  ····················  ····················  ····················
2338 MW349069.1 |Severe acute respiratory syndrome coro  ····················  ·A··················  ····················  ····················  ····················  ····················
2339 MW349070.1 |Severe acute respiratory syndrome coro  ····················  ·A··················  ····················  ····················  ····················  ····················
2340 MW349071.1 |Severe acute respiratory syndrome coro  ····················  ·A··················  ····················  ····················  ····················  ····················
2341 MW349072.1 |Severe acute respiratory syndrome coro  ····················  ·A··················  ····················  ····················  ····················  ····················
2342 MW349073.1 |Severe acute respiratory syndrome coro  ····················  ·A··················  ····················  ····················  ····················  ····················
2343 MW349074.1 |Severe acute respiratory syndrome coro  ····················  ·A··················  ····················  ····················  ····················  ····················
2344 MW349075.1 |Severe acute respiratory syndrome coro  ····················  ·A··················  ····················  ····················  ····················  ····················
2345 MW349076.1 |Severe acute respiratory syndrome coro  ····················  ·A··················  ····················  ····················  ····················  ····················
2346 MW349077.1 |Severe acute respiratory syndrome coro  ····················  ·A··················  ····················  ····················  ····················  ····················
2347 MW349078.1 |Severe acute respiratory syndrome coro  ····················  ·A··················  ····················  ····················  ····················  ····················
2348 MW349079.1 |Severe acute respiratory syndrome coro  ····················  ·A··················  ····················  ····················  ····················  ····················
2349 MW349080.1 |Severe acute respiratory syndrome coro  ····················  ·A··················  ····················  ····················  ····················  ····················
2350 MW349081.1 |Severe acute respiratory syndrome coro  ····················  ····················  ····················  ····················  ····················  ····················
2351 MW349082.1 |Severe acute respiratory syndrome coro  ····················  ·A··················  ····················  ····················  ····················  ····················
2352 MW349083.1 |Severe acute respiratory syndrome coro  ····················  ·A··················  ····················  ····················  ····················  ····················
2353 MW349084.1 |Severe acute respiratory syndrome coro  ····················  ·A··················  ····················  ····················  ····················  ····················
2354 MW349085.1 |Severe acute respiratory syndrome coro  ····················  ····················  ····················  ····················  ····················  ····················
2355 MW349086.1 |Severe acute respiratory syndrome coro  ····················  ·A··················  ····················  ····················  ····················  ····················
2356 MW349087.1 |Severe acute respiratory syndrome coro  ····················  ····················  ····················  ····················  ····················  ····················
2357 MW349088.1 |Severe acute respiratory syndrome coro  ····················  ·A··················  ····················  ····················  ····················  ····················
2358 MW349089.1 |Severe acute respiratory syndrome coro  ····················  ·A··················  ····················  ····················  ····················  ····················
2359 MW349090.1 |Severe acute respiratory syndrome coro  ····················  ·A··················  ····················  ····················  ····················  ····················
2360 MW349091.1 |Severe acute respiratory syndrome coro  ····················  ·A··················  ····················  ····················  ····················  ····················
2361 MW349092.1 |Severe acute respiratory syndrome coro  ····················  ····················  ····················  ····················  ····················  ····················
2362 MW349093.1 |Severe acute respiratory syndrome coro  ····················  ·A··················  ····················  ····················  ····················  ····················
2363 MW349094.1 |Severe acute respiratory syndrome coro  ····················  ····················  ····················  ····················  ····················  ····················
2364 MW349095.1 |Severe acute respiratory syndrome coro  ····················  ····················  ····················  ····················  ····················  ····················
2365 MW349096.1 |Severe acute respiratory syndrome coro  ····················  ····················  ····················  ····················  ····················  ····················
2366 MW349097.1 |Severe acute respiratory syndrome coro  ····················  ····················  ····················  ····················  ····················  ···R················
2367 MW349098.1 |Severe acute respiratory syndrome coro  ····················  ····················  ····················  ····················  ····················  ····················
2368 MW349099.1 |Severe acute respiratory syndrome coro  ····················  ····················  ····················  ····················  ····················  ····················
2369 MW349100.1 |Severe acute respiratory syndrome coro  ····················  ·A··················  ····················  ····················  ····················  ····················
2370 MW349101.1 |Severe acute respiratory syndrome coro  ····················  ····················  ····················  ····················  ····················  ····················
2371 MW349102.1 |Severe acute respiratory syndrome coro  ····················  ····················  ····················  ····················  ····················  ····················
2372 MW349103.1 |Severe acute respiratory syndrome coro  ····················  ····················  ····················  ····················  ····················  ····················
2373 MW349104.1 |Severe acute respiratory syndrome coro  ····················  ····················  ····················  ····················  ····················  ····················
2374 MW341443.1 |Severe acute respiratory syndrome coro  ····················  ····················  ····················  ····················  ····················  ····················
2375 MW341462.1 |Severe acute respiratory syndrome coro  ····················  ····················  ····················  ····················  ····················  ····················
2376 MW341463.1 |Severe acute respiratory syndrome coro  ····················  ····················  ····················  ····················  ····················  ····················
2377 MW341464.1 |Severe acute respiratory syndrome coro  ····················  ····················  ····················  ····················  ····················  ····················
2378 MW341465.1 |Severe acute respiratory syndrome coro  ····················  ····················  ····················  ····················  ····················  ····················
2379 MW341466.1 |Severe acute respiratory syndrome coro  ····················  ····················  ····················  ····················  ····················  ····················
2380 MW341468.1 |Severe acute respiratory syndrome coro  ····················  ····················  ····················  ····················  ····················  ····················
2381 MW341469.1 |Severe acute respiratory syndrome coro  ····················  ····················  ····················  ····················  ····················  ····················
2382 MW341470.1 |Severe acute respiratory syndrome coro  ····················  ····················  ····················  ····················  ····················  ····················
2383 MW341471.1 |Severe acute respiratory syndrome coro  ····················  ····················  ····················  ····················  ····················  ····················
2384 MW341472.1 |Severe acute respiratory syndrome coro  ····················  ····················  ····················  ····················  ····················  ····················
2385 MW341473.1 |Severe acute respiratory syndrome coro  ····················  ····················  ····················  ····················  ····················  ····················
2386 MW341474.1 |Severe acute respiratory syndrome coro  ····················  ····················  ····················  ····················  ····················  ····················
2387 MW341475.1 |Severe acute respiratory syndrome coro  ····················  ····················  ····················  ····················  ····················  ····················
2388 MW341476.1 |Severe acute respiratory syndrome coro  ····················  ····················  ····················  ····················  ····················  ····················
2389 MW341477.1 |Severe acute respiratory syndrome coro  ····················  ····················  ····················  ····················  ····················  ····················
2390 MW341478.1 |Severe acute respiratory syndrome coro  ····················  ····················  ····················  ····················  ····················  ····················
2391 MW341781.1 |Severe acute respiratory syndrome coro  ····················  ····················  ····················  ····················  ····················  ····················
2392 MW341793.1 |Severe acute respiratory syndrome coro  ····················  ····················  ····················  ····················  ····················  ····················
2393 MW341795.1 |Severe acute respiratory syndrome coro  ····················  ····················  ····················  ····················  ····················  ····················
2394 MW341796.1 |Severe acute respiratory syndrome coro  ····················  ····················  ····················  ····················  ····················  ····················
2395 MW341798.1 |Severe acute respiratory syndrome coro  ····················  ····················  ····················  ····················  ····················  ····················
2396 MW341799.1 |Severe acute respiratory syndrome coro  ····················  ····················  ····················  ····················  ····················  ····················
2397 MW341801.1 |Severe acute respiratory syndrome coro  ····················  ····················  ····················  ····················  ····················  ····················
2398 MW341802.1 |Severe acute respiratory syndrome coro  ····················  ····················  ····················  ····················  ····················  ····················
2399 MW341803.1 |Severe acute respiratory syndrome coro  ····················  ····················  ····················  ····················  ····················  ····················
2400 MW341804.1 |Severe acute respiratory syndrome coro  ····················  ····················  ····················  ····················  ····················  ····················
2401 MW341805.1 |Severe acute respiratory syndrome coro  ····················  ····················  ····················  ····················  ····················  ····················
2402 MW341807.1 |Severe acute respiratory syndrome coro  ····················  ····················  ····················  ····················  ····················  ····················
2403 MW341808.1 |Severe acute respiratory syndrome coro  ····················  ····················  ····················  ····················  ····················  ····················
2404 MW341809.1 |Severe acute respiratory syndrome coro  ····················  ····················  ····················  ····················  ····················  ····················
2405 MW341810.1 |Severe acute respiratory syndrome coro  ····················  ····················  ····················  ····················  ····················  ····················
2406 MW341811.1 |Severe acute respiratory syndrome coro  ····················  ····················  ····················  ····················  ····················  ····················
2407 MW341812.1 |Severe acute respiratory syndrome coro  ····················  ·A··················  ····················  ····················  ····················  ····················
2408 MW341813.1 |Severe acute respiratory syndrome coro  ····················  ····················  ····················  ····················  ····················  ····················
2409 MW341814.1 |Severe acute respiratory syndrome coro  ····················  ····················  ····················  ····················  ····················  ····················
2410 MW341815.1 |Severe acute respiratory syndrome coro  ····················  ····················  ····················  ····················  ····················  ····················
2411 MW341818.1 |Severe acute respiratory syndrome coro  ····················  ····················  ····················  ····················  ····················  ····················
2412 MW341820.1 |Severe acute respiratory syndrome coro  ····················  ····················  ····················  ····················  ····················  ····················
2413 MW341822.1 |Severe acute respiratory syndrome coro  ····················  ····················  ····················  ····················  ····················  ····················
2414 MW341823.1 |Severe acute respiratory syndrome coro  ····················  ····················  ····················  ····················  ····················  ····················
```

2415	MW341825.1	Severe acute respiratory syndrome coro	····················	····················	····················	····················	····················	····················
2416	MW341826.1	Severe acute respiratory syndrome coro	····················	····················	····················	····················	····················	····················
2417	MW341827.1	Severe acute respiratory syndrome coro	····················	·A··················	····················	····················	····················	····················
2418	MW341830.1	Severe acute respiratory syndrome coro	····················	····················	····················	····················	····················	····················
2419	MW341831.1	Severe acute respiratory syndrome coro	····················	····················	····················	····················	····················	····················
2420	MW341832.1	Severe acute respiratory syndrome coro	····················	··C·················	····················	····················	····················	····················
2421	MW341834.1	Severe acute respiratory syndrome coro	····················	····················	····················	····················	····················	····················
2422	MW341835.1	Severe acute respiratory syndrome coro	····················	····················	····················	····················	····················	····················
2423	MW341836.1	Severe acute respiratory syndrome coro	····················	··N·················	····················	····················	····················	····················
2424	MW341852.1	Severe acute respiratory syndrome coro	····················	····················	····················	····················	····················	····················
2425	MW341860.1	Severe acute respiratory syndrome coro	····················	····················	····················	····················	····················	····················
2426	MW341861.1	Severe acute respiratory syndrome coro	····················	····················	····················	····················	····················	····················
2427	MW341862.1	Severe acute respiratory syndrome coro	····················	····················	····················	····················	····················	····················
2428	MW341863.1	Severe acute respiratory syndrome coro	····················	····················	····················	····················	····················	····················
2429	MW341864.1	Severe acute respiratory syndrome coro	····················	····················	····················	····················	····················	····················
2430	MW341865.1	Severe acute respiratory syndrome coro	····················	····················	····················	····················	····················	····················
2431	MW341866.1	Severe acute respiratory syndrome coro	····················	····················	····················	····················	····················	····················
2432	MW341867.1	Severe acute respiratory syndrome coro	····················	····················	····················	····················	····················	····················
2433	MW341868.1	Severe acute respiratory syndrome coro	····················	····················	····················	····················	····················	····················
2434	MW341869.1	Severe acute respiratory syndrome coro	····················	·A··················	····················	····················	····················	····················
2435	MW341870.1	Severe acute respiratory syndrome coro	····················	····················	····················	····················	····················	····················
2436	MW341871.1	Severe acute respiratory syndrome coro	····················	····················	····················	····················	····················	····················
2437	MW341872.1	Severe acute respiratory syndrome coro	····················	····················	····················	····················	····················	····················
2438	MW341873.1	Severe acute respiratory syndrome coro	····················	····················	····················	····················	····················	····················
2439	MW341874.1	Severe acute respiratory syndrome coro	····················	····················	····················	····················	····················	····················
2440	MW341875.1	Severe acute respiratory syndrome coro	····················	····················	····················	····················	····················	····················
2441	MW341876.1	Severe acute respiratory syndrome coro	····················	·A··················	····················	····················	····················	····················
2442	MW341877.1	Severe acute respiratory syndrome coro	····················	····················	····················	····················	····················	····················
2443	MW341878.1	Severe acute respiratory syndrome coro	····················	····················	····················	····················	····················	····················
2444	MW341879.1	Severe acute respiratory syndrome coro	····················	····················	····················	····················	····················	····················
2445	MW341880.1	Severe acute respiratory syndrome coro	····················	····················	····················	····················	····················	····················
2446	MW341881.1	Severe acute respiratory syndrome coro	····················	····················	····················	····················	····················	····················
2447	MW341882.1	Severe acute respiratory syndrome coro	····················	····················	····················	····················	····················	····················
2448	MW341900.1	Severe acute respiratory syndrome coro	····················	····················	····················	····················	····················	····················
2449	MW341915.1	Severe acute respiratory syndrome coro	····················	····················	····················	····················	····················	····················
2450	MW341916.1	Severe acute respiratory syndrome coro	····················	····················	····················	····················	····················	····················
2451	MW341918.1	Severe acute respiratory syndrome coro	····················	····················	····················	····················	····················	····················
2452	MW341919.1	Severe acute respiratory syndrome coro	····················	····················	····················	····················	····················	····················
2453	MW341920.1	Severe acute respiratory syndrome coro	····················	····················	····················	····················	····················	····················
2454	MW341921.1	Severe acute respiratory syndrome coro	····················	····················	····················	····················	····················	····················
2455	MW341923.1	Severe acute respiratory syndrome coro	····················	····················	····················	····················	····················	····················
2456	MW341927.1	Severe acute respiratory syndrome coro	····················	····················	····················	····················	····················	····················
2457	MW341928.1	Severe acute respiratory syndrome coro	····················	····················	····················	····················	····················	····················
2458	MW341929.1	Severe acute respiratory syndrome coro	····················	····················	····················	····················	····················	····················
2459	MW341930.1	Severe acute respiratory syndrome coro	····················	····················	····················	····················	····················	····················
2460	MW341931.1	Severe acute respiratory syndrome coro	····················	····················	····················	····················	····················	····················
2461	MW341932.1	Severe acute respiratory syndrome coro	····················	····················	····················	····················	····················	····················
2462	MW341933.1	Severe acute respiratory syndrome coro	····················	····················	····················	····················	····················	····················
2463	MW341935.1	Severe acute respiratory syndrome coro	····················	····················	····················	····················	····················	····················
2464	MW341937.1	Severe acute respiratory syndrome coro	····················	····················	····················	····················	····················	····················
2465	MW341938.1	Severe acute respiratory syndrome coro	····················	····················	····················	····················	····················	····················
2466	MW341939.1	Severe acute respiratory syndrome coro	····················	····················	····················	····················	····················	····················
2467	MW341940.1	Severe acute respiratory syndrome coro	····················	····················	····················	····················	····················	····················
2468	MW341943.1	Severe acute respiratory syndrome coro	····················	····················	····················	····················	····················	····················
2469	MW341947.1	Severe acute respiratory syndrome coro	····················	····················	····················	····················	····················	····················
2470	MW341948.1	Severe acute respiratory syndrome coro	····················	····················	····················	····················	····················	····················
2471	MW341951.1	Severe acute respiratory syndrome coro	····················	····················	····················	····················	····················	····················
2472	MW341952.1	Severe acute respiratory syndrome coro	····················	····················	····················	····················	····················	····················
2473	MW341953.1	Severe acute respiratory syndrome coro	····················	····················	····················	····················	····················	····················
2474	MW341954.1	Severe acute respiratory syndrome coro	····················	····················	····················	····················	····················	····················
2475	MW341955.1	Severe acute respiratory syndrome coro	····················	·A··················	····················	····················	····················	····················
2476	MW341958.1	Severe acute respiratory syndrome coro	····················	····················	····················	····················	····················	····················
2477	MW341959.1	Severe acute respiratory syndrome coro	····················	····················	····················	····················	····················	····················
2478	MW341960.1	Severe acute respiratory syndrome coro	····················	····················	····················	····················	····················	····················
2479	MW341966.1	Severe acute respiratory syndrome coro	····················	····················	····················	····················	····················	····················
2480	MW341968.1	Severe acute respiratory syndrome coro	····················	····················	····················	····················	····················	····················
2481	MW341982.1	Severe acute respiratory syndrome coro	····················	····················	····················	····················	····················	····················
2482	MW341987.1	Severe acute respiratory syndrome coro	····················	····················	····················	····················	····················	····················
2483	MW341995.1	Severe acute respiratory syndrome coro	····················	····················	····················	····················	····················	····················
2484	MW342000.1	Severe acute respiratory syndrome coro	····················	····················	····················	····················	····················	····················
2485	MW342006.1	Severe acute respiratory syndrome coro	····················	····················	····················	····················	····················	····················
2486	MW342047.1	Severe acute respiratory syndrome coro	····················	····················	····················	····················	····················	····················
2487	MW342051.1	Severe acute respiratory syndrome coro	····················	····················	····················	····················	····················	····················
2488	MW342052.1	Severe acute respiratory syndrome coro	····················	····················	····················	····················	····················	····················
2489	MW342053.1	Severe acute respiratory syndrome coro	····················	····················	····················	····················	····················	····················
2490	MW342054.1	Severe acute respiratory syndrome coro	····················	····················	····················	····················	····················	····················
2491	MW342056.1	Severe acute respiratory syndrome coro	····················	····················	····················	····················	····················	····················
2492	MW342057.1	Severe acute respiratory syndrome coro	····················	····················	····················	····················	····················	····················

```
2493 MW342058.1 |Severe acute respiratory syndrome coro  ····················  ····················  ·····················  ····················  ····················  ····················
2494 MW342061.1 |Severe acute respiratory syndrome coro  ····················  ····················  ·····················  ····················  ····················  ····················
2495 MW342062.1 |Severe acute respiratory syndrome coro  ····················  ····················  ·····················  ····················  ····················  ····················
2496 MW342063.1 |Severe acute respiratory syndrome coro  ····················  ····················  ·····················  ····················  ····················  ····················
2497 MW342064.1 |Severe acute respiratory syndrome coro  ····················  ····················  ·····················  ····················  ····················  ····················
2498 MW342065.1 |Severe acute respiratory syndrome coro  ····················  ····················  ·····················  ····················  ····················  ····················
2499 MW342066.1 |Severe acute respiratory syndrome coro  ····················  ····················  ·····················  ····················  ····················  ····················
2500 MW342068.1 |Severe acute respiratory syndrome coro  ····················  ····················  ·····················  ····················  ····················  ····················
2501 MW342069.1 |Severe acute respiratory syndrome coro  ····················  ····················  ·····················  ····················  ····················  ····················
2502 MW342070.1 |Severe acute respiratory syndrome coro  ····················  ·N··················  ·····················  ····················  ····················  ····················
2503 MW342071.1 |Severe acute respiratory syndrome coro  ····················  ····················  ·····················  ····················  ····················  ····················
2504 MW342072.1 |Severe acute respiratory syndrome coro  ····················  ····················  ·····················  ····················  ····················  ····················
2505 MW342073.1 |Severe acute respiratory syndrome coro  ····················  ····················  ·····················  ····················  ····················  ····················
2506 MW342075.1 |Severe acute respiratory syndrome coro  ····················  ····················  ·····················  ····················  ····················  ····················
2507 MW342076.1 |Severe acute respiratory syndrome coro  ····················  ····················  ·····················  ····················  ····················  ····················
2508 MW342077.1 |Severe acute respiratory syndrome coro  ····················  ····················  ·····················  ····················  ····················  ····················
2509 MW342078.1 |Severe acute respiratory syndrome coro  ····················  ····················  ·····················  ····················  ····················  ····················
2510 MW342079.1 |Severe acute respiratory syndrome coro  ····················  ····················  ·····················  ····················  ····················  ····················
2511 MW342083.1 |Severe acute respiratory syndrome coro  ····················  ····················  ·····················  ····················  ····················  ····················
2512 MW342085.1 |Severe acute respiratory syndrome coro  ····················  ····················  ·····················  ····················  ····················  ····················
2513 MW342086.1 |Severe acute respiratory syndrome coro  ····················  ····················  ·····················  ····················  ····················  ····················
2514 MW342087.1 |Severe acute respiratory syndrome coro  ····················  ····················  ·····················  ····················  ····················  ····················
2515 MW342089.1 |Severe acute respiratory syndrome coro  ····N···············  ····················  ·····················  ····················  ····················  ····················
2516 MW342090.1 |Severe acute respiratory syndrome coro  ····················  ····················  ·····················  ····················  ····················  ····················
2517 MW342091.1 |Severe acute respiratory syndrome coro  ····················  ····················  ·····················  ····················  ····················  ····················
2518 MW342092.1 |Severe acute respiratory syndrome coro  ····················  ····················  ·····················  ····················  ····················  ····················
2519 MW342093.1 |Severe acute respiratory syndrome coro  ····················  ····················  ·····················  ····················  ····················  ····················
2520 MW342095.1 |Severe acute respiratory syndrome coro  ····················  ···A················  ·····················  ····················  ····················  ··A·················
2521 MW342096.1 |Severe acute respiratory syndrome coro  ····················  ····················  ·····················  ····················  ····················  ····················
2522 MW342097.1 |Severe acute respiratory syndrome coro  ····················  ····················  ·····················  ····················  ····················  ····················
2523 MW342706.1 |Severe acute respiratory syndrome coro  ····················  ····················  ·····················  ····················  ····················  ····················
2524 MW342708.1 |Severe acute respiratory syndrome coro  ····················  ····················  ·····················  ····················  ····················  ····················
2525 MW343734.1 |Severe acute respiratory syndrome coro  ····················  ····················  ·····················  ····················  ····················  ····················
2526 MW343735.1 |Severe acute respiratory syndrome coro  ····················  ····················  ·····················  ····················  ····················  ····················
2527 MW343736.1 |Severe acute respiratory syndrome coro  ····················  ····················  ·····················  ····················  ····················  ····················
2528 MW343737.1 |Severe acute respiratory syndrome coro  ····················  ····················  ·····················  ····················  ····················  ····················
2529 MW343738.1 |Severe acute respiratory syndrome coro  ····················  ····················  ·····················  ····················  ····················  ····················
2530 MW343739.1 |Severe acute respiratory syndrome coro  ····················  ····················  ·····················  ····················  ····················  ····················
2531 MW343740.1 |Severe acute respiratory syndrome coro  ····················  ····················  ·····················  ····················  ····················  ····················
2532 MW343741.1 |Severe acute respiratory syndrome coro  ····················  ····················  ·····················  ····················  ····················  ····················
2533 MW343742.1 |Severe acute respiratory syndrome coro  ····················  ····················  ·····················  ····················  ····················  ····················
2534 MW343743.1 |Severe acute respiratory syndrome coro  ····················  ····················  ·····················  ····················  ····················  ····················
2535 MW343744.1 |Severe acute respiratory syndrome coro  ····················  ····················  ·····················  ····················  ····················  ····················
2536 MW343745.1 |Severe acute respiratory syndrome coro  ····················  ····················  ·····················  ····················  ····················  ····················
2537 MW343746.1 |Severe acute respiratory syndrome coro  ····················  ····················  ·····················  ····················  ····················  ····················
2538 MW343747.1 |Severe acute respiratory syndrome coro  ····················  ····················  ·····················  ····················  ····················  ····················
2539 MW343748.1 |Severe acute respiratory syndrome coro  ····················  ····················  ·····················  ····················  ····················  ····················
2540 MW343749.1 |Severe acute respiratory syndrome coro  ····················  ····················  ·····················  ····················  ····················  ····················
2541 MW343750.1 |Severe acute respiratory syndrome coro  ····················  ····················  ·····················  ····················  ····················  ····················
2542 MW343751.1 |Severe acute respiratory syndrome coro  ····················  ····················  ·····················  ····················  ····················  ····················
2543 MW343752.1 |Severe acute respiratory syndrome coro  ····················  ····················  ·····················  ····················  ····················  ····················
2544 MW343753.1 |Severe acute respiratory syndrome coro  ····················  ····················  ·····················  ····················  ····················  ····················
2545 MW343754.1 |Severe acute respiratory syndrome coro  ····················  ····················  ·····················  ····················  ····················  ····················
2546 MW343755.1 |Severe acute respiratory syndrome coro  ····················  ····················  ·····················  ····················  ····················  ····················
2547 MW343756.1 |Severe acute respiratory syndrome coro  ····················  ····················  ·····················  ····················  ····················  ····················
2548 MW343757.1 |Severe acute respiratory syndrome coro  ····················  ····················  ·····················  ····················  ····················  ····················
2549 MW343758.1 |Severe acute respiratory syndrome coro  ····················  ····················  ·····················  ····················  ····················  ····················
2550 MW343759.1 |Severe acute respiratory syndrome coro  ····················  ····················  ·····················  ····················  ····················  ····················
2551 MW343760.1 |Severe acute respiratory syndrome coro  ····················  ····················  ·····················  ····················  ····················  ····················
2552 MW343761.1 |Severe acute respiratory syndrome coro  ····················  ····················  ·····················  ····················  ····················  ····················
2553 MW343762.1 |Severe acute respiratory syndrome coro  ····················  ····················  ·····················  ····················  ····················  ····················
2554 MW343763.1 |Severe acute respiratory syndrome coro  ····················  ····················  ·····················  ····················  ····················  ····················
2555 MW343764.1 |Severe acute respiratory syndrome coro  ····················  ····················  ·····················  ····················  ····················  ····················
2556 MW343765.1 |Severe acute respiratory syndrome coro  ····················  ····················  ·····················  ····················  ····················  ····················
2557 MW343766.1 |Severe acute respiratory syndrome coro  ····················  ····················  ·····················  ····················  ····················  ····················
2558 MW343767.1 |Severe acute respiratory syndrome coro  ····················  ····················  ·····················  ····················  ····················  ····················
2559 MW343768.1 |Severe acute respiratory syndrome coro  ····················  ····················  ·····················  ····················  ····················  ····················
2560 MW343769.1 |Severe acute respiratory syndrome coro  ····················  ····················  ·····················  ····················  ····················  ····················
2561 MW343770.1 |Severe acute respiratory syndrome coro  ····················  ····················  ·····················  ····················  ····················  ····················
2562 MW343771.1 |Severe acute respiratory syndrome coro  ····················  ····················  ·····················  ····················  ····················  ····················
2563 MW343772.1 |Severe acute respiratory syndrome coro  ····················  ····················  ·····················  ····················  ····················  ····················
2564 MW343773.1 |Severe acute respiratory syndrome coro  ····················  ····················  ·····················  ····················  ····················  ····················
2565 MW343774.1 |Severe acute respiratory syndrome coro  ····················  ····················  ·····················  ····················  ····················  ····················
2566 MW343775.1 |Severe acute respiratory syndrome coro  ····················  ····················  ·····················  ····················  ····················  ····················
2567 MW343776.1 |Severe acute respiratory syndrome coro  ····················  ····················  ·····················  ····················  ····················  ····················
2568 MW343777.1 |Severe acute respiratory syndrome coro  ····················  ····················  ·····················  ····················  ····················  ····················
2569 MW343778.1 |Severe acute respiratory syndrome coro  ····················  ····················  ·····················  ····················  ····················  ····················
2570 MW343779.1 |Severe acute respiratory syndrome coro  ····················  ····················  ·····················  ····················  ····················  ····················
```

2571 MW343780.1 |Severe acute respiratory syndrome coro ···················· ···················· ····················· ···················· ···················· ····················
2572 MW343781.1 |Severe acute respiratory syndrome coro ···················· ···················· ····················· ···················· ···················· ····················
2573 MW343782.1 |Severe acute respiratory syndrome coro ···················· ···················· ····················· ···················· ···················· ····················
2574 MW343783.1 |Severe acute respiratory syndrome coro ···················· ···················· ····················· ···················· ···················· ····················
2575 MW343784.1 |Severe acute respiratory syndrome coro ···················· ···················· ····················· ···················· ···················· ····················
2576 MW343785.1 |Severe acute respiratory syndrome coro ···················· ···················· ····················· ···················· ···················· ····················
2577 MW343786.1 |Severe acute respiratory syndrome coro ···················· ···················· ····················· ···················· ···················· ····················
2578 MW345264.1 |Severe acute respiratory syndrome coro ···················· ·A·················· ····················· ···················· ···················· ····················
2579 MW345265.1 |Severe acute respiratory syndrome coro ···················· ···················· ····················· ···················· ···················· ····················
2580 MW345266.1 |Severe acute respiratory syndrome coro ···················· ···················· ····················· ···················· ···················· ····················
2581 MW345267.1 |Severe acute respiratory syndrome coro ···················· ···················· ····················· ···················· ···················· ····················
2582 MW345268.1 |Severe acute respiratory syndrome coro ···················· ·A·················· ····················· ···················· ···················· ····················
2583 MW345269.1 |Severe acute respiratory syndrome coro ···················· ···················· ····················· ···················· ···················· ····················
2584 MW345270.1 |Severe acute respiratory syndrome coro ···················· ···················· ····················· ···················· ···················· ····················
2585 MW345271.1 |Severe acute respiratory syndrome coro ···················· ···················· ····················· ···················· ···················· ····················
2586 MW345272.1 |Severe acute respiratory syndrome coro ···················· ···················· ····················· ···················· ···················· ····················
2587 MW345273.1 |Severe acute respiratory syndrome coro ···················· ···················· ····················· ···················· ···················· ····················
2588 MW345274.1 |Severe acute respiratory syndrome coro ···················· ···················· ····················· ···················· ···················· ····················
2589 MW345275.1 |Severe acute respiratory syndrome coro ···················· ···················· ····················· ···················· ···················· ····················
2590 MW345276.1 |Severe acute respiratory syndrome coro ···················· ···················· ····················· ···················· ···················· ····················
2591 MW345277.1 |Severe acute respiratory syndrome coro ···················· ············C······· ····················· ···················· ···················· ····················
2592 MW345278.1 |Severe acute respiratory syndrome coro ···················· ···················· ····················· ···················· ···················· ····················
2593 MW345279.1 |Severe acute respiratory syndrome coro ···················· ···················· ····················· ···················· ···················· ····················
2594 MW345280.1 |Severe acute respiratory syndrome coro ···················· ···················· ····················· ···················· ···················· ····················
2595 MW345281.1 |Severe acute respiratory syndrome coro ···················· ···················· ····················· ···················· ···················· ····················
2596 MW345283.1 |Severe acute respiratory syndrome coro ···················· ···················· ····················· ···················· ···················· ····················
2597 MW345284.1 |Severe acute respiratory syndrome coro ···················· ···················· ····················· ···················· ···················· ····················
2598 MW345285.1 |Severe acute respiratory syndrome coro ···················· ···················· ····················· ···················· ···················· ····················
2599 MW345824.1 |Severe acute respiratory syndrome coro ···················· ···················· ··T·················· ···················· ···················· ····················
2600 MW345825.1 |Severe acute respiratory syndrome coro ···················· ···················· ····················· ···················· ···················· ····················
2601 MW332531.1 |Severe acute respiratory syndrome coro ···················· ···················· ····················· ···················· ···················· ····················
2602 MW332532.1 |Severe acute respiratory syndrome coro ···················· ···················· ····················· ···················· ···················· ····················
2603 MW332533.1 |Severe acute respiratory syndrome coro ···················· ···················· ····················· ···················· ···················· ····················
2604 MW332534.1 |Severe acute respiratory syndrome coro ···················· ···················· ····················· ···················· ···················· ····················
2605 MW332535.1 |Severe acute respiratory syndrome coro ···················· ···················· ····················· ···················· ···················· ····················
2606 MW332536.1 |Severe acute respiratory syndrome coro ···················· ···················· ····················· ···················· ···················· ····················
2607 MW332537.1 |Severe acute respiratory syndrome coro ···················· ···················· ····················· ···················· ···················· ····················
2608 MW332538.1 |Severe acute respiratory syndrome coro ···················· ···················· ····················· ···················· ···················· ····················
2609 MW332539.1 |Severe acute respiratory syndrome coro ···················· ···················· ····················· ···················· ···················· ····················
2610 MW332540.1 |Severe acute respiratory syndrome coro ···················· ···················· ····················· ···················· ···················· ····················
2611 MW332551.1 |Severe acute respiratory syndrome coro ···················· ···················· ····················· ···················· ···················· ····················
2612 MW332552.1 |Severe acute respiratory syndrome coro ···················· ···················· ····················· ···················· ···················· ····················
2613 MW332553.1 |Severe acute respiratory syndrome coro ···················· ···················· ····················· ···················· ···················· ····················
2614 MW332554.1 |Severe acute respiratory syndrome coro ···················· ···················· ····················· ···················· ···················· ····················
2615 MW332555.1 |Severe acute respiratory syndrome coro ···················· ···················· ····················· ···················· ···················· ····················
2616 MW332558.1 |Severe acute respiratory syndrome coro ···················· ···················· ····················· ···················· ···················· ····················
2617 MW332559.1 |Severe acute respiratory syndrome coro ········T··········· ···················· ····················· ···················· ···················· ····················
2618 MW332560.1 |Severe acute respiratory syndrome coro ···················· ···················· ····················· ···················· ···················· ····················
2619 MW332561.1 |Severe acute respiratory syndrome coro ···················· ···················· ····················· ···················· ···················· ····················
2620 MW332562.1 |Severe acute respiratory syndrome coro ···················· ···················· ····················· ···················· ···················· ····················
2621 MW332563.1 |Severe acute respiratory syndrome coro ···················· ···················· ····················· ···················· ···················· ····················
2622 MW332564.1 |Severe acute respiratory syndrome coro ···········T········ ···················· ····················· ···················· ···················· ····················
2623 MW332652.1 |Severe acute respiratory syndrome coro ···················· ···················· ····················· ···················· ···················· ····················
2624 MW332653.1 |Severe acute respiratory syndrome coro ···················· ···················· ····················· ···················· ···················· ····················
2625 MW332655.1 |Severe acute respiratory syndrome coro ···················· ···················· ····················· ···················· ···················· ····················
2626 MW332656.1 |Severe acute respiratory syndrome coro ···················· ···················· ····················· ···················· ···················· ····················
2627 MW332657.1 |Severe acute respiratory syndrome coro ···················· ···················· ····················· ···················· ···················· ····················
2628 MW332658.1 |Severe acute respiratory syndrome coro ···················· ···················· ····················· ···················· ···················· ····················
2629 MW332659.1 |Severe acute respiratory syndrome coro ···················· ···················· ····················· ···················· ···················· ····················
2630 MW332662.1 |Severe acute respiratory syndrome coro ···················· ···················· ····················· ···················· ···················· ····················
2631 MW332664.1 |Severe acute respiratory syndrome coro ···················· ···················· ····················· ···················· ···················· ····················
2632 MW332665.1 |Severe acute respiratory syndrome coro ···················· ···················· ····················· ···················· ···················· ····················
2633 MW332666.1 |Severe acute respiratory syndrome coro ···················· ···················· ····················· ···················· ···················· ····················
2634 MW332667.1 |Severe acute respiratory syndrome coro ···················· ···················· ····················· ···················· ···················· ····················
2635 MW332668.1 |Severe acute respiratory syndrome coro ···················· ···················· ····················· ···················· ···················· ····················
2636 MW332669.1 |Severe acute respiratory syndrome coro ···················· ···················· ····················· ···················· ···················· ····················
2637 MW332670.1 |Severe acute respiratory syndrome coro ···················· ···················· ····················· ···················· ···················· ····················
2638 MW332671.1 |Severe acute respiratory syndrome coro ···················· ···················· ····················· ···················· ···················· ····················
2639 MW332672.1 |Severe acute respiratory syndrome coro ···················· ···················· ····················· ···················· ···················· ····················
2640 MW332673.1 |Severe acute respiratory syndrome coro ···················· ···················· ····················· ···················· ···················· ····················
2641 MW332674.1 |Severe acute respiratory syndrome coro ···················· ···················· ····················· ···················· ···················· ····················
2642 MW332675.1 |Severe acute respiratory syndrome coro ···················· ···················· ····················· ···················· ···················· ····················
2643 MW332679.1 |Severe acute respiratory syndrome coro ···················· ···················· ····················· ···················· ···················· ····················
2644 MW332686.1 |Severe acute respiratory syndrome coro ···················· ···················· ····················· ···················· ···················· ····················
2645 MW332688.1 |Severe acute respiratory syndrome coro ···················· ···················· ····················· ···················· ···················· ····················
2646 MW332691.1 |Severe acute respiratory syndrome coro ···················· ···················· ····················· ···················· ···················· ····················
2647 MW332694.1 |Severe acute respiratory syndrome coro ···················· ···················· ····················· ···················· ···················· ····················
2648 MW332712.1 |Severe acute respiratory syndrome coro ···················· ···················· ····················· ···················· ···················· ····················

```
2649 MW332713.1 |Severe acute respiratory syndrome coro    ....................  ....................  ....................  ....................  ....................  ....................
2650 MW332714.1 |Severe acute respiratory syndrome coro    ....................  ....................  ....................  ....................  ....................  ....................
2651 MW332715.1 |Severe acute respiratory syndrome coro    ....................  ....................  ....................  ....................  ....................  ....................
2652 MW332720.1 |Severe acute respiratory syndrome coro    ....................  ....................  ....................  ....................  ....................  ....................
2653 MW332721.1 |Severe acute respiratory syndrome coro    ....................  ....................  ....................  ....................  ....................  ....................
2654 MW332722.1 |Severe acute respiratory syndrome coro    ....................  ....................  ....................  ....................  ....................  ....................
2655 MW332723.1 |Severe acute respiratory syndrome coro    ....................  ....................  ....................  ....................  ....................  ....................
2656 MW332725.1 |Severe acute respiratory syndrome coro    ....................  ....................  ....................  ....................  ....................  ....................
2657 MW332726.1 |Severe acute respiratory syndrome coro    ....................  ....................  ....................  ....................  ....................  ....................
2658 MW332728.1 |Severe acute respiratory syndrome coro    ....................  ....................  ....................  ....................  ....................  ....................
2659 MW332729.1 |Severe acute respiratory syndrome coro    ....................  ....................  ....................  ....................  ....................  ....................
2660 MW332731.1 |Severe acute respiratory syndrome coro    ....................  ....................  ....................  ....................  ....................  ....................
2661 MW332732.1 |Severe acute respiratory syndrome coro    ....................  ....................  ....................  ....................  ....................  ....................
2662 MW332733.1 |Severe acute respiratory syndrome coro    ....................  ....................  ....................  ....................  ....................  ....................
2663 MW332735.1 |Severe acute respiratory syndrome coro    ....................  ....................  ....................  ....................  ....................  ....................
2664 MW332736.1 |Severe acute respiratory syndrome coro    ....................  ....................  ....................  ....................  ....................  ....................
2665 MW332738.1 |Severe acute respiratory syndrome coro    ....................  ....................  ....................  ....................  ....................  ....................
2666 MW332739.1 |Severe acute respiratory syndrome coro    ....................  ....................  ....................  ....................  ....................  ....................
2667 MW332740.1 |Severe acute respiratory syndrome coro    ....................  ....................  ....................  ....................  ....................  ....................
2668 MW332741.1 |Severe acute respiratory syndrome coro    ....................  ....................  ....................  ....................  ....................  ....................
2669 MW332742.1 |Severe acute respiratory syndrome coro    ....................  ....................  ....................  ....................  ....................  ....................
2670 MW332744.1 |Severe acute respiratory syndrome coro    ....................  ....................  ....................  ....................  ....................  ....................
2671 MW332748.1 |Severe acute respiratory syndrome coro    ....................  .A..................  ....................  ....................  ....................  ....................
2672 MW332750.1 |Severe acute respiratory syndrome coro    ....................  ....................  ....................  ....................  ....................  ....................
2673 MW332751.1 |Severe acute respiratory syndrome coro    ....................  ....................  ....................  ....................  ....................  ....................
2674 MW332752.1 |Severe acute respiratory syndrome coro    ....................  ....................  ....................  ....................  ....................  ....................
2675 MW332753.1 |Severe acute respiratory syndrome coro    ....................  ....................  ....................  ....................  ....................  ....................
2676 MW332754.1 |Severe acute respiratory syndrome coro    ....................  ....................  ....................  ....................  ....................  ....................
2677 MW332755.1 |Severe acute respiratory syndrome coro    ....................  ....................  ....................  ....................  ....................  ....................
2678 MW332757.1 |Severe acute respiratory syndrome coro    ....................  ....................  ....................  ....................  ....................  ....................
2679 MW332758.1 |Severe acute respiratory syndrome coro    ....................  ....................  ....................  ....................  ....................  ....................
2680 MW332759.1 |Severe acute respiratory syndrome coro    ....................  ....................  ....................  ....................  ....................  ....................
2681 MW332761.1 |Severe acute respiratory syndrome coro    ....................  ....................  ....................  ....................  ....................  ....................
2682 MW332764.1 |Severe acute respiratory syndrome coro    ....................  ....................  ....................  ....................  ....................  ....................
2683 MW332766.1 |Severe acute respiratory syndrome coro    ....................  ....................  ....................  ....................  ....................  ....................
2684 MW332767.1 |Severe acute respiratory syndrome coro    ....................  ....................  ....................  ....................  ....................  ....................
2685 MW332768.1 |Severe acute respiratory syndrome coro    ....................  ....................  ....................  ....................  ....................  ....................
2686 MW332769.1 |Severe acute respiratory syndrome coro    ....................  ....................  ....................  ....................  ....................  ....................
2687 MW332770.1 |Severe acute respiratory syndrome coro    ....................  ....................  ....................  ....................  ....................  ....................
2688 MW332771.1 |Severe acute respiratory syndrome coro    ....................  ....................  ....................  ....................  ....................  ....................
2689 MW332772.1 |Severe acute respiratory syndrome coro    ....................  ....................  ....................  ....................  ....................  ....................
2690 MW332773.1 |Severe acute respiratory syndrome coro    ....................  ....................  ....................  ....................  ....................  ....................
2691 MW332775.1 |Severe acute respiratory syndrome coro    ....................  ....................  ....................  ....................  ....................  ....................
2692 MW332776.1 |Severe acute respiratory syndrome coro    ....................  ....................  ....................  ....................  ....................  ....................
2693 MW332777.1 |Severe acute respiratory syndrome coro    ....................  ....................  ....................  ....................  ....................  ....................
2694 MW332778.1 |Severe acute respiratory syndrome coro    ....................  ....................  ....................  ....................  ....................  ....................
2695 MW332779.1 |Severe acute respiratory syndrome coro    ....................  ....................  ....................  ....................  ....................  ....................
2696 MW332782.1 |Severe acute respiratory syndrome coro    ....................  ....................  ....................  ....................  ....................  ....................
2697 MW332784.1 |Severe acute respiratory syndrome coro    ....................  ....................  ....................  ....................  ....................  ....................
2698 MW332785.1 |Severe acute respiratory syndrome coro    ....................  ....................  ....................  ....................  ....................  ....................
2699 MW332786.1 |Severe acute respiratory syndrome coro    ....................  ....................  ....................  ....................  ....................  ....................
2700 MW332788.1 |Severe acute respiratory syndrome coro    ....................  ....................  ....................  ....................  ....................  ....................
2701 MW332790.1 |Severe acute respiratory syndrome coro    ....................  ....................  ....................  ....................  ....................  ....................
2702 MW332791.1 |Severe acute respiratory syndrome coro    ....................  ....................  ....................  ....................  ....................  ....................
2703 MW332793.1 |Severe acute respiratory syndrome coro    ....................  ....................  ....................  ....................  ....................  ....................
2704 MW332794.1 |Severe acute respiratory syndrome coro    ....................  ....................  ....................  ....................  ....................  ....................
2705 MW332796.1 |Severe acute respiratory syndrome coro    ....................  ....................  ....................  ....................  ....................  ....................
2706 MW332797.1 |Severe acute respiratory syndrome coro    ....................  ....................  ....................  ....................  ....................  ....................
2707 MW332803.1 |Severe acute respiratory syndrome coro    ....................  ....................  ....................  ....................  ....................  ....................
2708 MW332806.1 |Severe acute respiratory syndrome coro    ....................  ....................  ....................  ....................  ....................  ....................
2709 MW332808.1 |Severe acute respiratory syndrome coro    ....................  .............A......  ....................  ....................  ....................  ....................
2710 MW332811.1 |Severe acute respiratory syndrome coro    ....................  ....................  ....................  ....................  ....................  ....................
2711 MW332812.1 |Severe acute respiratory syndrome coro    ....................  ....................  ....................  ....................  ....................  ....................
2712 MW332814.1 |Severe acute respiratory syndrome coro    ....................  ....................  ....................  ....................  ....................  ....................
2713 MW332817.1 |Severe acute respiratory syndrome coro    ....................  ....................  ....................  ....................  ....................  ....................
2714 MW332820.1 |Severe acute respiratory syndrome coro    ....................  ....................  ....................  ....................  ....................  ....................
2715 MW332822.1 |Severe acute respiratory syndrome coro    ....................  ....................  ....................  ....................  ....................  ....................
2716 MW332823.1 |Severe acute respiratory syndrome coro    ....................  ....................  ....................  ....................  ....................  ....................
2717 MW332825.1 |Severe acute respiratory syndrome coro    ....................  ....................  ....................  ....................  ....................  ....................
2718 MW332827.1 |Severe acute respiratory syndrome coro    ....................  ....................  ....................  ....................  ....................  ....................
2719 MW332828.1 |Severe acute respiratory syndrome coro    ....................  ....................  ....................  ....................  ....................  ....................
2721 MW332832.1 |Severe acute respiratory syndrome coro    ....................  ....................  ....................  ....................  ....................  ....................
2722 MW332833.1 |Severe acute respiratory syndrome coro    ....................  ....................  ....................  ....................  ....................  ....................
2723 MW332842.1 |Severe acute respiratory syndrome coro    ....................  ....................  ....................  ....................  ....................  ....................
2724 MW332857.1 |Severe acute respiratory syndrome coro    ....................  ....................  ....................  ....................  ....................  ....................
2725 MW332858.1 |Severe acute respiratory syndrome coro    ....................  .A..................  ....................  ....................  ....................  ....................
2726 MW332859.1 |Severe acute respiratory syndrome coro    ....................  .............A......  ....................  ....................  ....................  ....................
2727 MW332860.1 |Severe acute respiratory syndrome coro    ....................  ....................  ....................  ....................  ....................  ....................
```

2728 MW332861.1 |Severe acute respiratory syndrome coro ···················· ···················· ····················· ···················· ···················· ····················
2729 MW332862.1 |Severe acute respiratory syndrome coro ···················· ·············A······ ····················· ···················· ···················· ····················
2730 MW332864.1 |Severe acute respiratory syndrome coro ···················· ···················· ····················· ···················· ···················· ····················
2731 MW332865.1 |Severe acute respiratory syndrome coro ···················· ···················· ····················· ···················· ···················· ····················
2732 MW332866.1 |Severe acute respiratory syndrome coro ···················· ···················· ····················· ···················· ···················· ····················
2733 MW332868.1 |Severe acute respiratory syndrome coro ···················· ···················· ····················· ···················· ···················· ····················
2734 MW332869.1 |Severe acute respiratory syndrome coro ···················· ···················· ····················· ···················· ···················· ····················
2735 MW332870.1 |Severe acute respiratory syndrome coro ···················· ···················· ····················· ···················· ···················· ····················
2736 MW332871.1 |Severe acute respiratory syndrome coro ···················· ···················· ····················· ···················· ···················· ····················
2737 MW332872.1 |Severe acute respiratory syndrome coro ···················· ···················· ····················· ···················· ···················· ····················
2738 MW332873.1 |Severe acute respiratory syndrome coro ···················· ···················· ····················· ···················· ···················· ····················
2739 MW332874.1 |Severe acute respiratory syndrome coro ···················· ···················· ····················· ···················· ···················· ····················
2740 MW332875.1 |Severe acute respiratory syndrome coro ···················· ·············A······ ····················· ···················· ···················· ····················
2741 MW332876.1 |Severe acute respiratory syndrome coro ···················· ···················· ····················· ···················· ···················· ····················
2742 MW332877.1 |Severe acute respiratory syndrome coro ···················· ···················· ····················· ···················· ···················· ····················
2743 MW332878.1 |Severe acute respiratory syndrome coro ···················· ···················· ····················· ···················· ···················· ····················
2744 MW332879.1 |Severe acute respiratory syndrome coro ···················· ···················· ····················· ···················· ···················· ····················
2745 MW332880.1 |Severe acute respiratory syndrome coro ···················· ···················· ····················· ···················· ···················· ····················
2746 MW332881.1 |Severe acute respiratory syndrome coro ···················· ···················· ····················· ···················· ···················· ····················
2747 MW332882.1 |Severe acute respiratory syndrome coro ···················· ···················· ····················· ···················· ···················· ····················
2748 MW332885.1 |Severe acute respiratory syndrome coro ···················· ···················· ····················· ···················· ···················· ····················
2749 MW332886.1 |Severe acute respiratory syndrome coro ···················· ·············A······ ····················· ···················· ···················· ····················
2750 MW332887.1 |Severe acute respiratory syndrome coro ···················· ···················· ····················· ···················· ···················· ····················
2751 MW332890.1 |Severe acute respiratory syndrome coro ···················· ···················· ····················· ···················· ···················· ····················
2752 MW332891.1 |Severe acute respiratory syndrome coro ···················· ···················· ····················· ···················· ···················· ····················
2753 MW332892.1 |Severe acute respiratory syndrome coro ···················· ···················· ····················· ···················· ···················· ····················
2754 MW332893.1 |Severe acute respiratory syndrome coro ···················· ···················· ····················· ···················· ···················· ····················
2755 MW332895.1 |Severe acute respiratory syndrome coro ···················· ···················· ····················· ···················· ···················· ····················
2756 MW332896.1 |Severe acute respiratory syndrome coro ···················· ···················· ····················· ···················· ···················· ····················
2757 MW332897.1 |Severe acute respiratory syndrome coro ···················· ···················· ····················· ···················· ···················· ····················
2758 MW332898.1 |Severe acute respiratory syndrome coro ···················· ···················· ····················· ···················· ···················· ····················
2759 MW332899.1 |Severe acute respiratory syndrome coro ···················· ···················· ····················· ···················· ···················· ····················
2760 MW332920.1 |Severe acute respiratory syndrome coro ···················· ···················· ····················· ···················· ···················· ····················
2761 MW332921.1 |Severe acute respiratory syndrome coro ···················· ···················· ····················· ···················· ···················· ····················
2762 MW332925.1 |Severe acute respiratory syndrome coro ···················· ···················· ····················· ···················· ···················· ····················
2763 MW332934.1 |Severe acute respiratory syndrome coro ···················· ···················· ····················· ···················· ···················· ····················
2764 MW332943.1 |Severe acute respiratory syndrome coro ···················· ···················· ····················· ···················· ···················· ····················
2765 MW332945.1 |Severe acute respiratory syndrome coro ···················· ···················· ····················· ···················· ···················· ····················
2766 MW332946.1 |Severe acute respiratory syndrome coro ···················· ···················· ····················· ···················· ···················· ····················
2767 MW332948.1 |Severe acute respiratory syndrome coro ···················· ···················· ····················· ···················· ···················· ····················
2768 MW332949.1 |Severe acute respiratory syndrome coro ···················· ···················· ····················· ···················· ···················· ····················
2769 MW332951.1 |Severe acute respiratory syndrome coro ···················· ···················· ····················· ···················· ···················· ····················
2770 MW332952.1 |Severe acute respiratory syndrome coro ···················· ···················· ····················· ···················· ···················· ····················
2771 MW332953.1 |Severe acute respiratory syndrome coro ···················· ···················· ····················· ···················· ···················· ····················
2772 MW332956.1 |Severe acute respiratory syndrome coro ···················· ···················· ····················· ···················· ···················· ····················
2773 MW332957.1 |Severe acute respiratory syndrome coro ···················· ···················· ····················· ···················· ···················· ····················
2774 MW332958.1 |Severe acute respiratory syndrome coro ···················· ···················· ····················· ···················· ···················· ····················
2775 MW332959.1 |Severe acute respiratory syndrome coro ···················· ···················· ····················· ···················· ···················· ····················
2776 MW332960.1 |Severe acute respiratory syndrome coro ···················· ···················· ····················· ···················· ···················· ····················
2777 MW332961.1 |Severe acute respiratory syndrome coro ···················· ···················· ····················· ···················· ···················· ····················
2778 MW332962.1 |Severe acute respiratory syndrome coro ···················· ···················· ····················· ···················· ···················· ····················
2779 MW332963.1 |Severe acute respiratory syndrome coro ···················· ···················· ····················· ···················· ···················· ····················
2780 MW332965.1 |Severe acute respiratory syndrome coro ···················· ···················· ····················· ···················· ···················· ····················
2781 MW332970.1 |Severe acute respiratory syndrome coro ···················· ·············A······ ····················· ···················· ···················· ····················
2782 MW332975.1 |Severe acute respiratory syndrome coro ···················· ···················· ····················· ···················· ···················· ····················
2783 MW332979.1 |Severe acute respiratory syndrome coro ···················· ···················· ····················· ···················· ···················· ····················
2784 MW332980.1 |Severe acute respiratory syndrome coro ···················· ···················· ····················· ···················· ···················· ····················
2785 MW332989.1 |Severe acute respiratory syndrome coro ···················· ···················· ····················· ···················· ···················· ····················
2786 MW332990.1 |Severe acute respiratory syndrome coro ···················· ···················· ····················· ···················· ···················· ····················
2787 MW332992.1 |Severe acute respiratory syndrome coro ···················· ···················· ····················· ···················· ···················· ····················
2788 MW332993.1 |Severe acute respiratory syndrome coro ···················· ···················· ····················· ···················· ···················· ····················
2789 MW332995.1 |Severe acute respiratory syndrome coro ···················· ···················· ····················· ···················· ···················· ····················
2790 MW332999.1 |Severe acute respiratory syndrome coro ···················· ···················· ····················· ···················· ···················· ····················
2791 MW333000.1 |Severe acute respiratory syndrome coro ···················· ···················· ····················· ···················· ···················· ····················
2792 MW333001.1 |Severe acute respiratory syndrome coro ···················· ···················· ····················· ···················· ···················· ····················
2793 MW333002.1 |Severe acute respiratory syndrome coro ···················· ···················· ····················· ···················· ···················· ····················
2794 MW333003.1 |Severe acute respiratory syndrome coro ···················· ···················· ····················· ···················· ···················· ····················
2795 MW333004.1 |Severe acute respiratory syndrome coro ···················· ···················· ····················· ···················· ···················· ····················
2796 MW333005.1 |Severe acute respiratory syndrome coro ···················· ···················· ····················· ···················· ···················· ····················
2797 MW333007.1 |Severe acute respiratory syndrome coro ···················· ···················· ····················· ···················· ···················· ····················
2798 MW333008.1 |Severe acute respiratory syndrome coro ···················· ···················· ····················· ···················· ···················· ····················
2799 MW333009.1 |Severe acute respiratory syndrome coro ···················· ···················· ····················· ···················· ···················· ····················
2800 MW333010.1 |Severe acute respiratory syndrome coro ···················· ···················· ····················· ···················· ···················· ····················
2801 MW333012.1 |Severe acute respiratory syndrome coro ···················· ···················· ····················· ···················· ···················· ····················
2802 MW333013.1 |Severe acute respiratory syndrome coro ···················· ···················· ····················· ···················· ···················· ····················
2803 MW333014.1 |Severe acute respiratory syndrome coro ···················· ···················· ····················· ···················· ···················· ····················
2804 MW333015.1 |Severe acute respiratory syndrome coro ···················· ···················· ····················· ···················· ···················· ····················
2805 MW333016.1 |Severe acute respiratory syndrome coro ···················· ···················· ····················· ···················· ···················· ····················

2806 MW333017.1 |Severe acute respiratory syndrome coro ....
2807 MW333018.1 |Severe acute respiratory syndrome coro ....
2808 MW333021.1 |Severe acute respiratory syndrome coro ....
2809 MW333022.1 |Severe acute respiratory syndrome coro ....
2810 MW333023.1 |Severe acute respiratory syndrome coro ....
2811 MW338760.1 |Severe acute respiratory syndrome coro ....
2812 MW338761.1 |Severe acute respiratory syndrome coro ....
2813 MW338762.1 |Severe acute respiratory syndrome coro ....
2814 MW338763.1 |Severe acute respiratory syndrome coro ....
2815 MW338764.1 |Severe acute respiratory syndrome coro ....
2816 MW338765.1 |Severe acute respiratory syndrome coro ....
2817 MW338766.1 |Severe acute respiratory syndrome coro ....
2818 MW338767.1 |Severe acute respiratory syndrome coro ....
2819 MW338768.1 |Severe acute respiratory syndrome coro ....
2820 MW338769.1 |Severe acute respiratory syndrome coro ....
2821 MW338770.1 |Severe acute respiratory syndrome coro ....
2822 MW338771.1 |Severe acute respiratory syndrome coro ....
2823 MW338772.1 |Severe acute respiratory syndrome coro ....
2824 MW338773.1 |Severe acute respiratory syndrome coro ....
2825 MW338774.1 |Severe acute respiratory syndrome coro ....
2826 MW338775.1 |Severe acute respiratory syndrome coro ....
2827 MW338776.1 |Severe acute respiratory syndrome coro ....
2828 MW338777.1 |Severe acute respiratory syndrome coro ....
2829 MW338778.1 |Severe acute respiratory syndrome coro ....
2830 MW338779.1 |Severe acute respiratory syndrome coro ....
2831 MW338780.1 |Severe acute respiratory syndrome coro ....
2832 MW338781.1 |Severe acute respiratory syndrome coro ....
2833 MW338782.1 |Severe acute respiratory syndrome coro ....
2834 MW338783.1 |Severe acute respiratory syndrome coro .... .A.................. ....
2835 MW338784.1 |Severe acute respiratory syndrome coro ....
2836 MW338785.1 |Severe acute respiratory syndrome coro ....
2837 MW338786.1 |Severe acute respiratory syndrome coro ....
2838 MW338787.1 |Severe acute respiratory syndrome coro ....
2839 MW338788.1 |Severe acute respiratory syndrome coro ....
2840 MW338789.1 |Severe acute respiratory syndrome coro ....
2841 MW338790.1 |Severe acute respiratory syndrome coro ....
2842 MW338791.1 |Severe acute respiratory syndrome coro ....
2843 MW338792.1 |Severe acute respiratory syndrome coro ....
2844 MW338793.1 |Severe acute respiratory syndrome coro ....
2845 MW338794.1 |Severe acute respiratory syndrome coro ....
2846 MW338795.1 |Severe acute respiratory syndrome coro ....
2847 MW338796.1 |Severe acute respiratory syndrome coro ....
2848 MW338797.1 |Severe acute respiratory syndrome coro ....
2849 MW338798.1 |Severe acute respiratory syndrome coro ....
2850 MW338799.1 |Severe acute respiratory syndrome coro ....
2851 MW338800.1 |Severe acute respiratory syndrome coro ....
2852 MW338801.1 |Severe acute respiratory syndrome coro ....
2853 MW338802.1 |Severe acute respiratory syndrome coro ....
2854 MW338803.1 |Severe acute respiratory syndrome coro ....
2855 MW338804.1 |Severe acute respiratory syndrome coro ....
2856 MW338805.1 |Severe acute respiratory syndrome coro ....
2857 MW338806.1 |Severe acute respiratory syndrome coro ....
2858 MW338807.1 |Severe acute respiratory syndrome coro .... .A.................. ....
2859 MW338808.1 |Severe acute respiratory syndrome coro ....
2860 MW338809.1 |Severe acute respiratory syndrome coro .... .A.................. ....
2861 MW340733.1 |Severe acute respiratory syndrome coro .... .A.................. ....
2862 MW340734.1 |Severe acute respiratory syndrome coro ....
2863 MW340735.1 |Severe acute respiratory syndrome coro .... .A.................. ....
2864 MW340736.1 |Severe acute respiratory syndrome coro ....
2865 MW340737.1 |Severe acute respiratory syndrome coro ....
2866 MW340738.1 |Severe acute respiratory syndrome coro ....
2867 MW340739.1 |Severe acute respiratory syndrome coro ....
2868 MW340740.1 |Severe acute respiratory syndrome coro ....
2869 MW340741.1 |Severe acute respiratory syndrome coro ....
2870 MW340742.1 |Severe acute respiratory syndrome coro ....
2871 MW340743.1 |Severe acute respiratory syndrome coro .... .A.................. ....
2872 MW340744.1 |Severe acute respiratory syndrome coro ....
2873 MW340745.1 |Severe acute respiratory syndrome coro ....
2874 MW340746.1 |Severe acute respiratory syndrome coro ....
2875 MW340747.1 |Severe acute respiratory syndrome coro ....A............... ....
2876 MW340748.1 |Severe acute respiratory syndrome coro ....
2877 MW340749.1 |Severe acute respiratory syndrome coro ....
2878 MW340750.1 |Severe acute respiratory syndrome coro ....
2879 MW340751.1 |Severe acute respiratory syndrome coro ....
2880 MW340752.1 |Severe acute respiratory syndrome coro ....
2881 MW340753.1 |Severe acute respiratory syndrome coro ....
2882 MW340754.1 |Severe acute respiratory syndrome coro ....
2883 MW340755.1 |Severe acute respiratory syndrome coro ....

2884	MW340756.1	|Severe acute respiratory syndrome coro	....	....	....	....	....	....
2885	MW340757.1	|Severe acute respiratory syndrome coro	....	....	....	....	....	....
2886	MW340758.1	|Severe acute respiratory syndrome coro	....	....	....	....	....	....
2887	MW340759.1	|Severe acute respiratory syndrome coro	....	....	....	....	....	....
2888	MW340760.1	|Severe acute respiratory syndrome coro	....	....	....	....	....	....
2889	MW340761.1	|Severe acute respiratory syndrome coro	....	....	....	....	....	....
2890	MW340763.1	|Severe acute respiratory syndrome coro	....	....	....	....	....	....
2891	MW340764.1	|Severe acute respiratory syndrome coro	....	....	....	....	....	....
2892	MW340765.1	|Severe acute respiratory syndrome coro	....	....	....	....	....	....
2893	MW340767.1	|Severe acute respiratory syndrome coro	....	.A..................	....	....	....	....
2894	MW340768.1	|Severe acute respiratory syndrome coro	....	.A..................	....	....	....	....
2895	MW340769.1	|Severe acute respiratory syndrome coro	....	....C...............	....	....	....	....
2896	MW340770.1	|Severe acute respiratory syndrome coro	....	....	....	....	....	....
2897	MW340771.1	|Severe acute respiratory syndrome coro	....	....	....	....	....	....
2898	MW340772.1	|Severe acute respiratory syndrome coro	....	....	....	....	....	....
2899	MW340773.1	|Severe acute respiratory syndrome coro	....	....	....	....	....	....
2900	MW340775.1	|Severe acute respiratory syndrome coro	....	....	....	....	....	....
2901	MW340776.1	|Severe acute respiratory syndrome coro	....	....	....	....	....	....
2902	MW340777.1	|Severe acute respiratory syndrome coro	....	....	....	....	....	....
2903	MW340778.1	|Severe acute respiratory syndrome coro	....	....	....	....	....	....
2904	MW340779.1	|Severe acute respiratory syndrome coro	....	....	....	....	....	....
2905	MW340780.1	|Severe acute respiratory syndrome coro	....	.A..................	....	....	....	....
2906	MW340781.1	|Severe acute respiratory syndrome coro	....	....	....	....	....	....
2907	MW340782.1	|Severe acute respiratory syndrome coro	....	.A..................	....	....	....	....
2908	MW340783.1	|Severe acute respiratory syndrome coro	....	.A..................	....	....	....	....
2909	MW340784.1	|Severe acute respiratory syndrome coro	....	....	....	....	....	....
2910	MW340785.1	|Severe acute respiratory syndrome coro	....	....	....	....	....	....
2911	MW340786.1	|Severe acute respiratory syndrome coro	....	....	....	....	....	....
2912	MW340787.1	|Severe acute respiratory syndrome coro	....	....	....	....	....	....
2913	MW340788.1	|Severe acute respiratory syndrome coro	....	....	....	....	....	....
2914	MW340789.1	|Severe acute respiratory syndrome coro	....	....	....	....	....	....
2915	MW340790.1	|Severe acute respiratory syndrome coro	....	....	....	....	....	....
2916	MW340791.1	|Severe acute respiratory syndrome coro	....	....	....	....	....	....
2917	MW340792.1	|Severe acute respiratory syndrome coro	....	....	....	....	....	....
2918	MW340793.1	|Severe acute respiratory syndrome coro	....	....	....	....	....	....
2919	MW340794.1	|Severe acute respiratory syndrome coro	....	....	....	....	....	....
2920	MW340795.1	|Severe acute respiratory syndrome coro	....	....	....	....	....	....
2921	MW340796.1	|Severe acute respiratory syndrome coro	....	....	....	....	....	....
2922	MW340797.1	|Severe acute respiratory syndrome coro	....	....	....	....	....	....
2923	MW340798.1	|Severe acute respiratory syndrome coro	....	....	....	....	....	....
2924	MW340799.1	|Severe acute respiratory syndrome coro	....	....	....	....	....	....
2925	MW341214.1	|Severe acute respiratory syndrome coro	....	....	....	....	....	....
2926	MW332213.1	|Severe acute respiratory syndrome coro	....	.A..................	....	....	....	..A.................
2927	MW332214.1	|Severe acute respiratory syndrome coro	....	....	....	....	....	....
2928	MW332215.1	|Severe acute respiratory syndrome coro	....	....	....	....	....	....
2929	MW332216.1	|Severe acute respiratory syndrome coro	....	....	....	....	....	....
2930	MW332217.1	|Severe acute respiratory syndrome coro	....	....	....	....	....	....
2931	MW332218.1	|Severe acute respiratory syndrome coro	....	....	....	....	....	....
2932	MW332219.1	|Severe acute respiratory syndrome coro	....	....	....	....	....	....
2933	MW332220.1	|Severe acute respiratory syndrome coro	....	....	....	....	....	....
2934	MW332221.1	|Severe acute respiratory syndrome coro	....	....	....	....	....	....
2935	MW332222.1	|Severe acute respiratory syndrome coro	....	...A................	....	....	....	..A.................
2936	MW332223.1	|Severe acute respiratory syndrome coro	....	....	....	....	....	....
2937	MW332224.1	|Severe acute respiratory syndrome coro	....	....	....	....	....	....
2938	MW332225.1	|Severe acute respiratory syndrome coro	....	....	....	....	....	....
2939	MW332226.1	|Severe acute respiratory syndrome coro	....	....	....	....	....	....
2940	MW332227.1	|Severe acute respiratory syndrome coro	....	....	....	....	....	....
2941	MW332228.1	|Severe acute respiratory syndrome coro	....	....	....	....	....	....
2942	MW332229.1	|Severe acute respiratory syndrome coro	....	....	....	....	....	....
2943	MW332230.1	|Severe acute respiratory syndrome coro	....	....	....	....	....	....
2944	MW332231.1	|Severe acute respiratory syndrome coro	....	....	....	....	....	....
2945	MW332232.1	|Severe acute respiratory syndrome coro	....	....	....	....	....	....
2946	MW332233.1	|Severe acute respiratory syndrome coro	....	....	....	....	....	....
2947	MW332235.1	|Severe acute respiratory syndrome coro	....	....	....	....	....	....
2948	MW332236.1	|Severe acute respiratory syndrome coro	....	....	....	....	....	....
2949	MW332237.1	|Severe acute respiratory syndrome coro	....	....	....	....	....	....
2950	MW332238.1	|Severe acute respiratory syndrome coro	....	....	....	....	....	....
2951	MW332239.1	|Severe acute respiratory syndrome coro	....	....	....	....	....	....
2952	MW332240.1	|Severe acute respiratory syndrome coro	....	....	....	....	....	....
2953	MW332241.1	|Severe acute respiratory syndrome coro	....	....	....	....	....	....
2954	MW327510.1	|Severe acute respiratory syndrome coro	....	....	....	....	....	....
2955	MW327511.1	|Severe acute respiratory syndrome coro	....	....	....	....	....	....
2956	MW327512.1	|Severe acute respiratory syndrome coro	....	....	....	....	....	....
2957	MW327513.1	|Severe acute respiratory syndrome coro	....	....	....	....	....	....
2958	MW327514.1	|Severe acute respiratory syndrome coro	....	....	....	....	....	....
2959	MW327515.1	|Severe acute respiratory syndrome coro	....	....	....	....	....	....
2960	MW327516.1	|Severe acute respiratory syndrome coro	....	....	....	....	....	....
2961	MW327517.1	|Severe acute respiratory syndrome coro	....	....	....	....	....	....

2962	MW327518.1	|Severe acute respiratory syndrome coro	····················	····················	····················	····················	····················	····················	
2963	MW327519.1	|Severe acute respiratory syndrome coro	····················	····················	····················	····················	····················	····················	
2964	MW327520.1	|Severe acute respiratory syndrome coro	····················	····················	····················	····················	····················	····················	
2965	MW327521.1	|Severe acute respiratory syndrome coro	····················	····················	····················	····················	····················	····················	
2966	MW327522.1	|Severe acute respiratory syndrome coro	····················	····················	····················	····················	····················	····················	
2967	MW327523.1	|Severe acute respiratory syndrome coro	····················	····················	····················	····················	····················	····················	
2968	MW327524.1	|Severe acute respiratory syndrome coro	····················	····················	····················	····················	····················	····················	
2969	MW327525.1	|Severe acute respiratory syndrome coro	····················	····················	····················	····················	····················	····················	
2970	MW327526.1	|Severe acute respiratory syndrome coro	····················	····················	····················	····················	····················	····················	
2971	MW327527.1	|Severe acute respiratory syndrome coro	····················	····················	····················	····················	····················	····················	
2972	MW327528.1	|Severe acute respiratory syndrome coro	····················	····················	····················	····················	····················	····················	
2973	MW327529.1	|Severe acute respiratory syndrome coro	····················	····················	····················	····················	····················	····················	
2974	MW327530.1	|Severe acute respiratory syndrome coro	····················	····················	····················	····················	····················	····················	
2975	MW327531.1	|Severe acute respiratory syndrome coro	····················	····················	····················	····················	····················	····················	
2976	MW327532.1	|Severe acute respiratory syndrome coro	····················	····················	····················	····················	····················	····················	
2977	MW327533.1	|Severe acute respiratory syndrome coro	····················	····················	····················	····················	····················	····················	
2978	MW327534.1	|Severe acute respiratory syndrome coro	····················	····················	····················	····················	····················	····················	
2979	MW327535.1	|Severe acute respiratory syndrome coro	····················	····················	····················	····················	····················	····················	
2980	MW327536.1	|Severe acute respiratory syndrome coro	····················	····················	····················	····················	····················	····················	
2981	MW327537.1	|Severe acute respiratory syndrome coro	····················	····················	····················	····················	····················	····················	
2982	MW327538.1	|Severe acute respiratory syndrome coro	····················	····················	····················	····················	····················	····················	
2983	MW327539.1	|Severe acute respiratory syndrome coro	····················	····················	····················	····················	····················	····················	
2985	MW320730.1	|Severe acute respiratory syndrome coro	····················	····················	····················	····················	····················	····················	
2986	MW320731.1	|Severe acute respiratory syndrome coro	····················	····················	····················	····················	····················	····················	
2987	MW320733.1	|Severe acute respiratory syndrome coro	····················	····················	····················	····················	····················	····················	
2988	MW320735.1	|Severe acute respiratory syndrome coro	····················	····················	····················	····················	····················	····················	
2989	MW320740.1	|Severe acute respiratory syndrome coro	····················	····················	····················	····················	····················	····················	
2990	MW320741.1	|Severe acute respiratory syndrome coro	····················	····················	····················	····················	····················	····················	
2991	MW320746.1	|Severe acute respiratory syndrome coro	····················	····················	····················	····················	····················	····················	
2993	MW320749.1	|Severe acute respiratory syndrome coro	····················	····················	····················	····················	····················	····················	
2995	MW320752.1	|Severe acute respiratory syndrome coro	····················	····················	····················	····················	····················	····················	
2996	MW320754.1	|Severe acute respiratory syndrome coro	····················	····················	····················	····················	····················	····················	
2997	MW320755.1	|Severe acute respiratory syndrome coro	····················	····················	····················	····················	····················	····················	
2998	MW320757.1	|Severe acute respiratory syndrome coro	····················	····················	····················	····················	····················	····················	
2999	MW320758.1	|Severe acute respiratory syndrome coro	····················	····················	····················	····················	····················	····················	
3000	MW320759.1	|Severe acute respiratory syndrome coro	····················	····················	····················	····················	····················	····················	
3001	MW320760.1	|Severe acute respiratory syndrome coro	····················	····················	····················	····················	····················	····················	
3002	MW320761.1	|Severe acute respiratory syndrome coro	····················	····················	····················	····················	····················	····················	
3003	MW320762.1	|Severe acute respiratory syndrome coro	····················	····················	····················	····················	····················	····················	
3004	MW320763.1	|Severe acute respiratory syndrome coro	····················	····················	····················	····················	····················	····················	
3005	MW320765.1	|Severe acute respiratory syndrome coro	····················	····················	····················	····················	····················	····················	
3006	MW320766.1	|Severe acute respiratory syndrome coro	····················	····················	····················	····················	····················	····················	
3007	MW320767.1	|Severe acute respiratory syndrome coro	····················	····················	····················	····················	····················	····················	
3008	MW320770.1	|Severe acute respiratory syndrome coro	····················	····················	····················	····················	NNN·················	····················	1
3010	MW320772.1	|Severe acute respiratory syndrome coro	····················	······A·············	····················	····················	····················	····················	
3011	MW320775.1	|Severe acute respiratory syndrome coro	····················	····················	····················	····················	····················	····················	
3012	MW320779.1	|Severe acute respiratory syndrome coro	····················	····················	····················	····················	····················	····················	
3013	MW320782.1	|Severe acute respiratory syndrome coro	····················	····················	····················	····················	····················	····················	
3014	MW320784.1	|Severe acute respiratory syndrome coro	····················	····················	····················	····················	····················	····················	
3015	MW320786.1	|Severe acute respiratory syndrome coro	····················	····················	····················	····················	····················	····················	
3016	MW320787.1	|Severe acute respiratory syndrome coro	····················	····················	····················	····················	····················	····················	
3017	MW320790.1	|Severe acute respiratory syndrome coro	····················	····················	····················	····················	····················	····················	
3018	MW320792.1	|Severe acute respiratory syndrome coro	····················	····················	····················	····················	····················	····················	
3019	MW320793.1	|Severe acute respiratory syndrome coro	····················	····················	····················	····················	····················	····················	
3020	MW320794.1	|Severe acute respiratory syndrome coro	····················	····················	····················	····················	····················	····················	
3021	MW320795.1	|Severe acute respiratory syndrome coro	····················	····················	····················	····················	····················	····················	
3022	MW320797.1	|Severe acute respiratory syndrome coro	····················	····················	····················	····················	····················	····················	
3023	MW320798.1	|Severe acute respiratory syndrome coro	····················	····················	····················	····················	····················	····················	
3024	MW320799.1	|Severe acute respiratory syndrome coro	····················	····················	····················	····················	····················	····················	
3025	MW320800.1	|Severe acute respiratory syndrome coro	····················	····················	····················	····················	····················	····················	
3026	MW320802.1	|Severe acute respiratory syndrome coro	····················	····················	····················	····················	·····Y··············	····················	
3027	MW320805.1	|Severe acute respiratory syndrome coro	····················	····················	····················	····················	····················	····················	
3028	MW320806.1	|Severe acute respiratory syndrome coro	····················	····················	····················	····················	····················	····················	
3029	MW320808.1	|Severe acute respiratory syndrome coro	····················	····················	····················	····················	····················	····················	
3030	MW320815.1	|Severe acute respiratory syndrome coro	····················	····················	····················	····················	····················	····················	
3031	MW320818.1	|Severe acute respiratory syndrome coro	····················	····················	····················	····················	····················	····················	
3032	MW320819.1	|Severe acute respiratory syndrome coro	····················	····················	····················	····················	····················	····················	
3033	MW320820.1	|Severe acute respiratory syndrome coro	····················	····················	····················	····················	····················	····················	
3034	MW320822.1	|Severe acute respiratory syndrome coro	····················	····················	····················	····················	····················	····················	
3035	MW320823.1	|Severe acute respiratory syndrome coro	····················	····················	····················	····················	····················	····················	
3036	MW320824.1	|Severe acute respiratory syndrome coro	····················	····················	····················	····················	····················	····················	
3037	MW320825.1	|Severe acute respiratory syndrome coro	····················	····················	····················	····················	····················	····················	
3038	MW320829.1	|Severe acute respiratory syndrome coro	····················	····················	····················	····················	····················	····················	
3039	MW320830.1	|Severe acute respiratory syndrome coro	····················	····················	····················	····················	····················	····················	
3040	MW320831.1	|Severe acute respiratory syndrome coro	····················	····················	····················	····················	····················	····················	
3041	MW320834.1	|Severe acute respiratory syndrome coro	····················	····················	····················	····················	····················	····················	
3042	MW320835.1	|Severe acute respiratory syndrome coro	····················	····················	····················	····················	····················	····················	
3043	MW320838.1	|Severe acute respiratory syndrome coro	····················	····················	····················	····················	····················	····················	

3044	MW320840.1	|Severe acute respiratory syndrome coro	....	....	....	....	....	....
3045	MW320841.1	|Severe acute respiratory syndrome coro	....	....	....	....	....	....
3046	MW320843.1	|Severe acute respiratory syndrome coro	....	....	....	....	....	....
3047	MW320846.1	|Severe acute respiratory syndrome coro	....	....	....	....	....	....
3048	MW320849.1	|Severe acute respiratory syndrome coro	....	....	....	....	....	....
3049	MW320851.1	|Severe acute respiratory syndrome coro	....	....	....	....	....	....
3050	MW320852.1	|Severe acute respiratory syndrome coro	....	....	....	....	....	....
3051	MW320856.1	|Severe acute respiratory syndrome coro	....	....	....	....	....	....
3052	MW320859.1	|Severe acute respiratory syndrome coro	....	....	....	....	....	....
3053	MW320861.1	|Severe acute respiratory syndrome coro	....	....	....	....	....	....
3055	MW320865.1	|Severe acute respiratory syndrome coro	....	....	....	....	....	....
3056	MW320867.1	|Severe acute respiratory syndrome coro	....	....	....	....	....	....
3057	MW320868.1	|Severe acute respiratory syndrome coro	....	....	....	....	....	....
3058	MW320869.1	|Severe acute respiratory syndrome coro	....	....	....	....	....	....
3059	MW320870.1	|Severe acute respiratory syndrome coro	....	....	....	....	....	....
3060	MW320873.1	|Severe acute respiratory syndrome coro	....	....	....	....	....	....
3061	MW320874.1	|Severe acute respiratory syndrome coro	....	....	....	....	....	....
3062	MW320876.1	|Severe acute respiratory syndrome coro	....	....	....	....	....	....
3063	MW320877.1	|Severe acute respiratory syndrome coro	....	....	....	....	....	....
3064	MW320879.1	|Severe acute respiratory syndrome coro	....	....	....	....	....	....
3065	MW320880.1	|Severe acute respiratory syndrome coro	....	....	....	....	....	....
3067	MW320882.1	|Severe acute respiratory syndrome coro	....	....	....	....	....	....
3068	MW320883.1	|Severe acute respiratory syndrome coro	....	....	....	....	....	....
3070	MW320889.1	|Severe acute respiratory syndrome coro	....	....	....	....	....	....
3071	MW320890.1	|Severe acute respiratory syndrome coro	....	....	....	....	....	....
3073	MW320892.1	|Severe acute respiratory syndrome coro	....	....	....	....	....	....
3074	MW320893.1	|Severe acute respiratory syndrome coro	....	....	....	....	....	....
3075	MW320894.1	|Severe acute respiratory syndrome coro	....	....	....	....	....	....
3076	MW320895.1	|Severe acute respiratory syndrome coro	....	....	....	....	....	....
3077	MW320897.1	|Severe acute respiratory syndrome coro	....	....	....	....	....	....
3078	MW320898.1	|Severe acute respiratory syndrome coro	....	....	....	....	....	....
3079	MW320901.1	|Severe acute respiratory syndrome coro	....	....	....	....	....	....
3080	MW320904.1	|Severe acute respiratory syndrome coro	....	....	....	....	....	....
3081	MW320906.1	|Severe acute respiratory syndrome coro	....	....	....	....	....	....
3083	MW320910.1	|Severe acute respiratory syndrome coro	....	....	....	....	....	....
3084	MW320912.1	|Severe acute respiratory syndrome coro	....	....	....	....	....	....
3086	MW320915.1	|Severe acute respiratory syndrome coro	....	....	....	....	....	....
3087	MW320916.1	|Severe acute respiratory syndrome coro	....	....	....	....	....	....
3088	MW320917.1	|Severe acute respiratory syndrome coro	....	....	....	....	....	....
3089	MW320919.1	|Severe acute respiratory syndrome coro	....	....	....	....	....	....
3091	MW320928.1	|Severe acute respiratory syndrome coro	....	....	....	....	....	....
3092	MW320929.1	|Severe acute respiratory syndrome coro	....	....	....	....	....	....
3095	MW320932.1	|Severe acute respiratory syndrome coro	....	....	....	....	....	....
3096	MW320934.1	|Severe acute respiratory syndrome coro	....	....	....	....	....	....
3098	MW320937.1	|Severe acute respiratory syndrome coro	....	....	....	....	....	....
3099	MW320941.1	|Severe acute respiratory syndrome coro	....	....	....	....	....	....
3100	MW320942.1	|Severe acute respiratory syndrome coro	....	....	....	....	....	....
3101	MW320943.1	|Severe acute respiratory syndrome coro	....	....	....	....	....	....
3102	MW320944.1	|Severe acute respiratory syndrome coro	....	....	....	....	....	....
3103	MW320947.1	|Severe acute respiratory syndrome coro	....	....	....	....	....	....
3104	MW320948.1	|Severe acute respiratory syndrome coro	....	....	....	....	....	....
3105	MW320949.1	|Severe acute respiratory syndrome coro	....	....	....	....	....	....
3106	MW320951.1	|Severe acute respiratory syndrome coro	....	....	....	....	....	....
3107	MW320952.1	|Severe acute respiratory syndrome coro	....	....	....	....	....	....
3109	MW320957.1	|Severe acute respiratory syndrome coro	....	....	....	....	....	....
3110	MW320958.1	|Severe acute respiratory syndrome coro	....	....	....	....	....	....
3112	MW320966.1	|Severe acute respiratory syndrome coro	....	....	....	....	....	....
3114	MW320968.1	|Severe acute respiratory syndrome coro	....	....	....	....	....	....
3115	MW320969.1	|Severe acute respiratory syndrome coro	....	....	....	....	....	....
3116	MW320970.1	|Severe acute respiratory syndrome coro	....	....	....	....	....	....
3117	MW320971.1	|Severe acute respiratory syndrome coro	....	....	....	....	....	....
3118	MW320973.1	|Severe acute respiratory syndrome coro	....	....	....	....	....	....
3119	MW320975.1	|Severe acute respiratory syndrome coro	....	....	....	....	....	....
3120	MW320976.1	|Severe acute respiratory syndrome coro	....	....	....	....	....	....
3122	MW320978.1	|Severe acute respiratory syndrome coro	....	....	....	....	....	....
3123	MW320980.1	|Severe acute respiratory syndrome coro	....	....	....	....	....	....
3124	MW320983.1	|Severe acute respiratory syndrome coro	....	....	....	....	....	....
3125	MW320984.1	|Severe acute respiratory syndrome coro	....	....	....	....	....	....
3126	MW320985.1	|Severe acute respiratory syndrome coro	....	....	....	....	....	....
3127	MW320986.1	|Severe acute respiratory syndrome coro	....	....	....	....	....	....
3128	MW320987.1	|Severe acute respiratory syndrome coro	....	....	....	....	....	....
3129	MW320989.1	|Severe acute respiratory syndrome coro	....	....	....	....	....	....
3130	MW320990.1	|Severe acute respiratory syndrome coro	....	....	....	....	....	....
3131	MW320992.1	|Severe acute respiratory syndrome coro	....	....	....	....	....	....
3132	MW320993.1	|Severe acute respiratory syndrome coro	....	....	....	....	....	....
3134	MW320995.1	|Severe acute respiratory syndrome coro	....	....	....	....	....	....
3135	MW320998.1	|Severe acute respiratory syndrome coro	....	....	....	....	....	....
3136	MW320999.1	|Severe acute respiratory syndrome coro	....	....	....	....	....	....

3138	MW321006.1	Severe acute respiratory syndrome coro	····················	····················	····················	····················	····················	····················	
3139	MW321008.1	Severe acute respiratory syndrome coro	····················	····················	····················	····················	····················	····················	
3140	MW321009.1	Severe acute respiratory syndrome coro	····················	····················	····················	····················	····················	····················	
3141	MW321010.1	Severe acute respiratory syndrome coro	····················	····················	····················	····················	····················	····················	
3142	MW321011.1	Severe acute respiratory syndrome coro	····················	····················	····················	····················	····················	····················	
3143	MW321014.1	Severe acute respiratory syndrome coro	····················	····················	····················	····················	····················	····················	
3144	MW321019.1	Severe acute respiratory syndrome coro	····················	····················	····················	····················	····················	····················	
3145	MW321022.1	Severe acute respiratory syndrome coro	····················	····················	····················	····················	····················	····················	
3146	MW321024.1	Severe acute respiratory syndrome coro	····················	····················	····················	····················	····················	····················	
3147	MW321025.1	Severe acute respiratory syndrome coro	····················	····················	····················	····················	····················	····················	
3148	MW321026.1	Severe acute respiratory syndrome coro	····················	····················	····················	····················	····················	····················	
3149	MW321030.1	Severe acute respiratory syndrome coro	····················	····················	····················	····················	····················	····················	
3150	MW321033.1	Severe acute respiratory syndrome coro	····················	····················	····················	····················	····················	····················	
3151	MW321036.1	Severe acute respiratory syndrome coro	····················	····················	····················	····················	····················	····················	
3154	MW321042.1	Severe acute respiratory syndrome coro	····················	····················	····················	····················	····················	····················	
3155	MW321043.1	Severe acute respiratory syndrome coro	····················	····················	····················	····················	····················	····················	
3156	MW321044.1	Severe acute respiratory syndrome coro	····················	····················	····················	····················	····················	····················	
3158	MW321046.1	Severe acute respiratory syndrome coro	····················	····················	····················	····················	····················	····················	
3159	MW321048.1	Severe acute respiratory syndrome coro	····················	····················	····················	····················	····················	····················	
3161	MW321052.1	Severe acute respiratory syndrome coro	····················	····················	····················	····················	····················	····················	
3162	MW321053.1	Severe acute respiratory syndrome coro	····················	····················	····················	····················	····················	····················	
3163	MW321054.1	Severe acute respiratory syndrome coro	····················	····················	····················	····················	····················	····················	
3164	MW321056.1	Severe acute respiratory syndrome coro	····················	····················	····················	····················	····················	····················	
3165	MW321057.1	Severe acute respiratory syndrome coro	····················	····················	····················	····················	····················	····················	
3166	MW321058.1	Severe acute respiratory syndrome coro	····················	····················	····················	····················	····················	····················	
3167	MW321059.1	Severe acute respiratory syndrome coro	····················	····················	····················	····················	····················	····················	
3168	MW321060.1	Severe acute respiratory syndrome coro	····················	····················	····················	····················	····················	····················	
3169	MW321062.1	Severe acute respiratory syndrome coro	····················	····················	····················	····················	····················	····················	
3170	MW321063.1	Severe acute respiratory syndrome coro	····················	····················	····················	····················	····················	····················	
3171	MW321064.1	Severe acute respiratory syndrome coro	····················	····················	····················	····················	····················	····················	
3173	MW321066.1	Severe acute respiratory syndrome coro	····················	····················	····················	····················	····················	····················	
3174	MW321067.1	Severe acute respiratory syndrome coro	····················	····················	····················	····················	····················	····················	
3175	MW321068.1	Severe acute respiratory syndrome coro	····················	····················	····················	····················	····················	····················	
3176	MW321070.1	Severe acute respiratory syndrome coro	····················	····················	····················	····················	····················	····················	
3177	MW321071.1	Severe acute respiratory syndrome coro	····················	····················	····················	····················	····················	····················	
3180	MW321080.1	Severe acute respiratory syndrome coro	····················	····················	····················	····················	····················	····················	
3181	MW321081.1	Severe acute respiratory syndrome coro	····················	····················	····················	····················	····················	····················	
3182	MW321082.1	Severe acute respiratory syndrome coro	····················	····················	····················	····················	····················	····················	
3183	MW321085.1	Severe acute respiratory syndrome coro	····················	····················	····················	····················	····················	····················	
3184	MW321086.1	Severe acute respiratory syndrome coro	····················	····················	····················	····················	····················	····················	
3186	MW321090.1	Severe acute respiratory syndrome coro	····················	····················	····················	····················	····················	····················	
3187	MW321091.1	Severe acute respiratory syndrome coro	····················	····················	····················	····················	····················	····················	
3189	MW321093.1	Severe acute respiratory syndrome coro	····················	····················	····················	····················	····················	····················	
3191	MW321098.1	Severe acute respiratory syndrome coro	····················	····················	····················	····················	····················	····················	
3192	MW321099.1	Severe acute respiratory syndrome coro	····················	····················	····················	····················	····················	····················	
3193	MW321101.1	Severe acute respiratory syndrome coro	····················	····················	····················	····················	····················	····················	
3194	MW321103.1	Severe acute respiratory syndrome coro	····················	····················	····················	····················	····················	····················	
3195	MW321104.1	Severe acute respiratory syndrome coro	····················	····················	····················	····················	····················	····················	
3196	MW321107.1	Severe acute respiratory syndrome coro	····················	····················	····················	····················	····················	····················	
3198	MW321111.1	Severe acute respiratory syndrome coro	····················	····················	····················	····················	····················	····················	
3199	MW321112.1	Severe acute respiratory syndrome coro	····················	····················	····················	····················	····················	····················	
3200	MW321113.1	Severe acute respiratory syndrome coro	····················	····················	····················	····················	····················	····················	
3201	MW321114.1	Severe acute respiratory syndrome coro	····················	····················	····················	····················	····················	····················	
3202	MW321115.1	Severe acute respiratory syndrome coro	····················	····················	····················	····················	····················	····················	
3203	MW321116.1	Severe acute respiratory syndrome coro	····················	····················	····················	····················	····················	····················	
3205	MW321119.1	Severe acute respiratory syndrome coro	····················	····················	····················	····················	····················	····················	
3206	MW321120.1	Severe acute respiratory syndrome coro	····················	····················	····················	····················	····················	····················	
3207	MW321121.1	Severe acute respiratory syndrome coro	····················	····················	····················	····················	····················	····················	
3208	MW321122.1	Severe acute respiratory syndrome coro	····················	····················	····················	····················	····················	····················	
3209	MW321123.1	Severe acute respiratory syndrome coro	····················	····················	····················	····················	····················	····················	
3210	MW321124.1	Severe acute respiratory syndrome coro	····················	····················	····················	····················	····················	····················	
3211	MW321125.1	Severe acute respiratory syndrome coro	····················	····················	····················	····················	····················	····················	
3212	MW321127.1	Severe acute respiratory syndrome coro	····················	····················	····················	····················	NNN·················	····················	1
3213	MW321128.1	Severe acute respiratory syndrome coro	····················	····················	····················	····················	····················	····················	
3214	MW321132.1	Severe acute respiratory syndrome coro	····················	····················	····················	····················	····················	····················	
3215	MW321134.1	Severe acute respiratory syndrome coro	····················	····················	····················	····················	····················	····················	
3216	MW321136.1	Severe acute respiratory syndrome coro	····················	····················	····················	····················	····················	····················	
3217	MW321137.1	Severe acute respiratory syndrome coro	····················	····················	····················	····················	····················	····················	
3218	MW321139.1	Severe acute respiratory syndrome coro	····················	····················	····················	····················	····················	····················	
3219	MW321143.1	Severe acute respiratory syndrome coro	····················	····················	····················	····················	····················	····················	
3220	MW321148.1	Severe acute respiratory syndrome coro	····················	····················	····················	····················	····················	····················	
3222	MW321150.1	Severe acute respiratory syndrome coro	····················	····················	····················	····················	····················	····················	
3223	MW321155.1	Severe acute respiratory syndrome coro	····················	····················	····················	····················	····················	····················	
3224	MW321157.1	Severe acute respiratory syndrome coro	········Y···········	····················	····················	····················	····················	····················	
3226	MW321160.1	Severe acute respiratory syndrome coro	····················	····················	····················	····················	····················	····················	
3227	MW321163.1	Severe acute respiratory syndrome coro	····················	····················	····················	····················	····················	····················	
3228	MW321165.1	Severe acute respiratory syndrome coro	····················	····················	····················	····················	····················	····················	
3229	MW321166.1	Severe acute respiratory syndrome coro	····················	····················	····················	····················	····················	NN··················	

3230 MW321167.1 |Severe acute respiratory syndrome coro ....
3231 MW321171.1 |Severe acute respiratory syndrome coro ....
3233 MW321173.1 |Severe acute respiratory syndrome coro ....
3234 MW321174.1 |Severe acute respiratory syndrome coro ....
3235 MW321176.1 |Severe acute respiratory syndrome coro ....
3236 MW321177.1 |Severe acute respiratory syndrome coro ....
3237 MW321179.1 |Severe acute respiratory syndrome coro ....
3238 MW321184.1 |Severe acute respiratory syndrome coro ....
3239 MW321186.1 |Severe acute respiratory syndrome coro ....
3240 MW321189.1 |Severe acute respiratory syndrome coro ....
3241 MW321193.1 |Severe acute respiratory syndrome coro ....
3242 MW321196.1 |Severe acute respiratory syndrome coro ....
3243 MW321197.1 |Severe acute respiratory syndrome coro ....
3244 MW321201.1 |Severe acute respiratory syndrome coro ....
3245 MW321202.1 |Severe acute respiratory syndrome coro ....
3246 MW321203.1 |Severe acute respiratory syndrome coro ....
3247 MW321206.1 |Severe acute respiratory syndrome coro ....
3248 MW321207.1 |Severe acute respiratory syndrome coro ....
3249 MW321208.1 |Severe acute respiratory syndrome coro ....
3250 MW321211.1 |Severe acute respiratory syndrome coro ....
3251 MW321212.1 |Severe acute respiratory syndrome coro ....
3252 MW321214.1 |Severe acute respiratory syndrome coro ....
3253 MW321216.1 |Severe acute respiratory syndrome coro ....
3254 MW321217.1 |Severe acute respiratory syndrome coro ....
3256 MW321220.1 |Severe acute respiratory syndrome coro ....
3257 MW321221.1 |Severe acute respiratory syndrome coro ....
3258 MW321225.1 |Severe acute respiratory syndrome coro ....
3259 MW321226.1 |Severe acute respiratory syndrome coro ....
3260 MW321228.1 |Severe acute respiratory syndrome coro ....
3261 MW321229.1 |Severe acute respiratory syndrome coro ....
3262 MW321230.1 |Severe acute respiratory syndrome coro ....
3263 MW321233.1 |Severe acute respiratory syndrome coro ....
3264 MW321238.1 |Severe acute respiratory syndrome coro .... NNN................. 1
3265 MW321242.1 |Severe acute respiratory syndrome coro ....
3266 MW321243.1 |Severe acute respiratory syndrome coro ....
3267 MW321244.1 |Severe acute respiratory syndrome coro ....
3268 MW321245.1 |Severe acute respiratory syndrome coro ....
3269 MW321247.1 |Severe acute respiratory syndrome coro ....
3271 MW321250.1 |Severe acute respiratory syndrome coro ....
3272 MW321251.1 |Severe acute respiratory syndrome coro ....
3273 MW321252.1 |Severe acute respiratory syndrome coro ....
3277 MW321261.1 |Severe acute respiratory syndrome coro ....
3278 MW321262.1 |Severe acute respiratory syndrome coro ....
3279 MW321264.1 |Severe acute respiratory syndrome coro ....
3280 MW321265.1 |Severe acute respiratory syndrome coro ....
3281 MW321267.1 |Severe acute respiratory syndrome coro ....
3282 MW321269.1 |Severe acute respiratory syndrome coro ....
3283 MW321270.1 |Severe acute respiratory syndrome coro ....
3284 MW321271.1 |Severe acute respiratory syndrome coro ....
3285 MW321272.1 |Severe acute respiratory syndrome coro ....
3287 MW321275.1 |Severe acute respiratory syndrome coro ....
3288 MW321276.1 |Severe acute respiratory syndrome coro ....
3290 MW321278.1 |Severe acute respiratory syndrome coro ....
3292 MW321282.1 |Severe acute respiratory syndrome coro ....
3293 MW321283.1 |Severe acute respiratory syndrome coro ....
3295 MW321285.1 |Severe acute respiratory syndrome coro ....
3296 MW321286.1 |Severe acute respiratory syndrome coro ....
3297 MW321289.1 |Severe acute respiratory syndrome coro ....
3298 MW321292.1 |Severe acute respiratory syndrome coro ....W.............
3299 MW321295.1 |Severe acute respiratory syndrome coro ....
3301 MW321298.1 |Severe acute respiratory syndrome coro ....
3302 MW321300.1 |Severe acute respiratory syndrome coro ....
3303 MW321303.1 |Severe acute respiratory syndrome coro ....
3304 MW321304.1 |Severe acute respiratory syndrome coro ....
3306 MW321316.1 |Severe acute respiratory syndrome coro ....
3307 MW321320.1 |Severe acute respiratory syndrome coro ....
3308 MW321321.1 |Severe acute respiratory syndrome coro ....
3309 MW321322.1 |Severe acute respiratory syndrome coro ....
3310 MW321326.1 |Severe acute respiratory syndrome coro ....
3311 MW321327.1 |Severe acute respiratory syndrome coro ....
3313 MW321329.1 |Severe acute respiratory syndrome coro ....
3314 MW321332.1 |Severe acute respiratory syndrome coro ....
3315 MW321336.1 |Severe acute respiratory syndrome coro ....
3316 MW321337.1 |Severe acute respiratory syndrome coro ....
3318 MW321343.1 |Severe acute respiratory syndrome coro ....
3319 MW321344.1 |Severe acute respiratory syndrome coro ....
3320 MW321345.1 |Severe acute respiratory syndrome coro ....
3321 MW321346.1 |Severe acute respiratory syndrome coro ....

3322	MW321349.1	Severe acute respiratory syndrome coro	····················	····················	····················	····················	····················	····················
3323	MW321350.1	Severe acute respiratory syndrome coro	····················	····················	····················	····················	····················	····················
3324	MW321355.1	Severe acute respiratory syndrome coro	····················	····················	····················	····················	····················	····················
3325	MW321359.1	Severe acute respiratory syndrome coro	····················	····················	····················	····················	····················	····················
3328	MW321368.1	Severe acute respiratory syndrome coro	····················	····················	····················	····················	····················	····················
3329	MW321369.1	Severe acute respiratory syndrome coro	····················	····················	····················	····················	····················	····················
3330	MW321370.1	Severe acute respiratory syndrome coro	····················	····················	····················	····················	····················	····················
3331	MW321371.1	Severe acute respiratory syndrome coro	····················	····················	····················	····················	····················	····················
3332	MW321373.1	Severe acute respiratory syndrome coro	····················	····················	····················	····················	····················	····················
3333	MW321374.1	Severe acute respiratory syndrome coro	····················	····················	····················	····················	····················	····················
3335	MW321377.1	Severe acute respiratory syndrome coro	····················	····················	····················	····················	····················	····················
3336	MW321382.1	Severe acute respiratory syndrome coro	····················	····················	····················	····················	····················	····················
3337	MW321383.1	Severe acute respiratory syndrome coro	····················	····················	····················	····················	····················	····················
3338	MW321385.1	Severe acute respiratory syndrome coro	····················	····················	····················	····················	····················	····················
3339	MW321388.1	Severe acute respiratory syndrome coro	····················	····················	····················	····················	····················	····················
3340	MW321390.1	Severe acute respiratory syndrome coro	····················	····················	····················	····················	····················	····················
3341	MW321391.1	Severe acute respiratory syndrome coro	····················	····················	····················	····················	····················	····················
3342	MW321396.1	Severe acute respiratory syndrome coro	····················	····················	····················	····················	····················	····················
3343	MW321398.1	Severe acute respiratory syndrome coro	····················	····················	····················	····················	····················	····················
3344	MW321399.1	Severe acute respiratory syndrome coro	····················	····················	····················	····················	····················	····················
3345	MW321401.1	Severe acute respiratory syndrome coro	····················	····················	····················	····················	····················	····················
3346	MW321402.1	Severe acute respiratory syndrome coro	····················	····················	····················	····················	····················	····················
3347	MW321403.1	Severe acute respiratory syndrome coro	····················	····················	····················	····················	····················	····················
3348	MW321404.1	Severe acute respiratory syndrome coro	····················	····················	····················	····················	····················	····················
3349	MW321408.1	Severe acute respiratory syndrome coro	····················	····················	····················	····················	····················	····················
3350	MW321411.1	Severe acute respiratory syndrome coro	····················	····················	····················	····················	····················	····················
3351	MW321412.1	Severe acute respiratory syndrome coro	····················	····················	····················	····················	····················	····················
3354	MW321415.1	Severe acute respiratory syndrome coro	····················	····················	····················	····················	····················	····················
3355	MW321417.1	Severe acute respiratory syndrome coro	····················	····················	····················	····················	····················	····················
3356	MW321420.1	Severe acute respiratory syndrome coro	····················	····················	····················	····················	····················	····················
3357	MW321421.1	Severe acute respiratory syndrome coro	····················	····················	····················	····················	····················	····················
3358	MW321425.1	Severe acute respiratory syndrome coro	····················	····················	····················	····················	····················	····················
3359	MW321426.1	Severe acute respiratory syndrome coro	····················	····················	····················	····················	····················	····················
3361	MW321429.1	Severe acute respiratory syndrome coro	····················	····················	····················	····················	·Y··················	·············S······
3362	MW321430.1	Severe acute respiratory syndrome coro	····················	····················	····················	····················	····················	····················
3364	MW315209.1	Severe acute respiratory syndrome coro	····················	·A··················	····················	····················	····················	····················
3365	MW315210.1	Severe acute respiratory syndrome coro	····················	·A··················	····················	····················	····················	····················
3366	MW315211.1	Severe acute respiratory syndrome coro	····················	·A··················	····················	····················	····················	····················
3367	MW315212.1	Severe acute respiratory syndrome coro	····················	·A··················	····················	····················	····················	····················
3368	MW315213.1	Severe acute respiratory syndrome coro	····················	·A··················	····················	····················	····················	····················
3369	MW315214.1	Severe acute respiratory syndrome coro	····················	·A··················	····················	····················	····················	····················
3370	MW315215.1	Severe acute respiratory syndrome coro	····················	·A··················	····················	····················	····················	····················
3371	MW315965.1	Severe acute respiratory syndrome coro	····················	····················	····················	····················	····················	····················
3372	MW315966.1	Severe acute respiratory syndrome coro	····················	····················	····················	····················	····················	····················
3373	MW315967.1	Severe acute respiratory syndrome coro	····················	····················	····················	····················	····················	····················
3374	MW315968.1	Severe acute respiratory syndrome coro	····················	····················	····················	····················	····················	····················
3375	MW315969.1	Severe acute respiratory syndrome coro	····················	····················	····················	····················	····················	····················
3376	MW315970.1	Severe acute respiratory syndrome coro	····················	····················	····················	····················	····················	····················
3377	MW315971.1	Severe acute respiratory syndrome coro	····················	····················	····················	····················	····················	····················
3378	MW315972.1	Severe acute respiratory syndrome coro	····················	····················	····················	····················	····················	····················
3379	MW315973.1	Severe acute respiratory syndrome coro	··T·················	····················	····················	····················	····················	····················
3380	MW315974.1	Severe acute respiratory syndrome coro	····················	····················	····················	····················	····················	····················
3381	MW315975.1	Severe acute respiratory syndrome coro	····················	····················	····················	····················	····················	····················
3382	MW315976.1	Severe acute respiratory syndrome coro	····················	·A··················	····················	····················	····················	····················
3383	MW315977.1	Severe acute respiratory syndrome coro	····················	····················	····················	····················	····················	····················
3384	MW315978.1	Severe acute respiratory syndrome coro	····················	····················	····················	····················	····················	····················
3385	MW315979.1	Severe acute respiratory syndrome coro	····················	····················	····················	····················	····················	····················
3386	MW315980.1	Severe acute respiratory syndrome coro	····················	····················	····················	····················	····················	····················
3387	MW315981.1	Severe acute respiratory syndrome coro	····················	····················	····················	····················	····················	····················
3388	MW315982.1	Severe acute respiratory syndrome coro	····················	····················	····················	····················	····················	····················
3389	MW315983.1	Severe acute respiratory syndrome coro	····················	····················	····················	····················	····················	····················
3390	MW315984.1	Severe acute respiratory syndrome coro	····················	····················	····················	····················	····················	····················
3391	MW315985.1	Severe acute respiratory syndrome coro	····················	····················	····················	····················	····················	····················
3392	MW316741.1	Severe acute respiratory syndrome coro	····················	····················	····················	····················	····················	····················
3393	MW309426.1	Severe acute respiratory syndrome coro	····················	····················	····················	····················	····················	····················
3394	MW309429.1	Severe acute respiratory syndrome coro	····················	····················	····················	····················	····················	····················
3395	MW309438.1	Severe acute respiratory syndrome coro	····················	····················	····················	····················	····················	····················
3396	MW309443.1	Severe acute respiratory syndrome coro	····················	···A················	····················	····················	····················	····················
3397	MW309444.1	Severe acute respiratory syndrome coro	····················	····················	····················	····················	····················	····················
3398	MW309446.1	Severe acute respiratory syndrome coro	····················	····················	····················	····················	····················	····················
3399	MW309449.1	Severe acute respiratory syndrome coro	····················	····················	····················	····················	····················	····················
3400	MW309463.1	Severe acute respiratory syndrome coro	····················	····················	····················	····················	····················	····················
3401	MW309470.1	Severe acute respiratory syndrome coro	····················	····················	····················	····················	····················	····················
3402	MW309474.1	Severe acute respiratory syndrome coro	····················	····················	····················	····················	····················	····················
3405	MW309482.1	Severe acute respiratory syndrome coro	····················	····················	····················	····················	····················	····················
3407	MW309485.1	Severe acute respiratory syndrome coro	····················	····················	····················	····················	····················	····················
3409	MW309488.1	Severe acute respiratory syndrome coro	····················	····················	····················	····················	····················	····················
3410	MW309493.1	Severe acute respiratory syndrome coro	····················	····················	····················	····················	····················	····················

3411 MW309496.1 |Severe acute respiratory syndrome coro ···················· ····A··············· ···················· ···················· ···················· ····················
3412 MW309829.1 |Severe acute respiratory syndrome coro ···················· ···················· ···················· ···················· ···················· ····················
3413 MW309830.1 |Severe acute respiratory syndrome coro ···················· ···················· ···················· ···················· ···················· ····················
3414 MW309831.1 |Severe acute respiratory syndrome coro ···················· ···················· ···················· ···················· ···················· ····················
3415 MW310413.1 |Severe acute respiratory syndrome coro ···················· ·A·················· ···················· ···················· ···················· ····················
3416 MW310414.1 |Severe acute respiratory syndrome coro ···················· ···················· ···················· ···················· ···················· ····················
3417 MW310415.1 |Severe acute respiratory syndrome coro ···················· ·A·················· ···················· ···················· ···················· ····················
3418 MW310416.1 |Severe acute respiratory syndrome coro ···················· ···················· ···················· ···················· ···················· ····················
3419 MW310417.1 |Severe acute respiratory syndrome coro ···················· ·A·················· ···················· ···················· ···················· ····················
3420 MW310418.1 |Severe acute respiratory syndrome coro ···················· ··········A········· ···················· ···················· ···················· ····················
3421 MW310419.1 |Severe acute respiratory syndrome coro ···················· ···················· ···················· ···················· ···················· ····················
3422 MW310420.1 |Severe acute respiratory syndrome coro ···················· ············C······· ···················· ···················· ···················· ····················
3423 MW310421.1 |Severe acute respiratory syndrome coro ···················· ············C······· ···················· ···················· ···················· ····················
3424 MW310422.1 |Severe acute respiratory syndrome coro ···················· ···················· ···················· ···················· ···················· ····················
3425 MW310423.1 |Severe acute respiratory syndrome coro ···················· ···················· ···················· ···················· ···················· ····················
3426 MW310424.1 |Severe acute respiratory syndrome coro ···················· ···················· ···················· ···················· ···················· ····················
3427 MW310425.1 |Severe acute respiratory syndrome coro ···················· ···················· ···················· ···················· ···················· ····················
3428 MW310426.1 |Severe acute respiratory syndrome coro ···················· ·A·················· ···················· ···················· ···················· ····················
3429 MW310427.1 |Severe acute respiratory syndrome coro ···················· ·············A······ ···················· ···················· ···················· ····················
3430 MW310428.1 |Severe acute respiratory syndrome coro ···················· ·············A······ ···················· ···················· ···················· ····················
3431 MW310429.1 |Severe acute respiratory syndrome coro ···················· ···················· ···········T········ ···················· ···················· ····················
3432 MW310430.1 |Severe acute respiratory syndrome coro ···················· ···················· ···················· ···················· ···················· ····················
3433 MW310431.1 |Severe acute respiratory syndrome coro ···················· ···················· ···················· ···················· ···················· ····················
3434 MW310432.1 |Severe acute respiratory syndrome coro ···················· ·A·················· ···················· ···················· ···················· ····················
3435 MW310433.1 |Severe acute respiratory syndrome coro ···················· ···················· ···················· ···················· ···················· ····················
3436 MW310434.1 |Severe acute respiratory syndrome coro ···················· ···················· ···················· ···················· ···················· ····················
3437 MW310435.1 |Severe acute respiratory syndrome coro ···················· ···················· ···················· ···················· ···················· ····················
3438 MW310436.1 |Severe acute respiratory syndrome coro ···················· ·A·················· ···················· ···················· ···················· ····················
3439 MW310437.1 |Severe acute respiratory syndrome coro ···················· ·A·················· ···················· ···················· ···················· ····················
3440 MW310438.1 |Severe acute respiratory syndrome coro ···················· ·A·················· ···················· ···················· ···················· ····················
3441 MW310439.1 |Severe acute respiratory syndrome coro ···················· ·A·················· ···················· ···················· ···················· ····················
3442 MW310440.1 |Severe acute respiratory syndrome coro ···················· ···················· ···················· ···················· ···················· ····················
3443 MW310441.1 |Severe acute respiratory syndrome coro ···················· ···················· ···················· ···················· ···················· ····················
3444 MW310442.1 |Severe acute respiratory syndrome coro ···················· ···················· ···················· ···················· ···················· ····················
3445 MW310443.1 |Severe acute respiratory syndrome coro ···················· ···················· ···················· ···················· ···················· ····················
3446 MW310444.1 |Severe acute respiratory syndrome coro ···················· ···················· ···················· ···················· ···················· ····················
3447 MW310445.1 |Severe acute respiratory syndrome coro ···················· ···················· ···················· ···················· ···················· ····················
3448 MW310446.1 |Severe acute respiratory syndrome coro ···················· ···················· ···················· ···················· ···················· ····················
3449 MW310447.1 |Severe acute respiratory syndrome coro ···················· ·A·················· ···················· ···················· ···················· ····················
3450 MW310448.1 |Severe acute respiratory syndrome coro ···················· ···················· ···················· ···················· ···················· ····················
3451 MW310449.1 |Severe acute respiratory syndrome coro ···················· ·A·················· ···················· ···················· ···················· ····················
3452 MW310450.1 |Severe acute respiratory syndrome coro ···················· ···················· ···················· ···················· ···················· ····················
3453 MW310451.1 |Severe acute respiratory syndrome coro ···················· ·A·················· ···················· ···················· ···················· ····················
3454 MW310452.1 |Severe acute respiratory syndrome coro ···················· ·A·················· ···················· ···················· ···················· ····················
3455 MW310453.1 |Severe acute respiratory syndrome coro ···················· ···················· ···················· ···················· ···················· ····················
3456 MW310454.1 |Severe acute respiratory syndrome coro ···················· ···················· ···················· ···················· ···················· ····················
3457 MW310455.1 |Severe acute respiratory syndrome coro ···················· ···················· ···················· ···················· ···················· ····················
3458 MW310456.1 |Severe acute respiratory syndrome coro ···················· ···················· ···················· ···················· ···················· ····················
3459 MW314605.1 |Severe acute respiratory syndrome coro ···················· ···················· ···················· ···················· ···················· ····················
3460 MW302336.1 |Severe acute respiratory syndrome coro ···················· ···················· ···················· ···················· ···················· ····················
3461 MW302341.1 |Severe acute respiratory syndrome coro ···················· ···················· ···················· ···················· ···················· ····················
3462 MW306294.1 |Severe acute respiratory syndrome coro ···················· ·A·················· ···················· ···················· ···················· ····················
3463 MW306295.1 |Severe acute respiratory syndrome coro ···················· ···················· ···················· ···················· ···················· ····················
3464 MW306296.1 |Severe acute respiratory syndrome coro ···················· ···················· ···················· ···················· ···················· ····················
3465 MW306297.1 |Severe acute respiratory syndrome coro ···················· ···················· ···················· ···················· ···················· ····················
3466 MW306298.1 |Severe acute respiratory syndrome coro ···················· ···················· ···················· ···················· ···················· ····················
3467 MW306299.1 |Severe acute respiratory syndrome coro ···················· ···················· ···················· ···················· ··········Y········· ····················
3468 MW306300.1 |Severe acute respiratory syndrome coro ···················· ···················· ···················· ···················· ···················· ····················
3469 MW306304.1 |Severe acute respiratory syndrome coro ···················· ·A·················· ···················· ···················· ···················· ····················
3470 MW306306.1 |Severe acute respiratory syndrome coro ···················· ·A·················· ···················· ···················· ···················· ····················
3471 MW306307.1 |Severe acute respiratory syndrome coro ···················· ·A·················· ···················· ···················· ···················· ····················
3472 MW306308.1 |Severe acute respiratory syndrome coro ···················· ·A·················· ···················· ···················· ···················· ····················
3473 MW306309.1 |Severe acute respiratory syndrome coro ···················· ·A·················· ···················· ···················· ···················· ····················
3474 MW306315.1 |Severe acute respiratory syndrome coro ···················· ···················· ···················· ···················· ···················· ····················
3475 MW306318.1 |Severe acute respiratory syndrome coro ···················· ·A·················· ···················· ···················· ···················· ····················
3476 MW306319.1 |Severe acute respiratory syndrome coro ···················· ·A·················· ···················· ···················· ···················· ····················
3477 MW306320.1 |Severe acute respiratory syndrome coro ···················· ·A·················· ···················· ···················· N··················· ····················
3480 MW306323.1 |Severe acute respiratory syndrome coro ···················· ·A·················· ···················· ···················· ···················· ····················
3481 MW306324.1 |Severe acute respiratory syndrome coro ···················· ···················· ···················· ···················· ···················· ····················
3482 MW306325.1 |Severe acute respiratory syndrome coro ···················· ···················· ···················· ···················· ···················· ····················
3484 MW306328.1 |Severe acute respiratory syndrome coro ···················· ·A·················· ···················· ···················· ···················· ····················
3485 MW306329.1 |Severe acute respiratory syndrome coro ···················· ·A·················· ···················· ···················· ···················· ····················
3486 MW306330.1 |Severe acute respiratory syndrome coro ···················· ·A·················· ···················· ···················· ···················· ····················
3487 MW306332.1 |Severe acute respiratory syndrome coro ···················· ···················· ···················· ···················· ···················· ····················
3488 MW306333.1 |Severe acute respiratory syndrome coro ···················· ·············A······ ···················· ···················· ···················· ····················
3489 MW306334.1 |Severe acute respiratory syndrome coro ···················· ·A·················· ···················· ···················· ···················· ····················
3490 MW306335.1 |Severe acute respiratory syndrome coro ···················· ·A·················· ···················· ···················· ···················· ····················
3491 MW306336.1 |Severe acute respiratory syndrome coro ···················· ·A·················· ···················· ···················· ···················· ····················

3492	MW306337.1	Severe acute respiratory syndrome coro	....	.A..................	....	....	....	....	
3493	MW306338.1	Severe acute respiratory syndrome coro	....	.A..................	....	....	....	....	
3494	MW306339.1	Severe acute respiratory syndrome coro	....	.A..................	....	....	....	....	
3495	MW306340.1	Severe acute respiratory syndrome coro	....	.A..................	....	....	....	....	
3496	MW306341.1	Severe acute respiratory syndrome coro	....	.A..................	....	....	....	....	
3497	MW306342.1	Severe acute respiratory syndrome coro	....	.A..................	....	....	....	....	
3498	MW306343.1	Severe acute respiratory syndrome coro	....	.A..................	....	....	....	....	
3499	MW306344.1	Severe acute respiratory syndrome coro	....	.A..................	....	....	....	....	
3500	MW306345.1	Severe acute respiratory syndrome coro	....	.A..................	....	....	....	....	
3501	MW306346.1	Severe acute respiratory syndrome coro	....	.A..................	....	....	....	....	
3502	MW306347.1	Severe acute respiratory syndrome coro	....	.A..................	....	....	....	....	
3503	MW306348.1	Severe acute respiratory syndrome coro	....	....	....	....	....	....	
3504	MW306349.1	Severe acute respiratory syndrome coro	....	....	....	....	....	....	
3505	MW306350.1	Severe acute respiratory syndrome coro	....	.A..................	....	....	....	....	
3506	MW306351.1	Severe acute respiratory syndrome coro	....	....	....	....	....	....	
3507	MW306353.1	Severe acute respiratory syndrome coro	....	.A..................	....	....	....	....	
3508	MW306355.1	Severe acute respiratory syndrome coro	....	.A..................	....	....	....	....	
3509	MW306356.1	Severe acute respiratory syndrome coro	....	....	....	....	....	....	
3510	MW306358.1	Severe acute respiratory syndrome coro	....	.A..................	....	....	....	....	
3512	MW306360.1	Severe acute respiratory syndrome coro	....	.A..................	....	....	....	....	
3513	MW306361.1	Severe acute respiratory syndrome coro	....	.A..................	....	....	....	....	
3514	MW306362.1	Severe acute respiratory syndrome coro	....	.A..................	....	....	....	....	
3515	MW306363.1	Severe acute respiratory syndrome coro	....	.A..................	....	....	....	....	
3517	MW306366.1	Severe acute respiratory syndrome coro	....	.A..................	....	....	....	....	
3518	MW306367.1	Severe acute respiratory syndrome coro	....	....	....	....	....	....	
3519	MW306369.1	Severe acute respiratory syndrome coro	....	....	....	....	....	....	
3520	MW306370.1	Severe acute respiratory syndrome coro	....	....	....	....	....	....	
3521	MW306371.1	Severe acute respiratory syndrome coro	....	....	....	....	NNN.................	....	1
3522	MW306372.1	Severe acute respiratory syndrome coro	....	....	....	....	....	....	
3523	MW306373.1	Severe acute respiratory syndrome coro	....	....	....	....	....	....	
3524	MW306374.1	Severe acute respiratory syndrome coro	....	....	....	....	....	....	
3525	MW306375.1	Severe acute respiratory syndrome coro	....	....	....	....	....	....	
3526	MW306376.1	Severe acute respiratory syndrome coro	....	....	....	....	....	....	
3527	MW306377.1	Severe acute respiratory syndrome coro	....	....	....	....	....	....	
3528	MW306378.1	Severe acute respiratory syndrome coro	....	....	....	....	....	....	
3529	MW306379.1	Severe acute respiratory syndrome coro	....	....	....	....	....	....	
3530	MW306385.1	Severe acute respiratory syndrome coro	....	.A..................	....	....	....	....	
3531	MW306386.1	Severe acute respiratory syndrome coro	....	.A..................	....	....	....	....	
3532	MW306387.1	Severe acute respiratory syndrome coro	....	.A..................	....	....	....	....	
3533	MW306388.1	Severe acute respiratory syndrome coro	....	.A..................	....	....	....	....	
3534	MW306389.1	Severe acute respiratory syndrome coro	....	.A..................	....	....	....	....	
3535	MW306391.1	Severe acute respiratory syndrome coro	....	.A..................	....	....	....	....	
3536	MW306392.1	Severe acute respiratory syndrome coro	....	.A..................	....	....	....	....	
3537	MW306393.1	Severe acute respiratory syndrome coro	....	.A..................	....	....	....	....	
3538	MW306397.1	Severe acute respiratory syndrome coro	....	.A..................	....	....	....	....	
3539	MW306398.1	Severe acute respiratory syndrome coro	....	.A..................	....	....	....	....	
3540	MW306399.1	Severe acute respiratory syndrome coro	....	.A..................	....	....	....	....	
3541	MW306401.1	Severe acute respiratory syndrome coro	....	.A..................	....	....	....	....	
3542	MW306402.1	Severe acute respiratory syndrome coro	....	.A..................	....	....	....	....	
3543	MW306403.1	Severe acute respiratory syndrome coro	....	.A..................	....	....	....	....	
3544	MW306404.1	Severe acute respiratory syndrome coro	....	.A..................	....	....	....	....	
3546	MW306406.1	Severe acute respiratory syndrome coro	....	....	....	....	....	....	
3547	MW306407.1	Severe acute respiratory syndrome coro	....	....	....	....	....	....	
3548	MW306409.1	Severe acute respiratory syndrome coro	....	....	....	....	....	....	
3549	MW306410.1	Severe acute respiratory syndrome coro	....	....	....	....	....	....	
3550	MW306411.1	Severe acute respiratory syndrome coro	....	....	....	....	....	....	
3551	MW306412.1	Severe acute respiratory syndrome coro	....	....	....	....	....	....	
3552	MW306413.1	Severe acute respiratory syndrome coro	....	....	....	....	....	....	
3553	MW306415.1	Severe acute respiratory syndrome coro	....	....	....	....	....	....	
3554	MW306416.1	Severe acute respiratory syndrome coro	....	....	....	....	....	....	
3555	MW306417.1	Severe acute respiratory syndrome coro	....	....	....	....	....	....	
3556	MW306418.1	Severe acute respiratory syndrome coro	....	....	....	....	....	....	
3557	MW306419.1	Severe acute respiratory syndrome coro	....	....	....	....	....	....	
3558	MW306420.1	Severe acute respiratory syndrome coro	....	.A..................	....	....	....	....	
3559	MW306421.1	Severe acute respiratory syndrome coro	....	....	....	....	....	....	
3560	MW306422.1	Severe acute respiratory syndrome coro	....	....	....	....	....	....	
3561	MW306424.1	Severe acute respiratory syndrome coro	....	....	....	....	....	....	
3562	MW306426.1	Severe acute respiratory syndrome coro	....	....	....	....	....	....	
3563	MW306427.1	Severe acute respiratory syndrome coro	....	....	....	....	....	....	
3564	MW306428.1	Severe acute respiratory syndrome coro	....	....	....	....	....	....	
3565	MW306429.1	Severe acute respiratory syndrome coro	....	....	....	....	....	....	
3566	MW306430.1	Severe acute respiratory syndrome coro	....	....	....	....	....	....	
3567	MW306431.1	Severe acute respiratory syndrome coro	....	....	....	....	....	....	
3568	MW306432.1	Severe acute respiratory syndrome coro	....	....	....	....	....	....	
3569	MW306433.1	Severe acute respiratory syndrome coro	....	....	....	....	....	....	
3570	MW306434.1	Severe acute respiratory syndrome coro	....	....	....	....	....	....	
3571	MW306435.1	Severe acute respiratory syndrome coro	....	....	....	....	....	....	
3572	MW306436.1	Severe acute respiratory syndrome coro	....	....	....	....	....	....	

3573	MW306437.1	Severe acute respiratory syndrome coro	····················	····················	····················	····················	····················	····················
3574	MW306438.1	Severe acute respiratory syndrome coro	····················	·A··················	····················	····················	····················	····················
3575	MW306439.1	Severe acute respiratory syndrome coro	····················	·A··················	····················	····················	····················	····················
3576	MW306440.1	Severe acute respiratory syndrome coro	····················	·A··················	····················	····················	····················	····················
3577	MW306441.1	Severe acute respiratory syndrome coro	····················	·A··················	····················	····················	····················	····················
3578	MW306442.1	Severe acute respiratory syndrome coro	····················	·A··················	····················	····················	····················	····················
3579	MW306443.1	Severe acute respiratory syndrome coro	····················	····A···············	····················	····················	····················	····················
3580	MW306444.1	Severe acute respiratory syndrome coro	····················	····················	····················	····················	····················	····················
3581	MW306445.1	Severe acute respiratory syndrome coro	····················	·A··················	····················	····················	····················	····················
3582	MW306446.1	Severe acute respiratory syndrome coro	····················	·A··················	····················	····················	····················	····················
3583	MW306447.1	Severe acute respiratory syndrome coro	····················	·A··················	····················	····················	····················	····················
3584	MW306448.1	Severe acute respiratory syndrome coro	····················	····················	····················	····················	····················	····················
3585	MW306449.1	Severe acute respiratory syndrome coro	····················	····················	····················	····················	····················	····················
3586	MW306450.1	Severe acute respiratory syndrome coro	····················	····················	····················	····················	····················	····················
3587	MW306666.1	Severe acute respiratory syndrome coro	····················	····················	····················	····················	····················	····················
3588	MW306667.1	Severe acute respiratory syndrome coro	····················	····················	····················	····················	····················	····················
3589	MW306668.1	Severe acute respiratory syndrome coro	····················	····················	····················	····················	····················	····················
3590	MW307809.1	Severe acute respiratory syndrome coro	····················	····················	····················	····················	····················	····················
3591	MW307831.1	Severe acute respiratory syndrome coro	····················	·A··················	····················	····················	····················	····················
3592	MW307889.1	Severe acute respiratory syndrome coro	····················	·A··················	····················	····················	····················	····················
3593	MW307977.1	Severe acute respiratory syndrome coro	····················	·A··················	····················	····················	····················	····················
3594	MW307979.1	Severe acute respiratory syndrome coro	····················	·A··················	····················	····················	····················	····················
3595	MW308137.1	Severe acute respiratory syndrome coro	····················	·A··················	····················	····················	····················	····················
3596	MW301121.1	Severe acute respiratory syndrome coro	····················	····················	····················	····················	····················	····················
3597	MW301192.1	Severe acute respiratory syndrome coro	····················	····················	····················	····················	····················	····················
3598	MW295556.1	Severe acute respiratory syndrome coro	····················	····················	····················	····················	····················	····················
3599	MW295640.1	Severe acute respiratory syndrome coro	····················	····················	····················	····················	····················	····················
3600	MW295643.1	Severe acute respiratory syndrome coro	····················	····················	····················	····················	····················	····················
3601	MW290937.1	Severe acute respiratory syndrome coro	····················	····················	····················	····················	····················	····················
3602	MW290938.1	Severe acute respiratory syndrome coro	····················	····················	····················	····················	····················	····················
3603	MW290940.1	Severe acute respiratory syndrome coro	····················	····················	····················	····················	····················	····················
3604	MW290941.1	Severe acute respiratory syndrome coro	····················	····················	····················	····················	····················	····················
3605	MW290942.1	Severe acute respiratory syndrome coro	····················	····················	····················	····················	····················	····················
3606	MW290947.1	Severe acute respiratory syndrome coro	····················	····················	····················	····················	····················	····················
3607	MW290948.1	Severe acute respiratory syndrome coro	····················	····················	····················	····················	····················	····················
3608	MW290951.1	Severe acute respiratory syndrome coro	····················	····················	····················	····················	····················	····················
3609	MW290952.1	Severe acute respiratory syndrome coro	····················	····················	····················	····················	····················	····················
3610	MW290960.1	Severe acute respiratory syndrome coro	····················	····················	····················	····················	····················	····················
3611	MW290964.1	Severe acute respiratory syndrome coro	····················	····················	····················	····················	····················	····················
3612	MW290965.1	Severe acute respiratory syndrome coro	····················	····················	····················	····················	····················	····················
3613	MW290966.1	Severe acute respiratory syndrome coro	····················	····················	····················	····················	····················	····················
3614	MW290969.1	Severe acute respiratory syndrome coro	····················	····················	····················	····················	····················	····················
3615	MW290970.1	Severe acute respiratory syndrome coro	····················	····················	····················	····················	····················	····················
3616	MW292629.1	Severe acute respiratory syndrome coro	····················	·A··················	····················	····················	····················	····················
3617	MW292630.1	Severe acute respiratory syndrome coro	····················	····················	····················	····················	····················	····················
3618	MW292632.1	Severe acute respiratory syndrome coro	····················	····················	····················	····················	····················	····················
3619	MW292633.1	Severe acute respiratory syndrome coro	····················	····················	····················	····················	····················	····················
3620	MW292634.1	Severe acute respiratory syndrome coro	····················	····A···············	····················	····················	····················	····················
3621	MW292635.1	Severe acute respiratory syndrome coro	····················	····················	····················	····················	····················	····················
3622	MW292636.1	Severe acute respiratory syndrome coro	····················	····················	····················	····················	····················	····················
3623	MW292637.1	Severe acute respiratory syndrome coro	····················	····················	····················	····················	····················	····················
3624	MW292638.1	Severe acute respiratory syndrome coro	····················	····················	····················	····················	····················	····················
3625	MW292639.1	Severe acute respiratory syndrome coro	····················	····················	··········T·········	····················	····················	····················
3626	MW292640.1	Severe acute respiratory syndrome coro	····················	·A··················	····················	····················	····················	····················
3627	MW292641.1	Severe acute respiratory syndrome coro	····················	·A··················	····················	····················	····················	····················
3628	MW292642.1	Severe acute respiratory syndrome coro	····················	····················	····················	····················	····················	····················
3629	MW292644.1	Severe acute respiratory syndrome coro	····················	····················	····················	····················	····················	····················
3630	MW292645.1	Severe acute respiratory syndrome coro	····················	····················	····················	····················	····················	····················
3631	MW292646.1	Severe acute respiratory syndrome coro	····················	····················	····················	····················	····················	····················
3632	MW292647.1	Severe acute respiratory syndrome coro	····················	····················	····················	····················	····················	····················
3633	MW292648.1	Severe acute respiratory syndrome coro	····················	····················	····················	····················	····················	····················
3634	MW292649.1	Severe acute respiratory syndrome coro	····················	·A··················	····················	····················	····················	····················
3635	MW292650.1	Severe acute respiratory syndrome coro	····················	····················	····················	····················	····················	····················
3636	MW292651.1	Severe acute respiratory syndrome coro	····················	············C·······	····················	····················	····················	····················
3637	MW292652.1	Severe acute respiratory syndrome coro	····················	············C·······	····················	····················	····················	····················
3638	MW292653.1	Severe acute respiratory syndrome coro	····················	·A··················	····················	····················	····················	····················
3639	MW292654.1	Severe acute respiratory syndrome coro	····················	············C·······	····················	····················	····················	····················
3640	MW292656.1	Severe acute respiratory syndrome coro	····················	····················	····················	····················	····················	····················
3641	MW292657.1	Severe acute respiratory syndrome coro	····················	····················	····················	····················	····················	····················
3642	MW292658.1	Severe acute respiratory syndrome coro	····················	····················	····················	····················	····················	····················
3643	MW292659.1	Severe acute respiratory syndrome coro	····················	·A··················	····················	····················	····················	····················
3644	MW292661.1	Severe acute respiratory syndrome coro	····················	·A··················	····················	····················	····················	····················
3645	MW292662.1	Severe acute respiratory syndrome coro	····················	·A··················	····················	····················	····················	····················
3646	MW292663.1	Severe acute respiratory syndrome coro	····················	····················	····················	····················	····················	····················
3647	MW292664.1	Severe acute respiratory syndrome coro	····················	····················	····················	····················	····················	····················
3648	MW292665.1	Severe acute respiratory syndrome coro	····················	····················	····················	····················	····················	····················
3649	MW294007.1	Severe acute respiratory syndrome coro	····················	····················	····················	····················	····················	····················
3650	MW294011.1	Severe acute respiratory syndrome coro	····················	····················	····················	····················	····················	····················

3651	MT994707.1	|Severe acute respiratory syndrome coro	····················	····················	····················	····················	····················	····················	
3652	MW288251.1	|Severe acute respiratory syndrome coro	····················	····················	····················	····················	····················	····················	
3653	MW288252.1	|Severe acute respiratory syndrome coro	····················	····················	····················	····················	····················	····················	
3654	MW288253.1	|Severe acute respiratory syndrome coro	····················	····················	····················	····················	····················	····················	
3655	MW288254.1	|Severe acute respiratory syndrome coro	····················	····················	····················	····················	····················	····················	
3656	MW288255.1	|Severe acute respiratory syndrome coro	····················	····················	····················	····················	····················	····················	
3657	MW288256.1	|Severe acute respiratory syndrome coro	····················	····················	····················	····················	····················	····················	
3658	MW288257.1	|Severe acute respiratory syndrome coro	····················	····················	····················	····················	····················	····················	
3659	MW288258.1	|Severe acute respiratory syndrome coro	····················	····················	····················	····················	····················	····················	
3660	MW288259.1	|Severe acute respiratory syndrome coro	····················	·A··················	····················	····················	····················	····················	
3661	MW288260.1	|Severe acute respiratory syndrome coro	····················	····················	····················	····················	····················	····················	
3662	MW288261.1	|Severe acute respiratory syndrome coro	····················	····················	····················	····················	····················	····················	
3663	MW288262.1	|Severe acute respiratory syndrome coro	····················	····················	····················	····················	····················	····················	
3664	MW288263.1	|Severe acute respiratory syndrome coro	····················	····················	····················	····················	····················	····················	
3665	MW288264.1	|Severe acute respiratory syndrome coro	····················	····················	····················	····················	····················	····················	
3666	MW288265.1	|Severe acute respiratory syndrome coro	····················	····················	····················	····················	····················	····················	
3667	MW288266.1	|Severe acute respiratory syndrome coro	····················	····················	····················	····················	····················	····················	
3668	MW288267.1	|Severe acute respiratory syndrome coro	····················	····················	····················	····················	····················	····················	
3669	MW288268.1	|Severe acute respiratory syndrome coro	····················	····················	····················	····················	NNN·················	····················	1
3670	MW288269.1	|Severe acute respiratory syndrome coro	····················	····················	····················	····················	····················	····················	
3671	MW288270.1	|Severe acute respiratory syndrome coro	····················	····················	····················	····················	····················	····················	
3672	MW288271.1	|Severe acute respiratory syndrome coro	····················	····················	····················	····················	····················	····················	
3673	MW288272.1	|Severe acute respiratory syndrome coro	····················	····················	····················	····················	····················	····················	
3674	MW288273.1	|Severe acute respiratory syndrome coro	····················	····················	····················	····················	····················	····················	
3675	MW288274.1	|Severe acute respiratory syndrome coro	····················	····················	····················	····················	····················	····················	
3676	MW288275.1	|Severe acute respiratory syndrome coro	····················	····················	····················	····················	····················	····················	
3677	MW288276.1	|Severe acute respiratory syndrome coro	····················	····················	····················	····················	····················	····················	
3678	MW288277.1	|Severe acute respiratory syndrome coro	····················	····················	····················	····················	····················	····················	
3679	MW288278.1	|Severe acute respiratory syndrome coro	····················	····················	····················	····················	····················	····················	
3680	MW288279.1	|Severe acute respiratory syndrome coro	····················	····················	····················	····················	····················	····················	
3681	MW288280.1	|Severe acute respiratory syndrome coro	····················	····················	····················	····················	····················	····················	
3682	MW288281.1	|Severe acute respiratory syndrome coro	····················	····················	····················	····················	····················	····················	
3683	MW288282.1	|Severe acute respiratory syndrome coro	····················	····················	····················	····················	····················	····················	
3684	MW288283.1	|Severe acute respiratory syndrome coro	····················	····················	····················	····················	······R·············	····················	
3685	MW288284.1	|Severe acute respiratory syndrome coro	····················	····················	····················	····················	····················	····················	
3686	MW288285.1	|Severe acute respiratory syndrome coro	····················	····················	····················	····················	····················	·········N··········	
3687	MW286518.1	|Severe acute respiratory syndrome coro	····················	····················	····················	····················	····················	····················	
3688	MW286523.1	|Severe acute respiratory syndrome coro	····················	····················	····················	····················	····················	····················	
3689	MW286525.1	|Severe acute respiratory syndrome coro	····················	····················	····················	····················	····················	····················	
3690	MW286533.1	|Severe acute respiratory syndrome coro	····················	····················	····················	····················	····················	····················	
3691	MW286535.1	|Severe acute respiratory syndrome coro	····················	····················	····················	····················	····················	····················	
3692	MW286539.1	|Severe acute respiratory syndrome coro	····················	····················	····················	····················	····················	····················	
3693	MW286543.1	|Severe acute respiratory syndrome coro	····················	····················	····················	····················	····················	····················	
3695	MW286558.1	|Severe acute respiratory syndrome coro	····················	····················	····················	····················	····················	····················	
3696	MW286559.1	|Severe acute respiratory syndrome coro	····················	····················	····················	····················	····················	····················	
3697	MW286563.1	|Severe acute respiratory syndrome coro	····················	····················	····················	····················	····················	····················	
3698	MW286564.1	|Severe acute respiratory syndrome coro	····················	····················	····················	····················	····················	····················	
3699	MW286578.1	|Severe acute respiratory syndrome coro	····················	····················	····················	····················	····················	····················	
3700	MW286579.1	|Severe acute respiratory syndrome coro	····················	····················	····················	····················	····················	····················	
3702	MW286581.1	|Severe acute respiratory syndrome coro	····················	····················	····················	····················	····················	····················	
3703	MW286582.1	|Severe acute respiratory syndrome coro	····················	····················	····················	····················	····················	····················	
3704	MW286583.1	|Severe acute respiratory syndrome coro	····················	····················	····················	····················	····················	····················	
3705	MW286584.1	|Severe acute respiratory syndrome coro	····················	····················	····················	····················	····················	····················	
3706	MW286585.1	|Severe acute respiratory syndrome coro	····················	····················	····················	····················	····················	····················	
3707	MW286590.1	|Severe acute respiratory syndrome coro	····················	····················	····················	····················	····················	····················	
3708	MW286592.1	|Severe acute respiratory syndrome coro	····················	····················	····················	····················	····················	····················	
3709	MW286593.1	|Severe acute respiratory syndrome coro	····················	····················	····················	····················	····················	····················	
3710	MW286594.1	|Severe acute respiratory syndrome coro	····················	····················	····················	····················	····················	····················	
3711	MW286595.1	|Severe acute respiratory syndrome coro	····················	····················	····················	····················	····················	····················	
3712	MW286597.1	|Severe acute respiratory syndrome coro	····················	····················	····················	····················	····················	····················	
3713	MW286598.1	|Severe acute respiratory syndrome coro	····················	····················	····················	····················	····················	····················	
3714	MW286601.1	|Severe acute respiratory syndrome coro	····················	····················	····················	····················	····················	····················	
3716	MW286607.1	|Severe acute respiratory syndrome coro	····················	····················	····················	····················	····················	····················	
3717	MW286608.1	|Severe acute respiratory syndrome coro	····················	····················	····················	····················	····················	····················	
3718	MW286612.1	|Severe acute respiratory syndrome coro	····················	····················	····················	····················	····················	····················	
3719	MW286628.1	|Severe acute respiratory syndrome coro	····················	····················	····················	····················	····················	····················	
3720	MW286629.1	|Severe acute respiratory syndrome coro	····················	····················	····················	····················	····················	····················	
3721	MW286633.1	|Severe acute respiratory syndrome coro	····················	····················	····················	····················	····················	····················	
3722	MW286634.1	|Severe acute respiratory syndrome coro	····················	····················	····················	····················	····················	····················	
3723	MW286635.1	|Severe acute respiratory syndrome coro	····················	····················	····················	····················	····················	····················	
3724	MW286636.1	|Severe acute respiratory syndrome coro	····················	····················	····················	····················	····················	····················	
3725	MW286637.1	|Severe acute respiratory syndrome coro	····················	····················	····················	····················	····················	····················	
3726	MW286638.1	|Severe acute respiratory syndrome coro	····················	····················	····················	····················	····················	····················	
3727	MW286639.1	|Severe acute respiratory syndrome coro	····················	····················	····················	····················	····················	····················	
3728	MW286642.1	|Severe acute respiratory syndrome coro	····················	····················	····················	····················	····················	····················	
3729	MW286644.1	|Severe acute respiratory syndrome coro	····················	····················	····················	····················	····················	····················	
3730	MW286647.1	|Severe acute respiratory syndrome coro	····················	····················	····················	····················	····················	····················	
3731	MW286649.1	|Severe acute respiratory syndrome coro	····················	····················	····················	····················	····················	····················	

3732 MW286650.1 |Severe acute respiratory syndrome coro ···················· ···················· ···················· ···················· ···················· ····················
3733 MW286652.1 |Severe acute respiratory syndrome coro ···················· ···················· ···················· ···················· ···················· ····················
3734 MW286654.1 |Severe acute respiratory syndrome coro ···················· ···················· ···················· ···················· ···················· ····················
3735 MW286655.1 |Severe acute respiratory syndrome coro ···················· ···················· ···················· ···················· ···················· ····················
3736 MW286656.1 |Severe acute respiratory syndrome coro ···················· ···················· ···················· ···················· ···················· ····················
3737 MW286661.1 |Severe acute respiratory syndrome coro ···················· ···················· ···················· ···················· ···················· ····················
3738 MW286665.1 |Severe acute respiratory syndrome coro ···················· ···················· ···················· ···················· ···················· ····················
3739 MW286668.1 |Severe acute respiratory syndrome coro ···················· ···················· ···················· ···················· ···················· ····················
3740 MW286669.1 |Severe acute respiratory syndrome coro ···················· ···················· ···················· ···················· ···················· ····················
3741 MW286670.1 |Severe acute respiratory syndrome coro ···················· ···················· ···················· ···················· ···················· ····················
3742 MW286671.1 |Severe acute respiratory syndrome coro ···················· ···················· ···················· ···················· ···················· ····················
3743 MW286676.1 |Severe acute respiratory syndrome coro ···················· ···················· ···················· ···················· ···················· ····················
3744 MW286677.1 |Severe acute respiratory syndrome coro ···················· ···················· ···················· ···················· ···················· ····················
3745 MW286678.1 |Severe acute respiratory syndrome coro ···················· ···················· ···················· ···················· ···················· ····················
3746 MW286680.1 |Severe acute respiratory syndrome coro ···················· ···················· ···················· ···················· ···················· ····················
3747 MW286682.1 |Severe acute respiratory syndrome coro ···················· ···················· ···················· ···················· ···················· ····················
3748 MW286686.1 |Severe acute respiratory syndrome coro ···················· ···················· ···················· ···················· ···················· ····················
3749 MW286690.1 |Severe acute respiratory syndrome coro ···················· ···················· ···················· ···················· ···················· ····················
3750 MW286698.1 |Severe acute respiratory syndrome coro ···················· ···················· ···················· ···················· ···················· ····················
3751 MW286700.1 |Severe acute respiratory syndrome coro ···················· ···················· ···················· ···················· ···················· ····················
3752 MW286702.1 |Severe acute respiratory syndrome coro ···················· ···················· ···················· ···················· ···················· ····················
3753 MW286705.1 |Severe acute respiratory syndrome coro ···················· ···················· ···················· ···················· ···················· ····················
3754 MW286708.1 |Severe acute respiratory syndrome coro ···················· ···················· ···················· ···················· ···················· ····················
3755 MW286712.1 |Severe acute respiratory syndrome coro ···················· ···················· ···················· ···················· ···················· ····················
3756 MW286714.1 |Severe acute respiratory syndrome coro ···················· ···················· ···················· ···················· ···················· ····················
3757 MW286715.1 |Severe acute respiratory syndrome coro ···················· ···················· ···················· ···················· ···················· ····················
3758 MW286716.1 |Severe acute respiratory syndrome coro ···················· ···················· ···················· ···················· ···················· ····················
3759 MW286717.1 |Severe acute respiratory syndrome coro ···················· ···················· ···················· ···················· ···················· ····················
3760 MW286718.1 |Severe acute respiratory syndrome coro ···················· ···················· ···················· ···················· ···················· ····················
3761 MW286719.1 |Severe acute respiratory syndrome coro ···················· ···················· ···················· ···················· ···················· ····················
3762 MW286720.1 |Severe acute respiratory syndrome coro ···················· ···················· ···················· ···················· ···················· ····················
3763 MW286721.1 |Severe acute respiratory syndrome coro ···················· ···················· ···················· ···················· ···················· ····················
3764 MW286722.1 |Severe acute respiratory syndrome coro ···················· ···················· ···················· ···················· ···················· ····················
3765 MW286725.1 |Severe acute respiratory syndrome coro ···················· ···················· ···················· ···················· ···················· ····················
3766 MW286726.1 |Severe acute respiratory syndrome coro ···················· ···················· ···················· ···················· ···················· ····················
3767 MW286727.1 |Severe acute respiratory syndrome coro ···················· ···················· ···················· ···················· ···················· ····················
3768 MW286728.1 |Severe acute respiratory syndrome coro ···················· ···················· ···················· ···················· ···················· ····················
3769 MW286729.1 |Severe acute respiratory syndrome coro ···················· ···················· ···················· ···················· ···················· ····················
3770 MW286730.1 |Severe acute respiratory syndrome coro ···················· ···················· ···················· ···················· ···················· ····················
3771 MW286732.1 |Severe acute respiratory syndrome coro ···················· ···················· ···················· ···················· ···················· ····················
3772 MW286733.1 |Severe acute respiratory syndrome coro ···················· ···················· ···················· ···················· ···················· ····················
3773 MW286734.1 |Severe acute respiratory syndrome coro ···················· ···················· ···················· ···················· ···················· ····················
3774 MW286735.1 |Severe acute respiratory syndrome coro ···················· ···················· ···················· ···················· ···················· ····················
3775 MW286737.1 |Severe acute respiratory syndrome coro ···················· ···················· ···················· ···················· ···················· ····················
3776 MW286739.1 |Severe acute respiratory syndrome coro ···················· ···················· ···················· ···················· ···················· ····················
3777 MW286740.1 |Severe acute respiratory syndrome coro ···················· ···················· ···················· ···················· ···················· ····················
3778 MW286741.1 |Severe acute respiratory syndrome coro ···················· ···················· ···················· ···················· ···················· ····················
3779 MW286742.1 |Severe acute respiratory syndrome coro ···················· ···················· ···················· ···················· ···················· ····················
3780 MW286743.1 |Severe acute respiratory syndrome coro ···················· ···················· ···················· ···················· ···················· ····················
3781 MW286745.1 |Severe acute respiratory syndrome coro ···················· ···················· ···················· ···················· ···················· ····················
3782 MW286746.1 |Severe acute respiratory syndrome coro ···················· ···················· ···················· ···················· ···················· ····················
3783 MW286749.1 |Severe acute respiratory syndrome coro ···················· ···················· ···················· ···················· ···················· ····················
3784 MW286751.1 |Severe acute respiratory syndrome coro ···················· ···················· ···················· ···················· ···················· ····················
3785 MW286752.1 |Severe acute respiratory syndrome coro ···················· ···················· ···················· ···················· ···················· ····················
3786 MW286755.1 |Severe acute respiratory syndrome coro ···················· ···················· ···················· ···················· ···················· ····················
3787 MW286757.1 |Severe acute respiratory syndrome coro ···················· ···················· ···················· ···················· ···················· ····················
3788 MW286759.1 |Severe acute respiratory syndrome coro ···················· ···················· ···················· ···················· ···················· ····················
3789 MW286760.1 |Severe acute respiratory syndrome coro ···················· ···················· ···················· ···················· ···················· ····················
3790 MW286761.1 |Severe acute respiratory syndrome coro ···················· ···················· ···················· ···················· ···················· ····················
3791 MW281864.1 |Severe acute respiratory syndrome coro ···················· ···················· ···················· ···················· ···················· ····················
3792 LC593801.1 |Severe acute respiratory syndrome coro ···················· ···················· ···················· ···················· ···················· ····················
3793 LC593802.1 |Severe acute respiratory syndrome coro ···················· ···················· ···················· ···················· ···················· ····················
3794 LC593803.1 |Severe acute respiratory syndrome coro ···················· ···················· ···················· ···················· ···················· ····················
3795 LC593804.1 |Severe acute respiratory syndrome coro ···················· ···················· ···················· ···················· ···················· ····················
3796 LC593805.1 |Severe acute respiratory syndrome coro ···················· ···················· ···················· ···················· ···················· ····················
3797 LC593806.1 |Severe acute respiratory syndrome coro ···················· ···················· ···················· ···················· ···················· ····················
3798 LC593807.1 |Severe acute respiratory syndrome coro ···················· ···················· ···················· ···················· ···················· ····················
3799 LC593808.1 |Severe acute respiratory syndrome coro ···················· ···················· ···················· ···················· ···················· ····················
3800 LC593809.1 |Severe acute respiratory syndrome coro ···················· ···················· ···················· ···················· ···················· ····················
3801 LC593810.1 |Severe acute respiratory syndrome coro ···················· ···················· ···················· ···················· ···················· ····················
3802 LC593811.1 |Severe acute respiratory syndrome coro ···················· ···················· ···················· ···················· ···················· ····················
3803 LC593812.1 |Severe acute respiratory syndrome coro ···················· ···················· ···················· ···················· ···················· ····················
3804 LC593813.1 |Severe acute respiratory syndrome coro ···················· ···················· ···················· ···················· ···················· ····················
3805 LC593814.1 |Severe acute respiratory syndrome coro ···················· ···················· ···················· ···················· ···················· ····················
3806 LC593815.1 |Severe acute respiratory syndrome coro ···················· ···················· ···················· ···················· ···················· ····················
3807 LC593816.1 |Severe acute respiratory syndrome coro ···················· ···················· ···················· ···················· ···················· ····················
3808 LC593817.1 |Severe acute respiratory syndrome coro ···················· ···················· ···················· ···················· ···················· ····················
3809 LC593818.1 |Severe acute respiratory syndrome coro ···················· ···················· ···················· ···················· ···················· ····················

3810 LC593819.1 |Severe acute respiratory syndrome coro ....
3811 MW276159.1 |Severe acute respiratory syndrome coro .... .A.................. ....
3812 MW276160.1 |Severe acute respiratory syndrome coro .... .A.................. ....
3813 MW276161.1 |Severe acute respiratory syndrome coro .... .A.................. ....
3814 MW276163.1 |Severe acute respiratory syndrome coro .... .A.................. ....
3815 MW276166.1 |Severe acute respiratory syndrome coro .... .A.................. ....
3816 MW276167.1 |Severe acute respiratory syndrome coro .... .A.................. ....
3817 MW276169.1 |Severe acute respiratory syndrome coro .... .A.................. ....
3818 MW276170.1 |Severe acute respiratory syndrome coro .... .A.................. ....
3819 MW276172.1 |Severe acute respiratory syndrome coro .... .A.................. ....
3820 MW276173.1 |Severe acute respiratory syndrome coro .... .A.................. ....
3821 MW276174.1 |Severe acute respiratory syndrome coro .... .A.................. ....
3822 MW276175.1 |Severe acute respiratory syndrome coro .... .A.................. .... N................... ....
3823 MW276178.1 |Severe acute respiratory syndrome coro .... .A.................. ....
3824 MW276182.1 |Severe acute respiratory syndrome coro .... .A.................. ....
3825 MW276184.1 |Severe acute respiratory syndrome coro .... .A.................. ....
3826 MW276185.1 |Severe acute respiratory syndrome coro .... .A.................. ....
3827 MW276189.1 |Severe acute respiratory syndrome coro ....
3828 MW276190.1 |Severe acute respiratory syndrome coro ....
3829 MW276191.1 |Severe acute respiratory syndrome coro ....
3830 MW276192.1 |Severe acute respiratory syndrome coro .... .A.................. ....
3831 MW276193.1 |Severe acute respiratory syndrome coro ....
3832 MW276194.1 |Severe acute respiratory syndrome coro .... .A.................. ....
3833 MW276196.1 |Severe acute respiratory syndrome coro .... .A.................. ....
3834 MW276197.1 |Severe acute respiratory syndrome coro .... .A.................. ....
3835 MW276198.1 |Severe acute respiratory syndrome coro ....
3836 MW276199.1 |Severe acute respiratory syndrome coro .... .A.................. ....
3837 MW276201.1 |Severe acute respiratory syndrome coro .... .A.................. ....
3838 MW276202.1 |Severe acute respiratory syndrome coro .... .A.................. ....
3839 MW276203.1 |Severe acute respiratory syndrome coro .... .A.................. ....
3840 MW276208.1 |Severe acute respiratory syndrome coro .... .A.................. ....
3841 MW276211.1 |Severe acute respiratory syndrome coro ....
3842 MW276213.1 |Severe acute respiratory syndrome coro ....
3843 MW276215.1 |Severe acute respiratory syndrome coro ....
3844 MW276217.1 |Severe acute respiratory syndrome coro ....
3845 MW276218.1 |Severe acute respiratory syndrome coro ....
3846 MW276219.1 |Severe acute respiratory syndrome coro ....
3847 MW276222.1 |Severe acute respiratory syndrome coro .... .A.................. ....
3848 MW276226.1 |Severe acute respiratory syndrome coro ....
3849 MW276227.1 |Severe acute respiratory syndrome coro ....
3850 MW276228.1 |Severe acute respiratory syndrome coro .... .A.................. ....
3851 MW276229.1 |Severe acute respiratory syndrome coro ....
3852 MW276230.1 |Severe acute respiratory syndrome coro ....
3853 MW276231.1 |Severe acute respiratory syndrome coro ....
3854 MW276232.1 |Severe acute respiratory syndrome coro .... .A.................. ....
3855 MW276240.1 |Severe acute respiratory syndrome coro ....
3856 MW276241.1 |Severe acute respiratory syndrome coro .... .A.................. ....
3857 MW276242.1 |Severe acute respiratory syndrome coro ....
3858 MW276243.1 |Severe acute respiratory syndrome coro ....
3859 MW276244.1 |Severe acute respiratory syndrome coro .... .A.................. ....
3860 MW276245.1 |Severe acute respiratory syndrome coro ....
3861 MW276247.1 |Severe acute respiratory syndrome coro ....
3862 MW276248.1 |Severe acute respiratory syndrome coro .... .A.................. ....
3863 MW276249.1 |Severe acute respiratory syndrome coro .... .A.................. ....
3864 MW276250.1 |Severe acute respiratory syndrome coro .... .A.................. ....
3865 MW276251.1 |Severe acute respiratory syndrome coro ....
3866 MW276252.1 |Severe acute respiratory syndrome coro ....
3867 MW276253.1 |Severe acute respiratory syndrome coro ....
3869 MW276255.1 |Severe acute respiratory syndrome coro ....
3870 MW276256.1 |Severe acute respiratory syndrome coro ....
3871 MW276257.1 |Severe acute respiratory syndrome coro ....
3872 MW276258.1 |Severe acute respiratory syndrome coro ....
3873 MW276259.1 |Severe acute respiratory syndrome coro ....
3874 MW276260.1 |Severe acute respiratory syndrome coro ....
3875 MW276261.1 |Severe acute respiratory syndrome coro ....
3876 MW276263.1 |Severe acute respiratory syndrome coro .... .A.................. ....
3877 MW276265.1 |Severe acute respiratory syndrome coro ....
3878 MW276268.1 |Severe acute respiratory syndrome coro ....
3879 MW276269.1 |Severe acute respiratory syndrome coro ....
3880 MW276270.1 |Severe acute respiratory syndrome coro ....
3881 MW276272.1 |Severe acute respiratory syndrome coro ....
3882 MW276278.1 |Severe acute respiratory syndrome coro .... .A.................. ....
3883 MW276279.1 |Severe acute respiratory syndrome coro ....
3884 MW276283.1 |Severe acute respiratory syndrome coro .... .A.................. ....
3885 MW276286.1 |Severe acute respiratory syndrome coro .... .A.................. ....
3886 MW276287.1 |Severe acute respiratory syndrome coro ....
3887 MW276288.1 |Severe acute respiratory syndrome coro ....
3888 MW276289.1 |Severe acute respiratory syndrome coro .... .A.................. ....

3889 MW276290.1 |Severe acute respiratory syndrome coro ···················· ·A·················· ····················· ···················· ···················· ····················
3890 MW276292.1 |Severe acute respiratory syndrome coro ···················· ···················· ····················· ···················· ···················· ····················
3891 MW276293.1 |Severe acute respiratory syndrome coro ···················· ···················· ····················· ···················· ···················· ····················
3892 MW276294.1 |Severe acute respiratory syndrome coro ···················· ·A·················· ····················· ···················· ···················· ····················
3893 MW276295.1 |Severe acute respiratory syndrome coro ···················· ·A·················· ····················· ···················· ···················· ····················
3894 MW276296.1 |Severe acute respiratory syndrome coro ···················· ·A·················· ····················· ···················· ···················· ····················
3895 MW276297.1 |Severe acute respiratory syndrome coro ···················· ·A·················· ····················· ···················· ···················· ····················
3896 MW276298.1 |Severe acute respiratory syndrome coro ···················· ·A·················· ····················· ···················· ···················· ····················
3897 MW276299.1 |Severe acute respiratory syndrome coro ···················· ···················· ····················· ···················· ···················· ····················
3898 MW276300.1 |Severe acute respiratory syndrome coro ···················· ···················· ····················· ···················· ···················· ····················
3899 MW276301.1 |Severe acute respiratory syndrome coro ···················· ···················· ····················· ···················· ···················· ····················
3900 MW276302.1 |Severe acute respiratory syndrome coro ···················· ·A·················· ····················· ···················· ···················· ····················
3901 MW276303.1 |Severe acute respiratory syndrome coro ···················· ·A·················· ····················· ···················· ···················· ····················
3902 MW276304.1 |Severe acute respiratory syndrome coro ···················· ·A·················· ····················· ···················· ···················· ····················
3903 MW276305.1 |Severe acute respiratory syndrome coro ···················· ·A·················· ····················· ···················· ···················· ····················
3904 MW276306.1 |Severe acute respiratory syndrome coro ···················· ·A·················· ····················· ···················· ···················· ····················
3905 MW276307.1 |Severe acute respiratory syndrome coro ···················· ·A·················· ····················· ···················· ···················· ····················
3906 MW276308.1 |Severe acute respiratory syndrome coro ···················· ·A·················· ····················· ···················· ···················· ····················
3907 MW276309.1 |Severe acute respiratory syndrome coro ···················· ·A·················· ····················· ···················· ···················· ····················
3908 MW276310.1 |Severe acute respiratory syndrome coro ···················· ···················· ····················· ···················· ···················· ····················
3909 MW276311.1 |Severe acute respiratory syndrome coro ···················· ·A·················· ····················· ···················· ···················· ····················
3910 MW276312.1 |Severe acute respiratory syndrome coro ···················· ···················· ····················· ···················· ···················· ····················
3911 MW276313.1 |Severe acute respiratory syndrome coro ···················· ·A·················· ····················· ···················· ···················· ····················
3912 MW276314.1 |Severe acute respiratory syndrome coro ···················· ···················· ····················· ···················· ···················· ····················
3913 MW276315.1 |Severe acute respiratory syndrome coro ···················· ·A·················· ····················· ···················· ···················· ····················
3914 MW276317.1 |Severe acute respiratory syndrome coro ···················· ·A·················· ····················· ···················· ···················· ····················
3915 MW276318.1 |Severe acute respiratory syndrome coro ···················· ·A·················· ····················· ···················· ···················· ····················
3916 MW276319.1 |Severe acute respiratory syndrome coro ···················· ·A·················· ····················· ···················· ···················· ····················
3917 MW276320.1 |Severe acute respiratory syndrome coro ···················· ···················· ····················· ···················· ···················· ····················
3918 MW276321.1 |Severe acute respiratory syndrome coro ···················· ·A·················· ····················· ···················· ···················· ····················
3919 MW276323.1 |Severe acute respiratory syndrome coro ···················· ···················· ····················· ···················· ···················· ····················
3920 MW276324.1 |Severe acute respiratory syndrome coro ···················· ···················· ····················· ···················· ···················· ····················
3921 MW276325.1 |Severe acute respiratory syndrome coro ···················· ···················· ····················· ···················· ···················· ····················
3922 MW276326.1 |Severe acute respiratory syndrome coro ···················· ···················· ····················· ···················· ···················· ····················
3923 MW276328.1 |Severe acute respiratory syndrome coro ···················· ···················· ····················· ···················· ···················· ····················
3924 MW276330.1 |Severe acute respiratory syndrome coro ···················· ···················· ····················· ···················· ···················· ····················
3925 MW276331.1 |Severe acute respiratory syndrome coro ···················· ···················· ····················· ···················· ···················· ····················
3926 MW276332.1 |Severe acute respiratory syndrome coro ···················· ···················· ····················· ···················· ···················· ····················
3927 MW276333.1 |Severe acute respiratory syndrome coro ···················· ···················· ····················· ···················· ···················· ····················
3928 MW276334.1 |Severe acute respiratory syndrome coro ···················· ···················· ····················· ···················· ···················· ····················
3929 MW276336.1 |Severe acute respiratory syndrome coro ···················· ···················· ····················· ···················· ···················· ····················
3930 MW276337.1 |Severe acute respiratory syndrome coro ···················· ···················· ····················· ···················· ···················· ····················
3931 MW276338.1 |Severe acute respiratory syndrome coro ···················· ···················· ····················· ···················· ···················· ····················
3932 MW276339.1 |Severe acute respiratory syndrome coro ···················· ···················· ····················· ···················· ···················· ····················
3933 MW276340.1 |Severe acute respiratory syndrome coro ···················· ···················· ····················· ···················· ···················· ····················
3934 MW276341.1 |Severe acute respiratory syndrome coro ···················· ···················· ····················· ···················· ···················· ····················
3935 MW276342.1 |Severe acute respiratory syndrome coro ···················· ···················· ····················· ···················· ···················· ····················
3936 MW276343.1 |Severe acute respiratory syndrome coro ···················· ···················· ····················· ···················· ···················· ····················
3937 MW276345.1 |Severe acute respiratory syndrome coro ···················· ···················· ····················· ···················· ···················· ····················
3938 MW276346.1 |Severe acute respiratory syndrome coro ···················· ···················· ····················· ···················· ···················· ····················
3939 MW276347.1 |Severe acute respiratory syndrome coro ···················· ·A·················· ····················· ···················· ···················· ····················
3940 MW276348.1 |Severe acute respiratory syndrome coro ···················· ·A·················· ····················· ···················· ···················· ····················
3941 MW276349.1 |Severe acute respiratory syndrome coro ···················· ·A·················· ····················· ···················· ···················· ····················
3942 MW276350.1 |Severe acute respiratory syndrome coro ···················· ·A·················· ····················· ···················· ···················· ····················
3943 MW276351.1 |Severe acute respiratory syndrome coro ···················· ·A·················· ····················· ···················· ···················· ····················
3944 MW276352.1 |Severe acute respiratory syndrome coro ···················· ·A·················· ····················· ···················· ···················· ····················
3945 MW276353.1 |Severe acute respiratory syndrome coro ···················· ···················· ····················· ···················· ···················· ····················
3946 MW276356.1 |Severe acute respiratory syndrome coro ···················· ···················· ····················· ···················· ···················· ····················
3947 MW276357.1 |Severe acute respiratory syndrome coro ···················· ···················· ····················· ···················· ···················· ····················
3948 MW276358.1 |Severe acute respiratory syndrome coro ···················· ···················· ····················· ···················· ···················· ····················
3949 MW276359.1 |Severe acute respiratory syndrome coro ···················· ···················· ····················· ···················· ···················· ····················
3951 MW276361.1 |Severe acute respiratory syndrome coro ···················· ···················· ····················· ···················· ···················· ····················
3952 MW276362.1 |Severe acute respiratory syndrome coro ···················· ·A·················· ····················· ···················· ···················· ····················
3953 MW276363.1 |Severe acute respiratory syndrome coro ···················· ···················· ····················· ···················· ···················· ····················
3954 MW276364.1 |Severe acute respiratory syndrome coro ···················· ···················· ····················· ···················· ···················· ····················
3955 MW276365.1 |Severe acute respiratory syndrome coro ···················· ···················· ····················· ···················· ···················· ····················
3956 MW276366.1 |Severe acute respiratory syndrome coro ···················· ···················· ····················· ···················· ···················· ····················
3957 MW276367.1 |Severe acute respiratory syndrome coro ···················· ·A·················· ····················· ···················· ···················· ····················
3958 MW276368.1 |Severe acute respiratory syndrome coro ···················· ···················· ····················· ···················· ···················· ····················
3959 MW276369.1 |Severe acute respiratory syndrome coro ···················· ·A·················· ····················· ···················· ···················· ····················
3960 MW276370.1 |Severe acute respiratory syndrome coro ···················· ···················· ····················· ···················· ···················· ····················
3961 MW276371.1 |Severe acute respiratory syndrome coro ···················· ···················· ····················· ···················· ···················· ····················
3962 MW276372.1 |Severe acute respiratory syndrome coro ···················· ···················· ····················· ···················· ···················· ····················
3963 MW276373.1 |Severe acute respiratory syndrome coro ···················· ···················· ····················· ···················· ···················· ····················
3964 MW276374.1 |Severe acute respiratory syndrome coro ···················· ·A·················· ····················· ···················· ···················· ····················
3965 MW276375.1 |Severe acute respiratory syndrome coro ···················· ···················· ····················· ···················· ···················· ····················
3966 MW276376.1 |Severe acute respiratory syndrome coro ···················· ···················· ····················· ···················· ···················· ····················
3967 MW276377.1 |Severe acute respiratory syndrome coro ···················· ·A·················· ····················· ···················· ···················· ····················

3968 MW276380.1 |Severe acute respiratory syndrome coro ···················· ·A·················· ···················· ···················· ···················· ····················
3969 MW276381.1 |Severe acute respiratory syndrome coro ···················· ·A·················· ···················· ···················· ···················· ····················
3970 MW276382.1 |Severe acute respiratory syndrome coro ···················· ·A·················· ···················· ···················· ···················· ····················
3971 MW276383.1 |Severe acute respiratory syndrome coro ···················· ·A·················· ···················· ···················· ···················· ····················
3972 MW276384.1 |Severe acute respiratory syndrome coro ···················· ·A·················· ···················· ···················· ···················· ····················
3975 MW276387.1 |Severe acute respiratory syndrome coro ···················· ···················· ···················· ···················· ···················· ····················
3976 MW276388.1 |Severe acute respiratory syndrome coro ···················· ···················· ···················· ···················· ···················· ····················
3977 MW276389.1 |Severe acute respiratory syndrome coro ···················· ···················· ···················· ···················· ···················· ····················
3978 MW276390.1 |Severe acute respiratory syndrome coro ···················· ···················· ···················· ···················· ···················· ····················
3979 MW276391.1 |Severe acute respiratory syndrome coro ···················· ···················· ···················· ···················· ···················· ····················
3980 MW276393.1 |Severe acute respiratory syndrome coro ···················· ·A·················· ···················· ···················· ··N················· ····················
3981 MW276394.1 |Severe acute respiratory syndrome coro ···················· ·A·················· ···················· ···················· ···················· ····················
3982 MW276395.1 |Severe acute respiratory syndrome coro ···················· ·A·················· ···················· ···················· ···················· ····················
3983 MW276396.1 |Severe acute respiratory syndrome coro ···················· ···················· ···················· ···················· ···················· ····················
3984 MW276397.1 |Severe acute respiratory syndrome coro ···················· ···················· ···················· ···················· ···················· ····················
3985 MW276398.1 |Severe acute respiratory syndrome coro ···················· ···················· ···················· ···················· ···················· ····················
3986 MW276399.1 |Severe acute respiratory syndrome coro ···················· ···················· ···················· ···················· ···················· ····················
3987 MW276400.1 |Severe acute respiratory syndrome coro ···················· ···················· ···················· ···················· ···················· ····················
3988 MW276401.1 |Severe acute respiratory syndrome coro ···················· ···················· ···················· ···················· ···················· ····················
3989 MW276402.1 |Severe acute respiratory syndrome coro ···················· ·A·················· ···················· ···················· ···················· ····················
3990 MW276403.1 |Severe acute respiratory syndrome coro ···················· ·A·················· ···················· ···················· ···················· ····················
3991 MW276404.1 |Severe acute respiratory syndrome coro ···················· ·A·················· ···················· ···················· ···················· ····················
3992 MW276406.1 |Severe acute respiratory syndrome coro ···················· ···················· ···················· ···················· ···················· ····················
3993 MW276407.1 |Severe acute respiratory syndrome coro ···················· ·A·················· ···················· ···················· ···················· ····················
3994 MW276408.1 |Severe acute respiratory syndrome coro ···················· ···················· ···················· ···················· ···················· ····················
3995 MW276409.1 |Severe acute respiratory syndrome coro ···················· ·A·················· ···················· ···················· ···················· ····················
3996 MW276410.1 |Severe acute respiratory syndrome coro ···················· ·A·················· ···················· ···················· ···················· ····················
3997 MW276411.1 |Severe acute respiratory syndrome coro ···················· ·A·················· ···················· ···················· ···················· ····················
3998 MW276413.1 |Severe acute respiratory syndrome coro ···················· ·A·················· ···················· ···················· ···················· ····················
3999 MW276415.1 |Severe acute respiratory syndrome coro ···················· ·A·················· ···················· ···················· ···················· ····················
4000 MW276416.1 |Severe acute respiratory syndrome coro ···················· ·A·················· ···················· ···················· ···················· ····················
4001 MW276417.1 |Severe acute respiratory syndrome coro ···················· ·A·················· ···················· ···················· ···················· ····················
4002 MW276418.1 |Severe acute respiratory syndrome coro ···················· ·A·················· ···················· ···················· ···················· ····················
4003 MW276419.1 |Severe acute respiratory syndrome coro ···················· ·A·················· ···················· ···················· ···················· ····················
4004 MW276420.1 |Severe acute respiratory syndrome coro ···················· ·A·················· ···················· ···················· ···················· ····················
4005 MW276421.1 |Severe acute respiratory syndrome coro ···················· ·A·················· ···················· ···················· ···················· ····················
4006 MW276422.1 |Severe acute respiratory syndrome coro ···················· ·A·················· ···················· ···················· ···················· ····················
4007 MW276423.1 |Severe acute respiratory syndrome coro ···················· ·A·················· ···················· ···················· ···················· ····················
4008 MW276424.1 |Severe acute respiratory syndrome coro ···················· ·A·················· ···················· ···················· ···················· ····················
4009 MW276425.1 |Severe acute respiratory syndrome coro ···················· ·A·················· ···················· ···················· ···················· ····················
4010 MW276426.1 |Severe acute respiratory syndrome coro ···················· ·A·················· ···················· ···················· ···················· ····················
4011 MW276427.1 |Severe acute respiratory syndrome coro ···················· ·A·················· ···················· ···················· ···················· ····················
4012 MW276428.1 |Severe acute respiratory syndrome coro ···················· ·A·················· ···················· ···················· ···················· ····················
4013 MW276429.1 |Severe acute respiratory syndrome coro ···················· ·A·················· ···················· ···················· ···················· ····················
4014 MW276430.1 |Severe acute respiratory syndrome coro ···················· ···················· ···················· ···················· ···················· ····················
4015 MW276431.1 |Severe acute respiratory syndrome coro ···················· ·A·················· ···················· ···················· ···················· ····················
4016 MW276432.1 |Severe acute respiratory syndrome coro ···················· ·A·················· ···················· ···················· ···················· ····················
4017 MW276433.1 |Severe acute respiratory syndrome coro ···················· ·A·················· ···················· ···················· ···················· ····················
4018 MW276434.1 |Severe acute respiratory syndrome coro ···················· ·A·················· ···················· ···················· ···················· ····················
4020 MW276437.1 |Severe acute respiratory syndrome coro ···················· ·A·················· ···················· ···················· ···················· ····················
4021 MW276438.1 |Severe acute respiratory syndrome coro ···················· ···················· ···················· ···················· ···················· ····················
4022 MW276440.1 |Severe acute respiratory syndrome coro ···················· ·A·················· ···················· ···················· ···················· ····················
4023 MW276441.1 |Severe acute respiratory syndrome coro ···················· ·A·················· ···················· ···················· ···················· ····················
4024 MW276442.1 |Severe acute respiratory syndrome coro ···················· ·A·················· ···················· ···················· ···················· ····················
4025 MW276443.1 |Severe acute respiratory syndrome coro ···················· ·A·················· ···················· ···················· ···················· ····················
4026 MW276444.1 |Severe acute respiratory syndrome coro ···················· ·A·················· ···················· ···················· ···················· ····················
4027 MW276445.1 |Severe acute respiratory syndrome coro ···················· ·A·················· ···················· ···················· ···················· ····················
4028 MW276446.1 |Severe acute respiratory syndrome coro ···················· ·A·················· ···················· ···················· ···················· ····················
4029 MW276447.1 |Severe acute respiratory syndrome coro ···················· ·A·················· ···················· ···················· ···················· ····················
4030 MW276448.1 |Severe acute respiratory syndrome coro ···················· ···················· ···················· ···················· ···················· ····················
4031 MW276449.1 |Severe acute respiratory syndrome coro ···················· ···················· ···················· ···················· ···················· ····················
4032 MW276450.1 |Severe acute respiratory syndrome coro ···················· ···················· ···················· ···················· ···················· ····················
4033 MW276451.1 |Severe acute respiratory syndrome coro ···················· ···················· ···················· ···················· ···················· ····················
4034 MW276452.1 |Severe acute respiratory syndrome coro ···················· ···················· ···················· ···················· ···················· ····················
4035 MW276453.1 |Severe acute respiratory syndrome coro ···················· ···················· ···················· ···················· ···················· ····················
4036 MW276454.1 |Severe acute respiratory syndrome coro ···················· ···················· ···················· ···················· ···················· ····················
4037 MW276455.1 |Severe acute respiratory syndrome coro ···················· ···················· ···················· ···················· ···················· ····················
4038 MW276456.1 |Severe acute respiratory syndrome coro ···················· ···················· ···················· ···················· ···················· ····················
4039 MW276458.1 |Severe acute respiratory syndrome coro ···················· ···················· ···················· ···················· ···················· ····················
4040 MW276463.1 |Severe acute respiratory syndrome coro ···················· ···················· ···················· ···················· ···················· ····················
4041 MW276464.1 |Severe acute respiratory syndrome coro ···················· ·A·················· ···················· ···················· ···················· ····················
4042 MW276465.1 |Severe acute respiratory syndrome coro ···················· ···················· ···················· ···················· ···················· ····················
4043 MW276466.1 |Severe acute respiratory syndrome coro ···················· ···················· ···················· ···················· ···················· ····················
4044 MW276467.1 |Severe acute respiratory syndrome coro ···················· ···················· ···················· ···················· ···················· ····················
4045 MW276468.1 |Severe acute respiratory syndrome coro ···················· ···················· ···················· ···················· ···················· ····················
4046 MW276469.1 |Severe acute respiratory syndrome coro ···················· ···················· ···················· ···················· ···················· ····················
4047 MW276470.1 |Severe acute respiratory syndrome coro ···················· ···················· ···················· ···················· ···················· ····················
4048 MW276471.1 |Severe acute respiratory syndrome coro ···················· ·A·················· ···················· ···················· ···················· ····················

```
4049 MW276473.1 |Severe acute respiratory syndrome coro   ····················  ····················  ····················  ····················  ····················  ····················
4050 MW276474.1 |Severe acute respiratory syndrome coro   ····················  ····················  ····················  ····················  ····················  ····················
4051 MW276475.1 |Severe acute respiratory syndrome coro   ····················  ·A··················  ····················  ····················  ····················  ····················
4052 MW276476.1 |Severe acute respiratory syndrome coro   ····················  ·A··················  ····················  ····················  ····················  ····················
4053 MW276477.1 |Severe acute respiratory syndrome coro   ····················  ····················  ····················  ····················  ····················  ····················
4054 MW276478.1 |Severe acute respiratory syndrome coro   ····················  ····················  ····················  ····················  ····················  ····················
4055 MW276479.1 |Severe acute respiratory syndrome coro   ····················  ····················  ····················  ····················  ····················  ····················
4056 MW276480.1 |Severe acute respiratory syndrome coro   ····················  ····················  ····················  ····················  ····················  ····················
4057 MW276481.1 |Severe acute respiratory syndrome coro   ····················  ····················  ····················  ····················  ····················  ····················
4058 MW276482.1 |Severe acute respiratory syndrome coro   ····················  ····················  ····················  ····················  ····················  ····················
4059 MW276483.1 |Severe acute respiratory syndrome coro   ····················  ····················  ····················  ····················  ····················  ····················
4060 MW276484.1 |Severe acute respiratory syndrome coro   ····················  ····················  ····················  ····················  ····················  ····················
4061 MW276485.1 |Severe acute respiratory syndrome coro   ····················  ·A··················  ····················  ····················  ····················  ····················
4062 MW276486.1 |Severe acute respiratory syndrome coro   ····················  ····················  ····················  ····················  ·············T······  ····················
4063 MW276487.1 |Severe acute respiratory syndrome coro   ····················  ····················  ····················  ····················  ····················  ····················
4064 MW276488.1 |Severe acute respiratory syndrome coro   ····················  ····················  ····················  ····················  ····················  ····················
4065 MW276489.1 |Severe acute respiratory syndrome coro   ····················  ····················  ····················  ····················  ····················  ····················
4066 MW276490.1 |Severe acute respiratory syndrome coro   ····················  ·A··················  ····················  ····················  ····················  ····················
4067 MW276491.1 |Severe acute respiratory syndrome coro   ····················  ·A··················  ····················  ····················  ····················  ····················
4068 MW276492.1 |Severe acute respiratory syndrome coro   ····················  ·A··················  ····················  ····················  ····················  ····················
4069 MW276493.1 |Severe acute respiratory syndrome coro   ····················  ····················  ··T·················  ····················  ····················  ····················
4070 MW276494.1 |Severe acute respiratory syndrome coro   ····················  ·A··················  ····················  ····················  ····················  ····················
4071 MW276495.1 |Severe acute respiratory syndrome coro   ····················  ····················  ····················  ····················  ····················  ····················
4072 MW276496.1 |Severe acute respiratory syndrome coro   ····················  ····················  ····················  ····················  ····················  ····················
4073 MW276498.1 |Severe acute respiratory syndrome coro   ····················  ····················  ····················  ····················  ····················  ····················
4074 MW276499.1 |Severe acute respiratory syndrome coro   ····················  ····················  ····················  ····················  ····················  ····················
4075 MW276504.1 |Severe acute respiratory syndrome coro   ····················  ····················  ····················  ····················  ····················  ····················
4076 MW276505.1 |Severe acute respiratory syndrome coro   ····················  ····················  ····················  ····················  ····················  ····················
4077 MW276506.1 |Severe acute respiratory syndrome coro   ····················  ····················  ····················  ····················  ····················  ····················
4078 MW276507.1 |Severe acute respiratory syndrome coro   ····················  ····················  ····················  ····················  ····················  ····················
4079 MW276508.1 |Severe acute respiratory syndrome coro   ····················  ····················  ····················  ····················  ····················  ····················
4080 MW276509.1 |Severe acute respiratory syndrome coro   ····················  ····················  ····················  ····················  ····················  ····················
4081 MW276510.1 |Severe acute respiratory syndrome coro   ····················  ····················  ····················  ····················  ····················  ····················
4082 MW276511.1 |Severe acute respiratory syndrome coro   ····················  ····················  ····················  ····················  ····················  ····················
4083 MW276512.1 |Severe acute respiratory syndrome coro   ····················  ····················  ····················  ····················  ····················  ····················
4084 MW276514.1 |Severe acute respiratory syndrome coro   ····················  ····················  ····················  ····················  ····················  ····················
4086 MW276519.1 |Severe acute respiratory syndrome coro   ····················  ····················  ····················  ····················  ····················  ····················
4088 MW276521.1 |Severe acute respiratory syndrome coro   ····················  ····················  ····················  ····················  ····················  ····················
4091 MW276524.1 |Severe acute respiratory syndrome coro   ····················  ····················  ····················  ····················  ····················  ····················
4093 MW276527.1 |Severe acute respiratory syndrome coro   ····················  ····················  ····················  ····················  ····················  ····················
4094 MW276528.1 |Severe acute respiratory syndrome coro   ····················  ····················  ····················  ····················  ····················  ····················
4095 MW276529.1 |Severe acute respiratory syndrome coro   ····················  ····················  ····················  ····················  ····················  ····················
4096 MW276533.1 |Severe acute respiratory syndrome coro   ····················  ····················  ····················  ····················  ····················  ····················
4097 MW276536.1 |Severe acute respiratory syndrome coro   ····················  ····················  ····················  ····················  NNN·················  ····················   1
4098 MW276537.1 |Severe acute respiratory syndrome coro   ····················  ·A··················  ····················  ····················  ····················  ····················
4099 MW276538.1 |Severe acute respiratory syndrome coro   ····················  ····················  ····················  ····················  ····················  ····················
4100 MW276539.1 |Severe acute respiratory syndrome coro   ····················  ····················  ····················  ····················  ····················  ····················
4101 MW276540.1 |Severe acute respiratory syndrome coro   ····················  ····················  ····················  ····················  ····················  ····················
4102 MW276542.1 |Severe acute respiratory syndrome coro   ····················  ····················  ····················  ····················  ····················  ····················
4103 MW276543.1 |Severe acute respiratory syndrome coro   ····················  ····················  ····················  ····················  ····················  ····················
4104 MW276544.1 |Severe acute respiratory syndrome coro   ····················  ····················  ····················  ····················  ····················  ····················
4105 MW276545.1 |Severe acute respiratory syndrome coro   ····················  ····················  ····················  ····················  ····················  ····················
4106 MW276546.1 |Severe acute respiratory syndrome coro   ····················  ····················  ····················  ····················  ····················  ····················
4109 MW276549.1 |Severe acute respiratory syndrome coro   ····················  ·A··················  ····················  ····················  ····················  ····················
4110 MW276550.1 |Severe acute respiratory syndrome coro   ····················  ·A··················  ····················  ····················  ····················  ····················
4111 MW276551.1 |Severe acute respiratory syndrome coro   ····················  ····················  ····················  ····················  ····················  ····················
4112 MW276552.1 |Severe acute respiratory syndrome coro   ····················  ·A··················  ····················  ····················  ····················  ····················
4113 MW276553.1 |Severe acute respiratory syndrome coro   ····················  ····················  ····················  ····················  ····················  ····················
4114 MW276554.1 |Severe acute respiratory syndrome coro   ····················  ····················  ····················  ····················  ····················  ····················
4115 MW276555.1 |Severe acute respiratory syndrome coro   ····················  ····················  ····················  ····················  ····················  ····················
4116 MW276556.1 |Severe acute respiratory syndrome coro   ····················  ·A··················  ····················  ····················  ····················  ····················
4117 MW276557.1 |Severe acute respiratory syndrome coro   ····················  ·A··················  ····················  ····················  ····················  ····················
4118 MW276558.1 |Severe acute respiratory syndrome coro   ····················  ·A··················  ····················  ····················  ····················  ····················
4119 MW276559.1 |Severe acute respiratory syndrome coro   ····················  ····················  ····················  ····················  ····················  ····················
4120 MW276560.1 |Severe acute respiratory syndrome coro   ····················  ·A··················  ····················  ····················  ····················  ····················
4121 MW276561.1 |Severe acute respiratory syndrome coro   ····················  ·A··················  ····················  ····················  ····················  ····················
4122 MW276562.1 |Severe acute respiratory syndrome coro   ····················  ·A··················  ····················  ····················  ····················  ····················
4123 MW276563.1 |Severe acute respiratory syndrome coro   ····················  ·A··················  ····················  ····················  ····················  ····················
4124 MW276564.1 |Severe acute respiratory syndrome coro   ····················  ·A··················  ····················  ····················  ····················  ····················
4126 MW276566.1 |Severe acute respiratory syndrome coro   ····················  ·A··················  ····················  ····················  ····················  ····················
4127 MW276567.1 |Severe acute respiratory syndrome coro   ····················  ·A··················  ····················  ····················  ····················  ····················
4128 MW276568.1 |Severe acute respiratory syndrome coro   ····················  ·A··················  ····················  ····················  ····················  ····················
4129 MW276569.1 |Severe acute respiratory syndrome coro   ····················  ·A··················  ····················  ····················  ····················  ····················
4130 MW276570.1 |Severe acute respiratory syndrome coro   ····················  ·A··················  ····················  ····················  ····················  ····················
4131 MW276571.1 |Severe acute respiratory syndrome coro   ····················  ·A··················  ····················  ····················  ····················  ····················
4132 MW276572.1 |Severe acute respiratory syndrome coro   ····················  ····················  ····················  ····················  ····················  ····················
4133 MW276573.1 |Severe acute respiratory syndrome coro   ····················  ····················  ····················  ····················  ····················  ····················
4134 MW276574.1 |Severe acute respiratory syndrome coro   ····················  ·A··················  ····················  ····················  ····················  ····················
```

4135	MW276575.1	|Severe acute respiratory syndrome coro	....	.A..................	....	....	....	....
4136	MW276577.1	|Severe acute respiratory syndrome coro	....	....	....	....	....	....
4137	MW276578.1	|Severe acute respiratory syndrome coro	....	....	....	....	....	....
4138	MW276579.1	|Severe acute respiratory syndrome coro	....	....	....	....	....	....
4139	MW276581.1	|Severe acute respiratory syndrome coro	....	.A..................	....	....	....	....
4140	MW276582.1	|Severe acute respiratory syndrome coro	....	.A..................	....	....	....	....
4141	MW276583.1	|Severe acute respiratory syndrome coro	....	.A..................	....	....	....	....
4142	MW276584.1	|Severe acute respiratory syndrome coro	....	.A..................	....	....	....	....
4143	MW276585.1	|Severe acute respiratory syndrome coro	....	.A..................	....	....	....	....
4144	MW276586.1	|Severe acute respiratory syndrome coro	....	.A..................	....	....	....	....
4145	MW276587.1	|Severe acute respiratory syndrome coro	....	.A..................	....	....	....	....
4147	MW276589.1	|Severe acute respiratory syndrome coro	....	.A..................	....	....	....	....
4149	MW276592.1	|Severe acute respiratory syndrome coro	....	.A..................	....	....	....	....
4150	MW276593.1	|Severe acute respiratory syndrome coro	....	.A..................	....	....	....	....
4151	MW276595.1	|Severe acute respiratory syndrome coro	....	.A..................	....	....	....	....
4152	MW276596.1	|Severe acute respiratory syndrome coro	....	.A..................	....	....	....	....
4153	MW276597.1	|Severe acute respiratory syndrome coro	....	.A..................	....	....	....	....
4154	MW276599.1	|Severe acute respiratory syndrome coro	....	.A..................	....	....	....	....
4155	MW276600.1	|Severe acute respiratory syndrome coro	....	....	....	....	....	....
4156	MW276601.1	|Severe acute respiratory syndrome coro	....	.A..................	....	....	....	....
4157	MW276603.1	|Severe acute respiratory syndrome coro	....	.A..................	....	....	....	....
4159	MW276606.1	|Severe acute respiratory syndrome coro	....	....	....	....	....	....
4160	MW276607.1	|Severe acute respiratory syndrome coro	....	....	....	....	....	....
4161	MW276608.1	|Severe acute respiratory syndrome coro	....	....	....	....	....	....
4162	MW276609.1	|Severe acute respiratory syndrome coro	....	....	....	....	....	....
4163	MW276610.1	|Severe acute respiratory syndrome coro	....	.............A......	....	....	....	....
4164	MW276612.1	|Severe acute respiratory syndrome coro	....	.A..................	....	....	....	....
4165	MW276613.1	|Severe acute respiratory syndrome coro	....	....	....	....	....	....
4166	MW276614.1	|Severe acute respiratory syndrome coro	....	.A..................	....	....	....	....
4167	MW276615.1	|Severe acute respiratory syndrome coro	....	.A..................	....	....	....	....
4168	MW276616.1	|Severe acute respiratory syndrome coro	....	.A..................	....	....	....	....
4169	MW276617.1	|Severe acute respiratory syndrome coro	....	....	....	....	....	....
4170	MW276618.1	|Severe acute respiratory syndrome coro	....	.A..................	....	....	....	....
4171	MW276621.1	|Severe acute respiratory syndrome coro	....	.A..................	....	....	....	....
4172	MW276622.1	|Severe acute respiratory syndrome coro	....	....	....	....	....	....
4173	MW276623.1	|Severe acute respiratory syndrome coro	....	.A..................	....	....	....	....
4174	MW276625.1	|Severe acute respiratory syndrome coro	....	....	...........T........	....	....	....
4175	MW276626.1	|Severe acute respiratory syndrome coro	....	....	....	....	....	....
4176	MW276627.1	|Severe acute respiratory syndrome coro	....	.A..................	....	....	....	....
4177	MW276629.1	|Severe acute respiratory syndrome coro	....	.A..................	....	....	....	....
4178	MW276630.1	|Severe acute respiratory syndrome coro	....	.A..................	....	....	....	....
4179	MW276631.1	|Severe acute respiratory syndrome coro	....	.A..................	....	....	....	....
4181	MW276634.1	|Severe acute respiratory syndrome coro	....	.A..................	....	....	....	....
4182	MW276635.1	|Severe acute respiratory syndrome coro	....	.A..................	....	....	....	....
4183	MW276636.1	|Severe acute respiratory syndrome coro	...T................	.A..................	....	....	....	....
4185	MW276638.1	|Severe acute respiratory syndrome coro	....	....	....	....	....	....
4186	MW276639.1	|Severe acute respiratory syndrome coro	....	.A..................	....	....	....	....
4187	MW276641.1	|Severe acute respiratory syndrome coro	....	.A..................	....	....	....	....
4189	MW276644.1	|Severe acute respiratory syndrome coro	....	....	....	....	....	....
4190	MW276645.1	|Severe acute respiratory syndrome coro	....	....	....	....	....	....
4191	MW276646.1	|Severe acute respiratory syndrome coro	....	....	....	....	....	....
4192	MW276647.1	|Severe acute respiratory syndrome coro	....	....	....	....	....	....
4193	MW276648.1	|Severe acute respiratory syndrome coro	....	....	....	....	....	....
4194	MW276649.1	|Severe acute respiratory syndrome coro	....	.A..................	....	....	....	....
4195	MW276651.1	|Severe acute respiratory syndrome coro	....	....	....	....	....	....
4196	MW276652.1	|Severe acute respiratory syndrome coro	....	....	....	....	....	....
4197	MW276653.1	|Severe acute respiratory syndrome coro	....	....	....	....	....	....
4198	MW276654.1	|Severe acute respiratory syndrome coro	....	....	....	....	....	....
4199	MW276656.1	|Severe acute respiratory syndrome coro	....	.A..................	....	....	....	....
4200	MW276657.1	|Severe acute respiratory syndrome coro	....	....	....	....	....	....
4201	MW276658.1	|Severe acute respiratory syndrome coro	...T................	.A..................	....	....	....	....
4202	MW276659.1	|Severe acute respiratory syndrome coro	....	....	....	....	....	....
4203	MW276661.1	|Severe acute respiratory syndrome coro	....	....	....	....	....	....
4204	MW276663.1	|Severe acute respiratory syndrome coro	....	.A..................	....	....	....	....
4206	MW276667.1	|Severe acute respiratory syndrome coro	....	....	....	....	....	....
4207	MW276669.1	|Severe acute respiratory syndrome coro	....	.A..................	....	....	....	....
4209	MW276671.1	|Severe acute respiratory syndrome coro	....	....	....	....	....	....
4210	MW276672.1	|Severe acute respiratory syndrome coro	....	.A..................	....	....	....	....
4211	MW276675.1	|Severe acute respiratory syndrome coro	....	....	....	....	....	....
4212	MW276679.1	|Severe acute respiratory syndrome coro	....	....	....	....	....	....
4213	MW276680.1	|Severe acute respiratory syndrome coro	....	....	....	....	....	....
4214	MW276681.1	|Severe acute respiratory syndrome coro	....	....	....	....	....	....
4215	MW276684.1	|Severe acute respiratory syndrome coro	....	....	....	....	....	....
4216	MW276686.1	|Severe acute respiratory syndrome coro	....	....	....	....	....	....
4217	MW276687.1	|Severe acute respiratory syndrome coro	....	....	....	....	....	....
4218	MW276688.1	|Severe acute respiratory syndrome coro	....	....	....	....	....	....
4219	MW276689.1	|Severe acute respiratory syndrome coro	....	....	....	....	....	....
4220	MW276690.1	|Severe acute respiratory syndrome coro	....	....	....	....	....	....

```
4221 MW276691.1 |Severe acute respiratory syndrome coro    ....................  ....................  ....................  ....................  ....................  ....................
4222 MW276692.1 |Severe acute respiratory syndrome coro    ....................  ....................  ....................  ....................  ....................  ....................
4223 MW276693.1 |Severe acute respiratory syndrome coro    ....................  ....................  ....................  ....................  ....................  ....................
4224 MW276695.1 |Severe acute respiratory syndrome coro    ....................  ....................  ....................  ....................  ....................  ....................
4225 MW276696.1 |Severe acute respiratory syndrome coro    ....................  ....................  ....................  ....................  ....................  ....................
4226 MW276697.1 |Severe acute respiratory syndrome coro    ....................  ....................  ....................  ....................  ....................  ....................
4227 MW276699.1 |Severe acute respiratory syndrome coro    ....................  ....................  ....................  ....................  ....................  ....................
4228 MW276700.1 |Severe acute respiratory syndrome coro    ....................  ....................  ....................  ....................  ....................  ....................
4229 MW276702.1 |Severe acute respiratory syndrome coro    ....................  ....................  ....................  ....................  ....................  ....................
4230 MW276703.1 |Severe acute respiratory syndrome coro    ....................  ....................  ....................  ....................  ....................  ....................
4231 MW276704.1 |Severe acute respiratory syndrome coro    ....................  ....................  ....................  ....................  ....................  ....................
4232 MW276705.1 |Severe acute respiratory syndrome coro    ....................  ....................  ....................  ....................  ....................  ....................
4233 MW276707.1 |Severe acute respiratory syndrome coro    ....................  ....................  ....................  ....................  ....................  ....................
4234 MW276708.1 |Severe acute respiratory syndrome coro    ....................  ....................  ....................  ....................  ....................  ....................
4235 MW276709.1 |Severe acute respiratory syndrome coro    ....................  ....................  ....................  ....................  ....................  ....................
4236 MW276710.1 |Severe acute respiratory syndrome coro    ....................  ....................  ....................  ....................  ....................  ....................
4237 MW276711.1 |Severe acute respiratory syndrome coro    ....................  ....................  ....................  ....................  ....................  ....................
4238 MW276712.1 |Severe acute respiratory syndrome coro    ....................  ....................  ....................  ....................  ....................  ....................
4239 MW276713.1 |Severe acute respiratory syndrome coro    ....................  ....................  ....................  ....................  ....................  ....................
4240 MW276714.1 |Severe acute respiratory syndrome coro    ....................  ....................  ....................  ....................  ....................  ....................
4241 MW276717.1 |Severe acute respiratory syndrome coro    ....................  ....................  ....................  ....................  ....................  ....................
4242 MW276718.1 |Severe acute respiratory syndrome coro    ....................  ....................  ....................  ....................  ....................  ....................
4243 MW276720.1 |Severe acute respiratory syndrome coro    ....................  ....................  ....................  ....................  ....................  ....................
4244 MW276721.1 |Severe acute respiratory syndrome coro    ....................  ....................  ....................  ....................  ....................  ....................
4245 MW276723.1 |Severe acute respiratory syndrome coro    ....................  ....................  ....................  ....................  ....................  ....................
4246 MW276724.1 |Severe acute respiratory syndrome coro    ....................  ....................  ....................  ....................  ....................  ....................
4247 MW276725.1 |Severe acute respiratory syndrome coro    ....................  ....................  ....................  ....................  ....................  ....................
4248 MW276726.1 |Severe acute respiratory syndrome coro    ....................  ....................  ....................  ....................  ....................  ....................
4249 MW276727.1 |Severe acute respiratory syndrome coro    ....................  ....................  ....................  ....................  ....................  ....................
4250 MW276728.1 |Severe acute respiratory syndrome coro    ....................  ....................  ....................  ....................  ....................  ....................
4251 MW276731.1 |Severe acute respiratory syndrome coro    ....................  ....................  ....................  ....................  ....................  ....................
4252 MW276732.1 |Severe acute respiratory syndrome coro    ....................  ....................  ....................  ....................  ....................  ....................
4253 MW276733.1 |Severe acute respiratory syndrome coro    ....................  ....................  ....................  ....................  ....................  ....................
4254 MW276734.1 |Severe acute respiratory syndrome coro    ....................  ....................  ....................  ....................  ....................  ....................
4255 MW276735.1 |Severe acute respiratory syndrome coro    ....................  ....................  ....................  ....................  ....................  ....................
4256 MW276736.1 |Severe acute respiratory syndrome coro    ....................  ....................  ....................  ....................  ....................  ....................
4257 MW276737.1 |Severe acute respiratory syndrome coro    ....................  ....................  ....................  ....................  ....................  ....................
4258 MW276738.1 |Severe acute respiratory syndrome coro    ....................  ....................  ....................  ....................  ....................  ....................
4259 MW276740.1 |Severe acute respiratory syndrome coro    ....................  ....................  ....................  ....................  ....................  ....................
4260 MW276741.1 |Severe acute respiratory syndrome coro    ....................  ....................  ....................  ....................  ....................  ....................
4261 MW276742.1 |Severe acute respiratory syndrome coro    ....................  ....................  ....................  ....................  ....................  ....................
4262 MW276743.1 |Severe acute respiratory syndrome coro    ....................  ....................  ....................  ....................  ....................  ....................
4263 MW276744.1 |Severe acute respiratory syndrome coro    ....................  ....................  ....................  ....................  ....................  ....................
4264 MW276745.1 |Severe acute respiratory syndrome coro    ....................  ....................  ....................  ....................  ....................  ....................
4266 MW276748.1 |Severe acute respiratory syndrome coro    ....................  ....................  ....................  ....................  ....................  ....................
4267 MW276749.1 |Severe acute respiratory syndrome coro    ....................  ....................  ....................  ....................  ....................  ....................
4268 MW276750.1 |Severe acute respiratory syndrome coro    ....................  ....................  ....................  ....................  ....................  ....................
4269 MW276751.1 |Severe acute respiratory syndrome coro    ....................  ....................  ....................  ....................  ....................  ....................
4270 MW276752.1 |Severe acute respiratory syndrome coro    ....................  ......A.............  ....................  ....................  ....................  ....................
4271 MW276753.1 |Severe acute respiratory syndrome coro    ....................  ....................  ....................  ....................  ....................  ....................
4272 MW276754.1 |Severe acute respiratory syndrome coro    ....................  ....................  ....................  ....................  ....................  ....................
4273 MW276755.1 |Severe acute respiratory syndrome coro    ....................  ....................  ....................  ....................  ....................  ....................
4274 MW276756.1 |Severe acute respiratory syndrome coro    ....................  ....................  ....................  ....................  ....................  ....................
4277 MW276760.1 |Severe acute respiratory syndrome coro    ....................  ....................  ....................  ....................  ....................  ....................
4278 MW276762.1 |Severe acute respiratory syndrome coro    ....................  ....................  ....................  ....................  ....................  ....................
4279 MW276763.1 |Severe acute respiratory syndrome coro    ....................  ....................  ....................  ....................  ....................  ....................
4280 MW276764.1 |Severe acute respiratory syndrome coro    ....................  ....................  ....................  ....................  ....................  ....................
4281 MW276766.1 |Severe acute respiratory syndrome coro    ....................  ....................  ....................  ....................  ....................  ....................
4282 MW276767.1 |Severe acute respiratory syndrome coro    ....................  ....................  ....................  ....................  ....................  ....................
4283 MW276768.1 |Severe acute respiratory syndrome coro    ....................  ....................  ....................  ....................  ....................  ....................
4284 MW276769.1 |Severe acute respiratory syndrome coro    ....................  ....................  ....................  ....................  ....................  ....................
4285 MW276770.1 |Severe acute respiratory syndrome coro    ....................  ....................  ....................  ....................  ....................  ....................
4286 MW276772.1 |Severe acute respiratory syndrome coro    ....................  ....................  ....................  ....................  ....................  ....................
4287 MW276773.1 |Severe acute respiratory syndrome coro    ....................  ....................  ....................  ....................  ....................  ....................
4288 MW276774.1 |Severe acute respiratory syndrome coro    ....................  ....................  ....................  ....................  ....................  ....................
4289 MW276775.1 |Severe acute respiratory syndrome coro    ....................  ....................  ....................  ....................  ....................  ....................
4290 MW276776.1 |Severe acute respiratory syndrome coro    ....................  ....................  ....................  ....................  ....................  ....................
4291 MW276777.1 |Severe acute respiratory syndrome coro    ....................  ....................  ....................  ....................  ....................  ....................
4292 MW276778.1 |Severe acute respiratory syndrome coro    ....................  ....................  ....................  ....................  ....................  ....................
4293 MW276779.1 |Severe acute respiratory syndrome coro    ....................  ....................  ....................  ....................  ....................  ....................
4294 MW276780.1 |Severe acute respiratory syndrome coro    ....................  ....................  ....................  ....................  ....................  ....................
4296 MW276782.1 |Severe acute respiratory syndrome coro    ....................  ....................  ....................  ....................  ....................  ....................
4297 MW276783.1 |Severe acute respiratory syndrome coro    ....................  ....................  ....................  ....................  ....................  ....................
4298 MW276784.1 |Severe acute respiratory syndrome coro    ....................  ....................  ....................  ....................  ....................  ....................
4299 MW276785.1 |Severe acute respiratory syndrome coro    ....................  ....................  ....................  ....................  ....................  ....................
4300 MW276786.1 |Severe acute respiratory syndrome coro    ....................  ....................  ....................  ....................  ....................  ....................
4301 MW276787.1 |Severe acute respiratory syndrome coro    ....................  ....................  ....................  ....................  ....................  ....................
4302 MW276788.1 |Severe acute respiratory syndrome coro    ....................  ....................  ....................  ....................  ....................  ....................
```

```
4303 MW276789.1 |Severe acute respiratory syndrome coro    ....................  ....................  ....................  ....................  ....................  ....................
4304 MW276791.1 |Severe acute respiratory syndrome coro    ....................  ....................  ....................  ....................  ....................  ....................
4305 MW276792.1 |Severe acute respiratory syndrome coro    ....................  ....................  ....................  ....................  ....................  ....................
4306 MW276793.1 |Severe acute respiratory syndrome coro    ....................  ....................  ....................  ....................  ....................  ....................
4307 MW276794.1 |Severe acute respiratory syndrome coro    ....................  ....................  ....................  ....................  ....................  ....................
4308 MW276795.1 |Severe acute respiratory syndrome coro    ....................  ....................  ....................  ....................  ....................  ....................
4309 MW276796.1 |Severe acute respiratory syndrome coro    ....................  ....................  ....................  ....................  ....................  ....................
4310 MW276797.1 |Severe acute respiratory syndrome coro    ....................  ....................  ....................  ....................  ....................  ....................
4311 MW276798.1 |Severe acute respiratory syndrome coro    ....................  ....................  ....................  ....................  ....................  ....................
4312 MW276799.1 |Severe acute respiratory syndrome coro    ....................  ....................  ....................  ....................  ....................  ....................
4313 MW276800.1 |Severe acute respiratory syndrome coro    ....................  ....................  ....................  ....................  ....................  ....................
4314 MW276802.1 |Severe acute respiratory syndrome coro    ....................  ....................  ....................  ....................  ....................  ....................
4315 MW276804.1 |Severe acute respiratory syndrome coro    ....................  ....................  ....................  ....................  ....................  ....................
4316 MW276805.1 |Severe acute respiratory syndrome coro    ....................  ....................  ....................  ....................  ....................  ....................
4317 MW276806.1 |Severe acute respiratory syndrome coro    ....................  ....................  ....................  ....................  ....................  ....................
4318 MW276807.1 |Severe acute respiratory syndrome coro    ....................  ....................  ....................  ....................  ....................  ....................
4319 MW276808.1 |Severe acute respiratory syndrome coro    ....................  ....................  ....................  ....................  ....................  ....................
4320 MW276809.1 |Severe acute respiratory syndrome coro    ....................  ....................  ....................  ....................  ....................  ....................
4321 MW276811.1 |Severe acute respiratory syndrome coro    ....................  ....................  ....................  ....................  ....................  ....................
4322 MW276812.1 |Severe acute respiratory syndrome coro    ....................  ....................  ....................  ....................  ....................  ....................
4323 MW276813.1 |Severe acute respiratory syndrome coro    ....................  ....................  ....................  ....................  ....................  ....................
4324 MW276814.1 |Severe acute respiratory syndrome coro    ....................  ....................  ....................  ....................  ....................  ....................
4325 MW276816.1 |Severe acute respiratory syndrome coro    ....................  ....................  ....................  ....................  ....................  ....................
4326 MW276817.1 |Severe acute respiratory syndrome coro    ....................  ....................  ....................  ....................  ....................  ....................
4327 MW276818.1 |Severe acute respiratory syndrome coro    ....................  ....................  ....................  ....................  ....................  ....................
4328 MW276820.1 |Severe acute respiratory syndrome coro    ....................  ....................  ....................  ....................  ....................  ....................
4329 MW276821.1 |Severe acute respiratory syndrome coro    ....................  ....................  ....................  ....................  ....................  ....................
4330 MW276822.1 |Severe acute respiratory syndrome coro    ....................  ....................  ....................  ....................  ....................  ....................
4331 MW276823.1 |Severe acute respiratory syndrome coro    ....................  ....................  ....................  ....................  ....................  ....................
4332 MW276824.1 |Severe acute respiratory syndrome coro    ....................  ....................  ....................  ....................  ....................  ....................
4333 MW276826.1 |Severe acute respiratory syndrome coro    ....................  ....................  ....................  ....................  ....................  ....................
4334 MW276827.1 |Severe acute respiratory syndrome coro    ....................  ....................  ....................  ....................  ....................  ....................
4335 MW276828.1 |Severe acute respiratory syndrome coro    ....................  ....................  ....................  ....................  ....................  ....................
4336 MW276829.1 |Severe acute respiratory syndrome coro    ....................  ....................  ....................  ....................  ....................  ....................
4337 MW276830.1 |Severe acute respiratory syndrome coro    ....................  ....................  ....................  ....................  ....................  ....................
4338 MW276831.1 |Severe acute respiratory syndrome coro    ....................  ....................  ....................  ....................  ....................  ....................
4339 MW276832.1 |Severe acute respiratory syndrome coro    ....................  ....................  ....................  ....................  ....................  ....................
4340 MW276833.1 |Severe acute respiratory syndrome coro    ....................  ....................  ....................  ....................  ....................  ....................
4341 MW276834.1 |Severe acute respiratory syndrome coro    ....................  ....................  ....................  ....................  ....................  ....................
4342 MW276835.1 |Severe acute respiratory syndrome coro    ....................  ....................  ....................  ....................  ....................  ....................
4343 MW276836.1 |Severe acute respiratory syndrome coro    ....................  ....................  ....................  ....................  ....................  ....................
4344 MW276837.1 |Severe acute respiratory syndrome coro    ....................  ....................  ....................  ....................  ....................  ....................
4345 MW276839.1 |Severe acute respiratory syndrome coro    ....................  ....................  ....................  ....................  ....................  ....................
4346 MW276840.1 |Severe acute respiratory syndrome coro    ....................  ....................  ....................  ....................  ....................  ....................
4347 MW276841.1 |Severe acute respiratory syndrome coro    ....................  ....................  ....................  ....................  ....................  ....................
4348 MW276842.1 |Severe acute respiratory syndrome coro    ....................  ....................  ....................  ....................  ....................  ....................
4349 MW276844.1 |Severe acute respiratory syndrome coro    ....................  ....................  ....................  ....................  ....................  ....................
4350 MW276845.1 |Severe acute respiratory syndrome coro    ....................  ....................  ....................  ....................  ....................  ....................
4351 MW276847.1 |Severe acute respiratory syndrome coro    ....................  ....................  ....................  ....................  ....................  ....................
4352 MW276850.1 |Severe acute respiratory syndrome coro    ....................  ....................  ....................  ....................  ....................  ....................
4353 MW276851.1 |Severe acute respiratory syndrome coro    ....................  ....................  ....................  ....................  ....................  ....................
4354 MW276852.1 |Severe acute respiratory syndrome coro    ....................  ....................  ....................  ....................  ....................  ....................
4355 MW276854.1 |Severe acute respiratory syndrome coro    ....................  ....................  ....................  ....................  ....................  ....................
4356 MW276856.1 |Severe acute respiratory syndrome coro    ....................  ....................  ....................  ....................  ....................  ....................
4357 MW276857.1 |Severe acute respiratory syndrome coro    ....................  ....................  ....................  ....................  ....................  ....................
4358 MW276858.1 |Severe acute respiratory syndrome coro    ....................  ....................  ....................  ....................  ....................  ....................
4359 MW276861.1 |Severe acute respiratory syndrome coro    ....................  ....................  ....................  ....................  ....................  ....................
4360 MW276863.1 |Severe acute respiratory syndrome coro    ....................  ....................  ....................  ....................  ....................  ....................
4361 MW276865.1 |Severe acute respiratory syndrome coro    ....................  ....................  ....................  ....................  ....................  ....................
4362 MW276866.1 |Severe acute respiratory syndrome coro    ....................  ....................  ....................  ....................  ....................  ....................
4363 MW276867.1 |Severe acute respiratory syndrome coro    ....................  ....................  ....................  ....................  ....................  ....................
4364 MW276868.1 |Severe acute respiratory syndrome coro    ....................  ....................  ....................  ....................  ....................  ....................
4365 MW276869.1 |Severe acute respiratory syndrome coro    ....................  ....................  ....................  ....................  ....................  ....................
4366 MW276870.1 |Severe acute respiratory syndrome coro    ....................  ....................  ....................  ....................  ....................  ....................
4367 MW276871.1 |Severe acute respiratory syndrome coro    ....................  ....................  ....................  ....................  NNN.................  ....................      1
4368 MW276872.1 |Severe acute respiratory syndrome coro    ....................  ....................  ....................  ....................  ....................  ....................
4369 MW276873.1 |Severe acute respiratory syndrome coro    ....................  ....................  ....................  ....................  ....................  ....................
4370 MW276874.1 |Severe acute respiratory syndrome coro    ....................  ....................  ....................  ....................  ....................  ....................
4371 MW276875.1 |Severe acute respiratory syndrome coro    ....................  ....................  ....................  ....................  ....................  ....................
4372 MW276876.1 |Severe acute respiratory syndrome coro    ....................  ....................  ....................  ....................  ....................  ....................
4373 MW276877.1 |Severe acute respiratory syndrome coro    ....................  ....................  ....................  ....................  ....................  ....................
4374 MW276879.1 |Severe acute respiratory syndrome coro    ....................  ....................  ....................  ....................  ....................  ....................
4375 MW276880.1 |Severe acute respiratory syndrome coro    ....................  ....................  ....................  ....................  ....................  ....................
4376 MW276882.1 |Severe acute respiratory syndrome coro    ....................  ....................  ....................  ....................  ....................  ....................
4377 MW276883.1 |Severe acute respiratory syndrome coro    ....................  ....................  ....................  ....................  ....................  ....................
4378 MW276885.1 |Severe acute respiratory syndrome coro    ....................  ....................  ....................  ....................  ....................  ....................
4379 MW276886.1 |Severe acute respiratory syndrome coro    ....................  ....................  ....................  ....................  ....................  ....................
4382 MW276889.1 |Severe acute respiratory syndrome coro    ....................  ....................  ....................  ....................  ....................  ....................
```

4383 MW276890.1 |Severe acute respiratory syndrome coro ···················· ···················· ···················· ···················· ···················· ····················
4384 MW276891.1 |Severe acute respiratory syndrome coro ···················· ···················· ···················· ···················· ···················· ····················
4385 MW276893.1 |Severe acute respiratory syndrome coro ···················· ···················· ···················· ···················· ···················· ····················
4386 MW276895.1 |Severe acute respiratory syndrome coro ···················· ···················· ···················· ···················· ···················· ····················
4387 MW276896.1 |Severe acute respiratory syndrome coro ···················· ···················· ···················· ···················· ···················· ····················
4388 MW276901.1 |Severe acute respiratory syndrome coro ···················· ···················· ···················· ···················· ···················· ····················
4389 MW276902.1 |Severe acute respiratory syndrome coro ···················· ···················· ···················· ···················· ···················· ····················
4390 MW276903.1 |Severe acute respiratory syndrome coro ···················· ···················· ···················· ···················· ···················· ····················
4391 MW276905.1 |Severe acute respiratory syndrome coro ········T··········· ···················· ···················· ···················· ···················· ····················
4392 MW276907.1 |Severe acute respiratory syndrome coro ···················· ···················· ···················· ···················· ···················· ····················
4393 MW276908.1 |Severe acute respiratory syndrome coro ···················· ···················· ···················· ···················· ···················· ····················
4394 MW276909.1 |Severe acute respiratory syndrome coro ···················· ···················· ···················· ···················· ···················· ····················
4395 MW276913.1 |Severe acute respiratory syndrome coro ···················· ···················· ···················· ···················· ···················· ····················
4396 MW276916.1 |Severe acute respiratory syndrome coro ···················· ···················· ···················· ···················· ···················· ····················
4397 MW276917.1 |Severe acute respiratory syndrome coro ···················· ···················· ···················· ···················· ···················· ····················
4398 MW276918.1 |Severe acute respiratory syndrome coro ···················· ···················· ···················· ···················· ···················· ····················
4399 MW276919.1 |Severe acute respiratory syndrome coro ···················· ···················· ···················· ···················· ···················· ····················
4400 MW276920.1 |Severe acute respiratory syndrome coro ···················· ···················· ···················· ···················· ···················· ····················
4401 MW276921.1 |Severe acute respiratory syndrome coro ···················· ···················· ···················· ···················· ···················· ····················
4402 MW276922.1 |Severe acute respiratory syndrome coro ···················· ···················· ···················· ···················· ···················· ····················
4404 MW276924.1 |Severe acute respiratory syndrome coro ···················· ···················· ···················· ···················· ···················· ····················
4405 MW276925.1 |Severe acute respiratory syndrome coro ···················· ···················· ···················· ···················· ···················· ····················
4406 MW276926.1 |Severe acute respiratory syndrome coro ···················· ···················· ···················· ···················· ···················· ····················
4407 MW276927.1 |Severe acute respiratory syndrome coro ···················· ···················· ···················· ···················· ···················· ····················
4408 MW276929.1 |Severe acute respiratory syndrome coro ···················· ···················· ···················· ···················· ···················· ····················
4409 MW276930.1 |Severe acute respiratory syndrome coro ···················· ···················· ···················· ···················· ···················· ····················
4410 MW276931.1 |Severe acute respiratory syndrome coro ···················· ···················· ···················· ···················· ···················· ····················
4411 MW276932.1 |Severe acute respiratory syndrome coro ···················· ···················· ···················· ···················· ···················· ····················
4412 MW276933.1 |Severe acute respiratory syndrome coro ···················· ···················· ···················· ···················· ···················· ····················
4413 MW276936.1 |Severe acute respiratory syndrome coro ···················· ···················· ···················· ···················· ···················· ····················
4414 MW276937.1 |Severe acute respiratory syndrome coro ···················· ···················· ···················· ···················· ···················· ····················
4415 MW276938.1 |Severe acute respiratory syndrome coro ···················· ···················· ···················· ···················· ···················· ····················
4416 MW276940.1 |Severe acute respiratory syndrome coro ···················· ···················· ···················· ···················· ···················· ····················
4417 MW276941.1 |Severe acute respiratory syndrome coro ···················· ···················· ···················· ···················· ···················· ····················
4418 MW276942.1 |Severe acute respiratory syndrome coro ···················· ···················· ···················· ···················· ···················· ····················
4419 MW276943.1 |Severe acute respiratory syndrome coro ···················· ···················· ···················· ···················· ···················· ····················
4420 MW276944.1 |Severe acute respiratory syndrome coro ···················· ···················· ···················· ···················· ···················· ····················
4421 MW276945.1 |Severe acute respiratory syndrome coro ···················· ···················· ···················· ···················· ···················· ····················
4422 MW276946.1 |Severe acute respiratory syndrome coro ···················· ···················· ···················· ···················· ···················· ····················
4423 MW276947.1 |Severe acute respiratory syndrome coro ···················· ···················· ···················· ···················· ···················· ····················
4424 MW276948.1 |Severe acute respiratory syndrome coro ···················· ···················· ···················· ···················· ···················· ····················
4425 MW276950.1 |Severe acute respiratory syndrome coro ···················· ···················· ···················· ···················· ···················· ····················
4426 MW276951.1 |Severe acute respiratory syndrome coro ···················· ···················· ···················· ···················· ···················· ····················
4427 MW276952.1 |Severe acute respiratory syndrome coro ···················· ···················· ···················· ···················· ···················· ····················
4428 MW276953.1 |Severe acute respiratory syndrome coro ···················· ···················· ···················· ···················· ···················· ····················
4430 MW276955.1 |Severe acute respiratory syndrome coro ···················· ···················· ···················· ···················· ···················· ····················
4431 MW276957.1 |Severe acute respiratory syndrome coro ···················· ···················· ···················· ···················· ···················· ····················
4432 MW276958.1 |Severe acute respiratory syndrome coro ···················· ···················· ···················· ···················· ···················· ····················
4433 MW276959.1 |Severe acute respiratory syndrome coro ···················· ···················· ···················· ···················· ···················· ····················
4434 MW276960.1 |Severe acute respiratory syndrome coro ···················· ···················· ···················· ···················· ···················· ····················
4435 MW276962.1 |Severe acute respiratory syndrome coro ···················· ···················· ···················· ···················· ···················· ····················
4436 MW276963.1 |Severe acute respiratory syndrome coro ···················· ···················· ···················· ···················· ···················· ····················
4437 MW276964.1 |Severe acute respiratory syndrome coro ···················· ···················· ···················· ···················· ···················· ····················
4438 MW276965.1 |Severe acute respiratory syndrome coro ···················· ···················· ···················· ···················· ···················· ····················
4439 MW276966.1 |Severe acute respiratory syndrome coro ···················· ···················· ···················· ···················· ···················· ····················
4440 MW276967.1 |Severe acute respiratory syndrome coro ···················· ···················· ···················· ···················· ···················· ····················
4441 MW276968.1 |Severe acute respiratory syndrome coro ···················· ···················· ···················· ···················· ···················· ····················
4442 MW276969.1 |Severe acute respiratory syndrome coro ···················· ···················· ···················· ···················· ···················· ····················
4443 MW276971.1 |Severe acute respiratory syndrome coro ···················· ···················· ···················· ···················· ···················· ····················
4444 MW276972.1 |Severe acute respiratory syndrome coro ···················· ···················· ···················· ···················· ···················· ····················
4445 MW276975.1 |Severe acute respiratory syndrome coro ···················· ···················· ···················· ···················· ···················· ····················
4446 MW276976.1 |Severe acute respiratory syndrome coro ···················· ···················· ···················· ···················· ···················· ····················
4447 MW276981.1 |Severe acute respiratory syndrome coro ···················· ···················· ···················· ···················· ···················· ····················
4448 MW276982.1 |Severe acute respiratory syndrome coro ···················· ···················· ···················· ···················· ···················· ····················
4449 MW276983.1 |Severe acute respiratory syndrome coro ···················· ···················· ···················· ···················· ···················· ····················
4450 MW276985.1 |Severe acute respiratory syndrome coro ···················· ···················· ···················· ···················· ···················· ····················
4451 MW276986.1 |Severe acute respiratory syndrome coro ···················· ···················· ···················· ···················· ···················· ····················
4452 MW276987.1 |Severe acute respiratory syndrome coro ···················· ···················· ···················· ···················· ···················· ····················
4453 MW276988.1 |Severe acute respiratory syndrome coro ···················· ···················· ···················· ···················· ···················· ····················
4454 MW276989.1 |Severe acute respiratory syndrome coro ···················· ···················· ···················· ···················· ···················· ····················
4455 MW276990.1 |Severe acute respiratory syndrome coro ···················· ···················· ···················· ···················· ···················· ····················
4456 MW276991.1 |Severe acute respiratory syndrome coro ···················· ···················· ···················· ···················· ···················· ····················
4457 MW276992.1 |Severe acute respiratory syndrome coro ···················· ···················· ···················· ···················· ···················· ····················
4458 MW276993.1 |Severe acute respiratory syndrome coro ···················· ···················· ···················· ···················· ···················· ····················
4459 MW276994.1 |Severe acute respiratory syndrome coro ···················· ···················· ···················· ···················· ···················· ····················
4460 MW276995.1 |Severe acute respiratory syndrome coro ···················· ···················· ···················· ···················· ···················· ····················
4461 MW277000.1 |Severe acute respiratory syndrome coro ···················· ···················· ···················· ···················· ···················· ····················
4462 MW277001.1 |Severe acute respiratory syndrome coro ···················· ···················· ···················· ···················· ···················· ····················

4463 MW277002.1 |Severe acute respiratory syndrome coro ....
4464 MW277003.1 |Severe acute respiratory syndrome coro ....
4465 MW277005.1 |Severe acute respiratory syndrome coro ....
4466 MW277006.1 |Severe acute respiratory syndrome coro ....
4467 MW277008.1 |Severe acute respiratory syndrome coro ....
4468 MW277009.1 |Severe acute respiratory syndrome coro ....
4469 MW277010.1 |Severe acute respiratory syndrome coro ....
4470 MW277012.1 |Severe acute respiratory syndrome coro ....
4471 MW277013.1 |Severe acute respiratory syndrome coro ....
4472 MW277016.1 |Severe acute respiratory syndrome coro ....
4473 MW277017.1 |Severe acute respiratory syndrome coro ....
4474 MW277018.1 |Severe acute respiratory syndrome coro ....
4475 MW277019.1 |Severe acute respiratory syndrome coro ....
4476 MW277021.1 |Severe acute respiratory syndrome coro ....
4477 MW277022.1 |Severe acute respiratory syndrome coro ....
4478 MW277023.1 |Severe acute respiratory syndrome coro ....
4479 MW277025.1 |Severe acute respiratory syndrome coro ....A............. ....
4480 MW277026.1 |Severe acute respiratory syndrome coro ....
4481 MW277027.1 |Severe acute respiratory syndrome coro ....
4482 MW277029.1 |Severe acute respiratory syndrome coro ....
4484 MW277031.1 |Severe acute respiratory syndrome coro ....
4486 MW277033.1 |Severe acute respiratory syndrome coro ....
4487 MW277034.1 |Severe acute respiratory syndrome coro ....
4488 MW277035.1 |Severe acute respiratory syndrome coro ....
4489 MW277037.1 |Severe acute respiratory syndrome coro ....
4490 MW277038.1 |Severe acute respiratory syndrome coro ....
4492 MW277042.1 |Severe acute respiratory syndrome coro ....
4493 MW277043.1 |Severe acute respiratory syndrome coro ....
4494 MW277045.1 |Severe acute respiratory syndrome coro ....
4495 MW277047.1 |Severe acute respiratory syndrome coro ....
4496 MW277048.1 |Severe acute respiratory syndrome coro ....
4497 MW277049.1 |Severe acute respiratory syndrome coro ....
4498 MW277054.1 |Severe acute respiratory syndrome coro ....
4499 MW277055.1 |Severe acute respiratory syndrome coro ....
4500 MW277056.1 |Severe acute respiratory syndrome coro ....
4501 MW277057.1 |Severe acute respiratory syndrome coro ....
4502 MW277058.1 |Severe acute respiratory syndrome coro ....
4503 MW277059.1 |Severe acute respiratory syndrome coro ....
4504 MW277060.1 |Severe acute respiratory syndrome coro ....
4505 MW277061.1 |Severe acute respiratory syndrome coro ....
4506 MW277062.1 |Severe acute respiratory syndrome coro ....
4507 MW277064.1 |Severe acute respiratory syndrome coro ....
4508 MW277065.1 |Severe acute respiratory syndrome coro ....
4509 MW277067.1 |Severe acute respiratory syndrome coro ....
4510 MW277068.1 |Severe acute respiratory syndrome coro ....
4511 MW277069.1 |Severe acute respiratory syndrome coro ....
4512 MW277073.1 |Severe acute respiratory syndrome coro ....
4513 MW277074.1 |Severe acute respiratory syndrome coro ....
4514 MW277075.1 |Severe acute respiratory syndrome coro ....
4515 MW277076.1 |Severe acute respiratory syndrome coro ....
4516 MW277077.1 |Severe acute respiratory syndrome coro ....
4517 MW277078.1 |Severe acute respiratory syndrome coro ....
4518 MW277079.1 |Severe acute respiratory syndrome coro ....
4519 MW277080.1 |Severe acute respiratory syndrome coro ....
4520 MW277081.1 |Severe acute respiratory syndrome coro ....
4521 MW277083.1 |Severe acute respiratory syndrome coro ....
4522 MW277084.1 |Severe acute respiratory syndrome coro ....
4523 MW277085.1 |Severe acute respiratory syndrome coro ....
4524 MW277086.1 |Severe acute respiratory syndrome coro ....
4525 MW277087.1 |Severe acute respiratory syndrome coro ....
4526 MW277088.1 |Severe acute respiratory syndrome coro ....
4527 MW277089.1 |Severe acute respiratory syndrome coro ....
4528 MW277091.1 |Severe acute respiratory syndrome coro ....
4529 MW277093.1 |Severe acute respiratory syndrome coro ....
4530 MW277094.1 |Severe acute respiratory syndrome coro ....
4531 MW277095.1 |Severe acute respiratory syndrome coro ....
4532 MW277096.1 |Severe acute respiratory syndrome coro ....
4533 MW277097.1 |Severe acute respiratory syndrome coro ....
4534 MW277098.1 |Severe acute respiratory syndrome coro ....
4535 MW277099.1 |Severe acute respiratory syndrome coro ....
4536 MW277100.1 |Severe acute respiratory syndrome coro ....
4537 MW277101.1 |Severe acute respiratory syndrome coro ....
4538 MW277103.1 |Severe acute respiratory syndrome coro ....
4539 MW277105.1 |Severe acute respiratory syndrome coro ....
4540 MW277106.1 |Severe acute respiratory syndrome coro ....
4541 MW277107.1 |Severe acute respiratory syndrome coro ....
4542 MW277113.1 |Severe acute respiratory syndrome coro ....
4543 MW277114.1 |Severe acute respiratory syndrome coro ....

4544 MW277115.1 |Severe acute respiratory syndrome coro ....
4545 MW277116.1 |Severe acute respiratory syndrome coro ....
4546 MW277117.1 |Severe acute respiratory syndrome coro ....
4547 MW277119.1 |Severe acute respiratory syndrome coro ....
4548 MW277120.1 |Severe acute respiratory syndrome coro ....
4549 MW277123.1 |Severe acute respiratory syndrome coro ....
4550 MW277124.1 |Severe acute respiratory syndrome coro ....
4551 MW277125.1 |Severe acute respiratory syndrome coro ....
4552 MW277126.1 |Severe acute respiratory syndrome coro ....
4553 MW277127.1 |Severe acute respiratory syndrome coro ....
4554 MW277129.1 |Severe acute respiratory syndrome coro ....
4555 MW277131.1 |Severe acute respiratory syndrome coro ....
4556 MW277132.1 |Severe acute respiratory syndrome coro ....
4557 MW277133.1 |Severe acute respiratory syndrome coro ....
4558 MW277134.1 |Severe acute respiratory syndrome coro ....
4559 MW277135.1 |Severe acute respiratory syndrome coro ....
4560 MW277136.1 |Severe acute respiratory syndrome coro ....
4561 MW277138.1 |Severe acute respiratory syndrome coro ....
4562 MW277141.1 |Severe acute respiratory syndrome coro ....
4564 MW277145.1 |Severe acute respiratory syndrome coro ....
4565 MW277146.1 |Severe acute respiratory syndrome coro ....
4568 MW277150.1 |Severe acute respiratory syndrome coro ....
4569 MW277152.1 |Severe acute respiratory syndrome coro ....
4570 MW277153.1 |Severe acute respiratory syndrome coro ....
4571 MW277154.1 |Severe acute respiratory syndrome coro ....
4572 MW277156.1 |Severe acute respiratory syndrome coro ....
4573 MW277157.1 |Severe acute respiratory syndrome coro ....
4574 MW277158.1 |Severe acute respiratory syndrome coro ....
4575 MW277160.1 |Severe acute respiratory syndrome coro ....
4576 MW277161.1 |Severe acute respiratory syndrome coro ....
4577 MW277162.1 |Severe acute respiratory syndrome coro ....
4578 MW277164.1 |Severe acute respiratory syndrome coro ....
4579 MW277166.1 |Severe acute respiratory syndrome coro ....
4580 MW277167.1 |Severe acute respiratory syndrome coro ....
4581 MW277168.1 |Severe acute respiratory syndrome coro ....
4582 MW277169.1 |Severe acute respiratory syndrome coro ....
4583 MW277170.1 |Severe acute respiratory syndrome coro ....
4584 MW277173.1 |Severe acute respiratory syndrome coro ....
4585 MW277174.1 |Severe acute respiratory syndrome coro ....
4586 MW277175.1 |Severe acute respiratory syndrome coro ....
4587 MW277176.1 |Severe acute respiratory syndrome coro ....
4588 MW277177.1 |Severe acute respiratory syndrome coro ....
4589 MW277179.1 |Severe acute respiratory syndrome coro ....
4590 MW277180.1 |Severe acute respiratory syndrome coro ....
4591 MW277181.1 |Severe acute respiratory syndrome coro ....
4592 MW277184.1 |Severe acute respiratory syndrome coro ....
4593 MW277185.1 |Severe acute respiratory syndrome coro ....
4594 MW277187.1 |Severe acute respiratory syndrome coro ....
4595 MW277188.1 |Severe acute respiratory syndrome coro ....
4596 MW277189.1 |Severe acute respiratory syndrome coro ....
4597 MW277190.1 |Severe acute respiratory syndrome coro ....
4598 MW277191.1 |Severe acute respiratory syndrome coro ....
4599 MW277192.1 |Severe acute respiratory syndrome coro ....
4600 MW277193.1 |Severe acute respiratory syndrome coro ....
4601 MW277194.1 |Severe acute respiratory syndrome coro ....
4603 MW277198.1 |Severe acute respiratory syndrome coro ....
4604 MW277199.1 |Severe acute respiratory syndrome coro ....
4605 MW277200.1 |Severe acute respiratory syndrome coro ....
4606 MW277201.1 |Severe acute respiratory syndrome coro ....
4607 MW277202.1 |Severe acute respiratory syndrome coro ....
4608 MW277203.1 |Severe acute respiratory syndrome coro ....
4609 MW277205.1 |Severe acute respiratory syndrome coro ....
4610 MW277206.1 |Severe acute respiratory syndrome coro ....
4611 MW277207.1 |Severe acute respiratory syndrome coro ....
4612 MW277208.1 |Severe acute respiratory syndrome coro ....
4613 MW277209.1 |Severe acute respiratory syndrome coro ....
4614 MW277210.1 |Severe acute respiratory syndrome coro ....
4615 MW277211.1 |Severe acute respiratory syndrome coro ....
4616 MW277212.1 |Severe acute respiratory syndrome coro ....
4617 MW277213.1 |Severe acute respiratory syndrome coro ....
4618 MW277214.1 |Severe acute respiratory syndrome coro ....
4619 MW277215.1 |Severe acute respiratory syndrome coro ....
4620 MW277217.1 |Severe acute respiratory syndrome coro ....
4621 MW277218.1 |Severe acute respiratory syndrome coro ....
4622 MW277219.1 |Severe acute respiratory syndrome coro ....
4623 MW277220.1 |Severe acute respiratory syndrome coro ....
4624 MW277221.1 |Severe acute respiratory syndrome coro ....
4625 MW277224.1 |Severe acute respiratory syndrome coro ....

4626 MW277227.1 |Severe acute respiratory syndrome coro ....
4627 MW277230.1 |Severe acute respiratory syndrome coro ....
4628 MW277232.1 |Severe acute respiratory syndrome coro ....
4629 MW277235.1 |Severe acute respiratory syndrome coro ....
4630 MW277236.1 |Severe acute respiratory syndrome coro ....
4631 MW277237.1 |Severe acute respiratory syndrome coro ....
4632 MW277240.1 |Severe acute respiratory syndrome coro ....
4633 MW277242.1 |Severe acute respiratory syndrome coro ....
4634 MW277243.1 |Severe acute respiratory syndrome coro ....
4635 MW277244.1 |Severe acute respiratory syndrome coro ....
4636 MW277245.1 |Severe acute respiratory syndrome coro ....
4637 MW277246.1 |Severe acute respiratory syndrome coro ....
4638 MW277247.1 |Severe acute respiratory syndrome coro ....
4639 MW277248.1 |Severe acute respiratory syndrome coro ....
4640 MW277249.1 |Severe acute respiratory syndrome coro ....
4641 MW277250.1 |Severe acute respiratory syndrome coro ....
4642 MW277251.1 |Severe acute respiratory syndrome coro ....
4643 MW277252.1 |Severe acute respiratory syndrome coro ....
4644 MW277253.1 |Severe acute respiratory syndrome coro ....
4645 MW277254.1 |Severe acute respiratory syndrome coro ....
4646 MW277255.1 |Severe acute respiratory syndrome coro ....
4647 MW277256.1 |Severe acute respiratory syndrome coro ....
4648 MW277257.1 |Severe acute respiratory syndrome coro ....
4649 MW277258.1 |Severe acute respiratory syndrome coro ....
4650 MW277259.1 |Severe acute respiratory syndrome coro ....
4651 MW277260.1 |Severe acute respiratory syndrome coro ....
4652 MW277261.1 |Severe acute respiratory syndrome coro ....
4653 MW277262.1 |Severe acute respiratory syndrome coro ....
4654 MW277263.1 |Severe acute respiratory syndrome coro ....
4655 MW277264.1 |Severe acute respiratory syndrome coro ....
4656 MW277265.1 |Severe acute respiratory syndrome coro ....
4657 MW277267.1 |Severe acute respiratory syndrome coro ....
4658 MW277269.1 |Severe acute respiratory syndrome coro ....
4659 MW277271.1 |Severe acute respiratory syndrome coro ....
4660 MW277272.1 |Severe acute respiratory syndrome coro ....A............. ....
4661 MW277273.1 |Severe acute respiratory syndrome coro ....
4662 MW277274.1 |Severe acute respiratory syndrome coro ....
4663 MW277275.1 |Severe acute respiratory syndrome coro ....
4664 MW277276.1 |Severe acute respiratory syndrome coro ....
4665 MW277277.1 |Severe acute respiratory syndrome coro ....
4666 MW277279.1 |Severe acute respiratory syndrome coro ....
4667 MW277281.1 |Severe acute respiratory syndrome coro ....
4668 MW277282.1 |Severe acute respiratory syndrome coro ....
4669 MW277283.1 |Severe acute respiratory syndrome coro ....
4670 MW277284.1 |Severe acute respiratory syndrome coro ....
4671 MW277285.1 |Severe acute respiratory syndrome coro ....A............. ....
4672 MW277286.1 |Severe acute respiratory syndrome coro ....
4673 MW277287.1 |Severe acute respiratory syndrome coro ....
4674 MW277289.1 |Severe acute respiratory syndrome coro ....
4675 MW277290.1 |Severe acute respiratory syndrome coro ....
4676 MW277291.1 |Severe acute respiratory syndrome coro ....
4677 MW277292.1 |Severe acute respiratory syndrome coro ....
4678 MW277293.1 |Severe acute respiratory syndrome coro ....
4679 MW277294.1 |Severe acute respiratory syndrome coro ....
4680 MW277297.1 |Severe acute respiratory syndrome coro ....
4681 MW277299.1 |Severe acute respiratory syndrome coro ....
4682 MW277300.1 |Severe acute respiratory syndrome coro ....
4683 MW277301.1 |Severe acute respiratory syndrome coro ....
4684 MW277302.1 |Severe acute respiratory syndrome coro ....
4685 MW277303.1 |Severe acute respiratory syndrome coro ....
4686 MW277304.1 |Severe acute respiratory syndrome coro ....
4687 MW277306.1 |Severe acute respiratory syndrome coro ....
4688 MW277307.1 |Severe acute respiratory syndrome coro ....
4689 MW277308.1 |Severe acute respiratory syndrome coro ....
4690 MW277309.1 |Severe acute respiratory syndrome coro ....
4691 MW277311.1 |Severe acute respiratory syndrome coro ....A............. ....
4692 MW277312.1 |Severe acute respiratory syndrome coro ....
4693 MW277313.1 |Severe acute respiratory syndrome coro ....
4694 MW277314.1 |Severe acute respiratory syndrome coro ....
4696 MW277316.1 |Severe acute respiratory syndrome coro ....
4697 MW277317.1 |Severe acute respiratory syndrome coro ....
4698 MW277318.1 |Severe acute respiratory syndrome coro ....
4699 MW277319.1 |Severe acute respiratory syndrome coro ....
4700 MW277320.1 |Severe acute respiratory syndrome coro ....
4701 MW277321.1 |Severe acute respiratory syndrome coro ....
4702 MW277322.1 |Severe acute respiratory syndrome coro ....
4703 MW277323.1 |Severe acute respiratory syndrome coro ....
4704 MW277324.1 |Severe acute respiratory syndrome coro ....

```
4705 MW277325.1 |Severe acute respiratory syndrome coro    ....................  ....................  ....................  ....................  ....................  ....................
4706 MW277327.1 |Severe acute respiratory syndrome coro    ....................  ....................  ....................  ....................  ....................  ....................
4707 MW277328.1 |Severe acute respiratory syndrome coro    ....................  ....................  ....................  ....................  ....................  ....................
4708 MW277329.1 |Severe acute respiratory syndrome coro    ....................  ....................  ....................  ....................  ....................  ....................
4709 MW277330.1 |Severe acute respiratory syndrome coro    ....................  ....................  ....................  ....................  ....................  ....................
4710 MW277331.1 |Severe acute respiratory syndrome coro    ....................  ....................  ....................  ....................  ....................  ....................
4711 MW277332.1 |Severe acute respiratory syndrome coro    ....................  ....................  ....................  ....................  ....................  ....................
4712 MW277334.1 |Severe acute respiratory syndrome coro    ....................  ....................  ....................  ....................  ....................  ....................
4713 MW277336.1 |Severe acute respiratory syndrome coro    ....................  ....................  ....................  ....................  ....................  ....................
4714 MW277337.1 |Severe acute respiratory syndrome coro    ....................  ....................  ....................  ....................  ....................  ....................
4715 MW277338.1 |Severe acute respiratory syndrome coro    ....................  ....................  ....................  ....................  ....................  ....................
4716 MW277339.1 |Severe acute respiratory syndrome coro    ....................  ....................  ....................  ....................  ....................  ....................
4717 MW277340.1 |Severe acute respiratory syndrome coro    ....................  ....................  ....................  ....................  ....................  ....................
4718 MW277341.1 |Severe acute respiratory syndrome coro    ....................  ....................  ....................  ....................  ....................  ....................
4719 MW277343.1 |Severe acute respiratory syndrome coro    ....................  ....................  ....................  ....................  ....................  ....................
4720 MW277344.1 |Severe acute respiratory syndrome coro    ....................  ....................  ....................  ....................  ....................  ....................
4721 MW277345.1 |Severe acute respiratory syndrome coro    ....................  ....................  ....................  ....................  ....................  ....................
4722 MW277346.1 |Severe acute respiratory syndrome coro    ....................  ....................  ....................  ....................  ....................  ....................
4723 MW277347.1 |Severe acute respiratory syndrome coro    ....................  ....................  ....................  ....................  ....................  ....................
4724 MW277348.1 |Severe acute respiratory syndrome coro    ....................  ....................  ....................  ....................  ....................  ....................
4725 MW277351.1 |Severe acute respiratory syndrome coro    ....................  ....................  ....................  ....................  ....................  ....................
4726 MW277352.1 |Severe acute respiratory syndrome coro    ....................  ....................  ....................  ....................  ....................  ....................
4727 MW277354.1 |Severe acute respiratory syndrome coro    ....................  ....................  ....................  ....................  ....................  ....................
4728 MW277355.1 |Severe acute respiratory syndrome coro    ....................  ....................  ....................  ....................  ....................  ....................
4729 MW277356.1 |Severe acute respiratory syndrome coro    ....................  ....................  ....................  ....................  ....................  ....................
4730 MW277357.1 |Severe acute respiratory syndrome coro    ....................  ....................  ....................  ....................  ....................  ....................
4731 MW277359.1 |Severe acute respiratory syndrome coro    ....................  ....................  ....................  ....................  ....................  ....................
4732 MW277361.1 |Severe acute respiratory syndrome coro    ....................  ....................  ....................  ....................  ....................  ....................
4733 MW277362.1 |Severe acute respiratory syndrome coro    ....................  ....................  ....................  ....................  ....................  ....................
4734 MW277363.1 |Severe acute respiratory syndrome coro    ....................  ....................  ....................  ....................  ....................  ....................
4735 MW277364.1 |Severe acute respiratory syndrome coro    ....................  ....................  ....................  ....................  ....................  ....................
4736 MW277367.1 |Severe acute respiratory syndrome coro    ....................  ....................  ....................  ....................  ....................  ....................
4737 MW277368.1 |Severe acute respiratory syndrome coro    ....................  ....................  ....................  ....................  ....................  ....................
4738 MW277369.1 |Severe acute respiratory syndrome coro    ....................  ....................  ....................  ....................  ....................  ....................
4739 MW277371.1 |Severe acute respiratory syndrome coro    ....................  ....................  ....................  ....................  ....................  ....................
4740 MW277372.1 |Severe acute respiratory syndrome coro    ....................  ....................  ....................  ....................  ....................  ....................
4741 MW277373.1 |Severe acute respiratory syndrome coro    ....................  ....................  ....................  ....................  ....................  ....................
4742 MW277376.1 |Severe acute respiratory syndrome coro    ....................  ....................  ....................  ....................  ....................  ....................
4743 MW277378.1 |Severe acute respiratory syndrome coro    ....................  ....................  ....................  ....................  ....................  ....................
4744 MW277379.1 |Severe acute respiratory syndrome coro    ....................  ....................  ....................  ....................  ....................  ....................
4745 MW277380.1 |Severe acute respiratory syndrome coro    ....................  ....................  ....................  ....................  ....................  ....................
4746 MW277381.1 |Severe acute respiratory syndrome coro    ....................  ....................  ....................  ....................  ....................  ....................
4747 MW277382.1 |Severe acute respiratory syndrome coro    ....................  ....................  ....................  ....................  ....................  ....................
4748 MW277385.1 |Severe acute respiratory syndrome coro    ....................  ....................  ....................  ....................  ....................  ....................
4749 MW277386.1 |Severe acute respiratory syndrome coro    ....................  ....................  ....................  ....................  ....................  ....................
4751 MW277388.1 |Severe acute respiratory syndrome coro    ....................  ....................  ....................  ....................  ....................  ....................
4752 MW277389.1 |Severe acute respiratory syndrome coro    ....................  ....................  ....................  ....................  ....................  ....................
4753 MW277390.1 |Severe acute respiratory syndrome coro    ....................  ....................  ....................  ....................  ....................  ....................
4754 MW277391.1 |Severe acute respiratory syndrome coro    ....................  ....................  ....................  ....................  ....................  ....................
4755 MW277392.1 |Severe acute respiratory syndrome coro    ....................  ....................  ....................  ....................  ....................  ....................
4756 MW277393.1 |Severe acute respiratory syndrome coro    ....................  ....................  ....................  ....................  ....................  ....................
4757 MW277394.1 |Severe acute respiratory syndrome coro    ....................  ....................  ....................  ....................  ....................  ....................
4758 MW277395.1 |Severe acute respiratory syndrome coro    ....................  ....................  ....................  ....................  ....................  ....................
4759 MW277396.1 |Severe acute respiratory syndrome coro    ....................  ....................  ....................  ....................  ....................  ....................
4760 MW277397.1 |Severe acute respiratory syndrome coro    ....................  ....................  ....................  ....................  ....................  ....................
4761 MW277398.1 |Severe acute respiratory syndrome coro    ....................  ....................  ....................  ....................  ....................  ....................
4762 MW277400.1 |Severe acute respiratory syndrome coro    ....................  ....................  ....................  ....................  ....................  ....................
4763 MW277402.1 |Severe acute respiratory syndrome coro    ....................  ....................  ....................  ....................  ....................  ....................
4764 MW277403.1 |Severe acute respiratory syndrome coro    ....................  ....................  ....................  ....................  ....................  ....................
4765 MW277404.1 |Severe acute respiratory syndrome coro    ....................  ....................  ....................  ....................  ....................  ....................
4767 MW277406.1 |Severe acute respiratory syndrome coro    ....................  ....................  ....................  ....................  ....................  ....................
4768 MW277408.1 |Severe acute respiratory syndrome coro    ....................  ....................  ....................  ....................  ....................  ....................
4769 MW277409.1 |Severe acute respiratory syndrome coro    ....................  ....................  ....................  ....................  ....................  ....................
4770 MW277410.1 |Severe acute respiratory syndrome coro    ....................  ....................  ....................  ....................  ....................  ....................
4771 MW277411.1 |Severe acute respiratory syndrome coro    ....................  ....................  ....................  ....................  ....................  ....................
4772 MW277413.1 |Severe acute respiratory syndrome coro    ....................  ....................  ....................  ....................  ....................  ....................
4773 MW277414.1 |Severe acute respiratory syndrome coro    ....................  ....................  ....................  ....................  ....................  ....................
4774 MW277416.1 |Severe acute respiratory syndrome coro    ....................  ....................  ....................  ....................  ....................  ....................
4775 MW277418.1 |Severe acute respiratory syndrome coro    ....................  ....................  ....................  ....................  ....................  ....................
4776 MW277419.1 |Severe acute respiratory syndrome coro    ....................  ....................  ....................  ....................  ....................  ....................
4777 MW277421.1 |Severe acute respiratory syndrome coro    ....................  ....................  ....................  ....................  ....................  ....................
4778 MW277422.1 |Severe acute respiratory syndrome coro    ....................  ....................  ....................  ....................  ....................  ....................
4779 MW277423.1 |Severe acute respiratory syndrome coro    ....................  ....................  ....................  ....................  ....................  ....................
4780 MW277424.1 |Severe acute respiratory syndrome coro    ....................  ....................  ....................  ....................  ....................  ....................
4781 MW277426.1 |Severe acute respiratory syndrome coro    ....................  ....................  ....................  ....................  ....................  ....................
4782 MW277427.1 |Severe acute respiratory syndrome coro    ....................  ....................  ....................  ....................  ....................  ....................
4783 MW277428.1 |Severe acute respiratory syndrome coro    ....................  ....................  ....................  ....................  ....................  ....................
4784 MW277429.1 |Severe acute respiratory syndrome coro    ....................  ....................  ....................  ....................  ....................  ....................
```

```
4785 MW277430.1 |Severe acute respiratory syndrome coro    ....................  ....................  ....................  ....................  ....................  ....................
4786 MW277431.1 |Severe acute respiratory syndrome coro    ....................  ....................  ....................  ....................  ....................  ....................
4787 MW277432.1 |Severe acute respiratory syndrome coro    ....................  ....................  ....................  ....................  ....................  ....................
4788 MW277433.1 |Severe acute respiratory syndrome coro    ....................  ....................  ....................  ....................  ....................  ....................
4790 MW277437.1 |Severe acute respiratory syndrome coro    ....................  ....................  ....................  ....................  ....................  ....................
4791 MW277438.1 |Severe acute respiratory syndrome coro    ....................  ....................  ....................  ....................  ....................  ....................
4792 MW277439.1 |Severe acute respiratory syndrome coro    ....................  ....................  ....................  ....................  ....................  ....................
4793 MW277440.1 |Severe acute respiratory syndrome coro    ....................  ....................  ....................  ....................  ....................  ....................
4794 MW277442.1 |Severe acute respiratory syndrome coro    ....................  ....................  ....................  ....................  ....................  ....................
4795 MW277443.1 |Severe acute respiratory syndrome coro    ....................  ....................  ....................  ....................  ....................  ....................
4796 MW277446.1 |Severe acute respiratory syndrome coro    ....................  ....................  ....................  ....................  ....................  ....................
4797 MW277447.1 |Severe acute respiratory syndrome coro    ....................  ....................  ....................  ....................  ....................  ....................
4798 MW277448.1 |Severe acute respiratory syndrome coro    ....................  ....................  ....................  ....................  ....................  ....................
4799 MW277449.1 |Severe acute respiratory syndrome coro    ....................  ....................  ....................  ....................  ....................  ....................
4800 MW277450.1 |Severe acute respiratory syndrome coro    ....................  ....................  ....................  ....................  ....................  ....................
4801 MW277452.1 |Severe acute respiratory syndrome coro    ....................  ....................  ....................  ....................  ....................  ....................
4802 MW277454.1 |Severe acute respiratory syndrome coro    ....................  ....................  ....................  ....................  ....................  ....................
4803 MW277455.1 |Severe acute respiratory syndrome coro    ....................  ....................  ....................  ....................  ....................  ....................
4804 MW277456.1 |Severe acute respiratory syndrome coro    ....................  ....................  ....................  ....................  ....................  ....................
4805 MW277457.1 |Severe acute respiratory syndrome coro    ....................  ....................  ....................  ....................  ....................  ....................
4806 MW277458.1 |Severe acute respiratory syndrome coro    ....................  ....................  ....................  ....................  ....................  ....................
4807 MW277459.1 |Severe acute respiratory syndrome coro    ....................  ....................  ....................  ....................  ....................  ....................
4808 MW277460.1 |Severe acute respiratory syndrome coro    ....................  ....................  ....................  ....................  ....................  ....................
4809 MW277462.1 |Severe acute respiratory syndrome coro    ....................  ....................  ....................  ....................  ....................  ....................
4810 MW277463.1 |Severe acute respiratory syndrome coro    ....................  ....................  ....................  ....................  ....................  ....................
4811 MW277464.1 |Severe acute respiratory syndrome coro    ....................  ....................  ....................  ....................  ....................  ....................
4812 MW277465.1 |Severe acute respiratory syndrome coro    ....................  ....................  ....................  ....................  ....................  ....................
4813 MW277466.1 |Severe acute respiratory syndrome coro    ....................  ....................  ....................  ....................  ....................  ....................
4814 MW277467.1 |Severe acute respiratory syndrome coro    ....................  ....................  ....................  ....................  ....................  ....................
4815 MW277468.1 |Severe acute respiratory syndrome coro    ....................  ....................  ....................  ....................  ....................  ....................
4816 MW277470.1 |Severe acute respiratory syndrome coro    ....................  ....................  ....................  ....................  ....................  ....................
4817 MW277471.1 |Severe acute respiratory syndrome coro    ....................  ....................  ....................  ....................  ....................  ....................
4818 MW277472.1 |Severe acute respiratory syndrome coro    ....................  ....................  ....................  ....................  ....................  ....................
4819 MW277474.1 |Severe acute respiratory syndrome coro    ....................  ....................  ....................  ....................  ....................  ....................
4821 MW277476.1 |Severe acute respiratory syndrome coro    ....................  ....................  ....................  ....................  ....................  ....................
4822 MW277477.1 |Severe acute respiratory syndrome coro    ....................  ....................  ....................  ....................  ....................  ....................
4823 MW277478.1 |Severe acute respiratory syndrome coro    ....................  ....................  ....................  ....................  ....................  ....................
4824 MW277480.1 |Severe acute respiratory syndrome coro    ....................  ....................  ....................  ....................  ....................  ....................
4825 MW277481.1 |Severe acute respiratory syndrome coro    ....................  ....................  ....................  ....................  ....................  ....................
4826 MW277483.1 |Severe acute respiratory syndrome coro    ....................  ....................  ....................  ....................  ....................  ....................
4827 MW277484.1 |Severe acute respiratory syndrome coro    ....................  ....................  ....................  ....................  ....................  ....................
4828 MW277485.1 |Severe acute respiratory syndrome coro    ....................  ....................  ....................  ....................  ....................  ....................
4829 MW277486.1 |Severe acute respiratory syndrome coro    ....................  ....................  ....................  ....................  ....................  ....................
4830 MW277487.1 |Severe acute respiratory syndrome coro    ....................  ....................  ....................  ....................  ....................  ....................
4831 MW277488.1 |Severe acute respiratory syndrome coro    ....................  ....................  ....................  ....................  ....................  ....................
4832 MW277490.1 |Severe acute respiratory syndrome coro    ....................  ....................  ....................  ....................  ....................  ....................
4833 MW277491.1 |Severe acute respiratory syndrome coro    ....................  ....................  ....................  ....................  ....................  ....................
4834 MW277492.1 |Severe acute respiratory syndrome coro    ....................  ....................  ....................  ....................  ....................  ....................
4835 MW277493.1 |Severe acute respiratory syndrome coro    ....................  ....................  ....................  ....................  ....................  ....................
4836 MW277494.1 |Severe acute respiratory syndrome coro    ....................  ....................  ....................  ....................  ....................  ....................
4837 MW277495.1 |Severe acute respiratory syndrome coro    ....................  ....................  ....................  ....................  ....................  ....................
4838 MW277496.1 |Severe acute respiratory syndrome coro    ....................  ....................  ....................  ....................  ....................  ....................
4839 MW277497.1 |Severe acute respiratory syndrome coro    ....................  ....................  ....................  ....................  ....................  ....................
4840 MW277498.1 |Severe acute respiratory syndrome coro    ....................  ....................  ....................  ....................  ....................  ....................
4841 MW277499.1 |Severe acute respiratory syndrome coro    ....................  ....................  ....................  ....................  ....................  ....................
4842 MW277500.1 |Severe acute respiratory syndrome coro    ....................  ....................  ....................  ....................  ....................  ....................
4843 MW277501.1 |Severe acute respiratory syndrome coro    ....................  ....................  ....................  ....................  ....................  ....................
4844 MW277502.1 |Severe acute respiratory syndrome coro    ....................  ....................  ....................  ....................  ....................  ....................
4845 MW277503.1 |Severe acute respiratory syndrome coro    ....................  ....................  ....................  ....................  ....................  ....................
4846 MW277506.1 |Severe acute respiratory syndrome coro    ....................  ....................  ....................  ....................  ....................  ....................
4847 MW277507.1 |Severe acute respiratory syndrome coro    ....................  ....................  ....................  ....................  ....................  ....................
4848 MW277508.1 |Severe acute respiratory syndrome coro    ....................  ....................  ....................  ....................  ....................  ....................
4850 MW277510.1 |Severe acute respiratory syndrome coro    ....................  ....................  ....................  ....................  ....................  ....................
4851 MW277511.1 |Severe acute respiratory syndrome coro    ....................  ....................  ....................  ....................  ....................  ....................
4852 MW277512.1 |Severe acute respiratory syndrome coro    ....................  ....................  ....................  ....................  ....................  ....................
4853 MW277513.1 |Severe acute respiratory syndrome coro    ....................  ....................  ....................  ....................  ....................  ....................
4854 MW277514.1 |Severe acute respiratory syndrome coro    ....................  ....................  ....................  ....................  ....................  ....................
4855 MW277515.1 |Severe acute respiratory syndrome coro    ....................  ....................  ....................  ....................  ....................  ....................
4856 MW277516.1 |Severe acute respiratory syndrome coro    ....................  ....................  ....................  ....................  ....................  ....................
4857 MW277517.1 |Severe acute respiratory syndrome coro    ....................  ....................  ....................  ....................  ....................  ....................
4858 MW277518.1 |Severe acute respiratory syndrome coro    ....................  ....................  ....................  ....................  ....................  ....................
4859 MW277519.1 |Severe acute respiratory syndrome coro    ....................  ....................  ....................  ....................  ....................  ....................
4860 MW277520.1 |Severe acute respiratory syndrome coro    ....................  ....................  ....................  ....................  ....................  ....................
4861 MW277521.1 |Severe acute respiratory syndrome coro    ....................  ....................  ....................  ....................  ....................  ....................
4862 MW277522.1 |Severe acute respiratory syndrome coro    ....................  ....................  ....................  ....................  ....................  ....................
4863 MW277525.1 |Severe acute respiratory syndrome coro    ....................  ....................  ....................  ....................  ....................  ....................
4864 MW277528.1 |Severe acute respiratory syndrome coro    ....................  ....................  ....................  ....................  ....................  ....................
4865 MW277529.1 |Severe acute respiratory syndrome coro    ....................  ....................  ....................  ....................  ....................  ....................
```

```
4866 MW277530.1 |Severe acute respiratory syndrome coro    ....................  ....................  ....................  ....................  ....................  ....................
4867 MW277532.1 |Severe acute respiratory syndrome coro    ....................  ....................  ....................  ....................  ....................  ....................
4868 MW277534.1 |Severe acute respiratory syndrome coro    ....................  ....................  ....................  ....................  ....................  ....................
4869 MW277535.1 |Severe acute respiratory syndrome coro    ....................  ....................  ....................  ....................  ....................  ....................
4870 MW277536.1 |Severe acute respiratory syndrome coro    ....................  ....................  ....................  ....................  ....................  ....................
4871 MW277537.1 |Severe acute respiratory syndrome coro    ....................  ....................  ....................  ....................  ....................  ....................
4872 MW277538.1 |Severe acute respiratory syndrome coro    ....................  ....................  ....................  ....................  ....................  ....................
4873 MW277539.1 |Severe acute respiratory syndrome coro    ....................  ....................  ....................  ....................  ....................  ....................
4874 MW277540.1 |Severe acute respiratory syndrome coro    ....................  ....................  ....................  ....................  ....................  ....................
4875 MW277544.1 |Severe acute respiratory syndrome coro    ....................  ....................  ....................  ....................  ....................  ....................
4876 MW277546.1 |Severe acute respiratory syndrome coro    ....................  ....................  ....................  ....................  ....................  ....................
4877 MW277547.1 |Severe acute respiratory syndrome coro    ....................  ....................  ....................  ....................  ....................  ....................
4878 MW277548.1 |Severe acute respiratory syndrome coro    ....................  ....................  ....................  ....................  ....................  ....................
4879 MW277550.1 |Severe acute respiratory syndrome coro    ....................  ....................  ....................  ....................  ....................  ....................
4880 MW277551.1 |Severe acute respiratory syndrome coro    ....................  ....................  ....................  ....................  ....................  ....................
4881 MW277553.1 |Severe acute respiratory syndrome coro    ....................  ....................  ....................  ....................  ....................  ....................
4882 MW277554.1 |Severe acute respiratory syndrome coro    ....................  ....................  ....................  ....................  ....................  ....................
4883 MW277555.1 |Severe acute respiratory syndrome coro    ....................  ....................  ....................  ....................  ....................  ....................
4884 MW277556.1 |Severe acute respiratory syndrome coro    ....................  ....................  ....................  ....................  ....................  ....................
4885 MW277557.1 |Severe acute respiratory syndrome coro    ....................  ....................  ....................  ....................  ....................  ....................
4886 MW277558.1 |Severe acute respiratory syndrome coro    ....................  ....................  ....................  ....................  ....................  ....................
4887 MW277559.1 |Severe acute respiratory syndrome coro    ....................  ....................  ....................  ....................  ....................  ....................
4888 MW277560.1 |Severe acute respiratory syndrome coro    ....................  ....................  ....................  ....................  ....................  ....................
4889 MW277561.1 |Severe acute respiratory syndrome coro    ....................  ....................  ....................  ....................  ....................  ....................
4890 MW277562.1 |Severe acute respiratory syndrome coro    ....................  ....................  ....................  ....................  ....................  ....................
4891 MW277563.1 |Severe acute respiratory syndrome coro    ....................  ....................  ....................  ....................  ....................  ....................
4892 MW277567.1 |Severe acute respiratory syndrome coro    ....................  ....................  ....................  ....................  ....................  ....................
4893 MW277568.1 |Severe acute respiratory syndrome coro    ....................  ....................  ....................  ....................  ....................  ....................
4894 MW277569.1 |Severe acute respiratory syndrome coro    ....................  ....................  ....................  ....................  ....................  ....................
4895 MW277571.1 |Severe acute respiratory syndrome coro    ....................  ....................  ....................  ....................  ....................  ....................
4896 MW277572.1 |Severe acute respiratory syndrome coro    ....................  ....................  ....................  ....................  ....................  ....................
4897 MW277574.1 |Severe acute respiratory syndrome coro    ....................  ....................  ....................  ....................  ....................  ....................
4898 MW277576.1 |Severe acute respiratory syndrome coro    ....................  ....................  ....................  ....................  ....................  ....................
4899 MW277577.1 |Severe acute respiratory syndrome coro    ....................  ....................  ....................  ....................  ....................  ....................
4900 MW277579.1 |Severe acute respiratory syndrome coro    ....................  ....................  ....................  ....................  ....................  ....................
4901 MW277581.1 |Severe acute respiratory syndrome coro    ....................  ....................  ....................  ....................  ....................  ....................
4902 MW277582.1 |Severe acute respiratory syndrome coro    ....................  ....................  ....................  ....................  ....................  ....................
4903 MW277583.1 |Severe acute respiratory syndrome coro    ....................  ....................  ....................  ....................  ....................  ....................
4904 MW277585.1 |Severe acute respiratory syndrome coro    ....................  ....................  ....................  ....................  ....................  ....................
4905 MW277586.1 |Severe acute respiratory syndrome coro    ....................  ....................  ....................  ....................  ....................  ....................
4906 MW277587.1 |Severe acute respiratory syndrome coro    ....................  ....................  ....................  ....................  ....................  ....................
4907 MW277588.1 |Severe acute respiratory syndrome coro    ....................  ....................  ....................  ....................  ....................  ....................
4908 MW277589.1 |Severe acute respiratory syndrome coro    ....................  ......A.............  ....................  ....................  ....................  ....................
4909 MW277590.1 |Severe acute respiratory syndrome coro    ....................  ....................  ....................  ....................  ....................  ....................
4910 MW277592.1 |Severe acute respiratory syndrome coro    ....................  ....................  ....................  ....................  ....................  ....................
4911 MW277594.1 |Severe acute respiratory syndrome coro    ....................  ....................  ....................  ....................  ....................  ....................
4912 MW277597.1 |Severe acute respiratory syndrome coro    ....................  ....................  ....................  ....................  ....................  ....................
4913 MW277598.1 |Severe acute respiratory syndrome coro    ....................  ....................  ....................  ....................  ....................  ....................
4914 MW277599.1 |Severe acute respiratory syndrome coro    ....................  ....................  ....................  ....................  ....................  ....................
4915 MW277600.1 |Severe acute respiratory syndrome coro    ....................  ....................  ....................  ....................  ....................  ....................
4916 MW277601.1 |Severe acute respiratory syndrome coro    ....................  ....................  ....................  ....................  ....................  ....................
4917 MW277602.1 |Severe acute respiratory syndrome coro    ....................  ....................  ....................  ....................  ....................  ....................
4919 MW277604.1 |Severe acute respiratory syndrome coro    ....................  ....................  ....................  ....................  ....................  ....................
4920 MW277607.1 |Severe acute respiratory syndrome coro    ....................  ....................  ....................  ....................  ....................  ....................
4921 MW277608.1 |Severe acute respiratory syndrome coro    ....................  ....................  ....................  ....................  ....................  ....................
4922 MW277609.1 |Severe acute respiratory syndrome coro    ....................  ....................  ....................  ....................  ....................  ....................
4923 MW277610.1 |Severe acute respiratory syndrome coro    ....................  ....................  ....................  ....................  ....................  ....................
4924 MW277611.1 |Severe acute respiratory syndrome coro    ....................  ....................  ....................  ....................  ....................  ....................
4925 MW277612.1 |Severe acute respiratory syndrome coro    ....................  ....................  ....................  ....................  ....................  ....................
4926 MW277613.1 |Severe acute respiratory syndrome coro    ....................  ....................  ....................  ....................  ....................  ....................
4927 MW277614.1 |Severe acute respiratory syndrome coro    ....................  ....................  ....................  ....................  ....................  ....................
4928 MW277615.1 |Severe acute respiratory syndrome coro    ....................  ....................  ....................  ....................  ....................  ....................
4929 MW277616.1 |Severe acute respiratory syndrome coro    ....................  ....................  ....................  ....................  ....................  ....................
4930 MW277617.1 |Severe acute respiratory syndrome coro    ....................  ....................  ....................  ....................  ....................  ....................
4931 MW277619.1 |Severe acute respiratory syndrome coro    ....................  ....................  ....................  ....................  ....................  ....................
4932 MW277620.1 |Severe acute respiratory syndrome coro    ....................  ....................  ....................  ....................  ....................  ....................
4933 MW277621.1 |Severe acute respiratory syndrome coro    ....................  ....................  ....................  ....................  ....................  ....................
4934 MW277622.1 |Severe acute respiratory syndrome coro    ....................  ....................  ....................  ....................  ....................  ....................
4935 MW277624.1 |Severe acute respiratory syndrome coro    ....................  ....................  ....................  ....................  ....................  ....................
4936 MW277625.1 |Severe acute respiratory syndrome coro    ....................  ......A.............  ....................  ....................  ....................  ....................
4937 MW277626.1 |Severe acute respiratory syndrome coro    ....................  ....................  ....................  ....................  ....................  ....................
4938 MW277628.1 |Severe acute respiratory syndrome coro    ....................  ....................  ....................  ....................  ....................  ....................
4939 MW277629.1 |Severe acute respiratory syndrome coro    ....................  ....................  ....................  ....................  ....................  ....................
4940 MW277630.1 |Severe acute respiratory syndrome coro    ....................  ....................  ....................  ....................  ....................  ....................
4941 MW277631.1 |Severe acute respiratory syndrome coro    ....................  ....................  ....................  ....................  ....................  ....................
4942 MW277632.1 |Severe acute respiratory syndrome coro    ....................  ....................  ....................  ....................  ....................  ....................
4943 MW277633.1 |Severe acute respiratory syndrome coro    ....................  ....................  ....................  ....................  ....................  ....................
4944 MW277635.1 |Severe acute respiratory syndrome coro    ....................  ....................  ....................  ....................  ....................  ....................
```

4945	MW277637.1	Severe acute respiratory syndrome coro	····················	····················	····················	····················	····················	····················	
4946	MW277638.1	Severe acute respiratory syndrome coro	····················	····················	····················	····················	····················	····················	
4947	MW277639.1	Severe acute respiratory syndrome coro	····················	····················	····················	····················	····················	····················	
4948	MW277640.1	Severe acute respiratory syndrome coro	····················	····················	····················	····················	····················	····················	
4949	MW277642.1	Severe acute respiratory syndrome coro	····················	····················	····················	····················	····················	····················	
4950	MW277643.1	Severe acute respiratory syndrome coro	····················	····················	····················	····················	····················	····················	
4951	MW277644.1	Severe acute respiratory syndrome coro	····················	····················	····················	····················	····················	····················	
4952	MW277645.1	Severe acute respiratory syndrome coro	····················	····················	····················	····················	····················	····················	
4953	MW277646.1	Severe acute respiratory syndrome coro	····················	····················	····················	····················	····················	····················	
4954	MW277647.1	Severe acute respiratory syndrome coro	····················	····················	····················	····················	····················	····················	
4955	MW277649.1	Severe acute respiratory syndrome coro	····················	····················	····················	····················	····················	····················	
4956	MW277650.1	Severe acute respiratory syndrome coro	····················	····················	····················	····················	····················	····················	
4957	MW277651.1	Severe acute respiratory syndrome coro	····················	····················	····················	····················	····················	····················	
4958	MW277652.1	Severe acute respiratory syndrome coro	····················	····················	····················	····················	····················	····················	
4959	MW277653.1	Severe acute respiratory syndrome coro	····················	····················	····················	····················	····················	····················	
4960	MW277654.1	Severe acute respiratory syndrome coro	····················	····················	····················	····················	····················	····················	
4961	MW277655.1	Severe acute respiratory syndrome coro	····················	····················	····················	····················	····················	····················	
4962	MW277656.1	Severe acute respiratory syndrome coro	····················	····················	····················	····················	····················	····················	
4963	MW277657.1	Severe acute respiratory syndrome coro	····················	····················	····················	····················	····················	····················	
4964	MW277658.1	Severe acute respiratory syndrome coro	····················	····················	····················	····················	····················	····················	
4965	MW277661.1	Severe acute respiratory syndrome coro	····················	····················	····················	····················	····················	····················	
4966	MW277662.1	Severe acute respiratory syndrome coro	····················	····················	····················	····················	····················	····················	
4967	MW279302.1	Severe acute respiratory syndrome coro	····················	····················	····················	····················	····················	···············A····	
4968	MW279303.1	Severe acute respiratory syndrome coro	····················	····················	····················	····················	····················	····················	
4969	MW279314.1	Severe acute respiratory syndrome coro	····················	····················	····················	····················	····················	····················	
4970	MW279315.1	Severe acute respiratory syndrome coro	····················	····················	····················	····················	····················	····················	
4971	MW279316.1	Severe acute respiratory syndrome coro	····················	····················	····················	····················	····················	····················	
4972	MW279317.1	Severe acute respiratory syndrome coro	····················	····················	····················	····················	····················	····················	
4973	MW279318.1	Severe acute respiratory syndrome coro	····················	····················	····················	····················	····················	····················	
4974	MW279319.1	Severe acute respiratory syndrome coro	····················	····················	····················	····················	····················	····················	
4975	MW279320.1	Severe acute respiratory syndrome coro	····················	····················	····················	····················	····················	····················	
4976	MW279321.1	Severe acute respiratory syndrome coro	····················	····················	····················	····················	····················	····················	
4977	MW279322.1	Severe acute respiratory syndrome coro	····················	····················	····················	····················	····················	····················	
4978	MW279323.1	Severe acute respiratory syndrome coro	····················	····················	····················	····················	····················	····················	
4979	MW279324.1	Severe acute respiratory syndrome coro	····················	····················	····················	····················	····················	····················	
4980	MW279325.1	Severe acute respiratory syndrome coro	····················	····················	····················	····················	····················	····················	
4981	MW279326.1	Severe acute respiratory syndrome coro	····················	·A··················	····················	····················	····················	····················	
4982	MW279327.1	Severe acute respiratory syndrome coro	····················	·A··················	····················	····················	····················	····················	
4983	MW279328.1	Severe acute respiratory syndrome coro	····················	·A··················	····················	····················	····················	····················	
4984	MW279329.1	Severe acute respiratory syndrome coro	····················	·A··················	····················	····················	NNN·················	····················	1
4985	MW279330.1	Severe acute respiratory syndrome coro	····················	·A··················	····················	····················	····················	····················	
4986	MW279331.1	Severe acute respiratory syndrome coro	····················	····················	····················	····················	····················	····················	
4987	MW279332.1	Severe acute respiratory syndrome coro	····················	·A··················	····················	····················	····················	····················	
4988	MW279333.1	Severe acute respiratory syndrome coro	····················	····················	····················	····················	····················	····················	
4989	MW279334.1	Severe acute respiratory syndrome coro	····················	····················	····················	····················	····················	····················	
4990	MW279335.1	Severe acute respiratory syndrome coro	····················	····················	····················	····················	····················	····················	
4991	MW279336.1	Severe acute respiratory syndrome coro	····················	····················	····················	····················	····················	····················	
4992	MW279337.1	Severe acute respiratory syndrome coro	····················	····················	····················	····················	····················	····················	
4993	MW279413.1	Severe acute respiratory syndrome coro	····················	····················	····················	····················	····················	····················	
4994	MW279414.1	Severe acute respiratory syndrome coro	····················	····················	····················	····················	····················	····················	
4995	MW279415.1	Severe acute respiratory syndrome coro	····················	····················	····················	····················	····················	····················	
4996	MW279416.1	Severe acute respiratory syndrome coro	····················	····················	····················	····················	····················	····················	
4997	MW279417.1	Severe acute respiratory syndrome coro	····················	····················	····················	····················	····················	····················	
4998	MW279418.1	Severe acute respiratory syndrome coro	····················	····················	····················	····················	····················	····················	
4999	MW279419.1	Severe acute respiratory syndrome coro	····················	····················	····················	····················	····················	····················	
5000	MW279420.1	Severe acute respiratory syndrome coro	····················	····················	····················	····················	····················	····················	
5001	MW279421.1	Severe acute respiratory syndrome coro	····················	····················	····················	····················	····················	····················	
5002	MW279422.1	Severe acute respiratory syndrome coro	····················	····················	····················	····················	····················	····················	
5003	MW279423.1	Severe acute respiratory syndrome coro	····················	····················	····················	····················	····················	····················	
5004	MW279424.1	Severe acute respiratory syndrome coro	····················	····················	····················	····················	····················	····················	
5005	MW279425.1	Severe acute respiratory syndrome coro	····················	····················	····················	····················	····················	····················	
5006	MW279426.1	Severe acute respiratory syndrome coro	····················	····················	····················	····················	····················	····················	
5007	MW279427.1	Severe acute respiratory syndrome coro	····················	····················	····················	····················	····················	····················	
5008	MW279428.1	Severe acute respiratory syndrome coro	····················	····················	····················	····················	····················	····················	
5009	MW279429.1	Severe acute respiratory syndrome coro	····················	····················	····················	····················	····················	····················	
5010	MW279430.1	Severe acute respiratory syndrome coro	····················	····················	····················	····················	····················	····················	
5011	MW279431.1	Severe acute respiratory syndrome coro	····················	····················	····················	····················	····················	····················	
5012	MW279432.1	Severe acute respiratory syndrome coro	····················	····················	····················	····················	····················	····················	
5013	MW279433.1	Severe acute respiratory syndrome coro	····················	····················	····················	····················	····················	····················	
5014	MW279434.1	Severe acute respiratory syndrome coro	····················	····················	····················	····················	····················	····················	
5015	MW279435.1	Severe acute respiratory syndrome coro	····················	····················	····················	····················	····················	····················	
5016	MW279436.1	Severe acute respiratory syndrome coro	····················	····················	····················	····················	····················	····················	
5017	MW279437.1	Severe acute respiratory syndrome coro	····················	····················	····················	····················	····················	····················	
5018	MW279438.1	Severe acute respiratory syndrome coro	····················	····················	····················	····················	····················	····················	
5019	MW279439.1	Severe acute respiratory syndrome coro	····················	····················	····················	····················	····················	····················	
5020	MW279440.1	Severe acute respiratory syndrome coro	····················	····················	····················	····················	····················	····················	
5021	MW279441.1	Severe acute respiratory syndrome coro	····················	····················	····················	····················	····················	····················	
5022	MW279442.1	Severe acute respiratory syndrome coro	····················	····················	····················	····················	····················	····················	

5023 MW279443.1 |Severe acute respiratory syndrome coro ···················· ···················· ···················· ···················· ···················· ····················
5024 MW279444.1 |Severe acute respiratory syndrome coro ···················· ···················· ···················· ···················· ···················· ····················
5025 MW279445.1 |Severe acute respiratory syndrome coro ···················· ···················· ···················· ···················· ···················· ····················
5026 MW279446.1 |Severe acute respiratory syndrome coro ···················· ···················· ···················· ···················· ···················· ····················
5027 MW279447.1 |Severe acute respiratory syndrome coro ···················· ···················· ···················· ···················· ···················· ····················
5028 MW279448.1 |Severe acute respiratory syndrome coro ···················· ···················· ···················· ···················· ···················· ····················
5029 MW279449.1 |Severe acute respiratory syndrome coro ···················· ···················· ···················· ···················· ···················· ····················
5030 MW279450.1 |Severe acute respiratory syndrome coro ···················· ···················· ···················· ···················· ···················· ····················
5031 MW279451.1 |Severe acute respiratory syndrome coro ···················· ···················· ···················· ···················· ···················· ····················
5032 MW279452.1 |Severe acute respiratory syndrome coro ···················· ···················· ···················· ···················· ···················· ····················
5033 MW279453.1 |Severe acute respiratory syndrome coro ···················· ···················· ···················· ···················· ···················· ····················
5034 MW279454.1 |Severe acute respiratory syndrome coro ···················· ···················· ···················· ···················· ···················· ····················
5035 MW279455.1 |Severe acute respiratory syndrome coro ···················· ·A·················· ···················· ···················· ···················· ····················
5036 MW279456.1 |Severe acute respiratory syndrome coro ···················· ···················· ···················· ···················· ···················· ····················
5037 MW279457.1 |Severe acute respiratory syndrome coro ···················· ···················· ···················· ···················· ···················· ····················
5038 MW279458.1 |Severe acute respiratory syndrome coro ···················· ···················· ···················· ···················· ···················· ····················
5039 MW279459.1 |Severe acute respiratory syndrome coro ···················· ···················· ···················· ···················· ···················· ····················
5040 MW279460.1 |Severe acute respiratory syndrome coro ···················· ···················· ···················· ···················· ···················· ····················
5041 MW280149.1 |Severe acute respiratory syndrome coro ···················· ···················· ···················· ···················· ···················· ····················
5042 MW280150.1 |Severe acute respiratory syndrome coro ···················· ·A·················· ···················· ···················· ···················· ····················
5043 MW280151.1 |Severe acute respiratory syndrome coro ···················· ···················· ···················· ···················· ···················· ····················
5044 MW280152.1 |Severe acute respiratory syndrome coro ···················· ···················· ···················· ···················· ···················· ····················
5045 MW280153.1 |Severe acute respiratory syndrome coro ···················· ···················· ···················· ···················· ···················· ····················
5046 MW280154.1 |Severe acute respiratory syndrome coro ···················· ·A·················· ···················· ···················· ···················· ····················
5047 MW280155.1 |Severe acute respiratory syndrome coro ···················· ···················· ···················· ···················· ···················· ····················
5048 MW280156.1 |Severe acute respiratory syndrome coro ···················· ···················· ···················· ···················· ···················· ····················
5049 MW280157.1 |Severe acute respiratory syndrome coro ···················· ···················· ···················· ···················· ···················· ····················
5050 MW280158.1 |Severe acute respiratory syndrome coro ···················· ············C······· ···················· ···················· ···················· ····················
5051 MW280159.1 |Severe acute respiratory syndrome coro ···················· ···················· ···················· ···················· ···················· ····················
5052 MW280162.1 |Severe acute respiratory syndrome coro ···················· ···················· ···················· ···················· ···················· ····················
5053 MW280163.1 |Severe acute respiratory syndrome coro ···················· ···················· ···················· ···················· ···················· ····················
5054 MW280165.1 |Severe acute respiratory syndrome coro ···················· ···················· ···················· ···················· ···················· ····················
5055 MW280166.1 |Severe acute respiratory syndrome coro ···················· ···················· ···················· ···················· ···················· ····················
5056 MW280167.1 |Severe acute respiratory syndrome coro ···················· ···················· ···················· ···················· ···················· ····················
5057 MW280168.1 |Severe acute respiratory syndrome coro ···················· ···················· ···················· ···················· ···················· ····················
5058 MW280169.1 |Severe acute respiratory syndrome coro ···················· ···················· ···················· ···················· ···················· ····················
5059 MW280170.1 |Severe acute respiratory syndrome coro ···················· ····A··············· ···················· ···················· ···················· ····················
5060 MW280171.1 |Severe acute respiratory syndrome coro ···················· ···················· ···················· ···················· ···················· ····················
5061 MW280172.1 |Severe acute respiratory syndrome coro ···················· ···················· ···················· ···················· ···················· ····················
5062 MW280173.1 |Severe acute respiratory syndrome coro ···················· ····A··············· ···················· ···················· ···················· ····················
5063 MW280174.1 |Severe acute respiratory syndrome coro ···················· ···················· ···················· ···················· ···················· ····················
5064 MW280175.1 |Severe acute respiratory syndrome coro ···················· ···················· ···················· ···················· ···················· ····················
5065 MW280176.1 |Severe acute respiratory syndrome coro ···················· ···················· ···················· ···················· ···················· ····················
5066 MW280177.1 |Severe acute respiratory syndrome coro ···················· ···················· ···················· ···················· ···················· ····················
5067 MW280178.1 |Severe acute respiratory syndrome coro ···················· ···················· ···················· ···················· ···················· ····················
5068 MW280179.1 |Severe acute respiratory syndrome coro ···················· ·A·················· ···················· ···················· ···················· ····················
5069 MW280180.1 |Severe acute respiratory syndrome coro ···················· ···················· ···················· ···················· ···················· ····················
5070 MW280181.1 |Severe acute respiratory syndrome coro ···················· ···················· ···················· ···················· ···················· ····················
5071 MW280182.1 |Severe acute respiratory syndrome coro ···················· ···················· ···················· ···················· ···················· ····················
5072 MW280183.1 |Severe acute respiratory syndrome coro ···················· ···················· ···················· ···················· ···················· ····················
5073 MW280184.1 |Severe acute respiratory syndrome coro ···················· ···················· ···················· ···················· ···················· ····················
5074 MW280185.1 |Severe acute respiratory syndrome coro ···················· ···················· ···················· ···················· ···················· ····················
5075 MW280187.1 |Severe acute respiratory syndrome coro ···················· ···················· ···················· ···················· ···················· ····················
5076 MW280450.1 |Severe acute respiratory syndrome coro ···················· ···················· ···················· ···················· ···················· ····················
5077 MW280451.1 |Severe acute respiratory syndrome coro ···················· ·A·················· ···················· ···················· ···················· ····················
5078 MW280452.1 |Severe acute respiratory syndrome coro ···················· ·A·················· ···················· ···················· ···················· ····················
5079 MW280453.1 |Severe acute respiratory syndrome coro ···················· ···················· ···················· ···················· ···················· ····················
5080 MW280454.1 |Severe acute respiratory syndrome coro ···················· ···················· ···················· ···················· ···················· ····················
5081 MW280456.1 |Severe acute respiratory syndrome coro ···················· ···················· ···················· ···················· ···················· ····················
5082 MW280457.1 |Severe acute respiratory syndrome coro ···················· ···················· ···················· ···················· ···················· ····················
5083 MW280458.1 |Severe acute respiratory syndrome coro ···················· ···················· ···················· ···················· ···················· ····················
5084 MW280459.1 |Severe acute respiratory syndrome coro ···················· ·A·················· ···················· ···················· ···················· ····················
5085 MW280460.1 |Severe acute respiratory syndrome coro ···················· ·A·················· ···················· ···················· ···················· ····················
5086 MW280461.1 |Severe acute respiratory syndrome coro ···················· ···················· ···················· ···················· ···················· ····················
5087 MW280462.1 |Severe acute respiratory syndrome coro ···················· ···················· ···················· ···················· ···················· ····················
5088 MW280464.1 |Severe acute respiratory syndrome coro ···················· ···················· ···················· ···················· ···················· ····················
5089 MW280465.1 |Severe acute respiratory syndrome coro ···················· ···················· ···················· ···················· ···················· ····················
5090 MW280467.1 |Severe acute respiratory syndrome coro ···················· ·A·················· ···········T········ ···················· ···················· ····················
5091 MW280468.1 |Severe acute respiratory syndrome coro ···················· ·A·················· ···················· ···················· ···················· ····················
5092 MW280469.1 |Severe acute respiratory syndrome coro ···················· ···················· ···················· ···················· ···················· ····················
5093 MW280470.1 |Severe acute respiratory syndrome coro ···················· ···················· ···················· ···················· ···················· ····················
5094 MW280471.1 |Severe acute respiratory syndrome coro ···················· ···················· ···················· ···················· ···················· ····················
5095 MW280472.1 |Severe acute respiratory syndrome coro ···················· ···················· ···················· ···················· ···················· ····················
5096 MW280473.1 |Severe acute respiratory syndrome coro ···················· ···················· ···················· ···················· ···················· ····················
5097 MW280474.1 |Severe acute respiratory syndrome coro ···················· ···················· ···················· ···················· ···················· ····················
5098 MW280475.1 |Severe acute respiratory syndrome coro ···················· ···················· ···················· ···················· ···················· ····················
5099 MW280476.1 |Severe acute respiratory syndrome coro ···················· ···················· ···················· ···················· ···················· ····················
5100 MW280478.1 |Severe acute respiratory syndrome coro ···················· ···················· ···················· ···················· ···················· ····················

```
5101 MW280479.1 |Severe acute respiratory syndrome coro   ....................  ....................  ....................  ....................  ....................  ....................
5102 MW280482.1 |Severe acute respiratory syndrome coro   ....................  .A..................  ....................  ....................  ....................  ....................
5103 MW280483.1 |Severe acute respiratory syndrome coro   ....................  ....................  ....................  ....................  ....................  ....................
5104 MW280485.1 |Severe acute respiratory syndrome coro   ....................  ....................  ....................  ....................  ....................  ....................
5105 MW280486.1 |Severe acute respiratory syndrome coro   ....................  ....................  ....................  ....................  ....................  ....................
5106 MW280487.1 |Severe acute respiratory syndrome coro   ....................  ....................  ....................  ....................  ....................  ....................
5107 MW280488.1 |Severe acute respiratory syndrome coro   ....................  .A..................  ....................  ....................  ....................  ....................
5108 MW280489.1 |Severe acute respiratory syndrome coro   ....................  .A..................  ....................  ....................  ....................  ....................
5109 MW280490.1 |Severe acute respiratory syndrome coro   ....................  ....................  ....................  ....................  ....................  ....................
5110 MW280491.1 |Severe acute respiratory syndrome coro   ....................  ....................  ....................  ....................  ....................  ....................
5111 MW280492.1 |Severe acute respiratory syndrome coro   ....................  ....................  ....................  ....................  ....................  ....................
5112 MW280493.1 |Severe acute respiratory syndrome coro   ....................  ....................  ....................  ....................  ....................  ....................
5113 MW280494.1 |Severe acute respiratory syndrome coro   ....................  ....................  ....................  ....................  ....................  ....................
5114 MW280495.1 |Severe acute respiratory syndrome coro   ....................  .A..................  ....................  ....................  ....................  ....................
5115 MW280496.1 |Severe acute respiratory syndrome coro   ....................  ....................  ....................  ....................  ....................  ....................
5116 MW280497.1 |Severe acute respiratory syndrome coro   ....................  ....................  ....................  ....................  ....................  ....................
5117 MW280498.1 |Severe acute respiratory syndrome coro   ....................  ....................  ....................  ....................  ....................  ....................
5118 MW280499.1 |Severe acute respiratory syndrome coro   ....................  .A..................  ....................  ....................  ....................  ....................
5119 MW280501.1 |Severe acute respiratory syndrome coro   ....................  ....................  ....................  ....................  ....................  ....................
5120 MW280502.1 |Severe acute respiratory syndrome coro   ....................  .A..................  ....................  ....................  ....................  ....................
5121 MW280503.1 |Severe acute respiratory syndrome coro   ....................  ....................  ....................  ....................  ....................  ....................
5122 MW280505.1 |Severe acute respiratory syndrome coro   ....................  ....................  ....................  ....................  ....................  ....................
5123 MW280507.1 |Severe acute respiratory syndrome coro   ....................  ....................  ....................  ....................  ....................  ....................
5124 MW280508.1 |Severe acute respiratory syndrome coro   ....................  ....................  ....................  ....................  ....................  ....................
5125 MW280509.1 |Severe acute respiratory syndrome coro   ....................  ....................  ....................  ....................  ....................  ....................
5126 MW280511.1 |Severe acute respiratory syndrome coro   ....................  ....................  ....................  ....................  ....................  ....................
5127 MW280512.1 |Severe acute respiratory syndrome coro   ....................  ....................  ....................  ....................  ....................  ....................
5128 MW280513.1 |Severe acute respiratory syndrome coro   ....................  ....................  ....................  ....................  ....................  ....................
5129 MW280516.1 |Severe acute respiratory syndrome coro   ....................  .A..................  ....................  ....................  ....................  ....................
5130 MW280517.1 |Severe acute respiratory syndrome coro   ....................  ....................  ....................  ....................  ....................  ....................
5131 MW280518.1 |Severe acute respiratory syndrome coro   ....................  ....................  ....................  ....................  ....................  ....................
5132 MW280519.1 |Severe acute respiratory syndrome coro   ....................  ....................  ....................  ....................  ....................  ....................
5133 MW280520.1 |Severe acute respiratory syndrome coro   ....................  ....................  ....................  ....................  ....................  ....................
5134 MW280521.1 |Severe acute respiratory syndrome coro   ....................  ....................  ....................  ....................  ....................  ....................
5135 MW280522.1 |Severe acute respiratory syndrome coro   ....................  .A..................  ....................  ....................  ....................  ....................
5136 MW280525.1 |Severe acute respiratory syndrome coro   ....................  ....................  ....................  ....................  ....................  ....................
5137 MW280527.1 |Severe acute respiratory syndrome coro   ....................  ....................  ....................  ....................  ....................  ....................
5138 MW280528.1 |Severe acute respiratory syndrome coro   ....................  ....................  ....................  ....................  ....................  ....................
5139 MW280529.1 |Severe acute respiratory syndrome coro   ....................  ....................  ....................  ....................  ....................  ....................
5140 MW280530.1 |Severe acute respiratory syndrome coro   ....................  ....................  ....................  ....................  ....................  ....................
5141 MW280531.1 |Severe acute respiratory syndrome coro   ....................  .............A......  ....................  ....................  ....................  ....................
5142 MW280533.1 |Severe acute respiratory syndrome coro   ....................  ....................  ....................  ....................  ....................  ....................
5143 MW280534.1 |Severe acute respiratory syndrome coro   ....................  ....................  ....................  ....................  ....................  ....................
5144 MW280537.1 |Severe acute respiratory syndrome coro   ....................  ....................  ....................  ....................  ....................  ....................
5145 MW280538.1 |Severe acute respiratory syndrome coro   ....................  ....................  ....................  ....................  ....................  ....................
5146 MW280539.1 |Severe acute respiratory syndrome coro   ....................  ....................  ....................  ....................  ....................  ....................
5147 MW280541.1 |Severe acute respiratory syndrome coro   ....................  .............A......  ....................  ....................  ....................  ....................
5148 MW280542.1 |Severe acute respiratory syndrome coro   ....................  .A..................  ....................  ....................  ....................  ....................
5149 MW280545.1 |Severe acute respiratory syndrome coro   ....................  .A..................  ....................  ....................  ....................  ....................
5150 MW280546.1 |Severe acute respiratory syndrome coro   ....................  ....................  ....................  ....................  ....................  ....................
5151 MW280547.1 |Severe acute respiratory syndrome coro   ....................  ....................  ....................  ....................  ....................  ....................
5152 MW280548.1 |Severe acute respiratory syndrome coro   ....................  ....................  ....................  ....................  ....................  ....................
5153 MW280549.1 |Severe acute respiratory syndrome coro   ....................  ....................  ....................  ....................  ....................  ....................
5154 MW280550.1 |Severe acute respiratory syndrome coro   ....................  ....................  ....................  ....................  ....................  ....................
5155 MW269882.1 |Severe acute respiratory syndrome coro   ....................  ....................  ....................  ....................  ....................  ....................
5156 MW269884.1 |Severe acute respiratory syndrome coro   ....................  ....................  ....................  ....................  ....................  ....................
5157 MW269885.1 |Severe acute respiratory syndrome coro   ....................  ....................  ....................  ....................  ....................  ....................
5158 MW269886.1 |Severe acute respiratory syndrome coro   ....................  ....................  ....................  ....................  ....................  ....................
5159 MW269887.1 |Severe acute respiratory syndrome coro   ....................  ....................  ....................  ....................  ....................  ....................
5160 MW269890.1 |Severe acute respiratory syndrome coro   ....................  ....................  ....................  ....................  ....................  ....................
5161 MW269891.1 |Severe acute respiratory syndrome coro   ....................  ....................  ....................  ....................  ....................  ....................
5162 MW269893.1 |Severe acute respiratory syndrome coro   ....................  ....................  ....................  ....................  ....................  ....................
5163 MW269895.1 |Severe acute respiratory syndrome coro   ....................  ....................  ....................  ....................  ....................  ....................
5164 MW269896.1 |Severe acute respiratory syndrome coro   ....................  ....................  ....................  ....................  ....................  ....................
5165 MW269898.1 |Severe acute respiratory syndrome coro   ....................  ....................  ....................  ....................  ....................  ....................
5166 MW269900.1 |Severe acute respiratory syndrome coro   ....................  ....................  ....................  ....................  ....................  ....................
5167 MW269902.1 |Severe acute respiratory syndrome coro   ....................  ....................  ....................  ....................  ....................  ....................
5168 MW269903.1 |Severe acute respiratory syndrome coro   ....................  ....................  ....................  ....................  ....................  ....................
5169 MW269904.1 |Severe acute respiratory syndrome coro   ....................  ....................  ....................  ....................  ....................  ....................
5170 MW273779.1 |Severe acute respiratory syndrome coro   ....................  ....................  ....................  ....................  ....................  ....................
5171 MW273783.1 |Severe acute respiratory syndrome coro   ....................  ....................  ....................  ....................  ....................  ....................
5172 MW273784.1 |Severe acute respiratory syndrome coro   ....................  ....................  ....................  ....................  ....................  ....................
5173 MW273785.1 |Severe acute respiratory syndrome coro   ....................  ....................  ....................  ....................  ....................  ....................
5174 MW273786.1 |Severe acute respiratory syndrome coro   ....................  ....................  ....................  ....................  ....................  ....................
5175 MW273787.1 |Severe acute respiratory syndrome coro   ....................  ....................  ....................  ....................  ....................  ....................
5176 MW273788.1 |Severe acute respiratory syndrome coro   ....................  ....................  ....................  ....................  ....................  ....................
5177 MW273789.1 |Severe acute respiratory syndrome coro   ....................  ....................  ....................  ....................  ....................  ....................
5178 MW273790.1 |Severe acute respiratory syndrome coro   ....................  ....................  ....................  ....................  ....................  ....................
```

5179	MW273791.1	|Severe acute respiratory syndrome coro	····················	····················	····················	····················	····················	····················
5180	MW273792.1	|Severe acute respiratory syndrome coro	····················	····················	····················	····················	····················	····················
5181	MW273793.1	|Severe acute respiratory syndrome coro	····················	····················	····················	····················	····················	····················
5182	MW273794.1	|Severe acute respiratory syndrome coro	····················	····················	····················	····················	····················	····················
5183	MW273795.1	|Severe acute respiratory syndrome coro	····················	····················	····················	····················	····················	····················
5184	MW273796.1	|Severe acute respiratory syndrome coro	····················	····················	····················	····················	····················	····················
5185	MW273797.1	|Severe acute respiratory syndrome coro	····················	····················	····················	····················	····················	····················
5186	MW273798.1	|Severe acute respiratory syndrome coro	····················	····················	····················	····················	····················	····················
5187	MW273799.1	|Severe acute respiratory syndrome coro	····················	····················	····················	····················	····················	····················
5188	MW273800.1	|Severe acute respiratory syndrome coro	····················	····················	····················	····················	····················	····················
5189	MW273801.1	|Severe acute respiratory syndrome coro	····················	····················	····················	····················	····················	····················
5190	MW273802.1	|Severe acute respiratory syndrome coro	····················	····················	····················	····················	····················	····················
5191	MW273803.1	|Severe acute respiratory syndrome coro	····················	····················	····················	····················	····················	····················
5192	MW273804.1	|Severe acute respiratory syndrome coro	····················	····················	····················	····················	····················	····················
5193	MW273805.1	|Severe acute respiratory syndrome coro	····················	····················	····················	····················	····················	····················
5194	MW273806.1	|Severe acute respiratory syndrome coro	····················	····················	····················	····················	····················	····················
5195	MW273807.1	|Severe acute respiratory syndrome coro	····················	····················	····················	····················	····················	····················
5196	MW273808.1	|Severe acute respiratory syndrome coro	····················	·A··················	····················	····················	····················	····················
5197	MW273809.1	|Severe acute respiratory syndrome coro	····················	·A··················	····················	····················	····················	····················
5198	MW273810.1	|Severe acute respiratory syndrome coro	····················	····················	····················	····················	····················	····················
5199	MW273811.1	|Severe acute respiratory syndrome coro	····················	····················	····················	····················	····················	····················
5200	MW273812.1	|Severe acute respiratory syndrome coro	····················	····················	····················	····················	····················	····················
5201	MW273813.1	|Severe acute respiratory syndrome coro	····················	····················	····················	····················	····················	····················
5202	MW273814.1	|Severe acute respiratory syndrome coro	····················	····················	····················	····················	····················	····················
5203	MW273815.1	|Severe acute respiratory syndrome coro	····················	····················	····················	····················	····················	····················
5204	MW273816.1	|Severe acute respiratory syndrome coro	····················	····················	····················	····················	····················	····················
5205	MW273817.1	|Severe acute respiratory syndrome coro	····················	····················	····················	····················	····················	····················
5206	MW273818.1	|Severe acute respiratory syndrome coro	····················	····················	····················	····················	····················	····················
5207	MW273819.1	|Severe acute respiratory syndrome coro	····················	····················	····················	····················	····················	····················
5208	MW273820.1	|Severe acute respiratory syndrome coro	····················	····················	····················	····················	····················	····················
5209	MW273821.1	|Severe acute respiratory syndrome coro	····················	····················	····················	····················	····················	····················
5210	MW273822.1	|Severe acute respiratory syndrome coro	····················	····················	····················	····················	····················	····················
5211	MW273823.1	|Severe acute respiratory syndrome coro	····················	····················	····················	····················	····················	····················
5212	MW273824.1	|Severe acute respiratory syndrome coro	····················	····················	····················	····················	····················	····················
5213	MW273825.1	|Severe acute respiratory syndrome coro	····················	····················	····················	····················	····················	····················
5214	MW273826.1	|Severe acute respiratory syndrome coro	····················	·A··················	····················	····················	····················	····················
5215	MW273827.1	|Severe acute respiratory syndrome coro	····················	····················	····················	····················	····················	····················
5216	MW273828.1	|Severe acute respiratory syndrome coro	····················	····················	····················	····················	····················	····················
5217	MW273829.1	|Severe acute respiratory syndrome coro	····················	····················	····················	····················	····················	····················
5218	MW273830.1	|Severe acute respiratory syndrome coro	····················	····················	····················	····················	····················	····················
5219	MW273831.1	|Severe acute respiratory syndrome coro	····················	····················	····················	····················	····················	····················
5220	MW273832.1	|Severe acute respiratory syndrome coro	····················	····················	····················	····················	····················	····················
5221	MW273833.1	|Severe acute respiratory syndrome coro	····················	····················	····················	····················	····················	····················
5222	MW273834.1	|Severe acute respiratory syndrome coro	····················	····················	····················	····················	····················	····················
5223	MW273835.1	|Severe acute respiratory syndrome coro	····················	····················	····················	····················	····················	····················
5224	MW273836.1	|Severe acute respiratory syndrome coro	····················	····················	····················	····················	····················	····················
5225	MW273837.1	|Severe acute respiratory syndrome coro	····················	····················	····················	····················	····················	····················
5226	MW273838.1	|Severe acute respiratory syndrome coro	····················	····················	····················	····················	····················	····················
5227	MW273839.1	|Severe acute respiratory syndrome coro	····················	····················	····················	····················	····················	····················
5228	MW273840.1	|Severe acute respiratory syndrome coro	····················	····················	····················	····················	····················	····················
5229	MW273841.1	|Severe acute respiratory syndrome coro	····················	····················	····················	····················	····················	····················
5230	MW273842.1	|Severe acute respiratory syndrome coro	····················	····················	····················	····················	····················	····················
5231	MW273843.1	|Severe acute respiratory syndrome coro	····················	····················	····················	····················	····················	····················
5232	MW273844.1	|Severe acute respiratory syndrome coro	····················	····················	····················	····················	····················	····················
5233	MW273845.1	|Severe acute respiratory syndrome coro	····················	····················	····················	····················	····················	····················
5234	MW273846.1	|Severe acute respiratory syndrome coro	····················	····················	····················	····················	····················	····················
5235	MW273847.1	|Severe acute respiratory syndrome coro	····················	····················	····················	····················	····················	····················
5236	MW273848.1	|Severe acute respiratory syndrome coro	····················	····················	····················	····················	····················	····················
5237	MW273849.1	|Severe acute respiratory syndrome coro	····················	····················	····················	····················	····················	····················
5238	MW273850.1	|Severe acute respiratory syndrome coro	····················	····················	····················	····················	····················	····················
5239	MW273851.1	|Severe acute respiratory syndrome coro	····················	····················	····················	····················	····················	····················
5240	MW273852.1	|Severe acute respiratory syndrome coro	····················	····················	····················	····················	····················	····················
5241	MW273853.1	|Severe acute respiratory syndrome coro	····················	····················	····················	····················	····················	····················
5242	MW273854.1	|Severe acute respiratory syndrome coro	····················	····················	····················	····················	····················	····················
5243	MW273855.1	|Severe acute respiratory syndrome coro	····················	····················	····················	····················	····················	····················
5244	MW273856.1	|Severe acute respiratory syndrome coro	····················	····················	····················	····················	····················	····················
5245	MW273857.1	|Severe acute respiratory syndrome coro	····················	····················	····················	····················	····················	····················
5246	MW273858.1	|Severe acute respiratory syndrome coro	····················	····················	····················	····················	····················	····················
5247	MW273859.1	|Severe acute respiratory syndrome coro	····················	····················	····················	····················	····················	····················
5248	MW273860.1	|Severe acute respiratory syndrome coro	····················	····················	····················	····················	····················	····················
5249	MW273861.1	|Severe acute respiratory syndrome coro	····················	····················	····················	····················	····················	····················
5250	MW273862.1	|Severe acute respiratory syndrome coro	····················	····················	····················	····················	····················	····················
5251	MW273863.1	|Severe acute respiratory syndrome coro	····················	····················	····················	····················	····················	····················
5252	MW273864.1	|Severe acute respiratory syndrome coro	····················	····················	····················	····················	····················	····················
5253	MW273865.1	|Severe acute respiratory syndrome coro	····················	····················	····················	····················	····················	····················
5254	MW273866.1	|Severe acute respiratory syndrome coro	····················	····················	····················	····················	····················	····················
5255	MW273867.1	|Severe acute respiratory syndrome coro	····················	····················	····················	····················	····················	····················
5256	MW273868.1	|Severe acute respiratory syndrome coro	····················	····················	····················	····················	····················	····················

5257	MW273869.1 |Severe acute respiratory syndrome coro	····················	····················	····················	····················	····················	····················
5258	MW273870.1 |Severe acute respiratory syndrome coro	····················	····················	····················	····················	····················	····················
5259	MW273871.1 |Severe acute respiratory syndrome coro	····················	····················	····················	····················	····················	····················
5260	MW273872.1 |Severe acute respiratory syndrome coro	····················	····················	····················	····················	····················	····················
5261	MW273873.1 |Severe acute respiratory syndrome coro	····················	····················	····················	····················	····················	····················
5262	MW273874.1 |Severe acute respiratory syndrome coro	····················	····················	····················	····················	····················	····················
5263	MW273875.1 |Severe acute respiratory syndrome coro	····················	····················	····················	····················	····················	····················
5264	MW273876.1 |Severe acute respiratory syndrome coro	····················	····················	····················	····················	····················	····················
5265	MW273877.1 |Severe acute respiratory syndrome coro	····················	····················	····················	····················	····················	····················
5266	MW273878.1 |Severe acute respiratory syndrome coro	····················	····················	····················	····················	····················	····················
5267	MW273879.1 |Severe acute respiratory syndrome coro	····················	····················	····················	····················	····················	····················
5268	MW273880.1 |Severe acute respiratory syndrome coro	····················	····················	····················	····················	····················	····················
5269	MW273881.1 |Severe acute respiratory syndrome coro	····················	····················	····················	····················	····················	····················
5270	MW273882.1 |Severe acute respiratory syndrome coro	····················	····················	····················	····················	····················	····················
5271	MW273883.1 |Severe acute respiratory syndrome coro	····················	····················	····················	····················	····················	····················
5272	MW273884.1 |Severe acute respiratory syndrome coro	····················	····················	····················	····················	····················	····················
5273	MW273885.1 |Severe acute respiratory syndrome coro	····················	····················	····················	····················	····················	····················
5274	MW273886.1 |Severe acute respiratory syndrome coro	····················	····················	····················	····················	····················	····················
5275	MW273887.1 |Severe acute respiratory syndrome coro	····················	····················	····················	····················	····················	····················
5276	MW273888.1 |Severe acute respiratory syndrome coro	····················	····················	····················	····················	····················	····················
5277	MW273889.1 |Severe acute respiratory syndrome coro	····················	····················	····················	····················	····················	····················
5278	MW273890.1 |Severe acute respiratory syndrome coro	····················	····················	····················	····················	····················	····················
5279	MW273891.1 |Severe acute respiratory syndrome coro	····················	····················	····················	····················	····················	····················
5280	MW273892.1 |Severe acute respiratory syndrome coro	····················	····················	····················	····················	····················	····················
5281	MW265603.1 |Severe acute respiratory syndrome coro	····················	····················	····················	····················	····················	····················
5282	MW266938.1 |Severe acute respiratory syndrome coro	····················	····················	····················	····················	····················	····················
5283	MW266939.1 |Severe acute respiratory syndrome coro	····················	····················	····················	····················	····················	····················
5284	MW266940.1 |Severe acute respiratory syndrome coro	····················	····················	····················	····················	····················	····················
5285	MW266941.1 |Severe acute respiratory syndrome coro	····················	····················	····················	····················	····················	····················
5286	MW266942.1 |Severe acute respiratory syndrome coro	····················	····················	····················	····················	····················	····················
5287	MW266943.1 |Severe acute respiratory syndrome coro	····················	····················	····················	····················	····················	····················
5288	MW266944.1 |Severe acute respiratory syndrome coro	····················	····················	····················	····················	····················	····················
5289	MW266945.1 |Severe acute respiratory syndrome coro	····················	····················	····················	····················	····················	····················
5290	MW266946.1 |Severe acute respiratory syndrome coro	····················	····················	····················	····················	····················	····················
5291	MW266947.1 |Severe acute respiratory syndrome coro	····················	····················	····················	····················	····················	····················
5292	MW266948.1 |Severe acute respiratory syndrome coro	····················	····················	····················	····················	····················	····················
5293	MW266949.1 |Severe acute respiratory syndrome coro	····················	····················	····················	····················	····················	····················
5294	MW266950.1 |Severe acute respiratory syndrome coro	····················	····················	····················	····················	····················	····················
5295	MW266951.1 |Severe acute respiratory syndrome coro	····················	····················	····················	····················	····················	····················
5296	MW266952.1 |Severe acute respiratory syndrome coro	····················	····················	····················	····················	····················	····················
5297	MW266953.1 |Severe acute respiratory syndrome coro	····················	····················	····················	····················	····················	····················
5298	MW266954.1 |Severe acute respiratory syndrome coro	····················	····················	····················	····················	····················	····················
5299	MW266955.1 |Severe acute respiratory syndrome coro	····················	····················	····················	····················	····················	····················
5300	MW266956.1 |Severe acute respiratory syndrome coro	····················	····················	····················	····················	····················	····················
5301	MW267131.1 |Severe acute respiratory syndrome coro	····················	····················	····················	····················	····················	····················
5302	MW268703.1 |Severe acute respiratory syndrome coro	····················	····················	····················	····················	····················	····················
5303	MW268704.1 |Severe acute respiratory syndrome coro	····················	····················	····················	····················	····················	····················
5304	MW268705.1 |Severe acute respiratory syndrome coro	····················	····················	····················	····················	····················	····················
5305	MW268706.1 |Severe acute respiratory syndrome coro	····················	····················	····················	····················	····················	····················
5306	MW268707.1 |Severe acute respiratory syndrome coro	···T················	····················	····················	····················	····················	····················
5307	MW268708.1 |Severe acute respiratory syndrome coro	···T················	····················	····················	····················	····················	····················
5308	MW268709.1 |Severe acute respiratory syndrome coro	····················	····················	····················	····················	····················	····················
5309	MW268710.1 |Severe acute respiratory syndrome coro	····················	·A··················	····················	····················	····················	····················
5310	MW268711.1 |Severe acute respiratory syndrome coro	····················	····················	····················	····················	····················	····················
5311	MW268712.1 |Severe acute respiratory syndrome coro	····················	····················	····················	····················	····················	····················
5312	MW268713.1 |Severe acute respiratory syndrome coro	····················	····················	····················	····················	····················	····················
5313	MW268715.1 |Severe acute respiratory syndrome coro	····················	····················	····················	····················	····················	····················
5314	MW268716.1 |Severe acute respiratory syndrome coro	····················	····················	····················	····················	····················	····················
5315	MW268717.1 |Severe acute respiratory syndrome coro	····················	····················	····················	····················	····················	····················
5316	MW268718.1 |Severe acute respiratory syndrome coro	····················	····················	····················	····················	····················	····················
5317	MW268720.1 |Severe acute respiratory syndrome coro	····················	····················	····················	····················	····················	····················
5318	MW268721.1 |Severe acute respiratory syndrome coro	····················	····················	····················	····················	····················	····················
5319	MW268722.1 |Severe acute respiratory syndrome coro	····················	····················	····················	····················	····················	····················
5320	MW268723.1 |Severe acute respiratory syndrome coro	····················	····················	····················	····················	····················	····················
5321	MW268724.1 |Severe acute respiratory syndrome coro	····················	····················	····················	····················	····················	····················
5322	MW268725.1 |Severe acute respiratory syndrome coro	····················	·············A······	····················	····················	····················	····················
5323	MW268726.1 |Severe acute respiratory syndrome coro	····················	····················	····················	····················	····················	····················
5324	MW268727.1 |Severe acute respiratory syndrome coro	····················	····················	····················	····················	····················	····················
5325	MW268728.1 |Severe acute respiratory syndrome coro	····················	····················	····················	····················	····················	····················
5326	MW264424.1 |Severe acute respiratory syndrome coro	····················	····················	····················	····················	····················	····················
5327	MW264435.1 |Severe acute respiratory syndrome coro	····················	····················	····················	····················	····················	····················
5328	MT982401.1 |Severe acute respiratory syndrome coro	····················	····················	····················	····················	····················	····················
5329	MT982402.1 |Severe acute respiratory syndrome coro	····················	····················	····················	····················	····················	····················
5330	MT982403.1 |Severe acute respiratory syndrome coro	····················	····················	····················	····················	····················	····················
5331	MT982404.1 |Severe acute respiratory syndrome coro	····················	····················	····················	····················	····················	····················
5332	MT982405.1 |Severe acute respiratory syndrome coro	····················	····················	····················	····················	····················	····················
5333	MT982406.1 |Severe acute respiratory syndrome coro	····················	····················	····················	····················	····················	····················
5334	MW255832.1 |Severe acute respiratory syndrome coro	····················	····················	····················	···········T········	····················	····················

5335 MW261767.1 |Severe acute respiratory syndrome coro ···················· ···················· ···················· ···················· ···················· ····················
5336 MW261770.1 |Severe acute respiratory syndrome coro ···················· ···················· ···················· ···················· ···················· ····················
5337 MW250244.1 |Severe acute respiratory syndrome coro ···················· ···················· ···················· ···················· ···················· ····················
5338 MW250352.1 |Severe acute respiratory syndrome coro ···················· ···················· ···················· ···················· ···················· ····················
5339 MW251511.1 |Severe acute respiratory syndrome coro ···················· ···················· ···················· ···················· ···················· ····················
5340 MW242950.1 |Severe acute respiratory syndrome coro ···················· ·A·················· ···················· ···················· ···················· ···A················
5341 MW242960.1 |Severe acute respiratory syndrome coro ···················· ·A·················· ···················· ···················· ···················· ···A················
5342 MW242961.1 |Severe acute respiratory syndrome coro ···················· ·A·················· ···················· ···················· ···················· ···A················
5343 MW242962.1 |Severe acute respiratory syndrome coro ···················· ···················· ···················· ···················· ···················· ····················
5344 MW242963.1 |Severe acute respiratory syndrome coro ···················· ·A·················· ···················· ···················· ···················· ···A················
5345 MW242964.1 |Severe acute respiratory syndrome coro ···················· ·A·················· ···················· ···················· ···················· ···A················
5346 MW242965.1 |Severe acute respiratory syndrome coro ···················· ·A·················· ···················· ···················· ···················· ···A················
5347 MW242999.1 |Severe acute respiratory syndrome coro ···················· ···················· ···················· ···················· ···················· ····················
5348 MW243003.1 |Severe acute respiratory syndrome coro ···················· ···················· ···················· ···················· ···················· ····················
5349 MW243586.1 |Severe acute respiratory syndrome coro ···················· ·A·················· ···················· ···················· ···················· ···A················
5350 MW243587.1 |Severe acute respiratory syndrome coro ···················· ·A·················· ···················· ···················· ···················· ···A················
5351 MW243995.1 |Severe acute respiratory syndrome coro ···················· ···················· ···················· ···················· ···················· ····················
5352 MW243996.1 |Severe acute respiratory syndrome coro ···················· ···················· ···················· ···················· ···················· ····················
5353 MW243997.1 |Severe acute respiratory syndrome coro ···················· ···················· ···················· ···················· ···················· ····················
5354 MW243998.1 |Severe acute respiratory syndrome coro ···················· ···················· ···················· ···················· ···················· ····················
5355 MW243999.1 |Severe acute respiratory syndrome coro ···················· ···················· ···················· ···················· ···················· ····················
5356 MW244000.1 |Severe acute respiratory syndrome coro ···················· ···················· ···················· ···················· ···················· ····················
5357 MW244001.1 |Severe acute respiratory syndrome coro ···················· ···················· ···················· ···················· ···················· ····················
5358 MW244002.1 |Severe acute respiratory syndrome coro ···················· ···················· ···················· ···················· ···················· ····················
5359 MW244003.1 |Severe acute respiratory syndrome coro ···················· ···················· ···················· ···················· ···················· ····················
5360 MW244004.1 |Severe acute respiratory syndrome coro ···················· ···················· ···················· ···················· ···················· ····················
5361 MW244005.1 |Severe acute respiratory syndrome coro ···················· ···················· ···················· ···················· ···················· ····················
5362 MW244006.1 |Severe acute respiratory syndrome coro ···················· ···················· ···················· ···················· ···················· ····················
5363 MW244007.1 |Severe acute respiratory syndrome coro ···················· ···················· ···················· ···················· ···················· ····················
5364 MW244008.1 |Severe acute respiratory syndrome coro ···················· ···················· ···················· ···················· ···················· ····················
5365 MW244009.1 |Severe acute respiratory syndrome coro ···················· ···················· ···················· ···················· ···················· ····················
5366 MW244010.1 |Severe acute respiratory syndrome coro ···················· ···················· ···················· ···················· ···················· ····················
5367 MW244011.1 |Severe acute respiratory syndrome coro ···················· ·A·················· ···················· ···················· ···················· ····················
5368 MW244012.1 |Severe acute respiratory syndrome coro ···················· ·A·················· ···················· ···················· ···················· ····················
5369 MW244013.1 |Severe acute respiratory syndrome coro ···················· ···················· ···················· ···················· ···················· ····················
5370 MW244014.1 |Severe acute respiratory syndrome coro ···················· ·····A·············· ···················· ···················· ···················· ····················
5371 MW244015.1 |Severe acute respiratory syndrome coro ···················· ···················· ···················· ···················· ···················· ····················
5372 MW244016.1 |Severe acute respiratory syndrome coro ···················· ···················· ···················· ···················· ···················· ····················
5373 MW244017.1 |Severe acute respiratory syndrome coro ···················· ············C······· ···················· ···················· ···················· ····················
5374 MW244018.1 |Severe acute respiratory syndrome coro ···················· ·A·················· ···················· ···················· ···················· ····················
5375 MW244019.1 |Severe acute respiratory syndrome coro ···················· ···················· ···················· ···················· ···················· ····················
5376 MW246592.1 |Severe acute respiratory syndrome coro ···················· ···················· ···················· ···················· ···················· ····················
5377 MW246593.1 |Severe acute respiratory syndrome coro ···················· ···················· ···················· ···················· ···················· ····················
5378 MW246594.1 |Severe acute respiratory syndrome coro ···················· ···················· ···················· ···················· ···················· ····················
5379 MW246595.1 |Severe acute respiratory syndrome coro ···················· ·············A······ ···················· ···················· ·NNN················ ···················· 1
5380 MW246596.1 |Severe acute respiratory syndrome coro ···················· ·············A······ ···················· ···················· ···················· ····················
5381 MW246597.1 |Severe acute respiratory syndrome coro ···················· ·A·················· ···················· ···················· ···················· ····················
5382 MW246598.1 |Severe acute respiratory syndrome coro ···················· ·············A······ ···················· ···················· ···················· ····················
5383 MW246599.1 |Severe acute respiratory syndrome coro ···················· ·············A······ ···················· ···················· ···················· ····················
5384 MW246600.1 |Severe acute respiratory syndrome coro ···················· ·············A······ ···················· ···················· ···················· ····················
5385 MW246601.1 |Severe acute respiratory syndrome coro ···················· ·············A······ ···················· ···················· ···················· ····················
5386 MW246602.1 |Severe acute respiratory syndrome coro ···················· ···················· ···················· ···················· ···················· ····················
5387 MW246603.1 |Severe acute respiratory syndrome coro ···················· ···················· ···················· ···················· ···················· ····················
5388 MW246604.1 |Severe acute respiratory syndrome coro ···················· ···················· ···················· ···················· ···················· ····················
5389 MW246605.1 |Severe acute respiratory syndrome coro ···················· ···················· ···················· ···················· ···················· ····················
5390 MW246606.1 |Severe acute respiratory syndrome coro ···················· ···················· ···················· ···················· ···················· ····················
5391 MW246607.1 |Severe acute respiratory syndrome coro ···················· ···················· ···················· ···················· ···················· ····················
5392 MW246608.1 |Severe acute respiratory syndrome coro ···················· ···················· ···················· ···················· ···················· ····················
5393 MW246609.1 |Severe acute respiratory syndrome coro ···················· ·············A······ ···················· ···················· ···················· ····················
5394 MW246610.1 |Severe acute respiratory syndrome coro ···················· ···················· ···················· ···················· ···················· ····················
5395 MW246611.1 |Severe acute respiratory syndrome coro ···················· ···················· ···················· ···················· ···················· ····················
5396 MW246612.1 |Severe acute respiratory syndrome coro ···················· ···················· ···················· ···················· ···················· ····················
5397 MW246613.1 |Severe acute respiratory syndrome coro ···················· ···················· ···················· ···················· ···················· ····················
5398 MW246614.1 |Severe acute respiratory syndrome coro ···················· ·············A······ ···················· ···················· ···················· ····················
5399 MW246615.1 |Severe acute respiratory syndrome coro ···················· ·············A······ ···················· ···················· ···················· ····················
5400 MW246616.1 |Severe acute respiratory syndrome coro ···················· ·············A······ ···················· ···················· ···················· ····················
5401 MW246617.1 |Severe acute respiratory syndrome coro ···················· ·············A······ ···················· ···················· ···················· ····················
5402 MW246618.1 |Severe acute respiratory syndrome coro ···················· ·············A······ ···················· ···················· ···················· ····················
5403 MW246619.1 |Severe acute respiratory syndrome coro ···················· ·············A······ ···················· ···················· ···················· ····················
5404 MW246620.1 |Severe acute respiratory syndrome coro ···················· ·············A······ ···················· ···················· ···················· ····················
5405 MW246621.1 |Severe acute respiratory syndrome coro ···················· ·············A······ ···················· ···················· ···················· ····················
5406 MW246622.1 |Severe acute respiratory syndrome coro ···················· ·············A······ ···················· ···················· ···················· ····················
5407 MW246623.1 |Severe acute respiratory syndrome coro ···················· ·············A······ ···················· ···················· ···················· ····················
5408 MW246624.1 |Severe acute respiratory syndrome coro ···················· ·············A······ ···················· ···················· ···················· ····················
5409 MW246625.1 |Severe acute respiratory syndrome coro ···················· ·············A······ ···················· ···················· ···················· ····················
5410 MW246626.1 |Severe acute respiratory syndrome coro ···················· ·············A······ ···················· ···················· ··N················· ····················
5411 MW246627.1 |Severe acute respiratory syndrome coro ···················· ·············A······ ···················· ···················· ···················· ····················
5412 MW246628.1 |Severe acute respiratory syndrome coro ···················· ·············A······ ···················· ···················· ···················· ····················

```
5413 MW246629.1 |Severe acute respiratory syndrome coro    ····················  ·A··················  ·····················  ····················  ····················  ····················
5414 MW242647.1 |Severe acute respiratory syndrome coro    ····················  ·A··················  ·····················  ····················  ····················  ····················
5415 MW242649.1 |Severe acute respiratory syndrome coro    ····················  ····················  ·····················  ····················  ····················  ····················
5416 MW242650.1 |Severe acute respiratory syndrome coro    ····················  ····················  ·····················  ····················  ····················  ····················
5417 MW242651.1 |Severe acute respiratory syndrome coro    ····················  ····················  ·····················  ····················  ····················  ····················
5418 MW242652.1 |Severe acute respiratory syndrome coro    ····················  ····················  ·····················  ····················  ····················  ····················
5419 MW242653.1 |Severe acute respiratory syndrome coro    ····················  ·A··················  ·····················  ····················  ····················  ···A················
5420 MW242655.1 |Severe acute respiratory syndrome coro    ····················  ····················  ·····················  ····················  ····················  ····················
5421 MW242663.1 |Severe acute respiratory syndrome coro    ····················  ·A··················  ·····················  ····················  ····················  ···A················
5422 MW242666.1 |Severe acute respiratory syndrome coro    ····················  ·A··················  ·····················  ····················  ····················  ···A················
5423 MW242667.1 |Severe acute respiratory syndrome coro    ····················  ····················  ·····················  ····················  ····················  ····················
5424 MW242670.1 |Severe acute respiratory syndrome coro    ····················  ·A··················  ·····················  ····················  ····················  ···A················
5425 MW242671.1 |Severe acute respiratory syndrome coro    ····················  ·A··················  ·····················  ····················  ····················  ···A················
5426 MW242689.1 |Severe acute respiratory syndrome coro    ····················  ·A··················  ·····················  ····················  ····················  ····················
5427 MW242691.1 |Severe acute respiratory syndrome coro    ····················  ····················  ·····················  ····················  ····················  ····················
5428 MW242692.1 |Severe acute respiratory syndrome coro    ····················  ····················  ·····················  ····················  ····················  ····················
5429 MW242704.1 |Severe acute respiratory syndrome coro    ····················  ····················  ·····················  ····················  ····················  ····················
5430 MW242705.1 |Severe acute respiratory syndrome coro    ····················  ····················  ·····················  ····················  ····················  ····················
5431 MW242706.1 |Severe acute respiratory syndrome coro    ····················  ·A··················  ·····················  ····················  ····················  ····················
5432 MW242707.1 |Severe acute respiratory syndrome coro    ····················  ····················  ·····················  ····················  ····················  ····················
5433 MW242708.1 |Severe acute respiratory syndrome coro    ····················  ····················  ·····················  ····················  ····················  ···A················
5434 MW242709.1 |Severe acute respiratory syndrome coro    ····················  ····················  ·····················  ····················  ····················  ····················
5435 MW242710.1 |Severe acute respiratory syndrome coro    ····················  ····················  ·····················  ····················  ····················  ····················
5436 MW242778.1 |Severe acute respiratory syndrome coro    ····················  ·A··················  ·····················  ····················  ····················  ···A················
5437 MW242779.1 |Severe acute respiratory syndrome coro    ····················  ·A··················  ·····················  ····················  ····················  ···A················
5438 MW242780.1 |Severe acute respiratory syndrome coro    ····················  ····················  ·····················  ····················  ····················  ····················
5439 MW242781.1 |Severe acute respiratory syndrome coro    ····················  ····················  ·····················  ····················  ····················  ····················
5440 MW242786.1 |Severe acute respiratory syndrome coro    ····················  ····················  ·····················  ····················  ····················  ····················
5441 MW185823.1 |Severe acute respiratory syndrome coro    ····················  ····················  ·····················  ····················  ····················  ····················
5442 MW185824.1 |Severe acute respiratory syndrome coro    ····················  ····················  ·····················  ····················  ····················  ····················
5443 MW185825.1 |Severe acute respiratory syndrome coro    ····················  ····················  ·····················  ····················  ····················  ····················
5444 MW185826.1 |Severe acute respiratory syndrome coro    ····················  ····················  ·····················  ····················  ····················  ····················
5445 MW185827.1 |Severe acute respiratory syndrome coro    ····················  ····················  ·····················  ····················  ····················  ····················
5446 MW237708.1 |Severe acute respiratory syndrome coro    ····················  ····················  ·····················  ····················  ····················  ····················
5447 MW237714.1 |Severe acute respiratory syndrome coro    ····················  ····················  ·····················  ····················  ····················  ····················
5448 MW237715.1 |Severe acute respiratory syndrome coro    ····················  ····················  ·····················  ····················  ····················  ····················
5449 MW237717.1 |Severe acute respiratory syndrome coro    ····················  ····················  ·····················  ····················  ····················  ····················
5450 MW237738.1 |Severe acute respiratory syndrome coro    ····················  ····················  ·····················  ····················  ····················  ····················
5451 MW237739.1 |Severe acute respiratory syndrome coro    ····················  ····················  ·····················  ····················  ····················  ····················
5452 MW237740.1 |Severe acute respiratory syndrome coro    ····················  ····················  ·····················  ····················  ····················  ····················
5453 MW237741.1 |Severe acute respiratory syndrome coro    ····················  ····················  ·····················  ····················  ····················  ····················
5454 MW237743.1 |Severe acute respiratory syndrome coro    ····················  ····················  ·····················  ····················  ····················  ····················
5455 MW237767.1 |Severe acute respiratory syndrome coro    ····················  ····················  ·····················  ····················  ····················  ····················
5456 MW237768.1 |Severe acute respiratory syndrome coro    ····················  ····················  ·····················  ····················  ····················  ····················
5457 MW240719.1 |Severe acute respiratory syndrome coro    ····················  ····················  ·····················  ····················  ····················  ····················
5458 MW240720.1 |Severe acute respiratory syndrome coro    ····················  ····················  ·····················  ····················  ····················  ····················
5459 MW240721.1 |Severe acute respiratory syndrome coro    ····················  ····················  ·····················  ····················  ····················  ····················
5460 MW240723.1 |Severe acute respiratory syndrome coro    ····················  ·············A······  ·····················  ····················  ····················  ····················
5461 MW240724.1 |Severe acute respiratory syndrome coro    ····················  ·A··················  ·····················  ····················  ····················  ····················
5462 MW240725.1 |Severe acute respiratory syndrome coro    ····················  ·A··················  ·····················  ····················  ····················  ····················
5463 MW240726.1 |Severe acute respiratory syndrome coro    ····················  ·A··················  ·····················  ····················  ····················  ····················
5464 MW240727.1 |Severe acute respiratory syndrome coro    ····················  ·A··················  ·····················  ····················  ····················  ····················
5465 MW240728.1 |Severe acute respiratory syndrome coro    ····················  ·A··················  ·····················  ····················  ····················  ····················
5466 MW240729.1 |Severe acute respiratory syndrome coro    ····················  ·A··················  ·····················  ····················  ····················  ····················
5467 MW240730.1 |Severe acute respiratory syndrome coro    ····················  ·A··················  ·····················  ····················  ····················  ····················
5468 MW240731.1 |Severe acute respiratory syndrome coro    ····················  ·A··················  ·····················  ····················  ····················  ····················
5469 MW240732.1 |Severe acute respiratory syndrome coro    ····················  ·A··················  ·····················  ····················  ····················  ····················
5470 MW240733.1 |Severe acute respiratory syndrome coro    ····················  ·A··················  ·····················  ····················  ····················  ····················
5471 MW240734.1 |Severe acute respiratory syndrome coro    ····················  ·A··················  ·····················  ····················  ····················  ····················
5472 MW240735.1 |Severe acute respiratory syndrome coro    ····················  ·A··················  ·····················  ····················  ····················  ····················
5473 MW240736.1 |Severe acute respiratory syndrome coro    ····················  ·A··················  ·····················  ····················  ····················  ····················
5474 MW240737.1 |Severe acute respiratory syndrome coro    ····················  ·A··················  ·····················  ····················  ····················  ····················
5475 MW240738.1 |Severe acute respiratory syndrome coro    ····················  ·A··················  ·····················  ····················  ····················  ····················
5476 MW240739.1 |Severe acute respiratory syndrome coro    ····················  ·A··················  ·····················  ····················  ····················  ····················
5477 MW240740.1 |Severe acute respiratory syndrome coro    ····················  ·A··················  ·····················  ····················  ····················  ····················
5478 MW240741.1 |Severe acute respiratory syndrome coro    ····················  ·A··················  ·····················  ····················  ····················  ····················
5479 MW240742.1 |Severe acute respiratory syndrome coro    ····················  ·A··················  ·····················  ····················  ····················  ····················
5480 MW240743.1 |Severe acute respiratory syndrome coro    ····················  ····················  ·····················  ····················  ····················  ····················
5481 MW240744.1 |Severe acute respiratory syndrome coro    ····················  ····················  ·····················  ····················  ····················  ····················
5482 MW240745.1 |Severe acute respiratory syndrome coro    ····················  ····················  ·····················  ····················  ····················  ····················
5483 MW240746.1 |Severe acute respiratory syndrome coro    ····················  ····················  ·····················  ····················  ····················  ····················
5484 MW240747.1 |Severe acute respiratory syndrome coro    ····················  ·A··················  ·····················  ····················  ····················  ····················
5485 MW240748.1 |Severe acute respiratory syndrome coro    ····················  ····················  ·····················  ····················  ····················  ····················
5486 MW240749.1 |Severe acute respiratory syndrome coro    ····················  ····················  ·····················  ····················  ····················  ····················
5487 MW240750.1 |Severe acute respiratory syndrome coro    ····················  ····················  ·····················  ····················  ····················  ····················
5488 MW240751.1 |Severe acute respiratory syndrome coro    ····················  ····················  ·····················  ····················  ····················  ····················
5489 MW240752.1 |Severe acute respiratory syndrome coro    ····················  ····················  ·····················  ····················  ····················  ····················
5490 MW240753.1 |Severe acute respiratory syndrome coro    ····················  ············C·······  ·····················  ····················  ····················  ····················
```

5491	MW240754.1	|Severe acute respiratory syndrome coro	····················	·A··················	····················	····················	····················	····················
5492	MW240755.1	|Severe acute respiratory syndrome coro	····················	·A··················	····················	····················	····················	····················
5493	MW240756.1	|Severe acute respiratory syndrome coro	····················	·A··················	····················	····················	····················	····················
5494	MW240757.1	|Severe acute respiratory syndrome coro	····················	·A··················	····················	····················	····················	····················
5495	MW240758.1	|Severe acute respiratory syndrome coro	····················	·A··················	····················	····················	····················	····················
5496	MW240759.1	|Severe acute respiratory syndrome coro	····················	····················	····················	····················	····················	····················
5497	MW240760.1	|Severe acute respiratory syndrome coro	····················	····················	····················	····················	····················	····················
5498	MW240761.1	|Severe acute respiratory syndrome coro	····················	····················	····················	····················	····················	····················
5499	MW240762.1	|Severe acute respiratory syndrome coro	····················	····················	····················	····················	····················	····················
5500	MW240763.1	|Severe acute respiratory syndrome coro	····················	····················	····················	····················	····················	····················
5501	MW241170.1	|Severe acute respiratory syndrome coro	····················	····················	····················	····················	····················	····················
5502	MW241172.1	|Severe acute respiratory syndrome coro	····················	····················	····················	····················	····················	····················
5503	MW241178.1	|Severe acute respiratory syndrome coro	····················	····················	····················	····················	····················	····················
5504	MW241179.1	|Severe acute respiratory syndrome coro	····················	····················	····················	····················	····················	····················
5505	MW241189.1	|Severe acute respiratory syndrome coro	····················	····················	····················	····················	····················	····················
5506	MW241191.1	|Severe acute respiratory syndrome coro	····················	····················	····················	····················	····················	····················
5507	MW241201.1	|Severe acute respiratory syndrome coro	····················	····················	····················	····················	····················	····················
5508	MW241203.1	|Severe acute respiratory syndrome coro	····················	····················	····················	····················	····················	····················
5509	MW241204.1	|Severe acute respiratory syndrome coro	····················	····················	····················	····················	····················	····················
5510	MW241336.1	|Severe acute respiratory syndrome coro	····················	····················	····················	····················	····················	····················
5511	MW241344.1	|Severe acute respiratory syndrome coro	····················	····················	····················	····················	····················	····················
5512	MW241347.1	|Severe acute respiratory syndrome coro	····················	····················	····················	····················	····················	····················
5513	MW241350.1	|Severe acute respiratory syndrome coro	····················	····················	····················	····················	····················	····················
5514	MW241361.1	|Severe acute respiratory syndrome coro	····················	····················	····················	····················	····················	····················
5515	MW241384.1	|Severe acute respiratory syndrome coro	····················	····················	····················	····················	····················	····················
5516	MW228050.1	|Severe acute respiratory syndrome coro	····················	····················	····················	····················	····················	····················
5517	MW228051.1	|Severe acute respiratory syndrome coro	····················	····················	····················	····················	····················	····················
5518	MW228052.1	|Severe acute respiratory syndrome coro	····················	····················	····················	····················	····················	····················
5519	MW228053.1	|Severe acute respiratory syndrome coro	····················	····················	····················	····················	····················	····················
5520	MW228054.1	|Severe acute respiratory syndrome coro	····················	····················	····················	····················	····················	····················
5521	MW228055.1	|Severe acute respiratory syndrome coro	····················	····················	····················	····················	····················	··A·················
5522	MW228056.1	|Severe acute respiratory syndrome coro	····················	····················	····················	····················	····················	··A·················
5523	MW228070.1	|Severe acute respiratory syndrome coro	····················	···A················	····················	····················	····················	····················
5524	MW228218.1	|Severe acute respiratory syndrome coro	····················	····················	····················	····················	····················	····················
5525	MW228219.1	|Severe acute respiratory syndrome coro	····················	····················	····················	····················	····················	····················
5526	MW228221.1	|Severe acute respiratory syndrome coro	····················	····················	····················	····················	····················	····················
5527	MW228222.1	|Severe acute respiratory syndrome coro	····················	·A··················	····················	····················	····················	····················
5528	MW228223.1	|Severe acute respiratory syndrome coro	····················	····················	····················	····················	····················	····················
5529	MW228224.1	|Severe acute respiratory syndrome coro	····················	···A················	····················	····················	····················	····················
5530	MW228225.1	|Severe acute respiratory syndrome coro	····················	····················	····················	····················	····················	····················
5531	MW228226.1	|Severe acute respiratory syndrome coro	····················	····················	····················	····················	····················	····················
5532	MW228227.1	|Severe acute respiratory syndrome coro	····················	·A··················	····················	····················	····················	····················
5533	MW228228.1	|Severe acute respiratory syndrome coro	····················	····················	····················	····················	····················	····················
5534	MW228229.1	|Severe acute respiratory syndrome coro	····················	····················	····················	····················	····················	····················
5535	MW228230.1	|Severe acute respiratory syndrome coro	····················	····················	····················	····················	····················	····················
5536	MW228231.1	|Severe acute respiratory syndrome coro	····················	····················	····················	····················	····················	····················
5537	MW228232.1	|Severe acute respiratory syndrome coro	····················	····················	····················	····················	····················	····················
5538	MW228233.1	|Severe acute respiratory syndrome coro	····················	····················	····················	····················	····················	····················
5539	MW228235.1	|Severe acute respiratory syndrome coro	····················	····················	····················	····················	····················	····················
5540	MW228236.1	|Severe acute respiratory syndrome coro	····················	····················	····················	····················	····················	····················
5541	MW228237.1	|Severe acute respiratory syndrome coro	····················	····················	····················	····················	····················	····················
5542	MW228238.1	|Severe acute respiratory syndrome coro	····················	·A··················	····················	····················	····················	····················
5543	MW228239.1	|Severe acute respiratory syndrome coro	····················	·A··················	····················	····················	····················	····················
5544	MW228240.1	|Severe acute respiratory syndrome coro	····················	····················	····················	····················	····················	····················
5545	MW228241.1	|Severe acute respiratory syndrome coro	····················	····················	····················	····················	····················	····················
5546	MW228242.1	|Severe acute respiratory syndrome coro	····················	····················	····················	····················	····················	····················
5547	MW228243.1	|Severe acute respiratory syndrome coro	····················	····················	····················	····················	····················	····················
5548	MW228244.1	|Severe acute respiratory syndrome coro	····················	·A··················	····················	····················	····················	····················
5549	MW228246.1	|Severe acute respiratory syndrome coro	····················	·A··················	····················	····················	····················	····················
5550	MW228247.1	|Severe acute respiratory syndrome coro	····················	·A··················	····················	····················	····················	····················
5551	MW228248.1	|Severe acute respiratory syndrome coro	····················	····················	····················	····················	····················	····················
5552	MW228249.1	|Severe acute respiratory syndrome coro	····················	····················	····················	····················	····················	····················
5553	MW228250.1	|Severe acute respiratory syndrome coro	····················	····················	····················	····················	····················	····················
5554	MW228251.1	|Severe acute respiratory syndrome coro	····················	····················	····················	····················	····················	····················
5555	MW228252.1	|Severe acute respiratory syndrome coro	····················	····················	····················	····················	····················	····················
5556	MW228254.1	|Severe acute respiratory syndrome coro	····················	····················	····················	····················	····················	····················
5557	MW228255.1	|Severe acute respiratory syndrome coro	····················	·A··················	····················	····················	····················	····················
5558	MW228256.1	|Severe acute respiratory syndrome coro	····················	····················	····················	····················	····················	····················
5559	MW228257.1	|Severe acute respiratory syndrome coro	····················	····················	····················	····················	····················	····················
5560	MW228258.1	|Severe acute respiratory syndrome coro	····················	·A··················	····················	····················	····················	····················
5561	MW228259.1	|Severe acute respiratory syndrome coro	····················	····················	····················	····················	····················	····················
5562	MW228260.1	|Severe acute respiratory syndrome coro	····················	····················	····················	····················	····················	····················
5563	MW228261.1	|Severe acute respiratory syndrome coro	····················	·A··················	····················	····················	····················	····················
5564	MW228262.1	|Severe acute respiratory syndrome coro	····················	····················	····················	····················	····················	····················
5565	MW228263.1	|Severe acute respiratory syndrome coro	····················	····················	····················	····················	····················	····················
5566	MW228264.1	|Severe acute respiratory syndrome coro	····················	····················	····················	····················	····················	····················
5567	MW228265.1	|Severe acute respiratory syndrome coro	····················	····················	····················	····················	····················	····················
5568	MW228266.1	|Severe acute respiratory syndrome coro	····················	····················	····················	····················	····················	····················

5569	MW228267.1	|Severe acute respiratory syndrome coro	····················	····················	····················	····················	····················	····················
5570	MW228268.1	|Severe acute respiratory syndrome coro	····················	····················	····················	····················	····················	····················
5571	MW228269.1	|Severe acute respiratory syndrome coro	····················	····················	····················	····················	····················	····················
5572	MW228270.1	|Severe acute respiratory syndrome coro	····················	····················	····················	····················	····················	····················
5573	MW228271.1	|Severe acute respiratory syndrome coro	····················	····················	····················	····················	····················	····················
5574	MW228272.1	|Severe acute respiratory syndrome coro	····················	····················	····················	····················	····················	····················
5575	MW228273.1	|Severe acute respiratory syndrome coro	····················	····················	····················	····················	····················	····················
5576	MW228274.1	|Severe acute respiratory syndrome coro	····················	····················	····················	····················	····················	····················
5577	MW228275.1	|Severe acute respiratory syndrome coro	····················	····················	····················	····················	····················	····················
5578	MW228276.1	|Severe acute respiratory syndrome coro	····················	····················	····················	····················	····················	····················
5579	MW228277.1	|Severe acute respiratory syndrome coro	····················	····················	····················	····················	····················	····················
5580	MW228279.1	|Severe acute respiratory syndrome coro	····················	····················	····················	····················	····················	····················
5581	MW228280.1	|Severe acute respiratory syndrome coro	····················	····················	····················	····················	····················	····················
5582	MW229264.1	|Severe acute respiratory syndrome coro	····················	····················	····················	····················	····················	····················
5583	MW227482.1	|Severe acute respiratory syndrome coro	····················	····················	····················	····················	····················	····················
5584	MW227483.1	|Severe acute respiratory syndrome coro	····················	····················	····················	····················	····················	····················
5585	MW227484.1	|Severe acute respiratory syndrome coro	····················	····················	····················	····················	····················	····················
5586	MW227490.1	|Severe acute respiratory syndrome coro	····················	····················	····················	····················	····················	····················
5587	MW227491.1	|Severe acute respiratory syndrome coro	····················	····················	····················	····················	····················	····················
5588	MW227492.1	|Severe acute respiratory syndrome coro	····················	····················	····················	····················	····················	····················
5589	MW227568.1	|Severe acute respiratory syndrome coro	····················	·A··················	····················	····················	·······T············	····················
5590	MT576563.1	|Severe acute respiratory syndrome coro	····················	····················	····················	····················	····················	····················
5591	MT835139.1	|Severe acute respiratory syndrome coro	····················	····················	····················	····················	····················	····················
5592	MT835140.1	|Severe acute respiratory syndrome coro	····················	·A··················	····················	····················	····················	····················
5593	MT835141.1	|Severe acute respiratory syndrome coro	····················	·A··················	····················	····················	····················	····················
5594	MT835142.1	|Severe acute respiratory syndrome coro	····················	····················	····················	····················	····················	····················
5595	MT835143.1	|Severe acute respiratory syndrome coro	····················	····················	····················	····················	····················	····················
5596	MW217299.1	|Severe acute respiratory syndrome coro	····················	····················	····················	····················	····················	····················
5597	MW217300.1	|Severe acute respiratory syndrome coro	····················	····················	····················	····················	····················	····················
5598	MW217301.1	|Severe acute respiratory syndrome coro	····················	····················	····················	····················	····················	····················
5599	MW217302.1	|Severe acute respiratory syndrome coro	····················	····················	····················	····················	····················	····················
5600	MW217303.1	|Severe acute respiratory syndrome coro	····················	····················	····················	····················	····················	····················
5601	MW217304.1	|Severe acute respiratory syndrome coro	····················	····A···············	····················	····················	····················	····················
5602	MW217305.1	|Severe acute respiratory syndrome coro	····················	····················	····················	····················	····················	····················
5603	MW217306.1	|Severe acute respiratory syndrome coro	····················	····················	····················	····················	····················	····················
5604	MW217308.1	|Severe acute respiratory syndrome coro	····················	····················	····················	····················	····················	····················
5605	MW217309.1	|Severe acute respiratory syndrome coro	····················	·A··················	····················	····················	····················	····················
5606	MW217310.1	|Severe acute respiratory syndrome coro	····················	····················	····················	····················	····················	····················
5607	MW217311.1	|Severe acute respiratory syndrome coro	····················	····················	····················	····················	····················	····················
5608	MW217313.1	|Severe acute respiratory syndrome coro	····················	····················	····················	····················	····················	····················
5609	MW217314.1	|Severe acute respiratory syndrome coro	····················	····················	····················	····················	····················	····················
5610	MW217315.1	|Severe acute respiratory syndrome coro	····················	····················	····················	····················	····················	····················
5611	MW217316.1	|Severe acute respiratory syndrome coro	····················	····················	····················	····················	····················	····················
5612	MW217317.1	|Severe acute respiratory syndrome coro	····················	····················	····················	····················	····················	····················
5613	MW217318.1	|Severe acute respiratory syndrome coro	····················	····················	····················	····················	····················	····················
5614	MW217319.1	|Severe acute respiratory syndrome coro	····················	····················	····················	····················	····················	····················
5615	MW217320.1	|Severe acute respiratory syndrome coro	····················	·A··················	····················	····················	····················	····················
5616	MW217321.1	|Severe acute respiratory syndrome coro	····················	·A··················	····················	····················	····················	····················
5617	MW217322.1	|Severe acute respiratory syndrome coro	····················	·A··················	····················	····················	····················	····················
5618	MW217323.1	|Severe acute respiratory syndrome coro	····················	·A··················	····················	····················	····················	····················
5619	MW217324.1	|Severe acute respiratory syndrome coro	····················	·A··················	····················	····················	····················	····················
5620	MW217325.1	|Severe acute respiratory syndrome coro	····················	·A··················	····················	····················	····················	····················
5621	MW217326.1	|Severe acute respiratory syndrome coro	····················	·A··················	····················	····················	····················	····················
5622	MW217327.1	|Severe acute respiratory syndrome coro	····················	····················	····················	····················	····················	····················
5623	MW217328.1	|Severe acute respiratory syndrome coro	····················	····················	····················	····················	····················	····················
5624	MW217330.1	|Severe acute respiratory syndrome coro	····················	·A··················	····················	····················	····················	····················
5625	MW217332.1	|Severe acute respiratory syndrome coro	····················	····················	····················	····················	····················	····················
5626	MW217334.1	|Severe acute respiratory syndrome coro	····················	····················	····················	····················	····················	····················
5627	MW217335.1	|Severe acute respiratory syndrome coro	····················	····················	····················	····················	····················	····················
5628	MW217336.1	|Severe acute respiratory syndrome coro	····················	····················	····················	····················	····················	····················
5629	MW217337.1	|Severe acute respiratory syndrome coro	····················	·A··················	····················	····················	····················	····················
5630	MW217338.1	|Severe acute respiratory syndrome coro	····················	····················	····················	····················	····················	····················
5631	MW217339.1	|Severe acute respiratory syndrome coro	····················	····················	····················	····················	····················	····················
5632	MW217340.1	|Severe acute respiratory syndrome coro	····················	····················	····················	····················	····················	····················
5633	MW217341.1	|Severe acute respiratory syndrome coro	····················	····················	····················	····················	····················	····················
5634	MW217343.1	|Severe acute respiratory syndrome coro	····················	····················	····················	····················	····················	····················
5635	MW217344.1	|Severe acute respiratory syndrome coro	····················	····················	····················	····················	····················	····················
5636	MW217345.1	|Severe acute respiratory syndrome coro	····················	·A··················	····················	····················	····················	····················
5637	MW217346.1	|Severe acute respiratory syndrome coro	····················	····················	····················	····················	····················	····················
5638	MW217347.1	|Severe acute respiratory syndrome coro	····················	····················	····················	····················	····················	····················
5639	MW217348.1	|Severe acute respiratory syndrome coro	····················	····················	····················	····················	····················	····················
5640	MW217349.1	|Severe acute respiratory syndrome coro	····················	····················	····················	····················	····················	····················
5641	MW217350.1	|Severe acute respiratory syndrome coro	····················	····················	····················	····················	····················	····················
5642	MW217351.1	|Severe acute respiratory syndrome coro	····················	····················	····················	····················	····················	····················
5643	MW217352.1	|Severe acute respiratory syndrome coro	····················	····················	····················	····················	····················	····················
5644	MW217353.1	|Severe acute respiratory syndrome coro	·T··················	····················	····················	····················	····················	····················
5645	MW217354.1	|Severe acute respiratory syndrome coro	····················	····················	····················	····················	····················	····················
5646	MW217355.1	|Severe acute respiratory syndrome coro	····················	····················	····················	····················	····················	····················

5647 MW217356.1 |Severe acute respiratory syndrome coro ···················· ···················· ···················· ···················· ···················· ····················
5648 MW217357.1 |Severe acute respiratory syndrome coro ···················· ···················· ···················· ···················· ···················· ····················
5649 MW217358.1 |Severe acute respiratory syndrome coro ···················· ···················· ···················· ···················· ···················· ····················
5650 MW217359.1 |Severe acute respiratory syndrome coro ···················· ·A·················· ···················· ···················· ···················· ····················
5651 MW217360.1 |Severe acute respiratory syndrome coro ···················· ···A················ ···················· ···················· ···················· ····················
5652 MW217361.1 |Severe acute respiratory syndrome coro ···················· ·A·················· ···················· ···················· ···················· ····················
5653 MW217362.1 |Severe acute respiratory syndrome coro ···················· ·A·················· ···················· ···················· ···················· ····················
5654 MW217363.1 |Severe acute respiratory syndrome coro ···················· ···A················ ···················· ···················· ···················· ····················
5655 MW217364.1 |Severe acute respiratory syndrome coro ···················· ·A·················· ···················· ···················· ···················· ····················
5656 MW217365.1 |Severe acute respiratory syndrome coro ···················· ···················· ···················· ···················· ···················· ····················
5657 MW217366.1 |Severe acute respiratory syndrome coro ···················· ···················· ···················· ···················· ···················· ····················
5658 MW217367.1 |Severe acute respiratory syndrome coro ···················· ·A·················· ···················· ···················· ···················· ····················
5659 MW217368.1 |Severe acute respiratory syndrome coro ···················· ···················· ···················· ···················· ···················· ····················
5660 MW217369.1 |Severe acute respiratory syndrome coro ···················· ···················· ···················· ···················· ···················· ····················
5661 MW217370.1 |Severe acute respiratory syndrome coro ···················· ···················· ···················· ···················· ···················· ····················
5662 MW217371.1 |Severe acute respiratory syndrome coro ···················· ···················· ···················· ···················· ···················· ····················
5663 MW217372.1 |Severe acute respiratory syndrome coro ···················· ···················· ···················· ···················· ···················· ····················
5664 MW217373.1 |Severe acute respiratory syndrome coro ···················· ···················· ···················· ···················· ···················· ····················
5665 MW217375.1 |Severe acute respiratory syndrome coro ···················· ···················· ···················· ···················· ···················· ····················
5666 MW217376.1 |Severe acute respiratory syndrome coro ···················· ···················· ···················· ···················· ···················· ····················
5667 MW217377.1 |Severe acute respiratory syndrome coro ···················· ···················· ···················· ···················· ···················· ····················
5668 MW217378.1 |Severe acute respiratory syndrome coro ···················· ···················· ···················· ···················· ···················· ····················
5669 MW217379.1 |Severe acute respiratory syndrome coro ···················· ···················· ···················· ···················· ···················· ····················
5670 MW217380.1 |Severe acute respiratory syndrome coro ···················· ···················· ···················· ···················· ···················· ····················
5671 MW219695.1 |Severe acute respiratory syndrome coro ···················· ···················· ···················· ···················· ···················· ····················
5672 MW221270.1 |Severe acute respiratory syndrome coro ···················· ··C················· ···················· ···················· ···················· ····················
5673 MW221275.1 |Severe acute respiratory syndrome coro ···················· ···················· ···················· ···················· ···················· ····················
5674 MW205968.1 |Severe acute respiratory syndrome coro ···················· ···················· ···················· ···················· ···················· ····················
5675 MW205969.1 |Severe acute respiratory syndrome coro ···················· ···················· ···················· ···················· ···················· ····················
5676 MW205970.1 |Severe acute respiratory syndrome coro ···················· ···················· ···················· ···················· ···················· ····················
5677 MW205971.1 |Severe acute respiratory syndrome coro ···················· ···················· ···················· ···················· ···················· ·········A··········
5678 MW205972.1 |Severe acute respiratory syndrome coro ···················· ···················· ···················· ···················· ···················· ····················
5679 MW205973.1 |Severe acute respiratory syndrome coro ···················· ···················· ···················· ···················· ···················· ····················
5680 MW205974.1 |Severe acute respiratory syndrome coro ···················· ···················· ···················· ···················· ···················· ····················
5681 MW205975.1 |Severe acute respiratory syndrome coro ···················· ···················· ···················· ···················· ···················· ····················
5682 MW205976.1 |Severe acute respiratory syndrome coro ···················· ···················· ···················· ···················· ···················· ····················
5683 MW205977.1 |Severe acute respiratory syndrome coro ···················· ···················· ···················· ···················· ···················· ····················
5684 MW205978.1 |Severe acute respiratory syndrome coro ···················· ···················· ···················· ···················· ···················· ····················
5685 MW205979.1 |Severe acute respiratory syndrome coro ···················· ···················· ···················· ···················· ···················· ····················
5686 MW205980.1 |Severe acute respiratory syndrome coro ···················· ···················· ···················· ···················· ···················· ····················
5687 MW205981.1 |Severe acute respiratory syndrome coro ···················· ···················· ···················· ···················· ···················· ····················
5688 MW205982.1 |Severe acute respiratory syndrome coro ···················· ···················· ···················· ···················· ···················· ····················
5689 MW205983.1 |Severe acute respiratory syndrome coro ···················· ···················· ···················· ···················· ···················· ····················
5690 MW205984.1 |Severe acute respiratory syndrome coro ···················· ···················· ···················· ···················· ···················· ····················
5691 MW205985.1 |Severe acute respiratory syndrome coro ···················· ···················· ···················· ···················· ···················· ····················
5692 MW205986.1 |Severe acute respiratory syndrome coro ···················· ···················· ···················· ···················· ···················· ····················
5693 MW205987.1 |Severe acute respiratory syndrome coro ···················· ···················· ···················· ···················· ···················· ····················
5694 MW205988.1 |Severe acute respiratory syndrome coro ···················· ···················· ···················· ···················· ···················· ····················
5695 MW205989.1 |Severe acute respiratory syndrome coro ···················· ···················· ···················· ···················· ···················· ····················
5696 MW205990.1 |Severe acute respiratory syndrome coro ···················· ···················· ···················· ···················· ···················· ····················
5697 MW205991.1 |Severe acute respiratory syndrome coro ···················· ···················· ···················· ···················· ···················· ····················
5698 MW205992.1 |Severe acute respiratory syndrome coro ···················· ·A·················· ···················· ···················· ···················· ····················
5699 MW205993.1 |Severe acute respiratory syndrome coro ···················· ···················· ···················· ···················· ···················· ····················
5700 MW205994.1 |Severe acute respiratory syndrome coro ···················· ···················· ···················· ···················· ···················· ····················
5701 MW205995.1 |Severe acute respiratory syndrome coro ···················· ···A················ ···················· ···················· ···················· ····················
5702 MW205996.1 |Severe acute respiratory syndrome coro ···················· ···················· ···················· ···················· ···················· ····················
5703 MW205997.1 |Severe acute respiratory syndrome coro ···················· ···················· ···················· ···················· ···················· ····················
5704 MW206007.1 |Severe acute respiratory syndrome coro ···················· ···················· ···················· ···················· ···················· ····················
5705 MW206008.1 |Severe acute respiratory syndrome coro ···················· ·A·················· ···················· ···················· ···················· ····················
5706 MW206009.1 |Severe acute respiratory syndrome coro ···················· ···················· ···················· ···················· ···················· ····················
5707 MW206010.1 |Severe acute respiratory syndrome coro ···················· ·A·················· ···················· ···················· ···················· ····················
5708 MW206011.1 |Severe acute respiratory syndrome coro ···················· ···················· ···················· ···················· ···················· ····················
5709 MW206012.1 |Severe acute respiratory syndrome coro ···················· ···················· ···················· ···················· ···················· ····················
5710 MW206013.1 |Severe acute respiratory syndrome coro ···················· ···················· ···················· ···················· ···················· ····················
5711 MW206014.1 |Severe acute respiratory syndrome coro ···················· ···················· ···················· ···················· ···················· ····················
5712 MW206015.1 |Severe acute respiratory syndrome coro ···················· ···················· ···················· ···················· ···················· ····················
5713 MW206016.1 |Severe acute respiratory syndrome coro ···················· ···················· ···················· ···················· ···················· ····················
5714 MW206017.1 |Severe acute respiratory syndrome coro ···················· ···················· ···················· ···················· ···················· ····················
5715 MW206018.1 |Severe acute respiratory syndrome coro ···················· ···················· ···················· ···················· ···················· ····················
5716 MW206019.1 |Severe acute respiratory syndrome coro ···················· ···················· ···················· ···················· ···················· ····················
5717 MW206020.1 |Severe acute respiratory syndrome coro ···················· ···················· ···················· ···················· ···················· ····················
5718 MW206021.1 |Severe acute respiratory syndrome coro ···················· ···················· ···················· ···················· ···················· ····················
5719 MW206022.1 |Severe acute respiratory syndrome coro ···················· ···················· ···················· ···················· ···················· ····················
5720 MW206023.1 |Severe acute respiratory syndrome coro ···················· ···················· ···················· ···················· ···················· ····················
5721 MW206024.1 |Severe acute respiratory syndrome coro ···················· ···················· ···················· ···················· ···················· ····················
5722 MW206025.1 |Severe acute respiratory syndrome coro ···················· ···················· ···················· ···················· ···················· ····················
5723 MW206026.1 |Severe acute respiratory syndrome coro ···················· ···················· ···················· ···················· ···················· ····················
5724 MW206027.1 |Severe acute respiratory syndrome coro ···················· ·A·················· ···················· ···················· ···················· ····················

5725 MW206028.1 |Severe acute respiratory syndrome coro ···················· ···················· ···················· ···················· ···················· ····················
5726 MW206029.1 |Severe acute respiratory syndrome coro ···················· ···················· ···················· ···················· ···················· ····················
5727 MW206030.1 |Severe acute respiratory syndrome coro ···················· ···················· ···················· ···················· ···················· ····················
5728 MW206031.1 |Severe acute respiratory syndrome coro ···················· ···················· ···················· ···················· ···················· ····················
5729 MW206032.1 |Severe acute respiratory syndrome coro ···················· ···················· ···················· ···················· ···················· ····················
5730 MW206033.1 |Severe acute respiratory syndrome coro ···················· ···················· ···················· ···················· ···················· ····················
5731 MW206034.1 |Severe acute respiratory syndrome coro ···················· ···················· ···················· ···················· ···················· ····················
5732 MW206035.1 |Severe acute respiratory syndrome coro ···················· ···················· ···················· ···················· ···················· ····················
5733 MW206036.1 |Severe acute respiratory syndrome coro ···················· ···················· ···················· ···················· ···················· ····················
5734 MW206037.1 |Severe acute respiratory syndrome coro ···················· ···················· ···················· ···················· ···················· ····················
5735 MW206038.1 |Severe acute respiratory syndrome coro ···················· ···················· ···················· ···················· ···················· ····················
5736 MW206039.1 |Severe acute respiratory syndrome coro ···················· ···················· ···················· ···················· ···················· ····················
5737 MW206040.1 |Severe acute respiratory syndrome coro ···················· ···················· ···················· ···················· ···················· ····················
5738 MW206041.1 |Severe acute respiratory syndrome coro ···················· ···················· ···················· ···················· ···················· ····················
5739 MW206042.1 |Severe acute respiratory syndrome coro ···················· ···················· ···················· ···················· ···················· ····················
5740 MW206043.1 |Severe acute respiratory syndrome coro ···················· ···················· ···················· ···················· ···················· ····················
5741 MW206044.1 |Severe acute respiratory syndrome coro ···················· ···················· ···················· ···················· ···················· ····················
5742 MW206045.1 |Severe acute respiratory syndrome coro ···················· ···················· ···················· ···················· ···················· ····················
5743 MW206046.1 |Severe acute respiratory syndrome coro ···················· ···················· ···················· ···················· ···················· ····················
5744 MW206047.1 |Severe acute respiratory syndrome coro ···················· ···················· ···················· ···················· ···················· ····················
5745 MW206048.1 |Severe acute respiratory syndrome coro ···················· ···················· ···················· ···················· ···················· ····················
5746 MW206049.1 |Severe acute respiratory syndrome coro ···················· ···················· ···················· ···················· ···················· ····················
5747 MW206050.1 |Severe acute respiratory syndrome coro ···················· ···················· ···················· ···················· ···················· ····················
5748 MW206051.1 |Severe acute respiratory syndrome coro ···················· ···················· ···················· ···················· ···················· ····················
5749 MW206052.1 |Severe acute respiratory syndrome coro ···················· ···················· ···················· ···················· ···················· ····················
5750 MW206053.1 |Severe acute respiratory syndrome coro ···················· ···················· ···················· ···················· ···················· ····················
5751 MW206054.1 |Severe acute respiratory syndrome coro ···················· ···················· ···················· ···················· ···················· ····················
5752 MW206055.1 |Severe acute respiratory syndrome coro ···················· ···················· ···················· ···················· ···················· ····················
5753 MW206056.1 |Severe acute respiratory syndrome coro ···················· ···················· ···················· ···················· ·········A·········· ····················
5754 MW206057.1 |Severe acute respiratory syndrome coro ···················· ···················· ···················· ···················· ···················· ····················
5755 MW206058.1 |Severe acute respiratory syndrome coro ···················· ···················· ···················· ···················· ···················· ····················
5756 MW206059.1 |Severe acute respiratory syndrome coro ···················· ···················· ···················· ···················· ···················· ····················
5757 MW206060.1 |Severe acute respiratory syndrome coro ···················· ···················· ···················· ···················· ···················· ····················
5758 MW206061.1 |Severe acute respiratory syndrome coro ···················· ···················· ···················· ···················· ···················· ····················
5759 MW206062.1 |Severe acute respiratory syndrome coro ···················· ···················· ···················· ···················· ···················· ····················
5760 MW206063.1 |Severe acute respiratory syndrome coro ···················· ···················· ···················· ···················· ···················· ····················
5761 MW206064.1 |Severe acute respiratory syndrome coro ···················· ···················· ···················· ···················· ···················· ····················
5762 MW206065.1 |Severe acute respiratory syndrome coro ···················· ···················· ···················· ···················· ·········A·········· ····················
5763 MW206066.1 |Severe acute respiratory syndrome coro ···················· ···················· ···················· ···················· ···················· ····················
5764 MW206067.1 |Severe acute respiratory syndrome coro ···················· ···················· ···················· ···················· ···················· ····················
5765 MW206068.1 |Severe acute respiratory syndrome coro ···················· ···················· ···················· ···················· ···················· ····················
5766 MW206069.1 |Severe acute respiratory syndrome coro ···················· ···················· ···················· ···················· ···················· ····················
5767 MW206070.1 |Severe acute respiratory syndrome coro ···················· ···················· ···················· ···················· ···················· ····················
5768 MW206071.1 |Severe acute respiratory syndrome coro ···················· ···················· ···················· ···················· ···················· ····················
5769 MW206072.1 |Severe acute respiratory syndrome coro ···················· ···················· ···················· ···················· ···················· ····················
5770 MW206073.1 |Severe acute respiratory syndrome coro ···················· ···················· ···················· ···················· ···················· ····················
5771 MW206074.1 |Severe acute respiratory syndrome coro ···················· ···················· ···················· ···················· ···················· ····················
5772 MW206075.1 |Severe acute respiratory syndrome coro ···················· ···················· ···················· ···················· ···················· ····················
5773 MW206076.1 |Severe acute respiratory syndrome coro ···················· ···················· ···················· ···················· ···················· ····················
5774 MW206077.1 |Severe acute respiratory syndrome coro ···················· ···················· ···················· ···················· ···················· ····················
5775 MW206078.1 |Severe acute respiratory syndrome coro ···················· ···················· ···················· ···················· ···················· ····················
5776 MW206079.1 |Severe acute respiratory syndrome coro ···················· ·A·················· ···················· ···················· ···················· ····················
5777 MW206080.1 |Severe acute respiratory syndrome coro ···················· ···················· ···················· ···················· ···················· ····················
5778 MW206081.1 |Severe acute respiratory syndrome coro ···················· ···················· ···················· ···················· ···················· ····················
5779 MW206082.1 |Severe acute respiratory syndrome coro ···················· ···················· ···················· ···················· ···················· ····················
5780 MW206083.1 |Severe acute respiratory syndrome coro ···················· ·A·················· ···················· ···················· ···················· ····················
5781 MW206084.1 |Severe acute respiratory syndrome coro ···················· ···················· ···················· ···················· ···················· ····················
5782 MW206085.1 |Severe acute respiratory syndrome coro ···················· ···················· ···················· ···················· ···················· ····················
5783 MW206086.1 |Severe acute respiratory syndrome coro ···················· ···················· ···················· ···················· ···················· ····················
5784 MW206087.1 |Severe acute respiratory syndrome coro ···················· ···················· ···················· ···················· ···················· ····················
5785 MW206088.1 |Severe acute respiratory syndrome coro ···················· ···················· ···················· ···················· ···················· ····················
5786 MW206089.1 |Severe acute respiratory syndrome coro ···················· ···················· ···················· ···················· ···················· ····················
5787 MW206090.1 |Severe acute respiratory syndrome coro ···················· ···················· ···················· ···················· ···················· ····················
5788 MW206091.1 |Severe acute respiratory syndrome coro ···················· ···················· ···················· ···················· ···················· ····················
5789 MW206092.1 |Severe acute respiratory syndrome coro ···················· ···················· ···················· ···················· ···················· ····················
5790 MW206093.1 |Severe acute respiratory syndrome coro ···················· ···················· ···················· ···················· ···················· ····················
5791 MW206094.1 |Severe acute respiratory syndrome coro ···················· ···················· ···················· ···················· ···········G········ ····················
5792 MW206095.1 |Severe acute respiratory syndrome coro ···················· ···················· ···················· ···················· ···················· ····················
5793 MW206096.1 |Severe acute respiratory syndrome coro ···················· ···················· ···················· ···················· ···················· ····················
5794 MW206097.1 |Severe acute respiratory syndrome coro ···················· ···················· ···················· ···················· ···················· ····················
5795 MW206098.1 |Severe acute respiratory syndrome coro ···················· ···················· ···················· ···················· ···················· ····················
5796 MW206099.1 |Severe acute respiratory syndrome coro ···················· ···················· ···················· ···················· ···················· ····················
5797 MW206100.1 |Severe acute respiratory syndrome coro ···················· ···················· ···················· ···················· ···················· ····················
5798 MW206101.1 |Severe acute respiratory syndrome coro ···················· ···················· ···················· ···················· ···················· ····················
5799 MW206102.1 |Severe acute respiratory syndrome coro ···················· ···················· ···················· ···················· ···················· ····················
5800 MW206103.1 |Severe acute respiratory syndrome coro ···················· ···················· ···················· ···················· ···················· ····················
5801 MW206104.1 |Severe acute respiratory syndrome coro ···················· ···················· ···················· ···················· ···················· ····················
5802 MW206105.1 |Severe acute respiratory syndrome coro ···················· ···················· ···················· ···················· ···················· ····················

5803 MW206106.1 |Severe acute respiratory syndrome coro ···················· ···················· ···················· ···················· ···················· ····················
5804 MW206107.1 |Severe acute respiratory syndrome coro ···················· ···················· ···················· ···················· ···················· ····················
5805 MW206108.1 |Severe acute respiratory syndrome coro ···················· ···················· ···················· ···················· ···················· ····················
5806 MW206109.1 |Severe acute respiratory syndrome coro ···················· ···················· ···················· ···················· ···················· ····················
5807 MW206110.1 |Severe acute respiratory syndrome coro ···················· ···················· ···················· ···················· ···················· ····················
5808 MW206111.1 |Severe acute respiratory syndrome coro ···················· ···················· ···················· ···················· ···················· ····················
5809 MW206112.1 |Severe acute respiratory syndrome coro ···················· ···················· ···················· ···················· ···················· ····················
5810 MW206113.1 |Severe acute respiratory syndrome coro ···················· ···················· ···················· ···················· ···················· ····················
5811 MW206114.1 |Severe acute respiratory syndrome coro ···················· ···················· ···················· ···················· ···················· ····················
5812 MW206115.1 |Severe acute respiratory syndrome coro ···················· ···················· ···················· ···················· ···················· ····················
5813 MW206116.1 |Severe acute respiratory syndrome coro ···················· ···················· ···················· ···················· ···················· ····················
5814 MW206117.1 |Severe acute respiratory syndrome coro ···················· ···················· ···················· ···················· ···················· ····················
5815 MW206118.1 |Severe acute respiratory syndrome coro ···················· ···················· ···················· ···················· ···················· ····················
5816 MW206119.1 |Severe acute respiratory syndrome coro ···················· ···················· ···················· ···················· ···················· ····················
5817 MW206120.1 |Severe acute respiratory syndrome coro ···················· ···················· ···················· ···················· ···················· ····················
5818 MW206121.1 |Severe acute respiratory syndrome coro ···················· ···················· ···················· ···················· ···················· ····················
5819 MW206122.1 |Severe acute respiratory syndrome coro ···················· ···················· ···················· ···················· ···················· ····················
5820 MW206123.1 |Severe acute respiratory syndrome coro ···················· ···················· ···················· ···················· ···················· ····················
5821 MW206124.1 |Severe acute respiratory syndrome coro ···················· ···················· ···················· ···················· ···················· ····················
5822 MW206125.1 |Severe acute respiratory syndrome coro ···················· ···················· ···················· ···················· ···················· ····················
5823 MW206126.1 |Severe acute respiratory syndrome coro ···················· ···················· ···················· ···················· ···················· ····················
5824 MW206127.1 |Severe acute respiratory syndrome coro ···················· ···················· ···················· ···················· ···················· ····················
5825 MW206128.1 |Severe acute respiratory syndrome coro ···················· ···················· ···················· ···················· ···················· ····················
5826 MW206129.1 |Severe acute respiratory syndrome coro ···················· ···················· ···················· ···················· ···················· ····················
5827 MW206130.1 |Severe acute respiratory syndrome coro ···················· ···················· ···················· ···················· ·········A·········· ····················
5828 MW206131.1 |Severe acute respiratory syndrome coro ···················· ···················· ···················· ···················· ···················· ····················
5829 MW206132.1 |Severe acute respiratory syndrome coro ···················· ···················· ···················· ···················· ···················· ····················
5830 MW206133.1 |Severe acute respiratory syndrome coro ···················· ···················· ···················· ···················· ···················· ····················
5831 MW206134.1 |Severe acute respiratory syndrome coro ···················· ···················· ···················· ···················· ···················· ····················
5832 MW206135.1 |Severe acute respiratory syndrome coro ···················· ···················· ···················· ···················· ···················· ····················
5833 MW206136.1 |Severe acute respiratory syndrome coro ···················· ·A·················· ···················· ···················· ···················· ····················
5834 MW206137.1 |Severe acute respiratory syndrome coro ···················· ···················· ···················· ···················· ···················· ····················
5835 MW206138.1 |Severe acute respiratory syndrome coro ···················· ···················· ···················· ···················· ···················· ····················
5836 MW206139.1 |Severe acute respiratory syndrome coro ···················· ···················· ···················· ···················· ···················· ····················
5837 MW206140.1 |Severe acute respiratory syndrome coro ···················· ···················· ···················· ···················· ···················· ····················
5838 MW206141.1 |Severe acute respiratory syndrome coro ···················· ···················· ···················· ···················· ···················· ····················
5839 MW206142.1 |Severe acute respiratory syndrome coro ···················· ···················· ···················· ···················· ···················· ····················
5840 MW206143.1 |Severe acute respiratory syndrome coro ···················· ···················· ···················· ···················· ···················· ····················
5841 MW206144.1 |Severe acute respiratory syndrome coro ···················· ···················· ···················· ···················· ···················· ····················
5842 MW206145.1 |Severe acute respiratory syndrome coro ···················· ···················· ···················· ···················· ···················· ····················
5843 MW206146.1 |Severe acute respiratory syndrome coro ···················· ···················· A··················· ···················· ···················· ····················
5844 MW206147.1 |Severe acute respiratory syndrome coro ···················· ···················· ···················· ···················· ·········A·········· ····················
5845 MW206148.1 |Severe acute respiratory syndrome coro ···················· ···················· ···················· ···················· ·········A·········· ····················
5846 MW206149.1 |Severe acute respiratory syndrome coro ···················· ···················· ···················· ···················· ···················· ····················
5847 MW206150.1 |Severe acute respiratory syndrome coro ···················· ···················· ···················· ···················· ···················· ····················
5848 MW206151.1 |Severe acute respiratory syndrome coro ···················· ···················· ···················· ···················· ···················· ····················
5849 MW206152.1 |Severe acute respiratory syndrome coro ···················· ···················· ···················· ···················· ···················· ····················
5850 MW206153.1 |Severe acute respiratory syndrome coro ···················· ···················· ···················· ···················· ···················· ····················
5851 MW206154.1 |Severe acute respiratory syndrome coro ···················· ···················· ···················· ···················· ···················· ····················
5852 MW206155.1 |Severe acute respiratory syndrome coro ···················· ···················· ···················· ···················· ···················· ····················
5853 MW206156.1 |Severe acute respiratory syndrome coro ···················· ···················· ···················· ···················· ···················· ····················
5854 MW206157.1 |Severe acute respiratory syndrome coro ···················· ···················· ···················· ···················· ···················· ····················
5855 MW206158.1 |Severe acute respiratory syndrome coro ···················· ···················· ···················· ···················· ···················· ····················
5856 MW206159.1 |Severe acute respiratory syndrome coro ···················· ···················· ···················· ···················· ···················· ····················
5857 MW206160.1 |Severe acute respiratory syndrome coro ···················· ···················· ···················· ···················· ···················· ····················
5858 MW206161.1 |Severe acute respiratory syndrome coro ···················· ···················· ···················· ···················· ···················· ····················
5859 MW206162.1 |Severe acute respiratory syndrome coro ···················· ···················· ···················· ···················· ···················· ····················
5860 MW206163.1 |Severe acute respiratory syndrome coro ···················· ·A·················· ···················· ···················· ···················· ····················
5861 MW206164.1 |Severe acute respiratory syndrome coro ···················· ·A·················· ···················· ···················· ···················· ····················
5862 MW206165.1 |Severe acute respiratory syndrome coro ···················· ···················· ···················· ···················· ···················· ····················
5863 MW206166.1 |Severe acute respiratory syndrome coro ···················· ·A·················· ···················· ···················· ···················· ····················
5864 MW206167.1 |Severe acute respiratory syndrome coro ···················· ···················· ···················· ···················· ···················· ····················
5865 MW206168.1 |Severe acute respiratory syndrome coro ···················· ···················· ···················· ···················· ···················· ····················
5866 MW206169.1 |Severe acute respiratory syndrome coro ···················· ···················· ···················· ···················· ·········A·········· ····················
5867 MW206170.1 |Severe acute respiratory syndrome coro ···················· ···················· ···················· ···················· ···················· ····················
5868 MW206171.1 |Severe acute respiratory syndrome coro ···················· ···················· ···················· ···················· ···················· ····················
5869 MW206172.1 |Severe acute respiratory syndrome coro ···················· ···················· ···················· ···················· ···················· ····················
5870 MW206173.1 |Severe acute respiratory syndrome coro ···················· ···················· ···················· ···················· ···················· ····················
5871 MW206174.1 |Severe acute respiratory syndrome coro ···················· ···················· ···················· ···················· ···················· ····················
5872 MW206175.1 |Severe acute respiratory syndrome coro ···················· ···················· ···················· ···················· ···················· ····················
5873 MW206176.1 |Severe acute respiratory syndrome coro ···················· ···················· ···················· ···················· ···················· ····················
5874 MW206177.1 |Severe acute respiratory syndrome coro ···················· ···················· ···················· ···················· ···················· ····················
5875 MW206178.1 |Severe acute respiratory syndrome coro ···················· ···················· ···················· ···················· ···················· ····················
5876 MW206179.1 |Severe acute respiratory syndrome coro ···················· ···················· ···················· ···················· ···················· ····················
5877 MW206180.1 |Severe acute respiratory syndrome coro ···················· ···················· ···················· ···················· ···················· ····················
5878 MW206181.1 |Severe acute respiratory syndrome coro ···················· ···················· ···················· ···················· ···················· ····················
5879 MW206182.1 |Severe acute respiratory syndrome coro ···················· ···················· ···················· ···················· ···················· ····················
5880 MW206183.1 |Severe acute respiratory syndrome coro ···················· ···················· ···················· ···················· ···················· ····················

```
5881 MW206184.1 |Severe acute respiratory syndrome coro    ....................  ....................  ....................  ....................  ....................  ....................
5882 MW206185.1 |Severe acute respiratory syndrome coro    ....................  ....................  ....................  ....................  .........A..........  ....................
5883 MW206186.1 |Severe acute respiratory syndrome coro    ....................  ....................  ....................  ....................  ....................  ....................
5884 MW206187.1 |Severe acute respiratory syndrome coro    ....................  ....................  ....................  ....................  ....................  ....................
5885 MW206188.1 |Severe acute respiratory syndrome coro    ....................  ....................  ....................  ....................  ....................  ....................
5886 MW206189.1 |Severe acute respiratory syndrome coro    ....................  ....................  ....................  ....................  ....................  ....................
5887 MW206190.1 |Severe acute respiratory syndrome coro    ....................  ....................  ....................  ....................  ....................  ....................
5888 MW206191.1 |Severe acute respiratory syndrome coro    ....................  ....................  ....................  ....................  ...C................  ....................
5889 MW206192.1 |Severe acute respiratory syndrome coro    ....................  ....................  ....................  ....................  ....................  ....................
5890 MW206193.1 |Severe acute respiratory syndrome coro    ....................  ....................  ....................  ....................  ....................  ....................
5891 MW206194.1 |Severe acute respiratory syndrome coro    ....................  ....................  ....................  ....................  ....................  ....................
5892 MW206195.1 |Severe acute respiratory syndrome coro    ....................  ....................  ....................  ....................  ....................  ....................
5893 MW206196.1 |Severe acute respiratory syndrome coro    ....................  ....................  ....................  ....................  ....................  ....................
5894 MW206197.1 |Severe acute respiratory syndrome coro    ....................  ....................  ....................  ....................  ....................  ....................
5895 MW206198.1 |Severe acute respiratory syndrome coro    ....................  ....................  ....................  ....................  ....................  ....................
5896 MW206199.1 |Severe acute respiratory syndrome coro    ....................  ....................  ....................  ....................  ....................  ....................
5897 MW206200.1 |Severe acute respiratory syndrome coro    ....................  ....................  ....................  ....................  ....................  ....................
5898 MW206201.1 |Severe acute respiratory syndrome coro    ....................  ....................  ....................  ....................  ....................  ....................
5899 MW206202.1 |Severe acute respiratory syndrome coro    ....................  ....................  ....................  ....................  ....................  ....................
5900 MW206203.1 |Severe acute respiratory syndrome coro    ....................  ....................  ....................  ....................  .........A..........  ....................
5901 MW206204.1 |Severe acute respiratory syndrome coro    ....................  ....................  ....................  ....................  ....................  ....................
5902 MW206205.1 |Severe acute respiratory syndrome coro    ....................  ....................  ....................  ....................  ....................  ....................
5903 MW206206.1 |Severe acute respiratory syndrome coro    ....................  ....................  ....................  ....................  ....................  ....................
5904 MW206207.1 |Severe acute respiratory syndrome coro    ....................  ....................  ....................  ....................  ....................  ....................
5905 MW206208.1 |Severe acute respiratory syndrome coro    ....................  ....................  ....................  ....................  ....................  ....................
5906 MW206209.1 |Severe acute respiratory syndrome coro    ....................  ....................  ....................  ....................  ....................  ....................
5907 MW206210.1 |Severe acute respiratory syndrome coro    ....................  ....................  ....................  ....................  ....................  ....................
5908 MW206211.1 |Severe acute respiratory syndrome coro    ....................  ....................  ....................  ....................  ....................  ....................
5909 MW206212.1 |Severe acute respiratory syndrome coro    ....................  ....................  ....................  ....................  ....................  ....................
5910 MW206213.1 |Severe acute respiratory syndrome coro    ....................  ....................  ....................  ....................  ....................  ....................
5911 MW206214.1 |Severe acute respiratory syndrome coro    ....................  ....................  ....................  ....................  ....................  ....................
5912 MW206215.1 |Severe acute respiratory syndrome coro    ....................  ....................  ....................  ....................  ....................  ....................
5913 MW206216.1 |Severe acute respiratory syndrome coro    ....................  ....................  ....................  ....................  ....................  ....................
5914 MW206217.1 |Severe acute respiratory syndrome coro    ....................  ....................  ....................  ....................  ....................  ....................
5915 MW206218.1 |Severe acute respiratory syndrome coro    ....................  ....................  ....................  ....................  ....................  ....................
5916 MW206219.1 |Severe acute respiratory syndrome coro    ....................  ....................  ....................  ....................  ....................  ....................
5917 MW206220.1 |Severe acute respiratory syndrome coro    ....................  ....................  ....................  ....................  ....................  ....................
5918 MW206221.1 |Severe acute respiratory syndrome coro    ....................  ....................  ....................  ....................  ....................  ....................
5919 MW206222.1 |Severe acute respiratory syndrome coro    ....................  ....................  ....................  ....................  ....................  ....................
5920 MW206223.1 |Severe acute respiratory syndrome coro    ....................  ....................  ....................  ....................  ....................  ....................
5921 MW206224.1 |Severe acute respiratory syndrome coro    ....................  ....................  ....................  ....................  ....................  ....................
5922 MW206225.1 |Severe acute respiratory syndrome coro    ....................  ....................  ....................  ....................  ....................  ....................
5923 MW206226.1 |Severe acute respiratory syndrome coro    ....................  ....................  ....................  ....................  ....................  ....................
5924 MW206227.1 |Severe acute respiratory syndrome coro    ....................  ....................  ....................  ....................  ....................  ....................
5925 MW206228.1 |Severe acute respiratory syndrome coro    ....................  ....................  ....................  ....................  ....................  ....................
5926 MW206229.1 |Severe acute respiratory syndrome coro    ....................  ....................  ....................  ....................  ....................  ....................
5927 MW206230.1 |Severe acute respiratory syndrome coro    ....................  .A..................  ....................  ....................  ....................  ....................
5928 MW206231.1 |Severe acute respiratory syndrome coro    ....................  ....................  ....................  ....................  ....................  ....................
5929 MW206232.1 |Severe acute respiratory syndrome coro    ....................  ....................  ....................  ....................  ....................  ....................
5930 MW206233.1 |Severe acute respiratory syndrome coro    ....................  ....................  ....................  ....................  ....................  ....................
5931 MW206234.1 |Severe acute respiratory syndrome coro    ....................  ....................  ....................  ....................  ....................  ....................
5932 MW206235.1 |Severe acute respiratory syndrome coro    ....................  ....................  ....................  ....................  .........A..........  ....................
5933 MW206236.1 |Severe acute respiratory syndrome coro    ....................  ....................  ....................  ....................  ....................  ....................
5934 MW206237.1 |Severe acute respiratory syndrome coro    ....................  ....................  ....................  ....................  ....................  ....................
5935 MW206238.1 |Severe acute respiratory syndrome coro    ....................  ....................  ....................  ....................  .........A..........  ....................
5936 MW206239.1 |Severe acute respiratory syndrome coro    ....................  ....................  ....................  ....................  ....................  ....................
5937 MW206240.1 |Severe acute respiratory syndrome coro    ....................  ....................  ....................  ....................  ....................  ....................
5938 MW206241.1 |Severe acute respiratory syndrome coro    ....................  ....................  ....................  ....................  ....................  ....................
5939 MW206242.1 |Severe acute respiratory syndrome coro    ....................  ....................  ....................  ....................  ....................  ....................
5940 MW206243.1 |Severe acute respiratory syndrome coro    ....................  ....................  ....................  ....................  ....................  ....................
5941 MW206244.1 |Severe acute respiratory syndrome coro    ....................  ....................  ....................  ....................  ....................  ....................
5942 MW206245.1 |Severe acute respiratory syndrome coro    ....................  ....................  ....................  ....................  ....................  ....................
5943 MW206246.1 |Severe acute respiratory syndrome coro    ....................  ....................  ....................  ....................  ....................  ....................
5944 MW206247.1 |Severe acute respiratory syndrome coro    ....................  ....................  ....................  ....................  ....................  ....................
5945 MW206248.1 |Severe acute respiratory syndrome coro    ....................  ....................  ....................  ....................  ....................  ....................
5946 MW206249.1 |Severe acute respiratory syndrome coro    ....................  ....................  ....................  ....................  ....................  ....................
5947 MW206250.1 |Severe acute respiratory syndrome coro    ....................  ....................  ....................  ....................  ....................  ....................
5948 MW206251.1 |Severe acute respiratory syndrome coro    ....................  ....................  ....................  ....................  ....................  ....................
5949 MW206252.1 |Severe acute respiratory syndrome coro    ....................  ....................  ....................  ....................  ....................  ....................
5950 MW206253.1 |Severe acute respiratory syndrome coro    ....................  ....................  ....................  ....................  ....................  ....................
5951 MW206254.1 |Severe acute respiratory syndrome coro    ....................  ....................  ....................  ....................  ....................  ....................
5952 MW206255.1 |Severe acute respiratory syndrome coro    ....................  ....................  ....................  ....................  ....................  ....................
5953 MW206256.1 |Severe acute respiratory syndrome coro    ....................  ....................  ....................  ....................  ....................  ....................
5954 MW206257.1 |Severe acute respiratory syndrome coro    ....................  ....................  ....................  ....................  ....................  ....................
5955 MW206258.1 |Severe acute respiratory syndrome coro    ....................  ....................  ....................  ....................  ....................  ....................
5956 MW206259.1 |Severe acute respiratory syndrome coro    ....................  ....................  ....................  ....................  .........A..........  ....................
5957 MW206260.1 |Severe acute respiratory syndrome coro    ....................  ....................  ....................  ....................  ....................  ....................
5958 MW206261.1 |Severe acute respiratory syndrome coro    ....................  ....................  ....................  ....................  ....................  ....................
```

5959	MW206262.1	|Severe acute respiratory syndrome coro	....	....	....	....	....	....
5960	MW206263.1	|Severe acute respiratory syndrome coro	....	....	....	....	....	....
5961	MW206264.1	|Severe acute respiratory syndrome coro	....	....	....	....	....	....
5962	MW206265.1	|Severe acute respiratory syndrome coro	....	....	....	....	....	....
5963	MW206266.1	|Severe acute respiratory syndrome coro	....	....	....	....	....	....
5964	MW206267.1	|Severe acute respiratory syndrome coro	....	....	....	....	....	....
5965	MW206268.1	|Severe acute respiratory syndrome coro	....	....	....	....	....	....
5966	MW206269.1	|Severe acute respiratory syndrome coro	....	....	....	....	....	....
5967	MW206270.1	|Severe acute respiratory syndrome coro	....	....	....	....	....	....
5968	MW206271.1	|Severe acute respiratory syndrome coro	....	....	....	....	....	....
5969	MW206272.1	|Severe acute respiratory syndrome coro	....	....	....	....	....	....
5970	MW206273.1	|Severe acute respiratory syndrome coro	....	....	....	....	....	....
5971	MW206274.1	|Severe acute respiratory syndrome coro	....	....	....	....	....	....
5972	MW206275.1	|Severe acute respiratory syndrome coro	....	....	....	....	....	....
5973	MW206279.1	|Severe acute respiratory syndrome coro	....	....	....	....	....	....
5974	MW206281.1	|Severe acute respiratory syndrome coro	....	....	....	....	....	....
5975	MW206282.1	|Severe acute respiratory syndrome coro	....	....	....	....	....	....
5976	MW206283.1	|Severe acute respiratory syndrome coro	....	....	....	....	....	....
5977	MW206284.1	|Severe acute respiratory syndrome coro	....	....	....	....	....	....
5978	MW206285.1	|Severe acute respiratory syndrome coro	....	....	....	....	....	....
5979	MW206286.1	|Severe acute respiratory syndrome coro	....	....	....	....	....	....
5980	MW206288.1	|Severe acute respiratory syndrome coro	....	.A..................	....	....	....	....
5981	MW206289.1	|Severe acute respiratory syndrome coro	....	....	....	....	....	....
5982	MW206290.1	|Severe acute respiratory syndrome coro	....	.A..................	....	....	....	....
5983	MW206291.1	|Severe acute respiratory syndrome coro	....	....	....	....	....	....
5984	MW206292.1	|Severe acute respiratory syndrome coro	....	....	....	....	....	....
5985	MW206293.1	|Severe acute respiratory syndrome coro	....	....	....	....	....	....
5986	MW206294.1	|Severe acute respiratory syndrome coro	....	....	....	....	....	....
5987	MW206295.1	|Severe acute respiratory syndrome coro	....	....	....	....	....	....
5988	MW206296.1	|Severe acute respiratory syndrome coro	....	....	....	....	....	....
5989	MW206297.1	|Severe acute respiratory syndrome coro	....	....	....	....	....	....
5990	MW206298.1	|Severe acute respiratory syndrome coro	....	....	....	....	....	....
5991	MW206299.1	|Severe acute respiratory syndrome coro	....	....	....	....	....	....
5992	MW206300.1	|Severe acute respiratory syndrome coro	....	....	....	....	....	....
5993	MW206301.1	|Severe acute respiratory syndrome coro	....	....	....	....	....	....
5994	MW206302.1	|Severe acute respiratory syndrome coro	....	....	....	....	....	....
5995	MW206303.1	|Severe acute respiratory syndrome coro	....	....	....	....	....	....
5996	MW206304.1	|Severe acute respiratory syndrome coro	....	....	....	....	....	....
5997	MW206305.1	|Severe acute respiratory syndrome coro	....	....	....	....	....	....
5998	MW206306.1	|Severe acute respiratory syndrome coro	....	....	....	....	....	....
5999	MW206307.1	|Severe acute respiratory syndrome coro	....	....	....	....	....	....
6000	MW206308.1	|Severe acute respiratory syndrome coro	....	.A..................	....	....	....	....
6001	MW206309.1	|Severe acute respiratory syndrome coro	....	.A..................	....	....	....	....
6002	MW206310.1	|Severe acute respiratory syndrome coro	....	....	....	....	....	....
6003	MW206311.1	|Severe acute respiratory syndrome coro	....	....	....	....	....	....
6004	MW206312.1	|Severe acute respiratory syndrome coro	....	.A..................	....	....	....	....
6005	MW206313.1	|Severe acute respiratory syndrome coro	....	....	....	....	....	....
6006	MW206314.1	|Severe acute respiratory syndrome coro	....	....	....	....	....	....
6007	MW206315.1	|Severe acute respiratory syndrome coro	....	....	....	....	....	....
6008	MW206316.1	|Severe acute respiratory syndrome coro	....	....	....	....	....	....
6009	MW206317.1	|Severe acute respiratory syndrome coro	....	.A..................	....	....	....	....
6010	MW206318.1	|Severe acute respiratory syndrome coro	....	....	....	....	....	....
6011	MW206319.1	|Severe acute respiratory syndrome coro	....	....	....	....	....	....
6012	MW206320.1	|Severe acute respiratory syndrome coro	....	....	....	....	....	....
6013	MW206321.1	|Severe acute respiratory syndrome coro	....	....	....	....	....	....
6014	MW206322.1	|Severe acute respiratory syndrome coro	....	.A..................	....	....	....	....
6015	MW206323.1	|Severe acute respiratory syndrome coro	....	....	....	....	....	....
6016	MW206324.1	|Severe acute respiratory syndrome coro	....	....	....	....	....	....
6017	MW206325.1	|Severe acute respiratory syndrome coro	....	....	....	....	....	....
6018	MW206326.1	|Severe acute respiratory syndrome coro	....	....	....	....	.........A..........	....
6019	MW206327.1	|Severe acute respiratory syndrome coro	....	....	....	....	....	....
6020	MW206328.1	|Severe acute respiratory syndrome coro	....	....	....	....	....	....
6021	MW206329.1	|Severe acute respiratory syndrome coro	....	....	....	....	....	....
6022	MW206330.1	|Severe acute respiratory syndrome coro	....	....	....	....	....	....
6023	MW206331.1	|Severe acute respiratory syndrome coro	....	....	....	....	....	....
6024	MW206332.1	|Severe acute respiratory syndrome coro	....	....	....	....	....	....
6025	MW206333.1	|Severe acute respiratory syndrome coro	....	....	....	....	....	....
6026	MW206334.1	|Severe acute respiratory syndrome coro	....	....	....	....	....	....
6027	MW206335.1	|Severe acute respiratory syndrome coro	....	....	....	....	....	....
6028	MW206336.1	|Severe acute respiratory syndrome coro	....	....	....	....	....	....
6029	MW206337.1	|Severe acute respiratory syndrome coro	....	....	....	....	....	....
6030	MW206338.1	|Severe acute respiratory syndrome coro	....	....	....	....	....	....
6031	MW206339.1	|Severe acute respiratory syndrome coro	....	.A..................	....	....	....	....
6032	MW206341.1	|Severe acute respiratory syndrome coro	....	....	....	....	....	....
6033	MW206342.1	|Severe acute respiratory syndrome coro	....	....	....	....	....	....
6034	MW206343.1	|Severe acute respiratory syndrome coro	....	....	....	....	....	....
6035	MW206344.1	|Severe acute respiratory syndrome coro	....	....	....	....	....	....
6036	MW206345.1	|Severe acute respiratory syndrome coro	....	....	....	....	....	....

6037	MW206346.1	|Severe acute respiratory syndrome coro	····················	·A··················	····················	····················	····················	····················
6038	MW206347.1	|Severe acute respiratory syndrome coro	····················	····················	····················	····················	·········T··········	····················
6039	MW206348.1	|Severe acute respiratory syndrome coro	····················	····················	····················	····················	····················	····················
6040	MW206349.1	|Severe acute respiratory syndrome coro	····················	·A··················	····················	····················	····················	····················
6041	MW206350.1	|Severe acute respiratory syndrome coro	····················	····················	····················	····················	····················	····················
6042	MW206351.1	|Severe acute respiratory syndrome coro	····················	····················	····················	····················	····················	····················
6043	MW206352.1	|Severe acute respiratory syndrome coro	····················	····················	····················	····················	····················	····················
6044	MW206353.1	|Severe acute respiratory syndrome coro	····················	····················	····················	····················	····················	····················
6045	MW206354.1	|Severe acute respiratory syndrome coro	····················	··G·················	····················	····················	····················	····················
6046	MW206355.1	|Severe acute respiratory syndrome coro	····················	····················	····················	····················	····················	····················
6047	MW206356.1	|Severe acute respiratory syndrome coro	····················	····················	····················	····················	····················	····················
6048	MW206357.1	|Severe acute respiratory syndrome coro	····················	····················	····················	····················	····················	····················
6049	MW206358.1	|Severe acute respiratory syndrome coro	····················	····················	····················	····················	····················	····················
6050	MW206359.1	|Severe acute respiratory syndrome coro	····················	····················	····················	····················	····················	····················
6051	MW206360.1	|Severe acute respiratory syndrome coro	····················	····················	····················	····················	····················	····················
6052	MW206361.1	|Severe acute respiratory syndrome coro	····················	····················	····················	····················	····················	····················
6053	MW206362.1	|Severe acute respiratory syndrome coro	····················	····················	····················	····················	····················	····················
6054	MW206363.1	|Severe acute respiratory syndrome coro	····················	····················	····················	····················	····················	····················
6055	MW206398.1	|Severe acute respiratory syndrome coro	····················	·A··················	····················	····················	····················	····················
6058	MW206409.1	|Severe acute respiratory syndrome coro	····················	·············A······	····················	····················	····················	····················
6059	MW206414.1	|Severe acute respiratory syndrome coro	····················	····················	····················	····················	····················	····················
6060	MW206415.1	|Severe acute respiratory syndrome coro	····················	····················	····················	····················	····················	····················
6061	MW206417.1	|Severe acute respiratory syndrome coro	····················	····················	····················	····················	····················	····················
6062	MW206418.1	|Severe acute respiratory syndrome coro	····················	····················	····················	····················	····················	····················
6063	MW206428.1	|Severe acute respiratory syndrome coro	····················	····················	····················	····················	····················	····················
6064	MW206431.1	|Severe acute respiratory syndrome coro	····················	····················	····················	····················	····················	····················
6065	MW206433.1	|Severe acute respiratory syndrome coro	····················	····················	····················	····················	····················	····················
6066	MW206434.1	|Severe acute respiratory syndrome coro	····················	····················	····················	····················	····················	····················
6067	MW206440.1	|Severe acute respiratory syndrome coro	····················	·A··················	····················	····················	····················	····················
6068	MW206448.1	|Severe acute respiratory syndrome coro	····················	·············A······	····················	····················	····················	····················
6069	MW206449.1	|Severe acute respiratory syndrome coro	····················	····················	····················	····················	····················	····················
6070	MW206450.1	|Severe acute respiratory syndrome coro	····················	·A··················	····················	····················	····················	····················
6072	MW206455.1	|Severe acute respiratory syndrome coro	····················	····················	····················	····················	····················	····················
6075	MW206459.1	|Severe acute respiratory syndrome coro	····················	····················	····················	····················	····················	····················
6076	MW206460.1	|Severe acute respiratory syndrome coro	····················	····················	····················	····················	····················	····················
6077	MW206479.1	|Severe acute respiratory syndrome coro	····················	····················	····················	····················	····················	····················
6078	MW206480.1	|Severe acute respiratory syndrome coro	····················	····················	····················	····················	····················	····················
6079	MW206481.1	|Severe acute respiratory syndrome coro	····················	····················	····················	····················	····················	····················
6080	MW206483.1	|Severe acute respiratory syndrome coro	····················	····················	····················	····················	····················	····················
6081	MW206485.1	|Severe acute respiratory syndrome coro	····················	····················	····················	····················	····················	····················
6082	MW206486.1	|Severe acute respiratory syndrome coro	····················	····················	····················	····················	····················	····················
6084	MW206490.1	|Severe acute respiratory syndrome coro	····················	····················	····················	····················	····················	····················
6085	MW206492.1	|Severe acute respiratory syndrome coro	····················	····················	····················	····················	····················	····················
6086	MW206493.1	|Severe acute respiratory syndrome coro	····················	····················	····················	····················	····················	····················
6087	MW206496.1	|Severe acute respiratory syndrome coro	····················	····················	····················	····················	····················	····················
6088	MW206501.1	|Severe acute respiratory syndrome coro	····················	····················	····················	····················	····················	····················
6089	MW206502.1	|Severe acute respiratory syndrome coro	····················	····················	····················	····················	····················	····················
6090	MW206506.1	|Severe acute respiratory syndrome coro	····················	····················	····················	····················	····················	····················
6091	MW206508.1	|Severe acute respiratory syndrome coro	····················	····················	····················	····················	····················	····················
6092	MW206509.1	|Severe acute respiratory syndrome coro	····················	····················	····················	····················	····················	····················
6093	MW206510.1	|Severe acute respiratory syndrome coro	····················	····················	····················	····················	····················	····················
6094	MW206511.1	|Severe acute respiratory syndrome coro	····················	····················	····················	····················	····················	····················
6095	MW206512.1	|Severe acute respiratory syndrome coro	····················	····················	····················	····················	····················	····················
6096	MW206513.1	|Severe acute respiratory syndrome coro	····················	····················	····················	····················	····················	····················
6097	MW206514.1	|Severe acute respiratory syndrome coro	····················	····················	····················	····················	····················	····················
6099	MW206516.1	|Severe acute respiratory syndrome coro	····················	····················	····················	····················	····················	····················
6100	MW206517.1	|Severe acute respiratory syndrome coro	····················	····················	····················	····················	····················	····················
6101	MW206518.1	|Severe acute respiratory syndrome coro	····················	····················	····················	····················	····················	····················
6102	MW206519.1	|Severe acute respiratory syndrome coro	····················	····················	····················	····················	····················	····················
6103	MW206521.1	|Severe acute respiratory syndrome coro	····················	····················	····················	····················	····················	····················
6104	MW206522.1	|Severe acute respiratory syndrome coro	····················	····················	····················	····················	····················	····················
6105	MW206523.1	|Severe acute respiratory syndrome coro	····················	····················	····················	····················	····················	····················
6106	MW206524.1	|Severe acute respiratory syndrome coro	····················	····················	····················	····················	····················	····················
6107	MW206525.1	|Severe acute respiratory syndrome coro	····················	····················	····················	····················	····················	····················
6108	MW206528.1	|Severe acute respiratory syndrome coro	····················	····················	····················	····················	····················	····················
6109	MW206529.1	|Severe acute respiratory syndrome coro	····················	····················	····················	····················	····················	····················
6110	MW206530.1	|Severe acute respiratory syndrome coro	····················	····················	····················	····················	····················	····················
6111	MW206531.1	|Severe acute respiratory syndrome coro	····················	····················	····················	····················	····················	····················
6112	MW206532.1	|Severe acute respiratory syndrome coro	····················	····················	····················	····················	····················	····················
6113	MW206533.1	|Severe acute respiratory syndrome coro	····················	····················	····················	····················	····················	····················
6114	MW206534.1	|Severe acute respiratory syndrome coro	····················	····················	····················	····················	····················	····················
6115	MW206535.1	|Severe acute respiratory syndrome coro	····················	····················	····················	····················	····················	····················
6116	MW206536.1	|Severe acute respiratory syndrome coro	····················	····················	····················	····················	····················	····················
6117	MW206539.1	|Severe acute respiratory syndrome coro	····················	····················	····················	····················	····················	····················
6118	MW206540.1	|Severe acute respiratory syndrome coro	····················	····················	····················	····················	····················	····················
6119	MW206541.1	|Severe acute respiratory syndrome coro	····················	····················	····················	····················	····················	····················
6120	MW206542.1	|Severe acute respiratory syndrome coro	····················	····················	····················	····················	····················	····················
6121	MW206547.1	|Severe acute respiratory syndrome coro	····················	····················	····················	····················	····················	····················

6123	MW206554.1	|Severe acute respiratory syndrome coro	....	....	....	....	....	....	
6124	MW206555.1	|Severe acute respiratory syndrome coro	....	....	....	....	....	....	
6126	MW206561.1	|Severe acute respiratory syndrome coro	....	....	....	....	....	....	
6127	MW206568.1	|Severe acute respiratory syndrome coro	....	....	....	....	....	....	
6128	MW206573.1	|Severe acute respiratory syndrome coro	....	....	....	....	....	....	
6129	MW206582.1	|Severe acute respiratory syndrome coro	....	....	....	....	....	....	
6130	MW206583.1	|Severe acute respiratory syndrome coro	....	....	....	....	....	....	
6131	MW206585.1	|Severe acute respiratory syndrome coro	....	....	....	....	....	....	
6132	MW206587.1	|Severe acute respiratory syndrome coro	....	....	....	....	....	....	
6133	MW206588.1	|Severe acute respiratory syndrome coro	....	....	....	....	....	....	
6134	MW206589.1	|Severe acute respiratory syndrome coro	....	....	....	....	.NNN................	....	1
6135	MW206590.1	|Severe acute respiratory syndrome coro	....	....	....	....	....	....	
6136	MW206593.1	|Severe acute respiratory syndrome coro	....	....	....	....	....	....	
6137	MW206594.1	|Severe acute respiratory syndrome coro	....	....	....	....	....	....	
6138	MW206595.1	|Severe acute respiratory syndrome coro	....	....	....	....	....	....	
6139	MW206596.1	|Severe acute respiratory syndrome coro	....	....	....	....	....	....	
6140	MW206597.1	|Severe acute respiratory syndrome coro	....	....	....	....	.NN.................	....	
6141	MW206600.1	|Severe acute respiratory syndrome coro	....	....	....	....	....	....	
6142	MW206601.1	|Severe acute respiratory syndrome coro	....	....	....	....	....	....	
6143	MW206604.1	|Severe acute respiratory syndrome coro	....	....	....	....	....	....	
6144	MW206605.1	|Severe acute respiratory syndrome coro	....	....	....	....	....	....	
6145	MW206606.1	|Severe acute respiratory syndrome coro	....	.A..................	....	....	....	....	
6146	MW206607.1	|Severe acute respiratory syndrome coro	....	....	....	....	....	....	
6147	MW206608.1	|Severe acute respiratory syndrome coro	....	....	....	....	....	....	
6149	MW206610.1	|Severe acute respiratory syndrome coro	....	....	....	....	....	....	
6150	MW206611.1	|Severe acute respiratory syndrome coro	....	....	....	....	....	....	
6151	MW206615.1	|Severe acute respiratory syndrome coro	....	....	....	....	....	....	
6152	MW206616.1	|Severe acute respiratory syndrome coro	....	....	....	....	....	....	
6153	MW206617.1	|Severe acute respiratory syndrome coro	....	....	....	....	....	....	
6154	MW206619.1	|Severe acute respiratory syndrome coro	....	....	....	....	....	....	
6155	MW206620.1	|Severe acute respiratory syndrome coro	....	....	....	....	....	....	
6156	MW206624.1	|Severe acute respiratory syndrome coro	....	....	....	....	....	....	
6157	MW206625.1	|Severe acute respiratory syndrome coro	....	....	....	....	....	....	
6158	MW206627.1	|Severe acute respiratory syndrome coro	....	....	....	....	....	....	
6159	MW206628.1	|Severe acute respiratory syndrome coro	....	....	....	....	....	....	
6160	MW206635.1	|Severe acute respiratory syndrome coro	....	....	....	....	....	....	
6161	MW208007.1	|Severe acute respiratory syndrome coro	....	....	....	....	....	....	
6162	MW208008.1	|Severe acute respiratory syndrome coro	....	....	....	....	....	....	
6163	MW208009.1	|Severe acute respiratory syndrome coro	....	....	....	....	....	....	
6164	MW208010.1	|Severe acute respiratory syndrome coro	....	....	....	....	....	....	
6165	MW208012.1	|Severe acute respiratory syndrome coro	....	......A.............	....	....	....	....	
6166	MW208013.1	|Severe acute respiratory syndrome coro	....	.A..................	....	....	....	....	
6167	MW208014.1	|Severe acute respiratory syndrome coro	....	....	....	....	....	....	
6168	MW208015.1	|Severe acute respiratory syndrome coro	....	.A..................	....	....	....	....	
6169	MW208016.1	|Severe acute respiratory syndrome coro	....	....	....	....	....	....	
6170	MW208017.1	|Severe acute respiratory syndrome coro	....	.A..................	....	....	....	....	
6171	MW208018.1	|Severe acute respiratory syndrome coro	....	....	....	....	....	....	
6172	MW208019.1	|Severe acute respiratory syndrome coro	....	......A.............	....	....	....	....	
6173	MW208020.1	|Severe acute respiratory syndrome coro	....	....	....	....	....	....	
6174	MW208021.1	|Severe acute respiratory syndrome coro	....	....	....	....	....	....	
6175	MW208022.1	|Severe acute respiratory syndrome coro	....	....	....	....	....	....	
6176	MW208023.1	|Severe acute respiratory syndrome coro	....	....	....	....	....	....	
6177	MW208024.1	|Severe acute respiratory syndrome coro	....	....	....	....	....	....	
6178	MW208025.1	|Severe acute respiratory syndrome coro	....	....	....	....	....	....	
6179	MW208026.1	|Severe acute respiratory syndrome coro	....	....	....	....	....	....	
6180	MW208027.1	|Severe acute respiratory syndrome coro	........T...........	.A..................	....	....	....	....	
6181	MW208028.1	|Severe acute respiratory syndrome coro	....	....	....	....	....	....	
6182	MW208029.1	|Severe acute respiratory syndrome coro	....	.A..................	....	....	....	....	
6183	MW208030.1	|Severe acute respiratory syndrome coro	....	....	....	....	....	....	
6184	MW208031.1	|Severe acute respiratory syndrome coro	....	....	....	....	....	....	
6185	MW208032.1	|Severe acute respiratory syndrome coro	....	....A...............	....	....	....	....	
6186	MW208033.1	|Severe acute respiratory syndrome coro	....	....	....	....	....	....	
6187	MW208034.1	|Severe acute respiratory syndrome coro	....	....	....	....	....	....	
6188	MW208036.1	|Severe acute respiratory syndrome coro	....	....	....	....	....	....	
6189	MW208037.1	|Severe acute respiratory syndrome coro	....	....	....	....	....	....	
6190	MW208039.1	|Severe acute respiratory syndrome coro	....	.A..................	....	....	....	....	
6191	MW210930.1	|Severe acute respiratory syndrome coro	....	.A..................	....	....	....	....	
6192	MW210931.1	|Severe acute respiratory syndrome coro	....	.A..................	....	....	....	....	
6193	MW210932.1	|Severe acute respiratory syndrome coro	....	.A..................	....	....	....	....	
6194	MW210933.1	|Severe acute respiratory syndrome coro	....	.A..................	....	....	....	....	
6195	MW210934.1	|Severe acute respiratory syndrome coro	....	.A..................	....	....	....	....	
6196	MW210935.1	|Severe acute respiratory syndrome coro	....	....	....	....	....	....	
6197	MW210936.1	|Severe acute respiratory syndrome coro	....	....	....	....	....	....	
6198	MW210937.1	|Severe acute respiratory syndrome coro	....	....	....	....	....	....	
6199	MW210938.1	|Severe acute respiratory syndrome coro	....	....	....	....	....	....	
6200	MW210939.1	|Severe acute respiratory syndrome coro	....	....	....	....	....	....	
6201	MW210940.1	|Severe acute respiratory syndrome coro	....	....	....	....	....	....	
6202	MW210941.1	|Severe acute respiratory syndrome coro	....	....	....	....	....	....	

6203	MW210942.1	|Severe acute respiratory syndrome coro	····················	····················	····················	····················	····················	····················	
6204	MW210960.1	|Severe acute respiratory syndrome coro	····················	·A··················	····················	····················	····················	····················	
6205	MW210965.1	|Severe acute respiratory syndrome coro	····················	····················	····················	····················	····················	····················	
6206	MW210975.1	|Severe acute respiratory syndrome coro	····················	····················	····················	····················	····················	····················	
6207	MW210976.1	|Severe acute respiratory syndrome coro	····················	····················	····················	····················	····················	····················	
6208	MW210977.1	|Severe acute respiratory syndrome coro	····················	····················	····················	····················	····················	····················	
6209	MW210978.1	|Severe acute respiratory syndrome coro	····················	····················	····················	····················	····················	····················	
6210	MW210979.1	|Severe acute respiratory syndrome coro	····················	····················	····················	····················	····················	····················	
6211	MW210980.1	|Severe acute respiratory syndrome coro	····················	····················	····················	····················	····················	····················	
6212	MW210982.1	|Severe acute respiratory syndrome coro	····················	····················	····················	····················	····················	····················	
6213	MW210983.1	|Severe acute respiratory syndrome coro	····················	····················	····················	····················	····················	····················	
6214	MW210984.1	|Severe acute respiratory syndrome coro	····················	····················	····················	····················	····················	····················	
6215	MW210985.1	|Severe acute respiratory syndrome coro	····················	····················	····················	····················	····················	····················	
6216	MW210986.1	|Severe acute respiratory syndrome coro	····················	····················	····················	····················	····················	····················	
6217	MW210987.1	|Severe acute respiratory syndrome coro	····················	····················	····················	····················	····················	····················	
6218	MW210988.1	|Severe acute respiratory syndrome coro	····················	····················	····················	····················	····················	····················	
6219	MW210989.1	|Severe acute respiratory syndrome coro	····················	····················	····················	····················	····················	····················	
6220	MW210990.1	|Severe acute respiratory syndrome coro	····················	····················	····················	····················	····················	····················	
6221	MW210991.1	|Severe acute respiratory syndrome coro	····················	····················	····················	····················	····················	····················	
6222	MW210992.1	|Severe acute respiratory syndrome coro	····················	····················	····················	····················	····················	····················	
6223	MW210993.1	|Severe acute respiratory syndrome coro	····················	····················	····················	····················	····················	····················	
6224	MW210994.1	|Severe acute respiratory syndrome coro	····················	····················	····················	····················	····················	····················	
6225	MW210995.1	|Severe acute respiratory syndrome coro	····················	····················	····················	····················	····················	····················	
6226	MW210996.1	|Severe acute respiratory syndrome coro	····················	····················	····················	····················	····················	····················	
6227	MW210997.1	|Severe acute respiratory syndrome coro	····················	····················	····················	····················	····················	····················	
6228	MW210998.1	|Severe acute respiratory syndrome coro	····················	·A··················	····················	····················	····················	····················	
6229	MW210999.1	|Severe acute respiratory syndrome coro	····················	·A··················	····················	····················	····················	····················	
6230	MW211001.1	|Severe acute respiratory syndrome coro	····················	·A··················	····················	····················	····················	····················	
6231	MW211002.1	|Severe acute respiratory syndrome coro	····················	····················	····················	····················	····················	····················	
6232	MW211003.1	|Severe acute respiratory syndrome coro	····················	····················	····················	····················	····················	····················	
6233	MW211004.1	|Severe acute respiratory syndrome coro	····················	····················	····················	····················	····················	····················	
6234	MW211005.1	|Severe acute respiratory syndrome coro	···········T········	····················	····················	····················	····················	····················	
6235	MW211006.1	|Severe acute respiratory syndrome coro	···········T········	····················	····················	····················	····················	····················	
6236	MW211008.1	|Severe acute respiratory syndrome coro	····················	····················	····················	····················	····················	····················	
6237	MW211009.1	|Severe acute respiratory syndrome coro	····················	····················	····················	····················	····················	····················	
6238	MW211010.1	|Severe acute respiratory syndrome coro	····················	····················	····················	····················	····················	····················	
6239	MW199143.1	|Severe acute respiratory syndrome coro	····················	····················	····················	····················	····················	····················	
6240	MW199149.1	|Severe acute respiratory syndrome coro	····················	····················	····················	····················	····················	····················	
6241	MW199150.1	|Severe acute respiratory syndrome coro	····················	····················	····················	····················	····················	····················	
6242	MW199186.1	|Severe acute respiratory syndrome coro	····················	····················	····················	····················	····················	····················	
6243	MW201576.1	|Severe acute respiratory syndrome coro	····················	·A··················	····················	····················	····················	····················	
6244	MW201577.1	|Severe acute respiratory syndrome coro	····················	····················	····················	····················	····················	····················	
6245	MW201578.1	|Severe acute respiratory syndrome coro	····················	····················	····················	····················	····················	····················	
6246	MW202150.1	|Severe acute respiratory syndrome coro	····················	·············A······	····················	····················	····················	····················	
6247	MW202151.1	|Severe acute respiratory syndrome coro	····················	····················	····················	····················	····················	····················	
6248	MW202152.1	|Severe acute respiratory syndrome coro	····················	·············A······	····················	····················	····················	····················	
6249	MW202153.1	|Severe acute respiratory syndrome coro	····················	·············A······	····················	····················	····················	····················	
6250	MW202154.1	|Severe acute respiratory syndrome coro	····················	·············A······	····················	····················	····················	····················	
6251	MW202155.1	|Severe acute respiratory syndrome coro	····················	·············A······	····················	····················	····················	····················	
6252	MW202156.1	|Severe acute respiratory syndrome coro	····················	·············A······	····················	····················	····················	····················	
6253	MW202157.1	|Severe acute respiratory syndrome coro	····················	·············A······	····················	····················	····················	····················	
6254	MW202158.1	|Severe acute respiratory syndrome coro	····················	·············A······	····················	····················	····················	····················	
6255	MW202159.1	|Severe acute respiratory syndrome coro	····················	·············A······	····················	····················	····················	····················	
6256	MW202160.1	|Severe acute respiratory syndrome coro	····················	·············A······	····················	····················	····················	····················	
6257	MW202161.1	|Severe acute respiratory syndrome coro	····················	·············A······	····················	····················	····················	····················	
6258	MW202162.1	|Severe acute respiratory syndrome coro	····················	·A··················	····················	····················	····················	····················	
6259	MW202163.1	|Severe acute respiratory syndrome coro	····················	·············A······	····················	····················	·NNN················	····················	1
6260	MW202164.1	|Severe acute respiratory syndrome coro	····················	·············A······	····················	····················	····················	····················	
6261	MW202165.1	|Severe acute respiratory syndrome coro	····················	····················	····················	····················	····················	····················	
6262	MW202166.1	|Severe acute respiratory syndrome coro	····················	·············A······	····················	····················	····················	····················	
6263	MW202167.1	|Severe acute respiratory syndrome coro	····················	·············A······	····················	····················	····················	····················	
6264	MW202168.1	|Severe acute respiratory syndrome coro	····················	····················	····················	····················	····················	····················	
6265	MW202169.1	|Severe acute respiratory syndrome coro	····················	····················	····················	····················	····················	····················	
6266	MW202170.1	|Severe acute respiratory syndrome coro	····················	·A··················	····················	····················	····················	····················	
6267	MW202171.1	|Severe acute respiratory syndrome coro	····················	····················	····················	····················	····················	····················	
6268	MW202172.1	|Severe acute respiratory syndrome coro	····················	····················	····················	····················	····················	····················	
6269	MW202173.1	|Severe acute respiratory syndrome coro	····················	·A··················	····················	····················	····················	····················	
6270	MW202174.1	|Severe acute respiratory syndrome coro	····················	······A······A······	····················	····················	····················	····················	
6271	MW202175.1	|Severe acute respiratory syndrome coro	····················	····················	····················	····················	····················	····················	
6272	MW202176.1	|Severe acute respiratory syndrome coro	····················	····················	····················	····················	····················	····················	
6273	MW202177.1	|Severe acute respiratory syndrome coro	····················	····················	····················	····················	····················	····················	
6274	MW202178.1	|Severe acute respiratory syndrome coro	····················	····················	····················	····················	····················	····················	
6275	MW202179.1	|Severe acute respiratory syndrome coro	····················	····················	····················	····················	····················	····················	
6276	MW202180.1	|Severe acute respiratory syndrome coro	····················	····················	····················	····················	····················	····················	
6277	MW202181.1	|Severe acute respiratory syndrome coro	····················	····················	····················	····················	····················	····················	
6278	MW202182.1	|Severe acute respiratory syndrome coro	····················	····················	····················	····················	····················	····················	
6279	MW202183.1	|Severe acute respiratory syndrome coro	····················	····················	····················	····················	····················	····················	
6280	MW202184.1	|Severe acute respiratory syndrome coro	····················	····················	····················	····················	····················	····················	

6281	MW202185.1	Severe acute respiratory syndrome coro	....	....	....	....	....	....	
6282	MW202186.1	Severe acute respiratory syndrome coro	....	....	....	....	....	....	
6283	MW202187.1	Severe acute respiratory syndrome coro	....	....	....	....	....	....	
6284	MW202188.1	Severe acute respiratory syndrome coro	....	....	....	....	.NN.................	....	
6285	MW202189.1	Severe acute respiratory syndrome coro	....	....	....	....	....	....	
6286	MW202190.1	Severe acute respiratory syndrome coro	....	....	....	....	....	....	
6287	MW202191.1	Severe acute respiratory syndrome coro	....	....	....	....	....	....	
6288	MW202192.1	Severe acute respiratory syndrome coro	....	....	....	....	....	....	
6289	MW202193.1	Severe acute respiratory syndrome coro	....	....	....	....	....	....	
6290	MW202194.1	Severe acute respiratory syndrome coro	....	....	....	....	....	....	
6291	MW202195.1	Severe acute respiratory syndrome coro	....	....	....	....	....	....	
6292	MW202196.1	Severe acute respiratory syndrome coro	....	....	....	....	....	....	
6293	MW202197.1	Severe acute respiratory syndrome coro	....	....	....	....	....	....	
6294	MW202198.1	Severe acute respiratory syndrome coro	....	....	....	....	....	....	
6295	MW202199.1	Severe acute respiratory syndrome coro	....	.............A......	....	....	....	....	
6296	MW202200.1	Severe acute respiratory syndrome coro	....	.............A......	....	....	....	....	
6297	MW202201.1	Severe acute respiratory syndrome coro	....	.............A......	....	....	....	....	
6298	MW202202.1	Severe acute respiratory syndrome coro	....	.............A......	....	....	....	....	
6299	MW202203.1	Severe acute respiratory syndrome coro	....	.............A......	....	....	....	....	
6300	MW202204.1	Severe acute respiratory syndrome coro	....	.............A......	....	....	....	....	
6301	MW202205.1	Severe acute respiratory syndrome coro	....	.............A......	....	....	....	....	
6302	MW202206.1	Severe acute respiratory syndrome coro	....	.............A......	....	....	....	....	
6303	MW202207.1	Severe acute respiratory syndrome coro	....	.............A......	....	....	....	....	
6304	MW202208.1	Severe acute respiratory syndrome coro	....	.............A......	....	....	....	....	
6305	MW202209.1	Severe acute respiratory syndrome coro	....	.............A......	....	....	....	....	
6306	MW202210.1	Severe acute respiratory syndrome coro	....	....	....	....	....	....	
6307	MW202211.1	Severe acute respiratory syndrome coro	....	....	....	....	....	....	
6308	MW202212.1	Severe acute respiratory syndrome coro	....	....	....	....	....	....	
6309	MW202213.1	Severe acute respiratory syndrome coro	....	....	....	....	....	....	
6310	MW202214.1	Severe acute respiratory syndrome coro	....	....	....	....	....	....	
6311	MW202215.1	Severe acute respiratory syndrome coro	....	....	....	....	....	....	
6312	MW202216.1	Severe acute respiratory syndrome coro	....	.............A......	....	....	....	....	
6313	MW202217.1	Severe acute respiratory syndrome coro	....	....	....	....	....	....	
6314	MW202218.1	Severe acute respiratory syndrome coro	....	....	....	....	....	....	
6315	MW202219.1	Severe acute respiratory syndrome coro	....	.A..................	....	....	....	....	
6316	MW202220.1	Severe acute respiratory syndrome coro	....	.............A......	....	....	....	....	
6317	MW202221.1	Severe acute respiratory syndrome coro	....	.............A......	....	....	....	....	
6318	MW202222.1	Severe acute respiratory syndrome coro	....	.............A......	....	....	....	....	
6319	MW202223.1	Severe acute respiratory syndrome coro	....	.............A......	....	....	....	....	
6320	MW202224.1	Severe acute respiratory syndrome coro	....	.............A......	....	....	....	....	
6321	MW202225.1	Severe acute respiratory syndrome coro	....	.............A......	....	....	NNN.................	....	1
6322	MW202226.1	Severe acute respiratory syndrome coro	....	.............A......	....	....	....	....	
6323	MW202227.1	Severe acute respiratory syndrome coro	....	.............A......	....	....	....	....	
6324	MW193407.1	Severe acute respiratory syndrome coro	....	....	....	....	....	....	
6325	MW193408.1	Severe acute respiratory syndrome coro	....	....	....	....	....	....	
6326	MW193889.1	Severe acute respiratory syndrome coro	....	....	....	....	....	....	
6327	MW193967.2	Severe acute respiratory syndrome coro	....	....	....	....	....	....	
6328	MW194121.1	Severe acute respiratory syndrome coro	....	....A...............	....	....	....	....	
6329	MW195531.1	Severe acute respiratory syndrome coro	....	....	....	....	....	....	
6330	MW195532.1	Severe acute respiratory syndrome coro	....	....	....	....	....	....	
6331	MW195533.1	Severe acute respiratory syndrome coro	....	.A..................	....	....	....	....	
6332	MW195534.1	Severe acute respiratory syndrome coro	....	....	....	....	....	....	
6333	MW195535.1	Severe acute respiratory syndrome coro	....	....	....	....	....	....	
6334	MW195536.1	Severe acute respiratory syndrome coro	....	....	....	....	....	....	
6335	MW195537.1	Severe acute respiratory syndrome coro	....	....	....	....	....	....	
6336	MW195538.1	Severe acute respiratory syndrome coro	....	....	....	....	....	....	
6337	MW195539.1	Severe acute respiratory syndrome coro	....	....	....	....	....	....	
6338	MW195540.1	Severe acute respiratory syndrome coro	....	....	....	....	....	....	
6339	MW195541.1	Severe acute respiratory syndrome coro	....	....	....	....	....	....	
6340	MW195542.1	Severe acute respiratory syndrome coro	....	....	....	....	....	....	
6341	MW195543.1	Severe acute respiratory syndrome coro	....	....	....	....	....	....	
6342	MW195544.1	Severe acute respiratory syndrome coro	....	.A..................	....	....	....	....	
6343	MW195545.1	Severe acute respiratory syndrome coro	....	.A..................	....	....	....	....	
6344	MW195546.1	Severe acute respiratory syndrome coro	....	.A..................	....	....	....	....	
6345	MW197470.1	Severe acute respiratory syndrome coro	....	....	....	....	....	....	
6346	MW197471.1	Severe acute respiratory syndrome coro	....	....	....	....	....	....	
6347	MW197472.1	Severe acute respiratory syndrome coro	....	....	....	....	....	....	
6348	MW197473.1	Severe acute respiratory syndrome coro	....	....	....	....	....	....	
6349	MW197474.1	Severe acute respiratory syndrome coro	....	....	....	....	....	....	
6350	MW197475.1	Severe acute respiratory syndrome coro	....	....	....	....	....	....	
6351	MW197476.1	Severe acute respiratory syndrome coro	....	....	....	....	....	....	
6352	MW197477.1	Severe acute respiratory syndrome coro	....	....	....	....	....	....	
6353	MW197479.1	Severe acute respiratory syndrome coro	....	....	....	....	....	....	
6354	MW197480.1	Severe acute respiratory syndrome coro	....	....	....	....	....	....	
6355	MW197481.1	Severe acute respiratory syndrome coro	....	....	....	....	....	....	
6356	MW197482.1	Severe acute respiratory syndrome coro	....	....	....	....	....	....	
6357	MW197483.1	Severe acute respiratory syndrome coro	....	.............A......	....	....	....	....	
6358	MW197485.1	Severe acute respiratory syndrome coro	....	....	....	....	....	....	

6359 MW197486.1 |Severe acute respiratory syndrome coro ···················· ·A·················· ···················· ···················· ···················· ····················
6360 MW197487.1 |Severe acute respiratory syndrome coro ···················· ···················· ···················· ···················· ···················· ····················
6361 MW197488.1 |Severe acute respiratory syndrome coro ···················· ···················· ···················· ···················· ···················· ····················
6362 MW197489.1 |Severe acute respiratory syndrome coro ···················· ·A·················· ···················· ···················· ···················· ····················
6363 MW197490.1 |Severe acute respiratory syndrome coro ···················· ···················· ···················· ···················· ···················· ····················
6364 MW197491.1 |Severe acute respiratory syndrome coro ···················· ···················· ···················· ···················· ···················· ····················
6365 MW197492.1 |Severe acute respiratory syndrome coro ···················· ···················· ···················· ···················· ···················· ····················
6366 MW197493.1 |Severe acute respiratory syndrome coro ···················· ···················· ···················· ···················· ···················· ····················
6367 MW197494.1 |Severe acute respiratory syndrome coro ···················· ···················· ···················· ···················· ···················· ····················
6368 MW197496.1 |Severe acute respiratory syndrome coro ···················· ·A·················· ···················· ···················· ···················· ····················
6369 MW197497.1 |Severe acute respiratory syndrome coro ···················· ·A·················· ···················· ···················· ···················· ····················
6370 MW197498.1 |Severe acute respiratory syndrome coro ···················· ···················· ···················· ···················· ···················· ····················
6371 MW059036.1 |Severe acute respiratory syndrome coro ···················· ···················· ···················· ···················· ···················· ····················
6372 MW181704.1 |Severe acute respiratory syndrome coro ···················· ···················· ···················· ···················· ···················· ····················
6373 MW181705.1 |Severe acute respiratory syndrome coro ···················· ···················· ···················· ···················· ···················· ····················
6374 MW181706.1 |Severe acute respiratory syndrome coro ···················· ···················· ···················· ···················· ···················· ····················
6375 MW181707.1 |Severe acute respiratory syndrome coro ···················· ···················· ···················· ···················· ···················· ····················
6376 MW181708.1 |Severe acute respiratory syndrome coro ···················· ···················· ···················· ···················· ···················· ····················
6377 MW181709.1 |Severe acute respiratory syndrome coro ···················· ···················· ···················· ···················· ···················· ····················
6378 MW181710.1 |Severe acute respiratory syndrome coro ···················· ···················· ···················· ···················· ···················· ····················
6379 MW181711.1 |Severe acute respiratory syndrome coro ···················· ···················· ···················· ···················· ···················· ····················
6380 MW181712.1 |Severe acute respiratory syndrome coro ···················· ···················· ···················· ···················· ···················· ····················
6381 MW181713.1 |Severe acute respiratory syndrome coro ···················· ···················· ···················· ···················· ···················· ····················
6382 MW181714.1 |Severe acute respiratory syndrome coro ···················· ···················· ···················· ···················· ···················· ····················
6383 MW181716.1 |Severe acute respiratory syndrome coro ···················· ···················· ···················· ···················· ···················· ····················
6384 MW181718.1 |Severe acute respiratory syndrome coro ···················· ···················· ···················· ···················· ···················· ····················
6385 MW181719.1 |Severe acute respiratory syndrome coro ···················· ···················· ···················· ···················· ···················· ····················
6386 MW181720.1 |Severe acute respiratory syndrome coro ···················· ···················· ···················· ···················· ···················· ····················
6387 MW181721.1 |Severe acute respiratory syndrome coro ···················· ···················· ···················· ···················· ···················· ····················
6388 MW181722.1 |Severe acute respiratory syndrome coro ···················· ···················· ···················· ···················· ···················· ····················
6389 MW181723.1 |Severe acute respiratory syndrome coro ···················· ···················· ···················· ···················· ···················· ····················
6390 MW181724.1 |Severe acute respiratory syndrome coro ···················· ···················· ···················· ···················· ···················· ····················
6391 MW181725.1 |Severe acute respiratory syndrome coro ···················· ···················· ···················· ···················· ···················· ····················
6392 MW181726.1 |Severe acute respiratory syndrome coro ···················· ···················· ···················· ···················· ···················· ····················
6393 MW181727.1 |Severe acute respiratory syndrome coro ···················· ···················· ···················· ···················· ···················· ····················
6394 MW181728.1 |Severe acute respiratory syndrome coro ···················· ···················· ···················· ···················· ···················· ····················
6395 MW181729.1 |Severe acute respiratory syndrome coro ···················· ···················· ···················· ···················· ···················· ····················
6396 MW181730.1 |Severe acute respiratory syndrome coro ···················· ···················· ···················· ···················· ···················· ····················
6397 MW181731.1 |Severe acute respiratory syndrome coro ···················· ···················· ···················· ···················· ···················· ····················
6398 MW181733.1 |Severe acute respiratory syndrome coro ···················· ···················· ···················· ···················· ···················· ····················
6399 MW181737.1 |Severe acute respiratory syndrome coro ···················· ···················· ···················· ···················· ···················· ····················
6400 MW181740.1 |Severe acute respiratory syndrome coro ···················· ·A·················· ···················· ···················· ···················· ··A·················
6401 MW181741.1 |Severe acute respiratory syndrome coro ···················· ·A·················· ···················· ···················· ···················· ··A·················
6402 MW181742.1 |Severe acute respiratory syndrome coro ···················· ·A·················· ···················· ···················· ···················· ··A·················
6403 MW181743.1 |Severe acute respiratory syndrome coro ···················· ·A·················· ···················· ···················· ···················· ··A·················
6404 MW181744.1 |Severe acute respiratory syndrome coro ···················· ···················· ···················· ···················· ···················· ····················
6405 MW181745.1 |Severe acute respiratory syndrome coro ···················· ···················· ···················· ···················· ···················· ····················
6406 MW181746.1 |Severe acute respiratory syndrome coro ···················· ···················· ···················· ···················· ···················· ····················
6407 MW181747.1 |Severe acute respiratory syndrome coro ···················· ···················· ···················· ···················· ···················· ····················
6408 MW181748.1 |Severe acute respiratory syndrome coro ···················· ···················· ···················· ···················· ···················· ····················
6409 MW181749.1 |Severe acute respiratory syndrome coro ···················· ···················· ···················· ···················· ···················· ····················
6410 MW181751.1 |Severe acute respiratory syndrome coro ···················· ···················· ···················· ···················· ···················· ····················
6411 MW181752.1 |Severe acute respiratory syndrome coro ···················· ···················· ···················· ···················· ···················· ····················
6412 MW181753.1 |Severe acute respiratory syndrome coro ···················· ·A·················· ···················· ···················· ···················· ··A·················
6413 MW181754.1 |Severe acute respiratory syndrome coro ···················· ·A·················· ···················· ···················· ···················· ··A·················
6414 MW181758.1 |Severe acute respiratory syndrome coro ···················· ·A·················· ···················· ···················· ···················· ····················
6415 MW181759.1 |Severe acute respiratory syndrome coro ···················· ···················· ···················· ···················· ···················· ····················
6416 MW181761.1 |Severe acute respiratory syndrome coro ···················· ·A·················· ···················· ···················· ···················· ··A·················
6417 MW181764.1 |Severe acute respiratory syndrome coro ···················· ···················· ···················· ···················· ···················· ····················
6418 MT412338.1 |Severe acute respiratory syndrome coro ···················· ···················· ···················· ···················· ···················· ····················
6419 MT412339.1 |Severe acute respiratory syndrome coro ···················· ···················· ···················· ···················· ···················· ····················
6420 MT412340.1 |Severe acute respiratory syndrome coro ···················· ···················· ···················· ···················· ···················· ····················
6421 MT581410.1 |Severe acute respiratory syndrome coro ···················· ···················· ···················· ···················· ···················· ····················
6422 MT581411.1 |Severe acute respiratory syndrome coro ···················· ···················· ···················· ···················· ···················· ····················
6423 MT581412.1 |Severe acute respiratory syndrome coro ···················· ···················· ···················· ···················· ···················· ····················
6424 MT581413.1 |Severe acute respiratory syndrome coro ···················· ···················· ···················· ···················· ···················· ····················
6425 MT581414.1 |Severe acute respiratory syndrome coro ···················· ···················· ···················· ···················· ···················· ····················
6426 MT581415.1 |Severe acute respiratory syndrome coro ···················· ···················· ···················· ···················· ···················· ····················
6427 MT581416.1 |Severe acute respiratory syndrome coro ···················· ···················· ···················· ···················· ···················· ····················
6428 MT581417.1 |Severe acute respiratory syndrome coro ···················· ···················· ···················· ···················· ···················· ····················
6429 MT581418.1 |Severe acute respiratory syndrome coro ···················· ···················· ···················· ···················· ···················· ····················
6430 MT581419.1 |Severe acute respiratory syndrome coro ···················· ···················· ···················· ···················· ···················· ····················
6431 MT581420.1 |Severe acute respiratory syndrome coro ···················· ···················· ···················· ···················· ···················· ····················
6432 MT581421.1 |Severe acute respiratory syndrome coro ···················· ···················· ···················· ···················· ···················· ····················
6433 MT581422.1 |Severe acute respiratory syndrome coro ···················· ···················· ···················· ···················· ···················· ····················
6434 MT581423.1 |Severe acute respiratory syndrome coro ···················· ···················· ···················· ···················· ···················· ····················
6435 MT581426.1 |Severe acute respiratory syndrome coro ···················· ···················· ···················· ···················· ···················· ····················
6436 MT581427.1 |Severe acute respiratory syndrome coro ···················· ···················· ···················· ···················· ···················· ····················

```
6437 MT581428.1 |Severe acute respiratory syndrome coro    ....................  ....................  ....................  ....................  ....................  ....................
6438 MT581429.1 |Severe acute respiratory syndrome coro    ....................  ....................  ....................  ....................  ....................  ....................
6439 MT581430.1 |Severe acute respiratory syndrome coro    ....................  ....................  ....................  ....................  ....................  ....................
6440 MT581431.1 |Severe acute respiratory syndrome coro    ....................  ....................  ....................  ....................  ....................  ....................
6441 MT581432.1 |Severe acute respiratory syndrome coro    ....................  ....................  ....................  ....................  ....................  ....................
6442 MT581434.1 |Severe acute respiratory syndrome coro    ....................  ....................  ....................  ....................  ....................  ....................
6443 MT581435.1 |Severe acute respiratory syndrome coro    ....................  ....................  ....................  ....................  ....................  ....................
6444 MT581436.1 |Severe acute respiratory syndrome coro    ....................  ....................  ....................  ....................  ....................  ....................
6445 MT607972.1 |Severe acute respiratory syndrome coro    ....................  ....................  ....................  ....................  ....................  ....................
6446 MT607973.1 |Severe acute respiratory syndrome coro    ....................  ....................  ....................  ....................  ....................  ....................
6447 MT607974.1 |Severe acute respiratory syndrome coro    ....................  ....................  ....................  ....................  ....................  ....................
6448 MT607975.1 |Severe acute respiratory syndrome coro    ....................  ....................  ....................  ....................  ....................  ....................
6449 MT607976.1 |Severe acute respiratory syndrome coro    ....................  ....................  ....................  ....................  ....................  ....................
6450 MT676412.1 |Severe acute respiratory syndrome coro    ....................  ....................  ....................  ....................  ....................  ....................
6451 MT676413.1 |Severe acute respiratory syndrome coro    ....................  ....................  ....................  ....................  ....................  ....................
6452 MT676414.1 |Severe acute respiratory syndrome coro    ....................  ....................  ....................  ....................  ....................  ....................
6453 MT676415.1 |Severe acute respiratory syndrome coro    ....................  ....................  ....................  ....................  ....................  ...............A....
6454 MT676416.1 |Severe acute respiratory syndrome coro    ....................  ....................  ....................  ....................  ....................  ....................
6455 MT676417.1 |Severe acute respiratory syndrome coro    ....................  ....................  ....................  ....................  ....................  ....................
6456 MT676418.1 |Severe acute respiratory syndrome coro    ....................  ....................  ....................  ....................  ....................  ....................
6457 MT676419.1 |Severe acute respiratory syndrome coro    ....................  ....................  ....................  ....................  ....................  ....................
6458 MT676420.1 |Severe acute respiratory syndrome coro    ....................  ....................  ....................  ....................  ....................  ....................
6459 MT676421.1 |Severe acute respiratory syndrome coro    ....................  ....................  ....................  ....................  ....................  ....................
6460 LR897977.1 |Severe acute respiratory syndrome coro    ......-.............  ....................  ....................  ....................  ....................  .......-............
6461 LR897981.1 |Severe acute respiratory syndrome coro    ......-.............  ....................  ....................  ....................  ....................  .......-............
6462 LR897983.1 |Severe acute respiratory syndrome coro    ......-.............  ....................  ....................  ....................  ....................  .......-............
6463 LR897985.1 |Severe acute respiratory syndrome coro    ......-.............  ....................  ....................  ....................  ....................  .......-............
6464 LR897986.1 |Severe acute respiratory syndrome coro    ......-.............  ....................  ....................  ....................  ....................  .......-............
6465 LR897987.1 |Severe acute respiratory syndrome coro    ......-.............  ....................  ....................  ....................  ....................  .......-............
6466 LR897989.1 |Severe acute respiratory syndrome coro    ......-.............  ....................  ....................  ....................  ....................  .......-............
6467 LR897990.1 |Severe acute respiratory syndrome coro    ......-.............  ....................  ....................  ....................  ....................  .......-............
6468 LR897991.1 |Severe acute respiratory syndrome coro    ......-.............  ....................  ....................  ....................  ....................  .......-............
6469 LR897992.1 |Severe acute respiratory syndrome coro    ......-.............  ....................  ....................  ....................  ....................  .......-............
6470 LR897993.1 |Severe acute respiratory syndrome coro    ......-.............  ....................  ....................  ....................  ....................  .......-............
6471 LR897994.1 |Severe acute respiratory syndrome coro    ......-.............  ....................  ....................  ....................  ....................  .......-............
6472 LR897995.1 |Severe acute respiratory syndrome coro    ......-.............  ....................  ....................  ....................  ....................  .......-............
6473 LR897996.1 |Severe acute respiratory syndrome coro    ......-.............  ....................  ....................  ....................  ....................  .......-............
6474 LR897997.1 |Severe acute respiratory syndrome coro    ......-.............  ....................  ....................  ....................  ....................  .......-............
6475 LR898015.1 |Severe acute respiratory syndrome coro    ......-.............  ....................  ....................  ....................  ....................  .......-............
6476 LR898016.1 |Severe acute respiratory syndrome coro    ......-.............  ....................  ....................  ....................  ....................  .......-............
6477 LR898017.1 |Severe acute respiratory syndrome coro    ......-.............  ....................  ....................  ....................  ....................  .......-............
6478 LR898018.1 |Severe acute respiratory syndrome coro    ......-.............  ....................  ....................  ....................  ....................  .......-............
6479 LR898019.1 |Severe acute respiratory syndrome coro    ......-.............  ....................  ....................  ....................  ....................  .......-............
6480 LR898020.1 |Severe acute respiratory syndrome coro    ......-.............  ....................  ....................  ....................  ....................  .......-............
6481 LR898021.1 |Severe acute respiratory syndrome coro    ......-.............  ....................  ....................  ....................  ....................  .......-............
6482 LR898022.1 |Severe acute respiratory syndrome coro    ......-.............  ....................  ....................  ....................  ....................  .......-............
6483 LR898023.1 |Severe acute respiratory syndrome coro    ......-.............  ....................  ....................  ....................  ....................  .......-............
6484 LR898037.1 |Severe acute respiratory syndrome coro    ......-.............  ....................  ....................  ....................  ....................  .......-............
6485 LR898038.1 |Severe acute respiratory syndrome coro    ......-.............  ....................  ....................  ....................  ....................  .......-............
6486 LR898039.1 |Severe acute respiratory syndrome coro    ......-.............  ....................  ....................  ....................  ....................  .......-............
6487 LR898040.1 |Severe acute respiratory syndrome coro    ......-.............  ....................  ....................  ....................  ....................  .......-............
6488 LR898041.1 |Severe acute respiratory syndrome coro    ......-.............  ....................  ....................  ....................  ....................  .......-............
6489 LR898042.1 |Severe acute respiratory syndrome coro    ......-.............  ....................  ....................  ....................  ....................  .......-............
6490 LR898043.1 |Severe acute respiratory syndrome coro    ......-.............  ....................  ....................  ....................  ....................  .......-............
6491 LR898044.1 |Severe acute respiratory syndrome coro    ......-.............  ....................  ....................  ....................  ....................  .......-............
6492 LR898045.1 |Severe acute respiratory syndrome coro    ......-.............  ....................  ....................  ....................  ....................  .......-............
6493 LR898046.1 |Severe acute respiratory syndrome coro    ......-.............  ....................  ....................  ....................  ....................  .......-............
6494 LR898047.1 |Severe acute respiratory syndrome coro    ......-.............  ....................  ....................  ....................  ....................  .......-............
6495 MW190183.1 |Severe acute respiratory syndrome coro    ....................  ....................  ....................  ....................  ....................  ....................
6496 MW190194.1 |Severe acute respiratory syndrome coro    ....................  ....................  ....................  ....................  ....................  ....................
6497 MW190195.1 |Severe acute respiratory syndrome coro    ....................  ....................  ....................  ....................  ....................  ....................
6498 MW190196.1 |Severe acute respiratory syndrome coro    ....................  ....................  ....................  ....................  ....................  ....................
6499 MW190197.1 |Severe acute respiratory syndrome coro    ....................  ....................  ....................  ....................  ....................  ....................
6500 MW190198.1 |Severe acute respiratory syndrome coro    ....................  ....................  ....................  ....................  ....................  ....................
6501 MW190199.1 |Severe acute respiratory syndrome coro    ....................  ....................  ....................  ....................  ....................  ....................
6502 MW190200.1 |Severe acute respiratory syndrome coro    ....................  ....................  ....................  ....................  ....................  ....................
6503 MW190201.1 |Severe acute respiratory syndrome coro    ....................  ....................  ....................  ....................  ....................  ....................
6504 MW190202.1 |Severe acute respiratory syndrome coro    ....................  ....................  ....................  ....................  ....................  ....................
6505 MW190203.1 |Severe acute respiratory syndrome coro    ....................  ....................  ....................  ....................  ....................  ....................
6506 MW190204.1 |Severe acute respiratory syndrome coro    ....................  ....................  ....................  ....................  ....................  ....................
6507 MW190205.1 |Severe acute respiratory syndrome coro    ....................  ....................  ....................  ....................  ....................  ....................
6508 MW190206.1 |Severe acute respiratory syndrome coro    ....................  ....................  ....................  ....................  ....................  ....................
6509 MW190207.1 |Severe acute respiratory syndrome coro    ....................  ....................  ....................  ....................  ....................  ....................
6510 MW190208.1 |Severe acute respiratory syndrome coro    ....................  ....................  ....................  ....................  ....................  ....................
6511 MW190209.1 |Severe acute respiratory syndrome coro    ....................  ....................  ....................  ....................  ....................  ....................
6512 MW190210.1 |Severe acute respiratory syndrome coro    ....................  ....................  ....................  ....................  ....................  ....................
6513 MW190211.1 |Severe acute respiratory syndrome coro    ....................  ....................  ....................  ....................  ....................  ....................
6514 MW190212.1 |Severe acute respiratory syndrome coro    ....................  ....................  ....................  ....................  ....................  ....................
```

```
6515 MW190213.1 |Severe acute respiratory syndrome coro    ....................  ....................  ....................  ....................  ....................  ....................
6516 MW190214.1 |Severe acute respiratory syndrome coro    ....................  ....................  ....................  ....................  ....................  ....................
6517 MW190215.1 |Severe acute respiratory syndrome coro    ....................  ....................  ....................  ....................  ....................  ....................
6518 MW190216.1 |Severe acute respiratory syndrome coro    ....................  ....................  ....................  ....................  ....................  ....................
6519 MW190217.1 |Severe acute respiratory syndrome coro    ....................  ....................  ....................  ....................  ....................  ....................
6520 MW190218.1 |Severe acute respiratory syndrome coro    ....................  ....................  ....................  ....................  ....................  ....................
6521 MW190219.1 |Severe acute respiratory syndrome coro    ....................  ....................  ....................  ....................  ....................  ....................
6522 MW190220.1 |Severe acute respiratory syndrome coro    ....................  ....................  ....................  ....................  ....................  ....................
6523 MW190221.1 |Severe acute respiratory syndrome coro    ....................  ....................  ....................  ....................  ....................  ....................
6524 MW190223.1 |Severe acute respiratory syndrome coro    ....................  ....................  ....................  ....................  ....................  ....................
6525 MW190224.1 |Severe acute respiratory syndrome coro    ....................  ....................  ....................  ....................  ....................  ....................
6526 MW190225.1 |Severe acute respiratory syndrome coro    ....................  ....................  ....................  ....................  ....................  ....................
6527 MW190226.1 |Severe acute respiratory syndrome coro    ....................  ....................  ....................  ....................  ....................  ....................
6528 MW190227.1 |Severe acute respiratory syndrome coro    ....................  ....................  ....................  ....................  ....................  ....................
6529 MW190228.1 |Severe acute respiratory syndrome coro    ....................  ....A...............  ....................  ....................  ....................  ....................
6530 MW190229.1 |Severe acute respiratory syndrome coro    ....................  ....................  ....................  ....................  ....................  ....................
6531 MW190230.1 |Severe acute respiratory syndrome coro    ....................  ....................  ....................  ....................  ....................  ....................
6532 MW190231.1 |Severe acute respiratory syndrome coro    ....................  ....................  ....................  ....................  ....................  ....................
6533 MW190232.1 |Severe acute respiratory syndrome coro    ....................  ....................  ....................  ....................  ....................  ....................
6534 MW190233.1 |Severe acute respiratory syndrome coro    ....................  ....................  ....................  ....................  ....................  ....................
6535 MW190234.1 |Severe acute respiratory syndrome coro    ....................  ....................  ....................  ....................  ....................  ....................
6536 MW190235.1 |Severe acute respiratory syndrome coro    ....................  ....................  ....................  ....................  ....................  ....................
6537 MW190236.1 |Severe acute respiratory syndrome coro    ....................  ....................  ....................  ....................  ....................  ....................
6538 MW190237.1 |Severe acute respiratory syndrome coro    ....................  ....................  ....................  ....................  ....................  ....................
6539 MW190238.1 |Severe acute respiratory syndrome coro    ....................  ....................  ....................  ....................  ....................  ....................
6540 MW190239.1 |Severe acute respiratory syndrome coro    ....................  ....................  ....................  ....................  ....................  ....................
6541 MW190240.1 |Severe acute respiratory syndrome coro    ....................  ....................  ....................  ....................  ....................  ....................
6542 MW190241.1 |Severe acute respiratory syndrome coro    ....................  ....................  ....................  ....................  ....................  ....................
6543 MW190242.1 |Severe acute respiratory syndrome coro    ....................  ....................  ....................  ....................  ....................  ....................
6544 MW190243.1 |Severe acute respiratory syndrome coro    ....................  ....................  ....................  ....................  ....................  ....................
6545 MW190244.1 |Severe acute respiratory syndrome coro    ....................  ....................  ....................  ....................  ....................  ....................
6546 MW190245.1 |Severe acute respiratory syndrome coro    ....................  ....................  ....................  ....................  ....................  ....................
6547 MW190246.1 |Severe acute respiratory syndrome coro    ....................  ....................  ....................  ....................  ....................  ....................
6548 MW190247.1 |Severe acute respiratory syndrome coro    ....................  ....................  ....................  ....................  ....................  ....................
6549 MW190248.1 |Severe acute respiratory syndrome coro    ....................  ....................  ....................  ....................  ....................  ....................
6550 MW190249.1 |Severe acute respiratory syndrome coro    ....................  ....................  ....................  ....................  ....................  ....................
6551 MW190250.1 |Severe acute respiratory syndrome coro    ....................  ....................  ....................  ....................  ....................  ....................
6552 MW190251.1 |Severe acute respiratory syndrome coro    ....................  ....................  ....................  ....................  ....................  ....................
6553 MW190252.1 |Severe acute respiratory syndrome coro    ....................  ....................  ....................  ....................  ....................  ....................
6554 MW190253.1 |Severe acute respiratory syndrome coro    ....................  ....................  ....................  ....................  ....................  ....................
6555 MW190254.1 |Severe acute respiratory syndrome coro    ....................  ....................  ....................  ....................  ....................  ....................
6556 MW190255.1 |Severe acute respiratory syndrome coro    ....................  ....................  ....................  ....................  ....................  ....................
6557 MW190256.1 |Severe acute respiratory syndrome coro    ...T................  ....................  ....................  ....................  ....................  ....................
6558 MW190257.1 |Severe acute respiratory syndrome coro    ....................  ....................  ....................  ....................  ....................  ....................
6559 MW190258.1 |Severe acute respiratory syndrome coro    ....................  ....................  ....................  ....................  ....................  ....................
6560 MW190259.1 |Severe acute respiratory syndrome coro    ....................  ....................  ....................  ....................  ....................  ....................
6561 MW190260.1 |Severe acute respiratory syndrome coro    ....................  ....................  ....................  ....................  ....................  ....................
6562 MW190261.1 |Severe acute respiratory syndrome coro    ....................  ....................  ....................  ....................  ....................  ....................
6563 MW190262.1 |Severe acute respiratory syndrome coro    ....................  ....................  ....................  ....................  ....................  ....................
6564 MW190263.1 |Severe acute respiratory syndrome coro    ....................  ....................  ....................  ....................  ....................  ....................
6565 MW190264.1 |Severe acute respiratory syndrome coro    ....................  ....................  ....................  ....................  ....................  ....................
6566 MW190265.1 |Severe acute respiratory syndrome coro    ....................  ....................  ....................  ....................  ....................  ....................
6567 MW190266.1 |Severe acute respiratory syndrome coro    ....................  ....................  ....................  ....................  ....................  ....................
6568 MW190267.1 |Severe acute respiratory syndrome coro    ....................  ....................  ....................  ....................  ....................  ....................
6569 MW190268.1 |Severe acute respiratory syndrome coro    ....................  ....................  ....................  ....................  ....................  ....................
6570 MW190269.1 |Severe acute respiratory syndrome coro    ....................  ....................  ....................  ....................  ....................  ....................
6571 MW190270.1 |Severe acute respiratory syndrome coro    ....................  ....................  ....................  ....................  ....................  ....................
6572 MW190271.1 |Severe acute respiratory syndrome coro    ....................  ....................  ....................  ....................  ....................  ....................
6573 MW190272.1 |Severe acute respiratory syndrome coro    ....................  ....................  ....................  ....................  ....................  ....................
6574 MW190273.1 |Severe acute respiratory syndrome coro    ....................  ....................  ....................  ....................  ....................  ....................
6575 MW190274.1 |Severe acute respiratory syndrome coro    ....................  ....................  ....................  ....................  ....................  ....................
6576 MW190275.1 |Severe acute respiratory syndrome coro    ....................  ....................  ....................  ....................  ....................  ....................
6577 MW190276.1 |Severe acute respiratory syndrome coro    ....................  ....................  ....................  ....................  ....................  ....................
6578 MW190277.1 |Severe acute respiratory syndrome coro    ....................  ....................  ....................  ....................  ....................  ....................
6579 MW190278.1 |Severe acute respiratory syndrome coro    ....................  ....................  ....................  ....................  ....................  ....................
6580 MW190279.1 |Severe acute respiratory syndrome coro    ....................  ....................  ....................  ....................  ....................  ....................
6581 MW190280.1 |Severe acute respiratory syndrome coro    ....................  ....................  ....................  ....................  ....................  ....................
6582 MW190281.1 |Severe acute respiratory syndrome coro    ....................  ....................  ....................  ....................  ....................  ....................
6583 MW190282.1 |Severe acute respiratory syndrome coro    ....................  ....................  ....................  ....................  ....................  ....................
6584 MW190283.1 |Severe acute respiratory syndrome coro    ....................  ....................  ....................  ....................  ....................  ....................
6585 MW190284.1 |Severe acute respiratory syndrome coro    ....................  ....................  ....................  ....................  ....................  ....................
6586 MW190285.1 |Severe acute respiratory syndrome coro    ....................  ....................  ....................  ....................  ....................  ....................
6587 MW190286.1 |Severe acute respiratory syndrome coro    ....................  ....................  ....................  ....................  ....................  ....................
6588 MW190287.1 |Severe acute respiratory syndrome coro    ....................  ....................  ....................  ....................  ....................  ....................
6589 MW190288.1 |Severe acute respiratory syndrome coro    ....................  ....................  ....................  ....................  ....................  ....................
6590 MW190289.1 |Severe acute respiratory syndrome coro    ....................  ....................  ....................  ....................  ....................  ....................
6591 MW190290.1 |Severe acute respiratory syndrome coro    ....................  ....................  ....................  ....................  ....................  ....................
6592 MW190291.1 |Severe acute respiratory syndrome coro    ....................  ....................  ....................  ....................  ....................  ....................
```

6593 MW190292.1 |Severe acute respiratory syndrome coro ....
6594 MW190293.1 |Severe acute respiratory syndrome coro ....
6595 MW190294.1 |Severe acute respiratory syndrome coro ....
6596 MW190295.1 |Severe acute respiratory syndrome coro ....
6597 MW190296.1 |Severe acute respiratory syndrome coro ....
6598 MW190297.1 |Severe acute respiratory syndrome coro ....
6599 MW190298.1 |Severe acute respiratory syndrome coro ....
6600 MW190299.1 |Severe acute respiratory syndrome coro ....
6601 MW190300.1 |Severe acute respiratory syndrome coro ....
6602 MW190301.1 |Severe acute respiratory syndrome coro ....
6603 MW190302.1 |Severe acute respiratory syndrome coro ....
6604 MW190303.1 |Severe acute respiratory syndrome coro ....
6605 MW190304.1 |Severe acute respiratory syndrome coro ....
6606 MW190305.1 |Severe acute respiratory syndrome coro ....
6607 MW190306.1 |Severe acute respiratory syndrome coro ....
6608 MW190307.1 |Severe acute respiratory syndrome coro ....
6609 MW190308.1 |Severe acute respiratory syndrome coro ....
6610 MW190309.1 |Severe acute respiratory syndrome coro ....
6611 MW190310.1 |Severe acute respiratory syndrome coro ....
6612 MW190311.1 |Severe acute respiratory syndrome coro ....
6613 MW190312.1 |Severe acute respiratory syndrome coro ....
6614 MW190313.1 |Severe acute respiratory syndrome coro ....
6615 MW190314.1 |Severe acute respiratory syndrome coro ....
6616 MW190315.1 |Severe acute respiratory syndrome coro ....
6617 MW190316.1 |Severe acute respiratory syndrome coro ....
6618 MW190317.1 |Severe acute respiratory syndrome coro ....
6619 MW190318.1 |Severe acute respiratory syndrome coro ....
6620 MW190319.1 |Severe acute respiratory syndrome coro ....
6621 MW190320.1 |Severe acute respiratory syndrome coro ....
6622 MW190321.1 |Severe acute respiratory syndrome coro ....
6623 MW190322.1 |Severe acute respiratory syndrome coro ....
6624 MW190323.1 |Severe acute respiratory syndrome coro ....
6625 MW190324.1 |Severe acute respiratory syndrome coro ....
6626 MW190325.1 |Severe acute respiratory syndrome coro ....
6627 MW190326.1 |Severe acute respiratory syndrome coro ....
6628 MW190327.1 |Severe acute respiratory syndrome coro ....
6629 MW190328.1 |Severe acute respiratory syndrome coro ....
6630 MW190329.1 |Severe acute respiratory syndrome coro ....
6631 MW190330.1 |Severe acute respiratory syndrome coro ....
6632 MW190331.1 |Severe acute respiratory syndrome coro ....
6633 MW190332.1 |Severe acute respiratory syndrome coro ....
6634 MW190333.1 |Severe acute respiratory syndrome coro ....
6635 MW190334.1 |Severe acute respiratory syndrome coro ....
6636 MW190335.1 |Severe acute respiratory syndrome coro ....
6637 MW190336.1 |Severe acute respiratory syndrome coro ....
6638 MW190337.1 |Severe acute respiratory syndrome coro ....
6639 MW190338.1 |Severe acute respiratory syndrome coro ....
6640 MW190339.1 |Severe acute respiratory syndrome coro ....
6641 MW190340.1 |Severe acute respiratory syndrome coro ....
6642 MW190341.1 |Severe acute respiratory syndrome coro ....
6643 MW190342.1 |Severe acute respiratory syndrome coro ....
6644 MW190343.1 |Severe acute respiratory syndrome coro ....
6645 MW190344.1 |Severe acute respiratory syndrome coro ....
6646 MW190345.1 |Severe acute respiratory syndrome coro ....
6647 MW190346.1 |Severe acute respiratory syndrome coro ....
6648 MW190347.1 |Severe acute respiratory syndrome coro ....
6649 MW190348.1 |Severe acute respiratory syndrome coro ....
6650 MW190349.1 |Severe acute respiratory syndrome coro ....
6651 MW190350.1 |Severe acute respiratory syndrome coro ....
6652 MW190351.1 |Severe acute respiratory syndrome coro ....
6653 MW190352.1 |Severe acute respiratory syndrome coro ....
6654 MW190353.1 |Severe acute respiratory syndrome coro ....
6655 MW190354.1 |Severe acute respiratory syndrome coro ....
6656 MW190355.1 |Severe acute respiratory syndrome coro ....
6657 MW190356.1 |Severe acute respiratory syndrome coro ....
6658 MW190357.1 |Severe acute respiratory syndrome coro ....
6659 MW190358.1 |Severe acute respiratory syndrome coro ....
6660 MW190359.1 |Severe acute respiratory syndrome coro ....
6661 MW190360.1 |Severe acute respiratory syndrome coro ....
6662 MW190361.1 |Severe acute respiratory syndrome coro ....
6663 MW190362.1 |Severe acute respiratory syndrome coro ....
6664 MW190363.1 |Severe acute respiratory syndrome coro ....
6665 MW190364.1 |Severe acute respiratory syndrome coro ....
6666 MW190365.1 |Severe acute respiratory syndrome coro ....
6667 MW190366.1 |Severe acute respiratory syndrome coro ....
6668 MW190367.1 |Severe acute respiratory syndrome coro ....
6669 MW190368.1 |Severe acute respiratory syndrome coro ....
6670 MW190369.1 |Severe acute respiratory syndrome coro ....

```
6671 MW190370.1 |Severe acute respiratory syndrome coro    ....................  ....................  .....................  ....................  ....................  ....................
6672 MW190372.1 |Severe acute respiratory syndrome coro    ....................  ....................  .....................  ....................  ....................  ....................
6673 MW190373.1 |Severe acute respiratory syndrome coro    ....................  ....................  .....................  ....................  ....................  ....................
6674 MW190374.1 |Severe acute respiratory syndrome coro    ....................  ....................  .....................  ....................  ....................  ....................
6675 MW190375.1 |Severe acute respiratory syndrome coro    ....................  ....................  .....................  ....................  ....................  ....................
6676 MW190376.1 |Severe acute respiratory syndrome coro    ....................  ....................  .....................  ....................  ....................  ....................
6677 MW190377.1 |Severe acute respiratory syndrome coro    ....................  ....................  .....................  ....................  ....................  ....................
6678 MW190378.1 |Severe acute respiratory syndrome coro    ....................  ....................  .....................  ....................  ....................  ....................
6679 MW190379.1 |Severe acute respiratory syndrome coro    ....................  ....................  .....................  ....................  ....................  ....................
6680 MW190380.1 |Severe acute respiratory syndrome coro    ....................  ....................  .....................  ....................  ....................  ....................
6681 MW190381.1 |Severe acute respiratory syndrome coro    ....................  ....................  .....................  ....................  ....................  ....................
6682 MW190382.1 |Severe acute respiratory syndrome coro    ....................  ....................  .....................  ....................  ....................  ....................
6683 MW190383.1 |Severe acute respiratory syndrome coro    ....................  ....................  .....................  ....................  ....................  ....................
6684 MW190384.1 |Severe acute respiratory syndrome coro    ....................  ....................  .....................  ....................  ....................  ....................
6685 MW190385.1 |Severe acute respiratory syndrome coro    ....................  ....................  .....................  ....................  ....................  ....................
6686 MW190386.1 |Severe acute respiratory syndrome coro    ....................  ....................  .....................  ....................  ....................  ....................
6687 MW190387.1 |Severe acute respiratory syndrome coro    ....................  ....................  .....................  ....................  ....................  ....................
6688 MW190388.1 |Severe acute respiratory syndrome coro    ....................  ....................  .....................  ....................  ....................  ....................
6689 MW190389.1 |Severe acute respiratory syndrome coro    ....................  ....................  .....................  ....................  ....................  ....................
6690 MW190390.1 |Severe acute respiratory syndrome coro    ....................  ....................  .....................  ....................  ....................  ....................
6691 MW190391.1 |Severe acute respiratory syndrome coro    ....................  ....................  .....................  ....................  ....................  ....................
6692 MW190392.1 |Severe acute respiratory syndrome coro    ....................  ....................  .....................  ....................  ....................  ....................
6693 MW190393.1 |Severe acute respiratory syndrome coro    ....................  ....................  .....................  ....................  ....................  ....................
6694 MW190394.1 |Severe acute respiratory syndrome coro    ....................  ....................  .....................  ....................  ....................  ....................
6695 MW190395.1 |Severe acute respiratory syndrome coro    ....................  ....................  .....................  ....................  ....................  ....................
6696 MW190396.1 |Severe acute respiratory syndrome coro    ....................  ....................  .....................  ....................  ....................  ....................
6697 MW190397.1 |Severe acute respiratory syndrome coro    ....................  ....................  .....................  ....................  ....................  ....................
6698 MW190398.1 |Severe acute respiratory syndrome coro    ....................  ....................  .....................  ....................  ....................  ....................
6699 MW190399.1 |Severe acute respiratory syndrome coro    ....................  ....................  .....................  ....................  ....................  ....................
6700 MW190400.1 |Severe acute respiratory syndrome coro    ....................  ....................  .....................  ....................  ....................  ....................
6701 MW190401.1 |Severe acute respiratory syndrome coro    ....................  ....................  .....................  ....................  ....................  ....................
6702 MW190402.1 |Severe acute respiratory syndrome coro    ....................  ....................  .....................  ....................  ....................  ....................
6703 MW190403.1 |Severe acute respiratory syndrome coro    ....................  ....................  .....................  ....................  ....................  ....................
6704 MW190404.1 |Severe acute respiratory syndrome coro    ....................  ....................  .....................  ....................  ....................  ....................
6705 MW190405.1 |Severe acute respiratory syndrome coro    ....................  ....................  .....................  ....................  ....................  ....................
6706 MW190406.1 |Severe acute respiratory syndrome coro    ....................  ....................  .....................  ....................  ....................  ....................
6707 MW190407.1 |Severe acute respiratory syndrome coro    ....................  ....................  .....................  ....................  ....................  ....................
6708 MW190408.1 |Severe acute respiratory syndrome coro    ....................  ....................  .....................  ....................  ....................  ....................
6709 MW190409.1 |Severe acute respiratory syndrome coro    ....................  ....................  .....................  ....................  ....................  ....................
6710 MW190410.1 |Severe acute respiratory syndrome coro    ....................  ....................  .....................  ....................  ....................  ....................
6711 MW190411.1 |Severe acute respiratory syndrome coro    ....................  ....................  .....................  ....................  ....................  ....................
6712 MW190412.1 |Severe acute respiratory syndrome coro    ....................  ....................  .....................  ....................  ....................  ....................
6713 MW190413.1 |Severe acute respiratory syndrome coro    ....................  ....................  .....................  ....................  ....................  ....................
6714 MW190414.1 |Severe acute respiratory syndrome coro    ....................  ....................  .....................  ....................  ....................  ....................
6715 MW190415.1 |Severe acute respiratory syndrome coro    ....................  ....................  .....................  ....................  ....................  ....................
6716 MW190416.1 |Severe acute respiratory syndrome coro    ....................  ....................  .....................  ....................  ....................  ....................
6717 MW190417.1 |Severe acute respiratory syndrome coro    ....................  ....................  .....................  ....................  ....................  ....................
6718 MW190418.1 |Severe acute respiratory syndrome coro    ....................  ....................  .....................  ....................  ....................  ....................
6719 MW190419.1 |Severe acute respiratory syndrome coro    ....................  ....................  .....................  ....................  ....................  ....................
6720 MW190420.1 |Severe acute respiratory syndrome coro    ....................  ....................  .....................  ....................  ....................  ....................
6721 MW190421.1 |Severe acute respiratory syndrome coro    ....................  ....................  .....................  ....................  ....................  ....................
6722 MW190422.1 |Severe acute respiratory syndrome coro    ....................  ....................  .....................  ....................  ....................  ....................
6723 MW190423.1 |Severe acute respiratory syndrome coro    ....................  ....................  .....................  ....................  ....................  ....................
6724 MW190424.1 |Severe acute respiratory syndrome coro    ....................  ....................  .....................  ....................  ....................  ....................
6725 MW190425.1 |Severe acute respiratory syndrome coro    ....................  ....................  .....................  ....................  ....................  ....................
6726 MW190426.1 |Severe acute respiratory syndrome coro    ....................  ....................  .....................  ....................  ....................  ....................
6727 MW190427.1 |Severe acute respiratory syndrome coro    ....................  ....................  .....................  ....................  ....................  ....................
6728 MW190428.1 |Severe acute respiratory syndrome coro    ....................  ....................  .....................  ....................  ....................  ....................
6729 MW190429.1 |Severe acute respiratory syndrome coro    ....................  ....................  .....................  ....................  ....................  ....................
6730 MW190430.1 |Severe acute respiratory syndrome coro    ....................  ....................  .....................  ....................  ....................  ....................
6731 MW190431.1 |Severe acute respiratory syndrome coro    ....................  ....................  .....................  ....................  ....................  ....................
6732 MW190432.1 |Severe acute respiratory syndrome coro    ....................  ....................  .....................  ....................  ....................  ....................
6733 MW190433.1 |Severe acute respiratory syndrome coro    ....................  ....................  .....................  ....................  ....................  ....................
6734 MW190434.1 |Severe acute respiratory syndrome coro    ....................  ....................  .....................  ....................  ....................  ....................
6735 MW190435.1 |Severe acute respiratory syndrome coro    ....................  ....................  .....................  ....................  ....................  ....................
6736 MW190436.1 |Severe acute respiratory syndrome coro    ....................  ....................  .....................  ....................  ....................  ....................
6737 MW190437.1 |Severe acute respiratory syndrome coro    ....................  ....................  .....................  ....................  ....................  ....................
6738 MW190438.1 |Severe acute respiratory syndrome coro    ....................  ....................  .....................  ....................  ....................  ....................
6739 MW190439.1 |Severe acute respiratory syndrome coro    ....................  ....................  .....................  ....................  ....................  ....................
6740 MW190440.1 |Severe acute respiratory syndrome coro    ....................  ....................  .....................  ....................  ....................  ....................
6741 MW190441.1 |Severe acute respiratory syndrome coro    ....................  ....................  .....................  ....................  ....................  ....................
6742 MW190442.1 |Severe acute respiratory syndrome coro    ....................  ....................  .....................  ....................  ....................  ....................
6743 MW190443.1 |Severe acute respiratory syndrome coro    ....................  ....................  .....................  ....................  ....................  ....................
6744 MW190444.1 |Severe acute respiratory syndrome coro    ....................  ....................  .....................  ....................  ....................  ....................
6745 MW190445.1 |Severe acute respiratory syndrome coro    ....................  ....................  .....................  ....................  ....................  ....................
6746 MW190446.1 |Severe acute respiratory syndrome coro    ....................  ....................  .....................  ....................  ....................  ....................
6747 MW190447.1 |Severe acute respiratory syndrome coro    ....................  ....................  .....................  ....................  ....................  ....................
6748 MW190448.1 |Severe acute respiratory syndrome coro    ....................  ....................  .....................  ....................  ....................  ....................
```

6749 MW190449.1 |Severe acute respiratory syndrome coro ···················· ···················· ···················· ···················· ···················· ····················
6750 MW190450.1 |Severe acute respiratory syndrome coro ···················· ···················· ···················· ···················· ···················· ····················
6751 MW190451.1 |Severe acute respiratory syndrome coro ···················· ···················· ···················· ···················· ···················· ····················
6752 MW190452.1 |Severe acute respiratory syndrome coro ···················· ···················· ···················· ···················· ···················· ····················
6753 MW190453.1 |Severe acute respiratory syndrome coro ···················· ···················· ···················· ···················· ···················· ····················
6754 MW190454.1 |Severe acute respiratory syndrome coro ···················· ···················· ···················· ···················· ···················· ····················
6755 MW190455.1 |Severe acute respiratory syndrome coro ···················· ···················· ···················· ···················· ···················· ····················
6756 MW190456.1 |Severe acute respiratory syndrome coro ···················· ···················· ···················· ···················· ···················· ····················
6757 MW190457.1 |Severe acute respiratory syndrome coro ···················· ···················· ···················· ···················· ···················· ····················
6758 MW190458.1 |Severe acute respiratory syndrome coro ···················· ···················· ···················· ···················· ···················· ····················
6759 MW190459.1 |Severe acute respiratory syndrome coro ···················· ···················· ···················· ···················· ···················· ····················
6760 MW190460.1 |Severe acute respiratory syndrome coro ···················· ···················· ···················· ···················· ···················· ····················
6761 MW190461.1 |Severe acute respiratory syndrome coro ···················· ···················· ···················· ···················· ···················· ····················
6762 MW190462.1 |Severe acute respiratory syndrome coro ···················· ···················· ···················· ···················· ···················· ····················
6763 MW190463.1 |Severe acute respiratory syndrome coro ···················· ···················· ···················· ···················· ···················· ····················
6764 MW190464.1 |Severe acute respiratory syndrome coro ···················· ···················· ···················· ···················· ···················· ····················
6765 MW190465.1 |Severe acute respiratory syndrome coro ···················· ···················· ···················· ···················· ···················· ····················
6766 MW190466.1 |Severe acute respiratory syndrome coro ···················· ···················· ···················· ···················· ···················· ····················
6767 MW190467.1 |Severe acute respiratory syndrome coro ···················· ···················· ···················· ···················· ···················· ····················
6768 MW190468.1 |Severe acute respiratory syndrome coro ···················· ···················· ···················· ···················· ···················· ····················
6769 MW190469.1 |Severe acute respiratory syndrome coro ···················· ···················· ···················· ···················· ···················· ····················
6770 MW190470.1 |Severe acute respiratory syndrome coro ···················· ···················· ···················· ···················· ···················· ····················
6771 MW190471.1 |Severe acute respiratory syndrome coro ···················· ···················· ···················· ···················· ···················· ····················
6772 MW190472.1 |Severe acute respiratory syndrome coro ···················· ···················· ···················· ···················· ···················· ····················
6773 MW190473.1 |Severe acute respiratory syndrome coro ···················· ···················· ···················· ···················· ···················· ····················
6774 MW190474.1 |Severe acute respiratory syndrome coro ···················· ···················· ···················· ···················· ···················· ····················
6775 MW190475.1 |Severe acute respiratory syndrome coro ···················· ···················· ···················· ···················· ···················· ····················
6776 MW190476.1 |Severe acute respiratory syndrome coro ···················· ···················· ···················· ···················· ···················· ····················
6777 MW190477.1 |Severe acute respiratory syndrome coro ···················· ···················· ···················· ···················· ···················· ····················
6778 MW190478.1 |Severe acute respiratory syndrome coro ···················· ···················· ···················· ···················· ···················· ····················
6779 MW190479.1 |Severe acute respiratory syndrome coro ···················· ···················· ···················· ···················· ···················· ····················
6780 MW190480.1 |Severe acute respiratory syndrome coro ···················· ···················· ···················· ···················· ···················· ····················
6781 MW190481.1 |Severe acute respiratory syndrome coro ···················· ···················· ···················· ···················· ···················· ····················
6782 MW190482.1 |Severe acute respiratory syndrome coro ···················· ···················· ···················· ···················· ···················· ····················
6783 MW190483.1 |Severe acute respiratory syndrome coro ···················· ···················· ···················· ···················· ···················· ····················
6784 MW190484.1 |Severe acute respiratory syndrome coro ···················· ···················· ···················· ···················· ···················· ····················
6785 MW190485.1 |Severe acute respiratory syndrome coro ···················· ···················· ···················· ···················· ···················· ····················
6786 MW190486.1 |Severe acute respiratory syndrome coro ···················· ···················· ···················· ···················· ···················· ····················
6787 MW190487.1 |Severe acute respiratory syndrome coro ···················· ···················· ···················· ···················· ···················· ····················
6788 MW190488.1 |Severe acute respiratory syndrome coro ···················· ···················· ···················· ···················· ···················· ····················
6789 MW190489.1 |Severe acute respiratory syndrome coro ···················· ···················· ···················· ···················· ···················· ····················
6790 MW190490.1 |Severe acute respiratory syndrome coro ···················· ···················· ···················· ···················· ···················· ····················
6791 MW190491.1 |Severe acute respiratory syndrome coro ···················· ···················· ···················· ···················· ···················· ····················
6792 MW190492.1 |Severe acute respiratory syndrome coro ···················· ·········A·········· ···················· ···················· ···················· ····················
6793 MW190493.1 |Severe acute respiratory syndrome coro ···················· ···················· ···················· ···················· ···················· ····················
6794 MW190495.1 |Severe acute respiratory syndrome coro ···················· ···················· ···················· ···················· ···················· ····················
6795 MW190496.1 |Severe acute respiratory syndrome coro ···················· ···················· ···················· ···················· ···················· ····················
6796 MW190497.1 |Severe acute respiratory syndrome coro ···················· ···················· ···················· ···················· ···················· ····················
6797 MW190498.1 |Severe acute respiratory syndrome coro ···················· ···················· ···················· ···················· ···················· ····················
6798 MW190499.1 |Severe acute respiratory syndrome coro ···················· ···················· ···················· ···················· ···················· ····················
6799 MW190500.1 |Severe acute respiratory syndrome coro ···················· ···················· ···················· ···················· ···················· ····················
6800 MW190501.1 |Severe acute respiratory syndrome coro ···················· ···················· ···················· ···················· ···················· ····················
6801 MW190502.1 |Severe acute respiratory syndrome coro ···················· ···················· ···················· ···················· ···················· ····················
6802 MW190503.1 |Severe acute respiratory syndrome coro ···················· ···················· ···················· ···················· ···················· ····················
6803 MW190504.1 |Severe acute respiratory syndrome coro ···················· ···················· ···················· ···················· ···················· ····················
6804 MW190505.1 |Severe acute respiratory syndrome coro ···················· ···················· ···················· ···················· ···················· ····················
6805 MW190506.1 |Severe acute respiratory syndrome coro ···················· ···················· ···················· ···················· ···················· ····················
6806 MW190507.1 |Severe acute respiratory syndrome coro ···················· ···················· ···················· ···················· ···················· ····················
6807 MW190508.1 |Severe acute respiratory syndrome coro ···················· ···················· ···················· ···················· ···················· ····················
6808 MW190509.1 |Severe acute respiratory syndrome coro ···················· ···················· ···················· ···················· ···················· ····················
6809 MW190510.1 |Severe acute respiratory syndrome coro ···················· ······A············· ···················· ···················· ···················· ····················
6810 MW190511.1 |Severe acute respiratory syndrome coro ···················· ···················· ···················· ···················· ···················· ····················
6811 MW190512.1 |Severe acute respiratory syndrome coro ···················· ···················· ···················· ···················· ···················· ····················
6812 MW190513.1 |Severe acute respiratory syndrome coro ···················· ···················· ···················· ···················· ···················· ····················
6813 MW190514.1 |Severe acute respiratory syndrome coro ···················· ···················· ···················· ···················· ···················· ····················
6814 MW190516.1 |Severe acute respiratory syndrome coro ···················· ···················· ···················· ···················· ···················· ····················
6815 MW190517.1 |Severe acute respiratory syndrome coro ···················· ···················· ···················· ···················· ···················· ····················
6816 MW190518.1 |Severe acute respiratory syndrome coro ···················· ···················· ···················· ···················· ···················· ····················
6817 MW190519.1 |Severe acute respiratory syndrome coro ···················· ···················· ···················· ···················· ···················· ····················
6818 MW190520.1 |Severe acute respiratory syndrome coro ···················· ···················· ···················· ···················· ···················· ····················
6819 MW190521.1 |Severe acute respiratory syndrome coro ···················· ···················· ···················· ···················· ···················· ····················
6820 MW190522.1 |Severe acute respiratory syndrome coro ···················· ···················· ···················· ···················· ···················· ····················
6821 MW190523.1 |Severe acute respiratory syndrome coro ···················· ···················· ···················· ···················· ···················· ····················
6822 MW190524.1 |Severe acute respiratory syndrome coro ···················· ···················· ···················· ···················· ···················· ····················
6823 MW190525.1 |Severe acute respiratory syndrome coro ···················· ···················· ···················· ···················· ···················· ····················
6824 MW190526.1 |Severe acute respiratory syndrome coro ···················· ···················· ···················· ···················· ···················· ····················
6825 MW190527.1 |Severe acute respiratory syndrome coro ···················· ···················· ···················· ···················· ···················· ····················
6826 MW190528.1 |Severe acute respiratory syndrome coro ···················· ···················· ···················· ···················· ···················· ····················

6827 MW190529.1 |Severe acute respiratory syndrome coro ....
6828 MW190530.1 |Severe acute respiratory syndrome coro ....
6829 MW190531.1 |Severe acute respiratory syndrome coro ....
6830 MW190532.1 |Severe acute respiratory syndrome coro ....
6831 MW190533.1 |Severe acute respiratory syndrome coro ....
6832 MW190534.1 |Severe acute respiratory syndrome coro ....
6833 MW190535.1 |Severe acute respiratory syndrome coro ....
6834 MW190536.1 |Severe acute respiratory syndrome coro ....
6835 MW190537.1 |Severe acute respiratory syndrome coro ....
6836 MW190538.1 |Severe acute respiratory syndrome coro ....
6837 MW190539.1 |Severe acute respiratory syndrome coro ....
6838 MW190540.1 |Severe acute respiratory syndrome coro ....
6839 MW190541.1 |Severe acute respiratory syndrome coro ....
6840 MW190542.1 |Severe acute respiratory syndrome coro ....
6841 MW190543.1 |Severe acute respiratory syndrome coro ....
6842 MW190545.1 |Severe acute respiratory syndrome coro ....
6843 MW190546.1 |Severe acute respiratory syndrome coro ....
6844 MW190547.1 |Severe acute respiratory syndrome coro ....
6845 MW190548.1 |Severe acute respiratory syndrome coro ....
6846 MW190549.1 |Severe acute respiratory syndrome coro ....
6847 MW190550.1 |Severe acute respiratory syndrome coro ....
6848 MW190551.1 |Severe acute respiratory syndrome coro ....
6849 MW190552.1 |Severe acute respiratory syndrome coro ....
6850 MW190553.1 |Severe acute respiratory syndrome coro ....
6851 MW190554.1 |Severe acute respiratory syndrome coro ....
6852 MW190556.1 |Severe acute respiratory syndrome coro ....
6853 MW190557.1 |Severe acute respiratory syndrome coro ....
6854 MW190558.1 |Severe acute respiratory syndrome coro ....
6855 MW190559.1 |Severe acute respiratory syndrome coro ....
6856 MW190560.1 |Severe acute respiratory syndrome coro ....
6857 MW190561.1 |Severe acute respiratory syndrome coro ....
6858 MW190562.1 |Severe acute respiratory syndrome coro ....
6859 MW190563.1 |Severe acute respiratory syndrome coro ....
6860 MW190564.1 |Severe acute respiratory syndrome coro ....
6861 MW190565.1 |Severe acute respiratory syndrome coro ....
6862 MW190566.1 |Severe acute respiratory syndrome coro ....
6863 MW190567.1 |Severe acute respiratory syndrome coro ....
6864 MW190568.1 |Severe acute respiratory syndrome coro ....
6865 MW190569.1 |Severe acute respiratory syndrome coro ....
6866 MW190570.1 |Severe acute respiratory syndrome coro ....
6867 MW190571.1 |Severe acute respiratory syndrome coro ....
6868 MW190572.1 |Severe acute respiratory syndrome coro ....
6869 MW190573.1 |Severe acute respiratory syndrome coro ....
6870 MW190574.1 |Severe acute respiratory syndrome coro ....
6871 MW190575.1 |Severe acute respiratory syndrome coro ....
6872 MW190576.1 |Severe acute respiratory syndrome coro ....
6873 MW190577.1 |Severe acute respiratory syndrome coro ....
6874 MW190578.1 |Severe acute respiratory syndrome coro ....
6875 MW190579.1 |Severe acute respiratory syndrome coro ....
6876 MW190580.1 |Severe acute respiratory syndrome coro ....
6877 MW190581.1 |Severe acute respiratory syndrome coro ....
6878 MW190582.1 |Severe acute respiratory syndrome coro ....
6879 MW190583.1 |Severe acute respiratory syndrome coro ....
6880 MW190584.1 |Severe acute respiratory syndrome coro ....
6881 MW190585.1 |Severe acute respiratory syndrome coro ....
6882 MW190586.1 |Severe acute respiratory syndrome coro ....
6883 MW190587.1 |Severe acute respiratory syndrome coro ....
6884 MW190588.1 |Severe acute respiratory syndrome coro ....
6885 MW190589.1 |Severe acute respiratory syndrome coro ....
6886 MW190590.1 |Severe acute respiratory syndrome coro ....
6887 MW190591.1 |Severe acute respiratory syndrome coro ....
6888 MW190592.1 |Severe acute respiratory syndrome coro ....
6889 MW190593.1 |Severe acute respiratory syndrome coro ....
6890 MW190594.1 |Severe acute respiratory syndrome coro ....
6891 MW190595.1 |Severe acute respiratory syndrome coro ....
6892 MW190596.1 |Severe acute respiratory syndrome coro ....
6893 MW190597.1 |Severe acute respiratory syndrome coro ....
6894 MW190598.1 |Severe acute respiratory syndrome coro ....
6895 MW190599.1 |Severe acute respiratory syndrome coro ....
6896 MW190600.1 |Severe acute respiratory syndrome coro ....
6897 MW190601.1 |Severe acute respiratory syndrome coro ....
6898 MW190602.1 |Severe acute respiratory syndrome coro ....
6899 MW190603.1 |Severe acute respiratory syndrome coro ....
6900 MW190604.1 |Severe acute respiratory syndrome coro ....
6901 MW190605.1 |Severe acute respiratory syndrome coro ....
6902 MW190606.1 |Severe acute respiratory syndrome coro ....
6903 MW190607.1 |Severe acute respiratory syndrome coro ....
6904 MW190608.1 |Severe acute respiratory syndrome coro ....

6905 MW190609.1 |Severe acute respiratory syndrome coro ....
6906 MW190610.1 |Severe acute respiratory syndrome coro ....
6907 MW190611.1 |Severe acute respiratory syndrome coro ....
6908 MW190612.1 |Severe acute respiratory syndrome coro ....
6909 MW190613.1 |Severe acute respiratory syndrome coro ....
6910 MW190614.1 |Severe acute respiratory syndrome coro ....
6911 MW190615.1 |Severe acute respiratory syndrome coro ....
6912 MW190616.1 |Severe acute respiratory syndrome coro ....
6913 MW190617.1 |Severe acute respiratory syndrome coro ....
6914 MW190618.1 |Severe acute respiratory syndrome coro ....
6915 MW190619.1 |Severe acute respiratory syndrome coro ....
6916 MW190620.1 |Severe acute respiratory syndrome coro ....
6917 MW190621.1 |Severe acute respiratory syndrome coro ....
6918 MW190622.1 |Severe acute respiratory syndrome coro ....
6919 MW190623.1 |Severe acute respiratory syndrome coro ....
6920 MW190624.1 |Severe acute respiratory syndrome coro ....
6921 MW190625.1 |Severe acute respiratory syndrome coro ....
6922 MW190626.1 |Severe acute respiratory syndrome coro ....
6923 MW190627.1 |Severe acute respiratory syndrome coro ....
6924 MW190628.1 |Severe acute respiratory syndrome coro ....
6925 MW190629.1 |Severe acute respiratory syndrome coro ....
6926 MW190631.1 |Severe acute respiratory syndrome coro ....
6927 MW190632.1 |Severe acute respiratory syndrome coro ....
6928 MW190633.1 |Severe acute respiratory syndrome coro ....
6929 MW190634.1 |Severe acute respiratory syndrome coro ....
6930 MW190635.1 |Severe acute respiratory syndrome coro ....
6931 MW190636.1 |Severe acute respiratory syndrome coro ....
6932 MW190637.1 |Severe acute respiratory syndrome coro ....
6933 MW190638.1 |Severe acute respiratory syndrome coro ....
6934 MW190639.1 |Severe acute respiratory syndrome coro ....
6935 MW190640.1 |Severe acute respiratory syndrome coro ....
6936 MW190641.1 |Severe acute respiratory syndrome coro ....
6937 MW190642.1 |Severe acute respiratory syndrome coro ....
6938 MW190643.1 |Severe acute respiratory syndrome coro ....
6939 MW190644.1 |Severe acute respiratory syndrome coro ....
6940 MW190646.1 |Severe acute respiratory syndrome coro ....
6941 MW190647.1 |Severe acute respiratory syndrome coro ....
6942 MW190648.1 |Severe acute respiratory syndrome coro ....
6943 MW190649.1 |Severe acute respiratory syndrome coro ....
6944 MW190650.1 |Severe acute respiratory syndrome coro ....
6945 MW190651.1 |Severe acute respiratory syndrome coro ....
6946 MW190652.1 |Severe acute respiratory syndrome coro ....
6947 MW190653.1 |Severe acute respiratory syndrome coro ....
6948 MW190654.1 |Severe acute respiratory syndrome coro ....
6949 MW190655.1 |Severe acute respiratory syndrome coro ....
6950 MW190656.1 |Severe acute respiratory syndrome coro ....
6951 MW190658.1 |Severe acute respiratory syndrome coro ....
6952 MW190659.1 |Severe acute respiratory syndrome coro ....
6953 MW190660.1 |Severe acute respiratory syndrome coro ....
6954 MW190661.1 |Severe acute respiratory syndrome coro ....
6955 MW190662.1 |Severe acute respiratory syndrome coro ....
6956 MW190663.1 |Severe acute respiratory syndrome coro ....
6957 MW190665.1 |Severe acute respiratory syndrome coro ....
6958 MW190666.1 |Severe acute respiratory syndrome coro ....
6959 MW190667.1 |Severe acute respiratory syndrome coro ....
6960 MW190668.1 |Severe acute respiratory syndrome coro ....
6961 MW190669.1 |Severe acute respiratory syndrome coro ....
6962 MW190670.1 |Severe acute respiratory syndrome coro ....
6963 MW190671.1 |Severe acute respiratory syndrome coro ....
6964 MW190672.1 |Severe acute respiratory syndrome coro ....
6965 MW190673.1 |Severe acute respiratory syndrome coro ....
6966 MW190674.1 |Severe acute respiratory syndrome coro ....
6967 MW190675.1 |Severe acute respiratory syndrome coro ....
6968 MW190676.1 |Severe acute respiratory syndrome coro ....
6969 MW190677.1 |Severe acute respiratory syndrome coro ....
6970 MW190678.1 |Severe acute respiratory syndrome coro ....
6971 MW190679.1 |Severe acute respiratory syndrome coro ....
6972 MW190680.1 |Severe acute respiratory syndrome coro ....
6973 MW190681.1 |Severe acute respiratory syndrome coro ....
6974 MW190682.1 |Severe acute respiratory syndrome coro ....
6975 MW190684.1 |Severe acute respiratory syndrome coro ....
6976 MW190685.1 |Severe acute respiratory syndrome coro ....
6977 MW190686.1 |Severe acute respiratory syndrome coro ....
6978 MW190687.1 |Severe acute respiratory syndrome coro ....
6979 MW190688.1 |Severe acute respiratory syndrome coro ....
6980 MW190690.1 |Severe acute respiratory syndrome coro ....
6981 MW190691.1 |Severe acute respiratory syndrome coro ....
6982 MW190692.1 |Severe acute respiratory syndrome coro ....

```
6983 MW190693.1 |Severe acute respiratory syndrome coro   ....................  ....................  ....................  ....................  ....................  ....................
6984 MW190694.1 |Severe acute respiratory syndrome coro   ....................  ....................  ....................  ....................  ....................  ....................
6985 MW190695.1 |Severe acute respiratory syndrome coro   ....................  ....................  ....................  ....................  ....................  ....................
6986 MW190696.1 |Severe acute respiratory syndrome coro   ....................  ....................  ....................  ....................  ....................  ....................
6987 MW190697.1 |Severe acute respiratory syndrome coro   ....................  ....................  ....................  ....................  ....................  ....................
6988 MW190698.1 |Severe acute respiratory syndrome coro   ....................  ....................  ....................  ....................  ....................  ....................
6989 MW190699.1 |Severe acute respiratory syndrome coro   ....................  ....................  ....................  ....................  ....................  ....................
6990 MW190700.1 |Severe acute respiratory syndrome coro   ....................  ....................  ....................  ....................  ....................  ....................
6991 MW190701.1 |Severe acute respiratory syndrome coro   ....................  ....................  ....................  ....................  ....................  ....................
6992 MW190702.1 |Severe acute respiratory syndrome coro   ....................  ....................  ....................  ....................  ....................  ....................
6993 MW190703.1 |Severe acute respiratory syndrome coro   ....................  ....................  ....................  ....................  ....................  ....................
6994 MW190704.1 |Severe acute respiratory syndrome coro   ....................  ....................  ....................  ....................  ....................  ....................
6995 MW190705.1 |Severe acute respiratory syndrome coro   ....................  ....................  ....................  ....................  ....................  ....................
6996 MW190706.1 |Severe acute respiratory syndrome coro   ....................  ....................  ....................  ....................  ....................  ....................
6997 MW190707.1 |Severe acute respiratory syndrome coro   ....................  ....................  ....................  ....................  ....................  ....................
6998 MW190708.1 |Severe acute respiratory syndrome coro   ....................  ....................  ....................  ....................  ....................  ....................
6999 MW190709.1 |Severe acute respiratory syndrome coro   ....................  ....................  ....................  ....................  ....................  ....................
7000 MW190710.1 |Severe acute respiratory syndrome coro   ....................  ....................  ....................  ....................  ....................  ....................
7001 MW190711.1 |Severe acute respiratory syndrome coro   ....................  ....................  ....................  ....................  ....................  ....................
7002 MW190712.1 |Severe acute respiratory syndrome coro   ....................  ....................  ....................  ....................  ....................  ....................
7003 MW190713.1 |Severe acute respiratory syndrome coro   ....................  ....................  ....................  ....................  ....................  ....................
7004 MW190714.1 |Severe acute respiratory syndrome coro   ....................  ....................  ....................  ....................  ....................  ....................
7005 MW190715.1 |Severe acute respiratory syndrome coro   ....................  ....................  ....................  ....................  ....................  ....................
7006 MW190716.1 |Severe acute respiratory syndrome coro   ....................  ....................  ....................  ....................  ....................  ....................
7007 MW190717.1 |Severe acute respiratory syndrome coro   ....................  ....................  ....................  ....................  ....................  ....................
7008 MW190718.1 |Severe acute respiratory syndrome coro   ....................  ....................  ....................  ....................  ....................  ....................
7009 MW190719.1 |Severe acute respiratory syndrome coro   ....................  ....................  ....................  ....................  ....................  ....................
7010 MW190720.1 |Severe acute respiratory syndrome coro   ....................  ....................  ....................  ....................  ....................  ....................
7011 MW190721.1 |Severe acute respiratory syndrome coro   ....................  ....................  ....................  ....................  ....................  ....................
7012 MW190722.1 |Severe acute respiratory syndrome coro   ....................  ....................  ....................  ....................  ....................  ....................
7013 MW190723.1 |Severe acute respiratory syndrome coro   ....................  ....................  ....................  ....................  ....................  ....................
7014 MW190724.1 |Severe acute respiratory syndrome coro   ....................  ....................  ....................  ....................  ....................  ....................
7015 MW190725.1 |Severe acute respiratory syndrome coro   ....................  ....................  ....................  ....................  ....................  ....................
7016 MW190726.1 |Severe acute respiratory syndrome coro   ....................  ....................  ....................  ....................  ....................  ....................
7017 MW190727.1 |Severe acute respiratory syndrome coro   ....................  ....................  ....................  ....................  ....................  ....................
7018 MW190729.1 |Severe acute respiratory syndrome coro   ....................  ....................  ....................  ....................  ....................  ....................
7019 MW190730.1 |Severe acute respiratory syndrome coro   ....................  ....................  ....................  ....................  ....................  ....................
7020 MW190731.1 |Severe acute respiratory syndrome coro   ....................  ....................  ....................  ....................  ....................  ....................
7021 MW190732.1 |Severe acute respiratory syndrome coro   ....................  ....................  ....................  ....................  ....................  ....................
7022 MW190733.1 |Severe acute respiratory syndrome coro   ....................  ....................  ....................  ....................  ....................  ....................
7023 MW190734.1 |Severe acute respiratory syndrome coro   ....................  ....................  ....................  ....................  ....................  ....................
7024 MW190735.1 |Severe acute respiratory syndrome coro   ....................  ....................  ....................  ....................  ....................  ....................
7025 MW190736.1 |Severe acute respiratory syndrome coro   ....................  ....................  ....................  ....................  ....................  ....................
7026 MW190737.1 |Severe acute respiratory syndrome coro   ....................  ....................  ....................  ....................  ....................  ....................
7027 MW190738.1 |Severe acute respiratory syndrome coro   ....................  ....................  ....................  ....................  ....................  ....................
7028 MW190739.1 |Severe acute respiratory syndrome coro   ....................  ....................  ....................  ....................  ....................  ....................
7029 MW190740.1 |Severe acute respiratory syndrome coro   ....................  ....................  ....................  ....................  ....................  ....................
7030 MW190741.1 |Severe acute respiratory syndrome coro   ....................  ....................  ....................  ....................  ....................  ....................
7031 MW190742.1 |Severe acute respiratory syndrome coro   ....................  ....................  ....................  ....................  ....................  ....................
7032 MW190743.1 |Severe acute respiratory syndrome coro   ....................  ....................  ....................  ....................  ....................  ....................
7033 MW190744.1 |Severe acute respiratory syndrome coro   ....................  ....................  ....................  ....................  ....................  ....................
7034 MW190745.1 |Severe acute respiratory syndrome coro   ....................  ....................  ....................  ....................  ....................  ....................
7035 MW190746.1 |Severe acute respiratory syndrome coro   ....................  ....................  ....................  ....................  ....................  ....................
7036 MW190747.1 |Severe acute respiratory syndrome coro   ....................  ....................  ....................  ....................  ....................  ....................
7037 MW190748.1 |Severe acute respiratory syndrome coro   ....................  ....................  ....................  ....................  ....................  ....................
7038 MW190749.1 |Severe acute respiratory syndrome coro   ....................  ....................  ....................  ....................  ....................  ....................
7039 MW190750.1 |Severe acute respiratory syndrome coro   ....................  ....................  ....................  ....................  ....................  ....................
7040 MW190751.1 |Severe acute respiratory syndrome coro   ....................  ....................  ....................  ....................  ....................  ....................
7041 MW190752.1 |Severe acute respiratory syndrome coro   ....................  ....................  ....................  ....................  ....................  ....................
7042 MW190753.1 |Severe acute respiratory syndrome coro   ....................  ....................  ....................  ....................  ....................  ....................
7043 MW190754.1 |Severe acute respiratory syndrome coro   ....................  ....................  ....................  ....................  ....................  ....................
7044 MW190755.1 |Severe acute respiratory syndrome coro   ....................  ....................  ....................  ....................  ....................  ....................
7045 MW190756.1 |Severe acute respiratory syndrome coro   ....................  ....................  ....................  ....................  ....................  ....................
7046 MW190757.1 |Severe acute respiratory syndrome coro   ....................  ....................  ....................  ....................  ....................  ....................
7047 MW190758.1 |Severe acute respiratory syndrome coro   ....................  ....................  ....................  ....................  ....................  ....................
7048 MW190759.1 |Severe acute respiratory syndrome coro   ....................  ....................  ....................  ....................  ....................  ....................
7049 MW190760.1 |Severe acute respiratory syndrome coro   ....................  ....................  ....................  ....................  ....................  ....................
7050 MW190761.1 |Severe acute respiratory syndrome coro   ....................  ....................  ....................  ....................  ....................  ....................
7051 MW190762.1 |Severe acute respiratory syndrome coro   ....................  ....................  ....................  ....................  ....................  ....................
7052 MW190763.1 |Severe acute respiratory syndrome coro   ....................  ....................  ....................  ....................  ....................  ....................
7053 MW190764.1 |Severe acute respiratory syndrome coro   ....................  ....................  ....................  ....................  ....................  ....................
7054 MW190765.1 |Severe acute respiratory syndrome coro   ....................  ....................  ....................  ....................  ....................  ....................
7055 MW190766.1 |Severe acute respiratory syndrome coro   ....................  ....................  ....................  ....................  ....................  ....................
7056 MW190767.1 |Severe acute respiratory syndrome coro   ....................  ....................  ....................  ....................  ....................  ....................
7057 MW190768.1 |Severe acute respiratory syndrome coro   ....................  ....................  ....................  ....................  ....................  ....................
7058 MW190769.1 |Severe acute respiratory syndrome coro   ....................  ....................  ....................  ....................  ....................  ....................
7059 MW190770.1 |Severe acute respiratory syndrome coro   ....................  ....................  ....................  ....................  ....................  ....................
7060 MW190771.1 |Severe acute respiratory syndrome coro   ....................  ....................  ....................  ....................  ....................  ....................
```

```
7061 MW190772.1 |Severe acute respiratory syndrome coro    .................... .................... .................... .................... .................... ....................
7062 MW190773.1 |Severe acute respiratory syndrome coro    .................... .................... .................... .................... .................... ....................
7063 MW190774.1 |Severe acute respiratory syndrome coro    .................... .................... .................... .................... .................... ....................
7064 MW190775.1 |Severe acute respiratory syndrome coro    .................... .................... .................... .................... .................... ....................
7065 MW190776.1 |Severe acute respiratory syndrome coro    .................... .................... .................... .................... .................... ....................
7066 MW190777.1 |Severe acute respiratory syndrome coro    .................... .................... .................... .................... .................... ....................
7067 MW190778.1 |Severe acute respiratory syndrome coro    .................... .................... .................... .................... .................... ....................
7068 MW190779.1 |Severe acute respiratory syndrome coro    .................... .................... .................... .................... .................... ....................
7069 MW190780.1 |Severe acute respiratory syndrome coro    .................... .................... .................... .................... .................... ....................
7070 MW190781.1 |Severe acute respiratory syndrome coro    .................... .................... .................... .................... .................... ....................
7071 MW190782.1 |Severe acute respiratory syndrome coro    .................... .................... .................... .................... .................... ....................
7072 MW190783.1 |Severe acute respiratory syndrome coro    .................... .................... .................... .................... .................... ....................
7073 MW190784.1 |Severe acute respiratory syndrome coro    .................... .................... .................... .................... .................... ....................
7074 MW190785.1 |Severe acute respiratory syndrome coro    .................... .................... .................... .................... .................... ....................
7075 MW190786.1 |Severe acute respiratory syndrome coro    .................... .................... .................... .................... .................... ....................
7076 MW190787.1 |Severe acute respiratory syndrome coro    .................... .................... .................... .................... .................... ....................
7077 MW190788.1 |Severe acute respiratory syndrome coro    .................... .................... .................... .................... .................... ....................
7078 MW190789.1 |Severe acute respiratory syndrome coro    .................... .................... .................... .................... .................... ....................
7079 MW190790.1 |Severe acute respiratory syndrome coro    .................... .................... .................... .................... .................... ....................
7080 MW190791.1 |Severe acute respiratory syndrome coro    .................... .................... .................... .................... .................... ....................
7081 MW190792.1 |Severe acute respiratory syndrome coro    .................... .................... .................... .................... .................... ....................
7082 MW190794.1 |Severe acute respiratory syndrome coro    .................... .................... .................... .................... .................... ....................
7083 MW190795.1 |Severe acute respiratory syndrome coro    .................... .................... .................... .................... .................... ....................
7084 MW190796.1 |Severe acute respiratory syndrome coro    .................... .................... .................... .................... .................... ....................
7085 MW190798.1 |Severe acute respiratory syndrome coro    .................... .................... .................... .................... .................... ....................
7086 MW190799.1 |Severe acute respiratory syndrome coro    .................... .................... .................... .................... .................... ....................
7087 MW190800.1 |Severe acute respiratory syndrome coro    .................... .................... .................... .................... .................... ....................
7088 MW190801.1 |Severe acute respiratory syndrome coro    .................... .................... .................... .................... .................... ....................
7089 MW190803.1 |Severe acute respiratory syndrome coro    .................... .................... .................... .................... .................... ....................
7090 MW190804.1 |Severe acute respiratory syndrome coro    .................... .................... .................... .................... .................... ....................
7091 MW190805.1 |Severe acute respiratory syndrome coro    .................... .................... .................... .................... .................... ....................
7092 MW190806.1 |Severe acute respiratory syndrome coro    .................... .................... .................... .................... .................... ....................
7093 MW190807.1 |Severe acute respiratory syndrome coro    .................... .................... .................... .................... .................... ....................
7094 MW190808.1 |Severe acute respiratory syndrome coro    .................... .................... .................... .................... .................... ....................
7095 MW190809.1 |Severe acute respiratory syndrome coro    .................... .................... .................... .................... .................... ....................
7096 MW190810.1 |Severe acute respiratory syndrome coro    .................... .................... .................... .................... .................... ....................
7097 MW190811.1 |Severe acute respiratory syndrome coro    .................... .................... .................... .................... .................... ....................
7098 MW190812.1 |Severe acute respiratory syndrome coro    .................... .................... .................... .................... .................... ....................
7099 MW190813.1 |Severe acute respiratory syndrome coro    .................... .................... .................... .................... .................... ....................
7100 MW190814.1 |Severe acute respiratory syndrome coro    .................... .................... .................... .................... .................... ....................
7101 MW190815.1 |Severe acute respiratory syndrome coro    .................... .................... .................... .................... .................... ....................
7102 MW190816.1 |Severe acute respiratory syndrome coro    .................... .................... .................... .................... .................... ....................
7103 MW190817.1 |Severe acute respiratory syndrome coro    .................... .................... .................... .................... .................... ....................
7104 MW190818.1 |Severe acute respiratory syndrome coro    .................... .................... .................... .................... .................... ....................
7105 MW190819.1 |Severe acute respiratory syndrome coro    .................... .................... .................... .................... .................... ....................
7106 MW190820.1 |Severe acute respiratory syndrome coro    .................... .................... .................... .................... .................... ....................
7107 MW190822.1 |Severe acute respiratory syndrome coro    .................... .................... .................... .................... .................... ....................
7108 MW190823.1 |Severe acute respiratory syndrome coro    .................... .................... .................... .................... .................... ....................
7109 MW190824.1 |Severe acute respiratory syndrome coro    .................... .................... .................... .................... .................... ....................
7110 MW190825.1 |Severe acute respiratory syndrome coro    .................... .................... .................... .................... .................... ....................
7111 MW190826.1 |Severe acute respiratory syndrome coro    .................... .................... .................... .................... .................... ....................
7112 MW190827.1 |Severe acute respiratory syndrome coro    .................... .................... .................... .................... .................... ....................
7113 MW190828.1 |Severe acute respiratory syndrome coro    .................... .................... .................... .................... .................... ....................
7114 MW190829.1 |Severe acute respiratory syndrome coro    .................... .................... .................... .................... .................... ....................
7115 MW190830.1 |Severe acute respiratory syndrome coro    .................... .................... .................... .................... .................... ....................
7116 MW190831.1 |Severe acute respiratory syndrome coro    .................... .................... .................... .................... .................... ....................
7117 MW190832.1 |Severe acute respiratory syndrome coro    .................... .................... .................... .................... .................... ....................
7118 MW190833.1 |Severe acute respiratory syndrome coro    .................... .................... .................... .................... .................... ....................
7119 MW190834.1 |Severe acute respiratory syndrome coro    .................... .................... .................... .................... .................... ....................
7120 MW190835.1 |Severe acute respiratory syndrome coro    .................... .................... .................... .................... .................... ....................
7121 MW190837.1 |Severe acute respiratory syndrome coro    .................... .................... .................... .................... .................... ....................
7122 MW190838.1 |Severe acute respiratory syndrome coro    .................... .................... .................... .................... .................... ....................
7123 MW190839.1 |Severe acute respiratory syndrome coro    .................... .................... .................... .................... .................... ....................
7124 MW190840.1 |Severe acute respiratory syndrome coro    .................... .................... .................... .................... .................... ....................
7125 MW190841.1 |Severe acute respiratory syndrome coro    .................... .................... .................... .................... .................... ....................
7126 MW190842.1 |Severe acute respiratory syndrome coro    .................... .................... .................... .................... .................... ....................
7127 MW190843.1 |Severe acute respiratory syndrome coro    .................... .................... .................... .................... .................... ....................
7128 MW190844.1 |Severe acute respiratory syndrome coro    .................... .................... .................... .................... .................... ....................
7129 MW190845.1 |Severe acute respiratory syndrome coro    .................... .................... .................... .................... .................... ....................
7130 MW190846.1 |Severe acute respiratory syndrome coro    .................... .................... .................... .................... .................... ....................
7131 MW190847.1 |Severe acute respiratory syndrome coro    .................... .................... .................... .................... .................... ....................
7132 MW190848.1 |Severe acute respiratory syndrome coro    .................... .................... .................... .................... .................... ....................
7133 MW190849.1 |Severe acute respiratory syndrome coro    .................... .................... .................... .................... .................... ....................
7134 MW190850.1 |Severe acute respiratory syndrome coro    .................... .................... .................... .................... .................... ....................
7135 MW190851.1 |Severe acute respiratory syndrome coro    .................... .................... .................... .................... .................... ....................
7136 MW190852.1 |Severe acute respiratory syndrome coro    .................... .................... .................... .................... .................... ....................
7137 MW190853.1 |Severe acute respiratory syndrome coro    .................... .................... .................... .................... .................... ....................
7138 MW190854.1 |Severe acute respiratory syndrome coro    .................... .................... .................... .................... .................... ....................
```

```
7139 MW190855.1 |Severe acute respiratory syndrome coro    ....................  ....................  ....................  ....................  ....................  ....................
7140 MW190856.1 |Severe acute respiratory syndrome coro    ....................  ....................  ....................  ....................  ....................  ....................
7141 MW190857.1 |Severe acute respiratory syndrome coro    ....................  ....................  ....................  ....................  ....................  ....................
7142 MW190858.1 |Severe acute respiratory syndrome coro    ....................  ....................  ....................  ....................  ....................  ....................
7143 MW190859.1 |Severe acute respiratory syndrome coro    ....................  ....................  ....................  ....................  ....................  ....................
7144 MW190860.1 |Severe acute respiratory syndrome coro    ....................  ....................  ....................  ....................  ....................  ....................
7145 MW190861.1 |Severe acute respiratory syndrome coro    ....................  ....................  ....................  ....................  ....................  ....................
7146 MW190862.1 |Severe acute respiratory syndrome coro    ....................  ....................  ....................  ....................  ....................  ....................
7147 MW190863.1 |Severe acute respiratory syndrome coro    ....................  ....................  ....................  ....................  ....................  ....................
7148 MW190864.1 |Severe acute respiratory syndrome coro    ....................  ....................  ....................  ....................  ....................  ....................
7149 MW190865.1 |Severe acute respiratory syndrome coro    ....................  ....................  ....................  ....................  ....................  ....................
7150 MW190866.1 |Severe acute respiratory syndrome coro    ....................  ....................  ....................  ....................  ....................  ....................
7151 MW190867.1 |Severe acute respiratory syndrome coro    ....................  ....................  ....................  ....................  ....................  ....................
7152 MW190868.1 |Severe acute respiratory syndrome coro    ....................  ....................  ....................  ....................  ....................  ....................
7153 MW190869.1 |Severe acute respiratory syndrome coro    ....................  ....................  ....................  ....................  ....................  ....................
7154 MW190870.1 |Severe acute respiratory syndrome coro    ....................  ....................  ....................  ....................  ....................  ....................
7155 MW190871.1 |Severe acute respiratory syndrome coro    ....................  ....................  ....................  ....................  ....................  ....................
7156 MW190872.1 |Severe acute respiratory syndrome coro    ....................  ....................  ....................  ....................  ....................  ....................
7157 MW190873.1 |Severe acute respiratory syndrome coro    ....................  ....................  ....................  ....................  ....................  ....................
7158 MW190874.1 |Severe acute respiratory syndrome coro    ....................  ....................  ....................  ....................  ....................  ....................
7159 MW190875.1 |Severe acute respiratory syndrome coro    ....................  ....................  ....................  ....................  ....................  ....................
7160 MW190876.1 |Severe acute respiratory syndrome coro    ....................  ....................  ....................  ....................  ....................  ....................
7161 MW190877.1 |Severe acute respiratory syndrome coro    ....................  ....................  ....................  ....................  ....................  ....................
7162 MW190878.1 |Severe acute respiratory syndrome coro    ....................  ....................  ....................  ....................  ....................  ....................
7163 MW190880.1 |Severe acute respiratory syndrome coro    ....................  ....................  ....................  ....................  ....................  ....................
7164 MW190881.1 |Severe acute respiratory syndrome coro    ....................  ....................  ....................  ....................  ....................  ....................
7165 MW190882.1 |Severe acute respiratory syndrome coro    ....................  ....................  ....................  ....................  ....................  ....................
7166 MW190883.1 |Severe acute respiratory syndrome coro    ....................  ....................  ....................  ....................  ....................  ....................
7167 MW190884.1 |Severe acute respiratory syndrome coro    ....................  ....................  ....................  ....................  ....................  ....................
7168 MW190885.1 |Severe acute respiratory syndrome coro    ....................  ....................  ....................  ....................  ....................  ....................
7169 MW190887.1 |Severe acute respiratory syndrome coro    ....................  ....................  ....................  ....................  ....................  ....................
7170 MW190888.1 |Severe acute respiratory syndrome coro    ....................  ....................  ....................  ....................  ....................  ....................
7171 MW190889.1 |Severe acute respiratory syndrome coro    ....................  ....................  ....................  ....................  ....................  ....................
7172 MW190890.1 |Severe acute respiratory syndrome coro    ....................  ....................  ....................  ....................  ....................  ....................
7173 MW190891.1 |Severe acute respiratory syndrome coro    ....................  ....................  ....................  ....................  ....................  ....................
7174 MW190892.1 |Severe acute respiratory syndrome coro    ....................  ....................  ....................  ....................  ....................  ....................
7175 MW190893.1 |Severe acute respiratory syndrome coro    ....................  ....................  ....................  ....................  ....................  ....................
7176 MW190894.1 |Severe acute respiratory syndrome coro    ....................  ....................  ....................  ....................  ....................  ....................
7177 MW190895.1 |Severe acute respiratory syndrome coro    ....................  ....................  ....................  ....................  ....................  ....................
7178 MW190896.1 |Severe acute respiratory syndrome coro    ....................  ....................  ....................  ....................  ....................  ....................
7179 MW190897.1 |Severe acute respiratory syndrome coro    ....................  ....................  ....................  ....................  ....................  ....................
7180 MW190898.1 |Severe acute respiratory syndrome coro    ....................  ....................  ....................  ....................  ....................  ....................
7181 MW190899.1 |Severe acute respiratory syndrome coro    ....................  ....................  ....................  ....................  ....................  ....................
7182 MW190900.1 |Severe acute respiratory syndrome coro    ....................  ....................  ....................  ....................  ....................  ....................
7183 MW190901.1 |Severe acute respiratory syndrome coro    ....................  ....................  ....................  ....................  ....................  ....................
7184 MW190902.1 |Severe acute respiratory syndrome coro    ....................  ....................  ....................  ....................  ....................  ....................
7185 MW190903.1 |Severe acute respiratory syndrome coro    ....................  ....................  ....................  ....................  ....................  ....................
7186 MW190904.1 |Severe acute respiratory syndrome coro    ....................  ....................  ....................  ....................  ....................  ....................
7187 MW190905.1 |Severe acute respiratory syndrome coro    ....................  ....................  ....................  ....................  ....................  ....................
7188 MW190906.1 |Severe acute respiratory syndrome coro    ....................  ....................  ....................  ....................  ....................  ....................
7189 MW190907.1 |Severe acute respiratory syndrome coro    ....................  ....................  ....................  ....................  ....................  ....................
7190 MW190908.1 |Severe acute respiratory syndrome coro    ....................  ....................  ....................  ....................  ....................  ....................
7191 MW190909.1 |Severe acute respiratory syndrome coro    ....................  ....................  ....................  ....................  ....................  ....................
7192 MW190910.1 |Severe acute respiratory syndrome coro    ....................  ....................  ....................  ....................  ....................  ....................
7193 MW190911.1 |Severe acute respiratory syndrome coro    ....................  ....................  ....................  ....................  ....................  ....................
7194 MW190912.1 |Severe acute respiratory syndrome coro    ....................  ....................  ....................  ....................  ....................  ....................
7195 MW190913.1 |Severe acute respiratory syndrome coro    ....................  ....................  ....................  ....................  ....................  ....................
7196 MW190914.1 |Severe acute respiratory syndrome coro    ....................  ....................  ....................  ....................  ....................  ....................
7197 MW190915.1 |Severe acute respiratory syndrome coro    ....................  ....................  ....................  ....................  ....................  ....................
7198 MW190916.1 |Severe acute respiratory syndrome coro    ....................  ....................  ....................  ....................  ....................  ....................
7199 MW190917.1 |Severe acute respiratory syndrome coro    ....................  ....................  ....................  ....................  ....................  ....................
7200 MW190918.1 |Severe acute respiratory syndrome coro    ....................  ....................  ....................  ....................  ....................  ....................
7201 MW190919.1 |Severe acute respiratory syndrome coro    ....................  ....................  ....................  ....................  ....................  ....................
7202 MW190920.1 |Severe acute respiratory syndrome coro    ....................  ....................  ....................  ....................  ....................  ....................
7203 MW190921.1 |Severe acute respiratory syndrome coro    ....................  ....................  ....................  ....................  ....................  ....................
7204 MW190922.1 |Severe acute respiratory syndrome coro    ....................  ....................  ....................  ....................  ....................  ....................
7205 MW190923.1 |Severe acute respiratory syndrome coro    ....................  ....................  ....................  ....................  ....................  ....................
7206 MW190924.1 |Severe acute respiratory syndrome coro    ....................  ....................  ....................  ....................  ....................  ....................
7207 MW190925.1 |Severe acute respiratory syndrome coro    ....................  ....................  ....................  ....................  ....................  ....................
7208 MW190926.1 |Severe acute respiratory syndrome coro    ....................  ....................  ....................  ....................  ....................  ....................
7209 MW190927.1 |Severe acute respiratory syndrome coro    ....................  ....................  ....................  ....................  ....................  ....................
7210 MW190928.1 |Severe acute respiratory syndrome coro    ....................  ....................  ....................  ....................  ....................  ....................
7211 MW190929.1 |Severe acute respiratory syndrome coro    ....................  ....................  ....................  ....................  ....................  ....................
7212 MW190930.1 |Severe acute respiratory syndrome coro    ....................  ....................  ....................  ....................  ....................  ....................
7213 MW190931.1 |Severe acute respiratory syndrome coro    ....................  ....................  ....................  ....................  ....................  ....................
7214 MW190932.1 |Severe acute respiratory syndrome coro    ....................  ....................  ....................  ....................  ....................  ....................
7215 MW190933.1 |Severe acute respiratory syndrome coro    ....................  ....................  ....................  ....................  ....................  ....................
7216 MW190934.1 |Severe acute respiratory syndrome coro    ....................  ....................  ....................  ....................  ....................  ....................
```

7217	MW190935.1	|Severe acute respiratory syndrome coro	....	....	....	....	....	....
7218	MW190936.1	|Severe acute respiratory syndrome coro	....	....	....	....	....	....
7219	MW190937.1	|Severe acute respiratory syndrome coro	....	....	....	....	....	....
7220	MW190938.1	|Severe acute respiratory syndrome coro	....	....	....	....	....	....
7221	MW190940.1	|Severe acute respiratory syndrome coro	....	....	....	....	....	....
7222	MW190941.1	|Severe acute respiratory syndrome coro	....	....	....	....	....	....
7223	MW190942.1	|Severe acute respiratory syndrome coro	....	....	....	....	....	....
7224	MW190943.1	|Severe acute respiratory syndrome coro	....	....	....	....	....	....
7225	MW190944.1	|Severe acute respiratory syndrome coro	....	....	....	....	....	....
7226	MW190945.1	|Severe acute respiratory syndrome coro	....	....	....	....	....	....
7227	MW190946.1	|Severe acute respiratory syndrome coro	....	....	....	....	....	....
7228	MW190947.1	|Severe acute respiratory syndrome coro	....	....	....	....	....	....
7229	MW190948.1	|Severe acute respiratory syndrome coro	....	....	....	....	....	....
7230	MW190949.1	|Severe acute respiratory syndrome coro	....	....	....	....	....	....
7231	MW190950.1	|Severe acute respiratory syndrome coro	....	....	....	....	....	....
7232	MW190951.1	|Severe acute respiratory syndrome coro	....	....	....	....	....	....
7233	MW190952.1	|Severe acute respiratory syndrome coro	....	....	....	....	....	....
7234	MW190953.1	|Severe acute respiratory syndrome coro	....	....	....	....	....	....
7235	MW190954.1	|Severe acute respiratory syndrome coro	....	....	....	....	....	....
7236	MW190955.1	|Severe acute respiratory syndrome coro	....	....	....	....	....	....
7237	MW190956.1	|Severe acute respiratory syndrome coro	....	....	....	....	....	....
7238	MW190957.1	|Severe acute respiratory syndrome coro	....	....	....	....	....	....
7239	MW190958.1	|Severe acute respiratory syndrome coro	....	....	....	....	....	....
7240	MW190959.1	|Severe acute respiratory syndrome coro	....	....	....	....	....	....
7241	MW190960.1	|Severe acute respiratory syndrome coro	....	....	....	....	....	....
7242	MW190961.1	|Severe acute respiratory syndrome coro	....	....	....	....	....	....
7243	MW190962.1	|Severe acute respiratory syndrome coro	....	....	....	....	....	....
7244	MW190963.1	|Severe acute respiratory syndrome coro	....	....	....	....	....	....
7245	MW190964.1	|Severe acute respiratory syndrome coro	....	....	....	....	....	....
7246	MW190965.1	|Severe acute respiratory syndrome coro	....	....	....	....	....	....
7247	MW190966.1	|Severe acute respiratory syndrome coro	....	....	....	....	....	....
7248	MW190967.1	|Severe acute respiratory syndrome coro	....	....	....	....	....	....
7249	MW190968.1	|Severe acute respiratory syndrome coro	....	....	....	....	....	....
7250	MW190969.1	|Severe acute respiratory syndrome coro	....	....	....	....	....	....
7251	MW190970.1	|Severe acute respiratory syndrome coro	....	....	....	....	....	....
7252	MW190971.1	|Severe acute respiratory syndrome coro	....	....	....	....	....	....
7253	MW190972.1	|Severe acute respiratory syndrome coro	....	....	....	....	....	....
7254	MW190973.1	|Severe acute respiratory syndrome coro	....	....	....	....	....	....
7255	MW190974.1	|Severe acute respiratory syndrome coro	....	....	....	....	....	....
7256	MW190975.1	|Severe acute respiratory syndrome coro	....	....	....	....	....	....
7257	MW190976.1	|Severe acute respiratory syndrome coro	....	....	....	....	....	....
7258	MW190977.1	|Severe acute respiratory syndrome coro	....	....	....	....	....	....
7259	MW190978.1	|Severe acute respiratory syndrome coro	....	....	....	....	....	....
7260	MW190979.1	|Severe acute respiratory syndrome coro	....	....	....	....	....	....
7261	MW190980.1	|Severe acute respiratory syndrome coro	....	....	....	....	....	....
7262	MW190981.1	|Severe acute respiratory syndrome coro	....	....	....	....	....	....
7263	MW190982.1	|Severe acute respiratory syndrome coro	....	....	....	....	....	....
7264	MW190983.1	|Severe acute respiratory syndrome coro	....	....	....	....	....	....
7265	MW190984.1	|Severe acute respiratory syndrome coro	....	....	....	....	....	....
7266	MW190985.1	|Severe acute respiratory syndrome coro	....	....	....	....	....	....
7267	MW190986.1	|Severe acute respiratory syndrome coro	....	....	....	....	....	....
7268	MW190987.1	|Severe acute respiratory syndrome coro	....	....	....	....	....	....
7269	MW190988.1	|Severe acute respiratory syndrome coro	....	....	....	....	....	....
7270	MW190989.1	|Severe acute respiratory syndrome coro	....	....	....	....	....	....
7271	MW190990.1	|Severe acute respiratory syndrome coro	....	....	....	....	....	....
7272	MW190991.1	|Severe acute respiratory syndrome coro	....	....	....	....	....	....
7273	MW190992.1	|Severe acute respiratory syndrome coro	....	....	....	....	....	....
7274	MW190993.1	|Severe acute respiratory syndrome coro	....	....	....	....	....	....
7275	MW190994.1	|Severe acute respiratory syndrome coro	....	....	....	....	....	....
7276	MW190995.1	|Severe acute respiratory syndrome coro	....	....	....	....	....	....
7277	MW190996.1	|Severe acute respiratory syndrome coro	....	....	....	....	....	....
7278	MW190999.1	|Severe acute respiratory syndrome coro	....	....	....	....	....	....
7279	MW191000.1	|Severe acute respiratory syndrome coro	....	....	....	....	....	....
7280	MW191001.1	|Severe acute respiratory syndrome coro	....	....	....	....	....	....
7281	MW191002.1	|Severe acute respiratory syndrome coro	....	....	....	....	....	....
7282	MW191003.1	|Severe acute respiratory syndrome coro	....	....	....	....	....	....
7283	MW191004.1	|Severe acute respiratory syndrome coro	....	....	....	....	....	....
7284	MW191005.1	|Severe acute respiratory syndrome coro	....	....	....	....	....	....
7285	MW191006.1	|Severe acute respiratory syndrome coro	....	....	....	....	....	....
7286	MW191007.1	|Severe acute respiratory syndrome coro	....	....	....	....	....	....
7287	MW191008.1	|Severe acute respiratory syndrome coro	....	....	....	....	......A.............	....
7288	MW191009.1	|Severe acute respiratory syndrome coro	....	....	....	....	....	....
7289	MW191010.1	|Severe acute respiratory syndrome coro	....	....	....	....	....	....
7290	MW191011.1	|Severe acute respiratory syndrome coro	....	....	....	....	....	....
7291	MW191012.1	|Severe acute respiratory syndrome coro	....	....	....	....	....	....
7292	MW191013.1	|Severe acute respiratory syndrome coro	....	....	....	....	....	....
7293	MW191014.1	|Severe acute respiratory syndrome coro	....	....	....	....	....	....
7294	MW191015.1	|Severe acute respiratory syndrome coro	....	....	....	....	....	....

```
7295 MW191016.1 |Severe acute respiratory syndrome coro    ....................  ....................  .....................  ....................  ....................  ....................
7296 MW191017.1 |Severe acute respiratory syndrome coro    ....................  ....................  .....................  ....................  ....................  ....................
7297 MW191018.1 |Severe acute respiratory syndrome coro    ....................  ....................  .....................  ....................  ....................  ....................
7298 MW191019.1 |Severe acute respiratory syndrome coro    ....................  ....................  .....................  ....................  ....................  ....................
7299 MW191020.1 |Severe acute respiratory syndrome coro    ....................  ....................  .....................  ....................  ....................  ....................
7300 MW191021.1 |Severe acute respiratory syndrome coro    ....................  ....................  .....................  ....................  ....................  ....................
7301 MW191022.1 |Severe acute respiratory syndrome coro    ....................  ....................  .....................  ....................  ....................  ....................
7302 MW191023.1 |Severe acute respiratory syndrome coro    ....................  ....................  .....................  ....................  ....................  ....................
7303 MW191025.1 |Severe acute respiratory syndrome coro    ....................  ....................  .....................  ....................  ....................  ....................
7304 MW191026.1 |Severe acute respiratory syndrome coro    ....................  ....................  .....................  ....................  ....................  ....................
7305 MW191027.1 |Severe acute respiratory syndrome coro    ....................  ....................  .....................  ....................  ....................  ....................
7306 MW191028.1 |Severe acute respiratory syndrome coro    ....................  ....................  .....................  ....................  ....................  ....................
7307 MW191029.1 |Severe acute respiratory syndrome coro    ....................  ....................  .....................  ....................  ....................  ....................
7308 MW191030.1 |Severe acute respiratory syndrome coro    ....................  ....................  .....................  ....................  ....................  ....................
7309 MW191032.1 |Severe acute respiratory syndrome coro    ....................  ....................  .....................  ....................  ....................  ....................
7310 MW191033.1 |Severe acute respiratory syndrome coro    ....................  ....................  .....................  ....................  ....................  ....................
7311 MW191034.1 |Severe acute respiratory syndrome coro    ....................  ....................  .....................  ....................  ....................  ....................
7312 MW191035.1 |Severe acute respiratory syndrome coro    ....................  ....................  .....................  ....................  ....................  ....................
7313 MW191036.1 |Severe acute respiratory syndrome coro    ....................  ....................  .....................  ....................  ....................  ....................
7314 MW191037.1 |Severe acute respiratory syndrome coro    ....................  ....................  .....................  ....................  ....................  ....................
7315 MW191038.1 |Severe acute respiratory syndrome coro    ....................  ....................  .....................  ....................  ....................  ....................
7316 MW191039.1 |Severe acute respiratory syndrome coro    ....................  ....................  .....................  ....................  ....................  ....................
7317 MW191040.1 |Severe acute respiratory syndrome coro    ....................  ....................  .....................  ....................  ....................  ....................
7318 MW191041.1 |Severe acute respiratory syndrome coro    ....................  ....................  .....................  ....................  ....................  ....................
7319 MW191042.1 |Severe acute respiratory syndrome coro    ....................  ....................  .....................  ....................  ....................  ....................
7320 MW191043.1 |Severe acute respiratory syndrome coro    ....................  ....................  .....................  ....................  ....................  ....................
7321 MW191044.1 |Severe acute respiratory syndrome coro    ....................  ....................  .....................  ....................  ....................  ....................
7322 MW191045.1 |Severe acute respiratory syndrome coro    ....................  ....................  .....................  ....................  ....................  ....................
7323 MW191046.1 |Severe acute respiratory syndrome coro    ....................  ....................  .....................  ....................  ....................  ....................
7324 MW191047.1 |Severe acute respiratory syndrome coro    ....................  ....................  .....................  ....................  ....................  ....................
7325 MW191048.1 |Severe acute respiratory syndrome coro    ....................  ....................  .....................  ....................  ....................  ....................
7326 MW191049.1 |Severe acute respiratory syndrome coro    ....................  ....................  .....................  ....................  ....................  ....................
7327 MW191050.1 |Severe acute respiratory syndrome coro    ....................  ....................  .....................  ....................  ....................  ....................
7328 MW191051.1 |Severe acute respiratory syndrome coro    ....................  ....................  .....................  ....................  ....................  ....................
7329 MW191052.1 |Severe acute respiratory syndrome coro    ....................  ....................  .....................  ....................  ....................  ....................
7330 MW191053.1 |Severe acute respiratory syndrome coro    ....................  ....................  .....................  ....................  ....................  ....................
7331 MW191054.1 |Severe acute respiratory syndrome coro    ....................  ....................  .....................  ....................  ....................  ....................
7332 MW191055.1 |Severe acute respiratory syndrome coro    ....................  ....................  .....................  ....................  ....................  ....................
7333 MW191056.1 |Severe acute respiratory syndrome coro    ....................  ....................  .....................  ....................  ....................  ....................
7334 MW191057.1 |Severe acute respiratory syndrome coro    ....................  ....................  .....................  ....................  ....................  ....................
7335 MW191058.1 |Severe acute respiratory syndrome coro    ....................  ....................  .....................  ....................  ....................  ....................
7336 MW191059.1 |Severe acute respiratory syndrome coro    ....................  ....................  .....................  ....................  ....................  ....................
7337 MW191060.1 |Severe acute respiratory syndrome coro    ....................  ....................  .....................  ....................  ....................  ....................
7338 MW191061.1 |Severe acute respiratory syndrome coro    ....................  ....................  .....................  ....................  ....................  ....................
7339 MW191062.1 |Severe acute respiratory syndrome coro    ....................  ....................  .....................  ....................  ....................  ....................
7340 MW191063.1 |Severe acute respiratory syndrome coro    ....................  ....................  .....................  ....................  ....................  ....................
7341 MW191065.1 |Severe acute respiratory syndrome coro    ....................  ....................  .....................  ....................  ....................  ....................
7342 MW191066.1 |Severe acute respiratory syndrome coro    ....................  ....................  .....................  ....................  ....................  ....................
7343 MW191067.1 |Severe acute respiratory syndrome coro    ....................  ....................  .....................  ....................  ....................  ....................
7344 MW191068.1 |Severe acute respiratory syndrome coro    ....................  ....................  .....................  ....................  ....................  ....................
7345 MW191069.1 |Severe acute respiratory syndrome coro    ....................  ....................  .....................  ....................  ....................  ....................
7346 MW191070.1 |Severe acute respiratory syndrome coro    ....................  ....................  .....................  ....................  ....................  ....................
7347 MW191071.1 |Severe acute respiratory syndrome coro    ....................  ....................  .....................  ....................  ....................  ....................
7348 MW191072.1 |Severe acute respiratory syndrome coro    ....................  ....................  .....................  ....................  ....................  ....................
7349 MW191073.1 |Severe acute respiratory syndrome coro    ....................  ....................  .....................  ....................  ....................  ....................
7350 MW191074.1 |Severe acute respiratory syndrome coro    ....................  ....................  .....................  ....................  ....................  ....................
7351 MW191075.1 |Severe acute respiratory syndrome coro    ....................  ....................  .....................  ....................  ....................  ....................
7352 MW191076.1 |Severe acute respiratory syndrome coro    ....................  ....................  .....................  ....................  ....................  ....................
7353 MW191077.1 |Severe acute respiratory syndrome coro    ....................  ....................  .....................  ....................  ....................  ....................
7354 MW191078.1 |Severe acute respiratory syndrome coro    ....................  ....................  .....................  ....................  ....................  ....................
7355 MW191079.1 |Severe acute respiratory syndrome coro    ....................  ....................  .....................  ....................  ....................  ....................
7356 MW191080.1 |Severe acute respiratory syndrome coro    ....................  ....................  .....................  ....................  ....................  ....................
7357 MW191081.1 |Severe acute respiratory syndrome coro    ....................  ....................  .....................  ....................  ....................  ....................
7358 MW191082.1 |Severe acute respiratory syndrome coro    ....................  ....................  .....................  ....................  ....................  ....................
7359 MW191083.1 |Severe acute respiratory syndrome coro    ....................  ....................  .....................  ....................  ....................  ....................
7360 MW191084.1 |Severe acute respiratory syndrome coro    ....................  ....................  .....................  ....................  ....................  ....................
7361 MW191085.1 |Severe acute respiratory syndrome coro    ....................  ....................  .....................  ....................  ....................  ....................
7362 MW191086.1 |Severe acute respiratory syndrome coro    ....................  ....................  .....................  ....................  ....................  ....................
7363 MW191087.1 |Severe acute respiratory syndrome coro    ....................  ....................  .....................  ....................  ....................  ....................
7364 MW191088.1 |Severe acute respiratory syndrome coro    ....................  ....................  .....................  ....................  ....................  ....................
7365 MW191089.1 |Severe acute respiratory syndrome coro    ....................  ....................  .....................  ....................  ....................  ....................
7366 MW191090.1 |Severe acute respiratory syndrome coro    ....................  ....................  .....................  ....................  ....................  ....................
7367 MW191091.1 |Severe acute respiratory syndrome coro    ....................  ....................  .....................  ....................  ....................  ....................
7368 MW191092.1 |Severe acute respiratory syndrome coro    ....................  ....................  .....................  ....................  ....................  ....................
7369 MW191093.1 |Severe acute respiratory syndrome coro    ....................  ....................  .....................  ....................  ....................  ....................
7370 MW191094.1 |Severe acute respiratory syndrome coro    ....................  ....................  .....................  ....................  ....................  ....................
7371 MW191095.1 |Severe acute respiratory syndrome coro    ....................  ....................  .....................  ....................  ....................  ....................
7372 MW191096.1 |Severe acute respiratory syndrome coro    ....................  ....................  .....................  ....................  ....................  ....................
```

7373 MW191097.1 |Severe acute respiratory syndrome coro ....
7374 MW191098.1 |Severe acute respiratory syndrome coro ....
7375 MW191099.1 |Severe acute respiratory syndrome coro ....
7376 MW191101.1 |Severe acute respiratory syndrome coro ....
7377 MW191102.1 |Severe acute respiratory syndrome coro ....
7378 MW191103.1 |Severe acute respiratory syndrome coro ....
7379 MW191104.1 |Severe acute respiratory syndrome coro ....
7380 MW191105.1 |Severe acute respiratory syndrome coro ....
7381 MW191106.1 |Severe acute respiratory syndrome coro ....
7382 MW191107.1 |Severe acute respiratory syndrome coro ....
7383 MW191108.1 |Severe acute respiratory syndrome coro ....
7384 MW191109.1 |Severe acute respiratory syndrome coro ....
7385 MW191110.1 |Severe acute respiratory syndrome coro ....
7386 MW191111.1 |Severe acute respiratory syndrome coro ....
7387 MW191112.1 |Severe acute respiratory syndrome coro ....
7388 MW191113.1 |Severe acute respiratory syndrome coro ....
7389 MW191114.1 |Severe acute respiratory syndrome coro ....
7390 MW191115.1 |Severe acute respiratory syndrome coro ....
7391 MW191116.1 |Severe acute respiratory syndrome coro ....
7392 MW191117.1 |Severe acute respiratory syndrome coro ....
7393 MW191118.1 |Severe acute respiratory syndrome coro ....
7394 MW191119.1 |Severe acute respiratory syndrome coro ....
7395 MW191120.1 |Severe acute respiratory syndrome coro ....
7396 MW191121.1 |Severe acute respiratory syndrome coro ....
7397 MW191122.1 |Severe acute respiratory syndrome coro ....
7398 MW191123.1 |Severe acute respiratory syndrome coro ....
7399 MW191124.1 |Severe acute respiratory syndrome coro ....
7400 MW191125.1 |Severe acute respiratory syndrome coro ....
7401 MW191126.1 |Severe acute respiratory syndrome coro ....
7402 MW191127.1 |Severe acute respiratory syndrome coro ....
7403 MW191128.1 |Severe acute respiratory syndrome coro ....
7404 MW191129.1 |Severe acute respiratory syndrome coro ....
7405 MW191130.1 |Severe acute respiratory syndrome coro ....
7406 MW191132.1 |Severe acute respiratory syndrome coro ....
7407 MW191134.1 |Severe acute respiratory syndrome coro ....
7408 MW191135.1 |Severe acute respiratory syndrome coro ....
7409 MW191136.1 |Severe acute respiratory syndrome coro ....
7410 MW191137.1 |Severe acute respiratory syndrome coro ....
7411 MW191138.1 |Severe acute respiratory syndrome coro ....
7412 MW191139.1 |Severe acute respiratory syndrome coro ....
7413 MW191140.1 |Severe acute respiratory syndrome coro ....
7414 MW191141.1 |Severe acute respiratory syndrome coro ....
7415 MW191142.1 |Severe acute respiratory syndrome coro ....
7416 MW191143.1 |Severe acute respiratory syndrome coro ....
7417 MW191144.1 |Severe acute respiratory syndrome coro ....
7418 MW191145.1 |Severe acute respiratory syndrome coro ....
7419 MW191146.1 |Severe acute respiratory syndrome coro ....
7420 MW191147.1 |Severe acute respiratory syndrome coro ....
7421 MW191148.1 |Severe acute respiratory syndrome coro ....
7422 MW191149.1 |Severe acute respiratory syndrome coro ....
7423 MW191150.1 |Severe acute respiratory syndrome coro ....
7424 MW191151.1 |Severe acute respiratory syndrome coro ....
7425 MW191152.1 |Severe acute respiratory syndrome coro ....
7426 MW191153.1 |Severe acute respiratory syndrome coro ....
7427 MW191154.1 |Severe acute respiratory syndrome coro ....
7428 MW191155.1 |Severe acute respiratory syndrome coro ....
7429 MW191156.1 |Severe acute respiratory syndrome coro ....
7430 MW191157.1 |Severe acute respiratory syndrome coro ....
7431 MW191158.1 |Severe acute respiratory syndrome coro ....
7432 MW191159.1 |Severe acute respiratory syndrome coro ....
7433 MW191160.1 |Severe acute respiratory syndrome coro ....
7434 MW191161.1 |Severe acute respiratory syndrome coro ....
7435 MW191163.1 |Severe acute respiratory syndrome coro ....
7436 MW191164.1 |Severe acute respiratory syndrome coro ....
7437 MW191165.1 |Severe acute respiratory syndrome coro ....
7438 MW191166.1 |Severe acute respiratory syndrome coro ....
7439 MW191167.1 |Severe acute respiratory syndrome coro ....
7440 MW191168.1 |Severe acute respiratory syndrome coro ....
7441 MW191169.1 |Severe acute respiratory syndrome coro ....
7442 MW191170.1 |Severe acute respiratory syndrome coro ....
7443 MW191171.1 |Severe acute respiratory syndrome coro ....
7444 MW191172.1 |Severe acute respiratory syndrome coro ....A...... ....
7445 MW191173.1 |Severe acute respiratory syndrome coro ....
7446 MW191174.1 |Severe acute respiratory syndrome coro ....
7447 MW191175.1 |Severe acute respiratory syndrome coro ....
7448 MW191176.1 |Severe acute respiratory syndrome coro ....
7449 MW191177.1 |Severe acute respiratory syndrome coro ....
7450 MW191178.1 |Severe acute respiratory syndrome coro ....

7451 MW191179.1 |Severe acute respiratory syndrome coro ....
7452 MW191180.1 |Severe acute respiratory syndrome coro ....
7453 MW191181.1 |Severe acute respiratory syndrome coro ....
7454 MW191182.1 |Severe acute respiratory syndrome coro ....
7455 MW191183.1 |Severe acute respiratory syndrome coro ....
7456 MW191184.1 |Severe acute respiratory syndrome coro ....
7457 MW191185.1 |Severe acute respiratory syndrome coro ....
7458 MW191186.1 |Severe acute respiratory syndrome coro ....
7459 MW191187.1 |Severe acute respiratory syndrome coro ....
7460 MW191188.1 |Severe acute respiratory syndrome coro ....
7461 MW191189.1 |Severe acute respiratory syndrome coro ....
7462 MW191190.1 |Severe acute respiratory syndrome coro ....
7463 MW191191.1 |Severe acute respiratory syndrome coro ....
7464 MW191192.1 |Severe acute respiratory syndrome coro ....
7465 MW191193.1 |Severe acute respiratory syndrome coro ....
7466 MW191194.1 |Severe acute respiratory syndrome coro ....
7467 MW191195.1 |Severe acute respiratory syndrome coro ....
7468 MW191196.1 |Severe acute respiratory syndrome coro ....
7469 MW191197.1 |Severe acute respiratory syndrome coro ....
7470 MW191198.1 |Severe acute respiratory syndrome coro ....
7471 MW191199.1 |Severe acute respiratory syndrome coro ....
7472 MW191200.1 |Severe acute respiratory syndrome coro ....
7473 MW191201.1 |Severe acute respiratory syndrome coro ....
7474 MW191202.1 |Severe acute respiratory syndrome coro ....
7475 MW191203.1 |Severe acute respiratory syndrome coro ....
7476 MW191204.1 |Severe acute respiratory syndrome coro ....
7477 MW191205.1 |Severe acute respiratory syndrome coro ....
7478 MW191206.1 |Severe acute respiratory syndrome coro ....
7479 MW191207.1 |Severe acute respiratory syndrome coro ....
7480 MW191208.1 |Severe acute respiratory syndrome coro ....
7481 MW191209.1 |Severe acute respiratory syndrome coro ....
7482 MW191210.1 |Severe acute respiratory syndrome coro ....
7483 MW191211.1 |Severe acute respiratory syndrome coro ....
7484 MW191212.1 |Severe acute respiratory syndrome coro ....
7485 MW191213.1 |Severe acute respiratory syndrome coro ....
7486 MW191214.1 |Severe acute respiratory syndrome coro ....
7487 MW191215.1 |Severe acute respiratory syndrome coro ....
7488 MW191216.1 |Severe acute respiratory syndrome coro ....
7489 MW191217.1 |Severe acute respiratory syndrome coro ....
7490 MW191218.1 |Severe acute respiratory syndrome coro ....
7491 MW191219.1 |Severe acute respiratory syndrome coro ....
7492 MW191220.1 |Severe acute respiratory syndrome coro ....
7493 MW191221.1 |Severe acute respiratory syndrome coro ....
7494 MW191222.1 |Severe acute respiratory syndrome coro ....
7495 MW191223.1 |Severe acute respiratory syndrome coro ....
7496 MW191224.1 |Severe acute respiratory syndrome coro ....
7497 MW191225.1 |Severe acute respiratory syndrome coro ....
7498 MW191226.1 |Severe acute respiratory syndrome coro ....
7499 MW191227.1 |Severe acute respiratory syndrome coro ....
7500 MW191228.1 |Severe acute respiratory syndrome coro ....
7501 MW191229.1 |Severe acute respiratory syndrome coro ....
7502 MW191230.1 |Severe acute respiratory syndrome coro ....
7503 MW191231.1 |Severe acute respiratory syndrome coro ....
7504 MW191232.1 |Severe acute respiratory syndrome coro ....
7505 MW191233.1 |Severe acute respiratory syndrome coro ....
7506 MW191234.1 |Severe acute respiratory syndrome coro ....
7507 MW191235.1 |Severe acute respiratory syndrome coro ....
7508 MW191236.1 |Severe acute respiratory syndrome coro ....
7509 MW191237.1 |Severe acute respiratory syndrome coro ....
7510 MW191238.1 |Severe acute respiratory syndrome coro ....
7511 MW191239.1 |Severe acute respiratory syndrome coro ....
7512 MW191240.1 |Severe acute respiratory syndrome coro ....
7513 MW191241.1 |Severe acute respiratory syndrome coro ....
7514 MW191242.1 |Severe acute respiratory syndrome coro ....
7515 MW191243.1 |Severe acute respiratory syndrome coro ....
7516 MW191244.1 |Severe acute respiratory syndrome coro ....
7517 MW191245.1 |Severe acute respiratory syndrome coro ....
7518 MW191246.1 |Severe acute respiratory syndrome coro ....
7519 MW191247.1 |Severe acute respiratory syndrome coro ....
7520 MW191248.1 |Severe acute respiratory syndrome coro ....
7521 MW191249.1 |Severe acute respiratory syndrome coro ....
7522 MW191250.1 |Severe acute respiratory syndrome coro ....
7523 MW191251.1 |Severe acute respiratory syndrome coro ....
7524 MW191252.1 |Severe acute respiratory syndrome coro ....
7525 MW191253.1 |Severe acute respiratory syndrome coro ....
7526 MW191255.1 |Severe acute respiratory syndrome coro ....
7527 MW191256.1 |Severe acute respiratory syndrome coro ....
7528 MW191257.1 |Severe acute respiratory syndrome coro ....

7529 MW191258.1 |Severe acute respiratory syndrome coro ....
7530 MW191259.1 |Severe acute respiratory syndrome coro ....
7531 MW191260.1 |Severe acute respiratory syndrome coro ....
7532 MW191261.1 |Severe acute respiratory syndrome coro ....
7533 MW191262.1 |Severe acute respiratory syndrome coro ....
7534 MW191263.1 |Severe acute respiratory syndrome coro ....
7535 MW191264.1 |Severe acute respiratory syndrome coro ....
7536 MW191265.1 |Severe acute respiratory syndrome coro ....
7537 MW191266.1 |Severe acute respiratory syndrome coro ....
7538 MW191267.1 |Severe acute respiratory syndrome coro ....
7539 MW191268.1 |Severe acute respiratory syndrome coro ....
7540 MW191269.1 |Severe acute respiratory syndrome coro ....
7541 MW191270.1 |Severe acute respiratory syndrome coro ....
7542 MW191271.1 |Severe acute respiratory syndrome coro ....
7543 MW191272.1 |Severe acute respiratory syndrome coro ....
7544 MW191273.1 |Severe acute respiratory syndrome coro ....
7545 MW191274.1 |Severe acute respiratory syndrome coro ....
7546 MW191275.1 |Severe acute respiratory syndrome coro ....
7547 MW191276.1 |Severe acute respiratory syndrome coro ....
7548 MW191277.1 |Severe acute respiratory syndrome coro ....
7549 MW191278.1 |Severe acute respiratory syndrome coro ....
7550 MW191279.1 |Severe acute respiratory syndrome coro ....
7551 MW191280.1 |Severe acute respiratory syndrome coro ....
7552 MW191281.1 |Severe acute respiratory syndrome coro ....
7553 MW191282.1 |Severe acute respiratory syndrome coro ....
7554 MW191283.1 |Severe acute respiratory syndrome coro ....
7555 MW191284.1 |Severe acute respiratory syndrome coro ....
7556 MW191285.1 |Severe acute respiratory syndrome coro ....
7557 MW191286.1 |Severe acute respiratory syndrome coro ....
7558 MW191287.1 |Severe acute respiratory syndrome coro ....
7559 MW191288.1 |Severe acute respiratory syndrome coro ....
7560 MW191289.1 |Severe acute respiratory syndrome coro ....
7561 MW191290.1 |Severe acute respiratory syndrome coro ....
7562 MW191292.1 |Severe acute respiratory syndrome coro ....
7563 MW191293.1 |Severe acute respiratory syndrome coro ....
7564 MW191294.1 |Severe acute respiratory syndrome coro ....
7565 MW191295.1 |Severe acute respiratory syndrome coro ....
7566 MW191296.1 |Severe acute respiratory syndrome coro ....
7567 MW191297.1 |Severe acute respiratory syndrome coro ....
7568 MW191298.1 |Severe acute respiratory syndrome coro ....
7569 MW191299.1 |Severe acute respiratory syndrome coro ....
7570 MW191300.1 |Severe acute respiratory syndrome coro ....
7571 MW191301.1 |Severe acute respiratory syndrome coro ....
7572 MW191302.1 |Severe acute respiratory syndrome coro ....
7573 MW191303.1 |Severe acute respiratory syndrome coro ....
7574 MW191304.1 |Severe acute respiratory syndrome coro ....
7575 MW191305.1 |Severe acute respiratory syndrome coro ....
7576 MW191306.1 |Severe acute respiratory syndrome coro ....
7577 MW191307.1 |Severe acute respiratory syndrome coro ....
7578 MW191308.1 |Severe acute respiratory syndrome coro ....
7579 MW191309.1 |Severe acute respiratory syndrome coro ....
7580 MW191310.1 |Severe acute respiratory syndrome coro ....
7581 MW191311.1 |Severe acute respiratory syndrome coro ....
7582 MW191312.1 |Severe acute respiratory syndrome coro ....
7583 MW191313.1 |Severe acute respiratory syndrome coro ....
7584 MW191314.1 |Severe acute respiratory syndrome coro ....
7585 MW191315.1 |Severe acute respiratory syndrome coro ....
7586 MW191316.1 |Severe acute respiratory syndrome coro ....
7587 MW191317.1 |Severe acute respiratory syndrome coro ....
7588 MW191318.1 |Severe acute respiratory syndrome coro ....
7589 MW191319.1 |Severe acute respiratory syndrome coro ....
7590 MW191320.1 |Severe acute respiratory syndrome coro ....
7591 MW191321.1 |Severe acute respiratory syndrome coro ....
7592 MW191322.1 |Severe acute respiratory syndrome coro ....
7593 MW191323.1 |Severe acute respiratory syndrome coro ....
7594 MW191324.1 |Severe acute respiratory syndrome coro ....
7595 MW191325.1 |Severe acute respiratory syndrome coro ....
7596 MW191326.1 |Severe acute respiratory syndrome coro ....
7597 MW191327.1 |Severe acute respiratory syndrome coro ....
7598 MW191328.1 |Severe acute respiratory syndrome coro ....
7599 MW191329.1 |Severe acute respiratory syndrome coro ....
7600 MW191330.1 |Severe acute respiratory syndrome coro ....
7601 MW191331.1 |Severe acute respiratory syndrome coro ....
7602 MW191332.1 |Severe acute respiratory syndrome coro ....
7603 MW191333.1 |Severe acute respiratory syndrome coro ....
7604 MW191334.1 |Severe acute respiratory syndrome coro ....
7605 MW191335.1 |Severe acute respiratory syndrome coro ....
7606 MW191336.1 |Severe acute respiratory syndrome coro ....

```
7607 MW191337.1 |Severe acute respiratory syndrome coro    ....................  ....................  .....................  ....................  ....................  ....................
7608 MW191338.1 |Severe acute respiratory syndrome coro    ....................  ....................  .....................  ....................  ....................  ....................
7609 MW191339.1 |Severe acute respiratory syndrome coro    ....................  ....................  .....................  ....................  ....................  ....................
7610 MW191340.1 |Severe acute respiratory syndrome coro    ....................  ....................  .....................  ....................  ....................  ....................
7611 MW191341.1 |Severe acute respiratory syndrome coro    ....................  ....................  .....................  ....................  ....................  ....................
7612 MW191342.1 |Severe acute respiratory syndrome coro    ....................  ....................  .....................  ....................  ....................  ....................
7613 MW191343.1 |Severe acute respiratory syndrome coro    ....................  ....................  .....................  ....................  ....................  ....................
7614 MW191344.1 |Severe acute respiratory syndrome coro    ....................  ....................  .....................  ....................  ....................  ....................
7615 MW191345.1 |Severe acute respiratory syndrome coro    ....................  ....................  .....................  ....................  ....................  ....................
7616 MW191346.1 |Severe acute respiratory syndrome coro    ....................  ....................  .....................  ....................  ....................  ....................
7617 MW191347.1 |Severe acute respiratory syndrome coro    ....................  ....................  .....................  ....................  ....................  ....................
7618 MW191348.1 |Severe acute respiratory syndrome coro    ....................  ....................  .....................  ....................  ....................  ....................
7619 MW191349.1 |Severe acute respiratory syndrome coro    ....................  ....................  .....................  ....................  ....................  ....................
7620 MW191350.1 |Severe acute respiratory syndrome coro    ....................  ....................  .....................  ....................  ....................  ....................
7621 MW191351.1 |Severe acute respiratory syndrome coro    ....................  ....................  .....................  ....................  ....................  ....................
7622 MW191352.1 |Severe acute respiratory syndrome coro    ....................  ....................  .....................  ....................  ....................  ....................
7623 MW191353.1 |Severe acute respiratory syndrome coro    ....................  ....................  .....................  ....................  ....................  ....................
7624 MW191354.1 |Severe acute respiratory syndrome coro    ....................  ....................  .....................  ....................  ....................  ....................
7625 MW191355.1 |Severe acute respiratory syndrome coro    ....................  ....................  .....................  ....................  ....................  ....................
7626 MW191356.1 |Severe acute respiratory syndrome coro    ....................  ....................  .....................  ....................  ....................  ....................
7627 MW191357.1 |Severe acute respiratory syndrome coro    ....................  ....................  .....................  ....................  ....................  ....................
7628 MW191358.1 |Severe acute respiratory syndrome coro    ....................  ....................  .....................  ....................  ....................  ....................
7629 MW191359.1 |Severe acute respiratory syndrome coro    ....................  ....................  .....................  ....................  ....................  ....................
7630 MW191360.1 |Severe acute respiratory syndrome coro    ....................  ....................  .....................  ....................  ....................  ....................
7631 MW191361.1 |Severe acute respiratory syndrome coro    ....................  ....................  .....................  ....................  ....................  ....................
7632 MW191362.1 |Severe acute respiratory syndrome coro    ....................  ....................  .....................  ....................  ....................  ....................
7633 MW191363.1 |Severe acute respiratory syndrome coro    ....................  ....................  .....................  ....................  ....................  ....................
7634 MW191364.1 |Severe acute respiratory syndrome coro    ....................  ....................  .....................  ....................  ....................  ....................
7635 MW191365.1 |Severe acute respiratory syndrome coro    ....................  ....................  .....................  ....................  ....................  ....................
7636 MW191366.1 |Severe acute respiratory syndrome coro    ....................  ....................  .....................  ....................  ....................  ....................
7637 MW191367.1 |Severe acute respiratory syndrome coro    ....................  ....................  .....................  ....................  ....................  ....................
7638 MW191368.1 |Severe acute respiratory syndrome coro    ....................  ....................  .....................  ....................  ....................  ....................
7639 MW191369.1 |Severe acute respiratory syndrome coro    ....................  ....................  .....................  ....................  ....................  ....................
7640 MW191370.1 |Severe acute respiratory syndrome coro    ....................  ....................  .....................  ....................  ....................  ....................
7641 MW191371.1 |Severe acute respiratory syndrome coro    ....................  ....................  .....................  ....................  ....................  ....................
7642 MW191372.1 |Severe acute respiratory syndrome coro    ....................  ....................  .....................  ....................  ....................  ....................
7643 MW191373.1 |Severe acute respiratory syndrome coro    ....................  ....................  .....................  ....................  ....................  ....................
7644 MW191374.1 |Severe acute respiratory syndrome coro    ....................  ....................  .....................  ....................  ....................  ....................
7645 MW191375.1 |Severe acute respiratory syndrome coro    ....................  ....................  .....................  ....................  ....................  ....................
7646 MW191376.1 |Severe acute respiratory syndrome coro    ....................  ....................  .....................  ....................  ....................  ....................
7647 MW191377.1 |Severe acute respiratory syndrome coro    ....................  ....................  .....................  ....................  ....................  ....................
7648 MW191378.1 |Severe acute respiratory syndrome coro    ....................  ....................  .....................  ....................  ....................  ....................
7649 MW191379.1 |Severe acute respiratory syndrome coro    ....................  ....................  .....................  ....................  ....................  ....................
7650 MW191380.1 |Severe acute respiratory syndrome coro    ....................  ....................  .....................  ....................  ....................  ....................
7651 MW191381.1 |Severe acute respiratory syndrome coro    ....................  ....................  .....................  ....................  ....................  ....................
7652 MW191382.1 |Severe acute respiratory syndrome coro    ....................  ....................  .....................  ....................  ....................  ....................
7653 MW191383.1 |Severe acute respiratory syndrome coro    ....................  ....................  .....................  ....................  ....................  ....................
7654 MW191384.1 |Severe acute respiratory syndrome coro    ....................  ....................  .....................  ....................  ....................  ....................
7655 MW191385.1 |Severe acute respiratory syndrome coro    ....................  ....................  .....................  ....................  ....................  ....................
7656 MW191386.1 |Severe acute respiratory syndrome coro    ....................  ....................  .....................  ....................  ....................  ....................
7657 MW191387.1 |Severe acute respiratory syndrome coro    ....................  ....................  .....................  ....................  ....................  ....................
7658 MW191388.1 |Severe acute respiratory syndrome coro    ....................  ....................  .....................  ....................  ....................  ....................
7659 MW191389.1 |Severe acute respiratory syndrome coro    ....................  ....................  .....................  ....................  ....................  ....................
7660 MW191390.1 |Severe acute respiratory syndrome coro    ....................  ....................  .....................  ....................  ....................  ....................
7661 MW191391.1 |Severe acute respiratory syndrome coro    ....................  ....................  .....................  ....................  ....................  ....................
7662 MW191392.1 |Severe acute respiratory syndrome coro    ....................  ....................  .....................  ....................  ....................  ....................
7663 MW191393.1 |Severe acute respiratory syndrome coro    ....................  ....................  .....................  ....................  ....................  ....................
7664 MW191394.1 |Severe acute respiratory syndrome coro    ....................  ....................  .....................  ....................  ....................  ....................
7665 MW191395.1 |Severe acute respiratory syndrome coro    ....................  ....................  .....................  ....................  ....................  ....................
7666 MW191396.1 |Severe acute respiratory syndrome coro    ....................  ....................  .....................  ....................  ....................  ....................
7667 MW191397.1 |Severe acute respiratory syndrome coro    ....................  ....................  .....................  ....................  ....................  ....................
7668 MW191398.1 |Severe acute respiratory syndrome coro    ....................  ....................  .....................  ....................  ....................  ....................
7669 MW191399.1 |Severe acute respiratory syndrome coro    ....................  ....................  .....................  ....................  ....................  ....................
7670 MW191400.1 |Severe acute respiratory syndrome coro    ....................  ....................  .....................  ....................  ....................  ....................
7671 MW191401.1 |Severe acute respiratory syndrome coro    ....................  ....................  .....................  ....................  ....................  ....................
7672 MW191402.1 |Severe acute respiratory syndrome coro    ....................  ....................  .....................  ....................  ....................  ....................
7673 MW191403.1 |Severe acute respiratory syndrome coro    ....................  ....................  .....................  ....................  ....................  ....................
7674 MW191404.1 |Severe acute respiratory syndrome coro    ....................  ....................  .....................  ....................  ....................  ....................
7675 MW191405.1 |Severe acute respiratory syndrome coro    ....................  ....................  .....................  ....................  ....................  ....................
7676 MW191406.1 |Severe acute respiratory syndrome coro    ....................  ....................  .....................  ....................  ....................  ....................
7677 MW191407.1 |Severe acute respiratory syndrome coro    ....................  ....................  .....................  ....................  ....................  ....................
7678 MW191408.1 |Severe acute respiratory syndrome coro    ....................  ....................  .....................  ....................  ....................  ....................
7679 MW191409.1 |Severe acute respiratory syndrome coro    ....................  ....................  .....................  ....................  ....................  ....................
7680 MW191410.1 |Severe acute respiratory syndrome coro    ....................  ....................  .....................  ....................  ....................  ....................
7681 MW191411.1 |Severe acute respiratory syndrome coro    ....................  ....................  .....................  ....................  ....................  ....................
7682 MW191412.1 |Severe acute respiratory syndrome coro    ....................  ....................  .....................  ....................  ....................  ....................
7683 MW191413.1 |Severe acute respiratory syndrome coro    ....................  ....................  .....................  ....................  ....................  ....................
7684 MW191414.1 |Severe acute respiratory syndrome coro    ....................  ....................  .....................  ....................  ....................  ....................
```

```
7685 MW191415.1 |Severe acute respiratory syndrome coro    ....................  ....................  .....................  ....................  ....................  ....................
7686 MW191416.1 |Severe acute respiratory syndrome coro    ....................  ....................  .....................  ....................  ....................  ....................
7687 MW191417.1 |Severe acute respiratory syndrome coro    ....................  ....................  .....................  ....................  ....................  ....................
7688 MW191418.1 |Severe acute respiratory syndrome coro    ....................  ....................  .....................  ....................  ....................  ....................
7689 MW191419.1 |Severe acute respiratory syndrome coro    ....................  ....................  .....................  ....................  ....................  ....................
7690 MW191420.1 |Severe acute respiratory syndrome coro    ....................  ....................  .....................  ....................  ....................  ....................
7691 MW191421.1 |Severe acute respiratory syndrome coro    ....................  ....................  .....................  ....................  ....................  ....................
7692 MW191422.1 |Severe acute respiratory syndrome coro    ....................  ....................  .....................  ....................  ....................  ....................
7693 MW191423.1 |Severe acute respiratory syndrome coro    ....................  ....................  .....................  ....................  ....................  ....................
7694 MW191424.1 |Severe acute respiratory syndrome coro    ....................  ....................  .....................  ....................  ....................  ....................
7695 MW191425.1 |Severe acute respiratory syndrome coro    ....................  ....................  .....................  ....................  ....................  ....................
7696 MW191426.1 |Severe acute respiratory syndrome coro    ....................  ....................  .....................  ....................  ....................  ....................
7697 MW191427.1 |Severe acute respiratory syndrome coro    ....................  ....................  .....................  ....................  ....................  ....................
7698 MW191428.1 |Severe acute respiratory syndrome coro    ....................  ....................  .....................  ....................  ....................  ....................
7699 MW191429.1 |Severe acute respiratory syndrome coro    ....................  ....................  .....................  ....................  ....................  ....................
7700 MW191430.1 |Severe acute respiratory syndrome coro    ....................  ....................  .....................  ....................  ....................  ....................
7701 MW191431.1 |Severe acute respiratory syndrome coro    ....................  ....................  .....................  ....................  ....................  ....................
7702 MW191432.1 |Severe acute respiratory syndrome coro    ....................  .A..................  .....................  ....................  ....................  ....................
7703 MW191433.1 |Severe acute respiratory syndrome coro    ....................  ....................  .....................  ....................  ....................  ....................
7704 MW191434.1 |Severe acute respiratory syndrome coro    ....................  ....................  .....................  ....................  ....................  ....................
7705 MW191435.1 |Severe acute respiratory syndrome coro    ....................  ....................  .....................  ....................  ....................  ....................
7706 MW191436.1 |Severe acute respiratory syndrome coro    ....................  ....................  .....................  ....................  ....................  ....................
7707 MW191437.1 |Severe acute respiratory syndrome coro    ....................  ....................  .....................  ....................  ....................  ....................
7708 MW191438.1 |Severe acute respiratory syndrome coro    ....................  ....................  .....................  ....................  ....................  ....................
7709 MW191439.1 |Severe acute respiratory syndrome coro    ....................  ....................  .....................  ....................  ....................  ....................
7710 MW191440.1 |Severe acute respiratory syndrome coro    ....................  ....................  .....................  ....................  ....................  ....................
7711 MW191441.1 |Severe acute respiratory syndrome coro    ....................  ....................  .....................  ....................  ....................  ....................
7712 MW191442.1 |Severe acute respiratory syndrome coro    ....................  ....................  .....................  ....................  ....................  ....................
7713 MW191443.1 |Severe acute respiratory syndrome coro    ....................  ....................  .....................  ....................  ....................  ....................
7714 MW191444.1 |Severe acute respiratory syndrome coro    ....................  ....................  .....................  ....................  ....................  ....................
7715 MW191445.1 |Severe acute respiratory syndrome coro    ....................  ....................  .....................  ....................  ....................  ....................
7716 MW191446.1 |Severe acute respiratory syndrome coro    ....................  ....................  .....................  ....................  ....................  ....................
7717 MW191447.1 |Severe acute respiratory syndrome coro    ....................  ....................  .....................  ....................  ....................  ....................
7718 MW191448.1 |Severe acute respiratory syndrome coro    ....................  ....................  .....................  ....................  ....................  ....................
7719 MW191449.1 |Severe acute respiratory syndrome coro    ....................  ....................  .....................  ....................  ....................  ....................
7720 MW191450.1 |Severe acute respiratory syndrome coro    ....................  ....................  .....................  ....................  ....................  ....................
7721 MW191452.1 |Severe acute respiratory syndrome coro    ....................  ....................  .....................  ....................  ....................  ....................
7722 MW191453.1 |Severe acute respiratory syndrome coro    ....................  ....................  .....................  ....................  ....................  ....................
7723 MW191454.1 |Severe acute respiratory syndrome coro    ....................  ....................  .....................  ....................  ....................  ....................
7724 MW191455.1 |Severe acute respiratory syndrome coro    ....................  ....................  .....................  ....................  ....................  ....................
7725 MW191456.1 |Severe acute respiratory syndrome coro    ....................  ....................  .....................  ....................  ....................  ....................
7726 MW191457.1 |Severe acute respiratory syndrome coro    ....................  ....................  .....................  ....................  ....................  ....................
7727 MW191458.1 |Severe acute respiratory syndrome coro    ....................  ....................  .....................  ....................  ....................  ....................
7728 MW191459.1 |Severe acute respiratory syndrome coro    ....................  ....................  .....................  ....................  ....................  ....................
7729 MW191460.1 |Severe acute respiratory syndrome coro    ....................  ....................  .....................  ....................  ....................  ....................
7730 MW191461.1 |Severe acute respiratory syndrome coro    ....................  ....................  .....................  ....................  ....................  ....................
7731 MW191462.1 |Severe acute respiratory syndrome coro    ....................  ....................  .....................  ....................  ....................  ....................
7732 MW191463.1 |Severe acute respiratory syndrome coro    ....................  ....................  .....................  ....................  ....................  ....................
7733 MW191464.1 |Severe acute respiratory syndrome coro    ....................  ....................  .....................  ....................  ....................  ....................
7734 MW191465.1 |Severe acute respiratory syndrome coro    ....................  ....................  .....................  ....................  ....................  ....................
7735 MW191466.1 |Severe acute respiratory syndrome coro    ....................  ....................  .....................  ....................  ....................  ....................
7736 MW191467.1 |Severe acute respiratory syndrome coro    ....................  ....................  .....................  ....................  ....................  ....................
7737 MW191468.1 |Severe acute respiratory syndrome coro    ....................  ....................  .....................  ....................  ....................  ....................
7738 MW191469.1 |Severe acute respiratory syndrome coro    ....................  ....................  .....................  ....................  ....................  ....................
7739 MW191470.1 |Severe acute respiratory syndrome coro    ....................  ....................  .....................  ....................  ....................  ....................
7740 MW191471.1 |Severe acute respiratory syndrome coro    ....................  ....................  .....................  ....................  ....................  ....................
7741 MW191472.1 |Severe acute respiratory syndrome coro    ....................  ....................  .....................  ....................  ....................  ....................
7742 MW191473.1 |Severe acute respiratory syndrome coro    ....................  ....................  .....................  ....................  ....................  ....................
7743 MW191474.1 |Severe acute respiratory syndrome coro    ....................  ....................  .....................  ....................  ....................  ....................
7744 MW191475.1 |Severe acute respiratory syndrome coro    ....................  ....................  .....................  ....................  ....................  ....................
7745 MW191476.1 |Severe acute respiratory syndrome coro    ....................  ....................  .....................  ....................  ....................  ....................
7746 MW191477.1 |Severe acute respiratory syndrome coro    ....................  ....................  .....................  ....................  ....................  ....................
7747 MW191478.1 |Severe acute respiratory syndrome coro    ....................  ....................  .....................  ....................  ....................  ....................
7748 MW191479.1 |Severe acute respiratory syndrome coro    ....................  ....................  .....................  ....................  ....................  ....................
7749 MW191480.1 |Severe acute respiratory syndrome coro    ....................  ....................  .....................  ....................  ....................  ....................
7750 MW191481.1 |Severe acute respiratory syndrome coro    ....................  ....................  .....................  ....................  ....................  ....................
7751 MW191482.1 |Severe acute respiratory syndrome coro    ....................  ....................  .....................  ....................  ....................  ....................
7752 MW191483.1 |Severe acute respiratory syndrome coro    ....................  ....................  .....................  ....................  ....................  ....................
7753 MW191484.1 |Severe acute respiratory syndrome coro    ....................  ....................  .....................  ....................  ....................  ....................
7754 MW191485.1 |Severe acute respiratory syndrome coro    ....................  ....................  .....................  ....................  ....................  ....................
7755 MW191486.1 |Severe acute respiratory syndrome coro    ....................  ....................  .....................  ....................  ....................  ....................
7756 MW191487.1 |Severe acute respiratory syndrome coro    ....................  ....................  .....................  ....................  ....................  ....................
7757 MW191488.1 |Severe acute respiratory syndrome coro    ....................  ....................  .....................  ....................  ....................  ....................
7758 MW191489.1 |Severe acute respiratory syndrome coro    ....................  ....................  .....................  ....................  ....................  ....................
7759 MW191490.1 |Severe acute respiratory syndrome coro    ....................  ....................  .....................  ....................  ....................  ....................
7760 MW191491.1 |Severe acute respiratory syndrome coro    ....................  ....................  .....................  ....................  ....................  ....................
7761 MW191492.1 |Severe acute respiratory syndrome coro    ....................  ....................  .....................  ....................  ....................  ....................
7762 MW191501.1 |Severe acute respiratory syndrome coro    ....................  ....................  .....................  ....................  ....................  ....................
```

7763	MW191509.1	|Severe acute respiratory syndrome coro	····················	····················	····················	····················	····················	····················
7764	MW191510.1	|Severe acute respiratory syndrome coro	····················	····················	····················	····················	····················	····················
7765	MW191511.1	|Severe acute respiratory syndrome coro	····················	····················	····················	····················	····················	····················
7766	MW191512.1	|Severe acute respiratory syndrome coro	····················	····················	····················	····················	····················	····················
7768	MW192064.1	|Severe acute respiratory syndrome coro	····················	····················	····················	····················	····················	····················
7769	MW192065.1	|Severe acute respiratory syndrome coro	····················	····················	····················	····················	····················	····················
7770	MW192066.1	|Severe acute respiratory syndrome coro	····················	····················	····················	····················	····················	····················
7771	MW192067.1	|Severe acute respiratory syndrome coro	····················	·············A······	····················	····················	····················	····················
7772	MW192068.1	|Severe acute respiratory syndrome coro	····················	····················	····················	····················	····················	····················
7773	MW192069.1	|Severe acute respiratory syndrome coro	····················	····················	····················	····················	····················	····················
7774	MW192070.1	|Severe acute respiratory syndrome coro	····················	····················	····················	····················	····················	····················
7775	MW192071.1	|Severe acute respiratory syndrome coro	····················	····················	····················	····················	····················	····················
7776	MW192072.1	|Severe acute respiratory syndrome coro	····················	····················	····················	····················	····················	····················
7777	MW192073.1	|Severe acute respiratory syndrome coro	····················	····················	····················	····················	····················	····················
7778	MW192074.1	|Severe acute respiratory syndrome coro	····················	····················	····················	····················	····················	····················
7779	MW192075.1	|Severe acute respiratory syndrome coro	····················	····················	····················	····················	····················	····················
7780	MW192076.1	|Severe acute respiratory syndrome coro	····················	····················	····················	····················	····················	····················
7781	MW192077.1	|Severe acute respiratory syndrome coro	····················	····················	····················	····················	·N··················	····················
7782	MW192078.1	|Severe acute respiratory syndrome coro	····················	·············A······	····················	····················	····················	····················
7783	MW192079.1	|Severe acute respiratory syndrome coro	····················	····················	····················	····················	····················	····················
7784	MW192080.1	|Severe acute respiratory syndrome coro	····················	····················	····················	····················	····················	····················
7785	MW192081.1	|Severe acute respiratory syndrome coro	····················	····················	····················	····················	····················	····················
7786	MW192082.1	|Severe acute respiratory syndrome coro	····················	····················	····················	····················	····················	····················
7787	MW192083.1	|Severe acute respiratory syndrome coro	····················	·············A······	····················	····················	····················	····················
7788	MW192084.1	|Severe acute respiratory syndrome coro	····················	····················	····················	····················	····················	····················
7789	MW192085.1	|Severe acute respiratory syndrome coro	····················	····················	····················	····················	····················	····················
7790	MW192086.1	|Severe acute respiratory syndrome coro	····················	····················	····················	····················	····················	····················
7791	MW192087.1	|Severe acute respiratory syndrome coro	····················	·A··················	····················	····················	····················	····················
7792	MW192088.1	|Severe acute respiratory syndrome coro	····················	····················	····················	····················	····················	····················
7793	MW192089.1	|Severe acute respiratory syndrome coro	····················	·A··················	····················	····················	····················	····················
7794	MW192090.1	|Severe acute respiratory syndrome coro	····················	····················	····················	····················	····················	····················
7795	MW192091.1	|Severe acute respiratory syndrome coro	····················	·A··················	····················	····················	····················	····················
7796	MW192092.1	|Severe acute respiratory syndrome coro	····················	····················	····················	····················	····················	····················
7797	MW192093.1	|Severe acute respiratory syndrome coro	····················	·A··················	····················	····················	····················	····················
7798	MW192094.1	|Severe acute respiratory syndrome coro	····················	·A··················	····················	····················	····················	····················
7799	MW192095.1	|Severe acute respiratory syndrome coro	····················	·A··················	····················	····················	····················	····················
7800	MW192096.1	|Severe acute respiratory syndrome coro	····················	·A··················	····················	····················	····················	····················
7801	MW192097.1	|Severe acute respiratory syndrome coro	····················	·A··················	····················	····················	····················	····················
7802	MW192098.1	|Severe acute respiratory syndrome coro	····················	·A··················	····················	····················	····················	····················
7803	MW192099.1	|Severe acute respiratory syndrome coro	····················	····················	····················	····················	····················	····················
7804	MW192100.1	|Severe acute respiratory syndrome coro	····················	····················	····················	····················	····················	····················
7805	MW192101.1	|Severe acute respiratory syndrome coro	····················	····················	····················	····················	····················	····················
7806	MW192102.1	|Severe acute respiratory syndrome coro	····················	·A··················	····················	····················	····················	····················
7807	MW192103.1	|Severe acute respiratory syndrome coro	····················	·A··················	····················	····················	····················	····················
7808	MW192104.1	|Severe acute respiratory syndrome coro	····················	·A··················	····················	····················	····················	····················
7809	MW192105.1	|Severe acute respiratory syndrome coro	····················	·············A······	····················	····················	····················	····················
7810	MW192106.1	|Severe acute respiratory syndrome coro	····················	·············A······	····················	····················	····················	····················
7811	MW192107.1	|Severe acute respiratory syndrome coro	····················	····················	····················	····················	····················	····················
7812	MW192108.1	|Severe acute respiratory syndrome coro	····················	····················	····················	····················	····················	····················
7813	MW192109.1	|Severe acute respiratory syndrome coro	····················	····················	····················	····················	····················	····················
7814	MW192110.1	|Severe acute respiratory syndrome coro	····················	····················	····················	····················	····················	····················
7815	MW192111.1	|Severe acute respiratory syndrome coro	····················	····················	····················	····················	····················	····················
7816	MW192112.1	|Severe acute respiratory syndrome coro	····················	····················	····················	····················	····················	····················
7817	MW192113.1	|Severe acute respiratory syndrome coro	····················	·A··················	····················	····················	····················	····················
7818	MW192114.1	|Severe acute respiratory syndrome coro	····················	·A··················	····················	····················	····················	····················
7819	MW192115.1	|Severe acute respiratory syndrome coro	····················	····················	····················	····················	····················	····················
7820	MW192116.1	|Severe acute respiratory syndrome coro	····················	····················	····················	····················	····················	····················
7821	MW192117.1	|Severe acute respiratory syndrome coro	····················	····················	····················	····················	····················	····················
7822	MW192118.1	|Severe acute respiratory syndrome coro	····················	····················	····················	····················	····················	····················
7823	MW192119.1	|Severe acute respiratory syndrome coro	····················	·A··················	····················	····················	····················	····················
7824	MW192120.1	|Severe acute respiratory syndrome coro	····················	·A··················	····················	····················	····················	····················
7825	MW192121.1	|Severe acute respiratory syndrome coro	····················	·A··················	····················	····················	····················	····················
7826	MW192122.1	|Severe acute respiratory syndrome coro	····················	····················	····················	····················	····················	····················
7827	MW192123.1	|Severe acute respiratory syndrome coro	····················	·A··················	····················	····················	····················	····················
7828	MW192124.1	|Severe acute respiratory syndrome coro	····················	·A··················	····················	····················	····················	····················
7829	MW192125.1	|Severe acute respiratory syndrome coro	····················	····················	····················	····················	····················	····················
7830	MW192126.1	|Severe acute respiratory syndrome coro	····················	····················	····················	····················	·NN·················	····················
7831	MW192127.1	|Severe acute respiratory syndrome coro	····················	····················	····················	····················	····················	····················
7832	MW192766.1	|Severe acute respiratory syndrome coro	····················	····················	····················	····················	····················	····················
7833	MW192771.1	|Severe acute respiratory syndrome coro	····················	····················	····················	····················	····················	····················
7834	MW192772.1	|Severe acute respiratory syndrome coro	····················	····················	····················	····················	····················	····················
7835	MW186669.1	|Severe acute respiratory syndrome coro	····················	····················	····················	····················	····················	····················
7836	MW181434.1	|Severe acute respiratory syndrome coro	····················	····················	····················	····················	····················	····················
7837	MW181442.1	|Severe acute respiratory syndrome coro	····················	····················	····················	····················	····················	····················
7838	MW181459.1	|Severe acute respiratory syndrome coro	····················	····················	····················	····················	····················	····················
7839	MW181466.1	|Severe acute respiratory syndrome coro	····················	····················	····················	····················	····················	····················
7840	MW181473.1	|Severe acute respiratory syndrome coro	····················	····················	····················	····················	····················	····················
7841	MW181474.1	|Severe acute respiratory syndrome coro	····················	····················	····················	····················	····················	····················

7842 MW181500.1 |Severe acute respiratory syndrome coro ···················· ···················· ···················· ···················· ···················· ····················
7843 MW181505.1 |Severe acute respiratory syndrome coro ···················· ···················· ···················· ···················· ···················· ····················
7844 MW181518.1 |Severe acute respiratory syndrome coro ···················· ···················· ···················· ···················· ···················· ····················
7845 MW181520.1 |Severe acute respiratory syndrome coro ···················· ···················· ···················· ···················· ···················· ····················
7846 MW181526.1 |Severe acute respiratory syndrome coro ···················· ···················· ···················· ···················· ···················· ····················
7847 MW181529.1 |Severe acute respiratory syndrome coro ···················· ···················· ···················· ···················· ···················· ····················
7848 MW181537.1 |Severe acute respiratory syndrome coro ···················· ···················· ···················· ···················· ···················· ····················
7849 MW181549.1 |Severe acute respiratory syndrome coro ···················· ···················· ···················· ···················· ···················· ····················
7850 MW181562.1 |Severe acute respiratory syndrome coro ···················· ···················· ···················· ···················· ···················· ····················
7851 MW181565.1 |Severe acute respiratory syndrome coro ···················· ·A·················· ···················· ···················· ···················· ····················
7852 MW181566.1 |Severe acute respiratory syndrome coro ···················· ···················· ···················· ···················· ···················· ····················
7853 MW181574.1 |Severe acute respiratory syndrome coro ···················· ···················· ···················· ···················· ···················· ····················
7854 MW181575.1 |Severe acute respiratory syndrome coro ···················· ·············A······ ···················· ···················· ···················· ····················
7855 MW181576.1 |Severe acute respiratory syndrome coro ···················· ·A·················· ···················· ···················· ···················· ····················
7856 MW181577.1 |Severe acute respiratory syndrome coro ···················· ···················· ···················· ···················· ···················· ····················
7857 MW181578.1 |Severe acute respiratory syndrome coro ···················· ···················· ···················· ···················· ···················· ····················
7858 MW181579.1 |Severe acute respiratory syndrome coro ···················· ···················· ···················· ···················· ···················· ····················
7859 MW181580.1 |Severe acute respiratory syndrome coro ···················· ···················· ···················· ···················· ···················· ····················
7860 MW181581.1 |Severe acute respiratory syndrome coro ···················· ···················· ···················· ···················· ···················· ····················
7861 MW181582.1 |Severe acute respiratory syndrome coro ···················· ···················· ···················· ···················· ···················· ····················
7862 MW181583.1 |Severe acute respiratory syndrome coro ···················· ···················· ···················· ···················· ···················· ····················
7863 MW181584.1 |Severe acute respiratory syndrome coro ···················· ···················· ···················· ···················· ···················· ····················
7864 MW181585.1 |Severe acute respiratory syndrome coro ···················· ···················· ···················· ···················· ···················· ····················
7865 MW181586.1 |Severe acute respiratory syndrome coro ···················· ···················· ···················· ···················· ···················· ····················
7866 MW181587.1 |Severe acute respiratory syndrome coro ···················· ············C······· ···················· ···················· ···················· ····················
7867 MW181588.1 |Severe acute respiratory syndrome coro ···················· ···················· ···················· ···················· ···················· ····················
7868 MW181589.1 |Severe acute respiratory syndrome coro ···················· ···················· ···················· ···················· ···················· ····················
7869 MW181590.1 |Severe acute respiratory syndrome coro ···················· ···················· ···················· ···················· ···················· ····················
7870 MW181591.1 |Severe acute respiratory syndrome coro ···················· ···················· ···················· ···················· ···················· ····················
7871 MW181592.1 |Severe acute respiratory syndrome coro ···················· ···················· ···················· ···················· ···················· ····················
7872 MW181593.1 |Severe acute respiratory syndrome coro ···················· ·A·················· ···················· ···················· ···················· ····················
7873 MW181621.1 |Severe acute respiratory syndrome coro ···················· ···················· ···················· ···················· ···················· ····················
7874 MW181771.1 |Severe acute respiratory syndrome coro ···················· ···················· ···················· ···················· ···················· ····················
7875 MW181772.1 |Severe acute respiratory syndrome coro ···················· ···················· ···················· ···················· ···················· ····················
7876 MW181773.1 |Severe acute respiratory syndrome coro ···················· ···················· ···················· ···················· ···················· ····················
7877 MW181797.1 |Severe acute respiratory syndrome coro ···················· ···················· ···················· ···················· ···················· ····················
7878 MW181803.1 |Severe acute respiratory syndrome coro ···················· ···················· ···················· ···················· ···················· ····················
7879 MW181804.1 |Severe acute respiratory syndrome coro ···················· ···················· ···················· ···················· ···················· ····················
7880 MW181805.1 |Severe acute respiratory syndrome coro ···················· ···················· ···················· ···················· ···················· ····················
7881 MW181806.1 |Severe acute respiratory syndrome coro ···················· ···················· ···················· ···················· ···················· ····················
7882 MW181807.1 |Severe acute respiratory syndrome coro ···················· ···················· ···················· ···················· ···················· ····················
7883 MW181811.1 |Severe acute respiratory syndrome coro ···················· ···················· ···················· ···················· ···················· ····················
7884 MW181814.1 |Severe acute respiratory syndrome coro ···················· ···················· ···················· ···················· ···················· ····················
7885 MW181828.1 |Severe acute respiratory syndrome coro ···················· ···················· ···················· ···················· ···················· ····················
7886 MW181829.1 |Severe acute respiratory syndrome coro ···················· ···················· ···················· ···················· ···················· ····················
7887 MW181831.2 |Severe acute respiratory syndrome coro ···················· ···················· ···················· ···················· ···················· ····················
7888 MW181832.1 |Severe acute respiratory syndrome coro ···················· ···················· ···················· ···················· ···················· ····················
7889 MW181833.1 |Severe acute respiratory syndrome coro ···················· ···················· ···················· ···················· ···················· ····················
7890 MW181834.1 |Severe acute respiratory syndrome coro ···················· ···················· ···················· ···················· ···················· ····················
7891 MW181835.1 |Severe acute respiratory syndrome coro ···················· ···················· ···················· ···················· ···················· ····················
7892 MW181837.1 |Severe acute respiratory syndrome coro ···················· ···················· ···················· ···················· ···················· ····················
7893 MW181838.2 |Severe acute respiratory syndrome coro ···················· ···················· ···················· ···················· ···················· ····················
7894 MW181839.1 |Severe acute respiratory syndrome coro ···················· ···················· ···················· ···················· ···················· ····················
7895 MW181840.1 |Severe acute respiratory syndrome coro ···················· ···················· ···················· ···················· ···················· ····················
7896 MW181843.1 |Severe acute respiratory syndrome coro ···················· ···················· ···················· ···················· ···················· ····················
7897 MW181844.1 |Severe acute respiratory syndrome coro ···················· ···················· ···················· ···················· ···················· ····················
7898 MW181845.1 |Severe acute respiratory syndrome coro ···················· ···················· ···················· ···················· ···················· ····················
7899 MW181846.1 |Severe acute respiratory syndrome coro ···················· ···················· ···················· ···················· ···················· ····················
7900 MW181847.1 |Severe acute respiratory syndrome coro ···················· ···················· ···················· ···················· ···················· ····················
7901 MW181848.1 |Severe acute respiratory syndrome coro ···················· ···················· ···················· ···················· ···················· ····················
7902 MW183931.1 |Severe acute respiratory syndrome coro ···················· ···················· ···················· ···················· ···················· ····················
7903 MW183932.1 |Severe acute respiratory syndrome coro ···················· ···················· ···················· ···················· ···················· ····················
7904 MW183934.1 |Severe acute respiratory syndrome coro ···················· ···················· ···················· ···················· ···················· ····················
7905 MW183935.1 |Severe acute respiratory syndrome coro ···················· ···················· ···················· ···················· ···················· ····················
7906 MW183936.1 |Severe acute respiratory syndrome coro ···················· ···················· ···················· ···················· ···················· ····················
7907 MW183938.1 |Severe acute respiratory syndrome coro ···················· ···················· ···················· ···················· ···················· ····················
7908 MW183939.1 |Severe acute respiratory syndrome coro ···················· ···················· ···················· ···················· ···················· ····················
7909 MW183941.1 |Severe acute respiratory syndrome coro ···················· ···················· ···················· ···················· ···················· ····················
7910 MW183942.1 |Severe acute respiratory syndrome coro ···················· ···················· ···················· ···················· ···················· ····················
7911 MW183943.1 |Severe acute respiratory syndrome coro ···················· ···················· ···················· ···················· ···················· ····················
7912 MW183944.1 |Severe acute respiratory syndrome coro ···················· ···················· ···················· ···················· ···················· ····················
7913 MW183945.1 |Severe acute respiratory syndrome coro ···················· ···················· ···················· ···················· ···················· ····················
7914 MW183946.1 |Severe acute respiratory syndrome coro ···················· ···················· ···················· ···················· ···················· ····················
7915 MW183947.1 |Severe acute respiratory syndrome coro ···················· ···················· ···················· ···················· ···················· ····················
7917 MW183951.1 |Severe acute respiratory syndrome coro ···················· ···················· ···················· ···················· ···················· ····················
7918 MW183952.1 |Severe acute respiratory syndrome coro ···················· ···················· ···················· ···················· ···················· ····················
7919 MW183953.1 |Severe acute respiratory syndrome coro ···················· ···················· ···················· ···················· ···················· ····················
7920 MW183956.1 |Severe acute respiratory syndrome coro ···················· ···················· ···················· ···················· ···················· ····················

7921	MW183957.1	|Severe acute respiratory syndrome coro	····················	····················	····················	····················	····················	····················	
7922	MW183959.1	|Severe acute respiratory syndrome coro	····················	····················	····················	····················	····················	····················	
7923	MW183960.1	|Severe acute respiratory syndrome coro	····················	····················	····················	····················	·NNN················	····················	1
7924	MW183961.1	|Severe acute respiratory syndrome coro	····················	····················	····················	····················	····················	····················	
7925	MW183962.1	|Severe acute respiratory syndrome coro	····················	····················	····················	····················	····················	····················	
7926	MW183963.1	|Severe acute respiratory syndrome coro	····················	····················	····················	····················	····················	····················	
7927	MW183964.1	|Severe acute respiratory syndrome coro	····················	····················	····················	····················	····················	····················	
7928	MW183965.1	|Severe acute respiratory syndrome coro	····················	····················	····················	····················	····················	····················	
7929	MW183966.1	|Severe acute respiratory syndrome coro	····················	····················	····················	····················	····················	····················	
7931	MW183970.1	|Severe acute respiratory syndrome coro	····················	····················	····················	····················	····················	····················	
7932	MW183971.1	|Severe acute respiratory syndrome coro	····················	····················	····················	····················	····················	····················	
7933	MW183972.1	|Severe acute respiratory syndrome coro	····················	····················	····················	····················	····················	····················	
7934	MW183975.1	|Severe acute respiratory syndrome coro	····················	····················	····················	····················	····················	····················	
7935	MW183976.1	|Severe acute respiratory syndrome coro	····················	····················	····················	····················	····················	····················	
7936	MW183977.1	|Severe acute respiratory syndrome coro	····················	····················	····················	····················	····················	····················	
7937	MW183978.1	|Severe acute respiratory syndrome coro	····················	····················	····················	····················	····················	····················	
7938	MW183980.1	|Severe acute respiratory syndrome coro	····················	····················	····················	····················	····················	····················	
7939	MW183981.1	|Severe acute respiratory syndrome coro	····················	····················	····················	····················	····················	····················	
7940	MW183982.1	|Severe acute respiratory syndrome coro	····················	····················	····················	····················	····················	····················	
7941	MW183983.1	|Severe acute respiratory syndrome coro	····················	····················	····················	····················	····················	····················	
7942	MW183984.1	|Severe acute respiratory syndrome coro	····················	····················	····················	····················	····················	····················	
7943	MW183985.1	|Severe acute respiratory syndrome coro	····················	····················	····················	····················	····················	····················	
7944	MW183986.1	|Severe acute respiratory syndrome coro	····················	····················	····················	····················	····················	····················	
7945	MW183987.1	|Severe acute respiratory syndrome coro	····················	····················	····················	····················	····················	····················	
7946	MW183988.1	|Severe acute respiratory syndrome coro	····················	····················	····················	····················	····················	····················	
7947	MW183989.1	|Severe acute respiratory syndrome coro	····················	····················	····················	····················	····················	····················	
7948	MW183990.1	|Severe acute respiratory syndrome coro	····················	····················	····················	····················	····················	····················	
7950	MW183994.1	|Severe acute respiratory syndrome coro	····················	····················	····················	····················	····················	····················	
7951	MW183995.1	|Severe acute respiratory syndrome coro	····················	····················	····················	····················	····················	····················	
7952	MW183996.1	|Severe acute respiratory syndrome coro	····················	····················	····················	····················	····················	····················	
7953	MW183997.1	|Severe acute respiratory syndrome coro	····················	····················	····················	····················	····················	····················	
7954	MW183998.1	|Severe acute respiratory syndrome coro	····················	····················	····················	····················	····················	····················	
7955	MW183999.1	|Severe acute respiratory syndrome coro	····················	····················	····················	····················	····················	····················	
7956	MW184000.1	|Severe acute respiratory syndrome coro	····················	····················	····················	····················	····················	····················	
7957	MW184001.1	|Severe acute respiratory syndrome coro	····················	····················	····················	····················	····················	····················	
7958	MW184002.1	|Severe acute respiratory syndrome coro	····················	····················	····················	····················	····················	····················	
7959	MW184003.1	|Severe acute respiratory syndrome coro	····················	····················	····················	····················	····················	····················	
7960	MW184004.1	|Severe acute respiratory syndrome coro	····················	····················	····················	····················	····················	····················	
7962	MW184007.1	|Severe acute respiratory syndrome coro	····················	····················	····················	····················	····················	····················	
7963	MW184009.1	|Severe acute respiratory syndrome coro	····················	····················	····················	····················	····················	····················	
7964	MW184010.1	|Severe acute respiratory syndrome coro	····················	····················	····················	····················	····················	····················	
7965	MW184013.1	|Severe acute respiratory syndrome coro	····················	····················	····················	····················	····················	····················	
7966	MW184014.1	|Severe acute respiratory syndrome coro	····················	····················	····················	····················	····················	····················	
7967	MW184015.1	|Severe acute respiratory syndrome coro	····················	····················	····················	····················	····················	····················	
7968	MW184016.1	|Severe acute respiratory syndrome coro	····················	····················	····················	····················	····················	····················	
7969	MW184019.1	|Severe acute respiratory syndrome coro	····················	····················	····················	····················	····················	····················	
7970	MW184020.1	|Severe acute respiratory syndrome coro	····················	····················	····················	····················	····················	····················	
7971	MW184021.1	|Severe acute respiratory syndrome coro	····················	····················	····················	····················	····················	····················	
7972	MW184022.1	|Severe acute respiratory syndrome coro	····················	····················	····················	····················	····················	····················	
7973	MW184023.1	|Severe acute respiratory syndrome coro	····················	····················	····················	····················	····················	····················	
7974	MW184025.1	|Severe acute respiratory syndrome coro	····················	····················	····················	····················	····················	····················	
7975	MW184026.1	|Severe acute respiratory syndrome coro	····················	····················	····················	····················	····················	····················	
7976	MW184027.1	|Severe acute respiratory syndrome coro	····················	····················	····················	····················	····················	····················	
7977	MW184029.1	|Severe acute respiratory syndrome coro	····················	····················	····················	····················	····················	····················	
7978	MW184030.1	|Severe acute respiratory syndrome coro	····················	····················	····················	····················	····················	····················	
7979	MW184032.1	|Severe acute respiratory syndrome coro	····················	·····A··············	····················	····················	····················	····················	
7980	MW184033.1	|Severe acute respiratory syndrome coro	····················	····················	····················	····················	····················	····················	
7981	MW184035.1	|Severe acute respiratory syndrome coro	····················	····················	····················	····················	····················	····················	
7982	MW184036.1	|Severe acute respiratory syndrome coro	····················	····················	····················	····················	····················	····················	
7983	MW184037.1	|Severe acute respiratory syndrome coro	····················	····················	····················	····················	····················	····················	
7984	MW184038.1	|Severe acute respiratory syndrome coro	····················	····················	····················	····················	····················	····················	
7985	MW184041.1	|Severe acute respiratory syndrome coro	····················	····················	····················	····················	····················	····················	
7986	MW184042.1	|Severe acute respiratory syndrome coro	····················	····················	····················	····················	····················	····················	
7987	MW184044.1	|Severe acute respiratory syndrome coro	····················	····················	····················	····················	····················	····················	
7988	MW184045.1	|Severe acute respiratory syndrome coro	····················	····················	····················	····················	····················	····················	
7989	MW184046.1	|Severe acute respiratory syndrome coro	····················	····················	····················	····················	····················	····················	
7990	MW184048.1	|Severe acute respiratory syndrome coro	····················	····················	····················	····················	····················	····················	
7991	MW184049.1	|Severe acute respiratory syndrome coro	····················	····················	····················	····················	····················	····················	
7992	MW184050.1	|Severe acute respiratory syndrome coro	····················	····················	····················	····················	····················	····················	
7993	MW184051.1	|Severe acute respiratory syndrome coro	····················	····················	····················	····················	····················	····················	
7994	MW184052.1	|Severe acute respiratory syndrome coro	····················	····················	····················	····················	····················	····················	
7995	MW184053.1	|Severe acute respiratory syndrome coro	····················	····················	····················	····················	····················	····················	
7996	MW184054.1	|Severe acute respiratory syndrome coro	····················	····················	····················	····················	····················	····················	
7997	MW184056.1	|Severe acute respiratory syndrome coro	····················	····················	····················	····················	····················	····················	
7998	MW184057.1	|Severe acute respiratory syndrome coro	····················	····················	····················	····················	····················	····················	
7999	MW184058.1	|Severe acute respiratory syndrome coro	····················	····················	····················	····················	····················	····················	
8000	MW184060.1	|Severe acute respiratory syndrome coro	····················	····················	····················	····················	····················	····················	
8001	MW184061.1	|Severe acute respiratory syndrome coro	····················	····················	····················	····················	····················	····················	

8002 MW184062.1 |Severe acute respiratory syndrome coro ···················· ···················· ····················· ···················· ···················· ····················
8003 MW184063.1 |Severe acute respiratory syndrome coro ···················· ···················· ····················· ···················· ···················· ····················
8004 MW184064.1 |Severe acute respiratory syndrome coro ···················· ···················· ····················· ···················· ···················· ····················
8005 MW184066.1 |Severe acute respiratory syndrome coro ···················· ···················· ····················· ···················· ···················· ····················
8006 MW184067.1 |Severe acute respiratory syndrome coro ···················· ···················· ····················· ···················· ···················· ····················
8007 MW184068.1 |Severe acute respiratory syndrome coro ···················· ···················· ····················· ···················· ···················· ····················
8008 MW184069.1 |Severe acute respiratory syndrome coro ···················· ···················· ····················· ···················· ···················· ····················
8009 MW184070.1 |Severe acute respiratory syndrome coro ···················· ···················· ····················· ···················· ···················· ····················
8010 MW184073.1 |Severe acute respiratory syndrome coro ···················· ···················· ····················· ···················· ···················· ····················
8011 MW184074.1 |Severe acute respiratory syndrome coro ···················· ···················· ····················· ···················· ···················· ····················
8012 MW184075.1 |Severe acute respiratory syndrome coro ···················· ···················· ····················· ···················· ···················· ····················
8013 MW184077.1 |Severe acute respiratory syndrome coro ···················· ···················· ····················· ···················· ···················· ····················
8014 MW184079.1 |Severe acute respiratory syndrome coro ···················· ···················· ····················· ···················· ···················· ····················
8015 MW184082.1 |Severe acute respiratory syndrome coro ···················· ···················· ····················· ···················· ···················· ····················
8016 MW184083.1 |Severe acute respiratory syndrome coro ···················· ···················· ····················· ···················· ···················· ····················
8017 MW184084.1 |Severe acute respiratory syndrome coro ···················· ···················· ····················· ···················· ···················· ····················
8018 MW184085.1 |Severe acute respiratory syndrome coro ···················· ···················· ····················· ···················· ···················· ····················
8019 MW184087.1 |Severe acute respiratory syndrome coro ···················· ···················· ····················· ···················· ···················· ····················
8020 MW184088.1 |Severe acute respiratory syndrome coro ···················· ···················· ····················· ···················· ···················· ····················
8021 MW184089.1 |Severe acute respiratory syndrome coro ···················· ···················· ····················· ···················· ···················· ····················
8022 MW184091.1 |Severe acute respiratory syndrome coro ···················· ···················· ····················· ···················· ···················· ····················
8023 MW184092.1 |Severe acute respiratory syndrome coro ···················· ···················· ····················· ···················· ···················· ····················
8024 MW184093.1 |Severe acute respiratory syndrome coro ···················· ···················· ····················· ···················· ···················· ····················
8025 MW184094.1 |Severe acute respiratory syndrome coro ···················· ···················· ····················· ···················· ···················· ····················
8026 MW184095.1 |Severe acute respiratory syndrome coro ···················· ···················· ····················· ···················· ···················· ····················
8027 MW184097.1 |Severe acute respiratory syndrome coro ···················· ···················· ····················· ···················· ···················· ····················
8028 MW184098.1 |Severe acute respiratory syndrome coro ···················· ···················· ····················· ···················· ···················· ····················
8029 MW184099.1 |Severe acute respiratory syndrome coro ···················· ···················· ····················· ···················· ···················· ····················
8030 MW184100.1 |Severe acute respiratory syndrome coro ···················· ···················· ····················· ···················· ···················· ····················
8031 MW184101.1 |Severe acute respiratory syndrome coro ···················· ···················· ····················· ···················· ···················· ····················
8032 MW184102.1 |Severe acute respiratory syndrome coro ···················· ···················· ····················· ···················· ···················· ····················
8033 MW184103.1 |Severe acute respiratory syndrome coro ···················· ···················· ····················· ···················· ···················· ····················
8034 MW184105.1 |Severe acute respiratory syndrome coro ···················· ···················· ····················· ···················· ···················· ····················
8035 MW184107.1 |Severe acute respiratory syndrome coro ···················· ···················· ····················· ···················· ···················· ····················
8036 MW184108.1 |Severe acute respiratory syndrome coro ···················· ···················· ····················· ···················· ···················· ····················
8037 MW184109.1 |Severe acute respiratory syndrome coro ···················· ···················· ····················· ···················· ···················· ····················
8038 MW184110.1 |Severe acute respiratory syndrome coro ···················· ···················· ····················· ···················· ···················· ····················
8039 MW184111.1 |Severe acute respiratory syndrome coro ···················· ······A············· ····················· ···················· ···················· ····················
8040 MW184112.1 |Severe acute respiratory syndrome coro ···················· ···················· ····················· ···················· ···················· ····················
8041 MW184113.1 |Severe acute respiratory syndrome coro ···················· ···················· ····················· ···················· ···················· ····················
8042 MW184115.1 |Severe acute respiratory syndrome coro ···················· ···················· ····················· ···················· ···················· ····················
8043 MW184116.1 |Severe acute respiratory syndrome coro ···················· ···················· ····················· ···················· ···················· ····················
8044 MW184117.1 |Severe acute respiratory syndrome coro ···················· ···················· ····················· ···················· ···················· ····················
8045 MW184121.1 |Severe acute respiratory syndrome coro ···················· ···················· ····················· ···················· ···················· ····················
8046 MW184122.1 |Severe acute respiratory syndrome coro ···················· ···················· ····················· ···················· ···················· ····················
8047 MW184123.1 |Severe acute respiratory syndrome coro ···················· ···················· ····················· ···················· ···················· ····················
8048 MW184124.1 |Severe acute respiratory syndrome coro ···················· ···················· ····················· ···················· ···················· ····················
8049 MW184125.1 |Severe acute respiratory syndrome coro ···················· ···················· ····················· ···················· ···················· ····················
8050 MW184126.1 |Severe acute respiratory syndrome coro ···················· ···················· ····················· ···················· ···················· ····················
8051 MW184127.1 |Severe acute respiratory syndrome coro ···················· ···················· ····················· ···················· ···················· ····················
8052 MW184128.1 |Severe acute respiratory syndrome coro ···················· ···················· ····················· ···················· ···················· ····················
8053 MW184129.1 |Severe acute respiratory syndrome coro ···················· ···················· ····················· ···················· ···················· ····················
8054 MW184130.1 |Severe acute respiratory syndrome coro ···················· ···················· ····················· ···················· ···················· ····················
8055 MW184131.1 |Severe acute respiratory syndrome coro ···················· ···················· ····················· ···················· ···················· ····················
8056 MW184132.1 |Severe acute respiratory syndrome coro ···················· ···················· ····················· ···················· ···················· ····················
8057 MW184134.1 |Severe acute respiratory syndrome coro ···················· ···················· ····················· ···················· ···················· ····················
8058 MW184136.1 |Severe acute respiratory syndrome coro ···················· ···················· ····················· ···················· ···················· ····················
8059 MW184137.1 |Severe acute respiratory syndrome coro ···················· ···················· ····················· ···················· ···················· ····················
8060 MW184138.1 |Severe acute respiratory syndrome coro ···················· ···················· ····················· ···················· ···················· ····················
8061 MW184139.1 |Severe acute respiratory syndrome coro ···················· ···················· ····················· ···················· ···················· ····················
8062 MW184140.1 |Severe acute respiratory syndrome coro ···················· ···················· ····················· ···················· ···················· ····················
8063 MW184141.1 |Severe acute respiratory syndrome coro ···················· ···················· ····················· ···················· ···················· ····················
8064 MW184142.1 |Severe acute respiratory syndrome coro ···················· ···················· ····················· ···················· ···················· ····················
8065 MW184143.1 |Severe acute respiratory syndrome coro ···················· ···················· ····················· ···················· ···················· ····················
8066 MW184144.1 |Severe acute respiratory syndrome coro ···················· ···················· ····················· ···················· ···················· ····················
8067 MW184148.1 |Severe acute respiratory syndrome coro ···················· ···················· ····················· ···················· ···················· ····················
8068 MW184149.1 |Severe acute respiratory syndrome coro ···················· ···················· ····················· ···················· ···················· ····················
8069 MW184150.1 |Severe acute respiratory syndrome coro ···················· ···················· ····················· ···················· ···················· ····················
8070 MW184151.1 |Severe acute respiratory syndrome coro ···················· ···················· ····················· ···················· ···················· ····················
8071 MW184152.1 |Severe acute respiratory syndrome coro ···················· ···················· ····················· ···················· ···················· ····················
8072 MW184153.1 |Severe acute respiratory syndrome coro ···················· ···················· ····················· ···················· ···················· ····················
8073 MW184154.1 |Severe acute respiratory syndrome coro ···················· ···················· ····················· ···················· ···················· ····················
8074 MW184155.1 |Severe acute respiratory syndrome coro ···················· ···················· ····················· ···················· ···················· ····················
8075 MW184156.1 |Severe acute respiratory syndrome coro ···················· ···················· ····················· ···················· ···················· ····················
8076 MW184157.1 |Severe acute respiratory syndrome coro ···················· ···················· ····················· ···················· ···················· ····················
8077 MW184158.1 |Severe acute respiratory syndrome coro ···················· ···················· ····················· ···················· ···················· ····················
8078 MW184159.1 |Severe acute respiratory syndrome coro ···················· ···················· ····················· ···················· ···················· ····················
8079 MW184160.1 |Severe acute respiratory syndrome coro ···················· ···················· ····················· ···················· ···················· ····················

```
8080 MW184161.1 |Severe acute respiratory syndrome coro    ....................  ....................  ....................  ....................  ....................  ....................
8081 MW184162.1 |Severe acute respiratory syndrome coro    ....................  ....................  ....................  ....................  ....................  ....................
8082 MW184163.1 |Severe acute respiratory syndrome coro    ....................  ....................  ....................  ....................  ....................  ....................
8083 MW184164.1 |Severe acute respiratory syndrome coro    ....................  ....................  ....................  ....................  ....................  ....................
8084 MW184166.1 |Severe acute respiratory syndrome coro    ....................  ....................  ....................  ....................  ....................  ....................
8085 MW184168.1 |Severe acute respiratory syndrome coro    ....................  ....................  ....................  ....................  ....................  ....................
8086 MW184169.1 |Severe acute respiratory syndrome coro    ....................  ....................  ....................  ....................  ....................  ....................
8087 MW184170.1 |Severe acute respiratory syndrome coro    ....................  ....................  ....................  ....................  ....................  ....................
8088 MW184171.1 |Severe acute respiratory syndrome coro    ....................  ....................  ....................  ....................  ....................  ....................
8089 MW184172.1 |Severe acute respiratory syndrome coro    ....................  ....................  ....................  ....................  ....................  ....................
8090 MW184174.1 |Severe acute respiratory syndrome coro    ....................  ....................  ....................  ....................  ....................  ....................
8091 MW184175.1 |Severe acute respiratory syndrome coro    ....................  ....................  ....................  ....................  ....................  ....................
8092 MW184176.1 |Severe acute respiratory syndrome coro    ....................  ....................  ....................  ....................  ....................  ....................
8093 MW184177.1 |Severe acute respiratory syndrome coro    ....................  ....................  ....................  ....................  ....................  ....................
8094 MW184178.1 |Severe acute respiratory syndrome coro    ....................  ....................  ....................  ....................  ....................  ....................
8095 MW184180.1 |Severe acute respiratory syndrome coro    ....................  ....................  ....................  ....................  ....................  ....................
8096 MW184182.1 |Severe acute respiratory syndrome coro    ....................  ....................  ....................  ....................  ....................  ....................
8097 MW184183.1 |Severe acute respiratory syndrome coro    ....................  ....................  ....................  ....................  ....................  ....................
8098 MW184184.1 |Severe acute respiratory syndrome coro    ....................  ....................  ....................  ....................  ....................  ....................
8099 MW184185.1 |Severe acute respiratory syndrome coro    ....................  ....................  ....................  ....................  ....................  ....................
8100 MW184186.1 |Severe acute respiratory syndrome coro    ....................  ....................  ....................  ....................  ....................  ....................
8101 MW184188.1 |Severe acute respiratory syndrome coro    ....................  ....................  ....................  ....................  ....................  ....................
8102 MW184190.1 |Severe acute respiratory syndrome coro    ....................  ....................  ....................  ....................  ....................  ....................
8103 MW184191.1 |Severe acute respiratory syndrome coro    ....................  ....................  ....................  ....................  ....................  ....................
8104 MW184194.1 |Severe acute respiratory syndrome coro    ....................  ....................  ....................  ....................  ....................  ....................
8105 MW184195.1 |Severe acute respiratory syndrome coro    ....................  ....................  ....................  ....................  ....................  ....................
8106 MW184196.1 |Severe acute respiratory syndrome coro    ....................  ....................  ....................  ....................  ....................  ....................
8107 MW184197.1 |Severe acute respiratory syndrome coro    ....................  ....................  ....................  ....................  ....................  ....................
8108 MW184198.1 |Severe acute respiratory syndrome coro    ....................  ....................  ....................  ....................  ....................  ....................
8109 MW184199.1 |Severe acute respiratory syndrome coro    ....................  ....................  ....................  ....................  ....................  ....................
8110 MW184200.1 |Severe acute respiratory syndrome coro    ....................  ....................  ....................  ....................  ....................  ....................
8111 MW184201.1 |Severe acute respiratory syndrome coro    ....................  ....................  ....................  ....................  ....................  ....................
8112 MW184202.1 |Severe acute respiratory syndrome coro    ....................  ....................  ....................  ....................  ....................  ....................
8113 MW184203.1 |Severe acute respiratory syndrome coro    ....................  ....................  ....................  ....................  ....................  ....................
8114 MW184205.1 |Severe acute respiratory syndrome coro    ....................  ....................  ....................  ....................  ....................  ....................
8115 MW184206.1 |Severe acute respiratory syndrome coro    ....................  ....................  ....................  ....................  ....................  ....................
8116 MW184207.1 |Severe acute respiratory syndrome coro    ....................  ....................  ....................  ....................  ....................  ....................
8117 MW184208.1 |Severe acute respiratory syndrome coro    ....................  ....................  ....................  ....................  ....................  ....................
8118 MW184209.1 |Severe acute respiratory syndrome coro    ....................  ....................  ....................  ....................  ....................  ....................
8119 MW184212.1 |Severe acute respiratory syndrome coro    ....................  ....................  ....................  ....................  ....................  ....................
8120 MW184213.1 |Severe acute respiratory syndrome coro    ....................  ....................  ....................  ....................  ....................  ....................
8121 MW184214.1 |Severe acute respiratory syndrome coro    ....................  ....................  ....................  ....................  ....................  ....................
8122 MW184215.1 |Severe acute respiratory syndrome coro    ....................  ....................  ....................  ....................  ....................  ....................
8123 MW184216.1 |Severe acute respiratory syndrome coro    ....................  ....................  ....................  ....................  ....................  ....................
8124 MW184217.1 |Severe acute respiratory syndrome coro    ....................  ....................  ....................  ....................  ....................  ....................
8125 MW184218.1 |Severe acute respiratory syndrome coro    ....................  ....................  ....................  ....................  ....................  ....................
8126 MW184219.1 |Severe acute respiratory syndrome coro    ....................  ....................  ....................  ....................  ....................  ....................
8127 MW184220.1 |Severe acute respiratory syndrome coro    ....................  ....................  ....................  ....................  ....................  ....................
8128 MW184222.1 |Severe acute respiratory syndrome coro    ....................  ....................  ....................  ....................  ....................  ....................
8129 MW184223.1 |Severe acute respiratory syndrome coro    ....................  ....................  ....................  ....................  ....................  ....................
8130 MW184226.1 |Severe acute respiratory syndrome coro    ....................  ....................  ....................  ....................  ....................  ....................
8131 MW184228.1 |Severe acute respiratory syndrome coro    ....................  ....................  ....................  ....................  ....................  ....................
8132 MW184230.1 |Severe acute respiratory syndrome coro    ....................  ....................  ....................  ....................  ....................  ....................
8133 MW184231.1 |Severe acute respiratory syndrome coro    ....................  ....................  ....................  ....................  ....................  ....................
8134 MW184232.1 |Severe acute respiratory syndrome coro    ....................  ....................  ....................  ....................  ....................  ....................
8135 MW184233.1 |Severe acute respiratory syndrome coro    ....................  ....................  ....................  ....................  ....................  ....................
8136 MW184234.1 |Severe acute respiratory syndrome coro    ....................  ....................  ....................  ....................  ....................  ....................
8137 MW184235.1 |Severe acute respiratory syndrome coro    ....................  ....................  ....................  ....................  ....................  ....................
8138 MW184236.1 |Severe acute respiratory syndrome coro    ....................  ....................  ....................  ....................  ....................  ....................
8139 MW184238.1 |Severe acute respiratory syndrome coro    ....................  ....................  ....................  ....................  ....................  ....................
8140 MW184239.1 |Severe acute respiratory syndrome coro    ....................  ....................  ....................  ....................  ....................  ....................
8141 MW184241.1 |Severe acute respiratory syndrome coro    ....................  ....................  ....................  ....................  ....................  ....................
8142 MW184243.1 |Severe acute respiratory syndrome coro    ....................  ....................  ....................  ....................  ....................  ....................
8143 MW184244.1 |Severe acute respiratory syndrome coro    ....................  ....................  ....................  ....................  ....................  ....................
8144 MW184245.1 |Severe acute respiratory syndrome coro    ....................  ....................  ....................  ....................  ....................  ....................
8145 MW184247.1 |Severe acute respiratory syndrome coro    ....................  ....................  ....................  ....................  ....................  ....................
8146 MW184248.1 |Severe acute respiratory syndrome coro    ....................  ....................  ....................  ....................  ....................  ....................
8147 MW184249.1 |Severe acute respiratory syndrome coro    ....................  ....................  ....................  ....................  ....................  ....................
8148 MW184250.1 |Severe acute respiratory syndrome coro    ....................  ....................  ....................  ....................  ....................  ....................
8149 MW184252.1 |Severe acute respiratory syndrome coro    ....................  ....................  ....................  ....................  ....................  ....................
8150 MW184254.1 |Severe acute respiratory syndrome coro    ....................  ....................  ....................  ....................  ....................  ....................
8151 MW184255.1 |Severe acute respiratory syndrome coro    ....................  ....................  ....................  ....................  ....................  ....................
8152 MW184256.1 |Severe acute respiratory syndrome coro    ....................  ....................  ....................  ....................  ....................  ....................
8153 MW184257.1 |Severe acute respiratory syndrome coro    ....................  ....................  ....................  ....................  ....................  ....................
8154 MW184258.1 |Severe acute respiratory syndrome coro    ....................  ....................  ....................  ....................  ....................  ....................
8155 MW184259.1 |Severe acute respiratory syndrome coro    ....................  ....................  ....................  ....................  ....................  ....................
8156 MW184260.1 |Severe acute respiratory syndrome coro    ....................  ....................  ....................  ....................  ....................  ....................
8157 MW184261.1 |Severe acute respiratory syndrome coro    ....................  ....................  ....................  ....................  ....................  ....................
```

8158	MW184263.1	Severe acute respiratory syndrome coro	····················	····················	·····················	····················	····················	····················
8159	MW184264.1	Severe acute respiratory syndrome coro	····················	····················	·····················	····················	····················	····················
8160	MW184266.1	Severe acute respiratory syndrome coro	····················	····················	·····················	····················	····················	····················
8162	MW184268.1	Severe acute respiratory syndrome coro	····················	····················	·····················	····················	····················	····················
8163	MW184269.1	Severe acute respiratory syndrome coro	····················	····················	·····················	····················	····················	····················
8164	MW184270.1	Severe acute respiratory syndrome coro	····················	····················	·····················	····················	····················	····················
8165	MW184271.1	Severe acute respiratory syndrome coro	····················	····················	·····················	····················	····················	····················
8166	MW184272.1	Severe acute respiratory syndrome coro	····················	····················	·····················	····················	····················	····················
8167	MW184273.1	Severe acute respiratory syndrome coro	····················	····················	·····················	····················	····················	····················
8168	MW184274.1	Severe acute respiratory syndrome coro	····················	····················	·····················	····················	····················	····················
8169	MW184275.1	Severe acute respiratory syndrome coro	····················	····················	·····················	····················	····················	····················
8170	MW184276.1	Severe acute respiratory syndrome coro	····················	····················	·····················	····················	····················	····················
8171	MW184277.1	Severe acute respiratory syndrome coro	····················	····················	·····················	····················	····················	····················
8172	MW184278.1	Severe acute respiratory syndrome coro	····················	····················	·····················	····················	····················	····················
8173	MW184279.1	Severe acute respiratory syndrome coro	····················	····················	·····················	····················	····················	····················
8174	MW184280.1	Severe acute respiratory syndrome coro	····················	····················	·····················	····················	····················	····················
8175	MW184281.1	Severe acute respiratory syndrome coro	····················	····················	·····················	····················	····················	····················
8176	MW184282.1	Severe acute respiratory syndrome coro	····················	····················	·····················	····················	····················	····················
8177	MW184283.1	Severe acute respiratory syndrome coro	····················	····················	·····················	····················	····················	····················
8179	MW184286.1	Severe acute respiratory syndrome coro	····················	····················	·····················	····················	····················	····················
8180	MW184288.1	Severe acute respiratory syndrome coro	····················	····················	·····················	····················	····················	····················
8181	MW184289.1	Severe acute respiratory syndrome coro	····················	····················	·····················	····················	····················	····················
8182	MW184290.1	Severe acute respiratory syndrome coro	····················	····················	·····················	····················	····················	····················
8183	MW184291.1	Severe acute respiratory syndrome coro	····················	····················	·····················	····················	····················	····················
8184	MW184293.1	Severe acute respiratory syndrome coro	····················	····················	·····················	····················	····················	····················
8185	MW184294.1	Severe acute respiratory syndrome coro	····················	····················	·····················	····················	····················	····················
8186	MW184295.1	Severe acute respiratory syndrome coro	····················	····················	·····················	····················	····················	····················
8187	MW184297.1	Severe acute respiratory syndrome coro	····················	····················	·····················	····················	····················	····················
8188	MW184298.1	Severe acute respiratory syndrome coro	····················	····················	·····················	····················	····················	····················
8189	MW184299.1	Severe acute respiratory syndrome coro	····················	····················	·····················	····················	····················	····················
8190	MW184300.1	Severe acute respiratory syndrome coro	····················	····················	·····················	····················	····················	····················
8191	MW184301.1	Severe acute respiratory syndrome coro	····················	····················	·····················	····················	····················	····················
8192	MW184302.1	Severe acute respiratory syndrome coro	····················	····················	·····················	····················	····················	····················
8193	MW184305.1	Severe acute respiratory syndrome coro	····················	····················	·····················	····················	····················	····················
8194	MW184306.1	Severe acute respiratory syndrome coro	····················	····················	·····················	····················	····················	····················
8195	MW184307.1	Severe acute respiratory syndrome coro	····················	····················	·····················	····················	····················	····················
8196	MW184308.1	Severe acute respiratory syndrome coro	····················	····················	·····················	····················	····················	····················
8197	MW184310.1	Severe acute respiratory syndrome coro	····················	····················	·····················	····················	····················	····················
8198	MW184311.1	Severe acute respiratory syndrome coro	····················	····················	·····················	····················	····················	····················
8199	MW184314.1	Severe acute respiratory syndrome coro	····················	····················	·····················	····················	····················	····················
8200	MW184315.1	Severe acute respiratory syndrome coro	····················	····················	·····················	····················	····················	····················
8201	MW184317.1	Severe acute respiratory syndrome coro	····················	····················	·····················	····················	····················	····················
8202	MW184318.1	Severe acute respiratory syndrome coro	····················	····················	·····················	····················	····················	····················
8203	MW184320.1	Severe acute respiratory syndrome coro	····················	····················	·····················	····················	····················	····················
8204	MW184321.1	Severe acute respiratory syndrome coro	····················	····················	·····················	····················	····················	····················
8205	MW184322.1	Severe acute respiratory syndrome coro	····················	····················	·····················	····················	····················	····················
8206	MW184323.1	Severe acute respiratory syndrome coro	····················	····················	·····················	····················	····················	····················
8207	MW184325.1	Severe acute respiratory syndrome coro	····················	····················	·····················	····················	····················	····················
8208	MW184326.1	Severe acute respiratory syndrome coro	····················	····················	·····················	····················	····················	····················
8209	MW184327.1	Severe acute respiratory syndrome coro	····················	····················	·····················	····················	····················	····················
8210	MW184328.1	Severe acute respiratory syndrome coro	····················	····················	··C··················	····················	····················	····················
8211	MW184329.1	Severe acute respiratory syndrome coro	····················	····················	·····················	····················	····················	····················
8212	MW184330.1	Severe acute respiratory syndrome coro	····················	····················	·····················	····················	····················	····················
8213	MW184332.1	Severe acute respiratory syndrome coro	····················	····················	·····················	····················	····················	····················
8214	MW184333.1	Severe acute respiratory syndrome coro	····················	····················	·····················	····················	····················	····················
8215	MW184335.1	Severe acute respiratory syndrome coro	····················	····················	·····················	····················	····················	····················
8216	MW184336.1	Severe acute respiratory syndrome coro	····················	····················	·····················	····················	····················	····················
8217	MW184337.1	Severe acute respiratory syndrome coro	····················	····················	·····················	····················	····················	····················
8218	MW184339.1	Severe acute respiratory syndrome coro	····················	····················	·····················	····················	····················	····················
8219	MW184340.1	Severe acute respiratory syndrome coro	····················	····················	·····················	····················	····················	····················
8220	MW184341.1	Severe acute respiratory syndrome coro	····················	····················	·····················	····················	····················	····················
8221	MW184343.1	Severe acute respiratory syndrome coro	····················	····················	·····················	····················	····················	····················
8222	MW184344.1	Severe acute respiratory syndrome coro	····················	····················	·····················	····················	····················	····················
8223	MW184345.1	Severe acute respiratory syndrome coro	····················	····················	·····················	····················	····················	····················
8224	MW184346.1	Severe acute respiratory syndrome coro	····················	····················	·····················	····················	····················	····················
8225	MW184347.1	Severe acute respiratory syndrome coro	····················	····················	·····················	····················	····················	····················
8226	MW184349.1	Severe acute respiratory syndrome coro	····················	····················	·····················	····················	····················	····················
8227	MW184350.1	Severe acute respiratory syndrome coro	····················	····················	·····················	····················	····················	····················
8228	MW184351.1	Severe acute respiratory syndrome coro	····················	····················	·····················	····················	····················	····················
8229	MW184352.1	Severe acute respiratory syndrome coro	····················	····················	·····················	····················	····················	····················
8230	MW184353.1	Severe acute respiratory syndrome coro	····················	····················	·····················	····················	····················	····················
8231	MW184354.1	Severe acute respiratory syndrome coro	····················	····················	·····················	····················	····················	····················
8232	MW184356.1	Severe acute respiratory syndrome coro	····················	····················	·····················	····················	····················	····················
8233	MW184357.1	Severe acute respiratory syndrome coro	····················	····················	·····················	····················	····················	····················
8234	MW184358.1	Severe acute respiratory syndrome coro	····················	····················	·····················	····················	····················	····················
8235	MW184359.1	Severe acute respiratory syndrome coro	····················	····················	·····················	····················	····················	····················
8236	MW184360.1	Severe acute respiratory syndrome coro	····················	····················	·····················	····················	····················	····················
8237	MW184361.1	Severe acute respiratory syndrome coro	····················	····················	·····················	····················	····················	····················

```
8238 MW184363.1 |Severe acute respiratory syndrome coro    ....................  ....................  ....................  ....................  ....................  ....................
8239 MW184365.1 |Severe acute respiratory syndrome coro    ....................  ....................  ....................  ....................  ....................  ....................
8240 MW184366.1 |Severe acute respiratory syndrome coro    ....................  ....................  ....................  ....................  ....................  ....................
8241 MW184367.1 |Severe acute respiratory syndrome coro    ....................  ....................  ....................  ....................  ....................  ....................
8242 MW184368.1 |Severe acute respiratory syndrome coro    ....................  ....................  ....................  ....................  ....................  ....................
8243 MW184370.1 |Severe acute respiratory syndrome coro    ....................  ....................  ....................  ....................  ....................  ....................
8244 MW184371.1 |Severe acute respiratory syndrome coro    ....................  ....................  ....................  ....................  ....................  ....................
8245 MW184372.1 |Severe acute respiratory syndrome coro    ....................  ....................  ....................  ....................  ....................  ....................
8246 MW184373.1 |Severe acute respiratory syndrome coro    ....................  ....................  ....................  ....................  ....................  ....................
8247 MW184374.1 |Severe acute respiratory syndrome coro    ....................  ....................  ....................  ....................  ....................  ....................
8248 MW184376.1 |Severe acute respiratory syndrome coro    ....................  ....................  ....................  ....................  ....................  ....................
8249 MW184377.1 |Severe acute respiratory syndrome coro    ....................  ......A.............  ....................  ....................  ....................  ....................
8250 MW184378.1 |Severe acute respiratory syndrome coro    ....................  ....................  ....................  ....................  ....................  ....................
8251 MW184379.1 |Severe acute respiratory syndrome coro    ....................  ....................  ....................  ....................  ....................  ....................
8252 MW184380.1 |Severe acute respiratory syndrome coro    ....................  ....................  ....................  ....................  ....................  ....................
8253 MW184381.1 |Severe acute respiratory syndrome coro    ....................  ....................  ....................  ....................  ....................  ....................
8254 MW184382.1 |Severe acute respiratory syndrome coro    ....................  ....................  ....................  ....................  ....................  ....................
8256 MW184384.1 |Severe acute respiratory syndrome coro    ....................  ....................  ....................  ....................  ....................  ....................
8257 MW184385.1 |Severe acute respiratory syndrome coro    ....................  ....................  ....................  ....................  ....................  ....................
8258 MW184387.1 |Severe acute respiratory syndrome coro    ....................  ....................  ....................  ....................  ....................  ....................
8259 MW184388.1 |Severe acute respiratory syndrome coro    ....................  ....................  ....................  ....................  ....................  ....................
8260 MW184389.1 |Severe acute respiratory syndrome coro    ....................  ....................  ....................  ....................  ....................  ....................
8261 MW184390.1 |Severe acute respiratory syndrome coro    ....................  ....................  ....................  ....................  ....................  ....................
8262 MW184392.1 |Severe acute respiratory syndrome coro    ....................  ....................  ....................  ....................  ....................  ....................
8263 MW184393.1 |Severe acute respiratory syndrome coro    ....................  ....................  ....................  ....................  ....................  ....................
8264 MW184394.1 |Severe acute respiratory syndrome coro    ....................  ....................  ....................  ....................  ....................  ....................
8265 MW184396.1 |Severe acute respiratory syndrome coro    ....................  ....................  ....................  ....................  ....................  ....................
8267 MW184398.1 |Severe acute respiratory syndrome coro    ....................  ....................  ....................  ....................  ....................  ....................
8268 MW184400.1 |Severe acute respiratory syndrome coro    ....................  ....................  ....................  ....................  ....................  ....................
8269 MW184401.1 |Severe acute respiratory syndrome coro    ....................  ....................  ....................  ....................  ....................  ....................
8270 MW184402.1 |Severe acute respiratory syndrome coro    ....................  ....................  ....................  ....................  ....................  ....................
8272 MW184404.1 |Severe acute respiratory syndrome coro    ....................  ....................  ....................  ....................  ....................  ....................
8273 MW184405.1 |Severe acute respiratory syndrome coro    ....................  ....................  ....................  ....................  ....................  ....................
8274 MW184406.1 |Severe acute respiratory syndrome coro    ....................  ....................  ....................  ....................  ....................  ....................
8275 MW184407.1 |Severe acute respiratory syndrome coro    ....................  ....................  ....................  ....................  ....................  ....................
8276 MW184408.1 |Severe acute respiratory syndrome coro    ....................  ....................  ....................  ....................  ....................  ....................
8277 MW184409.1 |Severe acute respiratory syndrome coro    ....................  ....................  ....................  ....................  ....................  ....................
8278 MW184410.1 |Severe acute respiratory syndrome coro    ....................  ....................  ....................  ....................  ....................  ....................
8279 MW184412.1 |Severe acute respiratory syndrome coro    ....................  ....................  ....................  ....................  ....................  ....................
8280 MW184413.1 |Severe acute respiratory syndrome coro    ....................  ....................  ....................  ....................  ....................  ....................
8281 MW184414.1 |Severe acute respiratory syndrome coro    ....................  ....................  ....................  ....................  ....................  ....................
8282 MW184415.1 |Severe acute respiratory syndrome coro    ....................  ....................  ....................  ....................  ....................  ....................
8283 MW184416.1 |Severe acute respiratory syndrome coro    ....................  ....................  ....................  ....................  ....................  ....................
8284 MW184418.1 |Severe acute respiratory syndrome coro    ....................  ....................  ....................  ....................  ....................  ....................
8285 MW184419.1 |Severe acute respiratory syndrome coro    ....................  ....................  ....................  ....................  ....................  ....................
8286 MW184420.1 |Severe acute respiratory syndrome coro    ....................  ....................  ....................  ....................  ....................  ....................
8287 MW184421.1 |Severe acute respiratory syndrome coro    ....................  ....................  ....................  ....................  ....................  ....................
8288 MW184422.1 |Severe acute respiratory syndrome coro    ....................  ....................  ....................  ....................  ....................  ....................
8289 MW184424.1 |Severe acute respiratory syndrome coro    ....................  ....................  ....................  ....................  ....................  ....................
8290 MW184425.1 |Severe acute respiratory syndrome coro    ....................  ....................  ....................  ....................  ....................  ....................
8291 MW184426.1 |Severe acute respiratory syndrome coro    ....................  ....................  ....................  ....................  ....................  ....................
8292 MW184428.1 |Severe acute respiratory syndrome coro    ....................  ....................  ....................  ....................  ....................  ....................
8293 MW184429.1 |Severe acute respiratory syndrome coro    ....................  ....................  ....................  ....................  ....................  ....................
8294 MW184430.1 |Severe acute respiratory syndrome coro    ....................  ....................  ....................  ....................  ....................  ....................
8295 MW184431.1 |Severe acute respiratory syndrome coro    ....................  ....................  ....................  ....................  ....................  ....................
8296 MW184432.1 |Severe acute respiratory syndrome coro    ....................  ....................  ....................  ....................  ....................  ....................
8297 MW184433.1 |Severe acute respiratory syndrome coro    ....................  ....................  ....................  ....................  ....................  ....................
8298 MW184434.1 |Severe acute respiratory syndrome coro    ....................  ....................  ....................  ....................  ....................  ....................
8299 MW184435.1 |Severe acute respiratory syndrome coro    ....................  ....................  ....................  ....................  ....................  ....................
8300 MW184437.1 |Severe acute respiratory syndrome coro    ....................  ....................  ....................  ....................  ....................  ....................
8301 MW184438.1 |Severe acute respiratory syndrome coro    ....................  ....................  ....................  ....................  ....................  ....................
8302 MW184439.1 |Severe acute respiratory syndrome coro    ....................  ....................  ....................  ....................  ....................  ....................
8304 MW184442.1 |Severe acute respiratory syndrome coro    ....................  ....................  ....................  ....................  ....................  ....................
8305 MW184444.1 |Severe acute respiratory syndrome coro    ....................  ....................  ....................  ....................  ....................  ....................
8306 MW184446.1 |Severe acute respiratory syndrome coro    ....................  ....................  ....................  ....................  ....................  ....................
8307 MW184447.1 |Severe acute respiratory syndrome coro    ....................  ....................  ....................  ....................  ....................  ....................
8308 MW184451.1 |Severe acute respiratory syndrome coro    ....................  ....................  ....................  ....................  ....................  ....................
8309 MW184452.1 |Severe acute respiratory syndrome coro    ....................  ....................  ....................  ....................  ....................  ....................
8310 MW184455.1 |Severe acute respiratory syndrome coro    ....................  ....................  ....................  ....................  ....................  ....................
8311 MW184459.1 |Severe acute respiratory syndrome coro    ....................  ....................  ....................  ....................  ....................  ....................
8313 MW184461.1 |Severe acute respiratory syndrome coro    ....................  ....................  ....................  ....................  ....................  ....................
8314 MW184462.1 |Severe acute respiratory syndrome coro    ....................  ....................  ....................  ....................  ....................  ....................
8315 MW184463.1 |Severe acute respiratory syndrome coro    ....................  ....................  ....................  ....................  ....................  ....................
8316 MW184467.1 |Severe acute respiratory syndrome coro    ....................  ....................  ....................  ....................  ....................  ....................
8317 MW184468.1 |Severe acute respiratory syndrome coro    ....................  ....................  ....................  ....................  ....................  ....................
8318 MW184470.1 |Severe acute respiratory syndrome coro    ....................  ....................  ....................  ....................  ....................  ....................
8319 MW184471.1 |Severe acute respiratory syndrome coro    ....................  ....................  ....................  ....................  ....................  ....................
8320 MW184473.1 |Severe acute respiratory syndrome coro    ....................  ....................  ....................  ....................  ....................  ....................
```

8321 MW184474.1 |Severe acute respiratory syndrome coro ....
8322 MW184475.1 |Severe acute respiratory syndrome coro ....
8323 MW184476.1 |Severe acute respiratory syndrome coro ....
8324 MW184479.1 |Severe acute respiratory syndrome coro ....
8325 MW184480.1 |Severe acute respiratory syndrome coro ....
8326 MW184481.1 |Severe acute respiratory syndrome coro ....
8327 MW184482.1 |Severe acute respiratory syndrome coro ....
8328 MW184483.1 |Severe acute respiratory syndrome coro ....
8329 MW184484.1 |Severe acute respiratory syndrome coro ....
8330 MW184485.1 |Severe acute respiratory syndrome coro ....
8332 MW184487.1 |Severe acute respiratory syndrome coro ....
8333 MW184489.1 |Severe acute respiratory syndrome coro ....
8334 MW184491.1 |Severe acute respiratory syndrome coro ....
8335 MW184493.1 |Severe acute respiratory syndrome coro ....
8336 MW184495.1 |Severe acute respiratory syndrome coro ....
8337 MW184496.1 |Severe acute respiratory syndrome coro ....
8338 MW184497.1 |Severe acute respiratory syndrome coro ....
8339 MW184498.1 |Severe acute respiratory syndrome coro ....
8340 MW184500.1 |Severe acute respiratory syndrome coro ....
8341 MW184503.1 |Severe acute respiratory syndrome coro ....
8342 MW184504.1 |Severe acute respiratory syndrome coro ....
8343 MW184505.1 |Severe acute respiratory syndrome coro ....
8344 MW184509.1 |Severe acute respiratory syndrome coro ....
8345 MW184510.1 |Severe acute respiratory syndrome coro ....
8346 MW184511.1 |Severe acute respiratory syndrome coro ....
8347 MW184513.1 |Severe acute respiratory syndrome coro ....
8348 MW184514.1 |Severe acute respiratory syndrome coro ....
8349 MW184518.1 |Severe acute respiratory syndrome coro ....
8350 MW184519.1 |Severe acute respiratory syndrome coro ....
8351 MW184522.1 |Severe acute respiratory syndrome coro ....
8352 MW184523.1 |Severe acute respiratory syndrome coro ....
8353 MW184524.1 |Severe acute respiratory syndrome coro ....
8354 MW184525.1 |Severe acute respiratory syndrome coro ....
8355 MW184526.1 |Severe acute respiratory syndrome coro ....
8356 MW184527.1 |Severe acute respiratory syndrome coro ....
8357 MW184528.1 |Severe acute respiratory syndrome coro ....
8358 MW184529.1 |Severe acute respiratory syndrome coro ....
8359 MW184530.1 |Severe acute respiratory syndrome coro ....
8360 MW184531.1 |Severe acute respiratory syndrome coro ....
8361 MW184532.1 |Severe acute respiratory syndrome coro ....
8362 MW184534.1 |Severe acute respiratory syndrome coro ....
8363 MW184535.1 |Severe acute respiratory syndrome coro ....
8364 MW184536.1 |Severe acute respiratory syndrome coro ....
8365 MW184537.1 |Severe acute respiratory syndrome coro ....
8366 MW184538.1 |Severe acute respiratory syndrome coro ....
8367 MW184539.1 |Severe acute respiratory syndrome coro ....
8368 MW184540.1 |Severe acute respiratory syndrome coro ....
8369 MW184541.1 |Severe acute respiratory syndrome coro ....A............. ....
8370 MW184542.1 |Severe acute respiratory syndrome coro ....
8371 MW184543.1 |Severe acute respiratory syndrome coro ....
8372 MW184544.1 |Severe acute respiratory syndrome coro ....
8373 MW184545.1 |Severe acute respiratory syndrome coro ....
8374 MW184547.1 |Severe acute respiratory syndrome coro ....
8375 MW184548.1 |Severe acute respiratory syndrome coro ....
8376 MW184550.1 |Severe acute respiratory syndrome coro ....
8377 MW184551.1 |Severe acute respiratory syndrome coro ....
8378 MW184552.1 |Severe acute respiratory syndrome coro ....
8379 MW184553.1 |Severe acute respiratory syndrome coro ....
8380 MW184555.1 |Severe acute respiratory syndrome coro ....
8381 MW184557.1 |Severe acute respiratory syndrome coro ....
8382 MW184558.1 |Severe acute respiratory syndrome coro ....
8384 MW184561.1 |Severe acute respiratory syndrome coro ....
8385 MW184562.1 |Severe acute respiratory syndrome coro ....
8386 MW184563.1 |Severe acute respiratory syndrome coro ....
8387 MW184564.1 |Severe acute respiratory syndrome coro ....
8388 MW184565.1 |Severe acute respiratory syndrome coro ....
8389 MW184566.1 |Severe acute respiratory syndrome coro ....
8390 MW184567.1 |Severe acute respiratory syndrome coro ....
8391 MW184568.1 |Severe acute respiratory syndrome coro ....
8392 MW184569.1 |Severe acute respiratory syndrome coro ....
8393 MW184570.1 |Severe acute respiratory syndrome coro ....
8394 MW184571.1 |Severe acute respiratory syndrome coro ....
8395 MW184572.1 |Severe acute respiratory syndrome coro ....
8396 MW184574.1 |Severe acute respiratory syndrome coro ....
8397 MW184575.1 |Severe acute respiratory syndrome coro ....
8398 MW184576.1 |Severe acute respiratory syndrome coro ....
8399 MW184577.1 |Severe acute respiratory syndrome coro ....
8400 MW184578.1 |Severe acute respiratory syndrome coro ....

8401	MW184579.1	|Severe acute respiratory syndrome coro	····················	····················	····················	····················	····················	····················
8402	MW184582.1	|Severe acute respiratory syndrome coro	····················	····················	····················	····················	····················	····················
8403	MW184583.1	|Severe acute respiratory syndrome coro	····················	····················	····················	····················	····················	····················
8404	MW184584.1	|Severe acute respiratory syndrome coro	····················	····················	····················	····················	····················	····················
8405	MW184585.1	|Severe acute respiratory syndrome coro	····················	····················	····················	····················	····················	····················
8407	MW184589.1	|Severe acute respiratory syndrome coro	····················	····················	····················	····················	····················	····················
8408	MW184590.1	|Severe acute respiratory syndrome coro	····················	····················	····················	····················	····················	····················
8409	MW184591.1	|Severe acute respiratory syndrome coro	····················	····················	····················	····················	····················	····················
8410	MW184592.1	|Severe acute respiratory syndrome coro	····················	····················	····················	····················	····················	····················
8411	MW184594.1	|Severe acute respiratory syndrome coro	····················	····················	····················	····················	····················	····················
8412	MW184595.1	|Severe acute respiratory syndrome coro	····················	····················	····················	····················	····················	····················
8413	MW184598.1	|Severe acute respiratory syndrome coro	····················	····················	····················	····················	····················	····················
8414	MW184599.1	|Severe acute respiratory syndrome coro	····················	····················	····················	····················	····················	····················
8415	MW184601.1	|Severe acute respiratory syndrome coro	····················	····················	····················	····················	····················	····················
8416	MW184603.1	|Severe acute respiratory syndrome coro	····················	····················	····················	····················	····················	····················
8417	MW184604.1	|Severe acute respiratory syndrome coro	····················	····················	····················	····················	····················	····················
8418	MW184605.1	|Severe acute respiratory syndrome coro	····················	····················	····················	····················	····················	····················
8419	MW184606.1	|Severe acute respiratory syndrome coro	····················	····················	····················	····················	····················	····················
8421	MW184609.1	|Severe acute respiratory syndrome coro	····················	····················	····················	····················	····················	····················
8422	MW184610.1	|Severe acute respiratory syndrome coro	····················	····················	····················	····················	····················	····················
8423	MW184611.1	|Severe acute respiratory syndrome coro	····················	····················	····················	····················	····················	····················
8424	MW184612.1	|Severe acute respiratory syndrome coro	····················	····················	····················	····················	····················	····················
8426	MW184614.1	|Severe acute respiratory syndrome coro	····················	····················	····················	····················	····················	····················
8427	MW184616.1	|Severe acute respiratory syndrome coro	····················	····················	····················	····················	····················	····················
8428	MW184617.1	|Severe acute respiratory syndrome coro	····················	····················	····················	····················	····················	····················
8429	MW184618.1	|Severe acute respiratory syndrome coro	····················	····················	····················	····················	····················	····················
8430	MW184621.1	|Severe acute respiratory syndrome coro	····················	····················	····················	····················	····················	····················
8431	MW184622.1	|Severe acute respiratory syndrome coro	····················	····················	····················	····················	····················	····················
8432	MW184623.1	|Severe acute respiratory syndrome coro	····················	····················	····················	····················	····················	····················
8433	MW184624.1	|Severe acute respiratory syndrome coro	····················	····················	····················	····················	····················	····················
8434	MW184625.1	|Severe acute respiratory syndrome coro	····················	····················	····················	····················	····················	····················
8435	MW184626.1	|Severe acute respiratory syndrome coro	····················	····················	····················	····················	····················	····················
8436	MW184627.1	|Severe acute respiratory syndrome coro	····················	····················	····················	····················	····················	····················
8437	MW184629.1	|Severe acute respiratory syndrome coro	····················	····················	····················	····················	····················	····················
8439	MW184632.1	|Severe acute respiratory syndrome coro	····················	····················	····················	····················	····················	····················
8440	MW184633.1	|Severe acute respiratory syndrome coro	····················	····················	····················	····················	····················	····················
8441	MW184634.1	|Severe acute respiratory syndrome coro	····················	····················	····················	····················	····················	····················
8442	MW184635.1	|Severe acute respiratory syndrome coro	····················	····················	····················	····················	····················	····················
8443	MW184636.1	|Severe acute respiratory syndrome coro	····················	····················	····················	····················	····················	····················
8444	MW184637.1	|Severe acute respiratory syndrome coro	····················	····················	····················	····················	····················	····················
8445	MW184638.1	|Severe acute respiratory syndrome coro	····················	····················	····················	····················	····················	····················
8446	MW184639.1	|Severe acute respiratory syndrome coro	····················	····················	····················	····················	····················	····················
8447	MW184640.1	|Severe acute respiratory syndrome coro	····················	····················	····················	····················	····················	····················
8448	MW184641.1	|Severe acute respiratory syndrome coro	····················	····················	····················	····················	····················	····················
8449	MW184642.1	|Severe acute respiratory syndrome coro	····················	····················	····················	····················	····················	····················
8450	MW184643.1	|Severe acute respiratory syndrome coro	····················	····················	····················	····················	····················	····················
8452	MW184646.1	|Severe acute respiratory syndrome coro	····················	····················	····················	····················	····················	····················
8453	MW184647.1	|Severe acute respiratory syndrome coro	····················	····················	····················	····················	····················	····················
8454	MW184648.1	|Severe acute respiratory syndrome coro	····················	····················	····················	····················	····················	····················
8456	MW184650.1	|Severe acute respiratory syndrome coro	····················	····················	····················	····················	····················	····················
8457	MW184652.1	|Severe acute respiratory syndrome coro	····················	····················	····················	····················	····················	····················
8458	MW184653.1	|Severe acute respiratory syndrome coro	····················	····················	····················	····················	····················	····················
8459	MW184654.1	|Severe acute respiratory syndrome coro	····················	····················	····················	····················	····················	····················
8460	MW184655.1	|Severe acute respiratory syndrome coro	····················	····················	····················	····················	····················	····················
8461	MW184657.1	|Severe acute respiratory syndrome coro	····················	····················	····················	····················	····················	····················
8462	MW184658.1	|Severe acute respiratory syndrome coro	····················	····················	····················	····················	····················	····················
8463	MW184659.1	|Severe acute respiratory syndrome coro	····················	····················	····················	····················	····················	····················
8464	MW184661.1	|Severe acute respiratory syndrome coro	····················	·····A··············	····················	····················	····················	····················
8465	MW184662.1	|Severe acute respiratory syndrome coro	····················	····················	····················	····················	····················	····················
8466	MW184663.1	|Severe acute respiratory syndrome coro	····················	····················	····················	····················	····················	····················
8467	MW184664.1	|Severe acute respiratory syndrome coro	····················	····················	····················	····················	····················	····················
8468	MW184665.1	|Severe acute respiratory syndrome coro	····················	····················	····················	····················	····················	····················
8469	MW184666.1	|Severe acute respiratory syndrome coro	····················	····················	····················	····················	····················	····················
8470	MW184668.1	|Severe acute respiratory syndrome coro	····················	····················	····················	····················	····················	····················
8471	MW184670.1	|Severe acute respiratory syndrome coro	····················	····················	····················	····················	····················	····················
8472	MW184671.1	|Severe acute respiratory syndrome coro	····················	····················	····················	····················	····················	····················
8473	MW184673.1	|Severe acute respiratory syndrome coro	····················	····················	····················	····················	····················	····················
8474	MW184674.1	|Severe acute respiratory syndrome coro	····················	····················	····················	····················	····················	····················
8475	MW184675.1	|Severe acute respiratory syndrome coro	····················	····················	····················	····················	····················	····················
8476	MW184676.1	|Severe acute respiratory syndrome coro	····················	····················	····················	····················	····················	····················
8477	MW184677.1	|Severe acute respiratory syndrome coro	····················	····················	····················	····················	····················	····················
8478	MW184678.1	|Severe acute respiratory syndrome coro	····················	····················	····················	····················	····················	····················
8479	MW184679.1	|Severe acute respiratory syndrome coro	····················	····················	····················	····················	····················	····················
8480	MW184680.1	|Severe acute respiratory syndrome coro	····················	····················	····················	····················	····················	····················
8481	MW184683.1	|Severe acute respiratory syndrome coro	····················	····················	····················	····················	····················	····················
8482	MW184689.1	|Severe acute respiratory syndrome coro	····················	····················	····················	····················	····················	····················
8483	MW184690.1	|Severe acute respiratory syndrome coro	····················	····················	····················	····················	····················	····················
8484	MW184691.1	|Severe acute respiratory syndrome coro	····················	····················	····················	····················	····················	····················

```
8485 MW184692.1 |Severe acute respiratory syndrome coro    ....................  ....................  ....................  ....................  ....................  ....................
8486 MW184694.1 |Severe acute respiratory syndrome coro    ....................  ....................  ....................  ....................  ....................  ....................
8487 MW184696.1 |Severe acute respiratory syndrome coro    ....................  ....................  ....................  ....................  ....................  ....................
8488 MW184697.1 |Severe acute respiratory syndrome coro    ....................  ....................  ....................  ....................  ....................  ....................
8489 MW184699.1 |Severe acute respiratory syndrome coro    ....................  ....................  ....................  ....................  ....................  ....................
8490 MW184700.1 |Severe acute respiratory syndrome coro    ....................  ....................  ....................  ....................  ....................  ....................
8491 MW184702.1 |Severe acute respiratory syndrome coro    ....................  ....................  ....................  ....................  ....................  ....................
8492 MW184703.1 |Severe acute respiratory syndrome coro    ....................  ....................  ....................  ....................  ....................  ....................
8493 MW184704.1 |Severe acute respiratory syndrome coro    ....................  ....................  ....................  ....................  ....................  ....................
8494 MW184705.1 |Severe acute respiratory syndrome coro    ....................  ....................  ....................  ....................  ....................  ....................
8495 MW184706.1 |Severe acute respiratory syndrome coro    ....................  ....................  ....................  ....................  ....................  ....................
8496 MW184707.1 |Severe acute respiratory syndrome coro    ....................  ....................  ....................  ....................  ....................  ....................
8497 MW184709.1 |Severe acute respiratory syndrome coro    ....................  ....................  ....................  ....................  ....................  ....................
8498 MW184710.1 |Severe acute respiratory syndrome coro    ....................  ....................  ....................  ....................  ....................  ....................
8499 MW184711.1 |Severe acute respiratory syndrome coro    ....................  ....................  ....................  ....................  ....................  ....................
8500 MW184712.1 |Severe acute respiratory syndrome coro    ....................  ....................  ....................  ....................  ....................  ....................
8501 MW184713.1 |Severe acute respiratory syndrome coro    ....................  ....................  ....................  ....................  ....................  ....................
8502 MW184714.1 |Severe acute respiratory syndrome coro    ....................  ....................  ....................  ....................  ....................  ....................
8503 MW184716.1 |Severe acute respiratory syndrome coro    ....................  ....................  ....................  ....................  ....................  ....................
8504 MW184718.1 |Severe acute respiratory syndrome coro    ....................  ....................  ....................  ....................  ....................  ....................
8505 MW184719.1 |Severe acute respiratory syndrome coro    ....................  ....................  ....................  ....................  ....................  ....................
8506 MW184720.1 |Severe acute respiratory syndrome coro    ....................  ....................  ....................  ....................  ....................  ....................
8507 MW184723.1 |Severe acute respiratory syndrome coro    ....................  ....................  ....................  ....................  ....................  ....................
8508 MW184725.1 |Severe acute respiratory syndrome coro    ....................  ....................  ....................  ....................  ....................  ....................
8509 MW184727.1 |Severe acute respiratory syndrome coro    ....................  ......A.............  ....................  ....................  ....................  ....................
8510 MW184729.1 |Severe acute respiratory syndrome coro    ....................  ....................  ....................  ....................  ....................  ....................
8511 MW184731.1 |Severe acute respiratory syndrome coro    ....................  ....................  ....................  ....................  ....................  ....................
8512 MW184732.1 |Severe acute respiratory syndrome coro    ....................  ....................  ....................  ....................  ....................  ....................
8513 MW184733.1 |Severe acute respiratory syndrome coro    ....................  ....................  ....................  ....................  ....................  ....................
8514 MW184735.1 |Severe acute respiratory syndrome coro    ....................  ....................  ....................  ....................  ....................  ....................
8515 MW184737.1 |Severe acute respiratory syndrome coro    ....................  ....................  ....................  ....................  ....................  ....................
8516 MW184739.1 |Severe acute respiratory syndrome coro    ....................  ....................  ....................  ....................  ....................  ....................
8517 MW184740.1 |Severe acute respiratory syndrome coro    ....................  ....................  ....................  ....................  ....................  ....................
8518 MW184741.1 |Severe acute respiratory syndrome coro    ....................  ....................  ....................  ....................  ....................  ....................
8519 MW184743.1 |Severe acute respiratory syndrome coro    ....................  ....................  ....................  ....................  ....................  ....................
8520 MW184745.1 |Severe acute respiratory syndrome coro    ....................  ....................  ....................  ....................  ....................  ....................
8521 MW184747.1 |Severe acute respiratory syndrome coro    ....................  ....................  ....................  ....................  ....................  ....................
8522 MW184748.1 |Severe acute respiratory syndrome coro    ....................  ....................  ....................  ....................  ....................  ....................
8523 MW184749.1 |Severe acute respiratory syndrome coro    ....................  ....................  ....................  ....................  ....................  ....................
8524 MW184750.1 |Severe acute respiratory syndrome coro    ....................  ....................  ....................  ....................  ....................  ....................
8525 MW184751.1 |Severe acute respiratory syndrome coro    ....................  ....................  ...C................  ....................  ....................  ....................
8526 MW184753.1 |Severe acute respiratory syndrome coro    ....................  ....................  ....................  ....................  ....................  ....................
8527 MW184754.1 |Severe acute respiratory syndrome coro    ....................  ....................  ....................  ....................  ....................  ....................
8528 MW184755.1 |Severe acute respiratory syndrome coro    ....................  ....................  ....................  ....................  ....................  ....................
8529 MW184756.1 |Severe acute respiratory syndrome coro    ....................  ....................  ....................  ....................  ....................  ....................
8530 MW184757.1 |Severe acute respiratory syndrome coro    ....................  ....................  ....................  ....................  ....................  ....................
8531 MW184759.1 |Severe acute respiratory syndrome coro    ....................  ....................  ....................  ....................  ....................  ....................
8532 MW184760.1 |Severe acute respiratory syndrome coro    ....................  ....................  ....................  ....................  ....................  ....................
8533 MW184761.1 |Severe acute respiratory syndrome coro    ....................  ....................  ....................  ....................  ....................  ....................
8534 MW184762.1 |Severe acute respiratory syndrome coro    ....................  ....................  ....................  ....................  ....................  ....................
8535 MW184763.1 |Severe acute respiratory syndrome coro    ....................  ....................  ....................  ....................  ....................  ....................
8536 MW184766.1 |Severe acute respiratory syndrome coro    ....................  ....................  ....................  ....................  ....................  ....................
8537 MW184767.1 |Severe acute respiratory syndrome coro    ....................  ....................  ....................  ....................  ....................  ....................
8538 MW184768.1 |Severe acute respiratory syndrome coro    ....................  ....................  ....................  ....................  ....................  ....................
8539 MW184770.1 |Severe acute respiratory syndrome coro    ....................  ....................  ....................  ....................  ....................  ....................
8540 MW184771.1 |Severe acute respiratory syndrome coro    ....................  ....................  ....................  ....................  ....................  ....................
8541 MW184772.1 |Severe acute respiratory syndrome coro    ....................  ....................  ....................  ....................  ....................  ....................
8542 MW184775.1 |Severe acute respiratory syndrome coro    ....................  ....................  ....................  ....................  ....................  ....................
8543 MW184776.1 |Severe acute respiratory syndrome coro    ....................  ....................  ....................  ....................  ....................  ....................
8544 MW184777.1 |Severe acute respiratory syndrome coro    ....................  ....................  ....................  ....................  ....................  ....................
8545 MW184780.1 |Severe acute respiratory syndrome coro    ....................  ....................  ....................  ....................  ....................  ....................
8546 MW184781.1 |Severe acute respiratory syndrome coro    ....................  ....................  ....................  ....................  ....................  ....................
8547 MW184782.1 |Severe acute respiratory syndrome coro    ....................  ....................  ....................  ....................  ....................  ....................
8548 MW184783.1 |Severe acute respiratory syndrome coro    ....................  ....................  ....................  ....................  ....................  ....................
8549 MW184784.1 |Severe acute respiratory syndrome coro    ....................  ....................  ....................  ....................  ....................  ....................
8550 MW184785.1 |Severe acute respiratory syndrome coro    ....................  ....................  ....................  ....................  ....................  ....................
8551 MW184786.1 |Severe acute respiratory syndrome coro    ....................  ....................  ....................  ....................  ....................  ....................
8552 MW184787.1 |Severe acute respiratory syndrome coro    ....................  ....................  ....................  ....................  ....................  ....................
8553 MW184788.1 |Severe acute respiratory syndrome coro    ....................  ....................  ....................  ....................  ....................  ....................
8554 MW184790.1 |Severe acute respiratory syndrome coro    ....................  ....................  ....................  ....................  ....................  ....................
8555 MW184791.1 |Severe acute respiratory syndrome coro    ....................  ....................  ....................  ....................  ....................  ....................
8556 MW184792.1 |Severe acute respiratory syndrome coro    ....................  ....................  ....................  ....................  ....................  ....................
8557 MW184793.1 |Severe acute respiratory syndrome coro    ....................  ....................  ....................  ....................  ....................  ....................
8558 MW184794.1 |Severe acute respiratory syndrome coro    ....................  ....................  ....................  ....................  ....................  ....................
8559 MW184795.1 |Severe acute respiratory syndrome coro    ....................  ....................  ....................  ....................  ....................  ....................
8560 MW184796.1 |Severe acute respiratory syndrome coro    ....................  ....................  ....................  ....................  ....................  ....................
8561 MW184797.1 |Severe acute respiratory syndrome coro    ....................  ....................  ....................  ....................  ....................  ....................
8562 MW184798.1 |Severe acute respiratory syndrome coro    ....................  ....................  ....................  ....................  ....................  ....................
```

8563	MW184799.1	|Severe acute respiratory syndrome coro	····················	····················	····················	····················	····················	····················
8564	MW184801.1	|Severe acute respiratory syndrome coro	····················	····················	····················	····················	····················	····················
8565	MW184802.1	|Severe acute respiratory syndrome coro	····················	····················	····················	····················	····················	····················
8566	MW184803.1	|Severe acute respiratory syndrome coro	····················	····················	····················	····················	····················	····················
8567	MW184804.1	|Severe acute respiratory syndrome coro	····················	····················	····················	····················	····················	····················
8568	MW184805.1	|Severe acute respiratory syndrome coro	····················	····················	····················	····················	····················	····················
8569	MW184806.1	|Severe acute respiratory syndrome coro	····················	····················	····················	····················	····················	····················
8570	MW184811.1	|Severe acute respiratory syndrome coro	····················	····················	····················	····················	····················	····················
8571	MW184813.1	|Severe acute respiratory syndrome coro	····················	····················	····················	····················	····················	····················
8573	MW184815.1	|Severe acute respiratory syndrome coro	····················	····················	····················	····················	····················	····················
8575	MW184817.1	|Severe acute respiratory syndrome coro	····················	····················	····················	····················	····················	····················
8576	MW184820.1	|Severe acute respiratory syndrome coro	····················	····················	····················	····················	····················	····················
8577	MW184821.1	|Severe acute respiratory syndrome coro	····················	····················	····················	····················	····················	····················
8578	MW184822.1	|Severe acute respiratory syndrome coro	····················	····················	····················	····················	····················	····················
8579	MW184823.1	|Severe acute respiratory syndrome coro	····················	····················	····················	····················	····················	····················
8580	MW184825.1	|Severe acute respiratory syndrome coro	····················	····················	····················	····················	····················	····················
8581	MW184827.1	|Severe acute respiratory syndrome coro	····················	····················	····················	····················	····················	····················
8582	MW184828.1	|Severe acute respiratory syndrome coro	····················	····················	····················	····················	····················	····················
8583	MW184829.1	|Severe acute respiratory syndrome coro	····················	····················	····················	····················	····················	····················
8584	MW184830.1	|Severe acute respiratory syndrome coro	····················	····················	····················	····················	····················	····················
8585	MW184831.1	|Severe acute respiratory syndrome coro	····················	····················	····················	····················	····················	····················
8586	MW184832.1	|Severe acute respiratory syndrome coro	····················	····················	····················	····················	····················	····················
8587	MW184833.1	|Severe acute respiratory syndrome coro	····················	····················	····················	····················	····················	····················
8588	MW184834.1	|Severe acute respiratory syndrome coro	····················	····················	····················	····················	····················	····················
8589	MW184835.1	|Severe acute respiratory syndrome coro	····················	····················	····················	····················	····················	····················
8590	MW184836.1	|Severe acute respiratory syndrome coro	····················	····················	····················	····················	····················	····················
8591	MW184837.1	|Severe acute respiratory syndrome coro	····················	····················	····················	····················	····················	····················
8592	MW184838.1	|Severe acute respiratory syndrome coro	····················	····················	····················	····················	····················	····················
8593	MW184839.1	|Severe acute respiratory syndrome coro	····················	····················	····················	····················	····················	····················
8594	MW184840.1	|Severe acute respiratory syndrome coro	····················	····················	····················	····················	····················	····················
8595	MW184841.1	|Severe acute respiratory syndrome coro	····················	····················	····················	····················	····················	····················
8596	MW184842.1	|Severe acute respiratory syndrome coro	····················	····················	····················	····················	····················	····················
8597	MW184844.1	|Severe acute respiratory syndrome coro	····················	····················	····················	····················	····················	····················
8598	MW184845.1	|Severe acute respiratory syndrome coro	····················	······A·············	····················	····················	····················	····················
8600	MW184849.1	|Severe acute respiratory syndrome coro	····················	····················	····················	····················	····················	····················
8601	MW184851.1	|Severe acute respiratory syndrome coro	····················	····················	····················	····················	····················	····················
8602	MW184852.1	|Severe acute respiratory syndrome coro	····················	····················	····················	····················	····················	····················
8603	MW184853.1	|Severe acute respiratory syndrome coro	····················	····················	····················	····················	····················	····················
8604	MW184854.1	|Severe acute respiratory syndrome coro	····················	····················	····················	····················	····················	····················
8605	MW184855.1	|Severe acute respiratory syndrome coro	····················	····················	····················	····················	····················	····················
8606	MW184856.1	|Severe acute respiratory syndrome coro	····················	····················	····················	····················	····················	····················
8607	MW184858.1	|Severe acute respiratory syndrome coro	····················	····················	····················	····················	····················	····················
8608	MW184859.1	|Severe acute respiratory syndrome coro	····················	····················	····················	····················	····················	····················
8609	MW184860.1	|Severe acute respiratory syndrome coro	····················	····················	····················	····················	····················	····················
8610	MW184861.1	|Severe acute respiratory syndrome coro	····················	····················	····················	····················	····················	····················
8611	MW184862.1	|Severe acute respiratory syndrome coro	····················	····················	····················	····················	····················	····················
8612	MW184864.1	|Severe acute respiratory syndrome coro	····················	····················	····················	····················	····················	····················
8613	MW184865.1	|Severe acute respiratory syndrome coro	····················	····················	····················	····················	····················	····················
8614	MW184866.1	|Severe acute respiratory syndrome coro	····················	····················	····················	····················	····················	····················
8615	MW184867.1	|Severe acute respiratory syndrome coro	····················	····················	····················	····················	····················	····················
8616	MW184869.1	|Severe acute respiratory syndrome coro	····················	····················	····················	····················	····················	····················
8617	MW184870.1	|Severe acute respiratory syndrome coro	····················	····················	····················	····················	····················	····················
8618	MW184871.1	|Severe acute respiratory syndrome coro	····················	····················	····················	····················	····················	····················
8619	MW184872.1	|Severe acute respiratory syndrome coro	····················	····················	····················	····················	····················	····················
8620	MW184874.1	|Severe acute respiratory syndrome coro	····················	····················	····················	····················	····················	····················
8621	MW184875.1	|Severe acute respiratory syndrome coro	····················	····················	····················	····················	····················	····················
8622	MW184876.1	|Severe acute respiratory syndrome coro	····················	····················	····················	····················	·········R··········	····················
8623	MW184877.1	|Severe acute respiratory syndrome coro	····················	····················	····················	····················	····················	····················
8624	MW184879.1	|Severe acute respiratory syndrome coro	····················	······A·············	····················	····················	····················	····················
8625	MW184882.1	|Severe acute respiratory syndrome coro	····················	····················	····················	····················	····················	····················
8626	MW184883.1	|Severe acute respiratory syndrome coro	····················	····················	····················	····················	····················	····················
8627	MW184884.1	|Severe acute respiratory syndrome coro	····················	····················	····················	····················	····················	····················
8628	MW184885.1	|Severe acute respiratory syndrome coro	····················	····················	····················	····················	····················	····················
8629	MW184886.1	|Severe acute respiratory syndrome coro	····················	····················	····················	····················	····················	····················
8630	MW184887.1	|Severe acute respiratory syndrome coro	····················	····················	····················	····················	····················	····················
8631	MW184888.1	|Severe acute respiratory syndrome coro	····················	····················	····················	····················	····················	····················
8633	MW184891.1	|Severe acute respiratory syndrome coro	····················	····················	····················	····················	····················	····················
8634	MW184892.1	|Severe acute respiratory syndrome coro	····················	····················	····················	····················	····················	····················
8635	MW184893.1	|Severe acute respiratory syndrome coro	····················	····················	····················	····················	····················	····················
8636	MW184894.1	|Severe acute respiratory syndrome coro	····················	····················	····················	····················	····················	····················
8637	MW184895.1	|Severe acute respiratory syndrome coro	····················	····················	····················	····················	····················	····················
8638	MW184896.1	|Severe acute respiratory syndrome coro	····················	····················	····················	····················	····················	····················
8639	MW184897.1	|Severe acute respiratory syndrome coro	····················	····················	····················	····················	····················	····················
8640	MW184899.1	|Severe acute respiratory syndrome coro	····················	····················	····················	····················	····················	····················
8641	MW184900.1	|Severe acute respiratory syndrome coro	····················	····················	····················	····················	····················	····················
8642	MW184901.1	|Severe acute respiratory syndrome coro	····················	····················	····················	····················	····················	····················
8643	MW184903.1	|Severe acute respiratory syndrome coro	····················	····················	····················	····················	····················	····················
8644	MW184904.1	|Severe acute respiratory syndrome coro	····················	····················	····················	····················	····················	····················

8645 MW184906.1 |Severe acute respiratory syndrome coro ....
8646 MW184907.1 |Severe acute respiratory syndrome coro ....
8647 MW184908.1 |Severe acute respiratory syndrome coro ....
8648 MW184909.1 |Severe acute respiratory syndrome coro ....
8649 MW184911.1 |Severe acute respiratory syndrome coro ....
8650 MW184914.1 |Severe acute respiratory syndrome coro ....
8651 MW184915.1 |Severe acute respiratory syndrome coro ....
8652 MW184916.1 |Severe acute respiratory syndrome coro ....
8653 MW184917.1 |Severe acute respiratory syndrome coro ....
8654 MW184918.1 |Severe acute respiratory syndrome coro ....
8655 MW184919.1 |Severe acute respiratory syndrome coro ....
8656 MW184922.1 |Severe acute respiratory syndrome coro ....
8657 MW184923.1 |Severe acute respiratory syndrome coro ....
8658 MW184924.1 |Severe acute respiratory syndrome coro ....
8659 MW184925.1 |Severe acute respiratory syndrome coro ....
8660 MW184926.1 |Severe acute respiratory syndrome coro ....
8661 MW184927.1 |Severe acute respiratory syndrome coro ....
8662 MW184928.1 |Severe acute respiratory syndrome coro ....
8663 MW184929.1 |Severe acute respiratory syndrome coro ....
8664 MW184930.1 |Severe acute respiratory syndrome coro ....
8665 MW184931.1 |Severe acute respiratory syndrome coro ....
8666 MW184933.1 |Severe acute respiratory syndrome coro ....
8667 MW184934.1 |Severe acute respiratory syndrome coro ....
8669 MW184938.1 |Severe acute respiratory syndrome coro ....
8670 MW184939.1 |Severe acute respiratory syndrome coro ....
8671 MW184940.1 |Severe acute respiratory syndrome coro ....
8672 MW184941.1 |Severe acute respiratory syndrome coro ....
8673 MW184944.1 |Severe acute respiratory syndrome coro ....
8674 MW184945.1 |Severe acute respiratory syndrome coro ....
8675 MW184947.1 |Severe acute respiratory syndrome coro ....
8676 MW184948.1 |Severe acute respiratory syndrome coro ....
8677 MW184949.1 |Severe acute respiratory syndrome coro ....
8678 MW184950.1 |Severe acute respiratory syndrome coro ....
8679 MW184951.1 |Severe acute respiratory syndrome coro ....
8680 MW184952.1 |Severe acute respiratory syndrome coro ....
8681 MW184953.1 |Severe acute respiratory syndrome coro ....
8682 MW184954.1 |Severe acute respiratory syndrome coro ....
8683 MW184956.1 |Severe acute respiratory syndrome coro ....
8684 MW184960.1 |Severe acute respiratory syndrome coro ....
8685 MW184961.1 |Severe acute respiratory syndrome coro ....
8686 MW184966.1 |Severe acute respiratory syndrome coro ....
8687 MW184968.1 |Severe acute respiratory syndrome coro ....
8688 MW184969.1 |Severe acute respiratory syndrome coro ....
8689 MW184970.1 |Severe acute respiratory syndrome coro ....
8690 MW184972.1 |Severe acute respiratory syndrome coro ....
8691 MW184973.1 |Severe acute respiratory syndrome coro ....
8692 MW184974.1 |Severe acute respiratory syndrome coro ....
8693 MW184975.1 |Severe acute respiratory syndrome coro ....
8694 MW184976.1 |Severe acute respiratory syndrome coro ....
8695 MW184977.1 |Severe acute respiratory syndrome coro ....
8696 MW184978.1 |Severe acute respiratory syndrome coro ....
8697 MW184979.1 |Severe acute respiratory syndrome coro ....
8698 MW184980.1 |Severe acute respiratory syndrome coro ....
8699 MW184981.1 |Severe acute respiratory syndrome coro ....
8700 MW184982.1 |Severe acute respiratory syndrome coro ....
8701 MW184983.1 |Severe acute respiratory syndrome coro ....
8702 MW184984.1 |Severe acute respiratory syndrome coro ....
8703 MW184987.1 |Severe acute respiratory syndrome coro ....
8704 MW184988.1 |Severe acute respiratory syndrome coro ....
8705 MW184989.1 |Severe acute respiratory syndrome coro ....
8706 MW184990.1 |Severe acute respiratory syndrome coro ....
8707 MW184991.1 |Severe acute respiratory syndrome coro ....
8708 MW184992.1 |Severe acute respiratory syndrome coro ....
8709 MW184993.1 |Severe acute respiratory syndrome coro ....
8710 MW184994.1 |Severe acute respiratory syndrome coro ....
8711 MW184995.1 |Severe acute respiratory syndrome coro ....
8712 MW184996.1 |Severe acute respiratory syndrome coro ....
8713 MW184997.1 |Severe acute respiratory syndrome coro ....
8714 MW184999.1 |Severe acute respiratory syndrome coro ....
8715 MW185001.1 |Severe acute respiratory syndrome coro ....
8716 MW185003.1 |Severe acute respiratory syndrome coro ....
8717 MW185004.1 |Severe acute respiratory syndrome coro ....
8718 MW185007.1 |Severe acute respiratory syndrome coro ....
8719 MW185008.1 |Severe acute respiratory syndrome coro ....
8720 MW185009.1 |Severe acute respiratory syndrome coro ....
8721 MW185010.1 |Severe acute respiratory syndrome coro ....
8722 MW185012.1 |Severe acute respiratory syndrome coro ....
8723 MW185013.1 |Severe acute respiratory syndrome coro ....

8724	MW185014.1	|Severe acute respiratory syndrome coro	....	....	....	....	....	....
8725	MW185015.1	|Severe acute respiratory syndrome coro	....	....	....	....	....	....
8726	MW185016.1	|Severe acute respiratory syndrome coro	....	....	....	....	....	....
8727	MW185017.1	|Severe acute respiratory syndrome coro	....	....	....	....	....	....
8728	MW185018.1	|Severe acute respiratory syndrome coro	....	....	....	....	....	....
8729	MW185020.1	|Severe acute respiratory syndrome coro	....	....	....	....	....	....
8730	MW185023.1	|Severe acute respiratory syndrome coro	....	....	....	....	....	....
8731	MW185024.1	|Severe acute respiratory syndrome coro	....	....	....	....	....	....
8732	MW185026.1	|Severe acute respiratory syndrome coro	....	....	....	....	....	....
8733	MW185027.1	|Severe acute respiratory syndrome coro	....	....	....	....	....	....
8734	MW185028.1	|Severe acute respiratory syndrome coro	....	....	....	....	....	....
8735	MW185029.1	|Severe acute respiratory syndrome coro	....	....	....	....	....	....
8736	MW185030.1	|Severe acute respiratory syndrome coro	....	......A.............	....	....	....	....
8737	MW185031.1	|Severe acute respiratory syndrome coro	....	....	....	....	....	....
8738	MW185032.1	|Severe acute respiratory syndrome coro	....	....	....	....	....	....
8739	MW185034.1	|Severe acute respiratory syndrome coro	....	....	....	....	....	....
8740	MW185035.1	|Severe acute respiratory syndrome coro	....	....	....	....	....	....
8741	MW185037.1	|Severe acute respiratory syndrome coro	....	....	....	....	....	....
8742	MW185038.1	|Severe acute respiratory syndrome coro	....	....	....	....	....	....
8743	MW185040.1	|Severe acute respiratory syndrome coro	....	....	....	....	....	....
8744	MW185042.1	|Severe acute respiratory syndrome coro	....	....	....	....	....	....
8745	MW185044.1	|Severe acute respiratory syndrome coro	....	....	....	....	....	....
8746	MW185045.1	|Severe acute respiratory syndrome coro	....	....	....	....	....	....
8747	MW185046.1	|Severe acute respiratory syndrome coro	....	....	....	....	....	....
8748	MW185047.1	|Severe acute respiratory syndrome coro	....	....	....	....	....	....
8749	MW185048.1	|Severe acute respiratory syndrome coro	....	....	....	....	....	....
8750	MW185049.1	|Severe acute respiratory syndrome coro	....	....	....	....	....	....
8752	MW185052.1	|Severe acute respiratory syndrome coro	....	....	....	....	....	....
8753	MW185053.1	|Severe acute respiratory syndrome coro	....	....	....	....	....	....
8754	MW185055.1	|Severe acute respiratory syndrome coro	....	....	....	....	....	....
8755	MW185057.1	|Severe acute respiratory syndrome coro	....	....	....	....	....	....
8756	MW185058.1	|Severe acute respiratory syndrome coro	....	....	....	....	....	....
8757	MW185059.1	|Severe acute respiratory syndrome coro	....	....	....	....	....	....
8758	MW185061.1	|Severe acute respiratory syndrome coro	....	....	....	....	....	....
8759	MW185062.1	|Severe acute respiratory syndrome coro	....	....	....	....	....	....
8760	MW185064.1	|Severe acute respiratory syndrome coro	....	....	....	....	....	....
8761	MW185065.1	|Severe acute respiratory syndrome coro	....	....	....	....	....	....
8762	MW185066.1	|Severe acute respiratory syndrome coro	....	....	....	....	....	....
8763	MW185067.1	|Severe acute respiratory syndrome coro	....	....	....	....	....	....
8764	MW185069.1	|Severe acute respiratory syndrome coro	....	....	....	....	....	....
8765	MW185070.1	|Severe acute respiratory syndrome coro	....	....	....	....	....	....
8766	MW185071.1	|Severe acute respiratory syndrome coro	....	....	....	....	....	....
8767	MW185072.1	|Severe acute respiratory syndrome coro	....	....	....	....	....	....
8768	MW185073.1	|Severe acute respiratory syndrome coro	....	....	....	....	....	....
8769	MW185074.1	|Severe acute respiratory syndrome coro	....	....	....	....	....	....
8770	MW185075.1	|Severe acute respiratory syndrome coro	....	....	....	....	....	....
8771	MW185076.1	|Severe acute respiratory syndrome coro	....	....	....	....	....	....
8772	MW185077.1	|Severe acute respiratory syndrome coro	....	....	....	....	....	....
8773	MW185078.1	|Severe acute respiratory syndrome coro	....	....	....	....	....	....
8774	MW185080.1	|Severe acute respiratory syndrome coro	....	....	....	....	....	....
8775	MW185081.1	|Severe acute respiratory syndrome coro	....	....	....	....	....	....
8777	MW185086.1	|Severe acute respiratory syndrome coro	....	....	....	....	....	....
8778	MW185087.1	|Severe acute respiratory syndrome coro	....	....	....	....	....	....
8779	MW185088.1	|Severe acute respiratory syndrome coro	....	....	....	....	....	....
8780	MW185089.1	|Severe acute respiratory syndrome coro	....	....	....	....	....	....
8781	MW185090.1	|Severe acute respiratory syndrome coro	....	....	....	....	....	....
8782	MW185091.1	|Severe acute respiratory syndrome coro	....	....	....	....	....	....
8783	MW185093.1	|Severe acute respiratory syndrome coro	....	....	....	....	....	....
8784	MW185094.1	|Severe acute respiratory syndrome coro	....	....	....	....	....	....
8785	MW185096.1	|Severe acute respiratory syndrome coro	....	....	....	....	....	....
8786	MW185098.1	|Severe acute respiratory syndrome coro	....	....	....	....	....	....
8787	MW185099.1	|Severe acute respiratory syndrome coro	....	....	....	....	....	....
8788	MW185101.1	|Severe acute respiratory syndrome coro	....	....	....	....	....	....
8789	MW185102.1	|Severe acute respiratory syndrome coro	....	....	....	....	....	....
8792	MW185105.1	|Severe acute respiratory syndrome coro	....	....	....	....	....	....
8793	MW185106.1	|Severe acute respiratory syndrome coro	....	....	....	....	....	....
8794	MW185107.1	|Severe acute respiratory syndrome coro	....	....	....	....	....	....
8795	MW185108.1	|Severe acute respiratory syndrome coro	....	....	....	....	....	....
8796	MW185109.1	|Severe acute respiratory syndrome coro	....	....	....	....	....	....
8797	MW185111.1	|Severe acute respiratory syndrome coro	....	....	....	....	....	....
8798	MW185113.1	|Severe acute respiratory syndrome coro	....	....	....	....	....	....
8799	MW185114.1	|Severe acute respiratory syndrome coro	....	....	....	....	....	....
8800	MW185116.1	|Severe acute respiratory syndrome coro	....	....	....	....	....	....
8801	MW185118.1	|Severe acute respiratory syndrome coro	....	....	....	....	....	....
8802	MW185119.1	|Severe acute respiratory syndrome coro	....	....	....	....	....	....
8803	MW185120.1	|Severe acute respiratory syndrome coro	....	....	....	....	....	....
8804	MW185121.1	|Severe acute respiratory syndrome coro	....	....	....	....	....	....
8805	MW185122.1	|Severe acute respiratory syndrome coro	....	....	....	....	....	....

8806 MW185123.1 |Severe acute respiratory syndrome coro ....
8808 MW185126.1 |Severe acute respiratory syndrome coro ....
8809 MW185128.1 |Severe acute respiratory syndrome coro ....
8810 MW185129.1 |Severe acute respiratory syndrome coro ....
8811 MW185130.1 |Severe acute respiratory syndrome coro ....
8812 MW185131.1 |Severe acute respiratory syndrome coro ....
8813 MW185132.1 |Severe acute respiratory syndrome coro ....
8814 MW185133.1 |Severe acute respiratory syndrome coro ....
8815 MW185135.1 |Severe acute respiratory syndrome coro ....
8816 MW185136.1 |Severe acute respiratory syndrome coro ....
8817 MW185137.1 |Severe acute respiratory syndrome coro ....
8818 MW185138.1 |Severe acute respiratory syndrome coro ....
8819 MW185140.1 |Severe acute respiratory syndrome coro ....
8820 MW185141.1 |Severe acute respiratory syndrome coro ....
8821 MW185142.1 |Severe acute respiratory syndrome coro ....
8822 MW185143.1 |Severe acute respiratory syndrome coro ....
8823 MW185144.1 |Severe acute respiratory syndrome coro ....
8824 MW185145.1 |Severe acute respiratory syndrome coro ....
8825 MW185146.1 |Severe acute respiratory syndrome coro ....
8826 MW185148.1 |Severe acute respiratory syndrome coro ....
8827 MW185150.1 |Severe acute respiratory syndrome coro ....
8828 MW185151.1 |Severe acute respiratory syndrome coro ....
8829 MW185152.1 |Severe acute respiratory syndrome coro ....
8830 MW185153.1 |Severe acute respiratory syndrome coro ....
8831 MW185155.1 |Severe acute respiratory syndrome coro ....
8832 MW185156.1 |Severe acute respiratory syndrome coro ....
8833 MW185158.1 |Severe acute respiratory syndrome coro ....
8834 MW185159.1 |Severe acute respiratory syndrome coro ....
8835 MW185161.1 |Severe acute respiratory syndrome coro ....
8836 MW185162.1 |Severe acute respiratory syndrome coro ....
8837 MW185164.1 |Severe acute respiratory syndrome coro ....
8838 MW185167.1 |Severe acute respiratory syndrome coro ....
8839 MW185168.1 |Severe acute respiratory syndrome coro ....
8840 MW185169.1 |Severe acute respiratory syndrome coro ....
8841 MW185171.1 |Severe acute respiratory syndrome coro ....
8842 MW185173.1 |Severe acute respiratory syndrome coro ....
8843 MW185174.1 |Severe acute respiratory syndrome coro ....
8844 MW185175.1 |Severe acute respiratory syndrome coro ....
8845 MW185176.1 |Severe acute respiratory syndrome coro ....
8846 MW185178.1 |Severe acute respiratory syndrome coro ....
8847 MW185179.1 |Severe acute respiratory syndrome coro ....
8848 MW185180.1 |Severe acute respiratory syndrome coro ....
8849 MW185181.1 |Severe acute respiratory syndrome coro ....
8850 MW185182.1 |Severe acute respiratory syndrome coro ....
8851 MW185184.1 |Severe acute respiratory syndrome coro ....
8852 MW185185.1 |Severe acute respiratory syndrome coro ....
8853 MW185186.1 |Severe acute respiratory syndrome coro ....
8855 MW185189.1 |Severe acute respiratory syndrome coro ....
8856 MW185191.1 |Severe acute respiratory syndrome coro ....
8857 MW185192.1 |Severe acute respiratory syndrome coro ....
8858 MW185193.1 |Severe acute respiratory syndrome coro ....
8859 MW185194.1 |Severe acute respiratory syndrome coro ....
8860 MW185196.1 |Severe acute respiratory syndrome coro ....
8862 MW185198.1 |Severe acute respiratory syndrome coro ....
8863 MW185199.1 |Severe acute respiratory syndrome coro ....
8865 MW185201.1 |Severe acute respiratory syndrome coro ....
8866 MW185202.1 |Severe acute respiratory syndrome coro ....
8867 MW185203.1 |Severe acute respiratory syndrome coro ....
8868 MW185207.1 |Severe acute respiratory syndrome coro ....
8869 MW185208.1 |Severe acute respiratory syndrome coro ....
8870 MW185210.1 |Severe acute respiratory syndrome coro ....
8871 MW185212.1 |Severe acute respiratory syndrome coro ....
8872 MW185213.1 |Severe acute respiratory syndrome coro ....
8873 MW185215.1 |Severe acute respiratory syndrome coro ....
8874 MW185216.1 |Severe acute respiratory syndrome coro ....
8875 MW185217.1 |Severe acute respiratory syndrome coro ....
8876 MW185218.1 |Severe acute respiratory syndrome coro ....
8877 MW185219.1 |Severe acute respiratory syndrome coro ....
8878 MW185221.1 |Severe acute respiratory syndrome coro ....
8879 MW185222.1 |Severe acute respiratory syndrome coro ....
8880 MW185223.1 |Severe acute respiratory syndrome coro ....
8881 MW185224.1 |Severe acute respiratory syndrome coro ....
8882 MW185226.1 |Severe acute respiratory syndrome coro ....
8883 MW185227.1 |Severe acute respiratory syndrome coro ....
8884 MW185228.1 |Severe acute respiratory syndrome coro ....
8885 MW185231.1 |Severe acute respiratory syndrome coro ....
8887 MW185233.1 |Severe acute respiratory syndrome coro ....
8888 MW185234.1 |Severe acute respiratory syndrome coro ....

8889 MW185237.1 |Severe acute respiratory syndrome coro ···················· ···················· ···················· ···················· ···················· ····················
8890 MW185238.1 |Severe acute respiratory syndrome coro ···················· ···················· ···················· ···················· ···················· ····················
8891 MW185239.1 |Severe acute respiratory syndrome coro ···················· ···················· ···················· ···················· ···················· ····················
8892 MW185242.1 |Severe acute respiratory syndrome coro ···················· ···················· ···················· ···················· ···················· ····················
8893 MW185245.1 |Severe acute respiratory syndrome coro ···················· ···················· ···················· ···················· ···················· ····················
8894 MW185247.1 |Severe acute respiratory syndrome coro ···················· ···················· ···················· ···················· ···················· ····················
8895 MW185248.1 |Severe acute respiratory syndrome coro ···················· ···················· ···················· ···················· ···················· ····················
8896 MW185249.1 |Severe acute respiratory syndrome coro ···················· ···················· ···················· ···················· ···················· ····················
8897 MW185250.1 |Severe acute respiratory syndrome coro ···················· ···················· ···················· ···················· ···················· ····················
8898 MW185251.1 |Severe acute respiratory syndrome coro ···················· ···················· ···················· ···················· ···················· ····················
8899 MW185252.1 |Severe acute respiratory syndrome coro ···················· ···················· ···················· ···················· ···················· ····················
8900 MW185253.1 |Severe acute respiratory syndrome coro ···················· ···················· ···················· ···················· ···················· ····················
8901 MW185256.1 |Severe acute respiratory syndrome coro ···················· ···················· ···················· ···················· ···················· ····················
8902 MW185257.1 |Severe acute respiratory syndrome coro ···················· ···················· ···················· ···················· ···················· ····················
8903 MW185258.1 |Severe acute respiratory syndrome coro ···················· ···················· ···················· ···················· ···················· ····················
8904 MW185259.1 |Severe acute respiratory syndrome coro ···················· ···················· ···················· ···················· ···················· ····················
8905 MW185261.1 |Severe acute respiratory syndrome coro ···················· ···················· ···················· ···················· ···················· ····················
8906 MW185262.1 |Severe acute respiratory syndrome coro ···················· ···················· ···················· ···················· ···················· ····················
8907 MW185263.1 |Severe acute respiratory syndrome coro ···················· ···················· ···················· ···················· ···················· ····················
8908 MW185264.1 |Severe acute respiratory syndrome coro ···················· ···················· ···················· ···················· ···················· ····················
8909 MW185265.1 |Severe acute respiratory syndrome coro ···················· ···················· ···················· ···················· ···················· ····················
8910 MW185266.1 |Severe acute respiratory syndrome coro ···················· ···················· ···················· ···················· ···················· ····················
8911 MW185267.1 |Severe acute respiratory syndrome coro ···················· ···················· ···················· ···················· ······R············· ····················
8912 MW185268.1 |Severe acute respiratory syndrome coro ···················· ···················· ···················· ···················· ···················· ····················
8913 MW185269.1 |Severe acute respiratory syndrome coro ···················· ···················· ···················· ···················· ···················· ····················
8914 MW185271.1 |Severe acute respiratory syndrome coro ···················· ···················· ···················· ···················· ···················· ····················
8915 MW185272.1 |Severe acute respiratory syndrome coro ···················· ···················· ···················· ···················· ···················· ····················
8916 MW185273.1 |Severe acute respiratory syndrome coro ···················· ···················· ···················· ···················· ···················· ····················
8917 MW185274.1 |Severe acute respiratory syndrome coro ···················· ···················· ···················· ···················· ···················· ····················
8918 MW185275.1 |Severe acute respiratory syndrome coro ···················· ···················· ···················· ···················· ···················· ····················
8919 MW185277.1 |Severe acute respiratory syndrome coro ···················· ···················· ···················· ···················· ···················· ····················
8920 MW185278.1 |Severe acute respiratory syndrome coro ···················· ···················· ···················· ···················· ···················· ····················
8921 MW185279.1 |Severe acute respiratory syndrome coro ···················· ···················· ···················· ···················· ···················· ····················
8922 MW185280.1 |Severe acute respiratory syndrome coro ···················· ···················· ···················· ···················· ···················· ····················
8923 MW185282.1 |Severe acute respiratory syndrome coro ···················· ···················· ···················· ···················· ···················· ····················
8924 MW185283.1 |Severe acute respiratory syndrome coro ···················· ···················· ···················· ···················· ···················· ····················
8925 MW185285.1 |Severe acute respiratory syndrome coro ···················· ···················· ···················· ···················· ···················· ····················
8927 MW185287.1 |Severe acute respiratory syndrome coro ···················· ···················· ···················· ···················· ···················· ····················
8928 MW185288.1 |Severe acute respiratory syndrome coro ···················· ···················· ···················· ···················· ···················· ····················
8929 MW185289.1 |Severe acute respiratory syndrome coro ···················· ···················· ···················· ···················· ···················· ····················
8930 MW185290.1 |Severe acute respiratory syndrome coro ···················· ···················· ···················· ···················· ···················· ····················
8931 MW185291.1 |Severe acute respiratory syndrome coro ···················· ···················· ···················· ···················· ···················· ····················
8932 MW185293.1 |Severe acute respiratory syndrome coro ···················· ···················· ···················· ···················· ···················· ····················
8933 MW185295.1 |Severe acute respiratory syndrome coro ···················· ···················· ···················· ···················· ···················· ····················
8934 MW185296.1 |Severe acute respiratory syndrome coro ···················· ···················· ···················· ···················· ···················· ····················
8935 MW185297.1 |Severe acute respiratory syndrome coro ···················· ···················· ···················· ···················· ···················· ····················
8936 MW185300.1 |Severe acute respiratory syndrome coro ···················· ···················· ···················· ···················· ···················· ····················
8937 MW185301.1 |Severe acute respiratory syndrome coro ···················· ···················· ···················· ···················· ···················· ····················
8938 MW185303.1 |Severe acute respiratory syndrome coro ···················· ···················· ···················· ···················· ···················· ····················
8939 MW185306.1 |Severe acute respiratory syndrome coro ···················· ···················· ···················· ···················· ···················· ····················
8940 MW185308.1 |Severe acute respiratory syndrome coro ···················· ···················· ···················· ···················· ···················· ····················
8941 MW185309.1 |Severe acute respiratory syndrome coro ···················· ···················· ···················· ···················· ···················· ····················
8942 MW185310.1 |Severe acute respiratory syndrome coro ···················· ···················· ···················· ···················· ···················· ····················
8943 MW185311.1 |Severe acute respiratory syndrome coro ···················· ···················· ···················· ···················· ···················· ····················
8944 MW185312.1 |Severe acute respiratory syndrome coro ···················· ···················· ···················· ···················· ···················· ····················
8945 MW185313.1 |Severe acute respiratory syndrome coro ···················· ···················· ···················· ···················· ···················· ····················
8946 MW185314.1 |Severe acute respiratory syndrome coro ···················· ···················· ···················· ···················· ···················· ····················
8947 MW185316.1 |Severe acute respiratory syndrome coro ···················· ···················· ···················· ···················· ···················· ····················
8948 MW185318.1 |Severe acute respiratory syndrome coro ···················· ···················· ···················· ···················· ···················· ····················
8949 MW185320.1 |Severe acute respiratory syndrome coro ···················· ···················· ···················· ···················· ···················· ····················
8950 MW185321.1 |Severe acute respiratory syndrome coro ···················· ···················· ···················· ···················· ···················· ····················
8951 MW185322.1 |Severe acute respiratory syndrome coro ···················· ···················· ···················· ···················· ···················· ····················
8952 MW185323.1 |Severe acute respiratory syndrome coro ···················· ···················· ···················· ···················· ···················· ····················
8954 MW185327.1 |Severe acute respiratory syndrome coro ···················· ···················· ···················· ···················· ···················· ····················
8955 MW185329.1 |Severe acute respiratory syndrome coro ···················· ···················· ···················· ···················· ···················· ····················
8956 MW185330.1 |Severe acute respiratory syndrome coro ···················· ···················· ···················· ···················· ···················· ····················
8957 MW185334.1 |Severe acute respiratory syndrome coro ···················· ···················· ···················· ···················· ···················· ····················
8958 MW185335.1 |Severe acute respiratory syndrome coro ···················· ···················· ···················· ···················· ···················· ····················
8959 MW185336.1 |Severe acute respiratory syndrome coro ···················· ···················· ···················· ···················· ···················· ····················
8960 MW185339.1 |Severe acute respiratory syndrome coro ···················· ···················· ···················· ···················· ···················· ····················
8961 MW185343.1 |Severe acute respiratory syndrome coro ···················· ···················· ···················· ···················· ···················· ····················
8962 MW185344.1 |Severe acute respiratory syndrome coro ···················· ···················· ···················· ···················· ···················· ····················
8963 MW185345.1 |Severe acute respiratory syndrome coro ···················· ···················· ···················· ···················· ···················· ····················
8964 MW185346.1 |Severe acute respiratory syndrome coro ···················· ···················· ···················· ···················· ···················· ····················
8965 MW185347.1 |Severe acute respiratory syndrome coro ···················· ···················· ···················· ···················· ···················· NNN················· 1
8966 MW185348.1 |Severe acute respiratory syndrome coro ···················· ···················· ···················· ···················· ···················· ····················
8967 MW185349.1 |Severe acute respiratory syndrome coro ···················· ···················· ···················· ···················· NNN················· ···················· 1
8968 MW185350.1 |Severe acute respiratory syndrome coro ···················· ···················· ···················· ···················· ···················· ····················

```
8969 MW185351.1 |Severe acute respiratory syndrome coro    ....................  ....................  ....................  ....................  ....................  ....................
8970 MW185352.1 |Severe acute respiratory syndrome coro    ....................  ....................  ....................  ....................  ....................  ....................
8971 MW185354.1 |Severe acute respiratory syndrome coro    ....................  ....................  ....................  ....................  ....................  ....................
8972 MW185356.1 |Severe acute respiratory syndrome coro    ....................  ....................  ....................  ....................  ....................  ....................
8973 MW185357.1 |Severe acute respiratory syndrome coro    ....................  ....................  ....................  ....................  ....................  ....................
8974 MW185359.1 |Severe acute respiratory syndrome coro    ....................  ....................  ....................  ....................  ....................  ....................
8975 MW185360.1 |Severe acute respiratory syndrome coro    ....................  ....................  ....................  ....................  ....................  ....................
8976 MW185362.1 |Severe acute respiratory syndrome coro    ....................  ....................  ....................  ....................  ....................  ....................
8977 MW185363.1 |Severe acute respiratory syndrome coro    ....................  ....................  ....................  ....................  ....................  ....................
8978 MW185364.1 |Severe acute respiratory syndrome coro    ....................  ....................  ....................  ....................  ....................  ....................
8979 MW185365.1 |Severe acute respiratory syndrome coro    ....................  ....................  ....................  ....................  ....................  ....................
8981 MW185367.1 |Severe acute respiratory syndrome coro    ....................  ....................  ....................  ....................  ....................  ....................
8982 MW185368.1 |Severe acute respiratory syndrome coro    ....................  ....................  ....................  ....................  ....................  ....................
8983 MW185369.1 |Severe acute respiratory syndrome coro    ....................  ....................  ....................  ....................  ....................  ....................
8984 MW185370.1 |Severe acute respiratory syndrome coro    ....................  ....................  ....................  ....................  ....................  ....................
8985 MW185371.1 |Severe acute respiratory syndrome coro    ....................  ....................  ....................  ....................  ....................  ....................
8986 MW185372.1 |Severe acute respiratory syndrome coro    ....................  ....................  ....................  ....................  ....................  ....................
8987 MW185373.1 |Severe acute respiratory syndrome coro    ....................  ....................  ....................  ....................  ....................  ....................
8988 MW185374.1 |Severe acute respiratory syndrome coro    ....................  ....................  ....................  ....................  ....................  ....................
8989 MW185375.1 |Severe acute respiratory syndrome coro    ....................  ....................  ....................  ....................  ....................  ....................
8990 MW185376.1 |Severe acute respiratory syndrome coro    ....................  ....................  ....................  ....................  ....................  ....................
8991 MW185377.1 |Severe acute respiratory syndrome coro    ....................  ....................  ....................  ....................  ....................  ....................
8992 MW185379.1 |Severe acute respiratory syndrome coro    ....................  ....................  ....................  ....................  ....................  ....................
8993 MW185380.1 |Severe acute respiratory syndrome coro    ....................  ....................  ....................  ....................  ....................  ....................
8994 MW185381.1 |Severe acute respiratory syndrome coro    ....................  ....................  ....................  ....................  ....................  ....................
8995 MW185382.1 |Severe acute respiratory syndrome coro    ....................  ....................  ....................  ....................  ....................  ....................
8996 MW185386.1 |Severe acute respiratory syndrome coro    ....................  ....................  ....................  ....................  ....................  ....................
8997 MW185389.1 |Severe acute respiratory syndrome coro    ....................  ....................  ....................  ....................  ....................  ....................
8998 MW185391.1 |Severe acute respiratory syndrome coro    ....................  ....................  ....................  ....................  ....................  ....................
8999 MW185392.1 |Severe acute respiratory syndrome coro    ....................  ....................  ....................  ....................  ....................  ....................
9000 MW185393.1 |Severe acute respiratory syndrome coro    ....................  ....................  ....................  ....................  ....................  ....................
9001 MW185394.1 |Severe acute respiratory syndrome coro    ....................  ....................  ....................  ....................  ....................  ....................
9002 MW185395.1 |Severe acute respiratory syndrome coro    ....................  ....................  ....................  ....................  ....................  ....................
9003 MW185396.1 |Severe acute respiratory syndrome coro    ....................  ....................  ....................  ....................  ....................  ....................
9004 MW185397.1 |Severe acute respiratory syndrome coro    ....................  ....................  ....................  ....................  ....................  ....................
9005 MW185398.1 |Severe acute respiratory syndrome coro    ....................  ....................  ....................  ....................  ....................  ....................
9006 MW185399.1 |Severe acute respiratory syndrome coro    ....................  ....................  ....................  ....................  ....................  ....................
9007 MW185400.1 |Severe acute respiratory syndrome coro    ....................  ....................  ....................  ....................  ....................  ....................
9008 MW185401.1 |Severe acute respiratory syndrome coro    ....................  ....................  ....................  ....................  ....................  ....................
9009 MW185402.1 |Severe acute respiratory syndrome coro    ....................  ....................  ....................  ....................  ....................  ....................
9010 MW185403.1 |Severe acute respiratory syndrome coro    ....................  ....................  ....................  ....................  ....................  ....................
9011 MW185406.1 |Severe acute respiratory syndrome coro    ....................  ....................  ....................  ....................  ....................  ....................
9012 MW185408.1 |Severe acute respiratory syndrome coro    ....................  ....................  ....................  ....................  ....................  ....................
9013 MW185409.1 |Severe acute respiratory syndrome coro    ....................  ....................  ....................  ....................  ....................  ....................
9014 MW185410.1 |Severe acute respiratory syndrome coro    ....................  ....................  ....................  ....................  ....................  ....................
9015 MW185411.1 |Severe acute respiratory syndrome coro    ....................  ....................  ....................  ....................  ....................  ....................
9016 MW185414.1 |Severe acute respiratory syndrome coro    ....................  ....................  ....................  ....................  ....................  ....................
9017 MW185415.1 |Severe acute respiratory syndrome coro    ....................  ....................  ....................  ....................  ....................  ....................
9018 MW185416.1 |Severe acute respiratory syndrome coro    ....................  ....................  ....................  ....................  ....................  ....................
9019 MW185417.1 |Severe acute respiratory syndrome coro    ....................  ....................  ....................  ....................  ....................  ....................
9020 MW185418.1 |Severe acute respiratory syndrome coro    ....................  ....................  ....................  ....................  ....................  ....................
9021 MW185419.1 |Severe acute respiratory syndrome coro    ....................  ....................  ....................  ....................  ....................  ....................
9022 MW185421.1 |Severe acute respiratory syndrome coro    ....................  ....................  ....................  ....................  ....................  ....................
9024 MW185423.1 |Severe acute respiratory syndrome coro    ....................  ....................  ....................  ....................  ....................  ....................
9025 MW185424.1 |Severe acute respiratory syndrome coro    ....................  ....................  ....................  ....................  ....................  ....................
9026 MW185427.1 |Severe acute respiratory syndrome coro    ....................  ....................  ....................  ....................  ....................  ....................
9027 MW185428.1 |Severe acute respiratory syndrome coro    ....................  ....................  ....................  ....................  ....................  ....................
9028 MW185432.1 |Severe acute respiratory syndrome coro    ....................  ....................  ....................  ....................  ....................  ....................
9029 MW185433.1 |Severe acute respiratory syndrome coro    ....................  ....................  ....................  ....................  ....................  ....................
9030 MW185434.1 |Severe acute respiratory syndrome coro    ....................  ....................  ....................  ....................  ....................  ....................
9031 MW185435.1 |Severe acute respiratory syndrome coro    ....................  ....................  ....................  ....................  ....................  ....................
9032 MW185436.1 |Severe acute respiratory syndrome coro    ....................  ....................  ....................  ....................  ....................  ....................
9033 MW185437.1 |Severe acute respiratory syndrome coro    ....................  ....................  ....................  ....................  ....................  ....................
9034 MW185440.1 |Severe acute respiratory syndrome coro    ....................  ....................  ....................  ....................  ....................  ....................
9035 MW185441.1 |Severe acute respiratory syndrome coro    ....................  ....................  ....................  ....................  ....................  ....................
9036 MW185442.1 |Severe acute respiratory syndrome coro    ....................  ....................  ....................  ....................  ....................  ....................
9037 MW185443.1 |Severe acute respiratory syndrome coro    ....................  ....................  ....................  ....................  ....................  ....................
9038 MW185444.1 |Severe acute respiratory syndrome coro    ....................  ....................  ....................  ....................  ....................  ....................
9039 MW185445.1 |Severe acute respiratory syndrome coro    ....................  ....................  ....................  ....................  ....................  ....................
9040 MW185446.1 |Severe acute respiratory syndrome coro    ....................  ....................  ....................  ....................  ....................  ....................
9041 MW185448.1 |Severe acute respiratory syndrome coro    ....................  ....................  ....................  ....................  ....................  ....................
9042 MW185450.1 |Severe acute respiratory syndrome coro    ....................  ....................  ....................  ....................  ....................  ....................
9043 MW185451.1 |Severe acute respiratory syndrome coro    ....................  ....................  ....................  ....................  ....................  ....................
9044 MW185453.1 |Severe acute respiratory syndrome coro    ....................  ....................  ....................  ....................  ....................  ....................
9045 MW185454.1 |Severe acute respiratory syndrome coro    ....................  ....................  ....................  ....................  ....................  ....................
9046 MW185458.1 |Severe acute respiratory syndrome coro    ....................  ....................  ....................  ....................  ....................  ....................
9047 MW185459.1 |Severe acute respiratory syndrome coro    ....................  ....................  ....................  ....................  ....................  ....................
9048 MW185462.1 |Severe acute respiratory syndrome coro    ....................  ....................  ....................  ....................  ....................  ....................
```

9049 MW185463.1 |Severe acute respiratory syndrome coro ....
9050 MW185466.1 |Severe acute respiratory syndrome coro ....
9051 MW185467.1 |Severe acute respiratory syndrome coro ....
9052 MW185468.1 |Severe acute respiratory syndrome coro ....
9053 MW185469.1 |Severe acute respiratory syndrome coro ....
9054 MW185470.1 |Severe acute respiratory syndrome coro ....
9055 MW185471.1 |Severe acute respiratory syndrome coro ....
9056 MW185472.1 |Severe acute respiratory syndrome coro ....
9057 MW185477.1 |Severe acute respiratory syndrome coro ....
9058 MW185478.1 |Severe acute respiratory syndrome coro ....
9059 MW185479.1 |Severe acute respiratory syndrome coro ....
9060 MW185480.1 |Severe acute respiratory syndrome coro ....
9061 MW185482.1 |Severe acute respiratory syndrome coro ....
9062 MW185484.1 |Severe acute respiratory syndrome coro ....
9063 MW185488.1 |Severe acute respiratory syndrome coro ....
9064 MW185489.1 |Severe acute respiratory syndrome coro ....
9065 MW185490.1 |Severe acute respiratory syndrome coro ....
9066 MW185491.1 |Severe acute respiratory syndrome coro ....
9067 MW185492.1 |Severe acute respiratory syndrome coro ....
9068 MW185493.1 |Severe acute respiratory syndrome coro ....
9069 MW185494.1 |Severe acute respiratory syndrome coro ....
9070 MW185496.1 |Severe acute respiratory syndrome coro ....
9071 MW185497.1 |Severe acute respiratory syndrome coro ....
9072 MW185498.1 |Severe acute respiratory syndrome coro ....
9073 MW185499.1 |Severe acute respiratory syndrome coro ....
9074 MW185500.1 |Severe acute respiratory syndrome coro ....
9075 MW185501.1 |Severe acute respiratory syndrome coro ....
9076 MW185503.1 |Severe acute respiratory syndrome coro ....
9077 MW185505.1 |Severe acute respiratory syndrome coro ....
9078 MW185506.1 |Severe acute respiratory syndrome coro ....
9079 MW185507.1 |Severe acute respiratory syndrome coro ....
9080 MW185508.1 |Severe acute respiratory syndrome coro ....
9082 MW185510.1 |Severe acute respiratory syndrome coro ....
9083 MW185513.1 |Severe acute respiratory syndrome coro ....
9084 MW185514.1 |Severe acute respiratory syndrome coro ....
9085 MW185515.1 |Severe acute respiratory syndrome coro ....
9086 MW185516.1 |Severe acute respiratory syndrome coro ....
9087 MW185517.1 |Severe acute respiratory syndrome coro ....
9088 MW185518.1 |Severe acute respiratory syndrome coro ....
9089 MW185520.1 |Severe acute respiratory syndrome coro ....
9090 MW185521.1 |Severe acute respiratory syndrome coro ....
9091 MW185523.1 |Severe acute respiratory syndrome coro ....
9092 MW185525.1 |Severe acute respiratory syndrome coro ....
9093 MW185526.1 |Severe acute respiratory syndrome coro ....
9094 MW185527.1 |Severe acute respiratory syndrome coro ....
9095 MW185528.1 |Severe acute respiratory syndrome coro ....
9096 MW185531.1 |Severe acute respiratory syndrome coro ....
9097 MW185532.1 |Severe acute respiratory syndrome coro ....
9098 MW185534.1 |Severe acute respiratory syndrome coro ....
9099 MW185535.1 |Severe acute respiratory syndrome coro ....
9100 MW185538.1 |Severe acute respiratory syndrome coro ....
9101 MW185539.1 |Severe acute respiratory syndrome coro ....
9102 MW185540.1 |Severe acute respiratory syndrome coro ....
9103 MW185541.1 |Severe acute respiratory syndrome coro ....
9104 MW185542.1 |Severe acute respiratory syndrome coro ....
9105 MW185543.1 |Severe acute respiratory syndrome coro ....
9106 MW185546.1 |Severe acute respiratory syndrome coro ....
9107 MW185547.1 |Severe acute respiratory syndrome coro ....
9108 MW185548.1 |Severe acute respiratory syndrome coro ....
9109 MW185549.1 |Severe acute respiratory syndrome coro ....
9110 MW185550.1 |Severe acute respiratory syndrome coro ....
9111 MW185552.1 |Severe acute respiratory syndrome coro ....
9112 MW185553.1 |Severe acute respiratory syndrome coro ....
9113 MW185554.1 |Severe acute respiratory syndrome coro ....
9114 MW185556.1 |Severe acute respiratory syndrome coro ....
9115 MW185557.1 |Severe acute respiratory syndrome coro ....
9116 MW185558.1 |Severe acute respiratory syndrome coro ....
9117 MW185559.1 |Severe acute respiratory syndrome coro ....
9118 MW185560.1 |Severe acute respiratory syndrome coro ....
9119 MW185562.1 |Severe acute respiratory syndrome coro ....
9120 MW185563.1 |Severe acute respiratory syndrome coro ....
9121 MW185565.1 |Severe acute respiratory syndrome coro ....
9122 MW185568.1 |Severe acute respiratory syndrome coro ....
9123 MW185569.1 |Severe acute respiratory syndrome coro ....
9124 MW185570.1 |Severe acute respiratory syndrome coro ....
9125 MW185572.1 |Severe acute respiratory syndrome coro ....
9126 MW185573.1 |Severe acute respiratory syndrome coro ....
9127 MW185574.1 |Severe acute respiratory syndrome coro ....

9128	MW185575.1	Severe acute respiratory syndrome coro	····················	····················	····················	····················	····················	····················	
9129	MW185576.1	Severe acute respiratory syndrome coro	····················	····················	····················	····················	····················	····················	
9130	MW185578.1	Severe acute respiratory syndrome coro	····················	····················	····················	····················	····················	····················	
9131	MW185579.1	Severe acute respiratory syndrome coro	····················	····················	····················	····················	····················	····················	
9132	MW185580.1	Severe acute respiratory syndrome coro	····················	····················	····················	····················	····················	····················	
9133	MW185581.1	Severe acute respiratory syndrome coro	····················	····················	····················	····················	····················	····················	
9134	MW185582.1	Severe acute respiratory syndrome coro	····················	····················	····················	····················	····················	····················	
9135	MW185584.1	Severe acute respiratory syndrome coro	····················	····················	····················	····················	····················	····················	
9136	MW185585.1	Severe acute respiratory syndrome coro	····················	····················	····················	····················	····················	····················	
9137	MW185586.1	Severe acute respiratory syndrome coro	····················	····················	····················	····················	····················	····················	
9138	MW185587.1	Severe acute respiratory syndrome coro	····················	····················	····················	····················	····················	····················	
9139	MW185588.1	Severe acute respiratory syndrome coro	····················	····················	····················	····················	····················	····················	
9140	MW185589.1	Severe acute respiratory syndrome coro	····················	····················	····················	····················	····················	····················	
9141	MW185590.1	Severe acute respiratory syndrome coro	····················	····················	····················	····················	····················	····················	
9142	MW185593.1	Severe acute respiratory syndrome coro	····················	····················	····················	····················	····················	····················	
9143	MW185595.1	Severe acute respiratory syndrome coro	····················	····················	····················	····················	····················	····················	
9144	MW185596.1	Severe acute respiratory syndrome coro	····················	····················	····················	····················	····················	····················	
9145	MW185597.1	Severe acute respiratory syndrome coro	····················	····················	····················	····················	····················	····················	
9146	MW185598.1	Severe acute respiratory syndrome coro	····················	····················	····················	····················	····················	····················	
9147	MW185599.1	Severe acute respiratory syndrome coro	····················	····················	····················	····················	····················	····················	
9148	MW185600.1	Severe acute respiratory syndrome coro	····················	····················	····················	····················	····················	····················	
9149	MW185603.1	Severe acute respiratory syndrome coro	····················	····················	····················	····················	····················	····················	
9150	MW185606.1	Severe acute respiratory syndrome coro	····················	····················	····················	····················	····················	····················	
9151	MW185607.1	Severe acute respiratory syndrome coro	····················	····················	····················	····················	····················	····················	
9152	MW185608.1	Severe acute respiratory syndrome coro	····················	····················	····················	····················	····················	····················	
9153	MW185609.1	Severe acute respiratory syndrome coro	····················	····················	····················	····················	····················	····················	
9154	MW185611.1	Severe acute respiratory syndrome coro	····················	····················	····················	····················	····················	····················	
9155	MW185614.1	Severe acute respiratory syndrome coro	····················	····················	····················	····················	····················	····················	
9156	MW185615.1	Severe acute respiratory syndrome coro	····················	····················	····················	····················	····················	····················	
9157	MW185617.1	Severe acute respiratory syndrome coro	····················	····················	····················	····················	····················	····················	
9158	MW185618.1	Severe acute respiratory syndrome coro	····················	····················	····················	····················	····················	····················	
9159	MW185619.1	Severe acute respiratory syndrome coro	····················	····················	····················	····················	····················	····················	
9160	MW185620.1	Severe acute respiratory syndrome coro	····················	····················	····················	····················	····················	····················	
9161	MW185621.1	Severe acute respiratory syndrome coro	····················	····················	····················	····················	····················	····················	
9162	MW185624.1	Severe acute respiratory syndrome coro	····················	····················	····················	····················	····················	····················	
9163	MW185625.1	Severe acute respiratory syndrome coro	····················	····················	····················	····················	····················	····················	
9164	MW185627.1	Severe acute respiratory syndrome coro	····················	····················	····················	····················	····················	····················	
9165	MW185628.1	Severe acute respiratory syndrome coro	····················	····················	····················	····················	····················	····················	
9166	MW185629.1	Severe acute respiratory syndrome coro	····················	····················	····················	····················	····················	····················	
9167	MW185630.1	Severe acute respiratory syndrome coro	····················	····················	····················	····················	····················	····················	
9168	MW185632.1	Severe acute respiratory syndrome coro	····················	······A·············	····················	····················	····················	····················	
9169	MW185636.1	Severe acute respiratory syndrome coro	····················	····················	····················	····················	····················	····················	
9170	MW185638.1	Severe acute respiratory syndrome coro	····················	····················	····················	····················	····················	····················	
9171	MW185640.1	Severe acute respiratory syndrome coro	····················	····················	····················	····················	····················	····················	
9172	MW185641.1	Severe acute respiratory syndrome coro	····················	····················	····················	····················	····················	····················	
9173	MW185642.1	Severe acute respiratory syndrome coro	····················	····················	····················	····················	····················	····················	
9174	MW185643.1	Severe acute respiratory syndrome coro	····················	····················	····················	····················	····················	····················	
9175	MW185645.1	Severe acute respiratory syndrome coro	····················	····················	····················	····················	····················	····················	
9176	MW185646.1	Severe acute respiratory syndrome coro	····················	····················	····················	····················	····················	····················	
9177	MW185647.1	Severe acute respiratory syndrome coro	····················	····················	····················	····················	····················	····················	
9178	MW185648.1	Severe acute respiratory syndrome coro	····················	····················	····················	····················	····················	····················	
9179	MW185649.1	Severe acute respiratory syndrome coro	····················	····················	····················	····················	····················	····················	
9180	MW185650.1	Severe acute respiratory syndrome coro	····················	····················	····················	····················	····················	····················	
9181	MW185652.1	Severe acute respiratory syndrome coro	····················	····················	····················	····················	····················	····················	
9182	MW185653.1	Severe acute respiratory syndrome coro	····················	····················	····················	····················	····················	····················	
9183	MW185654.1	Severe acute respiratory syndrome coro	····················	····················	····················	····················	····················	····················	
9184	MW185655.1	Severe acute respiratory syndrome coro	····················	····················	····················	····················	····················	····················	
9185	MW185657.1	Severe acute respiratory syndrome coro	····················	····················	····················	····················	····················	····················	
9186	MW185658.1	Severe acute respiratory syndrome coro	····················	····················	····················	····················	····················	····················	
9187	MW185659.1	Severe acute respiratory syndrome coro	····················	····················	····················	····················	····················	····················	
9188	MW185660.1	Severe acute respiratory syndrome coro	····················	····················	····················	····················	····················	····················	
9189	MW185661.1	Severe acute respiratory syndrome coro	····················	····················	····················	····················	····················	····················	
9190	MW185662.1	Severe acute respiratory syndrome coro	····················	····················	····················	····················	····················	····················	
9191	MW185663.1	Severe acute respiratory syndrome coro	····················	····················	····················	····················	····················	····················	
9192	MW185664.1	Severe acute respiratory syndrome coro	····················	····················	····················	····················	····················	····················	
9193	MW185665.1	Severe acute respiratory syndrome coro	····················	····················	····················	····················	····················	····················	
9194	MW185666.1	Severe acute respiratory syndrome coro	····················	····················	····················	····················	··················NN	····················	1
9195	MW185667.1	Severe acute respiratory syndrome coro	····················	····················	····················	····················	····················	····················	
9196	MW185669.1	Severe acute respiratory syndrome coro	····················	····················	····················	····················	····················	····················	
9197	MW185670.1	Severe acute respiratory syndrome coro	····················	····················	····················	····················	····················	····················	
9198	MW185671.1	Severe acute respiratory syndrome coro	····················	····················	····················	····················	····················	····················	
9199	MW185672.1	Severe acute respiratory syndrome coro	····················	····················	····················	····················	····················	····················	
9200	MW185674.1	Severe acute respiratory syndrome coro	····················	····················	····················	····················	····················	····················	
9201	MW185675.1	Severe acute respiratory syndrome coro	····················	····················	····················	····················	····················	····················	
9202	MW185676.1	Severe acute respiratory syndrome coro	····················	····················	····················	····················	····················	····················	
9203	MW185677.1	Severe acute respiratory syndrome coro	····················	····················	····················	····················	····················	····················	
9204	MW185678.1	Severe acute respiratory syndrome coro	····················	····················	····················	····················	····················	····················	
9205	MW185679.1	Severe acute respiratory syndrome coro	····················	····················	····················	····················	····················	····················	

9206 MW185680.1 |Severe acute respiratory syndrome coro ···················· ···················· ···················· ···················· ···················· ····················
9207 MW185681.1 |Severe acute respiratory syndrome coro ···················· ···················· ···················· ···················· ···················· ····················
9208 MW185682.1 |Severe acute respiratory syndrome coro ···················· ···················· ···················· ···················· ···················· ····················
9209 MW185683.1 |Severe acute respiratory syndrome coro ···················· ···················· ···················· ···················· ···················· ····················
9210 MW185685.1 |Severe acute respiratory syndrome coro ···················· ···················· ···················· ···················· ···················· ····················
9211 MW185686.1 |Severe acute respiratory syndrome coro ···················· ···················· ···················· ···················· ···················· ····················
9212 MW185688.1 |Severe acute respiratory syndrome coro ···················· ···················· ···················· ···················· ···················· ····················
9213 MW185691.1 |Severe acute respiratory syndrome coro ···················· ···················· ···················· ···················· ···················· ····················
9214 MW185692.1 |Severe acute respiratory syndrome coro ···················· ···················· ···················· ···················· ···················· ····················
9215 MW185694.1 |Severe acute respiratory syndrome coro ···················· ···················· ···················· ···················· ···················· ····················
9216 MW185695.1 |Severe acute respiratory syndrome coro ···················· ···················· ···················· ···················· ···················· ····················
9217 MW185696.1 |Severe acute respiratory syndrome coro ···················· ···················· ···················· ···················· ···················· ····················
9218 MW185697.1 |Severe acute respiratory syndrome coro ···················· ···················· ···················· ···················· ···················· ····················
9219 MW185698.1 |Severe acute respiratory syndrome coro ···················· ···················· ···················· ···················· ···················· ····················
9220 MW185699.1 |Severe acute respiratory syndrome coro ···················· ···················· ···················· ···················· ···················· ····················
9221 MW185700.1 |Severe acute respiratory syndrome coro ···················· ···················· ···················· ···················· ···················· ····················
9222 MW185701.1 |Severe acute respiratory syndrome coro ···················· ···················· ···················· ···················· ···················· ····················
9223 MW185703.1 |Severe acute respiratory syndrome coro ···················· ···················· ···················· ···················· ···················· ····················
9224 MW185704.1 |Severe acute respiratory syndrome coro ···················· ···················· ···················· ···················· ···················· ····················
9225 MW185705.1 |Severe acute respiratory syndrome coro ···················· ···················· ···················· ···················· ···················· ····················
9226 MW185706.1 |Severe acute respiratory syndrome coro ···················· ···················· ···················· ···················· ···················· ····················
9227 MW185707.1 |Severe acute respiratory syndrome coro ···················· ···················· ···················· ···················· ···················· ····················
9228 MW185708.1 |Severe acute respiratory syndrome coro ···················· ···················· ···················· ···················· ···················· ····················
9229 MW185709.1 |Severe acute respiratory syndrome coro ···················· ···················· ···················· ···················· ···················· ····················
9230 MW185710.1 |Severe acute respiratory syndrome coro ···················· ······A············· ···················· ···················· ···················· ····················
9231 MW185711.1 |Severe acute respiratory syndrome coro ···················· ···················· ···················· ···················· ···················· ····················
9232 MW185713.1 |Severe acute respiratory syndrome coro ···················· ···················· ···················· ···················· ···················· ····················
9233 MW185714.1 |Severe acute respiratory syndrome coro ···················· ···················· ···················· ···················· ···················· ····················
9234 MW185715.1 |Severe acute respiratory syndrome coro ···················· ···················· ···················· ···················· ···················· ····················
9235 MW185716.1 |Severe acute respiratory syndrome coro ···················· ···················· ···················· ···················· ···················· ····················
9236 MW185717.1 |Severe acute respiratory syndrome coro ···················· ···················· ···················· ···················· ···················· ····················
9237 MW185718.1 |Severe acute respiratory syndrome coro ···················· ···················· ···················· ···················· ···················· ····················
9238 MW185721.1 |Severe acute respiratory syndrome coro ···················· ···················· ···················· ···················· ···················· ····················
9239 MW185722.1 |Severe acute respiratory syndrome coro ···················· ···················· ···················· ···················· ···················· ····················
9240 MW185723.1 |Severe acute respiratory syndrome coro ···················· ···················· ···················· ···················· ···················· ····················
9241 MW185725.1 |Severe acute respiratory syndrome coro ···················· ···················· ···················· ···················· ···················· ····················
9242 MW185726.1 |Severe acute respiratory syndrome coro ···················· ···················· ···················· ···················· ···················· ····················
9243 MW185729.1 |Severe acute respiratory syndrome coro ···················· ···················· ···················· ···················· ···················· ····················
9244 MW185730.1 |Severe acute respiratory syndrome coro ···················· ···················· ···················· ···················· ···················· ····················
9245 MW185731.1 |Severe acute respiratory syndrome coro ···················· ···················· ···················· ···················· ···················· ····················
9246 MW185732.1 |Severe acute respiratory syndrome coro ···················· ···················· ···················· ···················· ···················· ····················
9247 MW185733.1 |Severe acute respiratory syndrome coro ···················· ···················· ···················· ···················· ···················· ····················
9248 MW185735.1 |Severe acute respiratory syndrome coro ···················· ···················· ···················· ···················· ···················· ····················
9249 MW185737.1 |Severe acute respiratory syndrome coro ···················· ···················· ···················· ···················· ···················· ····················
9250 MW185738.1 |Severe acute respiratory syndrome coro ···················· ···················· ···················· ···················· ···················· ····················
9251 MW185739.1 |Severe acute respiratory syndrome coro ···················· ···················· ···················· ···················· ···················· ····················
9252 MW185740.1 |Severe acute respiratory syndrome coro ···················· ···················· ···················· ···················· ···················· ····················
9253 MW185741.1 |Severe acute respiratory syndrome coro ···················· ···················· ···················· ···················· ···················· ····················
9254 MW185742.1 |Severe acute respiratory syndrome coro ···················· ···················· ···················· ···················· ···················· ····················
9255 MW185743.1 |Severe acute respiratory syndrome coro ···················· ···················· ···················· ···················· ···················· ····················
9256 MW185746.1 |Severe acute respiratory syndrome coro ···················· ···················· ···················· ···················· ···················· ····················
9257 MW185747.1 |Severe acute respiratory syndrome coro ···················· ···················· ···················· ···················· ···················· ····················
9258 MW185748.1 |Severe acute respiratory syndrome coro ···················· ···················· ···················· ···················· ···················· ····················
9259 MW185749.1 |Severe acute respiratory syndrome coro ···················· ···················· ···················· ···················· ···················· ····················
9260 MW185751.1 |Severe acute respiratory syndrome coro ···················· ···················· ···················· ···················· ···················· ····················
9261 MW185752.1 |Severe acute respiratory syndrome coro ···················· ···················· ···················· ···················· ···················· ····················
9262 MW185753.1 |Severe acute respiratory syndrome coro ···················· ···················· ···················· ···················· ···················· ····················
9263 MW185755.1 |Severe acute respiratory syndrome coro ···················· ···················· ···················· ···················· ···················· ····················
9264 MW185757.1 |Severe acute respiratory syndrome coro ···················· ···················· ···················· ···················· ···················· ····················
9265 MW185758.1 |Severe acute respiratory syndrome coro ···················· ···················· ···················· ···················· ···················· ····················
9266 MW185759.1 |Severe acute respiratory syndrome coro ···················· ···················· ···················· ···················· ···················· ····················
9267 MW185760.1 |Severe acute respiratory syndrome coro ···················· ···················· ···················· ···················· ···················· ····················
9268 MW185761.1 |Severe acute respiratory syndrome coro ···················· ···················· ···················· ···················· ···················· ····················
9269 MW185763.1 |Severe acute respiratory syndrome coro ···················· ···················· ···················· ···················· ···················· ····················
9270 MW185764.1 |Severe acute respiratory syndrome coro ···················· ···················· ···················· ···················· ···················· ····················
9271 MW185765.1 |Severe acute respiratory syndrome coro ···················· ···················· ···················· ···················· ···················· ····················
9272 MW185766.1 |Severe acute respiratory syndrome coro ···················· ···················· ···················· ···················· ···················· ····················
9273 MW185767.1 |Severe acute respiratory syndrome coro ···················· ···················· ···················· ···················· ···················· ····················
9274 MW185768.1 |Severe acute respiratory syndrome coro ···················· ···················· ···················· ···················· ···················· ····················
9275 MW185769.1 |Severe acute respiratory syndrome coro ···················· ···················· ···················· ···················· ···················· ····················
9276 MW185770.1 |Severe acute respiratory syndrome coro ···················· ···················· ···················· ···················· ···················· ····················
9277 MW185771.1 |Severe acute respiratory syndrome coro ···················· ···················· ···················· ···················· ···················· ····················
9278 MW185772.1 |Severe acute respiratory syndrome coro ···················· ···················· ···················· ···················· ···················· ····················
9279 MW185773.1 |Severe acute respiratory syndrome coro ···················· ···················· ···················· ···················· ···················· ····················
9281 MW185775.1 |Severe acute respiratory syndrome coro ···················· ···················· ···················· ···················· ···················· ····················
9282 MW185776.1 |Severe acute respiratory syndrome coro ···················· ···················· ···················· ···················· ···················· ····················
9283 MW185777.1 |Severe acute respiratory syndrome coro ···················· ···················· ···················· ···················· ···················· ····················
9284 MW185778.1 |Severe acute respiratory syndrome coro ···················· ···················· ···················· ···················· ···················· ····················

```
9285 MW185779.1 |Severe acute respiratory syndrome coro    ....................  ....................  ....................  ....................  ....................  ....................
9286 MW173245.1 |Severe acute respiratory syndrome coro    ....................  ....................  ....................  ....................  ....................  ....................
9287 MW173250.1 |Severe acute respiratory syndrome coro    ....................  .A..................  ....................  ....................  ....................  ....................
9288 MW173252.1 |Severe acute respiratory syndrome coro    ....................  ....................  ....................  ....................  ....................  ....................
9289 MW173253.1 |Severe acute respiratory syndrome coro    ....................  ....................  ....................  ....................  ....................  ....................
9290 MW173254.1 |Severe acute respiratory syndrome coro    ....................  ....................  ....................  ....................  ....................  ....................
9291 MW173298.1 |Severe acute respiratory syndrome coro    ....................  .A..................  ....................  ....................  ....................  ..A.................
9292 MW173401.1 |Severe acute respiratory syndrome coro    ....................  ....................  ....................  ....................  ....................  ....................
9293 MW173402.1 |Severe acute respiratory syndrome coro    ....................  .............A......  ....................  ....................  ....................  ....................
9294 MW173404.1 |Severe acute respiratory syndrome coro    ....................  .A..................  ....................  ....................  ....................  ....................
9295 MW173406.1 |Severe acute respiratory syndrome coro    ....................  ....................  ....................  ....................  ....................  ....................
9296 MW173407.1 |Severe acute respiratory syndrome coro    ....................  ....................  ....................  ....................  ....................  ....................
9297 MW173408.1 |Severe acute respiratory syndrome coro    ....................  ....................  ....................  ....................  ....................  ....................
9298 MW173409.1 |Severe acute respiratory syndrome coro    ....................  ....................  ....................  ....................  ....................  ....................
9299 MW173410.1 |Severe acute respiratory syndrome coro    ....................  ....................  ....................  ....................  ....................  ....................
9300 MW173412.1 |Severe acute respiratory syndrome coro    ....................  ....................  ....................  ....................  ....................  ....................
9301 MW173414.1 |Severe acute respiratory syndrome coro    ....................  ....................  ....................  ....................  ....................  ....................
9302 MW173417.1 |Severe acute respiratory syndrome coro    ....................  ....................  ....................  ....................  ....................  ....................
9303 MW173419.1 |Severe acute respiratory syndrome coro    ....................  .A..................  ....................  ....................  ....................  ....................
9304 MW173423.1 |Severe acute respiratory syndrome coro    ....................  .A..................  ....................  ....................  ....................  ....................
9305 MW173426.1 |Severe acute respiratory syndrome coro    ....................  .A..................  ....................  ....................  ....................  ....................
9306 MW173427.1 |Severe acute respiratory syndrome coro    ....................  ....................  ....................  ....................  ....................  ....................
9307 MW173433.1 |Severe acute respiratory syndrome coro    ....................  ....................  ....................  ....................  ....................  ....................
9308 MW173436.1 |Severe acute respiratory syndrome coro    ....................  ....................  ....................  ....................  ....................  ....................
9309 MW173440.1 |Severe acute respiratory syndrome coro    ....................  ....................  ....................  ....................  ....................  ....................
9310 MW173441.1 |Severe acute respiratory syndrome coro    ....................  ....................  ....................  ....................  ....................  ....................
9311 MW173442.1 |Severe acute respiratory syndrome coro    ....................  ....................  ....................  ....................  ....................  ....................
9312 MW173443.1 |Severe acute respiratory syndrome coro    ....................  ....................  ....................  ....................  ....................  ....................
9313 MW173444.1 |Severe acute respiratory syndrome coro    ....................  ....................  ....................  ....................  ....................  ....................
9315 MW173448.1 |Severe acute respiratory syndrome coro    ....................  ....................  ....................  ....................  ....................  ....................
9316 MW173452.1 |Severe acute respiratory syndrome coro    ....................  ....................  ....................  ....................  ....................  ....................
9317 MW173453.1 |Severe acute respiratory syndrome coro    ....................  ....................  ....................  ....................  ....................  ....................
9318 MW173454.1 |Severe acute respiratory syndrome coro    ....................  ....................  ....................  ....................  ....................  ....................
9319 MW173459.1 |Severe acute respiratory syndrome coro    ....................  ....................  ....................  ....................  ....................  ....................
9320 MW173460.1 |Severe acute respiratory syndrome coro    ....................  .A..................  ....................  ....................  ....................  ..A.................
9321 MW173463.1 |Severe acute respiratory syndrome coro    ....................  .A..................  ....................  ....................  ....................  ..A.................
9322 MW180935.1 |Severe acute respiratory syndrome coro    ....................  ....................  ....................  ....................  ....................  ....................
9323 MW180936.1 |Severe acute respiratory syndrome coro    ....................  ....................  ....................  ....................  ....................  ....................
9324 MW165854.1 |Severe acute respiratory syndrome coro    ....................  .A..................  ....................  ....................  ....................  ..A.................
9325 MW165857.1 |Severe acute respiratory syndrome coro    ....................  .A..................  ....................  ....................  ....................  ..A.................
9326 MW165861.1 |Severe acute respiratory syndrome coro    ....................  .A..................  ....................  ....................  ....................  ..A.................
9327 MW165862.1 |Severe acute respiratory syndrome coro    ....................  .A..................  ....................  ....................  ....................  ....................
9328 MW165865.1 |Severe acute respiratory syndrome coro    ....................  .A..................  ....................  ....................  ....................  ..A.................
9329 MW165869.1 |Severe acute respiratory syndrome coro    ....................  ....................  ....................  ....................  ....................  ....................
9330 MW165872.1 |Severe acute respiratory syndrome coro    ....................  ....................  ....................  ....................  ....................  ....................
9331 MW165873.1 |Severe acute respiratory syndrome coro    ....................  .A..................  ....................  ....................  ....................  ..A.................
9332 MW165876.1 |Severe acute respiratory syndrome coro    ....................  .A..................  ....................  ....................  ....................  ....................
9333 MW165885.1 |Severe acute respiratory syndrome coro    ....................  .A..................  ....................  ....................  ....................  ..A.................
9334 MW166053.1 |Severe acute respiratory syndrome coro    ....................  ....................  ....................  ....................  ....................  ....................
9335 MW166054.1 |Severe acute respiratory syndrome coro    ....................  ....................  ....................  ....................  ....................  ....................
9336 MW166055.1 |Severe acute respiratory syndrome coro    ....................  ....................  ....................  ....................  ....................  ....................
9337 MW166056.1 |Severe acute respiratory syndrome coro    ....................  ....................  ....................  ....................  ....................  ....................
9338 MW166064.1 |Severe acute respiratory syndrome coro    ....................  ....................  ....................  ....................  ....................  ....................
9339 MW166065.1 |Severe acute respiratory syndrome coro    ....................  ....................  ....................  ....................  ....................  ....................
9340 MW166066.1 |Severe acute respiratory syndrome coro    ....................  ....................  ....................  ....................  ....................  ....................
9342 MW166069.1 |Severe acute respiratory syndrome coro    ....................  ....................  ....................  ....................  ....................  ....................
9343 MW166070.1 |Severe acute respiratory syndrome coro    ....................  ....................  ....................  ....................  ....................  ....................
9345 MW166072.1 |Severe acute respiratory syndrome coro    ....................  ....................  ....................  ....................  ....................  ....................
9346 MW166073.1 |Severe acute respiratory syndrome coro    ....................  ....................  ....................  ....................  ....................  ....................
9347 MW166075.1 |Severe acute respiratory syndrome coro    ....................  ....................  ....................  ....................  ....................  ....................
9348 MW166077.1 |Severe acute respiratory syndrome coro    ....................  ....................  ....................  ....................  ....................  ....................
9349 MW166078.1 |Severe acute respiratory syndrome coro    ....................  ....................  ....................  ....................  ....................  ....................
9350 MW166079.1 |Severe acute respiratory syndrome coro    ....................  ....................  ....................  ....................  ....................  ....................
9351 MW166080.1 |Severe acute respiratory syndrome coro    ....................  ....................  ....................  ....................  ....................  ....................
9352 MW166081.1 |Severe acute respiratory syndrome coro    ....................  ....................  ....................  ....................  ....................  ....................
9353 MW166082.1 |Severe acute respiratory syndrome coro    ....................  ....................  ....................  ....................  ....................  ....................
9354 MW166083.1 |Severe acute respiratory syndrome coro    ....................  ....................  ....................  ....................  ....................  ....................
9355 MW166084.1 |Severe acute respiratory syndrome coro    ....................  ....................  ....................  ....................  ....................  ....................
9356 MW166086.1 |Severe acute respiratory syndrome coro    ....................  ....................  ....................  ....................  ....................  ....................
9357 MW166087.1 |Severe acute respiratory syndrome coro    ....................  ....................  ....................  ....................  ....................  ....................
9358 MW166088.1 |Severe acute respiratory syndrome coro    ....................  ....................  ....................  ....................  ....................  ....................
9359 MW166092.1 |Severe acute respiratory syndrome coro    ....................  ....................  ....................  ....................  ....................  ....................
9361 MW166096.1 |Severe acute respiratory syndrome coro    ....................  ....................  ....................  ....................  ....................  ....................
9362 MW166097.1 |Severe acute respiratory syndrome coro    ....................  ....................  ....................  ....................  ....................  ....................
9363 MW166098.1 |Severe acute respiratory syndrome coro    ....................  ....................  ....................  ....................  ....................  ....................
9364 MW166108.1 |Severe acute respiratory syndrome coro    ....................  ....................  ....................  ....................  ....................  ....................
9365 MW166110.1 |Severe acute respiratory syndrome coro    ....................  ....................  ....................  ....................  ....................  ....................
9366 MW166113.1 |Severe acute respiratory syndrome coro    ....................  ....................  ....................  ....................  ....................  ....................
```

9367 MW166116.1 |Severe acute respiratory syndrome coro ···················· ···················· ···················· ···················· ···················· ····················
9368 MW166117.1 |Severe acute respiratory syndrome coro ···················· ···················· ···················· ···················· ···················· ····················
9369 MW166118.1 |Severe acute respiratory syndrome coro ···················· ···················· ···················· ···················· ···················· ····················
9370 MW166119.1 |Severe acute respiratory syndrome coro ···················· ···················· ···················· ···················· ···················· ····················
9371 MW166120.1 |Severe acute respiratory syndrome coro ··············G····· ···················· ···················· ···················· ···················· ····················
9372 MW166122.1 |Severe acute respiratory syndrome coro ···················· ···················· ···················· ···················· ···················· ····················
9373 MW166123.1 |Severe acute respiratory syndrome coro ···················· ·········A·········· ···················· ···················· ···················· ····················
9374 MW166125.1 |Severe acute respiratory syndrome coro ···················· ···················· ···················· ···················· ···················· ····················
9375 MW166126.1 |Severe acute respiratory syndrome coro ···················· ···················· ···················· ···················· ···················· ····················
9376 MW166127.1 |Severe acute respiratory syndrome coro ···················· ···················· ···················· ···················· ···················· ····················
9377 MW166129.1 |Severe acute respiratory syndrome coro ···················· ···················· ···················· ···················· ···················· ····················
9378 MW166130.1 |Severe acute respiratory syndrome coro ···················· ···················· ···················· ···················· ···················· ····················
9379 MW166131.1 |Severe acute respiratory syndrome coro ···················· ···················· ···················· ···················· ···················· ····················
9380 MW166133.1 |Severe acute respiratory syndrome coro ···················· ···················· ···················· ···················· ···················· ····················
9381 MW166136.1 |Severe acute respiratory syndrome coro ···················· ···················· ···················· ···················· ···················· ····················
9382 MW166138.1 |Severe acute respiratory syndrome coro ···················· ···················· ···················· ···················· ···················· ····················
9383 MW166139.1 |Severe acute respiratory syndrome coro ···················· ···················· ···················· ···················· ···················· ····················
9384 MW166141.1 |Severe acute respiratory syndrome coro ···················· ···················· ···················· ···················· ···················· ····················
9385 MW166142.1 |Severe acute respiratory syndrome coro ···················· ···················· ···················· ···················· ···················· ····················
9386 MW166144.1 |Severe acute respiratory syndrome coro ···················· ···················· ···················· ···················· ···················· ····················
9387 MW166145.1 |Severe acute respiratory syndrome coro ···················· ···················· ···················· ···················· ···················· ····················
9388 MW166149.1 |Severe acute respiratory syndrome coro ···················· ···················· ···················· ···················· ···················· ····················
9389 MW166150.1 |Severe acute respiratory syndrome coro ···················· ···················· ···················· ···················· ···················· ····················
9390 MW166151.1 |Severe acute respiratory syndrome coro ···················· ···················· ···················· ···················· ···················· ····················
9391 MW166152.1 |Severe acute respiratory syndrome coro ···················· ···················· ···················· ···················· ···················· ····················
9392 MW166154.1 |Severe acute respiratory syndrome coro ···················· ···················· ···················· ···················· ···················· ····················
9393 MW166165.1 |Severe acute respiratory syndrome coro ···················· ···················· ···················· ···················· ···················· ····················
9394 MW166167.1 |Severe acute respiratory syndrome coro ···················· ···················· ···················· ···················· ···················· ····················
9395 MW166214.1 |Severe acute respiratory syndrome coro ···················· ·A·················· ···················· ···················· ···················· ···A················
9396 MW166221.1 |Severe acute respiratory syndrome coro ···················· ·A·················· ···················· ···················· ···················· ···A················
9397 MW166349.1 |Severe acute respiratory syndrome coro ···················· ·A·················· ···················· ···················· ···················· ···A················
9398 MW166864.1 |Severe acute respiratory syndrome coro ···················· ···················· ···················· ···················· ···················· ····················
9399 MW167078.1 |Severe acute respiratory syndrome coro ···················· ·A·················· ···················· ···················· ···················· ···A················
9400 MW167112.1 |Severe acute respiratory syndrome coro ···················· ·A·················· ···················· ···················· ···················· ···A················
9401 MW172414.1 |Severe acute respiratory syndrome coro ···················· ···················· ···················· ···················· ···················· ····················
9402 MW172415.1 |Severe acute respiratory syndrome coro ···················· ···················· ···················· ···················· ···················· ····················
9403 MW172507.1 |Severe acute respiratory syndrome coro ···················· ···················· ···················· ···················· ···················· ····················
9404 MW172508.1 |Severe acute respiratory syndrome coro ···················· ···················· ···················· ···················· ···················· ····················
9405 MW172509.1 |Severe acute respiratory syndrome coro ···················· ···················· ···················· ···················· ···················· ····················
9406 MW172510.1 |Severe acute respiratory syndrome coro ···················· ···················· ···················· ···················· ···················· ····················
9407 MW172513.1 |Severe acute respiratory syndrome coro ···················· ···················· ···················· ···················· ···················· ····················
9408 MW172514.1 |Severe acute respiratory syndrome coro ···················· ···················· ···················· ···················· ···················· ····················
9409 MW172515.1 |Severe acute respiratory syndrome coro ···················· ···················· ···················· ···················· ···················· ····················
9410 MW172517.1 |Severe acute respiratory syndrome coro ···················· ···················· ···················· ···················· ···················· ····················
9411 MW172518.1 |Severe acute respiratory syndrome coro ···················· ···················· ···················· ···················· ···················· ····················
9412 MW172520.1 |Severe acute respiratory syndrome coro ···················· ···················· ···················· ···················· ···················· ····················
9413 MW172558.1 |Severe acute respiratory syndrome coro ···················· ···················· ···················· ···················· ···················· ····················
9414 MW172563.1 |Severe acute respiratory syndrome coro ···················· ···················· ···················· ···················· ···················· ····················
9415 MW172570.1 |Severe acute respiratory syndrome coro ···················· ···················· ···················· ···················· ···················· ····················
9416 MW172571.1 |Severe acute respiratory syndrome coro ···················· ···················· ···················· ···················· ···················· ····················
9417 MW172572.1 |Severe acute respiratory syndrome coro ···················· ···················· ···················· ···················· ···················· ····················
9418 MW172574.1 |Severe acute respiratory syndrome coro ···················· ···················· ···················· ···················· ···················· ····················
9419 MW172576.1 |Severe acute respiratory syndrome coro ···················· ···················· ···················· ···················· ···················· ····················
9420 MW172579.1 |Severe acute respiratory syndrome coro ···················· ···················· ···················· ···················· ···················· ····················
9421 MW172581.1 |Severe acute respiratory syndrome coro ···················· ···················· ···················· ···················· ···················· ····················
9422 MW172721.1 |Severe acute respiratory syndrome coro ···················· ···················· ···················· ···················· ···················· ····················
9423 MW172727.1 |Severe acute respiratory syndrome coro ···················· ···················· ···················· ···················· ···················· ····················
9424 MW172730.1 |Severe acute respiratory syndrome coro ···················· ···················· ···················· ···················· ···················· ····················
9425 MW172733.1 |Severe acute respiratory syndrome coro ···················· ···················· ···················· ···················· ···················· ····················
9426 MW172737.1 |Severe acute respiratory syndrome coro ···················· ···················· ···················· ···················· ···················· ····················
9427 MW172742.1 |Severe acute respiratory syndrome coro ···················· ···················· ···················· ···················· ···················· ····················
9428 MW172756.1 |Severe acute respiratory syndrome coro ···················· ···················· ···················· ···················· ···················· ····················
9429 MW172762.1 |Severe acute respiratory syndrome coro ···················· ···················· ···················· ···················· ···················· ····················
9430 MW172766.1 |Severe acute respiratory syndrome coro ···················· ···················· ···················· ···················· ···················· ····················
9431 MW172768.1 |Severe acute respiratory syndrome coro ···················· ···················· ···················· ···················· ···················· ····················
9432 MW172771.1 |Severe acute respiratory syndrome coro ···················· ···················· ···················· ···················· ···················· ····················
9433 MW172813.1 |Severe acute respiratory syndrome coro ···················· ···················· ···················· ···················· ···················· ····················
9434 MW173146.1 |Severe acute respiratory syndrome coro ···················· ···················· ···················· ···················· ···················· ····················
9435 MW173147.1 |Severe acute respiratory syndrome coro ···················· ···················· ···················· ···················· ···················· ····················
9436 MW173148.1 |Severe acute respiratory syndrome coro ···················· ·A·················· ···················· ···················· ···················· ····················
9437 MW173149.1 |Severe acute respiratory syndrome coro ···················· ···················· ···················· ···················· ···················· ····················
9438 MW173150.1 |Severe acute respiratory syndrome coro ···················· ···················· ···················· ···················· ···················· ····················
9439 MW173151.1 |Severe acute respiratory syndrome coro ···················· ···················· ···················· ···················· ···················· ····················
9440 MW173152.1 |Severe acute respiratory syndrome coro ···················· ···················· ···················· ···················· ···················· ····················
9441 MW173153.1 |Severe acute respiratory syndrome coro ···················· ···················· ···················· ···················· ···················· ····················
9442 MW173154.1 |Severe acute respiratory syndrome coro ···················· ···················· ···················· ···················· ···················· ····················
9443 MW173155.1 |Severe acute respiratory syndrome coro ···················· ···················· ···················· ···················· ···················· ····················
9444 MW173156.1 |Severe acute respiratory syndrome coro ···················· ···················· ···················· ···················· ···················· ····················

9445	MW173157.1 |Severe acute respiratory syndrome coro	....	....	....	....	....	....
9446	MW173158.1 |Severe acute respiratory syndrome coro	....	....	....	....	....	....
9447	MW173159.1 |Severe acute respiratory syndrome coro	....	....	....	....	....	....
9448	MW173160.1 |Severe acute respiratory syndrome coro	....	....	....	....	....	....
9449	MW173161.1 |Severe acute respiratory syndrome coro	....	....	....	....	....	....
9450	MW173162.1 |Severe acute respiratory syndrome coro	....	....	....	....	....	....
9451	MW173163.1 |Severe acute respiratory syndrome coro	....	....	....	....	....	....
9452	MW173164.1 |Severe acute respiratory syndrome coro	....	.A..................	....	....	....	....
9453	MW173165.1 |Severe acute respiratory syndrome coro	....	....	....	....	....	....
9454	MW173166.1 |Severe acute respiratory syndrome coro	....	....	....	....	....	....
9455	MW173167.1 |Severe acute respiratory syndrome coro	....	....	....	....	....	....
9456	MW173168.1 |Severe acute respiratory syndrome coro	....	....	....	....	....	....
9457	MW173169.1 |Severe acute respiratory syndrome coro	....	....	....	....	....	....
9458	MW173170.1 |Severe acute respiratory syndrome coro	....	....	....	....	....	....
9459	MW173171.1 |Severe acute respiratory syndrome coro	....	....	....	....	....	....
9460	MW173212.1 |Severe acute respiratory syndrome coro	....	....	....	....	....	....
9461	MW173213.1 |Severe acute respiratory syndrome coro	....	....	....	....	....	....
9462	MW161259.1 |Severe acute respiratory syndrome coro	....	....	....	....	....	....
9463	MW153038.1 |Severe acute respiratory syndrome coro	....	....	....	....	....	....
9464	MW153039.1 |Severe acute respiratory syndrome coro	....	....	....	....	....	....
9467	MW153047.1 |Severe acute respiratory syndrome coro	....	....	....	....	....	....
9469	MW153051.1 |Severe acute respiratory syndrome coro	....	....	....	....	....	....
9473	MW153063.1 |Severe acute respiratory syndrome coro	....	....	....	....	....	....
9474	MW153072.1 |Severe acute respiratory syndrome coro	....	....	....	....	....	....
9475	MW153074.1 |Severe acute respiratory syndrome coro	....	....	....	....	....	....
9476	MW153075.1 |Severe acute respiratory syndrome coro	....	....	....	....	....	....
9478	MW153078.1 |Severe acute respiratory syndrome coro	....	....	....	....	....	....
9479	MW153079.1 |Severe acute respiratory syndrome coro	....	....	....	....	....	....
9481	MW153081.1 |Severe acute respiratory syndrome coro	....	....	....	....	....	....
9482	MW153082.1 |Severe acute respiratory syndrome coro	....	....	....	....	....	....
9483	MW153083.1 |Severe acute respiratory syndrome coro	....	....	....	....	....	....
9484	MW153084.1 |Severe acute respiratory syndrome coro	....	....	....	....	....	....
9485	MW153085.1 |Severe acute respiratory syndrome coro	....	....	....	....	....	....
9486	MW153086.1 |Severe acute respiratory syndrome coro	....	....	....	....	....	....
9487	MW153087.1 |Severe acute respiratory syndrome coro	....	....	....	....	....	....
9488	MW153088.1 |Severe acute respiratory syndrome coro	....	....	....	....	....	....
9489	MW153089.1 |Severe acute respiratory syndrome coro	....	....	....	....	....	....
9490	MW153090.1 |Severe acute respiratory syndrome coro	....	....	....	....	....	....
9491	MW153091.1 |Severe acute respiratory syndrome coro	....	....	....	....	....	....
9492	MW153094.1 |Severe acute respiratory syndrome coro	....	....	....	....	....	....
9493	MW153095.1 |Severe acute respiratory syndrome coro	....	....	....	....	....	....
9494	MW153096.1 |Severe acute respiratory syndrome coro	....	....	....	....	....	....
9495	MW153097.1 |Severe acute respiratory syndrome coro	....	....	....	....	....	....
9496	MW153098.1 |Severe acute respiratory syndrome coro	....	....	....	....	....	....
9497	MW153099.1 |Severe acute respiratory syndrome coro	....	....	....	....	....	....
9498	MW153100.1 |Severe acute respiratory syndrome coro	....	....	....	....	....	....
9499	MW153101.1 |Severe acute respiratory syndrome coro	....	....	....	....	....	....
9500	MW153102.1 |Severe acute respiratory syndrome coro	....	....	....	....	....	....
9501	MW153103.1 |Severe acute respiratory syndrome coro	....	....	....	....	....	....
9502	MW153104.1 |Severe acute respiratory syndrome coro	....	....	....	....	....	....
9503	MW153105.1 |Severe acute respiratory syndrome coro	....	....	....	....	....	....
9504	MW153106.1 |Severe acute respiratory syndrome coro	....	....	....	....	....	....
9505	MW153107.1 |Severe acute respiratory syndrome coro	....	....	....	....	....	....
9506	MW153108.1 |Severe acute respiratory syndrome coro	....	....	....	....	....	....
9507	MW153109.1 |Severe acute respiratory syndrome coro	....	....	....	....	....	....
9508	MW153110.1 |Severe acute respiratory syndrome coro	....	....	....	....	....	....
9509	MW153111.1 |Severe acute respiratory syndrome coro	....	....	....	....	....	....
9510	MW153112.1 |Severe acute respiratory syndrome coro	....	....	....	....	....	....
9511	MW153113.1 |Severe acute respiratory syndrome coro	....	....	....	....	....	....
9512	MW153114.1 |Severe acute respiratory syndrome coro	....	....	....	....	....	....
9513	MW153115.1 |Severe acute respiratory syndrome coro	....	....	....	....	....	....
9514	MW153116.1 |Severe acute respiratory syndrome coro	....	....	....	....	....	....
9515	MW153117.1 |Severe acute respiratory syndrome coro	....	....	....	....	....	....
9516	MW153118.1 |Severe acute respiratory syndrome coro	....	....	....	....	....	....
9517	MW153119.1 |Severe acute respiratory syndrome coro	....	....	....	....	....	....
9518	MW153120.1 |Severe acute respiratory syndrome coro	....	....	....	....	....	....
9519	MW153121.1 |Severe acute respiratory syndrome coro	....	....	....	....	....	....
9520	MW153122.1 |Severe acute respiratory syndrome coro	....	....	....	....	....	....
9521	MW153123.1 |Severe acute respiratory syndrome coro	....	....	....	....	....	....
9522	MW153126.1 |Severe acute respiratory syndrome coro	....	....	....	....	....	....
9523	MW153127.1 |Severe acute respiratory syndrome coro	....	....	....	....	....	....
9524	MW153128.1 |Severe acute respiratory syndrome coro	....	....	....	....	....	....
9525	MW153129.1 |Severe acute respiratory syndrome coro	....	....	....	....	....	....
9526	MW153130.1 |Severe acute respiratory syndrome coro	....	....	....	....	....	....
9527	MW153131.1 |Severe acute respiratory syndrome coro	....	....	....	....	....	....
9528	MW153132.1 |Severe acute respiratory syndrome coro	....	....	....	....	....	....
9529	MW153133.1 |Severe acute respiratory syndrome coro	....	....	....	....	....	....
9530	MW153134.1 |Severe acute respiratory syndrome coro	....	....	....	....	....	....

9531 MW153135.1 |Severe acute respiratory syndrome coro ···················· ···················· ····················· ···················· ···················· ····················
9532 MW153136.1 |Severe acute respiratory syndrome coro ···················· ···················· ····················· ···················· ···················· ····················
9533 MW153137.1 |Severe acute respiratory syndrome coro ···················· ···················· ····················· ···················· ···················· ····················
9534 MW153138.1 |Severe acute respiratory syndrome coro ···················· ······A············· ····················· ···················· ···················· ····················
9535 MW153139.1 |Severe acute respiratory syndrome coro ···················· ···················· ····················· ···················· ···················· ····················
9536 MW153140.1 |Severe acute respiratory syndrome coro ···················· ···················· ····················· ···················· ···················· ····················
9537 MW153142.1 |Severe acute respiratory syndrome coro ···················· ···················· ····················· ···················· ···················· ····················
9538 MW153143.1 |Severe acute respiratory syndrome coro ···················· ···················· ····················· ···················· ···················· ····················
9539 MW153144.1 |Severe acute respiratory syndrome coro ···················· ···················· ····················· ···················· ···················· ····················
9540 MW153145.1 |Severe acute respiratory syndrome coro ···················· ···················· ····················· ···················· ···················· ····················
9541 MW153146.1 |Severe acute respiratory syndrome coro ···················· ···················· ····················· ···················· ···················· ····················
9542 MW153148.1 |Severe acute respiratory syndrome coro ···················· ···················· ····················· ···················· ···················· ····················
9543 MW153149.1 |Severe acute respiratory syndrome coro ···················· ···················· ····················· ···················· ···················· ····················
9544 MW153151.1 |Severe acute respiratory syndrome coro ···················· ···················· ····················· ···················· ···················· ····················
9545 MW153152.1 |Severe acute respiratory syndrome coro ···················· ···················· ····················· ···················· ···················· ····················
9546 MW153153.1 |Severe acute respiratory syndrome coro ···················· ···················· ····················· ···················· ···················· ····················
9547 MW153154.1 |Severe acute respiratory syndrome coro ···················· ···················· ····················· ···················· ···················· ····················
9548 MW153155.1 |Severe acute respiratory syndrome coro ···················· ···················· ····················· ···················· ···················· ····················
9549 MW153156.1 |Severe acute respiratory syndrome coro ···················· ···················· ····················· ···················· ···················· ····················
9550 MW153157.1 |Severe acute respiratory syndrome coro ···················· ···················· ····················· ···················· ···················· ····················
9551 MW153158.1 |Severe acute respiratory syndrome coro ···················· ···················· ····················· ···················· ···················· ····················
9552 MW153159.1 |Severe acute respiratory syndrome coro ···················· ···················· ····················· ···················· ···················· ····················
9553 MW153160.1 |Severe acute respiratory syndrome coro ···················· ···················· ····················· ···················· ···················· ····················
9554 MW153162.1 |Severe acute respiratory syndrome coro ···················· ···················· ····················· ···················· ···················· ····················
9555 MW153163.1 |Severe acute respiratory syndrome coro ···················· ···················· ····················· ···················· ···················· ····················
9556 MW153164.1 |Severe acute respiratory syndrome coro ···················· ···················· ····················· ···················· ···················· ····················
9557 MW153165.1 |Severe acute respiratory syndrome coro ···················· ···················· ····················· ···················· ···················· ····················
9558 MW153166.1 |Severe acute respiratory syndrome coro ···················· ···················· ····················· ···················· ···················· ····················
9559 MW153167.1 |Severe acute respiratory syndrome coro ···················· ···················· ····················· ···················· ···················· ····················
9560 MW153168.1 |Severe acute respiratory syndrome coro ···················· ···················· ····················· ···················· ···················· ····················
9561 MW153169.1 |Severe acute respiratory syndrome coro ···················· ···················· ····················· ···················· ···················· ····················
9562 MW153170.1 |Severe acute respiratory syndrome coro ···················· ···················· ····················· ···················· ···················· ····················
9563 MW153172.1 |Severe acute respiratory syndrome coro ···················· ···················· ····················· ···················· ···················· ····················
9564 MW153173.1 |Severe acute respiratory syndrome coro ···················· ···················· ····················· ···················· ···················· ····················
9565 MW153175.1 |Severe acute respiratory syndrome coro ···················· ···················· ····················· ···················· ···················· ····················
9566 MW153176.1 |Severe acute respiratory syndrome coro ···················· ···················· ····················· ···················· ···················· ····················
9567 MW153177.1 |Severe acute respiratory syndrome coro ···················· ···················· ····················· ···················· ···················· ····················
9568 MW153178.1 |Severe acute respiratory syndrome coro ···················· ···················· ····················· ···················· ···················· ····················
9569 MW153181.1 |Severe acute respiratory syndrome coro ···················· ···················· ····················· ···················· ···················· ····················
9570 MW153182.1 |Severe acute respiratory syndrome coro ···················· ···················· ····················· ···················· ···················· ····················
9571 MW153183.1 |Severe acute respiratory syndrome coro ···················· ···················· ····················· ···················· ···················· ····················
9572 MW153184.1 |Severe acute respiratory syndrome coro ···················· ···················· ····················· ···················· ···················· ····················
9573 MW153185.1 |Severe acute respiratory syndrome coro ···················· ···················· ····················· ···················· ···················· ····················
9574 MW153187.1 |Severe acute respiratory syndrome coro ···················· ···················· ····················· ···················· ···················· ····················
9575 MW153188.1 |Severe acute respiratory syndrome coro ···················· ···················· ····················· ···················· ···················· ····················
9576 MW153189.1 |Severe acute respiratory syndrome coro ···················· ···················· ····················· ···················· ···················· ····················
9577 MW153190.1 |Severe acute respiratory syndrome coro ···················· ···················· ····················· ···················· ···················· ····················
9578 MW153191.1 |Severe acute respiratory syndrome coro ···················· ···················· ····················· ···················· ···················· ····················
9579 MW153192.1 |Severe acute respiratory syndrome coro ···················· ···················· ····················· ···················· ···················· ····················
9580 MW153193.1 |Severe acute respiratory syndrome coro ···················· ···················· ····················· ···················· ···················· ····················
9581 MW153194.1 |Severe acute respiratory syndrome coro ···················· ···················· ····················· ···················· ···················· ····················
9582 MW153195.1 |Severe acute respiratory syndrome coro ···················· ···················· ····················· ···················· ···················· ····················
9583 MW153196.1 |Severe acute respiratory syndrome coro ···················· ···················· ····················· ···················· ···················· ····················
9584 MW153197.1 |Severe acute respiratory syndrome coro ···················· ···················· ····················· ···················· ···················· ····················
9585 MW153198.1 |Severe acute respiratory syndrome coro ···················· ···················· ····················· ···················· ···················· ····················
9586 MW153199.1 |Severe acute respiratory syndrome coro ···················· ···················· ····················· ···················· ···················· ····················
9587 MW153200.1 |Severe acute respiratory syndrome coro ···················· ···················· ····················· ···················· ···················· ····················
9588 MW153202.1 |Severe acute respiratory syndrome coro ···················· ···················· ····················· ···················· ···················· ····················
9589 MW153203.1 |Severe acute respiratory syndrome coro ···················· ···················· ····················· ···················· ···················· ····················
9590 MW153204.1 |Severe acute respiratory syndrome coro ···················· ···················· ····················· ···················· ···················· ····················
9591 MW153205.1 |Severe acute respiratory syndrome coro ···················· ···················· ····················· ···················· ···················· ····················
9592 MW153206.1 |Severe acute respiratory syndrome coro ···················· ···················· ····················· ···················· ···················· ····················
9593 MW153208.1 |Severe acute respiratory syndrome coro ···················· ···················· ····················· ···················· ···················· ····················
9594 MW153209.1 |Severe acute respiratory syndrome coro ···················· ···················· ····················· ···················· ···················· ····················
9595 MW153210.1 |Severe acute respiratory syndrome coro ···················· ···················· ····················· ···················· ···················· ····················
9596 MW153211.1 |Severe acute respiratory syndrome coro ···················· ···················· ····················· ···················· ···················· ····················
9597 MW153212.1 |Severe acute respiratory syndrome coro ···················· ···················· ····················· ···················· ···················· ····················
9598 MW153213.1 |Severe acute respiratory syndrome coro ···················· ···················· ····················· ···················· ···················· ····················
9599 MW153214.1 |Severe acute respiratory syndrome coro ···················· ···················· ····················· ···················· ···················· ····················
9600 MW153215.1 |Severe acute respiratory syndrome coro ···················· ···················· ····················· ···················· ···················· ····················
9601 MW153216.1 |Severe acute respiratory syndrome coro ···················· ······A············· ····················· ···················· ···················· ····················
9602 MW153217.1 |Severe acute respiratory syndrome coro ···················· ···················· ····················· ···················· ···················· ····················
9603 MW153219.1 |Severe acute respiratory syndrome coro ···················· ···················· ····················· ···················· ···················· ····················
9604 MW153220.1 |Severe acute respiratory syndrome coro ···················· ···················· ····················· ···················· ···················· ····················
9605 MW153223.1 |Severe acute respiratory syndrome coro ···················· ···················· ····················· ···················· ···················· ····················
9606 MW153226.1 |Severe acute respiratory syndrome coro ···················· ···················· ····················· ···················· ···················· ····················
9607 MW153227.1 |Severe acute respiratory syndrome coro ···················· ···················· ····················· ···················· ···················· ····················
9608 MW153228.1 |Severe acute respiratory syndrome coro ···················· ···················· ····················· ···················· ···················· ····················

9609 MW153229.1 |Severe acute respiratory syndrome coro ....
9610 MW153230.1 |Severe acute respiratory syndrome coro ....
9611 MW153231.1 |Severe acute respiratory syndrome coro ....
9612 MW153232.1 |Severe acute respiratory syndrome coro ....
9613 MW153233.1 |Severe acute respiratory syndrome coro ....
9614 MW153234.1 |Severe acute respiratory syndrome coro ....
9615 MW153235.1 |Severe acute respiratory syndrome coro ....
9616 MW153236.1 |Severe acute respiratory syndrome coro ....
9617 MW153237.1 |Severe acute respiratory syndrome coro ....
9618 MW153238.1 |Severe acute respiratory syndrome coro ....
9619 MW153239.1 |Severe acute respiratory syndrome coro ....
9620 MW153240.1 |Severe acute respiratory syndrome coro ....
9621 MW153241.1 |Severe acute respiratory syndrome coro ....
9622 MW153242.1 |Severe acute respiratory syndrome coro ....
9623 MW153243.1 |Severe acute respiratory syndrome coro ....
9624 MW153244.1 |Severe acute respiratory syndrome coro ....
9625 MW153245.1 |Severe acute respiratory syndrome coro ....
9626 MW153246.1 |Severe acute respiratory syndrome coro ....
9627 MW153247.1 |Severe acute respiratory syndrome coro ....
9628 MW153249.1 |Severe acute respiratory syndrome coro ....
9629 MW153250.1 |Severe acute respiratory syndrome coro ....
9630 MW153251.1 |Severe acute respiratory syndrome coro ....
9631 MW153252.1 |Severe acute respiratory syndrome coro ....
9632 MW153253.1 |Severe acute respiratory syndrome coro ....
9633 MW153255.1 |Severe acute respiratory syndrome coro ....
9634 MW153258.1 |Severe acute respiratory syndrome coro ....
9635 MW153259.1 |Severe acute respiratory syndrome coro ....
9636 MW153260.1 |Severe acute respiratory syndrome coro ....
9637 MW153261.1 |Severe acute respiratory syndrome coro ....
9638 MW153262.1 |Severe acute respiratory syndrome coro ....
9639 MW153263.1 |Severe acute respiratory syndrome coro ....
9640 MW153264.1 |Severe acute respiratory syndrome coro ....
9641 MW153266.1 |Severe acute respiratory syndrome coro ....
9642 MW153267.1 |Severe acute respiratory syndrome coro ....
9643 MW153268.1 |Severe acute respiratory syndrome coro ....
9644 MW153269.1 |Severe acute respiratory syndrome coro ....
9645 MW153270.1 |Severe acute respiratory syndrome coro ....
9646 MW153271.1 |Severe acute respiratory syndrome coro ....
9647 MW153272.1 |Severe acute respiratory syndrome coro ....
9648 MW153273.1 |Severe acute respiratory syndrome coro ....
9649 MW153274.1 |Severe acute respiratory syndrome coro ....
9650 MW153275.1 |Severe acute respiratory syndrome coro ....
9651 MW153276.1 |Severe acute respiratory syndrome coro ....
9652 MW153277.1 |Severe acute respiratory syndrome coro ....
9653 MW153278.1 |Severe acute respiratory syndrome coro ....
9654 MW153279.1 |Severe acute respiratory syndrome coro ....
9655 MW153280.1 |Severe acute respiratory syndrome coro ....
9656 MW153281.1 |Severe acute respiratory syndrome coro ....
9657 MW153282.1 |Severe acute respiratory syndrome coro ....
9658 MW153283.1 |Severe acute respiratory syndrome coro ....
9659 MW153285.1 |Severe acute respiratory syndrome coro ....
9660 MW153286.1 |Severe acute respiratory syndrome coro ....
9661 MW153292.1 |Severe acute respiratory syndrome coro ....
9662 MW153293.1 |Severe acute respiratory syndrome coro ....
9663 MW153294.1 |Severe acute respiratory syndrome coro ....
9664 MW153295.1 |Severe acute respiratory syndrome coro ....
9665 MW153296.1 |Severe acute respiratory syndrome coro ....
9666 MW153299.1 |Severe acute respiratory syndrome coro ....
9667 MW153300.1 |Severe acute respiratory syndrome coro ....
9668 MW153301.1 |Severe acute respiratory syndrome coro ....
9669 MW153302.1 |Severe acute respiratory syndrome coro ....
9670 MW153304.1 |Severe acute respiratory syndrome coro ....
9671 MW153305.1 |Severe acute respiratory syndrome coro ....
9672 MW153306.1 |Severe acute respiratory syndrome coro ....
9673 MW153311.1 |Severe acute respiratory syndrome coro ....
9674 MW153316.1 |Severe acute respiratory syndrome coro ....
9675 MW153318.1 |Severe acute respiratory syndrome coro ....
9676 MW153320.1 |Severe acute respiratory syndrome coro ....
9677 MW153322.1 |Severe acute respiratory syndrome coro ....
9678 MW153323.1 |Severe acute respiratory syndrome coro ....
9679 MW153324.1 |Severe acute respiratory syndrome coro ....
9680 MW153325.1 |Severe acute respiratory syndrome coro ....
9681 MW153326.1 |Severe acute respiratory syndrome coro ....
9682 MW153327.1 |Severe acute respiratory syndrome coro ....
9683 MW153328.1 |Severe acute respiratory syndrome coro ....
9684 MW153329.1 |Severe acute respiratory syndrome coro ....
9685 MW153331.1 |Severe acute respiratory syndrome coro ....
9686 MW153332.1 |Severe acute respiratory syndrome coro ....

9687 MW153333.1 |Severe acute respiratory syndrome coro ....
9688 MW153334.1 |Severe acute respiratory syndrome coro ....
9689 MW153337.1 |Severe acute respiratory syndrome coro ....
9690 MW153338.1 |Severe acute respiratory syndrome coro ....
9691 MW153339.1 |Severe acute respiratory syndrome coro ....
9692 MW153340.1 |Severe acute respiratory syndrome coro ....
9693 MW153341.1 |Severe acute respiratory syndrome coro ....
9694 MW153342.1 |Severe acute respiratory syndrome coro ....A.............. ....
9695 MW153343.1 |Severe acute respiratory syndrome coro ....
9696 MW153345.1 |Severe acute respiratory syndrome coro ....
9697 MW153346.1 |Severe acute respiratory syndrome coro ....
9698 MW153347.1 |Severe acute respiratory syndrome coro ....
9699 MW153350.1 |Severe acute respiratory syndrome coro ....
9700 MW153351.1 |Severe acute respiratory syndrome coro ....
9701 MW153352.1 |Severe acute respiratory syndrome coro ....
9702 MW153354.1 |Severe acute respiratory syndrome coro ....
9703 MW153356.1 |Severe acute respiratory syndrome coro ....
9704 MW153357.1 |Severe acute respiratory syndrome coro ....
9705 MW153358.1 |Severe acute respiratory syndrome coro ....
9706 MW153360.1 |Severe acute respiratory syndrome coro ....
9707 MW153361.1 |Severe acute respiratory syndrome coro ....
9708 MW153363.1 |Severe acute respiratory syndrome coro ....
9709 MW153364.1 |Severe acute respiratory syndrome coro ....
9710 MW153365.1 |Severe acute respiratory syndrome coro ....
9711 MW153368.1 |Severe acute respiratory syndrome coro ....
9712 MW153369.1 |Severe acute respiratory syndrome coro ....
9713 MW153371.1 |Severe acute respiratory syndrome coro ....
9714 MW153372.1 |Severe acute respiratory syndrome coro ....
9715 MW153373.1 |Severe acute respiratory syndrome coro ....
9716 MW153374.1 |Severe acute respiratory syndrome coro ....
9717 MW153375.1 |Severe acute respiratory syndrome coro ....
9718 MW153376.1 |Severe acute respiratory syndrome coro ....
9719 MW153377.1 |Severe acute respiratory syndrome coro ....
9720 MW153379.1 |Severe acute respiratory syndrome coro ....
9721 MW153380.1 |Severe acute respiratory syndrome coro ....
9722 MW153381.1 |Severe acute respiratory syndrome coro ....
9723 MW153382.1 |Severe acute respiratory syndrome coro ....
9724 MW153383.1 |Severe acute respiratory syndrome coro ....
9725 MW153384.1 |Severe acute respiratory syndrome coro ....
9726 MW153385.1 |Severe acute respiratory syndrome coro ....
9727 MW153387.1 |Severe acute respiratory syndrome coro ....
9728 MW153389.1 |Severe acute respiratory syndrome coro ....
9729 MW153390.1 |Severe acute respiratory syndrome coro ....
9730 MW153392.1 |Severe acute respiratory syndrome coro ....
9731 MW153394.1 |Severe acute respiratory syndrome coro ....
9732 MW153395.1 |Severe acute respiratory syndrome coro ....
9733 MW153396.1 |Severe acute respiratory syndrome coro ....
9734 MW153397.1 |Severe acute respiratory syndrome coro ....
9735 MW153398.1 |Severe acute respiratory syndrome coro ....
9736 MW153399.1 |Severe acute respiratory syndrome coro ....
9737 MW153400.1 |Severe acute respiratory syndrome coro ....
9738 MW153401.1 |Severe acute respiratory syndrome coro ....
9739 MW153402.1 |Severe acute respiratory syndrome coro ....
9740 MW153403.1 |Severe acute respiratory syndrome coro ....
9741 MW153404.1 |Severe acute respiratory syndrome coro ....
9742 MW153405.1 |Severe acute respiratory syndrome coro ....
9743 MW153406.1 |Severe acute respiratory syndrome coro ....
9744 MW153407.1 |Severe acute respiratory syndrome coro ....
9745 MW153408.1 |Severe acute respiratory syndrome coro ....
9746 MW153409.1 |Severe acute respiratory syndrome coro ....
9747 MW153411.1 |Severe acute respiratory syndrome coro ....
9748 MW153412.1 |Severe acute respiratory syndrome coro ....
9749 MW153413.1 |Severe acute respiratory syndrome coro ....
9750 MW153415.1 |Severe acute respiratory syndrome coro ....
9751 MW153416.1 |Severe acute respiratory syndrome coro ....
9752 MW153417.1 |Severe acute respiratory syndrome coro ....
9753 MW153418.1 |Severe acute respiratory syndrome coro ....
9754 MW153420.1 |Severe acute respiratory syndrome coro ....
9755 MW153421.1 |Severe acute respiratory syndrome coro ....
9756 MW153422.1 |Severe acute respiratory syndrome coro ....
9757 MW153423.1 |Severe acute respiratory syndrome coro ....
9758 MW153424.1 |Severe acute respiratory syndrome coro ....
9759 MW153425.1 |Severe acute respiratory syndrome coro ....
9760 MW153426.1 |Severe acute respiratory syndrome coro ....
9761 MW153427.1 |Severe acute respiratory syndrome coro ....
9762 MW153428.1 |Severe acute respiratory syndrome coro ....
9763 MW153429.1 |Severe acute respiratory syndrome coro ....
9764 MW153430.1 |Severe acute respiratory syndrome coro ....

```
9765 MW153431.1 |Severe acute respiratory syndrome coro    .................... .................... .................... .................... .................... ....................
9766 MW153432.1 |Severe acute respiratory syndrome coro    .................... .................... .................... .................... .................... ....................
9767 MW153433.1 |Severe acute respiratory syndrome coro    .................... .................... .................... .................... .................... ....................
9768 MW153434.1 |Severe acute respiratory syndrome coro    .................... .................... .................... .................... .................... ....................
9769 MW153435.1 |Severe acute respiratory syndrome coro    .................... .................... .................... .................... .................... ....................
9770 MW153436.1 |Severe acute respiratory syndrome coro    .................... .................... .................... .................... .................... ....................
9771 MW153437.1 |Severe acute respiratory syndrome coro    .................... .................... .................... .................... .................... ....................
9772 MW153438.1 |Severe acute respiratory syndrome coro    .................... .................... .................... .................... .................... ....................
9773 MW153439.1 |Severe acute respiratory syndrome coro    .................... .................... .................... .................... .................... ....................
9774 MW153440.1 |Severe acute respiratory syndrome coro    .................... .................... .................... .................... .................... ....................
9775 MW153441.1 |Severe acute respiratory syndrome coro    .................... .................... .................... .................... .................... ....................
9776 MW153444.1 |Severe acute respiratory syndrome coro    .................... .................... .................... .................... .................... ....................
9777 MW153445.1 |Severe acute respiratory syndrome coro    .................... .................... .................... .................... .................... ....................
9778 MW153446.1 |Severe acute respiratory syndrome coro    .................... .................... .................... .................... .................... ....................
9779 MW153447.1 |Severe acute respiratory syndrome coro    .................... .................... .................... .................... .................... ....................
9780 MW153448.1 |Severe acute respiratory syndrome coro    .................... .................... .................... .................... .................... ....................
9781 MW153449.1 |Severe acute respiratory syndrome coro    .................... .................... .................... .................... .................... ....................
9782 MW153451.1 |Severe acute respiratory syndrome coro    .................... .................... .................... .................... .................... ....................
9783 MW153452.1 |Severe acute respiratory syndrome coro    .................... .................... .................... .................... .................... ....................
9784 MW153453.1 |Severe acute respiratory syndrome coro    .................... .................... .................... .................... .................... ....................
9785 MW153454.1 |Severe acute respiratory syndrome coro    .................... .................... .................... .................... .................... ....................
9786 MW153455.1 |Severe acute respiratory syndrome coro    .................... .................... .................... .................... .................... ....................
9787 MW153456.1 |Severe acute respiratory syndrome coro    .................... .................... .................... .................... .................... ....................
9788 MW153457.1 |Severe acute respiratory syndrome coro    .................... .................... .................... .................... .................... ....................
9789 MW153458.1 |Severe acute respiratory syndrome coro    .................... .................... .................... .................... .................... ....................
9790 MW153459.1 |Severe acute respiratory syndrome coro    .................... .................... .................... .................... .................... ....................
9791 MW153460.1 |Severe acute respiratory syndrome coro    .................... .................... .................... .................... .................... ....................
9792 MW153461.1 |Severe acute respiratory syndrome coro    .................... .................... .................... .................... .................... ....................
9793 MW153462.1 |Severe acute respiratory syndrome coro    .................... .................... .................... .................... .................... ....................
9794 MW153463.1 |Severe acute respiratory syndrome coro    .................... .................... .................... .................... .................... ....................
9795 MW153464.1 |Severe acute respiratory syndrome coro    .................... .................... .................... .................... .................... ....................
9796 MW153465.1 |Severe acute respiratory syndrome coro    .................... .................... .................... .................... .................... ....................
9797 MW153466.1 |Severe acute respiratory syndrome coro    .................... .................... .................... .................... .................... ....................
9798 MW153467.1 |Severe acute respiratory syndrome coro    .................... .................... .................... .................... .................... ....................
9799 MW153468.1 |Severe acute respiratory syndrome coro    .................... .................... .................... .................... .................... ....................
9800 MW153469.1 |Severe acute respiratory syndrome coro    .................... .................... .................... .................... .................... ....................
9801 MW153470.1 |Severe acute respiratory syndrome coro    .................... .................... .................... .................... .................... ....................
9802 MW153471.1 |Severe acute respiratory syndrome coro    .................... .................... .................... .................... .................... ....................
9803 MW153472.1 |Severe acute respiratory syndrome coro    .................... .................... .................... .................... .................... ....................
9804 MW153473.1 |Severe acute respiratory syndrome coro    .................... .................... .................... .................... .................... ....................
9805 MW153474.1 |Severe acute respiratory syndrome coro    .................... .................... .................... .................... .................... ....................
9806 MW153475.1 |Severe acute respiratory syndrome coro    .................... .................... .................... .................... .................... ....................
9807 MW153476.1 |Severe acute respiratory syndrome coro    .................... .................... .................... .................... .................... ....................
9808 MW153477.1 |Severe acute respiratory syndrome coro    .................... .................... .................... .................... .................... ....................
9809 MW153478.1 |Severe acute respiratory syndrome coro    .................... ......A............. .................... .................... .................... ....................
9810 MW153479.1 |Severe acute respiratory syndrome coro    .................... ......A............. .................... .................... .................... ....................
9811 MW153480.1 |Severe acute respiratory syndrome coro    .................... .................... .................... .................... .................... ....................
9812 MW153481.1 |Severe acute respiratory syndrome coro    .................... .................... .................... .................... .................... ....................
9813 MW153482.1 |Severe acute respiratory syndrome coro    .................... .................... .................... .................... .................... ....................
9814 MW153483.1 |Severe acute respiratory syndrome coro    .................... .................... .................... .................... .................... ....................
9815 MW153484.1 |Severe acute respiratory syndrome coro    .................... .................... .................... .................... .................... ....................
9816 MW153486.1 |Severe acute respiratory syndrome coro    .................... .................... .................... .................... .................... ....................
9817 MW153487.1 |Severe acute respiratory syndrome coro    .................... .................... .................... .................... .................... ....................
9818 MW153488.1 |Severe acute respiratory syndrome coro    .................... .................... .................... .................... .................... ....................
9819 MW153489.1 |Severe acute respiratory syndrome coro    .................... .................... .................... .................... .................... ....................
9820 MW153490.1 |Severe acute respiratory syndrome coro    .................... .................... .................... .................... .................... ....................
9821 MW153491.1 |Severe acute respiratory syndrome coro    .................... .................... .................... .................... .................... ....................
9822 MW153492.1 |Severe acute respiratory syndrome coro    .................... .................... .................... .................... .................... ....................
9823 MW153493.1 |Severe acute respiratory syndrome coro    .................... .................... .................... .................... .................... ....................
9824 MW153495.1 |Severe acute respiratory syndrome coro    .................... .................... .................... .................... .................... ....................
9825 MW153496.1 |Severe acute respiratory syndrome coro    .................... .................... .................... .................... .................... ....................
9826 MW153497.1 |Severe acute respiratory syndrome coro    .................... .................... .................... .................... .................... ....................
9827 MW153498.1 |Severe acute respiratory syndrome coro    .................... .................... .................... .................... .................... ....................
9828 MW153499.1 |Severe acute respiratory syndrome coro    .................... .................... .................... .................... .................... ....................
9829 MW153500.1 |Severe acute respiratory syndrome coro    .................... .................... .................... .................... .................... ....................
9830 MW153501.1 |Severe acute respiratory syndrome coro    .................... .................... .................... .................... .................... ....................
9831 MW153502.1 |Severe acute respiratory syndrome coro    .................... .................... .................... .................... .................... ....................
9832 MW153503.1 |Severe acute respiratory syndrome coro    .................... .................... .................... .................... .................... ....................
9833 MW153504.1 |Severe acute respiratory syndrome coro    .................... .................... .................... .................... .................... ....................
9834 MW153505.1 |Severe acute respiratory syndrome coro    .................... .................... .................... .................... .................... ....................
9835 MW153506.1 |Severe acute respiratory syndrome coro    .................... .................... .................... .................... .................... ....................
9836 MW153507.1 |Severe acute respiratory syndrome coro    .................... .................... .................... .................... .................... ....................
9837 MW153508.1 |Severe acute respiratory syndrome coro    .................... .................... .................... .................... .................... ....................
9838 MW153509.1 |Severe acute respiratory syndrome coro    .................... .................... .................... .................... .................... ....................
9839 MW153510.1 |Severe acute respiratory syndrome coro    .................... .................... .................... .................... .................... ....................
9840 MW153511.1 |Severe acute respiratory syndrome coro    .................... .................... .................... .................... .................... ....................
9841 MW153512.1 |Severe acute respiratory syndrome coro    .................... .................... .................... .................... .................... ....................
9842 MW153513.1 |Severe acute respiratory syndrome coro    .................... .................... .................... .................... .................... ....................
```

9843	MW153514.1 |Severe acute respiratory syndrome coro	....	....	....	....	....	....
9844	MW153515.1 |Severe acute respiratory syndrome coro	....	....	....	....	....	....
9845	MW153516.1 |Severe acute respiratory syndrome coro	....	....	....	....	....	....
9846	MW153517.1 |Severe acute respiratory syndrome coro	....	....	....	....	....	....
9847	MW153518.1 |Severe acute respiratory syndrome coro	....	....	....	....	....	....
9848	MW153520.1 |Severe acute respiratory syndrome coro	....	....	....	....	....	....
9849	MW153521.1 |Severe acute respiratory syndrome coro	....	....	....	....	....	....
9850	MW153522.1 |Severe acute respiratory syndrome coro	....	....	....	....	....	....
9851	MW153525.1 |Severe acute respiratory syndrome coro	....	....	....	....	....	....
9852	MW153526.1 |Severe acute respiratory syndrome coro	....	....	....	....	....	....
9853	MW153527.1 |Severe acute respiratory syndrome coro	....	....	....	....	....	....
9854	MW153528.1 |Severe acute respiratory syndrome coro	....	....	....	....	....	....
9855	MW153529.1 |Severe acute respiratory syndrome coro	....	....	....	....	....	....
9856	MW153530.1 |Severe acute respiratory syndrome coro	....	....	....	....	....	....
9857	MW153532.1 |Severe acute respiratory syndrome coro	....	....	....	....	....	....
9858	MW153533.1 |Severe acute respiratory syndrome coro	....	....	....	....	....	....
9859	MW153534.1 |Severe acute respiratory syndrome coro	....	....	....	....	....	....
9860	MW153536.1 |Severe acute respiratory syndrome coro	....	....	....	....	....	....
9861	MW153538.1 |Severe acute respiratory syndrome coro	....	....	....	....	....	....
9862	MW153540.1 |Severe acute respiratory syndrome coro	....	....	....	....	....	....
9863	MW153541.1 |Severe acute respiratory syndrome coro	....	....	....	....	....	....
9864	MW153543.1 |Severe acute respiratory syndrome coro	....	....	....	....	....	....
9865	MW153544.1 |Severe acute respiratory syndrome coro	....	....	....	....	....	....
9866	MW153545.1 |Severe acute respiratory syndrome coro	....	....	....	....	....	....
9867	MW153546.1 |Severe acute respiratory syndrome coro	....	....	....	....	....	....
9868	MW153547.1 |Severe acute respiratory syndrome coro	....	....	....	....	....	....
9869	MW153549.1 |Severe acute respiratory syndrome coro	....	....	....	....	....	....
9870	MW153550.1 |Severe acute respiratory syndrome coro	....	....	....	....	....	....
9871	MW153551.1 |Severe acute respiratory syndrome coro	....	....	....	....	....	....
9872	MW153552.1 |Severe acute respiratory syndrome coro	....	....	....	....	....	....
9873	MW153553.1 |Severe acute respiratory syndrome coro	....	....	....	....	....	....
9874	MW153554.1 |Severe acute respiratory syndrome coro	....	....	....	....	....	....
9875	MW153555.1 |Severe acute respiratory syndrome coro	....	....	....	....	....	....
9876	MW153556.1 |Severe acute respiratory syndrome coro	....	....	....	....	....	....
9877	MW153557.1 |Severe acute respiratory syndrome coro	....	....	....	....	....	....
9878	MW153558.1 |Severe acute respiratory syndrome coro	....	....	....	....	....	....
9879	MW153559.1 |Severe acute respiratory syndrome coro	....	....	....	....	....	....
9880	MW153560.1 |Severe acute respiratory syndrome coro	....	....	....	....	....	....
9881	MW153561.1 |Severe acute respiratory syndrome coro	....	....	....	....	....	....
9882	MW153562.1 |Severe acute respiratory syndrome coro	....	....	....	....	....	....
9883	MW153563.1 |Severe acute respiratory syndrome coro	....	....	....	....	....	....
9884	MW153564.1 |Severe acute respiratory syndrome coro	....	....	....	....	....	....
9885	MW153566.1 |Severe acute respiratory syndrome coro	....	....	....	....	....	....
9886	MW153567.1 |Severe acute respiratory syndrome coro	....	....	....	....	....	....
9887	MW153568.1 |Severe acute respiratory syndrome coro	....	....	....	....	....	....
9888	MW153569.1 |Severe acute respiratory syndrome coro	....	....	....	....	....	....
9889	MW153570.1 |Severe acute respiratory syndrome coro	....	....	....	....	....	....
9890	MW153571.1 |Severe acute respiratory syndrome coro	....	....	....	....	....	....
9891	MW153573.1 |Severe acute respiratory syndrome coro	....	....	....	....	....	....
9892	MW153574.1 |Severe acute respiratory syndrome coro	....	....	....	....	....	....
9893	MW153575.1 |Severe acute respiratory syndrome coro	....	....	....	....	....	....
9894	MW153576.1 |Severe acute respiratory syndrome coro	....	....	....	....	....	....
9895	MW153578.1 |Severe acute respiratory syndrome coro	....	....	....	....	....	....
9896	MW153579.1 |Severe acute respiratory syndrome coro	....	....	....	....	....	....
9897	MW153581.1 |Severe acute respiratory syndrome coro	....	....	....	....	....	....
9898	MW153582.1 |Severe acute respiratory syndrome coro	....	....	....	....	....	....
9899	MW153583.1 |Severe acute respiratory syndrome coro	....	....	....	....	....	....
9900	MW153584.1 |Severe acute respiratory syndrome coro	....	....	....	....	....	....
9901	MW153585.1 |Severe acute respiratory syndrome coro	....	....	....	....	....	....
9902	MW153586.1 |Severe acute respiratory syndrome coro	....	....	....	....	....	....
9903	MW153587.1 |Severe acute respiratory syndrome coro	....	....	....	....	....	....
9904	MW153588.1 |Severe acute respiratory syndrome coro	....	....	....	....	....	....
9905	MW153590.1 |Severe acute respiratory syndrome coro	....	....	....	....	....	....
9906	MW153591.1 |Severe acute respiratory syndrome coro	....	....	....	....	....	....
9907	MW153592.1 |Severe acute respiratory syndrome coro	....	....	....	....	....	....
9908	MW153593.1 |Severe acute respiratory syndrome coro	....	....	....	....	....	....
9909	MW153594.1 |Severe acute respiratory syndrome coro	....	....	....	....	....	....
9910	MW153595.1 |Severe acute respiratory syndrome coro	....	....	....	....	....	....
9911	MW153596.1 |Severe acute respiratory syndrome coro	....	....	....	....	....	....
9912	MW153597.1 |Severe acute respiratory syndrome coro	....	....	....	....	....	....
9913	MW153598.1 |Severe acute respiratory syndrome coro	....	....	....	....	....	....
9914	MW153600.1 |Severe acute respiratory syndrome coro	....	....	....	....	....	....
9915	MW153601.1 |Severe acute respiratory syndrome coro	....	....	....	....	....	....
9916	MW153602.1 |Severe acute respiratory syndrome coro	....	....	....	....	....	....
9917	MW153603.1 |Severe acute respiratory syndrome coro	....	....	....	....	....	....
9918	MW153605.1 |Severe acute respiratory syndrome coro	....	....	....	....	....	....
9919	MW153606.1 |Severe acute respiratory syndrome coro	....	....	....	....	....	....
9920	MW153607.1 |Severe acute respiratory syndrome coro	....	....	....	....	....	....

9921 MW153608.1 |Severe acute respiratory syndrome coro ···················· ···················· ····················· ···················· ···················· ····················
9922 MW153609.1 |Severe acute respiratory syndrome coro ···················· ···················· ····················· ···················· ···················· ····················
9923 MW153610.1 |Severe acute respiratory syndrome coro ···················· ···················· ····················· ···················· ···················· ····················
9924 MW153611.1 |Severe acute respiratory syndrome coro ···················· ···················· ····················· ···················· ···················· ····················
9925 MW153612.1 |Severe acute respiratory syndrome coro ···················· ···················· ····················· ···················· ···················· ····················
9926 MW153613.1 |Severe acute respiratory syndrome coro ···················· ···················· ····················· ···················· ···················· ····················
9927 MW153614.1 |Severe acute respiratory syndrome coro ···················· ···················· ····················· ···················· ···················· ····················
9928 MW153615.1 |Severe acute respiratory syndrome coro ···················· ···················· ····················· ···················· ···················· ····················
9929 MW153616.1 |Severe acute respiratory syndrome coro ···················· ···················· ····················· ···················· ···················· ····················
9930 MW153617.1 |Severe acute respiratory syndrome coro ···················· ···················· ····················· ···················· ···················· ····················
9931 MW153618.1 |Severe acute respiratory syndrome coro ···················· ···················· ····················· ···················· ···················· ····················
9932 MW153620.1 |Severe acute respiratory syndrome coro ···················· ···················· ····················· ···················· ···················· ····················
9933 MW153621.1 |Severe acute respiratory syndrome coro ···················· ···················· ····················· ···················· ···················· ····················
9934 MW153622.1 |Severe acute respiratory syndrome coro ···················· ···················· ····················· ···················· ···················· ····················
9935 MW153623.1 |Severe acute respiratory syndrome coro ···················· ···················· ····················· ···················· ···················· ····················
9936 MW153627.1 |Severe acute respiratory syndrome coro ···················· ···················· ····················· ···················· ···················· ····················
9937 MW153628.1 |Severe acute respiratory syndrome coro ···················· ···················· ····················· ···················· ···················· ····················
9938 MW153629.1 |Severe acute respiratory syndrome coro ···················· ···················· ····················· ···················· ···················· ····················
9939 MW153630.1 |Severe acute respiratory syndrome coro ···················· ···················· ····················· ···················· ···················· ····················
9940 MW153631.1 |Severe acute respiratory syndrome coro ···················· ···················· ····················· ···················· ···················· ····················
9941 MW153633.1 |Severe acute respiratory syndrome coro ···················· ···················· ····················· ···················· ···················· ····················
9942 MW153634.1 |Severe acute respiratory syndrome coro ···················· ···················· ····················· ···················· ···················· ····················
9943 MW153635.1 |Severe acute respiratory syndrome coro ···················· ···················· ····················· ···················· ···················· ····················
9944 MW153636.1 |Severe acute respiratory syndrome coro ···················· ···················· ····················· ···················· ···················· ····················
9945 MW153637.1 |Severe acute respiratory syndrome coro ···················· ···················· ····················· ···················· ···················· ····················
9946 MW153641.1 |Severe acute respiratory syndrome coro ···················· ···················· ····················· ···················· ···················· ····················
9947 MW153642.1 |Severe acute respiratory syndrome coro ···················· ···················· ····················· ···················· ···················· ····················
9948 MW153643.1 |Severe acute respiratory syndrome coro ···················· ···················· ····················· ···················· ···················· ····················
9949 MW153644.1 |Severe acute respiratory syndrome coro ···················· ···················· ····················· ···················· ···················· ····················
9950 MW153645.1 |Severe acute respiratory syndrome coro ···················· ···················· ····················· ···················· ···················· ····················
9951 MW153646.1 |Severe acute respiratory syndrome coro ···················· ···················· ····················· ···················· ···················· ····················
9952 MW153647.1 |Severe acute respiratory syndrome coro ···················· ···················· ····················· ···················· ···················· ····················
9953 MW153648.1 |Severe acute respiratory syndrome coro ···················· ···················· ····················· ···················· ···················· ····················
9954 MW153649.1 |Severe acute respiratory syndrome coro ···················· ···················· ····················· ···················· ···················· ····················
9955 MW153650.1 |Severe acute respiratory syndrome coro ···················· ···················· ····················· ···················· ···················· ····················
9956 MW153651.1 |Severe acute respiratory syndrome coro ···················· ···················· ····················· ···················· ···················· ····················
9957 MW153652.1 |Severe acute respiratory syndrome coro ···················· ···················· ····················· ···················· ···················· ····················
9958 MW153654.1 |Severe acute respiratory syndrome coro ···················· ···················· ····················· ···················· ···················· ····················
9959 MW153656.1 |Severe acute respiratory syndrome coro ···················· ···················· ····················· ···················· ···················· ····················
9960 MW153657.1 |Severe acute respiratory syndrome coro ···················· ···················· ····················· ···················· ···················· ····················
9961 MW153658.1 |Severe acute respiratory syndrome coro ···················· ···················· ····················· ···················· ···················· ····················
9962 MW153659.1 |Severe acute respiratory syndrome coro ···················· ···················· ····················· ···················· ···················· ····················
9963 MW153660.1 |Severe acute respiratory syndrome coro ···················· ···················· ····················· ···················· ···················· ····················
9964 MW153661.1 |Severe acute respiratory syndrome coro ···················· ···················· ····················· ···················· ···················· ····················
9965 MW153662.1 |Severe acute respiratory syndrome coro ···················· ···················· ····················· ···················· ···················· ····················
9966 MW153663.1 |Severe acute respiratory syndrome coro ···················· ···················· ····················· ···················· ···················· ····················
9967 MW153664.1 |Severe acute respiratory syndrome coro ···················· ···················· ····················· ···················· ···················· ····················
9968 MW153665.1 |Severe acute respiratory syndrome coro ···················· ···················· ····················· ···················· ···················· ····················
9969 MW153666.1 |Severe acute respiratory syndrome coro ···················· ···················· ····················· ···················· ···················· ····················
9970 MW153667.1 |Severe acute respiratory syndrome coro ···················· ···················· ····················· ···················· ···················· ····················
9971 MW153668.1 |Severe acute respiratory syndrome coro ···················· ···················· ····················· ···················· ···················· ····················
9972 MW153669.1 |Severe acute respiratory syndrome coro ···················· ···················· ····················· ···················· ···················· ····················
9973 MW153670.1 |Severe acute respiratory syndrome coro ···················· ···················· ····················· ···················· ···················· ····················
9974 MW153671.1 |Severe acute respiratory syndrome coro ···················· ···················· ····················· ···················· ···················· ····················
9975 MW153672.1 |Severe acute respiratory syndrome coro ···················· ···················· ····················· ···················· ···················· ····················
9976 MW153673.1 |Severe acute respiratory syndrome coro ···················· ···················· ····················· ···················· ···················· ····················
9977 MW153674.1 |Severe acute respiratory syndrome coro ···················· ···················· ····················· ···················· ···················· ····················
9978 MW153675.1 |Severe acute respiratory syndrome coro ···················· ···················· ····················· ···················· ···················· ····················
9979 MW153677.1 |Severe acute respiratory syndrome coro ···················· ···················· ····················· ···················· ···················· ····················
9980 MW153678.1 |Severe acute respiratory syndrome coro ···················· ···················· ····················· ···················· ···················· ····················
9981 MW153679.1 |Severe acute respiratory syndrome coro ···················· ···················· ····················· ···················· ···················· ····················
9982 MW153680.1 |Severe acute respiratory syndrome coro ···················· ···················· ····················· ···················· ···················· ····················
9983 MW153681.1 |Severe acute respiratory syndrome coro ···················· ···················· ····················· ···················· ···················· ····················
9984 MW153682.1 |Severe acute respiratory syndrome coro ···················· ···················· ····················· ···················· ···················· ····················
9985 MW153683.1 |Severe acute respiratory syndrome coro ···················· ···················· ····················· ···················· ···················· ····················
9986 MW153685.1 |Severe acute respiratory syndrome coro ···················· ···················· ····················· ···················· ···················· ····················
9987 MW153686.1 |Severe acute respiratory syndrome coro ···················· ···················· ····················· ···················· ···················· ····················
9988 MW153687.1 |Severe acute respiratory syndrome coro ···················· ···················· ····················· ···················· ···················· ····················
9989 MW153688.1 |Severe acute respiratory syndrome coro ···················· ···················· ····················· ···················· ···················· ····················
9990 MW153689.1 |Severe acute respiratory syndrome coro ···················· ···················· ····················· ···················· ···················· ····················
9991 MW153690.1 |Severe acute respiratory syndrome coro ········T··········· ···················· ····················· ···················· ···················· ····················
9992 MW153691.1 |Severe acute respiratory syndrome coro ···················· ···················· ····················· ···················· ···················· ····················
9993 MW153692.1 |Severe acute respiratory syndrome coro ···················· ···················· ····················· ···················· ···················· ····················
9994 MW153693.1 |Severe acute respiratory syndrome coro ···················· ···················· ····················· ···················· ···················· ····················
9995 MW153694.1 |Severe acute respiratory syndrome coro ···················· ···················· ····················· ···················· ···················· ····················
9996 MW153695.1 |Severe acute respiratory syndrome coro ···················· ···················· ····················· ···················· ···················· ····················
9997 MW153696.1 |Severe acute respiratory syndrome coro ···················· ···················· ····················· ···················· ···················· ····················
9998 MW153697.1 |Severe acute respiratory syndrome coro ···················· ···················· ····················· ···················· ···················· ····················

9999 MW153698.1 |Severe acute respiratory syndrome coro ···················· ···················· ···················· ···················· ···················· ····················
10000 MW153699.1 |Severe acute respiratory syndrome coro ···················· ···················· ···················· ···················· ···················· ····················
10001 MW153700.1 |Severe acute respiratory syndrome coro ···················· ···················· ···················· ···················· ···················· ····················
10002 MW153701.1 |Severe acute respiratory syndrome coro ···················· ···················· ···················· ···················· ···················· ····················
10003 MW153702.1 |Severe acute respiratory syndrome coro ···················· ···················· ···················· ···················· ···················· ····················
10004 MW153703.1 |Severe acute respiratory syndrome coro ···················· ···················· ···················· ···················· ···················· ····················
10005 MW153704.1 |Severe acute respiratory syndrome coro ···················· ···················· ···················· ···················· ···················· ····················
10006 MW153705.1 |Severe acute respiratory syndrome coro ···················· ···················· ···················· ···················· ···················· ····················
10007 MW153706.1 |Severe acute respiratory syndrome coro ···················· ···················· ···················· ···················· ···················· ····················
10008 MW153707.1 |Severe acute respiratory syndrome coro ···················· ···················· ···················· ···················· ···················· ····················
10009 MW153708.1 |Severe acute respiratory syndrome coro ···················· ···················· ···················· ···················· ···················· ····················
10010 MW153709.1 |Severe acute respiratory syndrome coro ···················· ···················· ···················· ···················· ···················· ····················
10011 MW153710.1 |Severe acute respiratory syndrome coro ···················· ···················· ···················· ···················· ···················· ····················
10012 MW153711.1 |Severe acute respiratory syndrome coro ···················· ···················· ···················· ···················· ···················· ····················
10013 MW153712.1 |Severe acute respiratory syndrome coro ···················· ···················· ···················· ···················· ···················· ····················
10014 MW153713.1 |Severe acute respiratory syndrome coro ···················· ······A············· ···················· ···················· ···················· ····················
10015 MW153714.1 |Severe acute respiratory syndrome coro ···················· ···················· ···················· ···················· ···················· ····················
10016 MW153716.1 |Severe acute respiratory syndrome coro ···················· ···················· ···················· ···················· ···················· ····················
10017 MW153718.1 |Severe acute respiratory syndrome coro ···················· ···················· ···················· ···················· ···················· ····················
10018 MW153719.1 |Severe acute respiratory syndrome coro ···················· ···················· ···················· ···················· ···················· ····················
10019 MW153721.1 |Severe acute respiratory syndrome coro ···················· ···················· ···················· ···················· ···················· ····················
10020 MW153722.1 |Severe acute respiratory syndrome coro ···················· ···················· ···················· ···················· ···················· ····················
10021 MW153724.1 |Severe acute respiratory syndrome coro ···················· ···················· ···················· ···················· ···················· ····················
10022 MW153726.1 |Severe acute respiratory syndrome coro ···················· ···················· ···················· ···················· ···················· ····················
10023 MW153727.1 |Severe acute respiratory syndrome coro ···················· ···················· ···················· ···················· ···················· ····················
10024 MW153728.1 |Severe acute respiratory syndrome coro ···················· ···················· ···················· ···················· ···················· ····················
10025 MW153729.1 |Severe acute respiratory syndrome coro ···················· ···················· ···················· ···················· ···················· ····················
10026 MW153730.1 |Severe acute respiratory syndrome coro ···················· ···················· ···················· ···················· ···················· ····················
10027 MW153731.1 |Severe acute respiratory syndrome coro ···················· ···················· ···················· ···················· ···················· ····················
10028 MW153732.1 |Severe acute respiratory syndrome coro ···················· ···················· ···················· ···················· ···················· ····················
10029 MW153733.1 |Severe acute respiratory syndrome coro ···················· ···················· ···················· ···················· ···················· ····················
10030 MW153734.1 |Severe acute respiratory syndrome coro ···················· ···················· ···················· ···················· ···················· ····················
10031 MW153735.1 |Severe acute respiratory syndrome coro ···················· ···················· ···················· ···················· ···················· ····················
10032 MW153736.1 |Severe acute respiratory syndrome coro ···················· ···················· ···················· ···················· ···················· ····················
10033 MW153737.1 |Severe acute respiratory syndrome coro ···················· ···················· ···················· ···················· ···················· ····················
10034 MW153738.1 |Severe acute respiratory syndrome coro ···················· ···················· ···················· ···················· ···················· ····················
10035 MW153739.1 |Severe acute respiratory syndrome coro ···················· ···················· ···················· ···················· ···················· ····················
10036 MW153740.1 |Severe acute respiratory syndrome coro ···················· ···················· ···················· ···················· ···················· ····················
10037 MW153741.1 |Severe acute respiratory syndrome coro ···················· ···················· ···················· ···················· ···················· ····················
10038 MW153742.1 |Severe acute respiratory syndrome coro ···················· ···················· ···················· ···················· ···················· ····················
10039 MW153743.1 |Severe acute respiratory syndrome coro ···················· ···················· ···················· ···················· ···················· ····················
10040 MW153744.1 |Severe acute respiratory syndrome coro ···················· ···················· ···················· ···················· ···················· ····················
10041 MW153746.1 |Severe acute respiratory syndrome coro ···················· ···················· ···················· ···················· ···················· ····················
10042 MW153747.1 |Severe acute respiratory syndrome coro ···················· ···················· ···················· ···················· ···················· ····················
10043 MW153748.1 |Severe acute respiratory syndrome coro ···················· ···················· ···················· ···················· ···················· ····················
10044 MW153749.1 |Severe acute respiratory syndrome coro ···················· ···················· ···················· ···················· ···················· ····················
10045 MW153750.1 |Severe acute respiratory syndrome coro ···················· ···················· ···················· ···················· ···················· ····················
10046 MW153751.1 |Severe acute respiratory syndrome coro ···················· ···················· ···················· ···················· ···················· ····················
10047 MW153752.1 |Severe acute respiratory syndrome coro ···················· ···················· ···················· ···················· ···················· ····················
10048 MW153753.1 |Severe acute respiratory syndrome coro ···················· ···················· ···················· ···················· ···················· ····················
10049 MW153754.1 |Severe acute respiratory syndrome coro ···················· ···················· ···················· ···················· ···················· ····················
10050 MW153755.1 |Severe acute respiratory syndrome coro ···················· ···················· ···················· ···················· ···················· ····················
10051 MW153756.1 |Severe acute respiratory syndrome coro ···················· ···················· ···················· ···················· ···················· ····················
10052 MW153757.1 |Severe acute respiratory syndrome coro ···················· ···················· ···················· ···················· ···················· ····················
10053 MW153758.1 |Severe acute respiratory syndrome coro ·····G·············· ···················· ···················· ···················· ···················· ····················
10054 MW153759.1 |Severe acute respiratory syndrome coro ···················· ···················· ···················· ···················· ···················· ····················
10055 MW153760.1 |Severe acute respiratory syndrome coro ···················· ···················· ···················· ···················· ···················· ····················
10056 MW153762.1 |Severe acute respiratory syndrome coro ···················· ···················· ···················· ···················· ···················· ····················
10057 MW153764.1 |Severe acute respiratory syndrome coro ···················· ···················· ···················· ···················· ···················· ····················
10058 MW153765.1 |Severe acute respiratory syndrome coro ···················· ···················· ···················· ···················· ···················· ····················
10059 MW153766.1 |Severe acute respiratory syndrome coro ···················· ···················· ···················· ···················· ···················· ····················
10060 MW153767.1 |Severe acute respiratory syndrome coro ···················· ···················· ···················· ···················· ···················· ····················
10061 MW153768.1 |Severe acute respiratory syndrome coro ···················· ···················· ···················· ···················· ···················· ····················
10062 MW153769.1 |Severe acute respiratory syndrome coro ···················· ···················· ···················· ···················· ···················· ····················
10063 MW153771.1 |Severe acute respiratory syndrome coro ···················· ···················· ···················· ···················· ···················· ····················
10064 MW153774.1 |Severe acute respiratory syndrome coro ···················· ···················· ···················· ···················· ···················· ····················
10065 MW153775.1 |Severe acute respiratory syndrome coro ···················· ···················· ···················· ···················· ···················· ····················
10066 MW153776.1 |Severe acute respiratory syndrome coro ···················· ···················· ···················· ···················· ···················· ····················
10067 MW153778.1 |Severe acute respiratory syndrome coro ···················· ···················· ···················· ···················· ···················· ····················
10068 MW153779.1 |Severe acute respiratory syndrome coro ···················· ···················· ···················· ···················· ···················· ····················
10069 MW153780.1 |Severe acute respiratory syndrome coro ···················· ···················· ···················· ···················· ···················· ····················
10070 MW153781.1 |Severe acute respiratory syndrome coro ···················· ···················· ···················· ···················· ···················· ····················
10071 MW153782.1 |Severe acute respiratory syndrome coro ···················· ···················· ···················· ···················· ···················· ····················
10072 MW153783.1 |Severe acute respiratory syndrome coro ···················· ···················· ···················· ···················· ···················· ····················
10073 MW153784.1 |Severe acute respiratory syndrome coro ···················· ···················· ···················· ···················· ···················· ····················
10074 MW153785.1 |Severe acute respiratory syndrome coro ···················· ···················· ···················· ···················· ···················· ····················
10075 MW153786.1 |Severe acute respiratory syndrome coro ···················· ···················· ···················· ···················· ···················· ····················
10076 MW153787.1 |Severe acute respiratory syndrome coro ···················· ···················· ···················· ···················· ···················· ····················

10077 MW153788.1 |Severe acute respiratory syndrome coro ....
10078 MW153789.1 |Severe acute respiratory syndrome coro ....
10079 MW153790.1 |Severe acute respiratory syndrome coro ....
10080 MW153791.1 |Severe acute respiratory syndrome coro ....
10081 MW153792.1 |Severe acute respiratory syndrome coro ....
10082 MW153793.1 |Severe acute respiratory syndrome coro ....
10083 MW153794.1 |Severe acute respiratory syndrome coro ....
10084 MW153795.1 |Severe acute respiratory syndrome coro ....
10085 MW153796.1 |Severe acute respiratory syndrome coro ....
10086 MW153797.1 |Severe acute respiratory syndrome coro ....
10087 MW153799.1 |Severe acute respiratory syndrome coro ....
10088 MW153801.1 |Severe acute respiratory syndrome coro ....
10089 MW153802.1 |Severe acute respiratory syndrome coro ....
10090 MW153803.1 |Severe acute respiratory syndrome coro ....
10091 MW153804.1 |Severe acute respiratory syndrome coro ....
10092 MW153805.1 |Severe acute respiratory syndrome coro ....
10093 MW153806.1 |Severe acute respiratory syndrome coro ....
10094 MW153807.1 |Severe acute respiratory syndrome coro ....
10095 MW153809.1 |Severe acute respiratory syndrome coro ....
10096 MW153811.1 |Severe acute respiratory syndrome coro ....
10097 MW153812.1 |Severe acute respiratory syndrome coro ....
10098 MW153813.1 |Severe acute respiratory syndrome coro ....
10099 MW153814.1 |Severe acute respiratory syndrome coro ....
10100 MW153815.1 |Severe acute respiratory syndrome coro ....
10101 MW153816.1 |Severe acute respiratory syndrome coro ....
10102 MW153817.1 |Severe acute respiratory syndrome coro ....
10103 MW153818.1 |Severe acute respiratory syndrome coro ....
10104 MW153819.1 |Severe acute respiratory syndrome coro ....
10105 MW153820.1 |Severe acute respiratory syndrome coro ....
10106 MW153821.1 |Severe acute respiratory syndrome coro ....
10107 MW153822.1 |Severe acute respiratory syndrome coro ....
10108 MW153823.1 |Severe acute respiratory syndrome coro ....
10109 MW153824.1 |Severe acute respiratory syndrome coro ....
10110 MW153825.1 |Severe acute respiratory syndrome coro ....
10111 MW153826.1 |Severe acute respiratory syndrome coro ....
10112 MW153827.1 |Severe acute respiratory syndrome coro ....
10113 MW153828.1 |Severe acute respiratory syndrome coro ....
10114 MW153829.1 |Severe acute respiratory syndrome coro ....
10115 MW153830.1 |Severe acute respiratory syndrome coro ....
10116 MW153831.1 |Severe acute respiratory syndrome coro ....
10117 MW153832.1 |Severe acute respiratory syndrome coro ....
10118 MW153833.1 |Severe acute respiratory syndrome coro ....
10119 MW153834.1 |Severe acute respiratory syndrome coro ....
10120 MW153835.1 |Severe acute respiratory syndrome coro ....
10121 MW153836.1 |Severe acute respiratory syndrome coro ....
10122 MW153837.1 |Severe acute respiratory syndrome coro ....
10123 MW153838.1 |Severe acute respiratory syndrome coro ....
10124 MW153839.1 |Severe acute respiratory syndrome coro ....
10125 MW153840.1 |Severe acute respiratory syndrome coro ....
10126 MW153841.1 |Severe acute respiratory syndrome coro ....
10127 MW153842.1 |Severe acute respiratory syndrome coro ....
10128 MW153843.1 |Severe acute respiratory syndrome coro ....
10129 MW153845.1 |Severe acute respiratory syndrome coro ....
10130 MW153846.1 |Severe acute respiratory syndrome coro ....
10131 MW153847.1 |Severe acute respiratory syndrome coro ....
10132 MW153848.1 |Severe acute respiratory syndrome coro ....
10133 MW153849.1 |Severe acute respiratory syndrome coro ....
10134 MW153850.1 |Severe acute respiratory syndrome coro ....
10135 MW153851.1 |Severe acute respiratory syndrome coro ....
10136 MW153853.1 |Severe acute respiratory syndrome coro ....
10137 MW153854.1 |Severe acute respiratory syndrome coro ....
10138 MW153856.1 |Severe acute respiratory syndrome coro ....
10139 MW153858.1 |Severe acute respiratory syndrome coro ....
10140 MW153859.1 |Severe acute respiratory syndrome coro ....
10141 MW153860.1 |Severe acute respiratory syndrome coro ....
10142 MW153861.1 |Severe acute respiratory syndrome coro ....
10143 MW153862.1 |Severe acute respiratory syndrome coro ....
10144 MW153863.1 |Severe acute respiratory syndrome coro ....
10145 MW153864.1 |Severe acute respiratory syndrome coro ....
10146 MW153865.1 |Severe acute respiratory syndrome coro ....
10147 MW153866.1 |Severe acute respiratory syndrome coro ....
10148 MW153867.1 |Severe acute respiratory syndrome coro ....
10149 MW153868.1 |Severe acute respiratory syndrome coro ....
10150 MW153869.1 |Severe acute respiratory syndrome coro ....
10151 MW153870.1 |Severe acute respiratory syndrome coro ....
10152 MW153871.1 |Severe acute respiratory syndrome coro ....
10153 MW153872.1 |Severe acute respiratory syndrome coro ....
10154 MW153873.1 |Severe acute respiratory syndrome coro ....

10155 MW153875.1 |Severe acute respiratory syndrome coro ....
10156 MW153876.1 |Severe acute respiratory syndrome coro ....
10157 MW153877.1 |Severe acute respiratory syndrome coro ....
10158 MW153878.1 |Severe acute respiratory syndrome coro ....
10159 MW153879.1 |Severe acute respiratory syndrome coro ....
10160 MW153880.1 |Severe acute respiratory syndrome coro ....
10161 MW153881.1 |Severe acute respiratory syndrome coro ....
10162 MW153882.1 |Severe acute respiratory syndrome coro ....
10163 MW153883.1 |Severe acute respiratory syndrome coro ....
10164 MW153884.1 |Severe acute respiratory syndrome coro ....
10165 MW153885.1 |Severe acute respiratory syndrome coro ....
10166 MW153888.1 |Severe acute respiratory syndrome coro ....
10167 MW153889.1 |Severe acute respiratory syndrome coro ....
10168 MW153891.1 |Severe acute respiratory syndrome coro ....
10169 MW153893.1 |Severe acute respiratory syndrome coro ....
10170 MW153896.1 |Severe acute respiratory syndrome coro ....
10171 MW153897.1 |Severe acute respiratory syndrome coro ....
10172 MW153898.1 |Severe acute respiratory syndrome coro ....
10173 MW153899.1 |Severe acute respiratory syndrome coro ....
10174 MW153900.1 |Severe acute respiratory syndrome coro ....
10175 MW153901.1 |Severe acute respiratory syndrome coro ....
10176 MW153902.1 |Severe acute respiratory syndrome coro ....
10177 MW153903.1 |Severe acute respiratory syndrome coro ....
10178 MW153905.1 |Severe acute respiratory syndrome coro ....
10179 MW153906.1 |Severe acute respiratory syndrome coro ....
10180 MW153907.1 |Severe acute respiratory syndrome coro ....
10181 MW153908.1 |Severe acute respiratory syndrome coro ....
10182 MW153909.1 |Severe acute respiratory syndrome coro ....
10183 MW153910.1 |Severe acute respiratory syndrome coro ....
10184 MW153911.1 |Severe acute respiratory syndrome coro ....
10185 MW153912.1 |Severe acute respiratory syndrome coro ....
10186 MW153913.1 |Severe acute respiratory syndrome coro ....
10187 MW153914.1 |Severe acute respiratory syndrome coro ....
10188 MW153915.1 |Severe acute respiratory syndrome coro ....
10189 MW153916.1 |Severe acute respiratory syndrome coro ....
10190 MW153917.1 |Severe acute respiratory syndrome coro ....
10191 MW153918.1 |Severe acute respiratory syndrome coro ....
10192 MW153919.1 |Severe acute respiratory syndrome coro ....
10193 MW153920.1 |Severe acute respiratory syndrome coro ....
10194 MW153921.1 |Severe acute respiratory syndrome coro ....
10195 MW153922.1 |Severe acute respiratory syndrome coro ....
10196 MW153923.1 |Severe acute respiratory syndrome coro ....
10197 MW153925.1 |Severe acute respiratory syndrome coro ....
10198 MW153926.1 |Severe acute respiratory syndrome coro ....
10199 MW153927.1 |Severe acute respiratory syndrome coro ....
10200 MW153928.1 |Severe acute respiratory syndrome coro ....
10201 MW153929.1 |Severe acute respiratory syndrome coro ....
10202 MW153931.1 |Severe acute respiratory syndrome coro ....
10203 MW153932.1 |Severe acute respiratory syndrome coro ....
10204 MW153933.1 |Severe acute respiratory syndrome coro ....
10205 MW153934.1 |Severe acute respiratory syndrome coro ....A............. ....
10206 MW153935.1 |Severe acute respiratory syndrome coro ....A............. ....
10207 MW153936.1 |Severe acute respiratory syndrome coro ....
10208 MW153937.1 |Severe acute respiratory syndrome coro ....
10209 MW153938.1 |Severe acute respiratory syndrome coro ....
10210 MW153939.1 |Severe acute respiratory syndrome coro ....A............. ....
10211 MW153940.1 |Severe acute respiratory syndrome coro ....A............. ....
10212 MW153941.1 |Severe acute respiratory syndrome coro ....
10213 MW153942.1 |Severe acute respiratory syndrome coro ....
10214 MW153943.1 |Severe acute respiratory syndrome coro ....
10215 MW153944.1 |Severe acute respiratory syndrome coro ....
10216 MW153945.1 |Severe acute respiratory syndrome coro ....A............. ....
10217 MW153946.1 |Severe acute respiratory syndrome coro ....
10218 MW153947.1 |Severe acute respiratory syndrome coro ....
10219 MW153948.1 |Severe acute respiratory syndrome coro ....
10220 MW153949.1 |Severe acute respiratory syndrome coro ....
10221 MW153950.1 |Severe acute respiratory syndrome coro ....
10222 MW153951.1 |Severe acute respiratory syndrome coro ....
10223 MW153952.1 |Severe acute respiratory syndrome coro ....
10224 MW153953.1 |Severe acute respiratory syndrome coro ....
10225 MW153954.1 |Severe acute respiratory syndrome coro ....
10226 MW153955.1 |Severe acute respiratory syndrome coro ....
10227 MW153956.1 |Severe acute respiratory syndrome coro ....
10228 MW153957.1 |Severe acute respiratory syndrome coro ....
10229 MW153958.1 |Severe acute respiratory syndrome coro ....
10230 MW153959.1 |Severe acute respiratory syndrome coro ....
10231 MW153960.1 |Severe acute respiratory syndrome coro ....
10232 MW153961.1 |Severe acute respiratory syndrome coro ....

10233 MW153962.1 |Severe acute respiratory syndrome coro ....
10234 MW153963.1 |Severe acute respiratory syndrome coro ....
10235 MW153964.1 |Severe acute respiratory syndrome coro ....
10236 MW153965.1 |Severe acute respiratory syndrome coro ....
10237 MW153966.1 |Severe acute respiratory syndrome coro ....
10238 MW153967.1 |Severe acute respiratory syndrome coro ....
10239 MW153968.1 |Severe acute respiratory syndrome coro ....
10240 MW153969.1 |Severe acute respiratory syndrome coro ....
10241 MW153970.1 |Severe acute respiratory syndrome coro ....
10242 MW153971.1 |Severe acute respiratory syndrome coro ....
10243 MW153972.1 |Severe acute respiratory syndrome coro ....
10244 MW153973.1 |Severe acute respiratory syndrome coro ....
10245 MW153974.1 |Severe acute respiratory syndrome coro ....
10246 MW153975.1 |Severe acute respiratory syndrome coro ....
10247 MW153976.1 |Severe acute respiratory syndrome coro ....
10248 MW153977.1 |Severe acute respiratory syndrome coro ....
10249 MW153978.1 |Severe acute respiratory syndrome coro ....
10250 MW153979.1 |Severe acute respiratory syndrome coro ....
10251 MW153980.1 |Severe acute respiratory syndrome coro ....
10252 MW153981.1 |Severe acute respiratory syndrome coro ....
10253 MW153982.1 |Severe acute respiratory syndrome coro ....
10254 MW153983.1 |Severe acute respiratory syndrome coro ....
10255 MW153984.1 |Severe acute respiratory syndrome coro ....
10256 MW153985.1 |Severe acute respiratory syndrome coro ....
10257 MW153986.1 |Severe acute respiratory syndrome coro ....
10258 MW153987.1 |Severe acute respiratory syndrome coro ....
10259 MW153988.1 |Severe acute respiratory syndrome coro ....
10260 MW153989.1 |Severe acute respiratory syndrome coro ....
10261 MW153990.1 |Severe acute respiratory syndrome coro ....
10262 MW153992.1 |Severe acute respiratory syndrome coro ....
10263 MW153993.1 |Severe acute respiratory syndrome coro ....
10264 MW153994.1 |Severe acute respiratory syndrome coro ....
10265 MW153995.1 |Severe acute respiratory syndrome coro ....
10266 MW153996.1 |Severe acute respiratory syndrome coro ....
10267 MW153997.1 |Severe acute respiratory syndrome coro ....
10268 MW153998.1 |Severe acute respiratory syndrome coro ....
10269 MW153999.1 |Severe acute respiratory syndrome coro ....
10270 MW154000.1 |Severe acute respiratory syndrome coro ....
10271 MW154001.1 |Severe acute respiratory syndrome coro ....
10272 MW154002.1 |Severe acute respiratory syndrome coro ....
10273 MW154003.1 |Severe acute respiratory syndrome coro ....
10274 MW154005.1 |Severe acute respiratory syndrome coro ....
10275 MW154006.1 |Severe acute respiratory syndrome coro ....
10276 MW154007.1 |Severe acute respiratory syndrome coro ....
10277 MW154008.1 |Severe acute respiratory syndrome coro ....
10278 MW154010.1 |Severe acute respiratory syndrome coro ....
10279 MW154011.1 |Severe acute respiratory syndrome coro ....
10280 MW154012.1 |Severe acute respiratory syndrome coro ....
10281 MW154013.1 |Severe acute respiratory syndrome coro ....
10282 MW154014.1 |Severe acute respiratory syndrome coro ....
10283 MW154015.1 |Severe acute respiratory syndrome coro ....
10284 MW154016.1 |Severe acute respiratory syndrome coro ....
10285 MW154017.1 |Severe acute respiratory syndrome coro ....
10286 MW154018.1 |Severe acute respiratory syndrome coro ....
10287 MW154019.1 |Severe acute respiratory syndrome coro ....
10288 MW154020.1 |Severe acute respiratory syndrome coro ....
10289 MW154021.1 |Severe acute respiratory syndrome coro ....
10290 MW154022.1 |Severe acute respiratory syndrome coro ....
10291 MW154023.1 |Severe acute respiratory syndrome coro ....
10292 MW154024.1 |Severe acute respiratory syndrome coro ....
10293 MW154025.1 |Severe acute respiratory syndrome coro ....
10294 MW154026.1 |Severe acute respiratory syndrome coro ....
10295 MW154027.1 |Severe acute respiratory syndrome coro ....
10296 MW154028.1 |Severe acute respiratory syndrome coro ....
10297 MW154029.1 |Severe acute respiratory syndrome coro ....
10298 MW154030.1 |Severe acute respiratory syndrome coro ....
10299 MW154031.1 |Severe acute respiratory syndrome coro ....
10300 MW154032.1 |Severe acute respiratory syndrome coro ....
10301 MW154033.1 |Severe acute respiratory syndrome coro ....
10302 MW154034.1 |Severe acute respiratory syndrome coro ....
10303 MW154035.1 |Severe acute respiratory syndrome coro ....
10304 MW154036.1 |Severe acute respiratory syndrome coro ....
10305 MW154037.1 |Severe acute respiratory syndrome coro ....
10306 MW154038.1 |Severe acute respiratory syndrome coro ....
10307 MW154039.1 |Severe acute respiratory syndrome coro ....
10308 MW154040.1 |Severe acute respiratory syndrome coro ....
10309 MW154042.1 |Severe acute respiratory syndrome coro ....
10310 MW154043.1 |Severe acute respiratory syndrome coro ....

10311 MW154044.1 |Severe acute respiratory syndrome coro ....
10312 MW154045.1 |Severe acute respiratory syndrome coro ....
10313 MW154046.1 |Severe acute respiratory syndrome coro ....
10314 MW154047.1 |Severe acute respiratory syndrome coro ....
10315 MW154048.1 |Severe acute respiratory syndrome coro ....
10316 MW154049.1 |Severe acute respiratory syndrome coro ....
10317 MW154050.1 |Severe acute respiratory syndrome coro ....
10318 MW154051.1 |Severe acute respiratory syndrome coro ....
10319 MW154052.1 |Severe acute respiratory syndrome coro ....
10320 MW154053.1 |Severe acute respiratory syndrome coro ....
10321 MW154054.1 |Severe acute respiratory syndrome coro ....
10322 MW154055.1 |Severe acute respiratory syndrome coro ....
10323 MW154056.1 |Severe acute respiratory syndrome coro ....
10324 MW154057.1 |Severe acute respiratory syndrome coro ....
10325 MW154058.1 |Severe acute respiratory syndrome coro ....
10326 MW154059.1 |Severe acute respiratory syndrome coro ....
10327 MW154060.1 |Severe acute respiratory syndrome coro ....
10328 MW154061.1 |Severe acute respiratory syndrome coro ....
10329 MW154062.1 |Severe acute respiratory syndrome coro ....
10330 MW154063.1 |Severe acute respiratory syndrome coro ....
10331 MW154064.1 |Severe acute respiratory syndrome coro ....
10332 MW154065.1 |Severe acute respiratory syndrome coro ....
10333 MW154066.1 |Severe acute respiratory syndrome coro ....
10334 MW154067.1 |Severe acute respiratory syndrome coro ....
10335 MW154068.1 |Severe acute respiratory syndrome coro ....
10336 MW154069.1 |Severe acute respiratory syndrome coro ....
10337 MW154070.1 |Severe acute respiratory syndrome coro ....
10338 MW154071.1 |Severe acute respiratory syndrome coro ....
10339 MW154072.1 |Severe acute respiratory syndrome coro ....
10340 MW154073.1 |Severe acute respiratory syndrome coro ....
10341 MW154074.1 |Severe acute respiratory syndrome coro ....
10342 MW154075.1 |Severe acute respiratory syndrome coro ....
10343 MW154076.1 |Severe acute respiratory syndrome coro ....
10344 MW154077.1 |Severe acute respiratory syndrome coro ....
10345 MW154078.1 |Severe acute respiratory syndrome coro ....
10346 MW154079.1 |Severe acute respiratory syndrome coro ....
10347 MW154080.1 |Severe acute respiratory syndrome coro ....
10348 MW154081.1 |Severe acute respiratory syndrome coro ....
10349 MW154082.1 |Severe acute respiratory syndrome coro ....
10350 MW154083.1 |Severe acute respiratory syndrome coro ....
10351 MW154084.1 |Severe acute respiratory syndrome coro ....
10352 MW154085.1 |Severe acute respiratory syndrome coro ....
10353 MW154086.1 |Severe acute respiratory syndrome coro ....
10354 MW154087.1 |Severe acute respiratory syndrome coro ....
10355 MW154088.1 |Severe acute respiratory syndrome coro ....
10356 MW154089.1 |Severe acute respiratory syndrome coro ....
10357 MW154090.1 |Severe acute respiratory syndrome coro ....
10358 MW154095.1 |Severe acute respiratory syndrome coro ....
10359 MW154096.1 |Severe acute respiratory syndrome coro ....
10360 MW154098.1 |Severe acute respiratory syndrome coro ....
10361 MW154099.1 |Severe acute respiratory syndrome coro ....
10362 MW154100.1 |Severe acute respiratory syndrome coro ....
10363 MW154101.1 |Severe acute respiratory syndrome coro ....
10364 MW154103.1 |Severe acute respiratory syndrome coro ....
10365 MW154104.1 |Severe acute respiratory syndrome coro ....
10366 MW154105.1 |Severe acute respiratory syndrome coro ....
10367 MW154106.1 |Severe acute respiratory syndrome coro ....
10368 MW154107.1 |Severe acute respiratory syndrome coro ....
10369 MW154108.1 |Severe acute respiratory syndrome coro ....
10370 MW154109.1 |Severe acute respiratory syndrome coro ....
10371 MW154110.1 |Severe acute respiratory syndrome coro ....
10372 MW154111.1 |Severe acute respiratory syndrome coro ....
10373 MW154112.1 |Severe acute respiratory syndrome coro ....
10374 MW154113.1 |Severe acute respiratory syndrome coro ....
10375 MW154115.1 |Severe acute respiratory syndrome coro ....
10376 MW154116.1 |Severe acute respiratory syndrome coro ....
10377 MW154117.1 |Severe acute respiratory syndrome coro ....
10378 MW154119.1 |Severe acute respiratory syndrome coro ....
10379 MW154120.1 |Severe acute respiratory syndrome coro ....
10380 MW154121.1 |Severe acute respiratory syndrome coro ....
10381 MW154122.1 |Severe acute respiratory syndrome coro ....
10382 MW154123.1 |Severe acute respiratory syndrome coro ....
10383 MW154124.1 |Severe acute respiratory syndrome coro ....
10384 MW154125.1 |Severe acute respiratory syndrome coro ....
10385 MW154126.1 |Severe acute respiratory syndrome coro ....
10386 MW154127.1 |Severe acute respiratory syndrome coro ....
10388 MW154130.1 |Severe acute respiratory syndrome coro ....
10389 MW154131.1 |Severe acute respiratory syndrome coro ....

```
10390 MW154132.1 |Severe acute respiratory syndrome coro    ....................  ....................  ....................  ....................  ....................  ....................
10391 MW154133.1 |Severe acute respiratory syndrome coro    ....................  ....................  ....................  ....................  ....................  ....................
10392 MW154134.1 |Severe acute respiratory syndrome coro    ....................  ....................  ....................  ....................  ....................  ....................
10393 MW154135.1 |Severe acute respiratory syndrome coro    ....................  ....................  ....................  ....................  ....................  ....................
10394 MW154136.1 |Severe acute respiratory syndrome coro    ....................  ....................  ....................  ....................  ....................  ....................
10395 MW154137.1 |Severe acute respiratory syndrome coro    ....................  ....................  ....................  ....................  ....................  ....................
10396 MW154138.1 |Severe acute respiratory syndrome coro    ....................  ....................  ....................  ....................  ....................  ....................
10397 MW154139.1 |Severe acute respiratory syndrome coro    ....................  ....................  ....................  ....................  ....................  ....................
10398 MW154140.1 |Severe acute respiratory syndrome coro    ....................  ....................  ....................  ....................  ....................  ....................
10399 MW154142.1 |Severe acute respiratory syndrome coro    ....................  ....................  ....................  ....................  ....................  ....................
10400 MW154143.1 |Severe acute respiratory syndrome coro    ....................  ....................  ....................  ....................  ....................  ....................
10401 MW154146.1 |Severe acute respiratory syndrome coro    ....................  ....................  ....................  ....................  ....................  ....................
10402 MW154147.1 |Severe acute respiratory syndrome coro    ....................  ....................  ....................  ....................  ....................  ....................
10403 MW154148.1 |Severe acute respiratory syndrome coro    ....................  ....................  ....................  ....................  ....................  ....................
10404 MW154149.1 |Severe acute respiratory syndrome coro    ....................  ....................  ....................  ....................  ....................  ....................
10405 MW154151.1 |Severe acute respiratory syndrome coro    ....................  ....................  ....................  ....................  ....................  ....................
10406 MW154152.1 |Severe acute respiratory syndrome coro    ....................  ....................  ....................  ....................  ....................  ....................
10407 MW154153.1 |Severe acute respiratory syndrome coro    ....................  ....................  ....................  ....................  ....................  ....................
10408 MW154154.1 |Severe acute respiratory syndrome coro    ....................  ....................  ....................  ....................  ....................  ....................
10409 MW154155.1 |Severe acute respiratory syndrome coro    ....................  ....................  ....................  ....................  ....................  ....................
10410 MW154156.1 |Severe acute respiratory syndrome coro    ....................  ....................  ....................  ....................  ....................  ....................
10411 MW154157.1 |Severe acute respiratory syndrome coro    ....................  ....................  ....................  ....................  ....................  ....................
10412 MW154158.1 |Severe acute respiratory syndrome coro    ....................  ....................  ....................  ....................  ....................  ....................
10413 MW154160.1 |Severe acute respiratory syndrome coro    ....................  ....................  ....................  ....................  ....................  ....................
10414 MW154161.1 |Severe acute respiratory syndrome coro    ....................  ....................  ....................  ....................  ....................  ....................
10415 MW154162.1 |Severe acute respiratory syndrome coro    ....................  ....................  ....................  ....................  ....................  ....................
10416 MW154163.1 |Severe acute respiratory syndrome coro    ....................  ....................  ....................  ....................  ....................  ....................
10417 MW154164.1 |Severe acute respiratory syndrome coro    ....................  ....................  ....................  ....................  ....................  ....................
10418 MW154165.1 |Severe acute respiratory syndrome coro    ....................  ....................  ....................  ....................  ....................  ....................
10419 MW154166.1 |Severe acute respiratory syndrome coro    ....................  ....................  ....................  ....................  ....................  ....................
10420 MW154168.1 |Severe acute respiratory syndrome coro    ....................  ....................  ....................  ....................  ....................  ....................
10421 MW154169.1 |Severe acute respiratory syndrome coro    ....................  ....................  ....................  ....................  ....................  ....................
10422 MW154171.1 |Severe acute respiratory syndrome coro    ....................  ....................  ....................  ....................  ....................  ....................
10423 MW154172.1 |Severe acute respiratory syndrome coro    ....................  ....................  ....................  ....................  ....................  ....................
10424 MW154173.1 |Severe acute respiratory syndrome coro    ....................  ....................  ....................  ....................  ....................  ....................
10425 MW154174.1 |Severe acute respiratory syndrome coro    ....................  ....................  ....................  ....................  ....................  ....................
10426 MW154177.1 |Severe acute respiratory syndrome coro    ....................  ....................  ....................  ....................  ....................  ....................
10427 MW154178.1 |Severe acute respiratory syndrome coro    ....................  ....................  ....................  ....................  ....................  ....................
10428 MW154179.1 |Severe acute respiratory syndrome coro    ....................  ....................  ....................  ....................  ....................  ....................
10429 MW154181.1 |Severe acute respiratory syndrome coro    ....................  ....................  ....................  ....................  ....................  ....................
10430 MW154182.1 |Severe acute respiratory syndrome coro    ....................  ....................  ....................  ....................  ....................  ....................
10431 MW154183.1 |Severe acute respiratory syndrome coro    ....................  ....................  ....................  ....................  ....................  ....................
10432 MW154184.1 |Severe acute respiratory syndrome coro    ....................  ....................  ....................  ....................  ....................  ....................
10433 MW154185.1 |Severe acute respiratory syndrome coro    ....................  ....................  ....................  ....................  ....................  ....................
10434 MW154186.1 |Severe acute respiratory syndrome coro    ....................  ....................  ....................  ....................  ....................  ....................
10435 MW154187.1 |Severe acute respiratory syndrome coro    ....................  ....................  ....................  ....................  ....................  ....................
10436 MW154188.1 |Severe acute respiratory syndrome coro    ....................  ....................  ....................  ....................  ....................  ....................
10437 MW154189.1 |Severe acute respiratory syndrome coro    ....................  ....................  ....................  ....................  ....................  ....................
10438 MW154190.1 |Severe acute respiratory syndrome coro    ....................  ....................  ....................  ....................  ....................  ....................
10439 MW154191.1 |Severe acute respiratory syndrome coro    ....................  ....................  ....................  ....................  ....................  ....................
10440 MW154192.1 |Severe acute respiratory syndrome coro    ....................  ....................  ....................  ....................  ....................  ....................
10441 MW154193.1 |Severe acute respiratory syndrome coro    ....................  ....................  ....................  ....................  ....................  ....................
10442 MW154194.1 |Severe acute respiratory syndrome coro    ....................  ....................  ....................  ....................  ....................  ....................
10443 MW154196.1 |Severe acute respiratory syndrome coro    ....................  ....................  ....................  ....................  ....................  ....................
10444 MW154197.1 |Severe acute respiratory syndrome coro    ....................  ....................  ....................  ....................  ....................  ....................
10445 MW154198.1 |Severe acute respiratory syndrome coro    ....................  ....................  ....................  ....................  ....................  ....................
10446 MW154199.1 |Severe acute respiratory syndrome coro    ....................  ....................  ....................  ....................  ....................  ....................
10447 MW154201.1 |Severe acute respiratory syndrome coro    ....................  ....................  ....................  ....................  ....................  ....................
10448 MW154202.1 |Severe acute respiratory syndrome coro    ....................  ....................  ....................  ....................  ....................  ....................
10449 MW154203.1 |Severe acute respiratory syndrome coro    ....................  ....................  ....................  ....................  ....................  ....................
10450 MW154204.1 |Severe acute respiratory syndrome coro    ....................  ....................  ....................  ....................  ....................  ....................
10451 MW154205.1 |Severe acute respiratory syndrome coro    ....................  ..........A.........  ....................  ....................  ....................  ....................
10452 MW154206.1 |Severe acute respiratory syndrome coro    ....................  ....................  ....................  ....................  ....................  ....................
10453 MW154207.1 |Severe acute respiratory syndrome coro    ....................  ....................  ....................  ....................  ....................  ....................
10454 MW154208.1 |Severe acute respiratory syndrome coro    ....................  ....................  ....................  ....................  ....................  ....................
10455 MW154209.1 |Severe acute respiratory syndrome coro    ....................  ....................  ....................  ....................  ....................  ....................
10456 MW154211.1 |Severe acute respiratory syndrome coro    ....................  ....................  ....................  ....................  ....................  ....................
10457 MW154212.1 |Severe acute respiratory syndrome coro    ....................  ....................  ....................  ....................  ....................  ....................
10458 MW154213.1 |Severe acute respiratory syndrome coro    ....................  ....................  ....................  ....................  ....................  ....................
10459 MW154214.1 |Severe acute respiratory syndrome coro    ....................  ....................  ....................  ....................  ....................  ....................
10460 MW154216.1 |Severe acute respiratory syndrome coro    ....................  ....................  ....................  ....................  ....................  ....................
10461 MW154217.1 |Severe acute respiratory syndrome coro    ....................  ....................  ....................  ....................  ....................  ....................
10462 MW154218.1 |Severe acute respiratory syndrome coro    ....................  ....................  ....................  ....................  ....................  ....................
10463 MW154219.1 |Severe acute respiratory syndrome coro    ....................  ....................  ....................  ....................  ....................  ....................
10464 MW154221.1 |Severe acute respiratory syndrome coro    ....................  ....................  ....................  ....................  ....................  ....................
10465 MW154222.1 |Severe acute respiratory syndrome coro    ....................  ....................  ....................  ....................  ....................  ....................
10466 MW154223.1 |Severe acute respiratory syndrome coro    ....................  ....................  ....................  ....................  ....................  ....................
10467 MW154224.1 |Severe acute respiratory syndrome coro    ....................  ....................  ....................  ....................  ....................  ....................
```

10468 MW154225.1 |Severe acute respiratory syndrome coro ....
10469 MW154226.1 |Severe acute respiratory syndrome coro ....
10470 MW154227.1 |Severe acute respiratory syndrome coro ....
10471 MW154228.1 |Severe acute respiratory syndrome coro ....
10472 MW154229.1 |Severe acute respiratory syndrome coro ....
10473 MW154230.1 |Severe acute respiratory syndrome coro ....
10474 MW154231.1 |Severe acute respiratory syndrome coro ....
10475 MW154232.1 |Severe acute respiratory syndrome coro ....
10476 MW154233.1 |Severe acute respiratory syndrome coro ....
10477 MW154234.1 |Severe acute respiratory syndrome coro ....
10478 MW154235.1 |Severe acute respiratory syndrome coro ....
10479 MW154236.1 |Severe acute respiratory syndrome coro ....
10480 MW154237.1 |Severe acute respiratory syndrome coro ....
10481 MW154238.1 |Severe acute respiratory syndrome coro ....
10482 MW154239.1 |Severe acute respiratory syndrome coro ....
10483 MW154240.1 |Severe acute respiratory syndrome coro ....
10484 MW154241.1 |Severe acute respiratory syndrome coro ....
10485 MW154242.1 |Severe acute respiratory syndrome coro ....
10486 MW154243.1 |Severe acute respiratory syndrome coro ....
10487 MW154244.1 |Severe acute respiratory syndrome coro ....
10488 MW154245.1 |Severe acute respiratory syndrome coro ....
10489 MW154247.1 |Severe acute respiratory syndrome coro ....A............. ....
10490 MW154248.1 |Severe acute respiratory syndrome coro ....
10491 MW154250.1 |Severe acute respiratory syndrome coro ....
10492 MW154251.1 |Severe acute respiratory syndrome coro ....
10493 MW154252.1 |Severe acute respiratory syndrome coro ....
10494 MW154253.1 |Severe acute respiratory syndrome coro ....
10495 MW154254.1 |Severe acute respiratory syndrome coro ....
10496 MW154255.1 |Severe acute respiratory syndrome coro ....
10497 MW154256.1 |Severe acute respiratory syndrome coro ....
10498 MW154258.1 |Severe acute respiratory syndrome coro ....
10499 MW154259.1 |Severe acute respiratory syndrome coro ....
10500 MW154260.1 |Severe acute respiratory syndrome coro ....
10501 MW154261.1 |Severe acute respiratory syndrome coro ....
10502 MW154262.1 |Severe acute respiratory syndrome coro ....
10503 MW154264.1 |Severe acute respiratory syndrome coro ....
10504 MW154265.1 |Severe acute respiratory syndrome coro ....
10505 MW154266.1 |Severe acute respiratory syndrome coro ....
10506 MW154267.1 |Severe acute respiratory syndrome coro ....
10507 MW154268.1 |Severe acute respiratory syndrome coro ....
10508 MW154269.1 |Severe acute respiratory syndrome coro ....
10509 MW154270.1 |Severe acute respiratory syndrome coro ....
10510 MW154271.1 |Severe acute respiratory syndrome coro ....
10511 MW154272.1 |Severe acute respiratory syndrome coro ....
10512 MW154273.1 |Severe acute respiratory syndrome coro ....
10513 MW154274.1 |Severe acute respiratory syndrome coro ....
10514 MW154275.1 |Severe acute respiratory syndrome coro ....
10515 MW154278.1 |Severe acute respiratory syndrome coro ....
10516 MW154279.1 |Severe acute respiratory syndrome coro ....
10517 MW154280.1 |Severe acute respiratory syndrome coro ....
10518 MW154281.1 |Severe acute respiratory syndrome coro ....
10519 MW154282.1 |Severe acute respiratory syndrome coro ....
10520 MW154283.1 |Severe acute respiratory syndrome coro ....
10521 MW154285.1 |Severe acute respiratory syndrome coro ....
10522 MW154287.1 |Severe acute respiratory syndrome coro ....
10523 MW154289.1 |Severe acute respiratory syndrome coro ....
10524 MW154290.1 |Severe acute respiratory syndrome coro ....
10525 MW154291.1 |Severe acute respiratory syndrome coro ....
10526 MW154292.1 |Severe acute respiratory syndrome coro ....
10527 MW154293.1 |Severe acute respiratory syndrome coro ....A......... ....
10528 MW154295.1 |Severe acute respiratory syndrome coro ....
10529 MW154296.1 |Severe acute respiratory syndrome coro ....
10530 MW154297.1 |Severe acute respiratory syndrome coro ....
10531 MW154298.1 |Severe acute respiratory syndrome coro ....A............. ....
10532 MW154299.1 |Severe acute respiratory syndrome coro ....
10533 MW154301.1 |Severe acute respiratory syndrome coro ....
10534 MW154302.1 |Severe acute respiratory syndrome coro ....
10535 MW154304.1 |Severe acute respiratory syndrome coro ....
10536 MW154305.1 |Severe acute respiratory syndrome coro ....
10537 MW154306.1 |Severe acute respiratory syndrome coro ....
10538 MW154307.1 |Severe acute respiratory syndrome coro ....
10539 MW154308.1 |Severe acute respiratory syndrome coro ....
10540 MW154309.1 |Severe acute respiratory syndrome coro ....
10541 MW154310.1 |Severe acute respiratory syndrome coro ....
10542 MW154311.1 |Severe acute respiratory syndrome coro ....
10543 MW154312.1 |Severe acute respiratory syndrome coro ....
10544 MW154313.1 |Severe acute respiratory syndrome coro ....
10545 MW154314.1 |Severe acute respiratory syndrome coro ....

10546 MW154315.1 |Severe acute respiratory syndrome coro ....
10547 MW154316.1 |Severe acute respiratory syndrome coro ....
10548 MW154317.1 |Severe acute respiratory syndrome coro ....
10549 MW154318.1 |Severe acute respiratory syndrome coro ....
10550 MW154319.1 |Severe acute respiratory syndrome coro ....
10551 MW154320.1 |Severe acute respiratory syndrome coro ....
10552 MW154321.1 |Severe acute respiratory syndrome coro ....
10553 MW154323.1 |Severe acute respiratory syndrome coro ....
10554 MW154324.1 |Severe acute respiratory syndrome coro ....
10555 MW154325.1 |Severe acute respiratory syndrome coro ....
10556 MW154326.1 |Severe acute respiratory syndrome coro ....
10557 MW154327.1 |Severe acute respiratory syndrome coro ....
10558 MW154329.1 |Severe acute respiratory syndrome coro ....
10559 MW154330.1 |Severe acute respiratory syndrome coro ....
10560 MW154331.1 |Severe acute respiratory syndrome coro ....
10561 MW154332.1 |Severe acute respiratory syndrome coro ....
10562 MW154333.1 |Severe acute respiratory syndrome coro ....
10563 MW154334.1 |Severe acute respiratory syndrome coro ....
10564 MW154335.1 |Severe acute respiratory syndrome coro ....
10565 MW154336.1 |Severe acute respiratory syndrome coro ....
10566 MW154338.1 |Severe acute respiratory syndrome coro ....
10567 MW154339.1 |Severe acute respiratory syndrome coro ....
10568 MW154340.1 |Severe acute respiratory syndrome coro ....
10569 MW154341.1 |Severe acute respiratory syndrome coro ....
10570 MW154342.1 |Severe acute respiratory syndrome coro ....
10571 MW154343.1 |Severe acute respiratory syndrome coro ....
10572 MW154345.1 |Severe acute respiratory syndrome coro ....
10573 MW154349.1 |Severe acute respiratory syndrome coro ....
10574 MW154350.1 |Severe acute respiratory syndrome coro ....
10575 MW154351.1 |Severe acute respiratory syndrome coro ....
10576 MW154352.1 |Severe acute respiratory syndrome coro ....
10577 MW154353.1 |Severe acute respiratory syndrome coro ....
10578 MW154355.1 |Severe acute respiratory syndrome coro ....
10579 MW154356.1 |Severe acute respiratory syndrome coro ....
10580 MW154357.1 |Severe acute respiratory syndrome coro ....
10581 MW154358.1 |Severe acute respiratory syndrome coro ....
10582 MW154359.1 |Severe acute respiratory syndrome coro ....
10583 MW154360.1 |Severe acute respiratory syndrome coro ....
10584 MW154362.1 |Severe acute respiratory syndrome coro ....
10585 MW154363.1 |Severe acute respiratory syndrome coro ....
10586 MW154364.1 |Severe acute respiratory syndrome coro ....
10587 MW154365.1 |Severe acute respiratory syndrome coro ....
10588 MW154366.1 |Severe acute respiratory syndrome coro ....
10589 MW154367.1 |Severe acute respiratory syndrome coro ....
10590 MW154368.1 |Severe acute respiratory syndrome coro ....
10591 MW154369.1 |Severe acute respiratory syndrome coro ....
10592 MW154370.1 |Severe acute respiratory syndrome coro ....
10593 MW154371.1 |Severe acute respiratory syndrome coro ....
10594 MW154372.1 |Severe acute respiratory syndrome coro ....
10595 MW154373.1 |Severe acute respiratory syndrome coro ....
10596 MW154374.1 |Severe acute respiratory syndrome coro ....
10597 MW154376.1 |Severe acute respiratory syndrome coro ....A............. ....
10598 MW154378.1 |Severe acute respiratory syndrome coro ....
10599 MW154379.1 |Severe acute respiratory syndrome coro ....
10600 MW154380.1 |Severe acute respiratory syndrome coro ....
10601 MW154381.1 |Severe acute respiratory syndrome coro ....
10602 MW154382.1 |Severe acute respiratory syndrome coro ....
10603 MW154383.1 |Severe acute respiratory syndrome coro ....
10604 MW154385.1 |Severe acute respiratory syndrome coro ....
10605 MW154386.1 |Severe acute respiratory syndrome coro ....
10606 MW154387.1 |Severe acute respiratory syndrome coro ....
10607 MW154388.1 |Severe acute respiratory syndrome coro ....
10608 MW154389.1 |Severe acute respiratory syndrome coro ....
10609 MW154390.1 |Severe acute respiratory syndrome coro ....
10610 MW154391.1 |Severe acute respiratory syndrome coro ....
10611 MW154392.1 |Severe acute respiratory syndrome coro ....
10612 MW154393.1 |Severe acute respiratory syndrome coro ....
10613 MW154394.1 |Severe acute respiratory syndrome coro ....
10614 MW154395.1 |Severe acute respiratory syndrome coro ....
10615 MW154397.1 |Severe acute respiratory syndrome coro ....
10616 MW154399.1 |Severe acute respiratory syndrome coro ....
10617 MW154400.1 |Severe acute respiratory syndrome coro ....
10618 MW154401.1 |Severe acute respiratory syndrome coro ....
10619 MW154402.1 |Severe acute respiratory syndrome coro ....
10620 MW154403.1 |Severe acute respiratory syndrome coro ....
10621 MW154404.1 |Severe acute respiratory syndrome coro ....
10622 MW154405.1 |Severe acute respiratory syndrome coro ....
10623 MW154406.1 |Severe acute respiratory syndrome coro ....

```
10624 MW154407.1 |Severe acute respiratory syndrome coro    ....................  ....................  .....................  ....................  ....................  ....................
10625 MW154408.1 |Severe acute respiratory syndrome coro    ....................  ....................  .....................  ....................  ....................  ....................
10626 MW154409.1 |Severe acute respiratory syndrome coro    ....................  ....................  .....................  ....................  ....................  ....................
10627 MW154411.1 |Severe acute respiratory syndrome coro    ....................  ....................  .....................  ....................  ....................  ....................
10628 MW154412.1 |Severe acute respiratory syndrome coro    ....................  ....................  .....................  ....................  ....................  ....................
10629 MW154413.1 |Severe acute respiratory syndrome coro    ....................  ....................  .....................  ....................  ....................  ....................
10630 MW154417.1 |Severe acute respiratory syndrome coro    ....................  ....................  .....................  ....................  ....................  ....................
10631 MW154419.1 |Severe acute respiratory syndrome coro    ....................  ....................  .....................  ....................  ....................  ....................
10632 MW154421.1 |Severe acute respiratory syndrome coro    ....................  ....................  .....................  ....................  ....................  ....................
10633 MW154423.1 |Severe acute respiratory syndrome coro    ....................  ....................  .....................  ....................  ....................  ....................
10634 MW154424.1 |Severe acute respiratory syndrome coro    ....................  ....................  .....................  ....................  ....................  ....................
10635 MW154425.1 |Severe acute respiratory syndrome coro    ....................  ....................  .....................  ....................  ....................  ....................
10636 MW154426.1 |Severe acute respiratory syndrome coro    ....................  ....................  .....................  ....................  ....................  ....................
10637 MW154428.1 |Severe acute respiratory syndrome coro    ....................  ....................  .....................  ....................  ....................  ....................
10638 MW154429.1 |Severe acute respiratory syndrome coro    ....................  ....................  .....................  ....................  ....................  ....................
10639 MW154430.1 |Severe acute respiratory syndrome coro    ....................  ....................  .....................  ....................  ....................  ....................
10640 MW154431.1 |Severe acute respiratory syndrome coro    ....................  ....................  .....................  ....................  ....................  ....................
10641 MW154432.1 |Severe acute respiratory syndrome coro    ....................  ....................  .....................  ....................  ....................  ....................
10642 MW154433.1 |Severe acute respiratory syndrome coro    ....................  ....................  .....................  ....................  ....................  ....................
```
[truncated: 4,351,781 more chars]
