# Supplementary material for: Development and implementation of a scalable and versatile test for COVID-19 diagnostics in rural communities
Source: Nat Commun. 2021 Jul 20;12:4400. doi: 10.1038/s41467-021-24552-4 (PMC8292415; doi:10.1038/s41467-021-24552-4)
Supplement: Supplementary file 4 — Supplementary Data 2 [file 41467_2021_24552_MOESM4_ESM.pdf]

Group 1 - Jan 2020 - Jun 2020

<table>
<tr><th>A</th><th>B</th><th>C</th><th>D</th><th>E</th><th>F</th><th>G</th><th>H</th><th>I</th><th>J</th><th>K</th><th>L</th></tr>
<tr><th>Database</th><th>Database ID</th><th>GISAID ID</th><th>SK_N forward GCTGCAATCGTGCTACAACT</th><th>SK_N reverse TGAACTGTTGCGACTACGTG</th><th>SK_E forward TTCGGAAGAGACAGGTACGTT</th><th>SK_E reverse CACACAATCGAAGCGCAGTA</th><th>SK_S forward GCTGGTGCTGCAGCTTATTA</th><th>SK_S reverse AGGGTCAAGTGCACAGTCTA</th><th>3' mismatches?</th><th>Multiple mismatches?</th><th>More than 1 primer set?</th></tr>
<tr><td>A</td><td>1</td><td>hCoV-19/Australia/NSW14/2020|EPI_ISL_413600|2020-0</td><td>····················</td><td>····················</td><td>····················</td><td>····················</td><td>····················</td><td>····················</td><td></td><td></td><td></td></tr>
<tr><td>A</td><td>2</td><td>hCoV-19/USA/WA13-UW9/2020|EPI_ISL_413601|2020-03-0</td><td>····················</td><td>····················</td><td>····················</td><td>····················</td><td>····················</td><td>····················</td><td></td><td></td><td></td></tr>
<tr><td>A</td><td>3</td><td>hCoV-19/USA/WA-S47/2020|EPI_ISL_417100|2020-02-29</td><td>····················</td><td>····················</td><td>····················</td><td>····················</td><td>····················</td><td>····················</td><td></td><td></td><td></td></tr>
<tr><td>A</td><td>4</td><td>hCoV-19/USA/WA-S48/2020|EPI_ISL_417101|2020-02-29</td><td>····················</td><td>····················</td><td>····················</td><td>····················</td><td>····················</td><td>····················</td><td></td><td></td><td></td></tr>
<tr><td>A</td><td>5</td><td>hCoV-19/USA/WA-S49/2020|EPI_ISL_417102|2020-03-04</td><td>····················</td><td>····················</td><td>····················</td><td>····················</td><td>····················</td><td>····················</td><td></td><td></td><td></td></tr>
<tr><td>A</td><td>6</td><td>hCoV-19/USA/WA-S77/2020|EPI_ISL_417130|2020-03-05</td><td>····················</td><td>····················</td><td>····················</td><td>····················</td><td>····················</td><td>····················</td><td></td><td></td><td></td></tr>
<tr><td>A</td><td>7</td><td>hCoV-19/USA/WA-S79/2020|EPI_ISL_417132|2020-03-05</td><td>····················</td><td>····················</td><td>····················</td><td>····················</td><td>····················</td><td>····················</td><td></td><td></td><td></td></tr>
<tr><td>A</td><td>8</td><td>hCoV-19/USA/WA-S80/2020|EPI_ISL_417133|2020-03-05</td><td>····················</td><td>····················</td><td>····················</td><td>····················</td><td>····················</td><td>····················</td><td></td><td></td><td></td></tr>
<tr><td>A</td><td>9</td><td>hCoV-19/USA/WA-S81/2020|EPI_ISL_417134|2020-02-26</td><td>····················</td><td>····················</td><td>····················</td><td>····················</td><td>····················</td><td>····················</td><td></td><td></td><td></td></tr>
<tr><td>A</td><td>10</td><td>hCoV-19/USA/WA-S82/2020|EPI_ISL_417135|2020-02-22</td><td>····················</td><td>····················</td><td>····················</td><td>····················</td><td>····················</td><td>····················</td><td></td><td></td><td></td></tr>
<tr><td>A</td><td>11</td><td>hCoV-19/USA/WA-S83/2020|EPI_ISL_417136|2020-03-05</td><td>····················</td><td>····················</td><td>····················</td><td>····················</td><td>····················</td><td>····················</td><td></td><td></td><td></td></tr>
<tr><td>A</td><td>12</td><td>hCoV-19/USA/WA-S84/2020|EPI_ISL_417137|2020-02-21</td><td>····················</td><td>····················</td><td>····················</td><td>····················</td><td>····················</td><td>····················</td><td></td><td></td><td></td></tr>
<tr><td>A</td><td>13</td><td>hCoV-19/USA/WA-S86/2020|EPI_ISL_417139|2020-03-01</td><td>····················</td><td>····················</td><td>····················</td><td>····················</td><td>····················</td><td>····················</td><td></td><td></td><td></td></tr>
<tr><td>A</td><td>14</td><td>hCoV-19/USA/WA-S87/2020|EPI_ISL_417140|2020-03-01</td><td>····················</td><td>····················</td><td>····················</td><td>····················</td><td>····················</td><td>····················</td><td></td><td></td><td></td></tr>
<tr><td>A</td><td>15</td><td>hCoV-19/USA/WA-S88/2020|EPI_ISL_417141|2020-03-01</td><td>····················</td><td>····················</td><td>····················</td><td>····················</td><td>····················</td><td>····················</td><td></td><td></td><td></td></tr>
<tr><td>A</td><td>16</td><td>hCoV-19/USA/WA-S89/2020|EPI_ISL_417142|2020-02-29</td><td>····················</td><td>····················</td><td>····················</td><td>····················</td><td>····················</td><td>····················</td><td></td><td></td><td></td></tr>
<tr><td>A</td><td>17</td><td>hCoV-19/USA/WA-S90/2020|EPI_ISL_417143|2020-02-29</td><td>····················</td><td>····················</td><td>····················</td><td>····················</td><td>····················</td><td>····················</td><td></td><td></td><td></td></tr>
<tr><td>A</td><td>18</td><td>hCoV-19/USA/WA-S91/2020|EPI_ISL_417144|2020-03-02</td><td>····················</td><td>····················</td><td>····················</td><td>····················</td><td>····················</td><td>····················</td><td></td><td></td><td></td></tr>
<tr><td>A</td><td>19</td><td>hCoV-19/USA/WA-S92/2020|EPI_ISL_417145|2020-02-29</td><td>····················</td><td>····················</td><td>····················</td><td>····················</td><td>····················</td><td>····················</td><td></td><td></td><td></td></tr>
<tr><td>A</td><td>20</td><td>hCoV-19/USA/WA-S93/2020|EPI_ISL_417146|2020-02-29</td><td>····················</td><td>····················</td><td>····················</td><td>····················</td><td>····················</td><td>····················</td><td></td><td></td><td></td></tr>
<tr><td>A</td><td>21</td><td>hCoV-19/USA/WA-S94/2020|EPI_ISL_417147|2020-02-28</td><td>····················</td><td>····················</td><td>····················</td><td>····················</td><td>····················</td><td>····················</td><td></td><td></td><td></td></tr>
<tr><td>A</td><td>22</td><td>hCoV-19/USA/WA-S95/2020|EPI_ISL_417148|2020-02-28</td><td>····················</td><td>····················</td><td>····················</td><td>····················</td><td>····················</td><td>····················</td><td></td><td></td><td></td></tr>
<tr><td>A</td><td>23</td><td>hCoV-19/USA/WA-S96/2020|EPI_ISL_417149|2020-02-28</td><td>····················</td><td>····················</td><td>····················</td><td>····················</td><td>····················</td><td>····················</td><td></td><td></td><td></td></tr>
<tr><td>A</td><td>24</td><td>hCoV-19/USA/WA-S97/2020|EPI_ISL_417150|2020-02-28</td><td>····················</td><td>····················</td><td>····················</td><td>····················</td><td>····················</td><td>····················</td><td></td><td></td><td></td></tr>
<tr><td>A</td><td>25</td><td>hCoV-19/USA/WA-S98/2020|EPI_ISL_417151|2020-02-29</td><td>····················</td><td>····················</td><td>····················</td><td>····················</td><td>····················</td><td>····················</td><td></td><td></td><td></td></tr>
<tr><td>A</td><td>26</td><td>hCoV-19/USA/WA-S99/2020|EPI_ISL_417152|2020-02-28</td><td>····················</td><td>····················</td><td>····················</td><td>····················</td><td>····················</td><td>····················</td><td></td><td></td><td></td></tr>
<tr><td>A</td><td>27</td><td>hCoV-19/USA/WA-S100/2020|EPI_ISL_417153|2020-02-29</td><td>····················</td><td>····················</td><td>····················</td><td>····················</td><td>····················</td><td>····················</td><td></td><td></td><td></td></tr>
<tr><td>A</td><td>28</td><td>hCoV-19/USA/WA-S101/2020|EPI_ISL_417154|2020-02-28</td><td>····················</td><td>····················</td><td>····················</td><td>····················</td><td>····················</td><td>····················</td><td></td><td></td><td></td></tr>
<tr><td>A</td><td>29</td><td>hCoV-19/USA/WA-S102/2020|EPI_ISL_417155|2020-02-28</td><td>····················</td><td>····················</td><td>····················</td><td>····················</td><td>····················</td><td>····················</td><td></td><td></td><td></td></tr>
<tr><td>A</td><td>30</td><td>hCoV-19/USA/WA-S103/2020|EPI_ISL_417156|2020-02-28</td><td>····················</td><td>····················</td><td>····················</td><td>····················</td><td>····················</td><td>····················</td><td></td><td></td><td></td></tr>
<tr><td>A</td><td>31</td><td>hCoV-19/USA/WA-S104/2020|EPI_ISL_417157|2020-03-05</td><td>····················</td><td>····················</td><td>····················</td><td>····················</td><td>····················</td><td>····················</td><td></td><td></td><td></td></tr>
<tr><td>A</td><td>32</td><td>hCoV-19/USA/WA-S105/2020|EPI_ISL_417158|2020-02-28</td><td>····················</td><td>····················</td><td>····················</td><td>····················</td><td>····················</td><td>····················</td><td></td><td></td><td></td></tr>
<tr><td>A</td><td>33</td><td>hCoV-19/USA/WA-S106/2020|EPI_ISL_417159|2020-02-29</td><td>····················</td><td>····················</td><td>····················</td><td>····················</td><td>····················</td><td>····················</td><td></td><td></td><td></td></tr>
<tr><td>A</td><td>34</td><td>hCoV-19/USA/WA-S107/2020|EPI_ISL_417160|2020-02-29</td><td>····················</td><td>····················</td><td>····················</td><td>····················</td><td>····················</td><td>····················</td><td></td><td></td><td></td></tr>
<tr><td>A</td><td>35</td><td>hCoV-19/USA/WA-S108/2020|EPI_ISL_417161|2020-02-29</td><td>····················</td><td>····················</td><td>····················</td><td>····················</td><td>····················</td><td>····················</td><td></td><td></td><td></td></tr>
<tr><td>A</td><td>36</td><td>hCoV-19/USA/WA-S109/2020|EPI_ISL_417162|2020-03-01</td><td>····················</td><td>····················</td><td>····················</td><td>····················</td><td>····················</td><td>····················</td><td></td><td></td><td></td></tr>
<tr><td>A</td><td>37</td><td>hCoV-19/USA/WA-S110/2020|EPI_ISL_417163|2020-03-05</td><td>····················</td><td>····················</td><td>····················</td><td>····················</td><td>····················</td><td>····················</td><td></td><td></td><td></td></tr>
<tr><td>A</td><td>38</td><td>hCoV-19/USA/WA-S111/2020|EPI_ISL_417164|2020-03-07</td><td>····················</td><td>····················</td><td>····················</td><td>····················</td><td>····················</td><td>····················</td><td></td><td></td><td></td></tr>
<tr><td>A</td><td>39</td><td>hCoV-19/USA/WA-S112/2020|EPI_ISL_417165|2020-03-08</td><td>····················</td><td>····················</td><td>····················</td><td>····················</td><td>····················</td><td>····················</td><td></td><td></td><td></td></tr>
<tr><td>A</td><td>40</td><td>hCoV-19/USA/WA-S113/2020|EPI_ISL_417166|2020-02-29</td><td>····················</td><td>····················</td><td>····················</td><td>····················</td><td>····················</td><td>····················</td><td></td><td></td><td></td></tr>
<tr><td>A</td><td>41</td><td>hCoV-19/USA/WA-S114/2020|EPI_ISL_417167|2020-03-05</td><td>····················</td><td>····················</td><td>····················</td><td>····················</td><td>····················</td><td>····················</td><td></td><td></td><td></td></tr>
<tr><td>A</td><td>42</td><td>hCoV-19/USA/WA-S115/2020|EPI_ISL_417168|2020-02-29</td><td>····················</td><td>····················</td><td>····················</td><td>····················</td><td>····················</td><td>····················</td><td></td><td></td><td></td></tr>
<tr><td>A</td><td>43</td><td>hCoV-19/USA/WA-S116/2020|EPI_ISL_417169|2020-03-02</td><td>····················</td><td>····················</td><td>····················</td><td>····················</td><td>····················</td><td>····················</td><td></td><td></td><td></td></tr>
<tr><td>A</td><td>44</td><td>hCoV-19/USA/WA-S117/2020|EPI_ISL_417170|2020-03-02</td><td>····················</td><td>····················</td><td>····················</td><td>····················</td><td>····················</td><td>····················</td><td></td><td></td><td></td></tr>
<tr><td>A</td><td>45</td><td>hCoV-19/USA/WA-S118/2020|EPI_ISL_417171|2020-03-01</td><td>····················</td><td>····················</td><td>····················</td><td>····················</td><td>····················</td><td>····················</td><td></td><td></td><td></td></tr>
<tr><td>A</td><td>46</td><td>hCoV-19/USA/WA-S119/2020|EPI_ISL_417172|2020-02-29</td><td>····················</td><td>····················</td><td>····················</td><td>····················</td><td>····················</td><td>····················</td><td></td><td></td><td></td></tr>
<tr><td>A</td><td>47</td><td>hCoV-19/USA/WA-S122/2020|EPI_ISL_417175|2020-03-02</td><td>····················</td><td>····················</td><td>····················</td><td>····················</td><td>····················</td><td>····················</td><td></td><td></td><td></td></tr>
<tr><td>A</td><td>48</td><td>hCoV-19/Hong Kong/HKPU1_2101/2020|EPI_ISL_417176|2</td><td>····················</td><td>····················</td><td>····················</td><td>····················</td><td>····················</td><td>····················</td><td></td><td></td><td></td></tr>
<tr><td>A</td><td>49</td><td>hCoV-19/Hong Kong/HKPU6_2101/2020|EPI_ISL_417178|2</td><td>····················</td><td>····················</td><td>····················</td><td>····················</td><td>····················</td><td>····················</td><td></td><td></td><td></td></tr>
<tr><td>A</td><td>50</td><td>hCoV-19/Hong Kong/HKPU19_0402/2020|EPI_ISL_417181|</td><td>····················</td><td>····················</td><td>····················</td><td>····················</td><td>····················</td><td>····················</td><td></td><td></td><td></td></tr>
<tr><td>A</td><td>51</td><td>hCoV-19/Hong Kong/HKPU28_3001/2020|EPI_ISL_417185|</td><td>····················</td><td>····················</td><td>····················</td><td>····················</td><td>····················</td><td>····················</td><td></td><td></td><td></td></tr>
<tr><td>A</td><td>52</td><td>hCoV-19/South Africa/R03006/2020|EPI_ISL_417186|20</td><td>····················</td><td>····················</td><td>····················</td><td>····················</td><td>····················</td><td>····················</td><td></td><td></td><td></td></tr>
<tr><td>A</td><td>53</td><td>hCoV-19/Hong Kong/HKPU29_0102/2020|EPI_ISL_417187|</td><td>····················</td><td>····················</td><td>····················</td><td>····················</td><td>····················</td><td>····················</td><td></td><td></td><td></td></tr>
<tr><td>A</td><td>54</td><td>hCoV-19/Hong Kong/HKPU30_2901/2020|EPI_ISL_417188|</td><td>····················</td><td>····················</td><td>····················</td><td>····················</td><td>····················</td><td>····················</td><td></td><td></td><td></td></tr>
<tr><td>A</td><td>55</td><td>hCoV-19/USA/MN5-MDH5/2020|EPI_ISL_417191|2020-03-1</td><td>····················</td><td>····················</td><td>····················</td><td>····················</td><td>····················</td><td>····················</td><td></td><td></td><td></td></tr>
<tr><td>A</td><td>56</td><td>hCoV-19/USA/MN25-MDH25/2020|EPI_ISL_417192|2020-03</td><td>····················</td><td>····················</td><td>····················</td><td>····················</td><td>····················</td><td>N···················</td><td></td><td></td><td></td></tr>
<tr><td>A</td><td>57</td><td>hCoV-19/Hong Kong/HKPU32_0402/2020|EPI_ISL_417193|</td><td>····················</td><td>····················</td><td>····················</td><td>····················</td><td>····················</td><td>····················</td><td></td><td></td><td></td></tr>
<tr><td>A</td><td>58</td><td>hCoV-19/USA/MN26-MDH26/2020|EPI_ISL_417194|2020-03</td><td>····················</td><td>····················</td><td>····················</td><td>····················</td><td>····················</td><td>····················</td><td></td><td></td><td></td></tr>
<tr><td>A</td><td>59</td><td>hCoV-19/USA/MN29-MDH29/2020|EPI_ISL_417196|2020-03</td><td>····················</td><td>····················</td><td>····················</td><td>····················</td><td>····················</td><td>····················</td><td></td><td></td><td></td></tr>
<tr><td>A</td><td>60</td><td>hCoV-19/Hong Kong/HKPU34_3001/2020|EPI_ISL_417197|</td><td>····················</td><td>····················</td><td>····················</td><td>····················</td><td>····················</td><td>····················</td><td></td><td></td><td></td></tr>
<tr><td>A</td><td>61</td><td>hCoV-19/Finland/13M83/2020|EPI_ISL_418400|2020-03-</td><td>····················</td><td>····················</td><td>····················</td><td>····················</td><td>····················</td><td>····················</td><td></td><td></td><td></td></tr>
<tr><td>A</td><td>62</td><td>hCoV-19/Finland/14M12/2020|EPI_ISL_418401|2020-03-</td><td>····················</td><td>····················</td><td>····················</td><td>····················</td><td>····················</td><td>····················</td><td></td><td></td><td></td></tr>
<tr><td>A</td><td>63</td><td>hCoV-19/Finland/14M13/2020|EPI_ISL_418402|2020-03-</td><td>····················</td><td>····················</td><td>····················</td><td>····················</td><td>····················</td><td>····················</td><td></td><td></td><td></td></tr>
<tr><td>A</td><td>64</td><td>hCoV-19/Finland/14M14/2020|EPI_ISL_418403|2020-03-</td><td>····················</td><td>····················</td><td>····················</td><td>····················</td><td>····················</td><td>····················</td><td></td><td></td><td></td></tr>
<tr><td>A</td><td>65</td><td>hCoV-19/Finland/14M16/2020|EPI_ISL_418404|2020-03-</td><td>····················</td><td>····················</td><td>····················</td><td>····················</td><td>····················</td><td>····················</td><td></td><td></td><td></td></tr>
<tr><td>A</td><td>66</td><td>hCoV-19/Finland/14M20/2020|EPI_ISL_418405|2020-03-</td><td>····················</td><td>····················</td><td>····················</td><td>····················</td><td>····················</td><td>····················</td><td></td><td></td><td></td></tr>
<tr><td>A</td><td>67</td><td>hCoV-19/Finland/14M26/2020|EPI_ISL_418406|2020-03-</td><td>····················</td><td>····················</td><td>····················</td><td>····················</td><td>····················</td><td>····················</td><td></td><td></td><td></td></tr>
<tr><td>A</td><td>68</td><td>hCoV-19/Finland/14M32/2020|EPI_ISL_418408|2020-03-</td><td>····················</td><td>····················</td><td>····················</td><td>····················</td><td>····················</td><td>····················</td><td></td><td></td><td></td></tr>
</table>

A	69	hCoV-19/Finland/14M74/2020|EPI_ISL_418409|2020-03-	····················	····················	····················	····················	····················	····················
A	70	hCoV-19/Finland/14M77/2020|EPI_ISL_418410|2020-03-	····················	····················	····················	····················	····················	····················
A	71	hCoV-19/Finland/14M82/2020|EPI_ISL_418411|2020-03-	····················	····················	····················	····················	····················	····················
A	72	hCoV-19/France/ARA-094100/2020|EPI_ISL_418412|2020	····················	····················	····················	····················	····················	····················
A	73	hCoV-19/France/ARA-09428/2020|EPI_ISL_418413|2020-	····················	····················	····················	····················	····················	····················
A	74	hCoV-19/France/ARA-09434/2020|EPI_ISL_418414|2020-	····················	····················	····················	····················	····················	····················
A	75	hCoV-19/France/ARA-09588/2020|EPI_ISL_418416|2020-	····················	····················	····················	····················	····················	····················
A	76	hCoV-19/France/ARA-09686/2020|EPI_ISL_418417|2020-	····················	····················	····················	····················	····················	····················
A	77	hCoV-19/France/ARA-10163/2020|EPI_ISL_418418|2020-	····················	····················	····················	····················	····················	····················
A	78	hCoV-19/France/ARA-10165/2020|EPI_ISL_418419|2020-	····················	····················	····················	····················	····················	····················
A	79	hCoV-19/France/ARA-10910/2020|EPI_ISL_418430|2020-	····················	····················	····················	····················	····················	····················
A	81	hCoV-19/France/ARA-11036/2020|EPI_ISL_418432|2020-	····················	····················	····················	····················	····················	····················
A	83	hCoV-19/Spain/CT-ISCIII-201397/2020|EPI_ISL_419707	····················	····················	····················	····················	····················	····················
A	84	hCoV-19/Spain/PV-ISCIII-201602/2020|EPI_ISL_419709	····················	····················	····················	····················	····················	····················
A	85	hCoV-19/USA/VA-DCLS-0016/2020|EPI_ISL_419710|2020-	····················	····················	····················	····················	····················	···A················
A	86	hCoV-19/USA/VA-DCLS-0017/2020|EPI_ISL_419711|2020-	····················	····················	····················	····················	····················	····················
A	87	hCoV-19/USA/VA-DCLS-0020/2020|EPI_ISL_419712|2020-	····················	····················	····················	····················	····················	····················
A	88	hCoV-19/USA/VA-DCLS-0021/2020|EPI_ISL_419713|2020-	····················	····················	····················	····················	····················	····················
A	89	hCoV-19/Australia/VIC108/2020|EPI_ISL_419714|2020-	····················	····················	····················	····················	····················	····················
A	90	hCoV-19/Australia/VIC111/2020|EPI_ISL_419715|2020-	····················	····················	····················	····················	····················	····················
A	91	hCoV-19/Australia/VIC110/2020|EPI_ISL_419716|2020-	····················	····················	····················	····················	····················	····················
A	92	hCoV-19/Australia/VIC115/2020|EPI_ISL_419717|2020-	····················	····················	····················	····················	····················	····················
A	93	hCoV-19/Australia/VIC109/2020|EPI_ISL_419718|2020-	····················	····················	····················	····················	····················	····················
A	94	hCoV-19/Australia/VIC118/2020|EPI_ISL_419719|2020-	····················	····················	····················	····················	····················	····················
A	95	hCoV-19/Australia/VIC107/2020|EPI_ISL_419720|2020-	····················	····················	····················	····················	····················	····················
A	96	hCoV-19/Australia/VIC116/2020|EPI_ISL_419721|2020-	····················	····················	····················	····················	····················	····················
A	97	hCoV-19/Australia/VIC120/2020|EPI_ISL_419722|2020-	····················	····················	····················	····················	····················	····················
A	98	hCoV-19/Australia/VIC121/2020|EPI_ISL_419723|2020-	····················	····················	····················	····················	····················	····················
A	99	hCoV-19/Australia/VIC114/2020|EPI_ISL_419725|2020-	····················	····················	····················	····················	····················	····················
A	100	hCoV-19/Australia/VIC119/2020|EPI_ISL_419726|2020-	····················	····················	····················	····················	····················	····················
A	101	hCoV-19/Australia/VIC117/2020|EPI_ISL_419727|2020-	····················	····················	····················	····················	····················	····················
A	102	hCoV-19/Australia/VIC126/2020|EPI_ISL_419728|2020-	····················	····················	····················	····················	····················	····················
A	103	hCoV-19/Australia/VIC127/2020|EPI_ISL_419729|2020-	····················	····················	····················	····················	····················	····················
A	104	hCoV-19/Australia/VIC128/2020|EPI_ISL_419730|2020-	····················	····················	····················	····················	····················	····················
A	105	hCoV-19/Australia/VIC123/2020|EPI_ISL_419731|2020-	····················	····················	····················	····················	····················	····················
A	106	hCoV-19/Australia/VIC129/2020|EPI_ISL_419732|2020-	····················	····················	····················	····················	····················	····················
A	107	hCoV-19/Australia/VIC13/2020|EPI_ISL_419733|2020-0	····················	····················	····················	····················	····················	····················
A	108	hCoV-19/Australia/VIC17/2020|EPI_ISL_419736|2020-0	····················	····················	····················	····················	····················	····················
A	109	hCoV-19/Australia/VIC18/2020|EPI_ISL_419737|2020-0	····················	····················	····················	····················	····················	····················
A	110	hCoV-19/Australia/VIC24/2020|EPI_ISL_419739|2020-0	····················	····················	····················	····················	····················	····················
A	111	hCoV-19/Australia/VIC21/2020|EPI_ISL_419740|2020-0	····················	····················	····················	····················	····················	····················
A	112	hCoV-19/Australia/VIC20/2020|EPI_ISL_419741|2020-0	····················	····················	····················	····················	····················	····················
A	113	hCoV-19/Australia/VIC22/2020|EPI_ISL_419742|2020-0	····················	····················	····················	····················	····················	····················
A	114	hCoV-19/Australia/VIC23/2020|EPI_ISL_419743|2020-0	····················	····················	····················	····················	····················	····················
A	115	hCoV-19/Australia/VIC25/2020|EPI_ISL_419744|2020-0	····················	····················	····················	····················	····················	····················
A	116	hCoV-19/Australia/VIC26/2020|EPI_ISL_419745|2020-0	····················	····················	····················	····················	····················	····················
A	117	hCoV-19/Australia/VIC27/2020|EPI_ISL_419746|2020-0	····················	····················	····················	····················	····················	····················
A	118	hCoV-19/Australia/VIC28/2020|EPI_ISL_419747|2020-0	····················	····················	····················	····················	····················	····················
A	119	hCoV-19/Australia/VIC29/2020|EPI_ISL_419748|2020-0	····················	····················	····················	····················	····················	····················
A	120	hCoV-19/Australia/VIC30/2020|EPI_ISL_419749|2020-0	····················	····················	····················	····················	····················	····················
A	121	hCoV-19/Australia/VIC41/2020|EPI_ISL_419760|2020-0	····················	····················	····················	····················	····················	····················
A	122	hCoV-19/Australia/VIC42/2020|EPI_ISL_419761|2020-0	····················	····················	····················	····················	····················	····················
A	123	hCoV-19/Australia/VIC48/2020|EPI_ISL_419762|2020-0	····················	····················	····················	····················	····················	····················
A	124	hCoV-19/Australia/VIC43/2020|EPI_ISL_419763|2020-0	····················	····················	····················	····················	····················	····················
A	125	hCoV-19/Australia/VIC49/2020|EPI_ISL_419764|2020-0	····················	····················	····················	····················	····················	····················
A	126	hCoV-19/Australia/VIC52/2020|EPI_ISL_419768|2020-0	····················	····················	····················	····················	····················	····················
A	127	hCoV-19/Australia/VIC78/2020|EPI_ISL_419790|2020-0	····················	····················	····················	····················	····················	····················
A	128	hCoV-19/Australia/VIC79/2020|EPI_ISL_419791|2020-0	····················	····················	····················	····················	····················	····················
A	129	hCoV-19/Australia/VIC80/2020|EPI_ISL_419792|2020-0	····················	····················	····················	····················	····················	····················
A	130	hCoV-19/Australia/VIC81/2020|EPI_ISL_419793|2020-0	····················	····················	····················	····················	····················	····················
A	131	hCoV-19/Australia/VIC82/2020|EPI_ISL_419794|2020-0	····················	····················	····················	····················	····················	····················
A	132	hCoV-19/Australia/VIC85/2020|EPI_ISL_419795|2020-0	····················	····················	····················	····················	····················	····················
A	133	hCoV-19/Australia/VIC86/2020|EPI_ISL_419796|2020-0	····················	····················	····················	····················	····················	····················
A	134	hCoV-19/Australia/VIC87/2020|EPI_ISL_419797|2020-0	····················	····················	····················	····················	····················	····················
A	135	hCoV-19/Australia/VIC88/2020|EPI_ISL_419798|2020-0	····················	····················	····················	····················	····················	····················
A	137	hCoV-19/England/20139053304/2020|EPI_ISL_420707|20	····················	····················	····················	····················	····················	····················
A	138	hCoV-19/England/20139053504/2020|EPI_ISL_420708|20	····················	····················	····················	····················	····················	····················
A	140	hCoV-19/England/20139055304/2020|EPI_ISL_420715|20	····················	····················	····················	····················	····················	····················
A	141	hCoV-19/England/20139056904/2020|EPI_ISL_420716|20	····················	····················	····················	····················	····················	····················
A	143	hCoV-19/England/20139057204/2020|EPI_ISL_420718|20	····················	····················	····················	····················	····················	····················
A	144	hCoV-19/England/20139057404/2020|EPI_ISL_420719|20	····················	····················	····················	····················	····················	····················
A	146	hCoV-19/England/20139057604/2020|EPI_ISL_420721|20	····················	····················	····················	····················	····················	····················
A	147	hCoV-19/England/20139057704/2020|EPI_ISL_420722|20	····················	····················	····················	····················	····················	····················
A	148	hCoV-19/England/20139058104/2020|EPI_ISL_420723|20	····················	····················	····················	····················	····················	····················
A	149	hCoV-19/England/20139058304/2020|EPI_ISL_420724|20	····················	····················	····················	····················	····················	····················
A	150	hCoV-19/England/20139059004/2020|EPI_ISL_420727|20	····················	····················	····················	····················	····················	····················
A	151	hCoV-19/England/20139059504/2020|EPI_ISL_420728|20	····················	····················	····················	····················	····················	····················
A	153	hCoV-19/England/20140038604/2020|EPI_ISL_420750|20	····················	····················	····················	····················	····················	····················

```
A     154 hCoV-19/England/20140038704/2020|EPI_ISL_420751|20   .................... .................... .................... .................... .................... ....................
A     155 hCoV-19/England/20142062604/2020|EPI_ISL_420774|20   .................... .................... .................... .................... .................... ....................
A     157 hCoV-19/USA/AZ_4811/2020|EPI_ISL_420784|2020-03-02   .................... .................... .................... .................... .................... ....................
A     158 hCoV-19/USA/FL_6318/2020|EPI_ISL_420785|2020-03-02   .................... .................... .................... .................... .................... ....................
A     159 hCoV-19/USA/GA_1320/2020|EPI_ISL_420786|2020-03-03   .................... .................... .................... .................... .................... ....................
A     160 hCoV-19/USA/GA_1299/2020|EPI_ISL_420787|2020-03-03   .................... .................... .................... .................... .................... ....................
A     161 hCoV-19/USA/GA_1445/2020|EPI_ISL_420788|2020-03-04   .................... .................... .................... .................... .................... ....................
A     162 hCoV-19/USA/IL_1375/2020|EPI_ISL_420789|2020-03-01   .................... .................... .................... .................... .................... ....................
A     163 hCoV-19/USA/IL_1293/2020|EPI_ISL_420790|2020-03-01   .................... .................... .................... .................... .................... ....................
A     164 hCoV-19/USA/NH_0004/2020|EPI_ISL_420791|2020-02-29   .................... .................... .................... .................... .................... ....................
A     165 hCoV-19/USA/NH_0008/2020|EPI_ISL_420792|2020-03-02   .................... .................... .................... .................... .................... ....................
A     166 hCoV-19/USA/NY_2929/2020|EPI_ISL_420793|2020-03-02   .................... .................... .................... .................... .................... ....................
A     167 hCoV-19/USA/OR_5430/2020|EPI_ISL_420794|2020-03-01   .................... .................... .................... .................... .................... ....................
A     168 hCoV-19/USA/RI_0556/2020|EPI_ISL_420795|2020-03-01   .................... .................... .................... .................... .................... ....................
A     169 hCoV-19/USA/TX_2039/2020|EPI_ISL_420796|2020-02-29   .................... .................... .................... .................... .................... ....................
A     170 hCoV-19/USA/TX_2817/2020|EPI_ISL_420797|2020-03-01   .................... .................... .................... .................... .................... ....................
A     171 hCoV-19/USA/TX_2967/2020|EPI_ISL_420798|2020-03-01   .................... .................... .................... .................... .................... ....................
A     172 hCoV-19/South Korea/BA-ACH_2604/2020|EPI_ISL_42079   .................... .................... .................... .................... .................... ....................
A     175 hCoV-19/Australia/NT17/2020|EPI_ISL_426904|2020      .................... .................... .................... .................... .................... ....................
A     179 hCoV-19/Sweden/20-08142/2020|EPI_ISL_429115|2020-0   .................... .................... .................... .................... .................... ....................
A     180 hCoV-19/Sweden/20-08143/2020|EPI_ISL_429116|2020-0   .................... .................... .................... .................... .................... ....................
A     181 hCoV-19/Sweden/20-08145/2020|EPI_ISL_429117|2020-0   .................... .................... .................... .................... .................... ....................
A     182 hCoV-19/Sweden/20-08146/2020|EPI_ISL_429118|2020-0   .................... .................... .................... .................... .................... ....................
A     183 hCoV-19/Sweden/20-08148/2020|EPI_ISL_429119|2020-0   .................... .................... .................... .................... .................... ....................
A     184 hCoV-19/Sweden/20-08149/2020|EPI_ISL_429120|2020-0   .................... .................... .................... .................... .................... ....................
A     185 hCoV-19/Sweden/20-08151/2020|EPI_ISL_429121|2020-0   .................... .................... .................... .................... .................... ....................
A     186 hCoV-19/Sweden/20-08155/2020|EPI_ISL_429122|2020-0   .................... .................... .................... .................... .................... ....................
A     187 hCoV-19/Sweden/20-08169/2020|EPI_ISL_429123|2020-0   .................... .................... .................... .................... .................... ....................
A     188 hCoV-19/Sweden/20-08170/2020|EPI_ISL_429124|2020-0   .................... .................... .................... .................... .................... ....................
A     189 hCoV-19/Sweden/20-08173/2020|EPI_ISL_429125|2020-0   .................... .................... .................... .................... .................... ....................
A     190 hCoV-19/Sweden/20-08320/2020|EPI_ISL_429126|2020-0   .................... .................... .................... .................... .................... ....................
A     191 hCoV-19/Sweden/20-08332/2020|EPI_ISL_429127|2020-0   .................... .................... .................... .................... .................... ....................
A     192 hCoV-19/Sweden/20-08680/2020|EPI_ISL_429128|2020-0   .................... .................... .................... .................... .................... ....................
A     193 hCoV-19/Sweden/20-08681/2020|EPI_ISL_429129|2020-0   .................... .................... .................... .................... .................... ....................
A     194 hCoV-19/Sweden/20-08683/2020|EPI_ISL_429130|2020-0   .................... .................... .................... .................... .................... ....................
A     195 hCoV-19/Sweden/20-08684/2020|EPI_ISL_429131|2020-0   .................... .................... .................... .................... .................... ....................
A     196 hCoV-19/Sweden/20-08685/2020|EPI_ISL_429132|2020-0   .................... .................... .................... .................... .................... ....................
A     197 hCoV-19/Sweden/20-08690/2020|EPI_ISL_429133|2020-0   .................... .................... .................... .................... .................... ....................
A     198 hCoV-19/Sweden/20-08693/2020|EPI_ISL_429134|2020-0   .................... .................... .................... .................... .................... ....................
A     199 hCoV-19/Sweden/20-08719/2020|EPI_ISL_429135|2020-0   .................... .................... .................... .................... .................... ....................
A     200 hCoV-19/Sweden/20-50048/2020|EPI_ISL_429136|2020-0   .................... .................... .................... .................... .................... ....................
A     201 hCoV-19/Sweden/20-50076/2020|EPI_ISL_429137|2020-0   .................... .................... .................... .................... .................... ....................
A     202 hCoV-19/Sweden/20-50087/2020|EPI_ISL_429138|2020-0   .................... .................... .................... .................... .................... ....................
A     203 hCoV-19/Sweden/20-50123/2020|EPI_ISL_429139|2020-0   .................... .................... .................... .................... .................... ....................
A     204 hCoV-19/Sweden/20-50125/2020|EPI_ISL_429140|2020-0   .................... .................... .................... .................... .................... ....................
A     205 hCoV-19/Sweden/20-50130/2020|EPI_ISL_429141|2020-0   .................... .................... .................... .................... .................... ....................
A     206 hCoV-19/Sweden/20-50131/2020|EPI_ISL_429142|2020-0   .................... .................... .................... .................... .................... ....................
A     207 hCoV-19/Sweden/20-50132/2020|EPI_ISL_429143|2020-0   .................... .................... .................... .................... .................... ....................
A     208 hCoV-19/Sweden/20-50133/2020|EPI_ISL_429144|2020-0   .................... .................... .................... .................... .................... ....................
A     209 hCoV-19/Sweden/20-50170/2020|EPI_ISL_429145|2020-0   .................... .................... .................... .................... .................... ....................
A     210 hCoV-19/Sweden/20-50171/2020|EPI_ISL_429146|2020-0   .................... .................... .................... .................... .................... ....................
A     211 hCoV-19/Sweden/20-50172/2020|EPI_ISL_429147|2020-0   .................... .................... .................... .................... .................... ....................
A     212 hCoV-19/Sweden/20-50173/2020|EPI_ISL_429148|2020-0   .................... .................... .................... .................... .................... ....................
A     213 hCoV-19/Sweden/20-50175/2020|EPI_ISL_429150|2020-0   .................... .................... .................... .................... .................... ....................
A     214 hCoV-19/Sweden/20-50176/2020|EPI_ISL_429151|2020-0   .................... .................... .................... .................... .................... ....................
A     215 hCoV-19/Sweden/20-50179/2020|EPI_ISL_429152|2020-0   .................... .................... .................... .................... .................... ....................
A     216 hCoV-19/Sweden/20-50180/2020|EPI_ISL_429153|2020-0   .................... .................... .................... .................... .................... ....................
A     217 hCoV-19/Sweden/20-50181/2020|EPI_ISL_429154|2020-0   .................... .................... .................... .................... .................... ....................
A     218 hCoV-19/Sweden/20-50230/2020|EPI_ISL_429155|2020-0   .................... .................... .................... .................... .................... ....................
A     219 hCoV-19/Sweden/20-50232/2020|EPI_ISL_429156|2020-0   .................... .................... .................... .................... .................... ....................
A     220 hCoV-19/Sweden/20-50234/2020|EPI_ISL_429157|2020-0   .................... .................... .................... .................... .................... ....................
A     221 hCoV-19/Sweden/20-50235/2020|EPI_ISL_429158|2020-0   .................... .................... .................... .................... .................... ....................
A     222 hCoV-19/Sweden/20-50312/2020|EPI_ISL_429160|2020-0   .................... .................... .................... .................... .................... ....................
A     223 hCoV-19/Sweden/20-50315/2020|EPI_ISL_429161|2020-0   .................... .................... .................... .................... .................... ....................
A     224 hCoV-19/Sweden/20-50357/2020|EPI_ISL_429162|2020-0   .................... .................... .................... .................... .................... ....................
A     225 hCoV-19/Sweden/20-50361/2020|EPI_ISL_429163|2020-0   .................... .................... .................... .................... .................... ....................
A     226 hCoV-19/Thailand/Bangkok-0025/2020|EPI_ISL_429164|   .................... .................... .................... .................... .................... ....................
A     227 hCoV-19/Thailand/Bangkok-0026/2020|EPI_ISL_429165|   .................... .................... .................... .................... .................... ....................
A     228 hCoV-19/Thailand/Bangkok-0028/2020|EPI_ISL_429166|   .................... .................... .................... .................... .................... ....................
A     229 hCoV-19/Thailand/Bangkok-0029/2020|EPI_ISL_429167|   .................... .................... .................... .................... .................... ....................
A     230 hCoV-19/Thailand/Bangkok-0033/2020|EPI_ISL_429169|   .................... .................... .................... .................... .................... ....................
A     231 hCoV-19/Thailand/Bangkok-0034/2020|EPI_ISL_429170|   .................... .................... .................... .................... .................... ....................
A     232 hCoV-19/Thailand/Bangkok-0035/2020|EPI_ISL_429171|   .................... .................... .................... .................... .................... ....................
A     233 hCoV-19/Thailand/Bangkok-0036/2020|EPI_ISL_429172|   .................... .................... .................... .................... .................... ....................
A     234 hCoV-19/Thailand/Bangkok-0037/2020|EPI_ISL_429173|   .................... .................... .................... .................... .................... ....................
A     235 hCoV-19/Thailand/Bangkok-0038/2020|EPI_ISL_429174|   .................... .................... .................... .................... .................... ....................
A     236 hCoV-19/Thailand/Bangkok-0039/2020|EPI_ISL_429175|   .................... .................... .................... .................... .................... ....................
A     237 hCoV-19/Thailand/Bangkok-0041/2020|EPI_ISL_429177|   .................... .................... .................... .................... .................... ....................
```

A	238	hCoV-19/Thailand/Bangkok-0042/2020|EPI_ISL_429178|	....	....	....	....	....	....
A	239	hCoV-19/Thailand/Bangkok-0043/2020|EPI_ISL_429179|	....	....	....	....	....	....
A	241	hCoV-19/Thailand/Bangkok-0046/2020|EPI_ISL_429182|	....	....	....	....	....	....
A	242	hCoV-19/Thailand/Bangkok-0047/2020|EPI_ISL_429183|	....	....	....	....	....	....
A	243	hCoV-19/Thailand/Bangkok-0048/2020|EPI_ISL_429184|	....	....	....	....	....	....
A	244	hCoV-19/Russia/StPetersburg-RII6027S/2020|EPI_ISL_	....	....	....	....	....	....
A	245	hCoV-19/Russia/StPetersburg-RII6031S/2020|EPI_ISL_	....	....	....	....	....	....
A	246	hCoV-19/Russia/StPetersburg-RII6039S/2020|EPI_ISL_	....	....	....	....	....	....
A	247	hCoV-19/Russia/StPetersburg-RII6043S/2020|EPI_ISL_	....	....	....	....	....	....
A	248	hCoV-19/Russia/StPetersburg-RII6053S/2020|EPI_ISL_	....	....	....	....	....	....
A	249	hCoV-19/Russia/StPetersburg-RII6055S/2020|EPI_ISL_	....	....	....	....	....	....
A	250	hCoV-19/Russia/StPetersburg-RII6056S/2020|EPI_ISL_	....	....	....	....	....	....
A	251	hCoV-19/Russia/StPetersburg-RII6060S/2020|EPI_ISL_	....	....	....	....	....	....
A	252	hCoV-19/Russia/StPetersburg-RII6061S/2020|EPI_ISL_	....	....	....	....	....	....
A	253	hCoV-19/Russia/StPetersburg-RII6063S/2020|EPI_ISL_	....	....	....	....	....	....
A	254	hCoV-19/Russia/StPetersburg-RII6065S/2020|EPI_ISL_	....	....	....	....	....	....
A	255	hCoV-19/Russia/StPetersburg-RII6086S/2020|EPI_ISL_	....	....	....	....	....	....
A	256	hCoV-19/Russia/Ulan-Ude-RII4562V/2020|EPI_ISL_4301	....	....	....	....	....	....
A	257	hCoV-19/Netherlands/Eindhoven_1363782/2020|EPI_ISL	....	....	....	....	....	....
A	258	hCoV-19/Netherlands/Haarlem_1363688/2020|EPI_ISL_4	....	....	....	....	....	....
A	259	hCoV-19/Netherlands/Hardinxveld_Giessendam_1364806	....	....	....	....	....	....
A	260	hCoV-19/Netherlands/Helmond_1363548/2020|EPI_ISL_4	....	....	....	....	....	....
A	261	hCoV-19/Netherlands/Houten_1363498/2020|EPI_ISL_41	....	....	....	....	....	....
A	262	hCoV-19/Netherlands/Naarden_1364774/2020|EPI_ISL_4	....	....	....	....	....	....
A	263	hCoV-19/Netherlands/Nootdorp_1364222/2020|EPI_ISL_	....	....	....	....	....	....
A	264	hCoV-19/Netherlands/Oisterwijk_1364072/2020|EPI_IS	....	....	....	....	....	....
A	265	hCoV-19/Netherlands/Rotterdam_1363790/2020|EPI_ISL	....	....	....	....	....	....
A	266	hCoV-19/Netherlands/Rotterdam_1364040/2020|EPI_ISL	....	....	....	....	....	....
A	267	hCoV-19/Netherlands/Rotterdam_1364740/2020|EPI_ISL	....	....	....	....	....	....
A	268	hCoV-19/Netherlands/Tilburg_1364286/2020|EPI_ISL_4	....	....	....	....	....	....
A	269	hCoV-19/Netherlands/Utrecht_1363564/2020|EPI_ISL_4	....	....	....	....	....	....
A	270	hCoV-19/Netherlands/Utrecht_1363628/2020|EPI_ISL_4	....	....	....	....	....	....
A	271	hCoV-19/Netherlands/Utrecht_1364066/2020|EPI_ISL_4	....	....	....	....	....	....
A	272	hCoV-19/Netherlands/Zeewolde_1365080/2020|EPI_ISL_	....	....	....	....	....	....
A	273	hCoV-19/Taiwan/NTU03/2020|EPI_ISL_413592|2020-03-0	....	....	....	....	....	....
A	274	hCoV-19/Luxembourg/Lux1/2020|EPI_ISL_413593|2020-0	....	....	....	....	....	....
A	275	hCoV-19/Australia/NSW08/2020|EPI_ISL_413594|2020-0	....	....	....	....	....	....
A	276	hCoV-19/Australia/NSW09/2020|EPI_ISL_413595|2020-0	....	....	....	....	....	....
A	277	hCoV-19/Australia/NSW10/2020|EPI_ISL_413596|2020-0	....	....	....	....	....	....
A	278	hCoV-19/Australia/NSW11/2020|EPI_ISL_413597|2020-0	....	....	....	....	....	....
A	279	hCoV-19/Australia/NSW12/2020|EPI_ISL_413598|2020-0	....	....	....	....	....	....
A	280	hCoV-19/Australia/NSW13/2020|EPI_ISL_413599|2020-0	....	....	....	....	....	....
A	281	hCoV-19/USA/WA-S50/2020|EPI_ISL_417103|2020-03-05	....	....	....	....	....	....
A	282	hCoV-19/USA/WA-S51/2020|EPI_ISL_417104|2020-03-03	....	....	....	....	....	....
A	283	hCoV-19/USA/WA-S52/2020|EPI_ISL_417105|2020-03-03	....	....	....	....	....	....
A	284	hCoV-19/USA/WA-S53/2020|EPI_ISL_417106|2020-03-03	....	....	....	....	....	....
A	285	hCoV-19/USA/WA-S54/2020|EPI_ISL_417107|2020-03-05	....	....	....	....	....	....
A	286	hCoV-19/USA/WA-S55/2020|EPI_ISL_417108|2020-02-29	....	....	....	....	....	....
A	287	hCoV-19/USA/WA-S57/2020|EPI_ISL_417110|2020-03-03	....	....	....	....	....	....
A	288	hCoV-19/USA/WA-S58/2020|EPI_ISL_417111|2020-03-05	....	....	....	....	....	....
A	289	hCoV-19/USA/WA-S59/2020|EPI_ISL_417112|2020-03-02	....	....	....	....	....	....
A	290	hCoV-19/USA/WA-S61/2020|EPI_ISL_417114|2020-03-05	....	....	....	....	....	....
A	291	hCoV-19/USA/WA-S62/2020|EPI_ISL_417115|2020-03-03	....	....	....	....	....	....
A	292	hCoV-19/USA/WA-S63/2020|EPI_ISL_417116|2020-03-04	....	....	....	....	....	....
A	293	hCoV-19/USA/WA-S64/2020|EPI_ISL_417117|2020-03-03	....	....	....	....	....	....
A	294	hCoV-19/USA/WA-S65/2020|EPI_ISL_417118|2020-03-03	....	....	....	....	....	....
A	295	hCoV-19/USA/WA-S66/2020|EPI_ISL_417119|2020-03-06	....	....	....	....	....	....
A	296	hCoV-19/USA/WA-S67/2020|EPI_ISL_417120|2020-03-04	....	....	....	....	....	....
A	297	hCoV-19/USA/WA-S68/2020|EPI_ISL_417121|2020-03-04	....	....	....	....	....	....
A	298	hCoV-19/USA/WA-S69/2020|EPI_ISL_417122|2020-03-05	....	....	....	....	....	....
A	299	hCoV-19/USA/WA-S70/2020|EPI_ISL_417123|2020-03-05	....	....	....	....	....	....
A	300	hCoV-19/USA/WA-S71/2020|EPI_ISL_417124|2020-03-05	....	....	....	....	....	....
A	301	hCoV-19/USA/WA-S72/2020|EPI_ISL_417125|2020-03-06	....	....	....	....	....	....
A	302	hCoV-19/USA/WA-S73/2020|EPI_ISL_417126|2020-03-05	....	....	....	....	....	....
A	303	hCoV-19/USA/WA-S74/2020|EPI_ISL_417127|2020-03-05	....	....	....	....	....	....
A	304	hCoV-19/USA/WA-S75/2020|EPI_ISL_417128|2020-03-05	....	....	....	....	....	....
A	305	hCoV-19/USA/WA-S76/2020|EPI_ISL_417129|2020-03-05	....	....	....	....	....	....
A	306	hCoV-19/France/ARA-10170/2020|EPI_ISL_418420|2020-	....	....	....	....	....	....
A	307	hCoV-19/France/ARA-10172/2020|EPI_ISL_418421|2020-	....	....	....	....	....	....
A	308	hCoV-19/France/ARA-10184/2020|EPI_ISL_418422|2020-	....	....	....	....	....	....
A	309	hCoV-19/France/ARA-10188/2020|EPI_ISL_418423|2020-	....	....	....	....	....	....
A	310	hCoV-19/France/ARA-10189/2020|EPI_ISL_418424|2020-	....	....	....	....	....	....
A	311	hCoV-19/France/ARA-10192/2020|EPI_ISL_418425|2020-	....	....	....	....	....	....
A	312	hCoV-19/France/ARA-10251/2020|EPI_ISL_418426|2020-	....	....	....	....	....	....
A	313	hCoV-19/France/ARA-10257/2020|EPI_ISL_418427|2020-	....	....	....	....	....	....
A	314	hCoV-19/France/ARA-10282/2020|EPI_ISL_418428|2020-	....	....	....	....	....	....
A	315	hCoV-19/France/ARA-10876/2020|EPI_ISL_418429|2020-	....	....	....	....	....	....
A	318	hCoV-19/Hangzhou/HZ48/2020|EPI_ISL_418441|2020-01-	....	....	....	....	....	....

```
A	319 hCoV-19/Hangzhou/HZ49/2020|EPI_ISL_418442|2020-01-	···················· ···················· ···················· ···················· ···················· ····················
A	320 hCoV-19/USA/NY-NYUMC39/2020|EPI_ISL_419700|2020-03	···················· ···················· ···················· ···················· ···················· ····················
A	321 hCoV-19/USA/NY-NYUMC41/2020|EPI_ISL_419702|2020-03	···················· ···················· ···················· ···················· ···················· ····················
A	322 hCoV-19/USA/NY-NYUMC43/2020|EPI_ISL_419704|2020-03	···················· ···················· ···················· ···················· ···················· ····················
A	323 hCoV-19/USA/NY-NYUMC44/2020|EPI_ISL_419705|2020-03	···················· ···················· ···················· ···················· ···················· ····················
A	324 hCoV-19/USA/VA-DCLS-0012/2020|EPI_ISL_419706|2020-	···················· ···················· ···················· ···················· ···················· ····················
A	325 hCoV-19/Australia/VIC31/2020|EPI_ISL_419750|2020-0	···················· ···················· ···················· ···················· ···················· ····················
A	326 hCoV-19/Australia/VIC32/2020|EPI_ISL_419751|2020-0	···················· ···················· ···················· ···················· ···················· ····················
A	327 hCoV-19/Australia/VIC33/2020|EPI_ISL_419752|2020-0	···················· ···················· ···················· ···················· ···················· ····················
A	328 hCoV-19/Australia/VIC34/2020|EPI_ISL_419753|2020-0	···················· ···················· ···················· ···················· ···················· ····················
A	329 hCoV-19/Australia/VIC37/2020|EPI_ISL_419754|2020-0	···················· ···················· ···················· ···················· ···················· ····················
A	330 hCoV-19/Australia/VIC35/2020|EPI_ISL_419755|2020-0	···················· ···················· ···················· ···················· ···················· ····················
A	331 hCoV-19/Australia/VIC38/2020|EPI_ISL_419756|2020-0	···················· ···················· ···················· ···················· ···················· ····················
A	332 hCoV-19/Australia/VIC39/2020|EPI_ISL_419757|2020-0	···················· ···················· ···················· ···················· ···················· ····················
A	333 hCoV-19/Australia/VIC40/2020|EPI_ISL_419758|2020-0	···················· ···················· ···················· ···················· ···················· ····················
A	334 hCoV-19/Australia/VIC47/2020|EPI_ISL_419759|2020-0	···················· ···················· ···················· ···················· ···················· ····················
A	335 hCoV-19/Australia/VIC36/2020|EPI_ISL_419765|2020-0	···················· ···················· ···················· ···················· ···················· ····················
A	336 hCoV-19/Australia/VIC50/2020|EPI_ISL_419766|2020-0	···················· ···················· ···················· ···················· ···················· ····················
A	337 hCoV-19/Australia/VIC44/2020|EPI_ISL_419767|2020-0	···················· ···················· ···················· ···················· ···················· ····················
A	338 hCoV-19/Australia/VIC53/2020|EPI_ISL_419769|2020-0	···················· ···················· ···················· ···················· ···················· ····················
A	339 hCoV-19/Australia/VIC54/2020|EPI_ISL_419770|2020-0	···················· ···················· ···················· ···················· ···················· ····················
A	340 hCoV-19/Australia/VIC59/2020|EPI_ISL_419771|2020-0	···················· ···················· ···················· ···················· ···················· ····················
A	341 hCoV-19/Australia/VIC60/2020|EPI_ISL_419772|2020-0	···················· ···················· ···················· ···················· ···················· ····················
A	342 hCoV-19/Australia/VIC61/2020|EPI_ISL_419773|2020-0	···················· ···················· ···················· ···················· ···················· ····················
A	343 hCoV-19/Australia/VIC62/2020|EPI_ISL_419774|2020-0	···················· ···················· ···················· ···················· ···················· ····················
A	344 hCoV-19/Australia/VIC63/2020|EPI_ISL_419775|2020-0	···················· ···················· ···················· ···················· ···················· ····················
A	345 hCoV-19/Australia/VIC55/2020|EPI_ISL_419776|2020-0	···················· ···················· ···················· ···················· ···················· ····················
A	346 hCoV-19/Australia/VIC56/2020|EPI_ISL_419777|2020-0	···················· ···················· ···················· ···················· ···················· ····················
A	347 hCoV-19/Australia/VIC64/2020|EPI_ISL_419778|2020-0	···················· ···················· ···················· ···················· ···················· ····················
A	348 hCoV-19/Australia/VIC57/2020|EPI_ISL_419779|2020-0	···················· ···················· ···················· ···················· ···················· ····················
A	349 hCoV-19/Australia/VIC66/2020|EPI_ISL_419780|2020-0	···················· ···················· ···················· ···················· ···················· ····················
A	350 hCoV-19/Australia/VIC67/2020|EPI_ISL_419781|2020-0	···················· ···················· ···················· ···················· ···················· ····················
A	351 hCoV-19/Australia/VIC68/2020|EPI_ISL_419782|2020-0	···················· ···················· ···················· ···················· ···················· ····················
A	352 hCoV-19/Australia/VIC69/2020|EPI_ISL_419783|2020-0	···················· ···················· ···················· ···················· ···················· ····················
A	353 hCoV-19/Australia/VIC58/2020|EPI_ISL_419784|2020-0	···················· ···················· ···················· ···················· ···················· ····················
A	354 hCoV-19/Australia/VIC73/2020|EPI_ISL_419785|2020-0	···················· ···················· ···················· ···················· ···················· ····················
A	355 hCoV-19/Australia/VIC70/2020|EPI_ISL_419786|2020-0	···················· ···················· ···················· ···················· ···················· ····················
A	356 hCoV-19/Australia/VIC75/2020|EPI_ISL_419787|2020-0	···················· ···················· ···················· ···················· ···················· ····················
A	357 hCoV-19/Australia/VIC76/2020|EPI_ISL_419788|2020-0	···················· ···················· ···················· ···················· ···················· ····················
A	358 hCoV-19/Australia/VIC77/2020|EPI_ISL_419789|2020-0	···················· ···················· ···················· ···················· ···················· ····················
A	360 hCoV-19/England/20139060004/2020|EPI_ISL_420731|20	···················· ···················· ···················· ···················· ···················· ····················
A	361 hCoV-19/England/20139060404/2020|EPI_ISL_420732|20	···················· ···················· ···················· ···················· ···················· ····················
A	362 hCoV-19/England/20139060504/2020|EPI_ISL_420733|20	···················· ···················· ···················· ···················· ···················· ····················
A	363 hCoV-19/England/20139060804/2020|EPI_ISL_420734|20	···················· ···················· ···················· ···················· ···················· ····················
A	364 hCoV-19/England/20139061104/2020|EPI_ISL_420735|20	···················· ···················· ···················· ···················· ···················· ···N····N·N·········	1
A	365 hCoV-19/England/20139061704/2020|EPI_ISL_420736|20	···················· ···················· ···················· ···················· ···················· ····················
A	367 hCoV-19/England/20139062704/2020|EPI_ISL_420742|20	···················· ···················· ···················· ···················· ···················· ····················
A	368 hCoV-19/England/20139062804/2020|EPI_ISL_420743|20	···················· ···················· ···················· ···················· ···················· ····················
A	370 hCoV-19/England/20139063504/2020|EPI_ISL_420745|20	···················· ···················· ···················· ···················· ···················· ····················
A	371 hCoV-19/England/20139063604/2020|EPI_ISL_420746|20	···················· ···················· ···················· ···················· ···················· ····················
A	372 hCoV-19/England/20139063704/2020|EPI_ISL_420747|20	···················· ···················· ···················· ···················· ···················· ····················
A	373 hCoV-19/England/20139063804/2020|EPI_ISL_420748|20	···················· ···················· ···················· ···················· ···················· ····················
A	374 hCoV-19/England/20140037504/2020|EPI_ISL_420749|20	···················· ···················· ···················· ···················· ···················· ····················
A	375 hCoV-19/England/20140039004/2020|EPI_ISL_420752|20	···················· ···················· ···················· ···················· ···················· ··N·················
A	376 hCoV-19/England/20140039104/2020|EPI_ISL_420753|20	···················· ···················· ···················· ···················· ····C··············· ····················
A	377 hCoV-19/England/20140039304/2020|EPI_ISL_420755|20	···················· ···················· ···················· ···················· ···················· ····················
A	378 hCoV-19/England/20140039404/2020|EPI_ISL_420756|20	···················· ···················· ···················· ···················· ···················· ····················
A	379 hCoV-19/England/20140039604/2020|EPI_ISL_420758|20	···················· ···················· ···················· ···················· ····C··············· ····················
A	380 hCoV-19/England/20140048704/2020|EPI_ISL_420761|20	···················· ···················· ···················· ···················· ···················· ····················
A	381 hCoV-19/England/20142022504/2020|EPI_ISL_420765|20	···················· ···················· ···················· ···················· ···················· ····················
A	382 hCoV-19/England/20142060404/2020|EPI_ISL_420766|20	···················· ···················· ···················· ···················· ···················· ····················
A	383 hCoV-19/England/20142060804/2020|EPI_ISL_420767|20	···················· ···················· ···················· ···················· ···················· ····················
A	384 hCoV-19/England/20142061004/2020|EPI_ISL_420769|20	···················· ···················· ···················· ···················· ···················· ····················
A	385 hCoV-19/England/20142061804/2020|EPI_ISL_420772|20	···················· ···················· ···················· ···················· ···················· ····················
A	387 hCoV-19/USA/WA-UW-1682/2020|EPI_ISL_424218|2020-03	···················· ···················· ···················· ···················· ···················· ····················
A	388 hCoV-19/USA/WA-UW-1695/2020|EPI_ISL_424219|2020-03	···················· ···················· ···················· ···················· ···················· ····················
A	390 hCoV-19/USA/WA-UW-1748/2020|EPI_ISL_424221|2020-03	···················· ···················· ···················· ···················· ···················· ····················
A	391 hCoV-19/USA/WA-UW-1700/2020|EPI_ISL_424222|2020-03	···················· ···················· ···················· ···················· ···················· ····················
A	392 hCoV-19/USA/WA-UW-1744/2020|EPI_ISL_424223|2020-03	···················· ···················· ···················· ···················· ···················· ····················
A	393 hCoV-19/USA/WA-UW-1745/2020|EPI_ISL_424224|2020-03	···················· ···················· ···················· ···················· ···················· ····················
A	394 hCoV-19/USA/WA-UW-1707/2020|EPI_ISL_424225|2020-03	···················· ···················· ···················· ···················· ···················· ····················
A	395 hCoV-19/USA/WA-UW-1705/2020|EPI_ISL_424226|2020-03	···················· ···················· ···················· ···················· ···················· ····················
A	396 hCoV-19/USA/WA-UW-1706/2020|EPI_ISL_424227|2020-03	···················· ···················· ···················· ···················· ···················· ····················
A	397 hCoV-19/USA/WA-UW-1702/2020|EPI_ISL_424228|2020-03	···················· ···················· ···················· ···················· ···················· ····················
A	398 hCoV-19/USA/WA-UW-1724/2020|EPI_ISL_424229|2020-03	···················· ···················· ···················· ···················· ···················· ····················
A	399 hCoV-19/USA/WA-UW-1709/2020|EPI_ISL_424230|2020-03	···················· ···················· ···················· ···················· ···················· ····················
A	400 hCoV-19/USA/WA-UW-1731/2020|EPI_ISL_424231|2020-03	···················· ···················· ···················· ···················· ···················· ····················
A	401 hCoV-19/USA/WA-UW-1708/2020|EPI_ISL_424232|2020-03	···················· ···················· ···················· ···················· ···················· ····················
```

A	402 hCoV-19/USA/WA-UW-1772/2020|EPI_ISL_424233|2020-03	····················	····················	·····················	····················	····················	····················
A	403 hCoV-19/USA/WA-UW-1722/2020|EPI_ISL_424234|2020-03	····················	····················	·····················	····················	····················	····················
A	404 hCoV-19/USA/WA-UW-1733/2020|EPI_ISL_424235|2020-03	····················	····················	·····················	····················	····················	····················
A	405 hCoV-19/USA/WA-UW-1732/2020|EPI_ISL_424236|2020-03	····················	····················	·····················	····················	····················	····················
A	406 hCoV-19/USA/WA-UW-1739/2020|EPI_ISL_424237|2020-03	····················	····················	·····················	····················	····················	····················
A	407 hCoV-19/USA/WA-UW-1741/2020|EPI_ISL_424238|2020-03	····················	····················	·····················	····················	····················	····················
A	408 hCoV-19/USA/WA-UW-1750/2020|EPI_ISL_424239|2020-03	····················	····················	·····················	····················	····················	····················
A	409 hCoV-19/USA/WA-UW-1730/2020|EPI_ISL_424240|2020-03	····················	····················	·····················	····················	····················	····················
A	410 hCoV-19/USA/WA-UW-1735/2020|EPI_ISL_424241|2020-03	····················	····················	·····················	····················	····················	····················
A	411 hCoV-19/USA/WA-UW-1753/2020|EPI_ISL_424242|2020-03	····················	····················	·····················	····················	····················	····················
A	412 hCoV-19/USA/WA-UW-1756/2020|EPI_ISL_424243|2020-03	····················	····················	·····················	····················	····················	····················
A	413 hCoV-19/USA/WA-UW-1770/2020|EPI_ISL_424244|2020-03	····················	····················	·····················	····················	····················	····················
A	414 hCoV-19/USA/WA-UW-1762/2020|EPI_ISL_424245|2020-03	····················	····················	·····················	····················	····················	····················
A	415 hCoV-19/USA/WA-UW-1758/2020|EPI_ISL_424246|2020-03	····················	····················	·····················	····················	····················	····················
A	416 hCoV-19/USA/WA-UW-1760/2020|EPI_ISL_424247|2020-03	····················	····················	·····················	····················	····················	····················
A	417 hCoV-19/USA/UN-UW-1773/2020|EPI_ISL_424248|2020-03	····················	····················	·····················	····················	····················	····················
A	418 hCoV-19/USA/UN-UW-1791/2020|EPI_ISL_424249|2020-03	····················	····················	·····················	····················	····················	····················
A	419 hCoV-19/USA/WA-UW-1785/2020|EPI_ISL_424250|2020-03	····················	····················	·····················	····················	····················	····················
A	420 hCoV-19/USA/WA-UW-1801/2020|EPI_ISL_424251|2020-03	····················	····················	·····················	····················	····················	····················
A	421 hCoV-19/USA/WA-UW-1840/2020|EPI_ISL_424252|2020-03	····················	····················	·····················	····················	····················	····················
A	422 hCoV-19/USA/WA-UW-1792/2020|EPI_ISL_424253|2020-03	····················	····················	·····················	····················	····················	····················
A	423 hCoV-19/USA/WA-UW-1796/2020|EPI_ISL_424254|2020-03	····················	····················	·····················	····················	····················	····················
A	424 hCoV-19/USA/UN-UW-1832/2020|EPI_ISL_424255|2020-03	····················	····················	·····················	····················	····················	····················
A	425 hCoV-19/USA/WA-UW-1774/2020|EPI_ISL_424256|2020-03	····················	····················	·····················	····················	····················	····················
A	426 hCoV-19/USA/WA-UW-1775/2020|EPI_ISL_424257|2020-03	····················	····················	·····················	····················	····················	····················
A	427 hCoV-19/USA/WA-UW-1778/2020|EPI_ISL_424258|2020-03	····················	····················	·····················	····················	····················	····················
A	428 hCoV-19/USA/WA-UW-1797/2020|EPI_ISL_424259|2020-03	····················	····················	·····················	····················	····················	····················
A	429 hCoV-19/USA/WA-UW-1779/2020|EPI_ISL_424260|2020-03	····················	····················	·····················	····················	····················	····················
A	430 hCoV-19/USA/WA-UW-1784/2020|EPI_ISL_424261|2020-03	····················	····················	·····················	····················	····················	····················
A	431 hCoV-19/USA/WA-UW-1782/2020|EPI_ISL_424262|2020-03	····················	····················	·····················	····················	····················	····················
A	432 hCoV-19/USA/WA-UW-1799/2020|EPI_ISL_424263|2020-03	····················	····················	·····················	····················	····················	····················
A	433 hCoV-19/USA/WA-UW-1805/2020|EPI_ISL_424264|2020-03	····················	····················	·····················	····················	····················	····················
A	434 hCoV-19/USA/UN-UW-1818/2020|EPI_ISL_424266|2020-03	····················	····················	·····················	····················	····················	····················
A	435 hCoV-19/USA/UN-UW-1817/2020|EPI_ISL_424267|2020-03	····················	····················	·····················	····················	····················	····················
A	436 hCoV-19/USA/UN-UW-1816/2020|EPI_ISL_424268|2020-03	····················	····················	·····················	····················	····················	····················
A	437 hCoV-19/USA/WA-UW-1828/2020|EPI_ISL_424269|2020-03	····················	····················	·····················	····················	····················	····················
A	438 hCoV-19/USA/WA-UW-1831/2020|EPI_ISL_424270|2020-03	····················	····················	·····················	····················	····················	····················
A	439 hCoV-19/USA/WA-UW-1827/2020|EPI_ISL_424271|2020-03	····················	····················	·····················	····················	····················	····················
A	440 hCoV-19/USA/WA-UW-1826/2020|EPI_ISL_424273|2020-03	····················	····················	·····················	····················	····················	····················
A	441 hCoV-19/USA/WA-UW-1821/2020|EPI_ISL_424275|2020-03	····················	····················	·····················	····················	····················	····················
A	442 hCoV-19/USA/WA-UW-1822/2020|EPI_ISL_424276|2020-03	····················	····················	·····················	····················	····················	····················
A	443 hCoV-19/USA/WA-UW-1866/2020|EPI_ISL_424278|2020-03	····················	····················	·····················	····················	····················	····················
A	444 hCoV-19/USA/WA-UW-1850/2020|EPI_ISL_424279|2020-03	····················	····················	·····················	····················	····················	····················
A	445 hCoV-19/USA/WA-UW-1853/2020|EPI_ISL_424290|2020-03	····················	····················	·····················	····················	····················	····················
A	446 hCoV-19/USA/WA-UW-1854/2020|EPI_ISL_424291|2020-03	····················	····················	·····················	····················	····················	····················
A	447 hCoV-19/USA/WA-UW-1855/2020|EPI_ISL_424292|2020-03	····················	····················	·····················	····················	····················	····················
A	448 hCoV-19/USA/WA-UW-1870/2020|EPI_ISL_424293|2020-03	····················	····················	·····················	····················	····················	····················
A	449 hCoV-19/USA/WA-UW-1872/2020|EPI_ISL_424294|2020-03	····················	····················	·····················	····················	····················	····················
A	450 hCoV-19/USA/WA-UW-1905/2020|EPI_ISL_424295|2020-03	····················	····················	·····················	····················	····················	····················
A	451 hCoV-19/USA/WA-UW-1919/2020|EPI_ISL_424296|2020-03	····················	····················	·····················	····················	····················	····················
A	452 hCoV-19/USA/WA-UW-1913/2020|EPI_ISL_424297|2020-03	····················	····················	·····················	····················	····················	····················
A	453 hCoV-19/USA/WA-UW-1920/2020|EPI_ISL_424298|2020-03	····················	····················	·····················	····················	····················	····················
A	454 hCoV-19/USA/WA-UW-1904/2020|EPI_ISL_424299|2020-03	····················	····················	·····················	····················	····················	····················
A	455 hCoV-19/England/NOTT-10E46F/2020|EPI_ISL_425548|20	····················	····················	·····················	····················	····················	····················
A	456 hCoV-19/England/NOTT-10E47E/2020|EPI_ISL_425549|20	····················	····················	·····················	····················	····················	····················
A	457 hCoV-19/England/NOTT-10E48D/2020|EPI_ISL_425550|20	····················	····················	·····················	····················	····················	····················
A	458 hCoV-19/England/NOTT-10E49C/2020|EPI_ISL_425551|20	····················	····················	·····················	····················	····················	····················
A	459 hCoV-19/England/NOTT-10E4C9/2020|EPI_ISL_425554|20	····················	····················	·····················	····················	····················	····················
A	460 hCoV-19/England/NOTT-10E55D/2020|EPI_ISL_425561|20	····················	····················	·····················	····················	····················	····················
A	461 hCoV-19/England/NOTT-10E57B/2020|EPI_ISL_425562|20	····················	····················	·····················	····················	····················	····················
A	462 hCoV-19/England/NOTT-10E58A/2020|EPI_ISL_425563|20	····················	····················	·····················	····················	····················	····················
A	463 hCoV-19/England/NOTT-10E5A8/2020|EPI_ISL_425565|20	····················	····················	·····················	····················	····················	····················
A	464 hCoV-19/England/NOTT-10E5B7/2020|EPI_ISL_425566|20	····················	····················	·····················	····················	····················	····················
A	465 hCoV-19/England/NOTT-10E5C6/2020|EPI_ISL_425567|20	····················	····················	·····················	····················	····················	····················
A	466 hCoV-19/England/NOTT-10E5D5/2020|EPI_ISL_425568|20	····················	····················	·····················	····················	····················	····················
A	467 hCoV-19/England/NOTT-10E5F3/2020|EPI_ISL_425570|20	····················	····················	·····················	····················	····················	····················
A	468 hCoV-19/England/NOTT-10E61E/2020|EPI_ISL_425571|20	····················	····················	·····················	····················	····················	····················
A	469 hCoV-19/England/NOTT-10E64B/2020|EPI_ISL_425573|20	····················	····················	·····················	····················	····················	····················
A	470 hCoV-19/England/NOTT-10E65A/2020|EPI_ISL_425574|20	····················	····················	·····················	····················	····················	····················
A	471 hCoV-19/England/NOTT-10E669/2020|EPI_ISL_425575|20	····················	····················	·····················	····················	····················	····················
A	472 hCoV-19/England/NOTT-10E678/2020|EPI_ISL_425576|20	····················	····················	·····················	····················	····················	····················
A	473 hCoV-19/England/NOTT-10E6A5/2020|EPI_ISL_425579|20	····················	····················	·····················	····················	····················	····················
A	474 hCoV-19/England/NOTT-10E6C3/2020|EPI_ISL_425581|20	····················	····················	·····················	····················	····················	····················
A	475 hCoV-19/England/NOTT-10E6D2/2020|EPI_ISL_425582|20	····················	····················	·····················	····················	····················	····················
A	476 hCoV-19/England/NOTT-10E70C/2020|EPI_ISL_425583|20	····················	····················	·····················	····················	····················	····················
A	477 hCoV-19/England/NOTT-10E71B/2020|EPI_ISL_425584|20	····················	····················	·····················	····················	····················	····················
A	478 hCoV-19/England/NOTT-10E739/2020|EPI_ISL_425586|20	····················	····················	·····················	····················	····················	····················
A	479 hCoV-19/England/NOTT-10E748/2020|EPI_ISL_425587|20	····················	····················	·····················	····················	····················	····················

A	480 hCoV-19/England/NOTT-10E775/2020|EPI_ISL_425590|20	....	....	....	....	....	....	
A	481 hCoV-19/England/NOTT-10E7A2/2020|EPI_ISL_425593|20	....	....	....	....	....	....	
A	482 hCoV-19/Australia/VIC912/2020|EPI_ISL_426881|2020-	....	....	....	....	....	....	
A	484 hCoV-19/Czech Republic/IAB_1/2020|EPI_ISL_426883|2	....	....	....	....	....	....	
A	485 hCoV-19/Czech Republic/IAB_4/2020|EPI_ISL_426884|2	....	....	....	....	....	....	
A	486 hCoV-19/Czech Republic/IAB_8/2020|EPI_ISL_426885|2	....	....	....	....	....	....	
A	487 hCoV-19/Czech Republic/IAB_9/2020|EPI_ISL_426886|2	....	....	....	....	....	....	
A	488 hCoV-19/Czech Republic/IAB_10/2020|EPI_ISL_426887|	....	....	....	....	....	....	
A	489 hCoV-19/Czech Republic/IAB_12/2020|EPI_ISL_426888|	....	....	....	....	....	....	
A	490 hCoV-19/Czech Republic/IAB_14/2020|EPI_ISL_426889|	....	....	....	....	....	................A...	
A	491 hCoV-19/Czech Republic/IAB_15/2020|EPI_ISL_426890|	....	....	....	....	....	....	
A	492 hCoV-19/Czech Republic/IAB_16/2020|EPI_ISL_426891|	....	....	....	....	....	....	
A	493 hCoV-19/Czech Republic/IAB_17/2020|EPI_ISL_426892|	....	....	....	....	....	....	
A	494 hCoV-19/Czech Republic/IAB_18/2020|EPI_ISL_426893|	....	....	....	....	....	....	
A	495 hCoV-19/Czech Republic/IAB_20/2020|EPI_ISL_426894|	....	....	....	....	....	....	
A	496 hCoV-19/Czech Republic/IAB_21/2020|EPI_ISL_426895|	....	....	....	....	....	....	
A	497 hCoV-19/Czech Republic/IAB_22/2020|EPI_ISL_426896|	....	....	....	....	....	....	
A	498 hCoV-19/Czech Republic/IAB_23/2020|EPI_ISL_426897|	....	....	....	....	....	....	
A	501 hCoV-19/Guangzhou/GZMU0057/2020|EPI_ISL_429100|202	....	....	....	....	....	....	
A	502 hCoV-19/Guangzhou/GZMU0058/2020|EPI_ISL_429101|202	....	....	....	....	....	....	
A	503 hCoV-19/Guangzhou/GZMU0059/2020|EPI_ISL_429102|202	....	....	....	....	....	....	
A	504 hCoV-19/Guangzhou/GZMU0060/2020|EPI_ISL_429103|202	....	....	....	....	....	....	
A	505 hCoV-19/Guangzhou/GZMU0036/2020|EPI_ISL_429104|202	....	....	....	....	....	....	
A	506 hCoV-19/Guangzhou/GZMU0055/2020|EPI_ISL_429105|202	....	....	....	....	....	....	
A	507 hCoV-19/Wuhan/OS52/2020|EPI_ISL_450500|2020-01-10	....	....	....	....	....	....	
A	508 hCoV-19/Wuhan/YB012611/2020|EPI_ISL_450501|2020-01	....	....	....	....	....	....	
A	509 hCoV-19/Wuhan/YB012605/2020|EPI_ISL_450502|2020-01	....	....	....	....	....	....	
A	510 hCoV-19/Wuhan/YB012602/2020|EPI_ISL_450503|2020-01	....	....	....	....	....	....	
A	511 hCoV-19/Wuhan/YB012506/2020|EPI_ISL_450504|2020-01	....	....	....	....	....	....	
A	512 hCoV-19/Brazil/SP02cc/2020|EPI_ISL_450506|2020-03-	....	....	....	....	....	....	
A	513 hCoV-19/South Korea/KUMC03/2020|EPI_ISL_413513|202	....	....	....	....	....	....	
A	514 hCoV-19/South Korea/KUMC04/2020|EPI_ISL_413514|202	....	....	....	....	....	....	
A	515 hCoV-19/South Korea/KUMC05/2020|EPI_ISL_413515|202	....	....	....	....	....	....	
A	516 hCoV-19/South Korea/KUMC06/2020|EPI_ISL_413516|202	....	....	....	....	....	....	
A	517 hCoV-19/Beijing/105/2020|EPI_ISL_413518|2020-01-26	....	....	....	....	....	....	
A	518 hCoV-19/Beijing/231/2020|EPI_ISL_413519|2020-01-28	....	....	....	....	....	....	
A	519 hCoV-19/Beijing/233/2020|EPI_ISL_413520|2020-01-28	....	....	....	....	....	....	
A	520 hCoV-19/Beijing/235/2020|EPI_ISL_413521|2020-01-28	....	....	....	....	....	....	
A	521 hCoV-19/India/MH-1-27/2020|EPI_ISL_413522|2020-01-	....	....	....	....	....	....	
A	522 hCoV-19/India/MH-1-31/2020|EPI_ISL_413523|2020-01-	....	....	....	....	....	....	
A	523 hCoV-19/Wales/PHW1/2020|EPI_ISL_413555|2020-02-27	....	....	....	....	....	....	
A	524 hCoV-19/Wales/PHW2/2020|EPI_ISL_413556|2020-03-04	....	....	....	....	....	....	
A	525 hCoV-19/USA/CA-CDPH-UC1/2020|EPI_ISL_413557|2020-0	....	....	....	....	....	....	
A	526 hCoV-19/USA/CA-CDPH-UC2/2020|EPI_ISL_413558|2020-0	....	....	....	....	....	....	
A	527 hCoV-19/USA/CA-CDPH-UC3/2020|EPI_ISL_413559|2020-0	....	....	....	....	....	....	
A	528 hCoV-19/USA/WA-S3/2020|EPI_ISL_413560|2020-02-28	....	....	....	....	....	....	
A	529 hCoV-19/USA/CA-CDPH-UC4/2020|EPI_ISL_413561|2020-0	....	....	....	....	....	....	
A	530 hCoV-19/USA/WA11-UW7/2020|EPI_ISL_413562|2020-03-0	....	....	....	....	....	....	
A	531 hCoV-19/USA/WA12-UW8/2020|EPI_ISL_413563|2020-03-0	....	....	....	....	....	....	
A	532 hCoV-19/Netherlands/Andel_1365066/2020|EPI_ISL_413	....	....	....	....	....	....	
A	533 hCoV-19/Netherlands/Blaricum_1364780/2020|EPI_ISL_	....	....	....	....	....	....	
A	534 hCoV-19/Netherlands/Dalen_1363624/2020|EPI_ISL_413	....	....	....	....	....	....	
A	535 hCoV-19/USA/WA-S37/2020|EPI_ISL_417090|2020-03-04	....	....	....	....	....	....	
A	536 hCoV-19/USA/WA-S38/2020|EPI_ISL_417091|2020-03-04	....	....	....	....	....	....	
A	537 hCoV-19/USA/WA-S39/2020|EPI_ISL_417092|2020-03-04	....	....	....	....	....	....	
A	538 hCoV-19/USA/WA-S40/2020|EPI_ISL_417093|2020-02-28	....	....	....	....	....	....	
A	539 hCoV-19/USA/WA-S42/2020|EPI_ISL_417095|2020-02-28	....	....	....	....	....	....	
A	540 hCoV-19/USA/WA-S43/2020|EPI_ISL_417096|2020-02-27	....	....	....	....	....	....	
A	541 hCoV-19/USA/WA-S44/2020|EPI_ISL_417097|2020-02-28	....	....	....	....	....	....	
A	542 hCoV-19/USA/WA-S45/2020|EPI_ISL_417098|2020-02-29	....	....	....	....	....	....	
A	543 hCoV-19/USA/WA-S46/2020|EPI_ISL_417099|2020-02-29	....	....	....	....	....	....	
A	544 hCoV-19/England/20108004702/2020|EPI_ISL_417250|20	....	....	....	....	....	....	
A	545 hCoV-19/England/20108006003/2020|EPI_ISL_417252|20	....	....	....	....	....	....	
A	547 hCoV-19/England/20108007002/2020|EPI_ISL_417255|20	....	....	....	....	....	....	
A	548 hCoV-19/England/20108007302/2020|EPI_ISL_417256|20	....	....	....	....	....	....	
A	550 hCoV-19/England/20108034006/2020|EPI_ISL_417258|20	....	....	....	....	....	....	
A	551 hCoV-19/England/20109035906/2020|EPI_ISL_417260|20	....	....	....	....	....	....	
A	552 hCoV-19/England/20109039306/2020|EPI_ISL_417262|20	....	....	....	....	....	....	
A	553 hCoV-19/England/20109050106/2020|EPI_ISL_417263|20	....	....	....	....	....	....	
A	554 hCoV-19/England/20109050306/2020|EPI_ISL_417264|20	....	....	....	....	....	....	
A	555 hCoV-19/England/20109050406/2020|EPI_ISL_417265|20	....	....	....	....	....	....	
A	556 hCoV-19/England/20109050506/2020|EPI_ISL_417266|20	....	....	....	....	....	....	
A	557 hCoV-19/England/20109050606/2020|EPI_ISL_417267|20	....	....	....	....	....	.NNN................	1
A	558 hCoV-19/England/20109050706/2020|EPI_ISL_417268|20	....	....	....	....	....	....	
A	559 hCoV-19/England/20109050806/2020|EPI_ISL_417269|20	....	....	....	....	....	....	
A	560 hCoV-19/England/20109056906/2020|EPI_ISL_417282|20	....	....	....	....	....	....	
A	561 hCoV-19/England/20109058906/2020|EPI_ISL_417283|20	....	....	....	....	....	....	
A	562 hCoV-19/England/20109093606/2020|EPI_ISL_417285|20	....	....	....	....	....	....	

A	563	hCoV-19/England/20109093706/2020|EPI_ISL_417286|20	····················	····················	·····················	····················	····················	····················
A	564	hCoV-19/England/20109093806/2020|EPI_ISL_417287|20	····················	····················	·····················	····················	····················	····················
A	565	hCoV-19/England/20109093906/2020|EPI_ISL_417288|20	····················	····················	·····················	····················	····················	····················
A	566	hCoV-19/England/20109094006/2020|EPI_ISL_417289|20	····················	····················	·····················	····················	····················	····················
A	567	hCoV-19/Ireland/CO-NVRL-22361/2020|EPI_ISL_418580|	····················	····················	·····················	····················	····················	····················
A	568	hCoV-19/Ireland/D-NVRL-22428/2020|EPI_ISL_418581|2	····················	····················	·····················	····················	····················	····················
A	569	hCoV-19/Ireland/LH-NVRL-22901/2020|EPI_ISL_418582|	····················	····················	·····················	····················	····················	····················
A	570	hCoV-19/Ireland/D-NVRL-24042/2020|EPI_ISL_418583|2	····················	····················	·····················	····················	····················	····················
A	571	hCoV-19/Ireland/WW-NVRL-24052/2020|EPI_ISL_418584|	····················	····················	·····················	····················	····················	····················
A	572	hCoV-19/USA/NY-NYUMC42/2020|EPI_ISL_419703|2020-03	····················	····················	·····················	····················	····················	····················
A	574	hCoV-19/Germany/BY-MVP-V2010492/2020|EPI_ISL_42089	····················	····················	·····················	····················	····················	····················
A	575	hCoV-19/USA/WA-UW-1723/2020|EPI_ISL_424200|2020-03	····················	····················	·····················	····················	····················	····················
A	576	hCoV-19/USA/WA-UW-1651/2020|EPI_ISL_424202|2020-03	····················	····················	·····················	····················	····················	····················
A	577	hCoV-19/USA/WA-UW-1652/2020|EPI_ISL_424203|2020-03	····················	····················	·····················	····················	····················	····················
A	578	hCoV-19/USA/WA-UW-1698/2020|EPI_ISL_424204|2020-03	····················	····················	·····················	····················	····················	····················
A	579	hCoV-19/USA/WA-UW-1697/2020|EPI_ISL_424205|2020-03	····················	····················	·····················	····················	····················	····················
A	580	hCoV-19/USA/WA-UW-1716/2020|EPI_ISL_424207|2020-03	····················	····················	·····················	····················	····················	····················
A	581	hCoV-19/USA/WA-UW-1712/2020|EPI_ISL_424208|2020-03	····················	····················	·····················	····················	····················	····················
A	582	hCoV-19/USA/WA-UW-1765/2020|EPI_ISL_424209|2020-03	····················	····················	·····················	····················	····················	····················
A	583	hCoV-19/USA/WA-UW-1718/2020|EPI_ISL_424210|2020-03	····················	····················	·····················	····················	····················	····················
A	584	hCoV-19/USA/WA-UW-1689/2020|EPI_ISL_424211|2020-03	····················	····················	·····················	····················	····················	····················
A	585	hCoV-19/USA/WA-UW-1715/2020|EPI_ISL_424212|2020-03	····················	····················	·····················	····················	····················	····················
A	586	hCoV-19/USA/WA-UW-1670/2020|EPI_ISL_424213|2020-03	····················	····················	·····················	····················	····················	····················
A	587	hCoV-19/USA/WA-UW-1677/2020|EPI_ISL_424214|2020-03	····················	····················	·····················	····················	····················	····················
A	588	hCoV-19/USA/WA-UW-1680/2020|EPI_ISL_424215|2020-03	····················	····················	·····················	····················	····················	····················
A	589	hCoV-19/USA/WA-UW-1690/2020|EPI_ISL_424216|2020-03	····················	····················	·····················	····················	····················	····················
A	590	hCoV-19/USA/WA-UW-1868/2020|EPI_ISL_424280|2020-03	····················	····················	·····················	····················	····················	····················
A	591	hCoV-19/USA/WA-UW-1864/2020|EPI_ISL_424281|2020-03	····················	····················	·····················	····················	····················	····················
A	592	hCoV-19/USA/WA-UW-1835/2020|EPI_ISL_424282|2020-03	····················	····················	·····················	····················	····················	····················
A	593	hCoV-19/USA/WA-UW-1863/2020|EPI_ISL_424284|2020-03	····················	····················	·····················	····················	····················	····················
A	594	hCoV-19/USA/OR-UW-1851/2020|EPI_ISL_424285|2020-03	····················	····················	·····················	····················	····················	····················
A	595	hCoV-19/USA/OR-UW-1849/2020|EPI_ISL_424286|2020-03	····················	····················	·····················	····················	····················	····················
A	596	hCoV-19/USA/WA-UW-1843/2020|EPI_ISL_424287|2020-03	····················	····················	·····················	····················	····················	····················
A	597	hCoV-19/USA/WA-UW-1846/2020|EPI_ISL_424288|2020-03	····················	····················	·····················	····················	····················	····················
A	598	hCoV-19/USA/WA-UW-1860/2020|EPI_ISL_424289|2020-03	····················	····················	·····················	····················	····················	····················
A	599	hCoV-19/England/NOTT-10E12C/2020|EPI_ISL_425501|20	····················	····················	·····················	····················	····················	····················
A	600	hCoV-19/England/NOTT-10E14A/2020|EPI_ISL_425502|20	····················	····················	·····················	····················	····················	····················
A	601	hCoV-19/England/NOTT-10E186/2020|EPI_ISL_425504|20	····················	····················	·····················	····················	····················	····················
A	602	hCoV-19/England/NOTT-10E195/2020|EPI_ISL_425505|20	····················	····················	·····················	····················	····················	····················
A	603	hCoV-19/England/NOTT-10E1A4/2020|EPI_ISL_425506|20	····················	····················	·····················	····················	····················	····················
A	604	hCoV-19/England/NOTT-10E1B3/2020|EPI_ISL_425507|20	····················	····················	·····················	····················	····················	····················
A	605	hCoV-19/England/NOTT-10E1C2/2020|EPI_ISL_425508|20	····················	····················	·····················	····················	····················	····················
A	606	hCoV-19/England/NOTT-10E1D1/2020|EPI_ISL_425509|20	····················	····················	·····················	····················	····················	····················
A	607	hCoV-19/England/NOTT-10E1E0/2020|EPI_ISL_425510|20	····················	····················	·····················	····················	····················	····················
A	608	hCoV-19/England/NOTT-10E1FF/2020|EPI_ISL_425511|20	····················	····················	·····················	····················	····················	····················
A	609	hCoV-19/England/NOTT-10E20B/2020|EPI_ISL_425512|20	····················	····················	·····················	····················	····················	····················
A	610	hCoV-19/England/NOTT-10E21A/2020|EPI_ISL_425513|20	····················	····················	·····················	····················	····················	····················
A	611	hCoV-19/England/NOTT-10E229/2020|EPI_ISL_425514|20	····················	····················	·····················	····················	····················	····················
A	612	hCoV-19/England/NOTT-10E238/2020|EPI_ISL_425515|20	····················	····················	·····················	····················	····················	····················
A	613	hCoV-19/England/NOTT-10E256/2020|EPI_ISL_425517|20	····················	····················	·····················	····················	····················	····················
A	614	hCoV-19/England/NOTT-10E265/2020|EPI_ISL_425518|20	····················	····················	·····················	····················	····················	····················
A	615	hCoV-19/England/NOTT-10E283/2020|EPI_ISL_425519|20	····················	····················	·····················	····················	····················	····················
A	616	hCoV-19/England/NOTT-10E2B0/2020|EPI_ISL_425522|20	····················	····················	·····················	····················	····················	····················
A	617	hCoV-19/England/NOTT-10E2CF/2020|EPI_ISL_425523|20	····················	····················	·····················	····················	····················	····················
A	618	hCoV-19/England/NOTT-10E2ED/2020|EPI_ISL_425525|20	····················	····················	·····················	····················	····················	····················
A	619	hCoV-19/England/NOTT-10E2FC/2020|EPI_ISL_425526|20	····················	····················	·····················	····················	····················	····················
A	620	hCoV-19/England/NOTT-10E308/2020|EPI_ISL_425527|20	····················	····················	·····················	····················	····················	····················
A	621	hCoV-19/England/NOTT-10E335/2020|EPI_ISL_425530|20	····················	····················	·····················	····················	····················	····················
A	622	hCoV-19/England/NOTT-10E362/2020|EPI_ISL_425532|20	····················	····················	·····················	····················	····················	····················
A	623	hCoV-19/England/NOTT-10E371/2020|EPI_ISL_425533|20	····················	····················	·····················	····················	····················	····················
A	624	hCoV-19/England/NOTT-10E39F/2020|EPI_ISL_425535|20	····················	····················	·····················	····················	····················	····················
A	625	hCoV-19/England/NOTT-10E3AE/2020|EPI_ISL_425536|20	····················	····················	·····················	····················	····················	····················
A	626	hCoV-19/England/NOTT-10E3BD/2020|EPI_ISL_425537|20	····················	····················	·····················	····················	····················	····················
A	627	hCoV-19/England/NOTT-10E423/2020|EPI_ISL_425544|20	····················	····················	·····················	····················	····················	····················
A	628	hCoV-19/England/NOTT-10E450/2020|EPI_ISL_425547|20	····················	····················	·····················	····················	····················	····················
A	629	hCoV-19/Australia/VIC534/2020|EPI_ISL_426800|2020-	····················	····················	·····················	····················	····················	····················
A	630	hCoV-19/Australia/VIC535/2020|EPI_ISL_426801|2020-	····················	····················	·····················	····················	····················	····················
A	631	hCoV-19/Australia/VIC536/2020|EPI_ISL_426802|2020-	····················	····················	·····················	····················	····················	·R··················
A	632	hCoV-19/Australia/VIC537/2020|EPI_ISL_426803|2020-	····················	····················	·····················	····················	····················	····················
A	633	hCoV-19/Australia/VIC538/2020|EPI_ISL_426804|2020-	····················	····················	·····················	····················	····················	····················
A	634	hCoV-19/Australia/VIC540/2020|EPI_ISL_426805|2020-	····················	····················	·····················	····················	····················	····················
A	635	hCoV-19/Australia/VIC541/2020|EPI_ISL_426806|2020-	····················	····················	·····················	····················	····················	····················
A	636	hCoV-19/Australia/VIC544/2020|EPI_ISL_426807|2020-	····················	····················	·····················	····················	····················	····················
A	637	hCoV-19/Australia/VIC546/2020|EPI_ISL_426809|2020-	····················	····················	·····················	····················	····················	····················
A	638	hCoV-19/Australia/VIC547/2020|EPI_ISL_426810|2020-	····················	····················	·····················	····················	····················	····················
A	639	hCoV-19/Australia/VIC548/2020|EPI_ISL_426811|2020-	····················	····················	·····················	····················	····················	····················
A	640	hCoV-19/Australia/VIC549/2020|EPI_ISL_426812|2020-	····················	····················	·····················	····················	····················	····················
A	642	hCoV-19/Australia/VIC553/2020|EPI_ISL_426814|2020-	····················	····················	·····················	····················	····················	····················

A	643	hCoV-19/Australia/VIC555/2020|EPI_ISL_426816|2020-	····················	····················	····················	····················	····················	····················
A	645	hCoV-19/Australia/VIC558/2020|EPI_ISL_426819|2020-	····················	····················	····················	····················	····················	····················
A	646	hCoV-19/Australia/VIC560/2020|EPI_ISL_426821|2020-	····················	····················	····················	····················	····················	····················
A	647	hCoV-19/Australia/VIC561/2020|EPI_ISL_426822|2020-	····················	····················	····················	····················	····················	····················
A	649	hCoV-19/Australia/VIC565/2020|EPI_ISL_426825|2020-	····················	····················	····················	····················	····················	····················
A	650	hCoV-19/Australia/VIC566/2020|EPI_ISL_426826|2020-	····················	····················	····················	····················	····················	····················
A	651	hCoV-19/Australia/VIC567/2020|EPI_ISL_426827|2020-	····················	····················	····················	····················	····················	····················
A	652	hCoV-19/Australia/VIC568/2020|EPI_ISL_426828|2020-	····················	····················	····················	····················	····················	····················
A	653	hCoV-19/Australia/VIC570/2020|EPI_ISL_426830|2020-	····················	····················	····················	····················	····················	····················
A	654	hCoV-19/Australia/VIC571/2020|EPI_ISL_426831|2020-	····················	····················	····················	····················	····················	····················
A	655	hCoV-19/Australia/VIC572/2020|EPI_ISL_426832|2020-	····················	····················	····················	····················	····················	····················
A	663	hCoV-19/Australia/VIC584/2020|EPI_ISL_426841|2020-	····················	····················	····················	····················	····················	····················
A	673	hCoV-19/Australia/VIC597/2020|EPI_ISL_426854|2020-	····················	····················	····················	····················	····················	····················
A	674	hCoV-19/Australia/VIC598/2020|EPI_ISL_426855|2020-	····················	····················	····················	····················	····················	····················
A	675	hCoV-19/Australia/VIC599/2020|EPI_ISL_426856|2020-	····················	····················	····················	····················	····················	····················
A	676	hCoV-19/Australia/VIC603/2020|EPI_ISL_426858|2020-	····················	····················	····················	····················	·············Y······	····················
A	677	hCoV-19/Australia/VIC605/2020|EPI_ISL_426860|2020-	····················	····················	····················	····················	····················	····················
A	678	hCoV-19/Australia/VIC606/2020|EPI_ISL_426861|2020-	····················	····················	····················	····················	····················	····················
A	682	hCoV-19/Australia/VIC612/2020|EPI_ISL_426866|2020-	····················	····················	····················	····················	····················	····················
A	684	hCoV-19/Australia/VIC617/2020|EPI_ISL_426872|2020-	····················	····················	····················	····················	····················	····················
A	686	hCoV-19/Australia/VIC630/2020|EPI_ISL_426874|2020-	····················	····················	····················	····················	····················	····················
A	689	hCoV-19/Australia/VIC636/2020|EPI_ISL_426877|2020-	····················	····················	····················	····················	····················	····················
A	691	hCoV-19/Guangzhou/GZMU0003/2020|EPI_ISL_429074|202	····················	····················	····················	····················	····················	····················
A	692	hCoV-19/Guangzhou/GZMU0008/2020|EPI_ISL_429075|202	····················	····················	····················	····················	····················	····················
A	693	hCoV-19/Guangzhou/GZMU0009/2020|EPI_ISL_429076|202	····················	····················	····················	····················	····················	····················
A	694	hCoV-19/Guangzhou/GZMU0010/2020|EPI_ISL_429077|202	····················	····················	····················	····················	····················	····················
A	695	hCoV-19/Guangzhou/GZMU0013/2020|EPI_ISL_429078|202	····················	····················	····················	····················	····················	····················
A	696	hCoV-19/Guangzhou/GZMU0017/2020|EPI_ISL_429079|202	····················	····················	····················	····················	····················	····················
A	697	hCoV-19/Guangzhou/GZMU0019/2020|EPI_ISL_429080|202	····················	····················	····················	····················	····················	····················
A	698	hCoV-19/Guangzhou/GZMU0020/2020|EPI_ISL_429081|202	····················	····················	····················	····················	····················	····················
A	699	hCoV-19/Guangzhou/GZMU0023/2020|EPI_ISL_429082|202	····················	····················	····················	····················	····················	····················
A	700	hCoV-19/Guangzhou/GZMU0024/2020|EPI_ISL_429083|202	····················	····················	····················	····················	····················	····················
A	701	hCoV-19/Guangzhou/GZMU0025/2020|EPI_ISL_429084|202	····················	····················	····················	····················	····················	····················
A	702	hCoV-19/Guangzhou/GZMU0033/2020|EPI_ISL_429085|202	····················	····················	····················	····················	····················	····················
A	703	hCoV-19/Guangzhou/GZMU0034/2020|EPI_ISL_429086|202	····················	····················	····················	····················	····················	····················
A	704	hCoV-19/Guangzhou/GZMU0035/2020|EPI_ISL_429088|202	····················	····················	····················	····················	····················	····················
A	705	hCoV-19/Guangzhou/GZMU0037/2020|EPI_ISL_429089|202	····················	····················	····················	····················	····················	····················
A	706	hCoV-19/Guangzhou/GZMU0039/2020|EPI_ISL_429090|202	····················	····················	····················	····················	····················	····················
A	707	hCoV-19/Guangzhou/GZMU0041/2020|EPI_ISL_429091|202	····················	····················	····················	····················	····················	····················
A	708	hCoV-19/Guangzhou/GZMU0045/2020|EPI_ISL_429092|202	····················	····················	····················	····················	····················	····················
A	709	hCoV-19/Guangzhou/GZMU0046/2020|EPI_ISL_429093|202	····················	····················	····················	····················	····················	····················
A	710	hCoV-19/Guangzhou/GZMU0049/2020|EPI_ISL_429094|202	····················	····················	····················	····················	····················	····················
A	711	hCoV-19/Guangzhou/GZMU0050/2020|EPI_ISL_429095|202	····················	····················	····················	····················	····················	····················
A	712	hCoV-19/Guangzhou/GZMU0051/2020|EPI_ISL_429096|202	····················	····················	····················	····················	····················	····················
A	713	hCoV-19/Guangzhou/GZMU0052/2020|EPI_ISL_429097|202	····················	····················	····················	····················	····················	····················
A	714	hCoV-19/Guangzhou/GZMU0054/2020|EPI_ISL_429098|202	····················	····················	····················	····················	····················	····················
A	715	hCoV-19/Italy/ABR-IZSGC-TE6571/2020|EPI_ISL_429231	····················	····················	····················	····················	····················	····················
A	717	hCoV-19/DRC/1131/2020|EPI_ISL_429255|2020-04-05	····················	····················	····················	····················	····················	····················
A	718	hCoV-19/Spain/MD-FISABIO-Vircell001/2020|EPI_ISL_4	····················	····················	····················	····················	····················	····················
A	720	hCoV-19/DRC/1249/2020|EPI_ISL_429259|2020-04-06	····················	····················	····················	····················	····················	····················
A	721	hCoV-19/Denmark/ALAB-HH01/2020|EPI_ISL_429262|2020	····················	····················	····················	····················	····················	····················
A	722	hCoV-19/Denmark/ALAB-HH02/2020|EPI_ISL_429263|2020	····················	····················	····················	····················	····················	····················
A	723	hCoV-19/Denmark/ALAB-HH03/2020|EPI_ISL_429264|2020	····················	····················	····················	····················	····················	····················
A	724	hCoV-19/Denmark/ALAB-HH04/2020|EPI_ISL_429265|2020	····················	····················	····················	····················	····················	····················
A	725	hCoV-19/Denmark/ALAB-HH05/2020|EPI_ISL_429266|2020	····················	····················	····················	····················	····················	····················
A	726	hCoV-19/Denmark/ALAB-HH06/2020|EPI_ISL_429267|2020	····················	····················	····················	····················	····················	····················
A	727	hCoV-19/Denmark/ALAB-HH07/2020|EPI_ISL_429268|2020	····················	····················	····················	····················	····················	····················
A	728	hCoV-19/Denmark/ALAB-HH08/2020|EPI_ISL_429269|2020	····················	····················	····················	····················	····················	····················
A	729	hCoV-19/USA/UT-0511/2020|EPI_ISL_430058|2020-04-03	····················	····················	····················	····················	····················	····················
A	730	hCoV-19/USA/UT-0513/2020|EPI_ISL_430059|2020-04-03	····················	····················	····················	····················	····················	····················
A	732	hCoV-19/USA/UT-0519/2020|EPI_ISL_430061|2020-04-03	····················	····················	····················	····················	····················	····················
A	733	hCoV-19/USA/UT-0520/2020|EPI_ISL_430062|2020-04-03	····················	····················	····················	····················	····················	····················
A	734	hCoV-19/Australia/VIC-CBA4/2020|EPI_ISL_430064|202	····················	····················	····················	····················	····················	····················
A	735	hCoV-19/Australia/VIC-CBA5/2020|EPI_ISL_430065|202	····················	····················	····················	····················	····················	····················
A	736	hCoV-19/Russia/StPetersburg-RII4705S/2020|EPI_ISL_	····················	····················	····················	····················	····················	····················
A	737	hCoV-19/Russia/StPetersburg-RII4712V/2020|EPI_ISL_	····················	····················	····················	····················	····················	····················
A	738	hCoV-19/Russia/StPetersburg-RII4780S/2020|EPI_ISL_	····················	····················	····················	····················	····················	····················
A	739	hCoV-19/Russia/StPetersburg-RII4917S/2020|EPI_ISL_	····················	····················	····················	····················	····················	····················
A	740	hCoV-19/Russia/StPetersburg-RII4936S/2020|EPI_ISL_	····················	····················	····················	····················	····················	····················
A	741	hCoV-19/Russia/StPetersburg-RII4937S/2020|EPI_ISL_	····················	····················	····················	····················	····················	····················
A	742	hCoV-19/Russia/StPetersburg-RII4938S/2020|EPI_ISL_	····················	····················	····················	····················	····················	····················
A	743	hCoV-19/Russia/StPetersburg-RII4944S/2020|EPI_ISL_	····················	····················	····················	····················	····················	····················
A	744	hCoV-19/Russia/StPetersburg-RII4949S/2020|EPI_ISL_	····················	····················	····················	····················	····················	····················
A	745	hCoV-19/Russia/StPetersburg-RII4969S/2020|EPI_ISL_	····················	····················	····················	····················	····················	····················
A	746	hCoV-19/Russia/StPetersburg-RII4970S/2020|EPI_ISL_	····················	····················	····················	····················	····················	····················
A	747	hCoV-19/Russia/StPetersburg-RII4972S/2020|EPI_ISL_	····················	····················	····················	····················	····················	····················
A	748	hCoV-19/Russia/StPetersburg-RII4980S/2020|EPI_ISL_	····················	····················	····················	····················	····················	····················
A	749	hCoV-19/Russia/StPetersburg-RII4983S/2020|EPI_ISL_	····················	····················	····················	····················	····················	····················

```
A 750 hCoV-19/Russia/StPetersburg-RII4984S/2020|EPI_ISL_ .................... .................... .................... .................... .................... ....................
A 751 hCoV-19/Russia/StPetersburg-RII4985S/2020|EPI_ISL_ .................... .................... .................... .................... .................... ....................
A 752 hCoV-19/Russia/StPetersburg-RII4988S/2020|EPI_ISL_ .................... .................... .................... .................... .................... ....................
A 753 hCoV-19/Russia/StPetersburg-RII5017V/2020|EPI_ISL_ .................... .................... .................... .................... .................... ....................
A 754 hCoV-19/Russia/StPetersburg-RII5029S/2020|EPI_ISL_ .................... .................... .................... .................... .................... ....................
A 755 hCoV-19/Russia/StPetersburg-RII5033S/2020|EPI_ISL_ .................... .................... .................... .................... .................... ....................
A 756 hCoV-19/Russia/StPetersburg-RII5047S/2020|EPI_ISL_ .................... .................... .................... .................... .................... ....................
A 757 hCoV-19/Russia/StPetersburg-RII5169S/2020|EPI_ISL_ .................... .................... .................... .................... .................... ....................
A 758 hCoV-19/Russia/StPetersburg-RII5170S/2020|EPI_ISL_ .................... .................... .................... .................... .................... ....................
A 759 hCoV-19/Russia/StPetersburg-RII5244S/2020|EPI_ISL_ .................... .................... .................... .................... .................... ....................
A 760 hCoV-19/Russia/StPetersburg-RII5247S/2020|EPI_ISL_ .................... .................... .................... .................... .................... ....................
A 761 hCoV-19/Russia/StPetersburg-RII5633S/2020|EPI_ISL_ .................... .................... .................... .................... .................... ....................
A 762 hCoV-19/Russia/StPetersburg-RII5634S/2020|EPI_ISL_ .................... .................... .................... .................... .................... ....................
A 763 hCoV-19/Russia/StPetersburg-RII5635S/2020|EPI_ISL_ .................... .................... .................... .................... .................... ....................
A 764 hCoV-19/Russia/StPetersburg-RII5642S/2020|EPI_ISL_ .................... .................... .................... .................... .................... ....................
A 765 hCoV-19/Russia/StPetersburg-RII5643S/2020|EPI_ISL_ .................... .................... .................... .................... .................... ....................
A 766 hCoV-19/Russia/StPetersburg-RII5644S/2020|EPI_ISL_ .................... .................... .................... .................... .................... ....................
A 767 hCoV-19/Russia/StPetersburg-RII5654S/2020|EPI_ISL_ .................... .................... .................... .................... .................... ....................
A 768 hCoV-19/Russia/StPetersburg-RII5942S/2020|EPI_ISL_ .................... .................... .................... .................... .................... ....................
A 769 hCoV-19/Guangdong/20SF174/2020|EPI_ISL_406531|2020 .................... .................... .................... .................... .................... ....................
A 770 hCoV-19/Guangzhou/20SF206/2020|EPI_ISL_406533|2020 .................... .................... .................... .................... .................... ....................
A 771 hCoV-19/Foshan/20SF207/2020|EPI_ISL_406534|2020-01 .................... .A.................. .................... .................... .................... ....................
A 772 hCoV-19/Foshan/20SF210/2020|EPI_ISL_406535|2020-01 .................... .................... .................... .................... .................... ....................
A 773 hCoV-19/Foshan/20SF211/2020|EPI_ISL_406536|2020-01 .................... .................... .................... .................... .................... ....................
A 774 hCoV-19/Guangdong/20SF201/2020|EPI_ISL_406538|2020 .................... .................... .................... .................... .................... ....................
A 775 hCoV-19/Shenzhen/SZTH-001/2020|EPI_ISL_406592|2020 .................... .................... .................... .................... .................... ....................
A 776 hCoV-19/Shenzhen/SZTH-002/2020|EPI_ISL_406593|2020 .................... .................... .................... .................... .................... ....................
A 777 hCoV-19/France/IDF-0372/2020|EPI_ISL_406596|2020-0 .................... .................... .................... .................... .................... ....................
A 778 hCoV-19/France/IDF-0373/2020|EPI_ISL_406597|2020-0 .................... .................... .................... .................... .................... ....................
A 779 hCoV-19/Australia/NSW01/2020|EPI_ISL_407893|2020-0 .................... .................... .................... .................... .................... ....................
A 780 hCoV-19/Australia/QLD01/2020|EPI_ISL_407894|2020-0 .................... .................... .................... .................... .................... ....................
A 781 hCoV-19/Australia/QLD02/2020|EPI_ISL_407896|2020-0 .................... .................... .................... .................... .................... ....................
A 782 hCoV-19/Weifang/WF0014/2020|EPI_ISL_413711|2020-02 .................... .................... .................... .................... .................... ....................
A 783 hCoV-19/USA/WI-UW-06/2020|EPI_ISL_417200|2020-03-2 .................... .................... .................... .................... .................... ....................
A 784 hCoV-19/USA/WI-UW-07/2020|EPI_ISL_417201|2020-03-2 .................... .................... .................... .................... .................... ....................
A 785 hCoV-19/USA/WI-UW-08/2020|EPI_ISL_417202|2020-03-2 .................... .................... .................... .................... .................... ....................
A 786 hCoV-19/USA/WI-UW-09/2020|EPI_ISL_417203|2020-03-2 .................... .................... .................... .................... .................... ....................
A 787 hCoV-19/USA/WI-UW-10/2020|EPI_ISL_417204|2020-03-2 .................... .................... .................... .................... .................... ....................
A 788 hCoV-19/New Zealand/CoV001/2020|EPI_ISL_417211|202 .................... .................... .................... .................... .................... ....................
A 789 hCoV-19/New Zealand/CoV002/2020|EPI_ISL_417212|202 .................... .................... .................... .................... .................... ....................
A 790 hCoV-19/England/20102068502/2020|EPI_ISL_417213|20 .................... .................... .................... .................... .................... ....................
A 793 hCoV-19/England/20104002606/2020|EPI_ISL_417220|20 .................... .................... .................... .................... .................... ....................
A 794 hCoV-19/England/20104003002/2020|EPI_ISL_417222|20 .................... .................... .................... .................... .................... ....................
A 795 hCoV-19/England/20104004902/2020|EPI_ISL_417226|20 .................... .................... .................... .................... .................... ....................
A 796 hCoV-19/England/20104007503/2020|EPI_ISL_417227|20 .................... .................... .................... .................... .................... ....................
A 797 hCoV-19/England/20104008402/2020|EPI_ISL_417228|20 .................... .................... .................... .................... .................... ....................
A 798 hCoV-19/England/20104008702/2020|EPI_ISL_417230|20 .................... .................... .................... .................... .................... ....................
A 799 hCoV-19/England/20104008802/2020|EPI_ISL_417231|20 .................... .................... .................... .................... .................... ....................
A 800 hCoV-19/England/20104008902/2020|EPI_ISL_417232|20 .................... .................... .................... .................... .................... ....................
A 801 hCoV-19/England/20104009002/2020|EPI_ISL_417233|20 .................... .................... .................... .................... .................... ....................
A 802 hCoV-19/England/20104009102/2020|EPI_ISL_417234|20 .................... .................... .................... .................... .................... ....................
A 803 hCoV-19/England/20104013703/2020|EPI_ISL_417235|20 .................... .................... .................... .................... .................... ....................
A 804 hCoV-19/England/20104015302/2020|EPI_ISL_417236|20 .................... .................... .................... .................... .................... ....................
A 805 hCoV-19/England/20104035803/2020|EPI_ISL_417238|20 .................... ......C............. .................... .................... .................... ....................
A 806 hCoV-19/England/20106003303/2020|EPI_ISL_417239|20 .................... .................... .................... .................... .................... ....................
A 807 hCoV-19/England/20106004803/2020|EPI_ISL_417240|20 .................... .................... .................... .................... .................... ....................
A 808 hCoV-19/England/20106005403/2020|EPI_ISL_417244|20 .................... .................... .................... .................... .................... ....................
A 809 hCoV-19/England/20106145903/2020|EPI_ISL_417246|20 .................... .................... .................... .................... .................... ....................
A 810 hCoV-19/England/20108003302/2020|EPI_ISL_417248|20 .................... .................... .................... .................... .................... ....................
A 811 hCoV-19/England/20109051806/2020|EPI_ISL_417270|20 .................... .................... .................... .................... .................... ....................
A 812 hCoV-19/England/20109052006/2020|EPI_ISL_417272|20 .................... .................... .................... .................... .................... ....................
A 813 hCoV-19/England/20109052106/2020|EPI_ISL_417273|20 .................... .................... .................... .................... .................... ....................
A 814 hCoV-19/England/20109052506/2020|EPI_ISL_417276|20 .................... .................... .................... .................... .................... ....................
A 815 hCoV-19/England/20109053406/2020|EPI_ISL_417278|20 .................... .................... .................... .................... .................... ....................
A 816 hCoV-19/England/20109053606/2020|EPI_ISL_417279|20 .................... .................... .................... .................... .................... ....................
A 817 hCoV-19/England/20109054806/2020|EPI_ISL_417280|20 .................... .................... .................... .................... .................... ....................
A 818 hCoV-19/England/20109094106/2020|EPI_ISL_417290|20 .................... .................... .................... .................... .................... ....................
A 819 hCoV-19/England/20109094206/2020|EPI_ISL_417291|20 .................... .................... .................... .................... .................... ....................
A 820 hCoV-19/England/20109094506/2020|EPI_ISL_417292|20 .................... .................... .................... .................... .................... ....................
A 821 hCoV-19/England/20109097506/2020|EPI_ISL_417293|20 .................... .................... .................... .................... .................... ....................
A 822 hCoV-19/England/20109098806/2020|EPI_ISL_417295|20 .................... .................... .................... .................... .................... ....................
A 823 hCoV-19/England/20109098906/2020|EPI_ISL_417296|20 .................... .................... .................... .................... .................... ....................
A 824 hCoV-19/England/20109099006/2020|EPI_ISL_417297|20 .................... .................... .................... .................... .................... ....................
A 825 hCoV-19/England/20109099106/2020|EPI_ISL_417298|20 .................... .................... .................... .................... .................... ....................
A 826 hCoV-19/England/20109099206/2020|EPI_ISL_417299|20 .................... .................... .................... .................... .................... ....................
A 827 hCoV-19/Hangzhou/HZ60/2020|EPI_ISL_418502|2020-01- .................... .................... .................... .................... .................... ....................
A 828 hCoV-19/Hangzhou/HZ62/2020|EPI_ISL_418503|2020-01- .................... .................... .................... .................... .................... ....................
A 829 hCoV-19/Hangzhou/HZ79/2020|EPI_ISL_418504|2020-01- .................... .................... .................... .................... .................... ....................
```

A	830	hCoV-19/Hangzhou/HZ90/2020|EPI_ISL_418506|2020-01-	....	....	....	....	....	....
A	831	hCoV-19/Hangzhou/HZ91/2020|EPI_ISL_418507|2020-01-	....	....	....	....	....	....
A	832	hCoV-19/Hangzhou/HZ162/2020|EPI_ISL_418508|2020-01	....	....	....	....	....	....
A	833	hCoV-19/Hangzhou/HZ178/2020|EPI_ISL_418509|2020-01	....	....	....	....	....	....
A	834	hCoV-19/Hangzhou/HZ185/2020|EPI_ISL_418510|2020-01	....	....	....	....	....	....
A	835	hCoV-19/Hangzhou/HZ477/2020|EPI_ISL_418511|2020-01	....	....	....	....	....	....
A	836	hCoV-19/Hangzhou/HZ481/2020|EPI_ISL_418512|2020-01	....	....	....	....	....	....
A	837	hCoV-19/Hangzhou/HZ551/2020|EPI_ISL_418513|2020-01	....	....	....	....	....	....
A	838	hCoV-19/Hangzhou/HZ576/2020|EPI_ISL_418514|2020-01	....	....	....	....	....	....
A	839	hCoV-19/Hangzhou/HZ638/2020|EPI_ISL_418515|2020-01	....	....	....	....	....	....
A	840	hCoV-19/Ireland/TA-NVRL-21023/2020|EPI_ISL_418516|	....	....	....	....	....	....
A	841	hCoV-19/Ireland/D-NVRL-21145/2020|EPI_ISL_418548|2	....	....	....	....	....	....
A	842	hCoV-19/Australia/VIC135/2020|EPI_ISL_419828|2020-	....	....	....	....	....	....
A	843	hCoV-19/Australia/VIC136/2020|EPI_ISL_419829|2020-	....	....	....	....	....	....
A	844	hCoV-19/Australia/VIC137/2020|EPI_ISL_419830|2020-	....	....	....	....	....	....
A	845	hCoV-19/Australia/NT01/2020|EPI_ISL_419831|2020	....	....	....	....	....	....
A	846	hCoV-19/Australia/NT02/2020|EPI_ISL_419832|2020	....	....	....	....	....	....
A	847	hCoV-19/Australia/VIC138/2020|EPI_ISL_419834|2020-	....	....	....	....	....	....
A	848	hCoV-19/Australia/NT05/2020|EPI_ISL_419835|2020	....	....	....	....	....	....
A	849	hCoV-19/Australia/VIC139/2020|EPI_ISL_419836|2020-	....	....	....	....	....	....
A	850	hCoV-19/Australia/VIC140/2020|EPI_ISL_419837|2020-	....	....	....	....	....	....
A	851	hCoV-19/Australia/VIC141/2020|EPI_ISL_419838|2020-	....	....	....	....	....	....
A	852	hCoV-19/Australia/VIC142/2020|EPI_ISL_419839|2020-	....	....	....	....	....	....
A	853	hCoV-19/Australia/VIC143/2020|EPI_ISL_419840|2020-	....	....	....	....	....	....
A	854	hCoV-19/Australia/VIC144/2020|EPI_ISL_419841|2020-	....	....	....	....	....	....
A	855	hCoV-19/Australia/VIC145/2020|EPI_ISL_419842|2020-	....	....	....	....	....	....
A	856	hCoV-19/Australia/VIC146/2020|EPI_ISL_419843|2020-	....	....	....	....	....	....
A	857	hCoV-19/Australia/VIC147/2020|EPI_ISL_419844|2020-	....	....	....	....	....	....
A	858	hCoV-19/Australia/VIC148/2020|EPI_ISL_419845|2020-	....	....	....	....	....	....
A	859	hCoV-19/Australia/VIC149/2020|EPI_ISL_419846|2020-	....	....	....	....	....	....
A	860	hCoV-19/Australia/VIC150/2020|EPI_ISL_419847|2020-	....	....	....	....	....	....
A	861	hCoV-19/Australia/VIC151/2020|EPI_ISL_419848|2020-	....	....	....	....	....	....
A	862	hCoV-19/Australia/VIC152/2020|EPI_ISL_419849|2020-	....	....	....	....	....	....
A	863	hCoV-19/Australia/VIC153/2020|EPI_ISL_419850|2020-	....	....	....	....	....	....
A	864	hCoV-19/Australia/VIC154/2020|EPI_ISL_419851|2020-	....	....	....	....	....	....
A	865	hCoV-19/Australia/VIC155/2020|EPI_ISL_419852|2020-	....	....	....	....	....	....
A	866	hCoV-19/Australia/VIC156/2020|EPI_ISL_419853|2020-	....	....	....	....	....	....
A	867	hCoV-19/Australia/VIC157/2020|EPI_ISL_419854|2020-	....	....	....	....	....	....
A	868	hCoV-19/Australia/VIC158/2020|EPI_ISL_419855|2020-	....	....	....	....	....	....
A	869	hCoV-19/Australia/VIC159/2020|EPI_ISL_419856|2020-	....	....	....	....	....	....
A	870	hCoV-19/Australia/VIC160/2020|EPI_ISL_419857|2020-	....	....	....	....	....	....
A	871	hCoV-19/Australia/VIC162/2020|EPI_ISL_419859|2020-	....	....	....	....	....	....
A	872	hCoV-19/Australia/VIC163/2020|EPI_ISL_419860|2020-	....	....	....	....	....	....
A	873	hCoV-19/Australia/VIC164/2020|EPI_ISL_419861|2020-	....	....	....	....	....	....
A	874	hCoV-19/Australia/VIC165/2020|EPI_ISL_419862|2020-	....	....	....	....	....	....
A	875	hCoV-19/Australia/VIC166/2020|EPI_ISL_419863|2020-	....	....	....	....	....	....
A	876	hCoV-19/Australia/VIC167/2020|EPI_ISL_419864|2020-	....	....	....	....	....	....
A	877	hCoV-19/Australia/VIC168/2020|EPI_ISL_419865|2020-	....	....	....	....	....	....
A	878	hCoV-19/Australia/VIC169/2020|EPI_ISL_419866|2020-	....	.............A......	....	....	....	....
A	879	hCoV-19/Australia/VIC170/2020|EPI_ISL_419867|2020-	....	....	....	....	....	....
A	880	hCoV-19/Australia/VIC171/2020|EPI_ISL_419868|2020-	....	....	....	....	....	....
A	881	hCoV-19/Australia/VIC172/2020|EPI_ISL_419869|2020-	....	....	....	....	....	....
A	882	hCoV-19/Australia/VIC173/2020|EPI_ISL_419870|2020-	....	....	....	....	....	....
A	883	hCoV-19/Australia/VIC174/2020|EPI_ISL_419871|2020-	....	....	....	....	....	....
A	884	hCoV-19/Australia/VIC175/2020|EPI_ISL_419872|2020-	....	....	....	....	....	....
A	885	hCoV-19/Australia/VIC176/2020|EPI_ISL_419873|2020-	....	....	....	....	....	....
A	886	hCoV-19/Australia/VIC177/2020|EPI_ISL_419874|2020-	....	....	....	....	....	....
A	887	hCoV-19/Australia/VIC178/2020|EPI_ISL_419875|2020-	....	....	....	....	....	....
A	888	hCoV-19/Australia/VIC179/2020|EPI_ISL_419876|2020-	....	....	....	....	....	....
A	889	hCoV-19/Australia/VIC180/2020|EPI_ISL_419877|2020-	....	....	....	....	....	....
A	890	hCoV-19/Australia/VIC181/2020|EPI_ISL_419878|2020-	....	....	....	....	....	....
A	891	hCoV-19/Australia/VIC183/2020|EPI_ISL_419879|2020-	....	....	....	....	....	....
A	892	hCoV-19/Australia/VIC184/2020|EPI_ISL_419880|2020-	....	....	....	....	....	....
A	893	hCoV-19/Australia/VIC185/2020|EPI_ISL_419881|2020-	....	....	....	....	....	....
A	894	hCoV-19/Australia/VIC186/2020|EPI_ISL_419882|2020-	....	....	....	....	....	....
A	895	hCoV-19/Australia/VIC187/2020|EPI_ISL_419883|2020-	....	....	....	....	....	....
A	896	hCoV-19/Australia/VIC188/2020|EPI_ISL_419884|2020-	....	....	....	....	....	....
A	897	hCoV-19/Australia/VIC189/2020|EPI_ISL_419885|2020-	....	....	....	....	....	....
A	898	hCoV-19/Australia/VIC190/2020|EPI_ISL_419886|2020-	....	....	....	....	....	....
A	899	hCoV-19/Australia/VIC191/2020|EPI_ISL_419887|2020-	....	....	....	....	....	....
A	900	hCoV-19/Australia/VIC192/2020|EPI_ISL_419888|2020-	....	....	....	....	....	....
A	901	hCoV-19/Australia/VIC193/2020|EPI_ISL_419889|2020-	....	....	....	....	....	....
A	902	hCoV-19/Australia/VIC194/2020|EPI_ISL_419890|2020-	.......C............	....	....	....	....	....
A	903	hCoV-19/Australia/VIC195/2020|EPI_ISL_419891|2020-	....	....	....	....	....	....
A	904	hCoV-19/Australia/VIC196/2020|EPI_ISL_419892|2020-	....	....	....	....	....	....
A	905	hCoV-19/Australia/VIC197/2020|EPI_ISL_419893|2020-	....	....	....	....	....	....
A	906	hCoV-19/Australia/VIC198/2020|EPI_ISL_419894|2020-	....	....	....	....	....	....
A	907	hCoV-19/Australia/VIC199/2020|EPI_ISL_419895|2020-	....	....	....	....	....	....

A	908	hCoV-19/Australia/VIC201/2020|EPI_ISL_419896|2020-	....	....	....	....	....	....	
A	909	hCoV-19/Australia/VIC202/2020|EPI_ISL_419897|2020-	....	....	....	....	....	....	
A	910	hCoV-19/Australia/VIC203/2020|EPI_ISL_419898|2020-	....	....	....	....	....	....	
A	911	hCoV-19/Australia/VIC204/2020|EPI_ISL_419899|2020-	....	....	....	....	....	....	
A	912	hCoV-19/USA/UT-0290/2020|EPI_ISL_420810|2020-03-27	....	....	....	....	....	....	
A	913	hCoV-19/USA/UT-0297/2020|EPI_ISL_420812|2020-03-27	....	....	....	....	....	....	
A	914	hCoV-19/USA/UT-0301/2020|EPI_ISL_420814|2020-03-27	....	....	....	....	....	....	
A	915	hCoV-19/USA/UT-0347/2020|EPI_ISL_420820|2020-03-25	....	....	....	....	....	....	
A	916	hCoV-19/USA/UT-0348/2020|EPI_ISL_420821|2020-03-26	....	....	....	....	....	....	
A	917	hCoV-19/USA/UT-0349/2020|EPI_ISL_420822|2020-03-25	....	....	....	....	....	....	
A	918	hCoV-19/USA/UT-0352/2020|EPI_ISL_420824|2020-03-26	....	....	....	....	....	....	
A	919	hCoV-19/USA/UT-0361/2020|EPI_ISL_420825|2020-03-25	....	....	....	....	....	....	
A	920	hCoV-19/DRC/253/2020|EPI_ISL_420838|2020-03-22	....	....	....	....	....	....	
A	921	hCoV-19/DRC/254/2020|EPI_ISL_420839|2020-03-22	....	....	....	....	....	....	
A	922	hCoV-19/DRC/299/2020|EPI_ISL_420840|2020-03-20	....	....	....	....	....	....	
A	923	hCoV-19/DRC/300/2020|EPI_ISL_420841|2020-03-22	....	....	....	....	....	....	
A	924	hCoV-19/DRC/307/2020|EPI_ISL_420842|2020-03	....	....	....	....	....	....	
A	925	hCoV-19/DRC/353/2020|EPI_ISL_420843|2020-03	....	....	....	....	....	....	
A	926	hCoV-19/DRC/355/2020|EPI_ISL_420844|2020-03	....	....	....	....	....	....	
A	927	hCoV-19/DRC/397/2020|EPI_ISL_420845|2020-03-26	....	....	....	....	....	....	
A	928	hCoV-19/DRC/400/2020|EPI_ISL_420846|2020-03	....	....	....	....	....	....	
A	929	hCoV-19/DRC/431/2020|EPI_ISL_420847|2020-03-26	....	....	....	....	....	....	
A	930	hCoV-19/DRC/445/2020|EPI_ISL_420848|2020-03	....	....	....	....	....	....	
A	931	hCoV-19/DRC/523/2020|EPI_ISL_420849|2020-03-28	....	....	....	....	....	....	
A	932	hCoV-19/DRC/352/2020|EPI_ISL_420850|2020-03	....	....	....	....	....	....	
A	933	hCoV-19/DRC/376/2020|EPI_ISL_420851|2020-03-25	....	....	....	....	....	....	
A	934	hCoV-19/DRC/396/2020|EPI_ISL_420852|2020-03-26	....	....	....	....	....	....	
A	935	hCoV-19/DRC/402/2020|EPI_ISL_420853|2020-03-26	....	....	....	....	....	....	
A	936	hCoV-19/DRC/521/2020|EPI_ISL_420854|2020-03-25	....	....	....	....	....	....	
A	937	hCoV-19/Australia/VIC-CBA1/2020|EPI_ISL_420855|202	....	....	....	....	....	....	
A	938	hCoV-19/Australia/VIC-CBA2/2020|EPI_ISL_420876|202	....	....	....	....	....	....	
A	939	hCoV-19/Australia/VIC-CBA3/2020|EPI_ISL_420877|202	....	....	....	....	....	....	
A	940	hCoV-19/Australia/QLDID929/2020|EPI_ISL_420878|202	....	....	....	....	....	....	
A	941	hCoV-19/Australia/QLDID931/2020|EPI_ISL_420879|202	....	....	....	....	....	....	
A	942	hCoV-19/Japan/Hu_DP_Kng_19-031/2020|EPI_ISL_420889	....	....	....	....	....	....	
A	943	hCoV-19/Japan/Hu_Kng_19-437/2020|EPI_ISL_420890|20	....	....	....	....	....	....	
A	944	hCoV-19/USA/WA-UW-1569/2020|EPI_ISL_423021|2020-03	....	....	....	....	....	....	
A	945	hCoV-19/USA/WA-UW-1562/2020|EPI_ISL_423022|2020-03	....	....	....	....	....	....	
A	946	hCoV-19/USA/UN-UW-1449/2020|EPI_ISL_423023|2020-03	....	....	....	....	....	....	
A	947	hCoV-19/USA/WA-UW-1435/2020|EPI_ISL_423024|2020-03	....	....	....	....	....	....	
A	948	hCoV-19/USA/WA-UW-1441/2020|EPI_ISL_423025|2020-03	....	....	....	....	....	....	
A	949	hCoV-19/USA/WA-UW-1440/2020|EPI_ISL_423026|2020-03	....	....	....	....	....	....	
A	950	hCoV-19/USA/WA-UW-1470/2020|EPI_ISL_423028|2020-03	....	....	....	....	....	....	
A	951	hCoV-19/England/20134016802/2020|EPI_ISL_423091|20	....	....	....	....	....	....	
A	952	hCoV-19/England/20134016902/2020|EPI_ISL_423092|20	....	....	....	....	....	....	
A	954	hCoV-19/England/20134017504/2020|EPI_ISL_423095|20	....	....	....	....	....	....	
A	955	hCoV-19/England/20134017904/2020|EPI_ISL_423096|20	....	....	....	....	....	....	
A	956	hCoV-19/England/20134018004/2020|EPI_ISL_423097|20	....	....	....	....	....	....	
A	958	hCoV-19/England/20134018602/2020|EPI_ISL_423099|20	....	....	....	....	....	....	
A	959	hCoV-19/USA/FL-UFL-UF1/2020|EPI_ISL_424350|2020-03	....	....	....	....	....	....	
A	960	hCoV-19/USA/FL-UFL-UF2/2020|EPI_ISL_424351|2020-03	....	....	....	....	....	....	
A	961	hCoV-19/Fuyang/FY002/2020|EPI_ISL_424352|2020-03-1	....	....	....	....	....	....	
A	962	hCoV-19/USA/NC_200173/2020|EPI_ISL_424353|2020-03	....	....	....	....	....	....	
A	963	hCoV-19/USA/NC_200189/2020|EPI_ISL_424354|2020-03	....	....	....	....	....	....	
A	964	hCoV-19/Beijing/Wuhan_IME-BJ01/2020|EPI_ISL_424355	....	....	....	....	....	....	
A	965	hCoV-19/Beijing/Wuhan_IME-BJ02/2020|EPI_ISL_424356	....	....	....	....	....	....	
A	966	hCoV-19/Beijing/Wuhan_IME-BJ03/2020|EPI_ISL_424357	....	....	....	....	....	....	
A	967	hCoV-19/Beijing/Wuhan_IME-BJ04/2020|EPI_ISL_424358	....	....	....	....	....	....	
A	968	hCoV-19/Beijing/Wuhan_IME-BJ05/2020|EPI_ISL_424359	....	....	....	....	....	....	
A	969	hCoV-19/Scotland/CVR139/2020|EPI_ISL_425682|2020-0	....	....	....	....	....	....	
A	970	hCoV-19/Scotland/CVR140/2020|EPI_ISL_425684|2020-0	....	....	....	....	....	....	
A	971	hCoV-19/Scotland/CVR141/2020|EPI_ISL_425685|2020-0	....	....	....	....	....	....	
A	972	hCoV-19/Scotland/CVR143/2020|EPI_ISL_425687|2020-0	....	....	....	....	....	....	
A	973	hCoV-19/Scotland/CVR144/2020|EPI_ISL_425688|2020-0	....	....	....	....	....	....	
A	974	hCoV-19/Scotland/CVR161/2020|EPI_ISL_425700|2020-0	....	.A..................	....	....	....	....	
A	975	hCoV-19/Scotland/CVR162/2020|EPI_ISL_425701|2020-0	....	....	....	....	....	....	
A	976	hCoV-19/Switzerland/GE-HUG-5737/2020|EPI_ISL_42920	....	....	....	....	....	....	
A	977	hCoV-19/Switzerland/GE-HUG-2297/2020|EPI_ISL_42920	....	....	....	....	....	....	
A	978	hCoV-19/Switzerland/GE-HUG-1736/2020|EPI_ISL_42920	....	....	....	....	....	....	
A	979	hCoV-19/Switzerland/GE-HUG-7617/2020|EPI_ISL_42920	....	....	....	....	....	....	
A	980	hCoV-19/Switzerland/GE-HUG-5007/2020|EPI_ISL_42920	....	....	....	....	....	....	
A	981	hCoV-19/Switzerland/GE-HUG-1184/2020|EPI_ISL_42920	....	....	....	....	....	....	
A	982	hCoV-19/Switzerland/GE-HUG-2164/2020|EPI_ISL_42920	....	....	....	....	....	....	
A	983	hCoV-19/Switzerland/GE-HUG-3650/2020|EPI_ISL_42920	....	...................A	....	....	....	....	1
A	984	hCoV-19/Switzerland/GE-HUG-8147/2020|EPI_ISL_42921	....	.A..................	....	....	....	....	
A	985	hCoV-19/Switzerland/GE-HUG-5708/2020|EPI_ISL_42921	....	....	....	....	....	....	
A	986	hCoV-19/Switzerland/GE-HUG-0056/2020|EPI_ISL_42921	....	....	....	....	....	....	
A	987	hCoV-19/Switzerland/GE-HUG-8086/2020|EPI_ISL_42921	....	....	....	....	....	....	

```
A    988 hCoV-19/Switzerland/GE-HUG-2759/2020|EPI_ISL_42921  ····················  ····················  ····················  ····················  ····················  ····················
A    989 hCoV-19/Switzerland/GE-HUG-4807/2020|EPI_ISL_42921  ····················  ····················  ····················  ····················  ····················  ····················
A    990 hCoV-19/Switzerland/GE-HUG-6414/2020|EPI_ISL_42921  ····················  ····················  ····················  ····················  ····················  ····················
A    991 hCoV-19/Switzerland/GE-HUG-0304/2020|EPI_ISL_42921  ····················  ·A··················  ····················  ····················  ····················  ····················
A    992 hCoV-19/Switzerland/GE-HUG-2453/2020|EPI_ISL_42921  ····················  ·A··················  ····················  ····················  ····················  ····················
A    993 hCoV-19/Switzerland/GE-HUG-6065/2020|EPI_ISL_42922  ····················  ····················  ····················  ····················  ····················  ····················
A    994 hCoV-19/Switzerland/GE-HUG-6099/2020|EPI_ISL_42922  ····················  ····················  ····················  ····················  ····················  ····················
A    995 hCoV-19/Switzerland/GE-HUG-3655/2020|EPI_ISL_42922  ····················  ····················  ····················  ····················  ····················  ····················
A    996 hCoV-19/Switzerland/GE-HUG-4644/2020|EPI_ISL_42922  ····················  ····················  ····················  ····················  ····················  ····················
A    997 hCoV-19/Italy/ABR-IZSGC-TE5551/2020|EPI_ISL_429226  ····················  ····················  ····················  ····················  ····················  ····················
A    998 hCoV-19/Italy/ABR-IZSGC-TE5553/2020|EPI_ISL_429227  ····················  ····················  ····················  ····················  ····················  ····················
A    999 hCoV-19/Italy/ABR-IZSGC-TE5674/2020|EPI_ISL_429228  ····················  ····················  ····················  ····················  ····················  ····················
A   1000 hCoV-19/Italy/ABR-IZSGC-TE6572/2020|EPI_ISL_429232  ····················  ····················  ····················  ····················  ····················  ····················
A   1001 hCoV-19/Italy/ABR-IZSGC-TE6580/2020|EPI_ISL_429235  ····················  ····················  ····················  ····················  ····················  ····················
A   1002 hCoV-19/Italy/ABR-AMVRC-TE7859/2020|EPI_ISL_429236  ····················  ····················  ····················  ····················  ····················  ····················
A   1003 hCoV-19/Yunnan/0306-466/2020|EPI_ISL_429239|2020-0  ····················  ····················  ····················  ····················  ····················  ····················
A   1004 hCoV-19/Denmark/ALAB-HH11/2020|EPI_ISL_429270|2020  ····················  ····················  ····················  ····················  ····················  ····················
A   1005 hCoV-19/Denmark/ALAB-HH13/2020|EPI_ISL_429271|2020  ····················  ····················  ····················  ····················  ····················  ····················
A   1006 hCoV-19/Denmark/ALAB-HH14/2020|EPI_ISL_429272|2020  ····················  ····················  ····················  ····················  ····················  ····················
A   1007 hCoV-19/Denmark/ALAB-HH15/2020|EPI_ISL_429273|2020  ····················  ····················  ····················  ····················  ····················  ····················
A   1008 hCoV-19/Denmark/ALAB-HH16/2020|EPI_ISL_429274|2020  ····················  ····················  ····················  ····················  ····················  ····················
A   1009 hCoV-19/Denmark/ALAB-HH17/2020|EPI_ISL_429275|2020  ····················  ····················  ····················  ····················  ····················  ····················
A   1010 hCoV-19/Denmark/ALAB-HH18/2020|EPI_ISL_429276|2020  ····················  ····················  ····················  ····················  ····················  ····················
A   1011 hCoV-19/Denmark/ALAB-HH19/2020|EPI_ISL_429277|2020  ····················  ····················  ····················  ····················  ····················  ····················
A   1012 hCoV-19/Denmark/ALAB-HH20/2020|EPI_ISL_429278|2020  ····················  ····················  ····················  ····················  ····················  ····················
A   1013 hCoV-19/Denmark/ALAB-HH21/2020|EPI_ISL_429279|2020  ····················  ····················  ····················  ····················  ····················  ····················
A   1014 hCoV-19/Denmark/ALAB-HH22/2020|EPI_ISL_429280|2020  ····················  ····················  ····················  ····················  ····················  ····················
A   1015 hCoV-19/Denmark/ALAB-HH23/2020|EPI_ISL_429281|2020  ····················  ····················  ····················  ····················  ····················  ····················
A   1016 hCoV-19/Denmark/ALAB-HH24/2020|EPI_ISL_429282|2020  ····················  ····················  ····················  ····················  ····················  ····················
A   1017 hCoV-19/Denmark/ALAB-HH25/2020|EPI_ISL_429283|2020  ····················  ····················  ····················  ····················  ····················  ····················
A   1018 hCoV-19/Denmark/ALAB-HH26/2020|EPI_ISL_429284|2020  ····················  ····················  ····················  ····················  ····················  ····················
A   1019 hCoV-19/Denmark/ALAB-HH27/2020|EPI_ISL_429285|2020  ····················  ····················  ····················  ····················  ····················  ····················
A   1020 hCoV-19/Denmark/ALAB-HH29/2020|EPI_ISL_429287|2020  ····················  ····················  ····················  ····················  ····················  ····················
A   1021 hCoV-19/Denmark/ALAB-HH31/2020|EPI_ISL_429289|2020  ····················  ····················  ····················  ····················  ····················  ····················
A   1022 hCoV-19/Denmark/ALAB-HH37/2020|EPI_ISL_429293|2020  ····················  ····················  ····················  ····················  ····················  ····················
A   1023 hCoV-19/Denmark/ALAB-HH39/2020|EPI_ISL_429295|2020  ····················  ····················  ····················  ····················  ····················  ····················
A   1024 hCoV-19/Denmark/ALAB-HH43/2020|EPI_ISL_429299|2020  ····················  ····················  ····················  ····················  ····················  ····················
A   1025 hCoV-19/Fujian/8/2020|EPI_ISL_411060|2020-01-21     ····················  ····················  ····················  ····················  ····················  ····················
A   1026 hCoV-19/Fujian/13/2020|EPI_ISL_411066|2020-01-22    ····················  ····················  ····················  ····················  ····················  ····················
A   1027 hCoV-19/Beijing/ICDC-DT005/2020|EPI_ISL_412386|202  ····················  ····················  ····················  ····················  ····················  ····················
A   1028 hCoV-19/Finland/FIN03032020A/2020|EPI_ISL_413602|2  ····················  ····················  ····················  ····················  ····················  ····················
A   1029 hCoV-19/Finland/FIN03032020B/2020|EPI_ISL_413603|2  ····················  ····················  ····················  ····················  ····················  ····················
A   1030 hCoV-19/Finland/FIN03032020C/2020|EPI_ISL_413604|2  ····················  ····················  ····················  ····················  ····················  ····················
A   1031 hCoV-19/USA/CruiseA-1/2020|EPI_ISL_413606|2020-02-  ····················  ····················  ····················  ····················  ····················  ····················
A   1032 hCoV-19/USA/CruiseA-2/2020|EPI_ISL_413607|2020-02-  ····················  ····················  ····················  ····················  ····················  ····················
A   1033 hCoV-19/USA/CruiseA-3/2020|EPI_ISL_413608|2020-02-  ····················  ····················  ····················  ····················  ····················  ····················
A   1034 hCoV-19/USA/CruiseA-4/2020|EPI_ISL_413609|2020-02-  ····················  ····················  ····················  ····················  ····················  ····················
A   1035 hCoV-19/USA/CruiseA-5/2020|EPI_ISL_413610|2020-02-  ····················  ····················  ····················  ····················  ····················  ····················
A   1036 hCoV-19/USA/CruiseA-6/2020|EPI_ISL_413611|2020-02-  ····················  ····················  ····················  ····················  ····················  ····················
A   1037 hCoV-19/USA/CruiseA-7/2020|EPI_ISL_413612|2020-02-  ····················  ····················  ····················  ····················  ····················  ····················
A   1038 hCoV-19/USA/CruiseA-8/2020|EPI_ISL_413613|2020-02-  ····················  ····················  ····················  ····················  ····················  ····················
A   1039 hCoV-19/USA/CruiseA-9/2020|EPI_ISL_413614|2020-02-  ····················  ····················  ····················  ····················  ····················  ····················
A   1040 hCoV-19/USA/CruiseA-10/2020|EPI_ISL_413615|2020-02  ····················  ····················  ····················  ····················  ····················  ····················
A   1041 hCoV-19/USA/CruiseA-11/2020|EPI_ISL_413616|2020-02  ····················  ····················  ····················  ····················  ····················  ····················
A   1042 hCoV-19/USA/CruiseA-12/2020|EPI_ISL_413617|2020-02  ····················  ····················  ····················  ····················  ····················  ····················
A   1043 hCoV-19/USA/CruiseA-13/2020|EPI_ISL_413618|2020-02  ····················  ····················  ····················  ····················  ····················  ····················
A   1044 hCoV-19/USA/CruiseA-14/2020|EPI_ISL_413619|2020-02  ····················  ····················  ····················  ····················  ····················  ····················
A   1045 hCoV-19/USA/CruiseA-15/2020|EPI_ISL_413620|2020-02  ····················  ····················  ····················  ····················  ····················  ····················
A   1046 hCoV-19/USA/CruiseA-16/2020|EPI_ISL_413621|2020-02  ····················  ····················  ····················  ····················  ····················  ····················
A   1047 hCoV-19/USA/CruiseA-17/2020|EPI_ISL_413622|2020-02  ····················  ····················  ····················  ····················  ····················  ····················
A   1048 hCoV-19/USA/CruiseA-18/2020|EPI_ISL_413623|2020-02  ····················  ····················  ····················  ····················  ····················  ····················
A   1049 hCoV-19/Weifang/WF0001/2020|EPI_ISL_413691|2020-01  ····················  ····················  ····················  ····················  ····················  ····················
A   1050 hCoV-19/Weifang/WF0002/2020|EPI_ISL_413692|2020-01  ····················  ····················  ····················  ····················  ····················  ····················
A   1051 hCoV-19/Weifang/WF0003/2020|EPI_ISL_413693|2020-01  ····················  ····················  ····················  ····················  ····················  ····················
A   1052 hCoV-19/Weifang/WF0004/2020|EPI_ISL_413694|2020-01  ····················  ····················  ····················  ····················  ····················  ····················
A   1053 hCoV-19/Weifang/WF0012/2020|EPI_ISL_413697|2020-02  ····················  ····················  ····················  ····················  ····················  ····················
A   1054 hCoV-19/Shandong/LY003-2/2020|EPI_ISL_414936|2020-  ····················  ····················  ····················  ····················  ····················  ····················
A   1055 hCoV-19/Shandong/LY004-2/2020|EPI_ISL_414937|2020-  ····················  ····················  ····················  ····················  ····················  ····················
A   1056 hCoV-19/Shandong/LY005-2/2020|EPI_ISL_414938|2020-  ····················  ····················  ····················  ····················  ····················  ····················
A   1057 hCoV-19/Shandong/LY006-2/2020|EPI_ISL_414939|2020-  ····················  ····················  ····················  ····················  ····················  ····················
A   1058 hCoV-19/Shandong/LY007-2/2020|EPI_ISL_414940|2020-  ····················  ····················  ····················  ····················  ····················  ····················
A   1059 hCoV-19/Shandong/LY008-2/2020|EPI_ISL_414941|2020-  ····················  ····················  ····················  ····················  ····················  ····················
A   1060 hCoV-19/Australia/VIC89/2020|EPI_ISL_419800|2020-0  ····················  ····················  ····················  ····················  ····················  ····················
A   1061 hCoV-19/Australia/VIC90/2020|EPI_ISL_419801|2020-0  ····················  ····················  ····················  ····················  ····················  ····················
A   1062 hCoV-19/Australia/VIC45/2020|EPI_ISL_419802|2020-0  ····················  ····················  ····················  ····················  ····················  ····················
A   1063 hCoV-19/Australia/VIC84/2020|EPI_ISL_419803|2020-0  ····················  ····················  ····················  ····················  ····················  ····················
A   1064 hCoV-19/Australia/VIC91/2020|EPI_ISL_419804|2020-0  ····················  ·A··················  ····················  ····················  ····················  ····················
A   1065 hCoV-19/Australia/VIC94/2020|EPI_ISL_419805|2020-0  ····················  ····················  ····················  ····················  ····················  ····················
```

A	1066 hCoV-19/Australia/VIC95/2020|EPI_ISL_419806|2020-0	····················	····················	····················	····················	····················	····················
A	1067 hCoV-19/Australia/VIC96/2020|EPI_ISL_419807|2020-0	····················	····················	····················	····················	····················	····················
A	1068 hCoV-19/Australia/VIC97/2020|EPI_ISL_419808|2020-0	····················	····················	····················	····················	····················	····················
A	1069 hCoV-19/Australia/VIC92/2020|EPI_ISL_419809|2020-0	····················	····················	····················	····················	····················	····················
A	1070 hCoV-19/Australia/VIC98/2020|EPI_ISL_419810|2020-0	····················	····················	····················	····················	····················	····················
A	1071 hCoV-19/Australia/VIC99/2020|EPI_ISL_419811|2020-0	····················	····················	····················	····················	····················	····················
A	1072 hCoV-19/Australia/VIC100/2020|EPI_ISL_419812|2020-	····················	····················	····················	····················	····················	····················
A	1073 hCoV-19/Australia/VIC101/2020|EPI_ISL_419813|2020-	····················	····················	····················	····················	····················	····················
A	1074 hCoV-19/Australia/VIC103/2020|EPI_ISL_419814|2020-	····················	····················	····················	····················	····················	····················
A	1075 hCoV-19/Australia/VIC46/2020|EPI_ISL_419816|2020-0	····················	····················	····················	····················	····················	····················
A	1076 hCoV-19/Australia/VIC102/2020|EPI_ISL_419817|2020-	····················	····················	····················	····················	····················	····················
A	1077 hCoV-19/Australia/VIC104/2020|EPI_ISL_419818|2020-	····················	····················	····················	····················	····················	····················
A	1078 hCoV-19/Australia/VIC105/2020|EPI_ISL_419819|2020-	····················	····················	····················	····················	····················	····················
A	1079 hCoV-19/Australia/VIC106/2020|EPI_ISL_419820|2020-	····················	·A··················	····················	····················	····················	····················
A	1080 hCoV-19/Australia/VIC112/2020|EPI_ISL_419821|2020-	····················	····················	····················	····················	····················	····················
A	1081 hCoV-19/Australia/VIC113/2020|EPI_ISL_419822|2020-	····················	····················	····················	····················	····················	····················
A	1082 hCoV-19/Australia/VIC130/2020|EPI_ISL_419823|2020-	····················	····················	····················	····················	····················	····················
A	1083 hCoV-19/Australia/VIC131/2020|EPI_ISL_419824|2020-	····················	····················	····················	····················	····················	····················
A	1084 hCoV-19/Australia/VIC132/2020|EPI_ISL_419825|2020-	····················	····················	····················	····················	····················	····················
A	1085 hCoV-19/Australia/VIC51/2020|EPI_ISL_419826|2020-0	····················	····················	····················	····················	····················	····················
A	1086 hCoV-19/Australia/VIC134/2020|EPI_ISL_419827|2020-	····················	····················	····················	····················	····················	····················
A	1087 hCoV-19/South Korea/BA-ACH_2718/2020|EPI_ISL_42080	····················	····················	····················	····················	····················	····················
A	1088 hCoV-19/South Korea/BA-ACH_2719/2020|EPI_ISL_42080	····················	····················	····················	····················	····················	····················
A	1089 hCoV-19/USA/UT-089/2020|EPI_ISL_420803|2020-03-12	····················	····················	····················	····················	····················	····················
A	1090 hCoV-19/USA/UT-0289/2020|EPI_ISL_420809|2020-03-24	····················	····················	····················	····················	····················	····················
A	1091 hCoV-19/USA/WA-UW-1448/2020|EPI_ISL_423007|2020-03	····················	····················	····················	····················	····················	····················
A	1092 hCoV-19/USA/WA-UW-1426/2020|EPI_ISL_423008|2020-03	····················	····················	····················	····················	····················	····················
A	1093 hCoV-19/USA/WA-UW-1446/2020|EPI_ISL_423010|2020-03	····················	····················	····················	····················	····················	····················
A	1094 hCoV-19/USA/WA-UW-1561/2020|EPI_ISL_423011|2020-03	····················	····················	····················	····················	····················	····················
A	1095 hCoV-19/USA/WA-UW-1588/2020|EPI_ISL_423012|2020-03	····················	····················	····················	····················	····················	····················
A	1096 hCoV-19/USA/WA-UW-1586/2020|EPI_ISL_423013|2020-03	····················	····················	····················	····················	····················	····················
A	1097 hCoV-19/USA/WA-UW-1587/2020|EPI_ISL_423014|2020-03	····················	····················	····················	····················	····················	····················
A	1098 hCoV-19/USA/WA-UW-1552/2020|EPI_ISL_423015|2020-03	····················	····················	····················	····················	····················	····················
A	1099 hCoV-19/USA/WA-UW-1578/2020|EPI_ISL_423016|2020-03	····················	····················	····················	····················	····················	····················
A	1100 hCoV-19/USA/WA-UW-1591/2020|EPI_ISL_423017|2020-03	····················	····················	····················	····················	····················	····················
A	1101 hCoV-19/USA/WA-UW-1576/2020|EPI_ISL_423018|2020-03	····················	····················	····················	····················	····················	····················
A	1102 hCoV-19/USA/WA-UW-1574/2020|EPI_ISL_423019|2020-03	····················	····················	····················	····················	····················	····················
A	1103 hCoV-19/USA/WA-UW-1570/2020|EPI_ISL_423020|2020-03	····················	····················	····················	····················	····················	····················
A	1104 hCoV-19/USA/WA-UW-1471/2020|EPI_ISL_423029|2020-03	····················	····················	····················	····················	····················	····················
A	1105 hCoV-19/USA/WA-UW-1433/2020|EPI_ISL_423030|2020-03	····················	····················	····················	····················	····················	····················
A	1106 hCoV-19/USA/WA-UW-1425/2020|EPI_ISL_423031|2020-03	····················	····················	····················	····················	····················	····················
A	1107 hCoV-19/USA/WA-UW-1608/2020|EPI_ISL_423032|2020-03	····················	····················	····················	····················	····················	····················
A	1108 hCoV-19/USA/WA-UW-1599/2020|EPI_ISL_423033|2020-03	····················	····················	····················	····················	····················	····················
A	1111 hCoV-19/Thailand/Bangkok-0020/2020|EPI_ISL_423041|	····················	····················	····················	····················	····················	····················
A	1112 hCoV-19/Thailand/Bangkok-0021/2020|EPI_ISL_423042|	····················	····················	····················	····················	····················	····················
A	1113 hCoV-19/Thailand/Bangkok-0022/2020|EPI_ISL_423043|	····················	····················	····················	····················	····················	····················
A	1114 hCoV-19/England/20134000704/2020|EPI_ISL_423044|20	····················	····················	····················	····················	····················	····················
A	1115 hCoV-19/England/20134000804/2020|EPI_ISL_423045|20	····················	····················	····················	····················	····················	····················
A	1116 hCoV-19/England/20134001104/2020|EPI_ISL_423047|20	····················	····················	····················	····················	····················	····················
A	1117 hCoV-19/England/20134001904/2020|EPI_ISL_423052|20	····················	····················	····················	····················	····················	····················
A	1118 hCoV-19/England/20134002104/2020|EPI_ISL_423053|20	····················	····················	····················	····················	····················	····················
A	1121 hCoV-19/England/20134003204/2020|EPI_ISL_423057|20	····················	····················	····················	····················	····················	····················
A	1122 hCoV-19/England/20134003304/2020|EPI_ISL_423058|20	····················	····················	····················	····················	····················	····················
A	1124 hCoV-19/England/20134004704/2020|EPI_ISL_423063|20	····················	····················	····················	····················	····················	····················
A	1125 hCoV-19/England/20134004904/2020|EPI_ISL_423065|20	····················	····················	····················	····················	····················	····················
A	1126 hCoV-19/England/20134005004/2020|EPI_ISL_423066|20	····················	····················	····················	····················	····················	····················
A	1127 hCoV-19/England/20134005104/2020|EPI_ISL_423067|20	····················	····················	····················	····················	····················	····················
A	1128 hCoV-19/England/20134005304/2020|EPI_ISL_423068|20	····················	····················	····················	····················	····················	····················
A	1130 hCoV-19/England/20134006504/2020|EPI_ISL_423070|20	····················	····················	····················	····················	····················	····················
A	1131 hCoV-19/England/20134006604/2020|EPI_ISL_423071|20	····················	····················	····················	····················	····················	····················
A	1132 hCoV-19/England/20134006704/2020|EPI_ISL_423072|20	····················	····················	····················	····················	····················	····················
A	1133 hCoV-19/England/20134007004/2020|EPI_ISL_423073|20	····················	····················	····················	····················	····················	····················
A	1134 hCoV-19/England/20134007104/2020|EPI_ISL_423074|20	····················	····················	····················	····················	····················	····················
A	1135 hCoV-19/England/20134007204/2020|EPI_ISL_423075|20	····················	····················	····················	····················	····················	················N···
A	1136 hCoV-19/England/20134009504/2020|EPI_ISL_423076|20	····················	····················	····················	····················	····················	····················
A	1137 hCoV-19/England/20134010904/2020|EPI_ISL_423078|20	····················	····················	····················	····················	····················	····················
A	1138 hCoV-19/England/20134011704/2020|EPI_ISL_423079|20	····················	····················	····················	····················	····················	····················
A	1139 hCoV-19/England/201340120/2020|EPI_ISL_423081|2020	····················	····················	····················	····················	····················	····················
A	1140 hCoV-19/England/201340127/2020|EPI_ISL_423084|2020	····················	····················	····················	····················	····················	····················
A	1141 hCoV-19/England/20134015504/2020|EPI_ISL_423085|20	····················	····················	····················	····················	··N·················	····················
A	1142 hCoV-19/England/20134015704/2020|EPI_ISL_423086|20	····················	····················	····················	····················	····················	····················
A	1144 hCoV-19/USA/WA-UW-1878/2020|EPI_ISL_424302|2020-03	····················	····················	····················	····················	····················	····················
A	1145 hCoV-19/USA/WA-UW-1930/2020|EPI_ISL_424304|2020-03	····················	····················	····················	····················	····················	····················
A	1146 hCoV-19/USA/WA-UW-1877/2020|EPI_ISL_424305|2020-03	····················	····················	····················	····················	····················	····················
A	1147 hCoV-19/USA/WA-UW-1874/2020|EPI_ISL_424306|2020-03	····················	····················	····················	····················	····················	····················
A	1148 hCoV-19/USA/OR-UW-1885/2020|EPI_ISL_424307|2020-03	····················	····················	····················	····················	····················	····················
A	1149 hCoV-19/USA/OR-UW-1887/2020|EPI_ISL_424308|2020-03	····················	····················	····················	····················	····················	····················
A	1150 hCoV-19/USA/WA-UW-1881/2020|EPI_ISL_424309|2020-03	····················	····················	····················	····················	····················	····················

A	1151	hCoV-19/USA/WA-UW-1888/2020|EPI_ISL_424310|2020-03	....	....	....	....	....	....
A	1152	hCoV-19/USA/WA-UW-1880/2020|EPI_ISL_424311|2020-03	....	....	....	....	....	....
A	1153	hCoV-19/USA/WA-UW-1955/2020|EPI_ISL_424312|2020-03	....	....	....	....	....	....
A	1154	hCoV-19/USA/WA-UW-1956/2020|EPI_ISL_424313|2020-03	....	....	....	....	....	....
A	1155	hCoV-19/USA/WA-UW-1921/2020|EPI_ISL_424314|2020-03	....	....	....	....	....	....
A	1156	hCoV-19/USA/ID-UW-1957/2020|EPI_ISL_424315|2020-03	....	....	....	....	....	....
A	1157	hCoV-19/USA/WA-UW-1986/2020|EPI_ISL_424338|2020-03	....	....	....	....	....	....
A	1158	hCoV-19/USA/WA-UW-2016/2020|EPI_ISL_424339|2020-03	....	....	....	....	....	....
A	1159	hCoV-19/USA/WA-UW-2009/2020|EPI_ISL_424340|2020-03	....	....	....	....	....	....
A	1160	hCoV-19/USA/WA-UW-2008/2020|EPI_ISL_424341|2020-03	....	....	....	....	....	....
A	1161	hCoV-19/Italy/LAZ-INMI-8/2020|EPI_ISL_424342|2020-	....	....	....	....	....	....
A	1162	hCoV-19/Italy/LAZ-INMI-9/2020|EPI_ISL_424343|2020-	....	....	....	....	....	....
A	1163	hCoV-19/Italy/LAZ-INMI10/2020|EPI_ISL_424344|2020-	....	....	....	....	....	....
A	1164	hCoV-19/Mexico/CMX-INER-01/2020|EPI_ISL_424345|202	....	....	....	....	....	....
A	1165	hCoV-19/USA/AK-PHL037/2020|EPI_ISL_424346|2020-03-	....	....	....	....	....	....
A	1166	hCoV-19/USA/AK-PHL077/2020|EPI_ISL_424347|2020-03-	....	....	....	....	....	....
A	1167	hCoV-19/Beijing/Wuhan_IME-BJ07/2020|EPI_ISL_424360	....	....	....	....	....	....
A	1168	hCoV-19/India/UN-1063/2020|EPI_ISL_424361|2020-03-	....	....	....	....	....	....
A	1169	hCoV-19/India/UN-1135/2020|EPI_ISL_424362|2020-03-	....	....	....	....	....	....
A	1170	hCoV-19/India/UN-1652/2020|EPI_ISL_424363|2020-03-	....	....	....	....	....	....
A	1171	hCoV-19/India/UN-3118/2020|EPI_ISL_424364|2020-03-	....	....	....	....	....	....
A	1172	hCoV-19/India/UN-3239/2020|EPI_ISL_424365|2020-03-	....	....	....	....	....	....
A	1173	hCoV-19/Turkey/ERAGEM-001/2020|EPI_ISL_424366|2020	....	....	....	....	....	....
A	1174	hCoV-19/Iceland/343/2020|EPI_ISL_424367|2020-03	....	....	....	....	....	....
A	1175	hCoV-19/Iceland/344/2020|EPI_ISL_424368|2020-03	....	....	....	....	....	....
A	1176	hCoV-19/Iceland/345/2020|EPI_ISL_424369|2020-03	....	....	....	....	....	....
A	1177	hCoV-19/Iceland/346/2020|EPI_ISL_424370|2020-03	....	....	....	....	....	....
A	1178	hCoV-19/Iceland/347/2020|EPI_ISL_424371|2020-03	....	....	....	....	....	....
A	1179	hCoV-19/Iceland/351/2020|EPI_ISL_424375|2020-03	....	....	....	....	....	....
A	1180	hCoV-19/Iceland/352/2020|EPI_ISL_424376|2020-03	....	....	....	....	....	....
A	1181	hCoV-19/Iceland/353/2020|EPI_ISL_424377|2020-03-18	....	....	....	....	....	....
A	1182	hCoV-19/Iceland/355/2020|EPI_ISL_424379|2020-03-19	....	....	....	....	....	....
A	1183	hCoV-19/Iceland/356/2020|EPI_ISL_424380|2020-03-19	....	....	....	....	....	....
A	1184	hCoV-19/Iceland/357/2020|EPI_ISL_424381|2020-03-19	....	....	....	....	....	....
A	1185	hCoV-19/Iceland/358/2020|EPI_ISL_424382|2020-03-19	....	....	....	....	....	....
A	1186	hCoV-19/Iceland/359/2020|EPI_ISL_424383|2020-03-19	....	....	....	....	....	....
A	1187	hCoV-19/Iceland/360/2020|EPI_ISL_424384|2020-03-19	....	....	....	....	....	....
A	1188	hCoV-19/Iceland/361/2020|EPI_ISL_424385|2020-03-19	....	....	....	....	....	....
A	1189	hCoV-19/Iceland/363/2020|EPI_ISL_424387|2020-03-19	....	....	....	....	....	....
A	1190	hCoV-19/Iceland/364/2020|EPI_ISL_424388|2020-03-19	....	....	....	....	....	....
A	1191	hCoV-19/Iceland/365/2020|EPI_ISL_424389|2020-03-19	....	....	....	....	....	....
A	1192	hCoV-19/Iceland/366/2020|EPI_ISL_424390|2020-03-19	....	....	....	....	....	....
A	1193	hCoV-19/Iceland/367/2020|EPI_ISL_424391|2020-03-19	....	....	....	....	....	....
A	1194	hCoV-19/Iceland/368/2020|EPI_ISL_424392|2020-03-19	....	....	....	....	....	....
A	1195	hCoV-19/Iceland/369/2020|EPI_ISL_424393|2020-03-19	....	....	....	....	....	....
A	1196	hCoV-19/Iceland/370/2020|EPI_ISL_424394|2020-03-19	....	....	....	....	....	....
A	1197	hCoV-19/Iceland/371/2020|EPI_ISL_424395|2020-03-19	....	....	....	....	....	....
A	1198	hCoV-19/Iceland/372/2020|EPI_ISL_424396|2020-03-19	....	....	....	....	....	....
A	1199	hCoV-19/Iceland/374/2020|EPI_ISL_424398|2020-03-19	....	....	....	....	....	....
A	1200	hCoV-19/Iceland/375/2020|EPI_ISL_424399|2020-03-19	....	....	....	....	....	....
A	1201	hCoV-19/England/NOTT-10E836/2020|EPI_ISL_425600|20	....	....	....	....	....	....
A	1202	hCoV-19/England/NOTT-10E845/2020|EPI_ISL_425601|20	....	....	....	....	....	....
A	1203	hCoV-19/England/NOTT-10E872/2020|EPI_ISL_425603|20	....	....	....	....	....	....
A	1204	hCoV-19/England/NOTT-10E8DC/2020|EPI_ISL_425609|20	....	....	....	....	....	....
A	1205	hCoV-19/England/NOTT-10E8FA/2020|EPI_ISL_425611|20	....	....	....	....	....	....
A	1206	hCoV-19/England/NOTT-10E933/2020|EPI_ISL_425614|20	....	....	....	....	....	....
A	1207	hCoV-19/England/NOTT-10E942/2020|EPI_ISL_425615|20	....	....	....	....	....	....
A	1208	hCoV-19/England/NOTT-10E951/2020|EPI_ISL_425616|20	....	....	....	....	....	....
A	1209	hCoV-19/England/NOTT-10E97F/2020|EPI_ISL_425618|20	....	....	....	....	....	....
A	1210	hCoV-19/England/NOTT-10E9AC/2020|EPI_ISL_425620|20	....	....	....	....	....	....
A	1211	hCoV-19/England/NOTT-10E9BB/2020|EPI_ISL_425621|20	....	....	....	....	....	....
A	1212	hCoV-19/England/NOTT-10E9CA/2020|EPI_ISL_425622|20	....	....	....	....	....	....
A	1213	hCoV-19/England/NOTT-10E9D9/2020|EPI_ISL_425623|20	....	....	....	....	....	....
A	1214	hCoV-19/England/NOTT-10EA8B/2020|EPI_ISL_425633|20	....	....	....	....	....	....
A	1215	hCoV-19/England/NOTT-10EAA9/2020|EPI_ISL_425635|20	....	....	....	....	....	....
A	1216	hCoV-19/England/NOTT-10EAC7/2020|EPI_ISL_425637|20	....	....	....	....	....	....
A	1217	hCoV-19/England/NOTT-10EAD6/2020|EPI_ISL_425638|20	....	....	....	....	....	....
A	1218	hCoV-19/England/NOTT-10EAE5/2020|EPI_ISL_425639|20	....	....	....	....	....	....
A	1219	hCoV-19/England/NOTT-10EAF4/2020|EPI_ISL_425640|20	....	....	....	....	....	....
A	1220	hCoV-19/England/NOTT-10EB1F/2020|EPI_ISL_425641|20	....	....	....	....	....	....
A	1221	hCoV-19/England/NOTT-10EB2E/2020|EPI_ISL_425642|20	....	....	....	....	....	....
A	1222	hCoV-19/England/NOTT-10EB3D/2020|EPI_ISL_425643|20	....	....	....	....	....	....
A	1223	hCoV-19/England/NOTT-10EB6A/2020|EPI_ISL_425644|20	....	....	....	....	....	....
A	1224	hCoV-19/England/NOTT-10EB79/2020|EPI_ISL_425645|20	....	....	....	....	....	....
A	1225	hCoV-19/Scotland/CVR126/2020|EPI_ISL_425669|2020-0	....	....	....	....	....	....
A	1226	hCoV-19/Scotland/CVR128/2020|EPI_ISL_425670|2020-0	....	....	....	....	....	....
A	1227	hCoV-19/Scotland/CVR129/2020|EPI_ISL_425671|2020-0	....	....	....	....	....	....
A	1228	hCoV-19/Scotland/CVR13/2020|EPI_ISL_425672|2020-03	....	....	....	....	....	....

A	1229	hCoV-19/Scotland/CVR131/2020|EPI_ISL_425674|2020-0	....	....	....	....	....	....
A	1230	hCoV-19/Scotland/CVR132/2020|EPI_ISL_425675|2020-0	....	....	....	....	....	....
A	1231	hCoV-19/Scotland/CVR133/2020|EPI_ISL_425676|2020-0	....	....	....	....	....	....
A	1232	hCoV-19/Scotland/CVR134/2020|EPI_ISL_425677|2020-0	....	....	....	....	....	....
A	1233	hCoV-19/Scotland/CVR135/2020|EPI_ISL_425678|2020-0	....	....	....	....	....	....
A	1234	hCoV-19/Scotland/CVR136/2020|EPI_ISL_425679|2020-0	....	....	....	....	....	....
A	1235	hCoV-19/Scotland/CVR15/2020|EPI_ISL_425692|2020-03	....	....	....	....	....	....
A	1236	hCoV-19/Scotland/CVR150/2020|EPI_ISL_425693|2020-0	....	....	....	....	....	....
A	1237	hCoV-19/Scotland/CVR152/2020|EPI_ISL_425695|2020-0	....	....	....	....	....	....
A	1238	hCoV-19/Scotland/CVR153/2020|EPI_ISL_425696|2020-0	....	....	....	....	....	....
A	1239	hCoV-19/Scotland/CVR155/2020|EPI_ISL_425697|2020-0	....	....	....	....	....	....
A	1240	hCoV-19/Scotland/CVR159/2020|EPI_ISL_425698|2020-0	....	....	....	....	....	....
A	1241	hCoV-19/Scotland/CVR16/2020|EPI_ISL_425699|2020-03	....	....	....	....	....	....
A	1252	hCoV-19/Australia/VIC623/2020|EPI_ISL_426927|2020-	....	....	....	....	....	....
A	1256	hCoV-19/Australia/VIC628/2020|EPI_ISL_426932|2020-	....	....	....	....	....	....
A	1258	hCoV-19/Australia/VIC638/2020|EPI_ISL_426934|2020-	....	....	....	....	....	....
A	1259	hCoV-19/Australia/VIC639/2020|EPI_ISL_426935|2020-	....	....	....	....	....	....
A	1260	hCoV-19/Australia/VIC644/2020|EPI_ISL_426940|2020-	....	....	....	....	....	....
A	1261	hCoV-19/Australia/VIC645/2020|EPI_ISL_426941|2020-	....	....	....	....	....	....
A	1262	hCoV-19/Australia/VIC646/2020|EPI_ISL_426942|2020-	....	....	....	....	....	....
A	1263	hCoV-19/Australia/VIC647/2020|EPI_ISL_426943|2020-	....	....	....	....	....	....
A	1265	hCoV-19/Australia/VIC652/2020|EPI_ISL_426948|2020-	....	....	....	....	....	....
A	1267	hCoV-19/Australia/VIC656/2020|EPI_ISL_426952|2020-	....	....	....	....	....	....
A	1272	hCoV-19/Australia/VIC685/2020|EPI_ISL_426980|2020-	....	....	....	....	....	....
A	1275	hCoV-19/Australia/VIC694/2020|EPI_ISL_426986|2020-	....	....	....	....	....	....
A	1276	hCoV-19/Australia/VIC696/2020|EPI_ISL_426988|2020-	....	....	....	....	....	....
A	1277	hCoV-19/Australia/VIC697/2020|EPI_ISL_426989|2020-	....	....	....	....	....	....
A	1278	hCoV-19/Switzerland/GE-HUG-3144/2020|EPI_ISL_42919	....	....	....	....	....	....
A	1279	hCoV-19/Switzerland/GE-HUG-0199-2/2020|EPI_ISL_429	....	.A..................	....	....	....	....
A	1280	hCoV-19/Switzerland/GE-HUG-0636/2020|EPI_ISL_42919	....	....	....	....	....	....
A	1281	hCoV-19/Portugal/CV62/2020|EPI_ISL_413647|2020-03-	....	....	....	....	....	....
A	1282	hCoV-19/Portugal/CV63/2020|EPI_ISL_413648|2020-03-	....	....	....	....	....	....
A	1283	hCoV-19/USA/WA14-UW10/2020|EPI_ISL_413649|2020-03-	....	....	....	....	....	....
A	1284	hCoV-19/USA/WA15-UW11/2020|EPI_ISL_413650|2020-03-	....	....	....	....	....	....
A	1285	hCoV-19/USA/WA16-UW12/2020|EPI_ISL_413651|2020-03-	....	....	....	....	....	....
A	1286	hCoV-19/USA/WA17-UW13/2020|EPI_ISL_413652|2020-03-	....	....	....	....	....	....
A	1287	hCoV-19/USA/WA18-UW14/2020|EPI_ISL_413653|2020-03-	....	....	....	....	....	....
A	1288	hCoV-19/France/HDF-1645/2020|EPI_ISL_418220|2020-0	....	....	....	....	....	....
A	1289	hCoV-19/France/HDF-1813/2020|EPI_ISL_418221|2020-0	....	....	....	....	....	....
A	1290	hCoV-19/France/CVL-2000/2020|EPI_ISL_418222|2020-0	....	....	....	....	....	....
A	1291	hCoV-19/France/HDF-2060/2020|EPI_ISL_418223|2020-0	....	....	....	....	....	....
A	1292	hCoV-19/France/HDF-2150/2020|EPI_ISL_418224|2020-0	....	....	....	....	....	....
A	1293	hCoV-19/France/HDF-2155/2020|EPI_ISL_418225|2020-0	....	....	....	....	....	....
A	1294	hCoV-19/France/HDF-2381/2020|EPI_ISL_418226|2020-0	....	....	....	....	....	....
A	1295	hCoV-19/France/HDF-2393/2020|EPI_ISL_418227|2020-0	....	....	....	....	....	....
A	1296	hCoV-19/France/HDF-2405/2020|EPI_ISL_418228|2020-0	....	....	....	....	....	....
A	1297	hCoV-19/France/IDF-2410/2020|EPI_ISL_418229|2020-0	....	....	....	....	....	....
A	1298	hCoV-19/France/IDF-2420/2020|EPI_ISL_418230|2020-0	....	....	....	....	....	....
A	1299	hCoV-19/France/HDF-2496/2020|EPI_ISL_418231|2020-0	....	....	....	....	....	....
A	1300	hCoV-19/France/IDF-2532/2020|EPI_ISL_418232|2020-0	....	....	....	....	....	....
A	1301	hCoV-19/France/IDF-2533/2020|EPI_ISL_418233|2020-0	....	....	....	....	....	....
A	1302	hCoV-19/France/IDF-2534/2020|EPI_ISL_418234|2020-0	....	....	....	....	....	....
A	1303	hCoV-19/France/IDF-2561/2020|EPI_ISL_418235|2020-0	....	....	....	....	....	....
A	1304	hCoV-19/France/HDF-2586/2020|EPI_ISL_418236|2020-0	....	....	....	....	....	....
A	1305	hCoV-19/France/HDF-2595/2020|EPI_ISL_418237|2020-0	....	....	....	....	....	....
A	1306	hCoV-19/France/HDF-2597/2020|EPI_ISL_418238|2020-0	....	....	....	....	....	....
A	1307	hCoV-19/France/HDF-2601/2020|EPI_ISL_418239|2020-0	....	....	....	....	....	....
A	1308	hCoV-19/France/IDF-2684/2020|EPI_ISL_418240|2020-0	....	....	....	....	....	....
A	1309	hCoV-19/Algeria/G0638_2264/2020|EPI_ISL_418241|202	....	....	....	....	....	....
A	1310	hCoV-19/Algeria/G0640_2265/2020|EPI_ISL_418242|202	....	....	....	....	....	....
A	1311	hCoV-19/Spain/AN-ISCIII-201272/2020|EPI_ISL_418243	....	....	....	....	....	....
A	1312	hCoV-19/Spain/AN-ISCIII-201373/2020|EPI_ISL_418244	....	....	....	....	....	....
A	1313	hCoV-19/Spain/CM-ISCIII-201328/2020|EPI_ISL_418245	....	....	....	....	....	....
A	1314	hCoV-19/Spain/CM-ISCIII-201329/2020|EPI_ISL_418246	....	....	....	....	....	....
A	1315	hCoV-19/Spain/CL-ISCIII-201061/2020|EPI_ISL_418247	....	....	....	....	....	....
A	1316	hCoV-19/Spain/CL-ISCIII-201323/2020|EPI_ISL_418248	....	....	....	....	....	....
A	1317	hCoV-19/Spain/CL-ISCIII-201372/2020|EPI_ISL_418249	....	....	....	....	....	....
A	1318	hCoV-19/Spain/CT-ISCIII-201396/2020|EPI_ISL_418250	....	....	....	....	....	....
A	1319	hCoV-19/Spain/MD-ISCIII-201105/2020|EPI_ISL_418251	....	....	....	....	....	....
A	1320	hCoV-19/Spain/MD-ISCIII-201449/2020|EPI_ISL_418252	....	....	....	....	....	....
A	1321	hCoV-19/Spain/PV-ISCIII-201382/2020|EPI_ISL_418253	....	....	....	....	....	....
A	1322	hCoV-19/Italy/ABR-IZSGC-TE4925/2020|EPI_ISL_418255	....	....	....	....	....	....
A	1323	hCoV-19/Italy/ABR-IZSGC-TE4880/2020|EPI_ISL_418256	....	....	....	....	....	....
A	1324	hCoV-19/Italy/ABR-IZSGC-TE5056/2020|EPI_ISL_418257	....	....	....	....	....	....
A	1325	hCoV-19/Italy/ABR-IZSGC-TE4953/2020|EPI_ISL_418258	....	....	....	....	....	....
A	1327	hCoV-19/Italy/ABR-IZSGC-TE4836/2020|EPI_ISL_418260	....	....	....	....	....	....
A	1328	hCoV-19/Colombia/DC-INS-78390/2020|EPI_ISL_418262|	....	....	....	....	....	....
A	1329	hCoV-19/Greece/10/2020|EPI_ISL_418263|2020-03-18	....	....	....	....	....	....

A	1330	hCoV-19/Greece/12/2020|EPI_ISL_418264|2020-03-18	....	....	....	....	....	....	
A	1331	hCoV-19/Greece/16/2020|EPI_ISL_418265|2020-03-18	....	....	....	....	....	....	
A	1332	hCoV-19/Vietnam/19-02S/2020|EPI_ISL_418267|2020-01	....	....	....	....	....	....	
A	1333	hCoV-19/Vietnam/19-01S/2020|EPI_ISL_418269|2020-01	....	....	....	....	....	....	
A	1334	hCoV-19/Belgium/JL-03044/2020|EPI_ISL_418270|2020-	....	....	....	....	....	....	
A	1338	hCoV-19/England/SHEF-BFDCD/2020|EPI_ISL_418291|202	....	....	....	....	....	....	
A	1339	hCoV-19/England/SHEF-BFDDC/2020|EPI_ISL_418292|202	....	....	....	....	....	....	
A	1340	hCoV-19/England/SHEF-BFDEB/2020|EPI_ISL_418293|202	....	....	....	....	....	....	
A	1341	hCoV-19/England/SHEF-BFDFA/2020|EPI_ISL_418294|202	....	....	....	....	....	....	
A	1342	hCoV-19/England/SHEF-BFE06/2020|EPI_ISL_418295|202	....	....	....	....	....	....	
A	1343	hCoV-19/England/SHEF-BFE15/2020|EPI_ISL_418296|202	....	....	....	....	....	....	
A	1344	hCoV-19/England/SHEF-BFE24/2020|EPI_ISL_418297|202	....	....	....	....	....	....	
A	1345	hCoV-19/England/SHEF-BFE33/2020|EPI_ISL_418298|202	....	....	....	....	....	....	
A	1346	hCoV-19/England/SHEF-BFE42/2020|EPI_ISL_418299|202	....	....	....	....	....	....	
A	1347	hCoV-19/Germany/NW-HHU-33/2020|EPI_ISL_419550|2020	....	....	....	....	....	....	
A	1348	hCoV-19/Germany/NW-HHU-34/2020|EPI_ISL_419551|2020	....	....	....	....	....	....	
A	1349	hCoV-19/Germany/NW-HHU-35/2020|EPI_ISL_419552|2020	....	....	....	....	....	....	
A	1350	hCoV-19/USA/RI_0520/2020|EPI_ISL_419553|2020-02-28	....	....	....	....	....	....	
A	1351	hCoV-19/USA/CA-CDC-2602/2020|EPI_ISL_419554|2020-0	....	....	....	....	....	....	
A	1352	hCoV-19/USA/WA_5030/2020|EPI_ISL_419555|2020-02-27	....	....	....	....	....	....	
A	1353	hCoV-19/USA/GA_2741/2020|EPI_ISL_419556|2020-02-29	....	....	....	....	....	....	
A	1354	hCoV-19/USA/GA_2742/2020|EPI_ISL_419557|2020-02-29	....	....	....	....	....	....	
A	1355	hCoV-19/USA/OR_2656/2020|EPI_ISL_419558|2020-02-27	....	....	....	....	....	....	
A	1356	hCoV-19/USA/FL_5125/2020|EPI_ISL_419559|2020-02-28	....	....	....	....	....	....	
A	1357	hCoV-19/USA/FL_5091/2020|EPI_ISL_419560|2020-02-28	....	....	....	....	....	....	
A	1358	hCoV-19/Luxembourg/LNS0000001/2020|EPI_ISL_419562|	....	....	....	....	....	....	
A	1359	hCoV-19/Luxembourg/LNS0156959/2020|EPI_ISL_419563|	....	....	....	....	....	....	
A	1360	hCoV-19/Luxembourg/LNS0366116/2020|EPI_ISL_419564|	....	....	....	....	....	....	
A	1361	hCoV-19/Luxembourg/LNS0641910/2020|EPI_ISL_419566|	....	....	....	....	....	....	
A	1362	hCoV-19/Luxembourg/LNS0756270/2020|EPI_ISL_419568|	....	....	....	....	....	....	
A	1363	hCoV-19/Luxembourg/LNS0945359/2020|EPI_ISL_419569|	....	....	....	....	....	....	
A	1364	hCoV-19/Luxembourg/LNS1234709/2020|EPI_ISL_419570|	....	....	....	....	....	....	
A	1365	hCoV-19/Luxembourg/LNS1874423/2020|EPI_ISL_419573|	....	....	....	....	....	....	
A	1366	hCoV-19/Luxembourg/LNS2614631/2020|EPI_ISL_419578|	....	....	....	....	....	....	
A	1367	hCoV-19/Luxembourg/LNS2886370/2020|EPI_ISL_419579|	....	....	....	....	....	....	
A	1368	hCoV-19/Luxembourg/LNS2907333/2020|EPI_ISL_419580|	....	....	....	....	....	....	
A	1369	hCoV-19/Luxembourg/LNS3156434/2020|EPI_ISL_419582|	....	....	....	....	....	....	
A	1370	hCoV-19/Luxembourg/LNS3588186/2020|EPI_ISL_419583|	....	....	....	....	....	....	
A	1371	hCoV-19/Luxembourg/LNS3711853/2020|EPI_ISL_419584|	....	....	....	....	....	....	
A	1372	hCoV-19/Luxembourg/LNS3879580/2020|EPI_ISL_419585|	....	....	....	....	....	....	
A	1373	hCoV-19/Luxembourg/LNS4134806/2020|EPI_ISL_419586|	....	....	....	....	....	....	
A	1374	hCoV-19/Luxembourg/LNS4691488/2020|EPI_ISL_419587|	....	....	....	....	....	....	
A	1375	hCoV-19/Luxembourg/LNS4836560/2020|EPI_ISL_419588|	....	....	....	....	....	....	
A	1376	hCoV-19/Luxembourg/LNS4845603/2020|EPI_ISL_419589|	....	...................A	....	....	....	....	1
A	1377	hCoV-19/Luxembourg/LNS5032431/2020|EPI_ISL_419590|	....	....	....	....	....	....	
A	1378	hCoV-19/Luxembourg/LNS5228153/2020|EPI_ISL_419591|	....	....	....	....	....	....	
A	1379	hCoV-19/Luxembourg/LNS5440244/2020|EPI_ISL_419592|	....	....	....	....	....	....	
A	1380	hCoV-19/Luxembourg/LNS6603085/2020|EPI_ISL_419593|	....	....	....	....	....	....	
A	1381	hCoV-19/Luxembourg/LNS7537751/2020|EPI_ISL_419594|	....	....	....	....	....	....	
A	1382	hCoV-19/Luxembourg/LNS7753431/2020|EPI_ISL_419595|	....	....	....	....	....	....	
A	1383	hCoV-19/Luxembourg/LNS7866283/2020|EPI_ISL_419596|	....	....	....	....	....	....	
A	1384	hCoV-19/Luxembourg/LNS7928872/2020|EPI_ISL_419597|	....	....	....	....	....	....	
A	1385	hCoV-19/Luxembourg/LNS8188502/2020|EPI_ISL_419598|	....	....	....	....	....	....	
A	1386	hCoV-19/Luxembourg/LNS8639502/2020|EPI_ISL_419599|	....	....	....	....	....	....	
A	1387	hCoV-19/Australia/WA02/2020|EPI_ISL_420532|2020-03	....	....	....	....	....	....	
A	1388	hCoV-19/Australia/WA03/2020|EPI_ISL_420533|2020-03	....	....	....	....	....	....	
A	1389	hCoV-19/Australia/WA06/2020|EPI_ISL_420534|2020-03	....	....	....	....	....	....	
A	1390	hCoV-19/Australia/WA04/2020|EPI_ISL_420536|2020-03	....	....	....	....	....	....	
A	1391	hCoV-19/Australia/WA07/2020|EPI_ISL_420537|2020-03	....	....	....	....	....	....	
A	1392	hCoV-19/Australia/WA09/2020|EPI_ISL_420538|2020-03	....	....	....	....	....	....	
A	1393	hCoV-19/Australia/WA10/2020|EPI_ISL_420539|2020-03	....	....	....	....	....	....	
A	1394	hCoV-19/Estonia/ChVir2148/2020|EPI_ISL_420540|2020	....	....	....	....	....	....	
A	1395	hCoV-19/Slovenia/808/2020|EPI_ISL_420541|2020-03-0	....	....	....	....	....	....	
A	1396	hCoV-19/India/UN-763/2020|EPI_ISL_420543|2020-03-0	....	....	....	....	....	....	
A	1397	hCoV-19/India/UN-2020763/2020|EPI_ISL_420544|2020	....	....	....	....	....	....	
A	1398	hCoV-19/India/UN-770/2020|EPI_ISL_420545|2020-03-0	....	....	....	....	....	....	
A	1399	hCoV-19/India/UN-2020770/2020|EPI_ISL_420546|2020	....	....	....	....	....	....	
A	1400	hCoV-19/India/UN-772/2020|EPI_ISL_420547|2020-03-0	....	....	....	....	....	....	
A	1401	hCoV-19/India/UN-2020772/2020|EPI_ISL_420548|2020	....	....	....	....	....	....	
A	1402	hCoV-19/India/UN-773/2020|EPI_ISL_420549|2020-03-0	....	....	....	....	....	....	
A	1403	hCoV-19/India/UN-2020773/2020|EPI_ISL_420550|2020	....	....	....	....	....	....	
A	1404	hCoV-19/India/UN-777/2020|EPI_ISL_420551|2020-03-0	....	....	....	....	....	....	
A	1405	hCoV-19/India/UN-2020777/2020|EPI_ISL_420552|2020	....	....	....	....	....	....	
A	1406	hCoV-19/India/UN-781/2020|EPI_ISL_420553|2020-03-0	....	....	....	....	....	....	
A	1407	hCoV-19/India/UN-2020781/2020|EPI_ISL_420554|2020	....	....	....	....	....	....	
A	1408	hCoV-19/India/UN-c32/2020|EPI_ISL_420555|2020-03-0	....	....	....	....	....	....	
A	1409	hCoV-19/India/UN-2020c32/2020|EPI_ISL_420556|2020	....	....	....	....	....	....	
A	1410	hCoV-19/Italy/ABR-IZSGC-TE5166/2020|EPI_ISL_420563	....	....	....	....	....	....	

```
A    1411 hCoV-19/Italy/ABR-IZSGC-TE5472/2020|EPI_ISL_420564    ····················  ····················  ····················  ····················  ····················  ····················
A    1412 hCoV-19/Italy/TE-IZSGC-TE5476/2020|EPI_ISL_420565|    ····················  ····················  ····················  ····················  ····················  ····················
A    1413 hCoV-19/Italy/ABR-IZSGC-TE5512/2020|EPI_ISL_420566    ····················  ····················  ····················  ·····N··············  ····················  ····················
A    1414 hCoV-19/Italy/ABR-IZSGC-TE5780/2020|EPI_ISL_420567    ····················  ····················  ····················  ····················  ····················  ····················
A    1415 hCoV-19/Italy/ABR-IZSGC-6193/2020|EPI_ISL_420568|2    ····················  ····················  ····················  ····················  ····················  ····················
A    1416 hCoV-19/Italy/ABR-IZSGC-TE6195/2020|EPI_ISL_420569    ····················  ····················  ····················  ····················  ····················  ····················
A    1417 hCoV-19/USA/NY-NYUMC55/2020|EPI_ISL_420570|2020-03    ····················  ····················  ····················  ····················  ····················  ····················
A    1418 hCoV-19/USA/NY-NYUMC56/2020|EPI_ISL_420571|2020-03    ····················  ····················  ····················  ····················  ····················  ····················
A    1419 hCoV-19/USA/NY-NYUMC57/2020|EPI_ISL_420572|2020-03    ····················  ····················  ····················  ····················  ····················  ····················
A    1420 hCoV-19/USA/NY-NYUMC58/2020|EPI_ISL_420573|2020-03    ····················  ····················  ····················  ····················  ····················  ····················
A    1421 hCoV-19/USA/NY-NYUMC60/2020|EPI_ISL_420575|2020-03    ····················  ····················  ····················  ····················  ····················  ····················
A    1422 hCoV-19/USA/NY-NYUMC61/2020|EPI_ISL_420576|2020-03    ····················  ····················  ····················  ····················  ····················  ····················
A    1423 hCoV-19/USA/NY-NYUMC62/2020|EPI_ISL_420577|2020-03    ····················  ····················  ····················  ····················  ····················  ····················
A    1424 hCoV-19/USA/NY-NYUMC63/2020|EPI_ISL_420578|2020-03    ····················  ····················  ····················  ····················  ····················  ····················
A    1425 hCoV-19/USA/NY-NYUMC64/2020|EPI_ISL_420579|2020-03    ····················  ····················  ····················  ····················  ····················  ····················
A    1426 hCoV-19/USA/NY-NYUMC65/2020|EPI_ISL_420580|2020-03    ····················  ····················  ····················  ····················  ····················  ····················
A    1427 hCoV-19/USA/NY-NYUMC66/2020|EPI_ISL_420581|2020-03    ····················  ····················  ····················  ····················  ····················  ····················
A    1428 hCoV-19/USA/NY-NYUMC67/2020|EPI_ISL_420582|2020-03    ····················  ····················  ····················  ····················  ····················  ····················
A    1429 hCoV-19/Italy/ABR-IZSGC-TE6222/2020|EPI_ISL_420583    ····················  ····················  ····················  ····················  ····················  ····················
A    1430 hCoV-19/USA/NY-NYUMC69/2020|EPI_ISL_420585|2020-03    ····················  ····················  ····················  ····················  ····················  ····················
A    1431 hCoV-19/USA/NY-NYUMC70/2020|EPI_ISL_420586|2020-03    ····················  ····················  ····················  ····················  ····················  ····················
A    1432 hCoV-19/USA/NY-NYUMC71/2020|EPI_ISL_420587|2020-03    ····················  ····················  ····················  ····················  ····················  ····················
A    1433 hCoV-19/USA/NY-NYUMC72/2020|EPI_ISL_420588|2020-03    ····················  ····················  ····················  ····················  ····················  ····················
A    1434 hCoV-19/USA/NY-NYUMC73/2020|EPI_ISL_420589|2020-03    ····················  ····················  ····················  ····················  ····················  ····················
A    1435 hCoV-19/USA/NY-NYUMC74/2020|EPI_ISL_420590|2020-03    ····················  ····················  ····················  ····················  ····················  ····················
A    1436 hCoV-19/USA/NY-NYUMC75/2020|EPI_ISL_420591|2020-03    ····················  ····················  ····················  ····················  ····················  ····················
A    1437 hCoV-19/England/20139008304/2020|EPI_ISL_421863|20    ····················  ····················  ····················  ····················  ····················  ····················
A    1438 hCoV-19/England/20139008404/2020|EPI_ISL_421864|20    ····················  ····················  ····················  ····················  ····················  ····················
A    1439 hCoV-19/England/20139008704/2020|EPI_ISL_421865|20    ····················  ····················  ····················  ····················  ····················  ····················
A    1440 hCoV-19/England/20139009004/2020|EPI_ISL_421866|20    ····················  ····················  ····················  ····················  ····················  ····················
A    1441 hCoV-19/England/20139009204/2020|EPI_ISL_421867|20    ····················  ····················  ····················  ····················  ····················  ····················
A    1442 hCoV-19/England/20139009504/2020|EPI_ISL_421868|20    ····················  ····················  ····················  ····················  ····················  ····················
A    1445 hCoV-19/England/20139010604/2020|EPI_ISL_421873|20    ····················  ····················  ····················  ····················  ····················  ····················
A    1448 hCoV-19/England/20139020204/2020|EPI_ISL_421879|20    ····················  ····················  ····················  ····················  ····················  ····················
A    1449 hCoV-19/England/20139021104/2020|EPI_ISL_421880|20    ····················  ····················  ····················  ····················  ····················  ····················
A    1450 hCoV-19/England/20139021604/2020|EPI_ISL_421882|20    ····················  ····················  ····················  ····················  ····················  ····················
A    1451 hCoV-19/England/20139022004/2020|EPI_ISL_421883|20    ····················  ····················  ····················  ····················  ····················  ····················
A    1452 hCoV-19/England/20139022204/2020|EPI_ISL_421884|20    ····················  ····················  ····················  ····················  ····················  ····················
A    1453 hCoV-19/England/20139022504/2020|EPI_ISL_421885|20    ····················  ····················  ····················  ····················  ····················  ····N···············
A    1454 hCoV-19/England/20139022704/2020|EPI_ISL_421886|20    ····················  ····················  ····················  ····················  NNN·················  ····················    1
A    1455 hCoV-19/England/20139023504/2020|EPI_ISL_421887|20    ····················  ····················  ····················  ····················  ····················  ····················
A    1457 hCoV-19/England/20139023804/2020|EPI_ISL_421889|20    ····················  ····················  ····················  ····················  ····················  ····················
A    1458 hCoV-19/England/20139024004/2020|EPI_ISL_421890|20    ····················  ····················  ····················  ····················  ····················  ····················
A    1462 hCoV-19/England/20139024804/2020|EPI_ISL_421894|20    ····················  ····················  ····················  ····················  ····················  ····················
A    1464 hCoV-19/England/20139026904/2020|EPI_ISL_421899|20    ····················  ····················  ····················  ····················  ····················  ····················
A    1465 hCoV-19/USA/WA-UW-1420/2020|EPI_ISL_423000|2020-03    ····················  ····················  ····················  ····················  ····················  ····················
A    1466 hCoV-19/USA/WA-UW-1567/2020|EPI_ISL_423001|2020-03    ····················  ····················  ····················  ····················  ····················  ····················
A    1467 hCoV-19/USA/WA-UW-1593/2020|EPI_ISL_423002|2020-03    ····················  ····················  ····················  ····················  ····················  ····················
A    1468 hCoV-19/USA/WA-UW-1583/2020|EPI_ISL_423003|2020-03    ····················  ····················  ····················  ····················  ····················  ····················
A    1469 hCoV-19/USA/WA-UW-1581/2020|EPI_ISL_423004|2020-03    ····················  ····················  ····················  ····················  ····················  ····················
A    1470 hCoV-19/USA/WA-UW-1603/2020|EPI_ISL_423005|2020-03    ····················  ····················  ····················  ····················  ····················  ····················
A    1471 hCoV-19/USA/WA-UW-1611/2020|EPI_ISL_423006|2020-03    ····················  ····················  ····················  ····················  ····················  ····················
A    1472 hCoV-19/USA/WA-UW-1879/2020|EPI_ISL_424300|2020-03    ····················  ····················  ····················  ····················  ····················  ····················
A    1473 hCoV-19/USA/WA-UW-1948/2020|EPI_ISL_424301|2020-03    ····················  ····················  ····················  ····················  ····················  ····················
A    1474 hCoV-19/USA/WA-UW-1890/2020|EPI_ISL_424316|2020-03    ····················  ····················  ····················  ····················  ····················  ····················
A    1475 hCoV-19/USA/WA-UW-1928/2020|EPI_ISL_424317|2020-03    ····················  ····················  ····················  ····················  ····················  ····················
A    1476 hCoV-19/USA/ID-UW-1938/2020|EPI_ISL_424318|2020-03    ····················  ····················  ····················  ····················  ····················  ····················
A    1477 hCoV-19/USA/ID-UW-1935/2020|EPI_ISL_424319|2020-03    ····················  ····················  ····················  ····················  ····················  ····················
A    1478 hCoV-19/USA/ID-UW-1925/2020|EPI_ISL_424321|2020-03    ····················  ····················  ····················  ····················  ····················  ····················
A    1479 hCoV-19/USA/ID-UW-1934/2020|EPI_ISL_424322|2020-03    ····················  ····················  ····················  ····················  ····················  ····················
A    1480 hCoV-19/USA/WA-UW-1944/2020|EPI_ISL_424323|2020-03    ····················  ····················  ····················  ····················  ····················  ····················
A    1481 hCoV-19/USA/WA-UW-1965/2020|EPI_ISL_424324|2020-03    ····················  ····················  ····················  ····················  ····················  ····················
A    1482 hCoV-19/USA/WA-UW-1954/2020|EPI_ISL_424325|2020-03    ····················  ····················  ····················  ····················  ····················  ····················
A    1483 hCoV-19/USA/WA-UW-1968/2020|EPI_ISL_424326|2020-03    ····················  ····················  ····················  ····················  ····················  ····················
A    1484 hCoV-19/USA/WA-UW-1984/2020|EPI_ISL_424329|2020-03    ····················  ····················  ····················  ····················  ····················  ····················
A    1485 hCoV-19/USA/WA-UW-1865/2020|EPI_ISL_424330|2020-03    ····················  ····················  ····················  ····················  ····················  ····················
A    1486 hCoV-19/USA/WA-UW-1907/2020|EPI_ISL_424331|2020-03    ····················  ····················  ····················  ····················  ····················  ····················
A    1487 hCoV-19/USA/WA-UW-1917/2020|EPI_ISL_424332|2020-03    ····················  ····················  ····················  ····················  ····················  ····················
A    1488 hCoV-19/USA/WA-UW-2006/2020|EPI_ISL_424333|2020-03    ····················  ····················  ····················  ····················  ····················  ····················
A    1489 hCoV-19/USA/WA-UW-1994/2020|EPI_ISL_424334|2020-03    ····················  ····················  ····················  ····················  ····················  ····················
A    1490 hCoV-19/USA/WA-UW-1998/2020|EPI_ISL_424335|2020-03    ····················  ····················  ····················  ····················  ····················  ····················
A    1491 hCoV-19/USA/WA-UW-1988/2020|EPI_ISL_424336|2020-03    ····················  ····················  ····················  ····················  ····················  ····················
A    1492 hCoV-19/USA/WA-UW-1977/2020|EPI_ISL_424337|2020-03    ····················  ····················  ····················  ····················  ····················  ····················
A    1493 hCoV-19/England/NOTT-10EA21/2020|EPI_ISL_425627|20    ····················  ····················  ····················  ····················  ····················  ····················
A    1494 hCoV-19/England/NOTT-10EA30/2020|EPI_ISL_425628|20    ····················  ····················  ····················  ····················  ····················  ····················
A    1495 hCoV-19/England/NOTT-10EA5E/2020|EPI_ISL_425630|20    ····················  ····················  ····················  ····················  ····················  ····················
A    1496 hCoV-19/England/NOTT-10EA6D/2020|EPI_ISL_425631|20    ····················  ····················  ····················  ····················  ····················  ····················
A    1497 hCoV-19/England/NOTT-10EA7C/2020|EPI_ISL_425632|20    ····················  ····················  ····················  ····················  ····················  ····················
```

A	1498	hCoV-19/Scotland/CVR100/2020|EPI_ISL_425647|2020-0	····················	····················	·····················	····················	····················	····················
A	1499	hCoV-19/Scotland/CVR101/2020|EPI_ISL_425648|2020-0	····················	····················	·····················	····················	····················	····················
A	1500	hCoV-19/Scotland/CVR103/2020|EPI_ISL_425650|2020-0	····················	····················	·····················	····················	····················	····················
A	1501	hCoV-19/Scotland/CVR104/2020|EPI_ISL_425651|2020-0	····················	····················	·····················	····················	····················	····················
A	1502	hCoV-19/Scotland/CVR106/2020|EPI_ISL_425652|2020-0	····················	····················	·····················	····················	····················	····················
A	1503	hCoV-19/Scotland/CVR107/2020|EPI_ISL_425653|2020-0	····················	····················	·····················	····················	····················	····················
A	1504	hCoV-19/Scotland/CVR108/2020|EPI_ISL_425654|2020-0	····················	····················	·····················	····················	····················	····················
A	1505	hCoV-19/Scotland/CVR110/2020|EPI_ISL_425656|2020-0	····················	····················	·····················	····················	····················	····················
A	1506	hCoV-19/Scotland/CVR112/2020|EPI_ISL_425657|2020-0	····················	····················	·····················	····················	····················	····················
A	1507	hCoV-19/Scotland/CVR113/2020|EPI_ISL_425658|2020-0	····················	····················	·····················	····················	····················	····················
A	1508	hCoV-19/Scotland/CVR114/2020|EPI_ISL_425659|2020-0	····················	····················	·····················	····················	····················	····················
A	1509	hCoV-19/Scotland/CVR115/2020|EPI_ISL_425660|2020-0	····················	····················	·····················	····················	····················	····················
A	1510	hCoV-19/Scotland/CVR118/2020|EPI_ISL_425662|2020-0	····················	····················	·····················	····················	····················	····················
A	1511	hCoV-19/Scotland/CVR12/2020|EPI_ISL_425663|2020-03	····················	····················	·····················	····················	····················	····················
A	1512	hCoV-19/Scotland/CVR125/2020|EPI_ISL_425668|2020-0	····················	····················	·····················	····················	····················	····················
A	1514	hCoV-19/Australia/VIC661/2020|EPI_ISL_426957|2020-	····················	····················	·····················	····················	····················	····················
A	1515	hCoV-19/Australia/VIC662/2020|EPI_ISL_426958|2020-	····················	····················	·····················	····················	····················	····················
A	1517	hCoV-19/Australia/VIC664/2020|EPI_ISL_426960|2020-	····················	····················	·····················	····················	····················	····················
A	1518	hCoV-19/Australia/VIC665/2020|EPI_ISL_426961|2020-	····················	····················	·····················	····················	····················	····················
A	1520	hCoV-19/Australia/VIC667/2020|EPI_ISL_426963|2020-	····················	····················	·····················	····················	····················	····················
A	1521	hCoV-19/Australia/VIC668/2020|EPI_ISL_426964|2020-	····················	····················	·····················	····················	····················	····················
A	1522	hCoV-19/Australia/VIC669/2020|EPI_ISL_426965|2020-	····················	····················	·····················	····················	····················	····················
A	1523	hCoV-19/Australia/VIC672/2020|EPI_ISL_426968|2020-	····················	····················	·····················	····················	····················	····················
A	1525	hCoV-19/Australia/VIC676/2020|EPI_ISL_426971|2020-	····················	····················	·····················	····················	····················	····················
A	1526	hCoV-19/Australia/VIC677/2020|EPI_ISL_426972|2020-	····················	····················	·····················	····················	····················	····················
A	1528	hCoV-19/Australia/VIC679/2020|EPI_ISL_426974|2020-	····················	····················	·····················	····················	····················	····················
A	1529	hCoV-19/Australia/VIC680/2020|EPI_ISL_426975|2020-	····················	····················	·····················	····················	····················	····················
A	1530	hCoV-19/Australia/VIC681/2020|EPI_ISL_426976|2020-	····················	····················	·····················	····················	····················	····················
A	1531	hCoV-19/Australia/VIC682/2020|EPI_ISL_426977|2020-	····················	····················	·····················	····················	····················	····················
A	1535	hCoV-19/Australia/VIC710/2020|EPI_ISL_426997|2020-	····················	····················	·····················	····················	····················	····················
A	1536	hCoV-19/Australia/VIC712/2020|EPI_ISL_426999|2020-	····················	····················	·····················	····················	····················	····················
A	1537	hCoV-19/Wuhan/IPBCAMS-WH-05/2020|EPI_ISL_403928|20	····················	····················	·····················	····················	····················	····················
A	1538	hCoV-19/Wuhan/IPBCAMS-WH-04/2019|EPI_ISL_403929|20	····················	····················	·····················	····················	····················	····················
A	1539	hCoV-19/Guangdong/20SF013/2020|EPI_ISL_403933|2020	····················	····················	·····················	····················	····················	····················
A	1540	hCoV-19/Guangdong/20SF014/2020|EPI_ISL_403934|2020	····················	····················	·····················	····················	····················	····················
A	1541	hCoV-19/Guangdong/20SF025/2020|EPI_ISL_403935|2020	····················	····················	·····················	····················	····················	····················
A	1542	hCoV-19/Guangdong/20SF028/2020|EPI_ISL_403936|2020	····················	····················	·····················	····················	····················	····················
A	1543	hCoV-19/Guangdong/20SF040/2020|EPI_ISL_403937|2020	····················	····················	·····················	····················	····················	····················
A	1544	hCoV-19/USA/AZ1/2020|EPI_ISL_406223|2020-01-22	····················	····················	·····················	····················	····················	····················
A	1545	hCoV-19/Hefei/2/2020|EPI_ISL_412026|2020-02-23	····················	····················	·····················	····················	····················	····················
A	1546	hCoV-19/Hong Kong/VM20001061-2/2020|EPI_ISL_412028	····················	····················	·····················	····················	····················	····················
A	1547	hCoV-19/Hong Kong/VB20024950-2/2020|EPI_ISL_412029	····················	····················	·····················	····················	····················	····················
A	1548	hCoV-19/Hong Kong/VB20026565-2/2020|EPI_ISL_412030	····················	····················	·····················	····················	····················	····················
A	1549	hCoV-19/Guangzhou/GZMU0016/2020|EPI_ISL_414663|202	····················	····················	·····················	····················	····················	····················
A	1550	hCoV-19/Guangzhou/GZMU0030/2020|EPI_ISL_414686|202	····················	····················	·····················	····················	····················	····················
A	1551	hCoV-19/Guangzhou/GZMU0031/2020|EPI_ISL_414687|202	····················	····················	·····················	····················	····················	····················
A	1552	hCoV-19/Guangzhou/GZMU0044/2020|EPI_ISL_414689|202	····················	····················	·····················	····················	····················	····················
A	1553	hCoV-19/Guangzhou/GZMU0047/2020|EPI_ISL_414690|202	····················	····················	·····················	····················	····················	····················
A	1554	hCoV-19/Guangzhou/GZMU0048/2020|EPI_ISL_414691|202	····················	····················	·····················	····················	····················	····················
A	1555	hCoV-19/Guangzhou/GZMU0014/2020|EPI_ISL_414692|202	····················	····················	·····················	····················	····················	····················
A	1556	hCoV-19/Wales/PHW32/2020|EPI_ISL_415920|2020-03-12	····················	····A···············	·····················	····················	····················	····················
A	1557	hCoV-19/USA/WI-GMF-00018/2020|EPI_ISL_418184|2020-	····················	····················	·····················	····················	····················	····················
A	1558	hCoV-19/USA/WI-GMF-00227/2020|EPI_ISL_418186|2020-	····················	····················	·····················	····················	····················	····················
A	1559	hCoV-19/USA/WI-GMF-00228/2020|EPI_ISL_418187|2020-	····················	····················	·····················	····················	····················	····················
A	1560	hCoV-19/USA/WI-GMF-00232/2020|EPI_ISL_418188|2020-	····················	····················	·····················	····················	····················	····················
A	1561	hCoV-19/USA/NY-NYUMC7/2020|EPI_ISL_418192|2020-03-	····················	····················	·····················	····················	····················	····················
A	1562	hCoV-19/USA/NY-NYUMC8/2020|EPI_ISL_418193|2020-03-	····················	····················	·····················	····················	····················	····················
A	1563	hCoV-19/USA/NY-NYUMC11/2020|EPI_ISL_418196|2020-03	····················	····················	·····················	····················	····················	····················
A	1564	hCoV-19/USA/NY-NYUMC12/2020|EPI_ISL_418197|2020-03	····················	····················	·····················	····················	····················	····················
A	1565	hCoV-19/USA/NY-NYUMC13/2020|EPI_ISL_418198|2020-03	····················	····················	·····················	····················	····················	····················
A	1566	hCoV-19/USA/NY-NYUMC14/2020|EPI_ISL_418199|2020-03	····················	····················	·····················	····················	····················	····················
A	1567	hCoV-19/USA/NY-NYUMC15/2020|EPI_ISL_418200|2020-03	····················	····················	·····················	····················	····················	····················
A	1568	hCoV-19/USA/NY-NYUMC18/2020|EPI_ISL_418203|2020-03	····················	····················	·····················	····················	····················	····················
A	1569	hCoV-19/USA/NY-NYUMC19/2020|EPI_ISL_418204|2020-03	····················	····················	·····················	····················	····················	····················
A	1570	hCoV-19/Senegal/003/2020|EPI_ISL_418206|2020-02-28	····················	····················	·····················	····················	····················	····················
A	1571	hCoV-19/Senegal/016/2020|EPI_ISL_418207|2020-03-02	····················	····················	·····················	····················	····················	····················
A	1572	hCoV-19/Senegal/020/2020|EPI_ISL_418208|2020-03-04	····················	····················	·····················	····················	····················	····················
A	1573	hCoV-19/Senegal/026/2020|EPI_ISL_418209|2020-03-03	····················	····················	·····················	····················	····················	····················
A	1574	hCoV-19/Senegal/073/2020|EPI_ISL_418210|2020-03-10	····················	····················	·····················	····················	····················	····················
A	1575	hCoV-19/Senegal/082/2020|EPI_ISL_418211|2020-03-11	····················	····················	·····················	····················	····················	····················
A	1576	hCoV-19/Senegal/087/2020|EPI_ISL_418212|2020-03-11	····················	····················	·····················	····················	····················	····················
A	1577	hCoV-19/Senegal/094/2020|EPI_ISL_418213|2020-03-12	····················	····················	·····················	····················	····················	····················
A	1578	hCoV-19/Senegal/119/2020|EPI_ISL_418215|2020-03-12	····················	····················	·····················	····················	····················	····················
A	1579	hCoV-19/Senegal/136/2020|EPI_ISL_418216|2020-03-13	····················	····················	·····················	····················	····················	····················
A	1580	hCoV-19/Senegal/139/2020|EPI_ISL_418217|2020-03-13	····················	····················	·····················	····················	····················	····················
A	1581	hCoV-19/France/HDF-1465/2020|EPI_ISL_418218|2020-0	····················	····················	·····················	····················	····················	····················
A	1582	hCoV-19/France/BRE-1623/2020|EPI_ISL_418219|2020-0	····················	····················	·····················	····················	····················	····················
A	1585	hCoV-19/England/SHEF-BFD63/2020|EPI_ISL_418286|202	····················	····················	·····················	····················	····················	····················

A	1586 hCoV-19/England/SHEF-BFD72/2020|EPI_ISL_418287|202	····················	····················	····················	····················	····················	····················
A	1587 hCoV-19/England/SHEF-BFD81/2020|EPI_ISL_418288|202	····················	····················	····················	····················	····················	····················
A	1588 hCoV-19/England/SHEF-BFDAF/2020|EPI_ISL_418289|202	····················	····················	····················	····················	····················	····················
A	1589 hCoV-19/England/SHEF-BFDBE/2020|EPI_ISL_418290|202	····················	····················	····················	····················	····················	····················
A	1590 hCoV-19/Wales/PHWC-24D87/2020|EPI_ISL_419508|2020-	····················	····A···············	····················	····················	····················	····················
A	1591 hCoV-19/USA/CT-Yale-013/2020|EPI_ISL_419517|2020-0	····················	····················	····················	····················	····················	····················
A	1592 hCoV-19/USA/CT-Yale-014/2020|EPI_ISL_419518|2020-0	····················	····················	····················	····················	····················	····················
A	1593 hCoV-19/USA/CT-Yale-034/2020|EPI_ISL_419524|2020-0	····················	····················	····················	····················	····················	····················
A	1594 hCoV-19/USA/CT-Yale-036/2020|EPI_ISL_419525|2020-0	····················	····················	····················	····················	····················	····················
A	1595 hCoV-19/USA/CT-Yale-040/2020|EPI_ISL_419528|2020-0	····················	····················	····················	····················	····················	····················
A	1596 hCoV-19/Germany/NW-HHU-24/2020|EPI_ISL_419541|2020	····················	····················	····················	····················	····················	····················
A	1597 hCoV-19/Germany/NW-HHU-25/2020|EPI_ISL_419542|2020	····················	····················	····················	····················	····················	····················
A	1598 hCoV-19/Germany/NW-HHU-26/2020|EPI_ISL_419543|2020	····················	····················	····················	····················	····················	····················
A	1599 hCoV-19/Germany/NW-HHU-27/2020|EPI_ISL_419544|2020	····················	····················	····················	····················	····················	····················
A	1600 hCoV-19/Germany/NW-HHU-28/2020|EPI_ISL_419545|2020	····················	····················	····················	····················	····················	····················
A	1601 hCoV-19/Germany/NW-HHU-29/2020|EPI_ISL_419546|2020	····················	····················	····················	····················	····················	····················
A	1602 hCoV-19/Germany/NW-HHU-30/2020|EPI_ISL_419547|2020	····················	····················	····················	····················	····················	····················
A	1603 hCoV-19/Germany/NW-HHU-31/2020|EPI_ISL_419548|2020	····················	····················	····················	····················	····················	····················
A	1604 hCoV-19/Germany/NW-HHU-32/2020|EPI_ISL_419549|2020	····················	····················	····················	····················	····················	····················
A	1605 hCoV-19/England/20132072802/2020|EPI_ISL_420508|20	····················	····················	····················	····················	····················	····················
A	1606 hCoV-19/England/20136015502/2020|EPI_ISL_420520|20	····················	····················	····················	····················	····················	····················
A	1607 hCoV-19/England/20138012802/2020|EPI_ISL_420521|20	····················	····················	····················	····················	····················	····················
A	1608 hCoV-19/England/20138014402/2020|EPI_ISL_420522|20	····················	····················	····················	····················	····················	····················
A	1609 hCoV-19/England/20139018202/2020|EPI_ISL_420523|20	····················	····················	····················	····················	····················	····················
A	1610 hCoV-19/England/20139018302/2020|EPI_ISL_420524|20	····················	····················	····················	····················	····················	····················
A	1611 hCoV-19/England/20139051702/2020|EPI_ISL_420529|20	····················	····················	····················	····················	····················	····················
A	1612 hCoV-19/Australia/WA01/2020|EPI_ISL_420531|2020-03	····················	····················	····················	····················	····················	····················
A	1613 hCoV-19/Argentina/C3013/2020|EPI_ISL_420598|2020-0	····················	···········A········	····················	····················	····················	····················
A	1614 hCoV-19/Argentina/C1374/2020|EPI_ISL_420599|2020-0	····················	····················	····················	····················	····················	····················
A	1615 hCoV-19/England/201380036/2020|EPI_ISL_421800|2020	····················	····················	····················	····················	····················	····················
A	1616 hCoV-19/England/201380037/2020|EPI_ISL_421801|2020	····················	····················	····················	····················	····················	····················
A	1617 hCoV-19/England/201380040/2020|EPI_ISL_421802|2020	····················	····················	····················	····················	····················	····················
A	1618 hCoV-19/England/201380041/2020|EPI_ISL_421803|2020	····················	····················	····················	····················	····················	····················
A	1619 hCoV-19/England/201380042/2020|EPI_ISL_421804|2020	····················	····················	····················	····················	··NN················	····················
A	1620 hCoV-19/England/201380044/2020|EPI_ISL_421805|2020	····················	····················	····················	····················	····················	····················
A	1621 hCoV-19/England/201380049/2020|EPI_ISL_421806|2020	····················	····················	····················	····················	····················	····················
A	1622 hCoV-19/England/201380050/2020|EPI_ISL_421807|2020	····················	····················	····················	····················	····················	····················
A	1623 hCoV-19/England/201380054/2020|EPI_ISL_421809|2020	····················	····················	····················	····················	····················	····················
A	1624 hCoV-19/England/201380055/2020|EPI_ISL_421810|2020	····················	····················	····················	····················	····················	····················
A	1625 hCoV-19/England/201380056/2020|EPI_ISL_421811|2020	····················	····················	····················	····················	····················	····················
A	1626 hCoV-19/England/201380061/2020|EPI_ISL_421813|2020	···T················	····················	····················	····················	····················	····················
A	1627 hCoV-19/England/201380063/2020|EPI_ISL_421815|2020	····················	····················	····················	····················	····················	····················
A	1628 hCoV-19/England/201380064/2020|EPI_ISL_421816|2020	····················	····················	····················	····················	····················	····················
A	1630 hCoV-19/England/201380070/2020|EPI_ISL_421818|2020	····················	····················	····················	····················	····················	····················
A	1631 hCoV-19/England/201380217/2020|EPI_ISL_421822|2020	····················	····················	····················	····················	····················	····················
A	1632 hCoV-19/England/201380221/2020|EPI_ISL_421823|2020	····················	····················	····················	····················	····················	····················
A	1633 hCoV-19/England/201380228/2020|EPI_ISL_421824|2020	····················	····················	····················	····················	····················	····················
A	1634 hCoV-19/England/201380229/2020|EPI_ISL_421825|2020	····················	····················	····················	····················	····················	····················
A	1635 hCoV-19/England/201380276/2020|EPI_ISL_421826|2020	····················	····················	····················	····················	····················	····················
A	1637 hCoV-19/England/201380280/2020|EPI_ISL_421828|2020	····················	····················	····················	····················	····················	····················
A	1641 hCoV-19/England/20138082004/2020|EPI_ISL_421834|20	····················	····················	····················	····················	····················	····················
A	1642 hCoV-19/England/20138082704/2020|EPI_ISL_421836|20	····················	····················	····················	····················	····················	····N···············
A	1643 hCoV-19/England/20138082804/2020|EPI_ISL_421837|20	····················	····················	····················	····················	····················	····················
A	1644 hCoV-19/England/20138083204/2020|EPI_ISL_421839|20	···T················	····················	····················	····················	····················	····················
A	1646 hCoV-19/England/20138086904/2020|EPI_ISL_421842|20	····················	····················	····················	····················	····················	····················
A	1647 hCoV-19/England/20138088004/2020|EPI_ISL_421845|20	····················	····················	····················	····················	····················	····················
A	1648 hCoV-19/England/20138088204/2020|EPI_ISL_421847|20	····················	····················	····················	····················	····················	····················
A	1649 hCoV-19/England/20139003004/2020|EPI_ISL_421848|20	····················	····················	····················	····················	····················	····················
A	1650 hCoV-19/England/20139003404/2020|EPI_ISL_421850|20	····················	····················	····················	····················	····················	····················
A	1651 hCoV-19/England/20139007204/2020|EPI_ISL_421859|20	····················	····················	····················	····················	····················	····················
A	1652 hCoV-19/England/20139007604/2020|EPI_ISL_421861|20	····················	····················	····················	····················	····················	····················
A	1653 hCoV-19/England/20139008204/2020|EPI_ISL_421862|20	····················	····················	····················	····················	····················	····················
A	1655 hCoV-19/England/20129017104/2020|EPI_ISL_424003|20	····················	····················	····················	····················	····················	··N·················
A	1657 hCoV-19/England/20129018904/2020|EPI_ISL_424007|20	····················	····················	····················	····················	····················	····················
A	1658 hCoV-19/England/20129020004/2020|EPI_ISL_424009|20	····················	····················	····················	····················	····················	····················
A	1659 hCoV-19/England/20129020304/2020|EPI_ISL_424010|20	····················	····················	····················	····················	····················	····················
A	1660 hCoV-19/England/20129020604/2020|EPI_ISL_424012|20	····················	····················	····················	····················	····················	····················
A	1662 hCoV-19/England/20129025404/2020|EPI_ISL_424017|20	····················	····················	····················	····················	····················	····················
A	1664 hCoV-19/England/20129032104/2020|EPI_ISL_424020|20	····················	····················	····················	····················	····················	····················
A	1666 hCoV-19/England/20129033804/2020|EPI_ISL_424024|20	····················	····················	····················	····················	····················	····················
A	1667 hCoV-19/England/20129033904/2020|EPI_ISL_424025|20	····················	····················	····················	····················	····················	····················
A	1668 hCoV-19/England/20129035304/2020|EPI_ISL_424028|20	····················	····················	····················	····················	····················	····················
A	1669 hCoV-19/England/20129035404/2020|EPI_ISL_424029|20	····················	····················	····················	····················	····················	····················
A	1670 hCoV-19/England/20129035804/2020|EPI_ISL_424031|20	····················	····················	····················	····················	····················	····················
A	1671 hCoV-19/England/20129036004/2020|EPI_ISL_424032|20	····················	····················	····················	····················	····················	····················
A	1672 hCoV-19/England/20129036404/2020|EPI_ISL_424034|20	····················	····················	····················	····················	····················	····················
A	1673 hCoV-19/England/20129036504/2020|EPI_ISL_424035|20	····················	····················	····················	····················	····················	····················
A	1674 hCoV-19/England/20129037204/2020|EPI_ISL_424037|20	····················	····················	····················	····················	····················	····················

A	1675	hCoV-19/England/20129037304/2020|EPI_ISL_424038|20	····················	····················	····················	····················	····················	····················
A	1677	hCoV-19/England/20129059204/2020|EPI_ISL_424054|20	····················	····················	····················	····················	····················	····················
A	1679	hCoV-19/England/20129085704/2020|EPI_ISL_424076|20	····················	····················	····················	····················	····················	····················
A	1680	hCoV-19/England/20129089504/2020|EPI_ISL_424078|20	····················	····················	····················	····················	····················	····················
A	1681	hCoV-19/England/20129096604/2020|EPI_ISL_424082|20	····················	····················	····················	····················	····················	····················
A	1682	hCoV-19/England/20129096804/2020|EPI_ISL_424084|20	····················	····················	····················	····················	····················	····················
A	1684	hCoV-19/England/CAMB-74571/2020|EPI_ISL_425307|202	····················	····················	····················	····················	····················	····················
A	1685	hCoV-19/England/CAMB-74580/2020|EPI_ISL_425308|202	····················	····················	····················	····················	····················	····················
A	1686	hCoV-19/England/CAMB-7459F/2020|EPI_ISL_425309|202	····················	····················	····················	····················	····················	····················
A	1687	hCoV-19/England/CAMB-745AE/2020|EPI_ISL_425310|202	····················	····················	····················	····················	····················	····················
A	1688	hCoV-19/England/CAMB-745BD/2020|EPI_ISL_425311|202	····················	····················	····················	····················	····················	····················
A	1689	hCoV-19/England/CAMB-745CC/2020|EPI_ISL_425312|202	····················	····················	····················	····················	····················	····················
A	1690	hCoV-19/England/CAMB-745DB/2020|EPI_ISL_425313|202	····················	····················	····················	····················	····················	····················
A	1691	hCoV-19/England/CAMB-745EA/2020|EPI_ISL_425314|202	····················	····················	····················	····················	····················	····················
A	1692	hCoV-19/England/CAMB-74605/2020|EPI_ISL_425315|202	····················	····················	····················	····················	····················	····················
A	1693	hCoV-19/England/CAMB-74614/2020|EPI_ISL_425316|202	····················	····················	····················	····················	····················	····················
A	1694	hCoV-19/England/CAMB-74623/2020|EPI_ISL_425317|202	····················	····················	····················	····················	····················	····················
A	1695	hCoV-19/England/CAMB-74641/2020|EPI_ISL_425318|202	····················	····················	····················	····················	····················	····················
A	1696	hCoV-19/England/CAMB-74650/2020|EPI_ISL_425319|202	····················	····················	····················	····················	····················	····················
A	1700	hCoV-19/England/CAMB-746BA/2020|EPI_ISL_425324|202	····················	····················	····················	····················	····················	····················
A	1701	hCoV-19/England/CAMB-746D8/2020|EPI_ISL_425325|202	····················	····················	····················	····················	····················	····················
A	1702	hCoV-19/England/CAMB-74702/2020|EPI_ISL_425326|202	····················	····················	····················	····················	····················	····················
A	1703	hCoV-19/England/CAMB-74711/2020|EPI_ISL_425327|202	····················	····················	····················	····················	····················	····················
A	1704	hCoV-19/England/CAMB-7475D/2020|EPI_ISL_425331|202	····················	····················	····················	····················	····················	····················
A	1705	hCoV-19/England/CAMB-7476C/2020|EPI_ISL_425332|202	····················	····················	····················	····················	····················	····················
A	1706	hCoV-19/England/CAMB-7477B/2020|EPI_ISL_425333|202	···T················	····················	····················	····················	····················	····················
A	1707	hCoV-19/England/CAMB-748E1/2020|EPI_ISL_425335|202	····················	····················	····················	····················	····················	····················
A	1708	hCoV-19/England/CAMB-7491B/2020|EPI_ISL_425338|202	····················	····················	····················	····················	····················	····················
A	1709	hCoV-19/England/CAMB-7492A/2020|EPI_ISL_425339|202	····················	····················	····················	····················	····················	····················
A	1711	hCoV-19/England/CAMB-74B42/2020|EPI_ISL_425348|202	····················	····················	····················	····················	····················	NN··················
A	1715	hCoV-19/England/CAMB-74C30/2020|EPI_ISL_425359|202	····················	····················	····················	····················	····················	····················
A	1716	hCoV-19/England/CAMB-74C5E/2020|EPI_ISL_425361|202	····················	····················	····················	····················	····················	····················
A	1717	hCoV-19/England/CAMB-74C8B/2020|EPI_ISL_425362|202	····················	····················	····················	····················	····················	····················
A	1718	hCoV-19/England/CAMB-74D5B/2020|EPI_ISL_425373|202	····················	····················	····················	····················	····················	····················
A	1719	hCoV-19/England/CAMB-74D6A/2020|EPI_ISL_425374|202	····················	····················	····················	····················	····················	····················
A	1720	hCoV-19/England/CAMB-74E49/2020|EPI_ISL_425378|202	····················	····················	····················	····················	····················	····················
A	1721	hCoV-19/England/CAMB-74E67/2020|EPI_ISL_425380|202	····················	····················	····················	····················	····················	····················
A	1722	hCoV-19/England/CAMB-74E85/2020|EPI_ISL_425382|202	····················	····················	····················	····················	····················	····················
A	1728	hCoV-19/Australia/VIC327/2020|EPI_ISL_426637|2020-	····················	····················	····················	····················	····················	····················
A	1729	hCoV-19/Australia/VIC328/2020|EPI_ISL_426638|2020-	····················	····················	····················	····················	····················	····················
A	1730	hCoV-19/Australia/VIC329/2020|EPI_ISL_426639|2020-	····················	····················	····················	····················	····················	····················
A	1731	hCoV-19/Australia/VIC330/2020|EPI_ISL_426640|2020-	····················	····················	····················	····················	····················	····················
A	1732	hCoV-19/Australia/VIC332/2020|EPI_ISL_426641|2020-	····················	····················	····················	····················	····················	····················
A	1733	hCoV-19/Australia/VIC333/2020|EPI_ISL_426642|2020-	····················	····················	····················	····················	····················	····················
A	1734	hCoV-19/Australia/VIC334/2020|EPI_ISL_426643|2020-	····················	····················	····················	····················	····················	····················
A	1735	hCoV-19/Australia/VIC335/2020|EPI_ISL_426644|2020-	····················	····················	····················	····················	····················	····················
A	1736	hCoV-19/Australia/VIC336/2020|EPI_ISL_426645|2020-	····················	····················	····················	····················	····················	····················
A	1737	hCoV-19/Australia/VIC337/2020|EPI_ISL_426646|2020-	····················	····················	····················	····················	····················	····················
A	1738	hCoV-19/Australia/VIC338/2020|EPI_ISL_426647|2020-	····················	····················	····················	····················	····················	····················
A	1739	hCoV-19/Australia/VIC339/2020|EPI_ISL_426648|2020-	····················	····················	····················	····················	····················	····················
A	1740	hCoV-19/Australia/VIC340/2020|EPI_ISL_426649|2020-	····················	····················	····················	····················	····················	····················
A	1741	hCoV-19/Australia/VIC341/2020|EPI_ISL_426650|2020-	····················	·A··················	····················	····················	····················	····················
A	1742	hCoV-19/Australia/VIC342/2020|EPI_ISL_426651|2020-	····················	····················	····················	····················	····················	····················
A	1743	hCoV-19/Australia/VIC343/2020|EPI_ISL_426652|2020-	····················	····················	····················	····················	····················	····················
A	1744	hCoV-19/Australia/VIC344/2020|EPI_ISL_426653|2020-	····················	····················	····················	····················	····················	····················
A	1745	hCoV-19/Australia/VIC345/2020|EPI_ISL_426654|2020-	····················	····················	····················	····················	····················	····················
A	1746	hCoV-19/Australia/VIC346/2020|EPI_ISL_426655|2020-	····················	····················	····················	····················	····················	····················
A	1747	hCoV-19/Australia/VIC347/2020|EPI_ISL_426656|2020-	····················	····················	····················	····················	····················	····················
A	1748	hCoV-19/Australia/VIC348/2020|EPI_ISL_426657|2020-	····················	····················	····················	····················	····················	····················
A	1749	hCoV-19/Australia/VIC350/2020|EPI_ISL_426658|2020-	····················	····················	····················	····················	····················	····················
A	1750	hCoV-19/Australia/VIC351/2020|EPI_ISL_426659|2020-	····················	····················	····················	····················	····················	····················
A	1751	hCoV-19/Australia/VIC353/2020|EPI_ISL_426661|2020-	····················	····················	····················	····················	····················	····················
A	1752	hCoV-19/Australia/VIC354/2020|EPI_ISL_426662|2020-	····················	····················	····················	····················	····················	····················
A	1753	hCoV-19/Australia/VIC355/2020|EPI_ISL_426663|2020-	····················	····················	····················	····················	····················	····················
A	1754	hCoV-19/Australia/VIC356/2020|EPI_ISL_426664|2020-	····················	····················	····················	····················	····················	····················
A	1755	hCoV-19/Australia/VIC357/2020|EPI_ISL_426665|2020-	····················	····················	····················	····················	····················	····················
A	1756	hCoV-19/Australia/VIC358/2020|EPI_ISL_426666|2020-	····················	····················	····················	····················	····················	····················
A	1757	hCoV-19/Australia/VIC359/2020|EPI_ISL_426667|2020-	····················	····················	····················	····················	····················	····················
A	1758	hCoV-19/Australia/VIC361/2020|EPI_ISL_426669|2020-	····················	····················	····················	····················	····················	····················
A	1759	hCoV-19/Australia/VIC362/2020|EPI_ISL_426670|2020-	····················	····················	····················	····················	····················	····················
A	1760	hCoV-19/Australia/VIC363/2020|EPI_ISL_426671|2020-	····················	····················	····················	····················	····················	····················
A	1761	hCoV-19/Australia/VIC364/2020|EPI_ISL_426672|2020-	····················	····················	····················	····················	····················	····················
A	1762	hCoV-19/Australia/VIC365/2020|EPI_ISL_426673|2020-	····················	····················	····················	····················	····················	····················
A	1763	hCoV-19/Australia/VIC366/2020|EPI_ISL_426674|2020-	····················	····················	····················	····················	····················	····················
A	1764	hCoV-19/Australia/VIC367/2020|EPI_ISL_426675|2020-	····················	····················	····················	····················	····················	····················
A	1765	hCoV-19/Australia/VIC368/2020|EPI_ISL_426676|2020-	····················	····················	····················	····················	····················	····················
A	1766	hCoV-19/Australia/VIC369/2020|EPI_ISL_426677|2020-	····················	····················	····················	····················	····················	····················
A	1767	hCoV-19/Australia/VIC372/2020|EPI_ISL_426679|2020-	····················	····················	····················	····················	····················	····················

A 1768 hCoV-19/Australia/VIC373/2020|EPI_ISL_426680|2020- ···················· ···················· ···················· ···················· ···················· ····················
A 1769 hCoV-19/Australia/VIC375/2020|EPI_ISL_426682|2020- ···················· ···················· ···················· ···················· ···················· ····················
A 1770 hCoV-19/Australia/VIC376/2020|EPI_ISL_426683|2020- ···················· ···················· ···················· ···················· ···················· ····················
A 1771 hCoV-19/Australia/VIC377/2020|EPI_ISL_426684|2020- ···················· ···················· ···················· ···················· ···················· ····················
A 1772 hCoV-19/Australia/VIC378/2020|EPI_ISL_426685|2020- ···················· ···················· ···················· ···················· ···················· ····················
A 1773 hCoV-19/Australia/VIC379/2020|EPI_ISL_426686|2020- ···················· ···················· ···················· ···················· ···················· ····················
A 1774 hCoV-19/Australia/VIC380/2020|EPI_ISL_426687|2020- ···················· ···················· ···················· ···················· ···················· ····················
A 1775 hCoV-19/Australia/VIC381/2020|EPI_ISL_426688|2020- ···················· ···················· ···················· ···················· ···················· ····················
A 1776 hCoV-19/Australia/VIC382/2020|EPI_ISL_426689|2020- ···················· ···················· ···················· ···················· ···················· ····················
A 1777 hCoV-19/Australia/VIC383/2020|EPI_ISL_426690|2020- ···················· ···················· ···················· ···················· ···················· ····················
A 1778 hCoV-19/Australia/VIC384/2020|EPI_ISL_426691|2020- ···················· ···················· ···················· ···················· ···················· ····················
A 1779 hCoV-19/Australia/VIC385/2020|EPI_ISL_426692|2020- ···················· ···················· ···················· ···················· ···················· ····················
A 1780 hCoV-19/Australia/VIC386/2020|EPI_ISL_426693|2020- ···················· ···················· ···················· ···················· ···················· ····················
A 1781 hCoV-19/Australia/VIC387/2020|EPI_ISL_426694|2020- ···················· ···················· ···················· ···················· ···················· ····················
A 1782 hCoV-19/Australia/VIC389/2020|EPI_ISL_426696|2020- ···················· ···················· ···················· ···················· ···················· ····················
A 1783 hCoV-19/Australia/VIC394/2020|EPI_ISL_426697|2020- ···················· ···················· ···················· ···················· ···················· ····················
A 1784 hCoV-19/Australia/VIC395/2020|EPI_ISL_426698|2020- ···················· ···················· ···················· ···················· ···················· ····················
A 1785 hCoV-19/Australia/VIC396/2020|EPI_ISL_426699|2020- ···················· ···················· ···················· ···················· ···················· ····················
A 1786 hCoV-19/India/DL-NIV-763/2020|EPI_ISL_547870|2020 ···················· ···················· ···················· ···················· ···················· ····················
A 1787 hCoV-19/India/DL-NIV-770/2020|EPI_ISL_547871|2020 ···················· ···················· ···················· ···················· ···················· ····················
A 1788 hCoV-19/India/DL-NIV-772/2020|EPI_ISL_547872|2020 ···················· ···················· ···················· ···················· ···················· ····················
A 1789 hCoV-19/India/DL-NIV-773/2020|EPI_ISL_547873|2020 ···················· ···················· ···················· ···················· ···················· ····················
A 1790 hCoV-19/India/DL-NIV-777/2020|EPI_ISL_547874|2020 ···················· ···················· ···················· ···················· ···················· ····················
A 1791 hCoV-19/India/DL-NIV-781/2020|EPI_ISL_547875|2020 ···················· ···················· ···················· ···················· ···················· ····················
A 1792 hCoV-19/India/UP-NIV-32/2020|EPI_ISL_547876|2020 ···················· ···················· ···················· ···················· ···················· ····················
A 1793 hCoV-19/Wuhan/IPBCAMS-WH-03/2019|EPI_ISL_403930|20 ···················· ···················· ···················· ···················· ···················· ····················
A 1794 hCoV-19/Wuhan/IPBCAMS-WH-02/2019|EPI_ISL_403931|20 ···················· ···················· ···················· ···················· ···················· ····················
A 1795 hCoV-19/Guangdong/20SF012/2020|EPI_ISL_403932|2020 ···················· ···················· ···················· ···················· ···················· ····················
A 1796 hCoV-19/Thailand/61/2020|EPI_ISL_403962|2020-01-08 ···················· ···················· ···················· ···················· ···················· ····················
A 1797 hCoV-19/Thailand/74/2020|EPI_ISL_403963|2020-01-13 ···················· ···················· ···················· ···················· ···················· ····················
A 1798 hCoV-19/France/GES-1583/2020|EPI_ISL_414600|2020-0 ···················· ···················· ···················· ···················· ···················· ····················
A 1799 hCoV-19/USA/WA-UW29/2020|EPI_ISL_414616|2020-03-08 ···················· ···················· ···················· ···················· ···················· ····················
A 1800 hCoV-19/USA/WA-UW30/2020|EPI_ISL_414617|2020-03-08 ···················· ···················· ···················· ···················· ···················· ····················
A 1801 hCoV-19/USA/WA-UW31/2020|EPI_ISL_414618|2020-03-08 ···················· ···················· ···················· ···················· ···················· ····················
A 1802 hCoV-19/USA/WA-UW32/2020|EPI_ISL_414619|2020-03-07 ···················· ···················· ···················· ···················· ···················· ····················
A 1803 hCoV-19/USA/WA-UW33/2020|EPI_ISL_414620|2020-03-08 ···················· ···················· ···················· ···················· ···················· ····················
A 1804 hCoV-19/USA/WA-UW34/2020|EPI_ISL_414621|2020-03-07 ···················· ···················· ···················· ···················· ···················· ····················
A 1805 hCoV-19/USA/WA-UW35/2020|EPI_ISL_414622|2020-03-08 ···················· ···················· ···················· ···················· ···················· ····················
A 1806 hCoV-19/France/GES-1583-2/2020|EPI_ISL_414623|2020 ···················· ···················· ···················· ···················· ···················· ····················
A 1807 hCoV-19/France/NOR-N1620/2020|EPI_ISL_414624|2020- ···················· ···················· ···················· ···················· ···················· ····················
A 1808 hCoV-19/France/PDL-1643/2020|EPI_ISL_414625|2020-0 ···················· ···················· ···················· ···················· ···················· ····················
A 1809 hCoV-19/France/HDF-1684/2020|EPI_ISL_414626|2020-0 ···················· ···················· ···················· ···················· ···················· ····················
A 1810 hCoV-19/France/HDF-1795/2020|EPI_ISL_414627|2020-0 ···················· ···················· ···················· ···················· ···················· ····················
A 1811 hCoV-19/France/HDF-1805/2020|EPI_ISL_414628|2020-0 ···················· ···················· ···················· ···················· ···················· ····················
A 1812 hCoV-19/France/HDF-1870/2020|EPI_ISL_414629|2020-0 ···················· ···················· ···················· ···················· ···················· ····················
A 1813 hCoV-19/France/HDF-1871/2020|EPI_ISL_414630|2020-0 ···················· ···················· ···················· ···················· ···················· ····················
A 1814 hCoV-19/France/GES-1973/2020|EPI_ISL_414631|2020-0 ···················· ···················· ···················· ···················· ···················· ····················
A 1815 hCoV-19/France/GES-1977/2020|EPI_ISL_414632|2020-0 ···················· ···················· ···················· ···················· ···················· ····················
A 1816 hCoV-19/France/IDF-1980/2020|EPI_ISL_414633|2020-0 ···················· ···················· ···················· ···················· ···················· ····················
A 1817 hCoV-19/France/HDF-1986/2020|EPI_ISL_414634|2020-0 ···················· ···················· ···················· ···················· ···················· ····················
A 1818 hCoV-19/France/HDF-1988/2020|EPI_ISL_414635|2020-0 ···················· ···················· ···················· ···················· ···················· ····················
A 1819 hCoV-19/France/HDF-1989/2020|EPI_ISL_414636|2020-0 ···················· ···················· ···················· ···················· ···················· ····················
A 1820 hCoV-19/France/HDF-1993/2020|EPI_ISL_414637|2020-0 ···················· ···················· ···················· ···················· ···················· ····················
A 1821 hCoV-19/France/HDF-1995/2020|EPI_ISL_414638|2020-0 ···················· ···················· ···················· ···················· ···················· ····················
A 1822 hCoV-19/Finland/FIN-313/2020|EPI_ISL_414641|2020-0 ···················· ···················· ···················· ···················· ···················· ····················
A 1823 hCoV-19/Finland/FIN-455/2020|EPI_ISL_414642|2020-0 ···················· ···················· ···················· ···················· ···················· ····················
A 1824 hCoV-19/Finland/FIN-508/2020|EPI_ISL_414643|2020-0 ···················· ···················· ···················· ···················· ···················· ····················
A 1825 hCoV-19/Finland/FIN-266/2020|EPI_ISL_414646|2020-0 ···················· ···················· ···················· ···················· ···················· ····················
A 1826 hCoV-19/USA/CA-PC101P/2020|EPI_ISL_414648|2020-03- ···················· ···················· ···················· ···················· ···················· ····················
A 1827 hCoV-19/Spain/MD-ISCIII-201442/2020|EPI_ISL_417010 ···················· ···················· ···················· ···················· ···················· ····················
A 1830 hCoV-19/Belgium/ULG-6216/2020|EPI_ISL_417014|2020- ···················· ···················· ···················· ···················· ···················· ····················
A 1831 hCoV-19/Belgium/ULG-6457/2020|EPI_ISL_417015|2020- ···················· ···················· ···················· ···················· ···················· ····················
A 1832 hCoV-19/Belgium/ULG-6503/2020|EPI_ISL_417016|2020- ···················· ···················· ···················· ···················· ···················· ····················
A 1833 hCoV-19/Belgium/ULG-6638/2020|EPI_ISL_417017|2020- ···················· ···················· ···················· ···················· ···················· ····················
A 1834 hCoV-19/Belgium/ULG-6670/2020|EPI_ISL_417018|2020- ···················· ···················· ···················· ···················· ···················· ····················
A 1835 hCoV-19/Belgium/ULG-6754/2020|EPI_ISL_417019|2020- ···················· ···················· ···················· ···················· ···················· ····················
A 1836 hCoV-19/Belgium/ULG-6939/2020|EPI_ISL_417020|2020- ···················· ···················· ···················· ···················· ···················· ····················
A 1837 hCoV-19/Belgium/ULG-6942/2020|EPI_ISL_417021|2020- ···················· ··········A········· ···················· ···················· ···················· ····················
A 1838 hCoV-19/Belgium/ULG-6948/2020|EPI_ISL_417022|2020- ···················· ··········A········· ···················· ···················· ···················· ····················
A 1839 hCoV-19/Belgium/ULG-6950/2020|EPI_ISL_417023|2020- ···················· ··········A········· ···················· ···················· ···················· ····················
A 1840 hCoV-19/Belgium/ULG-7019/2020|EPI_ISL_417025|2020- ···················· ···················· ···················· ···················· ···················· ····················
A 1841 hCoV-19/USA/UT-08/2020|EPI_ISL_417026|2020-03-20 ···················· ···················· ···················· ···················· ···················· ····················
A 1842 hCoV-19/USA/UT-09/2020|EPI_ISL_417027|2020-03-20 ···················· ···················· ···················· ···················· ···················· ····················
A 1843 hCoV-19/USA/UT-020/2020|EPI_ISL_417028|2020-03-20 ···················· ···················· ···················· ···················· ···················· ····················
A 1844 hCoV-19/Australia/NSW04/2020|EPI_ISL_417030|2020-0 ···················· ···················· ···················· ···················· ···················· ····················
A 1845 hCoV-19/Australia/QLDID919/2020|EPI_ISL_417031|202 ···················· ···················· ···················· ···················· ···················· ····················
A 1846 hCoV-19/Australia/QLDID920/2020|EPI_ISL_417032|202 ···················· ···················· ···················· ···················· ···················· ····················
A 1847 hCoV-19/Australia/QLDID921/2020|EPI_ISL_417033|202 ···················· ···················· ···················· ···················· ···················· ····················

A	1848	hCoV-19/Brazil/AM-02/2020|EPI_ISL_417034|2020-03-1	····················	····················	····················	····················	····················	····················
A	1849	hCoV-19/Hong Kong/VB20017970-2/2020|EPI_ISL_417064	····················	····················	····················	····················	····················	····················
A	1850	hCoV-19/USA/WA-S12/2020|EPI_ISL_417065|2020-03-03	····················	····················	····················	····················	····················	····················
A	1851	hCoV-19/USA/WA-S13/2020|EPI_ISL_417066|2020-03-03	····················	····················	····················	····················	····················	····················
A	1852	hCoV-19/USA/WA-S15/2020|EPI_ISL_417068|2020-03-02	····················	····················	····················	····················	····················	····················
A	1853	hCoV-19/USA/WA-S16/2020|EPI_ISL_417069|2020-03-03	····················	····················	····················	····················	····················	····················
A	1854	hCoV-19/USA/WA-S32/2020|EPI_ISL_417085|2020-03-02	····················	····················	····················	····················	····················	····················
A	1855	hCoV-19/USA/WA-S33/2020|EPI_ISL_417086|2020-03-01	····················	····················	····················	····················	····················	····················
A	1856	hCoV-19/USA/WA-S34/2020|EPI_ISL_417087|2020-03-02	····················	····················	····················	····················	····················	····················
A	1857	hCoV-19/USA/WA-S35/2020|EPI_ISL_417088|2020-03-02	····················	····················	····················	····················	····················	····················
A	1858	hCoV-19/USA/WA-S36/2020|EPI_ISL_417089|2020-03-02	····················	····················	····················	····················	····················	····················
A	1859	hCoV-19/Hungary/mbl2/2020|EPI_ISL_418183|2020-03-1	····················	····················	····················	····················	····················	····················
A	1860	hCoV-19/Canada/ON-PHL-1083/2020|EPI_ISL_418340|202	····················	····················	····················	····················	····················	····················
A	1861	hCoV-19/Canada/ON-PHL-0142/2020|EPI_ISL_418341|202	····················	····················	····················	····················	····················	····················
A	1862	hCoV-19/Canada/ON-PHL-0178/2020|EPI_ISL_418342|202	····················	····················	····················	····················	····················	····················
A	1863	hCoV-19/Canada/ON-PHL-6884/2020|EPI_ISL_418343|202	····················	····················	····················	····················	····················	····················
A	1864	hCoV-19/Canada/ON-PHL-2259/2020|EPI_ISL_418344|202	····················	····················	····················	····················	····················	····················
A	1865	hCoV-19/Canada/ON-PHL-8751/2020|EPI_ISL_418345|202	····················	····················	····················	····················	····················	····················
A	1866	hCoV-19/Canada/ON-PHL-0743/2020|EPI_ISL_418346|202	····················	····················	····················	····················	····················	····················
A	1867	hCoV-19/Canada/ON-PHL-3741/2020|EPI_ISL_418347|202	····················	····················	····················	····················	····················	····················
A	1868	hCoV-19/Canada/ON-PHL-3680/2020|EPI_ISL_418348|202	····················	····················	····················	····················	····················	····················
A	1869	hCoV-19/Canada/ON-PHL-3650/2020|EPI_ISL_418349|202	····················	····················	····················	····················	····················	····················
A	1870	hCoV-19/Canada/ON-PHL-3380/2020|EPI_ISL_418350|202	····················	····················	····················	····················	····················	····················
A	1871	hCoV-19/Canada/ON-PHL-3575/2020|EPI_ISL_418351|202	····················	····················	····················	····················	····················	····················
A	1872	hCoV-19/Canada/ON-PHL-5672/2020|EPI_ISL_418352|202	····················	····················	····················	····················	····················	····················
A	1873	hCoV-19/Canada/ON-PHL-0539/2020|EPI_ISL_418353|202	····················	····················	····················	····················	····················	····················
A	1874	hCoV-19/Canada/ON-PHL-4088/2020|EPI_ISL_418354|202	····················	····················	····················	····················	····················	····················
A	1875	hCoV-19/Canada/ON-PHL-U8150/2020|EPI_ISL_418355|20	····················	····················	····················	····················	····················	····················
A	1876	hCoV-19/Canada/ON-PHL-6980/2020|EPI_ISL_418356|202	····················	····················	····················	····················	····················	····················
A	1877	hCoV-19/Canada/ON-PHL-H6415/2020|EPI_ISL_418357|20	····················	····················	····················	····················	····················	····················
A	1878	hCoV-19/Canada/ON-PHL-5756/2020|EPI_ISL_418358|202	····················	····················	····················	····················	····················	··A·················
A	1879	hCoV-19/Canada/ON-PHL-5757/2020|EPI_ISL_418359|202	····················	····················	····················	····················	····················	··A·················
A	1880	hCoV-19/Canada/ON-PHL-3692/2020|EPI_ISL_418360|202	····················	····················	····················	····················	····················	····················
A	1881	hCoV-19/Canada/ON-PHL-1095/2020|EPI_ISL_418361|202	····················	····················	····················	····················	····················	····················
A	1882	hCoV-19/Canada/ON-PHL-3536/2020|EPI_ISL_418362|202	····················	····················	····················	····················	····················	····················
A	1883	hCoV-19/Canada/ON-PHL-3695/2020|EPI_ISL_418363|202	····················	····················	····················	····················	····················	····················
A	1884	hCoV-19/Canada/ON-PHL-6922/2020|EPI_ISL_418364|202	····················	····················	····················	····················	····················	····················
A	1885	hCoV-19/Canada/ON-PHL-0977/2020|EPI_ISL_418365|202	····················	····················	····················	····················	····················	····················
A	1886	hCoV-19/Canada/ON-PHL-3350/2020|EPI_ISL_418366|202	····················	····················	····················	····················	····················	····················
A	1887	hCoV-19/Canada/ON-PHL-7513/2020|EPI_ISL_418367|202	····················	····················	····················	····················	····················	····················
A	1888	hCoV-19/Canada/ON-PHL-3458/2020|EPI_ISL_418368|202	····················	····················	····················	····················	····················	····················
A	1889	hCoV-19/Canada/ON-PHL-8539/2020|EPI_ISL_418369|202	····················	····················	····················	····················	····················	····················
A	1890	hCoV-19/Canada/ON-PHL-0654/2020|EPI_ISL_418370|202	····················	····················	····················	····················	····················	····················
A	1891	hCoV-19/Canada/ON-PHL-2653/2020|EPI_ISL_418371|202	····················	····················	····················	····················	····················	····················
A	1892	hCoV-19/Canada/ON-PHL-8458/2020|EPI_ISL_418372|202	····················	····················	····················	····················	····················	····················
A	1893	hCoV-19/Canada/ON-PHL-7590/2020|EPI_ISL_418373|202	····················	····················	····················	····················	····················	····················
A	1894	hCoV-19/Canada/ON-PHL-4232/2020|EPI_ISL_418374|202	····················	····················	····················	····················	····················	····················
A	1895	hCoV-19/Canada/ON-PHL-0141/2020|EPI_ISL_418375|202	····················	····················	····················	····················	····················	····················
A	1896	hCoV-19/Canada/ON-PHL-3459/2020|EPI_ISL_418376|202	····················	····················	····················	····················	····················	····················
A	1897	hCoV-19/Canada/ON-PHL-1898/2020|EPI_ISL_418377|202	····················	····················	····················	····················	····················	····················
A	1898	hCoV-19/Canada/ON-PHL-0976/2020|EPI_ISL_418378|202	····················	····················	····················	····················	····················	····················
A	1899	hCoV-19/Canada/ON-PHL-7512/2020|EPI_ISL_418379|202	····················	····················	····················	····················	····················	····················
A	1900	hCoV-19/Canada/ON-PHL-3476/2020|EPI_ISL_418380|202	····················	····················	····················	····················	····················	····················
A	1901	hCoV-19/Canada/ON-PHL-2223/2020|EPI_ISL_418381|202	····················	····················	····················	····················	····················	····················
A	1902	hCoV-19/Canada/ON-PHL-5930/2020|EPI_ISL_418382|202	····················	····················	····················	····················	····················	····················
A	1903	hCoV-19/Canada/ON-PHL-2273/2020|EPI_ISL_418383|202	····················	····················	····················	····················	····················	····················
A	1904	hCoV-19/Finland/13M19/2020|EPI_ISL_418385|2020-03-	····················	····················	····················	····················	····················	····················
A	1905	hCoV-19/Finland/13M26/2020|EPI_ISL_418386|2020-03-	····················	····················	····················	····················	····················	····················
A	1906	hCoV-19/Finland/13M27/2020|EPI_ISL_418387|2020-03-	····················	····················	····················	····················	····················	····················
A	1907	hCoV-19/Finland/13M29/2020|EPI_ISL_418388|2020-03-	····················	····················	····················	····················	····················	····················
A	1908	hCoV-19/Finland/13M3/2020|EPI_ISL_418389|2020-03-1	····················	····················	····················	····················	····················	····················
A	1909	hCoV-19/Finland/13M33/2020|EPI_ISL_418390|2020-03-	····················	····················	····················	····················	····················	····················
A	1910	hCoV-19/Finland/13M57/2020|EPI_ISL_418391|2020-03-	····················	·A··················	····················	····················	····················	····················
A	1911	hCoV-19/Finland/13M58/2020|EPI_ISL_418392|2020-03-	····················	····················	····················	····················	····················	····················
A	1912	hCoV-19/Finland/13M60/2020|EPI_ISL_418393|2020-03-	····················	····················	····················	····················	····················	····················
A	1913	hCoV-19/Finland/13M64/2020|EPI_ISL_418394|2020-03-	····················	····················	····················	····················	····················	····················
A	1914	hCoV-19/Finland/13M65/2020|EPI_ISL_418395|2020-03-	····················	····················	····················	····················	····················	····················
A	1915	hCoV-19/Finland/13M69/2020|EPI_ISL_418396|2020-03-	····················	····················	····················	····················	····················	····················
A	1916	hCoV-19/Finland/13M77/2020|EPI_ISL_418397|2020-03-	····················	····················	····················	····················	····················	····················
A	1917	hCoV-19/Finland/13M82/2020|EPI_ISL_418399|2020-03-	····················	····················	····················	····················	····················	····················
A	1918	hCoV-19/Austria/CeMM0017/2020|EPI_ISL_419670|2020-	····················	····················	····················	····················	········N···········	····················
A	1919	hCoV-19/Austria/CeMM0018/2020|EPI_ISL_419671|2020-	····················	····················	····················	····················	····················	····················
A	1920	hCoV-19/Austria/CeMM0019/2020|EPI_ISL_419672|2020-	····················	····················	····················	····················	····················	····················
A	1921	hCoV-19/Austria/CeMM0020/2020|EPI_ISL_419673|2020-	····················	····················	····················	····················	····················	····················
A	1922	hCoV-19/Austria/CeMM0021/2020|EPI_ISL_419674|2020-	····················	····················	····················	····················	····················	····················
A	1923	hCoV-19/Spain/VC-FISABIO-11/2020|EPI_ISL_419675|20	····················	····················	····················	····················	····················	····················
A	1924	hCoV-19/Spain/VC-FISABIO-12/2020|EPI_ISL_419676|20	····················	····················	····················	····················	····················	····················
A	1925	hCoV-19/Spain/VC-FISABIO-13/2020|EPI_ISL_419677|20	····················	····················	····················	····················	····················	····················

A	1926 hCoV-19/Spain/VC-FISABIO-14/2020|EPI_ISL_419678|20	····················	····················	····················	····················	····················	····················
A	1927 hCoV-19/Spain/VC-FISABIO-15/2020|EPI_ISL_419679|20	····················	····················	····················	····················	····················	····················
A	1928 hCoV-19/Spain/VC-FISABIO-16/2020|EPI_ISL_419680|20	····················	····················	····················	····················	····················	····················
A	1929 hCoV-19/Spain/VC-FISABIO-17/2020|EPI_ISL_419681|20	····················	····················	····················	····················	····················	····················
A	1930 hCoV-19/Spain/VC-FISABIO-18/2020|EPI_ISL_419682|20	····················	····················	····················	····················	····················	····················
A	1931 hCoV-19/Spain/VC-FISABIO-19/2020|EPI_ISL_419683|20	····················	····················	····················	····················	····················	····················
A	1932 hCoV-19/Latvia/ChVir2025/2020|EPI_ISL_419691|2020-	····················	····················	····················	····················	····················	····················
A	1933 hCoV-19/Belarus/ChVir2072/2020|EPI_ISL_419692|2020	····················	·····C··············	····················	····················	····················	····················
A	1934 hCoV-19/Belarus/ChVir2073/2020|EPI_ISL_419693|2020	····················	····················	····················	····················	····················	····················
A	1935 hCoV-19/USA/NY-NYUMC35/2020|EPI_ISL_419696|2020-03	····················	····················	····················	····················	····················	····················
A	1936 hCoV-19/USA/NY-NYUMC36/2020|EPI_ISL_419697|2020-03	····················	····················	····················	····················	····················	····················
A	1937 hCoV-19/USA/NY-NYUMC37/2020|EPI_ISL_419698|2020-03	····················	····················	····················	····················	····················	····················
A	1938 hCoV-19/USA/NY-NYUMC38/2020|EPI_ISL_419699|2020-03	····················	····················	····················	····················	····················	····················
A	1939 hCoV-19/England/20139013406/2020|EPI_ISL_420661|20	····················	····················	····················	····················	····················	····················
A	1940 hCoV-19/England/20139037504/2020|EPI_ISL_420670|20	····················	····················	····················	····················	····················	····················
A	1941 hCoV-19/England/20139038104/2020|EPI_ISL_420672|20	····················	····················	····················	····················	····················	····················
A	1942 hCoV-19/England/20139039504/2020|EPI_ISL_420673|20	····················	····················	····················	····················	····················	····················
A	1945 hCoV-19/England/20139045904/2020|EPI_ISL_420687|20	····················	····················	····················	····················	····················	····················
A	1949 hCoV-19/England/20139047104/2020|EPI_ISL_420692|20	····················	····················	····················	····················	····················	····················
A	1950 hCoV-19/England/20139047404/2020|EPI_ISL_420693|20	····················	····················	····················	····················	····················	····················
A	1951 hCoV-19/England/20139047504/2020|EPI_ISL_420694|20	····················	····················	····················	····················	····················	····················
A	1952 hCoV-19/England/20139047604/2020|EPI_ISL_420695|20	····················	····················	····················	····················	····················	····················
A	1953 hCoV-19/England/20139047804/2020|EPI_ISL_420697|20	····················	····················	····················	····················	··Y·················	····················
A	1954 hCoV-19/England/20139048804/2020|EPI_ISL_420698|20	····················	····················	····················	····················	··N·················	····················
A	1955 hCoV-19/England/20146061504/2020|EPI_ISL_421986|20	····················	····················	····················	····················	····················	····················
A	1956 hCoV-19/England/CAMB-74508/2020|EPI_ISL_425300|202	····················	····················	····················	····················	····················	····················
A	1959 hCoV-19/England/CAMB-74544/2020|EPI_ISL_425304|202	····················	····················	····················	····················	····················	····················
A	1960 hCoV-19/England/CAMB-74553/2020|EPI_ISL_425305|202	····················	····················	····················	····················	····················	····················
A	1961 hCoV-19/USA/NY-NYUMC162/2020|EPI_ISL_426617|2020-0	····················	····················	····················	····················	····················	····················
A	1962 hCoV-19/USA/NY-NYUMC163/2020|EPI_ISL_426618|2020-0	····················	····················	····················	····················	····················	····················
A	1963 hCoV-19/USA/NY-NYUMC164/2020|EPI_ISL_426619|2020-0	····················	····················	····················	····················	····················	····················
A	1964 hCoV-19/USA/NY-NYUMC165/2020|EPI_ISL_426620|2020-0	····················	····················	····················	····················	····················	····················
A	1965 hCoV-19/USA/NY-NYUMC166/2020|EPI_ISL_426621|2020-0	····················	····················	····················	····················	····················	····················
A	1966 hCoV-19/USA/NY-NYUMC167/2020|EPI_ISL_426622|2020-0	····················	····················	····················	····················	····················	····················
A	1967 hCoV-19/USA/NY-NYUMC168/2020|EPI_ISL_426623|2020-0	····················	····················	····················	····················	····················	····················
A	1968 hCoV-19/USA/NY-NYUMC169/2020|EPI_ISL_426624|2020-0	····················	····················	····················	····················	····················	····················
A	1969 hCoV-19/USA/NY-NYUMC170/2020|EPI_ISL_426625|2020-0	····················	····················	····················	····················	····················	····················
A	1970 hCoV-19/USA/NY-NYUMC171/2020|EPI_ISL_426626|2020-0	····················	····················	····················	····················	····················	····················
A	1971 hCoV-19/USA/LA-BIE-096/2020|EPI_ISL_426627|2020-04	····················	····················	····················	····················	····················	····················
A	1972 hCoV-19/USA/LA-BIE-097/2020|EPI_ISL_426628|2020-04	····················	····················	····················	····················	····················	····················
A	1973 hCoV-19/Taiwan/TSGH-01/2020|EPI_ISL_426629|2020-02	····················	····················	····················	····················	····················	····················
A	1974 hCoV-19/Taiwan/TSGH-02/2020|EPI_ISL_426630|2020-03	····················	····················	····················	····················	····················	····················
A	1975 hCoV-19/Taiwan/TSGH-04/2020|EPI_ISL_426632|2020-03	····················	····················	····················	····················	····················	····················
A	1978 hCoV-19/Australia/NT19/2020|EPI_ISL_426636|2020	····················	····················	····················	····················	····················	····················
A	1979 hCoV-19/USA/CA-CZB0103/2020|EPI_ISL_429000|2020-03	····················	····················	····················	····················	····················	····················
A	1980 hCoV-19/USA/CA-CZB046/2020|EPI_ISL_429001|2020-03-	····················	····················	····················	····················	····················	····················
A	1981 hCoV-19/USA/CA-CZB098/2020|EPI_ISL_429002|2020-03-	····················	····················	····················	····················	····················	····················
A	1982 hCoV-19/USA/CA-CZB0136/2020|EPI_ISL_429003|2020-04	····················	····················	····················	····················	····················	····················
A	1983 hCoV-19/USA/CA-CZB085/2020|EPI_ISL_429004|2020-03-	····················	·A··················	····················	····················	····················	····················
A	1984 hCoV-19/USA/CA-CZB079/2020|EPI_ISL_429005|2020-03-	····················	····················	····················	····················	····················	····················
A	1985 hCoV-19/USA/CA-CZB095/2020|EPI_ISL_429006|2020-03-	····················	····················	····················	····················	····················	····················
A	1986 hCoV-19/USA/CA-CZB0458/2020|EPI_ISL_429007|2020-03	····················	····················	····················	····················	····················	····················
A	1987 hCoV-19/USA/CA-CZB091/2020|EPI_ISL_429008|2020-03-	····················	····················	····················	····················	····················	····················
A	1988 hCoV-19/USA/CA-CZB0122/2020|EPI_ISL_429009|2020-04	····················	····················	····················	····················	····················	····················
A	1989 hCoV-19/USA/CA-CZB0108/2020|EPI_ISL_429010|2020-03	····················	····················	····················	····················	····················	····················
A	1990 hCoV-19/USA/CA-CZB0147/2020|EPI_ISL_429011|2020-04	····················	····················	····················	····················	····················	····················
A	1991 hCoV-19/USA/CA-CZB016/2020|EPI_ISL_429012|2020-03-	····················	····················	····················	····················	····················	····················
A	1992 hCoV-19/USA/CA-CZB080/2020|EPI_ISL_429013|2020-03-	····················	····················	····················	····················	····················	····················
A	1993 hCoV-19/USA/CA-CZB0142/2020|EPI_ISL_429014|2020-04	····················	····················	····················	····················	····················	····················
A	1994 hCoV-19/USA/CA-CZB0709/2020|EPI_ISL_429016|2020-03	····················	···A················	····················	····················	····················	····················
A	1995 hCoV-19/USA/CA-CZB030/2020|EPI_ISL_429017|2020-03-	····················	····················	····················	····················	····················	····················
A	1996 hCoV-19/USA/CA-CZB0750/2020|EPI_ISL_429018|2020-04	····················	····················	····················	····················	····················	····················
A	1997 hCoV-19/USA/CA-CZB078/2020|EPI_ISL_429019|2020-03-	····················	····················	····················	····················	····················	····················
A	1998 hCoV-19/USA/CA-CZB0721/2020|EPI_ISL_429020|2020-03	····················	····················	····················	····················	····················	····················
A	1999 hCoV-19/USA/CA-CZB045/2020|EPI_ISL_429021|2020-03-	····················	····················	····················	····················	····················	····················
A	2000 hCoV-19/USA/CA-CZB0713/2020|EPI_ISL_429022|2020-03	····················	····················	····················	····················	····················	····················
A	2001 hCoV-19/USA/CA-CZB0740/2020|EPI_ISL_429023|2020-03	····················	····················	····················	····················	····················	····················
A	2002 hCoV-19/USA/CA-CZB0705/2020|EPI_ISL_429024|2020-03	····················	····················	····················	····················	····················	····················
A	2003 hCoV-19/USA/CA-CZB0737/2020|EPI_ISL_429025|2020-03	····················	····················	····················	····················	····················	····················
A	2004 hCoV-19/USA/CA-CZB0731/2020|EPI_ISL_429026|2020-03	····················	····················	····················	····················	····················	····················
A	2005 hCoV-19/USA/CA-CZB0137/2020|EPI_ISL_429027|2020-04	····················	····················	····················	····················	····················	····················
A	2006 hCoV-19/USA/CA-CZB0746/2020|EPI_ISL_429028|2020-04	····················	····················	····················	····················	····················	····················
A	2007 hCoV-19/USA/CA-CZB067/2020|EPI_ISL_429029|2020-03-	····················	····················	····················	····················	····················	····················
A	2008 hCoV-19/USA/CA-CZB089/2020|EPI_ISL_429050|2020-03-	····················	····················	····················	····················	····················	····················
A	2009 hCoV-19/USA/CA-CZB0741/2020|EPI_ISL_429051|2020-04	····················	····················	····················	····················	····················	····················
A	2010 hCoV-19/USA/CA-CZB0714/2020|EPI_ISL_429052|2020-03	····················	····················	····················	····················	····················	····················
A	2011 hCoV-19/USA/CA-CZB0167/2020|EPI_ISL_429053|2020-04	····················	····················	····················	····················	····················	····················
A	2012 hCoV-19/USA/CA-CZB0141/2020|EPI_ISL_429054|2020-04	····················	····················	····················	····················	····················	····················

A	2013	hCoV-19/USA/CA-CZB0726/2020|EPI_ISL_429055|2020-03	····················	····················	·····················	····················	····················	····················
A	2014	hCoV-19/USA/CA-CZB0101/2020|EPI_ISL_429056|2020-03	····················	····················	·····················	····················	····················	····················
A	2015	hCoV-19/USA/CA-CZB076/2020|EPI_ISL_429057|2020-03-	····················	····················	·····················	····················	····················	····················
A	2016	hCoV-19/USA/CA-CZB0114/2020|EPI_ISL_429058|2020-03	····················	····················	·····················	····················	····················	····················
A	2017	hCoV-19/Jordan/SR-042/2020|EPI_ISL_430000|2020-03-	····················	····················	·····················	····················	····················	····················
A	2018	hCoV-19/Jordan/SR-043/2020|EPI_ISL_430001|2020-03-	····················	····················	·····················	····················	····················	····················
A	2019	hCoV-19/Jordan/SR-044/2020|EPI_ISL_430002|2020-03-	····················	····················	·····················	····················	····················	····················
A	2020	hCoV-19/Jordan/SR-045/2020|EPI_ISL_430003|2020-03-	····················	····················	·····················	····················	····················	····················
A	2021	hCoV-19/Jordan/SR-046/2020|EPI_ISL_430004|2020-03-	····················	····················	·····················	····················	····················	····················
A	2022	hCoV-19/Jordan/SR-048/2020|EPI_ISL_430006|2020-03-	····················	····················	·····················	····················	····················	····················
A	2023	hCoV-19/Jordan/SR-049/2020|EPI_ISL_430007|2020-03-	····················	····················	·····················	····················	····················	····················
A	2024	hCoV-19/Jordan/SR-052/2020|EPI_ISL_430009|2020-03-	····················	····················	·····················	····················	····················	····················
A	2025	hCoV-19/Jordan/SR-053/2020|EPI_ISL_430011|2020-03-	····················	····················	·····················	····················	····················	····················
A	2026	hCoV-19/Jordan/SR-054/2020|EPI_ISL_430012|2020-03-	····················	····················	·····················	····················	····················	····················
A	2027	hCoV-19/Jordan/SR-057/2020|EPI_ISL_430015|2020-03-	····················	·············A······	·····················	····················	····················	····················
A	2028	hCoV-19/USA/CA-SR016/2020|EPI_ISL_430016|2020-03-2	····················	····················	·······A·············	····················	····················	····················
A	2029	hCoV-19/USA/UT-098/2020|EPI_ISL_430036|2020-03-14	····················	····················	·····················	····················	····················	····················
A	2030	hCoV-19/USA/UT-099/2020|EPI_ISL_430037|2020-03-14	····················	····················	·····················	····················	····················	····················
A	2031	hCoV-19/USA/UT-0100/2020|EPI_ISL_430038|2020-03-15	····················	····················	·····················	····················	····················	····················
A	2032	hCoV-19/USA/UT-0370/2020|EPI_ISL_430039|2020-03-30	····················	····················	·····················	····················	····················	····················
A	2033	hCoV-19/USA/UT-0443/2020|EPI_ISL_430041|2020-03-30	····················	····················	·····················	····················	····················	····················
A	2034	hCoV-19/USA/UT-0444/2020|EPI_ISL_430042|2020-03-30	····················	····················	·····················	····················	····················	····················
A	2035	hCoV-19/USA/UT-0445/2020|EPI_ISL_430043|2020-03-30	····················	····················	·····················	····················	····················	····················
A	2036	hCoV-19/USA/UT-0446/2020|EPI_ISL_430044|2020-03-30	····················	····················	·····················	····················	····················	····················
A	2037	hCoV-19/USA/UT-0447/2020|EPI_ISL_430045|2020-03-31	····················	····················	·····················	····················	····················	····················
A	2038	hCoV-19/USA/UT-0491/2020|EPI_ISL_430046|2020-04-02	····················	····················	·····················	····················	····················	····················
A	2039	hCoV-19/USA/UT-0492/2020|EPI_ISL_430047|2020-04-02	····················	····················	·····················	····················	····················	····················
A	2040	hCoV-19/USA/UT-0495/2020|EPI_ISL_430048|2020-04-02	····················	····················	·····················	····················	····················	····················
A	2041	hCoV-19/USA/UT-0500/2020|EPI_ISL_430050|2020-03-31	····················	····················	·····················	····················	····················	····················
A	2042	hCoV-19/USA/UT-0501/2020|EPI_ISL_430051|2020-04-02	····················	····················	·····················	····················	····················	····················
A	2043	hCoV-19/USA/UT-0502/2020|EPI_ISL_430052|2020-04-03	····················	····················	·····················	····················	····················	····················
A	2044	hCoV-19/USA/UT-0503/2020|EPI_ISL_430053|2020-04-02	····················	····················	·····················	····················	····················	····················
A	2045	hCoV-19/USA/UT-0506/2020|EPI_ISL_430054|2020-04-02	····················	····················	·····················	····················	····················	····················
A	2046	hCoV-19/USA/UT-0507/2020|EPI_ISL_430055|2020-04-02	····················	····················	·····················	····················	····················	····················
A	2048	hCoV-19/USA/UT-0510/2020|EPI_ISL_430057|2020-04-03	····················	····················	·····················	····················	····················	····················
A	2049	hCoV-19/Australia/NSW03/2020|EPI_ISL_408977|2020-0	····················	····················	·····················	····················	····················	····················
A	2050	hCoV-19/USA/WA4-UW2/2020|EPI_ISL_413455|2020-02-28	····················	····················	·····················	····················	····················	····················
A	2051	hCoV-19/USA/WA-S2/2020|EPI_ISL_413456|2020-02-21	····················	····················	·····················	····················	····················	····················
A	2052	hCoV-19/USA/WA6-UW3/2020|EPI_ISL_413457|2020-02-29	····················	····················	·····················	····················	····················	····················
A	2053	hCoV-19/USA/WA7-UW4/2020|EPI_ISL_413458|2020-03-01	····················	····················	·····················	····················	····················	····················
A	2054	hCoV-19/Japan/TK-20-31-3/2020|EPI_ISL_413459|2020-	····················	····················	·····················	····················	····················	····················
A	2055	hCoV-19/Belgium/ULG-3000/2020|EPI_ISL_417004|2020-	····················	····················	·····················	····················	····················	····················
A	2057	hCoV-19/Spain/GA-ISCIII-201663/2020|EPI_ISL_417007	····················	····················	·····················	····················	····················	····················
A	2058	hCoV-19/Belgium/ULG-3662/2020|EPI_ISL_417008|2020-	····················	····················	·····················	····················	····················	NN··················
A	2059	hCoV-19/Belgium/ULG-3665/2020|EPI_ISL_417009|2020-	····················	····················	·····················	····················	····················	····················
A	2060	hCoV-19/USA/WA-S17/2020|EPI_ISL_417070|2020-03-03	····················	····················	·····················	····················	····················	····················
A	2061	hCoV-19/USA/WA-S18/2020|EPI_ISL_417071|2020-03-03	····················	····················	·····················	····················	····················	····················
A	2062	hCoV-19/USA/WA-S19/2020|EPI_ISL_417072|2020-03-02	····················	····················	·····················	····················	····················	····················
A	2063	hCoV-19/USA/WA-S20/2020|EPI_ISL_417073|2020-03-02	····················	····················	·····················	····················	····················	····················
A	2064	hCoV-19/USA/WA-S21/2020|EPI_ISL_417074|2020-03-02	····················	····················	·····················	····················	····················	····················
A	2065	hCoV-19/USA/WA-S22/2020|EPI_ISL_417075|2020-03-02	····················	····················	·····················	····················	····················	····················
A	2066	hCoV-19/USA/WA-S23/2020|EPI_ISL_417076|2020-03-02	····················	····················	·····················	····················	····················	····················
A	2067	hCoV-19/USA/WA-S24/2020|EPI_ISL_417077|2020-03-02	····················	····················	·····················	····················	····················	····················
A	2068	hCoV-19/USA/WA-S26/2020|EPI_ISL_417079|2020-03-02	····················	····················	·····················	····················	····················	····················
A	2069	hCoV-19/USA/WA-S28/2020|EPI_ISL_417081|2020-03-02	····················	····················	·····················	····················	····················	····················
A	2070	hCoV-19/USA/WA-S29/2020|EPI_ISL_417082|2020-03-02	····················	····················	·····················	····················	····················	····················
A	2071	hCoV-19/England/SHEF-BFE51/2020|EPI_ISL_418300|202	····················	····················	·····················	····················	····················	····················
A	2072	hCoV-19/England/SHEF-BFE60/2020|EPI_ISL_418301|202	····················	····················	·····················	····················	····················	····················
A	2073	hCoV-19/England/SHEF-BFE9D/2020|EPI_ISL_418302|202	····················	····················	·····················	····················	····················	····················
A	2074	hCoV-19/England/SHEF-BFEAC/2020|EPI_ISL_418303|202	····················	····················	·····················	····················	····················	····················
A	2075	hCoV-19/England/SHEF-BFEBB/2020|EPI_ISL_418304|202	····················	····················	·····················	····················	····················	····················
A	2076	hCoV-19/England/SHEF-BFED9/2020|EPI_ISL_418305|202	····················	····················	·····················	····················	····················	····················
A	2077	hCoV-19/England/SHEF-BFEE8/2020|EPI_ISL_418306|202	····················	····················	·····················	····················	····················	····················
A	2078	hCoV-19/England/SHEF-BFF03/2020|EPI_ISL_418307|202	····················	····················	·····················	····················	····················	····················
A	2079	hCoV-19/England/SHEF-BFF12/2020|EPI_ISL_418308|202	····················	····················	·····················	····················	····················	····················
A	2080	hCoV-19/England/SHEF-BFF21/2020|EPI_ISL_418309|202	····················	····················	·····················	····················	····················	····················
A	2081	hCoV-19/England/SHEF-BFF30/2020|EPI_ISL_418310|202	····················	····················	·····················	····················	····················	····················
A	2082	hCoV-19/England/SHEF-BFF4F/2020|EPI_ISL_418311|202	····················	····················	·····················	····················	····················	····················
A	2083	hCoV-19/England/SHEF-BFF5E/2020|EPI_ISL_418312|202	····················	····················	·····················	····················	····················	····················
A	2084	hCoV-19/England/SHEF-BFF6D/2020|EPI_ISL_418313|202	····················	····················	·····················	····················	····················	····················
A	2085	hCoV-19/England/SHEF-BFF7C/2020|EPI_ISL_418314|202	····················	····················	·····················	····················	····················	····················
A	2086	hCoV-19/England/SHEF-BFF8B/2020|EPI_ISL_418315|202	····················	····················	·····················	····················	····················	····················
A	2087	hCoV-19/England/SHEF-BFF9A/2020|EPI_ISL_418316|202	····················	····················	·····················	····················	····················	····················
A	2088	hCoV-19/England/SHEF-BFFA9/2020|EPI_ISL_418317|202	····················	····················	·····················	····················	····················	····················
A	2089	hCoV-19/England/SHEF-BFFB8/2020|EPI_ISL_418318|202	····················	····················	·····················	····················	····················	····················
A	2090	hCoV-19/England/SHEF-BFFC7/2020|EPI_ISL_418319|202	····················	····················	·····················	····················	····················	····················
A	2091	hCoV-19/England/SHEF-BFFD6/2020|EPI_ISL_418320|202	····················	····················	·····················	····················	····················	····················
A	2092	hCoV-19/England/SHEF-BFFE5/2020|EPI_ISL_418321|202	····················	····················	·····················	····················	····················	····················

```
A 2093 hCoV-19/Canada/ON-PHL-3917/2020|EPI_ISL_418322|202 ···················· ···················· ···················· ···················· ···················· ····················
A 2094 hCoV-19/Canada/ON-PHL-3919/2020|EPI_ISL_418323|202 ···················· ···················· ···················· ···················· ···················· ····················
A 2095 hCoV-19/Canada/ON-PHL-3318/2020|EPI_ISL_418324|202 ···················· ···················· ···················· ···················· ···················· ····················
A 2096 hCoV-19/Canada/ON-PHL-5472/2020|EPI_ISL_418325|202 ···················· ···················· ···················· ···················· ···················· ····················
A 2097 hCoV-19/Canada/ON-PHL-7972/2020|EPI_ISL_418326|202 ···················· ···················· ···················· ···················· ···················· ····················
A 2098 hCoV-19/Canada/ON-PHL-4181/2020|EPI_ISL_418327|202 ···················· ·A·················· ···················· ···················· ···················· ····················
A 2099 hCoV-19/Canada/ON-PHL-3670/2020|EPI_ISL_418328|202 ···················· ···················· ···················· ···················· ···················· ····················
A 2100 hCoV-19/Canada/ON-PHL-5694/2020|EPI_ISL_418329|202 ···················· ···················· ···················· ···················· ···················· ····················
A 2101 hCoV-19/Canada/ON-PHL-8580/2020|EPI_ISL_418330|202 ···················· ···················· ···················· ···················· ···················· ····················
A 2102 hCoV-19/Canada/ON-PHL-6883/2020|EPI_ISL_418331|202 ···················· ···················· ···················· ···················· ···················· ····················
A 2103 hCoV-19/Canada/ON-PHL-0052/2020|EPI_ISL_418332|202 ···················· ···················· ···················· ···················· ···················· ····················
A 2104 hCoV-19/Canada/ON-PHL-3501/2020|EPI_ISL_418333|202 ···················· ···················· ···················· ···················· ···················· ····················
A 2105 hCoV-19/Canada/ON-PHL-4069/2020|EPI_ISL_418334|202 ···················· ···················· ···················· ···················· ···················· ····················
A 2106 hCoV-19/Canada/ON-PHL-4464/2020|EPI_ISL_418335|202 ···················· ···················· ···················· ···················· ···················· ····················
A 2107 hCoV-19/Canada/ON-PHL-5705/2020|EPI_ISL_418336|202 ···················· ···················· ···················· ···················· ···················· ····················
A 2108 hCoV-19/Canada/ON-PHL-3802/2020|EPI_ISL_418337|202 ···················· ···················· ···················· ···················· ···················· ····················
A 2109 hCoV-19/Canada/ON-PHL-5710/2020|EPI_ISL_418338|202 ···················· ···················· ···················· ···················· ···················· ····················
A 2110 hCoV-19/Canada/ON-PHL-3877/2020|EPI_ISL_418339|202 ···················· ···················· ···················· ···················· ···················· ····················
A 2111 hCoV-19/Luxembourg/LNS9080444/2020|EPI_ISL_419600| ···················· ···················· ···················· ···················· ···················· ····················
A 2112 hCoV-19/Luxembourg/LNS9086704/2020|EPI_ISL_419601| ···················· ···················· ···················· ···················· ···················· ····················
A 2113 hCoV-19/Luxembourg/LNS9324837/2020|EPI_ISL_419602| ···················· ···················· ···················· ···················· ···················· ····················
A 2114 hCoV-19/Luxembourg/LNS9512434/2020|EPI_ISL_419603| ···················· ···················· ···················· ···················· ···················· ····················
A 2115 hCoV-19/Luxembourg/LNS9627078/2020|EPI_ISL_419604| ···················· ···················· ···················· ···················· ···················· ····················
A 2116 hCoV-19/Luxembourg/LNS9862689/2020|EPI_ISL_419606| ···················· ···················· ···················· ···················· ···················· ····················
A 2117 hCoV-19/Luxembourg/LNS9982497/2020|EPI_ISL_419607| ···················· ···················· ···················· ···················· ···················· ····················
A 2118 hCoV-19/USA/WI-GMF-00466/2020|EPI_ISL_419651|2020- ···················· ···················· ···················· ···················· ···················· ····················
A 2119 hCoV-19/USA/WI-GMF-00281/2020|EPI_ISL_419652|2020- ···················· ···················· ···················· ···················· ···················· ····················
A 2120 hCoV-19/Austria/CeMM0001/2020|EPI_ISL_419654|2020- ···················· ···················· ···················· ···················· ···················· ····················
A 2121 hCoV-19/Austria/CeMM0002/2020|EPI_ISL_419655|2020- ···················· ···················· ···················· ···················· ···················· ····················
A 2122 hCoV-19/Austria/CeMM0003/2020|EPI_ISL_419656|2020- ···················· ···················· ···················· ···················· ···················· ····················
A 2123 hCoV-19/Austria/CeMM0004/2020|EPI_ISL_419657|2020- ···················· ···················· ···················· ···················· ···················· ····················
A 2124 hCoV-19/Austria/CeMM0005/2020|EPI_ISL_419658|2020- ···················· ···················· ···················· ···················· ···················· ····················
A 2125 hCoV-19/Austria/CeMM0006/2020|EPI_ISL_419659|2020- ···················· ···················· ···················· ···················· ···················· ····················
A 2126 hCoV-19/Austria/CeMM0007/2020|EPI_ISL_419660|2020- ···················· ···················· ···················· ···················· ···················· ····················
A 2127 hCoV-19/Austria/CeMM0008/2020|EPI_ISL_419661|2020- ···················· ···················· ···················· ···················· ········K··········· ····················
A 2128 hCoV-19/Austria/CeMM0009/2020|EPI_ISL_419662|2020- ···················· ···················· ···················· ···················· ···················· ····················
A 2129 hCoV-19/Austria/CeMM0011/2020|EPI_ISL_419664|2020- ···················· ···················· ···················· ···················· ···················· ····················
A 2130 hCoV-19/Austria/CeMM0012/2020|EPI_ISL_419665|2020- ···················· ···················· ···················· ···················· ···················· ····················
A 2131 hCoV-19/Austria/CeMM0013/2020|EPI_ISL_419666|2020- ···················· ···················· ···················· ···················· ···················· ····················
A 2132 hCoV-19/Austria/CeMM0014/2020|EPI_ISL_419667|2020- ···················· ···················· ···················· ···················· ···················· ····················
A 2133 hCoV-19/Austria/CeMM0016/2020|EPI_ISL_419669|2020- ···················· ···················· ···················· ···················· N··················· ····················
A 2134 hCoV-19/Argentina/C121/2020|EPI_ISL_420600|2020-03 ···················· ···················· ···················· ···················· ···················· ····················
A 2135 hCoV-19/France/ARA-12371/2020|EPI_ISL_420604|2020- ···················· ···················· ···················· ···················· ···················· ····················
A 2136 hCoV-19/France/ARA-12384/2020|EPI_ISL_420605|2020- ···················· ···················· ···················· ···················· ···················· ····················
A 2137 hCoV-19/France/ARA-12388/2020|EPI_ISL_420606|2020- ···················· ···················· ···················· ···················· ···················· ····················
A 2138 hCoV-19/France/ARA-12485/2020|EPI_ISL_420607|2020- ···················· ···················· ···················· ···················· ···················· ····················
A 2139 hCoV-19/France/ARA-12499/2020|EPI_ISL_420608|2020- ···················· ···················· ···················· ···················· ···················· ····················
A 2140 hCoV-19/France/ARA-12524/2020|EPI_ISL_420609|2020- ···················· ···················· ···················· ···················· ···················· ····················
A 2141 hCoV-19/France/ARA-12558/2020|EPI_ISL_420610|2020- ···················· ···················· ···················· ···················· ···················· ····················
A 2142 hCoV-19/France/ARA-12576/2020|EPI_ISL_420611|2020- ···················· ···················· ···················· ···················· ···················· ····················
A 2143 hCoV-19/France/ARA-12630/2020|EPI_ISL_420613|2020- ···················· ···················· ···················· ···················· ···················· ····················
A 2144 hCoV-19/France/ARA-12632/2020|EPI_ISL_420614|2020- ···················· ···················· ···················· ···················· ···················· ····················
A 2145 hCoV-19/France/ARA-12759/2020|EPI_ISL_420615|2020- ···················· ···················· ···················· ···················· ···················· ····················
A 2146 hCoV-19/France/ARA-1284/2020|EPI_ISL_420616|2020-0 ···················· ···················· ···················· ···················· ···················· ····················
A 2147 hCoV-19/France/ARA-12877/2020|EPI_ISL_420617|2020- ···················· ···················· ···················· ···················· ···················· ····················
A 2148 hCoV-19/France/ARA-12915/2020|EPI_ISL_420618|2020- ···················· ···················· ···················· ···················· ···················· ····················
A 2149 hCoV-19/France/ARA-12973/2020|EPI_ISL_420619|2020- ···················· ···················· ···················· ···················· ···················· ····················
A 2150 hCoV-19/France/ARA-12996/2020|EPI_ISL_420620|2020- ···················· ···················· ···················· ···················· ···················· ····················
A 2151 hCoV-19/France/ARA-1307/2020|EPI_ISL_420621|2020-0 ···················· ···················· ···················· ···················· ···················· ····················
A 2153 hCoV-19/France/ARA-13095/2020|EPI_ISL_420623|2020- ···················· ···················· ···················· ···················· ···················· ····················
A 2154 hCoV-19/France/ARA-13160/2020|EPI_ISL_420624|2020- ···················· ···················· ···················· ···················· ···················· ····················
A 2155 hCoV-19/France/ARA-1322/2020|EPI_ISL_420625|2020-0 ···················· ···················· ···················· ···················· ···················· ····················
A 2156 hCoV-19/USA/VA-DCLS-0024/2020|EPI_ISL_420628|2020- ···················· ···················· ···················· ···················· ···················· ····················
A 2157 hCoV-19/USA/VA-DCLS-0034/2020|EPI_ISL_420629|2020- ···················· ···················· ···················· ···················· ···················· ····················
A 2158 hCoV-19/USA/VA-DCLS-0038/2020|EPI_ISL_420630|2020- ···················· ···················· ···················· ···················· ···················· ····················
A 2159 hCoV-19/England/20130055502/2020|EPI_ISL_420633|20 ···T················ ···················· ···················· ···················· ···················· ····················
A 2160 hCoV-19/England/20136085404/2020|EPI_ISL_420636|20 ···················· ···················· ···················· ···················· ···················· ····················
A 2161 hCoV-19/England/20136088602/2020|EPI_ISL_420640|20 ···················· ···················· ···················· ···················· ···················· ····················
A 2162 hCoV-19/England/20136089402/2020|EPI_ISL_420642|20 ···················· ···················· ···················· ···················· ···················· ····················
A 2163 hCoV-19/England/20136094704/2020|EPI_ISL_420643|20 ···················· ···················· ···················· ···················· ···················· ····················
A 2164 hCoV-19/England/20136095604/2020|EPI_ISL_420645|20 ···················· ···················· ···················· ···················· ···················· ····················
A 2165 hCoV-19/England/20136096504/2020|EPI_ISL_420648|20 ···················· ···················· ···················· ···················· ···················· ····················
A 2166 hCoV-19/England/20136097204/2020|EPI_ISL_420652|20 ···················· ···················· ···················· ···················· ···················· ····················
A 2167 hCoV-19/England/20139027004/2020|EPI_ISL_421900|20 ···················· ···················· ···················· ···················· ···················· ····················
A 2169 hCoV-19/England/20139027404/2020|EPI_ISL_421904|20 ···················· ···················· ···················· ···················· ···················· ····················
A 2170 hCoV-19/England/20139027604/2020|EPI_ISL_421905|20 ···················· ···················· ···················· ···················· ···················· ····················
A 2171 hCoV-19/England/20139027704/2020|EPI_ISL_421906|20 ···················· ···················· ···················· ···················· ···················· ····················
A 2172 hCoV-19/England/20139027904/2020|EPI_ISL_421907|20 ···················· ···················· ···················· ···················· ···················· ····················
```

A	2173	hCoV-19/England/20139029104/2020|EPI_ISL_421909|20	····················	····················	····················	····················	····················	····················
A	2174	hCoV-19/England/20139029304/2020|EPI_ISL_421910|20	····················	····················	····················	····················	····················	····················
A	2175	hCoV-19/England/20139029404/2020|EPI_ISL_421911|20	····················	····················	····················	····················	····················	····················
A	2176	hCoV-19/England/20139030404/2020|EPI_ISL_421912|20	····················	····················	····················	····················	····················	····················
A	2177	hCoV-19/England/20139030704/2020|EPI_ISL_421913|20	····················	·············A······	····················	····················	····················	····················
A	2178	hCoV-19/England/20139031404/2020|EPI_ISL_421914|20	····················	····················	····················	····················	····················	····················
A	2179	hCoV-19/England/20139031504/2020|EPI_ISL_421915|20	····················	····················	····················	····················	····················	····················
A	2180	hCoV-19/England/20139031804/2020|EPI_ISL_421916|20	····················	····················	····················	····················	····················	····················
A	2181	hCoV-19/England/20139032404/2020|EPI_ISL_421918|20	····················	····················	····················	····················	····················	····················
A	2182	hCoV-19/England/20139033004/2020|EPI_ISL_421919|20	····················	····················	····················	····················	····················	····················
A	2184	hCoV-19/England/20139033404/2020|EPI_ISL_421921|20	····················	····················	····················	····················	····················	····················
A	2185	hCoV-19/England/20139036504/2020|EPI_ISL_421923|20	····················	····················	····················	····················	····················	····················
A	2188	hCoV-19/England/20144012204/2020|EPI_ISL_421930|20	····················	····················	····················	····················	··N·················	····················
A	2189	hCoV-19/England/20144013504/2020|EPI_ISL_421932|20	····················	····················	····················	····················	····················	····················
A	2190	hCoV-19/England/20144014004/2020|EPI_ISL_421934|20	····················	····················	····················	····················	····················	····················
A	2191	hCoV-19/England/20144014604/2020|EPI_ISL_421935|20	····················	····················	····················	····················	····················	····················
A	2193	hCoV-19/England/20144016604/2020|EPI_ISL_421937|20	····················	····················	····················	····················	····················	····················
A	2194	hCoV-19/England/20144018204/2020|EPI_ISL_421938|20	····················	····················	····················	····················	····················	····················
A	2195	hCoV-19/England/20144018704/2020|EPI_ISL_421939|20	····················	····················	····················	····················	····················	····················
A	2196	hCoV-19/England/20144019304/2020|EPI_ISL_421940|20	····················	····················	····················	····················	····················	····················
A	2197	hCoV-19/England/20144020004/2020|EPI_ISL_421942|20	····················	····················	····················	····················	····················	····················
A	2199	hCoV-19/England/20144037304/2020|EPI_ISL_421946|20	····················	····················	····················	····················	····················	····················
A	2201	hCoV-19/England/20144049404/2020|EPI_ISL_421948|20	····················	····················	····················	····················	····················	····················
A	2202	hCoV-19/England/20144049704/2020|EPI_ISL_421951|20	····················	····················	····················	····················	····················	····················
A	2203	hCoV-19/England/20144052104/2020|EPI_ISL_421953|20	····················	····················	····················	····················	····················	····················
A	2205	hCoV-19/England/20144067504/2020|EPI_ISL_421957|20	····················	····················	····················	····················	····················	····················
A	2206	hCoV-19/England/20144071004/2020|EPI_ISL_421958|20	····················	····················	····················	····················	····················	····················
A	2209	hCoV-19/England/20144076304/2020|EPI_ISL_421964|20	····················	····················	····················	····················	····················	····················
A	2210	hCoV-19/England/20146008104/2020|EPI_ISL_421974|20	····················	····················	····················	····················	····················	····················
A	2212	hCoV-19/England/20146019004/2020|EPI_ISL_421981|20	····················	····················	····················	····················	····················	····················
A	2213	hCoV-19/England/20146019704/2020|EPI_ISL_421982|20	····················	····················	····················	····················	····················	····················
A	2214	hCoV-19/England/20129125504/2020|EPI_ISL_424100|20	····················	····················	····················	····················	····················	····················
A	2215	hCoV-19/England/20129126204/2020|EPI_ISL_424101|20	····················	····················	····················	····················	····················	R···················
A	2217	hCoV-19/England/20129147804/2020|EPI_ISL_424116|20	····················	····················	····················	····················	····················	····················
A	2218	hCoV-19/England/20129148904/2020|EPI_ISL_424117|20	····················	····················	····················	····················	····················	····················
A	2219	hCoV-19/USA/WA-UW-1294/2020|EPI_ISL_424180|2020-03	····················	····················	····················	····················	····················	····················
A	2220	hCoV-19/USA/UN-UW-1402/2020|EPI_ISL_424181|2020-03	····················	····················	····················	····················	····················	····················
A	2221	hCoV-19/USA/WA-UW-1478/2020|EPI_ISL_424182|2020-03	····················	····················	····················	····················	····················	····················
A	2222	hCoV-19/USA/WA-UW-1436/2020|EPI_ISL_424183|2020-03	····················	····················	····················	····················	····················	····················
A	2223	hCoV-19/USA/WA-UW-1422/2020|EPI_ISL_424184|2020-03	····················	····················	····················	····················	····················	····················
A	2224	hCoV-19/USA/WA-UW-1644/2020|EPI_ISL_424186|2020-03	····················	····················	····················	····················	····················	····················
A	2225	hCoV-19/USA/WA-UW-1646/2020|EPI_ISL_424187|2020-03	····················	····················	····················	····················	····················	····················
A	2226	hCoV-19/USA/WA-UW-1645/2020|EPI_ISL_424188|2020-03	····················	····················	····················	····················	····················	····················
A	2227	hCoV-19/USA/WA-UW-01/2020|EPI_ISL_424189|2020-03	····················	····················	····················	····················	····················	····················
A	2228	hCoV-19/USA/WA-UW-1683/2020|EPI_ISL_424191|2020-03	····················	····················	····················	····················	····················	····················
A	2229	hCoV-19/USA/WA-UW-1688/2020|EPI_ISL_424192|2020-03	····················	····················	····················	····················	····················	····················
A	2230	hCoV-19/USA/WA-UW-1728/2020|EPI_ISL_424193|2020-03	····················	····················	····················	····················	····················	····················
A	2231	hCoV-19/USA/WA-UW-1661/2020|EPI_ISL_424194|2020-03	····················	····················	····················	····················	····················	····················
A	2232	hCoV-19/USA/WA-UW-1726/2020|EPI_ISL_424195|2020-03	····················	····················	····················	····················	····················	····················
A	2233	hCoV-19/USA/WA-UW-1666/2020|EPI_ISL_424196|2020-03	····················	····················	····················	····················	····················	····················
A	2234	hCoV-19/USA/WA-UW-1640/2020|EPI_ISL_424197|2020-03	····················	····················	····················	····················	····················	····················
A	2235	hCoV-19/USA/WA-UW-1656/2020|EPI_ISL_424198|2020-03	····················	····················	····················	····················	····················	····················
A	2236	hCoV-19/England/CAMB-76D95/2020|EPI_ISL_425427|202	····················	····················	····················	····················	····················	····················
A	2237	hCoV-19/England/CAMB-76DB3/2020|EPI_ISL_425428|202	····················	····················	····················	····················	····················	····················
A	2238	hCoV-19/England/CAMB-76DFF/2020|EPI_ISL_425430|202	····················	····················	····················	····················	····················	····················
A	2239	hCoV-19/England/CAMB-76E1A/2020|EPI_ISL_425431|202	····················	····················	····················	····················	····················	····················
A	2241	hCoV-19/England/CAMB-76E56/2020|EPI_ISL_425433|202	····················	····················	····················	····················	····················	····················
A	2242	hCoV-19/England/CAMB-76E74/2020|EPI_ISL_425434|202	····················	····················	····················	····················	····················	····················
A	2243	hCoV-19/England/CAMB-76E92/2020|EPI_ISL_425435|202	····················	····················	····················	····················	····················	····················
A	2244	hCoV-19/England/CAMB-76EB0/2020|EPI_ISL_425436|202	····················	····················	····················	····················	····················	····················
A	2245	hCoV-19/England/CAMB-76EDE/2020|EPI_ISL_425437|202	····················	····················	····················	····················	····················	····················
A	2246	hCoV-19/England/CAMB-76F17/2020|EPI_ISL_425439|202	····················	····················	····················	····················	····················	····················
A	2247	hCoV-19/England/CAMB-76F35/2020|EPI_ISL_425440|202	····················	····················	····················	····················	····················	····················
A	2248	hCoV-19/England/CAMB-76F62/2020|EPI_ISL_425441|202	····················	····················	····················	····················	····················	····················
A	2249	hCoV-19/England/CAMB-76F9F/2020|EPI_ISL_425443|202	····················	····················	····················	····················	····················	····················
A	2250	hCoV-19/England/CAMB-76FCC/2020|EPI_ISL_425446|202	····················	····················	····················	····················	····················	····················
A	2251	hCoV-19/England/NOTT-10DF14/2020|EPI_ISL_425469|20	····················	····················	····················	····················	····················	····················
A	2253	hCoV-19/Australia/VIC512/2020|EPI_ISL_426782|2020-	····················	····················	····················	····················	····················	····················
A	2254	hCoV-19/Australia/VIC513/2020|EPI_ISL_426783|2020-	····················	····················	····················	····················	····················	····················
A	2255	hCoV-19/Australia/VIC514/2020|EPI_ISL_426784|2020-	····················	····················	····················	····················	····················	····················
A	2256	hCoV-19/Australia/VIC516/2020|EPI_ISL_426786|2020-	····················	····················	····················	····················	····················	····················
A	2258	hCoV-19/Australia/VIC520/2020|EPI_ISL_426789|2020-	····················	····················	····················	····················	····················	····················
A	2259	hCoV-19/Australia/VIC522/2020|EPI_ISL_426790|2020-	····················	····················	····················	····················	····················	····················
A	2261	hCoV-19/Australia/VIC527/2020|EPI_ISL_426793|2020-	····················	····················	····················	····················	····················	····················
A	2262	hCoV-19/Australia/VIC530/2020|EPI_ISL_426796|2020-	····················	····················	····················	····················	····················	····················
A	2263	hCoV-19/Australia/VIC531/2020|EPI_ISL_426797|2020-	····················	····················	····················	····················	····················	····················
A	2264	hCoV-19/Australia/VIC532/2020|EPI_ISL_426798|2020-	····················	····················	····················	····················	····················	····················
A	2266	hCoV-19/USA/CA-CZB072/2020|EPI_ISL_429030|2020-03-	····················	····················	····················	····················	····················	····················

A	2267	hCoV-19/USA/CA-CZB0139/2020|EPI_ISL_429031|2020-04	····················	····················	·····················	····················	····················	····················
A	2268	hCoV-19/USA/CA-CZB082/2020|EPI_ISL_429032|2020-03-	····················	····················	·····················	····················	····················	····················
A	2269	hCoV-19/USA/CA-CZB086/2020|EPI_ISL_429033|2020-03-	····················	····················	·····················	····················	····················	····················
A	2270	hCoV-19/USA/CA-CZB0706/2020|EPI_ISL_429034|2020-03	····················	····················	·····················	····················	····················	····················
A	2271	hCoV-19/USA/CA-CZB042/2020|EPI_ISL_429035|2020-03-	····················	····················	·····················	····················	····················	····················
A	2272	hCoV-19/USA/CA-CZB0107/2020|EPI_ISL_429036|2020-03	····················	····················	·····················	····················	····················	····················
A	2273	hCoV-19/USA/CA-CZB09/2020|EPI_ISL_429037|2020-03-2	····················	····················	·····················	····················	····················	····················
A	2274	hCoV-19/USA/CA-CZB0707/2020|EPI_ISL_429038|2020-03	····················	····················	·····················	····················	····················	····················
A	2275	hCoV-19/USA/CA-CZB036/2020|EPI_ISL_429039|2020-03-	····················	····················	·····················	····················	····················	····················
A	2276	hCoV-19/USA/CA-CZB0701/2020|EPI_ISL_429040|2020-03	····················	····················	·····················	····················	····················	····················
A	2277	hCoV-19/USA/CA-CZB096/2020|EPI_ISL_429041|2020-03-	····················	····················	·····················	····················	····················	····················
A	2278	hCoV-19/USA/CA-CZB0710/2020|EPI_ISL_429042|2020-03	····················	····················	·····················	····················	····················	····················
A	2279	hCoV-19/USA/CA-CZB0156/2020|EPI_ISL_429043|2020-04	····················	····················	·····················	····················	····················	····················
A	2280	hCoV-19/USA/CA-CZB0158/2020|EPI_ISL_429044|2020-04	····················	····················	·····················	····················	····················	····················
A	2281	hCoV-19/USA/CA-CZB039/2020|EPI_ISL_429045|2020-03-	····················	····················	·····················	····················	····················	····················
A	2282	hCoV-19/USA/CA-CZB0125/2020|EPI_ISL_429046|2020-04	····················	····················	·····················	····················	····················	····················
A	2283	hCoV-19/USA/CA-CZB0154/2020|EPI_ISL_429047|2020-04	····················	····················	·····················	····················	····················	····················
A	2284	hCoV-19/USA/CA-CZB043/2020|EPI_ISL_429048|2020-03-	····················	····················	·····················	····················	····················	····················
A	2285	hCoV-19/USA/CA-CZB090/2020|EPI_ISL_429049|2020-03-	····················	····················	·····················	····················	····················	····················
A	2286	hCoV-19/Jordan/SR-056/2020|EPI_ISL_430014|2020-03-	····················	·············A······	·····················	····················	····················	····················
A	2287	hCoV-19/USA/UT-066/2020|EPI_ISL_430019|2020-03-16	····················	····················	·····················	····················	····················	····················
A	2288	hCoV-19/USA/UT-069/2020|EPI_ISL_430021|2020-03-16	····················	····················	·····················	····················	····················	····················
A	2289	hCoV-19/USA/UT-070/2020|EPI_ISL_430022|2020-03-16	····················	····················	·····················	····················	····················	····················
A	2290	hCoV-19/USA/UT-071/2020|EPI_ISL_430023|2020-03-16	····················	····················	·····················	····················	····················	····················
A	2291	hCoV-19/USA/UT-072/2020|EPI_ISL_430024|2020-03-16	····················	····················	·····················	····················	····················	····················
A	2292	hCoV-19/USA/UT-074/2020|EPI_ISL_430025|2020-03-17	····················	····················	·····················	····················	····················	····················
A	2293	hCoV-19/USA/UT-075/2020|EPI_ISL_430026|2020-03-16	····················	····················	·····················	····················	····················	····················
A	2294	hCoV-19/USA/UT-077/2020|EPI_ISL_430028|2020-03-17	····················	····················	·····················	····················	····················	····················
A	2295	hCoV-19/USA/UT-078/2020|EPI_ISL_430029|2020-03-15	····················	····················	·····················	····················	····················	····················
A	2296	hCoV-19/USA/UT-080/2020|EPI_ISL_430031|2020-03-15	····················	····················	·····················	····················	····················	····················
A	2297	hCoV-19/USA/UT-083/2020|EPI_ISL_430032|2020-03-17	····················	·············A······	·····················	····················	····················	····················
A	2298	hCoV-19/USA/UT-084/2020|EPI_ISL_430033|2020-03-17	····················	····················	·····················	····················	····················	····················
A	2300	hCoV-19/USA/UT-097/2020|EPI_ISL_430035|2020-03-15	····················	····················	·····················	····················	····················	····················
A	2301	hCoV-19/dog/Hong Kong/20-03695/2020|EPI_ISL_450403	····················	····················	·····················	····················	····················	····················
A	2302	hCoV-19/Hong Kong/VM20009579/2020|EPI_ISL_450404|2	····················	····················	·····················	····················	····················	····················
A	2303	hCoV-19/Hong Kong/VM2003179/2020|EPI_ISL_450405|20	····················	····················	·····················	····················	····················	····················
A	2304	hCoV-19/Wuhan/YB012504/2020|EPI_ISL_450489|2020-01	····················	····················	·····················	····················	····················	····················
A	2305	hCoV-19/Zhejiang/WZ-01/2020|EPI_ISL_404227|2020-01	····················	····················	·····················	····················	····················	····················
A	2306	hCoV-19/Zhejiang/WZ-02/2020|EPI_ISL_404228|2020-01	····················	····················	·····················	····················	····················	····················
A	2307	hCoV-19/USA/IL1/2020|EPI_ISL_404253|2020-01-21	····················	·R··················	·····················	····················	····················	····················
A	2308	hCoV-19/England/09c/2020|EPI_ISL_412116|2020-02-09	····················	····················	·····················	····················	····················	····················
A	2309	hCoV-19/USA/WA8-UW5/2020|EPI_ISL_413486|2020-03-01	····················	····················	·····················	····················	····················	····················
A	2310	hCoV-19/USA/WA9-UW6/2020|EPI_ISL_413487|2020-03-01	····················	····················	·····················	····················	····················	····················
A	2311	hCoV-19/Germany/NW-HHU-01/2020|EPI_ISL_413488|2020	····················	····················	·····················	····················	····················	····················
A	2312	hCoV-19/Italy/LOM-UniSR-1/2020|EPI_ISL_413489|2020	····················	····················	·····················	····················	····················	····················
A	2313	hCoV-19/New Zealand/20VR0174/2020|EPI_ISL_413490|2	····················	····················	·····················	····················	····················	····················
A	2314	hCoV-19/Luxembourg/LNS5731562/2020|EPI_ISL_417529|	····················	····················	·····················	····················	····················	····················
A	2315	hCoV-19/Luxembourg/LNS2128808/2020|EPI_ISL_417530|	····················	····················	·····················	····················	····················	····················
A	2316	hCoV-19/Luxembourg/LNS2013896/2020|EPI_ISL_417531|	····················	····················	·····················	····················	····················	····················
A	2317	hCoV-19/Luxembourg/LNS8489624/2020|EPI_ISL_417532|	····················	····················	·····················	····················	····················	····················
A	2318	hCoV-19/Luxembourg/LNS6282845/2020|EPI_ISL_417533|	····················	····················	·····················	····················	····················	····················
A	2319	hCoV-19/Luxembourg/LNS0158952/2020|EPI_ISL_417534|	····················	····················	·····················	····················	····················	····················
A	2320	hCoV-19/Iceland/1/2020|EPI_ISL_417535|2020-03-13	····················	····················	·····················	····················	····················	····················
A	2321	hCoV-19/Iceland/10/2020|EPI_ISL_417536|2020-03-16	····················	····················	·····················	····················	····················	····················
A	2322	hCoV-19/Iceland/100/2020|EPI_ISL_417537|2020-03-16	····················	····················	·····················	····················	····················	····················
A	2323	hCoV-19/Iceland/104/2020|EPI_ISL_417538|2020-03-17	····················	····················	·····················	····················	····················	····················
A	2324	hCoV-19/Iceland/11/2020|EPI_ISL_417539|2020-03-16	····················	····················	·····················	····················	····················	····················
A	2325	hCoV-19/Iceland/115/2020|EPI_ISL_417540|2020-03-17	····················	····················	·····················	····················	····················	····················
A	2326	hCoV-19/Iceland/116/2020|EPI_ISL_417541|2020-03-17	····················	····················	·····················	····················	····················	····················
A	2327	hCoV-19/Iceland/117/2020|EPI_ISL_417542|2020-03-17	····················	····················	·····················	····················	····················	····················
A	2328	hCoV-19/Iceland/118/2020|EPI_ISL_417543|2020-03-17	····················	····················	·····················	····················	····················	····················
A	2329	hCoV-19/Iceland/119/2020|EPI_ISL_417544|2020-03-17	····················	····················	·····················	····················	····················	····················
A	2330	hCoV-19/Iceland/120/2020|EPI_ISL_417545|2020-03-17	····················	····················	·····················	····················	····················	····················
A	2331	hCoV-19/Iceland/170/2020|EPI_ISL_417548|2020-03-17	····················	····················	·····················	····················	····················	····················
A	2332	hCoV-19/Iceland/171/2020|EPI_ISL_417549|2020-03-14	····················	····················	·····················	····················	····················	····················
A	2333	hCoV-19/Iceland/232/2020|EPI_ISL_417560|2020-03-17	····················	····················	·····················	····················	····················	····················
A	2334	hCoV-19/Iceland/233/2020|EPI_ISL_417561|2020-03-17	····················	····················	·····················	····················	····················	····················
A	2335	hCoV-19/Iceland/234/2020|EPI_ISL_417562|2020-03-17	····················	····················	·····················	····················	····················	····················
A	2336	hCoV-19/Iceland/235/2020|EPI_ISL_417563|2020-03-16	····················	····················	·····················	····················	····················	····················
A	2337	hCoV-19/Iceland/236/2020|EPI_ISL_417564|2020-03-17	····················	····················	·····················	····················	····················	····················
A	2338	hCoV-19/Iceland/237/2020|EPI_ISL_417565|2020-03-17	····················	····················	·····················	····················	····················	····················
A	2339	hCoV-19/Iceland/238/2020|EPI_ISL_417566|2020-03-17	····················	····················	·····················	····················	····················	····················
A	2340	hCoV-19/Iceland/239/2020|EPI_ISL_417567|2020-03-17	····················	····················	·····················	····················	····················	····················
A	2341	hCoV-19/Iceland/240/2020|EPI_ISL_417568|2020-03-17	····················	····················	·····················	····················	····················	····················
A	2342	hCoV-19/Iceland/241/2020|EPI_ISL_417569|2020-03-17	····················	····················	·····················	····················	····················	····················
A	2343	hCoV-19/Spain/CT-HUVH-VH1133/2020|EPI_ISL_418860|2	····················	····················	·····················	····················	····················	····················
A	2344	hCoV-19/Spain/CT-HUVH-VH2683/2020|EPI_ISL_418861|2	····················	····················	·····················	····················	····················	····················
A	2345	hCoV-19/Belgium/VS-030542/2020|EPI_ISL_418863|2020	····················	····················	·····················	····················	····················	····················

A	2346	hCoV-19/USA/VA-DCLS-0001/2020|EPI_ISL_418864|2020-	····················	····················	····················	····················	····················	····················
A	2347	hCoV-19/USA/WA-UW298/2020|EPI_ISL_418866|2020-03-1	····················	····················	····················	····················	····················	····················
A	2348	hCoV-19/USA/WA-UW299/2020|EPI_ISL_418867|2020-03-1	····················	····················	····················	····················	····················	····················
A	2349	hCoV-19/USA/WA-UW300/2020|EPI_ISL_418868|2020-03-1	····················	····················	····················	····················	····················	····················
A	2350	hCoV-19/USA/WA-UW301/2020|EPI_ISL_418869|2020-03-1	····················	····················	····················	····················	····················	····················
A	2351	hCoV-19/USA/WA-UW302/2020|EPI_ISL_418870|2020-03-1	····················	····················	····················	····················	····················	····················
A	2352	hCoV-19/USA/CT-UW303/2020|EPI_ISL_418871|2020-03-1	····················	····················	····················	····················	····················	····················
A	2353	hCoV-19/USA/WA-UW304/2020|EPI_ISL_418872|2020-03-1	····················	····················	····················	····················	····················	····················
A	2354	hCoV-19/USA/WA-UW305/2020|EPI_ISL_418873|2020-03-1	····················	····················	····················	····················	····················	····················
A	2355	hCoV-19/USA/WA-UW307/2020|EPI_ISL_418875|2020-03-1	····················	····················	····················	····················	····················	····················
A	2356	hCoV-19/USA/WA-UW308/2020|EPI_ISL_418876|2020-03-1	····················	····················	····················	····················	····················	····················
A	2357	hCoV-19/USA/WA-UW309/2020|EPI_ISL_418877|2020-03-2	····················	····················	····················	····················	····················	····················
A	2358	hCoV-19/USA/WA-UW310/2020|EPI_ISL_418878|2020-03-1	····················	····················	····················	····················	····················	····················
A	2359	hCoV-19/USA/WA-UW323/2020|EPI_ISL_418890|2020-03-1	····················	····················	····················	····················	····················	····················
A	2360	hCoV-19/USA/WA-UW324/2020|EPI_ISL_418891|2020-03-2	····················	····················	····················	····················	····················	····················
A	2361	hCoV-19/USA/WA-UW325/2020|EPI_ISL_418892|2020-03-1	····················	····················	····················	····················	····················	····················
A	2362	hCoV-19/USA/MN-UW326/2020|EPI_ISL_418893|2020-03-1	····················	····················	····················	····················	····················	····················
A	2363	hCoV-19/USA/CT-UW328/2020|EPI_ISL_418895|2020-03-1	····················	····················	····················	····················	····················	····················
A	2364	hCoV-19/USA/WA-UW330/2020|EPI_ISL_418897|2020-03-1	····················	····················	····················	····················	····················	····················
A	2365	hCoV-19/USA/CT-UW331/2020|EPI_ISL_418898|2020-03-1	····················	····················	····················	····················	····················	····················
A	2366	hCoV-19/USA/WA-UW332/2020|EPI_ISL_418899|2020-03-1	····················	····················	····················	····················	····················	····················
A	2367	hCoV-19/England/20140068404/2020|EPI_ISL_423400|20	····················	····················	····················	····················	····················	····················
A	2368	hCoV-19/England/20140068904/2020|EPI_ISL_423401|20	····················	····················	····················	····················	····················	····················
A	2370	hCoV-19/England/20129159604/2020|EPI_ISL_424120|20	····················	····················	····················	····················	····················	····················
A	2371	hCoV-19/England/20129160704/2020|EPI_ISL_424121|20	····················	····················	····················	····················	····················	····················
A	2372	hCoV-19/England/20129163604/2020|EPI_ISL_424126|20	····················	····················	····················	····················	····················	····················
A	2373	hCoV-19/England/20129170204/2020|EPI_ISL_424133|20	····················	····················	····················	····················	····················	····················
A	2374	hCoV-19/England/20129171604/2020|EPI_ISL_424140|20	····················	····················	····················	····················	····················	·N··················
A	2375	hCoV-19/England/20129174804/2020|EPI_ISL_424152|20	····················	····················	····················	····················	····················	····················
A	2376	hCoV-19/USA/WA-UW-1620/2020|EPI_ISL_424166|2020-03	····················	····················	····················	····················	····················	····················
A	2377	hCoV-19/USA/WA-UW-1616/2020|EPI_ISL_424167|2020-03	····················	····················	····················	····················	····················	····················
A	2378	hCoV-19/USA/WA-UW-1538/2020|EPI_ISL_424169|2020-03	····················	····················	····················	····················	····················	····················
A	2379	hCoV-19/USA/WA-UW-1595/2020|EPI_ISL_424170|2020-03	····················	····················	····················	····················	····················	····················
A	2380	hCoV-19/USA/WA-UW-1575/2020|EPI_ISL_424172|2020-03	····················	····················	····················	····················	····················	····················
A	2381	hCoV-19/USA/IL-UW-1311/2020|EPI_ISL_424173|2020-03	····················	····················	····················	····················	····················	····················
A	2382	hCoV-19/USA/CT-UW-1365/2020|EPI_ISL_424174|2020-03	····················	····················	····················	····················	····················	····················
A	2383	hCoV-19/USA/WA-UW-1362/2020|EPI_ISL_424175|2020-03	····················	····················	····················	····················	····················	····················
A	2384	hCoV-19/USA/WA-UW-1472/2020|EPI_ISL_424176|2020-03	····················	····················	····················	····················	····················	····················
A	2385	hCoV-19/USA/WA-UW-1342/2020|EPI_ISL_424178|2020-03	····················	····················	····················	····················	····················	····················
A	2386	hCoV-19/USA/WA-UW-1341/2020|EPI_ISL_424179|2020-03	····················	····················	····················	····················	····················	····················
A	2387	hCoV-19/Sweden/RV-FOI-1/2020|EPI_ISL_424703|2020-0	····················	····················	····················	····················	····················	····················
A	2398	hCoV-19/England/CAMB-76D1D/2020|EPI_ISL_425423|202	····················	····················	····················	····················	····················	····················
A	2399	hCoV-19/England/CAMB-76D3B/2020|EPI_ISL_425424|202	····················	····················	····················	····················	····················	····················
A	2400	hCoV-19/England/CAMB-76D59/2020|EPI_ISL_425425|202	····················	····················	····················	····················	····················	····················
A	2401	hCoV-19/England/CAMB-76FF9/2020|EPI_ISL_425449|202	····················	····················	····················	····················	····················	····················
A	2402	hCoV-19/England/CAMB-77004/2020|EPI_ISL_425450|202	····················	····················	····················	····················	····················	····················
A	2403	hCoV-19/England/CAMB-77013/2020|EPI_ISL_425451|202	····················	··G·················	····················	····················	····················	····················
A	2404	hCoV-19/England/CAMB-77022/2020|EPI_ISL_425452|202	····················	····················	····················	····················	····················	····················
A	2405	hCoV-19/England/CAMB-77031/2020|EPI_ISL_425453|202	····················	····················	····················	····················	····················	····················
A	2406	hCoV-19/England/CAMB-77040/2020|EPI_ISL_425454|202	····················	····················	····················	····················	····················	····················
A	2407	hCoV-19/England/CAMB-7705F/2020|EPI_ISL_425455|202	····················	····················	····················	····················	····················	····················
A	2408	hCoV-19/England/CAMB-7707D/2020|EPI_ISL_425457|202	····················	····················	····················	····················	····················	····················
A	2409	hCoV-19/England/CAMB-7708C/2020|EPI_ISL_425458|202	····················	····················	····················	····················	····················	····················
A	2410	hCoV-19/England/CAMB-770B9/2020|EPI_ISL_425460|202	····················	····················	····················	····················	····················	····················
A	2411	hCoV-19/England/NOTT-10DEAE/2020|EPI_ISL_425462|20	····················	····················	····················	····················	····················	····················
A	2412	hCoV-19/England/NOTT-10DECC/2020|EPI_ISL_425464|20	····················	····················	····················	····················	····················	····················
A	2413	hCoV-19/England/NOTT-10DEDB/2020|EPI_ISL_425465|20	····················	····················	····················	····················	····················	····················
A	2414	hCoV-19/England/NOTT-10DEEA/2020|EPI_ISL_425466|20	····················	····················	····················	····················	····················	····················
A	2415	hCoV-19/England/NOTT-10DF05/2020|EPI_ISL_425468|20	····················	····················	····················	····················	····················	····················
A	2416	hCoV-19/England/NOTT-10DF23/2020|EPI_ISL_425470|20	····················	····················	····················	····················	····················	····················
A	2417	hCoV-19/England/NOTT-10DF32/2020|EPI_ISL_425471|20	····················	····················	····················	····················	····················	····················
A	2418	hCoV-19/England/NOTT-10DF41/2020|EPI_ISL_425472|20	····················	····················	····················	····················	····················	····················
A	2419	hCoV-19/England/NOTT-10DF50/2020|EPI_ISL_425473|20	····················	····················	····················	····················	····················	····················
A	2420	hCoV-19/England/NOTT-10DF6F/2020|EPI_ISL_425474|20	····················	····················	····················	····················	····················	····················
A	2421	hCoV-19/England/NOTT-10DF7E/2020|EPI_ISL_425475|20	····················	····················	····················	····················	····················	····················
A	2422	hCoV-19/England/NOTT-10DF9C/2020|EPI_ISL_425476|20	····················	····················	····················	····················	····················	····················
A	2423	hCoV-19/England/NOTT-10DFAB/2020|EPI_ISL_425477|20	····················	····················	····················	····················	····················	····················
A	2424	hCoV-19/England/NOTT-10DFC9/2020|EPI_ISL_425479|20	····················	····················	····················	····················	····················	····················
A	2425	hCoV-19/England/NOTT-10DFD8/2020|EPI_ISL_425480|20	····················	····················	····················	····················	····················	····················
A	2426	hCoV-19/England/NOTT-10DFE7/2020|EPI_ISL_425481|20	····················	····················	····················	····················	····················	····················
A	2427	hCoV-19/England/NOTT-10DFF6/2020|EPI_ISL_425482|20	····················	····················	····················	····················	····················	····················
A	2428	hCoV-19/England/NOTT-10E001/2020|EPI_ISL_425483|20	····················	····················	····················	····················	····················	····················
A	2429	hCoV-19/England/NOTT-10E010/2020|EPI_ISL_425484|20	····················	····················	····················	····················	····················	····················
A	2430	hCoV-19/England/NOTT-10E02F/2020|EPI_ISL_425485|20	····················	····················	····················	····················	····················	····················
A	2431	hCoV-19/England/NOTT-10E03E/2020|EPI_ISL_425486|20	····················	····················	····················	····················	····················	····················
A	2432	hCoV-19/England/NOTT-10E04D/2020|EPI_ISL_425487|20	····················	····················	····················	····················	····················	····················
A	2433	hCoV-19/England/NOTT-10E05C/2020|EPI_ISL_425488|20	····················	····················	····················	····················	····················	····················
A	2434	hCoV-19/England/NOTT-10E06B/2020|EPI_ISL_425489|20	····················	····················	····················	····················	····················	····················

A	2435	hCoV-19/England/NOTT-10E07A/2020|EPI_ISL_425490|20	····················	····················	····················	····················	····················	····················
A	2436	hCoV-19/England/NOTT-10E089/2020|EPI_ISL_425491|20	····················	····················	····················	····················	····················	····················
A	2437	hCoV-19/England/NOTT-10E098/2020|EPI_ISL_425492|20	····················	····················	····················	····················	····················	····················
A	2438	hCoV-19/England/NOTT-10E0A7/2020|EPI_ISL_425493|20	····················	····················	····················	····················	····················	····················
A	2439	hCoV-19/England/NOTT-10E0B6/2020|EPI_ISL_425494|20	····················	····················	····················	····················	····················	····················
A	2440	hCoV-19/England/NOTT-10E0C5/2020|EPI_ISL_425495|20	····················	····················	····················	····················	····················	····················
A	2441	hCoV-19/England/NOTT-10E0D4/2020|EPI_ISL_425496|20	····················	····················	····················	····················	····················	····················
A	2442	hCoV-19/England/NOTT-10E0E3/2020|EPI_ISL_425497|20	····················	····················	····················	····················	····················	····················
A	2443	hCoV-19/England/NOTT-10E0F2/2020|EPI_ISL_425498|20	····················	····················	····················	····················	····················	····················
A	2444	hCoV-19/Australia/VIC397/2020|EPI_ISL_426700|2020-	····················	····················	····················	····················	····················	····················
A	2445	hCoV-19/Australia/VIC398/2020|EPI_ISL_426701|2020-	····················	····················	····················	····················	····················	····················
A	2446	hCoV-19/Australia/VIC399/2020|EPI_ISL_426702|2020-	····················	····················	····················	····················	····················	····················
A	2447	hCoV-19/Australia/VIC400/2020|EPI_ISL_426703|2020-	····················	····················	····················	····················	····················	····················
A	2448	hCoV-19/Australia/VIC402/2020|EPI_ISL_426705|2020-	····················	····················	····················	····················	····················	····················
A	2449	hCoV-19/Australia/VIC403/2020|EPI_ISL_426706|2020-	····················	····················	····················	····················	····················	····················
A	2450	hCoV-19/Australia/VIC404/2020|EPI_ISL_426707|2020-	····················	····················	····················	····················	····················	····················
A	2451	hCoV-19/Australia/VIC407/2020|EPI_ISL_426709|2020-	····················	····················	····················	····················	····················	····················
A	2469	hCoV-19/Australia/VIC444/2020|EPI_ISL_426741|2020-	····················	····················	····················	····················	····················	····················
A	2471	hCoV-19/Australia/VIC449/2020|EPI_ISL_426745|2020-	····················	····················	····················	····················	····················	····················
A	2476	hCoV-19/Australia/VIC458/2020|EPI_ISL_426752|2020-	····················	····················	····················	····················	····················	····················
A	2481	hCoV-19/Australia/VIC471/2020|EPI_ISL_426763|2020-	····················	····················	····················	····················	····················	···············R····
A	2482	hCoV-19/Australia/VIC492/2020|EPI_ISL_426765|2020-	····················	·············A······	····················	····················	····················	····················
A	2484	hCoV-19/Australia/VIC494/2020|EPI_ISL_426767|2020-	····················	····················	····················	····················	····················	····················
A	2487	hCoV-19/Australia/VIC497/2020|EPI_ISL_426770|2020-	····················	····················	····················	····················	····················	····················
A	2488	hCoV-19/Australia/VIC498/2020|EPI_ISL_426771|2020-	····················	····················	····················	····················	····················	····················
A	2489	hCoV-19/Australia/VIC500/2020|EPI_ISL_426772|2020-	····················	····················	····················	····················	····················	····················
A	2490	hCoV-19/Australia/VIC502/2020|EPI_ISL_426773|2020-	····················	····················	····················	····················	····················	····················
A	2492	hCoV-19/Australia/VIC504/2020|EPI_ISL_426775|2020-	····················	····················	····················	····················	····················	····················
A	2493	hCoV-19/Australia/VIC506/2020|EPI_ISL_426777|2020-	····················	····················	····················	····················	····················	····················
A	2494	hCoV-19/Australia/VIC508/2020|EPI_ISL_426778|2020-	····················	····················	····················	····················	····················	····················
A	2495	hCoV-19/Australia/VIC509/2020|EPI_ISL_426779|2020-	····················	····················	····················	····················	····················	····················
A	2496	hCoV-19/Sweden/RV-FOI-6/2020|EPI_ISL_428201|2020-0	····················	····················	····················	····················	····················	····················
A	2497	hCoV-19/Poland/Pom1/2020|EPI_ISL_428209|2020-04-06	····················	····················	····················	····················	····················	····················
A	2498	hCoV-19/USA/WI-UW-164/2020|EPI_ISL_428260|2020-03-	····················	····················	····················	····················	····················	····················
A	2499	hCoV-19/USA/WI-UW-165/2020|EPI_ISL_428261|2020-03-	····················	····················	····················	····················	····················	····················
A	2500	hCoV-19/USA/WI-UW-166/2020|EPI_ISL_428262|2020-03-	····················	····················	····················	····················	····················	····················
A	2501	hCoV-19/USA/WI-UW-167/2020|EPI_ISL_428263|2020-03-	····················	····················	····················	····················	····················	····················
A	2502	hCoV-19/USA/WI-UW-168/2020|EPI_ISL_428264|2020-03-	····················	····················	····················	····················	····················	····················
A	2503	hCoV-19/USA/WI-UW-169/2020|EPI_ISL_428265|2020-03-	····················	····················	····················	····················	····················	····················
A	2504	hCoV-19/USA/WI-UW-170/2020|EPI_ISL_428266|2020-03-	····················	····················	····················	····················	····················	····················
A	2505	hCoV-19/USA/WI-UW-171/2020|EPI_ISL_428267|2020-03-	····················	····················	····················	····················	····················	····················
A	2506	hCoV-19/USA/WI-UW-172/2020|EPI_ISL_428268|2020-03-	····················	····················	····················	····················	····················	····················
A	2507	hCoV-19/USA/WI-UW-173/2020|EPI_ISL_428269|2020-03-	····················	····················	····················	····················	····················	····················
A	2508	hCoV-19/USA/WI-UW-174/2020|EPI_ISL_428270|2020-03-	····················	····················	····················	····················	····················	····················
A	2509	hCoV-19/USA/WI-UW-175/2020|EPI_ISL_428271|2020-03-	····················	····················	····················	····················	····················	····················
A	2510	hCoV-19/USA/WI-UW-176/2020|EPI_ISL_428272|2020-03-	····················	····················	····················	····················	····················	····················
A	2511	hCoV-19/USA/WI-UW-177/2020|EPI_ISL_428273|2020-03-	····················	····················	····················	····················	····················	····················
A	2512	hCoV-19/USA/WI-UW-178/2020|EPI_ISL_428274|2020-03-	····················	····················	····················	····················	····················	····················
A	2513	hCoV-19/USA/WI-UW-179/2020|EPI_ISL_428275|2020-03-	····················	····················	····················	····················	····················	····················
A	2514	hCoV-19/USA/WI-UW-180/2020|EPI_ISL_428276|2020-03-	····················	····················	····················	····················	····················	····················
A	2515	hCoV-19/USA/WI-UW-181/2020|EPI_ISL_428277|2020-03-	····················	····················	····················	····················	····················	····················
A	2516	hCoV-19/USA/WI-UW-182/2020|EPI_ISL_428278|2020-03-	····················	····················	····················	····················	····················	····················
A	2517	hCoV-19/USA/WI-UW-183/2020|EPI_ISL_428279|2020-03-	····················	····················	····················	····················	····················	····················
A	2518	hCoV-19/USA/WI-UW-184/2020|EPI_ISL_428280|2020-03-	····················	····················	····················	····················	····················	····················
A	2519	hCoV-19/USA/WI-UW-185/2020|EPI_ISL_428281|2020-03-	····················	····················	····················	····················	····················	····················
A	2520	hCoV-19/USA/WI-UW-186/2020|EPI_ISL_428282|2020-03-	····················	····················	····················	····················	····················	····················
A	2521	hCoV-19/USA/WI-UW-187/2020|EPI_ISL_428283|2020-03-	····················	····················	····················	····················	····················	····················
A	2522	hCoV-19/USA/WI-UW-188/2020|EPI_ISL_428284|2020-03-	····················	····················	····················	····················	····················	····················
A	2523	hCoV-19/USA/WI-UW-189/2020|EPI_ISL_428285|2020-03-	····················	····················	····················	····················	····················	····················
A	2524	hCoV-19/USA/WI-UW-190/2020|EPI_ISL_428286|2020-03-	····················	·········A··········	····················	····················	····················	····················
A	2525	hCoV-19/USA/WI-UW-191/2020|EPI_ISL_428287|2020-03-	····················	····················	····················	····················	····················	····················
A	2526	hCoV-19/USA/WI-UW-192/2020|EPI_ISL_428288|2020-03-	····················	····················	····················	····················	····················	····················
A	2527	hCoV-19/USA/WI-UW-193/2020|EPI_ISL_428289|2020-03-	····················	····················	····················	····················	····················	····················
A	2528	hCoV-19/USA/WI-UW-194/2020|EPI_ISL_428290|2020-03-	····················	····················	····················	····················	····················	····················
A	2529	hCoV-19/USA/WI-UW-195/2020|EPI_ISL_428291|2020-03-	····················	····················	····················	····················	····················	····················
A	2530	hCoV-19/USA/WI-UW-196/2020|EPI_ISL_428292|2020-03-	····················	····················	····················	····················	····················	····················
A	2531	hCoV-19/USA/WI-UW-197/2020|EPI_ISL_428293|2020-03-	····················	····················	····················	····················	····················	····················
A	2532	hCoV-19/USA/WI-UW-198/2020|EPI_ISL_428294|2020-03-	····················	····················	····················	····················	····················	····················
A	2533	hCoV-19/USA/WI-UW-199/2020|EPI_ISL_428295|2020-03-	····················	····················	····················	····················	····················	····················
A	2534	hCoV-19/USA/WI-UW-200/2020|EPI_ISL_428296|2020-03-	····················	····················	····················	····················	····················	····················
A	2535	hCoV-19/USA/WI-UW-201/2020|EPI_ISL_428297|2020-03-	····················	····················	····················	····················	····················	····················
A	2536	hCoV-19/USA/WI-UW-202/2020|EPI_ISL_428298|2020-03-	····················	····················	····················	····················	····················	····················
A	2537	hCoV-19/USA/WI-UW-203/2020|EPI_ISL_428299|2020-03-	····················	····················	····················	····················	····················	····················
A	2538	hCoV-19/Denmark/ALAB-SSI412A/2020|EPI_ISL_429508|2	····················	····················	····················	····················	····················	····················
A	2539	hCoV-19/Denmark/ALAB-SSI413/2020|EPI_ISL_429509|20	····················	····················	····················	····················	····················	····················
A	2540	hCoV-19/Denmark/ALAB-SSI437/2020|EPI_ISL_429530|20	····················	····················	····················	····················	····················	····················
A	2541	hCoV-19/Denmark/ALAB-SSI444/2020|EPI_ISL_429533|20	····················	····················	····················	····················	····················	····················
A	2542	hCoV-19/Denmark/ALAB-SSI446/2020|EPI_ISL_429534|20	····················	····················	····················	····················	····················	····················

A	2543	hCoV-19/Denmark/ALAB-SSI448/2020|EPI_ISL_429535|20	....	....	....	....	....	....	
A	2544	hCoV-19/Denmark/ALAB-SSI449/2020|EPI_ISL_429536|20	....	....	....	....	....	....	
A	2545	hCoV-19/Denmark/ALAB-SSI450/2020|EPI_ISL_429537|20	....	....	....	....	....	....	
A	2546	hCoV-19/Denmark/ALAB-SSI451/2020|EPI_ISL_429538|20	....	....	....	....	....	....	
A	2547	hCoV-19/Denmark/ALAB-SSI454/2020|EPI_ISL_429539|20	....	....	....	....	....	....	
A	2548	hCoV-19/Denmark/ALAB-SSI456/2020|EPI_ISL_429540|20	....	....	....	....	....	....	
A	2549	hCoV-19/Denmark/ALAB-SSI461/2020|EPI_ISL_429542|20	....	....	....	....	....	....	
A	2550	hCoV-19/Denmark/ALAB-SSI462/2020|EPI_ISL_429543|20	....	....	....	....	....	....	
A	2551	hCoV-19/Denmark/ALAB-SSI463/2020|EPI_ISL_429544|20	....	....	....	....	....	....	
A	2552	hCoV-19/Denmark/ALAB-SSI464/2020|EPI_ISL_429545|20	....	....	....	....	....	....	
A	2553	hCoV-19/Denmark/ALAB-SSI466/2020|EPI_ISL_429546|20	....	....	....	....	....	....	
A	2554	hCoV-19/Denmark/ALAB-SSI467/2020|EPI_ISL_429547|20	....	....	....	....	....	....	
A	2555	hCoV-19/Denmark/ALAB-SSI468/2020|EPI_ISL_429548|20	....	....	....	....	....	....	
A	2556	hCoV-19/Denmark/ALAB-SSI469/2020|EPI_ISL_429549|20	....	....	....	....	....	....	
A	2557	hCoV-19/Denmark/ALAB-SSI595/2020|EPI_ISL_429590|20	....	....	....	....	....	....	
A	2558	hCoV-19/USA/WA-UW-4546/2020|EPI_ISL_429598|2020-03	....	....	....	....	....	....	
A	2559	hCoV-19/Germany/HH-UMC-01/2020|EPI_ISL_450413|2020	....	....	....	....	....	....	
A	2560	hCoV-19/Beijing/IME-HZ01/2020|EPI_ISL_450444|2020-	....	....	....	....	....	....	
A	2561	hCoV-19/Wuhan/WH03/2020|EPI_ISL_406800|2020-01-01	....	....	....	....	....	....	
A	2562	hCoV-19/Wuhan/WH04/2020|EPI_ISL_406801|2020-01-05	....	....	....	....	....	....	
A	2563	hCoV-19/USA/MN56-MDH56/2020|EPI_ISL_417500|2020-03	....	....	....	....	....	....	
A	2564	hCoV-19/USA/MN57-MDH57/2020|EPI_ISL_417501|2020-03	....	....	....	....	....	....	
A	2565	hCoV-19/USA/MN58-MDH58/2020|EPI_ISL_417502|2020-03	....	....	....	....	....	....	
A	2566	hCoV-19/USA/MN59-MDH59/2020|EPI_ISL_417503|2020-03	....	....	....	....	....	....	
A	2567	hCoV-19/USA/WI-UW-15/2020|EPI_ISL_417504|2020-03-1	....	....	....	....	....	....	
A	2568	hCoV-19/USA/WI-UW-11/2020|EPI_ISL_417505|2020-03-1	....	....	....	....	....	....	
A	2569	hCoV-19/USA/WI-UW-12/2020|EPI_ISL_417506|2020-03-1	....	....	....	....	....	....	
A	2570	hCoV-19/Taiwan/CGMH-CGU-07/2020|EPI_ISL_417520|202	....	....	....	....	....	....	
A	2571	hCoV-19/Taiwan/CGMH-CGU-08/2020|EPI_ISL_417521|202	....	....	....	....	....	....	
A	2572	hCoV-19/Taiwan/CGMH-CGU-09/2020|EPI_ISL_417522|202	....	....	....	....	....	....	
A	2573	hCoV-19/Taiwan/CGMH-CGU-10/2020|EPI_ISL_417523|202	....	....	....	....	....	....	
A	2574	hCoV-19/Taiwan/CGMH-CGU-11/2020|EPI_ISL_417524|202	....	....	....	....	....	....	
A	2575	hCoV-19/Luxembourg/LNS2848109/2020|EPI_ISL_417527|	....	....	....	....	....	....	
A	2576	hCoV-19/Luxembourg/LNS3694003/2020|EPI_ISL_417528|	....	....	....	....	....	....	
A	2577	hCoV-19/Iceland/184/2020|EPI_ISL_417550|2020-03-16	....	....	....	....	....	....	
A	2578	hCoV-19/Iceland/187/2020|EPI_ISL_417551|2020-03-16	....	....	....	....	....	....	
A	2579	hCoV-19/Iceland/2/2020|EPI_ISL_417552|2020-03-16	....	....	....	....	....	....	
A	2580	hCoV-19/Iceland/220/2020|EPI_ISL_417553|2020-03-16	....	....	....	....	....	....	
A	2581	hCoV-19/Iceland/227/2020|EPI_ISL_417555|2020-03-17	....	....	....	....	....	....	
A	2582	hCoV-19/Iceland/228/2020|EPI_ISL_417556|2020-03-17	....	....	....	....	....	....	
A	2583	hCoV-19/Iceland/229/2020|EPI_ISL_417557|2020-03-17	....	....	....	....	....	....	
A	2584	hCoV-19/Iceland/230/2020|EPI_ISL_417558|2020-03-17	....	....	....	....	....	....	
A	2585	hCoV-19/Iceland/242/2020|EPI_ISL_417570|2020-03-17	....	....	....	....	....	....	
A	2586	hCoV-19/Iceland/243/2020|EPI_ISL_417571|2020-03-17	....	....	....	....	....	....	
A	2587	hCoV-19/Iceland/244/2020|EPI_ISL_417572|2020-03-17	....	....	....	....	....	....	
A	2588	hCoV-19/Iceland/245/2020|EPI_ISL_417573|2020-03-16	....	....	....	....	....	....	
A	2589	hCoV-19/Iceland/246/2020|EPI_ISL_417574|2020-03-17	....	....	....	....	....	....	
A	2590	hCoV-19/Iceland/247/2020|EPI_ISL_417575|2020-03-17	....	....	....	....	....	....	
A	2591	hCoV-19/Iceland/248/2020|EPI_ISL_417576|2020-03-17	....	....	....	....	....	....	
A	2592	hCoV-19/Iceland/249/2020|EPI_ISL_417577|2020-03-17	....	....	....	....	....	....	
A	2593	hCoV-19/Iceland/252/2020|EPI_ISL_417580|2020-03-17	....	....	....	....	....	....	
A	2594	hCoV-19/Iceland/253/2020|EPI_ISL_417581|2020-03-17	....	....	....	....	....	....	
A	2595	hCoV-19/Iceland/254/2020|EPI_ISL_417582|2020-03-17	....	....	....	....	....	....	
A	2596	hCoV-19/Iceland/255/2020|EPI_ISL_417583|2020-03-17	....	....	....	....	....	....	
A	2597	hCoV-19/Iceland/256/2020|EPI_ISL_417584|2020-03-17	....	....	....	....	....	....	
A	2598	hCoV-19/Iceland/257/2020|EPI_ISL_417585|2020-03-17	....	....	....	....	....	....	
A	2599	hCoV-19/Iceland/258/2020|EPI_ISL_417586|2020-03-17	....	....	....	....	....	....	
A	2600	hCoV-19/Iceland/259/2020|EPI_ISL_417587|2020-03-17	....	....	....	....	....	....	
A	2601	hCoV-19/Iceland/260/2020|EPI_ISL_417588|2020-03-17	....	....	....	....	....	....	
A	2602	hCoV-19/Iceland/261/2020|EPI_ISL_417589|2020-03-17	....	....	....	....	....	....	
A	2603	hCoV-19/Iceland/262/2020|EPI_ISL_417590|2020-03-18	....	....	....	....	....	....	
A	2604	hCoV-19/Iceland/263/2020|EPI_ISL_417591|2020-03-17	....	....	....	....	....	....	
A	2605	hCoV-19/Iceland/264/2020|EPI_ISL_417592|2020-03-17	....	...................A	....	....	....	....	1
A	2606	hCoV-19/Iceland/267/2020|EPI_ISL_417595|2020-03-17	....	....	....	....	....	....	
A	2607	hCoV-19/Iceland/268/2020|EPI_ISL_417596|2020-03-17	....	....	....	....	....	....	
A	2608	hCoV-19/Iceland/269/2020|EPI_ISL_417597|2020-03-17	....	....	....	....	....	....	
A	2609	hCoV-19/Iceland/270/2020|EPI_ISL_417598|2020-03-17	....	....	....	....	....	....	
A	2610	hCoV-19/Iceland/271/2020|EPI_ISL_417599|2020-03-17	....	....	....	....	....	....	
A	2611	hCoV-19/Belgium/CS-031052/2020|EPI_ISL_418800|2020	....	....	....	....	....	....	
A	2612	hCoV-19/Australia/QLDID923/2020|EPI_ISL_418801|202	....	....	....	....	....	....	
A	2613	hCoV-19/Australia/QLDID924/2020|EPI_ISL_418802|202	....	....	....	....	....	....	
A	2614	hCoV-19/Australia/QLDID925/2020|EPI_ISL_418803|202	....	....	....	....	....	....	
A	2615	hCoV-19/Australia/QLDID926/2020|EPI_ISL_418804|202	....	....	....	....	....	....	
A	2616	hCoV-19/Belgium/VPE-030650/2020|EPI_ISL_418805|202	....	....	....	....	....	....	
A	2617	hCoV-19/Belgium/BG-030551/2020|EPI_ISL_418806|2020	....	....	....	....	....	....	
A	2618	hCoV-19/Australia/QLDID927/2020|EPI_ISL_418807|202	....	....	....	....	....	....	
A	2619	hCoV-19/Australia/QLDID928/2020|EPI_ISL_418808|202	....	....	....	....	....	....	
A	2620	hCoV-19/Japan/UT-NCGM02/2020|EPI_ISL_418809|2020-0	....	....	....	....	....	....	

A 2621 hCoV-19/Canada/NB_6/2020|EPI_ISL_418811|2020-03-12 ···················· ···················· ····················· ···················· ···················· ····················
A 2622 hCoV-19/Canada/MB_8/2020|EPI_ISL_418812|2020-03-12 ···················· ···················· ····················· ···················· ···················· ····················
A 2623 hCoV-19/Canada/MB_10/2020|EPI_ISL_418813|2020-03-1 ···················· ···················· ····················· ···················· ···················· ····················
A 2624 hCoV-19/Canada/NS_13/2020|EPI_ISL_418814|2020-03-1 ···················· ···················· ····················· ···················· ···················· ····················
A 2625 hCoV-19/Hong Kong/HKPU36-0702/2020|EPI_ISL_418815| ···················· ···················· ····················· ···················· ···················· ····················
A 2626 hCoV-19/Canada/BC_0443574/2020|EPI_ISL_418816|2020 ···················· ···················· ····················· ···················· ···················· ····················
A 2627 hCoV-19/Canada/BC_0554880/2020|EPI_ISL_418817|2020 ···················· ···················· ····················· ···················· ···················· ····················
A 2628 hCoV-19/Canada/BC_1318414/2020|EPI_ISL_418818|2020 ···················· ···················· ····················· ···················· ···················· ····················
A 2629 hCoV-19/Canada/BC_3808524/2020|EPI_ISL_418819|2020 ···················· ···················· ····················· ···················· ···················· ····················
A 2630 hCoV-19/Canada/BC_3842755/2020|EPI_ISL_418820|2020 ···················· ···················· ····················· ···················· ···················· ····················
A 2631 hCoV-19/Canada/BC_3968175/2020|EPI_ISL_418821|2020 ···················· ···················· ····················· ···················· ···················· ····················
A 2632 hCoV-19/Canada/BC_3972884/2020|EPI_ISL_418822|2020 ···················· ···················· ····················· ···················· ···················· ····················
A 2633 hCoV-19/Canada/BC_3989992/2020|EPI_ISL_418823|2020 ···················· ···················· ····················· ···················· ···················· ····················
A 2634 hCoV-19/Canada/BC_4078583/2020|EPI_ISL_418824|2020 ···················· ···················· ····················· ···················· ···················· ····················
A 2635 hCoV-19/Canada/BC_4118226/2020|EPI_ISL_418825|2020 ···················· ···················· ····················· ···················· ···················· ····················
A 2636 hCoV-19/Canada/BC_4122951/2020|EPI_ISL_418826|2020 ···················· ···················· ····················· ···················· ···················· ····················
A 2637 hCoV-19/Canada/BC_4540462/2020|EPI_ISL_418829|2020 ···················· ···················· ····················· ···················· ···················· ····················
A 2638 hCoV-19/Canada/BC_4799711/2020|EPI_ISL_418830|2020 ···················· ···················· ····················· ···················· ···················· ····················
A 2639 hCoV-19/Canada/BC_5275718/2020|EPI_ISL_418831|2020 ···················· ···················· ····················· ···················· ···················· ····················
A 2640 hCoV-19/Canada/BC_5282984/2020|EPI_ISL_418832|2020 ···················· ···················· ····················· ···················· ···················· ····················
A 2641 hCoV-19/Canada/BC_5306970/2020|EPI_ISL_418833|2020 ···················· ···················· ····················· ···················· ···················· ····················
A 2642 hCoV-19/Canada/BC_5522039/2020|EPI_ISL_418834|2020 ···················· ···················· ····················· ···················· ···················· ····················
A 2643 hCoV-19/Canada/BC_5979789/2020|EPI_ISL_418835|2020 ···················· ···················· ····················· ···················· ···················· ····················
A 2644 hCoV-19/Canada/BC_6004567/2020|EPI_ISL_418836|2020 ···················· ···················· ····················· ···················· ···················· ····················
A 2645 hCoV-19/Canada/BC_6129127/2020|EPI_ISL_418837|2020 ···················· ···················· ····················· ···················· ···················· ····················
A 2646 hCoV-19/Canada/BC_8606204/2020|EPI_ISL_418850|2020 ···················· ···················· ····················· ···················· ···················· ····················
A 2647 hCoV-19/Canada/BC_8622445/2020|EPI_ISL_418851|2020 ···················· ···················· ····················· ···················· ···················· ····················
A 2648 hCoV-19/Canada/BC_8718874/2020|EPI_ISL_418852|2020 ···················· ···················· ····················· ···················· ···················· ····················
A 2649 hCoV-19/Canada/BC_8894200/2020|EPI_ISL_418853|2020 ···················· ···················· ····················· ···················· ···················· ····················
A 2650 hCoV-19/Canada/BC_8896915/2020|EPI_ISL_418854|2020 ···················· ···················· ····················· ···················· ···················· ····················
A 2651 hCoV-19/Canada/BC_8897642/2020|EPI_ISL_418855|2020 ···················· ···················· ····················· ···················· ···················· ····················
A 2652 hCoV-19/Canada/BC_9446031/2020|EPI_ISL_418858|2020 ···················· ···················· ····················· ···················· ···················· ····················
A 2653 hCoV-19/USA/WA-UW313/2020|EPI_ISL_418880|2020-03-1 ···················· ···················· ····················· ···················· ···················· ····················
A 2654 hCoV-19/USA/WA-UW314/2020|EPI_ISL_418881|2020-03-1 ···················· ···················· ····················· ···················· ···················· ····················
A 2655 hCoV-19/USA/WA-UW315/2020|EPI_ISL_418882|2020-03-1 ···················· ···················· ····················· ···················· ···················· ····················
A 2656 hCoV-19/USA/WA-UW316/2020|EPI_ISL_418883|2020-03-1 ···················· ···················· ····················· ···················· ···················· ····················
A 2657 hCoV-19/USA/WA-UW317/2020|EPI_ISL_418884|2020-03-1 ···················· ···················· ····················· ···················· ···················· ····················
A 2658 hCoV-19/USA/WA-UW319/2020|EPI_ISL_418886|2020-03-1 ···················· ···················· ····················· ···················· ···················· ····················
A 2659 hCoV-19/USA/WA-UW320/2020|EPI_ISL_418887|2020-03-1 ···················· ···················· ····················· ···················· ···················· ····················
A 2660 hCoV-19/USA/WA-UW321/2020|EPI_ISL_418888|2020-03-1 ···················· ···················· ····················· ···················· ···················· ····················
A 2661 hCoV-19/USA/WA-UW322/2020|EPI_ISL_418889|2020-03-1 ···················· ···················· ····················· ···················· ···················· ····················
A 2662 hCoV-19/Wales/PHWC-25779/2020|EPI_ISL_422039|2020- ···················· ····A··············· ····················· ···················· ···················· ····················
A 2663 hCoV-19/Wales/PHWC-26D1C/2020|EPI_ISL_422040|2020- ···················· ····A··············· ····················· ···················· ···················· ····················
A 2664 hCoV-19/Wales/PHWC-25C3E/2020|EPI_ISL_422041|2020- ···················· ···················· ····················· ···················· ···················· ····················
A 2665 hCoV-19/Wales/PHWC-264BD/2020|EPI_ISL_422042|2020- ···················· ···················· ····················· ···················· ···················· ····················
A 2666 hCoV-19/Wales/PHWC-269DC/2020|EPI_ISL_422043|2020- ···················· ···················· ····················· ···················· ···················· ····················
A 2667 hCoV-19/Wales/PHWC-26E19/2020|EPI_ISL_422044|2020- ···················· ···················· ····················· ···················· ···················· ····················
A 2668 hCoV-19/Wales/PHWC-25AF8/2020|EPI_ISL_422045|2020- ···················· ···················· ····················· ···················· ···················· ····················
A 2669 hCoV-19/Wales/PHWC-2666C/2020|EPI_ISL_422046|2020- ···················· ···················· ····················· ···················· ···················· ····················
A 2670 hCoV-19/Wales/PHWC-26F34/2020|EPI_ISL_422047|2020- ···················· ···················· ····················· ···················· ···················· ····················
A 2671 hCoV-19/Wales/PHWC-26918/2020|EPI_ISL_422048|2020- ···················· ···················· ····················· ···················· ···················· ····················
A 2672 hCoV-19/Wales/PHWC-2575B/2020|EPI_ISL_422049|2020- ···················· ····A··············· ····················· ···················· ···················· ····················
A 2673 hCoV-19/Wales/PHWC-26DEF/2020|EPI_ISL_422050|2020- ···················· ····A··············· ····················· ···················· ···················· ····················
A 2674 hCoV-19/Wales/PHWC-26A51/2020|EPI_ISL_422051|2020- ···················· ····A··············· ····················· ···················· ···················· ····················
A 2675 hCoV-19/Wales/PHWC-26884/2020|EPI_ISL_422052|2020- ···················· ···················· ····················· ···················· ···················· ····················
A 2676 hCoV-19/Wales/PHWC-25ABC/2020|EPI_ISL_422053|2020- ···················· ···················· ····················· ···················· ···················· ····················
A 2677 hCoV-19/Wales/PHWC-26F9E/2020|EPI_ISL_422054|2020- ···················· ···················· ····················· ···················· ···················· ····················
A 2678 hCoV-19/Wales/PHWC-25E47/2020|EPI_ISL_422055|2020- ···················· ····A··············· ····················· ···················· ···················· ····················
A 2679 hCoV-19/Wales/PHWC-26CC4/2020|EPI_ISL_422056|2020- ···················· ····A··············· ····················· ···················· ···················· ····················
A 2680 hCoV-19/Wales/PHWC-268A2/2020|EPI_ISL_422057|2020- ···················· ···················· ····················· ···················· ···················· ····················
A 2681 hCoV-19/Wales/PHWC-26E91/2020|EPI_ISL_422058|2020- ···················· ···················· ····················· ···················· ···················· ····················
A 2682 hCoV-19/Wales/PHWC-26FE9/2020|EPI_ISL_422059|2020- ···················· ···················· ····················· ···················· ···················· ····················
A 2683 hCoV-19/Wales/PHWC-25FEA/2020|EPI_ISL_422060|2020- ···················· ···················· ····················· ···················· ···················· ····················
A 2684 hCoV-19/Wales/PHWC-26E82/2020|EPI_ISL_422061|2020- ···················· ···················· ····················· ···················· ···················· ····················
A 2685 hCoV-19/Wales/PHWC-269AF/2020|EPI_ISL_422062|2020- ···················· ·A·················· ····················· ···················· ···················· ····················
A 2686 hCoV-19/Wales/PHWC-25B6E/2020|EPI_ISL_422063|2020- ···················· ···················· ····················· ···················· ···················· ····················
A 2687 hCoV-19/Wales/PHWC-25C01/2020|EPI_ISL_422064|2020- ···················· ···················· ····················· ···················· ···················· ····················
A 2688 hCoV-19/Wales/PHWC-2566D/2020|EPI_ISL_422065|2020- ···················· ···················· ····················· ···················· ···················· ····················
A 2689 hCoV-19/Wales/PHWC-2573D/2020|EPI_ISL_422066|2020- ···················· ····A··············· ····················· ···················· ···················· ····················
A 2690 hCoV-19/Wales/PHWC-25973/2020|EPI_ISL_422067|2020- ···················· ···················· ····················· ···················· ···················· ····················
A 2691 hCoV-19/Wales/PHWC-26EFB/2020|EPI_ISL_422068|2020- ···················· ····A··············· ····················· ···················· ···················· ····················
A 2692 hCoV-19/Wales/PHWC-270A9/2020|EPI_ISL_422069|2020- ···················· ···················· ····················· ···················· ···················· ····················
A 2693 hCoV-19/Wales/PHWC-26839/2020|EPI_ISL_422070|2020- ···················· ···················· ····················· ···················· ···················· ····················
A 2694 hCoV-19/Wales/PHWC-257B5/2020|EPI_ISL_422071|2020- ···················· ···················· ····················· ···················· ···················· ····················
A 2695 hCoV-19/Wales/PHWC-26F52/2020|EPI_ISL_422072|2020- ···················· ···················· ····················· ···················· ···················· ····················
A 2696 hCoV-19/Wales/PHWC-260AA/2020|EPI_ISL_422073|2020- ···················· ···················· ····················· ···················· ···················· ····················
A 2697 hCoV-19/Wales/PHWC-26954/2020|EPI_ISL_422074|2020- ···················· ·A·················· ····················· ···················· ···················· ····················
A 2698 hCoV-19/Wales/PHWC-26E46/2020|EPI_ISL_422075|2020- ···················· ···················· ····················· ···················· ···················· ····················

A	2699	hCoV-19/Wales/PHWC-26D3A/2020|EPI_ISL_422076|2020-	....	....	....	....	....	....
A	2700	hCoV-19/Wales/PHWC-26A42/2020|EPI_ISL_422077|2020-	....	....A...............	....	....	....	....
A	2701	hCoV-19/Wales/PHWC-26013/2020|EPI_ISL_422078|2020-	....	....A...............	....	....	....	....
A	2702	hCoV-19/Wales/PHWC-26E37/2020|EPI_ISL_422079|2020-	....	....	....	....	....	....
A	2703	hCoV-19/Wales/PHWC-26E55/2020|EPI_ISL_422080|2020-	....	....	....	....	....	....
A	2704	hCoV-19/Wales/PHWC-26EBF/2020|EPI_ISL_422081|2020-	....	....	....	....	....	....
A	2705	hCoV-19/Wales/PHWC-26BD6/2020|EPI_ISL_422082|2020-	....	....	....	....	....	....
A	2706	hCoV-19/Wales/PHWC-2624A/2020|EPI_ISL_422083|2020-	....	....	....	....	....	....
A	2707	hCoV-19/Wales/PHWC-26DB2/2020|EPI_ISL_422084|2020-	....	....	....	....	....	....
A	2708	hCoV-19/Wales/PHWC-25FAE/2020|EPI_ISL_422085|2020-	....	....	....	....	....	....
A	2709	hCoV-19/Wales/PHWC-2708B/2020|EPI_ISL_422086|2020-	....	....	....	....	....	....
A	2710	hCoV-19/Wales/PHWC-26CA6/2020|EPI_ISL_422087|2020-	....	....A...............	....	....	....	....
A	2711	hCoV-19/Wales/PHWC-26DC1/2020|EPI_ISL_422088|2020-	....	....	....	....	....	....
A	2712	hCoV-19/Wales/PHWC-26DA3/2020|EPI_ISL_422089|2020-	....	....A...............	....	....	....	....
A	2713	hCoV-19/Wales/PHWC-26ECE/2020|EPI_ISL_422090|2020-	....	....	....	....	....	....
A	2714	hCoV-19/Wales/PHWC-264DB/2020|EPI_ISL_422091|2020-	....	....	....	....	....	....
A	2715	hCoV-19/Wales/PHWC-26857/2020|EPI_ISL_422092|2020-	....	....	....	....	....	....
A	2716	hCoV-19/Wales/PHWC-2707C/2020|EPI_ISL_422093|2020-	....	....	....	....	....	....
A	2717	hCoV-19/Wales/PHWC-2617A/2020|EPI_ISL_422094|2020-	....	....	....	....	....	....
A	2718	hCoV-19/Wales/PHWC-2705E/2020|EPI_ISL_422095|2020-	....	....	....	....	....	....
A	2719	hCoV-19/Wales/PHWC-2675A/2020|EPI_ISL_422096|2020-	....	....	....	....	....	....
A	2720	hCoV-19/Wales/PHWC-26D0D/2020|EPI_ISL_422097|2020-	....	....	....	....	....	....
A	2721	hCoV-19/Wales/PHWC-26848/2020|EPI_ISL_422098|2020-	....	....	....	....	....	....
A	2722	hCoV-19/Wales/PHWC-27021/2020|EPI_ISL_422099|2020-	....	....	....	....	....	....
A	2723	hCoV-19/England/20140001704/2020|EPI_ISL_423370|20	....	....	....	....	....	....
A	2724	hCoV-19/England/20140002204/2020|EPI_ISL_423371|20	....	....	....	....	....	....
A	2725	hCoV-19/England/20140002804/2020|EPI_ISL_423373|20	....	....	....	....	....	....
A	2726	hCoV-19/England/20140003402/2020|EPI_ISL_423375|20	....	....	....	....	....	....
A	2727	hCoV-19/England/20140003404/2020|EPI_ISL_423376|20	....	....	....	....	....	....N...............
A	2728	hCoV-19/England/20140004004/2020|EPI_ISL_423379|20	....	....	....	....	....	....
A	2730	hCoV-19/England/20140006204/2020|EPI_ISL_423381|20	....	....	....	....	....	....
A	2731	hCoV-19/England/20140007004/2020|EPI_ISL_423382|20	....	....	....	....	....	....
A	2732	hCoV-19/England/20140007602/2020|EPI_ISL_423383|20	....	....	....	....	....	....
A	2734	hCoV-19/England/20140009204/2020|EPI_ISL_423385|20	....	....	....	....	....	....
A	2736	hCoV-19/England/20140009404/2020|EPI_ISL_423387|20	....	....	....	....	....	....
A	2738	hCoV-19/England/20140064004/2020|EPI_ISL_423393|20	....	....	....	....	....	....
A	2739	hCoV-19/England/20140064504/2020|EPI_ISL_423394|20	....	....	....	....	....	....
A	2740	hCoV-19/England/20140064704/2020|EPI_ISL_423395|20	....	....	....	....	....	....
A	2741	hCoV-19/England/20140064804/2020|EPI_ISL_423396|20	....	....	....	....	....	....
A	2742	hCoV-19/England/20140066004/2020|EPI_ISL_423398|20	....	....	....	....	....	....
A	2743	hCoV-19/England/20140066204/2020|EPI_ISL_423399|20	....	....	....	....	....	....
A	2744	hCoV-19/Taiwan/TSGH-12/2020|EPI_ISL_428229|2020-03	....	....	....	....	....	....
A	2745	hCoV-19/Taiwan/TSGH-13/2020|EPI_ISL_428230|2020-03	....	....	....	....	....	....
A	2746	hCoV-19/Taiwan/TSGH-14/2020|EPI_ISL_428231|2020-03	....	....	....	....	....	....
A	2747	hCoV-19/USA/WI-UW-156/2020|EPI_ISL_428252|2020-03-	....	....	....	....	....	....
A	2748	hCoV-19/USA/WI-UW-157/2020|EPI_ISL_428253|2020-03-	....	....	....	....	....	....
A	2749	hCoV-19/USA/WI-UW-158/2020|EPI_ISL_428254|2020-03-	....	....	....	....	....	....
A	2750	hCoV-19/USA/WI-UW-159/2020|EPI_ISL_428255|2020-03-	....	....	....	....	....	....
A	2751	hCoV-19/USA/WI-UW-160/2020|EPI_ISL_428256|2020-03-	....	....	....	....	....	....
A	2752	hCoV-19/USA/WI-UW-161/2020|EPI_ISL_428257|2020-03-	....	....	....	....	....	....
A	2753	hCoV-19/USA/WI-UW-162/2020|EPI_ISL_428258|2020-03-	....	....	....	....	....	....
A	2754	hCoV-19/USA/WI-UW-163/2020|EPI_ISL_428259|2020-03-	....	....	....	....	....	....
A	2755	hCoV-19/Denmark/ALAB-SSI407/2020|EPI_ISL_429501|20	....	....	....	....	....	....
A	2756	hCoV-19/Denmark/ALAB-SSI408/2020|EPI_ISL_429502|20	....	....	....	....	....	....
A	2757	hCoV-19/Denmark/ALAB-SSI409/2020|EPI_ISL_429503|20	....	....	....	....	....	....
A	2758	hCoV-19/Denmark/ALAB-SSI411/2020|EPI_ISL_429505|20	....	....	....	....	....	....
A	2759	hCoV-19/Denmark/ALAB-SSI411A/2020|EPI_ISL_429506|2	....	....	....	....	....	....
A	2760	hCoV-19/Denmark/ALAB-SSI412/2020|EPI_ISL_429507|20	....	....	....	....	....	....
A	2761	hCoV-19/Denmark/ALAB-SSI413A/2020|EPI_ISL_429510|2	....	....	....	....	....	....
A	2762	hCoV-19/Denmark/ALAB-SSI414/2020|EPI_ISL_429511|20	....	....	....	....	....	....
A	2763	hCoV-19/Denmark/ALAB-SSI414A/2020|EPI_ISL_429512|2	....	....	....	....	....	....
A	2764	hCoV-19/Denmark/ALAB-SSI416/2020|EPI_ISL_429513|20	....	....	....	....	....	....
A	2765	hCoV-19/Denmark/ALAB-SSI417/2020|EPI_ISL_429514|20	....	....	....	....	....	....
A	2766	hCoV-19/Denmark/ALAB-SSI418/2020|EPI_ISL_429516|20	....	....	....	....	....	....
A	2767	hCoV-19/Denmark/ALAB-SSI419/2020|EPI_ISL_429517|20	....	....	....	....	....	....
A	2768	hCoV-19/Denmark/ALAB-SSI420/2020|EPI_ISL_429518|20	....	....	....	....	....	....
A	2769	hCoV-19/Denmark/ALAB-SSI420A/2020|EPI_ISL_429519|2	....	....	....	....	....	....
A	2770	hCoV-19/Denmark/ALAB-SSI423/2020|EPI_ISL_429520|20	....	....	....	....	....	....
A	2771	hCoV-19/Denmark/ALAB-SSI424/2020|EPI_ISL_429521|20	....	....	....	....	....	....
A	2772	hCoV-19/Denmark/ALAB-SSI425A/2020|EPI_ISL_429524|2	....	....	....	....	....	....
A	2773	hCoV-19/Denmark/ALAB-SSI426/2020|EPI_ISL_429525|20	....	....	....	....	....	....
A	2774	hCoV-19/Denmark/ALAB-SSI430/2020|EPI_ISL_429526|20	....	....	....	....	....	....
A	2775	hCoV-19/Denmark/ALAB-SSI431/2020|EPI_ISL_429527|20	....	....	....	....	....	....
A	2776	hCoV-19/Denmark/ALAB-SSI432/2020|EPI_ISL_429528|20	....	....	....	....	....	....
A	2777	hCoV-19/Denmark/ALAB-SSI434/2020|EPI_ISL_429529|20	....	....	....	....	....	....
A	2778	hCoV-19/Denmark/ALAB-SSI470/2020|EPI_ISL_429550|20	....	....	....	....	....	....
A	2779	hCoV-19/Denmark/ALAB-SSI471/2020|EPI_ISL_429551|20	....	....	....	....	....	....
A	2780	hCoV-19/Denmark/ALAB-SSI472/2020|EPI_ISL_429552|20	....	....	....	....	....	....

A	2781	hCoV-19/Denmark/ALAB-SSI473/2020|EPI_ISL_429553|20	....	....	....	....	....	....
A	2782	hCoV-19/Denmark/ALAB-SSI474/2020|EPI_ISL_429554|20	....	....	....	....	....	....
A	2783	hCoV-19/Denmark/ALAB-SSI475/2020|EPI_ISL_429555|20	....	....	....	....	....	....
A	2784	hCoV-19/Denmark/ALAB-SSI477/2020|EPI_ISL_429556|20	....	....	....	....	....	....
A	2785	hCoV-19/Denmark/ALAB-SSI478/2020|EPI_ISL_429557|20	....	....	....	....	....	....
A	2786	hCoV-19/Denmark/ALAB-SSI479/2020|EPI_ISL_429558|20	....	....	....	....	....	....
A	2787	hCoV-19/Denmark/ALAB-SSI480/2020|EPI_ISL_429559|20	....	....	....	....	....	....
A	2788	hCoV-19/Denmark/ALAB-SSI481/2020|EPI_ISL_429560|20	....	....	....	....	....	....
A	2789	hCoV-19/Denmark/ALAB-SSI482/2020|EPI_ISL_429561|20	....	....	....	....	....	....
A	2790	hCoV-19/Denmark/ALAB-SSI483/2020|EPI_ISL_429562|20	....	....	....	....	....	....
A	2791	hCoV-19/Denmark/ALAB-SSI484/2020|EPI_ISL_429563|20	....	....	....	....	....	....
A	2792	hCoV-19/Denmark/ALAB-SSI486/2020|EPI_ISL_429564|20	....	....	....	....	....	....
A	2793	hCoV-19/Denmark/ALAB-SSI488/2020|EPI_ISL_429565|20	....	....	....	....	....	....
A	2794	hCoV-19/Denmark/ALAB-SSI489/2020|EPI_ISL_429566|20	....	....	....	....	....	....
A	2795	hCoV-19/Denmark/ALAB-SSI490/2020|EPI_ISL_429567|20	....	....	....	....	....	....
A	2796	hCoV-19/Denmark/ALAB-SSI494/2020|EPI_ISL_429569|20	....	....	....	....	....	....
A	2797	hCoV-19/Denmark/ALAB-SSI495/2020|EPI_ISL_429570|20	....	....	....	....	....	....
A	2798	hCoV-19/Denmark/ALAB-SSI497/2020|EPI_ISL_429571|20	....	....	....	....	....	....
A	2799	hCoV-19/Denmark/ALAB-SSI501/2020|EPI_ISL_429572|20	....	....	....	....	....	....
A	2800	hCoV-19/Denmark/ALAB-SSI502/2020|EPI_ISL_429573|20	....	....	....	....	....	....
A	2801	hCoV-19/Denmark/ALAB-SSI506/2020|EPI_ISL_429574|20	....	....	....	....	....	....
A	2802	hCoV-19/Denmark/ALAB-SSI507/2020|EPI_ISL_429575|20	....	....	....	....	....	....
A	2803	hCoV-19/Denmark/ALAB-SSI509/2020|EPI_ISL_429577|20	....	....	....	....	....	....
A	2804	hCoV-19/Denmark/ALAB-SSI510/2020|EPI_ISL_429578|20	....	....	....	....	....	....
A	2805	hCoV-19/Denmark/ALAB-SSI511/2020|EPI_ISL_429579|20	....	....	....	....	....	....
A	2806	hCoV-19/Denmark/ALAB-SSI512/2020|EPI_ISL_429580|20	....	....	....	....	....	....
A	2807	hCoV-19/Denmark/ALAB-SSI513/2020|EPI_ISL_429581|20	....	....	....	....	....	....
A	2808	hCoV-19/Denmark/ALAB-SSI514/2020|EPI_ISL_429582|20	....	....	....	....	....	....
A	2809	hCoV-19/Denmark/ALAB-SSI515/2020|EPI_ISL_429583|20	....	....	....	....	....	....
A	2810	hCoV-19/Denmark/ALAB-SSI518/2020|EPI_ISL_429584|20	....	....	....	....	....	....
A	2811	hCoV-19/Denmark/ALAB-SSI520/2020|EPI_ISL_429585|20	....	....	....	....	....	....
A	2812	hCoV-19/Denmark/ALAB-SSI521/2020|EPI_ISL_429586|20	....	....	....	....	....	....
A	2813	hCoV-19/Denmark/ALAB-SSI522/2020|EPI_ISL_429587|20	....	....	....	....	....	....
A	2814	hCoV-19/Denmark/ALAB-SSI523/2020|EPI_ISL_429588|20	....	....	....	....	....	....
A	2815	hCoV-19/Denmark/ALAB-SSI592/2020|EPI_ISL_429589|20	....	....	....	....	....	....
A	2816	hCoV-19/Beijing/Beijing-01/2020|EPI_ISL_514752|202	....	....	....	....	....	....
A	2817	hCoV-19/Germany/BY-ChVir-929/2020|EPI_ISL_406862|2	....	....	....	....	....	....
A	2818	hCoV-19/USA/MA1/2020|EPI_ISL_409067|2020-01-29	....	.A..................	....	....	....	....
A	2819	hCoV-19/USA/CA-CDC-6/2020|EPI_ISL_410044|2020-01-2	....	....	....	....	....	....
A	2820	hCoV-19/USA/IL2/2020|EPI_ISL_410045|2020-01-28	....	....	....	....	....	....
A	2821	hCoV-19/USA/CA-CDC-9/2020|EPI_ISL_412862|2020-02-2	....	....	....	....	....	....
A	2822	hCoV-19/South Korea/KCDC05/2020|EPI_ISL_412869|202	....	....	....	....	....	....
A	2823	hCoV-19/South Korea/KCDC06/2020|EPI_ISL_412870|202	....	....	....	....	....	....
A	2824	hCoV-19/South Korea/KCDC07/2020|EPI_ISL_412871|202	....	....	....	....	....	....
A	2825	hCoV-19/South Korea/KCDC12/2020|EPI_ISL_412872|202	....	....	....	....	....	....
A	2826	hCoV-19/South Korea/KCDC24/2020|EPI_ISL_412873|202	....	....	....	....	....	....
A	2827	hCoV-19/Switzerland/TI-SNRCI-29919486/2020|EPI_ISL	....	....	....	....	....	....
A	2828	hCoV-19/Switzerland/GE-SNRCI-29933895/2020|EPI_ISL	....	....	....	....	....	....
A	2829	hCoV-19/Switzerland/AG-SNRCI-29940361/2020|EPI_ISL	....	....	....	....	....	....
A	2830	hCoV-19/Shanghai/SH0022/2020|EPI_ISL_416331|2020-0	....	....	....	....	....	....
A	2831	hCoV-19/USA/WI-UW-23/2020|EPI_ISL_417507|2020-03-1	....	....	....	....	....	....
A	2832	hCoV-19/USA/WI-UW-21/2020|EPI_ISL_417508|2020-03-1	....	....	....	....	....	....
A	2833	hCoV-19/USA/WI-UW-16/2020|EPI_ISL_417509|2020-03-1	....	....	....	....	....	....
A	2834	hCoV-19/USA/WI-UW-19/2020|EPI_ISL_417510|2020-03-1	....	....	....	....	....	....
A	2835	hCoV-19/USA/WI-UW-24/2020|EPI_ISL_417512|2020-03-1	....	....	....	....	....	....
A	2836	hCoV-19/USA/WI-UW-14/2020|EPI_ISL_417513|2020-03-1	....	....	....	....	....	....
A	2837	hCoV-19/USA/WI-UW-22/2020|EPI_ISL_417514|2020-03-1	....	....	....	....	....	....
A	2838	hCoV-19/USA/WI-UW-18/2020|EPI_ISL_417515|2020-03-1	....	....	....	....	....	....
A	2839	hCoV-19/USA/WI-UW-13/2020|EPI_ISL_417516|2020-03-1	....	....	....	....	....	....
A	2840	hCoV-19/USA/WI-UW-17/2020|EPI_ISL_417517|2020-03-1	....	....	....	....	....	....
A	2841	hCoV-19/Taiwan/CGMH-CGU-06/2020|EPI_ISL_417519|202	....	....	....	....	....	....
A	2842	hCoV-19/Taiwan/CGMH-CGU-12/2020|EPI_ISL_417525|202	....	....	....	....	....	....
A	2843	hCoV-19/Luxembourg/LNS9371718/2020|EPI_ISL_417526|	....	....	....	....	....	....
A	2845	hCoV-19/Iceland/334/2020|EPI_ISL_417663|2020-03-14	....	....	....	....	....	....
A	2846	hCoV-19/Canada/BC_6404889/2020|EPI_ISL_418838|2020	....	....	....	....	....	....
A	2847	hCoV-19/Canada/BC_6489864/2020|EPI_ISL_418839|2020	....	....	....	....	....	....
A	2848	hCoV-19/Canada/BC_6502001/2020|EPI_ISL_418840|2020	....	....	....	....	....	....
A	2849	hCoV-19/Canada/BC_6981299/2020|EPI_ISL_418841|2020	....	....	....	....	....	....
A	2850	hCoV-19/Canada/BC_6997898/2020|EPI_ISL_418842|2020	....	....	....	....	....	....
A	2851	hCoV-19/Canada/BC_7277381/2020|EPI_ISL_418843|2020	....	....	....	....	....	....
A	2852	hCoV-19/Canada/BC_7493713/2020|EPI_ISL_418844|2020	....	....	....	....	....	....
A	2853	hCoV-19/Canada/BC_7515663/2020|EPI_ISL_418845|2020	....	....	....	....	....	....
A	2854	hCoV-19/Canada/BC_7553799/2020|EPI_ISL_418846|2020	....	....	....	....	....	....
A	2855	hCoV-19/Canada/BC_8150537/2020|EPI_ISL_418847|2020	....	....	....	....	....	....
A	2856	hCoV-19/Canada/BC_8159203/2020|EPI_ISL_418848|2020	....	....	....	....	....	....
A	2857	hCoV-19/Canada/BC_8486790/2020|EPI_ISL_418849|2020	....	....	....	....	....	....
A	2858	hCoV-19/Canada/BC_9345042/2020|EPI_ISL_418856|2020	....	....	....	....	....	....
A	2859	hCoV-19/Canada/BC_9345715/2020|EPI_ISL_418857|2020	....	....	....	....	....	....

A 2860 hCoV-19/Hangzhou/ZJU-010/2020|EPI_ISL_418990|2020- ···················· ···················· ···················· ···················· ···················· ····················
A 2861 hCoV-19/Singapore/20/2020|EPI_ISL_418993|2020-01-2 ···················· ···················· ···················· ···················· ···················· ····················
A 2862 hCoV-19/Singapore/3Clin/2020|EPI_ISL_418994|2020-0 ···················· ···················· ···················· ···················· ···················· ····················
A 2864 hCoV-19/England/20148004904/2020|EPI_ISL_422001|20 ···················· ···················· ···················· ···················· ···················· ····················
A 2865 hCoV-19/England/20148006104/2020|EPI_ISL_422002|20 ···················· ···················· ···················· ···················· ···················· ····················
A 2866 hCoV-19/England/20148006304/2020|EPI_ISL_422003|20 ···················· ···················· ···················· ···················· ···················· ····················
A 2867 hCoV-19/England/20148007004/2020|EPI_ISL_422007|20 ···················· ···················· ···················· ···················· ···················· ····················
A 2869 hCoV-19/England/20148039404/2020|EPI_ISL_422009|20 ···················· ···················· ···················· ···················· ···················· ····················
A 2871 hCoV-19/England/20148046704/2020|EPI_ISL_422011|20 ···················· ···················· ···················· ···················· ···················· ····················
A 2872 hCoV-19/Wales/PHWC-26796/2020|EPI_ISL_422016|2020- ···················· ···················· ···················· ···················· ···················· ····················
A 2873 hCoV-19/Wales/PHWC-2711F/2020|EPI_ISL_422017|2020- ···················· ···················· ···················· ···················· ···················· ····················
A 2874 hCoV-19/Wales/PHWC-2673C/2020|EPI_ISL_422018|2020- ···················· ···················· ···················· ···················· ···················· ····················
A 2875 hCoV-19/Wales/PHWC-265E7/2020|EPI_ISL_422019|2020- ···················· ···················· ···················· ···················· ···················· ····················
A 2876 hCoV-19/Wales/PHWC-2682A/2020|EPI_ISL_422020|2020- ···················· ···················· ···················· ···················· ···················· ····················
A 2877 hCoV-19/Wales/PHWC-2665D/2020|EPI_ISL_422021|2020- ···················· ····A··············· ···················· ···················· ···················· ····················
A 2878 hCoV-19/Wales/PHWC-26D49/2020|EPI_ISL_422022|2020- ···················· ···················· ···················· ···················· ···················· ····················
A 2879 hCoV-19/Wales/PHWC-26981/2020|EPI_ISL_422023|2020- ···················· ···················· ···················· ···················· ···················· ····················
A 2880 hCoV-19/Wales/PHWC-26B12/2020|EPI_ISL_422024|2020- ···················· ···················· ···················· ···················· ···················· ····················
A 2881 hCoV-19/Wales/PHWC-263DE/2020|EPI_ISL_422025|2020- ···················· ···················· ···················· ···················· ···················· ····················
A 2882 hCoV-19/Wales/PHWC-263FC/2020|EPI_ISL_422026|2020- ···················· ····A··············· ···················· ···················· ···················· ····················
A 2883 hCoV-19/Wales/PHWC-2672D/2020|EPI_ISL_422027|2020- ···················· ···················· ···················· ···················· ···················· ····················
A 2884 hCoV-19/Wales/PHWC-2631A/2020|EPI_ISL_422028|2020- ···················· ····A··············· ···················· ···················· ···················· ····················
A 2885 hCoV-19/Wales/PHWC-25F44/2020|EPI_ISL_422029|2020- ···················· ···················· ···················· ···················· ···················· ····················
A 2886 hCoV-19/Wales/PHWC-270B8/2020|EPI_ISL_422030|2020- ···················· ···················· ···················· ···················· ···················· ····················
A 2887 hCoV-19/Wales/PHWC-26DD0/2020|EPI_ISL_422031|2020- ···················· ···················· ···················· ···················· ···················· ····················
A 2888 hCoV-19/Wales/PHWC-2649F/2020|EPI_ISL_422032|2020- ···················· ···················· ···················· ···················· ···················· ····················
A 2889 hCoV-19/Wales/PHWC-2681B/2020|EPI_ISL_422033|2020- ···················· ···················· ···················· ···················· ···················· ····················
A 2890 hCoV-19/Wales/PHWC-27012/2020|EPI_ISL_422034|2020- ···················· ···················· ···················· ···················· ···················· ····················
A 2891 hCoV-19/Wales/PHWC-26D58/2020|EPI_ISL_422035|2020- ···················· ···················· ···················· ···················· ···················· ····················
A 2892 hCoV-19/Wales/PHWC-26A15/2020|EPI_ISL_422036|2020- ···················· ···················· ···················· ···················· ···················· ····················
A 2893 hCoV-19/Wales/PHWC-2663F/2020|EPI_ISL_422037|2020- ···················· ···················· ···················· ···················· ···················· ····················
A 2894 hCoV-19/Wales/PHWC-2621D/2020|EPI_ISL_422038|2020- ···················· ···················· ···················· ···················· ···················· ····················
A 2896 hCoV-19/England/20138078204/2020|EPI_ISL_423324|20 ···················· ···················· ···················· ···················· ···················· ····················
A 2897 hCoV-19/England/20138084904/2020|EPI_ISL_423328|20 ···················· ···················· ···················· ···················· ···················· ····················
A 2898 hCoV-19/England/20139002204/2020|EPI_ISL_423337|20 ···················· ···················· ···················· ···················· ···················· ····················
A 2901 hCoV-19/England/20139016704/2020|EPI_ISL_423346|20 ···················· ···················· ···················· ···················· ···················· ····················
A 2904 hCoV-19/England/20139064904/2020|EPI_ISL_423352|20 ···················· ···················· ···················· ···················· ···················· ····················
A 2905 hCoV-19/England/20139065204/2020|EPI_ISL_423353|20 ···················· ···················· ···················· ···················· ···················· ····················
A 2906 hCoV-19/England/20139065404/2020|EPI_ISL_423354|20 ···················· ···················· ···················· ···················· ···················· ····················
A 2907 hCoV-19/England/20139065604/2020|EPI_ISL_423355|20 ···················· ···················· ···················· ···················· ···················· ····················
A 2908 hCoV-19/England/20139067504/2020|EPI_ISL_423356|20 ···················· ···················· ···················· ···················· ···················· ····················
A 2909 hCoV-19/England/20139068504/2020|EPI_ISL_423357|20 ···················· ···················· ···················· ···················· ···················· ····················
A 2910 hCoV-19/England/20139068804/2020|EPI_ISL_423358|20 ···················· ···················· ···················· ···················· ···················· ····················
A 2911 hCoV-19/England/20139069104/2020|EPI_ISL_423359|20 ···················· ···················· ···················· ···················· ···················· ··N···N·············
A 2912 hCoV-19/England/20139069404/2020|EPI_ISL_423360|20 ···················· ···················· ···················· ···················· ···················· ····················
A 2917 hCoV-19/England/20139072204/2020|EPI_ISL_423365|20 ···················· ···················· ···················· ···················· ···················· ····················
A 2918 hCoV-19/England/20140000304/2020|EPI_ISL_423366|20 ···················· ···················· ···················· ···················· ···················· ····················
A 2920 hCoV-19/England/20140000604/2020|EPI_ISL_423368|20 ···················· ···················· ···················· ···················· ···················· ····················
A 2922 hCoV-19/England/20144008304/2020|EPI_ISL_423500|20 ···················· ···················· ···················· ···················· ···················· ····················
A 2923 hCoV-19/England/20144008904/2020|EPI_ISL_423502|20 ···················· ···················· ···················· ···················· ···················· ····················
A 2924 hCoV-19/England/20144009004/2020|EPI_ISL_423503|20 ···················· ···················· ···················· ···················· ·············Y······ ····················
A 2925 hCoV-19/England/20144009104/2020|EPI_ISL_423504|20 ···················· ···················· ···················· ···················· ···················· ····················
A 2926 hCoV-19/England/20144011504/2020|EPI_ISL_423506|20 ···················· ···················· ···················· ···················· ···················· ····················
A 2928 hCoV-19/England/20144013704/2020|EPI_ISL_423508|20 ···················· ···················· ···················· ···················· ···················· ····················
A 2929 hCoV-19/England/20144014504/2020|EPI_ISL_423509|20 ···················· ···················· ···················· ···················· ···················· ····················
A 2932 hCoV-19/Iceland/593/2020|EPI_ISL_424613|2020-03-27 ···················· ···················· ···················· ···················· ···················· ····················
A 2933 hCoV-19/Iceland/594/2020|EPI_ISL_424614|2020-03-27 ···················· ···················· ···················· ···················· ···················· ····················
A 2934 hCoV-19/Iceland/595/2020|EPI_ISL_424615|2020-03-27 ···················· ···················· ···················· ···················· ···················· ····················
A 2935 hCoV-19/Iceland/596/2020|EPI_ISL_424616|2020-03-28 ···················· ···················· ···················· ···················· ···················· ····················
A 2936 hCoV-19/Iceland/597/2020|EPI_ISL_424617|2020-03-28 ···················· ···················· ···················· ···················· ···················· ····················
A 2937 hCoV-19/Iceland/599/2020|EPI_ISL_424619|2020-03-28 ···················· ···················· ···················· ···················· ···················· ····················
A 2938 hCoV-19/Iceland/601/2020|EPI_ISL_424621|2020-03-28 ···················· ···················· ···················· ···················· ···················· ····················
A 2939 hCoV-19/Iceland/602/2020|EPI_ISL_424622|2020-03-28 ···················· ···················· ···················· ···················· ···················· ····················
A 2940 hCoV-19/Iceland/604/2020|EPI_ISL_424624|2020-03-28 ···················· ···················· ···················· ···················· ···················· ····················
A 2941 hCoV-19/Mexico/CMX-INER-04/2020|EPI_ISL_424626|202 ···················· ···················· ···················· ···················· ···················· ····················
A 2942 hCoV-19/Mexico/CMX-INER-05/2020|EPI_ISL_424627|202 ···················· ···················· ···················· ···················· ···················· ····················
A 2943 hCoV-19/Belgium/ULG-10066/2020|EPI_ISL_424629|2020 ···················· ···················· ···················· ···················· ···················· ····················
A 2944 hCoV-19/Belgium/ULG-10067/2020|EPI_ISL_424630|2020 ···················· ···················· ···················· ···················· ···················· ····················
A 2945 hCoV-19/Belgium/ULG-10072/2020|EPI_ISL_424631|2020 ···················· ···················· ···················· ···················· ···················· ····················
A 2946 hCoV-19/Belgium/ULG-10069/2020|EPI_ISL_424633|2020 ···················· ···················· ···················· ···················· ···················· ····················
A 2947 hCoV-19/Belgium/ULG-10068/2020|EPI_ISL_424636|2020 ···················· ···················· ···················· ···················· ···················· ····················
A 2948 hCoV-19/Belgium/ULG-10078/2020|EPI_ISL_424637|2020 ···················· ···················· ···················· ···················· ···················· ····················
A 2949 hCoV-19/Belgium/ULG-10081/2020|EPI_ISL_424640|2020 ···················· ···················· ···················· ···················· ···················· ····················
A 2950 hCoV-19/Belgium/ULG-10080/2020|EPI_ISL_424641|2020 ···················· ···················· ···················· ···················· ···················· ····················
A 2951 hCoV-19/Belgium/ULG-10077/2020|EPI_ISL_424642|2020 ···················· ···················· ···················· ···················· ···················· ····················
A 2952 hCoV-19/Belgium/ULG-10089/2020|EPI_ISL_424643|2020 ···················· ···················· ···················· ···················· ···················· ····················
A 2953 hCoV-19/Belgium/ULG-10086/2020|EPI_ISL_424644|2020 ···················· ···················· ···················· ···················· ···················· ····················
A 2954 hCoV-19/Belgium/ULG-10085/2020|EPI_ISL_424645|2020 ···················· ···················· ···················· ···················· ···················· ····················

A	2955 hCoV-19/Belgium/ULG-10088/2020|EPI_ISL_424646|2020	....	....	....	....	....	....	
A	2956 hCoV-19/Belgium/ULG-10092/2020|EPI_ISL_424647|2020	....	....	....	....	....	....	
A	2957 hCoV-19/Belgium/ULG-10082/2020|EPI_ISL_424649|2020	....	....	....	....	....	....	
A	2958 hCoV-19/Belgium/ULG-10094/2020|EPI_ISL_424650|2020	....	....	....	....	....	....	
A	2959 hCoV-19/Belgium/ULG-10095/2020|EPI_ISL_424651|2020	....	....	....	....	....	....	
A	2960 hCoV-19/Belgium/ULG-10055/2020|EPI_ISL_424652|2020	....	....	....	....	....	....	
A	2961 hCoV-19/Belgium/ULG-10058/2020|EPI_ISL_424653|2020	....	....	....	....	....	....	
A	2962 hCoV-19/Belgium/ULG-10057/2020|EPI_ISL_424654|2020	....	....	....	....	....	....	
A	2963 hCoV-19/Belgium/ULG-10056/2020|EPI_ISL_424655|2020	....	....	....	....	....	....	
A	2964 hCoV-19/Belgium/ULG-10052/2020|EPI_ISL_424657|2020	....	....	....	....	....	....	
A	2965 hCoV-19/Belgium/ULG-10049/2020|EPI_ISL_424658|2020	....	....	....	....	....	....	
A	2966 hCoV-19/Belgium/ULG-10051/2020|EPI_ISL_424660|2020	....	....	....	....	....	....	
A	2967 hCoV-19/Belgium/ULG-10053/2020|EPI_ISL_424661|2020	....	....	....	....	....	....	
A	2968 hCoV-19/Belgium/ULG-10061/2020|EPI_ISL_424662|2020	....	..........A.........	....	....	....	....	
A	2969 hCoV-19/Belgium/ULG-10062/2020|EPI_ISL_424664|2020	....	....	....	....	....	....	
A	2970 hCoV-19/Mexico/CMX-InDRE_03/2020|EPI_ISL_424667|20	....	....	....	....	....	....	
A	2971 hCoV-19/USA/AZ-ASU2922/2020|EPI_ISL_424668|2020-03	....	....	....	....	....	....	
A	2972 hCoV-19/USA/AZ-ASU2923/2020|EPI_ISL_424669|2020-03	....	....	....	....	....	....	
A	2973 hCoV-19/Mexico/QUE-InDRE-04/2020|EPI_ISL_424670|20	....	....	....	....	....	....	
A	2974 hCoV-19/USA/AZ-ASU2936/2020|EPI_ISL_424671|2020-03	....	....	....	....	....	....	
A	2975 hCoV-19/Mexico/PUE-InDRE-05/2020|EPI_ISL_424672|20	....	....	....	....	....	....	
A	2976 hCoV-19/Mexico/CMX-InDRE-06/2020|EPI_ISL_424673|20	....	....	....	....	....	....	
A	2977 hCoV-19/USA/SC_3569/2020|EPI_ISL_424841|2020-03-06	....	....	....	....	....	....	
A	2978 hCoV-19/USA/SC_3575/2020|EPI_ISL_424842|2020-03-07	....	....	....	....	....	....	
A	2979 hCoV-19/USA/MA_0020/2020|EPI_ISL_424843|2020-03-05	....	....	....	....	....	....	
A	2980 hCoV-19/USA/MA_3672/2020|EPI_ISL_424844|2020-03-06	....	....	....	....	....	....	
A	2981 hCoV-19/USA/MA_3699/2020|EPI_ISL_424845|2020-03-06	....	....	....	....	....	....	
A	2982 hCoV-19/USA/MA_3878/2020|EPI_ISL_424846|2020-03-06	....	....	....	....	....	....	
A	2983 hCoV-19/USA/MA_4811/2020|EPI_ISL_424847|2020-03-07	....	....	....	....	....	....	
A	2984 hCoV-19/USA/AZ_8132/2020|EPI_ISL_424848|2020-03-09	....	....	....	....	....	....	
A	2985 hCoV-19/USA/AZ_8135/2020|EPI_ISL_424849|2020-03-09	....	....	....	....	....	....	
A	2986 hCoV-19/USA/IL_0087/2020|EPI_ISL_424850|2020-03-07	....	....	....	....	....	....	
A	2987 hCoV-19/USA/IL_0089/2020|EPI_ISL_424851|2020-03-07	....	....	....	....	....	....	
A	2988 hCoV-19/USA/DC_0004/2020|EPI_ISL_424852|2020-03-06	....	....	....	....	....	....	
A	2989 hCoV-19/USA/FL_9590/2020|EPI_ISL_424853|2020-03-05	....	....	....	....	....	....	
A	2990 hCoV-19/USA/FL_9655/2020|EPI_ISL_424854|2020-03-05	....	....	....	....	....	....	
A	2991 hCoV-19/Scotland/EDB214/2020|EPI_ISL_425944|2020-0	....	....	....	....	....	....	
A	2992 hCoV-19/Scotland/EDB216/2020|EPI_ISL_425945|2020-0	....	....	....	....	....	....	
A	2993 hCoV-19/Scotland/EDB218/2020|EPI_ISL_425946|2020-0	....	....	....	....	....	....	
A	2994 hCoV-19/Scotland/EDB219/2020|EPI_ISL_425947|2020-0	....	....	....	....	....	....	
A	2995 hCoV-19/Scotland/EDB222/2020|EPI_ISL_425948|2020-0	....	....	....	....	....	....	
A	2996 hCoV-19/Scotland/EDB226/2020|EPI_ISL_425949|2020-0	....	....	....	....	....	....	
A	2997 hCoV-19/Scotland/EDB227/2020|EPI_ISL_425950|2020-0	....	....	....	....	....	....	
A	2998 hCoV-19/Scotland/EDB232/2020|EPI_ISL_425951|2020-0	....	....	....	....	....	....	
A	2999 hCoV-19/Scotland/EDB234/2020|EPI_ISL_425952|2020-0	....	....	....	....	....	....	
A	3000 hCoV-19/Scotland/EDB245/2020|EPI_ISL_425953|2020-0	....	....	....	....	....	....	
A	3001 hCoV-19/Scotland/EDB248/2020|EPI_ISL_425954|2020-0	....	....	....	....	....	....	
A	3002 hCoV-19/Scotland/EDB252/2020|EPI_ISL_425955|2020-0	....	....	....	....	....	....	
A	3003 hCoV-19/Scotland/EDB254/2020|EPI_ISL_425956|2020-0	....	....	....	....	....	....	
A	3004 hCoV-19/Scotland/EDB270/2020|EPI_ISL_425958|2020-0	....	....	....	....	....	....	
A	3005 hCoV-19/Scotland/EDB273/2020|EPI_ISL_425959|2020-0	....	....	....	....	....	....	
A	3006 hCoV-19/Scotland/EDB275/2020|EPI_ISL_425961|2020-0	....	....	....	....	....	....	
A	3007 hCoV-19/Scotland/EDB278/2020|EPI_ISL_425962|2020-0	....	....	....	....	....	....	
A	3008 hCoV-19/Scotland/EDB283/2020|EPI_ISL_425963|2020-0	....	....	....	....	....	....	
A	3009 hCoV-19/Scotland/EDB287/2020|EPI_ISL_425964|2020-0	....	....	....	....	....	....	
A	3010 hCoV-19/Scotland/EDB288/2020|EPI_ISL_425965|2020-0	....	....	....	....	....	....	
A	3011 hCoV-19/Scotland/EDB297/2020|EPI_ISL_425966|2020-0	....	....	....	....	....	....	
A	3012 hCoV-19/Scotland/EDB300/2020|EPI_ISL_425967|2020-0	....	....	....	....	....	....	
A	3013 hCoV-19/Scotland/EDB314/2020|EPI_ISL_425968|2020-0	....	....	....	....	....	....	
A	3014 hCoV-19/Scotland/EDB318/2020|EPI_ISL_425969|2020-0	....	....	....	....	....	....	
A	3015 hCoV-19/Scotland/EDB325/2020|EPI_ISL_425971|2020-0	....	....	....	....	....	....	
A	3016 hCoV-19/Scotland/EDB326/2020|EPI_ISL_425972|2020-0	....	....	....	....	....	....	
A	3019 hCoV-19/Scotland/EDB342/2020|EPI_ISL_425976|2020-0	....	....	....	....	....	....	
A	3020 hCoV-19/Scotland/EDB354/2020|EPI_ISL_425978|2020-0	....	....	....	....	....	....	
A	3021 hCoV-19/Scotland/EDB355/2020|EPI_ISL_425979|2020-0	....	....	....	....	....	....	
A	3022 hCoV-19/Scotland/EDB356/2020|EPI_ISL_425980|2020-0	....	....	....	....	....	....	
A	3023 hCoV-19/Scotland/EDB363/2020|EPI_ISL_425982|2020-0	....	...................A	....	....	....	....	1
A	3025 hCoV-19/Scotland/EDB373/2020|EPI_ISL_425985|2020-0	....	....	....	....	....	....	
A	3026 hCoV-19/Scotland/EDB374/2020|EPI_ISL_425986|2020-0	....	....	....	....	....	....	
A	3027 hCoV-19/Scotland/EDB378/2020|EPI_ISL_425987|2020-0	....	....	....	....	....	....	
A	3028 hCoV-19/Scotland/EDB379/2020|EPI_ISL_425988|2020-0	....	....	....	....	....	....	
A	3029 hCoV-19/Scotland/EDB383/2020|EPI_ISL_425990|2020-0	....	....	....	....	....	....	
A	3030 hCoV-19/Scotland/EDB386/2020|EPI_ISL_425991|2020-0	....	....	....	....	....	....	
A	3031 hCoV-19/Scotland/EDB389/2020|EPI_ISL_425992|2020-0	....	....	....	....	....	....	
A	3032 hCoV-19/Scotland/EDB390/2020|EPI_ISL_425993|2020-0	....	....	....	....	....	....	
A	3033 hCoV-19/Scotland/EDB391/2020|EPI_ISL_425994|2020-0	....	....	....	....	....	....	
A	3034 hCoV-19/Scotland/EDB392/2020|EPI_ISL_425995|2020-0	....	....	....	....	....	....	
A	3035 hCoV-19/Scotland/EDB396/2020|EPI_ISL_425996|2020-0	..................T.	....	....	....	....	....	1

A	3036	hCoV-19/Scotland/EDB397/2020|EPI_ISL_425997|2020-0	····················	····················	····················	····················	····················	····················
A	3037	hCoV-19/Scotland/EDB399/2020|EPI_ISL_425998|2020-0	····················	····················	····················	····················	····················	····················
A	3038	hCoV-19/Scotland/EDB401/2020|EPI_ISL_425999|2020-0	····················	····················	····················	····················	····················	····················
A	3039	hCoV-19/Australia/VIC779/2020|EPI_ISL_427054|2020-	····················	····················	····················	····················	····················	····················
A	3040	hCoV-19/Australia/VIC780/2020|EPI_ISL_427055|2020-	····················	····················	····················	····················	····················	····················
A	3044	hCoV-19/Australia/VIC785/2020|EPI_ISL_427060|2020-	····················	····················	····················	····················	····················	····················
A	3061	hCoV-19/Australia/VIC807/2020|EPI_ISL_427081|2020-	····················	····················	····················	····················	····················	····················
A	3062	hCoV-19/Australia/VIC808/2020|EPI_ISL_427082|2020-	····················	····················	····················	····················	····················	····················
A	3063	hCoV-19/Australia/VIC812/2020|EPI_ISL_427085|2020-	····················	····················	····················	····················	····················	····················
A	3064	hCoV-19/Australia/VIC820/2020|EPI_ISL_427092|2020-	····················	····················	····················	····················	····················	····················
A	3065	hCoV-19/Australia/VIC821/2020|EPI_ISL_427093|2020-	····················	····················	····················	····················	····················	····················
A	3066	hCoV-19/Australia/VIC823/2020|EPI_ISL_427094|2020-	····················	····················	····················	····················	····················	····················
A	3067	hCoV-19/Australia/VIC824/2020|EPI_ISL_427095|2020-	····················	····················	····················	····················	····················	····················
A	3068	hCoV-19/Australia/VIC828/2020|EPI_ISL_427096|2020-	····················	····················	····················	····················	····················	····················
A	3069	hCoV-19/USA/CT-Yale-076/2020|EPI_ISL_428384|2020-0	····················	····················	····················	····················	····················	····················
A	3070	hCoV-19/USA/CT-Yale-066/2020|EPI_ISL_428385|2020-0	····················	····················	····················	····················	····················	····················
A	3071	hCoV-19/USA/CT-Yale-062/2020|EPI_ISL_428387|2020-0	····················	····················	····················	····················	····················	····················
A	3072	hCoV-19/USA/CT-Yale-060/2020|EPI_ISL_428391|2020-0	····················	····················	····················	····················	··············N·····	····················
A	3073	hCoV-19/Wales/PHW04/2020|EPI_ISL_415041|2020-03-07	····················	····················	····················	····················	····················	····················
A	3074	hCoV-19/Shanghai/SH0005/2020|EPI_ISL_416319|2020-0	····················	····················	····················	····················	····················	····················
A	3075	hCoV-19/Shanghai/SH0007/2020|EPI_ISL_416320|2020-0	····················	····················	····················	····················	····················	····················
A	3076	hCoV-19/Shanghai/SH0008/2020|EPI_ISL_416321|2020-0	····················	····················	····················	····················	····················	····················
A	3077	hCoV-19/Shanghai/SH0009/2020|EPI_ISL_416322|2020-0	····················	····················	····················	····················	····················	····················
A	3078	hCoV-19/Shanghai/SH0010/2020|EPI_ISL_416323|2020-0	····················	····················	····················	····················	····················	····················
A	3079	hCoV-19/Shanghai/SH0011/2020|EPI_ISL_416324|2020-0	····················	····················	····················	····················	····················	····················
A	3080	hCoV-19/Shanghai/SH0012/2020|EPI_ISL_416325|2020-0	····················	····················	····················	····················	····················	····················
A	3081	hCoV-19/Shanghai/SH0013/2020|EPI_ISL_416326|2020-0	····················	····················	····················	····················	····················	····················
A	3082	hCoV-19/Shanghai/SH0020/2020|EPI_ISL_416329|2020-0	····················	····················	····················	····················	····················	····················
A	3083	hCoV-19/Shanghai/SH0021/2020|EPI_ISL_416330|2020-0	····················	····················	····················	····················	····················	····················
A	3084	hCoV-19/Shanghai/SH0023/2020|EPI_ISL_416332|2020-0	····················	····················	····················	····················	····················	····················
A	3085	hCoV-19/Shanghai/SH0024/2020|EPI_ISL_416333|2020-0	····················	····················	····················	····················	····················	····················
A	3086	hCoV-19/Shanghai/SH0025/2020|EPI_ISL_416334|2020-0	····················	····················	····················	····················	····················	····················
A	3087	hCoV-19/Shanghai/SH0026/2020|EPI_ISL_416335|2020-0	····················	····················	····················	····················	····················	····················
A	3088	hCoV-19/Shanghai/SH0027/2020|EPI_ISL_416336|2020-0	····················	····················	····················	····················	····················	····················
A	3089	hCoV-19/Shanghai/SH0028/2020|EPI_ISL_416337|2020-0	····················	····················	····················	····················	····················	····················
A	3090	hCoV-19/Shanghai/SH0029/2020|EPI_ISL_416338|2020-0	····················	····················	····················	····················	····················	····················
A	3091	hCoV-19/Shanghai/SH0030/2020|EPI_ISL_416339|2020-0	····················	····················	····················	····················	····················	····················
A	3092	hCoV-19/Shanghai/SH0031/2020|EPI_ISL_416340|2020-0	····················	····················	····················	····················	····················	····················
A	3093	hCoV-19/Shanghai/SH0032/2020|EPI_ISL_416341|2020-0	····················	····················	····················	····················	····················	····················
A	3094	hCoV-19/Shanghai/SH0033/2020|EPI_ISL_416342|2020-0	····················	····················	····················	····················	····················	····················
A	3095	hCoV-19/Shanghai/SH0039/2020|EPI_ISL_416348|2020-0	····················	····················	····················	····················	····················	····················
A	3096	hCoV-19/Shanghai/SH0040/2020|EPI_ISL_416349|2020-0	····················	····················	····················	····················	····················	····················
A	3098	hCoV-19/Shanghai/SH0043/2020|EPI_ISL_416352|2020-0	····················	····················	····················	····················	····················	····················
A	3099	hCoV-19/Shanghai/SH0044/2020|EPI_ISL_416353|2020-0	····················	····················	····················	····················	····················	····················
A	3100	hCoV-19/Shanghai/SH0045/2020|EPI_ISL_416354|2020-0	····················	····················	····················	····················	····················	····················
A	3101	hCoV-19/Shanghai/SH0047/2020|EPI_ISL_416355|2020-0	····················	····················	····················	····················	····················	····················
A	3102	hCoV-19/Shanghai/SH0050/2020|EPI_ISL_416358|2020-0	····················	·A··················	····················	····················	····················	····················
A	3103	hCoV-19/Shanghai/SH0051/2020|EPI_ISL_416359|2020-0	····················	····················	····················	····················	····················	····················
A	3104	hCoV-19/Shanghai/SH0053/2020|EPI_ISL_416361|2020-0	····················	····················	····················	····················	····················	····················
A	3105	hCoV-19/Shanghai/SH0054/2020|EPI_ISL_416362|2020-0	····················	··········A·········	····················	····················	····················	····················
A	3106	hCoV-19/Shanghai/SH0055/2020|EPI_ISL_416363|2020-0	····················	····················	····················	····················	····················	····················
A	3107	hCoV-19/Shanghai/SH0056/2020|EPI_ISL_416364|2020-0	····················	····················	····················	····················	····················	····················
A	3108	hCoV-19/Shanghai/SH0058/2020|EPI_ISL_416365|2020-0	····················	·A··················	····················	····················	····················	····················
A	3109	hCoV-19/Shanghai/SH0059/2020|EPI_ISL_416366|2020-0	····················	····················	····················	····················	····················	····················
A	3110	hCoV-19/Shanghai/SH0060/2020|EPI_ISL_416367|2020-0	····················	····················	····················	····················	····················	····················
A	3111	hCoV-19/Shanghai/SH0065/2020|EPI_ISL_416368|2020-0	····················	····················	····················	····················	····················	····NN··············
A	3112	hCoV-19/Shanghai/SH0066/2020|EPI_ISL_416369|2020-0	····················	····················	····················	····················	····················	····················
A	3113	hCoV-19/Shanghai/SH0067/2020|EPI_ISL_416370|2020-0	····················	····················	····················	····················	····················	····················
A	3114	hCoV-19/Shanghai/SH0069/2020|EPI_ISL_416372|2020-0	····················	····················	····················	····················	····················	····················
A	3115	hCoV-19/Shanghai/SH0070/2020|EPI_ISL_416373|2020-0	····················	····················	····················	····················	····················	····················
A	3116	hCoV-19/Shanghai/SH0073/2020|EPI_ISL_416376|2020-0	····················	····················	····················	····················	····················	····················
A	3117	hCoV-19/Shanghai/SH0074/2020|EPI_ISL_416377|2020-0	····················	····················	····················	····················	····················	····················
A	3118	hCoV-19/Shanghai/SH0075/2020|EPI_ISL_416378|2020-0	····················	····················	····················	····················	····················	N···················
A	3119	hCoV-19/Shanghai/SH0076/2020|EPI_ISL_416379|2020-0	····················	····················	····················	····················	····················	····················
A	3120	hCoV-19/Shanghai/SH0077/2020|EPI_ISL_416380|2020-0	····················	····················	····················	····················	····················	····················
A	3121	hCoV-19/Shanghai/SH0079/2020|EPI_ISL_416381|2020-0	····················	····················	····················	····················	····················	····················
A	3122	hCoV-19/Shanghai/SH0080/2020|EPI_ISL_416382|2020-0	····················	····················	····················	····················	····················	····················
A	3123	hCoV-19/Shanghai/SH0083/2020|EPI_ISL_416384|2020-0	····················	····················	····················	····················	····················	····················
A	3124	hCoV-19/Shanghai/SH0088/2020|EPI_ISL_416387|2020-0	····················	····················	····················	····················	····················	····················
A	3125	hCoV-19/Shanghai/SH0093/2020|EPI_ISL_416389|2020-0	····················	····A···············	····················	····················	····················	····················
A	3126	hCoV-19/Shanghai/SH0094/2020|EPI_ISL_416390|2020-0	····················	····················	····················	····················	····················	····················
A	3127	hCoV-19/Shanghai/SH0101/2020|EPI_ISL_416393|2020-0	····················	····················	····················	····················	····················	····················
A	3128	hCoV-19/Shanghai/SH0102/2020|EPI_ISL_416394|2020-0	····················	····················	····················	····················	····················	····················
A	3129	hCoV-19/Shanghai/SH0106/2020|EPI_ISL_416396|2020-0	····················	····················	····················	····················	····················	····················
A	3130	hCoV-19/Shanghai/SH0107/2020|EPI_ISL_416397|2020-0	····················	····················	····················	····················	····················	····················
A	3131	hCoV-19/Shanghai/SH0109/2020|EPI_ISL_416398|2020-0	····················	····················	····················	····················	····················	····················
A	3132	hCoV-19/Shanghai/SH0110/2020|EPI_ISL_416399|2020-0	····················	····················	····················	····················	····················	····················
A	3133	hCoV-19/Iceland/321/2020|EPI_ISL_417650|2020-03-18	····················	····················	····················	····················	····················	····················

A	3134	hCoV-19/Iceland/322/2020|EPI_ISL_417651|2020-03-18	····················	····················	····················	····················	····················	····················
A	3135	hCoV-19/Iceland/323/2020|EPI_ISL_417652|2020-03-18	····················	····················	····················	····················	····················	····················
A	3136	hCoV-19/Iceland/324/2020|EPI_ISL_417653|2020-03-18	····················	····················	····················	····················	····················	····················
A	3137	hCoV-19/Iceland/325/2020|EPI_ISL_417654|2020-03-18	····················	····················	····················	····················	····················	····················
A	3138	hCoV-19/Iceland/326/2020|EPI_ISL_417655|2020-03-14	····················	····················	····················	····················	····················	····················
A	3140	hCoV-19/Iceland/330/2020|EPI_ISL_417659|2020-03-14	····················	····················	····················	····················	····················	····················
A	3142	hCoV-19/Iceland/335/2020|EPI_ISL_417664|2020-03-13	····················	····················	····················	····················	····················	····················
A	3143	hCoV-19/Iceland/336/2020|EPI_ISL_417665|2020-03-14	····················	····················	····················	····················	····················	····················
A	3144	hCoV-19/Iceland/337/2020|EPI_ISL_417666|2020-03-15	····················	····················	····················	····················	····················	····················
A	3145	hCoV-19/Iceland/338/2020|EPI_ISL_417667|2020-03-15	····················	····················	····················	····················	····················	····················
A	3146	hCoV-19/Iceland/339/2020|EPI_ISL_417668|2020-03-15	····················	····················	····················	····················	····················	····················
A	3147	hCoV-19/Iceland/341/2020|EPI_ISL_417670|2020-03-15	····················	····················	····················	····················	·········N··········	····················
A	3148	hCoV-19/Iceland/38/2020|EPI_ISL_417672|2020-03-16	····················	····················	····················	····················	····················	····················
A	3150	hCoV-19/Iceland/98/2020|EPI_ISL_417676|2020-03-16	····················	····················	····················	····················	····················	····················
A	3151	hCoV-19/Iceland/39/2020|EPI_ISL_417677|2020-03-04	····················	····················	····················	····················	····················	····················
A	3152	hCoV-19/Iceland/185/2020|EPI_ISL_417678|2020-03-16	····················	····················	····················	····················	····················	····················
A	3154	hCoV-19/Iceland/177/2020|EPI_ISL_417680|2020-03-15	····················	····················	····················	····················	····················	····················
A	3155	hCoV-19/Iceland/37/2020|EPI_ISL_417681|2020-03-04	····················	····················	····················	····················	····················	····················
A	3156	hCoV-19/Iceland/101/2020|EPI_ISL_417682|2020-03-10	····················	····················	····················	····················	····················	····················
A	3157	hCoV-19/Iceland/47/2020|EPI_ISL_417683|2020-03-05	····················	····················	····················	····················	····················	····················
A	3158	hCoV-19/Iceland/54/2020|EPI_ISL_417684|2020-03-06	····················	····················	····················	····················	····················	····················
A	3159	hCoV-19/Iceland/215/2020|EPI_ISL_417685|2020-03-16	····················	····················	····················	····················	····················	····················
A	3160	hCoV-19/Iceland/55/2020|EPI_ISL_417687|2020-03-06	····················	····················	····················	····················	····················	····················
A	3161	hCoV-19/Iceland/12/2020|EPI_ISL_417688|2020-03-01	····················	····················	····················	····················	····················	····················
A	3162	hCoV-19/Iceland/160/2020|EPI_ISL_417689|2020-03-13	····················	····················	····················	····················	····················	····················
A	3163	hCoV-19/Iceland/125/2020|EPI_ISL_417690|2020-03-11	····················	····················	····················	····················	····················	····················
A	3164	hCoV-19/Iceland/111/2020|EPI_ISL_417691|2020-03-11	····················	····················	····················	····················	····················	····················
A	3165	hCoV-19/Iceland/57/2020|EPI_ISL_417692|2020-03-06	····················	····················	····················	····················	····················	····················
A	3166	hCoV-19/Iceland/25/2020|EPI_ISL_417693|2020-03-02	····················	····················	····················	····················	····················	····················
A	3167	hCoV-19/Iceland/3/2020|EPI_ISL_417694|2020-03-13	····················	····················	····················	····················	····················	····················
A	3168	hCoV-19/Iceland/20/2020|EPI_ISL_417695|2020-03-02	····················	····················	····················	····················	····················	····················
A	3169	hCoV-19/Iceland/135/2020|EPI_ISL_417696|2020-03-12	····················	····················	····················	····················	····················	····················
A	3171	hCoV-19/Iceland/103/2020|EPI_ISL_417698|2020-03-10	····················	····················	····················	····················	····················	····················
A	3172	hCoV-19/Iceland/206/2020|EPI_ISL_417699|2020-03-16	····················	····················	····················	····················	····················	····················
A	3173	hCoV-19/USA/NY-NYUMC34/2020|EPI_ISL_418980|2020-03	····················	····················	····················	····················	····················	····················
A	3174	hCoV-19/Belgium/VRAR-030643/2020|EPI_ISL_418981|20	····················	····················	····················	····················	····················	····················
A	3175	hCoV-19/Belgium/HAA-030953/2020|EPI_ISL_418982|202	····················	····················	····················	····················	····················	····················
A	3176	hCoV-19/Belgium/DWAEE-030954/2020|EPI_ISL_418983|2	····················	····················	····················	····················	····················	····················
A	3177	hCoV-19/Belgium/BG-030955/2020|EPI_ISL_418984|2020	····················	····················	····················	····················	····················	····················
A	3178	hCoV-19/Belgium/LT-030956/2020|EPI_ISL_418985|2020	····················	····················	····················	····················	····················	····················
A	3179	hCoV-19/Belgium/RS-030257/2020|EPI_ISL_418986|2020	····················	····················	····················	····················	····················	····················
A	3180	hCoV-19/Belgium/CG-030158/2020|EPI_ISL_418987|2020	····················	····················	····················	····················	····················	····················
A	3181	hCoV-19/Belgium/JRH-030459/2020|EPI_ISL_418988|202	····················	····················	····················	····················	····················	····················
A	3182	hCoV-19/Belgium/RT-030460/2020|EPI_ISL_418989|2020	····················	····················	····················	····················	····················	····················
A	3183	hCoV-19/Hangzhou/ZJU-011/2020|EPI_ISL_418991|2020-	····················	····················	····················	····················	····················	····················
A	3184	hCoV-19/Singapore/17/2020|EPI_ISL_418992|2020-02-1	····················	····················	····················	····················	····················	····················
A	3185	hCoV-19/Singapore/12Clin/2020|EPI_ISL_418995|2020-	····················	····················	····················	····················	····················	····················
A	3186	hCoV-19/Singapore/15/2020|EPI_ISL_418996|2020-01-2	····················	····················	····················	····················	····················	····················
A	3187	hCoV-19/Singapore/16/2020|EPI_ISL_418997|2020-02-0	····················	····················	····················	····················	····················	····················
A	3188	hCoV-19/Singapore/14Clin/2020|EPI_ISL_418998|2020-	····················	····················	····················	····················	····················	····················
A	3189	hCoV-19/Singapore/18/2020|EPI_ISL_418999|2020-03-0	····················	····················	····················	····················	····················	····················
A	3190	hCoV-19/Wales/PHWC-269CD/2020|EPI_ISL_422176|2020-	····················	····················	····················	····················	····················	····················
A	3191	hCoV-19/Wales/PHWC-2668A/2020|EPI_ISL_422177|2020-	····················	····A···············	····················	····················	····················	····················
A	3192	hCoV-19/Wales/PHWC-26D67/2020|EPI_ISL_422178|2020-	····················	····················	····················	····················	····················	····················
A	3194	hCoV-19/Australia/VIC718/2020|EPI_ISL_427002|2020-	····················	····················	····················	····················	····················	····················
A	3198	hCoV-19/Australia/VIC731/2020|EPI_ISL_427010|2020-	····················	····················	····················	····················	····················	····················
A	3201	hCoV-19/Australia/VIC734/2020|EPI_ISL_427013|2020-	····················	····················	····················	····················	····················	····················
A	3207	hCoV-19/Australia/VIC741/2020|EPI_ISL_427019|2020-	····················	····················	····················	····················	····················	····················
A	3215	hCoV-19/Australia/VIC752/2020|EPI_ISL_427029|2020-	····················	····················	····················	····················	····················	····················
A	3221	hCoV-19/Greece/13/2020|EPI_ISL_427043|2020-03-18	····················	····················	····················	····················	····················	····················
A	3226	hCoV-19/USA/WI-UW-204/2020|EPI_ISL_428300|2020-03-	····················	····················	····················	····················	····················	····················
A	3227	hCoV-19/USA/WI-UW-205/2020|EPI_ISL_428301|2020-03-	····················	····················	····················	····················	····················	····················
A	3228	hCoV-19/USA/WI-UW-206/2020|EPI_ISL_428302|2020-03-	····················	····················	····················	····················	····················	····················
A	3229	hCoV-19/USA/WI-UW-207/2020|EPI_ISL_428303|2020-03-	····················	····················	····················	····················	····················	····················
A	3230	hCoV-19/USA/WI-UW-208/2020|EPI_ISL_428304|2020-03-	····················	····················	····················	····················	····················	····················
A	3231	hCoV-19/USA/WI-UW-209/2020|EPI_ISL_428305|2020-03-	····················	····················	····················	····················	····················	····················
A	3232	hCoV-19/USA/WI-UW-210/2020|EPI_ISL_428306|2020-03-	····················	····················	····················	····················	····················	····················
A	3233	hCoV-19/USA/WI-UW-211/2020|EPI_ISL_428307|2020-03-	····················	····················	····················	····················	····················	····················
A	3234	hCoV-19/USA/WI-UW-212/2020|EPI_ISL_428308|2020-03-	····················	····················	····················	····················	····················	····················
A	3235	hCoV-19/USA/WI-UW-213/2020|EPI_ISL_428309|2020-03-	····················	····················	····················	····················	····················	····················
A	3236	hCoV-19/USA/WI-UW-214/2020|EPI_ISL_428310|2020-03-	····················	····················	····················	····················	····················	····················
A	3237	hCoV-19/USA/WI-UW-215/2020|EPI_ISL_428311|2020-03-	····················	····················	····················	····················	····················	····················
A	3238	hCoV-19/USA/WI-UW-216/2020|EPI_ISL_428312|2020-03-	····················	····················	····················	····················	····················	····················
A	3239	hCoV-19/USA/WI-UW-217/2020|EPI_ISL_428313|2020-03-	····················	····················	····················	····················	····················	····················
A	3240	hCoV-19/USA/WI-UW-218/2020|EPI_ISL_428314|2020-03-	····················	····················	····················	····················	····················	····················
A	3241	hCoV-19/USA/WI-UW-219/2020|EPI_ISL_428315|2020-03-	····················	····················	····················	····················	····················	····················
A	3242	hCoV-19/USA/WI-UW-220/2020|EPI_ISL_428316|2020-03-	····················	····················	····················	····················	····················	····················
A	3243	hCoV-19/USA/WI-UW-221/2020|EPI_ISL_428317|2020-03-	····················	····················	····················	····················	····················	····················

A	3244	hCoV-19/USA/WI-UW-222/2020|EPI_ISL_428318|2020-03-	....	....	....	....	....	....	
A	3245	hCoV-19/USA/WI-UW-223/2020|EPI_ISL_428319|2020-03-	....	....	....	....	....	....	
A	3246	hCoV-19/USA/WI-UW-224/2020|EPI_ISL_428320|2020-03-	....	....	....	....	....	....	
A	3247	hCoV-19/USA/WI-UW-225/2020|EPI_ISL_428321|2020-03-	....	....	....	....	....	....	
A	3248	hCoV-19/USA/WI-UW-226/2020|EPI_ISL_428322|2020-03-	....	....	....	....	....	....	
A	3249	hCoV-19/USA/WI-UW-227/2020|EPI_ISL_428323|2020-03-	....	....	....	....	....	....	
A	3250	hCoV-19/USA/WI-UW-228/2020|EPI_ISL_428324|2020-03-	....	....	....	....	....	....	
A	3251	hCoV-19/USA/WI-UW-229/2020|EPI_ISL_428325|2020-03-	....	....	....	....	....	....	
A	3252	hCoV-19/USA/WI-UW-230/2020|EPI_ISL_428326|2020-03-	....	....	....	....	....	....	
A	3253	hCoV-19/USA/WI-UW-231/2020|EPI_ISL_428327|2020-03-	....	....	....	....	....	....	
A	3254	hCoV-19/USA/WI-UW-232/2020|EPI_ISL_428328|2020-03-	....	....	....	....	....	....	
A	3255	hCoV-19/USA/WI-UW-233/2020|EPI_ISL_428329|2020-03-	....	....	....	....	....	....	
A	3256	hCoV-19/USA/WI-UW-234/2020|EPI_ISL_428330|2020-03-	....	....	....	....	....	....	
A	3257	hCoV-19/USA/WI-UW-235/2020|EPI_ISL_428331|2020-03-	....	....	....	....	....	....	
A	3258	hCoV-19/USA/WI-UW-236/2020|EPI_ISL_428332|2020-03-	....	....	....	....	....	....	
A	3259	hCoV-19/USA/WI-UW-237/2020|EPI_ISL_428333|2020-03-	....	....	....	....	....	....	
A	3260	hCoV-19/USA/WI-UW-238/2020|EPI_ISL_428334|2020-03-	....	....	....	....	....	....	
A	3261	hCoV-19/USA/WI-UW-239/2020|EPI_ISL_428335|2020-03-	....	....	....	....	....	....	
A	3262	hCoV-19/USA/WI-UW-240/2020|EPI_ISL_428336|2020-03-	....	....	....	....	....	....	
A	3263	hCoV-19/USA/WI-UW-241/2020|EPI_ISL_428337|2020-03-	....	....	....	....	....	....	
A	3264	hCoV-19/USA/WI-UW-242/2020|EPI_ISL_428338|2020-03-	....	....	....	....	....	....	
A	3265	hCoV-19/USA/WI-UW-243/2020|EPI_ISL_428339|2020-03-	....	....	....	....	....	....	
A	3266	hCoV-19/USA/WI-UW-244/2020|EPI_ISL_428340|2020-03-	....	....	....	....	....	....	
A	3267	hCoV-19/USA/WI-UW-245/2020|EPI_ISL_428341|2020-03-	....	....	....	....	....	....	
A	3268	hCoV-19/USA/WI-UW-246/2020|EPI_ISL_428342|2020-03-	....	....	....	....	....	....	
A	3269	hCoV-19/USA/WI-UW-247/2020|EPI_ISL_428343|2020-03-	....	....	....	....	....	....	
A	3270	hCoV-19/USA/WI-UW-248/2020|EPI_ISL_428344|2020-03-	....	....	....	....	....	....	
A	3271	hCoV-19/USA/WI-UW-249/2020|EPI_ISL_428345|2020-03-	....	....	....	....	....	....	
A	3272	hCoV-19/Turkey/IST-GLAB-012/2020|EPI_ISL_428346|20	....	....	....	....	....	....	
A	3273	hCoV-19/France/IDF-3384/2020|EPI_ISL_428347|2020-0	....	....	....	....	....	....	
A	3274	hCoV-19/France/GES-3372/2020|EPI_ISL_428348|2020-0	....	....	....	....	....	....	
A	3275	hCoV-19/France/IDF-3386/2020|EPI_ISL_428349|2020-0	....	....	....	....	....	....	
A	3276	hCoV-19/France/HDF-3419/2020|EPI_ISL_428350|2020-0	....	....	....	....	....	....	
A	3277	hCoV-19/France/IDF-3509/2020|EPI_ISL_428351|2020-0	....	....	....	....	....	....	
A	3278	hCoV-19/France/IDF-3518/2020|EPI_ISL_428352|2020-0	....	....	....	....	....	....	
A	3279	hCoV-19/France/HDF-3534/2020|EPI_ISL_428353|2020-0	....	....	....	....	....	....	
A	3280	hCoV-19/France/IDF-3577/2020|EPI_ISL_428354|2020-0	....	....	....	....	....	....	
A	3281	hCoV-19/France/IDF-2251/2020|EPI_ISL_428355|2020-0	....	....	....	....	....	....	
A	3282	hCoV-19/France/IDF-2252/2020|EPI_ISL_428356|2020-0	....	....	....	....	....	....	
A	3283	hCoV-19/France/IDF-2253/2020|EPI_ISL_428357|2020-0	....	....	....	....	....	....	
A	3284	hCoV-19/France/HDF-3598/2020|EPI_ISL_428358|2020-0	....	....	....	....	....	....	
A	3285	hCoV-19/France/HDF-3677/2020|EPI_ISL_428359|2020-0	....	....	....	....	....	....	
A	3286	hCoV-19/France/HDF-3678/2020|EPI_ISL_428360|2020-0	....	....	....	....	....	....	
A	3287	hCoV-19/France/IDF-3703/2020|EPI_ISL_428361|2020-0	....	....	....	....	....	....	
A	3288	hCoV-19/France/IDF-3745/2020|EPI_ISL_428363|2020-0	....	....	....	....	....	....	
A	3289	hCoV-19/France/IDF-3930/2020|EPI_ISL_428365|2020-0	....	....	....	....	....	....	
A	3290	hCoV-19/France/HDF-4220/2020|EPI_ISL_428366|2020-0	....	....	....	....	....	....	
A	3291	hCoV-19/France/NOR-N4427/2020|EPI_ISL_428367|2020-	....	....	....	....	....	....	
A	3292	hCoV-19/Turkey/IST-GLAB-013/2020|EPI_ISL_428368|20	....	....	....	....	....	....	
A	3293	hCoV-19/USA/WA-UW-4649/2020|EPI_ISL_429629|2020-04	....	....	....	....	....	....	
A	3294	hCoV-19/USA/WA-UW-4677/2020|EPI_ISL_429630|2020-04	....	....	....	....	....	....	
A	3295	hCoV-19/USA/WA-UW-4695/2020|EPI_ISL_429631|2020-04	....	....	....	....	....	....	
A	3296	hCoV-19/USA/WA-UW-4705/2020|EPI_ISL_429632|2020-04	....	....	....	....	....	....	
A	3297	hCoV-19/USA/WA-UW-4892/2020|EPI_ISL_429634|2020-04	....	....	....	....	....	....	
A	3298	hCoV-19/USA/WA-UW-4897/2020|EPI_ISL_429635|2020-04	....	....	....	....	....	....	
A	3299	hCoV-19/USA/WA-UW-4921/2020|EPI_ISL_429636|2020-04	....	....	....	....	....	....	
A	3300	hCoV-19/USA/WA-UW-4925/2020|EPI_ISL_429637|2020-04	....	....	....	....	....	....	
A	3301	hCoV-19/USA/WA-UW-4999/2020|EPI_ISL_429638|2020-04	....	....	....	....	....	....	
A	3302	hCoV-19/USA/WA-UW-5025/2020|EPI_ISL_429639|2020-04	....	....	....	....	....	....	
A	3303	hCoV-19/USA/CT-UW-5038/2020|EPI_ISL_429641|2020-04	....	....	....	....	....	....	
A	3304	hCoV-19/USA/CT-UW-5043/2020|EPI_ISL_429642|2020-04	....	...................A	....	....	....	....	1
A	3305	hCoV-19/USA/UN-UW-5044/2020|EPI_ISL_429643|2020-04	....	....	....	....	....	....	
A	3306	hCoV-19/USA/WA-UW-5065/2020|EPI_ISL_429644|2020-04	....	....	....	....	....	....	
A	3307	hCoV-19/USA/CT-UW-5073/2020|EPI_ISL_429645|2020-04	....	....	....	....	....	....	
A	3308	hCoV-19/USA/WA-UW-5095/2020|EPI_ISL_429647|2020-04	....	....	....	....	....	....	
A	3309	hCoV-19/USA/WA-UW-5144/2020|EPI_ISL_429649|2020-04	....	....	....	....	....	....	
A	3310	hCoV-19/USA/WA-UW-5152/2020|EPI_ISL_429650|2020-04	....	....	....	....	....	....	
A	3311	hCoV-19/USA/WA-UW-5158/2020|EPI_ISL_429651|2020-04	....	....	....	....	....	....	
A	3312	hCoV-19/USA/WA-UW-5164/2020|EPI_ISL_429652|2020-04	....	....	....	....	....	....	
A	3313	hCoV-19/USA/WA-UW-5166/2020|EPI_ISL_429653|2020-04	....	....	....	....	....	....	
A	3314	hCoV-19/USA/WA-UW-5169/2020|EPI_ISL_429654|2020-04	....	....	....	....	....	....	
A	3315	hCoV-19/USA/WA-UW-5170/2020|EPI_ISL_429655|2020-04	....	....	....	....	....	....	
A	3316	hCoV-19/USA/UN-UW-5172/2020|EPI_ISL_429656|2020-04	....	....	....	....	....	....	
A	3317	hCoV-19/Croatia/OY-S1/2020|EPI_ISL_429659|2020-03	....	....	....	....	....	....	
A	3318	hCoV-19/Croatia/8U-S17/2020|EPI_ISL_429663|2020-03	....	....	....	....	....	....	
A	3319	hCoV-19/Brazil/MG-CV4/2020|EPI_ISL_429667|2020-03-	....	....	....	....	....	....	
A	3320	hCoV-19/Brazil/MG-CV6/2020|EPI_ISL_429669|2020-03-	....	....	....	....	....	....	
A	3321	hCoV-19/Brazil/MG-CV8/2020|EPI_ISL_429671|2020-03-	....	....	....	....	....	....	

A	3322	hCoV-19/Brazil/MG-CV12/2020|EPI_ISL_429674|2020-03	····················	····················	····················	····················	····················	····················
A	3323	hCoV-19/Brazil/MG-CV16/2020|EPI_ISL_429676|2020-03	····················	····················	····················	····················	····················	····················
A	3324	hCoV-19/Brazil/MG-CV21/2020|EPI_ISL_429681|2020-03	····················	····················	····················	····················	····················	····················
A	3325	hCoV-19/Brazil/MG-CV31/2020|EPI_ISL_429687|2020-03	····················	····················	····················	····················	····················	····················
A	3326	hCoV-19/Brazil/MG-CV32/2020|EPI_ISL_429688|2020-03	····················	····················	····················	····················	····················	····················
A	3327	hCoV-19/Brazil/MG-CV33/2020|EPI_ISL_429689|2020-03	····················	····················	····················	····················	····················	····················
A	3328	hCoV-19/Brazil/MG-CV42/2020|EPI_ISL_429695|2020-03	····················	····················	····················	····················	····················	····················
A	3329	hCoV-19/Hangzhou/HZ-1/2020|EPI_ISL_406970|2020-01-	····················	····················	····················	····················	····················	····················
A	3330	hCoV-19/Singapore/1/2020|EPI_ISL_406973|2020-01-23	····················	····················	····················	····················	····················	····················
A	3331	hCoV-19/Hong Kong/CUHK1/2020|EPI_ISL_416314|2020-0	····················	····················	····················	····················	····················	····················
A	3332	hCoV-19/Shanghai/SH0002/2020|EPI_ISL_416316|2020-0	····················	····················	····················	····················	····················	····················
A	3333	hCoV-19/Shanghai/SH0003/2020|EPI_ISL_416317|2020-0	····················	····················	····················	····················	····················	····················
A	3334	hCoV-19/Shanghai/SH0004/2020|EPI_ISL_416318|2020-0	····················	····················	····················	····················	····················	····················
A	3335	hCoV-19/Iceland/272/2020|EPI_ISL_417600|2020-03-17	····················	····················	····················	····················	····················	····················
A	3336	hCoV-19/Iceland/273/2020|EPI_ISL_417601|2020-03-17	····················	····················	····················	····················	····················	····················
A	3337	hCoV-19/Iceland/274/2020|EPI_ISL_417602|2020-03-17	····················	····················	····················	····················	····················	····················
A	3338	hCoV-19/Iceland/275/2020|EPI_ISL_417603|2020-03-17	····················	····················	····················	····················	····················	····················
A	3339	hCoV-19/Iceland/276/2020|EPI_ISL_417604|2020-03-17	····················	····················	····················	····················	····················	····················
A	3340	hCoV-19/Iceland/277/2020|EPI_ISL_417605|2020-03-17	····················	····················	····················	····················	····················	····················
A	3341	hCoV-19/Iceland/278/2020|EPI_ISL_417606|2020-03-17	····················	····················	····················	····················	····················	····················
A	3342	hCoV-19/Iceland/279/2020|EPI_ISL_417607|2020-03-17	····················	····················	····················	····················	····················	····················
A	3343	hCoV-19/Iceland/280/2020|EPI_ISL_417608|2020-03-17	····················	····················	····················	····················	····················	····················
A	3344	hCoV-19/Iceland/281/2020|EPI_ISL_417609|2020-03-17	····················	····················	····················	····················	····················	····················
A	3345	hCoV-19/Iceland/282/2020|EPI_ISL_417610|2020-03-17	····················	····················	····················	····················	····················	····················
A	3346	hCoV-19/Iceland/283/2020|EPI_ISL_417611|2020-03-17	····················	····················	····················	····················	····················	····················
A	3347	hCoV-19/Iceland/284/2020|EPI_ISL_417612|2020-03-17	····················	····················	····················	····················	····················	····················
A	3348	hCoV-19/Iceland/285/2020|EPI_ISL_417613|2020-03-17	····················	····················	····················	····················	····················	····················
A	3349	hCoV-19/Iceland/286/2020|EPI_ISL_417614|2020-03-18	····················	····················	····················	····················	····················	····················
A	3350	hCoV-19/Iceland/287/2020|EPI_ISL_417615|2020-03-18	····················	····················	····················	····················	····················	····················
A	3351	hCoV-19/Iceland/288/2020|EPI_ISL_417616|2020-03-18	····················	····················	····················	····················	····················	····················
A	3352	hCoV-19/Iceland/289/2020|EPI_ISL_417617|2020-03-18	····················	····················	····················	····················	····················	····················
A	3353	hCoV-19/Iceland/29/2020|EPI_ISL_417618|2020-03-16	····················	····················	····················	····················	····················	····················
A	3354	hCoV-19/Iceland/290/2020|EPI_ISL_417619|2020-03-18	····················	····················	····················	····················	····················	····················
A	3355	hCoV-19/Iceland/291/2020|EPI_ISL_417620|2020-03-18	····················	····················	····················	····················	····················	····················
A	3356	hCoV-19/Iceland/292/2020|EPI_ISL_417621|2020-03-18	····················	····················	····················	····················	····················	····················
A	3357	hCoV-19/Iceland/293/2020|EPI_ISL_417622|2020-03-18	····················	····················	····················	····················	····················	····················
A	3358	hCoV-19/Iceland/294/2020|EPI_ISL_417623|2020-03-18	····················	····················	····················	····················	····················	····················
A	3359	hCoV-19/Iceland/295/2020|EPI_ISL_417624|2020-03-18	····················	····················	····················	····················	····················	····················
A	3360	hCoV-19/Iceland/296/2020|EPI_ISL_417625|2020-03-18	····················	····················	····················	····················	····················	····················
A	3361	hCoV-19/Iceland/297/2020|EPI_ISL_417626|2020-03-18	····················	····················	····················	····················	····················	····················
A	3362	hCoV-19/Iceland/298/2020|EPI_ISL_417627|2020-03-18	····················	····················	····················	····················	····················	····················
A	3363	hCoV-19/Iceland/299/2020|EPI_ISL_417628|2020-03-18	····················	····················	····················	····················	····················	····················
A	3364	hCoV-19/Iceland/300/2020|EPI_ISL_417629|2020-03-18	····················	····················	····················	····················	····················	····················
A	3365	hCoV-19/Iceland/301/2020|EPI_ISL_417630|2020-03-18	····················	····················	····················	····················	····················	····················
A	3366	hCoV-19/Iceland/302/2020|EPI_ISL_417631|2020-03-18	····················	····················	····················	····················	····················	····················
A	3367	hCoV-19/Iceland/303/2020|EPI_ISL_417632|2020-03-18	····················	····················	····················	····················	····················	····················
A	3368	hCoV-19/Iceland/304/2020|EPI_ISL_417633|2020-03-17	····················	····················	····················	····················	····················	····················
A	3369	hCoV-19/Iceland/305/2020|EPI_ISL_417634|2020-03-18	····················	····················	····················	····················	····················	····················
A	3370	hCoV-19/Iceland/306/2020|EPI_ISL_417635|2020-03-18	····················	····················	····················	····················	····················	····················
A	3371	hCoV-19/Iceland/307/2020|EPI_ISL_417636|2020-03-18	····················	····················	····················	····················	····················	····················
A	3372	hCoV-19/Iceland/308/2020|EPI_ISL_417637|2020-03-18	····················	····················	····················	····················	····················	····················
A	3373	hCoV-19/Iceland/309/2020|EPI_ISL_417638|2020-03-18	····················	····················	····················	····················	····················	····················
A	3374	hCoV-19/Iceland/310/2020|EPI_ISL_417639|2020-03-18	····················	····················	····················	····················	····················	····················
A	3375	hCoV-19/Iceland/311/2020|EPI_ISL_417640|2020-03-18	····················	····················	····················	····················	····················	····················
A	3376	hCoV-19/Iceland/312/2020|EPI_ISL_417641|2020-03-18	····················	····················	····················	····················	····················	····················
A	3377	hCoV-19/Iceland/313/2020|EPI_ISL_417642|2020-03-18	····················	····················	····················	····················	····················	····················
A	3378	hCoV-19/Iceland/314/2020|EPI_ISL_417643|2020-03-18	····················	····················	····················	····················	····················	····················
A	3379	hCoV-19/Iceland/315/2020|EPI_ISL_417644|2020-03-18	····················	····················	····················	····················	····················	····················
A	3380	hCoV-19/Iceland/316/2020|EPI_ISL_417645|2020-03-18	····················	····················	····················	····················	····················	····················
A	3381	hCoV-19/Iceland/317/2020|EPI_ISL_417646|2020-03-18	····················	····················	····················	····················	····················	····················
A	3382	hCoV-19/Iceland/318/2020|EPI_ISL_417647|2020-03-18	····················	····················	····················	····················	····················	····················
A	3383	hCoV-19/Iceland/319/2020|EPI_ISL_417648|2020-03-18	····················	····················	····················	····················	····················	····················
A	3384	hCoV-19/Iceland/320/2020|EPI_ISL_417649|2020-03-18	····················	····················	····················	····················	····················	····················
A	3385	hCoV-19/USA/WA-UW333/2020|EPI_ISL_418900|2020-03-1	····················	····················	····················	····················	····················	····················
A	3386	hCoV-19/USA/MN-UW334/2020|EPI_ISL_418901|2020-03-1	····················	····················	····················	····················	····················	····················
A	3387	hCoV-19/USA/MN-UW335/2020|EPI_ISL_418902|2020-03-1	····················	····················	····················	····················	····················	····················
A	3388	hCoV-19/USA/WA-UW336/2020|EPI_ISL_418903|2020-03-1	····················	····················	····················	····················	····················	····················
A	3389	hCoV-19/USA/WA-UW337/2020|EPI_ISL_418904|2020-03-1	····················	····················	····················	····················	····················	····················
A	3390	hCoV-19/USA/WA-UW338/2020|EPI_ISL_418905|2020-03-1	····················	····················	····················	····················	····················	····················
A	3391	hCoV-19/USA/WA-UW339/2020|EPI_ISL_418906|2020-03-1	····················	····················	····················	····················	····················	····················
A	3392	hCoV-19/USA/WA-UW340/2020|EPI_ISL_418907|2020-03-1	····················	····················	····················	····················	····················	····················
A	3393	hCoV-19/USA/WA-UW341/2020|EPI_ISL_418908|2020-03-1	····················	····················	····················	····················	····················	····················
A	3394	hCoV-19/USA/WA-UW342/2020|EPI_ISL_418909|2020-03-1	····················	····················	····················	····················	····················	····················
A	3395	hCoV-19/USA/WA-UW343/2020|EPI_ISL_418910|2020-03-1	····················	····················	····················	····················	····················	····················
A	3396	hCoV-19/USA/WA-UW344/2020|EPI_ISL_418911|2020-03-1	····················	····················	····················	····················	····················	····················
A	3397	hCoV-19/USA/WA-UW345/2020|EPI_ISL_418912|2020-03-1	····················	····················	····················	····················	····················	····················
A	3398	hCoV-19/USA/WA-UW346/2020|EPI_ISL_418913|2020-03-1	····················	····················	····················	····················	····················	····················
A	3399	hCoV-19/USA/IL-UW348/2020|EPI_ISL_418915|2020-03-1	····················	····················	····················	····················	····················	····················

A	3400	hCoV-19/USA/IL-UW349/2020|EPI_ISL_418916|2020-03-1	····················	·A··················	····················	····················	····················	····················
A	3401	hCoV-19/USA/IL-UW350/2020|EPI_ISL_418917|2020-03-1	····················	····················	····················	····················	····················	····················
A	3402	hCoV-19/USA/IL-UW351/2020|EPI_ISL_418918|2020-03-1	····················	····················	····················	····················	····················	····················
A	3403	hCoV-19/USA/IL-UW352/2020|EPI_ISL_418919|2020-03-1	····················	····················	····················	····················	····················	····················
A	3404	hCoV-19/USA/IL-UW353/2020|EPI_ISL_418920|2020-03-1	····················	····················	····················	····················	····················	····················
A	3405	hCoV-19/USA/UN-UW354/2020|EPI_ISL_418921|2020-03-1	····················	····················	····················	····················	····················	····················
A	3406	hCoV-19/USA/WA-UW355/2020|EPI_ISL_418922|2020-03-1	····················	····················	····················	····················	····················	····················
A	3407	hCoV-19/USA/WA-UW356/2020|EPI_ISL_418923|2020-03-1	····················	····················	····················	····················	····················	····················
A	3408	hCoV-19/USA/WA-UW357/2020|EPI_ISL_418924|2020-03-1	····················	····················	····················	····················	····················	····················
A	3409	hCoV-19/USA/UN-UW358/2020|EPI_ISL_418925|2020-03	····················	····················	····················	····················	····················	····················
A	3410	hCoV-19/USA/WA-UW359/2020|EPI_ISL_418926|2020-03-1	····················	····················	····················	····················	····················	····················
A	3411	hCoV-19/USA/WA-UW360/2020|EPI_ISL_418927|2020-03-1	····················	····················	····················	····················	····················	····················
A	3412	hCoV-19/USA/WA-UW361/2020|EPI_ISL_418928|2020-03-1	····················	····················	····················	····················	····················	····················
A	3413	hCoV-19/USA/WA-UW362/2020|EPI_ISL_418929|2020-03-1	····················	····················	····················	····················	····················	····················
A	3414	hCoV-19/USA/WA-UW363/2020|EPI_ISL_418930|2020-03-1	····················	····················	····················	····················	····················	····················
A	3415	hCoV-19/USA/WA-UW364/2020|EPI_ISL_418931|2020-03-1	····················	····················	····················	····················	····················	····················
A	3416	hCoV-19/USA/WA-UW365/2020|EPI_ISL_418932|2020-03-1	····················	····················	····················	····················	····················	····················
A	3417	hCoV-19/USA/WA-UW366/2020|EPI_ISL_418933|2020-03-1	····················	····················	····················	····················	····················	····················
A	3418	hCoV-19/USA/WA-UW367/2020|EPI_ISL_418934|2020-03-1	····················	····················	····················	····················	····················	····················
A	3419	hCoV-19/USA/WA-UW368/2020|EPI_ISL_418935|2020-03-1	····················	····················	····················	····················	····················	····················
A	3420	hCoV-19/USA/WA-UW369/2020|EPI_ISL_418936|2020-03-1	····················	····················	····················	····················	····················	····················
A	3421	hCoV-19/USA/WA-UW370/2020|EPI_ISL_418937|2020-03-1	····················	····················	····················	····················	····················	····················
A	3422	hCoV-19/USA/WA-UW371/2020|EPI_ISL_418938|2020-03-1	····················	····················	····················	····················	····················	····················
A	3423	hCoV-19/USA/WA-UW372/2020|EPI_ISL_418939|2020-03-1	····················	····················	····················	····················	····················	····················
A	3424	hCoV-19/USA/WA-UW373/2020|EPI_ISL_418940|2020-03-1	····················	····················	····················	····················	····················	····················
A	3425	hCoV-19/USA/WA-UW374/2020|EPI_ISL_418941|2020-03	····················	····················	····················	····················	····················	····················
A	3426	hCoV-19/USA/WA-UW375/2020|EPI_ISL_418942|2020-03-1	····················	····················	····················	····················	····················	····················
A	3427	hCoV-19/USA/WA-UW376/2020|EPI_ISL_418943|2020-03-1	····················	····················	····················	····················	····················	····················
A	3428	hCoV-19/USA/WA-UW378/2020|EPI_ISL_418944|2020-03-1	····················	····················	····················	····················	····················	····················
A	3429	hCoV-19/USA/WA-UW-1238/2020|EPI_ISL_418945|2020-03	····················	····················	····················	····················	····················	····················
A	3430	hCoV-19/USA/WA-UW383/2020|EPI_ISL_418948|2020-03-1	····················	····················	····················	····················	····················	····················
A	3431	hCoV-19/USA/WA-UW384/2020|EPI_ISL_418949|2020-03-1	····················	····················	····················	····················	····················	····················
A	3432	hCoV-19/USA/WA-UW385/2020|EPI_ISL_418950|2020-03-1	····················	····················	····················	····················	····················	····················
A	3433	hCoV-19/USA/WA-UW386/2020|EPI_ISL_418951|2020-03-1	····················	····················	····················	····················	····················	····················
A	3434	hCoV-19/USA/WA-UW387/2020|EPI_ISL_418952|2020-03-1	····················	····················	····················	····················	····················	····················
A	3435	hCoV-19/USA/WA-UW388/2020|EPI_ISL_418953|2020-03-1	····················	····················	····················	····················	····················	····················
A	3436	hCoV-19/USA/WA-UW389/2020|EPI_ISL_418954|2020-03-1	····················	····················	····················	····················	····················	····················
A	3437	hCoV-19/USA/WA-UW390/2020|EPI_ISL_418955|2020-03-1	····················	····················	····················	····················	····················	····················
A	3438	hCoV-19/USA/VA-DCLS-0002/2020|EPI_ISL_418956|2020-	····················	····················	····················	····················	····················	····················
A	3439	hCoV-19/USA/VA-DCLS-0003/2020|EPI_ISL_418957|2020-	····················	····················	····················	····················	····················	····················
A	3440	hCoV-19/USA/VA-DCLS-0004/2020|EPI_ISL_418958|2020-	····················	····················	····················	····················	····················	····················
A	3441	hCoV-19/USA/UT-019/2020|EPI_ISL_418962|2020-03-13	····················	····················	····················	····················	····················	····················
A	3442	hCoV-19/USA/UT-021/2020|EPI_ISL_418963|2020-03-14	····················	····················	····················	····················	····················	····················
A	3443	hCoV-19/USA/UT-022/2020|EPI_ISL_418964|2020-03-14	····················	····················	····················	····················	····················	····················
A	3444	hCoV-19/USA/UT-023/2020|EPI_ISL_418965|2020-03-11	····················	····················	····················	····················	····················	····················
A	3445	hCoV-19/USA/UT-025/2020|EPI_ISL_418967|2020-03-11	····················	····················	····················	····················	····················	····················
A	3446	hCoV-19/USA/NY-NYUMC26/2020|EPI_ISL_418972|2020-03	····················	····················	····················	····················	····················	····················
A	3447	hCoV-19/USA/NY-NYUMC27/2020|EPI_ISL_418973|2020-03	····················	····················	····················	····················	····················	····················
A	3448	hCoV-19/USA/NY-NYUMC28/2020|EPI_ISL_418974|2020-03	····················	····················	····················	····················	····················	····················
A	3449	hCoV-19/USA/NY-NYUMC29/2020|EPI_ISL_418975|2020-03	····················	····················	····················	····················	····················	····················
A	3450	hCoV-19/USA/NY-NYUMC30/2020|EPI_ISL_418976|2020-03	····················	····················	····················	····················	····················	····················
A	3451	hCoV-19/USA/NY-NYUMC31/2020|EPI_ISL_418977|2020-03	····················	····················	····················	····················	····················	····················
A	3452	hCoV-19/USA/NY-NYUMC32/2020|EPI_ISL_418978|2020-03	····················	····················	····················	····················	····················	····················
A	3453	hCoV-19/USA/NY-NYUMC33/2020|EPI_ISL_418979|2020-03	····················	····················	····················	NN··················	····················	····················
A	3454	hCoV-19/Wales/PHWC-260B9/2020|EPI_ISL_422100|2020-	····················	····················	····················	····················	····················	····················
A	3455	hCoV-19/Wales/PHWC-26444/2020|EPI_ISL_422102|2020-	····················	····················	····················	····················	····················	····················
A	3456	hCoV-19/Wales/PHWC-26C00/2020|EPI_ISL_422103|2020-	········Y···········	····················	····················	····················	····················	····················
A	3457	hCoV-19/Wales/PHWC-26022/2020|EPI_ISL_422104|2020-	····················	····A···············	····················	····················	····················	····················
A	3458	hCoV-19/Wales/PHWC-26F25/2020|EPI_ISL_422105|2020-	····················	····················	····················	····················	····················	····················
A	3459	hCoV-19/Wales/PHWC-260F5/2020|EPI_ISL_422106|2020-	····················	····A···············	····················	····················	····················	····················
A	3460	hCoV-19/Wales/PHWC-25FF9/2020|EPI_ISL_422107|2020-	····················	····················	····················	····················	····················	····················
A	3461	hCoV-19/Wales/PHWC-2581C/2020|EPI_ISL_422108|2020-	····················	····················	····················	····················	····················	····················
A	3462	hCoV-19/Wales/PHWC-266F3/2020|EPI_ISL_422109|2020-	····················	····················	····················	····················	····················	····················
A	3463	hCoV-19/Wales/PHWC-260D7/2020|EPI_ISL_422110|2020-	····················	····················	····················	····················	····················	····················
A	3464	hCoV-19/Wales/PHWC-260C8/2020|EPI_ISL_422111|2020-	····················	····················	····················	····················	····················	····················
A	3465	hCoV-19/Wales/PHWC-26417/2020|EPI_ISL_422112|2020-	····················	····················	····················	····················	····················	····················
A	3466	hCoV-19/Wales/PHWC-26CB5/2020|EPI_ISL_422113|2020-	····················	····M···············	····················	····················	····················	····················
A	3467	hCoV-19/Wales/PHWC-25BC8/2020|EPI_ISL_422114|2020-	····················	····················	····················	····················	····················	····················
A	3468	hCoV-19/Wales/PHWC-26EDD/2020|EPI_ISL_422115|2020-	····················	····················	····················	····················	····················	····················
A	3469	hCoV-19/Wales/PHWC-27003/2020|EPI_ISL_422125|2020-	····················	····················	····················	····················	····················	····················
A	3470	hCoV-19/Wales/PHWC-26699/2020|EPI_ISL_422127|2020-	····················	····················	····················	····················	····················	····················
A	3471	hCoV-19/Wales/PHWC-266A8/2020|EPI_ISL_422128|2020-	····················	····················	····················	····················	····················	····················
A	3472	hCoV-19/Wales/PHWC-267A5/2020|EPI_ISL_422129|2020-	····················	····················	····················	····················	····················	····················
A	3473	hCoV-19/Wales/PHWC-26A9D/2020|EPI_ISL_422130|2020-	····················	····················	····················	····················	····················	····················
A	3474	hCoV-19/Wales/PHWC-27030/2020|EPI_ISL_422131|2020-	····················	····················	····················	····················	····················	····················
A	3475	hCoV-19/Wales/PHWC-26F07/2020|EPI_ISL_422132|2020-	····················	····················	····················	····················	····················	····················
A	3476	hCoV-19/Wales/PHWC-270C7/2020|EPI_ISL_422133|2020-	····················	····················	····················	····················	····················	····················
A	3477	hCoV-19/Wales/PHWC-25E65/2020|EPI_ISL_422135|2020-	····················	····················	····················	····················	····················	····················

A	3478 hCoV-19/Wales/PHWC-25EB0/2020|EPI_ISL_422136|2020-	····················	····A···············	····················	····················	····················	····················	
A	3479 hCoV-19/Wales/PHWC-26EEC/2020|EPI_ISL_422137|2020-	····················	····················	····················	····················	····················	····················	
A	3480 hCoV-19/Wales/PHWC-26D94/2020|EPI_ISL_422160|2020-	····················	····················	····················	····················	····················	····················	
A	3481 hCoV-19/Wales/PHWC-26CE2/2020|EPI_ISL_422161|2020-	····················	····················	····················	····················	····················	····················	
A	3482 hCoV-19/Wales/PHWC-26611/2020|EPI_ISL_422162|2020-	····················	·R··················	····················	····················	····················	····················	
A	3483 hCoV-19/Wales/PHWC-26D85/2020|EPI_ISL_422163|2020-	····················	····A···············	····················	····················	····················	····················	
A	3484 hCoV-19/Wales/PHWC-26620/2020|EPI_ISL_422164|2020-	····················	····················	····················	····················	····················	····················	
A	3485 hCoV-19/Wales/PHWC-26C3D/2020|EPI_ISL_422165|2020-	····················	····················	····················	····················	····················	····················	
A	3486 hCoV-19/Wales/PHWC-26927/2020|EPI_ISL_422166|2020-	····················	····················	····················	····················	····················	····················	
A	3487 hCoV-19/Wales/PHWC-266B7/2020|EPI_ISL_422167|2020-	····················	····················	····················	····················	····················	····················	
A	3488 hCoV-19/Wales/PHWC-25F62/2020|EPI_ISL_422168|2020-	····················	····················	····················	····················	····················	····················	
A	3489 hCoV-19/Wales/PHWC-26BA9/2020|EPI_ISL_422170|2020-	····················	····················	····················	····················	····················	····················	
A	3490 hCoV-19/Wales/PHWC-26FDA/2020|EPI_ISL_422171|2020-	····················	····················	····················	····················	····················	····················	
A	3491 hCoV-19/Wales/PHWC-26C4C/2020|EPI_ISL_422173|2020-	····················	····················	····················	····················	····················	····················	
A	3492 hCoV-19/Wales/PHWC-26F70/2020|EPI_ISL_422174|2020-	····················	····················	····················	····················	····················	····················	
A	3493 hCoV-19/Wales/PHWC-25B7D/2020|EPI_ISL_422180|2020-	····················	····················	····················	····················	····················	····················	
A	3494 hCoV-19/Wales/PHWC-261F2/2020|EPI_ISL_422181|2020-	····················	····················	····················	····················	····················	····················	
A	3495 hCoV-19/Wales/PHWC-267D2/2020|EPI_ISL_422182|2020-	····················	····················	····················	····················	····················	····················	
A	3496 hCoV-19/Wales/PHWC-26875/2020|EPI_ISL_422184|2020-	····················	····················	····················	····················	····················	····················	
A	3497 hCoV-19/Wales/PHWC-2680C/2020|EPI_ISL_422185|2020-	····················	····················	····················	····················	····················	····················	
A	3498 hCoV-19/Wales/PHWC-26B4F/2020|EPI_ISL_422186|2020-	····················	····················	····················	····················	····················	····················	
A	3499 hCoV-19/Wales/PHWC-2709A/2020|EPI_ISL_422187|2020-	····················	····················	····················	····················	····················	····················	
A	3500 hCoV-19/Wales/PHWC-26480/2020|EPI_ISL_422191|2020-	····················	·A··················	····················	····················	····················	····················	
A	3501 hCoV-19/Wales/PHWC-265BA/2020|EPI_ISL_422192|2020-	····················	····················	····················	····················	····················	····················	
A	3502 hCoV-19/Wales/PHWC-26963/2020|EPI_ISL_422193|2020-	····················	····················	····················	····················	····················	····················	
A	3503 hCoV-19/Wales/PHWC-2612F/2020|EPI_ISL_422194|2020-	····················	····················	····················	····················	····················	····················	
A	3504 hCoV-19/Wales/PHWC-2659C/2020|EPI_ISL_422195|2020-	····················	····A···············	····················	····················	····················	····················	
A	3506 hCoV-19/England/20140069404/2020|EPI_ISL_423404|20	····················	····················	····················	····················	····················	····················	
A	3509 hCoV-19/England/20140070504/2020|EPI_ISL_423408|20	····················	····················	····················	····················	····················	····················	
A	3510 hCoV-19/England/20140070604/2020|EPI_ISL_423409|20	····················	····················	····················	····················	····················	····················	
A	3511 hCoV-19/England/20140070804/2020|EPI_ISL_423410|20	····················	····················	····················	····················	····················	····················	
A	3512 hCoV-19/England/20140071104/2020|EPI_ISL_423411|20	····················	····················	····················	····················	····················	····················	
A	3514 hCoV-19/England/20140071504/2020|EPI_ISL_423413|20	····················	····················	····················	····················	····················	····················	
A	3515 hCoV-19/England/20140071604/2020|EPI_ISL_423414|20	····················	····················	····················	····················	····················	····················	
A	3516 hCoV-19/England/20140071704/2020|EPI_ISL_423415|20	····················	····················	····················	····················	····················	····················	
A	3517 hCoV-19/England/20140072304/2020|EPI_ISL_423417|20	····················	····················	····················	····················	····················	····················	
A	3518 hCoV-19/England/20140072504/2020|EPI_ISL_423418|20	····················	····················	····················	····················	····················	····················	
A	3524 hCoV-19/England/20142002704/2020|EPI_ISL_423424|20	····················	····················	····················	····················	····················	····················	
A	3525 hCoV-19/England/20142002904/2020|EPI_ISL_423425|20	····················	····················	····················	····················	····················	····················	
A	3526 hCoV-19/England/20142003904/2020|EPI_ISL_423426|20	····················	····A···············	····················	····················	····················	····················	
A	3528 hCoV-19/England/20142014504/2020|EPI_ISL_423430|20	····················	····················	····················	····················	····················	····················	
A	3529 hCoV-19/England/20142015104/2020|EPI_ISL_423432|20	····················	····················	····················	····················	····················	····················	
A	3530 hCoV-19/England/20142016204/2020|EPI_ISL_423435|20	····················	····················	····················	····················	····················	····················	
A	3531 hCoV-19/England/20142016504/2020|EPI_ISL_423436|20	····················	····················	····················	····················	····················	····················	
A	3532 hCoV-19/England/20142016704/2020|EPI_ISL_423437|20	····················	····················	····················	····················	····················	····················	
A	3533 hCoV-19/England/20142018004/2020|EPI_ISL_423438|20	····················	····················	····················	····················	····················	····················	
A	3535 hCoV-19/England/20142018804/2020|EPI_ISL_423441|20	····················	····················	····················	····················	····················	····················	
A	3538 hCoV-19/England/20142019704/2020|EPI_ISL_423444|20	····················	····················	····················	····················	····················	·NN·········N·······	1
A	3539 hCoV-19/England/20142021604/2020|EPI_ISL_423446|20	····················	····················	····················	····················	····················	····················	
A	3542 hCoV-19/England/20142079504/2020|EPI_ISL_423460|20	····················	····················	····················	····················	····················	····················	
A	3543 hCoV-19/England/20142079804/2020|EPI_ISL_423461|20	····················	····················	····················	····················	····················	····················	
A	3545 hCoV-19/England/20142083604/2020|EPI_ISL_423465|20	····················	····················	····················	····················	··················N·	····················	1
A	3546 hCoV-19/England/20142083804/2020|EPI_ISL_423466|20	····················	····················	····················	····················	····················	····················	
A	3547 hCoV-19/England/20142130404/2020|EPI_ISL_423467|20	····················	····················	····················	····················	····················	····················	
A	3548 hCoV-19/England/20142130504/2020|EPI_ISL_423468|20	····················	····················	····················	····················	····················	····················	
A	3552 hCoV-19/England/20144006304/2020|EPI_ISL_423493|20	····················	····················	····················	····················	····················	····················	
A	3554 hCoV-19/England/20144006704/2020|EPI_ISL_423495|20	····················	····················	····················	····················	····················	····················	
A	3555 hCoV-19/England/20144006904/2020|EPI_ISL_423496|20	····················	····················	····················	····················	····················	····················	
A	3556 hCoV-19/England/20144007604/2020|EPI_ISL_423497|20	····················	····················	····················	····················	····················	·NN·················	
A	3557 hCoV-19/England/20144008004/2020|EPI_ISL_423498|20	····················	····················	····················	····················	····················	···N················	
A	3559 hCoV-19/USA/WA-UW-4549/2020|EPI_ISL_429600|2020-03	····················	····················	····················	····················	····················	····················	
A	3560 hCoV-19/USA/ID-UW-4552/2020|EPI_ISL_429601|2020-03	····················	····················	····················	····················	····················	····················	
A	3561 hCoV-19/USA/WA-UW-4564/2020|EPI_ISL_429602|2020-03	····················	····················	····················	····················	····················	····················	
A	3562 hCoV-19/USA/WA-UW-4565/2020|EPI_ISL_429603|2020-03	····················	····················	····················	····················	····················	····················	
A	3563 hCoV-19/USA/WA-UW-4568/2020|EPI_ISL_429604|2020-03	····················	····················	····················	····················	····················	····················	
A	3564 hCoV-19/USA/WA-UW-4578/2020|EPI_ISL_429605|2020-03	····················	····················	····················	····················	····················	····················	
A	3565 hCoV-19/USA/WA-UW-4579/2020|EPI_ISL_429606|2020-03	····················	····················	····················	····················	····················	····················	
A	3566 hCoV-19/USA/WA-UW-4593/2020|EPI_ISL_429608|2020-03	····················	····················	····················	····················	····················	····················	
A	3567 hCoV-19/USA/WA-UW-4594/2020|EPI_ISL_429609|2020-03	····················	····················	····················	····················	····················	····················	
A	3568 hCoV-19/USA/ID-UW-4598/2020|EPI_ISL_429610|2020-03	····················	····················	····················	····················	····················	····················	
A	3569 hCoV-19/USA/WA-UW-4602/2020|EPI_ISL_429611|2020-03	····················	····················	····················	····················	····················	····················	
A	3570 hCoV-19/USA/WA-UW-4609/2020|EPI_ISL_429612|2020-03	····················	····················	····················	····················	····················	····················	
A	3571 hCoV-19/USA/WA-UW-4611/2020|EPI_ISL_429613|2020-03	····················	····················	····················	····················	····················	····················	
A	3572 hCoV-19/USA/WA-UW-4612/2020|EPI_ISL_429614|2020-03	····················	····················	····················	····················	····················	····················	
A	3573 hCoV-19/USA/WA-UW-4703/2020|EPI_ISL_429615|2020-03	····················	····················	····················	····················	····················	····················	
A	3574 hCoV-19/USA/WA-UW-4732/2020|EPI_ISL_429616|2020-03	····················	····················	····················	····················	····················	····················	
A	3575 hCoV-19/USA/WA-UW-4734/2020|EPI_ISL_429617|2020-03	····················	····················	····················	····················	····················	····················	
A	3576 hCoV-19/USA/WA-UW-4741/2020|EPI_ISL_429618|2020-03	····················	····················	····················	····················	····················	····················	

A	3577 hCoV-19/USA/WA-UW-4760/2020|EPI_ISL_429621|2020-03	····················	····················	····················	····················	····················	····················
A	3578 hCoV-19/USA/WA-UW-4763/2020|EPI_ISL_429622|2020-03	····················	····················	····················	····················	····················	····················
A	3579 hCoV-19/USA/WA-UW-4766/2020|EPI_ISL_429623|2020-03	····················	····················	····················	····················	····················	····················
A	3580 hCoV-19/USA/WA-UW-4769/2020|EPI_ISL_429624|2020-03	····················	····················	····················	····················	····················	····················
A	3581 hCoV-19/USA/WA-UW-4770/2020|EPI_ISL_429625|2020-03	····················	····················	····················	····················	····················	····················
A	3582 hCoV-19/USA/WA-UW-4775/2020|EPI_ISL_429626|2020-03	····················	····················	····················	····················	····················	····················
A	3583 hCoV-19/USA/WA-UW-4777/2020|EPI_ISL_429627|2020-03	····················	····················	····················	····················	····················	····················
A	3584 hCoV-19/USA/UN-UW-4645/2020|EPI_ISL_429628|2020-04	····················	····················	····················	····················	····················	····················
A	3585 hCoV-19/Belgium/GHB-03021/2020|EPI_ISL_407976|2020	····················	····················	····················	····················	····················	····················
A	3586 hCoV-19/Singapore/2/2020|EPI_ISL_407987|2020-01-25	····················	····················	····················	····················	····················	····················
A	3587 hCoV-19/Singapore/3/2020|EPI_ISL_407988|2020-02-01	····················	····················	····················	····················	····················	····················
A	3588 hCoV-19/Weifang/WF0029/2020|EPI_ISL_413809|2020-02	····················	····················	····················	····················	····················	····················
A	3589 hCoV-19/England/20110000606/2020|EPI_ISL_417301|20	····················	····················	····················	····················	····················	····················
A	3590 hCoV-19/England/20110000706/2020|EPI_ISL_417302|20	····················	····················	····················	····················	····················	····················
A	3591 hCoV-19/England/20110015106/2020|EPI_ISL_417306|20	····················	····················	····················	····················	····················	····················
A	3592 hCoV-19/England/20110023706/2020|EPI_ISL_417307|20	····················	····················	····················	····················	····················	····················
A	3593 hCoV-19/England/20110058706/2020|EPI_ISL_417311|20	····················	····················	····················	····················	····················	····················
A	3594 hCoV-19/England/20110059306/2020|EPI_ISL_417312|20	····················	····················	····················	····················	····················	····················
A	3595 hCoV-19/England/20110097506/2020|EPI_ISL_417313|20	····················	····················	····················	····················	····················	····················
A	3597 hCoV-19/England/20118044606/2020|EPI_ISL_417315|20	····················	····················	····················	····················	····················	····················
A	3598 hCoV-19/USA/CA-SCCPHD-UC13/2020|EPI_ISL_417317|202	····················	····················	····················	····················	····················	····················
A	3599 hCoV-19/USA/CA-SCCPHD-UC14/2020|EPI_ISL_417318|202	····················	····················	····················	····················	····················	····················
A	3600 hCoV-19/France/ARA-06813/2020|EPI_ISL_417340|2020-	····················	····················	····················	····················	····················	····················
A	3601 hCoV-19/USA/WA-UW192/2020|EPI_ISL_417341|2020-03-1	····················	····················	····················	····················	····················	····················
A	3602 hCoV-19/USA/WA-UW193/2020|EPI_ISL_417342|2020-03-1	····················	····················	····················	····················	····················	····················
A	3603 hCoV-19/USA/WA-UW194/2020|EPI_ISL_417343|2020-03-1	····················	····················	····················	····················	····················	····················
A	3604 hCoV-19/USA/WA-UW195/2020|EPI_ISL_417344|2020-03-1	····················	····················	····················	····················	····················	····················
A	3605 hCoV-19/USA/WA-UW221/2020|EPI_ISL_417370|2020-03-1	····················	····················	····················	····················	····················	····················
A	3606 hCoV-19/USA/WA-UW222/2020|EPI_ISL_417371|2020-03-1	····················	····················	····················	····················	····················	····················
A	3607 hCoV-19/USA/CT-UW223/2020|EPI_ISL_417372|2020-03-1	····················	····················	····················	····················	····················	····················
A	3608 hCoV-19/USA/CT-UW224/2020|EPI_ISL_417373|2020-03-1	····················	····················	····················	····················	····················	····················
A	3609 hCoV-19/USA/WA-UW225/2020|EPI_ISL_417374|2020-03-1	····················	····················	····················	····················	····················	····················
A	3610 hCoV-19/USA/WA-UW226/2020|EPI_ISL_417375|2020-03-1	····················	····················	····················	····················	····················	····················
A	3611 hCoV-19/USA/WA-UW227/2020|EPI_ISL_417376|2020-03-1	····················	····················	····················	····················	····················	····················
A	3612 hCoV-19/USA/WA-UW228/2020|EPI_ISL_417377|2020-03-1	····················	····················	····················	····················	····················	····················
A	3613 hCoV-19/USA/WA-UW229/2020|EPI_ISL_417378|2020-03-1	····················	····················	····················	····················	····················	····················
A	3614 hCoV-19/USA/WA-UW230/2020|EPI_ISL_417379|2020-03-1	····················	····················	····················	····················	····················	····················
A	3615 hCoV-19/USA/WA-UW231/2020|EPI_ISL_417380|2020-03-1	····················	····················	····················	····················	····················	····················
A	3616 hCoV-19/USA/WA-UW232/2020|EPI_ISL_417381|2020-03-1	····················	····················	····················	····················	····················	····················
A	3617 hCoV-19/USA/WA-UW233/2020|EPI_ISL_417382|2020-03-1	····················	····················	····················	····················	····················	····················
A	3618 hCoV-19/Australia/NSW15/2020|EPI_ISL_417383|2020-0	····················	····················	····················	····················	····················	····················
A	3619 hCoV-19/Australia/NSW18/2020|EPI_ISL_417384|2020-0	····················	····················	····················	····················	····················	····················
A	3620 hCoV-19/Australia/NSW19/2020|EPI_ISL_417385|2020-0	····················	····················	····················	····················	····················	····················
A	3621 hCoV-19/Australia/NSW21/2020|EPI_ISL_417386|2020-0	····················	····················	····················	····················	····················	····················
A	3622 hCoV-19/Australia/NSW24/2020|EPI_ISL_417387|2020-0	····················	····················	····················	····················	····················	····················
A	3623 hCoV-19/Australia/NSW25/2020|EPI_ISL_417388|2020-0	····················	····················	····················	····················	····················	····················
A	3624 hCoV-19/Australia/NSW26/2020|EPI_ISL_417389|2020-0	····················	····················	····················	····················	····················	····················
A	3625 hCoV-19/Australia/NSW27/2020|EPI_ISL_417390|2020-0	····················	····················	····················	····················	····················	····················
A	3626 hCoV-19/Australia/NSW28/2020|EPI_ISL_417391|2020-0	····················	····················	····················	····················	····················	····················
A	3627 hCoV-19/Australia/NSW31/2020|EPI_ISL_417392|2020-0	····················	····················	····················	····················	····················	····················
A	3628 hCoV-19/Australia/NSW35/2020|EPI_ISL_417393|2020-0	····················	····················	····················	····················	····················	····················
A	3629 hCoV-19/Australia/NSW36/2020|EPI_ISL_417394|2020-0	····················	····················	····················	····················	····················	····················
A	3630 hCoV-19/Australia/NSW37/2020|EPI_ISL_417395|2020-0	····················	····················	····················	····················	····················	····················
A	3631 hCoV-19/Australia/NSW38/2020|EPI_ISL_417396|2020-0	····················	····················	····················	····················	····················	····················
A	3632 hCoV-19/Australia/NSW39/2020|EPI_ISL_417397|2020-0	····················	····················	····················	····················	····················	····················
A	3633 hCoV-19/Australia/NSW40/2020|EPI_ISL_417398|2020-0	····················	····················	····················	····················	····················	····················
A	3634 hCoV-19/Australia/NSW41/2020|EPI_ISL_417399|2020-0	····················	····················	····················	····················	····················	····················
A	3635 hCoV-19/Belgium/ULG-6972/2020|EPI_ISL_418624|2020-	····················	····················	····················	····················	····················	····················
A	3636 hCoV-19/Belgium/ULG-6688/2020|EPI_ISL_418625|2020-	····················	····················	····················	····················	····················	····················
A	3637 hCoV-19/Belgium/ULG-7242/2020|EPI_ISL_418626|2020-	····················	··········A·········	····················	····················	····················	····················
A	3638 hCoV-19/Belgium/ULG-6743/2020|EPI_ISL_418627|2020-	····················	····················	····················	····················	····················	····················
A	3640 hCoV-19/Belgium/ULG-7773/2020|EPI_ISL_418629|2020-	····················	····················	····················	····················	····················	····················
A	3641 hCoV-19/Belgium/ULG-7729/2020|EPI_ISL_418630|2020-	····················	····················	····················	····················	····················	····················
A	3642 hCoV-19/Belgium/ULG-6649/2020|EPI_ISL_418631|2020-	····················	····················	····················	····················	····················	····················
A	3643 hCoV-19/Belgium/ULG-7500/2020|EPI_ISL_418632|2020-	····················	····················	····················	····················	····················	····················
A	3645 hCoV-19/Belgium/ULG-8595/2020|EPI_ISL_418634|2020-	····················	····················	····················	····················	····················	····················
A	3646 hCoV-19/Belgium/ULG-8532/2020|EPI_ISL_418635|2020-	····················	····················	····················	····················	····················	····················
A	3647 hCoV-19/Belgium/ULG-8541/2020|EPI_ISL_418636|2020-	····················	····················	····················	····················	····················	····················
A	3648 hCoV-19/Belgium/ULG-8578/2020|EPI_ISL_418638|2020-	····················	····················	····················	····················	····················	····················
A	3649 hCoV-19/Belgium/ULG-8617/2020|EPI_ISL_418639|2020-	····················	····················	····················	····················	····················	····················
A	3650 hCoV-19/Belgium/ULG-8634/2020|EPI_ISL_418640|2020-	····················	····················	····················	····················	··············N·····	····················
A	3651 hCoV-19/Belgium/ULG-9558/2020|EPI_ISL_418645|2020-	····················	····················	····················	····················	····················	····················
A	3652 hCoV-19/Belgium/ULG-9572/2020|EPI_ISL_418646|2020-	····················	····················	····················	····················	····················	····················
A	3653 hCoV-19/Belgium/ULG-9618/2020|EPI_ISL_418648|2020-	····················	····················	····················	····················	····················	····················
A	3655 hCoV-19/Belgium/ULG-9620/2020|EPI_ISL_418650|2020-	····················	····················	····················	····················	····················	····················
A	3656 hCoV-19/Belgium/ULG-9634/2020|EPI_ISL_418651|2020-	····················	····················	····················	····················	····················	····················
A	3657 hCoV-19/Belgium/ULG-9641/2020|EPI_ISL_418652|2020-	····················	····················	····················	····················	····················	····················
A	3658 hCoV-19/Belgium/ULG-9647/2020|EPI_ISL_418653|2020-	····················	····················	····················	····················	····················	····················

A	3659 hCoV-19/Belgium/ULG-9694/2020|EPI_ISL_418654|2020-	····················	····················	····················	····················	····················	····················	
A	3660 hCoV-19/Belgium/ULG-9714/2020|EPI_ISL_418655|2020-	····················	····················	····················	····················	····················	····················	
A	3661 hCoV-19/Belgium/ULG-9715/2020|EPI_ISL_418656|2020-	····················	····················	····················	····················	··············N·····	NN·····N············	1
A	3662 hCoV-19/Belgium/ULG-9716/2020|EPI_ISL_418657|2020-	····················	····················	····················	····················	····················	····················	
A	3663 hCoV-19/Belgium/ULG-9725/2020|EPI_ISL_418659|2020-	····················	····················	····················	····················	····················	····················	
A	3664 hCoV-19/England/20116025603/2020|EPI_ISL_418670|20	····················	····················	····················	····················	····················	····················	
A	3665 hCoV-19/England/20119026102/2020|EPI_ISL_418671|20	····················	····················	····················	····················	····················	····················	
A	3666 hCoV-19/England/20122032802/2020|EPI_ISL_418673|20	····················	····················	····················	····················	····················	····················	
A	3667 hCoV-19/England/20122073102/2020|EPI_ISL_418675|20	····················	····················	····················	····················	····················	····················	
A	3668 hCoV-19/Australia/VIC254/2020|EPI_ISL_419949|2020-	····················	·A··················	····················	····················	····················	····················	
A	3669 hCoV-19/Australia/VIC257/2020|EPI_ISL_419950|2020-	····················	·A··················	····················	····················	····················	····················	
A	3670 hCoV-19/Australia/VIC258/2020|EPI_ISL_419951|2020-	····················	····················	····················	····················	····················	····················	
A	3671 hCoV-19/Australia/VIC259/2020|EPI_ISL_419952|2020-	····················	····················	····················	····················	····················	····················	
A	3672 hCoV-19/Australia/VIC260/2020|EPI_ISL_419953|2020-	····················	····················	····················	····················	····················	····················	
A	3673 hCoV-19/Australia/VIC261/2020|EPI_ISL_419954|2020-	····················	····················	····················	····················	····················	····················	
A	3674 hCoV-19/Australia/VIC262/2020|EPI_ISL_419955|2020-	····················	····················	····················	····················	····················	····················	
A	3675 hCoV-19/Australia/VIC263/2020|EPI_ISL_419956|2020-	····················	····················	····················	····················	····················	····················	
A	3676 hCoV-19/Australia/VIC264/2020|EPI_ISL_419957|2020-	····················	····················	····················	····················	······A·············	····················	
A	3677 hCoV-19/Australia/VIC265/2020|EPI_ISL_419958|2020-	····················	··M·················	····················	····················	····················	····················	
A	3678 hCoV-19/Australia/VIC266/2020|EPI_ISL_419959|2020-	····················	····················	····················	····················	······A·············	····················	
A	3679 hCoV-19/Australia/VIC267/2020|EPI_ISL_419960|2020-	····················	····················	····················	····················	····················	····················	
A	3680 hCoV-19/Australia/VIC268/2020|EPI_ISL_419961|2020-	····················	····················	····················	····················	····················	····················	
A	3681 hCoV-19/Australia/VIC269/2020|EPI_ISL_419962|2020-	····················	····················	····················	····················	····················	····················	
A	3682 hCoV-19/Australia/VIC270/2020|EPI_ISL_419963|2020-	····················	····················	····················	····················	····················	····················	
A	3683 hCoV-19/Australia/VIC271/2020|EPI_ISL_419964|2020-	····················	····················	····················	····················	····················	····················	
A	3684 hCoV-19/Australia/VIC272/2020|EPI_ISL_419965|2020-	····················	····················	····················	····················	····················	····················	
A	3685 hCoV-19/Australia/VIC273/2020|EPI_ISL_419966|2020-	····················	····················	····················	····················	····················	····················	
A	3686 hCoV-19/Australia/VIC274/2020|EPI_ISL_419967|2020-	····················	····················	····················	····················	····················	····················	
A	3687 hCoV-19/Australia/VIC275/2020|EPI_ISL_419968|2020-	····················	····················	····················	····················	····················	····················	
A	3688 hCoV-19/Australia/VIC276/2020|EPI_ISL_419969|2020-	····················	····················	····················	····················	····················	····················	
A	3689 hCoV-19/Australia/VIC277/2020|EPI_ISL_419970|2020-	····················	····················	····················	····················	····················	····················	
A	3690 hCoV-19/Australia/VIC278/2020|EPI_ISL_419971|2020-	····················	····················	····················	····················	····················	····················	
A	3691 hCoV-19/Australia/VIC279/2020|EPI_ISL_419972|2020-	····················	····················	····················	····················	····················	····················	
A	3692 hCoV-19/Australia/VIC280/2020|EPI_ISL_419973|2020-	····················	····················	····················	····················	····················	····················	
A	3693 hCoV-19/Australia/VIC283/2020|EPI_ISL_419974|2020-	····················	····················	····················	····················	····················	····················	
A	3694 hCoV-19/Australia/VIC284/2020|EPI_ISL_419975|2020-	····················	····················	····················	····················	····················	····················	
A	3695 hCoV-19/Australia/VIC285/2020|EPI_ISL_419976|2020-	····················	····················	····················	····················	····················	····················	
A	3696 hCoV-19/Australia/VIC286/2020|EPI_ISL_419977|2020-	····················	····················	····················	····················	····················	····················	
A	3697 hCoV-19/Australia/VIC287/2020|EPI_ISL_419978|2020-	····················	····················	····················	····················	····················	····················	
A	3698 hCoV-19/Australia/VIC288/2020|EPI_ISL_419979|2020-	····················	····················	····················	····················	····················	····················	
A	3699 hCoV-19/Australia/VIC289/2020|EPI_ISL_419980|2020-	····················	····················	····················	····················	····················	····················	
A	3700 hCoV-19/Australia/VIC291/2020|EPI_ISL_419982|2020-	····················	····················	····················	····················	····················	····················	
A	3701 hCoV-19/Australia/VIC292/2020|EPI_ISL_419983|2020-	····················	····················	····················	····················	····················	····················	
A	3702 hCoV-19/Australia/VIC293/2020|EPI_ISL_419984|2020-	····················	····················	····················	····················	····················	····················	
A	3703 hCoV-19/Australia/VIC294/2020|EPI_ISL_419985|2020-	····················	····················	····················	····················	····················	····················	
A	3704 hCoV-19/Australia/VIC295/2020|EPI_ISL_419986|2020-	····················	····················	····················	····················	····················	····················	
A	3705 hCoV-19/Australia/VIC296/2020|EPI_ISL_419987|2020-	····················	····················	····················	····················	····················	····················	
A	3706 hCoV-19/Australia/VIC297/2020|EPI_ISL_419988|2020-	····················	····················	····················	····················	····················	····················	
A	3707 hCoV-19/Australia/VIC298/2020|EPI_ISL_419989|2020-	····················	····················	····················	····················	····················	····················	
A	3708 hCoV-19/Wales/PHWC-24CB7/2020|EPI_ISL_420937|2020-	····················	····A···············	····················	····················	····················	····················	
A	3709 hCoV-19/Wales/PHWC-24DD2/2020|EPI_ISL_420940|2020-	····················	····················	····················	····················	····················	····················	
A	3710 hCoV-19/Wales/PHWC-24F36/2020|EPI_ISL_420943|2020-	····················	····················	····················	····················	····················	····················	
A	3711 hCoV-19/Wales/PHWC-24FFA/2020|EPI_ISL_420944|2020-	····················	····················	····················	····················	····················	····················	
A	3712 hCoV-19/Wales/PHWC-2522D/2020|EPI_ISL_420949|2020-	····················	····················	····················	····················	····················	····················	
A	3713 hCoV-19/Wales/PHWC-24CF3/2020|EPI_ISL_420951|2020-	····················	····A···············	····················	····················	····················	····················	
A	3714 hCoV-19/Wales/PHWC-24F72/2020|EPI_ISL_420960|2020-	····················	····A···············	····················	····················	····················	····················	
A	3715 hCoV-19/Wales/PHWC-24F81/2020|EPI_ISL_420961|2020-	····················	····A···············	····················	····················	····················	····················	
A	3716 hCoV-19/Wales/PHWC-25050/2020|EPI_ISL_420965|2020-	····················	····················	····················	····················	····················	····················	
A	3717 hCoV-19/Wales/PHWC-25111/2020|EPI_ISL_420969|2020-	····················	····A···············	····················	····················	····················	····················	
A	3718 hCoV-19/Wales/PHWC-25120/2020|EPI_ISL_420970|2020-	····················	····A···············	····················	····················	····················	····················	
A	3719 hCoV-19/Wales/PHWC-25418/2020|EPI_ISL_420990|2020-	····················	····················	····················	····················	····················	····················	
A	3720 hCoV-19/Wales/PHWC-25B04/2020|EPI_ISL_422116|2020-	····················	····················	····················	····················	····················	····················	
A	3721 hCoV-19/Wales/PHWC-25F71/2020|EPI_ISL_422117|2020-	····················	····················	····················	····················	····················	····················	
A	3722 hCoV-19/Wales/PHWC-25F9F/2020|EPI_ISL_422118|2020-	····················	····················	····················	····················	····················	····················	
A	3723 hCoV-19/Wales/PHWC-26C5B/2020|EPI_ISL_422119|2020-	····················	····················	····················	····················	····················	····················	
A	3724 hCoV-19/Wales/PHWC-26FBC/2020|EPI_ISL_422120|2020-	····················	····················	····················	····················	····················	····················	
A	3725 hCoV-19/Wales/PHWC-26C97/2020|EPI_ISL_422121|2020-	····················	····A···············	····················	····················	····················	····················	
A	3726 hCoV-19/Wales/PHWC-26E73/2020|EPI_ISL_422122|2020-	····················	····················	····················	····················	····················	····················	
A	3727 hCoV-19/Wales/PHWC-26FCB/2020|EPI_ISL_422123|2020-	····················	····················	····················	····················	····················	····················	
A	3728 hCoV-19/Wales/PHWC-2715B/2020|EPI_ISL_422124|2020-	····················	····················	····················	····················	····················	····················	
A	3729 hCoV-19/Wales/PHWC-267B4/2020|EPI_ISL_422126|2020-	····················	····················	····················	····················	····················	····················	
A	3730 hCoV-19/Wales/PHWC-259A0/2020|EPI_ISL_422138|2020-	····················	····················	····················	····················	····················	····················	
A	3731 hCoV-19/Wales/PHWC-25867/2020|EPI_ISL_422139|2020-	····················	····A···············	····················	····················	····················	····················	
A	3732 hCoV-19/Wales/PHWC-26F8F/2020|EPI_ISL_422140|2020-	····················	····················	····················	····················	····················	····················	
A	3733 hCoV-19/Wales/PHWC-26893/2020|EPI_ISL_422141|2020-	····················	····················	····················	····················	····················	····················	
A	3734 hCoV-19/Wales/PHWC-2613E/2020|EPI_ISL_422142|2020-	····················	····················	····················	····················	····················	····················	
A	3735 hCoV-19/Wales/PHWC-26E64/2020|EPI_ISL_422143|2020-	····················	····················	····················	····················	····················	····················	
A	3736 hCoV-19/Wales/PHWC-26F43/2020|EPI_ISL_422144|2020-	····················	····················	····················	····················	····················	····················	

A	3737	hCoV-19/Wales/PHWC-26EA0/2020|EPI_ISL_422145|2020-	····················	····················	····················	····················	····················	····················
A	3738	hCoV-19/Wales/PHWC-26D2B/2020|EPI_ISL_422146|2020-	····················	····A···············	····················	····················	····················	····················
A	3739	hCoV-19/Wales/PHWC-2706D/2020|EPI_ISL_422147|2020-	····················	····················	····················	····················	····················	····················
A	3740	hCoV-19/Wales/PHWC-26BC7/2020|EPI_ISL_422148|2020-	····················	····················	····················	····················	····················	····················
A	3741	hCoV-19/Wales/PHWC-26004/2020|EPI_ISL_422149|2020-	····················	····················	····················	····················	····················	····················
A	3742	hCoV-19/Wales/PHWC-25E0B/2020|EPI_ISL_422150|2020-	····················	····················	····················	····················	····················	····················
A	3743	hCoV-19/Wales/PHWC-26C1F/2020|EPI_ISL_422151|2020-	····················	····················	····················	····················	····················	····················
A	3744	hCoV-19/Wales/PHWC-26FAD/2020|EPI_ISL_422152|2020-	····················	····A···············	····················	····················	····················	····················
A	3745	hCoV-19/Wales/PHWC-257C4/2020|EPI_ISL_422153|2020-	····················	····A···············	····················	····················	····················	····················
A	3746	hCoV-19/Wales/PHWC-25C10/2020|EPI_ISL_422154|2020-	····················	····················	····················	····················	····················	····················
A	3747	hCoV-19/Wales/PHWC-2713D/2020|EPI_ISL_422155|2020-	····················	····················	····················	····················	····················	····················
A	3748	hCoV-19/Wales/PHWC-26C88/2020|EPI_ISL_422156|2020-	····················	····················	····················	····················	····················	····················
A	3749	hCoV-19/Wales/PHWC-26787/2020|EPI_ISL_422157|2020-	····················	····················	····················	····················	····················	····················
A	3750	hCoV-19/Wales/PHWC-266D5/2020|EPI_ISL_422158|2020-	····················	····················	····················	····················	····················	····················
A	3751	hCoV-19/Wales/PHWC-26E28/2020|EPI_ISL_422159|2020-	····················	····················	····················	····················	····················	····················
A	3752	hCoV-19/England/20142022104/2020|EPI_ISL_423447|20	····················	····················	····················	····················	····················	····················
A	3753	hCoV-19/England/20142023704/2020|EPI_ISL_423448|20	····················	····················	····················	····················	····················	····················
A	3758	hCoV-19/England/20142026304/2020|EPI_ISL_423453|20	····················	····················	····················	····················	····················	····················
A	3763	hCoV-19/England/20144000204/2020|EPI_ISL_423472|20	····················	····················	····················	····················	····················	····················
A	3764	hCoV-19/England/20144000404/2020|EPI_ISL_423473|20	····················	····················	····················	····················	····················	····················
A	3766	hCoV-19/England/20144001204/2020|EPI_ISL_423476|20	····················	····················	····················	····················	····················	····················
A	3768	hCoV-19/England/20144002904/2020|EPI_ISL_423479|20	····················	····················	····················	····················	····················	····················
A	3770	hCoV-19/England/20144003604/2020|EPI_ISL_423481|20	····················	····················	····················	····················	····················	····················
A	3772	hCoV-19/England/20144004504/2020|EPI_ISL_423486|20	····················	····················	····················	····················	····················	····················
A	3773	hCoV-19/England/20144004704/2020|EPI_ISL_423487|20	····················	····················	····················	····················	····················	····················
A	3774	hCoV-19/England/20144004804/2020|EPI_ISL_423488|20	····················	····················	····················	····················	····················	····················
A	3775	hCoV-19/Scotland/CVR69/2020|EPI_ISL_425800|2020-03	····················	····················	····················	····················	····················	····················
A	3776	hCoV-19/Scotland/CVR70/2020|EPI_ISL_425801|2020-03	····················	····················	····················	····················	····················	····················
A	3777	hCoV-19/Scotland/CVR71/2020|EPI_ISL_425802|2020-03	····················	····················	····················	····················	····················	····················
A	3778	hCoV-19/Scotland/CVR72/2020|EPI_ISL_425803|2020-03	····················	····················	····················	····················	····················	····················
A	3779	hCoV-19/Scotland/CVR74/2020|EPI_ISL_425804|2020-03	····················	····················	····················	····················	····················	····················
A	3780	hCoV-19/Scotland/CVR75/2020|EPI_ISL_425805|2020-03	····················	····················	····················	····················	····················	····················
A	3781	hCoV-19/Scotland/CVR76/2020|EPI_ISL_425806|2020-03	····················	····················	····················	····················	····················	····················
A	3782	hCoV-19/Scotland/CVR77/2020|EPI_ISL_425807|2020-03	····················	····················	····················	····················	····················	····················
A	3783	hCoV-19/Scotland/CVR78/2020|EPI_ISL_425808|2020-03	····················	····················	····················	····················	····················	····················
A	3784	hCoV-19/Scotland/CVR79/2020|EPI_ISL_425809|2020-03	····················	····················	····················	····················	····················	····················
A	3785	hCoV-19/Scotland/CVR80/2020|EPI_ISL_425810|2020-03	····················	····················	····················	····················	····················	····················
A	3786	hCoV-19/Scotland/CVR83/2020|EPI_ISL_425811|2020-03	····················	····················	····················	····················	····················	····················
A	3787	hCoV-19/Scotland/CVR85/2020|EPI_ISL_425812|2020-03	····················	····················	····················	····················	····················	····················
A	3788	hCoV-19/Scotland/CVR89/2020|EPI_ISL_425813|2020-03	····················	····················	····················	····················	····················	····················
A	3789	hCoV-19/Scotland/CVR91/2020|EPI_ISL_425815|2020-03	····················	·A··················	····················	····················	····················	····················
A	3790	hCoV-19/Scotland/CVR96/2020|EPI_ISL_425816|2020-03	····················	·A··················	····················	····················	····················	····················
A	3791	hCoV-19/Scotland/CVR97/2020|EPI_ISL_425817|2020-03	····················	····················	····················	····················	····················	····················
A	3792	hCoV-19/Scotland/CVR99/2020|EPI_ISL_425818|2020-03	····················	····················	····················	····················	····················	····················
A	3793	hCoV-19/Scotland/EDB002/2020|EPI_ISL_425819|2020-0	····················	····················	····················	····················	····················	····················
A	3795	hCoV-19/Scotland/EDB015/2020|EPI_ISL_425821|2020-0	····················	····················	····················	····················	····················	····················
A	3796	hCoV-19/Scotland/EDB016/2020|EPI_ISL_425822|2020-0	····················	····················	····················	····················	····················	····················
A	3797	hCoV-19/Denmark/ALAB-HH46/2020|EPI_ISL_429300|2020	····················	····················	····················	····················	····················	····················
A	3798	hCoV-19/Denmark/ALAB-HH49/2020|EPI_ISL_429301|2020	····················	····················	····················	····················	····················	····················
A	3799	hCoV-19/Denmark/ALAB-HH50/2020|EPI_ISL_429302|2020	····················	····················	····················	····················	····················	····················
A	3800	hCoV-19/Denmark/ALAB-HH51/2020|EPI_ISL_429303|2020	····················	····················	····················	····················	····················	····················
A	3801	hCoV-19/Denmark/ALAB-HH56/2020|EPI_ISL_429304|2020	····················	····················	····················	····················	····················	····················
A	3802	hCoV-19/Denmark/ALAB-HH57/2020|EPI_ISL_429305|2020	····················	····················	····················	····················	····················	····················
A	3803	hCoV-19/Denmark/ALAB-HH60/2020|EPI_ISL_429306|2020	····················	····················	····················	····················	····················	····················
A	3804	hCoV-19/Denmark/ALAB-HH61/2020|EPI_ISL_429307|2020	····················	····················	····················	····················	····················	····················
A	3805	hCoV-19/Denmark/ALAB-HH62/2020|EPI_ISL_429308|2020	····················	····················	····················	····················	····················	····················
A	3806	hCoV-19/Denmark/ALAB-HH63/2020|EPI_ISL_429309|2020	····················	····················	····················	····················	····················	····················
A	3807	hCoV-19/Denmark/ALAB-SSI121/2020|EPI_ISL_429350|20	····················	····················	····················	····················	····················	····················
A	3808	hCoV-19/Denmark/ALAB-SSI122/2020|EPI_ISL_429351|20	····················	····················	····················	····················	····················	····················
A	3809	hCoV-19/Denmark/ALAB-SSI123/2020|EPI_ISL_429352|20	····················	····················	····················	····················	····················	····················
A	3810	hCoV-19/Denmark/ALAB-SSI125/2020|EPI_ISL_429353|20	····················	····················	····················	····················	····················	····················
A	3811	hCoV-19/Denmark/ALAB-SSI126/2020|EPI_ISL_429354|20	····················	····················	····················	····················	····················	····················
A	3812	hCoV-19/Denmark/ALAB-SSI128/2020|EPI_ISL_429355|20	····················	····················	····················	····················	····················	····················
A	3813	hCoV-19/Denmark/ALAB-SSI129/2020|EPI_ISL_429356|20	····················	····················	····················	····················	····················	····················
A	3814	hCoV-19/Denmark/ALAB-SSI132/2020|EPI_ISL_429357|20	····················	····················	····················	····················	····················	····················
A	3815	hCoV-19/Denmark/ALAB-SSI139/2020|EPI_ISL_429358|20	····················	····················	····················	····················	····················	····················
A	3816	hCoV-19/Denmark/ALAB-SSI142/2020|EPI_ISL_429360|20	····················	····················	····················	····················	····················	····················
A	3817	hCoV-19/Denmark/ALAB-SSI143/2020|EPI_ISL_429361|20	····················	····················	····················	····················	····················	····················
A	3818	hCoV-19/Denmark/ALAB-SSI146/2020|EPI_ISL_429364|20	····················	····················	····················	····················	····················	····················
A	3819	hCoV-19/Denmark/ALAB-SSI150/2020|EPI_ISL_429368|20	····················	····················	····················	····················	····················	····················
A	3820	hCoV-19/Denmark/ALAB-SSI151/2020|EPI_ISL_429369|20	····················	····················	····················	····················	····················	····················
A	3821	hCoV-19/Denmark/ALAB-SSI154/2020|EPI_ISL_429370|20	····················	····················	····················	····················	····················	····················
A	3822	hCoV-19/Denmark/ALAB-SSI155/2020|EPI_ISL_429371|20	····················	····················	····················	····················	····················	····················
A	3823	hCoV-19/Denmark/ALAB-SSI156/2020|EPI_ISL_429372|20	····················	····················	····················	····················	····················	····················
A	3824	hCoV-19/Denmark/ALAB-SSI157/2020|EPI_ISL_429373|20	····················	····················	····················	····················	····················	····················
A	3825	hCoV-19/Denmark/ALAB-SSI158/2020|EPI_ISL_429374|20	····················	····················	····················	····················	····················	····················
A	3826	hCoV-19/Denmark/ALAB-SSI159/2020|EPI_ISL_429375|20	····················	····················	····················	····················	····················	····················
A	3827	hCoV-19/Denmark/ALAB-SSI160/2020|EPI_ISL_429376|20	····················	····················	····················	····················	····················	····················

A	3828	hCoV-19/Denmark/ALAB-SSI162/2020|EPI_ISL_429377|20	....	....	....	....	....	....
A	3829	hCoV-19/Denmark/ALAB-SSI163/2020|EPI_ISL_429378|20	....	....	....	....	....	....
A	3830	hCoV-19/Denmark/ALAB-SSI164/2020|EPI_ISL_429379|20	....	....	....	....	....	....
A	3831	hCoV-19/Denmark/ALAB-SSI167/2020|EPI_ISL_429381|20	....	....	....	....	....	....
A	3832	hCoV-19/Denmark/ALAB-SSI168/2020|EPI_ISL_429382|20	....	....	....	....	....	....
A	3833	hCoV-19/Denmark/ALAB-SSI169/2020|EPI_ISL_429383|20	....	....	....	....	....	....
A	3834	hCoV-19/Denmark/ALAB-SSI171/2020|EPI_ISL_429384|20	....	....	....	....	....	....
A	3835	hCoV-19/Denmark/ALAB-SSI172/2020|EPI_ISL_429385|20	....	....	....	....	....	....
A	3836	hCoV-19/Denmark/ALAB-SSI173/2020|EPI_ISL_429386|20	....	....	....	....	....	....
A	3837	hCoV-19/Denmark/ALAB-SSI174/2020|EPI_ISL_429387|20	....	....	....	....	....	....
A	3838	hCoV-19/Denmark/ALAB-SSI194/2020|EPI_ISL_429395|20	....	....	....	....	....	....
A	3839	hCoV-19/Denmark/ALAB-SSI195/2020|EPI_ISL_429396|20	....	....	....	....	....	....
A	3840	hCoV-19/Denmark/ALAB-SSI201/2020|EPI_ISL_429399|20	....	....	....	....	....	....
A	3841	hCoV-19/Wales/PHW35/2020|EPI_ISL_416024|2020-03-11	....	....	....	....	....	....
A	3842	hCoV-19/Wales/PHW21/2020|EPI_ISL_416026|2020-03-11	....	....	....	....	....	....
A	3843	hCoV-19/Brazil/SP-07/2020|EPI_ISL_416028|2020-03-0	....	....	....	....	....	....
A	3844	hCoV-19/Brazil/SP-08/2020|EPI_ISL_416029|2020-03-0	....	....	....	....	....	....
A	3845	hCoV-19/Brazil/SP-09/2020|EPI_ISL_416031|2020-03-0	....	....	....	....	....	....
A	3846	hCoV-19/Brazil/SP-10/2020|EPI_ISL_416032|2020-03-0	....	....	....	....	....	....
A	3847	hCoV-19/Brazil/SP-11/2020|EPI_ISL_416033|2020-03-0	....	....	....	....	....	....
A	3848	hCoV-19/Brazil/SP-12/2020|EPI_ISL_416034|2020-03-0	....	....	....	....	....	....
A	3849	hCoV-19/Brazil/SP-13/2020|EPI_ISL_416035|2020-03-0	....	....	....	....	....	....
A	3850	hCoV-19/Brazil/SP-14/2020|EPI_ISL_416036|2020-03-0	....	....	....	....	....	....
A	3851	hCoV-19/USA/CA-CDPH-UC26/2020|EPI_ISL_417330|2020-	....	....	....	....	....	....
A	3853	hCoV-19/France/ARA-06056/2020|EPI_ISL_417334|2020-	....	....	....	....	....	....
A	3854	hCoV-19/France/ARA-06573/2020|EPI_ISL_417335|2020-	....	....	....	....	....	....
A	3855	hCoV-19/France/ARA-0668/2020|EPI_ISL_417336|2020-0	....	....	....	....	....	....
A	3856	hCoV-19/France/ARA-06625/2020|EPI_ISL_417337|2020-	....	....	....	....	....	....
A	3857	hCoV-19/France/ARA-06756/2020|EPI_ISL_417338|2020-	....	....	....	....	....	....
A	3858	hCoV-19/France/ARA-06820/2020|EPI_ISL_417339|2020-	....	....	....	....	....	....
A	3859	hCoV-19/USA/WA-UW196/2020|EPI_ISL_417345|2020-03-1	....	....	....	....	....	....
A	3860	hCoV-19/USA/WA-UW197/2020|EPI_ISL_417346|2020-03-1	....	....	....	....	....	....
A	3861	hCoV-19/USA/WA-UW198/2020|EPI_ISL_417347|2020-03-1	....	....	....	....	....	....
A	3862	hCoV-19/USA/WA-UW199/2020|EPI_ISL_417348|2020-03-1	....	....	....	....	....	....
A	3863	hCoV-19/USA/WA-UW200/2020|EPI_ISL_417349|2020-03-1	....	....	....	....	....	....
A	3864	hCoV-19/USA/WA-UW201/2020|EPI_ISL_417350|2020-03-1	....	....	....	....	....	....
A	3865	hCoV-19/USA/WA-UW202/2020|EPI_ISL_417351|2020-03-1	....	....	....	....	....	....
A	3866	hCoV-19/USA/WA-UW203/2020|EPI_ISL_417352|2020-03-1	....	....	....	....	....	....
A	3867	hCoV-19/USA/WA-UW204/2020|EPI_ISL_417353|2020-03-1	....	....	....	....	....	....
A	3868	hCoV-19/USA/WA-UW205/2020|EPI_ISL_417354|2020-03-1	....	....	....	....	....	....
A	3869	hCoV-19/USA/WA-UW206/2020|EPI_ISL_417355|2020-03-1	....	....	....	....	....	....
A	3870	hCoV-19/USA/WA-UW207/2020|EPI_ISL_417356|2020-03-1	....	....	....	....	....	....
A	3871	hCoV-19/USA/WA-UW209/2020|EPI_ISL_417358|2020-03-1	....	....	....	....	....	....
A	3872	hCoV-19/USA/WA-UW210/2020|EPI_ISL_417359|2020-03-1	....	....	....	....	....	....
A	3873	hCoV-19/USA/WA-UW211/2020|EPI_ISL_417360|2020-03-1	....	....	....	....	....	....
A	3874	hCoV-19/USA/WA-UW212/2020|EPI_ISL_417361|2020-03-1	....	....	....	....	....	....
A	3875	hCoV-19/USA/CT-UW213/2020|EPI_ISL_417362|2020-03-1	....	....	....	....	....	....
A	3876	hCoV-19/USA/WA-UW214/2020|EPI_ISL_417363|2020-03-1	....	....	....	....	....	....
A	3877	hCoV-19/USA/WA-UW215/2020|EPI_ISL_417364|2020-03-1	....	....	....	....	....	....
A	3878	hCoV-19/USA/WA-UW216/2020|EPI_ISL_417365|2020-03-1	....	....	....	....	....	....
A	3879	hCoV-19/USA/WA-UW217/2020|EPI_ISL_417366|2020-03-1	....	....	....	....	....	....
A	3880	hCoV-19/USA/WA-UW218/2020|EPI_ISL_417367|2020-03-1	....	....	....	....	....	....
A	3881	hCoV-19/USA/UN-UW219/2020|EPI_ISL_417368|2020-03-1	....	....	....	....	....	....
A	3882	hCoV-19/USA/WA-UW220/2020|EPI_ISL_417369|2020-03-1	....	....	....	....	....	....
A	3883	hCoV-19/Belgium/ULG-9732/2020|EPI_ISL_418660|2020-	....	....	....	....	....	....
A	3884	hCoV-19/Belgium/ULG-9735/2020|EPI_ISL_418661|2020-	....	....	....	....	....	....
A	3885	hCoV-19/Belgium/ULG-9739/2020|EPI_ISL_418663|2020-	....	....	....	....	....	....
A	3886	hCoV-19/Belgium/ULG-9741/2020|EPI_ISL_418664|2020-	....	....	....	....	....	....
A	3887	hCoV-19/England/20109047803/2020|EPI_ISL_418667|20	....	....	....	....	....	....
A	3888	hCoV-19/England/20110136302/2020|EPI_ISL_418668|20	....	....	....	....	....	....
A	3889	hCoV-19/England/20116022902/2020|EPI_ISL_418669|20	....	....	....	....	....	....
A	3890	hCoV-19/England/20120002102/2020|EPI_ISL_418672|20	....	....	....	....	....	....
A	3891	hCoV-19/England/20122074602/2020|EPI_ISL_418676|20	....	....	....	....	....	....
A	3892	hCoV-19/England/20122087702/2020|EPI_ISL_418677|20	....	....	....	....	....	....
A	3893	hCoV-19/England/20122088102/2020|EPI_ISL_418678|20	....	....	....	....	....	....
A	3894	hCoV-19/England/20122119202/2020|EPI_ISL_418679|20	....	....	....	....	....	....
A	3895	hCoV-19/England/20122119302/2020|EPI_ISL_418680|20	....	....	....	....	....	....
A	3896	hCoV-19/England/20122119502/2020|EPI_ISL_418681|20	....	....	....	....	....	....
A	3900	hCoV-19/England/20124002002/2020|EPI_ISL_418687|20	....	....	....	....	....	....
A	3902	hCoV-19/England/20124003702/2020|EPI_ISL_418690|20	....	....	....	....	....	....
A	3903	hCoV-19/England/20124003902/2020|EPI_ISL_418691|20	....	....	....	....	....	....
A	3904	hCoV-19/England/20124004302/2020|EPI_ISL_418692|20	....	....	....	....	....	....
A	3906	hCoV-19/England/20124009602/2020|EPI_ISL_418696|20	....	....	....	....	....	....
A	3908	hCoV-19/England/20124012702/2020|EPI_ISL_418698|20	....	....	....	....	....	....
A	3909	hCoV-19/Australia/VIC205/2020|EPI_ISL_419900|2020-	....	....	....	....	....	....
A	3910	hCoV-19/Australia/VIC206/2020|EPI_ISL_419901|2020-	....	....	....	....	....	....
A	3911	hCoV-19/Australia/VIC207/2020|EPI_ISL_419902|2020-	....	....	....	....	....	....
A	3912	hCoV-19/Australia/VIC208/2020|EPI_ISL_419903|2020-	....	....	....	....	....	....

A	3913	hCoV-19/Australia/VIC209/2020|EPI_ISL_419904|2020-	····················	····················	····················	····················	····················	····················
A	3914	hCoV-19/Australia/VIC210/2020|EPI_ISL_419905|2020-	····················	·A··················	····················	····················	····················	····················
A	3915	hCoV-19/Australia/VIC211/2020|EPI_ISL_419906|2020-	····················	····················	····················	····················	····················	····················
A	3917	hCoV-19/Australia/VIC213/2020|EPI_ISL_419908|2020-	····················	····················	····················	····················	····················	····················
A	3918	hCoV-19/Australia/VIC214/2020|EPI_ISL_419909|2020-	····················	····················	····················	····················	····················	····················
A	3919	hCoV-19/Australia/VIC215/2020|EPI_ISL_419910|2020-	····················	····················	····················	····················	····················	····················
A	3920	hCoV-19/Australia/VIC216/2020|EPI_ISL_419911|2020-	····················	····················	····················	····················	····················	····················
A	3921	hCoV-19/Australia/VIC217/2020|EPI_ISL_419912|2020-	····················	····················	····················	····················	······A·············	····················
A	3922	hCoV-19/Australia/VIC218/2020|EPI_ISL_419913|2020-	····················	····················	····················	····················	····················	····················
A	3923	hCoV-19/Australia/VIC219/2020|EPI_ISL_419914|2020-	····················	····················	····················	····················	····················	····················
A	3924	hCoV-19/Australia/VIC220/2020|EPI_ISL_419915|2020-	····················	····················	····················	····················	····················	····················
A	3925	hCoV-19/Australia/VIC221/2020|EPI_ISL_419916|2020-	····················	····················	····················	····················	····················	····················
A	3926	hCoV-19/Australia/VIC222/2020|EPI_ISL_419917|2020-	····················	····················	····················	····················	····················	····················
A	3927	hCoV-19/Australia/VIC223/2020|EPI_ISL_419918|2020-	····················	····················	····················	····················	····················	····················
A	3928	hCoV-19/Australia/VIC224/2020|EPI_ISL_419919|2020-	····················	····················	····················	····················	······A·············	····················
A	3929	hCoV-19/Australia/VIC225/2020|EPI_ISL_419920|2020-	····················	····················	····················	····················	····················	····················
A	3930	hCoV-19/Australia/VIC226/2020|EPI_ISL_419921|2020-	····················	····················	····················	····················	····················	····················
A	3931	hCoV-19/Australia/VIC227/2020|EPI_ISL_419922|2020-	····················	····················	····················	····················	····················	····················
A	3932	hCoV-19/Australia/VIC228/2020|EPI_ISL_419923|2020-	····················	····················	····················	····················	····················	····················
A	3933	hCoV-19/Australia/VIC229/2020|EPI_ISL_419924|2020-	····················	····················	····················	····················	····················	····················
A	3934	hCoV-19/Australia/VIC230/2020|EPI_ISL_419925|2020-	····················	····················	····················	····················	····················	····················
A	3935	hCoV-19/Australia/VIC231/2020|EPI_ISL_419926|2020-	····················	····················	····················	····················	····················	····················
A	3936	hCoV-19/Australia/VIC236/2020|EPI_ISL_419931|2020-	····················	····················	····················	····················	····················	····················
A	3937	hCoV-19/Australia/VIC237/2020|EPI_ISL_419932|2020-	····················	····················	····················	····················	····················	····················
A	3938	hCoV-19/Australia/VIC238/2020|EPI_ISL_419933|2020-	····················	····················	····················	····················	····················	····················
A	3939	hCoV-19/Australia/VIC242/2020|EPI_ISL_419937|2020-	····················	····················	····················	····················	····················	····················
A	3940	hCoV-19/Australia/VIC243/2020|EPI_ISL_419938|2020-	····················	····················	····················	····················	····················	····················
A	3941	hCoV-19/Australia/VIC244/2020|EPI_ISL_419939|2020-	····················	····················	····················	····················	····················	····················
A	3942	hCoV-19/Australia/VIC245/2020|EPI_ISL_419940|2020-	····················	····················	····················	····················	····················	····················
A	3943	hCoV-19/Australia/VIC246/2020|EPI_ISL_419941|2020-	····················	····················	····················	····················	····················	····················
A	3944	hCoV-19/Australia/VIC247/2020|EPI_ISL_419942|2020-	····················	····················	····················	····················	····················	····················
A	3945	hCoV-19/Australia/VIC248/2020|EPI_ISL_419943|2020-	····················	····················	····················	····················	····················	····················
A	3946	hCoV-19/Australia/VIC249/2020|EPI_ISL_419944|2020-	····················	····················	····················	····················	····················	····················
A	3947	hCoV-19/Australia/VIC250/2020|EPI_ISL_419945|2020-	····················	····················	····················	····················	····················	····················
A	3948	hCoV-19/Australia/VIC251/2020|EPI_ISL_419946|2020-	····················	····················	····················	····················	····················	····················
A	3949	hCoV-19/Australia/VIC252/2020|EPI_ISL_419947|2020-	····················	····················	····················	····················	····················	····················
A	3950	hCoV-19/Australia/VIC253/2020|EPI_ISL_419948|2020-	····················	····················	····················	····················	····················	····················
A	3951	hCoV-19/Australia/VIC299/2020|EPI_ISL_419990|2020-	····················	····················	····················	····················	····················	····················
A	3952	hCoV-19/Australia/VIC300/2020|EPI_ISL_419991|2020-	····················	····················	····················	····················	····················	····················
A	3953	hCoV-19/Australia/VIC301/2020|EPI_ISL_419992|2020-	····················	····················	····················	····················	····················	····················
A	3954	hCoV-19/Australia/VIC302/2020|EPI_ISL_419993|2020-	····················	····················	····················	····················	····················	····················
A	3955	hCoV-19/Australia/VIC303/2020|EPI_ISL_419994|2020-	····················	····················	····················	····················	····················	····················
A	3956	hCoV-19/Australia/VIC304/2020|EPI_ISL_419995|2020-	····················	····················	····················	····················	····················	····················
A	3957	hCoV-19/Australia/VIC305/2020|EPI_ISL_419996|2020-	····················	····················	····················	····················	····················	····················
A	3958	hCoV-19/Australia/VIC306/2020|EPI_ISL_419997|2020-	····················	····················	····················	····················	····················	····················
A	3959	hCoV-19/Australia/VIC307/2020|EPI_ISL_419998|2020-	····················	····················	····················	····················	····················	····················
A	3960	hCoV-19/Australia/VIC308/2020|EPI_ISL_419999|2020-	····················	····················	····················	····················	····················	····················
A	3962	hCoV-19/Germany/BY-MVP-V2010840/2020|EPI_ISL_42090	····················	····················	····················	····················	····················	····················
A	3963	hCoV-19/Germany/BY-MVP-V2011376/2020|EPI_ISL_42090	····················	····················	····················	····················	····················	····················
A	3964	hCoV-19/Germany/BY-MVP-V2011395/2020|EPI_ISL_42090	····················	····················	····················	····················	····················	····················
A	3966	hCoV-19/Germany/BY-MVP-V2011673/2020|EPI_ISL_42090	····················	····················	····················	····················	····················	····················
A	3967	hCoV-19/Germany/BY-MVP-V2011675/2020|EPI_ISL_42090	····················	····················	····················	····················	····················	····················
A	3968	hCoV-19/Wales/PHWC-25005/2020|EPI_ISL_420929|2020-	····················	····················	····················	····················	····················	····················
A	3969	hCoV-19/Wales/PHWC-2516C/2020|EPI_ISL_420974|2020-	····················	····················	····················	····················	····················	····················
A	3970	hCoV-19/Wales/PHWC-251B7/2020|EPI_ISL_420976|2020-	····················	····················	····················	····················	····················	····················
A	3971	hCoV-19/Wales/PHWC-2523C/2020|EPI_ISL_420977|2020-	····················	····················	····················	····················	····················	····················
A	3972	hCoV-19/Wales/PHWC-253B1/2020|EPI_ISL_420978|2020-	····················	····················	····················	····················	····················	····················
A	3973	hCoV-19/Wales/PHWC-251D5/2020|EPI_ISL_420982|2020-	····················	····················	····················	····················	····················	····················
A	3974	hCoV-19/Wales/PHWC-252B4/2020|EPI_ISL_420985|2020-	····················	····················	····················	····················	····················	····················
A	3975	hCoV-19/Wales/PHWC-253DF/2020|EPI_ISL_420988|2020-	····················	····A···············	····················	····················	····················	····················
A	3976	hCoV-19/Wales/PHWC-25463/2020|EPI_ISL_420994|2020-	····················	····A···············	····················	····················	····················	····················
A	3977	hCoV-19/Wales/PHWC-25472/2020|EPI_ISL_420995|2020-	····················	····A···············	····················	····················	····················	····················
A	3978	hCoV-19/Wales/PHWC-254AF/2020|EPI_ISL_420998|2020-	····················	····················	····················	····················	····················	····················
A	3979	hCoV-19/England/20134025204/2020|EPI_ISL_423129|20	····················	····················	····················	····················	····················	····················
A	3981	hCoV-19/England/20134025704/2020|EPI_ISL_423131|20	····················	····················	····················	····················	····················	····················
A	3982	hCoV-19/England/20134025904/2020|EPI_ISL_423132|20	····················	····················	····················	····················	····················	····················
A	3984	hCoV-19/England/20134027504/2020|EPI_ISL_423136|20	····················	····················	····················	····················	····················	····················
A	3985	hCoV-19/England/20134027604/2020|EPI_ISL_423137|20	····················	····················	····················	····················	····················	····················
A	3986	hCoV-19/England/20134027904/2020|EPI_ISL_423138|20	····················	····················	····················	····················	····················	····················
A	3990	hCoV-19/England/20134060504/2020|EPI_ISL_423147|20	····················	····················	····················	····················	····················	····················
A	3992	hCoV-19/England/20134060904/2020|EPI_ISL_423149|20	····················	····················	····················	····················	·N··················	····················
A	3993	hCoV-19/England/20134064004/2020|EPI_ISL_423150|20	····················	····················	····················	····················	····················	····················
A	3994	hCoV-19/England/20134064204/2020|EPI_ISL_423151|20	····················	····················	····················	····················	····················	····················
A	3995	hCoV-19/England/20134064904/2020|EPI_ISL_423152|20	····················	····················	····················	····················	····················	····················
A	3996	hCoV-19/England/20134067304/2020|EPI_ISL_423154|20	····················	····················	····················	····················	····················	····················
A	3998	hCoV-19/England/20134068504/2020|EPI_ISL_423157|20	····················	····················	····················	····················	····················	····················
A	3999	hCoV-19/England/20134068604/2020|EPI_ISL_423158|20	····················	····················	····················	····················	····················	····················
A	4001	hCoV-19/England/20134068904/2020|EPI_ISL_423160|20	····················	···G················	····················	····················	····················	····················

A	4003 hCoV-19/England/20134071804/2020|EPI_ISL_423163|20	····················	····················	····················	····················	····················	····················	
A	4006 hCoV-19/England/20134077504/2020|EPI_ISL_423170|20	····················	····················	····················	····················	····················	····················	
A	4007 hCoV-19/England/20134078004/2020|EPI_ISL_423172|20	····················	····················	····················	····················	····················	····················	
A	4009 hCoV-19/England/20134078404/2020|EPI_ISL_423174|20	····················	····················	····················	····················	····················	····················	
A	4011 hCoV-19/England/20134078804/2020|EPI_ISL_423176|20	····················	····················	····················	····················	····················	····················	
A	4013 hCoV-19/England/20134079504/2020|EPI_ISL_423178|20	····················	····················	····················	····················	····················	····················	
A	4014 hCoV-19/England/20134081904/2020|EPI_ISL_423181|20	··T·················	····················	····················	····················	····················	····················	
A	4015 hCoV-19/England/20134082304/2020|EPI_ISL_423185|20	····················	····················	····················	····················	····················	·N·N················	
A	4017 hCoV-19/England/201360001/2020|EPI_ISL_423194|2020	····················	····················	····················	····················	····················	····················	
A	4018 hCoV-19/England/201360003/2020|EPI_ISL_423195|2020	····················	····················	····················	····················	····················	····················	
A	4019 hCoV-19/England/201360015/2020|EPI_ISL_423196|2020	····················	····················	····················	····················	····················	·NN·················	
A	4020 hCoV-19/England/201360018/2020|EPI_ISL_423198|2020	····················	····················	····················	····················	····················	····················	
A	4022 hCoV-19/Iceland/435/2020|EPI_ISL_424459|2020-03-20	····················	····················	····················	····················	····················	····················	
A	4023 hCoV-19/Iceland/436/2020|EPI_ISL_424460|2020-03-20	····················	····················	····················	····················	····················	····················	
A	4024 hCoV-19/Iceland/437/2020|EPI_ISL_424461|2020-03-20	····················	····················	····················	····················	····················	····················	
A	4025 hCoV-19/Iceland/438/2020|EPI_ISL_424462|2020-03-20	····················	····················	····················	····················	····················	····················	
A	4026 hCoV-19/Iceland/439/2020|EPI_ISL_424463|2020-03-20	····················	····················	····················	····················	····················	····················	
A	4027 hCoV-19/Iceland/441/2020|EPI_ISL_424465|2020-03-20	····················	····················	····················	····················	····················	····················	
A	4028 hCoV-19/Iceland/442/2020|EPI_ISL_424466|2020-03-20	····················	····················	····················	····················	····················	····················	
A	4029 hCoV-19/Iceland/443/2020|EPI_ISL_424467|2020-03-20	····················	····················	····················	····················	····················	····················	
A	4030 hCoV-19/Iceland/444/2020|EPI_ISL_424468|2020-03-20	····················	····················	····················	····················	····················	····················	
A	4031 hCoV-19/Iceland/445/2020|EPI_ISL_424469|2020-03-20	····················	····················	····················	····················	····················	····················	
A	4032 hCoV-19/Iceland/446/2020|EPI_ISL_424470|2020-03-20	····················	····················	····················	····················	····················	····················	
A	4033 hCoV-19/Iceland/447/2020|EPI_ISL_424471|2020-03-20	····················	····················	····················	····················	····················	····················	
A	4034 hCoV-19/Iceland/448/2020|EPI_ISL_424472|2020-03-20	····················	····················	····················	····················	····················	····················	
A	4035 hCoV-19/Iceland/449/2020|EPI_ISL_424473|2020-03-20	····················	····················	····················	····················	····················	····················	
A	4036 hCoV-19/Iceland/450/2020|EPI_ISL_424474|2020-03-20	····················	····················	····················	····················	····················	····················	
A	4037 hCoV-19/Iceland/451/2020|EPI_ISL_424475|2020-03-20	····················	····················	····················	····················	····················	····················	
A	4038 hCoV-19/Iceland/452/2020|EPI_ISL_424476|2020-03-19	····················	····················	····················	····················	····················	····················	
A	4039 hCoV-19/Iceland/454/2020|EPI_ISL_424477|2020-03-20	····················	····················	····················	····················	····················	····················	
A	4040 hCoV-19/Iceland/455/2020|EPI_ISL_424478|2020-03-20	····················	····················	····················	····················	····················	····················	
A	4041 hCoV-19/Iceland/456/2020|EPI_ISL_424479|2020-03-20	····················	····················	····················	····················	····················	····················	
A	4042 hCoV-19/Iceland/457/2020|EPI_ISL_424480|2020-03-20	····················	····················	····················	····················	····················	····················	
A	4043 hCoV-19/Iceland/459/2020|EPI_ISL_424482|2020-03-20	····················	····················	····················	····················	····················	····················	
A	4044 hCoV-19/Iceland/460/2020|EPI_ISL_424483|2020-03-20	····················	····················	····················	····················	····················	····················	
A	4045 hCoV-19/Iceland/461/2020|EPI_ISL_424484|2020-03-20	····················	····················	····················	····················	····················	····················	
A	4046 hCoV-19/Iceland/462/2020|EPI_ISL_424485|2020-03-20	····················	····················	····················	····················	····················	····················	
A	4047 hCoV-19/Iceland/463/2020|EPI_ISL_424486|2020-03-20	····················	····················	····················	····················	····················	····················	
A	4048 hCoV-19/Iceland/464/2020|EPI_ISL_424487|2020-03-20	····················	····················	····················	····················	····················	····················	
A	4049 hCoV-19/Iceland/466/2020|EPI_ISL_424489|2020-03-20	····················	····················	····················	····················	····················	····················	
A	4050 hCoV-19/Iceland/467/2020|EPI_ISL_424490|2020-03-20	····················	····················	····················	····················	····················	····················	
A	4051 hCoV-19/Iceland/468/2020|EPI_ISL_424491|2020-03-20	····················	····················	····················	····················	····················	····················	
A	4052 hCoV-19/Iceland/469/2020|EPI_ISL_424492|2020-03-21	····················	····················	····················	····················	····················	····················	
A	4053 hCoV-19/Iceland/470/2020|EPI_ISL_424493|2020-03-21	····················	····················	····················	····················	····················	····················	
A	4054 hCoV-19/Iceland/471/2020|EPI_ISL_424494|2020-03-20	····················	····················	····················	····················	····················	····················	
A	4055 hCoV-19/Iceland/472/2020|EPI_ISL_424495|2020-03-20	····················	····················	····················	····················	····················	····················	
A	4056 hCoV-19/Iceland/473/2020|EPI_ISL_424496|2020-03-20	····················	····················	····················	····················	····················	····················	
A	4057 hCoV-19/Iceland/474/2020|EPI_ISL_424497|2020-03-20	····················	····················	····················	····················	····················	····················	
A	4058 hCoV-19/Iceland/475/2020|EPI_ISL_424498|2020-03-20	····················	····················	····················	····················	····················	····················	
A	4059 hCoV-19/Iceland/476/2020|EPI_ISL_424499|2020-03-20	····················	····················	····················	····················	····················	····················	
A	4060 hCoV-19/Scotland/CVR62/2020|EPI_ISL_425790|2020-03	····················	····················	····················	····················	····················	····················	
A	4061 hCoV-19/Scotland/CVR63/2020|EPI_ISL_425791|2020-03	····················	····················	····················	····················	····················	····················	
A	4062 hCoV-19/Scotland/CVR632/2020|EPI_ISL_425792|2020-0	····················	·A··················	····················	····················	····················	····················	
A	4063 hCoV-19/Scotland/CVR634/2020|EPI_ISL_425793|2020-0	····················	····················	····················	····················	····················	····················	
A	4064 hCoV-19/Scotland/CVR635/2020|EPI_ISL_425794|2020-0	····················	···················A	····················	····················	····················	····················	1
A	4065 hCoV-19/Scotland/CVR64/2020|EPI_ISL_425795|2020-03	····················	····················	····················	····················	····················	····················	
A	4066 hCoV-19/Scotland/CVR65/2020|EPI_ISL_425796|2020-03	····················	····················	····················	····················	····················	····················	
A	4067 hCoV-19/Scotland/CVR66/2020|EPI_ISL_425797|2020-03	····················	····················	····················	····················	····················	····················	
A	4068 hCoV-19/Scotland/CVR67/2020|EPI_ISL_425798|2020-03	····················	····················	····················	····················	····················	····················	
A	4069 hCoV-19/Scotland/CVR68/2020|EPI_ISL_425799|2020-03	····················	····················	····················	····················	····················	····················	
A	4070 hCoV-19/Denmark/ALAB-HH64/2020|EPI_ISL_429310|2020	····················	····················	····················	····················	····················	····················	
A	4071 hCoV-19/Denmark/ALAB-HH65/2020|EPI_ISL_429311|2020	····················	····················	····················	····················	····················	····················	
A	4072 hCoV-19/Denmark/ALAB-HH66/2020|EPI_ISL_429312|2020	····················	····················	····················	····················	····················	····················	
A	4073 hCoV-19/Denmark/ALAB-HH74/2020|EPI_ISL_429315|2020	····················	····················	····················	····················	····················	····················	
A	4074 hCoV-19/Denmark/ALAB-HH75/2020|EPI_ISL_429316|2020	····················	····················	····················	····················	····················	····················	
A	4075 hCoV-19/Denmark/ALAB-HH77/2020|EPI_ISL_429318|2020	····················	····················	····················	····················	····················	····················	
A	4076 hCoV-19/Denmark/ALAB-HH80/2020|EPI_ISL_429321|2020	····················	····················	····················	····················	····················	····················	
A	4077 hCoV-19/Denmark/ALAB-HH81/2020|EPI_ISL_429322|2020	····················	····················	····················	····················	····················	····················	
A	4078 hCoV-19/Denmark/ALAB-HH82/2020|EPI_ISL_429323|2020	····················	····················	····················	····················	····················	····················	
A	4079 hCoV-19/Denmark/ALAB-HH83/2020|EPI_ISL_429324|2020	····················	····················	····················	····················	····················	····················	
A	4080 hCoV-19/Denmark/ALAB-HH84/2020|EPI_ISL_429325|2020	····················	····················	····················	····················	····················	····················	
A	4081 hCoV-19/Denmark/ALAB-HH87/2020|EPI_ISL_429327|2020	····················	····················	····················	····················	····················	····················	
A	4082 hCoV-19/Denmark/ALAB-HH88/2020|EPI_ISL_429328|2020	····················	····················	····················	····················	····················	····················	
A	4083 hCoV-19/Denmark/ALAB-HH89/2020|EPI_ISL_429329|2020	····················	····················	····················	····················	····················	····················	
A	4084 hCoV-19/Denmark/ALAB-HH92/2020|EPI_ISL_429331|2020	····················	····················	····················	····················	····················	····················	
A	4085 hCoV-19/Denmark/ALAB-SSI103/2020|EPI_ISL_429335|20	····················	····················	····················	····················	····················	····················	
A	4086 hCoV-19/Denmark/ALAB-SSI105/2020|EPI_ISL_429337|20	····················	····················	····················	····················	····················	····················	
A	4087 hCoV-19/Denmark/ALAB-SSI107/2020|EPI_ISL_429339|20	····················	····················	····················	····················	····················	····················	

A	4088	hCoV-19/Denmark/ALAB-SSI108/2020|EPI_ISL_429340|20	····················	····················	····················	····················	····················	····················
A	4089	hCoV-19/Denmark/ALAB-SSI109/2020|EPI_ISL_429341|20	····················	····················	····················	····················	····················	····················
A	4090	hCoV-19/Denmark/ALAB-SSI110/2020|EPI_ISL_429342|20	····················	····················	····················	····················	····················	····················
A	4091	hCoV-19/Denmark/ALAB-SSI111/2020|EPI_ISL_429343|20	····················	····················	····················	····················	····················	····················
A	4092	hCoV-19/Denmark/ALAB-SSI113/2020|EPI_ISL_429344|20	····················	····················	····················	····················	····················	····················
A	4093	hCoV-19/Denmark/ALAB-SSI114/2020|EPI_ISL_429345|20	····················	····················	····················	····················	····················	····················
A	4094	hCoV-19/Denmark/ALAB-SSI117/2020|EPI_ISL_429346|20	····················	····················	····················	····················	····················	····················
A	4095	hCoV-19/Denmark/ALAB-SSI119/2020|EPI_ISL_429348|20	····················	····················	····················	····················	····················	····················
A	4096	hCoV-19/Denmark/ALAB-SSI120/2020|EPI_ISL_429349|20	····················	····················	····················	····················	····················	····················
A	4097	hCoV-19/Jingzhou/HBCDC-HB-01/2020|EPI_ISL_412459|2	····················	····················	····················	····················	····················	····················
A	4098	hCoV-19/Weifang/WF0015/2020|EPI_ISL_413729|2020-02	····················	····················	····················	····················	····················	····················
A	4099	hCoV-19/Weifang/WF0016/2020|EPI_ISL_413746|2020-02	····················	····················	····················	····················	····················	····················
A	4100	hCoV-19/Weifang/WF0018/2020|EPI_ISL_413748|2020-02	····················	····················	····················	····················	····················	····················
A	4101	hCoV-19/Weifang/WF0019/2020|EPI_ISL_413749|2020-02	····················	····················	····················	····················	····················	····················
A	4102	hCoV-19/Weifang/WF0020/2020|EPI_ISL_413750|2020-02	····················	····················	····················	····················	····················	····················
A	4103	hCoV-19/Weifang/WF0021/2020|EPI_ISL_413751|2020-02	····················	····················	····················	····················	····················	····················
A	4104	hCoV-19/Weifang/WF0024/2020|EPI_ISL_413753|2020-02	····················	····················	····················	····················	····················	····················
A	4105	hCoV-19/Weifang/WF0026/2020|EPI_ISL_413761|2020-02	····················	····················	····················	····················	····················	····················
A	4106	hCoV-19/Weifang/WF0028/2020|EPI_ISL_413791|2020-02	····················	····················	····················	····················	····················	····················
A	4107	hCoV-19/USA/CA-CDPH-UC6/2020|EPI_ISL_413924|2020-0	····················	····················	····················	····················	····················	····················
A	4108	hCoV-19/USA/CA-CDPH-UC7/2020|EPI_ISL_413925|2020-0	····················	····················	····················	····················	····················	····················
A	4109	hCoV-19/USA/CA-CDPH-UC9/2020|EPI_ISL_413928|2020-0	····················	····················	····················	····················	····················	····················
A	4110	hCoV-19/Italy/MAR-UnivPM-1/2020|EPI_ISL_417491|202	····················	····················	····················	····················	····················	····················
A	4111	hCoV-19/USA/MN45-MDH45/2020|EPI_ISL_417492|2020-03	····················	····················	····················	····················	····················	····················
A	4112	hCoV-19/USA/MN46-MDH46/2020|EPI_ISL_417493|2020-03	····················	····················	····················	····················	····················	····················
A	4113	hCoV-19/USA/MN47-MDH47/2020|EPI_ISL_417494|2020-03	····················	····················	····················	····················	····················	····················
A	4114	hCoV-19/USA/MN48-MDH48/2020|EPI_ISL_417495|2020-03	····················	····················	····················	····················	····················	····················
A	4115	hCoV-19/USA/MN51-MDH51/2020|EPI_ISL_417496|2020-03	····················	····················	····················	····················	····················	····················
A	4116	hCoV-19/USA/MN52-MDH52/2020|EPI_ISL_417497|2020-03	····················	····················	····················	····················	····················	····················
A	4117	hCoV-19/USA/MN53-MDH53/2020|EPI_ISL_417498|2020-03	····················	····················	····················	····················	····················	····················
A	4118	hCoV-19/USA/MN55-MDH55/2020|EPI_ISL_417499|2020-03	····················	····················	····················	····················	····················	····················
A	4119	hCoV-19/Australia/VIC232/2020|EPI_ISL_419927|2020-	····················	····················	····················	····················	····················	····················
A	4120	hCoV-19/Australia/VIC233/2020|EPI_ISL_419928|2020-	····················	····················	····················	····················	····················	····················
A	4121	hCoV-19/Australia/VIC234/2020|EPI_ISL_419929|2020-	····················	····················	····················	····················	····················	····················
A	4122	hCoV-19/Australia/VIC239/2020|EPI_ISL_419934|2020-	····················	····················	····················	····················	····················	····················
A	4123	hCoV-19/Australia/VIC240/2020|EPI_ISL_419935|2020-	····················	····················	····················	····················	····················	····················
A	4124	hCoV-19/Australia/VIC241/2020|EPI_ISL_419936|2020-	····················	····················	····················	····················	····················	····················
A	4125	hCoV-19/England/20136087804/2020|EPI_ISL_420910|20	····················	····················	····················	····················	····················	····················
A	4126	hCoV-19/Germany/BY-MVP-V2012856/2020|EPI_ISL_42091	····················	····················	····················	····················	····················	····················
A	4127	hCoV-19/England/20134018902/2020|EPI_ISL_423100|20	····················	····················	····················	····················	····················	····················
A	4128	hCoV-19/England/20134019102/2020|EPI_ISL_423101|20	····················	····················	····················	····················	····················	N···················
A	4129	hCoV-19/England/20134019202/2020|EPI_ISL_423102|20	····················	····················	····················	····················	····················	····················
A	4130	hCoV-19/England/20134019304/2020|EPI_ISL_423103|20	····················	····················	····················	····················	····················	····················
A	4131	hCoV-19/England/20134019402/2020|EPI_ISL_423104|20	····················	····················	····················	····················	····················	····················
A	4132	hCoV-19/England/20134019502/2020|EPI_ISL_423105|20	····················	····················	····················	····················	····················	····················
A	4134	hCoV-19/England/20134020004/2020|EPI_ISL_423108|20	····················	····················	····················	····················	····················	···N················
A	4135	hCoV-19/England/20134020104/2020|EPI_ISL_423109|20	····················	····················	····················	····················	····················	····················
A	4136	hCoV-19/England/20134020504/2020|EPI_ISL_423110|20	····················	····················	····················	····················	····················	····················
A	4138	hCoV-19/England/20134021004/2020|EPI_ISL_423112|20	····················	····················	····················	····················	····················	····················
A	4139	hCoV-19/England/20134021104/2020|EPI_ISL_423113|20	····················	····················	····················	····················	····················	····················
A	4140	hCoV-19/England/20134021504/2020|EPI_ISL_423114|20	····················	····················	····················	····················	····················	····················
A	4141	hCoV-19/England/20134021804/2020|EPI_ISL_423117|20	····················	····················	····················	····················	····················	····················
A	4142	hCoV-19/England/20134021904/2020|EPI_ISL_423118|20	····················	····················	····················	····················	····················	····················
A	4143	hCoV-19/England/20134022104/2020|EPI_ISL_423119|20	····················	····················	····················	····················	····················	····················
A	4145	hCoV-19/England/20134023104/2020|EPI_ISL_423122|20	····················	····················	····················	····················	····················	····················
A	4146	hCoV-19/England/20134023204/2020|EPI_ISL_423123|20	····················	····················	····················	····················	····················	····················
A	4148	hCoV-19/England/20134024104/2020|EPI_ISL_423126|20	····················	····················	····················	····················	····················	····················
A	4149	hCoV-19/England/201340860/2020|EPI_ISL_423192|2020	····················	····················	····················	····················	····················	····················
A	4150	hCoV-19/Iceland/376/2020|EPI_ISL_424400|2020-03-19	····················	····················	····················	····················	····················	····················
A	4151	hCoV-19/Iceland/377/2020|EPI_ISL_424401|2020-03-19	····················	····················	····················	····················	····················	····················
A	4152	hCoV-19/Iceland/378/2020|EPI_ISL_424402|2020-03-19	····················	····················	····················	····················	····················	····················
A	4153	hCoV-19/Iceland/379/2020|EPI_ISL_424403|2020-03-18	····················	····················	····················	····················	····················	····················
A	4154	hCoV-19/Iceland/380/2020|EPI_ISL_424404|2020-03-19	····················	····················	····················	····················	····················	····················
A	4155	hCoV-19/Iceland/381/2020|EPI_ISL_424405|2020-03-19	····················	····················	····················	····················	····················	····················
A	4156	hCoV-19/Iceland/382/2020|EPI_ISL_424406|2020-03-19	····················	····················	····················	····················	····················	····················
A	4157	hCoV-19/Iceland/383/2020|EPI_ISL_424407|2020-03-19	····················	····················	····················	····················	····················	····················
A	4158	hCoV-19/Iceland/385/2020|EPI_ISL_424409|2020-03-19	····················	····················	····················	····················	····················	····················
A	4159	hCoV-19/Iceland/386/2020|EPI_ISL_424410|2020-03-19	····················	····················	····················	····················	····················	····················
A	4160	hCoV-19/Iceland/387/2020|EPI_ISL_424411|2020-03-19	····················	····················	····················	····················	····················	····················
A	4161	hCoV-19/Iceland/388/2020|EPI_ISL_424412|2020-03-19	····················	····················	····················	····················	····················	····················
A	4162	hCoV-19/Iceland/389/2020|EPI_ISL_424413|2020-03-19	····················	····················	····················	····················	····················	····················
A	4163	hCoV-19/Iceland/390/2020|EPI_ISL_424414|2020-03-19	····················	····················	····················	····················	····················	····················
A	4164	hCoV-19/Iceland/391/2020|EPI_ISL_424415|2020-03-19	····················	····················	····················	····················	····················	····················
A	4165	hCoV-19/Iceland/392/2020|EPI_ISL_424416|2020-03-19	····················	····················	····················	····················	····················	····················
A	4166	hCoV-19/Iceland/393/2020|EPI_ISL_424417|2020-03-19	····················	····················	····················	····················	····················	····················
A	4167	hCoV-19/Iceland/394/2020|EPI_ISL_424418|2020-03-19	····················	····················	····················	····················	····················	····················
A	4168	hCoV-19/Iceland/395/2020|EPI_ISL_424419|2020-03-19	····················	····················	····················	····················	····················	····················
A	4169	hCoV-19/Iceland/396/2020|EPI_ISL_424420|2020-03-19	····················	····················	····················	····················	····················	····················

```
A 4170 hCoV-19/Iceland/398/2020|EPI_ISL_424422|2020-03-19 ···················· ···················· ····················· ···················· ···················· ····················
A 4171 hCoV-19/Iceland/399/2020|EPI_ISL_424423|2020-03-19 ···················· ···················· ····················· ···················· ···················· ····················
A 4172 hCoV-19/Iceland/400/2020|EPI_ISL_424424|2020-03-19 ···················· ···················· ····················· ···················· ···················· ····················
A 4173 hCoV-19/Iceland/401/2020|EPI_ISL_424425|2020-03-19 ···················· ···················· ····················· ···················· ···················· ····················
A 4174 hCoV-19/Iceland/402/2020|EPI_ISL_424426|2020-03-19 ···················· ···················· ····················· ···················· ···················· ····················
A 4175 hCoV-19/Iceland/403/2020|EPI_ISL_424427|2020-03-19 ···················· ···················· ····················· ···················· ···················· ····················
A 4176 hCoV-19/Iceland/404/2020|EPI_ISL_424428|2020-03-19 ···T················ ···················· ····················· ···················· ···················· ····················
A 4177 hCoV-19/Iceland/405/2020|EPI_ISL_424429|2020-03-19 ···················· ···················· ····················· ···················· ···················· ····················
A 4178 hCoV-19/Iceland/406/2020|EPI_ISL_424430|2020-03-19 ···················· ···················· ····················· ···················· ···················· ····················
A 4179 hCoV-19/Iceland/407/2020|EPI_ISL_424431|2020-03-19 ···················· ···················· ····················· ···················· ···················· ····················
A 4180 hCoV-19/Iceland/408/2020|EPI_ISL_424432|2020-03-19 ···················· ···················· ····················· ···················· ···················· ····················
A 4181 hCoV-19/Iceland/409/2020|EPI_ISL_424433|2020-03-19 ···················· ···················· ····················· ···················· ···················· ····················
A 4182 hCoV-19/Iceland/410/2020|EPI_ISL_424434|2020-03-19 ···················· ···················· ····················· ···················· ···················· ····················
A 4183 hCoV-19/Iceland/411/2020|EPI_ISL_424435|2020-03-19 ···················· ···················· ····················· ···················· ···················· ····················
A 4184 hCoV-19/Iceland/412/2020|EPI_ISL_424436|2020-03-19 ···················· ···················· ····················· ···················· ···················· ····················
A 4185 hCoV-19/Iceland/413/2020|EPI_ISL_424437|2020-03-19 ···················· ···················· ····················· ···················· ···················· ····················
A 4186 hCoV-19/Iceland/414/2020|EPI_ISL_424438|2020-03-19 ···················· ···················· ····················· ···················· ···················· ····················
A 4187 hCoV-19/Iceland/415/2020|EPI_ISL_424439|2020-03-19 ···················· ···················· ····················· ···················· ···················· ····················
A 4188 hCoV-19/Iceland/416/2020|EPI_ISL_424440|2020-03-19 ···················· ···················· ····················· ···················· ···················· ····················
A 4189 hCoV-19/Iceland/417/2020|EPI_ISL_424441|2020-03-19 ···················· ···················· ····················· ···················· ···················· ····················
A 4190 hCoV-19/Iceland/418/2020|EPI_ISL_424442|2020-03-19 ···················· ···················A ····················· ···················· ···················· ···················· 1
A 4191 hCoV-19/Iceland/419/2020|EPI_ISL_424443|2020-03-19 ···················· ···················· ····················· ···················· ···················· ····················
A 4192 hCoV-19/Iceland/420/2020|EPI_ISL_424444|2020-03-19 ···················· ···················· ····················· ···················· ···················· ····················
A 4193 hCoV-19/Iceland/421/2020|EPI_ISL_424445|2020-03-19 ···················· ···················· ····················· ···················· ···················· ····················
A 4194 hCoV-19/Iceland/422/2020|EPI_ISL_424446|2020-03-19 ···················· ···················· ····················· ···················· ···················· ····················
A 4195 hCoV-19/Iceland/423/2020|EPI_ISL_424447|2020-03-19 ···················· ···················· ····················· ···················· ···················· ····················
A 4196 hCoV-19/Iceland/424/2020|EPI_ISL_424448|2020-03-20 ···················· ···················· ····················· ···················· ···················· ····················
A 4197 hCoV-19/Iceland/425/2020|EPI_ISL_424449|2020-03-20 ···················· ···················· ····················· ···················· ···················· ····················
A 4198 hCoV-19/Iceland/426/2020|EPI_ISL_424450|2020-03-20 ···················· ···················· ····················· ···················· ···················· ····················
A 4199 hCoV-19/Iceland/427/2020|EPI_ISL_424451|2020-03-20 ···················· ···················· ····················· ···················· ···················· ····················
A 4200 hCoV-19/Iceland/428/2020|EPI_ISL_424452|2020-03-20 ···················· ···················· ····················· ···················· ···················· ····················
A 4201 hCoV-19/Iceland/429/2020|EPI_ISL_424453|2020-03-20 ···················· ···················· ····················· ···················· ···················· ····················
A 4202 hCoV-19/Iceland/430/2020|EPI_ISL_424454|2020-03-20 ···················· ···················· ····················· ···················· ···················· ····················
A 4203 hCoV-19/Iceland/431/2020|EPI_ISL_424455|2020-03-20 ···················· ···················· ····················· ···················· ···················· ····················
A 4204 hCoV-19/Iceland/432/2020|EPI_ISL_424456|2020-03-20 ···················· ···················· ····················· ···················· ···················· ····················
A 4205 hCoV-19/Iceland/433/2020|EPI_ISL_424457|2020-03-20 ···················· ···················· ····················· ···················· ···················· ····················
A 4206 hCoV-19/Iceland/434/2020|EPI_ISL_424458|2020-03-20 ···················· ···················· ····················· ···················· ···················· ····················
A 4207 hCoV-19/Iceland/581/2020|EPI_ISL_424601|2020-03-27 ···················· ···················· ····················· ···················· ···················· ····················
A 4208 hCoV-19/Iceland/582/2020|EPI_ISL_424602|2020-03-27 ···················· ···················· ····················· ···················· ···················· ····················
A 4209 hCoV-19/Iceland/583/2020|EPI_ISL_424603|2020-03-27 ···················· ···················· ····················· ···················· ···················· ····················
A 4210 hCoV-19/Iceland/584/2020|EPI_ISL_424604|2020-03-27 ···················· ···················· ····················· ···················· ···················· ····················
A 4211 hCoV-19/Iceland/585/2020|EPI_ISL_424605|2020-03-27 ···················· ···················· ····················· ···················· ···················· ····················
A 4212 hCoV-19/Iceland/586/2020|EPI_ISL_424606|2020-03-28 ···················· ···················· ····················· ···················· ···················· ····················
A 4213 hCoV-19/Iceland/588/2020|EPI_ISL_424608|2020-03-29 ···················· ···················· ····················· ···················· ···················· ····················
A 4214 hCoV-19/Iceland/589/2020|EPI_ISL_424609|2020-03-29 ···················· ···················· ····················· ···················· ···················· ····················
A 4215 hCoV-19/Iceland/590/2020|EPI_ISL_424610|2020-03-26 ···················· ···················· ····················· ···················· ···················· ····················
A 4216 hCoV-19/Iceland/592/2020|EPI_ISL_424612|2020-03-27 ···················· ···················· ····················· ···················· ···················· ····················
A 4217 hCoV-19/Scotland/CVR163/2020|EPI_ISL_425702|2020-0 ···················· ···················· ····················· ···················· ···················· ····················
A 4218 hCoV-19/Scotland/CVR164/2020|EPI_ISL_425703|2020-0 ···················· ···················· ····················· ···················· ···················· ····················
A 4219 hCoV-19/Scotland/CVR165/2020|EPI_ISL_425704|2020-0 ···················· ···················· ····················· ···················· ···················· ····················
A 4220 hCoV-19/Scotland/CVR166/2020|EPI_ISL_425705|2020-0 ···················· ···················· ····················· ···················· ···················· ····················
A 4221 hCoV-19/Scotland/CVR168/2020|EPI_ISL_425707|2020-0 ···················· ···················· ····················· ···················· ···················· ····················
A 4222 hCoV-19/Scotland/CVR169/2020|EPI_ISL_425708|2020-0 ···················· ···················· ····················· ···················· ···················· ····················
A 4223 hCoV-19/Scotland/CVR17/2020|EPI_ISL_425709|2020-03 ···················· ···················· ····················· ···················· ···················· ····················
A 4224 hCoV-19/Scotland/CVR171/2020|EPI_ISL_425711|2020-0 ···················· ···················· ····················· ···················· ···················· ····················
A 4225 hCoV-19/Scotland/CVR173/2020|EPI_ISL_425713|2020-0 ···················· ···················· ····················· ···················· ···················· ····················
A 4226 hCoV-19/Scotland/CVR175/2020|EPI_ISL_425715|2020-0 ···················· ···················· ····················· ···················· ···················· ····················
A 4227 hCoV-19/Scotland/CVR176/2020|EPI_ISL_425716|2020-0 ···················· ···················· ····················· ···················· ···················· ····················
A 4228 hCoV-19/Scotland/CVR177/2020|EPI_ISL_425717|2020-0 ···················· ···················· ····················· ···················· ···················· ····················
A 4229 hCoV-19/Scotland/CVR18/2020|EPI_ISL_425720|2020-03 ···················· ···················· ····················· ···················· ···················· ····················
A 4230 hCoV-19/Scotland/CVR180/2020|EPI_ISL_425721|2020-0 ···················· ···················· ····················· ···················· ···················· ····················
A 4231 hCoV-19/Scotland/CVR183/2020|EPI_ISL_425722|2020-0 ···················· ···················· ····················· ···················· ···················· ····················
A 4232 hCoV-19/Scotland/CVR19/2020|EPI_ISL_425724|2020-03 ···················· ···················· ····················· ···················· ···················· ····················
A 4233 hCoV-19/Scotland/CVR190/2020|EPI_ISL_425725|2020-0 ···················· ···················· ····················· ···················· ···················· ····················
A 4234 hCoV-19/Scotland/CVR191/2020|EPI_ISL_425726|2020-0 ···················· ···················· ····················· ···················· ···················· ····················
A 4235 hCoV-19/Scotland/CVR195/2020|EPI_ISL_425727|2020-0 ···················· ···················· ····················· ···················· ···················· ····················
A 4236 hCoV-19/Scotland/CVR196/2020|EPI_ISL_425728|2020-0 ···················· ·A·················· ····················· ···················· ···················· ····················
A 4237 hCoV-19/Scotland/CVR199/2020|EPI_ISL_425729|2020-0 ···················· ···················· ····················· ···················· ···················· ····················
A 4238 hCoV-19/Scotland/CVR20/2020|EPI_ISL_425730|2020-03 ···················· ···················· ····················· ···················· ···················· ····················
A 4239 hCoV-19/Scotland/CVR200/2020|EPI_ISL_425731|2020-0 ···················· ···················· ····················· ···················· ···················· ····················
A 4240 hCoV-19/Scotland/CVR201/2020|EPI_ISL_425732|2020-0 ···················· ···················· ····················· ···················· ···················· ····················
A 4241 hCoV-19/Scotland/CVR202/2020|EPI_ISL_425733|2020-0 ···················· ···················· ····················· ···················· ···················· ····················
A 4242 hCoV-19/Scotland/CVR203/2020|EPI_ISL_425734|2020-0 ···················· ···················· ····················· ···················· ···················· ····················
A 4243 hCoV-19/Scotland/CVR205/2020|EPI_ISL_425735|2020-0 ···················· ···················· ····················· ···················· ···················· ····················
A 4244 hCoV-19/Scotland/CVR206/2020|EPI_ISL_425736|2020-0 ···················· ···················· ····················· ···················· ···················· ····················
A 4245 hCoV-19/Scotland/CVR207/2020|EPI_ISL_425737|2020-0 ···················· ···················· ····················· ···················· ···················· ····················
A 4246 hCoV-19/Scotland/CVR21/2020|EPI_ISL_425739|2020-03 ···················· ···················· ····················· ···················· ···················· ····················
A 4247 hCoV-19/Scotland/CVR211/2020|EPI_ISL_425740|2020-0 ···················· ···················· ····················· ···················· ···················· ····················
```

A	4248 hCoV-19/Scotland/CVR212/2020|EPI_ISL_425741|2020-0	·····················	·····················	·····················	·····················	·····················	·····················	
A	4249 hCoV-19/Scotland/CVR213/2020|EPI_ISL_425742|2020-0	·····················	·····················	·····················	·····················	·····················	·····················	
A	4250 hCoV-19/Scotland/CVR215/2020|EPI_ISL_425743|2020-0	·····················	·····················	·····················	·····················	·····················	·····················	
A	4251 hCoV-19/Scotland/CVR216/2020|EPI_ISL_425744|2020-0	·····················	·····················	·····················	·····················	·····················	·····················	
A	4252 hCoV-19/Scotland/CVR218/2020|EPI_ISL_425745|2020-0	·····················	·A···················	·····················	·····················	·····················	·····················	
A	4253 hCoV-19/Scotland/CVR219/2020|EPI_ISL_425746|2020-0	·····················	·····················	·····················	·····················	·····················	·····················	
A	4254 hCoV-19/Scotland/CVR220/2020|EPI_ISL_425747|2020-0	·····················	·····················	·····················	·····················	·····················	·····················	
A	4255 hCoV-19/Scotland/CVR222/2020|EPI_ISL_425748|2020-0	·····················	·····················	·····················	·····················	·····················	·····················	
A	4256 hCoV-19/Scotland/CVR23/2020|EPI_ISL_425749|2020-03	·····················	·····················	·····················	·····················	·····················	·····················	
A	4257 hCoV-19/Scotland/CVR235/2020|EPI_ISL_425750|2020-0	·····················	·····················	·····················	·····················	·····················	·····················	
A	4258 hCoV-19/Scotland/CVR236/2020|EPI_ISL_425751|2020-0	·····················	·····················	·····················	·····················	·····················	·····················	
A	4259 hCoV-19/Scotland/CVR24/2020|EPI_ISL_425752|2020-03	·····················	·····················	·····················	·····················	·····················	·····················	
A	4260 hCoV-19/Scotland/CVR264/2020|EPI_ISL_425754|2020-0	·····················	·····················	·····················	·····················	·····················	·····················	
A	4261 hCoV-19/Scotland/CVR268/2020|EPI_ISL_425755|2020-0	·····················	·····················	·····················	·····················	·····················	·····················	
A	4262 hCoV-19/Scotland/CVR27/2020|EPI_ISL_425756|2020-03	·····················	·····················	·····················	·····················	·····················	·····················	
A	4263 hCoV-19/Scotland/CVR28/2020|EPI_ISL_425757|2020-03	·····················	·····················	·····················	·····················	·····················	·····················	
A	4264 hCoV-19/Scotland/CVR29/2020|EPI_ISL_425758|2020-03	·····················	·····················	·····················	·····················	·····················	·····················	
A	4265 hCoV-19/Scotland/CVR30/2020|EPI_ISL_425759|2020-03	·····················	·····················	·····················	·····················	·····················	·····················	
A	4266 hCoV-19/Scotland/CVR31/2020|EPI_ISL_425760|2020-03	·····················	·····················	·····················	·····················	·····················	·····················	
A	4267 hCoV-19/Scotland/CVR32/2020|EPI_ISL_425761|2020-03	·····················	·····················	·····················	·····················	·····················	·····················	
A	4268 hCoV-19/Scotland/CVR33/2020|EPI_ISL_425762|2020-03	·····················	·A···················	·····················	·····················	·····················	·····················	
A	4269 hCoV-19/Scotland/CVR34/2020|EPI_ISL_425763|2020-03	·····················	·····················	·····················	·····················	·····················	·····················	
A	4270 hCoV-19/Scotland/CVR35/2020|EPI_ISL_425764|2020-03	·····················	·····················	·····················	·····················	·····················	·····················	
A	4271 hCoV-19/Scotland/CVR36/2020|EPI_ISL_425765|2020-03	·····················	·····················	·····················	·····················	·····················	·····················	
A	4272 hCoV-19/Scotland/CVR37/2020|EPI_ISL_425766|2020-03	·····················	·····················	·····················	·····················	·····················	·····················	
A	4273 hCoV-19/Scotland/CVR38/2020|EPI_ISL_425767|2020-03	·····················	·····················	·····················	·····················	·····················	·····················	
A	4274 hCoV-19/Scotland/CVR40/2020|EPI_ISL_425768|2020-03	·····················	·····················	·····················	·····················	·····················	·····················	
A	4275 hCoV-19/Scotland/CVR41/2020|EPI_ISL_425769|2020-03	·····················	·····················	·····················	·····················	·····················	·····················	
A	4276 hCoV-19/Scotland/CVR42/2020|EPI_ISL_425770|2020-03	·····················	·····················	·····················	·····················	·····················	·····················	
A	4277 hCoV-19/Scotland/CVR44/2020|EPI_ISL_425772|2020-03	·····················	·····················	·····················	·····················	·····················	·····················	
A	4278 hCoV-19/Scotland/CVR45/2020|EPI_ISL_425773|2020-03	·····················	·····················	·····················	·····················	·····················	·····················	
A	4279 hCoV-19/Scotland/CVR46/2020|EPI_ISL_425774|2020-03	·····················	·····················	·····················	·····················	·····················	·····················	
A	4280 hCoV-19/Scotland/CVR462/2020|EPI_ISL_425775|2020-0	·····················	·····················	·····················	·····················	·····················	·····················	
A	4281 hCoV-19/Scotland/CVR47/2020|EPI_ISL_425776|2020-03	·····················	·····················	·····················	·····················	·····················	·····················	
A	4282 hCoV-19/Scotland/CVR49/2020|EPI_ISL_425777|2020-03	·····················	·····················	·····················	·····················	·····················	·····················	
A	4283 hCoV-19/Scotland/CVR50/2020|EPI_ISL_425778|2020-03	·····················	·····················	·····················	·····················	·····················	·····················	
A	4284 hCoV-19/Scotland/CVR51/2020|EPI_ISL_425779|2020-03	·····················	·····················	·····················	·····················	·····················	·····················	
A	4285 hCoV-19/Scotland/CVR52/2020|EPI_ISL_425780|2020-03	·····················	·····················	·····················	·····················	·····················	·····················	
A	4286 hCoV-19/Scotland/CVR53/2020|EPI_ISL_425782|2020-03	·····················	·····················	·····················	·····················	·····················	·····················	
A	4287 hCoV-19/Scotland/CVR54/2020|EPI_ISL_425783|2020-03	·····················	·····················	·····················	·····················	·····················	·····················	
A	4288 hCoV-19/Scotland/CVR55/2020|EPI_ISL_425784|2020-03	·····················	·····················	·····················	·····················	·····················	·····················	
A	4289 hCoV-19/Scotland/CVR56/2020|EPI_ISL_425785|2020-03	·····················	·····················	·····················	·····················	·····················	·····················	
A	4290 hCoV-19/Scotland/CVR562/2020|EPI_ISL_425786|2020-0	·····················	·····················	·····················	·····················	·····················	·····················	
A	4291 hCoV-19/Scotland/CVR57/2020|EPI_ISL_425787|2020-03	·····················	·····················	·····················	·····················	·····················	·····················	
A	4292 hCoV-19/Scotland/CVR58/2020|EPI_ISL_425788|2020-03	·····················	·····················	·····················	·····················	·····················	·····················	
A	4293 hCoV-19/Scotland/CVR60/2020|EPI_ISL_425789|2020-03	·····················	·····················	·····················	·····················	·····················	·····················	
A	4294 hCoV-19/Scotland/EDB113/2020|EPI_ISL_425900|2020-0	·····················	·····················	·····················	·····················	·····················	·····················	
A	4296 hCoV-19/Scotland/EDB118/2020|EPI_ISL_425902|2020-0	·····················	·····················	·····················	·····················	·····················	·····················	
A	4298 hCoV-19/Scotland/EDB120/2020|EPI_ISL_425904|2020-0	·····················	·····················	·····················	·····················	·····················	·····················	
A	4300 hCoV-19/Scotland/EDB124/2020|EPI_ISL_425906|2020-0	·····················	·····················	·····················	·····················	·····················	·····················	
A	4301 hCoV-19/Scotland/EDB125/2020|EPI_ISL_425907|2020-0	·····················	·····················	·····················	·····················	·····················	·····················	
A	4303 hCoV-19/Scotland/EDB132/2020|EPI_ISL_425911|2020-0	·····················	·····················	·····················	·····················	·····················	·····················	
A	4304 hCoV-19/Scotland/EDB142/2020|EPI_ISL_425912|2020-0	·····················	·····················	·····················	·····················	·····················	·····················	
A	4306 hCoV-19/Scotland/EDB146/2020|EPI_ISL_425915|2020-0	·····················	·····················	·····················	·····················	·····················	·····················	
A	4307 hCoV-19/Scotland/EDB148/2020|EPI_ISL_425916|2020-0	·····················	·····················	·····················	·····················	·····················	·····················	
A	4308 hCoV-19/Scotland/EDB149/2020|EPI_ISL_425917|2020-0	·····················	·····················	·····················	·····················	·····················	·····················	
A	4309 hCoV-19/Scotland/EDB150/2020|EPI_ISL_425918|2020-0	·····················	·····················	·····················	·····················	·····················	·····················	
A	4310 hCoV-19/Scotland/EDB151/2020|EPI_ISL_425919|2020-0	·····················	·A···················	·····················	·····················	·····················	·····················	
A	4311 hCoV-19/Scotland/EDB155/2020|EPI_ISL_425920|2020-0	·····················	·····················	·····················	·····················	·····················	·····················	
A	4312 hCoV-19/Scotland/EDB156/2020|EPI_ISL_425921|2020-0	·····················	·····················	·····················	·····················	·······N·N····N······	·······NN············	1
A	4316 hCoV-19/Scotland/EDB188/2020|EPI_ISL_425932|2020-0	·····················	·····················	·····················	·····················	·····················	·····················	
A	4317 hCoV-19/Scotland/EDB189/2020|EPI_ISL_425933|2020-0	·····················	·····················	·····················	·····················	·····················	·····················	
A	4320 hCoV-19/Scotland/EDB193/2020|EPI_ISL_425936|2020-0	·····················	·····················	·····················	·····················	·····················	·······NN············	
A	4323 hCoV-19/Scotland/EDB198/2020|EPI_ISL_425939|2020-0	·····················	·····················	·····················	·····················	·····················	·····················	
A	4327 hCoV-19/Scotland/EDB212/2020|EPI_ISL_425943|2020-0	·····················	·····················	·····················	·····················	·····················	·····················	
A	4328 hCoV-19/Sweden/RV-FOI-4/2020|EPI_ISL_428148|2020-0	·····················	·····················	·····················	·····················	·····················	·····················	
A	4329 hCoV-19/Denmark/ALAB-SSI361/2020|EPI_ISL_429471|20	·····················	·····················	·····················	·····················	·····················	·····················	
A	4330 hCoV-19/Denmark/ALAB-SSI364/2020|EPI_ISL_429472|20	·····················	·····················	·····················	·····················	·····················	·····················	
A	4331 hCoV-19/Denmark/ALAB-SSI365/2020|EPI_ISL_429473|20	·····················	·····················	·····················	·····················	·····················	·····················	
A	4332 hCoV-19/Denmark/ALAB-SSI366/2020|EPI_ISL_429474|20	·····················	·····················	·····················	·····················	·····················	·····················	
A	4333 hCoV-19/Denmark/ALAB-SSI370/2020|EPI_ISL_429476|20	·····················	·····················	·····················	·····················	·····················	·····················	
A	4334 hCoV-19/Denmark/ALAB-SSI371/2020|EPI_ISL_429477|20	·····················	·····················	·····················	·····················	·····················	·····················	
A	4335 hCoV-19/Denmark/ALAB-SSI372/2020|EPI_ISL_429478|20	·····················	·····················	·····················	·····················	·····················	·····················	
A	4336 hCoV-19/Denmark/ALAB-SSI374/2020|EPI_ISL_429479|20	·····················	·A···················	·····················	·····················	·····················	·····················	
A	4337 hCoV-19/Denmark/ALAB-SSI375/2020|EPI_ISL_429480|20	·····················	·····················	·····················	·····················	·····················	·····················	
A	4338 hCoV-19/Denmark/ALAB-SSI376/2020|EPI_ISL_429481|20	·····················	·····················	·····················	·····················	·····················	·····················	
A	4339 hCoV-19/Denmark/ALAB-SSI377/2020|EPI_ISL_429482|20	·····················	·····················	·····················	·····················	·····················	·····················	
A	4340 hCoV-19/Denmark/ALAB-SSI383/2020|EPI_ISL_429483|20	·····················	·····················	·····················	·····················	·····················	·····················	

A	4341	hCoV-19/Denmark/ALAB-SSI384/2020|EPI_ISL_429484|20	....	....	....	....	....	....
A	4342	hCoV-19/Denmark/ALAB-SSI385/2020|EPI_ISL_429485|20	....	....	....	....	....	....
A	4343	hCoV-19/Denmark/ALAB-SSI390/2020|EPI_ISL_429486|20	....	....	....	....	....	....
A	4344	hCoV-19/Denmark/ALAB-SSI393/2020|EPI_ISL_429488|20	....	....	....	....	....	....
A	4345	hCoV-19/Denmark/ALAB-SSI394/2020|EPI_ISL_429489|20	....	....	....	....	....	....
A	4346	hCoV-19/Denmark/ALAB-SSI395/2020|EPI_ISL_429490|20	....	....	....	....	....	....
A	4347	hCoV-19/Denmark/ALAB-SSI399/2020|EPI_ISL_429492|20	....	....	....	....	....	....
A	4348	hCoV-19/Denmark/ALAB-SSI400/2020|EPI_ISL_429493|20	....	....	....	....	....	....
A	4349	hCoV-19/Denmark/ALAB-SSI402/2020|EPI_ISL_429495|20	....	....	....	....	....	....
A	4350	hCoV-19/Denmark/ALAB-SSI403A/2020|EPI_ISL_429496|2	....	....	....	....	....	....
A	4351	hCoV-19/Denmark/ALAB-SSI404/2020|EPI_ISL_429497|20	....	....	....	....	....	....
A	4352	hCoV-19/Denmark/ALAB-SSI405/2020|EPI_ISL_429499|20	....	....	....	....	....	....
A	4353	hCoV-19/Wuhan/WHU01/2020|EPI_ISL_406716|2020-01-02	....	....	....	....	....	....
A	4354	hCoV-19/Wuhan/WHU02/2020|EPI_ISL_406717|2020-01-02	....	....	....	....	....	....
A	4355	hCoV-19/Wuhan/WH01/2019|EPI_ISL_406798|2019-12-26	....	....	....	....	....	....
A	4356	hCoV-19/Guangdong/2020XN4373-P0039/2020|EPI_ISL_41	....	....	....	....	....	....
A	4357	hCoV-19/Guangdong/2020XN4433-P0040/2020|EPI_ISL_41	....	....	....	....	....	....
A	4358	hCoV-19/Guangdong/2020XN4243-P0035/2020|EPI_ISL_41	....	....	....	....	....	....
A	4359	hCoV-19/Guangdong/2020XN4475-P0042/2020|EPI_ISL_41	....	....	....	....	....	....
A	4360	hCoV-19/Guangdong/GD2020234-P0023/2020|EPI_ISL_413	....	....	....	....	....	....
A	4361	hCoV-19/Guangdong/GD2020233-P0027/2020|EPI_ISL_413	....	....	....	....	....	....
A	4362	hCoV-19/Guangdong/2020XN4448-P0002/2020|EPI_ISL_41	....	....	....	....	....	....
A	4363	hCoV-19/Guangdong/2020XN4459-P0041/2020|EPI_ISL_41	....	....	....	....	....	....
A	4364	hCoV-19/Guangdong/2020XN4273-P0036/2020|EPI_ISL_41	....	....	....	....	....	....
A	4365	hCoV-19/Guangdong/GD2020080-P0010/2020|EPI_ISL_413	....	....	....	....	....	....
A	4366	hCoV-19/Guangdong/GD2020086-P0021/2020|EPI_ISL_413	....	....	....	....	....	....
A	4367	hCoV-19/Guangdong/GD2020087-P0008/2020|EPI_ISL_413	....	....	....	....	....	....
A	4368	hCoV-19/Guangdong/GD2020246-P0028/2020|EPI_ISL_413	....	....	....	....	....	....
A	4369	hCoV-19/Denmark/SSI-05/2020|EPI_ISL_416140|2020-03	....	....	....	....	....	....
A	4370	hCoV-19/Denmark/SSI-09/2020|EPI_ISL_416141|2020-03	....	....	....	....	....	....
A	4371	hCoV-19/Denmark/SSI-01/2020|EPI_ISL_416142|2020-02	....	....	....	....	....	....
A	4372	hCoV-19/Denmark/SSI-02/2020|EPI_ISL_416143|2020-02	....	....	....	....	....	....
A	4373	hCoV-19/Denmark/SSI-03/2020|EPI_ISL_416144|2020-03	....	....	....	....	....	....
A	4374	hCoV-19/Denmark/SSI-04/2020|EPI_ISL_416153|2020-03	....	....	....	....	....	....
A	4375	hCoV-19/Australia/NSW44/2020|EPI_ISL_417400|2020-0	....	....	....	....	....	....
A	4376	hCoV-19/Australia/NSW45/2020|EPI_ISL_417401|2020-0	....	....	....	....	....	....
A	4377	hCoV-19/Australia/NSW47/2020|EPI_ISL_417402|2020-0	....	....	....	....	....	....
A	4378	hCoV-19/Australia/NSW48/2020|EPI_ISL_417403|2020-0	....	....	....	....	....	....
A	4379	hCoV-19/Australia/NSW50/2020|EPI_ISL_417404|2020-0	....	....	....	....	....	....
A	4380	hCoV-19/Australia/NSW52/2020|EPI_ISL_417405|2020-0	....	....	....	....	....	....
A	4381	hCoV-19/Australia/NSW53/2020|EPI_ISL_417406|2020-0	....	....	....	....	....	....
A	4382	hCoV-19/Australia/NSW54/2020|EPI_ISL_417407|2020-0	....	....	....	....	....	....
A	4383	hCoV-19/Australia/NSW56/2020|EPI_ISL_417408|2020-0	....	....	....	....	....	....
A	4384	hCoV-19/Australia/NSW57/2020|EPI_ISL_417409|2020-0	....	....	....	....	....	....
A	4385	hCoV-19/Australia/NSW58/2020|EPI_ISL_417410|2020-0	....	....	....	....	....	....
A	4386	hCoV-19/Australia/NSW61/2020|EPI_ISL_417411|2020-0	....	....	....	....	....	....
A	4387	hCoV-19/Australia/NSW62/2020|EPI_ISL_417412|2020-0	....	....	....	....	....	....
A	4388	hCoV-19/Italy/FVG-ICGEB-S1/2020|EPI_ISL_417418|202	....	....	....	....	....	....
A	4389	hCoV-19/Italy/FVG-ICGEB-S5/2020|EPI_ISL_417419|202	....	....	....	....	....	....
A	4390	hCoV-19/NanChang/JX216/2020|EPI_ISL_417420|2020-03	....	....	....	....	....	....
A	4391	hCoV-19/Italy/FVG-ICGEB-S8/2020|EPI_ISL_417421|202	....	....	....	....	....	....
A	4392	hCoV-19/Belgium/DHWM-03041/2020|EPI_ISL_417422|202	....	....	....	....	....	....
A	4393	hCoV-19/Belgium/KA-03042/2020|EPI_ISL_417424|2020-	....	....	....	....	....	....
A	4394	hCoV-19/Belgium/VBK-03061/2020|EPI_ISL_417425|2020	....	....	....	....	....	....
A	4395	hCoV-19/Belgium/SQ-03043/2020|EPI_ISL_417426|2020-	....	....	....	....	....	....
A	4396	hCoV-19/Belgium/VI-03027/2020|EPI_ISL_417427|2020-	....	....	....	....	....	....
A	4397	hCoV-19/Belgium/FR-03029/2020|EPI_ISL_417428|2020-	....	....	....	....	....	....
A	4398	hCoV-19/Belgium/MMJ-03034/2020|EPI_ISL_417429|2020	....	....	....	....	....	....
A	4399	hCoV-19/Belgium/RT-03035/2020|EPI_ISL_417430|2020-	....	....	....	....	....	....
A	4401	hCoV-19/DRC/KN-0038/2020|EPI_ISL_417434|2020-03-14	....	....	....	....	....	....
A	4403	hCoV-19/DRC/KN-0051/2020|EPI_ISL_417436|2020-03-17	....	....	....	....	....	....
A	4404	hCoV-19/DRC/KN-0054/2020|EPI_ISL_417437|2020-03-17	....	....	....	....	....	....
A	4405	hCoV-19/DRC/KN-0058/2020|EPI_ISL_417438|2020-03-17	....	....	....	....	....	....
A	4406	hCoV-19/DRC/KN-0059/2020|EPI_ISL_417439|2020-03-17	....	....	....	....	....	....
A	4407	hCoV-19/DRC/KN-0060/2020|EPI_ISL_417440|2020-03-17	....	....	....	....	....	....
A	4408	hCoV-19/DRC/KN-0070/2020|EPI_ISL_417441|2020-03-17	....	....	....	....	....	....
A	4409	hCoV-19/DRC/KN-0072/2020|EPI_ISL_417442|2020-03-18	....	....	....	....	....	....
A	4410	hCoV-19/Hong Kong/XM-PII-S4/2020|EPI_ISL_417443|20	....	....	....	....	....	....
A	4411	hCoV-19/Pakistan/Gilgit1/2020|EPI_ISL_417444|2020-	....	....	....	....	....	....
A	4412	hCoV-19/Italy/LOM-UniMI01/2020|EPI_ISL_417445|2020	....	....	....	....	....	....
A	4413	hCoV-19/Italy/LOM-UniMI03/2020|EPI_ISL_417447|2020	....	....	....	....	....	....
A	4414	hCoV-19/USA/WA-UW234/2020|EPI_ISL_417448|2020-03-1	....	....	....	....	....	....
A	4415	hCoV-19/USA/WA-UW235/2020|EPI_ISL_417449|2020-03-1	....	....	....	....	....	....
A	4416	hCoV-19/USA/WA-UW236/2020|EPI_ISL_417450|2020-03-1	....	....	....	....	....	....
A	4417	hCoV-19/USA/WA-UW237/2020|EPI_ISL_417451|2020-03-1	....	....	....	....	....	....
A	4418	hCoV-19/USA/CT-UW238/2020|EPI_ISL_417452|2020-03-1	....	....	....	....	....	....
A	4419	hCoV-19/USA/WA-UW239/2020|EPI_ISL_417453|2020-03-1	....	....	....	....	....	....
A	4420	hCoV-19/USA/WA-UW240/2020|EPI_ISL_417454|2020-03-1	....	....	....	....	....	....

A 4421 hCoV-19/USA/WA-UW241/2020|EPI_ISL_417455|2020-03-1 ....
A 4422 hCoV-19/USA/WA-UW242/2020|EPI_ISL_417456|2020-03-1 ....
A 4423 hCoV-19/Germany/NW-HHU-12/2020|EPI_ISL_417457|2020 ....
A 4424 hCoV-19/Germany/NW-HHU-13/2020|EPI_ISL_417458|2020 ....
A 4425 hCoV-19/Germany/NW-HHU-14/2020|EPI_ISL_417459|2020 ....
A 4426 hCoV-19/Germany/NW-HHU-15/2020|EPI_ISL_417460|2020 ....
A 4427 hCoV-19/Germany/NW-HHU-16/2020|EPI_ISL_417461|2020 ....
A 4428 hCoV-19/Germany/NW-HHU-17/2020|EPI_ISL_417462|2020 ....
A 4429 hCoV-19/Germany/NW-HHU-18/2020|EPI_ISL_417463|2020 ....
A 4430 hCoV-19/Germany/NW-HHU-19/2020|EPI_ISL_417464|2020 ....
A 4431 hCoV-19/Germany/NW-HHU-20/2020|EPI_ISL_417465|2020 ....
A 4432 hCoV-19/Germany/NW-HHU-21/2020|EPI_ISL_417466|2020 ....
A 4433 hCoV-19/Germany/NW-HHU-22/2020|EPI_ISL_417467|2020 ....
A 4434 hCoV-19/Germany/NW-HHU-23/2020|EPI_ISL_417468|2020 ....
A 4435 hCoV-19/USA/MN31-MDH31/2020|EPI_ISL_417469|2020-03 ....
A 4436 hCoV-19/USA/MN32-MDH32/2020|EPI_ISL_417470|2020-03 ....
A 4437 hCoV-19/USA/MN34-MDH34/2020|EPI_ISL_417471|2020-03 ....
A 4438 hCoV-19/USA/MN35-MDH35/2020|EPI_ISL_417472|2020-03 ....
A 4439 hCoV-19/USA/MN36-MDH36/2020|EPI_ISL_417473|2020-03 ....
A 4440 hCoV-19/USA/MN38-MDH38/2020|EPI_ISL_417474|2020-03 ....
A 4441 hCoV-19/USA/MN39-MDH39/2020|EPI_ISL_417475|2020-03 ....
A 4442 hCoV-19/USA/MN40-MDH40/2020|EPI_ISL_417476|2020-03 ....
A 4443 hCoV-19/USA/MN41-MDH41/2020|EPI_ISL_417477|2020-03 ....
A 4444 hCoV-19/USA/MN42-MDH42/2020|EPI_ISL_417478|2020-03 ....
A 4445 hCoV-19/USA/MN43-MDH43/2020|EPI_ISL_417479|2020-03 ....
A 4446 hCoV-19/USA/MN44-MDH44/2020|EPI_ISL_417480|2020-03 ....
A 4447 hCoV-19/Iceland/LUH-01/2020|EPI_ISL_417481|2020-03 ....
A 4448 hCoV-19/Norway/1379/2020|EPI_ISL_417483|2020-02-27 ....
A 4449 hCoV-19/Norway/1380/2020|EPI_ISL_417484|2020-02-26 ....
A 4450 hCoV-19/Norway/1526/2020|EPI_ISL_417485|2020-03-02 ....
A 4451 hCoV-19/Norway/1538/2020|EPI_ISL_417486|2020-03-01 ....
A 4452 hCoV-19/Norway/1539/2020|EPI_ISL_417487|2020-03-01 ....
A 4453 hCoV-19/Norway/1493/2020|EPI_ISL_417488|2020-02-29 ....
A 4454 hCoV-19/England/20124013202/2020|EPI_ISL_418700|20 ....
A 4455 hCoV-19/England/20124020402/2020|EPI_ISL_418701|20 ....
A 4456 hCoV-19/England/20124020802/2020|EPI_ISL_418702|20 ....
A 4457 hCoV-19/England/20124021402/2020|EPI_ISL_418704|20 ....
A 4458 hCoV-19/England/20124029802/2020|EPI_ISL_418706|20 ....
A 4459 hCoV-19/England/20124030003/2020|EPI_ISL_418707|20 ....
A 4460 hCoV-19/England/20124030802/2020|EPI_ISL_418708|20 ....
A 4461 hCoV-19/England/20124034002/2020|EPI_ISL_418709|20 ....
A 4462 hCoV-19/England/20124034702/2020|EPI_ISL_418711|20 ....
A 4465 hCoV-19/England/20124036902/2020|EPI_ISL_418718|20 ....
A 4468 hCoV-19/England/20124049402/2020|EPI_ISL_418723|20 ....
A 4469 hCoV-19/England/20124095502/2020|EPI_ISL_418729|20 ....
A 4471 hCoV-19/England/20124097102/2020|EPI_ISL_418734|20 ....
A 4474 hCoV-19/England/20124101102/2020|EPI_ISL_418739|20 ....
A 4477 hCoV-19/England/20126007002/2020|EPI_ISL_418750|20 ....
A 4478 hCoV-19/England/20126007102/2020|EPI_ISL_418751|20 ....
A 4479 hCoV-19/England/20126012702/2020|EPI_ISL_418756|20 ....
A 4480 hCoV-19/England/20126035502/2020|EPI_ISL_418764|20 ....
A 4481 hCoV-19/England/20126096602/2020|EPI_ISL_418770|20 ....
A 4482 hCoV-19/USA/WA-NH2/2020|EPI_ISL_418771|2020-03-13 ....
A 4483 hCoV-19/USA/WA-NH3/2020|EPI_ISL_418772|2020-03-13 ....
A 4484 hCoV-19/USA/WA-NH4/2020|EPI_ISL_418773|2020-03-13 ....
A 4485 hCoV-19/USA/WA-NH5/2020|EPI_ISL_418774|2020-03-13 ....
A 4486 hCoV-19/USA/WA-NH6/2020|EPI_ISL_418775|2020-03-13 ....
A 4487 hCoV-19/USA/WA-NH7/2020|EPI_ISL_418776|2020-03-13 ....
A 4488 hCoV-19/USA/WA-NH8/2020|EPI_ISL_418777|2020-03-13 ....
A 4489 hCoV-19/USA/WA-NH9/2020|EPI_ISL_418778|2020-03-13 ....
A 4490 hCoV-19/USA/WA-NH10/2020|EPI_ISL_418779|2020-03-13 ....
A 4491 hCoV-19/USA/WA-NH11/2020|EPI_ISL_418780|2020-03-13 ....
A 4492 hCoV-19/USA/WA-NH12/2020|EPI_ISL_418781|2020-03-13 ....
A 4493 hCoV-19/USA/WA-NH13/2020|EPI_ISL_418782|2020-03-13 ....
A 4494 hCoV-19/USA/WA-NH14/2020|EPI_ISL_418783|2020-03-13 ....
A 4495 hCoV-19/USA/WA-NH17/2020|EPI_ISL_418784|2020-03-13 ....
A 4496 hCoV-19/USA/WA-NH18/2020|EPI_ISL_418785|2020-03-13 ....
A 4497 hCoV-19/USA/WA-NH19/2020|EPI_ISL_418786|2020-03-13 ....
A 4498 hCoV-19/USA/WA-NH20/2020|EPI_ISL_418787|2020-03-13 ....
A 4499 hCoV-19/USA/WA-NH21/2020|EPI_ISL_418788|2020-03-13 ....
A 4500 hCoV-19/USA/WA-NH22/2020|EPI_ISL_418789|2020-03-13 ....
A 4501 hCoV-19/USA/WA-NH23/2020|EPI_ISL_418790|2020-03-13 ....
A 4502 hCoV-19/USA/WA-NH24/2020|EPI_ISL_418791|2020-03-13 ....
A 4503 hCoV-19/Belgium/GL-030546/2020|EPI_ISL_418792|2020 ....
A 4504 hCoV-19/Belgium/SS-031047/2020|EPI_ISL_418793|2020 ....
A 4505 hCoV-19/Belgium/VHRA-030644/2020|EPI_ISL_418794|20 ....
A 4506 hCoV-19/Belgium/NKR-030645/2020|EPI_ISL_418795|202 ....
A 4507 hCoV-19/Belgium/VDLH-030548/2020|EPI_ISL_418796|20 ....

A	4508	hCoV-19/Belgium/VDLH-030548-2/2020|EPI_ISL_418797|	····················	····················	····················	····················	····················	····················		
A	4509	hCoV-19/Belgium/GS-030549/2020|EPI_ISL_418798|2020	····················	····················	····················	····················	····················	····················		
A	4510	hCoV-19/Australia/QLDID922/2020|EPI_ISL_418799|202	····················	····················	····················	····················	····················	····················		
A	4511	hCoV-19/England/201360220/2020|EPI_ISL_423250|2020	····················	····················	····················	····················	····················	····················		
A	4512	hCoV-19/England/201360221/2020|EPI_ISL_423251|2020	····················	····················	····················	····················	····················	····················		
A	4513	hCoV-19/England/201360223/2020|EPI_ISL_423253|2020	····················	····················	····················	····················	····················	····················		
A	4514	hCoV-19/England/201360224/2020|EPI_ISL_423254|2020	····················	····················	····················	····················	····················	····················		
A	4516	hCoV-19/England/201360236/2020|EPI_ISL_423256|2020	····················	····················	····················	····················	····················	····················		
A	4518	hCoV-19/England/201360251/2020|EPI_ISL_423259|2020	····················	····················	····················	····················	·N················NN	··N·················	1	1
A	4519	hCoV-19/England/201360275/2020|EPI_ISL_423260|2020	····················	····················	····················	····················	····················	·······R············		
A	4520	hCoV-19/England/201360276/2020|EPI_ISL_423261|2020	····················	····················	····················	····················	····················	····················		
A	4521	hCoV-19/England/201360277/2020|EPI_ISL_423262|2020	····················	····················	····················	····················	····················	····················		
A	4522	hCoV-19/England/201360281/2020|EPI_ISL_423263|2020	····················	····················	····················	····················	····················	····················		
A	4523	hCoV-19/England/201360317/2020|EPI_ISL_423264|2020	····················	····················	····················	····················	····················	····················		
A	4524	hCoV-19/England/201360318/2020|EPI_ISL_423265|2020	····················	····················	····················	····················	····················	NN·N················		1
A	4526	hCoV-19/England/20138007104/2020|EPI_ISL_423269|20	····················	····················	····················	····················	····················	···N················		
A	4528	hCoV-19/England/20138010904/2020|EPI_ISL_423277|20	····················	····················	····················	····················	····················	····················		
A	4529	hCoV-19/England/20138017804/2020|EPI_ISL_423288|20	····················	····················	····················	····················	····················	····················		
A	4530	hCoV-19/England/20138019104/2020|EPI_ISL_423292|20	····················	····················	····················	····················	····················	····················		
A	4531	hCoV-19/Iceland/477/2020|EPI_ISL_424500|2020-03-20	····················	····················	····················	····················	····················	····················		
A	4532	hCoV-19/Iceland/479/2020|EPI_ISL_424502|2020-03-20	····················	····················	····················	····················	····················	····················		
A	4533	hCoV-19/Iceland/480/2020|EPI_ISL_424503|2020-03-20	····················	····················	····················	····················	····················	····················		
A	4534	hCoV-19/Iceland/482/2020|EPI_ISL_424505|2020-03-20	····················	····················	····················	····················	····················	····················		
A	4535	hCoV-19/Iceland/483/2020|EPI_ISL_424506|2020-03-20	····················	····················	····················	····················	····················	····················		
A	4536	hCoV-19/Iceland/484/2020|EPI_ISL_424507|2020-03-20	····················	····················	····················	····················	····················	····················		
A	4537	hCoV-19/Iceland/485/2020|EPI_ISL_424508|2020-03-20	····················	····················	····················	····················	····················	····················		
A	4538	hCoV-19/Iceland/487/2020|EPI_ISL_424509|2020-03-20	····················	····················	····················	····················	····················	····················		
A	4539	hCoV-19/Iceland/488/2020|EPI_ISL_424510|2020-03-20	····················	····················	····················	····················	····················	····················		
A	4540	hCoV-19/Iceland/489/2020|EPI_ISL_424511|2020-03-20	····················	····················	····················	····················	····················	····················		
A	4541	hCoV-19/Iceland/490/2020|EPI_ISL_424512|2020-03-20	····················	····················	····················	····················	····················	····················		
A	4542	hCoV-19/Iceland/491/2020|EPI_ISL_424513|2020-03-20	····················	····················	····················	····················	····················	····················		
A	4543	hCoV-19/Iceland/560/2020|EPI_ISL_424580|2020-03-27	····················	····················	····················	····················	····················	····················		
A	4544	hCoV-19/Iceland/561/2020|EPI_ISL_424581|2020-03-28	····················	····················	····················	····················	····················	····················		
A	4545	hCoV-19/Iceland/562/2020|EPI_ISL_424582|2020-03-27	····················	····················	····················	····················	····················	····················		
A	4546	hCoV-19/Iceland/564/2020|EPI_ISL_424584|2020-03-28	····················	····················	····················	····················	····················	····················		
A	4547	hCoV-19/Iceland/565/2020|EPI_ISL_424585|2020-03-28	····················	····················	····················	····················	····················	····················		
A	4548	hCoV-19/Iceland/568/2020|EPI_ISL_424588|2020-03-28	····················	····················	····················	····················	····················	····················		
A	4549	hCoV-19/Iceland/569/2020|EPI_ISL_424589|2020-03-28	····················	····················	····················	····················	····················	····················		
A	4550	hCoV-19/Iceland/570/2020|EPI_ISL_424590|2020-03-28	····················	····················	····················	····················	····················	····················		
A	4551	hCoV-19/Iceland/571/2020|EPI_ISL_424591|2020-03-28	····················	····················	····················	····················	····················	····················		
A	4552	hCoV-19/Iceland/572/2020|EPI_ISL_424592|2020-03-28	····················	····················	····················	····················	····················	····················		
A	4553	hCoV-19/Iceland/573/2020|EPI_ISL_424593|2020-03-28	····················	····················	····················	····················	····················	····················		
A	4554	hCoV-19/Iceland/575/2020|EPI_ISL_424595|2020-03-28	····················	····················	····················	····················	····················	····················		
A	4555	hCoV-19/Iceland/576/2020|EPI_ISL_424596|2020-03-28	····················	····················	····················	····················	····················	····················		
A	4556	hCoV-19/Iceland/577/2020|EPI_ISL_424597|2020-03-27	····················	····················	····················	····················	····················	····················		
A	4557	hCoV-19/Iceland/578/2020|EPI_ISL_424598|2020-03-27	····················	····················	····················	····················	····················	····················		
A	4558	hCoV-19/Iceland/579/2020|EPI_ISL_424599|2020-03-27	····················	····················	····················	····················	····················	····················		
A	4559	hCoV-19/Scotland/EDB024/2020|EPI_ISL_425830|2020-0	····················	····················	····················	····················	····················	NN··················		
A	4561	hCoV-19/Scotland/EDB030/2020|EPI_ISL_425836|2020-0	····················	····················	····················	····················	····················	····················		
A	4562	hCoV-19/Scotland/EDB033/2020|EPI_ISL_425839|2020-0	····················	····················	····················	····················	····················	····················		
A	4563	hCoV-19/Scotland/EDB036/2020|EPI_ISL_425842|2020-0	····················	····················	····················	····················	····················	····················		
A	4564	hCoV-19/Scotland/EDB037/2020|EPI_ISL_425843|2020-0	····················	·············A······	····················	····················	····················	····················		
A	4565	hCoV-19/Scotland/EDB038/2020|EPI_ISL_425844|2020-0	····················	····················	····················	····················	····················	····················		
A	4566	hCoV-19/Scotland/EDB157/2020|EPI_ISL_425922|2020-0	····················	····················	····················	····················	····················	····················		
A	4568	hCoV-19/Scotland/EDB162/2020|EPI_ISL_425924|2020-0	····················	····················	····················	····················	····················	····················		
A	4569	hCoV-19/Scotland/EDB168/2020|EPI_ISL_425925|2020-0	····················	····················	····················	····················	····················	····················		
A	4571	hCoV-19/Scotland/EDB181/2020|EPI_ISL_425927|2020-0	····················	····················	····················	····················	····················	····················		
A	4573	hCoV-19/Denmark/ALAB-SSI204/2020|EPI_ISL_429402|20	····················	····················	····················	····················	····················	····················		
A	4574	hCoV-19/Denmark/ALAB-SSI207/2020|EPI_ISL_429404|20	····················	····················	····················	····················	····················	····················		
A	4575	hCoV-19/Denmark/ALAB-SSI209/2020|EPI_ISL_429405|20	····················	····················	····················	····················	····················	····················		
A	4576	hCoV-19/Denmark/ALAB-SSI213/2020|EPI_ISL_429406|20	····················	····················	····················	····················	····················	····················		
A	4577	hCoV-19/Denmark/ALAB-SSI215/2020|EPI_ISL_429407|20	····················	····················	····················	····················	····················	····················		
A	4578	hCoV-19/Denmark/ALAB-SSI216/2020|EPI_ISL_429408|20	····················	····················	····················	····················	····················	····················		
A	4579	hCoV-19/Denmark/ALAB-SSI219/2020|EPI_ISL_429409|20	····················	····················	····················	····················	····················	····················		
A	4580	hCoV-19/Denmark/ALAB-SSI221/2020|EPI_ISL_429410|20	····················	····················	····················	····················	····················	····················		
A	4581	hCoV-19/Denmark/ALAB-SSI235/2020|EPI_ISL_429414|20	····················	····················	····················	····················	····················	····················		
A	4582	hCoV-19/Denmark/ALAB-SSI258/2020|EPI_ISL_429420|20	····················	····················	····················	····················	····················	····················		
A	4583	hCoV-19/Denmark/ALAB-SSI259/2020|EPI_ISL_429421|20	····················	····················	····················	····················	····················	····················		
A	4584	hCoV-19/Denmark/ALAB-SSI262/2020|EPI_ISL_429424|20	····················	····················	····················	····················	····················	····················		
A	4585	hCoV-19/Denmark/ALAB-SSI263/2020|EPI_ISL_429425|20	····················	····················	····················	····················	····················	····················		
A	4586	hCoV-19/Denmark/ALAB-SSI264/2020|EPI_ISL_429426|20	····················	····················	····················	····················	····················	····················		
A	4587	hCoV-19/Denmark/ALAB-SSI265/2020|EPI_ISL_429427|20	····················	····················	····················	····················	····················	····················		
A	4588	hCoV-19/Denmark/ALAB-SSI268/2020|EPI_ISL_429428|20	····················	····················	····················	····················	····················	····················		
A	4589	hCoV-19/Denmark/ALAB-SSI271/2020|EPI_ISL_429431|20	····················	····················	····················	····················	····················	····················		
A	4590	hCoV-19/Denmark/ALAB-SSI272/2020|EPI_ISL_429433|20	····················	····················	····················	····················	····················	····················		
A	4591	hCoV-19/Denmark/ALAB-SSI273/2020|EPI_ISL_429434|20	····················	····················	····················	····················	····················	····················		
A	4592	hCoV-19/Denmark/ALAB-SSI279/2020|EPI_ISL_429439|20	····················	····················	····················	····················	····················	····················		
A	4593	hCoV-19/Denmark/ALAB-SSI280/2020|EPI_ISL_429440|20	····················	····················	····················	····················	····················	····················		

A	4594	hCoV-19/Denmark/ALAB-SSI29/2020|EPI_ISL_429447|202	....	....	....	....	....	....
A	4595	hCoV-19/Denmark/ALAB-SSI293/2020|EPI_ISL_429448|20	....	....	....	....	....	....
A	4596	hCoV-19/Denmark/ALAB-SSI298/2020|EPI_ISL_429449|20	....	....	....	....	....	....
A	4597	hCoV-19/Denmark/ALAB-SSI299/2020|EPI_ISL_429450|20	....	....	....	....	....	....
A	4598	hCoV-19/Denmark/ALAB-SSI300/2020|EPI_ISL_429451|20	....	....	....	....	....	....
A	4599	hCoV-19/Denmark/ALAB-SSI301/2020|EPI_ISL_429452|20	....	....	....	....	....	....
A	4600	hCoV-19/Denmark/ALAB-SSI302/2020|EPI_ISL_429453|20	....	....	....	....	....	....
A	4601	hCoV-19/Denmark/ALAB-SSI346/2020|EPI_ISL_429460|20	....	....	....	....	....	....
A	4602	hCoV-19/Denmark/ALAB-SSI347/2020|EPI_ISL_429461|20	....	....	....	....	....	....
A	4603	hCoV-19/Denmark/ALAB-SSI349/2020|EPI_ISL_429463|20	....	....	....	....	....	....
A	4604	hCoV-19/Denmark/ALAB-SSI351/2020|EPI_ISL_429464|20	....	....	....	....	....	....
A	4605	hCoV-19/Denmark/ALAB-SSI352/2020|EPI_ISL_429465|20	....	....	....	....	....	....
A	4606	hCoV-19/Denmark/ALAB-SSI354/2020|EPI_ISL_429466|20	....	....	....	....	....	....
A	4607	hCoV-19/Denmark/ALAB-SSI357/2020|EPI_ISL_429468|20	....	....	....	....	....	....
A	4608	hCoV-19/Denmark/ALAB-SSI358/2020|EPI_ISL_429469|20	....	....	....	....	....	....
A	4609	hCoV-19/Japan/NA-20-05-1/2020|EPI_ISL_410531|2020-	....	....	....	....	....	....
A	4610	hCoV-19/Japan/OS-20-07-1/2020|EPI_ISL_410532|2020-	....	....	....	....	....	....
A	4611	hCoV-19/Singapore/4/2020|EPI_ISL_410535|2020-02-03	....	....	....	....	....	....
A	4612	hCoV-19/Singapore/5/2020|EPI_ISL_410536|2020-02-06	....	....	....	....	....	....
A	4613	hCoV-19/Singapore/6/2020|EPI_ISL_410537|2020-02-09	....	....	....	....	....	....
A	4614	hCoV-19/Italy/LAZ-INMI1-isl/2020|EPI_ISL_410545|20	....	....	....	....	....	....
A	4615	hCoV-19/Italy/LAZ-INMI1-cs/2020|EPI_ISL_410546|202	....	....	....	....	....	....
A	4616	hCoV-19/France/IDF-0571/2020|EPI_ISL_411218|2020-0	....	....	....	....	....	....
A	4617	hCoV-19/France/IDF-0386-islP1/2020|EPI_ISL_411219|	....	....	....	....	....	....
A	4618	hCoV-19/France/IDF-0386-islP3/2020|EPI_ISL_411220|	....	....	....	....	....	....
A	4619	hCoV-19/Guangdong/GDSZ202008-P0020/2020|EPI_ISL_41	....	....	....	....	....	....
A	4620	hCoV-19/Guangdong/GDSZ202015-P0019/2020|EPI_ISL_41	....	....	....	....	....	....
A	4621	hCoV-19/Guangdong/GDFS2020052-P0025/2020|EPI_ISL_4	....	....	....	....	....	....
A	4622	hCoV-19/Guangdong/GD2020115-P0009/2020|EPI_ISL_413	....	....	....	....	....	....
A	4623	hCoV-19/Belgium/ULG-10026/2020|EPI_ISL_421200|2020	....	....	....	....	....	....
A	4624	hCoV-19/Belgium/ULG-10027/2020|EPI_ISL_421201|2020	....	....	....	....	....	....
A	4625	hCoV-19/Belgium/ULG-10028/2020|EPI_ISL_421202|2020	....	....	....	....	....	....
A	4626	hCoV-19/Hangzhou/HZCDC6789/2020|EPI_ISL_421221|202	....	....	....	....	....	....
A	4627	hCoV-19/Hangzhou/HZCDC0162/2020|EPI_ISL_421224|202	....	....	....	....	....	....
A	4628	hCoV-19/USA/NY-PV09126/2020|EPI_ISL_422500|2020-03	....	....	....	....	....	....
A	4629	hCoV-19/USA/NY-PV09127/2020|EPI_ISL_422501|2020-03	....	....	....	....	....	....
A	4630	hCoV-19/USA/NY-PV09128/2020|EPI_ISL_422502|2020-03	....	....	....	....	....	....
A	4631	hCoV-19/USA/NY-PV09129/2020|EPI_ISL_422503|2020-03	....	....	....	....	....	....
A	4632	hCoV-19/USA/NY-PV09130/2020|EPI_ISL_422504|2020-03	....	....	....	....	....	....
A	4633	hCoV-19/USA/NY-PV09132/2020|EPI_ISL_422505|2020-03	....	....	....	....	....	....
A	4634	hCoV-19/USA/NY-PV09134/2020|EPI_ISL_422506|2020-03	....	....	....	....	....	....
A	4635	hCoV-19/USA/NY-PV09135/2020|EPI_ISL_422507|2020-03	....	....	....	....	....	....
A	4636	hCoV-19/USA/NY-PV09137/2020|EPI_ISL_422508|2020-03	....	....	....	....	....	....
A	4637	hCoV-19/USA/NY-PV09138/2020|EPI_ISL_422509|2020-03	....	....	....	....	....	....
A	4638	hCoV-19/USA/NY-PV09139/2020|EPI_ISL_422510|2020-03	....	....	....	....	....	....
A	4639	hCoV-19/USA/NY-PV09140/2020|EPI_ISL_422511|2020-03	....	....	....	....	....	....
A	4640	hCoV-19/USA/NY-PV09142/2020|EPI_ISL_422513|2020-03	....	....	....	....	....	....
A	4641	hCoV-19/USA/NY-PV09143/2020|EPI_ISL_422514|2020-03	....	....	....	....	....	....
A	4642	hCoV-19/USA/NY-PV09144/2020|EPI_ISL_422515|2020-03	....	....	....	....	....	....
A	4643	hCoV-19/USA/NY-PV09145/2020|EPI_ISL_422516|2020-03	....	....	....	....	....	....
A	4644	hCoV-19/USA/NY-PV09147/2020|EPI_ISL_422517|2020-03	....	....	....	....	....	....
A	4645	hCoV-19/USA/NY-PV09151/2020|EPI_ISL_422519|2020-03	....	....	....	....	....	....
A	4646	hCoV-19/USA/NY-PV09152/2020|EPI_ISL_422520|2020-03	....	....	....	....	....	....
A	4647	hCoV-19/USA/NY-PV09153/2020|EPI_ISL_422521|2020-03	....	....	....	....	....	....
A	4648	hCoV-19/USA/NY-PV09154/2020|EPI_ISL_422522|2020-03	....	....	....	....	....	....
A	4649	hCoV-19/USA/NY-PV09156/2020|EPI_ISL_422523|2020-03	....	....	....	....	....	....
A	4650	hCoV-19/USA/NY-PV09157/2020|EPI_ISL_422524|2020-03	....	....	....	....	....	....
A	4651	hCoV-19/USA/NY-PV09158/2020|EPI_ISL_422525|2020-03	....	....	....	....	....	....
A	4652	hCoV-19/USA/NY-PV09163/2020|EPI_ISL_422527|2020-03	....	....	....	....	....	....
A	4653	hCoV-19/USA/NY-PV09164/2020|EPI_ISL_422528|2020-03	....	....	....	....	....	....
A	4654	hCoV-19/USA/NY-PV09165/2020|EPI_ISL_422529|2020-03	....	....	....	....	....	....
A	4655	hCoV-19/USA/NY-PV09168/2020|EPI_ISL_422530|2020-03	....	....	....	....	....	....
A	4656	hCoV-19/USA/NY-PV09169/2020|EPI_ISL_422531|2020-03	....	....	....	....	....	....
A	4657	hCoV-19/USA/NY-PV09170/2020|EPI_ISL_422532|2020-03	....	....	....	....	....	....
A	4658	hCoV-19/USA/NY-PV09171/2020|EPI_ISL_422533|2020-03	....	....	....	....	....	....
A	4659	hCoV-19/USA/NY-PV09197/2020|EPI_ISL_422552|2020-03	....	....	....	....	....	....
A	4660	hCoV-19/USA/NY-PV09198/2020|EPI_ISL_422553|2020-03	....	....	....	....	....	....
A	4661	hCoV-19/USA/NY-PV09199/2020|EPI_ISL_422554|2020-03	....	....	....	....	....	....
A	4662	hCoV-19/USA/NY-PV09200/2020|EPI_ISL_422555|2020-03	....	....	....	....	....	....
A	4663	hCoV-19/England/201360051/2020|EPI_ISL_423201|2020	....	....	....	....	....	....
A	4665	hCoV-19/England/201360053/2020|EPI_ISL_423203|2020	....	....	....	....	....	....
A	4666	hCoV-19/England/201360055/2020|EPI_ISL_423204|2020	....	....	....	....	....	....
A	4670	hCoV-19/England/201360075/2020|EPI_ISL_423210|2020	....	....	....	....	....	....
A	4671	hCoV-19/England/201360119/2020|EPI_ISL_423214|2020	....	....	....	....	....	....
A	4672	hCoV-19/England/20136012004/2020|EPI_ISL_423215|20	....	....	....	....	....	....
A	4673	hCoV-19/England/201360121/2020|EPI_ISL_423216|2020	....	....	....	....	....	....
A	4674	hCoV-19/England/201360126/2020|EPI_ISL_423218|2020	....	....	....	....	....	....
A	4675	hCoV-19/England/201360127/2020|EPI_ISL_423219|2020	....	....	....	....	....	....

A	4676 hCoV-19/England/201360131/2020|EPI_ISL_423221|2020	····················	····················	····················	····················	····················	····················		
A	4677 hCoV-19/England/201360136/2020|EPI_ISL_423223|2020	····················	····················	····················	····················	··N·················	····················		
A	4678 hCoV-19/England/201360137/2020|EPI_ISL_423224|2020	····················	····················	····················	····················	····················	····················		
A	4679 hCoV-19/England/201360143/2020|EPI_ISL_423227|2020	····················	····················	····················	····················	····················	····················		
A	4680 hCoV-19/England/201360171/2020|EPI_ISL_423228|2020	····················	····················	····················	····················	····················	····················		
A	4681 hCoV-19/England/201360178/2020|EPI_ISL_423231|2020	····················	····················	····················	····················	····················	····················		
A	4682 hCoV-19/England/201360181/2020|EPI_ISL_423232|2020	····················	····················	····················	····················	····················	····················		
A	4684 hCoV-19/England/201360191/2020|EPI_ISL_423235|2020	····················	····················	····················	····················	····················	····················		
A	4687 hCoV-19/England/201360201/2020|EPI_ISL_423238|2020	····················	····················	····················	····················	····················	····················		
A	4689 hCoV-19/England/201360203/2020|EPI_ISL_423240|2020	····················	····················	····················	····················	····················	····················		
A	4690 hCoV-19/England/201360206/2020|EPI_ISL_423241|2020	····················	····················	····················	····················	····················	·NN·················		
A	4691 hCoV-19/England/201360210/2020|EPI_ISL_423242|2020	····················	····················	····················	····················	····················	····················		
A	4692 hCoV-19/England/201360211/2020|EPI_ISL_423243|2020	····················	····················	····················	····················	····················	····················		
A	4693 hCoV-19/England/201360213/2020|EPI_ISL_423244|2020	····················	····················	····················	····················	····················	····················		
A	4694 hCoV-19/England/201360215/2020|EPI_ISL_423246|2020	····················	····················	····················	····················	····················	····················		
A	4696 hCoV-19/England/20132080004/2020|EPI_ISL_423803|20	····················	····················	····················	····················	····················	····················		
A	4698 hCoV-19/England/20132080804/2020|EPI_ISL_423808|20	····················	····················	····················	····················	····················	····················		
A	4699 hCoV-19/England/20132081104/2020|EPI_ISL_423809|20	····················	····················	····················	····················	····················	····················		
A	4703 hCoV-19/England/20132082004/2020|EPI_ISL_423813|20	····················	····················	····················	····················	····················	····················		
A	4704 hCoV-19/England/20132082504/2020|EPI_ISL_423817|20	····················	····················	····················	····················	····················	····················		
A	4705 hCoV-19/England/20132090404/2020|EPI_ISL_423818|20	····················	····················	····················	····················	····················	····················		
A	4706 hCoV-19/England/20132090504/2020|EPI_ISL_423819|20	····················	····················	····················	····················	····················	····················		
A	4708 hCoV-19/England/20132091704/2020|EPI_ISL_423823|20	····················	····················	····················	····················	····················	····················		
A	4709 hCoV-19/England/20132091804/2020|EPI_ISL_423824|20	····················	····················	····················	····················	····················	····················		
A	4710 hCoV-19/England/20132091904/2020|EPI_ISL_423825|20	····················	····················	····················	····················	····················	·N·········N·N······		1
A	4711 hCoV-19/England/20132095704/2020|EPI_ISL_423828|20	····················	····················	····················	····················	····················	····················		
A	4712 hCoV-19/England/20132096004/2020|EPI_ISL_423829|20	····················	····················	····················	····················	····················	····················		
A	4713 hCoV-19/England/20132096304/2020|EPI_ISL_423830|20	····················	····················	····················	····················	····················	····················		
A	4715 hCoV-19/England/20132100404/2020|EPI_ISL_423834|20	····················	····················	····················	····················	····················	····················		
A	4716 hCoV-19/England/20132100704/2020|EPI_ISL_423835|20	····················	····················	····················	····················	····················	····················		
A	4717 hCoV-19/England/20132104104/2020|EPI_ISL_423837|20	····················	····················	····················	····················	····················	····················		
A	4718 hCoV-19/England/20132104404/2020|EPI_ISL_423838|20	····················	····················	····················	····················	····················	····················		
A	4719 hCoV-19/England/20132105704/2020|EPI_ISL_423839|20	····················	····················	····················	····················	····················	····················		
A	4720 hCoV-19/England/20132106104/2020|EPI_ISL_423841|20	····················	····················	····················	····················	····················	····················		
A	4721 hCoV-19/England/20132107304/2020|EPI_ISL_423844|20	····················	····················	····················	····················	····················	····················		
A	4722 hCoV-19/England/20132107704/2020|EPI_ISL_423845|20	····················	····················	····················	····················	····················	····················		
A	4723 hCoV-19/England/20132108004/2020|EPI_ISL_423847|20	····················	····················	····················	····················	····················	····················		
A	4725 hCoV-19/England/20132108704/2020|EPI_ISL_423852|20	····················	····················	····················	····················	····················	····················		
A	4726 hCoV-19/England/20124036306/2020|EPI_ISL_423857|20	····················	····················	····················	····················	····················	····················		
A	4727 hCoV-19/England/20126000104/2020|EPI_ISL_423859|20	····················	····················	····················	····················	····················	····················		
A	4728 hCoV-19/England/20126000404/2020|EPI_ISL_423861|20	····················	····················	····················	····················	····················	····················		
A	4730 hCoV-19/England/20126001204/2020|EPI_ISL_423863|20	····················	····················	····················	····················	····················	··NN················		
A	4731 hCoV-19/England/20126001404/2020|EPI_ISL_423864|20	····················	····················	····················	····················	·················NNN	····················	1	1
A	4732 hCoV-19/England/20126009204/2020|EPI_ISL_423883|20	····················	····················	····················	····················	····················	····················		
A	4733 hCoV-19/Iceland/492/2020|EPI_ISL_424514|2020-03-20	····················	····················	····················	····················	····················	····················		
A	4734 hCoV-19/Iceland/493/2020|EPI_ISL_424515|2020-03-20	····················	····················	····················	····················	····················	····················		
A	4735 hCoV-19/Iceland/494/2020|EPI_ISL_424516|2020-03-20	····················	····················	····················	····················	····················	····················		
A	4736 hCoV-19/Iceland/495/2020|EPI_ISL_424517|2020-03-21	····················	····················	····················	····················	····················	····················		
A	4737 hCoV-19/Iceland/496/2020|EPI_ISL_424518|2020-03-21	····················	····················	····················	····················	····················	····················		
A	4738 hCoV-19/Iceland/497/2020|EPI_ISL_424519|2020-03-21	····················	····················	····················	····················	····················	····················		
A	4739 hCoV-19/Iceland/498/2020|EPI_ISL_424520|2020-03-21	····················	····················	····················	····················	····················	····················		
A	4740 hCoV-19/Iceland/499/2020|EPI_ISL_424521|2020-03-21	····················	····················	····················	····················	····················	····················		
A	4741 hCoV-19/Iceland/500/2020|EPI_ISL_424522|2020-03-21	····················	····················	····················	····················	····················	····················		
A	4742 hCoV-19/Iceland/501/2020|EPI_ISL_424523|2020-03-21	····················	····················	····················	····················	····················	····················		
A	4743 hCoV-19/Iceland/502/2020|EPI_ISL_424524|2020-03-21	····················	····················	····················	····················	····················	····················		
A	4744 hCoV-19/Iceland/503/2020|EPI_ISL_424525|2020-03-21	····················	····················	····················	····················	····················	····················		
A	4745 hCoV-19/Iceland/504/2020|EPI_ISL_424526|2020-03-21	····················	····················	····················	····················	····················	····················		
A	4746 hCoV-19/Iceland/505/2020|EPI_ISL_424527|2020-03-21	····················	····················	····················	····················	····················	····················		
A	4747 hCoV-19/Iceland/506/2020|EPI_ISL_424528|2020-03-21	····················	····················	····················	····················	····················	····················		
A	4748 hCoV-19/Iceland/507/2020|EPI_ISL_424529|2020-03-20	····················	····················	····················	····················	····················	····················		
A	4749 hCoV-19/Iceland/508/2020|EPI_ISL_424530|2020-03-20	····················	····················	····················	····················	····················	····················		
A	4750 hCoV-19/Iceland/509/2020|EPI_ISL_424531|2020-03-20	····················	····················	····················	····················	····················	····················		
A	4751 hCoV-19/Iceland/510/2020|EPI_ISL_424532|2020-03-20	····················	····················	····················	····················	····················	····················		
A	4752 hCoV-19/Iceland/511/2020|EPI_ISL_424533|2020-03-20	····················	····················	····················	····················	····················	····················		
A	4753 hCoV-19/Iceland/512/2020|EPI_ISL_424534|2020-03	····················	····················	····················	····················	····················	····················		
A	4754 hCoV-19/Iceland/513/2020|EPI_ISL_424535|2020-03-21	····················	····················	····················	····················	····················	····················		
A	4755 hCoV-19/Iceland/514/2020|EPI_ISL_424536|2020-03-21	····················	····················	····················	····················	····················	····················		
A	4756 hCoV-19/Iceland/515/2020|EPI_ISL_424537|2020-03	····················	····················	····················	····················	····················	····················		
A	4757 hCoV-19/Iceland/516/2020|EPI_ISL_424538|2020-03-24	····················	····················	····················	····················	····················	····················		
A	4758 hCoV-19/Iceland/519/2020|EPI_ISL_424540|2020-03	····················	····················	····················	····················	····················	····················		
A	4759 hCoV-19/Iceland/520/2020|EPI_ISL_424541|2020-03	····················	····················	····················	····················	····················	····················		
A	4760 hCoV-19/Iceland/521/2020|EPI_ISL_424542|2020-03	····················	····················	····················	····················	····················	····················		
A	4761 hCoV-19/Iceland/522/2020|EPI_ISL_424543|2020-03-25	····················	····················	····················	····················	····················	····················		
A	4762 hCoV-19/Iceland/524/2020|EPI_ISL_424545|2020-03-25	····················	····················	····················	····················	····················	····················		
A	4763 hCoV-19/Iceland/525/2020|EPI_ISL_424546|2020-03-25	····················	····················	····················	····················	····················	····················		
A	4764 hCoV-19/Iceland/526/2020|EPI_ISL_424547|2020-03	····················	····················	····················	····················	····················	····················		
A	4765 hCoV-19/Iceland/527/2020|EPI_ISL_424548|2020-03	····················	····················	····················	····················	····················	····················		
A	4766 hCoV-19/Iceland/528/2020|EPI_ISL_424549|2020-03	····················	····················	····················	····················	····················	····················		

A	4767	hCoV-19/Iceland/529/2020|EPI_ISL_424550|2020-03	....	....	....	....	....	....	
A	4768	hCoV-19/Iceland/530/2020|EPI_ISL_424551|2020-03-25	....	....	....	....	....	....	
A	4769	hCoV-19/Iceland/535/2020|EPI_ISL_424555|2020-03-29	....	....	....	....	....	....	
A	4770	hCoV-19/Iceland/536/2020|EPI_ISL_424556|2020-03-29	....	....	....	....	....	....	
A	4771	hCoV-19/Iceland/537/2020|EPI_ISL_424557|2020-03-27	....	....	....	....	....	....	
A	4772	hCoV-19/Iceland/538/2020|EPI_ISL_424558|2020-03-27	....	....	....	....	....	....	
A	4773	hCoV-19/Iceland/539/2020|EPI_ISL_424559|2020-03-27	....	....	....	....	....	....	
A	4774	hCoV-19/Iceland/540/2020|EPI_ISL_424560|2020-03-27	....	....	....	....	....	....	
A	4775	hCoV-19/Iceland/542/2020|EPI_ISL_424562|2020-03-26	....	....	....	....	....	....	
A	4776	hCoV-19/Iceland/543/2020|EPI_ISL_424563|2020-03-27	....	....	....	....	....	....	
A	4777	hCoV-19/Iceland/544/2020|EPI_ISL_424564|2020-03-27	....	....	....	....	....	....	
A	4778	hCoV-19/Iceland/547/2020|EPI_ISL_424567|2020-03-28	....	....	....	....	....	....	
A	4779	hCoV-19/Iceland/548/2020|EPI_ISL_424568|2020-03-28	....	....	....	....	....	....	
A	4780	hCoV-19/Iceland/550/2020|EPI_ISL_424570|2020-03-28	....	....	....	....	....	....	
A	4781	hCoV-19/Iceland/551/2020|EPI_ISL_424571|2020-03-27	....	....	....	....	....	....	
A	4782	hCoV-19/Iceland/552/2020|EPI_ISL_424572|2020-03-26	....	...................A	....	....	....	....	1
A	4783	hCoV-19/Iceland/553/2020|EPI_ISL_424573|2020-03-27	....	....	....	....	....	....	
A	4784	hCoV-19/Iceland/554/2020|EPI_ISL_424574|2020-03-27	....	....	....	....	....	....	
A	4785	hCoV-19/Iceland/555/2020|EPI_ISL_424575|2020-03-28	....	....	....	....	....	....	
A	4786	hCoV-19/Iceland/556/2020|EPI_ISL_424576|2020-03-27	....	....	....	....	....	....	
A	4787	hCoV-19/Iceland/557/2020|EPI_ISL_424577|2020-03-27	....	....	....	....	....	....	
A	4788	hCoV-19/Iceland/558/2020|EPI_ISL_424578|2020-03-27	....	....	....	....	....	....	
A	4789	hCoV-19/Scotland/EDB017/2020|EPI_ISL_425823|2020-0	....	....	....	....	....	....	
A	4791	hCoV-19/Scotland/EDB019/2020|EPI_ISL_425825|2020-0	....	....	....	....	....	....	
A	4792	hCoV-19/Scotland/EDB020/2020|EPI_ISL_425826|2020-0	....	....	....	....	....	....	
A	4793	hCoV-19/Scotland/EDB021/2020|EPI_ISL_425827|2020-0	....	....	....	....	....	....	
A	4794	hCoV-19/Scotland/EDB022/2020|EPI_ISL_425828|2020-0	....	....	....	....	....	....	
A	4795	hCoV-19/Scotland/EDB023/2020|EPI_ISL_425829|2020-0	....	....	....	....	....	N...................	
A	4796	hCoV-19/Scotland/EDB039/2020|EPI_ISL_425845|2020-0	....	....	....	....	....	....	
A	4798	hCoV-19/Scotland/EDB042/2020|EPI_ISL_425847|2020-0	....	....	....	....	....	....	
A	4800	hCoV-19/Scotland/EDB045/2020|EPI_ISL_425849|2020-0	....	....	....	....	.............N......	....	
A	4801	hCoV-19/Scotland/EDB046/2020|EPI_ISL_425850|2020-0	....	....	....	....	....	....	
A	4802	hCoV-19/Scotland/EDB047/2020|EPI_ISL_425851|2020-0	....	....	....	....	....	....	
A	4803	hCoV-19/Scotland/EDB048/2020|EPI_ISL_425852|2020-0	....	....	....	....	....	....	
A	4805	hCoV-19/Scotland/EDB052/2020|EPI_ISL_425856|2020-0	....	....	....	....	....	NN.....N............	1
A	4807	hCoV-19/Scotland/EDB057/2020|EPI_ISL_425861|2020-0	....	....	....	....	....	....	
A	4808	hCoV-19/Scotland/EDB058/2020|EPI_ISL_425862|2020-0	....	....	....	....	....	....	
A	4809	hCoV-19/Scotland/EDB063/2020|EPI_ISL_425863|2020-0	....	....	....	....	....	....	
A	4810	hCoV-19/Scotland/EDB065/2020|EPI_ISL_425864|2020-0	....	....	....	....	....	....	
A	4812	hCoV-19/Scotland/EDB067/2020|EPI_ISL_425866|2020-0	....	....	....	....	....	....	
A	4813	hCoV-19/Scotland/EDB068/2020|EPI_ISL_425867|2020-0	....	....	....	....	....	....	
A	4814	hCoV-19/Scotland/EDB070/2020|EPI_ISL_425868|2020-0	....	....	....	....	....	....	
A	4815	hCoV-19/Scotland/EDB071/2020|EPI_ISL_425869|2020-0	....	....	....	....	.............N......	NN..................	
A	4816	hCoV-19/Scotland/EDB073/2020|EPI_ISL_425870|2020-0	....	....	....	....	....	....	
A	4818	hCoV-19/Scotland/EDB075/2020|EPI_ISL_425872|2020-0	....	....	....	....	....	....	
A	4819	hCoV-19/Scotland/EDB081/2020|EPI_ISL_425873|2020-0	....	....	....	....	....	....	
A	4821	hCoV-19/Scotland/EDB083/2020|EPI_ISL_425875|2020-0	....	....	....	....	....	....	
A	4822	hCoV-19/Scotland/EDB084/2020|EPI_ISL_425876|2020-0	....	....	....	....	....	....	
A	4823	hCoV-19/Scotland/EDB085/2020|EPI_ISL_425877|2020-0	....	.A..................	....	....	....	....	
A	4824	hCoV-19/Scotland/EDB086/2020|EPI_ISL_425878|2020-0	....	....	....	....	....	....	
A	4825	hCoV-19/Scotland/EDB087/2020|EPI_ISL_425879|2020-0	....	....	....	....	....	....	
A	4826	hCoV-19/Scotland/EDB089/2020|EPI_ISL_425880|2020-0	....	....	....	....	....	....	
A	4827	hCoV-19/Scotland/EDB090/2020|EPI_ISL_425881|2020-0	....	....	....	....	....	....	
A	4828	hCoV-19/Scotland/EDB091/2020|EPI_ISL_425882|2020-0	....	....	....	....	....	....	
A	4831	hCoV-19/Scotland/EDB095/2020|EPI_ISL_425885|2020-0	....	....	....	....	....	....	
A	4833	hCoV-19/Scotland/EDB097/2020|EPI_ISL_425887|2020-0	....	....	....	....	....	....	
A	4834	hCoV-19/Scotland/EDB098/2020|EPI_ISL_425888|2020-0	....	....	....	....	....	....	
A	4835	hCoV-19/Scotland/EDB099/2020|EPI_ISL_425889|2020-0	....	....	....	....	....	....	
A	4837	hCoV-19/Scotland/EDB101/2020|EPI_ISL_425891|2020-0	....	....	....	....	....	....	
A	4838	hCoV-19/Scotland/EDB102/2020|EPI_ISL_425892|2020-0	....	....	....	....	....	....	
A	4839	hCoV-19/Scotland/EDB103/2020|EPI_ISL_425893|2020-0	....	....	....	....	....	....	
A	4842	hCoV-19/Scotland/EDB108/2020|EPI_ISL_425896|2020-0	....	....	....	....	....	....	
A	4843	hCoV-19/Scotland/EDB109/2020|EPI_ISL_425897|2020-0	....	....	....	....	....	....	
A	4845	hCoV-19/Scotland/EDB111/2020|EPI_ISL_425899|2020-0	....	....	....	....	....	....	
A	4846	hCoV-19/USA/WA-UW-2074/2020|EPI_ISL_426080|2020-03	....	....	....	....	....	....	
A	4847	hCoV-19/USA/WA-UW-2078/2020|EPI_ISL_426081|2020-03	....	....	....	....	....	....	
A	4848	hCoV-19/USA/WA-UW-2082/2020|EPI_ISL_426082|2020-03	....	....	....	....	....	....	
A	4849	hCoV-19/USA/WA-UW-2087/2020|EPI_ISL_426083|2020-03	....	....	....	....	....	....	
A	4850	hCoV-19/USA/WA-UW-2091/2020|EPI_ISL_426084|2020-03	....	....	....	....	....	....	
A	4851	hCoV-19/USA/WA-UW-2094/2020|EPI_ISL_426085|2020-03	....	....	....	....	....	....	
A	4852	hCoV-19/USA/WA-UW-2100/2020|EPI_ISL_426086|2020-03	....	....	....	....	....	....	
A	4853	hCoV-19/USA/WA-UW-2104/2020|EPI_ISL_426087|2020-03	....	....	....	....	....	....	
A	4854	hCoV-19/USA/WA-UW-2108/2020|EPI_ISL_426089|2020-03	....	....	....	....	....	....	
A	4855	hCoV-19/USA/WA-UW-2112/2020|EPI_ISL_426090|2020-03	....	....	....	....	....	....	
A	4856	hCoV-19/USA/WA-UW-2119/2020|EPI_ISL_426091|2020-03	....	....	....	....	....	....	
A	4857	hCoV-19/USA/WA-UW-2131/2020|EPI_ISL_426092|2020-03	....	....	....	....	....	....	
A	4858	hCoV-19/USA/WA-UW-2132/2020|EPI_ISL_426093|2020-03	....	....	....	....	....	....	
A	4859	hCoV-19/USA/WA-UW-2137/2020|EPI_ISL_426094|2020-03	....	....	....	....	....	....	

A	4860	hCoV-19/USA/WA-UW-2140/2020|EPI_ISL_426095|2020-03	....	....	....	....	....	....
A	4861	hCoV-19/USA/WA-UW-2141/2020|EPI_ISL_426096|2020-03	....	....	....	....	....	....
A	4862	hCoV-19/USA/WA-UW-2142/2020|EPI_ISL_426097|2020-03	....	....	....	....	....	....
A	4863	hCoV-19/USA/WA-UW-2144/2020|EPI_ISL_426098|2020-03	....	....	....	....	....	....
A	4864	hCoV-19/USA/WA-UW-2145/2020|EPI_ISL_426099|2020-03	....	....	....	....	....	....
A	4865	hCoV-19/Switzerland/GE-SNRCI-29945373/2020|EPI_ISL	....	....	....	....	....	....
A	4866	hCoV-19/Switzerland/BL-SNRCI-29950902/2020|EPI_ISL	....	....	....	....	....	....
A	4867	hCoV-19/Switzerland/GE-SNRCI-29949586/2020|EPI_ISL	....	....	....	....	....	....
A	4868	hCoV-19/Switzerland/VD-SNRCI-29965615/2020|EPI_ISL	....	....	....	....	....	....
A	4869	hCoV-19/Scotland/CVR05/2020|EPI_ISL_414027|2020-03	....	....	....	....	....	....
A	4870	hCoV-19/England/200641094/2020|EPI_ISL_414040|2020	....	....	....	....	....	....
A	4871	hCoV-19/England/200690245/2020|EPI_ISL_414041|2020	....	....	....	....	....	....
A	4872	hCoV-19/England/200690300/2020|EPI_ISL_414042|2020	....	....	....	....	....	....
A	4873	hCoV-19/England/200690306/2020|EPI_ISL_414043|2020	....	....	....	....	....	....
A	4874	hCoV-19/England/200690756/2020|EPI_ISL_414044|2020	....	....	....	....	....	....
A	4875	hCoV-19/Brazil/RJ-314/2020|EPI_ISL_414045|2020-03-	....	....	....	....	....	....
A	4876	hCoV-19/Japan/DP0438/2020|EPI_ISL_416600|2020-02-1	....	....	....	....	....	....
A	4877	hCoV-19/Japan/DP0457/2020|EPI_ISL_416601|2020-02-1	....	....	....	....	....	....
A	4878	hCoV-19/Japan/DP0462/2020|EPI_ISL_416602|2020-02-1	....	....	....	....	....	....
A	4879	hCoV-19/Japan/DP0464/2020|EPI_ISL_416603|2020-02-1	....	....	....	....	....	....
A	4880	hCoV-19/Japan/DP0476/2020|EPI_ISL_416604|2020-02-1	....	....	....	....	....	....
A	4881	hCoV-19/Japan/DP0481/2020|EPI_ISL_416605|2020-02-1	....	....	....	....	....	....
A	4882	hCoV-19/Japan/DP0482/2020|EPI_ISL_416606|2020-02-1	....	....	....	....	....	....
A	4883	hCoV-19/Japan/DP0543/2020|EPI_ISL_416607|2020-02-1	....	....	....	....	....	....
A	4884	hCoV-19/Japan/DP0544/2020|EPI_ISL_416608|2020-02-1	....	....	....	....	....	....
A	4885	hCoV-19/Japan/DP0568/2020|EPI_ISL_416609|2020-02-1	....	....	....	....	....	....
A	4886	hCoV-19/Japan/DP0588/2020|EPI_ISL_416610|2020-02-1	....	....	....	....	....	....
A	4887	hCoV-19/Japan/DP0644/2020|EPI_ISL_416611|2020-02-1	....	....	....	....	....	....
A	4888	hCoV-19/Japan/DP0645/2020|EPI_ISL_416612|2020-02-1	....	....	....	....	....	....
A	4889	hCoV-19/Japan/DP0654/2020|EPI_ISL_416613|2020-02-1	....	....	....	....	....	....
A	4890	hCoV-19/Japan/DP0687/2020|EPI_ISL_416614|2020-02-1	....	....	....	....	....	....
A	4891	hCoV-19/Japan/DP0690/2020|EPI_ISL_416615|2020-02-1	....	....	....	....	....	....
A	4892	hCoV-19/Japan/DP0803/2020|EPI_ISL_416630|2020-02-1	....	....	....	....	....	....
A	4893	hCoV-19/Japan/DP0804/2020|EPI_ISL_416631|2020-02-1	....	....	....	....	....	....
A	4894	hCoV-19/Japan/DP0827/2020|EPI_ISL_416632|2020-02-1	....	....	....	....	....	....
A	4895	hCoV-19/Japan/DP0880/2020|EPI_ISL_416633|2020-02-1	....	....	....	....	....	....
A	4896	hCoV-19/USA/WA-UW98/2020|EPI_ISL_416636|2020-03-12	....	....	....	....	....	....
A	4897	hCoV-19/USA/WA-UW99/2020|EPI_ISL_416637|2020-03-12	....	....	....	....	....	....
A	4898	hCoV-19/USA/WA-UW142/2020|EPI_ISL_416680|2020-03-1	....	....	....	....	....	....
A	4899	hCoV-19/USA/WA-UW143/2020|EPI_ISL_416681|2020-03-1	....	....	....	....	....	....
A	4900	hCoV-19/USA/WA-UW144/2020|EPI_ISL_416682|2020-03-0	....	....	....	....	....	....
A	4901	hCoV-19/USA/WA-UW145/2020|EPI_ISL_416683|2020-03-1	....	....	....	....	....	....
A	4902	hCoV-19/USA/WA-UW146/2020|EPI_ISL_416684|2020-03-1	....	....	....	....	....	....
A	4903	hCoV-19/USA/WA-UW147/2020|EPI_ISL_416685|2020-03-1	....	....	....	....	....	....
A	4904	hCoV-19/USA/WA-UW148/2020|EPI_ISL_416686|2020-03-1	....	....	....	....	....	....
A	4905	hCoV-19/USA/WA-UW149/2020|EPI_ISL_416687|2020-03-1	....	....	....	....	....	....
A	4906	hCoV-19/USA/WA-UW150/2020|EPI_ISL_416688|2020-03-1	....	....	....	....	....	....
A	4907	hCoV-19/USA/WA-UW151/2020|EPI_ISL_416689|2020-03-1	....	....	....	....	....	....
A	4908	hCoV-19/USA/WA-UW152/2020|EPI_ISL_416690|2020-03-1	....	....	....	....	....	....
A	4909	hCoV-19/USA/WA-UW153/2020|EPI_ISL_416691|2020-03-1	....	....	....	....	....	....
A	4910	hCoV-19/USA/WA-UW154/2020|EPI_ISL_416692|2020-03-1	....	....	....	....	....	....
A	4911	hCoV-19/USA/WA-UW155/2020|EPI_ISL_416693|2020-03-1	....	....	....	....	....	....
A	4912	hCoV-19/USA/WA-UW156/2020|EPI_ISL_416694|2020-03-1	....	....	....	....	....	....
A	4913	hCoV-19/USA/CT-UW158/2020|EPI_ISL_416696|2020-03-1	....	....	....	....	....	....
A	4914	hCoV-19/USA/CT-UW159/2020|EPI_ISL_416697|2020-03-1	....	....	....	....	....	....
A	4915	hCoV-19/USA/WA-UW160/2020|EPI_ISL_416698|2020-03-1	....	....	....	....	....	....
A	4916	hCoV-19/USA/WA-UW161/2020|EPI_ISL_416699|2020-03-1	....	....	....	....	....	....
A	4917	hCoV-19/USA/CA-CZB05/2020|EPI_ISL_417931|2020-03-1	....	....	....	....	....	....
A	4918	hCoV-19/USA/CA-CZB06/2020|EPI_ISL_417932|2020-03-1	....	....	....	....	....	....
A	4919	hCoV-19/USA/CA-CZB07/2020|EPI_ISL_417933|2020-03-1	....	....	....	....	....	....
A	4920	hCoV-19/USA/CA-CZB011a/2020|EPI_ISL_417935|2020-03	....	....	....	....	....	....
A	4921	hCoV-19/USA/CA-CZB013/2020|EPI_ISL_417937|2020-03-	....	....	....	....	....	....
A	4922	hCoV-19/USA/CA-CZB014/2020|EPI_ISL_417938|2020-03-	....	....	....	....	....	....
A	4923	hCoV-19/USA/CA-CZB015a/2020|EPI_ISL_417939|2020-03	....	....	....	....	....	....
A	4924	hCoV-19/DRC/73/2020|EPI_ISL_417941|2020-03-18	....	....	....	....	....	....
A	4925	hCoV-19/DRC/80/2020|EPI_ISL_417942|2020-03-18	....	....	....	....	....	....
A	4926	hCoV-19/DRC/81/2020|EPI_ISL_417944|2020-03-18	....	....	....	....	....	....
A	4927	hCoV-19/DRC/82/2020|EPI_ISL_417946|2020-03-18	....	....	....	....	....	....
A	4928	hCoV-19/USA/UT-012/2020|EPI_ISL_417960|2020-03-12	....	....	....	....	....	....
A	4929	hCoV-19/Spain/MD-HLP-H9-38/2020|EPI_ISL_417961|202	....	....	....	....	....	....
A	4930	hCoV-19/Spain/MD-HLP-H10-39/2020|EPI_ISL_417963|20	....	....	....	....	....	....
A	4931	hCoV-19/USA/UT-014/2020|EPI_ISL_417964|2020-03-13	....	....	....	....	....	....
A	4932	hCoV-19/Spain/MD-HLP-H11-40/2020|EPI_ISL_417967|20	....	....	....	....	....	....
A	4933	hCoV-19/Belgium/ULG-10012/2020|EPI_ISL_421190|2020	....	....	....	....	....	....
A	4934	hCoV-19/Belgium/ULG-10013/2020|EPI_ISL_421191|2020	....	....	....	....	....	....
A	4935	hCoV-19/Belgium/ULG-10014/2020|EPI_ISL_421192|2020	....	....	....	....	....	....
A	4936	hCoV-19/Belgium/ULG-10015/2020|EPI_ISL_421193|2020	....	....	....	....	....	....
A	4937	hCoV-19/Belgium/ULG-10016/2020|EPI_ISL_421194|2020	....	....	....	....	....	....

A	4938 hCoV-19/Belgium/ULG-10017/2020|EPI_ISL_421195|2020	····················	····················	·····················	····················	····················	····················
A	4939 hCoV-19/Belgium/ULG-10018/2020|EPI_ISL_421196|2020	····················	····················	·····················	····················	····················	····················
A	4940 hCoV-19/Belgium/ULG-10024/2020|EPI_ISL_421198|2020	····················	····················	·····················	····················	····················	····················
A	4941 hCoV-19/Belgium/ULG-10029/2020|EPI_ISL_421203|2020	····················	····················	·····················	····················	····················	····················
A	4942 hCoV-19/Belgium/ULG-10030/2020|EPI_ISL_421204|2020	····················	····················	·····················	····················	····················	····················
A	4943 hCoV-19/Belgium/ULG-10031/2020|EPI_ISL_421205|2020	····················	····················	·····················	····················	····················	····················
A	4944 hCoV-19/Belgium/ULG-10032/2020|EPI_ISL_421206|2020	····················	····················	·····················	····················	····················	····················
A	4945 hCoV-19/Belgium/ULG-10033/2020|EPI_ISL_421207|2020	····················	····················	·····················	····················	····················	····················
A	4946 hCoV-19/Belgium/ULG-10043/2020|EPI_ISL_421210|2020	····················	····················	·····················	····················	····················	····················
A	4947 hCoV-19/Belgium/ULG-10045/2020|EPI_ISL_421212|2020	····················	····················	·····················	····················	····················	····················
A	4948 hCoV-19/Belgium/ULG-10046/2020|EPI_ISL_421213|2020	····················	····················	·····················	····················	····················	····················
A	4949 hCoV-19/Belgium/ULG-10047/2020|EPI_ISL_421214|2020	····················	····················	·····················	····················	····················	····················
A	4950 hCoV-19/Hangzhou/HZCDC6706/2020|EPI_ISL_421222|202	····················	····················	·····················	····················	····················	····················
A	4951 hCoV-19/USA/NY-PV09172/2020|EPI_ISL_422534|2020-03	····················	····················	·····················	····················	····················	····················
A	4952 hCoV-19/USA/NY-PV09176/2020|EPI_ISL_422535|2020-03	····················	····················	·····················	····················	····················	····················
A	4953 hCoV-19/USA/NY-PV09177/2020|EPI_ISL_422536|2020-03	····················	····················	·····················	····················	····················	····················
A	4954 hCoV-19/USA/NY-PV09178/2020|EPI_ISL_422537|2020-03	····················	····················	·····················	····················	····················	····················
A	4955 hCoV-19/USA/NY-PV09180/2020|EPI_ISL_422539|2020-03	····················	····················	·····················	····················	····················	····················
A	4956 hCoV-19/USA/NY-PV09181/2020|EPI_ISL_422540|2020-03	····················	····················	·····················	····················	····················	····················
A	4957 hCoV-19/USA/NY-PV09182/2020|EPI_ISL_422541|2020-03	····················	····················	·····················	····················	····················	····················
A	4958 hCoV-19/USA/NY-PV09183/2020|EPI_ISL_422542|2020-03	····················	····················	·····················	····················	····················	····················
A	4959 hCoV-19/USA/NY-PV09186/2020|EPI_ISL_422543|2020-03	····················	····················	·····················	····················	····················	····················
A	4960 hCoV-19/USA/NY-PV09187/2020|EPI_ISL_422544|2020-03	····················	····················	·····················	····················	····················	····················
A	4961 hCoV-19/USA/NY-PV09189/2020|EPI_ISL_422546|2020-03	····················	·······C············	·····················	····················	····················	····················
A	4962 hCoV-19/USA/NY-PV09190/2020|EPI_ISL_422547|2020-03	····················	····················	·····················	····················	····················	····················
A	4963 hCoV-19/USA/NY-PV09192/2020|EPI_ISL_422548|2020-03	····················	····················	·····················	····················	····················	····················
A	4964 hCoV-19/USA/NY-PV09193/2020|EPI_ISL_422549|2020-03	····················	····················	·····················	····················	····················	····················
A	4965 hCoV-19/USA/NY-PV09194/2020|EPI_ISL_422550|2020-03	····················	····················	·····················	····················	····················	····················
A	4966 hCoV-19/USA/NY-PV09195/2020|EPI_ISL_422551|2020-03	····················	····················	·····················	····················	····················	····················
A	4967 hCoV-19/England/20126001604/2020|EPI_ISL_423865|20	····················	·A··················	·····················	····················	····················	····················
A	4969 hCoV-19/England/20126001904/2020|EPI_ISL_423867|20	····················	····················	·····················	····················	····················	····················
A	4970 hCoV-19/England/20126003406/2020|EPI_ISL_423877|20	····················	····················	·····················	····················	····················	····················
A	4971 hCoV-19/Scotland/EDB407/2020|EPI_ISL_426000|2020-0	····················	····················	·····················	····················	····················	····················
A	4972 hCoV-19/Scotland/EDB409/2020|EPI_ISL_426001|2020-0	····················	····················	·····················	····················	····················	····················
A	4973 hCoV-19/Scotland/EDB410/2020|EPI_ISL_426002|2020-0	····················	····················	·····················	····················	····················	····················
A	4974 hCoV-19/Scotland/EDB412/2020|EPI_ISL_426003|2020-0	····················	····················	·····················	····················	····················	····················
A	4975 hCoV-19/Scotland/EDB413/2020|EPI_ISL_426004|2020-0	····················	····················	·····················	····················	····················	····················
A	4976 hCoV-19/Scotland/EDB420/2020|EPI_ISL_426005|2020-0	····················	····················	·····················	····················	····················	····················
A	4977 hCoV-19/Scotland/EDB421/2020|EPI_ISL_426006|2020-0	····················	····················	·····················	····················	····················	····················
A	4978 hCoV-19/Scotland/EDB442/2020|EPI_ISL_426007|2020-0	····················	····················	·····················	····················	····················	····················
A	4979 hCoV-19/Scotland/EDB501/2020|EPI_ISL_426008|2020-0	····················	····················	·····················	····················	····················	····················
A	4980 hCoV-19/Scotland/EDB502/2020|EPI_ISL_426009|2020-0	····················	····················	·····················	····················	····················	····················
A	4981 hCoV-19/Scotland/EDB508/2020|EPI_ISL_426010|2020-0	····················	····················	·····················	····················	····················	····················
A	4982 hCoV-19/Scotland/EDB513/2020|EPI_ISL_426011|2020-0	····················	····················	·····················	····················	····················	····················
A	4983 hCoV-19/Scotland/EDB517/2020|EPI_ISL_426012|2020-0	····················	····················	·····················	····················	····················	····················
A	4984 hCoV-19/Scotland/EDB530/2020|EPI_ISL_426013|2020-0	····················	····················	·····················	····················	····················	····················
A	4985 hCoV-19/Scotland/EDB533/2020|EPI_ISL_426014|2020-0	····················	····················	·····················	····················	····················	····················
A	4986 hCoV-19/Scotland/EDB551/2020|EPI_ISL_426015|2020-0	····················	····················	·····················	····················	····················	····················
A	4987 hCoV-19/Scotland/EDB555/2020|EPI_ISL_426016|2020-0	····················	····················	·····················	····················	····················	····················
A	4988 hCoV-19/Scotland/EDB557/2020|EPI_ISL_426017|2020-0	····················	····················	·····················	····················	····················	····················
A	4989 hCoV-19/Scotland/EDB560/2020|EPI_ISL_426019|2020-0	····················	····················	·····················	····················	····················	····················
A	4990 hCoV-19/Scotland/EDB561/2020|EPI_ISL_426020|2020-0	····················	····················	·····················	····················	····················	····················
A	4991 hCoV-19/Scotland/EDB562/2020|EPI_ISL_426021|2020-0	····················	·A··················	·····················	····················	····················	····················
A	4992 hCoV-19/Scotland/EDB563/2020|EPI_ISL_426022|2020-0	····················	····················	·····················	····················	····················	····················
A	4993 hCoV-19/Scotland/EDB565/2020|EPI_ISL_426023|2020-0	·T··················	····················	·····················	····················	····················	····················
A	4994 hCoV-19/Scotland/EDB566/2020|EPI_ISL_426024|2020-0	····················	····················	·····················	····················	····················	····················
A	4995 hCoV-19/USA/NY-Wadsworth-10496-01/2020|EPI_ISL_426	····················	····················	·····················	····················	····················	····················
A	4996 hCoV-19/USA/NY-Wadsworth-10528-01/2020|EPI_ISL_426	····················	····················	·····················	····················	····················	····················
A	4997 hCoV-19/USA/NY-Wadsworth-10683-01/2020|EPI_ISL_426	····················	····················	·····················	····················	····················	····················
A	4998 hCoV-19/USA/NY-Wadsworth-10690-01/2020|EPI_ISL_426	····················	····················	·····················	····················	····················	····················
A	4999 hCoV-19/USA/NY-Wadsworth-10695-01/2020|EPI_ISL_426	····················	····················	·····················	····················	····················	····················
A	5000 hCoV-19/USA/NY-Wadsworth-10703-01/2020|EPI_ISL_426	····················	····················	·····················	····················	····················	····················
A	5001 hCoV-19/USA/NY-Wadsworth-10704-01/2020|EPI_ISL_426	····················	····················	·····················	····················	····················	····················
A	5002 hCoV-19/USA/NY-Wadsworth-10707-01/2020|EPI_ISL_426	····················	····················	·····················	····················	····················	····················
A	5003 hCoV-19/USA/NY-Wadsworth-10935-01/2020|EPI_ISL_426	····················	····················	·····················	····················	····················	····················
A	5004 hCoV-19/USA/NY-Wadsworth-10955-01/2020|EPI_ISL_426	····················	····················	·····················	····················	····················	····················
A	5005 hCoV-19/USA/NY-Wadsworth-10957-02/2020|EPI_ISL_426	····················	····················	·····················	····················	····················	····················
A	5006 hCoV-19/USA/NY-Wadsworth-10958-01/2020|EPI_ISL_426	····················	····················	·····················	····················	····················	····················
A	5007 hCoV-19/USA/NY-Wadsworth-10999-01/2020|EPI_ISL_426	····················	····················	·····················	····················	····················	····················
A	5008 hCoV-19/USA/NY-Wadsworth-11000-01/2020|EPI_ISL_426	····················	····················	·····················	····················	····················	····················
A	5009 hCoV-19/USA/NY-Wadsworth-11003-01/2020|EPI_ISL_426	····················	····················	·····················	····················	····················	····················
A	5010 hCoV-19/USA/NY-Wadsworth-11017-01/2020|EPI_ISL_426	····················	····················	·····················	····················	····················	····················
A	5011 hCoV-19/USA/NY-Wadsworth-11180-01/2020|EPI_ISL_426	····················	····················	·····················	····················	····················	····················
A	5012 hCoV-19/USA/NY-Wadsworth-11202-01/2020|EPI_ISL_426	····················	····················	·····················	····················	····················	····················
A	5013 hCoV-19/USA/NY-Wadsworth-11284-01/2020|EPI_ISL_426	····················	····················	·····················	····················	····················	····················
A	5014 hCoV-19/USA/NY-Wadsworth-11291-01/2020|EPI_ISL_426	····················	····················	·····················	····················	····················	····················
A	5015 hCoV-19/USA/NY-Wadsworth-11344-01/2020|EPI_ISL_426	····················	····················	·····················	····················	····················	····················
A	5016 hCoV-19/USA/NY-Wadsworth-11353-01/2020|EPI_ISL_426	····················	····················	·····················	····················	····················	····················

A	5017 hCoV-19/USA/NY-Wadsworth-11354-01/2020|EPI_ISL_426	....	....	....	....	....	....
A	5018 hCoV-19/USA/NY-Wadsworth-11379-01/2020|EPI_ISL_426	....	....	....	....	....	....
A	5019 hCoV-19/USA/NY-Wadsworth-11380-01/2020|EPI_ISL_426	....	....	....	....	....	....
A	5020 hCoV-19/USA/NY-Wadsworth-11666-01/2020|EPI_ISL_426	....	....	....	....	....	....
A	5021 hCoV-19/Czech Republic/2282/2020|EPI_ISL_426051|20	....	....	....	....	....	....
A	5022 hCoV-19/USA/CT-UW-4344/2020|EPI_ISL_426052|2020-03	....	....	....	....	....	....
A	5023 hCoV-19/USA/CT-UW-4346/2020|EPI_ISL_426053|2020-03	....	....	....	....	....	....
A	5024 hCoV-19/USA/CT-UW-4347/2020|EPI_ISL_426054|2020-03	....	....	....	....	....	....
A	5025 hCoV-19/USA/CT-UW-4366/2020|EPI_ISL_426055|2020-03	....	....	....	....	....	....
A	5026 hCoV-19/USA/CT-UW-4372/2020|EPI_ISL_426056|2020-03	....	....	....	....	....	....
A	5027 hCoV-19/USA/ID-UW-2127/2020|EPI_ISL_426057|2020-03	....	....	....	....	....	....
A	5028 hCoV-19/USA/ID-UW-2134/2020|EPI_ISL_426058|2020-03	....	....	....	....	....	....
A	5029 hCoV-19/USA/ID-UW-2159/2020|EPI_ISL_426059|2020-03	....	....	....	....	....	....
A	5030 hCoV-19/USA/ID-UW-2160/2020|EPI_ISL_426060|2020-03	....	....	....	....	....	....
A	5031 hCoV-19/USA/ID-UW-2174/2020|EPI_ISL_426061|2020-03	....	....	....	....	....	....
A	5032 hCoV-19/USA/ID-UW-2198/2020|EPI_ISL_426062|2020-03	....	....	....	....	....	....
A	5033 hCoV-19/USA/ID-UW-2255/2020|EPI_ISL_426063|2020-03	....	....	....	....	....	....
A	5034 hCoV-19/USA/ID-UW-4355/2020|EPI_ISL_426064|2020-03	....	....	....	....	....	....
A	5035 hCoV-19/USA/ID-UW-4357/2020|EPI_ISL_426065|2020-03	....	....	....	....	....	....
A	5036 hCoV-19/USA/ID-UW-4375/2020|EPI_ISL_426066|2020-03	....	....	....	....	....	....
A	5037 hCoV-19/USA/ID-UW-4378/2020|EPI_ISL_426067|2020-03	....	....	....	....	....	....
A	5038 hCoV-19/USA/ID-UW-4380/2020|EPI_ISL_426068|2020-03	....	....	....	....	....	....
A	5039 hCoV-19/USA/ID-UW-4396/2020|EPI_ISL_426069|2020-03	....	....	....	....	....	....
A	5040 hCoV-19/USA/ID-UW-4400/2020|EPI_ISL_426070|2020-03	....	....	....	....	....	....
A	5041 hCoV-19/USA/ID-UW-4427/2020|EPI_ISL_426072|2020-03	....	....	....	....	....	....
A	5042 hCoV-19/USA/ID-UW-4433/2020|EPI_ISL_426073|2020-03	....	....	....	....	....	....
A	5043 hCoV-19/USA/ID-UW-4448/2020|EPI_ISL_426074|2020-03	....	....	....	....	....	....
A	5044 hCoV-19/USA/ID-UW-4461/2020|EPI_ISL_426075|2020-03	....	....	....	....	....	....
A	5045 hCoV-19/USA/OR-UW-4388/2020|EPI_ISL_426076|2020-03	....	....	....	....	....	....
A	5046 hCoV-19/USA/UN-UW-2069/2020|EPI_ISL_426077|2020-03	....	....	....	....	....	....
A	5047 hCoV-19/USA/UN-UW-2139/2020|EPI_ISL_426078|2020-03	....	....	....	....	....	....
A	5048 hCoV-19/USA/WA-UW-2072/2020|EPI_ISL_426079|2020-03	....	....	....	....	....	....
A	5049 hCoV-19/Russia/StPetersburg-RII4711S/2020|EPI_ISL_	....	....	....	....	....	....
A	5050 hCoV-19/Russia/StPetersburg-RII4713S/2020|EPI_ISL_	....	....	....	....	....	....
A	5051 hCoV-19/Russia/StPetersburg-RII4723S/2020|EPI_ISL_	....	....	....	....	....	....
A	5052 hCoV-19/Russia/StPetersburg-RII4724S/2020|EPI_ISL_	....	....	....	....	....	....
A	5053 hCoV-19/Russia/StPetersburg-RII4726S/2020|EPI_ISL_	....	....	....	....	....	....
A	5054 hCoV-19/Russia/StPetersburg-RII4728S/2020|EPI_ISL_	....	....	....	....	....	....
A	5055 hCoV-19/Russia/StPetersburg-RII4781S/2020|EPI_ISL_	....	....	....	....	....	....
A	5056 hCoV-19/Russia/StPetersburg-RII4326S/2020|EPI_ISL_	....	....	....	....	....	....
A	5057 hCoV-19/Russia/StPetersburg-RII4328S/2020|EPI_ISL_	....	....	....	....	....	....
A	5058 hCoV-19/Russia/StPetersburg-RII4332S/2020|EPI_ISL_	....	....	....	....	....	....
A	5059 hCoV-19/Belgium/ULG-10098/2020|EPI_ISL_427349|2020	....	....	....	....	....	....
A	5060 hCoV-19/Belgium/ULG-10099/2020|EPI_ISL_427350|2020	....	....	....	....	....	....
A	5061 hCoV-19/Belgium/ULG-10100/2020|EPI_ISL_427351|2020	....	....	....	....	....	....
A	5062 hCoV-19/Belgium/ULG-10101/2020|EPI_ISL_427352|2020	....	....	....	....	....	....
A	5063 hCoV-19/Belgium/ULG-10103/2020|EPI_ISL_427354|2020	....	....	....	....	....	....
A	5064 hCoV-19/Belgium/ULG-10106/2020|EPI_ISL_427357|2020	....	....	....	....	....	....
A	5065 hCoV-19/Belgium/ULG-10107/2020|EPI_ISL_427358|2020	....	....	....	....	....	....
A	5066 hCoV-19/Belgium/ULG-10108/2020|EPI_ISL_427359|2020	....	....	....	....	....	....
A	5067 hCoV-19/Belgium/ULG-10115/2020|EPI_ISL_427364|2020	....	....	....	....	....	....
A	5068 hCoV-19/Belgium/ULG-10118/2020|EPI_ISL_427366|2020	....	....	....	....	....	....
A	5069 hCoV-19/Belgium/ULG-10122/2020|EPI_ISL_427370|2020	....	....	....	....	....	....
A	5070 hCoV-19/Belgium/ULG-10124/2020|EPI_ISL_427371|2020	....	....	....	....	....	....
A	5071 hCoV-19/Belgium/ULG-10126/2020|EPI_ISL_427372|2020	....	....	....	....	....	....
A	5072 hCoV-19/Belgium/ULG-10134/2020|EPI_ISL_427379|2020	....	....	....	....	....	....
A	5073 hCoV-19/Belgium/ULG-10142/2020|EPI_ISL_427385|2020	....	....	....	....	....	....
A	5074 hCoV-19/Belgium/ULG-10144/2020|EPI_ISL_427388|2020	....	....	....	....	....	....
A	5075 hCoV-19/Belgium/ULG-10125/2020|EPI_ISL_427390|2020	....	....	....	....	....	....
A	5076 hCoV-19/Turkey/IST-GLAB-008/2020|EPI_ISL_427391|20	....	....	....	....	....	....
A	5077 hCoV-19/Taiwan/TSGH-05/2020|EPI_ISL_427392|2020-03	....	....	....	....	....	....
A	5078 hCoV-19/Taiwan/TSGH-06/2020|EPI_ISL_427393|2020-03	....	....	....	....	....	....
A	5079 hCoV-19/Taiwan/TSGH-07/2020|EPI_ISL_427394|2020-03	....	....	....	....	....	....
A	5080 hCoV-19/Taiwan/TSGH-08/2020|EPI_ISL_427395|2020-03	....	....	....	....	....	....
A	5081 hCoV-19/Taiwan/TSGH-11/2020|EPI_ISL_427398|2020-03	....	....	....	....	....	....
A	5082 hCoV-19/Sri Lanka/COV38/2020|EPI_ISL_428670|2020-0	....	....	....	....	....	....
A	5083 hCoV-19/Sri Lanka/COV53/2020|EPI_ISL_428671|2020-0	....	....	....	....	....	....
A	5084 hCoV-19/Sri Lanka/COV91/2020|EPI_ISL_428672|2020-0	....	....	....	....	....	....
A	5085 hCoV-19/Sri Lanka/COV486/2020|EPI_ISL_428673|2020-	....	....	....	....	....	....
A	5086 hCoV-19/Spain/MD-H12-LP11-2271/2020|EPI_ISL_428674	....	....	....	....	....	....
A	5087 hCoV-19/Spain/MD-H12-LP16-6193/2020|EPI_ISL_428675	....	....	....	....	....	....
A	5088 hCoV-19/Spain/MD-H12-LP19-4952/2020|EPI_ISL_428676	....	....	....	....	....	....
A	5089 hCoV-19/Spain/MD-H12-LP20-2327/2020|EPI_ISL_428677	....	....	....	....	....	....
A	5090 hCoV-19/Spain/MD-H12-LP22-5885/2020|EPI_ISL_428678	....	....	....	....	....	....
A	5091 hCoV-19/Spain/MD-H12-LP23-5852/2020|EPI_ISL_428679	....	....	....	....	....	....
A	5092 hCoV-19/Spain/MD-H12-LP24-5999/2020|EPI_ISL_428680	....	....	....	....	....	....
A	5093 hCoV-19/Spain/MD-H12-LP27-2548/2020|EPI_ISL_428681	....	....	....	....	....	....
A	5094 hCoV-19/Spain/MD-H12-LP30-2226/2020|EPI_ISL_428682	....	....	....	....	....	....

A	5095 hCoV-19/Spain/MD-H12-13/2020|EPI_ISL_428683|2020-0	····················	····················	·····················	····················	····················	····················
A	5096 hCoV-19/Spain/MD-H12-20/2020|EPI_ISL_428684|2020-0	····················	····················	·····················	····················	····················	····················
A	5097 hCoV-19/Spain/MD-H12-23/2020|EPI_ISL_428686|2020-0	····················	····················	·····················	····················	····················	····················
A	5098 hCoV-19/Spain/MD-H12-24/2020|EPI_ISL_428687|2020-0	····················	····················	·····················	····················	····················	····················
A	5099 hCoV-19/Spain/MD-H12-25/2020|EPI_ISL_428688|2020-0	····················	····················	·····················	····················	····················	····················
A	5100 hCoV-19/Spain/MD-H12-26/2020|EPI_ISL_428689|2020-0	····················	····················	·····················	····················	····················	····················
A	5101 hCoV-19/Spain/MD-H12-27/2020|EPI_ISL_428690|2020-0	····················	····················	·····················	····················	····················	····················
A	5102 hCoV-19/Spain/MD-H12-28/2020|EPI_ISL_428691|2020-0	····················	····················	·····················	····················	····················	····················
A	5103 hCoV-19/Spain/MD-H12-29/2020|EPI_ISL_428692|2020-0	····················	····················	·····················	····················	····················	····················
A	5104 hCoV-19/Spain/MD-H12-30/2020|EPI_ISL_428693|2020-0	····················	····················	·····················	····················	····················	····················
A	5105 hCoV-19/Spain/MD-H12-31/2020|EPI_ISL_428694|2020-0	····················	····················	·····················	····················	····················	····················
A	5106 hCoV-19/Spain/MD-H12-32/2020|EPI_ISL_428695|2020-0	····················	····················	·····················	····················	····················	····················
A	5107 hCoV-19/Spain/MD-H12-33/2020|EPI_ISL_428696|2020-0	····················	····················	·····················	····················	····················	····················
A	5108 hCoV-19/Spain/MD-H12-34/2020|EPI_ISL_428697|2020-0	····················	····················	·····················	····················	····················	····················
A	5109 hCoV-19/Spain/MD-H12-35/2020|EPI_ISL_428698|2020-0	····················	····················	·····················	····················	····················	····················
A	5110 hCoV-19/Spain/MD-H12-36/2020|EPI_ISL_428699|2020-0	····················	····················	·····················	····················	····················	····················
A	5111 hCoV-19/USA/CA-SR007/2020|EPI_ISL_429990|2020-03-2	····················	····················	·····················	····················	····················	····················
A	5112 hCoV-19/USA/CA-SR017/2020|EPI_ISL_429991|2020-03-2	····················	····················	·····················	····················	····················	····················
A	5113 hCoV-19/Jordan/SR-032/2020|EPI_ISL_429992|2020-03-	····················	····················	·····················	····················	····················	····················
A	5114 hCoV-19/Jordan/SR-033/2020|EPI_ISL_429993|2020-03-	····················	····················	·····················	····················	····················	····················
A	5115 hCoV-19/Jordan/SR-034/2020|EPI_ISL_429994|2020-03-	····················	····················	·····················	····················	····················	····················
A	5117 hCoV-19/Jordan/SR-036/2020|EPI_ISL_429996|2020-03-	····················	····················	·····················	····················	····················	····················
A	5118 hCoV-19/Jordan/SR-037/2020|EPI_ISL_429997|2020-03-	····················	·············A······	·····················	····················	····················	····················
A	5119 hCoV-19/Jordan/SR-039/2020|EPI_ISL_429998|2020-03-	····················	····················	·····················	····················	····················	····················
A	5120 hCoV-19/Jordan/SR-041/2020|EPI_ISL_429999|2020-03-	····················	····················	·····················	····················	····················	····················
A	5121 hCoV-19/France/ARA-739/2020|EPI_ISL_410486|2020-02	····················	····················	·····················	····················	····················	····················
A	5122 hCoV-19/Jiangsu/JS01/2020|EPI_ISL_411950|2020-01-2	····················	····················	·····················	····················	····················	····················
A	5123 hCoV-19/Sweden/01/2020|EPI_ISL_411951|2020-02-07	····················	····················	·····················	····················	····················	····················
A	5124 hCoV-19/Jiangsu/JS02/2020|EPI_ISL_411952|2020-01-2	····················	····················	·····················	····················	····················	····················
A	5125 hCoV-19/Jiangsu/JS03/2020|EPI_ISL_411953|2020-01-2	····················	····················	·····················	····················	····················	····················
A	5126 hCoV-19/USA/CA-CDC-7/2020|EPI_ISL_411954|2020-02-0	····················	····················	·····················	····················	····················	····················
A	5127 hCoV-19/USA/CA-CDC-8/2020|EPI_ISL_411955|2020-02-1	····················	····················	·····················	····················	····················	····················
A	5128 hCoV-19/USA/TX1/2020|EPI_ISL_411956|2020-02-11	····················	····················	·····················	····················	····················	····················
A	5129 hCoV-19/England/200940527/2020|EPI_ISL_414005|2020	····················	····················	·····················	····················	····················	····················
A	5130 hCoV-19/England/200990724/2020|EPI_ISL_414006|2020	····················	····················	·····················	····················	····················	····················
A	5131 hCoV-19/England/200990725/2020|EPI_ISL_414007|2020	····················	····················	·····················	····················	····················	····················
A	5132 hCoV-19/England/200960041/2020|EPI_ISL_414008|2020	····················	····················	·····················	····················	····················	····················
A	5133 hCoV-19/England/200960515/2020|EPI_ISL_414009|2020	····················	····················	·····················	····················	····················	····················
A	5134 hCoV-19/England/200981386/2020|EPI_ISL_414010|2020	···T················	····················	·····················	····················	····················	····················
A	5135 hCoV-19/England/200990006/2020|EPI_ISL_414011|2020	····················	····················	·····················	····················	····················	····················
A	5136 hCoV-19/England/200990723/2020|EPI_ISL_414012|2020	····················	····················	·····················	····················	····················	····················
A	5137 hCoV-19/England/201000003/2020|EPI_ISL_414013|2020	····················	····················	·····················	····················	····················	····················
A	5138 hCoV-19/Brazil/SP-03/2020|EPI_ISL_414014|2020-03-0	····················	····················	·····················	····················	····················	····················
A	5139 hCoV-19/Brazil/SP-06/2020|EPI_ISL_414015|2020-02-2	····················	····················	·····················	····················	····················	····················
A	5140 hCoV-19/Brazil/SP-05/2020|EPI_ISL_414016|2020-02-2	····················	····················	·····················	····················	····················	····················
A	5141 hCoV-19/Brazil/SP-04/2020|EPI_ISL_414017|2020-03-0	····················	····················	·····················	····················	····················	····················
A	5142 hCoV-19/Switzerland/GE-SNRCI-29943121/2020|EPI_ISL	····················	····················	·····················	····················	····················	····················
A	5143 hCoV-19/Japan/DP0699/2020|EPI_ISL_416617|2020-02-1	····················	····················	·····················	····················	····················	····················
A	5144 hCoV-19/Japan/DP0700/2020|EPI_ISL_416618|2020-02-1	····················	····················	·····················	····················	····················	····················
A	5145 hCoV-19/Japan/DP0703/2020|EPI_ISL_416619|2020-02-1	····················	····················	·····················	····················	····················	····················
A	5146 hCoV-19/Japan/DP0724/2020|EPI_ISL_416620|2020-02-1	····················	····················	·····················	····················	····················	····················
A	5147 hCoV-19/Japan/DP0743/2020|EPI_ISL_416621|2020-02-1	····················	····················	·····················	····················	····················	····················
A	5148 hCoV-19/Japan/DP0752/2020|EPI_ISL_416622|2020-02-1	····················	····················	·····················	····················	····················	····················
A	5149 hCoV-19/Japan/DP0764/2020|EPI_ISL_416624|2020-02-1	····················	····················	·····················	····················	····················	····················
A	5150 hCoV-19/Japan/DP0765/2020|EPI_ISL_416625|2020-02-1	····················	····················	·····················	····················	····················	····················
A	5151 hCoV-19/Japan/DP0779/2020|EPI_ISL_416626|2020-02-1	····················	····················	·····················	····················	····················	····················
A	5152 hCoV-19/Japan/DP0785/2020|EPI_ISL_416627|2020-02-1	····················	····················	·····················	····················	····················	····················
A	5153 hCoV-19/Japan/DP0786/2020|EPI_ISL_416628|2020-02-1	····················	····················	·····················	····················	····················	····················
A	5154 hCoV-19/Japan/DP0802/2020|EPI_ISL_416629|2020-02-1	····················	····················	·····················	····················	····················	····················
A	5155 hCoV-19/Japan/DP0890/2020|EPI_ISL_416634|2020-02-1	····················	····················	·····················	····················	····················	····················
A	5156 hCoV-19/USA/WA-UW97/2020|EPI_ISL_416635|2020-03-12	····················	····················	·····················	····················	····················	····················
A	5157 hCoV-19/USA/WA-UW100/2020|EPI_ISL_416638|2020-03-1	····················	····················	·····················	····················	····················	····················
A	5158 hCoV-19/USA/WA-UW101/2020|EPI_ISL_416639|2020-03-1	····················	····················	·····················	····················	····················	····················
A	5159 hCoV-19/USA/WA-UW103/2020|EPI_ISL_416641|2020-03-1	····················	····················	·····················	····················	····················	····················
A	5160 hCoV-19/USA/WA-UW104/2020|EPI_ISL_416642|2020-03-1	····················	····················	·····················	····················	····················	····················
A	5161 hCoV-19/USA/WA-UW105/2020|EPI_ISL_416643|2020-03-1	····················	····················	·····················	····················	····················	····················
A	5162 hCoV-19/USA/WA-UW106/2020|EPI_ISL_416644|2020-03-1	····················	····················	·····················	····················	····················	····················
A	5163 hCoV-19/USA/WA-UW108/2020|EPI_ISL_416646|2020-03-1	····················	····················	·····················	····················	····················	····················
A	5164 hCoV-19/USA/WA-UW109/2020|EPI_ISL_416647|2020-03-1	····················	····················	·····················	····················	····················	····················
A	5165 hCoV-19/USA/WA-UW110/2020|EPI_ISL_416648|2020-03-1	····················	····················	·····················	····················	····················	····················
A	5166 hCoV-19/USA/WA-UW111/2020|EPI_ISL_416649|2020-03-1	····················	····················	·····················	····················	····················	····················
A	5167 hCoV-19/USA/WA-UW112/2020|EPI_ISL_416650|2020-03-1	····················	····················	·····················	····················	····················	····················
A	5168 hCoV-19/USA/WA-UW113/2020|EPI_ISL_416651|2020-03-1	····················	····················	·····················	····················	····················	····················
A	5169 hCoV-19/USA/WA-UW114/2020|EPI_ISL_416652|2020-03-1	····················	····················	·····················	····················	····················	····················
A	5170 hCoV-19/USA/WA-UW115/2020|EPI_ISL_416653|2020-03-1	····················	····················	·····················	····················	····················	····················
A	5171 hCoV-19/USA/WA-UW116/2020|EPI_ISL_416654|2020-03-1	····················	····················	·····················	····················	····················	····················
A	5172 hCoV-19/USA/WA-UW117/2020|EPI_ISL_416655|2020-03-1	····················	····················	·····················	····················	····················	····················
A	5173 hCoV-19/USA/WA-UW118/2020|EPI_ISL_416656|2020-03-1	····················	····················	·····················	····················	····················	····················

A	5174	hCoV-19/USA/WA-UW119/2020|EPI_ISL_416657|2020-03-1	....	....	....	....	....	....
A	5175	hCoV-19/USA/WA-UW120/2020|EPI_ISL_416658|2020-03-1	....	....	....	....	....	....
A	5176	hCoV-19/USA/WA-UW121/2020|EPI_ISL_416659|2020-03-1	....	....	....	....	....	....
A	5177	hCoV-19/USA/WA-UW122/2020|EPI_ISL_416660|2020-03-1	....	....	....	....	....	....
A	5178	hCoV-19/USA/WA-UW123/2020|EPI_ISL_416661|2020-03-1	....	....	....	....	....	....
A	5179	hCoV-19/USA/WA-UW124/2020|EPI_ISL_416662|2020-03-1	....	....	....	....	....	....
A	5180	hCoV-19/USA/WA-UW125/2020|EPI_ISL_416663|2020-03-1	....	....	....	....	....	....
A	5181	hCoV-19/USA/WA-UW126/2020|EPI_ISL_416664|2020-03-1	....	....	....	....	....	....
A	5182	hCoV-19/USA/WA-UW127/2020|EPI_ISL_416665|2020-03-1	....	....	....	....	....	....
A	5183	hCoV-19/USA/WA-UW128/2020|EPI_ISL_416666|2020-03-1	....	....	....	....	....	....
A	5184	hCoV-19/USA/WA-UW129/2020|EPI_ISL_416667|2020-03-1	....	....	....	....	....	....
A	5185	hCoV-19/USA/WA-UW130/2020|EPI_ISL_416668|2020-03-1	....	....	....	....	....	....
A	5186	hCoV-19/USA/WA-UW131/2020|EPI_ISL_416669|2020-03-1	....	....	....	....	....	....
A	5187	hCoV-19/USA/WA-UW133/2020|EPI_ISL_416671|2020-03-1	....	....	....	....	....	....
A	5188	hCoV-19/USA/OR-UW135/2020|EPI_ISL_416673|2020-03-1	....	....	....	....	....	....
A	5189	hCoV-19/USA/WA-UW136/2020|EPI_ISL_416674|2020-03-1	....	....	....	....	....	....
A	5190	hCoV-19/USA/WA-UW137/2020|EPI_ISL_416675|2020-03-1	....	....	....	....	....	....
A	5191	hCoV-19/USA/WA-UW138/2020|EPI_ISL_416676|2020-03-1	....	....	....	....	....	....
A	5192	hCoV-19/USA/WA-UW139/2020|EPI_ISL_416677|2020-03-1	....	....	....	....	....	....
A	5193	hCoV-19/USA/WA-UW140/2020|EPI_ISL_416678|2020-03-1	....	....	....	....	....	....
A	5194	hCoV-19/USA/WA-UW141/2020|EPI_ISL_416679|2020-03-1	....	....	....	....	....	....
A	5195	hCoV-19/Malaysia/189332/2020|EPI_ISL_417917|2020-0	....	....	....	....	....	....
A	5196	hCoV-19/Malaysia/188407/2020|EPI_ISL_417918|2020-0	....	....	....	....	....	....
A	5197	hCoV-19/Italy/LAZ-INMI3/2020|EPI_ISL_417921|2020-0	....	....	....	....	....	....
A	5198	hCoV-19/Italy/LAZ-INMI4/2020|EPI_ISL_417922|2020-0	....	....	....	....	....	....
A	5199	hCoV-19/Italy/LAZ-INMI5/2020|EPI_ISL_417923|2020-0	....	....	....	....	....	....
A	5200	hCoV-19/Colombia/ANT-INS-79256/2020|EPI_ISL_417924	....	....	....	....	....	....
A	5201	hCoV-19/DRC/94/2020|EPI_ISL_417947|2020-03-19	....	....	....	....	....	....
A	5202	hCoV-19/DRC/108/2020|EPI_ISL_417948|2020-03-19	....	....	....	....	....	....
A	5203	hCoV-19/DRC/158/2020|EPI_ISL_417950|2020-03-20	....	....	....	....	....	....
A	5204	hCoV-19/Spain/MD-HLP-H3-10/2020|EPI_ISL_417954|202	....	....	....	....	....	....
A	5205	hCoV-19/DRC/191/2020|EPI_ISL_417955|2020-03-21	....	....	....	....	....	....
A	5206	hCoV-19/USA/UT-010/2020|EPI_ISL_417958|2020-03-10	....	....	....	....	....	....
A	5208	hCoV-19/USA/UT-016/2020|EPI_ISL_417966|2020-03-13	....	....	....	....	....	....
A	5209	hCoV-19/Spain/MD-HLP-LP10-12/2020|EPI_ISL_417969|2	....	....	....	....	....	....
A	5210	hCoV-19/USA/UT-027/2020|EPI_ISL_417970|2020-03-16	....	....	....	....	....	....
A	5211	hCoV-19/USA/UT-028/2020|EPI_ISL_417971|2020-03-19	....	....	....	....	....	....
A	5212	hCoV-19/Spain/MD-HLP-LP12-21/2020|EPI_ISL_417972|2	....	....	....	....	....	....
A	5213	hCoV-19/USA/UT-031/2020|EPI_ISL_417973|2020-03-19	....	....	....	....	....	....
A	5214	hCoV-19/USA/UT-032/2020|EPI_ISL_417974|2020-03-19	....	....	....	....	....	....
A	5215	hCoV-19/Spain/MD-HLP-LP14-3/2020|EPI_ISL_417975|20	....	....	....	....	....	....
A	5216	hCoV-19/USA/UT-033/2020|EPI_ISL_417976|2020-03-19	....	....	....	....	....	....
A	5217	hCoV-19/USA/UT-034/2020|EPI_ISL_417977|2020-03-20	....	....	....	....	....	....
A	5218	hCoV-19/Spain/MD-HLP-LP15-4/2020|EPI_ISL_417978|20	....	....	....	....	....	....
A	5219	hCoV-19/Spain/MD-HLP-R2-15/2020|EPI_ISL_417979|202	....	....	....	....	....	....
A	5220	hCoV-19/Spain/MD-HLP-R5-8/2020|EPI_ISL_417980|2020	....	....	....	....	....	....
A	5221	hCoV-19/Spain/MD-HLP-R10-33/2020|EPI_ISL_417981|20	....	....	....	....	....	....
A	5222	hCoV-19/Portugal/PT0001b/2020|EPI_ISL_417986|2020-	....	....	....	....	....	....
A	5223	hCoV-19/Portugal/PT0003/2020|EPI_ISL_417987|2020-0	....	....	....	....	....	....
A	5224	hCoV-19/Portugal/PT0004/2020|EPI_ISL_417988|2020-0	....	....	....	....	....	....
A	5225	hCoV-19/Portugal/PT0005/2020|EPI_ISL_417989|2020-0	....	....	....	....	....	....
A	5226	hCoV-19/Portugal/PT0006a/2020|EPI_ISL_417990|2020-	....	....	....	....	....	....
A	5227	hCoV-19/Portugal/PT0006b/2020|EPI_ISL_417991|2020-	....	....	....	....	....	....
A	5228	hCoV-19/Portugal/PT0007/2020|EPI_ISL_417992|2020-0	....	....	....	....	....	....
A	5229	hCoV-19/Portugal/PT0008/2020|EPI_ISL_417993|2020-0	....	....	....	....	....	....
A	5230	hCoV-19/Portugal/PT0009/2020|EPI_ISL_417994|2020-0	....	....	....	....	....	....
A	5231	hCoV-19/Portugal/PT0010/2020|EPI_ISL_417995|2020-0	....	....	....	....	....	....
A	5232	hCoV-19/Portugal/PT0011/2020|EPI_ISL_417996|2020-0	....	....	....	....	....	....
A	5233	hCoV-19/Portugal/PT0012/2020|EPI_ISL_417997|2020-0	....	....	....	....	....	....
A	5234	hCoV-19/Portugal/PT0013/2020|EPI_ISL_417998|2020-0	....	....	....	....	....	....
A	5235	hCoV-19/Portugal/PT0014/2020|EPI_ISL_417999|2020-0	....	....	....	....	....	....
A	5236	hCoV-19/Spain/MD-H12-1301/2020|EPI_ISL_421171|2020	....	....	....	....	....	....
A	5237	hCoV-19/Spain/MD-H12-H12-1502/2020|EPI_ISL_421172|	....	....	....	....	....	....
A	5238	hCoV-19/Spain/MD-H12-H12-1703/2020|EPI_ISL_421173|	....	....	....	....	....	....
A	5239	hCoV-19/Spain/MD-H12-1804/2020|EPI_ISL_421174|2020	....	....	....	....	....	....
A	5240	hCoV-19/Spain/MD-H12-H12-1905/2020|EPI_ISL_421175|	....	....	....	....	....	....
A	5241	hCoV-19/Spain/MD-H12-2006/2020|EPI_ISL_421176|2020	....	....	....	....	....	....
A	5242	hCoV-19/Spain/MD-H12-H12-2107/2020|EPI_ISL_421177|	....	....	....	....	....	....
A	5243	hCoV-19/Spain/MD-H12-H12-2208/2020|EPI_ISL_421178|	....	....	....	....	....	....
A	5244	hCoV-19/Spain/MD-H12-H12-2309/2020|EPI_ISL_421179|	....	....	....	....	....	....
A	5245	hCoV-19/Spain/MD-H12-2410/2020|EPI_ISL_421180|2020	....	....	....	....	....	....
A	5246	hCoV-19/Belgium/ULG-10001/2020|EPI_ISL_421182|2020	....	....	....	....	....	....
A	5247	hCoV-19/Belgium/ULG-10003/2020|EPI_ISL_421183|2020	....	....	....	....	....	....
A	5248	hCoV-19/Belgium/ULG-10004/2020|EPI_ISL_421184|2020	....	....	....	....	....	....
A	5249	hCoV-19/Belgium/ULG-10006/2020|EPI_ISL_421185|2020	....	....	....	....	....	....
A	5250	hCoV-19/Belgium/ULG-10007/2020|EPI_ISL_421186|2020	....	....	....	....	....	....
A	5251	hCoV-19/Belgium/ULG-10008/2020|EPI_ISL_421187|2020	....	....	....	....	....	....
A	5252	hCoV-19/Belgium/ULG-10009/2020|EPI_ISL_421188|2020	....	....	....	....	....	....

A 5253 hCoV-19/Belgium/ULG-10011/2020|EPI_ISL_421189|2020 ....
A 5254 hCoV-19/USA/WI-UW-42/2020|EPI_ISL_421300|2020-03-1 ....
A 5255 hCoV-19/USA/WI-UW-43/2020|EPI_ISL_421301|2020-03-1 ....
A 5256 hCoV-19/Singapore/42/2020|EPI_ISL_422435|2020-03-3 ....
A 5257 hCoV-19/Italy/VEN-IZSVe-21-26/2020|EPI_ISL_422437| ....
A 5258 hCoV-19/Italy/VEN-IZSVe-21-37/2020|EPI_ISL_422438| ....
A 5259 hCoV-19/USA/WI-GMF-00498/2020|EPI_ISL_422453|2020- ....
A 5260 hCoV-19/USA/WI-GMF-00557/2020|EPI_ISL_422459|2020- ....
A 5261 hCoV-19/USA/WI-GMF-00707/2020|EPI_ISL_422461|2020- ....
A 5262 hCoV-19/USA/WI-GMF-00744/2020|EPI_ISL_422462|2020- ....
A 5263 hCoV-19/USA/WI-GMF-00588/2020|EPI_ISL_422463|2020- ....
A 5264 hCoV-19/USA/WI-GMF-00441/2020|EPI_ISL_422465|2020- ....
A 5265 hCoV-19/USA/NY-PV09045/2020|EPI_ISL_422488|2020-03 ....
A 5266 hCoV-19/USA/NY-PV09057/2020|EPI_ISL_422489|2020-03 ....
A 5267 hCoV-19/USA/NY-PV09069/2020|EPI_ISL_422490|2020-03 ....
A 5268 hCoV-19/USA/NY-PV09115/2020|EPI_ISL_422491|2020-03 ....
A 5269 hCoV-19/USA/NY-PV09116/2020|EPI_ISL_422492|2020-03 ....
A 5270 hCoV-19/USA/NY-PV09119/2020|EPI_ISL_422494|2020-03 ....
A 5271 hCoV-19/USA/NY-PV09120/2020|EPI_ISL_422495|2020-03 ....
A 5272 hCoV-19/USA/NY-PV09121/2020|EPI_ISL_422496|2020-03 ....
A 5273 hCoV-19/USA/NY-PV09122/2020|EPI_ISL_422497|2020-03 ....
A 5274 hCoV-19/USA/NY-PV09123/2020|EPI_ISL_422498|2020-03 ....
A 5275 hCoV-19/USA/NY-PV09125/2020|EPI_ISL_422499|2020-03 ....
A 5276 hCoV-19/Netherlands/NA_291/2020|EPI_ISL_422600|202 ....
A 5277 hCoV-19/Netherlands/NA_292/2020|EPI_ISL_422601|202 ....
A 5278 hCoV-19/Netherlands/NA_293/2020|EPI_ISL_422602|202 ....
A 5279 hCoV-19/Netherlands/NA_294/2020|EPI_ISL_422603|202 ....
A 5280 hCoV-19/Netherlands/NA_295/2020|EPI_ISL_422604|202 ....
A 5281 hCoV-19/Netherlands/NA_296/2020|EPI_ISL_422605|202 ....
A 5282 hCoV-19/Netherlands/NA_297/2020|EPI_ISL_422606|202 ....
A 5283 hCoV-19/Netherlands/NA_298/2020|EPI_ISL_422607|202 ....
A 5284 hCoV-19/Netherlands/NA_299/2020|EPI_ISL_422608|202 ....
A 5285 hCoV-19/Netherlands/NA_300/2020|EPI_ISL_422609|202 ....
A 5286 hCoV-19/Netherlands/NA_301/2020|EPI_ISL_422610|202 ....
A 5287 hCoV-19/Netherlands/NA_313/2020|EPI_ISL_422622|202 ....
A 5288 hCoV-19/Netherlands/NA_316/2020|EPI_ISL_422625|202 ....
A 5289 hCoV-19/Netherlands/NA_317/2020|EPI_ISL_422626|202 ....
A 5290 hCoV-19/Netherlands/ZuidHolland_100/2020|EPI_ISL_4 ....A........ ....
A 5291 hCoV-19/Netherlands/ZuidHolland_101/2020|EPI_ISL_4 ....
A 5292 hCoV-19/Netherlands/ZuidHolland_94/2020|EPI_ISL_42 ....
A 5293 hCoV-19/Netherlands/ZuidHolland_95/2020|EPI_ISL_42 ....
A 5294 hCoV-19/Netherlands/ZuidHolland_96/2020|EPI_ISL_42 ....
A 5295 hCoV-19/England/20132031404/2020|EPI_ISL_423769|20 ....
A 5296 hCoV-19/England/20132032504/2020|EPI_ISL_423771|20 ....
A 5297 hCoV-19/England/20132032604/2020|EPI_ISL_423772|20 ....
A 5299 hCoV-19/England/20132032804/2020|EPI_ISL_423774|20 ....
A 5300 hCoV-19/England/20132032904/2020|EPI_ISL_423775|20 ....
A 5302 hCoV-19/England/20132035904/2020|EPI_ISL_423779|20 ....
A 5303 hCoV-19/England/20132036004/2020|EPI_ISL_423780|20 ....
A 5304 hCoV-19/England/20132047304/2020|EPI_ISL_423782|20 ....
A 5306 hCoV-19/England/20132054604/2020|EPI_ISL_423786|20 ....
A 5307 hCoV-19/England/20132054704/2020|EPI_ISL_423787|20 ....
A 5308 hCoV-19/England/20132066404/2020|EPI_ISL_423790|20 ....
A 5310 hCoV-19/England/20132068204/2020|EPI_ISL_423796|20 ....
A 5311 hCoV-19/England/20132068304/2020|EPI_ISL_423797|20 ....
A 5312 hCoV-19/England/20132069004/2020|EPI_ISL_423798|20 ....N...........N.....
A 5313 hCoV-19/England/20132069104/2020|EPI_ISL_423799|20 ....
A 5314 hCoV-19/England/20128002204/2020|EPI_ISL_423923|20 ....
A 5315 hCoV-19/England/20128003004/2020|EPI_ISL_423924|20 ....
A 5316 hCoV-19/England/20128003506/2020|EPI_ISL_423926|20 ....
A 5317 hCoV-19/England/20128003904/2020|EPI_ISL_423927|20 ....
A 5319 hCoV-19/England/20128011804/2020|EPI_ISL_423933|20 ....
A 5320 hCoV-19/England/20128012704/2020|EPI_ISL_423935|20 ....
A 5325 hCoV-19/England/20128041604/2020|EPI_ISL_423963|20 ....
A 5326 hCoV-19/Brazil/RJ-477/2020|EPI_ISL_427300|2020-03- ....
A 5327 hCoV-19/Brazil/RJ-477i/2020|EPI_ISL_427301|2020-03 ....
A 5328 hCoV-19/Brazil/RJ-763/2020|EPI_ISL_427302|2020-03- ....
A 5329 hCoV-19/Brazil/RJ-818/2020|EPI_ISL_427303|2020-03- ....
A 5330 hCoV-19/Brazil/RJ-872/2020|EPI_ISL_427304|2020-03- ....
A 5331 hCoV-19/Brazil/SC-766/2020|EPI_ISL_427305|2020-03- ....
A 5332 hCoV-19/Brazil/SC-769/2020|EPI_ISL_427306|2020-03- ....
A 5333 hCoV-19/Russia/StPetersburg-RII4144S/2020|EPI_ISL_ ....
A 5334 hCoV-19/Russia/StPetersburg-RII4307V/2020|EPI_ISL_ ....
A 5335 hCoV-19/Russia/StPetersburg-RII4308V/2020|EPI_ISL_ ....
A 5336 hCoV-19/Russia/StPetersburg-RII4382V/2020|EPI_ISL_ ....
A 5337 hCoV-19/Russia/StPetersburg-RII4384V/2020|EPI_ISL_ ....
A 5338 hCoV-19/Russia/StPetersburg-RII4386V/2020|EPI_ISL_ ....
A 5339 hCoV-19/Russia/StPetersburg-RII4532V/2020|EPI_ISL_ ....

A 5340 hCoV-19/Russia/StPetersburg-RII4546V/2020|EPI_ISL_ ....
A 5341 hCoV-19/Russia/Ulan-Ude-RII4560S/2020|EPI_ISL_4273 ....
A 5342 hCoV-19/Russia/StPetersburg-RII4584S/2020|EPI_ISL_ ....
A 5343 hCoV-19/Russia/StPetersburg-RII4585S/2020|EPI_ISL_ ....
A 5344 hCoV-19/Russia/StPetersburg-RII4591S/2020|EPI_ISL_ ....
A 5345 hCoV-19/Russia/StPetersburg-RII4597S/2020|EPI_ISL_ ....
A 5346 hCoV-19/Russia/StPetersburg-RII4637S/2020|EPI_ISL_ ....
A 5347 hCoV-19/Russia/StPetersburg-RII4649S/2020|EPI_ISL_ ....
A 5348 hCoV-19/Russia/StPetersburg-RII4655S/2020|EPI_ISL_ ....
A 5349 hCoV-19/Russia/StPetersburg-RII4678S/2020|EPI_ISL_ ....
A 5350 hCoV-19/Russia/StPetersburg-RII4686S/2020|EPI_ISL_ ....
A 5351 hCoV-19/Russia/StPetersburg-RII4693S/2020|EPI_ISL_ ....
A 5352 hCoV-19/Russia/StPetersburg-RII4694S/2020|EPI_ISL_ ....
A 5353 hCoV-19/Russia/StPetersburg-RII4696S/2020|EPI_ISL_ ....
A 5354 hCoV-19/Russia/StPetersburg-RII4697S/2020|EPI_ISL_ ....
A 5355 hCoV-19/Russia/StPetersburg-RII4707S/2020|EPI_ISL_ ....
A 5356 hCoV-19/France/HDF-1463/2020|EPI_ISL_429968|2020-0 ....
A 5357 hCoV-19/USA/VA-DCLS-0069/2020|EPI_ISL_429969|2020- ....
A 5358 hCoV-19/USA/VA-DCLS-0070/2020|EPI_ISL_429970|2020- ....
A 5359 hCoV-19/USA/VA-DCLS-0071/2020|EPI_ISL_429971|2020- ....
A 5360 hCoV-19/USA/VA-DCLS-0072/2020|EPI_ISL_429972|2020- ....
A 5361 hCoV-19/USA/VA-DCLS-0073/2020|EPI_ISL_429973|2020- ....
A 5362 hCoV-19/USA/VA-DCLS-0075/2020|EPI_ISL_429974|2020- ....
A 5363 hCoV-19/USA/VA-DCLS-0076/2020|EPI_ISL_429975|2020- ....
A 5364 hCoV-19/USA/VA-DCLS-0077/2020|EPI_ISL_429976|2020- ....
A 5365 hCoV-19/USA/VA-DCLS-0078/2020|EPI_ISL_429977|2020- ....
A 5366 hCoV-19/USA/VA-DCLS-0079/2020|EPI_ISL_429978|2020- ....
A 5367 hCoV-19/USA/VA-DCLS-0080/2020|EPI_ISL_429979|2020- ....
A 5368 hCoV-19/USA/VA-DCLS-0081/2020|EPI_ISL_429980|2020- ....
A 5369 hCoV-19/USA/VA-DCLS-0084/2020|EPI_ISL_429982|2020- ....
A 5370 hCoV-19/USA/VA-DCLS-0086/2020|EPI_ISL_429983|2020- ....
A 5371 hCoV-19/USA/VA-DCLS-0087/2020|EPI_ISL_429984|2020- ....
A 5372 hCoV-19/USA/VA-DCLS-0088/2020|EPI_ISL_429985|2020- ....
A 5373 hCoV-19/USA/VA-DCLS-0089/2020|EPI_ISL_429986|2020- ....
A 5374 hCoV-19/USA/VA-DCLS-0090/2020|EPI_ISL_429987|2020- ....
A 5375 hCoV-19/USA/VA-DCLS-0091/2020|EPI_ISL_429988|2020- ....
A 5376 hCoV-19/USA/VA-DCLS-0092/2020|EPI_ISL_429989|2020- ....
A 5377 hCoV-19/Cambodia/0012/2020|EPI_ISL_411902|2020-01- ....
A 5378 hCoV-19/Taiwan/CGMH-CGU-01/2020|EPI_ISL_411915|202 ....
A 5379 hCoV-19/Taiwan/3/2020|EPI_ISL_411926|2020-01-24 ....
A 5380 hCoV-19/Taiwan/4/2020|EPI_ISL_411927|2020-01-28 ....
A 5381 hCoV-19/Netherlands/NA_14/2020|EPI_ISL_415470|2020 ....
A 5382 hCoV-19/Netherlands/NA_15/2020|EPI_ISL_415471|2020 ....
A 5383 hCoV-19/Netherlands/NA_16/2020|EPI_ISL_415472|2020 ....
A 5384 hCoV-19/Netherlands/NA_17/2020|EPI_ISL_415473|2020 ....
A 5385 hCoV-19/Netherlands/NA_18/2020|EPI_ISL_415474|2020 ....
A 5386 hCoV-19/Netherlands/NA_19/2020|EPI_ISL_415475|2020 ....
A 5387 hCoV-19/Netherlands/NA_2/2020|EPI_ISL_415476|2020- ....
A 5388 hCoV-19/Netherlands/NA_21/2020|EPI_ISL_415478|2020 ....
A 5389 hCoV-19/Netherlands/NA_22/2020|EPI_ISL_415479|2020 ....
A 5390 hCoV-19/Netherlands/NA_23/2020|EPI_ISL_415480|2020 ....
A 5391 hCoV-19/Netherlands/NA_24/2020|EPI_ISL_415481|2020 ....
A 5392 hCoV-19/Netherlands/NA_25/2020|EPI_ISL_415482|2020 ....
A 5393 hCoV-19/Netherlands/NA_26/2020|EPI_ISL_415483|2020 ....
A 5394 hCoV-19/Netherlands/NA_27/2020|EPI_ISL_415484|2020 ....
A 5395 hCoV-19/Netherlands/NA_28/2020|EPI_ISL_415485|2020 ....
A 5396 hCoV-19/Netherlands/NA_29/2020|EPI_ISL_415486|2020 ....
A 5397 hCoV-19/Netherlands/NA_30/2020|EPI_ISL_415487|2020 ....
A 5398 hCoV-19/Netherlands/NA_31/2020|EPI_ISL_415488|2020 ....
A 5399 hCoV-19/Netherlands/NA_32/2020|EPI_ISL_415489|2020 ....
A 5400 hCoV-19/Netherlands/NA_34/2020|EPI_ISL_415491|2020 ....
A 5401 hCoV-19/Netherlands/NA_35/2020|EPI_ISL_415492|2020 ....
A 5402 hCoV-19/Netherlands/NA_4/2020|EPI_ISL_415493|2020 ....
A 5403 hCoV-19/Netherlands/NA_5/2020|EPI_ISL_415494|2020 ....
A 5404 hCoV-19/Netherlands/NA_6/2020|EPI_ISL_415495|2020- ....
A 5405 hCoV-19/Netherlands/NA_7/2020|EPI_ISL_415496|2020- ....
A 5406 hCoV-19/Netherlands/NA_8/2020|EPI_ISL_415497|2020- ....
A 5407 hCoV-19/Netherlands/NA_9/2020|EPI_ISL_415498|2020- ....
A 5408 hCoV-19/Netherlands/NoordBrabant_41/2020|EPI_ISL_4 ....
A 5409 hCoV-19/Singapore/21/2020|EPI_ISL_419000|2020-02-1 ....
A 5410 hCoV-19/Singapore/19/2020|EPI_ISL_419001|2020-03-0 ....
A 5411 hCoV-19/Australia/VIC309/2020|EPI_ISL_420000|2020- ....
A 5412 hCoV-19/Australia/VIC310/2020|EPI_ISL_420001|2020- ....
A 5413 hCoV-19/Australia/VIC311/2020|EPI_ISL_420002|2020- ....
A 5414 hCoV-19/Australia/VIC312/2020|EPI_ISL_420003|2020- ....
A 5415 hCoV-19/Australia/VIC313/2020|EPI_ISL_420004|2020- ....
A 5416 hCoV-19/Australia/VIC314/2020|EPI_ISL_420005|2020- ....
A 5417 hCoV-19/Australia/VIC315/2020|EPI_ISL_420006|2020- ....

A	5418	hCoV-19/Australia/VIC316/2020|EPI_ISL_420007|2020-	····················	····················	····················	····················	····················	····················
A	5419	hCoV-19/Australia/VIC317/2020|EPI_ISL_420008|2020-	····················	····················	····················	····················	····················	····················
A	5420	hCoV-19/Australia/VIC318/2020|EPI_ISL_420009|2020-	····················	····················	····················	····················	····················	····················
A	5421	hCoV-19/Australia/VIC319/2020|EPI_ISL_420010|2020-	····················	····················	····················	····················	····················	····················
A	5422	hCoV-19/Australia/VIC320/2020|EPI_ISL_420011|2020-	····················	····················	····················	····················	····················	····················
A	5423	hCoV-19/Australia/VIC321/2020|EPI_ISL_420012|2020-	····················	····················	····················	····················	····················	····················
A	5424	hCoV-19/Australia/VIC322/2020|EPI_ISL_420013|2020-	····················	····················	····················	····················	····················	····················
A	5425	hCoV-19/Australia/VIC323/2020|EPI_ISL_420014|2020-	····················	····················	····················	····················	····················	····················
A	5426	hCoV-19/USA/WI-UW-44/2020|EPI_ISL_421302|2020-03-1	····················	····················	····················	····················	····················	····················
A	5427	hCoV-19/USA/WI-UW-45/2020|EPI_ISL_421303|2020-03-2	····················	····················	····················	····················	····················	····················
A	5428	hCoV-19/USA/WI-UW-46/2020|EPI_ISL_421304|2020-03-2	····················	····················	····················	····················	····················	····················
A	5429	hCoV-19/USA/WI-UW-47/2020|EPI_ISL_421305|2020-03-1	····················	····················	····················	····················	····················	····················
A	5430	hCoV-19/USA/WI-UW-48/2020|EPI_ISL_421306|2020-03-2	····················	····················	····················	····················	····················	····················
A	5431	hCoV-19/USA/WI-UW-49/2020|EPI_ISL_421307|2020-03-2	····················	····················	····················	····················	····················	····················
A	5432	hCoV-19/USA/WI-UW-50/2020|EPI_ISL_421308|2020-03-2	····················	····················	····················	····················	····················	····················
A	5433	hCoV-19/USA/WI-UW-51/2020|EPI_ISL_421309|2020-03-2	····················	····················	····················	····················	····················	····················
A	5434	hCoV-19/USA/WI-UW-52/2020|EPI_ISL_421310|2020-03-1	····················	····················	····················	····················	····················	····················
A	5435	hCoV-19/USA/WI-UW-53/2020|EPI_ISL_421311|2020-03-1	····················	····················	····················	····················	····················	····················
A	5436	hCoV-19/USA/WI-UW-54/2020|EPI_ISL_421312|2020-03-2	····················	····················	····················	····················	····················	····················
A	5437	hCoV-19/USA/WI-UW-55/2020|EPI_ISL_421313|2020-03-2	····················	····················	····················	····················	····················	····················
A	5438	hCoV-19/USA/WI-UW-56/2020|EPI_ISL_421314|2020-03-1	····················	····················	····················	····················	····················	····················
A	5439	hCoV-19/USA/WI-UW-57/2020|EPI_ISL_421315|2020-03-1	····················	····················	····················	····················	····················	····················
A	5440	hCoV-19/USA/WI-UW-58/2020|EPI_ISL_421316|2020-03-2	····················	····················	····················	····················	····················	····················
A	5441	hCoV-19/USA/WI-UW-59/2020|EPI_ISL_421317|2020-03-2	····················	····················	····················	····················	····················	····················
A	5442	hCoV-19/USA/WI-UW-60/2020|EPI_ISL_421318|2020-03-1	····················	····················	····················	····················	····················	····················
A	5443	hCoV-19/USA/WI-UW-61/2020|EPI_ISL_421319|2020-03-2	····················	····················	····················	····················	····················	····················
A	5444	hCoV-19/USA/WI-UW-62/2020|EPI_ISL_421320|2020-03-2	····················	····················	····················	····················	····················	····················
A	5445	hCoV-19/USA/WI-UW-63/2020|EPI_ISL_421321|2020-03-2	····················	····················	····················	····················	····················	····················
A	5446	hCoV-19/USA/WI-UW-64/2020|EPI_ISL_421322|2020-03-2	····················	····················	····················	····················	····················	····················
A	5447	hCoV-19/USA/WI-UW-65/2020|EPI_ISL_421323|2020-03-2	····················	····················	····················	····················	····················	····················
A	5448	hCoV-19/USA/WI-UW-66/2020|EPI_ISL_421324|2020-03-2	····················	····················	····················	····················	····················	N···················
A	5449	hCoV-19/USA/WI-UW-67/2020|EPI_ISL_421325|2020-03-2	····················	····················	····················	····················	····················	····················
A	5450	hCoV-19/USA/WI-UW-68/2020|EPI_ISL_421326|2020-03-2	····················	····················	····················	····················	····················	····················
A	5451	hCoV-19/USA/WI-UW-69/2020|EPI_ISL_421327|2020-03-1	····················	····················	····················	····················	····················	····················
A	5452	hCoV-19/USA/WI-UW-70/2020|EPI_ISL_421328|2020-03-1	····················	····················	····················	····················	····················	····················
A	5453	hCoV-19/USA/WI-UW-71/2020|EPI_ISL_421329|2020-03-2	····················	····················	····················	····················	····················	····················
A	5454	hCoV-19/USA/WI-UW-72/2020|EPI_ISL_421330|2020-03-2	····················	····················	····················	····················	····················	····················
A	5455	hCoV-19/USA/WI-UW-73/2020|EPI_ISL_421331|2020-03-2	····················	····················	····················	····················	····················	····················
A	5456	hCoV-19/USA/WI-UW-74/2020|EPI_ISL_421332|2020-03-2	····················	····················	····················	····················	····················	····················
A	5457	hCoV-19/USA/WI-UW-75/2020|EPI_ISL_421333|2020-03-2	····················	····················	····················	····················	····················	····················
A	5458	hCoV-19/USA/WI-UW-76/2020|EPI_ISL_421334|2020-03-2	····················	····················	····················	····················	····················	····················
A	5459	hCoV-19/USA/WI-UW-77/2020|EPI_ISL_421335|2020-03-1	····················	····················	····················	····················	····················	····················
A	5460	hCoV-19/USA/WI-UW-78/2020|EPI_ISL_421336|2020-03-2	····················	····················	····················	····················	····················	····················
A	5461	hCoV-19/USA/WI-UW-79/2020|EPI_ISL_421338|2020-03-2	····················	····················	····················	····················	····················	····················
A	5462	hCoV-19/USA/WI-UW-80/2020|EPI_ISL_421339|2020-03-2	····················	····················	····················	····················	····················	····················
A	5463	hCoV-19/USA/WI-UW-81/2020|EPI_ISL_421340|2020-03-2	····················	····················	····················	····················	····················	····················
A	5464	hCoV-19/USA/WI-UW-82/2020|EPI_ISL_421341|2020-03-2	····················	····················	····················	····················	····················	····················
A	5465	hCoV-19/USA/WI-UW-83/2020|EPI_ISL_421342|2020-03-2	····················	····················	····················	····················	····················	····················
A	5466	hCoV-19/USA/WI-UW-84/2020|EPI_ISL_421343|2020-03-2	····················	····················	····················	····················	····················	····················
A	5467	hCoV-19/Netherlands/NA_302/2020|EPI_ISL_422611|202	····················	····················	····················	····················	····················	····················
A	5468	hCoV-19/Netherlands/NA_303/2020|EPI_ISL_422612|202	····················	··········A·········	····················	····················	····················	····················
A	5469	hCoV-19/Netherlands/NA_306/2020|EPI_ISL_422615|202	····················	····················	····················	····················	····················	····················
A	5470	hCoV-19/Netherlands/NA_307/2020|EPI_ISL_422616|202	····················	····················	····················	····················	····················	····················
A	5471	hCoV-19/Netherlands/NA_311/2020|EPI_ISL_422620|202	····················	····················	····················	····················	····················	····················
A	5472	hCoV-19/Netherlands/NA_312/2020|EPI_ISL_422621|202	····················	····················	····················	····················	····················	····················
A	5473	hCoV-19/Netherlands/NA_314/2020|EPI_ISL_422623|202	····················	····················	····················	····················	····················	····················
A	5474	hCoV-19/Netherlands/ZuidHolland_97/2020|EPI_ISL_42	····················	····················	····················	····················	····················	····················
A	5475	hCoV-19/Netherlands/ZuidHolland_98/2020|EPI_ISL_42	····················	····················	····················	····················	····················	····················
A	5476	hCoV-19/Netherlands/ZuidHolland_99/2020|EPI_ISL_42	····················	····················	····················	····················	····················	····················
A	5477	hCoV-19/Czech Republic/2741/2020|EPI_ISL_422636|20	····················	····················	····················	····················	····················	····················
A	5478	hCoV-19/Netherlands/Gelderland_10/2020|EPI_ISL_422	····················	····················	····················	····················	····················	····················
A	5479	hCoV-19/Netherlands/Gelderland_11/2020|EPI_ISL_422	····················	····················	····················	····················	····················	····················
A	5480	hCoV-19/Netherlands/Gelderland_12/2020|EPI_ISL_422	····················	····················	····················	····················	····················	····················
A	5481	hCoV-19/Netherlands/Gelderland_5/2020|EPI_ISL_4226	····················	····················	····················	····················	····················	····················
A	5482	hCoV-19/Netherlands/Gelderland_6/2020|EPI_ISL_4226	····················	····················	····················	····················	····················	····················
A	5483	hCoV-19/Netherlands/Gelderland_7/2020|EPI_ISL_4226	····················	····················	····················	····················	····················	····················
A	5484	hCoV-19/Netherlands/Gelderland_8/2020|EPI_ISL_4226	····················	····················	····················	····················	····················	····················
A	5485	hCoV-19/Netherlands/Gelderland_9/2020|EPI_ISL_4226	····················	····················	····················	····················	····················	····················
A	5486	hCoV-19/Netherlands/Limburg_10/2020|EPI_ISL_422646	····················	····················	····················	····················	····················	····················
A	5487	hCoV-19/Netherlands/Limburg_8/2020|EPI_ISL_422647|	····················	····················	····················	····················	····················	····················
A	5488	hCoV-19/Netherlands/Limburg_9/2020|EPI_ISL_422648|	····················	····················	····················	····················	····················	····················
A	5489	hCoV-19/Netherlands/NA_100/2020|EPI_ISL_422649|202	····················	····················	····················	····················	····················	····················
A	5490	hCoV-19/Netherlands/NA_101/2020|EPI_ISL_422650|202	····················	····················	····················	····················	····················	····················
A	5491	hCoV-19/Netherlands/NA_102/2020|EPI_ISL_422651|202	····················	····················	····················	····················	····················	····················
A	5492	hCoV-19/Netherlands/NA_103/2020|EPI_ISL_422652|202	····················	····················	····················	····················	····················	····················
A	5493	hCoV-19/Netherlands/NA_104/2020|EPI_ISL_422653|202	····················	····················	····················	····················	····················	····················
A	5494	hCoV-19/Netherlands/NA_106/2020|EPI_ISL_422655|202	····················	····················	····················	····················	····················	····················
A	5495	hCoV-19/Netherlands/NA_107/2020|EPI_ISL_422656|202	····················	····················	····················	····················	····················	····················

A	5496 hCoV-19/Netherlands/NA_109/2020|EPI_ISL_422657|202	····················	····················	····················	····················	····················	····················
A	5497 hCoV-19/Netherlands/NA_111/2020|EPI_ISL_422659|202	····················	····················	····················	····················	····················	····················
A	5498 hCoV-19/Netherlands/NA_112/2020|EPI_ISL_422660|202	····················	····················	····················	····················	····················	····················
A	5499 hCoV-19/Netherlands/NA_113/2020|EPI_ISL_422661|202	····················	····················	····················	····················	····················	····················
A	5500 hCoV-19/Netherlands/NA_114/2020|EPI_ISL_422662|202	····················	····················	····················	····················	····················	····················
A	5501 hCoV-19/Netherlands/NA_117/2020|EPI_ISL_422664|202	····················	····················	····················	····················	···T················	····················
A	5502 hCoV-19/Netherlands/NA_118/2020|EPI_ISL_422665|202	····················	····················	····················	····················	···T················	····················
A	5503 hCoV-19/Netherlands/NA_119/2020|EPI_ISL_422666|202	····················	····················	····················	····················	····················	····················
A	5504 hCoV-19/Netherlands/NA_120/2020|EPI_ISL_422667|202	····················	····················	····················	····················	····················	····················
A	5505 hCoV-19/Netherlands/NA_121/2020|EPI_ISL_422668|202	····················	····················	····················	····················	····················	····················
A	5506 hCoV-19/Netherlands/NA_122/2020|EPI_ISL_422669|202	····················	····················	····················	····················	····················	····················
A	5507 hCoV-19/Netherlands/NA_126/2020|EPI_ISL_422670|202	····················	····················	····················	····················	····················	····················
A	5508 hCoV-19/Netherlands/NA_127/2020|EPI_ISL_422671|202	····················	····················	····················	····················	····················	····················
A	5509 hCoV-19/Netherlands/NA_128/2020|EPI_ISL_422672|202	····················	····················	····················	····················	····················	····················
A	5510 hCoV-19/Netherlands/NA_129/2020|EPI_ISL_422673|202	····················	····················	····················	····················	····················	····················
A	5511 hCoV-19/Netherlands/NA_130/2020|EPI_ISL_422674|202	····················	····················	····················	····················	····················	····················
A	5512 hCoV-19/Netherlands/NA_131/2020|EPI_ISL_422675|202	····················	····················	····················	····················	····················	····················
A	5513 hCoV-19/Netherlands/NA_132/2020|EPI_ISL_422676|202	····················	····················	····················	····················	····················	····················
A	5514 hCoV-19/England/20126097804/2020|EPI_ISL_423906|20	····················	····················	····················	····················	····················	····················
A	5515 hCoV-19/England/20126100404/2020|EPI_ISL_423907|20	····················	····················	····················	····················	····················	····················
A	5517 hCoV-19/England/20126103304/2020|EPI_ISL_423911|20	····················	····················	····················	····················	····················	····················
A	5518 hCoV-19/England/20128000604/2020|EPI_ISL_423918|20	····················	····················	····················	····················	····················	····················
A	5519 hCoV-19/England/20128015104/2020|EPI_ISL_423942|20	····················	····················	····················	····················	····················	····················
A	5520 hCoV-19/England/20128015604/2020|EPI_ISL_423943|20	····················	····················	····················	····················	····················	····················
A	5521 hCoV-19/England/20128018804/2020|EPI_ISL_423948|20	····················	····················	····················	····················	····················	····················
A	5523 hCoV-19/England/20128036004/2020|EPI_ISL_423952|20	····················	····················	····················	····················	····················	····················
A	5524 hCoV-19/England/20128085204/2020|EPI_ISL_423964|20	····················	····················	····················	····················	····················	····················
A	5525 hCoV-19/England/20128090404/2020|EPI_ISL_423965|20	····················	····················	····················	····················	····················	····················
A	5527 hCoV-19/England/20128094904/2020|EPI_ISL_423969|20	····················	····················	····················	····················	····················	····················
A	5529 hCoV-19/England/20129000404/2020|EPI_ISL_423973|20	····················	····················	····················	····················	····················	····················
A	5530 hCoV-19/England/20129002004/2020|EPI_ISL_423976|20	····················	····················	····················	····················	····················	····················
A	5531 hCoV-19/England/20129009304/2020|EPI_ISL_423979|20	····················	····················	····················	····················	····················	····················
A	5532 hCoV-19/England/20129012304/2020|EPI_ISL_423990|20	····················	····················	····················	····················	····················	····················
A	5533 hCoV-19/England/20129015404/2020|EPI_ISL_423998|20	····················	····················	····················	····················	····················	····················
A	5534 hCoV-19/England/20129015704/2020|EPI_ISL_423999|20	····················	····················	····················	····················	····················	····················
A	5535 hCoV-19/USA/WA-UW-2230/2020|EPI_ISL_426120|2020-03	····················	····················	····················	····················	····················	····················
A	5536 hCoV-19/USA/WA-UW-2232/2020|EPI_ISL_426121|2020-03	····················	····················	····················	····················	····················	····················
A	5537 hCoV-19/USA/WA-UW-2234/2020|EPI_ISL_426122|2020-03	····················	····················	····················	····················	····················	····················
A	5538 hCoV-19/USA/WA-UW-2235/2020|EPI_ISL_426123|2020-03	····················	····················	····················	····················	····················	····················
A	5539 hCoV-19/USA/WA-UW-2236/2020|EPI_ISL_426124|2020-03	····················	····················	····················	····················	····················	····················
A	5540 hCoV-19/USA/WA-UW-2238/2020|EPI_ISL_426125|2020-03	····················	····················	····················	····················	····················	····················
A	5541 hCoV-19/USA/WA-UW-2240/2020|EPI_ISL_426126|2020-03	····················	····················	····················	····················	····················	····················
A	5542 hCoV-19/USA/WA-UW-2244/2020|EPI_ISL_426127|2020-03	····················	····················	····················	····················	····················	····················
A	5543 hCoV-19/USA/WA-UW-2245/2020|EPI_ISL_426128|2020-03	····················	····················	····················	····················	····················	····················
A	5544 hCoV-19/USA/WA-UW-2247/2020|EPI_ISL_426129|2020-03	····················	····················	····················	····················	····················	····················
A	5545 hCoV-19/USA/WA-UW-2249/2020|EPI_ISL_426130|2020-03	····················	····················	····················	····················	····················	····················
A	5546 hCoV-19/USA/WA-UW-2250/2020|EPI_ISL_426131|2020-03	····················	····················	····················	····················	····················	····················
A	5547 hCoV-19/USA/WA-UW-2253/2020|EPI_ISL_426132|2020-03	····················	····················	····················	····················	····················	····················
A	5548 hCoV-19/USA/WA-UW-2258/2020|EPI_ISL_426133|2020-03	····················	····················	····················	····················	····················	····················
A	5549 hCoV-19/USA/WA-UW-4404/2020|EPI_ISL_426135|2020-03	····················	····················	····················	····················	····················	····················
A	5550 hCoV-19/USA/WA-UW-4406/2020|EPI_ISL_426136|2020-03	····················	····················	····················	····················	····················	····················
A	5551 hCoV-19/USA/WA-UW-4407/2020|EPI_ISL_426137|2020-03	····················	····················	····················	····················	····················	····················
A	5552 hCoV-19/USA/WI-GMF-00986/2020|EPI_ISL_426159|2020-	····················	····················	····················	····················	····················	····················
A	5553 hCoV-19/USA/WI-GMF-00921/2020|EPI_ISL_426160|2020-	····················	····················	····················	····················	····················	····················
A	5554 hCoV-19/USA/WI-GMF-00928/2020|EPI_ISL_426161|2020-	····················	····················	····················	····················	····················	····················
A	5555 hCoV-19/South Korea/KCDC2003/2020|EPI_ISL_426163|2	····················	····················	····················	····················	····················	····················
A	5556 hCoV-19/South Korea/KCDC2004/2020|EPI_ISL_426164|2	····················	····················	····················	····················	····················	····················
A	5557 hCoV-19/South Korea/KCDC2005/2020|EPI_ISL_426166|2	····················	····················	····················	····················	····················	····················
A	5558 hCoV-19/South Korea/KCDC2003/2020|EPI_ISL_426167|2	····················	····················	····················	····················	····················	····················
A	5559 hCoV-19/South Korea/KCDC2006/2020|EPI_ISL_426168|2	····················	····················	····················	····················	····················	····················
A	5560 hCoV-19/South Korea/KCDC2007/2020|EPI_ISL_426169|2	····················	····················	····················	····················	····················	····················
A	5561 hCoV-19/South Korea/KCDC2008/2020|EPI_ISL_426171|2	····················	····················	····················	····················	····················	····················
A	5562 hCoV-19/South Korea/KCDC2009/2020|EPI_ISL_426173|2	····················	····················	····················	····················	····················	····················
A	5563 hCoV-19/South Korea/KCDC2010/2020|EPI_ISL_426180|2	····················	····················	····················	····················	····················	····················
A	5564 hCoV-19/South Korea/KCDC2011/2020|EPI_ISL_426181|2	····················	····················	····················	····················	····················	····················
A	5565 hCoV-19/South Korea/KCDC2012/2020|EPI_ISL_426182|2	····················	····················	····················	····················	····················	····················
A	5566 hCoV-19/South Korea/KCDC2013/2020|EPI_ISL_426183|2	····················	····················	····················	····················	····················	····················
A	5567 hCoV-19/South Korea/KCDC2014/2020|EPI_ISL_426187|2	····················	····················	····················	····················	····················	····················
A	5568 hCoV-19/USA/IL-UW-143/2020|EPI_ISL_427450|2020-04-	····················	····················	····················	····················	····················	····················
A	5569 hCoV-19/USA/WI-UW-144/2020|EPI_ISL_427451|2020-04-	····················	····················	····················	····················	····················	····················
A	5570 hCoV-19/USA/WI-UW-145/2020|EPI_ISL_427452|2020-04-	····················	····················	····················	····················	····················	····················
A	5571 hCoV-19/USA/WI-UW-146/2020|EPI_ISL_427453|2020-04-	····················	····················	····················	····················	····················	····················
A	5572 hCoV-19/USA/WI-UW-147/2020|EPI_ISL_427454|2020-04-	····················	····················	····················	····················	····················	····················
A	5573 hCoV-19/USA/WI-UW-148/2020|EPI_ISL_427455|2020-04-	····················	····················	····················	····················	····················	····················
A	5574 hCoV-19/USA/IL-UW-149/2020|EPI_ISL_427456|2020-04-	····················	····················	····················	····················	····················	····················
A	5575 hCoV-19/USA/WI-UW-150/2020|EPI_ISL_427457|2020-04-	····················	····················	····················	····················	····················	····················
A	5576 hCoV-19/USA/WI-UW-151/2020|EPI_ISL_427458|2020-04-	····················	····················	····················	····················	····················	····················
A	5577 hCoV-19/USA/WI-UW-152/2020|EPI_ISL_427459|2020-04-	····················	····················	····················	····················	····················	····················

A	5578 hCoV-19/USA/WI-UW-153/2020|EPI_ISL_427460|2020-04-	····················	····················	····················	····················	····················	····················
A	5579 hCoV-19/USA/WI-UW-154/2020|EPI_ISL_427461|2020-04-	····················	····················	····················	····················	····················	····················
A	5580 hCoV-19/USA/WI-UW-155/2020|EPI_ISL_427462|2020-04-	····················	····················	····················	····················	····················	····················
A	5581 hCoV-19/USA/NY-NYUMC172/2020|EPI_ISL_427469|2020-0	····················	····················	····················	····················	····················	····················
A	5582 hCoV-19/USA/NY-NYUMC173/2020|EPI_ISL_427470|2020-0	····················	····················	····················	····················	····················	····················
A	5583 hCoV-19/USA/NY-NYUMC174/2020|EPI_ISL_427471|2020-0	····················	····················	····················	····················	····················	····················
A	5584 hCoV-19/USA/NY-NYUMC175/2020|EPI_ISL_427472|2020-0	····················	····················	····················	····················	····················	····················
A	5585 hCoV-19/USA/NY-NYUMC177/2020|EPI_ISL_427473|2020-0	····················	····················	····················	····················	····················	····················
A	5586 hCoV-19/USA/NY-NYUMC178/2020|EPI_ISL_427474|2020-0	····················	····················	····················	····················	····················	····················
A	5587 hCoV-19/USA/NY-NYUMC179/2020|EPI_ISL_427475|2020-0	····················	····················	····················	····················	····················	····················
A	5588 hCoV-19/USA/NY-NYUMC180/2020|EPI_ISL_427476|2020-0	····················	····················	····················	····················	····················	····················
A	5589 hCoV-19/USA/NY-NYUMC181/2020|EPI_ISL_427477|2020-0	····················	····················	····················	····················	····················	····················
A	5590 hCoV-19/USA/NY-NYUMC182/2020|EPI_ISL_427478|2020-0	····················	····················	····················	····················	····················	····················
A	5591 hCoV-19/USA/NY-NYUMC183/2020|EPI_ISL_427479|2020-0	····················	····················	····················	····················	····················	····················
A	5592 hCoV-19/USA/NY-NYUMC184/2020|EPI_ISL_427480|2020-0	····················	····················	····················	····················	····················	····················
A	5593 hCoV-19/USA/NY-NYUMC185/2020|EPI_ISL_427481|2020-0	····················	····················	····················	····················	····················	····················
A	5594 hCoV-19/USA/NY-NYUMC186/2020|EPI_ISL_427482|2020-0	····················	····················	····················	····················	····················	······G·············
A	5595 hCoV-19/USA/NY-NYUMC188/2020|EPI_ISL_427484|2020-0	····················	····················	····················	····················	····················	····················
A	5596 hCoV-19/USA/NY-NYUMC189/2020|EPI_ISL_427485|2020-0	····················	····················	····················	····················	····················	····················
A	5597 hCoV-19/USA/NY-NYUMC192/2020|EPI_ISL_427487|2020-0	····················	····················	····················	····················	····················	····················
A	5598 hCoV-19/USA/NY-NYUMC193/2020|EPI_ISL_427488|2020-0	····················	····················	····················	····················	····················	····················
A	5599 hCoV-19/USA/NY-NYUMC194/2020|EPI_ISL_427489|2020-0	····················	····················	····················	····················	····················	····················
A	5600 hCoV-19/USA/NY-NYUMC195/2020|EPI_ISL_427490|2020-0	····················	····················	····················	····················	····················	····················
A	5601 hCoV-19/USA/NY-NYUMC196/2020|EPI_ISL_427491|2020-0	····················	····················	····················	····················	····················	····················
A	5602 hCoV-19/USA/NY-NYUMC197/2020|EPI_ISL_427492|2020-0	····················	····················	····················	····················	····················	····················
A	5603 hCoV-19/USA/NY-NYUMC198/2020|EPI_ISL_427493|2020-0	····················	····················	····················	····················	····················	····················
A	5604 hCoV-19/USA/NY-NYUMC199/2020|EPI_ISL_427494|2020-0	····················	····················	····················	····················	····················	····················
A	5605 hCoV-19/USA/NY-NYUMC200/2020|EPI_ISL_427495|2020-0	····················	····················	····················	····················	····················	····················
A	5606 hCoV-19/USA/NY-NYUMC201/2020|EPI_ISL_427496|2020-0	····················	····················	····················	····················	····················	····················
A	5607 hCoV-19/USA/NY-NYUMC203/2020|EPI_ISL_427498|2020-0	····················	····················	····················	····················	····················	····················
A	5608 hCoV-19/USA/NY-NYUMC266/2020|EPI_ISL_428780|2020-0	····················	····················	····················	····················	····················	····················
A	5609 hCoV-19/USA/NY-NYUMC267/2020|EPI_ISL_428781|2020-0	····················	····················	····················	····················	····················	····················
A	5610 hCoV-19/USA/NY-NYUMC268/2020|EPI_ISL_428782|2020-0	····················	····················	····················	····················	····················	····················
A	5611 hCoV-19/USA/NY-NYUMC269/2020|EPI_ISL_428783|2020-0	····················	····················	····················	····················	····················	····················
A	5612 hCoV-19/USA/NY-NYUMC270/2020|EPI_ISL_428784|2020-0	····················	····················	····················	····················	····················	····················
A	5613 hCoV-19/USA/NY-NYUMC271/2020|EPI_ISL_428785|2020-0	····················	····················	····················	····················	····················	····················
A	5614 hCoV-19/USA/NY-NYUMC272/2020|EPI_ISL_428786|2020-0	····················	····················	····················	····················	····················	····················
A	5615 hCoV-19/USA/NY-NYUMC273/2020|EPI_ISL_428787|2020-0	····················	····················	····················	····················	····················	····················
A	5616 hCoV-19/USA/NY-NYUMC274/2020|EPI_ISL_428788|2020-0	····················	····················	····················	····················	····················	····················
A	5617 hCoV-19/USA/NY-NYUMC275/2020|EPI_ISL_428789|2020-0	····················	····················	····················	····················	····················	····················
A	5618 hCoV-19/USA/NY-NYUMC276/2020|EPI_ISL_428790|2020-0	····················	····················	····················	····················	····················	····················
A	5619 hCoV-19/USA/NY-NYUMC277/2020|EPI_ISL_428791|2020-0	····················	····················	····················	····················	····················	····················
A	5620 hCoV-19/USA/NY-NYUMC278/2020|EPI_ISL_428792|2020-0	····················	····················	····················	····················	····················	····················
A	5621 hCoV-19/USA/NY-NYUMC279/2020|EPI_ISL_428793|2020-0	····················	····················	····················	····················	····················	····················
A	5622 hCoV-19/USA/NY-NYUMC281/2020|EPI_ISL_428795|2020-0	····················	····················	····················	····················	····················	····················
A	5623 hCoV-19/USA/NY-NYUMC283/2020|EPI_ISL_428797|2020-0	····················	····················	····················	····················	····················	····················
A	5624 hCoV-19/USA/NY-NYUMC284/2020|EPI_ISL_428798|2020-0	····················	····················	····················	····················	····················	····················
A	5625 hCoV-19/USA/NY-NYUMC285/2020|EPI_ISL_428799|2020-0	····················	····················	····················	····················	····················	····················
A	5626 hCoV-19/USA/WA-UW-328/2020|EPI_ISL_524437|2020-03-	····················	····················	····················	····················	····················	····················
A	5627 hCoV-19/USA/WA-UW-334/2020|EPI_ISL_524438|2020-03-	····················	····················	····················	····················	····················	····················
A	5628 hCoV-19/USA/WA-UW-350/2020|EPI_ISL_524439|2020-03-	····················	····················	····················	····················	····················	····················
A	5629 hCoV-19/USA/WA-UW-351/2020|EPI_ISL_524440|2020-03-	····················	····················	····················	····················	····················	····················
A	5630 hCoV-19/USA/WA-UW-352/2020|EPI_ISL_524441|2020-03-	····················	····················	····················	····················	····················	····················
A	5631 hCoV-19/USA/WA-UW-354/2020|EPI_ISL_524442|2020-03-	····················	····················	····················	····················	····················	····················
A	5632 hCoV-19/USA/WA-UW-380/2020|EPI_ISL_524443|2020-03-	····················	····················	····················	····················	····················	····················
A	5633 hCoV-19/Wuhan/IVDC-HB-01/2019|EPI_ISL_402119|2019-	····················	····················	····················	····················	····················	····················
A	5634 hCoV-19/Wuhan/IVDC-HB-04/2020|EPI_ISL_402120|2020-	····················	····················	····················	····················	····················	····················
A	5635 hCoV-19/Wuhan/IVDC-HB-05/2019|EPI_ISL_402121|2019-	····················	····················	····················	····················	····················	····················
A	5636 hCoV-19/Wuhan/IPBCAMS-WH-01/2019|EPI_ISL_402123|20	····················	····················	····················	····················	····················	····················
A	5637 hCoV-19/Wuhan/WIV04/2019|EPI_ISL_402124|2019-12-30	····················	····················	····················	····················	····················	····················
A	5638 hCoV-19/Wuhan/Hu-1/2019|EPI_ISL_402125|2019-12-31	····················	····················	····················	····················	····················	····················
A	5639 hCoV-19/Wuhan/WIV02/2019|EPI_ISL_402127|2019-12-30	····················	····················	····················	····················	····················	····················
A	5640 hCoV-19/Wuhan/WIV05/2019|EPI_ISL_402128|2019-12-30	····················	····················	····················	····················	····················	····················
A	5641 hCoV-19/Wuhan/WIV06/2019|EPI_ISL_402129|2019-12-30	····················	····················	····················	····················	····················	····················
A	5642 hCoV-19/Wuhan/WIV07/2019|EPI_ISL_402130|2019-12-30	····················	····················	····················	····················	····················	····················
A	5643 hCoV-19/Wuhan/HBCDC-HB-01/2019|EPI_ISL_402132|2019	····················	····················	····················	····················	····················	····················
A	5644 hCoV-19/South Korea/SNU01/2020|EPI_ISL_411929|2020	····················	····················	····················	··········T·········	····················	····················
A	5645 hCoV-19/Switzerland/GE-HUG-1422/2020|EPI_ISL_41545	····················	····················	····················	····················	····················	····················
A	5646 hCoV-19/Switzerland/GE-HUG-0199/2020|EPI_ISL_41545	····················	····················	····················	····················	····················	····················
A	5647 hCoV-19/Switzerland/BE-HUG-6651/2020|EPI_ISL_41545	····················	····················	····················	····················	····················	····················
A	5648 hCoV-19/Switzerland/AG-HUG-7120/2020|EPI_ISL_41545	····················	····················	····················	····················	····················	····················
A	5649 hCoV-19/Switzerland/GE-HUG-8102/2020|EPI_ISL_41545	····················	····················	····················	····················	····················	····················
A	5650 hCoV-19/Switzerland/GE-HUG-VD0503/2020|EPI_ISL_415	····················	····················	····················	····················	····················	····················
A	5651 hCoV-19/Netherlands/Flevoland_1/2020|EPI_ISL_41546	····················	····················	····················	····················	····················	····················
A	5652 hCoV-19/Netherlands/Gelderland_1-2/2020|EPI_ISL_41	····················	····················	····················	····················	····················	····················
A	5653 hCoV-19/Netherlands/Gelderland_2/2020|EPI_ISL_4154	····················	····················	····················	····················	····················	····················
A	5654 hCoV-19/Netherlands/Gelderland_3/2020|EPI_ISL_4154	····················	····················	····················	····················	····················	····················
A	5655 hCoV-19/Netherlands/Limburg_7/2020|EPI_ISL_415464|	····················	····················	····················	····················	····················	····················

A	5656 hCoV-19/Netherlands/NA_1/2020|EPI_ISL_415465|2020-	....	....	....	....	....	....
A	5657 hCoV-19/Netherlands/NA_10/2020|EPI_ISL_415466|2020	....	....	....	....	....	....
A	5658 hCoV-19/Netherlands/NA_11/2020|EPI_ISL_415467|2020	....	....	....	....	....	....
A	5659 hCoV-19/Netherlands/NA_12/2020|EPI_ISL_415468|2020	....	....	....	....	....	....
A	5660 hCoV-19/Netherlands/NA_13/2020|EPI_ISL_415469|2020	....	....	....	....	....	....
A	5661 hCoV-19/USA/WA-UW177/2020|EPI_ISL_416715|2020-03-1	....	....	....	....	....	....
A	5662 hCoV-19/USA/WA-UW178/2020|EPI_ISL_416716|2020-03-1	....	....	....	....	....	....
A	5663 hCoV-19/USA/WA-UW179/2020|EPI_ISL_416717|2020-03-1	....	....	....	....	....	....
A	5664 hCoV-19/USA/WA-UW180/2020|EPI_ISL_416718|2020-03-1	....	....	....	....	....	....
A	5665 hCoV-19/USA/WA-UW181/2020|EPI_ISL_416719|2020-03-1	....	....	....	....	....	....
A	5666 hCoV-19/USA/WA-UW183/2020|EPI_ISL_416721|2020-03-1	....	....	....	....	....	....
A	5667 hCoV-19/USA/WA-UW184/2020|EPI_ISL_416722|2020-03-1	....	....	....	....	....	....
A	5668 hCoV-19/USA/WA-UW185/2020|EPI_ISL_416723|2020-03-1	....	....	....	....	....	....
A	5669 hCoV-19/USA/WA-UW186/2020|EPI_ISL_416724|2020-03-1	....	....	....	....	....	....
A	5670 hCoV-19/USA/WA-UW187/2020|EPI_ISL_416725|2020-03-1	....	....	....	....	....	....
A	5671 hCoV-19/USA/WA-UW188/2020|EPI_ISL_416726|2020-03-1	....	....	....	....	....	....
A	5672 hCoV-19/USA/WA-UW189/2020|EPI_ISL_416727|2020-03-1	....	....	....	....	....	....
A	5673 hCoV-19/USA/WA-UW190/2020|EPI_ISL_416728|2020-03-1	....	....	....	....	....	....
A	5674 hCoV-19/USA/WA-UW191/2020|EPI_ISL_416729|2020-03-1	....	....	....	....	....	....
A	5675 hCoV-19/England/SHEF-BFCB1/2020|EPI_ISL_416730|202	....	....	....	....	....	....
A	5676 hCoV-19/England/SHEF-BFCC0/2020|EPI_ISL_416731|202	....	....	....	....	....	....
A	5677 hCoV-19/England/SHEF-BFCDF/2020|EPI_ISL_416732|202	....	....	....	....	....	....
A	5678 hCoV-19/England/SHEF-BFCEE/2020|EPI_ISL_416733|202	....	....	....	....	....	....
A	5679 hCoV-19/England/SHEF-BFCFD/2020|EPI_ISL_416734|202	....	....	....	....	....	....
A	5681 hCoV-19/England/SHEF-BFD18/2020|EPI_ISL_416736|202	....	....	....	....	....	....
A	5683 hCoV-19/England/SHEF-BFD36/2020|EPI_ISL_416738|202	....	....	....	....	....	....
A	5684 hCoV-19/England/SHEF-BFD45/2020|EPI_ISL_416739|202	....	....	....	....	....	....
A	5685 hCoV-19/England/SHEF-BFD54/2020|EPI_ISL_416740|202	....	....	....	....	....	....
A	5686 hCoV-19/Lithuania/ChVir1632/2020|EPI_ISL_416741|20	....	....	....	....	....	....
A	5687 hCoV-19/Czech Republic/ChVir1630/2020|EPI_ISL_4167	....	....	....	....	....	....
A	5688 hCoV-19/Czech Republic/ChVir1912/2020|EPI_ISL_4167	....	....	....	....	....	....
A	5689 hCoV-19/Hungary/mbl49/2020|EPI_ISL_416744|2020-03-	....	....	....	....	....	....
A	5690 hCoV-19/France/ARA-1733/2020|EPI_ISL_416745|2020-0	....	....	....	....	....	....
A	5691 hCoV-19/France/ARA-425/2020|EPI_ISL_416746|2020-03	....	....	....	....	....	....
A	5692 hCoV-19/France/ARA-487/2020|EPI_ISL_416747|2020-03	....	....	....	....	....	....
A	5693 hCoV-19/France/ARA-508/2020|EPI_ISL_416748|2020-03	....	....	....	....	....	....
A	5694 hCoV-19/France/ARA-532/2020|EPI_ISL_416749|2020-03	....	....	....	....	....	....
A	5695 hCoV-19/France/ARA-683/2020|EPI_ISL_416750|2020-03	....	....	....	....	....	....
A	5696 hCoV-19/France/ARA-651/2020|EPI_ISL_416751|2020-03	....	....	....	....	....	....
A	5697 hCoV-19/France/ARA-650/2020|EPI_ISL_416752|2020-03	....	....	....	....	....	....
A	5698 hCoV-19/France/ARA-06464 /2020|EPI_ISL_416753|2020	....	....	....	....	....	....
A	5699 hCoV-19/France/ARA-06487/2020|EPI_ISL_416754|2020-	....	....	....	....	....	....
A	5700 hCoV-19/France/ARA-06531/2020|EPI_ISL_416756|2020-	....	....	....	....	....	....
A	5701 hCoV-19/France/ARA-06678/2020|EPI_ISL_416757|2020-	....	....	....	....	....	....
A	5702 hCoV-19/France/ARA-0693/2020|EPI_ISL_416758|2020-0	....	....	....	....	....	....
A	5703 hCoV-19/Hangzhou/HZCDC0135/2020|EPI_ISL_421225|202	....	....	....	....	....	....
A	5704 hCoV-19/Hangzhou/HZCDC0119/2020|EPI_ISL_421226|202	....	....	....	....	....	....
A	5705 hCoV-19/Hangzhou/HZCDC0091L/2020|EPI_ISL_421227|20	....	....	....	....	....	....
A	5706 hCoV-19/Hangzhou/HZCDC0091/2020|EPI_ISL_421228|202	....	....	....	....	....	....
A	5707 hCoV-19/Hangzhou/HZCDC0090L/2020|EPI_ISL_421229|20	....	....	....	....	....	....
A	5708 hCoV-19/Hangzhou/HZCDC0090/2020|EPI_ISL_421230|202	....	....	....	....	....	....
A	5709 hCoV-19/Hangzhou/HZCDC0049L/2020|EPI_ISL_421231|20	....	....	....	....	....	....
A	5710 hCoV-19/Hangzhou/HZCDC0048L/2020|EPI_ISL_421232|20	....	....	....	....	....	....
A	5711 hCoV-19/Hangzhou/HZCDC0048/2020|EPI_ISL_421233|202	....	....	....	....	....	....
A	5712 hCoV-19/Hangzhou/HZCDC0025/2020|EPI_ISL_421234|202	....	....	....	....	....	....
A	5713 hCoV-19/Hangzhou/HZCDC0013/2020|EPI_ISL_421235|202	....	....	....	....	....	....
A	5714 hCoV-19/Hangzhou/HZCDC0012/2020|EPI_ISL_421236|202	....	....	....	....	....	....
A	5715 hCoV-19/Jiujiang/JX22/2020|EPI_ISL_421237|2020-01-	....	....	....	....	....	....
A	5716 hCoV-19/Nanchang/JX174/2020|EPI_ISL_421238|2020-01	....	....	....	....	....	....
A	5717 hCoV-19/Shangrao/JX105/2020|EPI_ISL_421239|2020-01	....	....	....	....	....	....
A	5718 hCoV-19/Shangrao/JX1948/2020|EPI_ISL_421240|2020-0	....	....	....	....	....	....
A	5719 hCoV-19/Nanchang/JX177/2020|EPI_ISL_421241|2020-01	....	....	....	....	....	....
A	5720 hCoV-19/Ganzhou/JX81/2020|EPI_ISL_421242|2020-01-2	....	....	....	....	....	....
A	5721 hCoV-19/Nanchang/JX14/2020|EPI_ISL_421243|2020-01-	....	....	....	....	....	....
A	5722 hCoV-19/Shangrao/JX29/2020|EPI_ISL_421244|2020-01-	....	....	....	....	....	....
A	5723 hCoV-19/Nanchang/JX90/2020|EPI_ISL_421245|2020-01-	....	....	....	....	....	....
A	5724 hCoV-19/Nanchang/JXN3T4/2020|EPI_ISL_421246|2020-0	....	....	....	....	....	....
A	5725 hCoV-19/Nanchang/JX149/2020|EPI_ISL_421247|2020-01	....	....	....	....	....	....
A	5726 hCoV-19/Nanchang/JX39/2020|EPI_ISL_421248|2020-01-	....	....	....	....	....	....
A	5727 hCoV-19/Xinyu/JX122/2020|EPI_ISL_421249|2020-01-25	....	....	....	....	....	....
A	5728 hCoV-19/Shangrao/JX1177/2020|EPI_ISL_421250|2020-0	....	.A..................	....	....	....	....
A	5729 hCoV-19/Shangrao/JX1176/2020|EPI_ISL_421251|2020-0	....	.A..................	....	....	....	....
A	5730 hCoV-19/Pingxiang/JX5/2020|EPI_ISL_421252|2020-01-	....	....	....	....	....	....
A	5731 hCoV-19/Jian/JX129/2020|EPI_ISL_421253|2020-01-26	....	....	....	....	....	....
A	5732 hCoV-19/Shangrao/JX1178/2020|EPI_ISL_421254|2020-0	....	....	....	....	....	....
A	5733 hCoV-19/Jian/JX169/2020|EPI_ISL_421256|2020-01-27	....	....	....	....	....	....
A	5734 hCoV-19/Shangrao/JX1215/2020|EPI_ISL_421257|2020-0	....	....	....	....	....	....
A	5735 hCoV-19/Shangrao/JX1974/2020|EPI_ISL_421258|2020-0	....	....	....	....	....	....

```
A    5736 hCoV-19/Pingxiang/JX151/2020|EPI_ISL_421259|2020-0    ....................  ....................  ....................  ....................  ....................  ....................
A    5737 hCoV-19/Xinyu/JX124/2020|EPI_ISL_421260|2020-01-26    ....................  ....................  ....................  ....................  ....................  ....................
A    5738 hCoV-19/Nanchang/JX176/2020|EPI_ISL_421261|2020-01    ....................  ....................  ....................  ....................  ....................  ....................
A    5739 hCoV-19/Nanchang/JX155/2020|EPI_ISL_421262|2020-01    ....................  ....................  ....................  ....................  ....................  ....................
A    5740 hCoV-19/USA/WI-UW-29/2020|EPI_ISL_421287|2020-03-2    ....................  ....................  ....................  ....................  ....................  ....................
A    5741 hCoV-19/USA/WI-UW-30/2020|EPI_ISL_421288|2020-03-2    ....................  ....................  ....................  ....................  ....................  ....................
A    5742 hCoV-19/USA/WI-UW-31/2020|EPI_ISL_421289|2020-03-2    ....................  ....................  ....................  ....................  ....................  ....................
A    5743 hCoV-19/USA/NY-PV09301/2020|EPI_ISL_422556|2020-03    ....................  ....................  ....................  ....................  ....................  ....................
A    5744 hCoV-19/USA/NY-PV09303/2020|EPI_ISL_422557|2020-03    ....................  ....................  ....................  ....................  ....................  ....................
A    5745 hCoV-19/USA/NY-PV09304/2020|EPI_ISL_422558|2020-03    ....................  ....................  ....................  ....................  ....................  ....................
A    5746 hCoV-19/USA/NY-PV09305/2020|EPI_ISL_422559|2020-03    ....................  ....................  ....................  ....................  ....................  ....................
A    5747 hCoV-19/USA/NY-PV09307/2020|EPI_ISL_422560|2020-03    ....................  ....................  ....................  ....................  ....................  ....................
A    5748 hCoV-19/USA/NY-PV09308/2020|EPI_ISL_422561|2020-03    ....................  ....................  ....................  ....................  ....................  ....................
A    5749 hCoV-19/USA/NY-PV09309/2020|EPI_ISL_422562|2020-03    ....................  ....................  ....................  ....................  ....................  ....................
A    5750 hCoV-19/Netherlands/Friesland_10/2020|EPI_ISL_4225    ....................  ....................  ....................  ....................  ....................  ....................
A    5751 hCoV-19/Netherlands/Friesland_3/2020|EPI_ISL_42256    ....................  ....................  ....................  ....................  ....................  ....................
A    5752 hCoV-19/Netherlands/Friesland_4/2020|EPI_ISL_42256    ....................  ....................  ....................  ....................  ....................  ....................
A    5753 hCoV-19/Netherlands/Friesland_6/2020|EPI_ISL_42257    ....................  ....................  ....................  ....................  ....................  ....................
A    5754 hCoV-19/Netherlands/Friesland_7/2020|EPI_ISL_42257    ....................  ....................  ....................  ....................  ....................  ....................
A    5755 hCoV-19/Netherlands/NA_265/2020|EPI_ISL_422576|202    ....................  ....................  ....................  ....................  ....................  ....................
A    5756 hCoV-19/Netherlands/NA_266/2020|EPI_ISL_422577|202    ....................  ....................  ....................  ....................  ....................  ....................
A    5757 hCoV-19/Netherlands/NA_267/2020|EPI_ISL_422578|202    ....................  ....................  ....................  ....................  ....................  ....................
A    5758 hCoV-19/Netherlands/NA_268/2020|EPI_ISL_422579|202    ....................  ....................  ....................  ....................  ....................  ....................
A    5759 hCoV-19/Netherlands/NA_269/2020|EPI_ISL_422580|202    ....................  ....................  ....................  ....................  ....................  ....................
A    5760 hCoV-19/Netherlands/NA_270/2020|EPI_ISL_422581|202    ....................  ....................  ....................  ....................  ....................  ....................
A    5761 hCoV-19/Netherlands/NA_271/2020|EPI_ISL_422582|202    ....................  ....................  ....................  ....................  ....................  ....................
A    5762 hCoV-19/Netherlands/NA_272/2020|EPI_ISL_422583|202    ....................  ....................  ....................  ....................  ....................  ....................
A    5763 hCoV-19/Netherlands/NA_274/2020|EPI_ISL_422585|202    ....................  ....................  ....................  ....................  ....................  ....................
A    5764 hCoV-19/Netherlands/NA_275/2020|EPI_ISL_422586|202    ....................  ....................  ....................  ....................  ....................  ....................
A    5765 hCoV-19/Netherlands/NA_276/2020|EPI_ISL_422587|202    ....................  ....................  ....................  ....................  ....................  ....................
A    5766 hCoV-19/Netherlands/NA_277/2020|EPI_ISL_422588|202    ....................  ....................  ....................  ....................  ....................  ....................
A    5767 hCoV-19/Netherlands/NA_282/2020|EPI_ISL_422591|202    ....................  ....................  ....................  ....................  ....................  ....................
A    5768 hCoV-19/Netherlands/NA_283/2020|EPI_ISL_422592|202    ....................  ....................  ....................  ....................  ....................  ....................
A    5769 hCoV-19/Netherlands/NA_284/2020|EPI_ISL_422593|202    ....................  ....................  ....................  ....................  ....................  ....................
A    5770 hCoV-19/Netherlands/NA_285/2020|EPI_ISL_422594|202    ....................  ....................  ....................  ....................  ....................  ....................
A    5771 hCoV-19/Netherlands/NA_286/2020|EPI_ISL_422595|202    ....................  ....................  ....................  ....................  ....................  ....................
A    5772 hCoV-19/Netherlands/NA_287/2020|EPI_ISL_422596|202    ....................  ....................  ....................  ....................  ....................  ....................
A    5773 hCoV-19/Netherlands/NA_288/2020|EPI_ISL_422597|202    ....................  ....................  ....................  ....................  ....................  ....................
A    5774 hCoV-19/Netherlands/NA_289/2020|EPI_ISL_422598|202    ....................  ...........A........  ....................  ....................  ....................  ....................
A    5775 hCoV-19/Netherlands/NA_290/2020|EPI_ISL_422599|202    ....................  ....................  ....................  ....................  ....................  ....................
A    5776 hCoV-19/England/20126009804/2020|EPI_ISL_423887|20    ....................  ....................  ....................  ....................  ....................  ....................
A    5777 hCoV-19/England/20126063104/2020|EPI_ISL_423903|20    ....................  ....................  ....................  ....................  ....................  ....................
A    5778 hCoV-19/USA/WA-UW-2146/2020|EPI_ISL_426100|2020-03    ....................  ....................  ....................  ....................  ....................  ....................
A    5779 hCoV-19/USA/WA-UW-2149/2020|EPI_ISL_426101|2020-03    ....................  ....................  ....................  ....................  ....................  ....................
A    5780 hCoV-19/USA/WA-UW-2164/2020|EPI_ISL_426102|2020-03    ....................  ....................  ....................  ....................  ....................  ....................
A    5781 hCoV-19/USA/WA-UW-2169/2020|EPI_ISL_426103|2020-03    ....................  ....................  ....................  ....................  ....................  ....................
A    5782 hCoV-19/USA/WA-UW-2179/2020|EPI_ISL_426105|2020-03    ....................  ....................  ....................  ....................  ....................  ....................
A    5783 hCoV-19/USA/WA-UW-2180/2020|EPI_ISL_426106|2020-03    ....................  ....................  ....................  ....................  ....................  ....................
A    5784 hCoV-19/USA/WA-UW-2181/2020|EPI_ISL_426107|2020-03    ....................  ....................  ....................  ....................  ....................  ....................
A    5785 hCoV-19/USA/WA-UW-2192/2020|EPI_ISL_426109|2020-03    ....................  ....................  ....................  ....................  ....................  ....................
A    5786 hCoV-19/USA/WA-UW-2203/2020|EPI_ISL_426111|2020-03    ....................  ....................  ....................  ....................  ....................  ....................
A    5787 hCoV-19/USA/WA-UW-2208/2020|EPI_ISL_426112|2020-03    ....................  ....................  ....................  ....................  ....................  ....................
A    5788 hCoV-19/USA/WA-UW-2212/2020|EPI_ISL_426113|2020-03    ....................  ....................  ....................  ....................  ....................  ....................
A    5789 hCoV-19/USA/WA-UW-2213/2020|EPI_ISL_426114|2020-03    ....................  ....................  ....................  ....................  ....................  ....................
A    5790 hCoV-19/USA/WA-UW-2214/2020|EPI_ISL_426115|2020-03    ....................  ....................  ....................  ....................  ....................  ....................
A    5791 hCoV-19/USA/WA-UW-2215/2020|EPI_ISL_426116|2020-03    ....................  ....................  ....................  ....................  ....................  ....................
A    5792 hCoV-19/USA/WA-UW-2218/2020|EPI_ISL_426117|2020-03    ....................  ....................  ....................  ....................  ....................  ....................
A    5793 hCoV-19/USA/WA-UW-2219/2020|EPI_ISL_426118|2020-03    ....................  ....................  ....................  ....................  ....................  ....................
A    5794 hCoV-19/USA/WA-UW-2228/2020|EPI_ISL_426119|2020-03    ....................  ....................  ....................  ....................  ....................  ....................
A    5795 hCoV-19/India/UN-c31/2020|EPI_ISL_426179|2020-03-0    ....................  ....................  ....................  ....................  ....................  ....................
A    5796 hCoV-19/Qatar/QA13/2020|EPI_ISL_427408|2020-03-23     ....................  ....................  ....................  ....................  ....................  ....................
A    5797 hCoV-19/Qatar/QA20/2020|EPI_ISL_427416|2020-03-23     ....................  ....................  ....................  ....................  ....................  ....................
A    5798 hCoV-19/Qatar/QA21/2020|EPI_ISL_427417|2020-03-23     ....................  ....................  ....................  ....................  ....................  ....................
A    5799 hCoV-19/Qatar/QA22/2020|EPI_ISL_427418|2020-03-23     ....................  ....................  ....................  ....................  ....................  ....................
A    5800 hCoV-19/USA/WI-UW-120/2020|EPI_ISL_427427|2020-04-    ....................  ....................  ....................  ....................  ....................  ....................
A    5801 hCoV-19/USA/IA-UW-121/2020|EPI_ISL_427428|2020-04-    ....................  ....................  ....................  ....................  ....................  ....................
A    5802 hCoV-19/USA/WI-UW-122/2020|EPI_ISL_427429|2020-04-    ....................  ....................  ....................  ....................  ....................  ....................
A    5803 hCoV-19/USA/IL-UW-123/2020|EPI_ISL_427430|2020-04-    ....................  ....................  ....................  ....................  ....................  ....................
A    5804 hCoV-19/USA/WI-UW-124/2020|EPI_ISL_427431|2020-04-    ....................  ....................  ....................  ....................  ....................  ....................
A    5805 hCoV-19/USA/IL-UW-125/2020|EPI_ISL_427432|2020-04-    ....................  ....................  ....................  ....................  ....................  ....................
A    5806 hCoV-19/USA/IL-UW-126/2020|EPI_ISL_427433|2020-04-    ....................  ....................  ....................  ....................  ....................  ....................
A    5807 hCoV-19/USA/WI-UW-127/2020|EPI_ISL_427434|2020-04-    ....................  ....................  ....................  ....................  ....................  ....................
A    5808 hCoV-19/USA/WI-UW-128/2020|EPI_ISL_427435|2020-04-    ....................  ....................  ....................  ....................  ....................  ....................
A    5809 hCoV-19/USA/WI-UW-129/2020|EPI_ISL_427436|2020-04-    ....................  ....................  ....................  ....................  ....................  ....................
A    5810 hCoV-19/USA/IL-UW-130/2020|EPI_ISL_427437|2020-04-    ....................  ....................  ....................  ....................  ....................  ....................
A    5811 hCoV-19/USA/WI-UW-131/2020|EPI_ISL_427438|2020-04-    ....................  ....................  ....................  ....................  ....................  ....................
A    5812 hCoV-19/USA/WI-UW-132/2020|EPI_ISL_427439|2020-04-    ....................  ....................  ....................  ....................  ....................  ....................
A    5813 hCoV-19/USA/IL-UW-133/2020|EPI_ISL_427440|2020-04-    ....................  ....................  ....................  ....................  ....................  ....................
```

A	5814 hCoV-19/USA/WI-UW-134/2020|EPI_ISL_427441|2020-04-	····················	····················	····················	····················	····················	····················
A	5815 hCoV-19/USA/WI-UW-135/2020|EPI_ISL_427442|2020-04-	····················	····················	····················	····················	····················	····················
A	5816 hCoV-19/USA/WI-UW-136/2020|EPI_ISL_427443|2020-04-	····················	····················	····················	····················	····················	····················
A	5817 hCoV-19/USA/IL-UW-137/2020|EPI_ISL_427444|2020-04-	····················	····················	····················	····················	····················	····················
A	5818 hCoV-19/USA/IL-UW-138/2020|EPI_ISL_427445|2020-04-	····················	····················	····················	····················	····················	····················
A	5819 hCoV-19/USA/IL-UW-139/2020|EPI_ISL_427446|2020-04-	····················	····················	····················	····················	····················	····················
A	5820 hCoV-19/USA/WI-UW-140/2020|EPI_ISL_427447|2020-04-	····················	····················	····················	····················	····················	····················
A	5821 hCoV-19/USA/IL-UW-141/2020|EPI_ISL_427448|2020-04-	····················	····················	····················	····················	····················	····················
A	5822 hCoV-19/USA/IL-UW-142/2020|EPI_ISL_427449|2020-04-	····················	····················	····················	····················	····················	····················
A	5823 hCoV-19/Spain/MD-H12-2501/2020|EPI_ISL_428700|2020	····················	····················	····················	····················	····················	····················
A	5824 hCoV-19/Spain/MD-H12-2902/2020|EPI_ISL_428701|2020	····················	····················	····················	····················	····················	····················
A	5825 hCoV-19/Spain/MD-H12-3304/2020|EPI_ISL_428703|2020	····················	····················	····················	····················	····················	····················
A	5826 hCoV-19/Spain/MD-H12-3405/2020|EPI_ISL_428704|2020	····················	····················	····················	····················	····················	····················
A	5827 hCoV-19/Spain/MD-H12-3506/2020|EPI_ISL_428705|2020	····················	····················	····················	····················	····················	····················
A	5828 hCoV-19/Spain/MD-H12-3607/2020|EPI_ISL_428706|2020	····················	····················	····················	····················	····················	····················
A	5829 hCoV-19/Spain/MD-H12-3708/2020|EPI_ISL_428707|2020	····················	····················	····················	····················	····················	····················
A	5830 hCoV-19/Spain/MD-H12-3809/2020|EPI_ISL_428708|2020	····················	····················	····················	····················	····················	····················
A	5831 hCoV-19/Spain/MD-H12-3910/2020|EPI_ISL_428709|2020	····················	····················	····················	····················	····················	····················
A	5832 hCoV-19/Spain/MD-H12-4011/2020|EPI_ISL_428710|2020	····················	····················	····················	····················	····················	····················
A	5833 hCoV-19/Spain/MD-H12-4112/2020|EPI_ISL_428711|2020	····················	····················	····················	····················	····················	····················
A	5834 hCoV-19/Turkey/HSGM-4706/2020|EPI_ISL_428712|2020-	····················	····················	····················	····················	····················	····················
A	5835 hCoV-19/Turkey/HSGM-5516/2020|EPI_ISL_428713|2020-	····················	····················	····················	····················	····················	····················
A	5836 hCoV-19/Turkey/HSGM-5602/2020|EPI_ISL_428714|2020-	····················	····················	····················	····················	····················	····················
A	5837 hCoV-19/Turkey/HSGM-5624/2020|EPI_ISL_428715|2020-	····················	····················	····················	····················	····················	····················
A	5838 hCoV-19/Turkey/HSGM-5711/2020|EPI_ISL_428716|2020-	····················	·A··················	····················	····················	····················	··A·················
A	5839 hCoV-19/Turkey/HSGM-5769/2020|EPI_ISL_428717|2020-	····················	····················	····················	····················	····················	····················
A	5840 hCoV-19/Turkey/HSGM-5770/2020|EPI_ISL_428718|2020-	····················	····················	····················	····················	····················	····················
A	5841 hCoV-19/Turkey/HSGM-7718/2020|EPI_ISL_428719|2020-	····················	····················	····················	····················	····················	····················
A	5842 hCoV-19/Turkey/HSGM-8001/2020|EPI_ISL_428720|2020-	····················	····················	····················	····················	····················	····················
A	5843 hCoV-19/Turkey/HSGM-8003/2020|EPI_ISL_428721|2020-	····················	····················	····················	····················	····················	····················
A	5844 hCoV-19/Turkey/HSGM-8010/2020|EPI_ISL_428722|2020-	····················	····················	····················	····················	····················	····················
A	5845 hCoV-19/Turkey/HSGM-8964/2020|EPI_ISL_428723|2020-	····················	·A··················	····················	····················	····················	··A·················
A	5846 hCoV-19/USA/WI-UW-250/2020|EPI_ISL_428724|2020-03-	····················	····················	····················	····················	····················	····················
A	5847 hCoV-19/USA/WI-UW-251/2020|EPI_ISL_428725|2020-03-	····················	····················	····················	····················	····················	····················
A	5848 hCoV-19/USA/WI-UW-252/2020|EPI_ISL_428726|2020-03-	····················	····················	····················	····················	····················	····················
A	5849 hCoV-19/USA/WI-UW-253/2020|EPI_ISL_428727|2020-03-	····················	····················	····················	····················	····················	····················
A	5850 hCoV-19/USA/WI-UW-254/2020|EPI_ISL_428728|2020-04-	····················	····················	····················	····················	····················	····················
A	5851 hCoV-19/USA/WI-UW-255/2020|EPI_ISL_428729|2020-04-	····················	····················	····················	····················	····················	····················
A	5852 hCoV-19/USA/WI-UW-256/2020|EPI_ISL_428730|2020-04-	····················	····················	····················	····················	····················	····················
A	5853 hCoV-19/USA/WI-UW-257/2020|EPI_ISL_428731|2020-04-	····················	····················	····················	····················	····················	····················
A	5854 hCoV-19/USA/WI-UW-258/2020|EPI_ISL_428732|2020-04-	····················	····················	····················	····················	····················	····················
A	5855 hCoV-19/USA/NY-NYUMC243/2020|EPI_ISL_428757|2020-0	····················	·············A······	····················	····················	····················	····················
A	5856 hCoV-19/USA/NY-NYUMC244/2020|EPI_ISL_428758|2020-0	····················	····················	····················	····················	····················	····················
A	5857 hCoV-19/USA/NY-NYUMC245/2020|EPI_ISL_428759|2020-0	····················	····················	····················	····················	····················	····················
A	5858 hCoV-19/USA/NY-NYUMC246/2020|EPI_ISL_428760|2020-0	····················	····················	····················	····················	····················	····················
A	5859 hCoV-19/USA/NY-NYUMC247/2020|EPI_ISL_428761|2020-0	····················	····················	····················	····················	····················	····················
A	5860 hCoV-19/USA/NY-NYUMC248/2020|EPI_ISL_428762|2020-0	····················	····················	····················	····················	····················	····················
A	5861 hCoV-19/USA/NY-NYUMC249/2020|EPI_ISL_428763|2020-0	····················	····················	····················	····················	····················	····················
A	5862 hCoV-19/USA/NY-NYUMC250/2020|EPI_ISL_428764|2020-0	····················	····················	····················	····················	····················	····················
A	5863 hCoV-19/USA/NY-NYUMC251/2020|EPI_ISL_428765|2020-0	····················	····················	····················	····················	····················	····················
A	5864 hCoV-19/USA/NY-NYUMC252/2020|EPI_ISL_428766|2020-0	····················	····················	····················	····················	····················	····················
A	5865 hCoV-19/USA/NY-NYUMC253/2020|EPI_ISL_428767|2020-0	····················	····················	····················	····················	····················	····················
A	5866 hCoV-19/USA/NY-NYUMC254/2020|EPI_ISL_428768|2020-0	····················	····················	····················	····················	····················	····················
A	5867 hCoV-19/USA/NY-NYUMC255/2020|EPI_ISL_428769|2020-0	····················	····················	····················	····················	····················	····················
A	5868 hCoV-19/USA/NY-NYUMC256/2020|EPI_ISL_428770|2020-0	····················	····················	····················	····················	····················	····················
A	5869 hCoV-19/USA/NY-NYUMC258/2020|EPI_ISL_428772|2020-0	····················	····················	····················	····················	····················	····················
A	5870 hCoV-19/USA/NY-NYUMC259/2020|EPI_ISL_428773|2020-0	····················	····················	····················	····················	····················	····················
A	5871 hCoV-19/USA/NY-NYUMC260/2020|EPI_ISL_428774|2020-0	····················	····················	····················	····················	····················	····················
A	5872 hCoV-19/USA/NY-NYUMC261/2020|EPI_ISL_428775|2020-0	····················	····················	····················	····················	····················	····················
A	5873 hCoV-19/USA/NY-NYUMC262/2020|EPI_ISL_428776|2020-0	····················	····················	····················	····················	····················	····················
A	5874 hCoV-19/USA/NY-NYUMC263/2020|EPI_ISL_428777|2020-0	····················	····················	····················	····················	····················	····················
A	5875 hCoV-19/USA/NY-NYUMC264/2020|EPI_ISL_428778|2020-0	····················	····················	····················	····················	····················	····················
A	5876 hCoV-19/USA/NY-NYUMC265/2020|EPI_ISL_428779|2020-0	····················	····················	····················	····················	····················	····················
A	5877 hCoV-19/Hong Kong/HK20/2020|EPI_ISL_434560|2020-01	····················	····················	····················	····················	····················	····················
A	5878 hCoV-19/Japan/TKYE6968/2020|EPI_ISL_434561|2020-02	····················	····················	····················	····················	····················	····················
A	5879 hCoV-19/Japan/TKYE6947/2020|EPI_ISL_434562|2020-03	····················	····················	····················	····················	····················	····················
A	5880 hCoV-19/Hong Kong/HKU-902a/2020|EPI_ISL_434563|202	····················	····················	····················	····················	····················	····················
A	5881 hCoV-19/Hong Kong/HKU-902b/2020|EPI_ISL_434564|202	····················	····················	····················	····················	····················	····················
A	5882 hCoV-19/Hong Kong/HKU-903a/2020|EPI_ISL_434565|202	····················	····················	····················	····················	····················	····················
A	5883 hCoV-19/Hong Kong/HKU-903b/2020|EPI_ISL_434566|202	····················	····················	····················	····················	····················	····················
A	5884 hCoV-19/Hong Kong/HKU-907a/2020|EPI_ISL_434567|202	····················	····················	····················	····················	····················	····················
A	5885 hCoV-19/Hong Kong/HKU-907b/2020|EPI_ISL_434568|202	····················	····················	····················	····················	····················	····················
A	5886 hCoV-19/Hong Kong/HKU-908a/2020|EPI_ISL_434569|202	····················	····················	····················	····················	····················	····················
A	5887 hCoV-19/Hong Kong/HKU-908b/2020|EPI_ISL_434570|202	····················	····················	····················	····················	····················	····················
A	5888 hCoV-19/Hong Kong/HKU-001a/2020|EPI_ISL_434571|202	····················	····················	····················	····················	····················	····················
A	5889 hCoV-19/USA/CA-CDC-3/2020|EPI_ISL_408008|2020-01-2	····················	·A··················	····················	····················	····················	····················
A	5890 hCoV-19/USA/CA-CDC-4/2020|EPI_ISL_408009|2020-01-2	····················	·A··················	····················	····················	····················	····················
A	5891 hCoV-19/USA/CA-CDC-5/2020|EPI_ISL_408010|2020-01-2	····················	····················	····················	····················	····················	····················

```
A                5892 hCoV-19/Nepal/61/2020|EPI_ISL_410301|2020-01-13     ····················  ····················  ·····················  ·····················  ····················  ····················
A                5893 hCoV-19/Germany/BW-ChVir-1577/2020|EPI_ISL_412912|  ····················  ····················  ·····················  ·····················  ····················  ····················
A                5894 hCoV-19/Brazil/SP-01/2020|EPI_ISL_412964|2020-02-2  ····················  ····················  ·····················  ·····················  ····················  ····················
A                5895 hCoV-19/Canada/BC_37_0-2/2020|EPI_ISL_412965|2020-  ····················  ····················  ·····················  ·····················  ····················  ····················
A                5896 hCoV-19/Guangzhou/IQTC01/2020|EPI_ISL_412966|2020-  ····················  ····················  ·····················  ·····················  ····················  ····················
A                5897 hCoV-19/Japan/Hu_DP_Kng_19-020/2020|EPI_ISL_412968  ····················  ····················  ·····················  ·····················  ····················  ····················
A                5898 hCoV-19/USA/WA2/2020|EPI_ISL_412970|2020-02-24      ····················  ····················  ·····················  ·····················  ····················  ····················
A                5899 hCoV-19/Mexico/CMX-InDRE-01/2020|EPI_ISL_412972|20  ····················  ····················  ·····················  ·····················  ····················  ····················
A                5900 hCoV-19/Italy/LOM-ASST-CDG1/2020|EPI_ISL_412973|20  ····················  ····················  ·····················  ·····················  ····················  ····················
A                5901 hCoV-19/Italy/LAZ-INMI-SPL1/2020|EPI_ISL_412974|20  ····················  ····················  ·····················  ·····················  ····················  ····················
A                5902 hCoV-19/Australia/NSW05/2020|EPI_ISL_412975|2020-0  ····················  ····················  ·····················  ·····················  ····················  ····················
A                5903 hCoV-19/Wuhan/HBCDC-HB-02/2020|EPI_ISL_412978|2020  ····················  ····················  ·····················  ·····················  ····················  ····················
A                5904 hCoV-19/Wuhan/HBCDC-HB-03/2020|EPI_ISL_412979|2020  ····················  ····················  ·····················  ·····················  ····················  ····················
A                5905 hCoV-19/Wuhan/HBCDC-HB-04/2020|EPI_ISL_412980|2020  ····················  ····················  ·····················  ·····················  ····················  ····················
A                5906 hCoV-19/Wuhan/HBCDC-HB-05/2020|EPI_ISL_412981|2020  ····················  ····················  ·····················  ·····················  ····················  ····················
A                5907 hCoV-19/Wuhan/HBCDC-HB-06/2020|EPI_ISL_412982|2020  ····················  ····················  ·····················  ·····················  ····················  ····················
A                5908 hCoV-19/Tianmen/HBCDC-HB-07/2020|EPI_ISL_412983|20  ····················  ····················  ·····················  ·····················  ····················  ····················
A                5909 hCoV-19/Brazil/ES-225/2020|EPI_ISL_415128|2020-02-  ····················  ····················  ·····················  ·····················  ····················  ····················
A                5910 hCoV-19/England/20099038206/2020|EPI_ISL_415129|20  ····················  ····················  ·····················  ·····················  ····················  ····················
A                5911 hCoV-19/England/20100121006/2020|EPI_ISL_415140|20  ····················  ····················  ·····················  ·····················  ····················  ····················
A                5912 hCoV-19/England/20100121007/2020|EPI_ISL_415141|20  ····················  ····················  ·····················  ·····················  ····················  ····················
A                5913 hCoV-19/England/20100122106/2020|EPI_ISL_415142|20  ····················  ····················  ·····················  ·····················  ····················  ····················
A                5914 hCoV-19/England/20100122107/2020|EPI_ISL_415143|20  ····················  ····················  ·····················  ·····················  ····················  ····················
A                5915 hCoV-19/England/20102000106/2020|EPI_ISL_415144|20  ····················  ····················  ·····················  ·····················  ····················  ····················
A                5916 hCoV-19/England/20102000306/2020|EPI_ISL_415146|20  ····················  ····················  ·····················  ·····················  ····················  ····················
A                5917 hCoV-19/USA/WA-UW84/2020|EPI_ISL_416440|2020-03-10  ····················  ····················  ·····················  ·····················  ····················  ····················
A                5918 hCoV-19/USA/WA-UW85/2020|EPI_ISL_416441|2020-03-10  ····················  ····················  ·····················  ·····················  ····················  ····················
A                5919 hCoV-19/USA/WA-UW86/2020|EPI_ISL_416442|2020-03-10  ····················  ····················  ·····················  ·····················  ····················  ····················
A                5920 hCoV-19/USA/WA-UW87/2020|EPI_ISL_416443|2020-03-10  ····················  ····················  ·····················  ·····················  ····················  ····················
A                5921 hCoV-19/USA/WA-UW88/2020|EPI_ISL_416444|2020-03-10  ····················  ····················  ·····················  ·····················  ····················  ····················
A                5922 hCoV-19/USA/WA-UW89/2020|EPI_ISL_416445|2020-03-10  ····················  ····················  ·····················  ·····················  ····················  ····················
A                5923 hCoV-19/USA/WA-UW90/2020|EPI_ISL_416446|2020-03-10  ····················  ····················  ·····················  ·····················  ····················  ····················
A                5924 hCoV-19/USA/WA-UW91/2020|EPI_ISL_416447|2020-03-10  ····················  ····················  ·····················  ·····················  ····················  ····················
A                5925 hCoV-19/USA/WA-UW92/2020|EPI_ISL_416448|2020-03-11  ····················  ····················  ·····················  ·····················  ····················  ····················
A                5926 hCoV-19/USA/WA-UW93/2020|EPI_ISL_416449|2020-03-11  ····················  ····················  ·····················  ·····················  ····················  ····················
A                5927 hCoV-19/USA/WA-UW94/2020|EPI_ISL_416450|2020-03-11  ····················  ····················  ·····················  ·····················  ····················  ····················
A                5928 hCoV-19/USA/WA-UW95/2020|EPI_ISL_416451|2020-03-10  ····················  ····················  ·····················  ·····················  ····················  ····················
A                5929 hCoV-19/USA/WA-UW96/2020|EPI_ISL_416452|2020-03-10  ····················  ····················  ·····················  ·····················  ····················  ····················
A                5930 hCoV-19/USA/WA-UW36/2020|EPI_ISL_416453|2020-03-07  ····················  ····················  ·····················  ·····················  ····················  ····················
A                5931 hCoV-19/USA/WA-UW37/2020|EPI_ISL_416454|2020-03-06  ····················  ····················  ·····················  ·····················  ····················  ····················
A                5932 hCoV-19/USA/WA-UW38/2020|EPI_ISL_416455|2020-03-07  ····················  ····················  ·····················  ·····················  ····················  ····················
A                5933 hCoV-19/USA/WA-UW39/2020|EPI_ISL_416456|2020-03-06  ····················  ····················  ·····················  ·····················  ····················  ····················
A                5934 hCoV-19/USA/CA-MG0987/2020|EPI_ISL_416457|2020-03-  ····················  ····················  ·····················  ·····················  ····················  ····················
A                5935 hCoV-19/Kuwait/KU12/2020|EPI_ISL_416458|2020-03-02  ····················  ····················  ·····················  ·····················  ····················  ····················
A                5936 hCoV-19/Belgium/DB-03023/2020|EPI_ISL_416470|2020-  ····················  ····················  ·····················  ·····················  ····················  ····················
A                5937 hCoV-19/Belgium/DBD-03024/2020|EPI_ISL_416471|2020  ····················  ····················  ·····················  ·····················  ····················  ····················
A                5938 hCoV-19/Belgium/UMF-03025/2020|EPI_ISL_416472|2020  ····················  ····················  ·····················  ·····················  ····················  ····················
A                5939 hCoV-19/Belgium/DBA-03032/2020|EPI_ISL_416475|2020  ····················  ····················  ·····················  ·····················  ····················  ····················
A                5940 hCoV-19/Georgia/Tb-673/2020|EPI_ISL_416478|2020-03  ····················  ····················  ·····················  ·····················  ····················  ····················
A                5941 hCoV-19/Georgia/Tb-273/2020|EPI_ISL_416479|2020-03  ····················  ····················  ·····················  ·····················  ····················  ····················
A                5942 hCoV-19/USA/WA-UW162/2020|EPI_ISL_416700|2020-03-1  ····················  ····················  ·····················  ·····················  ····················  ····················
A                5943 hCoV-19/USA/WA-UW163/2020|EPI_ISL_416701|2020-03-1  ····················  ····················  ·····················  ·····················  ····················  ····················
A                5944 hCoV-19/USA/WA-UW164/2020|EPI_ISL_416702|2020-03-1  ····················  ····················  ·····················  ·····················  ····················  ····················
A                5945 hCoV-19/USA/WA-UW165/2020|EPI_ISL_416703|2020-03-1  ····················  ····················  ·····················  ·····················  ····················  ····················
A                5946 hCoV-19/USA/WA-UW167/2020|EPI_ISL_416705|2020-03-1  ····················  ····················  ·····················  ·····················  ····················  ····················
A                5947 hCoV-19/USA/WA-UW168/2020|EPI_ISL_416706|2020-03-1  ····················  ····················  ·····················  ·····················  ····················  ····················
A                5948 hCoV-19/USA/WA-UW169/2020|EPI_ISL_416707|2020-03-1  ····················  ····················  ·····················  ·····················  ····················  ····················
A                5949 hCoV-19/USA/WA-UW170/2020|EPI_ISL_416708|2020-03-1  ····················  ····················  ·····················  ·····················  ····················  ····················
A                5950 hCoV-19/USA/WA-UW171/2020|EPI_ISL_416709|2020-03-1  ····················  ····················  ·····················  ·····················  ····················  ····················
A                5951 hCoV-19/USA/WA-UW172/2020|EPI_ISL_416710|2020-03-1  ····················  ····················  ·····················  ·····················  ····················  ····················
A                5952 hCoV-19/USA/WA-UW173/2020|EPI_ISL_416711|2020-03-1  ····················  ····················  ·····················  ·····················  ····················  ····················
A                5953 hCoV-19/USA/WA-UW174/2020|EPI_ISL_416712|2020-03-1  ····················  ····················  ·····················  ·····················  ····················  ····················
A                5954 hCoV-19/USA/WA-UW175/2020|EPI_ISL_416713|2020-03-1  ····················  ····················  ·····················  ·····················  ····················  ····················
A                5955 hCoV-19/USA/WA-UW176/2020|EPI_ISL_416714|2020-03-1  ····················  ····················  ·····················  ·····················  ····················  ····················
A                5956 hCoV-19/Iceland/112/2020|EPI_ISL_417770|2020-03-11  ····················  ·A··················  ·····················  ·····················  ····················  ····················
A                5957 hCoV-19/Iceland/131/2020|EPI_ISL_417771|2020-03-12  ····················  ····················  ·····················  ·····················  ····················  ····················
A                5958 hCoV-19/Iceland/162/2020|EPI_ISL_417772|2020-03-14  ····················  ····················  ·····················  ·····················  ····················  ····················
A                5959 hCoV-19/Iceland/30/2020|EPI_ISL_417773|2020-03-03    ····················  ····················  ·····················  ·····················  ····················  ····················
A                5960 hCoV-19/Iceland/175/2020|EPI_ISL_417774|2020-03-16  ····················  ····················  ·····················  ·····················  ····················  ····················
A                5961 hCoV-19/Iceland/163/2020|EPI_ISL_417775|2020-03-14  ····················  ····················  ·····················  ·····················  ····················  ····················
A                5962 hCoV-19/Iceland/102/2020|EPI_ISL_417777|2020-03-10  ····················  ·A··················  ·····················  ·····················  ····················  ····················
A                5963 hCoV-19/Iceland/106/2020|EPI_ISL_417779|2020-03-11  ····················  ····················  ·····················  ·····················  ····················  ····················
A                5965 hCoV-19/Iceland/123/2020|EPI_ISL_417783|2020-03-11  ····················  ····················  ·····················  ·····················  ····················  ····················
A                5966 hCoV-19/Iceland/124/2020|EPI_ISL_417784|2020-03-12  ····················  ····················  ·····················  ·····················  ····················  ····················
A                5967 hCoV-19/Iceland/126/2020|EPI_ISL_417785|2020-03-12  ····················  ····················  ·····················  ·····················  ····················  ····················
A                5968 hCoV-19/Iceland/128/2020|EPI_ISL_417786|2020-03-12  ····················  ····················  ·····················  ·····················  ····················  ····················
A                5969 hCoV-19/Iceland/132/2020|EPI_ISL_417787|2020-03-12  ····················  ····················  ·····················  ·····················  ····················  ····················
A                5970 hCoV-19/Iceland/133/2020|EPI_ISL_417788|2020-03-12  ····················  ····················  ·····················  ·····················  ····················  ····················
```

A	5971	hCoV-19/Iceland/136/2020|EPI_ISL_417789|2020-03-12	....	....	....	....	....	....
A	5972	hCoV-19/USA/WY-WYPHL001/2020|EPI_ISL_421272|2020-0	....	....	....	....	....	....
A	5973	hCoV-19/Russia/Moscow_PMVL-1/2020|EPI_ISL_421275|2	....	....	....	....	....	....
A	5974	hCoV-19/USA/IN-Lilly-IPB0170-4bc20/2020|EPI_ISL_42	....	....	....	....	....	....
A	5975	hCoV-19/USA/IN-Lilly-IPB0170-4bc21/2020|EPI_ISL_42	....	....	....	....	....	....
A	5976	hCoV-19/USA/WI-UW-25/2020|EPI_ISL_421283|2020-03-2	....	....	....	....	....	....
A	5977	hCoV-19/USA/WI-UW-26/2020|EPI_ISL_421284|2020-03-2	....	....	....	....	....	....
A	5978	hCoV-19/USA/WI-UW-27/2020|EPI_ISL_421285|2020-03-2	....	....	....	....	....	....
A	5979	hCoV-19/USA/WI-UW-28/2020|EPI_ISL_421286|2020-03-2	....	....	....	....	....	....
A	5980	hCoV-19/USA/WI-UW-32/2020|EPI_ISL_421290|2020-03-2	....	....	....	....	....	....
A	5981	hCoV-19/USA/WI-UW-33/2020|EPI_ISL_421291|2020-03-2	....	....	....	....	....	....
A	5982	hCoV-19/USA/WI-UW-34/2020|EPI_ISL_421292|2020-03-2	....	....	....	....	....	....
A	5983	hCoV-19/USA/WI-UW-35/2020|EPI_ISL_421293|2020-03-2	....	....	....	....	....	....
A	5984	hCoV-19/USA/WI-UW-36/2020|EPI_ISL_421294|2020-03-2	....	....	....	....	....	....
A	5985	hCoV-19/USA/WI-UW-37/2020|EPI_ISL_421295|2020-03-2	....	....	....	....	....	....
A	5986	hCoV-19/USA/WI-UW-38/2020|EPI_ISL_421296|2020-03-2	....	....	....	....	....	....
A	5987	hCoV-19/USA/WI-UW-39/2020|EPI_ISL_421297|2020-03-2	....	....	....	....	....	....
A	5988	hCoV-19/USA/WI-UW-40/2020|EPI_ISL_421298|2020-03-2	....	....	....	....	....	....
A	5989	hCoV-19/USA/WI-UW-41/2020|EPI_ISL_421299|2020-03-2	....	....	....	....	....	....
A	5991	hCoV-19/England/20130052804/2020|EPI_ISL_423632|20	....	....	....	....	....	....
A	5992	hCoV-19/England/20130061404/2020|EPI_ISL_423639|20	....	....	....	....	....	....
A	5993	hCoV-19/USA/IA_6390/2020|EPI_ISL_424900|2020-03-07	....	....	....	....	....	....
A	5994	hCoV-19/USA/NJ_3201/2020|EPI_ISL_424901|2020-03-04	....	....	....	....	....	....
A	5995	hCoV-19/USA/SC_3520/2020|EPI_ISL_424902|2020-03-05	....	....	....	....	....	....
A	5996	hCoV-19/USA/SC_3570/2020|EPI_ISL_424903|2020-03-07	....	....	....	....	....	....
A	5997	hCoV-19/USA/SC_3571/2020|EPI_ISL_424904|2020-03-07	....	....	....	....	....	....
A	5998	hCoV-19/USA/SC_3572/2020|EPI_ISL_424905|2020-03-05	....	....	....	....	....	....
A	5999	hCoV-19/USA/NY_1922/2020|EPI_ISL_424906|2020-03-04	....	....	....	....	....	....
A	6000	hCoV-19/USA/VA_6171/2020|EPI_ISL_424907|2020-03-06	....	....	....	....	....	....
A	6001	hCoV-19/USA/MA_8932/2020|EPI_ISL_424908|2020-03-05	....	....	....	....	....	....
A	6002	hCoV-19/USA/MA_8933/2020|EPI_ISL_424909|2020-03-05	....	....	....	....	....	....
A	6003	hCoV-19/USA/MA_9703/2020|EPI_ISL_424910|2020-03-05	....	....	....	....	....	....
A	6004	hCoV-19/USA/MA_9704/2020|EPI_ISL_424911|2020-03-04	....	....	....	....	....	....
A	6005	hCoV-19/USA/MA_9889/2020|EPI_ISL_424912|2020-03-04	....	....	....	....	....	....
A	6006	hCoV-19/USA/MA_1355/2020|EPI_ISL_424913|2020-03-05	....	....	....	....	....	....
A	6007	hCoV-19/USA/MA_2652/2020|EPI_ISL_424914|2020-03-06	....	....	....	....	....	....
A	6008	hCoV-19/USA/MA_3614/2020|EPI_ISL_424915|2020-03-06	....	....	....	....	....	....
A	6009	hCoV-19/USA/MA_3616/2020|EPI_ISL_424916|2020-03-06	....	....	....	....	....	....
A	6010	hCoV-19/USA/MA_3623/2020|EPI_ISL_424917|2020-03-06	....	....	....	....	....	....
A	6011	hCoV-19/USA/MA_3626/2020|EPI_ISL_424918|2020-03-06	....	....	....	....	....	....
A	6012	hCoV-19/USA/MA_3642/2020|EPI_ISL_424919|2020-03-07	....	....	....	....	....	....
A	6013	hCoV-19/USA/MA_3653/2020|EPI_ISL_424920|2020-03-06	....	....	....	....	....	....
A	6014	hCoV-19/USA/NY-NYUMC122/2020|EPI_ISL_424929|2020-0	....	....	....	....	....	....
A	6015	hCoV-19/USA/NY-NYUMC123/2020|EPI_ISL_424930|2020-0	....	....	....	....	....	....
A	6016	hCoV-19/USA/NY-NYUMC124/2020|EPI_ISL_424931|2020-0	....	....	....	....	....	....
A	6017	hCoV-19/USA/NY-NYUMC125/2020|EPI_ISL_424932|2020-0	....	....	....	....	....	....
A	6018	hCoV-19/USA/NY-NYUMC126/2020|EPI_ISL_424933|2020-0	....	....	....	....	....	....
A	6019	hCoV-19/USA/NY-NYUMC127/2020|EPI_ISL_424934|2020-0	....	....	....	....	....	....
A	6020	hCoV-19/USA/NY-NYUMC128/2020|EPI_ISL_424935|2020-0	....	....	....	....	....	....
A	6021	hCoV-19/USA/NY-NYUMC129/2020|EPI_ISL_424936|2020-0	....	....	....	....	....	....
A	6022	hCoV-19/USA/NY-NYUMC130/2020|EPI_ISL_424937|2020-0	....	....	....	....	....	....
A	6023	hCoV-19/USA/NY-NYUMC131/2020|EPI_ISL_424938|2020-0	....	....	....	....	....	....
A	6024	hCoV-19/USA/NY-NYUMC132/2020|EPI_ISL_424939|2020-0	....	....	....	....	....	....
A	6025	hCoV-19/USA/NY-NYUMC133/2020|EPI_ISL_424940|2020-0	....	....	....	....	....	....
A	6026	hCoV-19/USA/NY-NYUMC134/2020|EPI_ISL_424941|2020-0	....	....	....	....	....	....
A	6027	hCoV-19/USA/NY-NYUMC135/2020|EPI_ISL_424942|2020-0	....	....	....	....	....	....
A	6028	hCoV-19/USA/NY-NYUMC136/2020|EPI_ISL_424943|2020-0	....	....	....	....	....	....
A	6029	hCoV-19/USA/NY-NYUMC137/2020|EPI_ISL_424944|2020-0	....	....	....	....	....	....
A	6030	hCoV-19/USA/NY-NYUMC138/2020|EPI_ISL_424945|2020-0	....	....	....	....	....	....
A	6031	hCoV-19/USA/NY-NYUMC139/2020|EPI_ISL_424946|2020-0	....	....	....	....	....	....
A	6032	hCoV-19/USA/NY-NYUMC140/2020|EPI_ISL_424947|2020-0	....	....	....	....	....	....
A	6033	hCoV-19/USA/NY-NYUMC141/2020|EPI_ISL_424948|2020-0	....	....	....	....	....	....
A	6034	hCoV-19/USA/NY-NYUMC142/2020|EPI_ISL_424949|2020-0	....	....	....	....	....	....
A	6035	hCoV-19/USA/NY-NYUMC143/2020|EPI_ISL_424950|2020-0	....	....	....	....	....	....
A	6036	hCoV-19/USA/NY-NYUMC144/2020|EPI_ISL_424951|2020-0	....	....	....	....	....	....
A	6037	hCoV-19/USA/NY-NYUMC145/2020|EPI_ISL_424952|2020-0	....	....	....	....	....	....
A	6038	hCoV-19/USA/NY-NYUMC146/2020|EPI_ISL_424953|2020-0	....	....	....	....	....	....
A	6039	hCoV-19/USA/NY-NYUMC147/2020|EPI_ISL_424954|2020-0	....	....	....	....	....	....
A	6040	hCoV-19/USA/NY-NYUMC148/2020|EPI_ISL_424955|2020-0	....	....	....	....	....	....
A	6041	hCoV-19/USA/NY-NYUMC149/2020|EPI_ISL_424956|2020-0	....	....	....	....	....	....
A	6042	hCoV-19/USA/NY-NYUMC150/2020|EPI_ISL_424957|2020-0	....	....	....	....	....	....
A	6043	hCoV-19/USA/NY-NYUMC151/2020|EPI_ISL_424958|2020-0	....	....	....	....	....	....
A	6044	hCoV-19/USA/NY-NYUMC152/2020|EPI_ISL_424959|2020-0	....	....	....	....	....	....
A	6045	hCoV-19/USA/NY-NYUMC153/2020|EPI_ISL_424960|2020-0	....	....	....	....	....	....
A	6046	hCoV-19/USA/NY-NYUMC154/2020|EPI_ISL_424961|2020-0	....	....	....	....	....	....
A	6047	hCoV-19/USA/NY-NYUMC155/2020|EPI_ISL_424962|2020-0	....	....	....	....	....	....
A	6048	hCoV-19/USA/NY-NYUMC156/2020|EPI_ISL_424963|2020-0	....	....	....	....	....	....
A	6049	hCoV-19/USA/NY-NYUMC157/2020|EPI_ISL_424964|2020-0	....	....	....	....	....	....

A	6050 hCoV-19/USA/NY-NYUMC158/2020|EPI_ISL_424965|2020-0	····················	····················	····················	····················	····················	····················
A	6051 hCoV-19/USA/NY-NYUMC159/2020|EPI_ISL_424966|2020-0	····················	····················	····················	····················	····················	····················
A	6052 hCoV-19/USA/NY-NYUMC160/2020|EPI_ISL_424967|2020-0	····················	····················	····················	····················	····················	····················
A	6053 hCoV-19/USA/NY-NYUMC161/2020|EPI_ISL_424968|2020-0	····················	····················	····················	····················	····················	····················
A	6054 hCoV-19/Taiwan/CGMH-CGU-13/2020|EPI_ISL_424969|202	····················	····················	····················	····················	····················	····················
A	6055 hCoV-19/Taiwan/CGMH-CGU-14/2020|EPI_ISL_424970|202	····················	····················	····················	····················	····················	····················
A	6056 hCoV-19/Taiwan/CGMH-CGU-15/2020|EPI_ISL_424971|202	····················	····················	····················	····················	····················	····················
A	6057 hCoV-19/Taiwan/CGMH-CGU-16/2020|EPI_ISL_424972|202	····················	····················	····················	····················	····················	····················
A	6058 hCoV-19/Taiwan/CGMH-CGU-17/2020|EPI_ISL_424973|202	····················	····················	····················	····················	····················	····················
A	6059 hCoV-19/Taiwan/CGMH-CGU-18/2020|EPI_ISL_424974|202	····················	····················	····················	····················	····················	····················
A	6060 hCoV-19/Taiwan/CGMH-CGU-19/2020|EPI_ISL_424975|202	····················	····················	····················	····················	····················	····················
A	6062 hCoV-19/Australia/VIC851/2020|EPI_ISL_427111|2020-	····················	····················	····················	····················	····················	····················
A	6063 hCoV-19/Australia/VIC853/2020|EPI_ISL_427113|2020-	····················	····················	····················	····················	····················	····················
A	6064 hCoV-19/Australia/VIC856/2020|EPI_ISL_427115|2020-	····················	····················	····················	····················	····················	····················
A	6065 hCoV-19/Australia/VIC857/2020|EPI_ISL_427116|2020-	····················	····················	····················	····················	····················	····················
A	6066 hCoV-19/Australia/VIC858/2020|EPI_ISL_427117|2020-	····················	····················	····················	····················	····················	····················
A	6067 hCoV-19/Australia/VIC860/2020|EPI_ISL_427118|2020-	····················	····················	····················	····················	····················	····················
A	6068 hCoV-19/Australia/VIC862/2020|EPI_ISL_427119|2020-	····················	····················	····················	····················	····················	····················
A	6069 hCoV-19/Australia/VIC863/2020|EPI_ISL_427120|2020-	····················	····················	····················	····················	····················	····················
A	6070 hCoV-19/Australia/VIC864/2020|EPI_ISL_427121|2020-	····················	····················	····················	····················	····················	····················
A	6071 hCoV-19/Australia/VIC867/2020|EPI_ISL_427122|2020-	····················	····················	····················	····················	····················	····················
A	6072 hCoV-19/Australia/VIC868/2020|EPI_ISL_427123|2020-	····················	····················	····················	····················	····················	····················
A	6073 hCoV-19/Australia/VIC869/2020|EPI_ISL_427124|2020-	····················	····················	····················	····················	····················	····················
A	6075 hCoV-19/Australia/VIC871/2020|EPI_ISL_427126|2020-	····················	····················	····················	····················	····················	····················
A	6076 hCoV-19/Australia/VIC873/2020|EPI_ISL_427128|2020-	····················	····················	····················	····················	····················	····················
A	6077 hCoV-19/Australia/VIC874/2020|EPI_ISL_427129|2020-	····················	····················	····················	····················	····················	····················
A	6078 hCoV-19/USA/ID-UW-4266/2020|EPI_ISL_427170|2020-03	····················	····················	····················	····················	····················	····················
A	6079 hCoV-19/USA/ID-UW-4462/2020|EPI_ISL_427171|2020-03	····················	····················	····················	····················	····················	····················
A	6080 hCoV-19/USA/ID-UW-4486/2020|EPI_ISL_427172|2020-03	····················	····················	····················	····················	····················	····················
A	6081 hCoV-19/USA/OR-UW-4300/2020|EPI_ISL_427173|2020-03	····················	····················	····················	····················	····················	····················
A	6082 hCoV-19/USA/WA-UW-3809/2020|EPI_ISL_427174|2020-03	····················	····················	····················	····················	····················	····················
A	6083 hCoV-19/USA/WA-UW-3810/2020|EPI_ISL_427175|2020-03	····················	····················	····················	····················	····················	····················
A	6084 hCoV-19/USA/WA-UW-3814/2020|EPI_ISL_427177|2020-03	····················	····················	····················	····················	····················	····················
A	6085 hCoV-19/USA/WA-UW-3815/2020|EPI_ISL_427178|2020-03	····················	····················	····················	····················	····················	····················
A	6086 hCoV-19/USA/WA-UW-3816/2020|EPI_ISL_427179|2020-03	····················	····················	····················	····················	····················	····················
A	6087 hCoV-19/USA/WA-UW-3819/2020|EPI_ISL_427180|2020-03	····················	····················	····················	····················	····················	····················
A	6088 hCoV-19/USA/WA-UW-3826/2020|EPI_ISL_427181|2020-03	····················	····················	····················	····················	····················	····················
A	6089 hCoV-19/USA/WA-UW-3827/2020|EPI_ISL_427182|2020-03	····················	····················	····················	····················	····················	····················
A	6090 hCoV-19/USA/WA-UW-3828/2020|EPI_ISL_427183|2020-03	····················	····················	····················	····················	····················	····················
A	6091 hCoV-19/USA/WA-UW-3829/2020|EPI_ISL_427184|2020-03	····················	····················	····················	····················	····················	····················
A	6092 hCoV-19/USA/WA-UW-3832/2020|EPI_ISL_427185|2020-03	····················	····················	····················	····················	····················	····················
A	6093 hCoV-19/USA/WA-UW-3834/2020|EPI_ISL_427187|2020-03	····················	····················	····················	····················	····················	····················
A	6094 hCoV-19/USA/WA-UW-3838/2020|EPI_ISL_427188|2020-03	····················	····················	····················	····················	····················	····················
A	6095 hCoV-19/USA/WA-UW-3839/2020|EPI_ISL_427189|2020-03	····················	····················	····················	····················	····················	····················
A	6096 hCoV-19/USA/WA-UW-3841/2020|EPI_ISL_427190|2020-03	····················	····················	····················	····················	····················	····················
A	6097 hCoV-19/USA/WA-UW-3843/2020|EPI_ISL_427191|2020-03	····················	····················	····················	····················	····················	····················
A	6098 hCoV-19/USA/WA-UW-3844/2020|EPI_ISL_427192|2020-03	····················	····················	····················	····················	····················	····················
A	6099 hCoV-19/USA/WA-UW-3850/2020|EPI_ISL_427193|2020-03	····················	····················	····················	····················	····················	····················
A	6100 hCoV-19/USA/WA-UW-3855/2020|EPI_ISL_427194|2020-03	····················	····················	····················	····················	····················	····················
A	6101 hCoV-19/USA/WA-UW-3858/2020|EPI_ISL_427195|2020-03	····················	····················	····················	····················	····················	····················
A	6102 hCoV-19/USA/WA-UW-3862/2020|EPI_ISL_427196|2020-03	····················	····················	····················	····················	····················	····················
A	6103 hCoV-19/USA/WA-UW-3868/2020|EPI_ISL_427197|2020-03	····················	····················	····················	····················	····················	····················
A	6104 hCoV-19/USA/WA-UW-3871/2020|EPI_ISL_427198|2020-03	····················	····················	····················	····················	····················	····················
A	6105 hCoV-19/USA/WA-UW-3880/2020|EPI_ISL_427199|2020-03	····················	····················	····················	····················	····················	····················
A	6106 hCoV-19/Guangdong/20SF1107/2020|EPI_ISL_428441|202	····················	····················	····················	····················	····················	····················
A	6107 hCoV-19/Guangdong/20SF1136/2020|EPI_ISL_428442|202	····················	····················	····················	····················	····················	····················
A	6108 hCoV-19/Guangdong/20SF115/2020|EPI_ISL_428443|2020	····················	····················	····················	····················	····················	····················
A	6109 hCoV-19/Guangdong/20SF1152/2020|EPI_ISL_428444|202	····················	·A··················	····················	····················	····················	····················
A	6110 hCoV-19/Guangdong/20SF1153/2020|EPI_ISL_428445|202	····················	·A··················	····················	····················	····················	····················
A	6111 hCoV-19/Guangdong/20SF1159/2020|EPI_ISL_428446|202	····················	····················	····················	····················	····················	····················
A	6112 hCoV-19/Guangdong/20SF117/2020|EPI_ISL_428447|2020	····················	····················	····················	····················	····················	····················
A	6113 hCoV-19/Guangdong/20SF118/2020|EPI_ISL_428448|2020	····················	····················	····················	····················	····················	····················
A	6114 hCoV-19/Guangdong/20SF123/2020|EPI_ISL_428449|2020	····················	····················	····················	····················	····················	····················
A	6115 hCoV-19/Guangdong/20SF190/2020|EPI_ISL_428452|2020	····················	·A··················	····················	····················	····················	····················
A	6116 hCoV-19/Guangdong/20SF198/2020|EPI_ISL_428454|2020	····················	····················	····················	····················	····················	····················
A	6117 hCoV-19/Guangdong/20SF200/2020|EPI_ISL_428455|2020	····················	····················	····················	····················	····················	····················
A	6118 hCoV-19/Guangdong/20SF243/2020|EPI_ISL_428456|2020	····················	····················	····················	····················	····················	····················
A	6119 hCoV-19/Guangdong/20SF254/2020|EPI_ISL_428457|2020	····················	····················	····················	····················	····················	····················
A	6120 hCoV-19/Guangdong/20SF2546/2020|EPI_ISL_428458|202	····················	····················	····················	····················	····················	····················
A	6121 hCoV-19/Luxembourg/LNS4530810/2020|EPI_ISL_429770|	····················	····················	····················	····················	····················	····················
A	6122 hCoV-19/Luxembourg/LNS4107430/2020|EPI_ISL_429772|	····················	····················	····················	····················	····················	····················
A	6123 hCoV-19/Luxembourg/LNS3257811/2020|EPI_ISL_429773|	····················	····················	····················	····················	····················	····················
A	6124 hCoV-19/Luxembourg/LNS5611124/2020|EPI_ISL_429774|	····················	····················	····················	····················	····················	····················
A	6125 hCoV-19/Luxembourg/LNS7299024/2020|EPI_ISL_429775|	····················	····················	····················	····················	····················	····················
A	6126 hCoV-19/Luxembourg/LNS8726389/2020|EPI_ISL_429776|	····················	····················	····················	····················	····················	····················
A	6127 hCoV-19/Luxembourg/LNS0994857/2020|EPI_ISL_429777|	····················	····················	····················	····················	····················	····················
A	6128 hCoV-19/Luxembourg/LNS2580723/2020|EPI_ISL_429778|	····················	····················	····················	····················	····················	····················
A	6129 hCoV-19/Luxembourg/LNS7357097/2020|EPI_ISL_429779|	····················	····················	····················	····················	····················	····················

A 6130 hCoV-19/Luxembourg/LNS5680075/2020|EPI_ISL_429780| ....
A 6131 hCoV-19/Luxembourg/LNS0709551/2020|EPI_ISL_429781| ....
A 6132 hCoV-19/Luxembourg/LNS3169999/2020|EPI_ISL_429782| ....
A 6133 hCoV-19/Luxembourg/LNS5677421/2020|EPI_ISL_429784| ....
A 6134 hCoV-19/Luxembourg/LNS4818581/2020|EPI_ISL_429786| ....
A 6135 hCoV-19/Luxembourg/LNS3283768/2020|EPI_ISL_429787| ....
A 6136 hCoV-19/Guangzhou/IQTC03/2020|EPI_ISL_529149|2020- ....
A 6137 hCoV-19/Guangzhou/IQTC04/2020|EPI_ISL_529150|2020- ....
A 6138 hCoV-19/USA/CT-UW420/2020|EPI_ISL_529155|2020-03-2 ....
A 6139 hCoV-19/USA/CT-UW423/2020|EPI_ISL_529156|2020-03-2 ....
A 6140 hCoV-19/USA/CT-UW427/2020|EPI_ISL_529157|2020-03-2 ....
A 6141 hCoV-19/USA/CT-UW430/2020|EPI_ISL_529159|2020-03-2 ....
A 6142 hCoV-19/USA/ID-UW-2173/2020|EPI_ISL_529160|2020-03 ....
A 6143 hCoV-19/USA/ID-UW-2195/2020|EPI_ISL_529161|2020-03 ....
A 6144 hCoV-19/USA/OR-UW409/2020|EPI_ISL_529170|2020-03-2 ....
A 6145 hCoV-19/Brazil/BA-312/2020|EPI_ISL_415105|2020-03- ....
A 6146 hCoV-19/England/20099079106/2020|EPI_ISL_415130|20 ....
A 6147 hCoV-19/England/20100004706/2020|EPI_ISL_415133|20 ....
A 6148 hCoV-19/England/20100004806/2020|EPI_ISL_415134|20 ....
A 6150 hCoV-19/England/20102000206/2020|EPI_ISL_415145|20 ....Y........
A 6151 hCoV-19/England/20102000506/2020|EPI_ISL_415147|20 ....
A 6154 hCoV-19/USA/NY2-PV08100/2020|EPI_ISL_415151|2020-0 ....
A 6155 hCoV-19/Panama/328677/2020|EPI_ISL_415152|2020-03- ....
A 6156 hCoV-19/Belgium/VLM-03011/2020|EPI_ISL_415153|2020 ....
A 6157 hCoV-19/Belgium/BM-03012/2020|EPI_ISL_415154|2020- ....
A 6158 hCoV-19/Belgium/VAG-03013/2020|EPI_ISL_415155|2020 ....
A 6159 hCoV-19/Belgium/SH-03014/2020|EPI_ISL_415156|2020- ....
A 6160 hCoV-19/Belgium/BC-03016/2020|EPI_ISL_415157|2020- ....
A 6161 hCoV-19/Belgium/QKJ-03015/2020|EPI_ISL_415158|2020 ....
A 6162 hCoV-19/Belgium/BA-02291/2020|EPI_ISL_415159|2020- ....
A 6163 hCoV-19/Shanghai/SH0112/2020|EPI_ISL_416400|2020-0 ....
A 6164 hCoV-19/Shanghai/SH0114/2020|EPI_ISL_416401|2020-0 ....
A 6165 hCoV-19/Shanghai/SH0115/2020|EPI_ISL_416402|2020-0 ....
A 6166 hCoV-19/Shanghai/SH0117/2020|EPI_ISL_416403|2020-0 ....
A 6167 hCoV-19/Shanghai/SH0119/2020|EPI_ISL_416404|2020-0 ....
A 6168 hCoV-19/Shanghai/SH0121/2020|EPI_ISL_416405|2020-0 ....
A 6169 hCoV-19/Shanghai/SH0125/2020|EPI_ISL_416406|2020-0 ....
A 6170 hCoV-19/Shanghai/SH0126/2020|EPI_ISL_416407|2020-0 ....
A 6171 hCoV-19/Shanghai/SH0128/2020|EPI_ISL_416409|2020-0 ....
A 6172 hCoV-19/Australia/VIC02/2020|EPI_ISL_416410|2020-0 ....
A 6173 hCoV-19/Australia/VIC03/2020|EPI_ISL_416411|2020-0 ....
A 6174 hCoV-19/Australia/VIC04/2020|EPI_ISL_416412|2020-0 ....
A 6175 hCoV-19/Australia/VIC05/2020|EPI_ISL_416413|2020-0 ....
A 6176 hCoV-19/Australia/VIC07/2020|EPI_ISL_416415|2020-0 ....
A 6177 hCoV-19/Hungary/mbl1/2020|EPI_ISL_416426|2020-03-1 ....
A 6178 hCoV-19/Vietnam/38142/2020|EPI_ISL_416427|2020-01- ....
A 6179 hCoV-19/Vietnam/39607/2020|EPI_ISL_416428|2020-03- ....
A 6180 hCoV-19/Vietnam/CM99/2020|EPI_ISL_416429|2020-02-1 ....
A 6181 hCoV-19/Vietnam/CM295/2020|EPI_ISL_416430|2020-03- ....
A 6182 hCoV-19/Vietnam/CM296/2020|EPI_ISL_416431|2020-03- ....
A 6183 hCoV-19/Saudi Arabia/KAIMRC-Alghoribi/2020|EPI_ISL ....
A 6184 hCoV-19/USA/WA-UW77/2020|EPI_ISL_416433|2020-03-10 ....
A 6185 hCoV-19/USA/WA-UW78/2020|EPI_ISL_416434|2020-03-10 ....
A 6186 hCoV-19/USA/WA-UW79/2020|EPI_ISL_416435|2020-03-10 ....
A 6187 hCoV-19/USA/WA-UW80/2020|EPI_ISL_416436|2020-03-10 ....
A 6188 hCoV-19/USA/WA-UW81/2020|EPI_ISL_416437|2020-03-10 ....
A 6189 hCoV-19/USA/WA-UW82/2020|EPI_ISL_416438|2020-03-10 ....
A 6190 hCoV-19/USA/WA-UW83/2020|EPI_ISL_416439|2020-03-10 ....
A 6191 hCoV-19/USA/WA-S5/2020|EPI_ISL_416460|2020-02-29 ....
A 6192 hCoV-19/USA/WA-S6/2020|EPI_ISL_416461|2020-02-29 ....
A 6193 hCoV-19/USA/WA-S7/2020|EPI_ISL_416462|2020-02-24 ....
A 6194 hCoV-19/USA/WA-S10/2020|EPI_ISL_416465|2020-02-29 ....
A 6195 hCoV-19/USA/WA-S11/2020|EPI_ISL_416466|2020-03-03 ....
A 6196 hCoV-19/Belgium/MTR-03021/2020|EPI_ISL_416467|2020 ....
A 6197 hCoV-19/Belgium/GMH-03022/2020|EPI_ISL_416468|2020 ....
A 6198 hCoV-19/Belgium/SN-03031/2020|EPI_ISL_416469|2020- ....
A 6199 hCoV-19/Belgium/MTR-03026/2020|EPI_ISL_416476|2020 ....
A 6200 hCoV-19/Georgia/Tb-390/2020|EPI_ISL_416477|2020-03 ....
A 6201 hCoV-19/Georgia/Tb-537/2020|EPI_ISL_416480|2020-03 ....
A 6202 hCoV-19/Georgia/Tb-712/2020|EPI_ISL_416481|2020-03 ....
A 6203 hCoV-19/Georgia/Tb/2020|EPI_ISL_416482|2020-03-13 ....
A 6204 hCoV-19/Spain/VC-FISABIO-5/2020|EPI_ISL_416484|202 ....
A 6205 hCoV-19/Spain/VC-FISABIO-6/2020|EPI_ISL_416485|202 ....
A 6206 hCoV-19/Spain/VC-FISABIO-7/2020|EPI_ISL_416486|202 ....
A 6207 hCoV-19/Spain/VC-FISABIO-8/2020|EPI_ISL_416487|202 ....
A 6208 hCoV-19/Poland/PL_P1/2020|EPI_ISL_416488|2020-03-0 ....
A 6209 hCoV-19/USA/WI-UW-02/2020|EPI_ISL_416489|2020-03-1 ....
A 6210 hCoV-19/USA/WI-UW-04/2020|EPI_ISL_416491|2020-03-1 ....

A	6211 hCoV-19/USA/WI-UW-05/2020|EPI_ISL_416492|2020-03-1	····················	····················	····················	····················	····················	····················		
A	6212 hCoV-19/France/HDF-2196/2020|EPI_ISL_416493|2020-0	····················	····················	····················	····················	····················	····················		
A	6213 hCoV-19/France/NOR-N2223/2020|EPI_ISL_416494|2020-	····················	····················	····················	····················	····················	····················		
A	6214 hCoV-19/France/HDF-2234/2020|EPI_ISL_416495|2020-0	····················	····················	····················	····················	····················	····················		
A	6215 hCoV-19/France/HDF-2237/2020|EPI_ISL_416496|2020-0	····················	····················	····················	····················	····················	····················		
A	6216 hCoV-19/France/HDF-2239/2020|EPI_ISL_416497|2020-0	····················	····················	····················	····················	····················	····················		
A	6217 hCoV-19/France/IDF-2256/2020|EPI_ISL_416498|2020-0	····················	····················	····················	····················	····················	····················		
A	6218 hCoV-19/France/IDF-2278/2020|EPI_ISL_416499|2020-0	····················	····················	····················	····················	····················	····················		
A	6219 hCoV-19/Iceland/173/2020|EPI_ISL_417700|2020-03-15	····················	····················	····················	····················	····················	····················		
A	6222 hCoV-19/Iceland/179/2020|EPI_ISL_417703|2020-03-16	····················	····················	····················	····················	····················	····················		
A	6223 hCoV-19/Iceland/92/2020|EPI_ISL_417704|2020-03-10	····················	····················	····················	····················	····················	················N·NN	1	1
A	6224 hCoV-19/Iceland/65/2020|EPI_ISL_417705|2020-03-07	····················	····················	····················	····················	····················	····················		
A	6225 hCoV-19/Iceland/199/2020|EPI_ISL_417706|2020-03-16	····················	····················	····················	····················	····················	····················		
A	6226 hCoV-19/Iceland/189/2020|EPI_ISL_417709|2020-03-16	····················	····················	····················	····················	····················	····················		
A	6227 hCoV-19/Iceland/150/2020|EPI_ISL_417711|2020-03-13	····················	····················	····················	····················	····················	····················		
A	6228 hCoV-19/Iceland/172/2020|EPI_ISL_417712|2020-03-15	····················	····················	····················	····················	····················	····················		
A	6229 hCoV-19/Iceland/130/2020|EPI_ISL_417713|2020-03-12	····················	····················	····················	····················	····················	····················		
A	6230 hCoV-19/Iceland/129/2020|EPI_ISL_417714|2020-03-12	····················	····················	····················	····················	····················	····················		
A	6231 hCoV-19/Iceland/45/2020|EPI_ISL_417715|2020-03-04	····················	····················	····················	····················	····················	····················		
A	6232 hCoV-19/Iceland/174/2020|EPI_ISL_417716|2020-03-16	····················	····················	····················	····················	····················	····················		
A	6233 hCoV-19/Iceland/195/2020|EPI_ISL_417717|2020-03-16	····················	····················	····················	····················	····················	····················		
A	6234 hCoV-19/Iceland/49/2020|EPI_ISL_417718|2020-03-05	····················	····················	····················	····················	····················	····················		
A	6235 hCoV-19/Iceland/138/2020|EPI_ISL_417720|2020-03-12	····················	····················	····················	····················	····················	····················		
A	6236 hCoV-19/Iceland/27/2020|EPI_ISL_417721|2020-03-03	····················	····················	····················	····················	····················	····················		
A	6237 hCoV-19/Iceland/110/2020|EPI_ISL_417722|2020-03-11	····················	····················	····················	····················	····················	····················		
A	6238 hCoV-19/Iceland/182/2020|EPI_ISL_417724|2020-03-16	····················	····················	····················	····················	····················	····················		
A	6239 hCoV-19/Iceland/161/2020|EPI_ISL_417725|2020-03-14	····················	····················	····················	····················	····················	····················		
A	6241 hCoV-19/Iceland/127/2020|EPI_ISL_417727|2020-03-12	····················	····················	····················	····················	····················	····················		
A	6242 hCoV-19/Iceland/70/2020|EPI_ISL_417728|2020-03-08	····················	····················	····················	····················	····················	····················		
A	6243 hCoV-19/Iceland/93/2020|EPI_ISL_417730|2020-03-10	····················	····················	····················	····················	····················	····················		
A	6244 hCoV-19/Iceland/61/2020|EPI_ISL_417731|2020-03-06	····················	····················	····················	····················	····················	····················		
A	6245 hCoV-19/Iceland/94/2020|EPI_ISL_417732|2020-03-10	····················	····················	····················	····················	····················	····················		
A	6246 hCoV-19/Iceland/207/2020|EPI_ISL_417733|2020-03-16	····················	····················	····················	····················	····················	····················		
A	6247 hCoV-19/Iceland/22/2020|EPI_ISL_417734|2020-03-02	····················	····················	····················	····················	····················	····················		
A	6248 hCoV-19/Iceland/134/2020|EPI_ISL_417735|2020-03-12	····················	····················	····················	····················	····················	····················		
A	6249 hCoV-19/Iceland/14/2020|EPI_ISL_417736|2020-03-01	····················	····················	····················	····················	····················	····················		
A	6250 hCoV-19/Iceland/155/2020|EPI_ISL_417737|2020-03-15	····················	····················	····················	····················	····················	····················		
A	6251 hCoV-19/Iceland/89/2020|EPI_ISL_417738|2020-03-10	····················	····················	····················	····················	····················	····················		
A	6252 hCoV-19/Iceland/5/2020|EPI_ISL_417739|2020-03-13	····················	····················	····················	····················	····················	····················		
A	6253 hCoV-19/Iceland/7/2020|EPI_ISL_417740|2020-03-15	····················	····················	····················	····················	····················	····················		
A	6254 hCoV-19/Iceland/145/2020|EPI_ISL_417741|2020-03-13	····················	····················	····················	····················	····················	····················		
A	6255 hCoV-19/Iceland/197/2020|EPI_ISL_417742|2020-03-16	····················	····················	····················	····················	····················	····················		
A	6256 hCoV-19/Iceland/200/2020|EPI_ISL_417743|2020-03-16	····················	····················	····················	····················	····················	····················		
A	6257 hCoV-19/Iceland/137/2020|EPI_ISL_417744|2020-03-13	····················	····················	····················	····················	····················	····················		
A	6258 hCoV-19/Iceland/152/2020|EPI_ISL_417745|2020-03-13	····················	····················	····················	····················	····················	····················		
A	6259 hCoV-19/Iceland/183/2020|EPI_ISL_417746|2020-03-16	····················	····················	····················	····················	····················	····················		
A	6261 hCoV-19/Iceland/109/2020|EPI_ISL_417748|2020-03-11	····················	····················	····················	····················	····················	····················		
A	6262 hCoV-19/Iceland/69/2020|EPI_ISL_417749|2020-03-08	····················	····················	····················	····················	····················	····················		
A	6263 hCoV-19/Iceland/202/2020|EPI_ISL_417750|2020-03-16	····················	····················	····················	····················	····················	····················		
A	6264 hCoV-19/Iceland/213/2020|EPI_ISL_417752|2020-03-16	····················	····················	····················	····················	····················	····················		
A	6265 hCoV-19/Iceland/180/2020|EPI_ISL_417753|2020-03-16	····················	····················	····················	····················	····················	····················		
A	6266 hCoV-19/Iceland/226/2020|EPI_ISL_417754|2020-03-16	····················	····················	····················	····················	····················	····················		
A	6267 hCoV-19/Iceland/169/2020|EPI_ISL_417755|2020-03-13	····················	····················	····················	····················	····················	····················		
A	6268 hCoV-19/Iceland/62/2020|EPI_ISL_417757|2020-03-06	····················	····················	····················	····················	····················	····················		
A	6269 hCoV-19/Iceland/164/2020|EPI_ISL_417758|2020-03-14	····················	····················	····················	····················	····················	····················		
A	6270 hCoV-19/Iceland/166/2020|EPI_ISL_417759|2020-03-13	····················	····················	····················	····················	····················	····················		
A	6271 hCoV-19/Iceland/84/2020|EPI_ISL_417761|2020-03-10	····················	····················	····················	····················	····················	····················		
A	6272 hCoV-19/Iceland/191/2020|EPI_ISL_417762|2020-03-16	····················	····················	····················	····················	····················	····················		
A	6273 hCoV-19/Iceland/211/2020|EPI_ISL_417763|2020-03-16	····················	····················	····················	····················	····················	····················		
A	6274 hCoV-19/Iceland/209/2020|EPI_ISL_417764|2020-03-16	····················	····················	····················	····················	····················	····················		
A	6275 hCoV-19/Iceland/13/2020|EPI_ISL_417765|2020-02-27	····················	····················	····················	····················	····················	····················		
A	6276 hCoV-19/Iceland/208/2020|EPI_ISL_417766|2020-03-16	····················	·A··················	····················	····················	····················	····················		
A	6278 hCoV-19/Iceland/139/2020|EPI_ISL_417790|2020-03-12	····················	····················	····················	····················	····················	····················		
A	6279 hCoV-19/Iceland/140/2020|EPI_ISL_417791|2020-03-12	····················	····················	····················	····················	····················	····················		
A	6280 hCoV-19/Iceland/141/2020|EPI_ISL_417792|2020-03-13	····················	····················	····················	····················	····················	····················		
A	6281 hCoV-19/Iceland/142/2020|EPI_ISL_417793|2020-03-13	····················	····················	····················	····················	····················	····················		
A	6282 hCoV-19/Iceland/144/2020|EPI_ISL_417795|2020-03-13	····················	····················	····················	····················	····················	····················		
A	6283 hCoV-19/Iceland/146/2020|EPI_ISL_417796|2020-03-13	····················	····················	····················	····················	····················	····················		
A	6284 hCoV-19/Iceland/147/2020|EPI_ISL_417797|2020-03-13	····················	····················	····················	····················	····················	····················		
A	6285 hCoV-19/Iceland/148/2020|EPI_ISL_417798|2020-03-13	····················	·A··················	····················	····················	····················	····················		
A	6286 hCoV-19/Iceland/149/2020|EPI_ISL_417799|2020-03-13	····················	····················	····················	····················	····················	····················		
A	6287 hCoV-19/Australia/VIC836/2020|EPI_ISL_427100|2020-	····················	····················	····················	····················	····················	····················		
A	6288 hCoV-19/Australia/VIC837/2020|EPI_ISL_427101|2020-	····················	····················	····················	····················	····················	····················		
A	6289 hCoV-19/Australia/VIC842/2020|EPI_ISL_427102|2020-	····················	····················	····················	····················	····················	····················		
A	6290 hCoV-19/Australia/VIC843/2020|EPI_ISL_427103|2020-	····················	····················	····················	····················	····················	····················		
A	6291 hCoV-19/Australia/VIC845/2020|EPI_ISL_427105|2020-	····················	····················	····················	····················	····················	····················		
A	6292 hCoV-19/Australia/VIC846/2020|EPI_ISL_427106|2020-	····················	····················	····················	····················	····················	····················		
A	6293 hCoV-19/Australia/VIC847/2020|EPI_ISL_427107|2020-	····················	····················	····················	····················	····················	····················		

A	6294 hCoV-19/Australia/VIC848/2020|EPI_ISL_427108|2020-	....	....	....	....	....	....
A	6295 hCoV-19/Australia/VIC849/2020|EPI_ISL_427109|2020-	....	....	....	....	....	....
A	6296 hCoV-19/Australia/VIC875/2020|EPI_ISL_427130|2020-	....	....	....	....	....	....
A	6297 hCoV-19/Australia/VIC876/2020|EPI_ISL_427131|2020-	....	....	....	....	....	....
A	6298 hCoV-19/Australia/VIC878/2020|EPI_ISL_427133|2020-	....	....	....	....	....	....
A	6299 hCoV-19/Australia/VIC879/2020|EPI_ISL_427134|2020-	....	....	....	....	....	....
A	6300 hCoV-19/Australia/VIC881/2020|EPI_ISL_427135|2020-	....	....	....	....	....	....
A	6301 hCoV-19/Australia/VIC883/2020|EPI_ISL_427136|2020-	....	....	....	....	....	....
A	6303 hCoV-19/Australia/VIC886/2020|EPI_ISL_427139|2020-	....	....	....	....	....	....
A	6304 hCoV-19/Australia/VIC887/2020|EPI_ISL_427140|2020-	....	....	....	....	....	....
A	6306 hCoV-19/Australia/VIC893/2020|EPI_ISL_427145|2020-	....	....	....	....	....	....
A	6308 hCoV-19/Australia/VIC895/2020|EPI_ISL_427147|2020-	....	....	....	....	....	....
A	6309 hCoV-19/Australia/VIC898/2020|EPI_ISL_427148|2020-	....	....	....	....	....	....
A	6310 hCoV-19/Australia/VIC899/2020|EPI_ISL_427149|2020-	....	....	....	....	....	....
A	6315 hCoV-19/Australia/VIC931/2020|EPI_ISL_427159|2020-	....	....	....	....	....	....
A	6316 hCoV-19/Australia/VIC932/2020|EPI_ISL_427160|2020-	....	....	....	....	....	....
A	6317 hCoV-19/USA/CT-UW-3845/2020|EPI_ISL_427161|2020-03	....	....	....	....	....	....
A	6318 hCoV-19/USA/CT-UW-4238/2020|EPI_ISL_427162|2020-03	....	....	....	....	....	....
A	6319 hCoV-19/USA/CT-UW-4244/2020|EPI_ISL_427163|2020-03	....	....	....	....	....	....
A	6320 hCoV-19/USA/CT-UW-4251/2020|EPI_ISL_427164|2020-03	....	....	....	....	....	....
A	6322 hCoV-19/USA/ID-UW-3888/2020|EPI_ISL_427166|2020-03	....	....	....	....	....	....
A	6323 hCoV-19/USA/ID-UW-3892/2020|EPI_ISL_427167|2020-03	....	....	....	....	....	....
A	6324 hCoV-19/USA/ID-UW-4239/2020|EPI_ISL_427169|2020-03	....	....	....	....	....	....
A	6325 hCoV-19/Guangdong/20SF273/2020|EPI_ISL_428460|2020	....	....	....	....	....	....
A	6327 hCoV-19/Guangdong/20SF4047/2020|EPI_ISL_428462|202	....	....	....	....	....	....
A	6328 hCoV-19/Guangdong/20SF4051/2020|EPI_ISL_428463|202	....	....	....	....	....	....
A	6329 hCoV-19/Guangdong/20SF602/2020|EPI_ISL_428464|2020	....	....	....	....	....	....
A	6330 hCoV-19/Guangdong/20SF616/2020|EPI_ISL_428465|2020	....	....	....	....	....	....
A	6331 hCoV-19/Guangdong/20SF629/2020|EPI_ISL_428466|2020	....	....	....	....	....	....
A	6332 hCoV-19/Guangdong/20SF630/2020|EPI_ISL_428467|2020	....	....	....	....	....	....
A	6333 hCoV-19/Guangdong/20SF632/2020|EPI_ISL_428468|2020	....	....	....	....	....	....
A	6334 hCoV-19/Guangdong/20SF665/2020|EPI_ISL_428469|2020	....	....	....	....	....	....
A	6335 hCoV-19/Guangdong/20SF684/2020|EPI_ISL_428470|2020	....	....	....	....	....	....
A	6336 hCoV-19/Guangdong/20SF685/2020|EPI_ISL_428471|2020	....	....	....	....	....	....
A	6337 hCoV-19/Guangdong/20SF753/2020|EPI_ISL_428472|2020	....	....	....	....	....	....
A	6338 hCoV-19/Guangdong/20SF758/2020|EPI_ISL_428473|2020	....	....	....	....	....	....
A	6339 hCoV-19/Guangdong/20SF808/2020|EPI_ISL_428474|2020	....	....	....	....	....	....
A	6340 hCoV-19/Guangdong/20SF812/2020|EPI_ISL_428475|2020	....	....	....	....	....	....
A	6341 hCoV-19/Guangdong/20SF813/2020|EPI_ISL_428476|2020	....	....	....	....	....	....
A	6342 hCoV-19/Guangdong/20SF822/2020|EPI_ISL_428477|2020	....	....	....	....	....	....
A	6343 hCoV-19/Guangdong/20SF840/2020|EPI_ISL_428478|2020	....	....	....	....	....	....
A	6344 hCoV-19/India/KA-nimh-0113/2020|EPI_ISL_428479|202	....	....	....	....	....	....
A	6346 hCoV-19/India/KA-nimh-0130/2020|EPI_ISL_428481|202	....	....	....	....	....	....
A	6347 hCoV-19/India/KA-nimh-0182/2020|EPI_ISL_428482|202	....	....	....	....	....	....
A	6348 hCoV-19/India/KA-nimh-0318/2020|EPI_ISL_428483|202	....	....	....	....	....	....
A	6349 hCoV-19/India/KA-nimh-0351/2020|EPI_ISL_428484|202	....	....	....	....	....	....
A	6350 hCoV-19/India/KA-nimh-0996/2020|EPI_ISL_428486|202	....	....	....	....	....	....
A	6351 hCoV-19/India/KA-nimh-1071/2020|EPI_ISL_428487|202	....	....	....	....	....	....
A	6352 hCoV-19/Taiwan/8/2020|EPI_ISL_428488|2020-01-31	....	....	....	....	....	....
A	6353 hCoV-19/Taiwan/5/2020|EPI_ISL_428489|2020-01-31	....	....	....	....	....	....
A	6354 hCoV-19/Taiwan/78/2020|EPI_ISL_428490|2020-03-16	....	....	....	....	....	....
A	6355 hCoV-19/Taiwan/58/2020|EPI_ISL_428491|2020-03-15	....	....	....	....	....	....
A	6357 hCoV-19/Croatia/AU-S10/2020|EPI_ISL_429705|2020-03	....	....	....	....	....	....
A	6358 hCoV-19/Luxembourg/LNS3652733/2020|EPI_ISL_429706|	....	....	....	....	....	....
A	6359 hCoV-19/Luxembourg/LNS4641661/2020|EPI_ISL_429707|	....	....	....	....	....	....
A	6360 hCoV-19/Luxembourg/LNS5041225/2020|EPI_ISL_429710|	....	....	....	....	....	....
A	6361 hCoV-19/Luxembourg/LNS5519604/2020|EPI_ISL_429711|	....	....	....	....	....	....
A	6362 hCoV-19/Luxembourg/LNS9831617/2020|EPI_ISL_429713|	....	....	....	....	....	....
A	6363 hCoV-19/Luxembourg/LNS7469301/2020|EPI_ISL_429715|	....	....	....	....	....	....
A	6364 hCoV-19/Luxembourg/LNS7032690/2020|EPI_ISL_429716|	....	....	....	....	....	....
A	6365 hCoV-19/Luxembourg/LNS1995247/2020|EPI_ISL_429718|	....	....	....	....	....	....
A	6366 hCoV-19/Luxembourg/LNS5277361/2020|EPI_ISL_429723|	....	....	....	....	....	....
A	6367 hCoV-19/Luxembourg/LNS3212178/2020|EPI_ISL_429728|	....	....	....	....	....	....
A	6368 hCoV-19/Luxembourg/LNS0481305/2020|EPI_ISL_429729|	....	....	....	....	....	....
A	6369 hCoV-19/Luxembourg/LNS7438855/2020|EPI_ISL_429730|	....	....	....	....	....	....
A	6370 hCoV-19/Luxembourg/LNS7580831/2020|EPI_ISL_429732|	....	....	....	....	....	....
A	6371 hCoV-19/Luxembourg/LNS4423730/2020|EPI_ISL_429733|	....	....	....	....	....	....
A	6372 hCoV-19/Luxembourg/LNS6783622/2020|EPI_ISL_429734|	....	....	....	....	....	....
A	6373 hCoV-19/Luxembourg/LNS5358505/2020|EPI_ISL_429735|	....	....	....	....	....	....
A	6374 hCoV-19/Luxembourg/LNS7640155/2020|EPI_ISL_429736|	....	....	....	....	....	....
A	6375 hCoV-19/Luxembourg/LNS4254328/2020|EPI_ISL_429737|	....	....	....	....	....	....
A	6376 hCoV-19/Luxembourg/LNS5488745/2020|EPI_ISL_429738|	....	....	....	....	....	....
A	6377 hCoV-19/Luxembourg/LNS5549490/2020|EPI_ISL_429740|	....	....	....	....	....	....
A	6378 hCoV-19/Luxembourg/LNS6533667/2020|EPI_ISL_429741|	....	....	....	....	....	....
A	6379 hCoV-19/Luxembourg/LNS1658150/2020|EPI_ISL_429742|	....	....	....	....	....	....
A	6380 hCoV-19/Luxembourg/LNS6325758/2020|EPI_ISL_429743|	....	....	....	....	....	....
A	6381 hCoV-19/Luxembourg/LNS2913521/2020|EPI_ISL_429746|	....	....	....	....	....	....
A	6382 hCoV-19/Luxembourg/LNS4830703/2020|EPI_ISL_429748|	....	....	....	....	....	....

A	6383 hCoV-19/Luxembourg/LNS3989293/2020|EPI_ISL_429749|	....	....	....	....	....	....	
A	6384 hCoV-19/Luxembourg/LNS9598665/2020|EPI_ISL_429750|	....	....	....	....	....	....	
A	6385 hCoV-19/Luxembourg/LNS4245799/2020|EPI_ISL_429753|	....	....	....	....	....	....	
A	6386 hCoV-19/Luxembourg/LNS8772889/2020|EPI_ISL_429755|	....	....	....	....	....	....	
A	6387 hCoV-19/Luxembourg/LNS4946091/2020|EPI_ISL_429756|	....	....	....	....	....	....	
A	6388 hCoV-19/Luxembourg/LNS7006156/2020|EPI_ISL_429757|	....	....	....	....	....	....	
A	6389 hCoV-19/Luxembourg/LNS8167314/2020|EPI_ISL_429758|	....	....	....	....	....	....	
A	6390 hCoV-19/Luxembourg/LNS9455951/2020|EPI_ISL_429759|	....	....	....	....	....	....	
A	6391 hCoV-19/Luxembourg/LNS0211710/2020|EPI_ISL_429760|	....	....	....	....	....	....	
A	6392 hCoV-19/Luxembourg/LNS6277439/2020|EPI_ISL_429762|	....	....	....	....	....	....	
A	6393 hCoV-19/Luxembourg/LNS1703112/2020|EPI_ISL_429764|	....	....	....	....	....	....	
A	6394 hCoV-19/Luxembourg/LNS0814393/2020|EPI_ISL_429766|	....	....	....	....	....	....	
A	6395 hCoV-19/Luxembourg/LNS3437586/2020|EPI_ISL_429767|	....	....	....	....	....	....	
A	6396 hCoV-19/Luxembourg/LNS7721101/2020|EPI_ISL_429769|	....	....	....	....	....	....	
A	6397 hCoV-19/Luxembourg/LNS7007586/2020|EPI_ISL_429791|	....	....	....	....	....	....	
A	6398 hCoV-19/Croatia/LG-S2/2020|EPI_ISL_429792|2020-03	....	....	....	....	....	....	
A	6399 hCoV-19/Luxembourg/LNS7812353/2020|EPI_ISL_429793|	....	....	....	....	....	....	
A	6400 hCoV-19/Luxembourg/LNS6479831/2020|EPI_ISL_429794|	....	....	....	....	....	....	
A	6401 hCoV-19/Taiwan/NTU02/2020|EPI_ISL_410218|2020-02-0	....	....	....	....	....	....	
A	6402 hCoV-19/Wuhan/HBCDC-HB-02/2019|EPI_ISL_412898|2019	....	....	....	....	....	....	
A	6403 hCoV-19/Wuhan/HBCDC-HB-03/2019|EPI_ISL_412899|2019	....	....	....	....	....	....	
A	6404 hCoV-19/Japan/DP0005/2020|EPI_ISL_416565|2020-02-1	....	....	....	....	....	....	
A	6405 hCoV-19/Japan/DP0027/2020|EPI_ISL_416566|2020-02-1	....	....	....	....	....	....	
A	6406 hCoV-19/Japan/DP0037/2020|EPI_ISL_416567|2020-02-1	....	....	....	....	....	....	
A	6407 hCoV-19/Japan/DP0059/2020|EPI_ISL_416569|2020-02-1	....	....	....	....	....	....	
A	6408 hCoV-19/Japan/DP0065/2020|EPI_ISL_416570|2020-02-1	....	....	....	....	....	....	
A	6409 hCoV-19/Japan/DP0077/2020|EPI_ISL_416571|2020-02-1	....	....	....	....	....	....	
A	6410 hCoV-19/Japan/DP0078/2020|EPI_ISL_416572|2020-02-1	....	....	....	....	....	....	
A	6411 hCoV-19/Japan/DP0104/2020|EPI_ISL_416573|2020-02-1	....	....	....	....	....	....	
A	6412 hCoV-19/Japan/DP0107/2020|EPI_ISL_416574|2020-02-1	....	....	....	....	....	....	
A	6413 hCoV-19/Japan/DP0121/2020|EPI_ISL_416575|2020-02-1	....	....	....	....	....	....	
A	6414 hCoV-19/Japan/DP0133/2020|EPI_ISL_416576|2020-02-1	....	....	....	....	....	....	
A	6415 hCoV-19/Japan/DP0134/2020|EPI_ISL_416577|2020-02-1	....	....	....	....	....	....	
A	6416 hCoV-19/Japan/DP0152/2020|EPI_ISL_416578|2020-02-1	....	....	....	....	....	....	
A	6417 hCoV-19/Japan/DP0158/2020|EPI_ISL_416579|2020-02-1	....	....	....	....	....	....	
A	6418 hCoV-19/Japan/DP0184/2020|EPI_ISL_416580|2020-02-1	....	....	....	....	....	....	
A	6419 hCoV-19/Japan/DP0190/2020|EPI_ISL_416581|2020-02-1	....	....	....	....	....	....	
A	6420 hCoV-19/Japan/DP0191/2020|EPI_ISL_416582|2020-02-1	....	....	....	....	....	....	
A	6421 hCoV-19/Japan/DP0196/2020|EPI_ISL_416583|2020-02-1	....	....	....	....	....	....	
A	6422 hCoV-19/Japan/DP0200/2020|EPI_ISL_416584|2020-02-1	....	....	....	....	....	....	
A	6423 hCoV-19/Japan/DP0236/2020|EPI_ISL_416585|2020-02-1	....	....	....	....	....	....	
A	6424 hCoV-19/Japan/DP0274/2020|EPI_ISL_416586|2020-02-1	....	....	....	....	....	....	
A	6425 hCoV-19/Japan/DP0278/2020|EPI_ISL_416587|2020-02-1	....	....	....	....	....	....	
A	6426 hCoV-19/Japan/DP0287/2020|EPI_ISL_416589|2020-02-1	....	....	....	....	....	....	
A	6427 hCoV-19/Japan/DP0289/2020|EPI_ISL_416590|2020-02-1	....	....	....	....	....	....	
A	6428 hCoV-19/Japan/DP0290/2020|EPI_ISL_416591|2020-02-1	....	....	....	....	....	....	
A	6429 hCoV-19/Japan/DP0294/2020|EPI_ISL_416592|2020-02-1	....	....	....	....	....	....	
A	6430 hCoV-19/Japan/DP0311/2020|EPI_ISL_416593|2020-02-1	....	....	....	....	....	....	
A	6431 hCoV-19/Japan/DP0319/2020|EPI_ISL_416594|2020-02-1	....	....	....	....	....	....	
A	6432 hCoV-19/Japan/DP0328/2020|EPI_ISL_416595|2020-02-1	....	....	....	....	....	....	
A	6433 hCoV-19/Japan/DP0344/2020|EPI_ISL_416596|2020-02-1	....	....	....	....	....	....	
A	6434 hCoV-19/Japan/DP0346/2020|EPI_ISL_416597|2020-02-1	....	....	....	....	....	....	
A	6435 hCoV-19/Japan/DP0357/2020|EPI_ISL_416598|2020-02-1	....	....	....	....	....	....	
A	6436 hCoV-19/Japan/DP0361/2020|EPI_ISL_416599|2020-02-1	....	....	....	....	....	....	
A	6437 hCoV-19/Wales/PHWC-26A7F/2020|EPI_ISL_422200|2020-	....	....A...............	....	....	....	....	
A	6438 hCoV-19/Wales/PHWC-25EA1/2020|EPI_ISL_422202|2020-	....	....	....	....	....	....	
A	6439 hCoV-19/Wales/PHWC-26F16/2020|EPI_ISL_422204|2020-	....	....	....	....	....	....	
A	6440 hCoV-19/Wales/PHWC-268DF/2020|EPI_ISL_422209|2020-	....	....	....	....	....	....	
A	6441 hCoV-19/Wales/PHWC-26F61/2020|EPI_ISL_422214|2020-	....	....	....	....	....	....	
A	6442 hCoV-19/Wales/PHWC-26E0A/2020|EPI_ISL_422215|2020-	....	....M...............	....	....	....	....	
A	6443 hCoV-19/Taiwan/NTU04/2020|EPI_ISL_422407|2020-03-0	....	....	....	....	....	....	
A	6444 hCoV-19/Taiwan/NTU05/2020|EPI_ISL_422408|2020-03-1	....	....	....	....	....	....	
A	6445 hCoV-19/Taiwan/NTU06/2020|EPI_ISL_422409|2020-03-1	....	....	....	....	....	....	
A	6446 hCoV-19/Taiwan/NTU07/2020|EPI_ISL_422410|2020-03-1	....	....	....	....	....	....	
A	6447 hCoV-19/Taiwan/NTU08/2020|EPI_ISL_422411|2020-03-1	....	....	....	....	....	....	
A	6448 hCoV-19/Taiwan/NTU09/2020|EPI_ISL_422412|2020-03-1	....	....	....	....	....	....	
A	6449 hCoV-19/Taiwan/NTU11/2020|EPI_ISL_422413|2020-03-1	....	....	....	....	....	....	
A	6450 hCoV-19/Taiwan/NTU12/2020|EPI_ISL_422414|2020-03-1	....	....	....	....	....	....	
A	6451 hCoV-19/Taiwan/NTU13/2020|EPI_ISL_422415|2020-03-1	....	....	....	....	....	....	
A	6452 hCoV-19/Taiwan/NTU14/2020|EPI_ISL_422416|2020-03-1	....	....	....	....	....	....	
A	6453 hCoV-19/Taiwan/NTU15/2020|EPI_ISL_422417|2020-03-1	....	...................A	....	....	....	....	1
A	6454 hCoV-19/Taiwan/NTU16/2020|EPI_ISL_422418|2020-03-1	....	....	....	....	....	....	
A	6455 hCoV-19/Taiwan/NTU17/2020|EPI_ISL_422419|2020-03-1	....	....	....	....	....	....	
A	6456 hCoV-19/Taiwan/NTU19/2020|EPI_ISL_422420|2020-03-1	....	....	....	....	....	....	
A	6457 hCoV-19/Taiwan/NTU20/2020|EPI_ISL_422421|2020-03-1	....	....	....	....	....	....	
A	6458 hCoV-19/Taiwan/NTU21/2020|EPI_ISL_422422|2020-03-1	....	....	....	....	....	....	
A	6459 hCoV-19/Kuwait/KU006/2020|EPI_ISL_422424|2020-03-1	....	....	....	....	....	....	
A	6460 hCoV-19/Zhejiang/HZ103/2020|EPI_ISL_422425|2020-01	....	....	....	....	....	....	

A	6461 hCoV-19/Kuwait/KU005/2020|EPI_ISL_422426|2020-03-1	····················	····················	····················	····················	····················	····················
A	6462 hCoV-19/Kuwait/KU008/2020|EPI_ISL_422427|2020-03-1	····················	····················	····················	····················	····················	····················
A	6463 hCoV-19/Singapore/35/2020|EPI_ISL_422428|2020-02-0	····················	····················	····················	····················	····················	····················
A	6464 hCoV-19/Singapore/36/2020|EPI_ISL_422429|2020-02-1	····················	····················	····················	····················	····················	····················
A	6465 hCoV-19/Singapore/37/2020|EPI_ISL_422430|2020-03-1	····················	····················	····················	····················	····················	····················
A	6466 hCoV-19/Singapore/38/2020|EPI_ISL_422431|2020-03-3	····················	····················	····················	····················	····················	····················
A	6467 hCoV-19/Singapore/39/2020|EPI_ISL_422432|2020-03-3	····················	····················	····················	····················	····················	····················
A	6468 hCoV-19/Singapore/40/2020|EPI_ISL_422433|2020-03-3	····················	····················	····················	····················	····················	····················
A	6469 hCoV-19/Singapore/41/2020|EPI_ISL_422434|2020-03-3	····················	····················	····················	····················	····················	····················
A	6472 hCoV-19/England/20144024304/2020|EPI_ISL_423532|20	····················	····················	····················	····················	····················	····················
A	6473 hCoV-19/England/20144036004/2020|EPI_ISL_423535|20	····················	····················	····················	····················	····················	····················
A	6475 hCoV-19/England/20144061104/2020|EPI_ISL_423550|20	····················	····················	····················	····················	····················	····················
A	6476 hCoV-19/England/20144064004/2020|EPI_ISL_423554|20	····················	····················	····················	····················	····················	····················
A	6477 hCoV-19/England/20144066304/2020|EPI_ISL_423555|20	···T················	····················	····················	····················	····················	····················
A	6479 hCoV-19/England/20144071504/2020|EPI_ISL_423559|20	····················	····················	····················	····················	····················	····················
A	6480 hCoV-19/England/20144073804/2020|EPI_ISL_423561|20	····················	····················	····················	····················	····················	····················
A	6481 hCoV-19/England/20144074504/2020|EPI_ISL_423563|20	····················	····················	····················	····················	····················	····················
A	6483 hCoV-19/England/20144076204/2020|EPI_ISL_423570|20	····················	····················	····················	····················	····················	····················
A	6484 hCoV-19/England/20146004804/2020|EPI_ISL_423573|20	····················	····················	····················	····················	····················	····················
A	6485 hCoV-19/England/20146005804/2020|EPI_ISL_423574|20	····················	····················	····················	····················	····················	····················
A	6488 hCoV-19/England/20146025304/2020|EPI_ISL_423582|20	····················	····················	····················	····················	····················	····················
A	6489 hCoV-19/England/20146060504/2020|EPI_ISL_423583|20	····················	····················	····················	····················	····················	····················
A	6492 hCoV-19/England/20148006604/2020|EPI_ISL_423588|20	····················	····················	····················	····················	····················	····················
A	6494 hCoV-19/England/20148043704/2020|EPI_ISL_423591|20	····················	····················	····················	····················	····················	····················
A	6496 hCoV-19/England/20132001104/2020|EPI_ISL_423704|20	····················	····················	····················	····················	····················	····················
A	6497 hCoV-19/England/20132003004/2020|EPI_ISL_423711|20	····················	····················	····················	····················	····················	····················
A	6498 hCoV-19/England/20132003204/2020|EPI_ISL_423712|20	····················	····················	····················	····················	····················	····················
A	6500 hCoV-19/England/20132004804/2020|EPI_ISL_423717|20	····················	····················	····················	····················	··N········N········	····················
A	6501 hCoV-19/England/20132004904/2020|EPI_ISL_423718|20	····················	····················	····················	····················	····················	····················
A	6502 hCoV-19/England/20132005204/2020|EPI_ISL_423720|20	····················	····················	····················	····················	····················	····················
A	6503 hCoV-19/England/20132005704/2020|EPI_ISL_423723|20	····················	····················	····················	····················	····················	····················
A	6504 hCoV-19/England/20132017604/2020|EPI_ISL_423727|20	····················	····················	····················	····················	····················	····················
A	6505 hCoV-19/England/20132018004/2020|EPI_ISL_423731|20	····················	····················	····················	····················	····················	····················
A	6507 hCoV-19/England/20132018704/2020|EPI_ISL_423733|20	····················	····················	····················	····················	····················	····················
A	6508 hCoV-19/England/20132018804/2020|EPI_ISL_423734|20	····················	····················	····················	····················	····················	····················
A	6509 hCoV-19/England/20132018904/2020|EPI_ISL_423735|20	····················	····················	····················	····················	····················	····················
A	6511 hCoV-19/England/20132021004/2020|EPI_ISL_423741|20	····················	····················	····················	····················	····················	····················
A	6512 hCoV-19/England/20132023204/2020|EPI_ISL_423749|20	····················	····················	····················	····················	····················	····················
A	6513 hCoV-19/England/20132023704/2020|EPI_ISL_423752|20	····················	····················	····················	····················	····················	····················
A	6514 hCoV-19/England/20132023904/2020|EPI_ISL_423753|20	····················	····················	····················	····················	····················	····················
A	6515 hCoV-19/England/20132024604/2020|EPI_ISL_423758|20	····················	····················	····················	····················	····················	····················
A	6516 hCoV-19/England/20132026904/2020|EPI_ISL_423761|20	····················	····················	····················	····················	····················	····················
A	6517 hCoV-19/England/20132027004/2020|EPI_ISL_423762|20	····················	····················	····················	····················	····················	····················
A	6518 hCoV-19/England/20132027504/2020|EPI_ISL_423765|20	····················	····················	····················	····················	····················	····················
A	6519 hCoV-19/USA/FL_7169/2020|EPI_ISL_424855|2020-03-02	····················	····················	····················	····················	····················	····················
A	6520 hCoV-19/USA/FL_9656/2020|EPI_ISL_424856|2020-03-06	····················	····················	····················	····················	····················	····················
A	6521 hCoV-19/USA/FL_1177/2020|EPI_ISL_424857|2020-03-06	····················	····················	····················	····················	····················	····················
A	6522 hCoV-19/USA/GA_1621/2020|EPI_ISL_424858|2020-03-04	····················	····················	····················	····················	····················	····················
A	6523 hCoV-19/USA/GA_1847/2020|EPI_ISL_424859|2020-03-05	····················	····················	····················	····················	····················	····················
A	6524 hCoV-19/USA/GA_2055/2020|EPI_ISL_424860|2020-03	····················	····················	····················	····················	····················	····················
A	6525 hCoV-19/USA/GA_2118/2020|EPI_ISL_424861|2020-03-08	····················	····················	····················	····················	····················	····················
A	6526 hCoV-19/USA/GA_2120/2020|EPI_ISL_424862|2020-03	····················	····················	····················	····················	····················	····················
A	6527 hCoV-19/USA/GA_2185/2020|EPI_ISL_424863|2020-03	····················	····················	····················	····················	····················	····················
A	6528 hCoV-19/USA/GA_2343/2020|EPI_ISL_424864|2020-03-10	····················	····················	····················	····················	····················	····················
A	6529 hCoV-19/USA/IA_6394/2020|EPI_ISL_424865|2020-03-08	····················	····················	····················	····················	····················	····················
A	6530 hCoV-19/USA/IN_2001/2020|EPI_ISL_424866|2020-03-09	····················	····················	····················	····················	····················	····················
A	6531 hCoV-19/USA/KS_4126/2020|EPI_ISL_424867|2020-03-06	····················	····················	····················	····················	····················	····················
A	6532 hCoV-19/USA/LA_0842/2020|EPI_ISL_424868|2020-03-09	····················	····················	····················	····················	····················	····················
A	6533 hCoV-19/USA/MD_0026/2020|EPI_ISL_424869|2020-03-04	····················	····················	····················	····················	····················	····················
A	6534 hCoV-19/USA/MO_4470/2020|EPI_ISL_424870|2020-03-06	····················	····················	····················	····················	····················	····················
A	6535 hCoV-19/USA/NC_6999/2020|EPI_ISL_424871|2020-03-06	····················	····················	····················	····················	····················	····················
A	6536 hCoV-19/USA/NC_0024/2020|EPI_ISL_424872|2020-03-08	····················	····················	····················	····················	····················	····················
A	6537 hCoV-19/USA/NC_0031/2020|EPI_ISL_424873|2020-03-07	····················	····················	····················	····················	····················	····················
A	6538 hCoV-19/USA/NE_6605/2020|EPI_ISL_424874|2020-03-05	····················	····················	····················	····················	····················	····················
A	6539 hCoV-19/USA/NE_7025/2020|EPI_ISL_424875|2020-03-07	····················	····················	····················	····················	····················	····················
A	6540 hCoV-19/USA/NH_0029/2020|EPI_ISL_424876|2020-03-06	····················	····················	····················	····················	····················	····················
A	6541 hCoV-19/USA/NH_0033/2020|EPI_ISL_424877|2020-03-06	····················	····················	····················	····················	····················	····················
A	6542 hCoV-19/USA/NJ_3592/2020|EPI_ISL_424878|2020-03-04	····················	····················	····················	····················	····················	····················
A	6543 hCoV-19/USA/NV_0052/2020|EPI_ISL_424879|2020-03-09	····················	····················	····················	····················	····················	····················
A	6544 hCoV-19/USA/OH_0023/2020|EPI_ISL_424880|2020-03-08	····················	····················	····················	····················	····················	····················
A	6545 hCoV-19/USA/PA_1802/2020|EPI_ISL_424881|2020-03-05	····················	····················	····················	····················	····················	····················
A	6546 hCoV-19/USA/PA_1881/2020|EPI_ISL_424882|2020-03-05	····················	····················	····················	····················	····················	····················
A	6547 hCoV-19/USA/PA_2317/2020|EPI_ISL_424883|2020-03-07	····················	····················	····················	····················	····················	····················
A	6548 hCoV-19/USA/PA_2937/2020|EPI_ISL_424884|2020-03-08	····················	····················	····················	····················	····················	····················
A	6549 hCoV-19/USA/PA_4395/2020|EPI_ISL_424885|2020-03-09	····················	····················	····················	····················	····················	····················
A	6550 hCoV-19/USA/PA_4405/2020|EPI_ISL_424886|2020-03-06	····················	····················	····················	····················	····················	····················
A	6551 hCoV-19/USA/RI_0702/2020|EPI_ISL_424887|2020-03-05	····················	····················	····················	····················	····················	····················
A	6552 hCoV-19/USA/SC_6370/2020|EPI_ISL_424888|2020-03-08	····················	····················	····················	····················	····················	····················

A	6553	hCoV-19/USA/UT_8199/2020|EPI_ISL_424889|2020-03-05	....	....	....	....	....	....
A	6554	hCoV-19/USA/UT_8906/2020|EPI_ISL_424890|2020-03-07	....	....	....	....	....	....
A	6555	hCoV-19/USA/VA_6352/2020|EPI_ISL_424891|2020-03-08	....	....	....	....	....	....
A	6556	hCoV-19/USA/VA_6377/2020|EPI_ISL_424892|2020-03-08	....	....	....	....	....	....
A	6557	hCoV-19/USA/VA_6382/2020|EPI_ISL_424893|2020-03-08	....	....	....	....	....	....
A	6558	hCoV-19/USA/VA_6389/2020|EPI_ISL_424894|2020-03-08	....	....	....	....	....	....
A	6559	hCoV-19/USA/IA_6396/2020|EPI_ISL_424895|2020-03-08	....	....	....	....	....	....
A	6560	hCoV-19/USA/IA_6395/2020|EPI_ISL_424896|2020-03-08	....	....	....	....	....	....
A	6561	hCoV-19/USA/IA_6401/2020|EPI_ISL_424897|2020-03-08	....	....	....	....	....	....
A	6562	hCoV-19/USA/IA_6399/2020|EPI_ISL_424898|2020-03-08	....	....	....	....	....	....
A	6563	hCoV-19/USA/IA_6391/2020|EPI_ISL_424899|2020-03-07	....	....	....	....	....	....
A	6564	hCoV-19/USA/WA-UW-3918/2020|EPI_ISL_427209|2020-03	....	....	....	....	....	....
A	6565	hCoV-19/USA/WA-UW-3923/2020|EPI_ISL_427210|2020-03	....	....	....	....	....	....
A	6566	hCoV-19/USA/WA-UW-3932/2020|EPI_ISL_427211|2020-03	....	....	....	....	....	....
A	6567	hCoV-19/USA/WA-UW-3935/2020|EPI_ISL_427212|2020-03	....	....	....	....	....	....
A	6568	hCoV-19/USA/WA-UW-3939/2020|EPI_ISL_427213|2020-03	....	....	....	....	....	....
A	6569	hCoV-19/USA/WA-UW-3940/2020|EPI_ISL_427214|2020-03	....	....	....	....	....	....
A	6570	hCoV-19/USA/WA-UW-3941/2020|EPI_ISL_427215|2020-03	....	....	....	....	....	....
A	6571	hCoV-19/USA/WA-UW-3942/2020|EPI_ISL_427216|2020-03	....	....	....	....	....	....
A	6572	hCoV-19/USA/WA-UW-3946/2020|EPI_ISL_427217|2020-03	....	....	....	....	....	....
A	6573	hCoV-19/USA/WA-UW-3950/2020|EPI_ISL_427218|2020-03	....	....	....	....	....	....
A	6574	hCoV-19/USA/WA-UW-3954/2020|EPI_ISL_427219|2020-03	....	....	....	....	....	....
A	6575	hCoV-19/USA/WA-UW-3959/2020|EPI_ISL_427220|2020-03	....	....	....	....	....	....
A	6576	hCoV-19/USA/WA-UW-3964/2020|EPI_ISL_427222|2020-03	....	....	....	....	....	....
A	6577	hCoV-19/USA/WA-UW-3969/2020|EPI_ISL_427223|2020-03	....	....	....	....	....	....
A	6578	hCoV-19/USA/WA-UW-3983/2020|EPI_ISL_427224|2020-03	....	....	....	....	....	....
A	6579	hCoV-19/USA/WA-UW-3984/2020|EPI_ISL_427225|2020-03	....	....	....	....	....	....
A	6580	hCoV-19/USA/WA-UW-3985/2020|EPI_ISL_427226|2020-03	....	....	....	....	....	....
A	6581	hCoV-19/USA/WA-UW-3986/2020|EPI_ISL_427227|2020-03	....	....	....	....	....	....
A	6582	hCoV-19/USA/WA-UW-3988/2020|EPI_ISL_427228|2020-03	....	....	....	....	....	....
A	6583	hCoV-19/USA/WA-UW-3989/2020|EPI_ISL_427229|2020-03	....	....	....	....	....	....
A	6584	hCoV-19/USA/WA-UW-3990/2020|EPI_ISL_427230|2020-03	....	....	....	....	....	....
A	6585	hCoV-19/USA/WA-UW-3994/2020|EPI_ISL_427232|2020-03	....	....	....	....	....	....
A	6586	hCoV-19/USA/WA-UW-3996/2020|EPI_ISL_427233|2020-03	....	....	....	....	....	....
A	6587	hCoV-19/USA/WA-UW-3997/2020|EPI_ISL_427234|2020-03	....	....	....	....	....	....
A	6588	hCoV-19/USA/WA-UW-4035/2020|EPI_ISL_427235|2020-03	....	....	....	....	....	....
A	6589	hCoV-19/USA/WA-UW-4038/2020|EPI_ISL_427236|2020-03	....	....	....	....	....	....
A	6590	hCoV-19/USA/WA-UW-4039/2020|EPI_ISL_427237|2020-03	....	....	....	....	....	....
A	6591	hCoV-19/USA/WA-UW-4068/2020|EPI_ISL_427238|2020-04	....	....	....	....	....	....
A	6592	hCoV-19/USA/WA-UW-4082/2020|EPI_ISL_427239|2020-04	....	....	....	....	....	....
A	6593	hCoV-19/USA/WA-UW-4105/2020|EPI_ISL_427240|2020-03	....	....	....	....	....	....
A	6594	hCoV-19/USA/WA-UW-4109/2020|EPI_ISL_427241|2020-03	....	....	....	....	....	....
A	6595	hCoV-19/USA/WA-UW-4111/2020|EPI_ISL_427242|2020-03	....	....	....	....	....	....
A	6596	hCoV-19/USA/WA-UW-4180/2020|EPI_ISL_427245|2020-04	....	....	....	....	....	....
A	6597	hCoV-19/USA/WA-UW-4188/2020|EPI_ISL_427246|2020-03	....	....	....	....	....	....
A	6598	hCoV-19/USA/WA-UW-4197/2020|EPI_ISL_427247|2020-03	....	....	....	....	....	....
A	6599	hCoV-19/USA/WA-UW-4213/2020|EPI_ISL_427248|2020-03	....	....	....	....	....	....
A	6600	hCoV-19/USA/WA-UW-4217/2020|EPI_ISL_427249|2020-03	....	....	....	....	....	....
A	6601	hCoV-19/USA/WA-UW-4227/2020|EPI_ISL_427250|2020-03	....	....	....	....	....	....
A	6602	hCoV-19/USA/WA-UW-4228/2020|EPI_ISL_427251|2020-03	....	....	....	....	....	....
A	6603	hCoV-19/USA/WA-UW-4281/2020|EPI_ISL_427252|2020-03	....	....	....	....	....	....
A	6604	hCoV-19/USA/WA-UW-4289/2020|EPI_ISL_427253|2020-03	....	....	....	....	....	....
A	6605	hCoV-19/USA/WA-UW-4290/2020|EPI_ISL_427254|2020-03	....	....	....	....	....	....
A	6606	hCoV-19/USA/WA-UW-4500/2020|EPI_ISL_427270|2020-03	....	....	....	....	....	....
A	6607	hCoV-19/USA/AZ-TG271866/2020|EPI_ISL_427271|2020-0	....	....	....	....	....	....
A	6608	hCoV-19/USA/AZ-TG271868/2020|EPI_ISL_427272|2020-0	....	....	....	....	....	....
A	6609	hCoV-19/USA/MN-MDH-117/2020|EPI_ISL_427273|2020-03	....	....	....	....	....	....
A	6610	hCoV-19/USA/MN-MDH-118/2020|EPI_ISL_427274|2020-03	....	....	....	....	....	....
A	6611	hCoV-19/USA/MN-MDH-119/2020|EPI_ISL_427275|2020-03	....	....	....	....	....	....
A	6612	hCoV-19/USA/MN-MDH-120/2020|EPI_ISL_427276|2020-03	....	....	....	....	....	....
A	6613	hCoV-19/USA/MN-MDH-121/2020|EPI_ISL_427277|2020-03	....	....	....	....	....	....
A	6614	hCoV-19/USA/MN-MDH-138/2020|EPI_ISL_427278|2020-03	....	....	....	....	....	....
A	6615	hCoV-19/USA/MN-MDH-140/2020|EPI_ISL_427279|2020-03	....	....	....	....	....	....
A	6616	hCoV-19/USA/MN-MDH-141/2020|EPI_ISL_427280|2020-03	....	....	....	....	....	....
A	6617	hCoV-19/USA/MN-MDH-142/2020|EPI_ISL_427281|2020-03	....	....	....	....	....	....
A	6618	hCoV-19/USA/MN-MDH-143/2020|EPI_ISL_427282|2020-03	....	....	....	....	....	....
A	6619	hCoV-19/USA/MN-MDH-144/2020|EPI_ISL_427283|2020-03	....	....	....	....	....	....
A	6620	hCoV-19/USA/MN-MDH-145/2020|EPI_ISL_427284|2020-03	....	....	....	....	....	....
A	6621	hCoV-19/USA/MN-MDH-146/2020|EPI_ISL_427285|2020-03	....	....	....	....	....	....
A	6622	hCoV-19/USA/MN-MDH-147/2020|EPI_ISL_427286|2020-03	....	....	....	....	....	....
A	6623	hCoV-19/USA/MN-MDH-149/2020|EPI_ISL_427287|2020-03	....	....	....	....	....	....
A	6624	hCoV-19/USA/OH-OSUP0016/2020|EPI_ISL_427288|2020-0	....	....	....	....	....	....
A	6625	hCoV-19/USA/OH-OSUP0017/2020|EPI_ISL_427290|2020-0	....	....	....	....	....	....
A	6626	hCoV-19/Brazil/AL-837/2020|EPI_ISL_427292|2020-03-	....	....	....	....	....	....
A	6627	hCoV-19/Brazil/BA-510/2020|EPI_ISL_427293|2020-03-	....	....	....	....	....	....
A	6628	hCoV-19/Brazil/DF-615i/2020|EPI_ISL_427294|2020-03	....	....	....	....	....	....
A	6629	hCoV-19/Brazil/DF-619i/2020|EPI_ISL_427295|2020-03	....	....	....	....	....	....
A	6630	hCoV-19/Brazil/DF-861/2020|EPI_ISL_427296|2020-03-	....	....	....	....	....	....

A	6631	hCoV-19/Brazil/DF-862/2020|EPI_ISL_427297|2020-03-	····················	····················	····················	····················	····················	····················	
A	6632	hCoV-19/Brazil/DF-891/2020|EPI_ISL_427298|2020-03-	····················	····················	····················	····················	····················	····················	
A	6633	hCoV-19/Brazil/RJ-352/2020|EPI_ISL_427299|2020-03-	····················	····················	····················	····················	····················	····················	
A	6634	hCoV-19/Luxembourg/LNS6137379/2020|EPI_ISL_429722|	····················	····················	····················	····················	····················	····················	
A	6635	hCoV-19/Luxembourg/LNS9984061/2020|EPI_ISL_429724|	····················	····················	····················	····················	····················	····················	
A	6636	hCoV-19/Luxembourg/LNS0756265/2020|EPI_ISL_429725|	····················	·A··················	····················	····················	····················	····················	
A	6637	hCoV-19/Luxembourg/LNS9102295/2020|EPI_ISL_429727|	····················	····················	····················	····················	····················	····················	
A	6638	hCoV-19/Turkey/HSGM-8990/2020|EPI_ISL_429870|2020-	····················	····················	····················	····················	····················	····················	
A	6639	hCoV-19/Turkey/HSGM-10241/2020|EPI_ISL_429871|2020	····················	····················	····················	····················	····················	····················	
A	6640	hCoV-19/Turkey/HSGM-11518/2020|EPI_ISL_429872|2020	····················	····················	····················	····················	····················	····················	
A	6641	hCoV-19/Turkey/HSGM-11520/2020|EPI_ISL_429873|2020	····················	····················	····················	····················	····················	····················	
A	6642	hCoV-19/USA/CA-SCCPHD-UC34/2020|EPI_ISL_429879|202	····················	····················	····················	····················	····················	NNN·················	1
A	6643	hCoV-19/Taiwan/299/2020|EPI_ISL_429882|2020-03-29	····················	····················	····················	····················	····················	····················	
A	6644	hCoV-19/Taiwan/108/2020|EPI_ISL_429883|2020-03-23	····················	····················	····················	····················	····················	····················	
A	6645	hCoV-19/Taiwan/284/2020|EPI_ISL_429884|2020-03-30	····················	····················	····················	····················	····················	····················	
A	6646	hCoV-19/USA/WA-UW398/2020|EPI_ISL_529200|2020-03-2	····················	····················	····················	····················	····················	····················	
A	6647	hCoV-19/USA/WA-UW391/2020|EPI_ISL_529208|2020-03-3	····················	····················	····················	····················	····················	····················	
A	6648	hCoV-19/USA/WA-UW392/2020|EPI_ISL_529209|2020-03-2	····················	····················	····················	····················	····················	····················	
A	6649	hCoV-19/USA/WA-UW408/2020|EPI_ISL_529210|2020-03-3	····················	····················	····················	····················	····················	····················	
A	6650	hCoV-19/USA/WA-UW410/2020|EPI_ISL_529211|2020-03-3	····················	····················	····················	····················	····················	····················	
A	6651	hCoV-19/USA/WA-UW413/2020|EPI_ISL_529212|2020-03-2	····················	····················	····················	····················	····················	····················	
A	6652	hCoV-19/Wuhan/IME-WH01/2020|EPI_ISL_529213|2019-12	····················	····················	····················	····················	····················	····················	
A	6653	hCoV-19/Wuhan/IME-WH02/2020|EPI_ISL_529214|2019-12	····················	····················	····················	····················	····················	····················	
A	6654	hCoV-19/Wuhan/IME-WH03/2020|EPI_ISL_529215|2019-12	····················	····················	····················	····················	····················	····················	
A	6655	hCoV-19/Wuhan/IME-WH04/2020|EPI_ISL_529216|2019-12	····················	····················	····················	····················	····················	····················	
A	6656	hCoV-19/Wuhan/IME-WH05/2020|EPI_ISL_529217|2019-12	····················	····················	····················	····················	····················	····················	
A	6657	hCoV-19/Shenzhen/HKU-SZ-005/2020|EPI_ISL_405839|20	····················	····················	····················	····················	····················	····················	
A	6658	hCoV-19/Shenzhen/HKU-SZ-002/2020|EPI_ISL_406030|20	····················	····················	····················	····················	····················	····················	
A	6659	hCoV-19/USA/CA-CDC-1/2020|EPI_ISL_406034|2020-01-2	····················	····················	····················	····················	····················	····················	
A	6660	hCoV-19/USA/CA2/2020|EPI_ISL_406036|2020-01-22	····················	····················	····················	····················	····················	····················	
A	6661	hCoV-19/Japan/TY-WK-012/2020|EPI_ISL_408665|2020-0	····················	····················	····················	····················	····················	····················	
A	6662	hCoV-19/Japan/TY-WK-501/2020|EPI_ISL_408666|2020-0	····················	····················	····················	····················	····················	····················	
A	6663	hCoV-19/Japan/TY-WK-521/2020|EPI_ISL_408667|2020-0	····················	····················	····················	····················	····················	····················	
A	6664	hCoV-19/Vietnam/VR03-38142/2020|EPI_ISL_408668|202	····················	····················	····················	····················	····················	····················	
A	6665	hCoV-19/Japan/KY-V-029/2020|EPI_ISL_408669|2020-01	····················	····················	····················	····················	····················	····················	
A	6666	hCoV-19/USA/WI1/2020|EPI_ISL_408670|2020-01-31	····················	····················	····················	····················	····················	····················	
A	6667	hCoV-19/France/IDF-0515-isl/2020|EPI_ISL_410984|20	····················	····················	····················	····················	····················	····················	
A	6668	hCoV-19/France/IDF-2279/2020|EPI_ISL_416500|2020-0	····················	····················	····················	····················	····················	····················	
A	6669	hCoV-19/France/IDF-2284/2020|EPI_ISL_416501|2020-0	····················	····················	····················	····················	····················	····················	
A	6670	hCoV-19/France/BRE-2330/2020|EPI_ISL_416502|2020-0	····················	····················	····················	····················	····················	····················	
A	6671	hCoV-19/France/BRE-2334/2020|EPI_ISL_416503|2020-0	····················	····················	····················	····················	····················	····················	
A	6672	hCoV-19/France/BRE-2335/2020|EPI_ISL_416504|2020-0	····················	····················	····················	····················	····················	····················	
A	6673	hCoV-19/France/BRE-2336/2020|EPI_ISL_416505|2020-0	····················	····················	····················	····················	····················	····················	
A	6674	hCoV-19/France/BRE-2337/2020|EPI_ISL_416506|2020-0	····················	····················	····················	····················	····················	····················	
A	6675	hCoV-19/France/BRE-2340/2020|EPI_ISL_416507|2020-0	····················	····················	····················	····················	····················	····················	
A	6676	hCoV-19/France/BRE-2343/2020|EPI_ISL_416508|2020-0	····················	····················	····················	····················	····················	····················	
A	6677	hCoV-19/France/BRE-2344/2020|EPI_ISL_416509|2020-0	····················	····················	····················	····················	····················	····················	
A	6678	hCoV-19/France/BRE-2346/2020|EPI_ISL_416510|2020-0	····················	····················	····················	····················	····················	····················	
A	6679	hCoV-19/France/BRE-2348/2020|EPI_ISL_416511|2020-0	····················	····················	····················	····················	····················	····················	
A	6680	hCoV-19/France/BRE-2349/2020|EPI_ISL_416512|2020-0	····················	····················	····················	····················	····················	····················	
A	6681	hCoV-19/France/BRE-2351/2020|EPI_ISL_416513|2020-0	····················	····················	····················	····················	····················	····················	
A	6682	hCoV-19/Australia/VIC08/2020|EPI_ISL_416514|2020-0	····················	····················	····················	····················	····················	····················	
A	6683	hCoV-19/New Zealand/20VR0189/2020|EPI_ISL_416519|2	····················	····················	····················	····················	····················	····················	
A	6684	hCoV-19/USA/WI-UW-03/2020|EPI_ISL_416523|2020-03-1	····················	····················	····················	····················	····················	····················	
A	6685	hCoV-19/Japan/SMU-0311S2/2020|EPI_ISL_416524|2020-	····················	····················	····················	····················	····················	····················	
A	6686	hCoV-19/Japan/SMU-0311S3/2020|EPI_ISL_416525|2020-	····················	····················	····················	····················	····················	····················	
A	6687	hCoV-19/New Zealand/20VR0275/2020|EPI_ISL_416538|2	····················	····················	····················	····················	····················	····················	
A	6688	hCoV-19/New Zealand/20VR0276/2020|EPI_ISL_416539|2	····················	····················	····················	····················	····················	····················	
A	6689	hCoV-19/Kuwait/KU09/2020|EPI_ISL_416541|2020-03-02	····················	····················	····················	····················	····················	····················	
A	6690	hCoV-19/Kuwait/KU17/2020|EPI_ISL_416542|2020-03-02	····················	····················	····················	····················	····················	····················	
A	6691	hCoV-19/Kuwait/KU18/2020|EPI_ISL_416543|2020-03-02	····················	····················	····················	····················	····················	····················	
A	6692	hCoV-19/Iceland/15/2020|EPI_ISL_417800|2020-03-02	····················	····················	····················	····················	····················	····················	
A	6693	hCoV-19/Iceland/151/2020|EPI_ISL_417801|2020-03-13	····················	····················	····················	····················	····················	····················	
A	6694	hCoV-19/Iceland/153/2020|EPI_ISL_417802|2020-03-13	····················	····················	····················	····················	····················	····················	
A	6695	hCoV-19/Iceland/156/2020|EPI_ISL_417803|2020-03-13	····················	····················	····················	····················	····················	····················	
A	6696	hCoV-19/Iceland/157/2020|EPI_ISL_417804|2020-03-13	····················	····················	····················	····················	····················	····················	
A	6697	hCoV-19/Iceland/158/2020|EPI_ISL_417805|2020-03-13	····················	····················	····················	····················	····················	····················	
A	6698	hCoV-19/Iceland/159/2020|EPI_ISL_417806|2020-03-13	····················	····················	····················	····················	····················	····················	
A	6699	hCoV-19/Iceland/165/2020|EPI_ISL_417808|2020-03-15	····················	····················	····················	····················	····················	····················	
A	6700	hCoV-19/Iceland/167/2020|EPI_ISL_417809|2020-03-15	····················	····················	····················	····················	····················	····················	
A	6701	hCoV-19/Iceland/168/2020|EPI_ISL_417810|2020-03-13	····················	····················	····················	····················	····················	····················	
A	6702	hCoV-19/Iceland/176/2020|EPI_ISL_417812|2020-03-14	····················	····················	····················	····················	····················	····················	
A	6703	hCoV-19/Iceland/178/2020|EPI_ISL_417813|2020-03-15	····················	····················	····················	····················	····················	····················	
A	6704	hCoV-19/Iceland/181/2020|EPI_ISL_417814|2020-03-16	····················	····················	····················	····················	····················	····················	
A	6705	hCoV-19/Iceland/186/2020|EPI_ISL_417815|2020-03-16	····················	····················	····················	····················	····················	····················	
A	6706	hCoV-19/Iceland/188/2020|EPI_ISL_417816|2020-03-16	····················	····················	····················	····················	····················	····················	
A	6707	hCoV-19/Iceland/19/2020|EPI_ISL_417817|2020-03-02	····················	····················	····················	····················	····················	····················	
A	6708	hCoV-19/Iceland/190/2020|EPI_ISL_417818|2020-03-16	····················	····················	····················	····················	····················	····················	

A	6709 hCoV-19/Iceland/192/2020|EPI_ISL_417819|2020-03-15	····················	····················	····················	····················	····················	····················		
A	6710 hCoV-19/Iceland/193/2020|EPI_ISL_417820|2020-03-16	····················	····················	····················	····················	····················	····················		
A	6711 hCoV-19/Iceland/196/2020|EPI_ISL_417822|2020-03-16	····················	····················	····················	····················	····················	····················		
A	6712 hCoV-19/Iceland/198/2020|EPI_ISL_417823|2020-03-16	····················	····················	····················	····················	····················	····················		
A	6713 hCoV-19/Iceland/201/2020|EPI_ISL_417824|2020-03-16	····················	····················	····················	····················	····················	····················		
A	6714 hCoV-19/Iceland/203/2020|EPI_ISL_417825|2020-03-16	····················	····················	····················	····················	····················	····················		
A	6715 hCoV-19/Iceland/204/2020|EPI_ISL_417826|2020-03-16	····················	····················	····················	····················	····················	····················		
A	6716 hCoV-19/Iceland/205/2020|EPI_ISL_417827|2020-03-16	····················	····················	····················	····················	····················	····················		
A	6718 hCoV-19/Iceland/210/2020|EPI_ISL_417829|2020-03-16	····················	····················	····················	····················	····················	····················		
A	6719 hCoV-19/Iceland/212/2020|EPI_ISL_417830|2020-03-16	····················	····················	····················	····················	····················	····················		
A	6720 hCoV-19/Iceland/214/2020|EPI_ISL_417831|2020-03-16	····················	····················	····················	····················	····················	····················		
A	6721 hCoV-19/Iceland/216/2020|EPI_ISL_417832|2020-03-16	····················	····················	····················	····················	····················	····················		
A	6722 hCoV-19/Iceland/217/2020|EPI_ISL_417833|2020-03-16	····················	····················	····················	····················	····················	····················		
A	6723 hCoV-19/Iceland/218/2020|EPI_ISL_417834|2020-03-16	····················	····················	····················	····················	····················	····················		
A	6724 hCoV-19/Iceland/219/2020|EPI_ISL_417835|2020-03-16	····················	····················	····················	····················	····················	····················		
A	6725 hCoV-19/Iceland/221/2020|EPI_ISL_417836|2020-03-16	····················	····················	····················	····················	····················	····················		
A	6726 hCoV-19/Iceland/222/2020|EPI_ISL_417837|2020-03-16	····················	····················	····················	····················	····················	····················		
A	6727 hCoV-19/Iceland/223/2020|EPI_ISL_417838|2020-03-16	····················	····················	····················	····················	····················	····················		
A	6728 hCoV-19/Iceland/224/2020|EPI_ISL_417839|2020-03-16	····················	····················	····················	····················	····················	····················		
A	6729 hCoV-19/Iceland/23/2020|EPI_ISL_417840|2020-03-02	····················	····················	····················	····················	····················	····················		
A	6730 hCoV-19/Iceland/26/2020|EPI_ISL_417841|2020-03-03	····················	····················	····················	····················	····················	····················		
A	6731 hCoV-19/Iceland/31/2020|EPI_ISL_417842|2020-03-03	····················	····················	····················	····················	····················	····················		
A	6733 hCoV-19/Iceland/36/2020|EPI_ISL_417844|2020-03-04	····················	····················	····················	····················	····················	····················		
A	6734 hCoV-19/Iceland/4/2020|EPI_ISL_417845|2020-03-13	····················	····················	····················	····················	····················	····················		
A	6735 hCoV-19/Iceland/40/2020|EPI_ISL_417846|2020-03-04	····················	····················	····················	····················	····················	····················		
A	6737 hCoV-19/Iceland/52/2020|EPI_ISL_417850|2020-03-05	····················	····················	····················	····················	····················	····NN··N···········		1
A	6738 hCoV-19/Iceland/53/2020|EPI_ISL_417851|2020-03-05	····················	····················	····················	····················	····················	····················		
A	6739 hCoV-19/Iceland/56/2020|EPI_ISL_417852|2020-03-06	····················	····················	····················	····················	····················	····················		
A	6740 hCoV-19/Iceland/59/2020|EPI_ISL_417853|2020-03-06	····················	····················	····················	····················	····················	····················		
A	6741 hCoV-19/Iceland/6/2020|EPI_ISL_417854|2020-03-13	····················	····················	····················	····················	····················	····················		
A	6742 hCoV-19/Iceland/63/2020|EPI_ISL_417855|2020-03-07	····················	····················	····················	····················	····················	····················		
A	6743 hCoV-19/Iceland/64/2020|EPI_ISL_417856|2020-03-07	····················	····················	····················	····················	····················	·················NNN	1	1
A	6744 hCoV-19/Iceland/66/2020|EPI_ISL_417857|2020-03-07	····················	····················	····················	····················	····················	····················		
A	6745 hCoV-19/Iceland/72/2020|EPI_ISL_417860|2020-03-08	····················	····················	····················	····················	····················	····················		
A	6746 hCoV-19/Iceland/73/2020|EPI_ISL_417861|2020-03-09	····················	····················	····················	····················	····················	····················		
A	6747 hCoV-19/Iceland/77/2020|EPI_ISL_417862|2020-03-09	····················	····················	····················	····················	····················	····················		
A	6748 hCoV-19/Iceland/78/2020|EPI_ISL_417863|2020-03-09	····················	····················	····················	····················	····················	····················		
A	6749 hCoV-19/Iceland/8/2020|EPI_ISL_417864|2020-03-15	····················	····················	····················	····················	····················	····················		
A	6750 hCoV-19/Iceland/80/2020|EPI_ISL_417865|2020-03-09	····················	····················	····················	····················	····················	····················		
A	6751 hCoV-19/Iceland/81/2020|EPI_ISL_417866|2020-03-09	····················	····················	····················	····················	····················	····················		
A	6752 hCoV-19/Iceland/82/2020|EPI_ISL_417867|2020-03-09	····················	····················	····················	····················	····················	····················		
A	6753 hCoV-19/Iceland/83/2020|EPI_ISL_417868|2020-03-09	····················	····················	····················	····················	····················	····················		
A	6754 hCoV-19/Iceland/9/2020|EPI_ISL_417871|2020-03-11	····················	····················	····················	····················	····················	····················		
A	6755 hCoV-19/Iceland/90/2020|EPI_ISL_417872|2020-03-10	····················	····················	····················	····················	····················	····················		
A	6756 hCoV-19/Iceland/91/2020|EPI_ISL_417873|2020-03-10	····················	····················	····················	····················	····················	····················		
A	6759 hCoV-19/Iceland/97/2020|EPI_ISL_417876|2020-03-10	····················	····················	····················	····················	····················	····················		
A	6760 hCoV-19/Slovakia/SK-BMC1/2020|EPI_ISL_417877|2020-	····················	····················	····················	····················	····················	····················		
A	6761 hCoV-19/Slovakia/SK-BMC2/2020|EPI_ISL_417878|2020-	····················	····················	····················	····················	····················	····················		
A	6762 hCoV-19/Slovakia/SK-BMC5/2020|EPI_ISL_417879|2020-	····················	····················	····················	····················	····················	····················		
A	6763 hCoV-19/Slovakia/SK-BMC6/2020|EPI_ISL_417880|2020-	····················	····················	····················	····················	····················	····················		
A	6764 hCoV-19/Portugal/PT0025/2020|EPI_ISL_418010|2020-0	····················	····················	····················	····················	····················	····················		
A	6765 hCoV-19/Portugal/PT0026/2020|EPI_ISL_418011|2020-0	····················	····················	····················	····················	····················	····················		
A	6766 hCoV-19/Portugal/PT0027/2020|EPI_ISL_418012|2020-0	····················	····················	····················	····················	····················	····················		
A	6767 hCoV-19/Portugal/PT0028/2020|EPI_ISL_418013|2020-0	····················	····················	····················	····················	····················	····················		
A	6768 hCoV-19/Portugal/PT0029/2020|EPI_ISL_418014|2020-0	····················	····················	····················	····················	····················	····················		
A	6769 hCoV-19/Portugal/PT0030/2020|EPI_ISL_418015|2020-0	····················	····················	····················	····················	····················	····················		
A	6770 hCoV-19/Portugal/PT0031/2020|EPI_ISL_418016|2020-0	····················	····················	····················	····················	····················	····················		
A	6771 hCoV-19/Portugal/PT0032/2020|EPI_ISL_418017|2020-0	····················	····················	····················	····················	····················	····················		
A	6772 hCoV-19/Portugal/PT0033/2020|EPI_ISL_418018|2020-0	····················	····················	····················	····················	····················	····················		
A	6773 hCoV-19/Portugal/PT0034/2020|EPI_ISL_418019|2020-0	····················	····················	····················	····················	····················	····················		
A	6774 hCoV-19/Japan/P5-2/2020|EPI_ISL_419310|2020-03-13	····················	····················	····················	····················	····················	····················		
A	6775 hCoV-19/Japan/P5-3/2020|EPI_ISL_419311|2020-03-13	····················	····················	····················	····················	····················	····················		
A	6776 hCoV-19/USA/NY-NYUMC49/2020|EPI_ISL_420300|2020-03	····················	····················	····················	····················	····················	····················		
A	6777 hCoV-19/USA/NY-NYUMC50/2020|EPI_ISL_420301|2020-03	····················	····················	····················	····················	····················	····················		
A	6778 hCoV-19/USA/NY-NYUMC51/2020|EPI_ISL_420302|2020-03	····················	····················	····················	····················	····················	····················		
A	6779 hCoV-19/USA/AK-PHL002/2020|EPI_ISL_420303|2020-03-	····················	····················	····················	····················	····················	····················		
A	6780 hCoV-19/USA/AK-PHL003/2020|EPI_ISL_420304|2020-03-	····················	····················	····················	····················	····················	····················		
A	6781 hCoV-19/USA/AK-PHL006/2020|EPI_ISL_420305|2020-03-	····················	····················	····················	····················	····················	····················		
A	6782 hCoV-19/USA/AK-PHL015/2020|EPI_ISL_420306|2020-03-	····················	····················	····················	····················	····················	····················		
A	6783 hCoV-19/USA/NY-NYUMC52/2020|EPI_ISL_420307|2020-03	····················	····················	····················	····················	····················	····················		
A	6784 hCoV-19/USA/NY-NYUMC53/2020|EPI_ISL_420308|2020-03	····················	····················	····················	····················	····················	····················		
A	6785 hCoV-19/USA/NY-NYUMC54/2020|EPI_ISL_420309|2020-03	····················	····················	····················	····················	····················	····················		
A	6786 hCoV-19/Belgium/MCW-030574/2020|EPI_ISL_420330|202	····················	····················	····················	····················	····················	····················		
A	6787 hCoV-19/Belgium/LY-030575/2020|EPI_ISL_420331|2020	····················	····················	····················	····················	····················	····················		
A	6788 hCoV-19/Belgium/WAM-030676/2020|EPI_ISL_420332|202	····················	····················	····················	····················	····················	····················		
A	6789 hCoV-19/Belgium/RS-030677/2020|EPI_ISL_420333|2020	····················	····················	····················	····················	····················	····················		
A	6790 hCoV-19/Wales/PHWC-25524/2020|EPI_ISL_421002|2020-	····················	····A···············	····················	····················	····················	····················		
A	6791 hCoV-19/Wales/PHWC-25542/2020|EPI_ISL_421003|2020-	····················	····················	····················	····················	····················	····················		

A	6792	hCoV-19/Wales/PHWC-25551/2020|EPI_ISL_421004|2020-	···················· ···················· ····················· ···················· ···················· ····················
A	6793	hCoV-19/Wales/PHWC-25560/2020|EPI_ISL_421005|2020-	···················· ···················· ····················· ···················· ···················· ····················
A	6794	hCoV-19/Wales/PHWC-2557F/2020|EPI_ISL_421006|2020-	···················· ···················· ····················· ···················· ···················· ····················
A	6795	hCoV-19/Wales/PHWC-255AC/2020|EPI_ISL_421008|2020-	···················· ···················· ····················· ···················· ···················· ····················
A	6796	hCoV-19/Wales/PHWC-255D9/2020|EPI_ISL_421009|2020-	···················· ····A··············· ····················· ···················· ···················· ····················
A	6797	hCoV-19/Wales/PHWC-255E8/2020|EPI_ISL_421010|2020-	···················· ····A··············· ····················· ···················· ···················· ····················
A	6798	hCoV-19/Wales/PHWC-255F7/2020|EPI_ISL_421011|2020-	···················· ···················· ····················· ···················· ···················· ····················
A	6799	hCoV-19/USA/NY-PV09075/2020|EPI_ISL_421621|2020-03	···················· ···················· ····················· ···················· ···················· ····················
A	6800	hCoV-19/USA/NY-PV09077/2020|EPI_ISL_421623|2020-03	···················· ···················· ····················· ···················· ···················· ····················
A	6801	hCoV-19/USA/NY-PV09079/2020|EPI_ISL_421624|2020-03	···················· ···················· ····················· ···················· ···················· ····················
A	6802	hCoV-19/USA/NY-PV09083/2020|EPI_ISL_421626|2020-03	···················· ···················· ····················· ···················· ···················· ····················
A	6803	hCoV-19/USA/NY-PV09087/2020|EPI_ISL_421627|2020-03	···················· ···················· ····················· ···················· ···················· ····················
A	6804	hCoV-19/USA/NY-PV09088/2020|EPI_ISL_421628|2020-03	···················· ···················· ····················· ···················· ···················· ····················
A	6805	hCoV-19/USA/NY-PV09090/2020|EPI_ISL_421629|2020-03	···················· ···················· ····················· ···················· ···················· ····················
A	6806	hCoV-19/USA/NY-PV09092/2020|EPI_ISL_421630|2020-03	···················· ···················· ····················· ···················· ···················· ····················
A	6807	hCoV-19/USA/NY-PV09097/2020|EPI_ISL_421631|2020-03	···················· ···················· ····················· ···················· ···················· ····················
A	6808	hCoV-19/USA/NY-PV09098/2020|EPI_ISL_421632|2020-03	···················· ···················· ····················· ···················· ···················· ····················
A	6809	hCoV-19/USA/NY-PV09099/2020|EPI_ISL_421633|2020-03	···················· ···················· ····················· ···················· ···················· ····················
A	6810	hCoV-19/USA/NY-PV09102/2020|EPI_ISL_421634|2020-03	···················· ···················· ····················· ···················· ···················· ····················
A	6811	hCoV-19/USA/NY-PV09103/2020|EPI_ISL_421635|2020-03	···················· ···················· ····················· ···················· ···················· ····················
A	6812	hCoV-19/Australia/NSWID930/2020|EPI_ISL_421636|202	···················· ···················· ····················· ···················· ···················· ····················
A	6813	hCoV-19/Taiwan/144/2020|EPI_ISL_421641|2020-03-19	···················· ···················· ····················· ···················· ···················· ····················
A	6814	hCoV-19/USA/OH-OSUP0109/2020|EPI_ISL_421660|2020-0	···················· ···················· ····················· ···················· ···················· ····················
A	6815	hCoV-19/India/UN-1073/2020|EPI_ISL_421662|2020-03-	···················· ···················· ····················· ···················· ···················· ····················
A	6816	hCoV-19/India/UN-1093/2020|EPI_ISL_421663|2020-03-	···················· ···················· ····················· ···················· ···················· ····················
A	6817	hCoV-19/India/UN-1100/2020|EPI_ISL_421664|2020-03-	···················· ···················· ····················· ···················· ···················· ····················
A	6818	hCoV-19/Netherlands/ZuidHolland_84/2020|EPI_ISL_42	···················· ···················· ····················· ···················· ···················· ····················
A	6819	hCoV-19/Netherlands/ZuidHolland_86/2020|EPI_ISL_42	···················· ···················· ····················· ···················· ···················· ····················
A	6820	hCoV-19/Netherlands/ZuidHolland_88/2020|EPI_ISL_42	···················· ···················· ····················· ···················· ···················· ····················
A	6821	hCoV-19/Netherlands/ZuidHolland_89/2020|EPI_ISL_42	···················· ···················· ····················· ···················· ···················· ····················
A	6822	hCoV-19/Netherlands/ZuidHolland_90/2020|EPI_ISL_42	···················· ···················· ····················· ···················· ···················· ····················
A	6823	hCoV-19/Netherlands/ZuidHolland_93/2020|EPI_ISL_42	···················· ···················· ····················· ···················· ···················· ····················
A	6824	hCoV-19/USA/IL-UW-1265/2020|EPI_ISL_422961|2020-03	···················· ···················· ····················· ···················· ···················· ····················
A	6825	hCoV-19/USA/UN-UW-1312/2020|EPI_ISL_422962|2020-03	···················· ···················· ····················· ···················· ···················· ····················
A	6826	hCoV-19/USA/WA-UW-1271/2020|EPI_ISL_422963|2020-03	···················· ···················· ····················· ···················· ···················· ····················
A	6827	hCoV-19/USA/WA-UW-1304/2020|EPI_ISL_422964|2020-03	···················· ···················· ····················· ···················· ···················· ····················
A	6828	hCoV-19/USA/WA-UW-1315/2020|EPI_ISL_422965|2020-03	···················· ···················· ····················· ···················· ···················· ····················
A	6829	hCoV-19/USA/WA-UW-1329/2020|EPI_ISL_422966|2020-03	···················· ···················· ····················· ···················· ···················· ····················
A	6830	hCoV-19/USA/WA-UW-1302/2020|EPI_ISL_422967|2020-03	···················· ···················· ····················· ···················· ···················· ····················
A	6831	hCoV-19/USA/UN-UW-1331/2020|EPI_ISL_422968|2020-03	···················· ···················· ····················· ···················· ···················· ····················
A	6832	hCoV-19/USA/WA-UW-1287/2020|EPI_ISL_422969|2020-03	···················· ···················· ····················· ···················· ···················· ····················
A	6833	hCoV-19/USA/WA-UW-1269/2020|EPI_ISL_422970|2020-03	···················· ···················· ····················· ···················· ···················· ····················
A	6834	hCoV-19/USA/WA-UW-1320/2020|EPI_ISL_422971|2020-03	···················· ···················· ····················· ···················· ···················· ····················
A	6835	hCoV-19/USA/WA-UW-1319/2020|EPI_ISL_422972|2020-03	···················· ···················· ····················· ···················· ···················· ····················
A	6836	hCoV-19/USA/WA-UW-1308/2020|EPI_ISL_422973|2020-03	···················· ···················· ····················· ···················· ···················· ····················
A	6837	hCoV-19/USA/WA-UW-1295/2020|EPI_ISL_422990|2020-03	···················· ···················· ····················· ···················· ···················· ····················
A	6838	hCoV-19/USA/WA-UW-1314/2020|EPI_ISL_422991|2020-03	···················· ···················· ····················· ···················· ···················· ····················
A	6839	hCoV-19/USA/WA-UW-1347/2020|EPI_ISL_422992|2020-03	···················· ···················· ····················· ···················· ···················· ····················
A	6840	hCoV-19/USA/WA-UW-1321/2020|EPI_ISL_422993|2020-03	···················· ···················· ····················· ···················· ···················· ····················
A	6841	hCoV-19/USA/WA-UW-1322/2020|EPI_ISL_422994|2020-03	···················· ···················· ····················· ···················· ···················· ····················
A	6842	hCoV-19/USA/WA-UW-1334/2020|EPI_ISL_422995|2020-03	···················· ···················· ····················· ···················· ···················· ····················
A	6843	hCoV-19/England/20130063804/2020|EPI_ISL_423648|20	···················· ···················· ····················· ···················· ···················· ····················
A	6845	hCoV-19/England/20130065304/2020|EPI_ISL_423654|20	···················· ···················· ····················· ···················· ···················· ····················
A	6846	hCoV-19/England/20130072204/2020|EPI_ISL_423665|20	···················· ···················· ····················· ···················· ···················· ····················
A	6849	hCoV-19/England/20130092104/2020|EPI_ISL_423679|20	···················· ···················· ····················· ···················· ···················· ····················
A	6850	hCoV-19/England/20130092404/2020|EPI_ISL_423682|20	···················· ···················· ····················· ···················· ···················· ····················
A	6852	hCoV-19/Taiwan/CGMH-CGU-20/2020|EPI_ISL_424978|202	···················· ···················· ····················· ···················· ···················· ····················
A	6853	hCoV-19/USA/NC_200191/2020|EPI_ISL_424983|2020-03	···················· ···················· ····················· ···················· ···················· ····················
A	6854	hCoV-19/USA/NC_200181/2020|EPI_ISL_424987|2020-03	···················· ···················· ····················· ···················· ···················· ····················
A	6855	hCoV-19/France/OCC-KRA-ROB/2020|EPI_ISL_424993|202	···················· ···················· ····················· ···················· ···················· ····················
A	6856	hCoV-19/USA/WA-UW-3881/2020|EPI_ISL_427200|2020-03	···················· ···················· ····················· ···················· ···················· ····················
A	6857	hCoV-19/USA/WA-UW-3883/2020|EPI_ISL_427201|2020-03	···················· ···················· ····················· ···················· ···················· ····················
A	6858	hCoV-19/USA/WA-UW-3889/2020|EPI_ISL_427202|2020-03	···················· ···················· ····················· ···················· ···················· ····················
A	6859	hCoV-19/USA/WA-UW-3895/2020|EPI_ISL_427203|2020-03	···················· ···················· ····················· ···················· ···················· ····················
A	6860	hCoV-19/USA/WA-UW-3900/2020|EPI_ISL_427204|2020-03	···················· ···················· ····················· ···················· ···················· ····················
A	6861	hCoV-19/USA/WA-UW-3903/2020|EPI_ISL_427205|2020-03	···················· ···················· ····················· ···················· ···················· ····················
A	6862	hCoV-19/USA/WA-UW-3908/2020|EPI_ISL_427206|2020-03	···················· ···················· ····················· ···················· ···················· ····················
A	6863	hCoV-19/USA/WA-UW-3911/2020|EPI_ISL_427207|2020-03	···················· ···················· ····················· ···················· ···················· ····················
A	6864	hCoV-19/USA/WA-UW-3917/2020|EPI_ISL_427208|2020-03	···················· ···················· ····················· ···················· ···················· ····················
A	6865	hCoV-19/USA/WA-UW-4291/2020|EPI_ISL_427255|2020-03	···················· ···················· ····················· ···················· ···················· ····················
A	6866	hCoV-19/USA/WA-UW-4292/2020|EPI_ISL_427256|2020-03	···················· ···················· ····················· ···················· ···················· ····················
A	6867	hCoV-19/USA/WA-UW-4315/2020|EPI_ISL_427257|2020-03	···················· ···················· ····················· ···················· ···················· ····················
A	6868	hCoV-19/USA/WA-UW-4316/2020|EPI_ISL_427258|2020-03	···················· ···················· ····················· ···················· ···················· ····················
A	6869	hCoV-19/USA/WA-UW-4318/2020|EPI_ISL_427259|2020-03	···················· ···················· ····················· ···················· ···················· ····················
A	6870	hCoV-19/USA/WA-UW-4321/2020|EPI_ISL_427260|2020-03	···················· ···················· ····················· ···················· ···················· ····················
A	6871	hCoV-19/USA/WA-UW-4322/2020|EPI_ISL_427261|2020-03	···················· ···················· ····················· ···················· ···················· ····················
A	6872	hCoV-19/USA/WA-UW-4324/2020|EPI_ISL_427262|2020-03	···················· ···················· ····················· ···················· ···················· ····················
A	6873	hCoV-19/USA/WA-UW-4326/2020|EPI_ISL_427263|2020-03	···················· ···················· ····················· ···················· ···················· ····················

A	6874 hCoV-19/USA/WA-UW-4329/2020|EPI_ISL_427264|2020-03	····················	····················	····················	····················	····················	····················
A	6875 hCoV-19/USA/WA-UW-4330/2020|EPI_ISL_427265|2020-03	····················	····················	····················	····················	····················	····················
A	6876 hCoV-19/USA/WA-UW-4335/2020|EPI_ISL_427266|2020-03	····················	····················	····················	····················	····················	····················
A	6877 hCoV-19/USA/WA-UW-4340/2020|EPI_ISL_427268|2020-03	····················	····················	····················	····················	····················	····················
A	6878 hCoV-19/USA/WA-UW-4499/2020|EPI_ISL_427269|2020-03	····················	····················	····················	····················	····················	····················
A	6879 hCoV-19/Croatia/17-S7/2020|EPI_ISL_429802|2020-03	····················	····················	····················	····················	····················	····················
A	6880 hCoV-19/Croatia/I7-S21/2020|EPI_ISL_429803|2020-03	····················	····················	····················	····················	····················	····················
A	6881 hCoV-19/Croatia/7R-S8/2020|EPI_ISL_429805|2020-03	····················	····················	····················	····················	····················	····················
A	6882 hCoV-19/Canada/NB_1/2020|EPI_ISL_429806|2020-03-09	····················	····················	····················	····················	····················	····················
A	6883 hCoV-19/Canada/MB_2/2020|EPI_ISL_429807|2020-03-10	····················	····················	····················	····················	····················	····················
A	6884 hCoV-19/Canada/NB_21/2020|EPI_ISL_429811|2020-03-1	····················	····················	····················	····················	····················	····················
A	6885 hCoV-19/Canada/NS_30/2020|EPI_ISL_429812|2020-03-1	····················	····················	····················	····················	····················	····················
A	6886 hCoV-19/Canada/NS_31/2020|EPI_ISL_429813|2020-03-1	····················	····················	····················	····················	····················	····················
A	6887 hCoV-19/Canada/NS_32/2020|EPI_ISL_429814|2020-03-1	····················	····················	····················	····················	····················	····················
A	6888 hCoV-19/Canada/NL_34/2020|EPI_ISL_429815|2020-03-1	····················	····················	····················	····················	····················	····················
A	6889 hCoV-19/Canada/NB_35/2020|EPI_ISL_429816|2020-03-1	····················	····················	····················	····················	····················	····················
A	6890 hCoV-19/Canada/MB_36/2020|EPI_ISL_429817|2020-03-1	····················	····················	····················	····················	····················	····················
A	6891 hCoV-19/Canada/MB_37/2020|EPI_ISL_429818|2020-03-1	····················	····················	····················	····················	····················	····················
A	6892 hCoV-19/Canada/MB_38/2020|EPI_ISL_429819|2020-03-1	····················	····················	····················	····················	····················	····················
A	6893 hCoV-19/Canada/MB_39/2020|EPI_ISL_429820|2020-03-1	····················	····················	····················	····················	····················	····················
A	6894 hCoV-19/USA/WI-GMF-00822/2020|EPI_ISL_429843|2020-	····················	····················	····················	····················	····················	····················
A	6895 hCoV-19/USA/WI-GMF-00857/2020|EPI_ISL_429844|2020-	····················	····················	····················	····················	····················	····················
A	6896 hCoV-19/USA/WI-GMF-01047/2020|EPI_ISL_429845|2020-	····················	····················	····················	····················	····················	····················
A	6897 hCoV-19/USA/WI-GMF-01158/2020|EPI_ISL_429846|2020-	····················	····················	····················	····················	····················	····················
A	6898 hCoV-19/USA/WI-GMF-01159/2020|EPI_ISL_429847|2020-	····················	····················	····················	····················	····················	····················
A	6899 hCoV-19/USA/WI-GMF-01316/2020|EPI_ISL_429848|2020-	····················	····················	····················	····················	····················	····················
A	6900 hCoV-19/Lishui/LS111/2020|EPI_ISL_429852|2020-01-2	····················	····················	····················	····················	····················	····················
A	6901 hCoV-19/Lishui/LS556/2020|EPI_ISL_429853|2020-01-2	····················	····················	····················	····················	····················	····················
A	6902 hCoV-19/Lishui/LS557/2020|EPI_ISL_429854|2020-02-0	····················	····················	····················	····················	····················	····················
A	6903 hCoV-19/Turkey/HSGM-8968/2020|EPI_ISL_429861|2020-	····················	·A··················	····················	····················	····················	···A················
A	6904 hCoV-19/Turkey/HSGM-8970/2020|EPI_ISL_429862|2020-	····················	····················	····················	····················	····················	····················
A	6905 hCoV-19/Turkey/HSGM-8975/2020|EPI_ISL_429863|2020-	····················	·A··················	····················	····················	····················	···A················
A	6906 hCoV-19/Turkey/HSGM-8979/2020|EPI_ISL_429864|2020-	····················	····················	····················	····················	····················	····················
A	6907 hCoV-19/Turkey/HSGM-8992/2020|EPI_ISL_429865|2020-	····················	····················	····················	····················	····················	····················
A	6908 hCoV-19/Turkey/HSGM-4236/2020|EPI_ISL_429866|2020-	····················	····················	····················	····················	····················	····················
A	6909 hCoV-19/Turkey/HSGM-4505/2020|EPI_ISL_429867|2020-	····················	····················	····················	····················	····················	····················
A	6910 hCoV-19/Turkey/HSGM-4598/2020|EPI_ISL_429868|2020-	····················	····················	····················	····················	····················	····················
A	6911 hCoV-19/Turkey/HSGM-4701/2020|EPI_ISL_429869|2020-	····················	·A··················	····················	····················	····················	···A················
A	6912 hCoV-19/Germany/BY-ChVir-1019/2020|EPI_ISL_450199|	····················	····················	····················	····················	····················	····················
A	6913 hCoV-19/Taiwan/2/2020|EPI_ISL_406031|2020-01-23	····················	····················	····················	····················	····················	····················
A	6914 hCoV-19/Portugal/PT0015/2020|EPI_ISL_418000|2020-0	····················	····················	····················	····················	····················	····················
A	6915 hCoV-19/Portugal/PT0016/2020|EPI_ISL_418001|2020-0	····················	····················	····················	····················	····················	····················
A	6916 hCoV-19/Portugal/PT0017/2020|EPI_ISL_418002|2020-0	····················	····················	····················	····················	····················	····················
A	6917 hCoV-19/Portugal/PT0018/2020|EPI_ISL_418003|2020-0	····················	····················	····················	····················	····················	····················
A	6918 hCoV-19/Portugal/PT0019/2020|EPI_ISL_418004|2020-0	····················	····················	····················	····················	····················	····················
A	6919 hCoV-19/Portugal/PT0020/2020|EPI_ISL_418005|2020-0	····················	····················	····················	····················	····················	····················
A	6920 hCoV-19/Portugal/PT0021/2020|EPI_ISL_418006|2020-0	····················	····················	····················	····················	····················	····················
A	6921 hCoV-19/Portugal/PT0022/2020|EPI_ISL_418007|2020-0	····················	····················	····················	····················	····················	····················
A	6922 hCoV-19/Portugal/PT0023/2020|EPI_ISL_418008|2020-0	····················	····················	····················	····················	····················	····················
A	6923 hCoV-19/Portugal/PT0024/2020|EPI_ISL_418009|2020-0	····················	····················	····················	····················	····················	····················
A	6924 hCoV-19/Portugal/PT0035/2020|EPI_ISL_418020|2020-0	····················	····················	····················	····················	····················	····················
A	6925 hCoV-19/Portugal/PT0036/2020|EPI_ISL_418021|2020-0	····················	····················	····················	····················	····················	····················
A	6926 hCoV-19/Portugal/PT0037/2020|EPI_ISL_418022|2020-0	····················	····················	····················	····················	····················	····················
A	6927 hCoV-19/Portugal/PT0038/2020|EPI_ISL_418023|2020-0	····················	····················	····················	····················	····················	····················
A	6928 hCoV-19/Portugal/PT0039/2020|EPI_ISL_418024|2020-0	····················	····················	····················	····················	····················	····················
A	6929 hCoV-19/Portugal/PT0040/2020|EPI_ISL_418025|2020-0	····················	····················	····················	····················	····················	····················
A	6930 hCoV-19/Portugal/PT0041/2020|EPI_ISL_418026|2020-0	····················	····················	····················	····················	····················	····················
A	6931 hCoV-19/Portugal/PT0042/2020|EPI_ISL_418027|2020-0	····················	····················	····················	····················	····················	····················
A	6932 hCoV-19/USA/MN-UW243/2020|EPI_ISL_418028|2020-03-1	····················	····················	····················	····················	····················	····················
A	6933 hCoV-19/USA/WA-UW244/2020|EPI_ISL_418029|2020-03-1	····················	····················	····················	····················	····················	····················
A	6934 hCoV-19/USA/WA-UW245/2020|EPI_ISL_418030|2020-03-1	····················	····················	····················	····················	····················	····················
A	6935 hCoV-19/USA/WA-UW247/2020|EPI_ISL_418032|2020-03-1	····················	····················	····················	····················	····················	····················
A	6936 hCoV-19/USA/WA-UW248/2020|EPI_ISL_418033|2020-03-1	····················	····················	····················	····················	····················	····················
A	6937 hCoV-19/USA/WA-UW249/2020|EPI_ISL_418034|2020-03-1	····················	····················	····················	····················	····················	····················
A	6938 hCoV-19/USA/MN-UW251/2020|EPI_ISL_418036|2020-03-1	····················	····················	····················	····················	····················	····················
A	6939 hCoV-19/USA/WA-UW252/2020|EPI_ISL_418037|2020-03-1	····················	····················	····················	····················	····················	····················
A	6940 hCoV-19/USA/WA-UW253/2020|EPI_ISL_418038|2020-03-1	····················	····················	····················	····················	····················	····················
A	6941 hCoV-19/USA/CT-UW254/2020|EPI_ISL_418039|2020-03-1	····················	····················	····················	····················	····················	····················
A	6942 hCoV-19/USA/WA-UW255/2020|EPI_ISL_418040|2020-03-1	····················	····················	····················	····················	····················	····················
A	6943 hCoV-19/USA/CT-UW256/2020|EPI_ISL_418041|2020-03-1	····················	····················	····················	····················	····················	····················
A	6944 hCoV-19/USA/MN-UW257/2020|EPI_ISL_418042|2020-03-1	····················	····················	····················	····················	····················	····················
A	6945 hCoV-19/USA/CT-UW258/2020|EPI_ISL_418043|2020-03-1	····················	····················	····················	····················	····················	····················
A	6946 hCoV-19/USA/CT-UW259/2020|EPI_ISL_418044|2020-03-1	····················	····················	····················	····················	····················	····················
A	6947 hCoV-19/USA/CT-UW260/2020|EPI_ISL_418045|2020-03-1	····················	····················	····················	····················	····················	····················
A	6948 hCoV-19/USA/WA-UW261/2020|EPI_ISL_418046|2020-03-1	····················	····················	····················	····················	····················	····················
A	6949 hCoV-19/USA/WA-UW262/2020|EPI_ISL_418047|2020-03-1	····················	····················	····················	····················	····················	····················
A	6950 hCoV-19/USA/MN-UW264/2020|EPI_ISL_418049|2020-03-1	····················	····················	····················	····················	····················	····················
A	6951 hCoV-19/USA/WA-UW265/2020|EPI_ISL_418050|2020-03-1	····················	····················	····················	····················	····················	····················

A	6952 hCoV-19/USA/MN-UW266/2020|EPI_ISL_418051|2020-03-1	....	....	....	....	....	....
A	6953 hCoV-19/USA/WA-UW268/2020|EPI_ISL_418053|2020-03-1	....	....	....	....	....	....
A	6954 hCoV-19/USA/WA-UW269/2020|EPI_ISL_418054|2020-03-1	....	....	....	....	....	....
A	6955 hCoV-19/USA/WA-UW270/2020|EPI_ISL_418055|2020-03-1	....	....	....	....	....	....
A	6956 hCoV-19/USA/CT-UW271/2020|EPI_ISL_418056|2020-03-1	....	....	....	....	....	....
A	6957 hCoV-19/USA/CT-UW272/2020|EPI_ISL_418057|2020-03-1	....	....	....	....	....	....
A	6958 hCoV-19/USA/CT-UW273/2020|EPI_ISL_418058|2020-03-1	....	....	....	....	....	....
A	6959 hCoV-19/USA/WA-UW274/2020|EPI_ISL_418059|2020-03-1	....	....	....	....	....	....
A	6960 hCoV-19/Japan/P3-2/2020|EPI_ISL_419300|2020-03-20	....	....	....	....	....	....
A	6961 hCoV-19/Japan/P4-1/2020|EPI_ISL_419301|2020-03-11	....	....	....	....	....	....
A	6962 hCoV-19/Japan/P4-2/2020|EPI_ISL_419302|2020-03-12	....	....	....	....	....	....
A	6963 hCoV-19/Japan/P4-3/2020|EPI_ISL_419303|2020-03-12	....	....	....	....	....	....
A	6964 hCoV-19/Japan/P4-4/2020|EPI_ISL_419304|2020-03-17	....	....	....	....	....	....
A	6965 hCoV-19/Japan/P4-5/2020|EPI_ISL_419305|2020-03-18	....	....	....	....	....	....
A	6966 hCoV-19/Japan/P4-6/2020|EPI_ISL_419306|2020-03-20	....	....	....	....	....	....
A	6967 hCoV-19/Japan/P4-7/2020|EPI_ISL_419307|2020-03-20	....	....	....	....	....	....
A	6968 hCoV-19/Japan/P4-8/2020|EPI_ISL_419308|2020-03-19	....	....	....	....	....	....
A	6969 hCoV-19/Japan/P5-1/2020|EPI_ISL_419309|2020-03-12	....	....	....	....	....	....
A	6970 hCoV-19/Portugal/IGC00009/2020|EPI_ISL_419386|2020	....	....	....	....	....	....
A	6971 hCoV-19/Portugal/IGC00010/2020|EPI_ISL_419387|2020	....	....	....	....	....	....
A	6972 hCoV-19/USA/MN60-MDH60/2020|EPI_ISL_419388|2020-03	....	....	....	....	....	....
A	6973 hCoV-19/Norway/2114/2020|EPI_ISL_420312|2020-03-18	....	....	....	....	....	....
A	6974 hCoV-19/Norway/1953/2020|EPI_ISL_420313|2020-03-10	....	....	....	....	....	....
A	6975 hCoV-19/Belgium/NL-030447/2020|EPI_ISL_420314|2020	....	....	....	....	....	....
A	6976 hCoV-19/Belgium/RYR-030649/2020|EPI_ISL_420315|202	....	....	....	....	....	....
A	6977 hCoV-19/Belgium/QOJ-030751/2020|EPI_ISL_420316|202	....	....	....	....	....	....
A	6978 hCoV-19/Belgium/RJ-030552/2020|EPI_ISL_420317|2020	....	....	....	....	....	....
A	6979 hCoV-19/Belgium/TG-030757/2020|EPI_ISL_420318|2020	....	....	....	....	....	....
A	6980 hCoV-19/Belgium/GJ-030458/2020|EPI_ISL_420319|2020	....	....	....	....	....	....
A	6981 hCoV-19/Belgium/030959/2020|EPI_ISL_420320|2020-03	....	....	....	....	....	....
A	6982 hCoV-19/Belgium/WWM-030665/2020|EPI_ISL_420321|202	....	....	....	....	....	....
A	6983 hCoV-19/Belgium/DBOM-030566/2020|EPI_ISL_420322|20	....	....	....	....	....	....
A	6984 hCoV-19/Belgium/BJ-030767/2020|EPI_ISL_420323|2020	....	....	....	....	....	....
A	6985 hCoV-19/Belgium/DVBJ-030468/2020|EPI_ISL_420324|20	....	....	....	....	....	....
A	6986 hCoV-19/Belgium/DLDJ-030569/2020|EPI_ISL_420325|20	....	....	....	....	....	....
A	6987 hCoV-19/Belgium/AKM-030670/2020|EPI_ISL_420326|202	....	....	....	....	....	....
A	6988 hCoV-19/Belgium/HL-030771/2020|EPI_ISL_420327|2020	....	....	....	....	....	....
A	6989 hCoV-19/Belgium/VGA-030672/2020|EPI_ISL_420328|202	....	....	....	....	....	....
A	6990 hCoV-19/Belgium/GE-030573/2020|EPI_ISL_420329|2020	....	....	....	....	....	....
A	6991 hCoV-19/Belgium/GOF-030578/2020|EPI_ISL_420334|202	....	....	....	....	....	....
A	6992 hCoV-19/Belgium/CD-030679/2020|EPI_ISL_420335|2020	....	....	....	....	....	....
A	6993 hCoV-19/Belgium/PA-030680/2020|EPI_ISL_420336|2020	....	....	....	....	....	....
A	6994 hCoV-19/Belgium/PAN-030681/2020|EPI_ISL_420337|202	....	....	....	....	....	....
A	6995 hCoV-19/Belgium/AC-030982/2020|EPI_ISL_420338|2020	....	....	....	....	....	....
A	6996 hCoV-19/Belgium/SJ-030583/2020|EPI_ISL_420339|2020	....	....	....	....	....	....
A	6997 hCoV-19/Belgium/MJP-030684/2020|EPI_ISL_420340|202	....	....	....	....	....	....
A	6998 hCoV-19/Belgium/LM-030685/2020|EPI_ISL_420341|2020	....	....	....	....	....	....
A	6999 hCoV-19/Belgium/ECC-030686/2020|EPI_ISL_420342|202	....	....	....	....	....	....
A	7000 hCoV-19/Belgium/BM-030687/2020|EPI_ISL_420343|2020	....	....	....	....	....	....
A	7001 hCoV-19/Belgium/RJL-030588/2020|EPI_ISL_420344|202	....	....	....	....	....	....
A	7002 hCoV-19/Belgium/MC-030689/2020|EPI_ISL_420345|2020	....	....	....	....	....	....
A	7003 hCoV-19/Belgium/SB-030990/2020|EPI_ISL_420346|2020	....	....	....	....	....	....
A	7004 hCoV-19/Belgium/DA-030691/2020|EPI_ISL_420347|2020	....	....	....	....	....	....
A	7005 hCoV-19/Belgium/FM-030592/2020|EPI_ISL_420348|2020	....	....	....	....	....	....
A	7006 hCoV-19/Belgium/DD-030593/2020|EPI_ISL_420349|2020	....	....	....	....	....	....
A	7007 hCoV-19/Belgium/BDW-030694/2020|EPI_ISL_420350|202	....	....	....	....	....	....
A	7008 hCoV-19/Belgium/VMC-030695/2020|EPI_ISL_420351|202	....	....	....	....	....	....
A	7009 hCoV-19/Belgium/DCS-030796/2020|EPI_ISL_420352|202	....	....	....	....	....	....
A	7010 hCoV-19/Belgium/LD-030597/2020|EPI_ISL_420353|2020	....	....	....	....	....	....
A	7011 hCoV-19/Belgium/BS-030598/2020|EPI_ISL_420354|2020	....	....	....	....	....	....
A	7012 hCoV-19/Belgium/RR-030699/2020|EPI_ISL_420355|2020	....	....	....	....	....	....
A	7013 hCoV-19/Belgium/MJP-0306100/2020|EPI_ISL_420356|20	....	....	....	....	....	....
A	7014 hCoV-19/Belgium/JLJ-0320101/2020|EPI_ISL_420357|20	....	....	....	....	....	....
A	7015 hCoV-19/Belgium/VD-0318102/2020|EPI_ISL_420358|202	....	....	....	....	....	....
A	7016 hCoV-19/Belgium/UM-0318103/2020|EPI_ISL_420359|202	....	....	....	....	....	....
A	7017 hCoV-19/Belgium/VL-0318104/2020|EPI_ISL_420360|202	....	....	....	....	....	....
A	7018 hCoV-19/Belgium/GE-0317105/2020|EPI_ISL_420361|202	....	....	....	....	....	....
A	7019 hCoV-19/Belgium/DA-0319106/2020|EPI_ISL_420362|202	....	....	....	....	....	....
A	7020 hCoV-19/Belgium/VNP-0320107/2020|EPI_ISL_420363|20	....	....	....	....	....	....
A	7021 hCoV-19/Belgium/HP-0319108/2020|EPI_ISL_420364|202	....	....	....	....	....	....
A	7022 hCoV-19/Belgium/OT-0319109/2020|EPI_ISL_420365|202	....	....	....	....	....	....
A	7023 hCoV-19/Belgium/KKN-0319184/2020|EPI_ISL_420366|20	....	....	....	....	....	....
A	7024 hCoV-19/Belgium/JK-0305110/2020|EPI_ISL_420367|202	....	....	....	....	....	....
A	7025 hCoV-19/Belgium/CA-0319111/2020|EPI_ISL_420368|202	....	....	....	....	....	....
A	7026 hCoV-19/Belgium/SR-0319112/2020|EPI_ISL_420369|202	....	....	....	....	....	....
A	7027 hCoV-19/Belgium/SC-0319113/2020|EPI_ISL_420370|202	....	....	....	....	....	....
A	7028 hCoV-19/Belgium/VRJM-0323114/2020|EPI_ISL_420371|2	....	....	....	....	....	....
A	7029 hCoV-19/Belgium/LPDC-0323115/2020|EPI_ISL_420372|2	....	....	....	....	....	....

A	7030	hCoV-19/Belgium/EBN-0323116/2020|EPI_ISL_420373|20	····················	····················	····················	····················	····················	····················
A	7031	hCoV-19/Belgium/DV-0324117/2020|EPI_ISL_420374|202	····················	····················	····················	····················	····················	····················
A	7032	hCoV-19/Belgium/VHV-0324118/2020|EPI_ISL_420375|20	····················	····················	····················	····················	····················	····················
A	7033	hCoV-19/Belgium/CF-0324119/2020|EPI_ISL_420376|202	····················	····················	····················	····················	····················	····················
A	7034	hCoV-19/Belgium/SEM-0324120/2020|EPI_ISL_420377|20	····················	····················	····················	····················	····················	····················
A	7035	hCoV-19/USA/NY-PV09044/2020|EPI_ISL_421607|2020-03	····················	····················	····················	····················	····················	····················
A	7036	hCoV-19/USA/NY-PV09056/2020|EPI_ISL_421611|2020-03	····················	····················	····················	····················	····················	····················
A	7037	hCoV-19/USA/NY-PV09060/2020|EPI_ISL_421612|2020-03	····················	····················	····················	····················	····················	····················
A	7038	hCoV-19/USA/NY-PV09061/2020|EPI_ISL_421613|2020-03	···T················	····················	····················	····················	····················	····················
A	7039	hCoV-19/USA/NY-PV09063/2020|EPI_ISL_421614|2020-03	····················	····················	····················	····················	····················	····················
A	7040	hCoV-19/USA/NY-PV09064/2020|EPI_ISL_421615|2020-03	·T··················	····················	····················	····················	····················	····················
A	7041	hCoV-19/USA/NY-PV09067/2020|EPI_ISL_421616|2020-03	····················	····················	····················	····················	····················	····················
A	7042	hCoV-19/USA/NY-PV09068/2020|EPI_ISL_421617|2020-03	····················	····················	····················	····················	····················	····················
A	7043	hCoV-19/USA/NJ-PV09072/2020|EPI_ISL_421619|2020-03	····················	····················	····················	····················	····················	····················
A	7044	hCoV-19/USA/NY-PV09073/2020|EPI_ISL_421620|2020-03	····················	····················	····················	····················	····················	····················
A	7045	hCoV-19/Taiwan/225/2020|EPI_ISL_421651|2020-03-22	····················	····················	····················	····················	····················	····················
A	7046	hCoV-19/Kuwait/KU001/2020|EPI_ISL_421652|2020-03-1	····················	····················	····················	····················	····················	····················
A	7047	hCoV-19/Latvia/01/2020|EPI_ISL_421653|2020-03-25	····················	····················	····················	····················	····················	····················
A	7048	hCoV-19/Latvia/02/2020|EPI_ISL_421654|2020-03-25	····················	····················	····················	····················	····················	····················
A	7049	hCoV-19/Latvia/03/2020|EPI_ISL_421655|2020-03-25	····················	····················	····················	····················	····················	····················
A	7050	hCoV-19/Latvia/04/2020|EPI_ISL_421656|2020-03-25	····················	····················	····················	····················	····················	····················
A	7051	hCoV-19/India/UN-1104/2020|EPI_ISL_421665|2020-03-	····················	····················	····················	····················	····················	····················
A	7052	hCoV-19/India/UN-1111/2020|EPI_ISL_421666|2020-03-	····················	····················	····················	····················	····················	····················
A	7053	hCoV-19/India/UN-1115/2020|EPI_ISL_421667|2020-03-	····················	····················	····················	····················	····················	····················
A	7054	hCoV-19/India/UN-1125/2020|EPI_ISL_421668|2020-03-	····················	····················	····················	····················	····················	····················
A	7055	hCoV-19/India/UN-1616/2020|EPI_ISL_421669|2020-03-	····················	····················	····················	····················	····················	····················
A	7056	hCoV-19/India/UN-1617/2020|EPI_ISL_421670|2020-03-	····················	····················	····················	····················	····················	····················
A	7057	hCoV-19/India/UN-1621/2020|EPI_ISL_421671|2020-03-	····················	····················	····················	····················	····················	····················
A	7058	hCoV-19/India/UN-1644/2020|EPI_ISL_421672|2020-03-	····················	····················	····················	····················	····················	····················
A	7059	hCoV-19/USA/OH-OSUP0112/2020|EPI_ISL_421675|2020-0	····················	····················	····················	····················	····················	····················
A	7060	hCoV-19/USA/MN124-MDH124/2020|EPI_ISL_421683|2020-	····················	····················	····················	····················	····················	····················
A	7061	hCoV-19/USA/MN128-MDH128/2020|EPI_ISL_421684|2020-	····················	····················	····················	····················	····················	····················
A	7062	hCoV-19/USA/MN129-MDH129/2020|EPI_ISL_421685|2020-	····················	····················	····················	····················	····················	····················
A	7063	hCoV-19/USA/MN130-MDH130/2020|EPI_ISL_421686|2020-	····················	····················	····················	····················	····················	····················
A	7064	hCoV-19/USA/MN131-MDH131/2020|EPI_ISL_421687|2020-	····················	····················	····················	····················	····················	····················
A	7065	hCoV-19/USA/MN132-MDH132/2020|EPI_ISL_421688|2020-	····················	····················	····················	····················	····················	····················
A	7066	hCoV-19/USA/MN133-MDH133/2020|EPI_ISL_421689|2020-	····················	····················	····················	····················	····················	····················
A	7067	hCoV-19/USA/MN134-MDH134/2020|EPI_ISL_421690|2020-	····················	····················	····················	····················	····················	····················
A	7068	hCoV-19/USA/MN135-MDH135/2020|EPI_ISL_421691|2020-	····················	····················	····················	····················	····················	····················
A	7069	hCoV-19/USA/MN136-MDH136/2020|EPI_ISL_421692|2020-	····················	····················	····················	····················	····················	····················
A	7070	hCoV-19/Netherlands/ZuidHolland_102/2020|EPI_ISL_4	····················	····················	····················	····················	····················	····················
A	7071	hCoV-19/Netherlands/ZuidHolland_103/2020|EPI_ISL_4	····················	····················	····················	····················	····················	····················
A	7072	hCoV-19/Netherlands/ZuidHolland_104/2020|EPI_ISL_4	····················	····················	····················	····················	····················	····················
A	7073	hCoV-19/Netherlands/ZuidHolland_32/2020|EPI_ISL_42	····················	····················	····················	····················	····················	····················
A	7074	hCoV-19/Netherlands/ZuidHolland_33/2020|EPI_ISL_42	····················	····················	····················	····················	····················	····················
A	7075	hCoV-19/Netherlands/ZuidHolland_35/2020|EPI_ISL_42	····················	····················	····················	····················	····················	····················
A	7076	hCoV-19/Netherlands/ZuidHolland_36/2020|EPI_ISL_42	····················	····················	····················	····················	····················	····················
A	7077	hCoV-19/Netherlands/ZuidHolland_37/2020|EPI_ISL_42	····················	····················	····················	····················	····················	····················
A	7078	hCoV-19/Netherlands/ZuidHolland_38/2020|EPI_ISL_42	····················	····················	····················	····················	····················	····················
A	7079	hCoV-19/Netherlands/ZuidHolland_39/2020|EPI_ISL_42	····················	····················	····················	····················	····················	····················
A	7080	hCoV-19/Netherlands/ZuidHolland_40/2020|EPI_ISL_42	····················	····················	····················	····················	····················	····················
A	7081	hCoV-19/Netherlands/ZuidHolland_41/2020|EPI_ISL_42	····················	····················	····················	····················	····················	····················
A	7082	hCoV-19/Netherlands/ZuidHolland_42/2020|EPI_ISL_42	····················	····················	····················	····················	····················	····················
A	7083	hCoV-19/Netherlands/ZuidHolland_43/2020|EPI_ISL_42	····················	····················	····················	····················	····················	····················
A	7084	hCoV-19/Netherlands/ZuidHolland_44/2020|EPI_ISL_42	····················	····················	····················	····················	····················	····················
A	7085	hCoV-19/Netherlands/ZuidHolland_45/2020|EPI_ISL_42	····················	····················	····················	····················	····················	····················
A	7086	hCoV-19/Netherlands/ZuidHolland_47/2020|EPI_ISL_42	····················	····················	····················	····················	····················	····················
A	7087	hCoV-19/Netherlands/ZuidHolland_48/2020|EPI_ISL_42	········T···········	····················	····················	····················	····················	····················
A	7088	hCoV-19/Netherlands/ZuidHolland_52/2020|EPI_ISL_42	····················	····················	····················	····················	····················	····················
A	7089	hCoV-19/Netherlands/ZuidHolland_55/2020|EPI_ISL_42	····················	····················	····················	····················	····················	····················
A	7090	hCoV-19/Netherlands/ZuidHolland_56/2020|EPI_ISL_42	····················	····················	····················	····················	····················	····················
A	7091	hCoV-19/Netherlands/ZuidHolland_57/2020|EPI_ISL_42	····················	····················	····················	····················	····················	····················
A	7092	hCoV-19/Netherlands/ZuidHolland_58/2020|EPI_ISL_42	····················	····················	····················	····················	····················	····················
A	7093	hCoV-19/Netherlands/ZuidHolland_61/2020|EPI_ISL_42	····················	····················	····················	····················	····················	····················
A	7094	hCoV-19/Netherlands/ZuidHolland_70/2020|EPI_ISL_42	····················	····················	····················	····················	····················	····················
A	7095	hCoV-19/Netherlands/ZuidHolland_72/2020|EPI_ISL_42	····················	····················	····················	····················	····················	····················
A	7096	hCoV-19/Netherlands/ZuidHolland_74/2020|EPI_ISL_42	····················	····················	····················	····················	····················	····················
A	7097	hCoV-19/Netherlands/ZuidHolland_75/2020|EPI_ISL_42	····················	····················	····················	····················	····················	····················
A	7098	hCoV-19/Netherlands/ZuidHolland_76/2020|EPI_ISL_42	····················	····················	····················	····················	····················	····················
A	7099	hCoV-19/Netherlands/ZuidHolland_77/2020|EPI_ISL_42	····················	····················	····················	····················	····················	····················
A	7100	hCoV-19/Netherlands/ZuidHolland_79/2020|EPI_ISL_42	····················	····················	····················	····················	····················	····················
A	7101	hCoV-19/Netherlands/ZuidHolland_80/2020|EPI_ISL_42	····················	····················	····················	····················	····················	····················
A	7102	hCoV-19/Netherlands/ZuidHolland_81/2020|EPI_ISL_42	····················	····················	····················	····················	····················	····················
A	7103	hCoV-19/Netherlands/ZuidHolland_82/2020|EPI_ISL_42	····················	····················	····················	····················	····················	····················
A	7104	hCoV-19/Netherlands/ZuidHolland_83/2020|EPI_ISL_42	····················	····················	····················	····················	····················	····················
A	7105	hCoV-19/USA/WA-UW-1279/2020|EPI_ISL_422974|2020-03	····················	····················	····················	····················	····················	····················
A	7106	hCoV-19/USA/WA-UW-1327/2020|EPI_ISL_422975|2020-03	····················	····················	····················	····················	····················	····················
A	7107	hCoV-19/USA/WA-UW-1306/2020|EPI_ISL_422976|2020-03	····················	····················	····················	····················	····················	····················

A	7108 hCoV-19/USA/WA-UW-1309/2020|EPI_ISL_422977|2020-03	....	....	....	....	....	....
A	7109 hCoV-19/USA/UN-UW-1291/2020|EPI_ISL_422978|2020-03	....	....	....	....	....	....
A	7110 hCoV-19/USA/UN-UW-1292/2020|EPI_ISL_422979|2020-03	....	....	....	....	....	....
A	7111 hCoV-19/USA/UN-UW-1307/2020|EPI_ISL_422980|2020-03	....	....	....	....	....	....
A	7112 hCoV-19/USA/WA-UW-1417/2020|EPI_ISL_422981|2020-03	....	....	....	....	....	....
A	7113 hCoV-19/USA/UN-UW-1403/2020|EPI_ISL_422982|2020-03	....	....	....	....	....	....
A	7114 hCoV-19/USA/UN-UW-1407/2020|EPI_ISL_422983|2020-03	....	....	....	....	....	....
A	7115 hCoV-19/USA/WA-UW-1300/2020|EPI_ISL_422984|2020-03	....	....	....	....	....	....
A	7116 hCoV-19/USA/WA-UW-1338/2020|EPI_ISL_422986|2020-03	....	....	....	....	....	....
A	7117 hCoV-19/USA/WA-UW-1353/2020|EPI_ISL_422987|2020-03	....	....	....	....	....	....
A	7118 hCoV-19/USA/WA-UW-1296/2020|EPI_ISL_422988|2020-03	....	....	....	....	....	....
A	7119 hCoV-19/USA/WA-UW-1297/2020|EPI_ISL_422989|2020-03	....	....	....	....	....	....
A	7120 hCoV-19/USA/WA-UW-1346/2020|EPI_ISL_422996|2020-03	....	....	....	....	....	....
A	7121 hCoV-19/USA/WA-UW-1351/2020|EPI_ISL_422997|2020-03	....	....	....	....	....	....
A	7122 hCoV-19/USA/WA-UW-1349/2020|EPI_ISL_422999|2020-03	....	....	....	....	....	....
A	7123 hCoV-19/USA/WI-UW-93/2020|EPI_ISL_425150|2020-04-0	....	....	....	....	....	....
A	7124 hCoV-19/USA/WI-UW-94/2020|EPI_ISL_425151|2020-03-3	....	....	....	....	....	....
A	7125 hCoV-19/USA/WI-UW-95/2020|EPI_ISL_425152|2020-03-3	....	....	....	....	....	....
A	7126 hCoV-19/USA/WI-UW-96/2020|EPI_ISL_425153|2020-04-0	....	....	....	....	....	....
A	7127 hCoV-19/USA/WI-UW-97/2020|EPI_ISL_425154|2020-03-3	....	....	....	....	....	....
A	7128 hCoV-19/USA/WI-UW-98/2020|EPI_ISL_425155|2020-03-3	....	....	....	....	....	....
A	7129 hCoV-19/USA/WI-UW-99/2020|EPI_ISL_425156|2020-04-0	....	....	....	....	....	....
A	7130 hCoV-19/USA/WI-UW-100/2020|EPI_ISL_425157|2020-04-	....	....	....	....	....	....
A	7131 hCoV-19/USA/WI-UW-101/2020|EPI_ISL_425158|2020-03-	....	....	....	....	....	....
A	7132 hCoV-19/USA/WI-UW-102/2020|EPI_ISL_425159|2020-03-	....	....	....	....	....	....
A	7133 hCoV-19/USA/WI-UW-103/2020|EPI_ISL_425160|2020-04-	....	....	....	....	....	....
A	7134 hCoV-19/USA/WI-UW-104/2020|EPI_ISL_425161|2020-04-	....	....	....	....	....	....
A	7135 hCoV-19/USA/WI-UW-105/2020|EPI_ISL_425162|2020-03-	....	....	....	....	....	....
A	7136 hCoV-19/USA/WI-UW-106/2020|EPI_ISL_425163|2020-03-	....	....	....	....	....	....
A	7137 hCoV-19/USA/WI-UW-107/2020|EPI_ISL_425164|2020-03-	....	....	....	....	....	....
A	7138 hCoV-19/USA/WI-UW-108/2020|EPI_ISL_425165|2020-03-	....	....	....	....	....	....
A	7139 hCoV-19/USA/WI-UW-109/2020|EPI_ISL_425166|2020-03-	....	....	....	....	....	....
A	7140 hCoV-19/USA/WI-UW-110/2020|EPI_ISL_425167|2020-03-	....	....	....	....	....	....
A	7141 hCoV-19/USA/WI-UW-111/2020|EPI_ISL_425168|2020-03-	....	....	....	....	....	....
A	7142 hCoV-19/USA/WI-UW-112/2020|EPI_ISL_425169|2020-03-	....	....	....	....	....	....
A	7143 hCoV-19/USA/CO-UW-113/2020|EPI_ISL_425170|2020-03-	....	....	....	....	....	....
A	7144 hCoV-19/USA/WI-UW-114/2020|EPI_ISL_425171|2020-03-	....	....	....	....	....	....
A	7145 hCoV-19/USA/WI-UW-115/2020|EPI_ISL_425172|2020-03-	....	....	....	....	....	....
A	7146 hCoV-19/USA/WI-UW-116/2020|EPI_ISL_425173|2020-03-	....	....	....	....	....	....
A	7147 hCoV-19/USA/WI-UW-117/2020|EPI_ISL_425174|2020-03-	....	....	....	....	....	....
A	7148 hCoV-19/USA/WI-UW-118/2020|EPI_ISL_425175|2020-03-	....	....	....	....	....	....
A	7149 hCoV-19/USA/WI-UW-119/2020|EPI_ISL_425176|2020-03	....	....	....	....	....	....
A	7150 hCoV-19/Canada/ON_ON-VIDO-01-2/2020|EPI_ISL_425177	....	.A..................	....	....	....	....
A	7151 hCoV-19/Spain/VC-FISABIO-54/2020|EPI_ISL_425178|20	....	....	....	....	....	....
A	7152 hCoV-19/Spain/VC-FISABIO-55/2020|EPI_ISL_425179|20	....	....	....	....	....	....
A	7153 hCoV-19/Spain/VC-FISABIO-56/2020|EPI_ISL_425180|20	....	....	....	....	....	....
A	7154 hCoV-19/Spain/VC-FISABIO-57/2020|EPI_ISL_425181|20	....	....	....	....	....	....
A	7155 hCoV-19/Spain/VC-FISABIO-58/2020|EPI_ISL_425182|20	....	....	....	....	....	....
A	7156 hCoV-19/Spain/VC-FISABIO-59/2020|EPI_ISL_425183|20	....	....	....	....	....	....
A	7157 hCoV-19/Spain/VC-FISABIO-60/2020|EPI_ISL_425184|20	....	....	....	....	....	....
A	7158 hCoV-19/Spain/VC-FISABIO-61/2020|EPI_ISL_425185|20	....	....	....	....	....	....
A	7159 hCoV-19/Spain/VC-FISABIO-62/2020|EPI_ISL_425186|20	....	....	....	....	....	....
A	7160 hCoV-19/Spain/VC-FISABIO-63/2020|EPI_ISL_425187|20	....	....	....	....	....	....
A	7161 hCoV-19/Spain/VC-FISABIO-64/2020|EPI_ISL_425188|20	....	....	....	....	....	....
A	7162 hCoV-19/Spain/VC-FISABIO-65/2020|EPI_ISL_425189|20	....	....	....	....	....	....
A	7163 hCoV-19/Spain/VC-FISABIO-69/2020|EPI_ISL_425193|20	....	....	....	....	....	....
A	7164 hCoV-19/Spain/VC-FISABIO-72/2020|EPI_ISL_425196|20	....	....	....	....	....	....
A	7165 hCoV-19/Spain/VC-FISABIO-73/2020|EPI_ISL_425197|20	....	....	....	....	....	....
A	7166 hCoV-19/Spain/VC-FISABIO-75/2020|EPI_ISL_425199|20	....	....	....	....	....	....
A	7167 hCoV-19/USA/AZ-TG271872/2020|EPI_ISL_426485|2020-0	....	....	....	....	....	....
A	7168 hCoV-19/Germany/BY-ChVir-929-2/2020|EPI_ISL_450198	....	....	....	....	....	....
A	7169 hCoV-19/Hangzhou/HZCDC0001/2020|EPI_ISL_407313|202	....	....	....	....	....	....
A	7170 hCoV-19/Netherlands/Gelderland_1/2020|EPI_ISL_4144	....	....	....	....	....	....
A	7171 hCoV-19/Netherlands/Limburg_2/2020|EPI_ISL_414424|	....	....	....	....	....	....
A	7172 hCoV-19/Netherlands/Limburg_3/2020|EPI_ISL_414425|	....	....	....	....	....	....
A	7173 hCoV-19/Netherlands/Limburg_4/2020|EPI_ISL_414426|	....	....	....	....	....	....
A	7174 hCoV-19/Netherlands/NoordBrabant_1/2020|EPI_ISL_41	....	....	....	....	....	....
A	7175 hCoV-19/Netherlands/NoordBrabant_3/2020|EPI_ISL_41	....	....	....	....	....	....
A	7176 hCoV-19/Netherlands/NoordBrabant_5/2020|EPI_ISL_41	....	....	....	....	....	....
A	7177 hCoV-19/Netherlands/NoordBrabant_11/2020|EPI_ISL_4	....	....	....	....	....	....
A	7178 hCoV-19/Netherlands/NoordHolland_1/2020|EPI_ISL_41	....	....	....	....	....	....
A	7179 hCoV-19/Netherlands/Overijssel_1/2020|EPI_ISL_4144	....	....	....	....	....	....
A	7180 hCoV-19/Netherlands/Utrecht_1/2020|EPI_ISL_414435|	....	....	....	....	....	....
A	7181 hCoV-19/Netherlands/Utrecht_2/2020|EPI_ISL_414436|	....	....	....	....	....	....
A	7182 hCoV-19/Netherlands/Utrecht_3/2020|EPI_ISL_414437|	....	....	....	....	....	....
A	7183 hCoV-19/Netherlands/Utrecht_4/2020|EPI_ISL_414438|	....	....	....	....	....	....
A	7184 hCoV-19/Netherlands/Utrecht_5/2020|EPI_ISL_414439|	....	....	....	....	....	....
A	7185 hCoV-19/Netherlands/Utrecht_7/2020|EPI_ISL_414440|	....	....	....	....	....	....

A	7186	hCoV-19/Netherlands/Utrecht_8/2020|EPI_ISL_414441|	····················	····················	·····················	····················	····················	····················
A	7187	hCoV-19/Netherlands/Utrecht_10/2020|EPI_ISL_414442	····················	····················	·····················	····················	····················	····················
A	7188	hCoV-19/Netherlands/Utrecht_11/2020|EPI_ISL_414443	····················	····················	·····················	····················	····················	····················
A	7189	hCoV-19/Netherlands/ZuidHolland_1/2020|EPI_ISL_414	····················	····················	·····················	····················	····················	····················
A	7190	hCoV-19/Netherlands/ZuidHolland_9/2020|EPI_ISL_414	····················	····················	·····················	····················	····················	····················
A	7191	hCoV-19/Netherlands/ZuidHolland_10/2020|EPI_ISL_41	····················	····················	·····················	····················	····················	····················
A	7192	hCoV-19/Netherlands/Limburg_5/2020|EPI_ISL_414448|	····················	····················	·····················	····················	····················	····················
A	7193	hCoV-19/Netherlands/NoordBrabant_2/2020|EPI_ISL_41	····················	····················	·····················	····················	····················	····················
A	7194	hCoV-19/Netherlands/NoordBrabant_6/2020|EPI_ISL_41	····················	····················	·····················	····················	····················	····················
A	7195	hCoV-19/Netherlands/NoordBrabant_12/2020|EPI_ISL_4	····················	····················	·····················	····················	····················	····················
A	7196	hCoV-19/Netherlands/NoordBrabant_14/2020|EPI_ISL_4	····················	····················	·····················	····················	····················	····················
A	7197	hCoV-19/Netherlands/NoordBrabant_16/2020|EPI_ISL_4	····················	····················	·····················	····················	····················	····················
A	7198	hCoV-19/Netherlands/NoordBrabant_17/2020|EPI_ISL_4	···T················	····················	·····················	····················	····················	····················
A	7199	hCoV-19/Netherlands/NoordBrabant_18/2020|EPI_ISL_4	····················	····················	·····················	····················	····················	····················
A	7200	hCoV-19/Netherlands/Overijssel_2/2020|EPI_ISL_4144	····················	····················	·····················	····················	····················	····················
A	7201	hCoV-19/Netherlands/Utrecht_6/2020|EPI_ISL_414461|	····················	····················	·····················	····················	····················	····················
A	7202	hCoV-19/Netherlands/Utrecht_12/2020|EPI_ISL_414462	····················	····················	·····················	····················	····················	····················
A	7203	hCoV-19/Netherlands/Utrecht_13/2020|EPI_ISL_414463	····················	····················	·····················	····················	····················	····················
A	7204	hCoV-19/Netherlands/Utrecht_14/2020|EPI_ISL_414464	····················	····················	·····················	····················	····················	····················
A	7205	hCoV-19/Netherlands/ZuidHolland_7/2020|EPI_ISL_414	····················	····················	·····················	····················	····················	····················
A	7206	hCoV-19/Netherlands/ZuidHolland_8/2020|EPI_ISL_414	····················	····················	·····················	····················	····················	····················
A	7207	hCoV-19/Netherlands/ZuidHolland_11/2020|EPI_ISL_41	····················	····················	·····················	····················	····················	····················
A	7208	hCoV-19/Netherlands/ZuidHolland_13/2020|EPI_ISL_41	····················	····················	·····················	····················	····················	····················
A	7209	hCoV-19/Netherlands/ZuidHolland_14/2020|EPI_ISL_41	····················	····················	·····················	····················	····················	····················
A	7210	hCoV-19/USA/NY1-PV08001/2020|EPI_ISL_414476|2020-0	····················	····················	·····················	····················	····················	····················
A	7211	hCoV-19/Czech Republic/951/2020|EPI_ISL_414477|202	····················	····················	·····················	····················	····················	····················
A	7212	hCoV-19/USA/CruiseA-19/2020|EPI_ISL_414479|2020-02	····················	····················	·····················	····················	····················	····················
A	7213	hCoV-19/USA/CruiseA-21/2020|EPI_ISL_414480|2020-02	····················	····················	·····················	····················	····················	····················
A	7214	hCoV-19/USA/CruiseA-22/2020|EPI_ISL_414481|2020-02	····················	····················	·····················	····················	····················	····················
A	7215	hCoV-19/USA/CruiseA-23/2020|EPI_ISL_414482|2020-02	····················	····················	·····················	····················	····················	····················
A	7216	hCoV-19/USA/CruiseA-24/2020|EPI_ISL_414483|2020-02	····················	····················	·····················	····················	····················	····················
A	7217	hCoV-19/USA/CruiseA-25/2020|EPI_ISL_414484|2020-02	····················	····················	·····················	····················	····················	····················
A	7218	hCoV-19/USA/CruiseA-26/2020|EPI_ISL_414485|2020-02	····················	····················	·····················	····················	····················	····················
A	7219	hCoV-19/Ireland/CO-NVRL-20134/2020|EPI_ISL_414487|	····················	····················	·····················	····················	····················	····················
A	7222	hCoV-19/Germany/NW-HHU-02-1/2020|EPI_ISL_414497|20	····················	····················	·····················	····················	····················	····················
A	7223	hCoV-19/Germany/NW-HHU-03/2020|EPI_ISL_414498|2020	····················	····················	·····················	····················	····················	····················
A	7224	hCoV-19/Germany/NW-HHU-04/2020|EPI_ISL_414499|2020	····················	····················	·····················	····················	····················	····················
A	7225	hCoV-19/Peru/LIM-010/2020|EPI_ISL_415787|2020-03-1	····················	····················	·····················	····················	····················	····················
A	7226	hCoV-19/England/SHEF-C0190/2020|EPI_ISL_420257|202	····················	····················	·····················	····················	····················	····················
A	7228	hCoV-19/England/SHEF-C0181/2020|EPI_ISL_420260|202	····················	····················	·····················	····················	····················	····················
A	7229	hCoV-19/England/SHEF-C0725/2020|EPI_ISL_420261|202	····················	····················	·····················	····················	····················	····················
A	7230	hCoV-19/England/SHEF-C0752/2020|EPI_ISL_420262|202	····················	····················	·····················	····················	····················	····················
A	7231	hCoV-19/England/SHEF-C04E2/2020|EPI_ISL_420263|202	····················	····················	·····················	····················	····················	····················
A	7232	hCoV-19/England/SHEF-C06FB/2020|EPI_ISL_420264|202	····················	····················	·····················	····················	····················	····················
A	7233	hCoV-19/England/SHEF-C078F/2020|EPI_ISL_420265|202	····················	····················	·····················	····················	····················	····················
A	7234	hCoV-19/England/SHEF-C0057/2020|EPI_ISL_420266|202	····················	····················	·····················	····················	····················	····················
A	7235	hCoV-19/England/SHEF-C00DF/2020|EPI_ISL_420267|202	····················	····················	·····················	····················	····················	····················
A	7236	hCoV-19/England/SHEF-C0093/2020|EPI_ISL_420268|202	····················	····················	·····················	····················	····················	····················
A	7237	hCoV-19/England/SHEF-C04F1/2020|EPI_ISL_420269|202	····················	····················	·····················	····················	····················	····················
A	7238	hCoV-19/England/SHEF-C0707/2020|EPI_ISL_420270|202	····················	····················	·····················	····················	····················	····················
A	7239	hCoV-19/England/SHEF-C0075/2020|EPI_ISL_420271|202	····················	····················	·····················	····················	····················	····················
A	7240	hCoV-19/England/SHEF-C002A/2020|EPI_ISL_420272|202	····················	····················	·····················	····················	····················	····················
A	7241	hCoV-19/England/SHEF-C000C/2020|EPI_ISL_420273|202	····················	····················	·····················	····················	····················	····················
A	7242	hCoV-19/England/SHEF-C00EE/2020|EPI_ISL_420274|202	····················	····················	·····················	····················	····················	····················
A	7243	hCoV-19/England/SHEF-C0039/2020|EPI_ISL_420275|202	····················	····················	·····················	····················	····················	····················
A	7244	hCoV-19/England/SHEF-C00B1/2020|EPI_ISL_420276|202	····················	····················	·····················	····················	····················	····················
A	7245	hCoV-19/England/SHEF-C0066/2020|EPI_ISL_420277|202	····················	····················	·····················	····················	····················	····················
A	7246	hCoV-19/England/SHEF-C0646/2020|EPI_ISL_420278|202	····················	····················	·····················	····················	····················	····················
A	7247	hCoV-19/USA/NY-NYUMC89/2020|EPI_ISL_421590|2020-03	····················	····················	·····················	····················	····················	····················
A	7248	hCoV-19/USA/NY-NYUMC90/2020|EPI_ISL_421591|2020-03	····················	····················	·····················	····················	····················	····················
A	7249	hCoV-19/USA/NY-NYUMC91/2020|EPI_ISL_421592|2020-03	····················	····················	·····················	····················	····················	····················
A	7250	hCoV-19/USA/NY-PV09006/2020|EPI_ISL_421593|2020-03	····················	····················	·····················	····················	····················	····················
A	7251	hCoV-19/USA/NY-PV09019/2020|EPI_ISL_421594|2020-03	····················	····················	·····················	····················	····················	····················
A	7252	hCoV-19/USA/NY-PV09020/2020|EPI_ISL_421595|2020-03	····················	····················	·····················	····················	····················	····················
A	7253	hCoV-19/USA/NY-PV09021/2020|EPI_ISL_421596|2020-03	····················	····················	·····················	····················	····················	····················
A	7254	hCoV-19/USA/NY-PV09023/2020|EPI_ISL_421597|2020-03	····················	····················	·····················	····················	····················	····················
A	7255	hCoV-19/USA/NY-PV09025/2020|EPI_ISL_421598|2020-03	····················	····················	·····················	····················	····················	····················
A	7256	hCoV-19/USA/NY-PV09028/2020|EPI_ISL_421599|2020-03	····················	····················	·····················	····················	····················	····················
A	7257	hCoV-19/USA/NY-PV09029/2020|EPI_ISL_421600|2020-03	····················	····················	·····················	····················	····················	····················
A	7258	hCoV-19/USA/NY-PV09032/2020|EPI_ISL_421602|2020-03	····················	····················	·····················	····················	····················	····················
A	7259	hCoV-19/USA/NY-PV09037/2020|EPI_ISL_421603|2020-03	····················	····················	·····················	····················	····················	····················
A	7260	hCoV-19/USA/NY-PV09039/2020|EPI_ISL_421604|2020-03	····················	····················	·····················	····················	····················	····················
A	7261	hCoV-19/USA/NY-PV09041/2020|EPI_ISL_421605|2020-03	····················	····················	·····················	····················	····················	····················
A	7262	hCoV-19/USA/NY-PV09043/2020|EPI_ISL_421606|2020-03	····················	····················	·····················	····················	····················	····················
A	7263	hCoV-19/USA/NY-PV09046/2020|EPI_ISL_421608|2020-03	····················	····················	·····················	····················	····················	····················
A	7264	hCoV-19/Netherlands/ZuidHolland_62/2020|EPI_ISL_42	····················	····················	·····················	····················	····················	····················
A	7265	hCoV-19/Netherlands/ZuidHolland_64/2020|EPI_ISL_42	····················	····················	·····················	····················	····················	····················
A	7266	hCoV-19/Netherlands/ZuidHolland_66/2020|EPI_ISL_42	····················	····················	·····················	····················	····················	····················

A	7267 hCoV-19/Netherlands/ZuidHolland_67/2020|EPI_ISL_42	····················	····················	····················	····················	····················	····················
A	7268 hCoV-19/Netherlands/ZuidHolland_71/2020|EPI_ISL_42	····················	····················	····················	····················	····················	····················
A	7269 hCoV-19/South Korea/KCDC2001/2020|EPI_ISL_425117|2	····················	····················	····················	····················	····················	····················
A	7270 hCoV-19/South Korea/KCDC2002/2020|EPI_ISL_425118|2	····················	····················	····················	····················	····················	····················
A	7271 hCoV-19/Germany/NW-HHU-37/2020|EPI_ISL_425121|2020	····················	····················	····················	····················	····················	····················
A	7272 hCoV-19/Germany/NW-HHU-38/2020|EPI_ISL_425122|2020	····················	····················	····················	····················	····················	····················
A	7273 hCoV-19/Germany/NW-HHU-39/2020|EPI_ISL_425123|2020	····················	····················	····················	····················	····················	G···················
A	7274 hCoV-19/Germany/NW-HHU-40/2020|EPI_ISL_425124|2020	····················	····················	····················	····················	····················	····················
A	7275 hCoV-19/Germany/NW-HHU-41/2020|EPI_ISL_425125|2020	····················	····················	····················	····················	····················	····················
A	7276 hCoV-19/Germany/NW-HHU-42/2020|EPI_ISL_425126|2020	····················	····················	····················	····················	····················	····················
A	7277 hCoV-19/Germany/NW-HHU-42-2/2020|EPI_ISL_425127|20	····················	····················	····················	····················	····················	····················
A	7278 hCoV-19/Germany/NW-HHU-42-3/2020|EPI_ISL_425128|20	····················	····················	····················	····················	··············N·····	····················
A	7279 hCoV-19/Germany/NW-HHU-46/2020|EPI_ISL_425130|2020	····················	····················	····················	····················	····················	····················
A	7280 hCoV-19/Germany/NW-HHU-47/2020|EPI_ISL_425131|2020	····················	····················	····················	····················	····················	····················
A	7281 hCoV-19/Germany/NW-HHU-48/2020|EPI_ISL_425132|2020	····················	····················	····················	····················	····················	····················
A	7282 hCoV-19/Germany/NW-HHU-43/2020|EPI_ISL_425138|2020	····················	····················	····················	····················	····················	····················
A	7283 hCoV-19/Germany/NW-HHU-44/2020|EPI_ISL_425139|2020	····················	····················	····················	····················	····················	····················
A	7284 hCoV-19/USA/WI-UW-85/2020|EPI_ISL_425142|2020-04-0	····················	····················	····················	····················	····················	····················
A	7285 hCoV-19/USA/WI-UW-86/2020|EPI_ISL_425143|2020-04-0	····················	····················	····················	····················	····················	····················
A	7286 hCoV-19/USA/WI-UW-87/2020|EPI_ISL_425144|2020-04-0	····················	····················	····················	····················	····················	····················
A	7287 hCoV-19/USA/WI-UW-88/2020|EPI_ISL_425145|2020-04-0	····················	····················	····················	····················	····················	····················
A	7288 hCoV-19/USA/WI-UW-89/2020|EPI_ISL_425146|2020-03-3	····················	····················	····················	····················	····················	····················
A	7289 hCoV-19/USA/WI-UW-90/2020|EPI_ISL_425147|2020-04-0	····················	····················	····················	····················	····················	····················
A	7290 hCoV-19/USA/WI-UW-91/2020|EPI_ISL_425148|2020-04-0	····················	····················	····················	····················	····················	····················
A	7291 hCoV-19/USA/WI-UW-92/2020|EPI_ISL_425149|2020-03-3	····················	····················	····················	····················	····················	····················
A	7292 hCoV-19/India/GJ-GBRC1/2020|EPI_ISL_426414|2020-04	····················	····················	····················	····················	····················	····················
A	7293 hCoV-19/India/GJ-GBRC1s/2020|EPI_ISL_426415|2020-0	····················	····················	····················	····················	····················	····················
A	7294 hCoV-19/USA/CT_9849/2020|EPI_ISL_426416|2020-03-08	····················	····················	····················	····················	····················	····················
A	7295 hCoV-19/USA/GA_2070/2020|EPI_ISL_426417|2020-03-07	····················	····················	····················	····················	····················	····················
A	7296 hCoV-19/USA/GA_2147/2020|EPI_ISL_426418|2020-03	····················	····················	····················	····················	····················	····················
A	7297 hCoV-19/USA/GA_2275/2020|EPI_ISL_426419|2020-03	····················	····················	····················	····················	····················	····················
A	7298 hCoV-19/USA/HI_4970/2020|EPI_ISL_426420|2020-03-05	····················	·A··················	····················	····················	····················	····················
A	7299 hCoV-19/USA/HI_7881/2020|EPI_ISL_426421|2020-03-07	····················	····················	····················	····················	····················	····················
A	7300 hCoV-19/USA/MD_0027/2020|EPI_ISL_426425|2020-03-04	····················	····················	····················	····················	····················	····················
A	7301 hCoV-19/USA/MN_0100/2020|EPI_ISL_426426|2020-03-05	····················	····················	····················	····················	····················	····················
A	7302 hCoV-19/USA/MN_0101/2020|EPI_ISL_426427|2020-03-07	····················	····················	····················	····················	····················	····················
A	7303 hCoV-19/USA/NC_0025/2020|EPI_ISL_426428|2020-03-08	····················	····················	····················	····················	····················	····················
A	7304 hCoV-19/USA/NV_0016/2020|EPI_ISL_426429|2020-03-07	····················	····················	····················	····················	····················	····················
A	7305 hCoV-19/USA/OH_0019/2020|EPI_ISL_426430|2020-03-08	····················	····················	····················	····················	····················	····················
A	7306 hCoV-19/USA/OH_0020/2020|EPI_ISL_426431|2020-03-07	····················	····················	····················	····················	····················	····················
A	7307 hCoV-19/USA/PA_2734/2020|EPI_ISL_426432|2020-03-07	····················	····················	····················	····················	····················	····················
A	7308 hCoV-19/USA/PA_2735/2020|EPI_ISL_426433|2020-03-07	····················	····················	····················	····················	····················	····················
A	7309 hCoV-19/USA/PA_2887/2020|EPI_ISL_426434|2020-03-08	····················	····················	····················	····················	····················	····················
A	7310 hCoV-19/USA/RI_0882/2020|EPI_ISL_426435|2020-03-09	····················	····················	····················	····················	····················	····················
A	7311 hCoV-19/USA/WA_0418/2020|EPI_ISL_426436|2020-03-19	····················	····················	····················	····················	····················	····················
A	7312 hCoV-19/USA/WA_0422/2020|EPI_ISL_426437|2020-03-19	····················	····················	····················	····················	····················	····················
A	7313 hCoV-19/USA/WA_0427/2020|EPI_ISL_426438|2020-03-19	····················	····················	····················	····················	····················	····················
A	7314 hCoV-19/USA/WA_0428/2020|EPI_ISL_426439|2020-03-19	····················	····················	····················	····················	····················	····················
A	7315 hCoV-19/USA/WA_0429/2020|EPI_ISL_426440|2020-03-19	····················	····················	····················	····················	····················	····················
A	7316 hCoV-19/USA/WA_0432/2020|EPI_ISL_426441|2020-03-19	····················	····················	····················	····················	····················	····················
A	7317 hCoV-19/USA/WA_0434/2020|EPI_ISL_426442|2020-03-19	····················	····················	····················	····················	····················	····················
A	7318 hCoV-19/USA/WA_0440/2020|EPI_ISL_426443|2020-03-19	····················	····················	····················	····················	····················	····················
A	7319 hCoV-19/USA/WA_0441/2020|EPI_ISL_426444|2020-03-19	····················	····················	····················	····················	····················	····················
A	7320 hCoV-19/USA/WA_0442/2020|EPI_ISL_426445|2020-03-19	····················	····················	····················	····················	····················	····················
A	7321 hCoV-19/USA/WA_0447/2020|EPI_ISL_426446|2020-03-19	····················	····················	····················	····················	····················	····················
A	7322 hCoV-19/USA/WA_0448/2020|EPI_ISL_426447|2020-03-19	····················	····················	····················	····················	····················	····················
A	7323 hCoV-19/USA/WA_0457/2020|EPI_ISL_426448|2020-03-20	····················	····················	····················	····················	····················	····················
A	7324 hCoV-19/USA/WA_0478/2020|EPI_ISL_426449|2020-03-19	····················	····················	····················	····················	····················	····················
A	7325 hCoV-19/USA/WA_0480/2020|EPI_ISL_426450|2020-03-19	····················	····················	····················	····················	····················	····················
A	7326 hCoV-19/USA/WA_0484/2020|EPI_ISL_426451|2020-03-19	····················	····················	····················	····················	····················	····················
A	7327 hCoV-19/USA/WA_0711/2020|EPI_ISL_426452|2020-03-19	····················	····················	····················	····················	····················	····················
A	7328 hCoV-19/USA/WA_0797/2020|EPI_ISL_426453|2020-03-20	····················	····················	····················	····················	····················	····················
A	7329 hCoV-19/USA/VA-DCLS-0041/2020|EPI_ISL_426454|2020-	····················	····················	····················	····················	····················	····················
A	7330 hCoV-19/USA/VA-DCLS-0047/2020|EPI_ISL_426455|2020-	····················	····················	····················	····················	····················	····················
A	7331 hCoV-19/USA/VA-DCLS-0048/2020|EPI_ISL_426456|2020-	····················	····················	····················	····················	····················	····················
A	7332 hCoV-19/USA/VA-DCLS-0049/2020|EPI_ISL_426457|2020-	····················	····················	····················	····················	····················	····················
A	7333 hCoV-19/USA/VA-DCLS-0050/2020|EPI_ISL_426458|2020-	····················	····················	····················	····················	····················	····················
A	7334 hCoV-19/USA/VA-DCLS-0051/2020|EPI_ISL_426459|2020-	····················	····················	····················	····················	····················	····················
A	7335 hCoV-19/USA/VA-DCLS-0052/2020|EPI_ISL_426460|2020-	····················	····················	····················	····················	····················	····················
A	7336 hCoV-19/USA/VA-DCLS-0053/2020|EPI_ISL_426461|2020-	····················	····················	····················	····················	····················	····················
A	7337 hCoV-19/USA/VA-DCLS-0054/2020|EPI_ISL_426462|2020-	····················	····················	····················	····················	····················	····················
A	7338 hCoV-19/USA/VA-DCLS-0055/2020|EPI_ISL_426463|2020-	····················	····················	····················	····················	····················	····················
A	7339 hCoV-19/USA/VA-DCLS-0056/2020|EPI_ISL_426464|2020-	····················	····················	····················	··············A·····	····················	····················
A	7340 hCoV-19/USA/VA-DCLS-0057/2020|EPI_ISL_426465|2020-	····················	····················	····················	····················	····················	····················
A	7341 hCoV-19/USA/VA-DCLS-0058/2020|EPI_ISL_426466|2020-	····················	····················	····················	····················	····················	····················
A	7342 hCoV-19/USA/VA-DCLS-0059/2020|EPI_ISL_426467|2020-	····················	····················	····················	····················	····················	····················
A	7343 hCoV-19/USA/VA-DCLS-0060/2020|EPI_ISL_426468|2020-	····················	····················	····················	····················	····················	····················
A	7344 hCoV-19/USA/VA-DCLS-0061/2020|EPI_ISL_426469|2020-	····················	····················	····················	····················	····················	····················

A	7345 hCoV-19/USA/VA-DCLS-0063/2020|EPI_ISL_426470|2020-	····················	····················	····················	····················	····················	····················	
A	7346 hCoV-19/USA/VA-DCLS-0064/2020|EPI_ISL_426471|2020-	····················	····················	····················	····················	····················	····················	
A	7347 hCoV-19/USA/VA-DCLS-0065/2020|EPI_ISL_426472|2020-	····················	····················	····················	····················	····················	····················	
A	7348 hCoV-19/USA/VA-DCLS-0066/2020|EPI_ISL_426473|2020-	····················	····················	····················	····················	····················	····················	
A	7349 hCoV-19/USA/VA-DCLS-0067/2020|EPI_ISL_426474|2020-	····················	····················	····················	····················	····················	····················	
A	7350 hCoV-19/USA/VA-DCLS-0068/2020|EPI_ISL_426475|2020-	····················	····················	····················	····················	····················	····················	
A	7351 hCoV-19/Australia/NSW213/2020|EPI_ISL_427726|2020-	····················	····················	····················	····················	····················	····················	
A	7352 hCoV-19/Australia/NSW214/2020|EPI_ISL_427727|2020-	····················	····················	····················	····················	····················	····················	
A	7353 hCoV-19/Australia/NSW113/2020|EPI_ISL_427728|2020-	····················	····················	····················	····················	····················	····················	
A	7354 hCoV-19/Australia/NSW77/2020|EPI_ISL_427729|2020-0	····················	····················	····················	····················	····················	····················	
A	7355 hCoV-19/Australia/NSW215/2020|EPI_ISL_427730|2020-	····················	····················	····················	····················	····················	····················	
A	7356 hCoV-19/Australia/NSW165/2020|EPI_ISL_427731|2020-	····················	····················	····················	····················	····················	····················	
A	7357 hCoV-19/Australia/NSW216/2020|EPI_ISL_427732|2020-	····················	····················	····················	····················	····················	····················	
A	7358 hCoV-19/Australia/NSW67/2020|EPI_ISL_427733|2020-0	····················	····················	····················	····················	····················	····················	
A	7359 hCoV-19/Australia/NSW176/2020|EPI_ISL_427734|2020-	····················	····················	····················	····················	····················	····················	
A	7360 hCoV-19/Australia/NSW158/2020|EPI_ISL_427735|2020-	····················	····················	····················	····················	····················	····················	
A	7361 hCoV-19/Australia/NSW190/2020|EPI_ISL_427736|2020-	····················	····················	····················	····················	····················	····················	
A	7362 hCoV-19/Australia/NSW143/2020|EPI_ISL_427737|2020-	····················	····················	····················	····················	····················	····················	
A	7363 hCoV-19/Australia/NSW144/2020|EPI_ISL_427738|2020-	····················	····················	····················	····················	····················	····················	
A	7364 hCoV-19/Australia/NSW168/2020|EPI_ISL_427739|2020-	····················	····················	····················	····················	····················	····················	
A	7365 hCoV-19/Australia/NSW189/2020|EPI_ISL_427740|2020-	····················	····················	····················	····················	····················	····················	
A	7366 hCoV-19/Australia/NSW110/2020|EPI_ISL_427741|2020-	····················	····················	····················	····················	····················	····················	
A	7367 hCoV-19/Australia/NSW134/2020|EPI_ISL_427742|2020-	····················	····················	····················	····················	····················	····················	
A	7368 hCoV-19/Australia/NSW171/2020|EPI_ISL_427743|2020-	····················	····················	····················	····················	····················	····················	
A	7369 hCoV-19/Australia/NSW72/2020|EPI_ISL_427744|2020-0	····················	····················	····················	····················	····················	····················	
A	7370 hCoV-19/Australia/NSW205/2020|EPI_ISL_427745|2020-	····················	····················	····················	····················	····················	····················	
A	7371 hCoV-19/Australia/NSW197/2020|EPI_ISL_427746|2020-	····················	····················	····················	····················	····················	····················	
A	7372 hCoV-19/Australia/NSW199/2020|EPI_ISL_427747|2020-	····················	····················	····················	····················	····················	····················	
A	7373 hCoV-19/Australia/NSW106/2020|EPI_ISL_427748|2020-	····················	····················	····················	····················	····················	····················	
A	7374 hCoV-19/Australia/NSW142/2020|EPI_ISL_427749|2020-	····················	····················	····················	····················	····················	····················	
A	7375 hCoV-19/Australia/NSW73/2020|EPI_ISL_427750|2020-0	····················	····················	····················	····················	····················	····················	
A	7376 hCoV-19/Australia/NSW68/2020|EPI_ISL_427751|2020-0	····················	····················	····················	····················	····················	····················	
A	7377 hCoV-19/Australia/NSW184/2020|EPI_ISL_427752|2020-	····················	····················	····················	····················	····················	····················	
A	7378 hCoV-19/Australia/NSW208/2020|EPI_ISL_427753|2020-	····················	····················	····················	····················	····················	····················	
A	7379 hCoV-19/Australia/NSW109/2020|EPI_ISL_427754|2020-	····················	····················	····················	····················	····················	····················	
A	7380 hCoV-19/Australia/NSW209/2020|EPI_ISL_427755|2020-	····················	····················	····················	····················	····················	····················	
A	7381 hCoV-19/Australia/NSW117/2020|EPI_ISL_427756|2020-	····················	····················	····················	····················	····················	····················	
A	7382 hCoV-19/Australia/NSW70/2020|EPI_ISL_427757|2020-0	····················	····················	····················	····················	····················	····················	
A	7383 hCoV-19/Australia/NSW162/2020|EPI_ISL_427758|2020-	····················	····················	····················	····················	····················	····················	
A	7384 hCoV-19/Australia/NSW71/2020|EPI_ISL_427759|2020-0	····················	····················	····················	····················	····················	····················	
A	7385 hCoV-19/Australia/NSW129/2020|EPI_ISL_427760|2020-	····················	····················	····················	····················	····················	····················	
A	7386 hCoV-19/Australia/NSW138/2020|EPI_ISL_427761|2020-	····················	····················	····················	····················	····················	····················	
A	7387 hCoV-19/Australia/NSW202/2020|EPI_ISL_427762|2020-	····················	····················	····················	····················	········T···········	····················	
A	7388 hCoV-19/Australia/NSW194/2020|EPI_ISL_427763|2020-	····················	····················	····················	····················	····················	····················	
A	7389 hCoV-19/Australia/NSW147/2020|EPI_ISL_427764|2020-	····················	····················	····················	····················	····················	····················	
A	7390 hCoV-19/Australia/NSW203/2020|EPI_ISL_427765|2020-	····················	····················	····················	····················	····················	····················	
A	7391 hCoV-19/Australia/NSW210/2020|EPI_ISL_427766|2020-	····················	····················	····················	····················	····················	····················	
A	7392 hCoV-19/Australia/NSW195/2020|EPI_ISL_427767|2020-	····················	····················	····················	····················	····················	····················	
A	7393 hCoV-19/Australia/NSW130/2020|EPI_ISL_427768|2020-	····················	····················	····················	····················	····················	····················	
A	7394 hCoV-19/Australia/NSW97/2020|EPI_ISL_427769|2020-0	····················	····················	····················	····················	····················	····················	
A	7395 hCoV-19/Australia/NSW78/2020|EPI_ISL_427770|2020-0	····················	····················	····················	····················	····················	····················	
A	7396 hCoV-19/Australia/NSW166/2020|EPI_ISL_427771|2020-	····················	····················	····················	····················	····················	····················	
A	7397 hCoV-19/Australia/NSW107/2020|EPI_ISL_427772|2020-	····················	····················	····················	····················	····················	····················	
A	7398 hCoV-19/Australia/NSW64/2020|EPI_ISL_427773|2020-0	····················	····················	····················	····················	····················	····················	
A	7399 hCoV-19/Australia/NSW98/2020|EPI_ISL_427774|2020-0	····················	····················	····················	····················	····················	····················	
A	7400 hCoV-19/Australia/NSW149/2020|EPI_ISL_427775|2020-	····················	····················	····················	····················	····················	····················	
A	7401 hCoV-19/Australia/NSW89/2020|EPI_ISL_427776|2020-0	····················	····················	····················	····················	····················	····················	
A	7402 hCoV-19/Australia/NSW99/2020|EPI_ISL_427777|2020-0	····················	····················	····················	····················	····················	····················	
A	7403 hCoV-19/Australia/NSW122/2020|EPI_ISL_427778|2020-	····················	····················	····················	····················	····················	····················	
A	7404 hCoV-19/Australia/NSW169/2020|EPI_ISL_427779|2020-	····················	····················	····················	····················	····················	····················	
A	7405 hCoV-19/Australia/NSW123/2020|EPI_ISL_427780|2020-	····················	····················	····················	····················	····················	····················	
A	7406 hCoV-19/Australia/NSW159/2020|EPI_ISL_427781|2020-	··················T·	····················	····················	····················	····················	····················	1
A	7407 hCoV-19/Australia/NSW124/2020|EPI_ISL_427782|2020-	····················	····················	····················	····················	····················	····················	
A	7408 hCoV-19/Australia/NSW131/2020|EPI_ISL_427783|2020-	····················	····················	····················	····················	····················	····················	
A	7409 hCoV-19/Australia/NSW160/2020|EPI_ISL_427784|2020-	····················	····················	····················	····················	····················	····················	
A	7410 hCoV-19/Australia/NSW125/2020|EPI_ISL_427785|2020-	····················	····················	····················	····················	····················	····················	
A	7411 hCoV-19/Australia/NSW191/2020|EPI_ISL_427786|2020-	····················	····················	····················	····················	····················	····················	
A	7412 hCoV-19/Australia/NSW132/2020|EPI_ISL_427787|2020-	····················	····················	····················	····················	····················	····················	
A	7413 hCoV-19/Australia/NSW161/2020|EPI_ISL_427788|2020-	····················	····················	····················	····················	····················	····················	
A	7414 hCoV-19/Australia/NSW163/2020|EPI_ISL_427789|2020-	····················	····················	····················	····················	····················	····················	
A	7415 hCoV-19/Australia/NSW79/2020|EPI_ISL_427790|2020-0	····················	····················	····················	····················	····················	····················	
A	7416 hCoV-19/Australia/NSW133/2020|EPI_ISL_427791|2020-	····················	····················	····················	····················	····················	····················	
A	7417 hCoV-19/Australia/NSW204/2020|EPI_ISL_427792|2020-	····················	····················	····················	····················	····················	····················	
A	7418 hCoV-19/Australia/NSW185/2020|EPI_ISL_427793|2020-	····················	····················	····················	····················	····················	····················	
A	7419 hCoV-19/Australia/NSW126/2020|EPI_ISL_427794|2020-	····················	····················	····················	····················	····················	····················	
A	7420 hCoV-19/Australia/NSW139/2020|EPI_ISL_427795|2020-	····················	····················	····················	····················	····················	····················	
A	7421 hCoV-19/Australia/NSW150/2020|EPI_ISL_427796|2020-	····················	····················	····················	····················	····················	····················	
A	7422 hCoV-19/Australia/NSW192/2020|EPI_ISL_427797|2020-	····················	····················	····················	····················	····················	····················	

A	7423 hCoV-19/Australia/NSW193/2020|EPI_ISL_427798|2020-	····················	····················	····················	····················	····················	····················	
A	7424 hCoV-19/Australia/NSW140/2020|EPI_ISL_427799|2020-	····················	····················	····················	····················	····················	····················	
A	7425 hCoV-19/Australia/QLD09/2020|EPI_ISL_414414|2020-0	····················	····················	····················	····················	····················	····················	
A	7426 hCoV-19/Switzerland/GE-HUG-1402/2020|EPI_ISL_41570	····················	····················	····················	····················	····················	····················	
A	7427 hCoV-19/Switzerland/BS-HUG-0914/2020|EPI_ISL_41570	····················	····················	····················	····················	····················	····················	
A	7428 hCoV-19/Switzerland/SZ-HUG-1417/2020|EPI_ISL_41570	····················	····················	····················	····················	····················	····················	
A	7429 hCoV-19/Switzerland/TI-HUG-2045/2020|EPI_ISL_41570	····················	····················	····················	····················	····················	····················	
A	7430 hCoV-19/Switzerland/BE-HUG-2536/2020|EPI_ISL_41570	····················	····················	····················	····················	····················	····················	
A	7431 hCoV-19/Switzerland/GE-HUG-4135/2020|EPI_ISL_41570	····················	····················	····················	····················	····················	····················	
A	7432 hCoV-19/Switzerland/GE-HUG-06207/2020|EPI_ISL_4157	····················	····················	····················	····················	····················	····················	
A	7433 hCoV-19/Switzerland/GE-HUG-6679/2020|EPI_ISL_41570	····················	····················	····················	····················	····················	····················	
A	7434 hCoV-19/Switzerland/GE-HUG-4984/2020|EPI_ISL_41570	····················	···················A	····················	····················	····················	····················	1
A	7435 hCoV-19/Russia/StPetersburg-3524/2020|EPI_ISL_4157	····················	····················	····················	····················	····················	····················	
A	7436 hCoV-19/Taiwan/CGMH-CGU-03/2020|EPI_ISL_415741|202	····················	····················	····················	····················	····················	····················	
A	7437 hCoV-19/Taiwan/CGMH-CGU-04/2020|EPI_ISL_415742|202	····················	····················	····················	····················	····················	····················	
A	7438 hCoV-19/Taiwan/CGMH-CGU-05/2020|EPI_ISL_415743|202	····················	····················	····················	····················	····················	····················	
A	7439 hCoV-19/Wales/PHWC-23CD6/2020|EPI_ISL_418103|2020-	····················	····A···············	····················	····················	····················	····················	
A	7440 hCoV-19/Wales/PHWC-23D6A/2020|EPI_ISL_418110|2020-	····················	····A···············	····················	····················	····················	····················	
A	7441 hCoV-19/Wales/PHWC-23E2B/2020|EPI_ISL_418117|2020-	····················	····················	····················	····················	····················	····················	
A	7442 hCoV-19/Wales/PHWC-23E67/2020|EPI_ISL_418120|2020-	····················	····A···············	····················	····················	····················	····················	
A	7443 hCoV-19/Wales/PHWC-23E85/2020|EPI_ISL_418122|2020-	····················	····················	····················	····················	····················	····················	
A	7444 hCoV-19/Wales/PHWC-23FFB/2020|EPI_ISL_418136|2020-	····················	····A···············	····················	····················	····················	····················	
A	7445 hCoV-19/Wales/PHWC-24006/2020|EPI_ISL_418137|2020-	····················	····················	····················	····················	····················	····················	
A	7446 hCoV-19/Wales/PHWC-24024/2020|EPI_ISL_418139|2020-	····················	····················	····················	····················	····················	····················	
A	7447 hCoV-19/Wales/PHWC-24033/2020|EPI_ISL_418140|2020-	····················	····················	····················	····················	····················	····················	
A	7448 hCoV-19/Wales/PHWC-240D9/2020|EPI_ISL_418143|2020-	····················	····A···············	····················	····················	····················	····················	
A	7449 hCoV-19/Wales/PHWC-240E8/2020|EPI_ISL_418144|2020-	····················	····A···············	····················	····················	····················	····················	
A	7450 hCoV-19/Wales/PHWC-240F7/2020|EPI_ISL_418145|2020-	····················	····A···············	····················	····················	····················	····················	
A	7451 hCoV-19/Wales/PHWC-24121/2020|EPI_ISL_418147|2020-	····················	····A···············	····················	····················	····················	····················	
A	7452 hCoV-19/Wales/PHWC-2415E/2020|EPI_ISL_418149|2020-	····················	····················	····················	····················	····················	····················	
A	7453 hCoV-19/Wales/PHWC-2416D/2020|EPI_ISL_418150|2020-	····················	····A···············	····················	····················	····················	····················	
A	7454 hCoV-19/Wales/PHWC-2419A/2020|EPI_ISL_418151|2020-	····················	····A···············	····················	····················	····················	····················	
A	7455 hCoV-19/Wales/PHWC-2422E/2020|EPI_ISL_418155|2020-	····················	····A···············	····················	····················	····················	····················	
A	7456 hCoV-19/Wales/PHWC-2423D/2020|EPI_ISL_418156|2020-	····················	····A···············	····················	····················	····················	····················	
A	7457 hCoV-19/Wales/PHWC-24279/2020|EPI_ISL_418158|2020-	····················	····A···············	····················	····················	····················	····················	
A	7458 hCoV-19/Wales/PHWC-24288/2020|EPI_ISL_418159|2020-	····················	····A···············	····················	····················	····················	····················	
A	7459 hCoV-19/Wales/PHWC-242C4/2020|EPI_ISL_418163|2020-	····················	····A···············	····················	····················	····················	····················	
A	7460 hCoV-19/Japan/P1/2020|EPI_ISL_419296|2020-03-08	····················	····················	····················	····················	····················	····················	
A	7461 hCoV-19/Japan/P2-1/2020|EPI_ISL_419297|2020-03-10	····················	····················	····················	····················	····················	····················	
A	7462 hCoV-19/Japan/P2-2/2020|EPI_ISL_419298|2020-03-10	····················	····················	····················	····················	····················	····················	
A	7463 hCoV-19/Japan/P3-1/2020|EPI_ISL_419299|2020-03-09	····················	····················	····················	····················	····················	····················	
A	7464 hCoV-19/Wales/PHWC-243D0/2020|EPI_ISL_419406|2020-	····················	····················	····················	····················	····················	····················	
A	7465 hCoV-19/Wales/PHWC-243EF/2020|EPI_ISL_419407|2020-	····················	····A···············	····················	····················	····················	····················	
A	7466 hCoV-19/Wales/PHWC-24419/2020|EPI_ISL_419409|2020-	····················	····A···············	····················	····················	····················	····················	
A	7467 hCoV-19/Wales/PHWC-244EC/2020|EPI_ISL_419420|2020-	····················	····A···············	····················	····················	····················	····················	
A	7468 hCoV-19/Wales/PHWC-244FB/2020|EPI_ISL_419421|2020-	····················	····M···············	····················	····················	····················	····················	
A	7469 hCoV-19/Wales/PHWC-24507/2020|EPI_ISL_419422|2020-	····················	····A···············	····················	····················	····················	····················	
A	7470 hCoV-19/Wales/PHWC-24525/2020|EPI_ISL_419424|2020-	····················	····A···············	····················	····················	····················	····················	
A	7471 hCoV-19/Wales/PHWC-24534/2020|EPI_ISL_419425|2020-	····················	····················	····················	····················	····················	····················	
A	7472 hCoV-19/Wales/PHWC-2458F/2020|EPI_ISL_419428|2020-	····················	····················	····················	····················	····················	····················	
A	7473 hCoV-19/Wales/PHWC-2465F/2020|EPI_ISL_419434|2020-	····················	····················	····················	····················	····················	····················	
A	7474 hCoV-19/Wales/PHWC-2469B/2020|EPI_ISL_419438|2020-	····················	····················	····················	····················	····················	····················	
A	7475 hCoV-19/Wales/PHWC-246AA/2020|EPI_ISL_419439|2020-	····················	····A···············	····················	····················	····················	····················	
A	7476 hCoV-19/Wales/PHWC-246B9/2020|EPI_ISL_419440|2020-	····················	····················	····················	····················	····················	····················	
A	7477 hCoV-19/Wales/PHWC-246E6/2020|EPI_ISL_419443|2020-	····················	····················	····················	····················	····················	····················	
A	7478 hCoV-19/Wales/PHWC-247F2/2020|EPI_ISL_419448|2020-	····················	····A···············	····················	····················	····················	····················	
A	7479 hCoV-19/Wales/PHWC-2480E/2020|EPI_ISL_419449|2020-	····················	····A···············	····················	····················	····················	····················	
A	7480 hCoV-19/Wales/PHWC-248B3/2020|EPI_ISL_419453|2020-	····················	····················	····················	····················	····················	····················	
A	7481 hCoV-19/Wales/PHWC-24A35/2020|EPI_ISL_419472|2020-	····················	····A···············	····················	····················	····················	····················	
A	7482 hCoV-19/Wales/PHWC-24A62/2020|EPI_ISL_419474|2020-	····················	····A···············	····················	····················	····················	····················	
A	7483 hCoV-19/Wales/PHWC-24BC9/2020|EPI_ISL_419496|2020-	····················	····················	····················	····················	····················	····················	
A	7484 hCoV-19/Wales/PHWC-24BE7/2020|EPI_ISL_419498|2020-	····················	····A···············	····················	····················	····················	····················	
A	7485 hCoV-19/Wales/PHWC-24BF6/2020|EPI_ISL_419499|2020-	····················	····A···············	····················	····················	····················	····················	
A	7486 hCoV-19/England/SHEF-C0172/2020|EPI_ISL_420259|202	····················	····················	····················	····················	····················	····················	
A	7487 hCoV-19/England/SHEF-C05A3/2020|EPI_ISL_420279|202	····················	····················	····················	····················	····················	····················	
A	7488 hCoV-19/England/SHEF-C06A0/2020|EPI_ISL_420280|202	····················	····················	····················	····················	····················	····················	
A	7489 hCoV-19/England/SHEF-BFFF4/2020|EPI_ISL_420281|202	····················	····················	····················	····················	····················	····················	
A	7490 hCoV-19/England/SHEF-C05C1/2020|EPI_ISL_420282|202	····················	····················	····················	····················	····················	····················	
A	7491 hCoV-19/England/SHEF-C001B/2020|EPI_ISL_420283|202	····················	····················	····················	····················	····················	····················	
A	7492 hCoV-19/England/SHEF-C0576/2020|EPI_ISL_420284|202	····················	····················	····················	····················	····················	····················	
A	7493 hCoV-19/England/SHEF-C060A/2020|EPI_ISL_420285|202	····················	····················	····················	····················	····················	····················	
A	7494 hCoV-19/England/SHEF-C00A2/2020|EPI_ISL_420286|202	····················	····················	····················	····················	····················	····················	
A	7495 hCoV-19/England/SHEF-C0048/2020|EPI_ISL_420287|202	····················	····················	····················	····················	····················	····················	
A	7496 hCoV-19/England/SHEF-C00FD/2020|EPI_ISL_420288|202	····················	····················	····················	····················	····················	····················	
A	7497 hCoV-19/England/SHEF-C0619/2020|EPI_ISL_420289|202	····················	····················	····················	····················	····················	····················	
A	7499 hCoV-19/England/SHEF-C00C0/2020|EPI_ISL_420291|202	····················	····················	····················	····················	····················	····················	
A	7500 hCoV-19/England/SHEF-C0655/2020|EPI_ISL_420292|202	····················	····················	····················	····················	····················	····················	
A	7501 hCoV-19/tiger/USA/NY-040420/2020|EPI_ISL_420293|20	····················	····················	····················	····················	····················	····················	

A	7502	hCoV-19/Slovenia/4584/2020|EPI_ISL_420294|2020-03-	····················	····················	·····················	····················	····················	····················
A	7503	hCoV-19/Slovenia/12304/2020|EPI_ISL_420295|2020-03	····················	····················	·····················	····················	····················	····················
A	7504	hCoV-19/USA/NY-NYUMC45/2020|EPI_ISL_420296|2020-03	····················	····················	·····················	····················	····················	····················
A	7505	hCoV-19/USA/NY-NYUMC46/2020|EPI_ISL_420297|2020-03	····················	····················	·····················	····················	····················	····················
A	7506	hCoV-19/USA/NY-NYUMC47/2020|EPI_ISL_420298|2020-03	····················	····················	·····················	····················	····················	····················
A	7507	hCoV-19/USA/NY-NYUMC48/2020|EPI_ISL_420299|2020-03	····················	····················	·····················	····················	····················	····················
A	7508	hCoV-19/Belgium/VT-0325143/2020|EPI_ISL_420400|202	····················	····················	·····················	····················	····················	····················
A	7509	hCoV-19/Belgium/PM-0324144/2020|EPI_ISL_420401|202	····················	····················	·····················	····················	····················	····················
A	7510	hCoV-19/Belgium/PC-0325147/2020|EPI_ISL_420404|202	····················	····················	·····················	····················	····················	····················
A	7511	hCoV-19/Belgium/LJ-0325148/2020|EPI_ISL_420405|202	····················	····················	·····················	····················	····················	····················
A	7512	hCoV-19/Belgium/BEG-0324149/2020|EPI_ISL_420406|20	····················	····················	·····················	····················	····················	····················
A	7513	hCoV-19/Belgium/CK-0324150/2020|EPI_ISL_420407|202	····················	····················	·····················	····················	····················	····················
A	7514	hCoV-19/Belgium/HCM-0324151/2020|EPI_ISL_420408|20	····················	····················	·····················	····················	····················	····················
A	7515	hCoV-19/Belgium/ZF-0325152/2020|EPI_ISL_420409|202	····················	····················	·····················	····················	····················	····················
A	7516	hCoV-19/Belgium/VPMV-0325153/2020|EPI_ISL_420410|2	····················	····················	·····················	····················	····················	····················
A	7517	hCoV-19/Belgium/BEG-0323154/2020|EPI_ISL_420411|20	····················	····················	·····················	····················	····················	····················
A	7518	hCoV-19/Belgium/BJ-0324192/2020|EPI_ISL_420412|202	····················	····················	·····················	····················	····················	····················
A	7519	hCoV-19/Belgium/DJMG-0325156/2020|EPI_ISL_420413|2	····················	····················	·····················	····················	····················	····················
A	7520	hCoV-19/Belgium/DRV-0325157/2020|EPI_ISL_420414|20	····················	····················	·····················	····················	····················	····················
A	7521	hCoV-19/Belgium/DCS-0325158/2020|EPI_ISL_420415|20	····················	····················	·····················	····················	····················	····················
A	7522	hCoV-19/Belgium/FMC-0323159/2020|EPI_ISL_420416|20	····················	····················	·····················	····················	····················	····················
A	7523	hCoV-19/Belgium/BCM-0324160/2020|EPI_ISL_420417|20	····················	····················	·····················	····················	····················	····················
A	7524	hCoV-19/Belgium/NIM-0324161/2020|EPI_ISL_420418|20	····················	····················	·····················	····················	····················	····················
A	7525	hCoV-19/Belgium/VLG-0325162/2020|EPI_ISL_420419|20	····················	····················	·····················	····················	····················	····················
A	7526	hCoV-19/Belgium/DCB-0324163/2020|EPI_ISL_420420|20	····················	····················	·····················	····················	····················	····················
A	7527	hCoV-19/Belgium/ML-0324164/2020|EPI_ISL_420421|202	····················	····················	·····················	····················	····················	····················
A	7528	hCoV-19/Belgium/JJC-0325165/2020|EPI_ISL_420422|20	····················	····················	·····················	····················	····················	····················
A	7529	hCoV-19/Belgium/PJM-0325166/2020|EPI_ISL_420423|20	····················	····················	·····················	····················	····················	····················
A	7530	hCoV-19/Belgium/CCM-0324167/2020|EPI_ISL_420424|20	····················	····················	·····················	····················	····················	····················
A	7531	hCoV-19/Belgium/RC-0324168/2020|EPI_ISL_420425|202	····················	····················	·····················	····················	····················	····················
A	7532	hCoV-19/Belgium/NMA-0324169/2020|EPI_ISL_420426|20	····················	····················	·····················	····················	····················	····················
A	7533	hCoV-19/Belgium/STM-0324170/2020|EPI_ISL_420427|20	····················	····················	·····················	····················	····················	····················
A	7534	hCoV-19/Belgium/VM-0325171/2020|EPI_ISL_420428|202	····················	····················	·····················	····················	····················	····················
A	7535	hCoV-19/Belgium/NKMA-0324172/2020|EPI_ISL_420429|2	····················	····················	·····················	····················	····················	····················
A	7536	hCoV-19/Belgium/QL-0324173/2020|EPI_ISL_420430|202	····················	····················	·····················	····················	····················	····················
A	7537	hCoV-19/Belgium/AGR-0324174/2020|EPI_ISL_420431|20	····················	····················	·····················	····················	····················	····················
A	7538	hCoV-19/Belgium/CJM-0323175/2020|EPI_ISL_420432|20	····················	····················	·····················	····················	····················	····················
A	7539	hCoV-19/Belgium/DNS-0324176/2020|EPI_ISL_420433|20	····················	····················	·····················	····················	····················	····················
A	7540	hCoV-19/Belgium/RHA-0324177/2020|EPI_ISL_420434|20	····················	····················	·····················	····················	····················	····················
A	7541	hCoV-19/Belgium/CJE-0325178/2020|EPI_ISL_420435|20	····················	····················	·····················	····················	····················	····················
A	7542	hCoV-19/Belgium/GLA-0324179/2020|EPI_ISL_420436|20	····················	····················	·····················	····················	····················	····················
A	7543	hCoV-19/Belgium/DMM-0325180/2020|EPI_ISL_420437|20	····················	····················	·····················	····················	····················	····················
A	7544	hCoV-19/Belgium/BJ-0325181/2020|EPI_ISL_420438|202	····················	····················	·····················	····················	····················	····················
A	7545	hCoV-19/Belgium/PC-0325182/2020|EPI_ISL_420439|202	····················	····················	·····················	····················	····················	····················
A	7546	hCoV-19/Belgium/BS-0325183/2020|EPI_ISL_420440|202	····················	····················	·····················	····················	····················	····················
A	7547	hCoV-19/Belgium/RA-030664/2020|EPI_ISL_420441|2020	····················	····················	·····················	····················	····················	····················
A	7548	hCoV-19/Belgium/BGM-030444/2020|EPI_ISL_420442|202	····················	····················	·····················	····················	····················	····················
A	7549	hCoV-19/Belgium/RA-030445/2020|EPI_ISL_420443|2020	····················	····················	·····················	····················	····················	····················
A	7550	hCoV-19/Belgium/MF-030546/2020|EPI_ISL_420444|2020	····················	····················	·····················	····················	····················	····················
A	7551	hCoV-19/Belgium/FAE-030948/2020|EPI_ISL_420445|202	····················	····················	·····················	····················	····················	····················
A	7552	hCoV-19/Belgium/DAL-030650/2020|EPI_ISL_420446|202	····················	····················	·····················	····················	····················	····················
A	7553	hCoV-19/Belgium/DJ-030452/2020|EPI_ISL_420447|2020	····················	····················	·····················	····················	····················	····················
A	7554	hCoV-19/Belgium/GSA-030454/2020|EPI_ISL_420448|202	····················	····················	·····················	····················	····················	····················
A	7555	hCoV-19/Belgium/VHC-030655/2020|EPI_ISL_420449|202	····················	····················	·····················	····················	····················	····················
A	7556	hCoV-19/Belgium/KN-030756/2020|EPI_ISL_420450|2020	····················	····················	·····················	····················	····················	····················
A	7557	hCoV-19/Belgium/HC-030760/2020|EPI_ISL_420451|2020	····················	····················	·····················	····················	····················	····················
A	7558	hCoV-19/Belgium/RDC-030961/2020|EPI_ISL_420452|202	····················	····················	·····················	····················	····················	····················
A	7559	hCoV-19/Belgium/VF-030562/2020|EPI_ISL_420453|2020	····················	····················	·····················	····················	····················	····················
A	7560	hCoV-19/Belgium/VJP-030463/2020|EPI_ISL_420454|202	····················	····················	·····················	····················	····················	····················
A	7561	hCoV-19/Australia/WA11/2020|EPI_ISL_420456|2020-02	····················	····················	·····················	····················	····················	····················
A	7562	hCoV-19/England/20114052703/2020|EPI_ISL_420458|20	····················	····················	·····················	····················	····················	····················
A	7563	hCoV-19/England/20120033002/2020|EPI_ISL_420464|20	····················	····················	·····················	····················	····················	····················
A	7564	hCoV-19/England/20126063003/2020|EPI_ISL_420466|20	···T················	····················	·····················	····················	····················	····················
A	7565	hCoV-19/England/20128035202/2020|EPI_ISL_420477|20	····················	····················	·····················	····················	····················	····················
A	7566	hCoV-19/England/20128046202/2020|EPI_ISL_420478|20	····················	····················	·····················	····················	····················	····················
A	7567	hCoV-19/England/20128046302/2020|EPI_ISL_420479|20	····················	····················	·····················	····················	····················	····················
A	7568	hCoV-19/England/20129040102/2020|EPI_ISL_420488|20	····················	····················	·····················	····················	····················	····················
A	7570	hCoV-19/USA/NY-NYUMC87/2020|EPI_ISL_421588|2020-03	····················	····················	·····················	····················	····················	····················
A	7571	hCoV-19/USA/NY-NYUMC88/2020|EPI_ISL_421589|2020-03	····················	····················	·····················	····················	····················	····················
A	7572	hCoV-19/USA/NY-NYUMC94/2020|EPI_ISL_421706|2020-03	····················	····················	·····················	····················	····················	····················
A	7573	hCoV-19/USA/NY-NYUMC95/2020|EPI_ISL_421707|2020-03	····················	····················	·····················	····················	····················	····················
A	7574	hCoV-19/USA/NY-NYUMC97/2020|EPI_ISL_421709|2020-03	····················	····················	·····················	····················	····················	····················
A	7575	hCoV-19/USA/NY-NYUMC98/2020|EPI_ISL_421710|2020-03	····················	····················	·····················	····················	····················	····················
A	7576	hCoV-19/USA/NY-NYUMC99/2020|EPI_ISL_421711|2020-03	····················	····················	·····················	····················	····················	····················
A	7577	hCoV-19/USA/NY-NYUMC100/2020|EPI_ISL_421712|2020-0	····················	····················	·····················	····················	····················	····················
A	7578	hCoV-19/USA/NY-NYUMC102/2020|EPI_ISL_421714|2020-0	····················	····················	·····················	····················	····················	····················
A	7579	hCoV-19/USA/NY-NYUMC103/2020|EPI_ISL_421715|2020-0	····················	····················	·····················	····················	····················	····················
A	7580	hCoV-19/USA/NY-NYUMC105/2020|EPI_ISL_421717|2020-0	····················	····················	·····················	····················	····················	····················

A	7581	hCoV-19/USA/NJ-NYUMC106/2020|EPI_ISL_421718|2020-0	....	....	....	....	....	....
A	7582	hCoV-19/USA/NY-NYUMC107/2020|EPI_ISL_421719|2020-0	....	....	....	....	....	....
A	7583	hCoV-19/USA/NY-NYUMC121/2020|EPI_ISL_421733|2020-0	....	....	....	....	....	....
A	7584	hCoV-19/Luxembourg/LNS3307371/2020|EPI_ISL_421735|	....	....	....	....	....	....
A	7585	hCoV-19/Luxembourg/LNS2334563/2020|EPI_ISL_421737|	....	....	....	....	....	....
A	7586	hCoV-19/Luxembourg/LNS3404815/2020|EPI_ISL_421738|	....	....	....	....	....	....
A	7587	hCoV-19/Luxembourg/LNS1644489/2020|EPI_ISL_421740|	....	....	....	....	....	....
A	7588	hCoV-19/Luxembourg/LNS6689955/2020|EPI_ISL_421741|	....	....	....	....	....	....
A	7589	hCoV-19/Luxembourg/LNS6500589/2020|EPI_ISL_421742|	....	....	....	....	....	....
A	7590	hCoV-19/Luxembourg/LNS0272441/2020|EPI_ISL_421743|	....	....	....	....	....	....
A	7591	hCoV-19/Luxembourg/LNS7074589/2020|EPI_ISL_421744|	....	....	....	....	....	....
A	7592	hCoV-19/Luxembourg/LNS2299410/2020|EPI_ISL_421745|	....	....	....	....	....	....
A	7593	hCoV-19/Luxembourg/LNS0819394/2020|EPI_ISL_421746|	....	....	....	....	....	....
A	7594	hCoV-19/Luxembourg/LNS3770609/2020|EPI_ISL_421747|	....	....	....	....	....	....
A	7595	hCoV-19/Luxembourg/LNS3052946/2020|EPI_ISL_421748|	....	....	....	....	....	....
A	7596	hCoV-19/Luxembourg/LNS3035957/2020|EPI_ISL_421750|	....	....	....	....	....	....
A	7597	hCoV-19/Luxembourg/LNS4569788/2020|EPI_ISL_421752|	....	....	....	....	....	....
A	7598	hCoV-19/Luxembourg/LNS7991123/2020|EPI_ISL_421753|	....	....	....	....	....	....
A	7599	hCoV-19/Luxembourg/LNS0814536/2020|EPI_ISL_421754|	....	....	....	....	....	....
A	7600	hCoV-19/Luxembourg/LNS6897699/2020|EPI_ISL_421755|	....	....	....	....	....	....
A	7601	hCoV-19/Luxembourg/LNS8124566/2020|EPI_ISL_421756|	....	....	....	....	....	....
A	7602	hCoV-19/Luxembourg/LNS7914820/2020|EPI_ISL_421757|	....	....	....	....	....	....
A	7603	hCoV-19/Luxembourg/LNS4235576/2020|EPI_ISL_421759|	....	....	....	....	....	....
A	7604	hCoV-19/Luxembourg/LNS2458673/2020|EPI_ISL_421760|	....	....	....	....	....	....
A	7605	hCoV-19/Luxembourg/LNS8602041/2020|EPI_ISL_421761|	....	....	....	....	....	....
A	7606	hCoV-19/Luxembourg/LNS9510711/2020|EPI_ISL_421762|	....	....	....	....	....	....
A	7607	hCoV-19/England/201360017/2020|EPI_ISL_421768|2020	....	....	....	....	....	....
A	7609	hCoV-19/England/201360185/2020|EPI_ISL_421771|2020	....	....	....	....	....	....
A	7610	hCoV-19/England/201360187/2020|EPI_ISL_421772|2020	....	....	....	....	....	....
A	7611	hCoV-19/England/201360289/2020|EPI_ISL_421773|2020	....	....	....	....	....	....
A	7612	hCoV-19/England/201360295/2020|EPI_ISL_421774|2020	....	....	....	....	....	....
A	7613	hCoV-19/England/201360297/2020|EPI_ISL_421776|2020	....	....	....	....	....	....
A	7614	hCoV-19/England/201360298/2020|EPI_ISL_421777|2020	....	....	....	....	....	....
A	7615	hCoV-19/England/201360299/2020|EPI_ISL_421778|2020	....	....	....	....	....	....
A	7616	hCoV-19/England/201360300/2020|EPI_ISL_421779|2020	....	....	....	....	....	....
A	7617	hCoV-19/England/201360492/2020|EPI_ISL_421780|2020	....	....	....	....	....	....
A	7618	hCoV-19/England/201360499/2020|EPI_ISL_421781|2020	....	....	....	....	....	....
A	7619	hCoV-19/England/201360506/2020|EPI_ISL_421782|2020	....	....	....	....	....	....
A	7620	hCoV-19/England/201360508/2020|EPI_ISL_421783|2020	....	....	....	....	....	....
A	7621	hCoV-19/England/201361007/2020|EPI_ISL_421784|2020	....	....	....	....	....	....
A	7622	hCoV-19/England/201361008/2020|EPI_ISL_421785|2020	....	....	....	....	....	....
A	7623	hCoV-19/England/201361012/2020|EPI_ISL_421787|2020	....	....	....	....	....	....
A	7625	hCoV-19/England/201361022/2020|EPI_ISL_421790|2020	....	....	....	....	....	....
A	7626	hCoV-19/England/201361040/2020|EPI_ISL_421791|2020	....	....	....	....	....	....
A	7628	hCoV-19/England/201380024/2020|EPI_ISL_421794|2020	....	....	....	....	....	....
A	7629	hCoV-19/England/201380025/2020|EPI_ISL_421795|2020	....	....	....	....	....	....
A	7630	hCoV-19/England/201380028/2020|EPI_ISL_421796|2020	....	....	....	....	....	....
A	7631	hCoV-19/England/201380030/2020|EPI_ISL_421797|2020	....	....	....	....	....	....
A	7632	hCoV-19/England/201380034/2020|EPI_ISL_421798|2020	....	....	....	....	....	....
A	7634	hCoV-19/England/CAMB-73A46/2020|EPI_ISL_425270|202	....	....	....	....	....	....
A	7636	hCoV-19/England/CAMB-73ABF/2020|EPI_ISL_425275|202	....	....	....	....	....	....
A	7637	hCoV-19/England/CAMB-73ACE/2020|EPI_ISL_425276|202	....	....	....	....	....	....
A	7638	hCoV-19/England/CAMB-73ADD/2020|EPI_ISL_425277|202	....	....	....	....	....	....
A	7639	hCoV-19/England/CAMB-73AEC/2020|EPI_ISL_425278|202	....	....	....	....	....	....
A	7640	hCoV-19/England/CAMB-74359/2020|EPI_ISL_425280|202	....	....	....	....	....	....
A	7641	hCoV-19/England/CAMB-74368/2020|EPI_ISL_425281|202	....	....	....	....	....	....
A	7642	hCoV-19/England/CAMB-74386/2020|EPI_ISL_425283|202	....	....	....	....	....	....
A	7643	hCoV-19/England/CAMB-743E0/2020|EPI_ISL_425286|202	....	....	....	....	....	....
A	7644	hCoV-19/England/CAMB-743FF/2020|EPI_ISL_425287|202	....	....	....	....	....	....
A	7645	hCoV-19/England/CAMB-7440B/2020|EPI_ISL_425288|202	....	....	....	....	....	....
A	7646	hCoV-19/England/CAMB-74438/2020|EPI_ISL_425290|202	....	....	....	....	....	....
A	7647	hCoV-19/England/CAMB-74465/2020|EPI_ISL_425292|202	....	....	....	....	....	....
A	7648	hCoV-19/England/CAMB-74474/2020|EPI_ISL_425293|202	....	....	....	....	....	....
A	7649	hCoV-19/England/CAMB-74483/2020|EPI_ISL_425294|202	....	....	....	....	....	....
A	7650	hCoV-19/England/CAMB-74492/2020|EPI_ISL_425295|202	....	....	....	....	....	....
A	7651	hCoV-19/England/CAMB-744A1/2020|EPI_ISL_425296|202	....	....	....	....	....	....
A	7652	hCoV-19/England/CAMB-744CF/2020|EPI_ISL_425298|202	....	....	....	....	....	....
A	7653	hCoV-19/Australia/NSW137/2020|EPI_ISL_427700|2020-	....	....	....	....	....	....
A	7654	hCoV-19/Australia/NSW180/2020|EPI_ISL_427701|2020-	....	....	....	....	....	....
A	7655	hCoV-19/Australia/NSW88/2020|EPI_ISL_427702|2020-0	....	....	....	....	....	....
A	7656	hCoV-19/Australia/NSW75/2020|EPI_ISL_427703|2020-0	....	....	....	....	....	....
A	7657	hCoV-19/Australia/NSW76/2020|EPI_ISL_427704|2020-0	....	....	....	....	....	....
A	7658	hCoV-19/Australia/NSW69/2020|EPI_ISL_427705|2020-0	....	....	....	....	....	....
A	7659	hCoV-19/Australia/NSW74/2020|EPI_ISL_427706|2020-0	....	....	....	....	....	....
A	7660	hCoV-19/Australia/NSW198/2020|EPI_ISL_427707|2020-	....	....	....	....	....	....
A	7661	hCoV-19/Australia/NSW145/2020|EPI_ISL_427708|2020-	....	....	....	....	....	....
A	7662	hCoV-19/Australia/NSW181/2020|EPI_ISL_427709|2020-	....	....	....	....	....	....
A	7663	hCoV-19/Australia/NSW201/2020|EPI_ISL_427710|2020-	....	....	....	....	....	....

```
A 7664 hCoV-19/Australia/NSW83/2020|EPI_ISL_427711|2020-0 ···················· ···················· ···················· ···················· ···················· ····················
A 7665 hCoV-19/Australia/NSW91/2020|EPI_ISL_427712|2020-0 ···················· ···················· ···················· ···················· ···················· ····················
A 7666 hCoV-19/Australia/NSW182/2020|EPI_ISL_427713|2020- ···················· ···················· ···················· ···················· ···················· ····················
A 7667 hCoV-19/Australia/NSW183/2020|EPI_ISL_427714|2020- ···················· ···················· ···················· ···················· ···················· ····················
A 7668 hCoV-19/Australia/NSW164/2020|EPI_ISL_427715|2020- ···················· ···················· ···················· ···················· ···················· ····················
A 7669 hCoV-19/Australia/NSW94/2020|EPI_ISL_427716|2020-0 ···················· ···················· ···················· ···················· ···················· ····················
A 7670 hCoV-19/Australia/NSW101/2020|EPI_ISL_427717|2020- ···················· ···················· ···················· ···················· ···················· ····················
A 7671 hCoV-19/Australia/NSW85/2020|EPI_ISL_427718|2020-0 ···················· ···················· ···················· ···················· ···················· ····················
A 7672 hCoV-19/Australia/NSW156/2020|EPI_ISL_427719|2020- ···················· ···················· ···················· ···················· ···················· ····················
A 7673 hCoV-19/Australia/NSW81/2020|EPI_ISL_427720|2020-0 ···················· ···················· ···················· ···················· ···················· ····················
A 7674 hCoV-19/Australia/NSW151/2020|EPI_ISL_427721|2020- ···················· ···················· ···················· ···················· ···················· ····················
A 7675 hCoV-19/Australia/NSW100/2020|EPI_ISL_427722|2020- ···················· ···················· ···················· ···················· ···················· ····················
A 7676 hCoV-19/Australia/NSW84/2020|EPI_ISL_427723|2020-0 ···················· ···················· ···················· ···················· ···················· ····················
A 7677 hCoV-19/Australia/NSW114/2020|EPI_ISL_427724|2020- ···················· ···················· ···················· ···················· ···················· ····················
A 7678 hCoV-19/Australia/NSW86/2020|EPI_ISL_427725|2020-0 ···················· ···················· ···················· ···················· ···················· ····················
A 7679 hCoV-19/Germany/BY-ChVir-1530/2020|EPI_ISL_450210| ···················· ···················· ···················· ···················· ···················· ····················
A 7680 hCoV-19/Germany/BY-ChVir-1536/2020|EPI_ISL_450211| ···················· ···················· ···················· ···················· ···················· ····················
A 7681 hCoV-19/Australia/NSW06/2020|EPI_ISL_413213|2020-0 ···················· ···················· ···················· ···················· ···················· ····················
A 7682 hCoV-19/Australia/NSW07/2020|EPI_ISL_413214|2020-0 ···················· ···················· ···················· ···················· ···················· ····················
A 7683 hCoV-19/Scotland/CVR01/2020|EPI_ISL_413221|2020-02 ···················· ···················· ···················· ···················· ···················· ····················
A 7684 hCoV-19/England/Sheff01/2020|EPI_ISL_414500|2020-0 ···················· ···················· ···················· ···················· ···················· ····················
A 7685 hCoV-19/England/Sheff02/2020|EPI_ISL_414501|2020-0 ···················· ···················· ···················· ···················· ···················· ····················
A 7686 hCoV-19/Germany/NW-HHU-06/2020|EPI_ISL_414505|2020 ···················· ···················· ···················· ···················· ···················· ····················
A 7687 hCoV-19/Germany/NW-HHU-08/2020|EPI_ISL_414508|2020 ···················· ···················· ···················· ···················· ···················· ····················
A 7688 hCoV-19/Germany/NW-HHU-09/2020|EPI_ISL_414509|2020 ···················· ···················· ···················· ···················· ···················· ····················
A 7689 hCoV-19/Shanghai/SH01/2020|EPI_ISL_414510|2020-02- ···················· ···················· ···················· ···················· ···················· ····················
A 7690 hCoV-19/Japan/TKYE6182/2020|EPI_ISL_414511|2020-01 ···················· ···················· ···················· ···················· ···················· ····················
A 7691 hCoV-19/Netherlands/NoordBrabant_28/2020|EPI_ISL_4 ···················· ···················· ···················· ···················· ···················· ····················
A 7692 hCoV-19/Netherlands/NoordBrabant_30/2020|EPI_ISL_4 ···················· ···················· ···················· ···················· ···················· ····················
A 7693 hCoV-19/Netherlands/NoordBrabant_32/2020|EPI_ISL_4 ···················· ···················· ···················· ···················· ···················· ····················
A 7694 hCoV-19/Netherlands/NoordBrabant_33/2020|EPI_ISL_4 ···················· ···················· ···················· ···················· ···················· ····················
A 7695 hCoV-19/Netherlands/NoordBrabant_34/2020|EPI_ISL_4 ···················· ···················· ···············C···· ···················· ···················· ····················
A 7696 hCoV-19/Netherlands/NoordBrabant_35/2020|EPI_ISL_4 ···················· ···················· ···················· ···················· ···················· ····················
A 7697 hCoV-19/Netherlands/NoordBrabant_36/2020|EPI_ISL_4 ···················· ···················· ···················· ···················· ···················· ····················
A 7698 hCoV-19/Netherlands/NoordBrabant_39/2020|EPI_ISL_4 ···················· ···················· ···················· ···················· ···················· ····················
A 7699 hCoV-19/Netherlands/NoordHolland_2/2020|EPI_ISL_41 ···················· ···················· ···················· ···················· ···················· ····················
A 7700 hCoV-19/Netherlands/Utrecht_47/2020|EPI_ISL_414551 ···················· ···················· ···················· ···················· ···················· ····················
A 7701 hCoV-19/Netherlands/Utrecht_46/2020|EPI_ISL_414552 ···················· ···················· ···················· ···················· ···················· ····················
A 7702 hCoV-19/Netherlands/Utrecht_15/2020|EPI_ISL_414554 ···················· ···················· ···················· ···················· ···················· ····················
A 7703 hCoV-19/Netherlands/Utrecht_16/2020|EPI_ISL_414555 ···················· ···················· ···················· ···················· ···················· ····················
A 7704 hCoV-19/Netherlands/ZuidHolland_2/2020|EPI_ISL_414 ···················· ···················· ···················· ···················· ···················· ····················
A 7705 hCoV-19/Netherlands/ZuidHolland_15/2020|EPI_ISL_41 ···················· ···················· ···················· ···················· ···················· ····················
A 7706 hCoV-19/Netherlands/ZuidHolland_16/2020|EPI_ISL_41 ···················· ···················· ···················· ···················· ···················· ····················
A 7707 hCoV-19/Netherlands/ZuidHolland_17/2020|EPI_ISL_41 ···················· ···················· ···················· ···················· ···················· ····················
A 7708 hCoV-19/Netherlands/ZuidHolland_18/2020|EPI_ISL_41 ···················· ···················· ···················· ···················· ···················· ····················
A 7709 hCoV-19/Netherlands/ZuidHolland_19/2020|EPI_ISL_41 ···················· ···················· ···················· ···················· ···················· ····················
A 7710 hCoV-19/Netherlands/ZuidHolland_20/2020|EPI_ISL_41 ···················· ···················· ···················· ···················· ···················· ····················
A 7711 hCoV-19/Netherlands/ZuidHolland_21/2020|EPI_ISL_41 ···················· ···················· ···················· ···················· ···················· ····················
A 7712 hCoV-19/Netherlands/ZuidHolland_22/2020|EPI_ISL_41 ···················· ···················· ···················· ···················· ···················· ····················
A 7713 hCoV-19/Netherlands/ZuidHolland_24/2020|EPI_ISL_41 ···················· ···················· ···················· ···················· ···················· ····················
A 7714 hCoV-19/Hong Kong/VM20002582-2/2020|EPI_ISL_414569 ···················· ···················· ···················· ···················· ···················· ····················
A 7715 hCoV-19/Hong Kong/VM20002849/2020|EPI_ISL_414571|2 ···················· ···················· ···················· ···················· ···················· ····················
A 7716 hCoV-19/Chile/ML-ISPCH-1/2020|EPI_ISL_414577|2020- ···················· ···················· ···················· ···················· ···················· ····················
A 7717 hCoV-19/Chile/ML-ISPCH-2/2020|EPI_ISL_414578|2020- ···················· ···················· ···················· ···················· ···················· ····················
A 7718 hCoV-19/Chile/RM-ISPCH-1/2020|EPI_ISL_414579|2020- ···················· ···················· ···················· ···················· ···················· ····················
A 7719 hCoV-19/Chile/RM-ISPCH-2/2020|EPI_ISL_414580|2020- ···················· ···················· ···················· ···················· ···················· ····················
A 7720 hCoV-19/Ireland/LK-NVRL-19934/2020|EPI_ISL_414586| ···················· ···················· ···················· ···················· ···················· ····················
A 7721 hCoV-19/Ireland/LK-NVRL-19935/2020|EPI_ISL_414587| ···················· ···················· ···················· ···················· ···················· ····················
A 7722 hCoV-19/USA/MN2-MDH2/2020|EPI_ISL_414589|2020-03-0 ···················· ···················· ···················· ···················· ···················· ····················
A 7723 hCoV-19/USA/MN3-MDH3/2020|EPI_ISL_414590|2020-03-0 ···················· ···················· ···················· ···················· ···················· ···············A····
A 7724 hCoV-19/USA/WA-UW22/2020|EPI_ISL_414591|2020-03-06 ···················· ···················· ···················· ···················· ···················· ····················
A 7725 hCoV-19/USA/WA-UW23/2020|EPI_ISL_414592|2020-03-06 ···················· ···················· ···················· ···················· ···················· ····················
A 7726 hCoV-19/USA/WA-UW24/2020|EPI_ISL_414593|2020-03-05 ···················· ···················· ···················· ···················· ···················· ····················
A 7727 hCoV-19/USA/WA-UW25/2020|EPI_ISL_414594|2020-03-05 ···················· ···················· ···················· ···················· ···················· ····················
A 7728 hCoV-19/USA/WA-UW26/2020|EPI_ISL_414595|2020-03-05 ···················· ···················· ···················· ···················· ···················· ····················
A 7729 hCoV-19/USA/WA-UW27/2020|EPI_ISL_414596|2020-03-04 ···················· ···················· ···················· ···················· ···················· ····················
A 7730 hCoV-19/USA/WA-UW28/2020|EPI_ISL_414597|2020-03-04 ···················· ···················· ···················· ···················· ···················· ····················
A 7731 hCoV-19/Spain/VC-FISABIO-3/2020|EPI_ISL_414598|202 ···················· ···················· ···················· ···················· ···················· ····················
A 7732 hCoV-19/USA/WA-UW275/2020|EPI_ISL_418060|2020-03-1 ···················· ···················· ···················· ···················· ···················· ····················
A 7733 hCoV-19/USA/UN-UW276/2020|EPI_ISL_418061|2020-03-1 ···················· ···················· ···················· ···················· ···················· ····················
A 7734 hCoV-19/USA/WA-UW277/2020|EPI_ISL_418062|2020-03-1 ···················· ···················· ···················· ···················· ···················· ····················
A 7735 hCoV-19/USA/WA-UW279/2020|EPI_ISL_418064|2020-03-1 ···················· ···················· ···················· ···················· ···················· ····················
A 7736 hCoV-19/USA/WA-UW282/2020|EPI_ISL_418067|2020-03-1 ···················· ···················· ···················· ···················· ···················· ····················
A 7737 hCoV-19/USA/WA-UW284/2020|EPI_ISL_418069|2020-03-1 ···················· ···················· ···················· ···················· ···················· ····················
A 7738 hCoV-19/USA/WA-UW285/2020|EPI_ISL_418070|2020-03-1 ···················· ···················· ···················· ···················· ···················· ····················
A 7739 hCoV-19/USA/WA-UW286/2020|EPI_ISL_418071|2020-03-1 ···················· ···················· ···················· ···················· ···················· ····················
A 7740 hCoV-19/USA/WA-UW287/2020|EPI_ISL_418072|2020-03-1 ···················· ···················· ···················· ···················· ···················· ····················
A 7741 hCoV-19/USA/WA-UW288/2020|EPI_ISL_418073|2020-03-1 ···················· ···················· ···················· ···················· ···················· ····················
```

A	7742	hCoV-19/USA/WA-UW289/2020|EPI_ISL_418074|2020-03-1	····················	····················	····················	····················	····················	····················	
A	7743	hCoV-19/USA/WA-UW290/2020|EPI_ISL_418075|2020-03-1	····················	····················	····················	····················	····················	····················	
A	7744	hCoV-19/USA/WA-UW291/2020|EPI_ISL_418076|2020-03-1	····················	····················	····················	····················	····················	····················	
A	7745	hCoV-19/USA/WA-UW292/2020|EPI_ISL_418077|2020-03-1	····················	····················	····················	····················	····················	····················	
A	7746	hCoV-19/USA/WA-UW293/2020|EPI_ISL_418078|2020-03-1	····················	····················	····················	····················	····················	····················	
A	7747	hCoV-19/USA/WA-UW294/2020|EPI_ISL_418079|2020-03-1	····················	····················	····················	····················	····················	····················	
A	7748	hCoV-19/USA/WA-UW295/2020|EPI_ISL_418080|2020-03-1	····················	····················	····················	····················	····················	····················	
A	7749	hCoV-19/USA/WA-UW296/2020|EPI_ISL_418081|2020-03-1	····················	····················	····················	····················	····················	····················	
A	7750	hCoV-19/USA/WA-UW297/2020|EPI_ISL_418082|2020-03-1	····················	····················	····················	····················	····················	····················	
A	7751	hCoV-19/Wales/PHWC-23B60/2020|EPI_ISL_418093|2020-	····················	····················	····················	····················	····················	····················	
A	7752	hCoV-19/Wales/PHWC-23BAC/2020|EPI_ISL_418096|2020-	····················	····················	····················	····················	····················	····················	
A	7753	hCoV-19/USA/MN62-MDH62/2020|EPI_ISL_419390|2020-03	····················	····················	····················	····················	····················	····················	
A	7754	hCoV-19/USA/MN63-MDH63/2020|EPI_ISL_419391|2020-03	····················	····················	····················	····················	····················	····················	
A	7755	hCoV-19/USA/MN64-MDH64/2020|EPI_ISL_419392|2020-03	····················	····················	····················	····················	····················	····················	
A	7756	hCoV-19/USA/MN65-MDH65/2020|EPI_ISL_419393|2020-03	····················	····················	····················	····················	····················	····················	
A	7757	hCoV-19/USA/MN66-MDH66/2020|EPI_ISL_419394|2020-03	····················	····················	····················	····················	····················	····················	
A	7758	hCoV-19/USA/MN67-MDH67/2020|EPI_ISL_419395|2020-03	····················	····················	····················	····················	····················	····················	
A	7759	hCoV-19/USA/MN68-MDH68/2020|EPI_ISL_419396|2020-03	····················	····················	····················	····················	····················	····················	
A	7760	hCoV-19/USA/MN69-MDH69/2020|EPI_ISL_419397|2020-03	····················	····················	····················	····················	····················	····················	
A	7761	hCoV-19/Wales/PHWC-24349/2020|EPI_ISL_419399|2020-	····················	····················	····················	····················	····················	····················	
A	7762	hCoV-19/Wales/PHWC-24455/2020|EPI_ISL_419412|2020-	····················	····A···············	····················	····················	····················	····················	
A	7763	hCoV-19/Wales/PHWC-244CE/2020|EPI_ISL_419418|2020-	····················	····A···············	····················	····················	····················	····················	
A	7764	hCoV-19/Belgium/MSE-0324121/2020|EPI_ISL_420378|20	····················	····················	····················	····················	····················	····················	
A	7765	hCoV-19/Belgium/RGE-0325122/2020|EPI_ISL_420379|20	····················	····················	····················	····················	····················	····················	
A	7766	hCoV-19/Belgium/DPG-0325123/2020|EPI_ISL_420380|20	····················	····················	····················	····················	····················	····················	
A	7767	hCoV-19/Belgium/FFJ-0324124/2020|EPI_ISL_420381|20	····················	····················	····················	····················	····················	····················	
A	7768	hCoV-19/Belgium/UNJP-0324125/2020|EPI_ISL_420382|2	····················	····················	····················	····················	····················	····················	
A	7769	hCoV-19/Belgium/NJC-0324126/2020|EPI_ISL_420383|20	····················	····················	····················	····················	····················	····················	
A	7770	hCoV-19/Belgium/NML-0325127/2020|EPI_ISL_420384|20	····················	····················	····················	····················	····················	····················	
A	7771	hCoV-19/Belgium/EC-0325128/2020|EPI_ISL_420385|202	····················	····················	····················	····················	····················	····················	
A	7772	hCoV-19/Belgium/MC-0323129/2020|EPI_ISL_420386|202	····················	····················	····················	····················	····················	····················	
A	7773	hCoV-19/Belgium/MNL-0324130/2020|EPI_ISL_420387|20	····················	····················	····················	····················	····················	····················	
A	7774	hCoV-19/Belgium/MSE-0324131/2020|EPI_ISL_420388|20	····················	····················	····················	····················	····················	····················	
A	7775	hCoV-19/Belgium/GMJ-0324132/2020|EPI_ISL_420389|20	····················	····················	····················	····················	····················	····················	
A	7776	hCoV-19/Belgium/LV-0324133/2020|EPI_ISL_420390|202	····················	····················	····················	····················	····················	····················	
A	7777	hCoV-19/Belgium/MS-0324134/2020|EPI_ISL_420391|202	····················	····················	····················	····················	····················	····················	
A	7778	hCoV-19/Belgium/DME-0324135/2020|EPI_ISL_420392|20	····················	····················	····················	····················	····················	····················	
A	7779	hCoV-19/Belgium/VWJM-0324136/2020|EPI_ISL_420393|2	····················	····················	····················	····················	····················	····················	
A	7780	hCoV-19/Belgium/OS-0325137/2020|EPI_ISL_420394|202	····················	····················	····················	····················	····················	····················	
A	7781	hCoV-19/Belgium/RS-0324138/2020|EPI_ISL_420395|202	····················	····················	····················	····················	····················	····················	
A	7782	hCoV-19/Belgium/GM-0323139/2020|EPI_ISL_420396|202	····················	····················	····················	····················	····················	····················	
A	7783	hCoV-19/Belgium/DB-0323140/2020|EPI_ISL_420397|202	····················	····················	····················	····················	····················	····················	
A	7784	hCoV-19/Belgium/NA-0323141/2020|EPI_ISL_420398|202	····················	····················	····················	····················	····················	····················	
A	7785	hCoV-19/Belgium/MAC-0324142/2020|EPI_ISL_420399|20	····················	····················	····················	····················	····················	····················	
A	7786	hCoV-19/Belgium/GJJ-0325145/2020|EPI_ISL_420402|20	····················	····················	····················	····················	····················	····················	
A	7787	hCoV-19/Belgium/DVG-0325146/2020|EPI_ISL_420403|20	····················	····················	····················	····················	····················	····················	
A	7788	hCoV-19/USA/NY-NYUMC108/2020|EPI_ISL_421720|2020-0	····················	····················	····················	····················	····················	····················	
A	7789	hCoV-19/USA/NY-NYUMC109/2020|EPI_ISL_421721|2020-0	····················	····················	····················	····················	····················	····················	
A	7790	hCoV-19/USA/NY-NYUMC110/2020|EPI_ISL_421722|2020-0	····················	····················	····················	····················	····················	····················	
A	7791	hCoV-19/USA/NJ-NYUMC111/2020|EPI_ISL_421723|2020-0	····················	····················	····················	····················	····················	····················	
A	7792	hCoV-19/USA/NY-NYUMC112/2020|EPI_ISL_421724|2020-0	····················	····················	····················	····················	····················	····················	
A	7793	hCoV-19/USA/NY-NYUMC113/2020|EPI_ISL_421725|2020-0	····················	····················	····················	····················	····················	····················	
A	7794	hCoV-19/USA/NY-NYUMC114/2020|EPI_ISL_421726|2020-0	····················	····················	····················	····················	····················	····················	
A	7795	hCoV-19/USA/NY-NYUMC115/2020|EPI_ISL_421727|2020-0	····················	····················	····················	····················	····················	····················	
A	7796	hCoV-19/USA/NY-NYUMC116/2020|EPI_ISL_421728|2020-0	····················	····················	····················	····················	····················	····················	
A	7797	hCoV-19/USA/NY-NYUMC117/2020|EPI_ISL_421729|2020-0	····················	····················	····················	····················	····················	····················	
A	7798	hCoV-19/USA/NY-NYUMC118/2020|EPI_ISL_421730|2020-0	····················	····················	····················	····················	····················	····················	
A	7799	hCoV-19/USA/NY-NYUMC119/2020|EPI_ISL_421731|2020-0	····················	····················	····················	····················	····················	····················	
A	7800	hCoV-19/USA/NY-NYUMC120/2020|EPI_ISL_421732|2020-0	····················	····················	····················	····················	····················	····················	
A	7801	hCoV-19/Luxembourg/LNS7970956/2020|EPI_ISL_421734|	····················	····················	····················	····················	····················	····················	
A	7802	hCoV-19/Spain/VC-FISABIO-77/2020|EPI_ISL_425201|20	····················	····················	····················	····················	····················	····················	
A	7803	hCoV-19/Spain/VC-FISABIO-79/2020|EPI_ISL_425203|20	····················	····················	····················	····················	····················	····················	
A	7804	hCoV-19/Spain/VC-FISABIO-81/2020|EPI_ISL_425205|20	····················	····················	····················	····················	····················	····················	
A	7805	hCoV-19/Spain/VC-FISABIO-86/2020|EPI_ISL_425210|20	····················	····················	····················	····················	····················	····················	
A	7806	hCoV-19/Spain/VC-FISABIO-88/2020|EPI_ISL_425212|20	····················	····················	····················	····················	····················	····················	
A	7807	hCoV-19/Spain/VC-FISABIO-89/2020|EPI_ISL_425213|20	····················	····················	····················	····················	····················	····················	
A	7808	hCoV-19/Spain/VC-FISABIO-90/2020|EPI_ISL_425214|20	····················	····················	····················	····················	····················	····················	
A	7809	hCoV-19/Spain/VC-FISABIO-92/2020|EPI_ISL_425216|20	····················	····················	····················	····················	····················	····················	
A	7810	hCoV-19/Spain/VC-FISABIO-93/2020|EPI_ISL_425217|20	····················	····················	····················	····················	····················	····················	
A	7811	hCoV-19/Spain/VC-FISABIO-94/2020|EPI_ISL_425218|20	····················	····················	····················	····················	····················	····················	
A	7812	hCoV-19/Spain/VC-FISABIO-95/2020|EPI_ISL_425219|20	····················	····················	····················	····················	····················	····················	
A	7813	hCoV-19/Spain/VC-FISABIO-96/2020|EPI_ISL_425220|20	····················	····················	····················	····················	····················	····················	
A	7814	hCoV-19/Spain/VC-FISABIO-97/2020|EPI_ISL_425221|20	····················	····················	····················	····················	····················	····················	
A	7815	hCoV-19/USA/NY-Wadsworth-10495-01/2020|EPI_ISL_425	····················	····················	····················	····················	····················	····················	
A	7816	hCoV-19/Czech Republic/2556/2020|EPI_ISL_425228|20	····················	····················	····················	····················	····················	····················	
A	7817	hCoV-19/Czech Republic/2122/2020|EPI_ISL_425229|20	····················	····················	····················	····················	····················	···············A····	
A	7818	hCoV-19/England/CAMB-7374F/2020|EPI_ISL_425231|202	····················	···················A	····················	····················	····················	····················	1
A	7819	hCoV-19/England/CAMB-7375E/2020|EPI_ISL_425232|202	····················	····················	····················	····················	····················	····················	

A	7820	hCoV-19/England/CAMB-7376D/2020|EPI_ISL_425233|202	....	....	....	....	....	....
A	7821	hCoV-19/England/CAMB-7377C/2020|EPI_ISL_425234|202	....	....	....	....	....	....
A	7822	hCoV-19/England/CAMB-7378B/2020|EPI_ISL_425235|202	....	....	....	....	....	....
A	7823	hCoV-19/England/CAMB-7379A/2020|EPI_ISL_425236|202	....	....	....	....	....	....
A	7825	hCoV-19/England/CAMB-737B8/2020|EPI_ISL_425238|202	....	....	....	....	....	....
A	7826	hCoV-19/England/CAMB-737C7/2020|EPI_ISL_425239|202	....	....	....	....	....	....
A	7827	hCoV-19/England/CAMB-737D6/2020|EPI_ISL_425240|202	....	....	....	....	....	....
A	7828	hCoV-19/England/CAMB-737F4/2020|EPI_ISL_425242|202	....	....	....	....	....	....
A	7829	hCoV-19/England/CAMB-73800/2020|EPI_ISL_425243|202	....	....	....	....	....	....
A	7830	hCoV-19/England/CAMB-7381F/2020|EPI_ISL_425244|202	....	....	....	....	....	....
A	7831	hCoV-19/England/CAMB-7386A/2020|EPI_ISL_425246|202	....	....	....	....	....	....
A	7832	hCoV-19/England/CAMB-73888/2020|EPI_ISL_425248|202	....	....	....	....	....	....
A	7833	hCoV-19/England/CAMB-738A6/2020|EPI_ISL_425249|202	....	....	....	....	....	....
A	7834	hCoV-19/England/CAMB-738B5/2020|EPI_ISL_425250|202	....	....	....	....	....	....
A	7835	hCoV-19/England/CAMB-738C4/2020|EPI_ISL_425251|202	....	....	....	....	....	....
A	7836	hCoV-19/England/CAMB-738D3/2020|EPI_ISL_425252|202	....	....	....	....	....	....
A	7837	hCoV-19/England/CAMB-738E2/2020|EPI_ISL_425253|202	....	....	....	....	....	....
A	7838	hCoV-19/England/CAMB-7391C/2020|EPI_ISL_425255|202	....	....	....	....	....	....
A	7839	hCoV-19/England/CAMB-73949/2020|EPI_ISL_425257|202	....	....	....	....	....	....
A	7840	hCoV-19/England/CAMB-73976/2020|EPI_ISL_425258|202	....	....	....	....	....	....
A	7841	hCoV-19/England/CAMB-73985/2020|EPI_ISL_425259|202	....	....	....	....	....	....
A	7842	hCoV-19/England/CAMB-73994/2020|EPI_ISL_425260|202	....	....	....	....	....	....
A	7843	hCoV-19/England/CAMB-739A3/2020|EPI_ISL_425261|202	....	....	....	....	....	....
A	7844	hCoV-19/England/CAMB-739B2/2020|EPI_ISL_425262|202	....	....	....	....	....	....
A	7845	hCoV-19/England/CAMB-739D0/2020|EPI_ISL_425263|202	....	....	....	....	....	....
A	7846	hCoV-19/England/CAMB-739EF/2020|EPI_ISL_425264|202	....	....	....	....	....	....
A	7847	hCoV-19/England/CAMB-73A0A/2020|EPI_ISL_425266|202	....	....	....	....	....	....
A	7848	hCoV-19/England/CAMB-73A19/2020|EPI_ISL_425267|202	....	....	....	....	....	....
A	7849	hCoV-19/England/CAMB-73A28/2020|EPI_ISL_425268|202	....	....	....	....	....	....
A	7850	hCoV-19/England/CAMB-73A37/2020|EPI_ISL_425269|202	....	....	....	....	....	NN..................
A	7851	hCoV-19/USA/AZ-TG268099/2020|EPI_ISL_426500|2020-0	....	....	....	....	....	....
A	7852	hCoV-19/USA/AZ-TG268183/2020|EPI_ISL_426501|2020-0	....	....	....	....	....	....
A	7853	hCoV-19/USA/AZ-TG268188/2020|EPI_ISL_426502|2020-0	....	....	....	....	....	....
A	7854	hCoV-19/USA/AZ-TG268281/2020|EPI_ISL_426503|2020-0	....	....	....	....	....	....
A	7855	hCoV-19/USA/AZ-TG268282/2020|EPI_ISL_426504|2020-0	....	....	....	....	....	....
A	7856	hCoV-19/USA/AZ-TG268289/2020|EPI_ISL_426505|2020-0	....	....	....	....	....	....
A	7857	hCoV-19/USA/AZ-TG268350/2020|EPI_ISL_426506|2020-0	....	....	....	....	....	....
A	7858	hCoV-19/USA/AZ-TG268360/2020|EPI_ISL_426507|2020-0	....	....	....	....	....	....
A	7859	hCoV-19/USA/AZ-TG268417/2020|EPI_ISL_426508|2020-0	....	....	....	....	....	....
A	7860	hCoV-19/USA/AZ-TG268629/2020|EPI_ISL_426509|2020-0	....	....	....	....	....	....
A	7861	hCoV-19/USA/AZ-TG268657/2020|EPI_ISL_426510|2020-0	....	....	....	....	....	....
A	7862	hCoV-19/USA/AZ-TG268770/2020|EPI_ISL_426511|2020-0	....	....	....	....	....	....
A	7863	hCoV-19/USA/AZ-TG268893/2020|EPI_ISL_426512|2020-0	....	....	....	....	....	....
A	7864	hCoV-19/USA/AZ-TG268903/2020|EPI_ISL_426513|2020-0	....	....	....	....	....	....
A	7865	hCoV-19/USA/AZ-TG268905/2020|EPI_ISL_426514|2020-0	....	....	....	....	....	....
A	7866	hCoV-19/USA/AZ-TG268909/2020|EPI_ISL_426515|2020-0	....	....	....	....	....	....
A	7867	hCoV-19/USA/AZ-TG268911/2020|EPI_ISL_426516|2020-0	....	....	....	....	....	....
A	7868	hCoV-19/USA/AZ-TG268913/2020|EPI_ISL_426517|2020-0	....	....	....	....	....	....
A	7869	hCoV-19/USA/AZ-TG268915/2020|EPI_ISL_426518|2020-0	....	....	....	....	....	....
A	7870	hCoV-19/USA/AZ-TG268917/2020|EPI_ISL_426519|2020-0	....	....	....	....	....	....
A	7871	hCoV-19/USA/AZ-TG269027/2020|EPI_ISL_426520|2020-0	....	....	....	....	....	....
A	7872	hCoV-19/USA/AZ-TG269238/2020|EPI_ISL_426521|2020-0	....	....	....	....	....	....
A	7873	hCoV-19/USA/AZ-TG269290/2020|EPI_ISL_426522|2020-0	....	....	....	....	....	....
A	7874	hCoV-19/USA/AZ-TG269358/2020|EPI_ISL_426523|2020-0	....	....	....	....	....	....
A	7875	hCoV-19/USA/AZ-TG269439/2020|EPI_ISL_426524|2020-0	....	....	....	....	....	....
A	7876	hCoV-19/USA/AZ-TG269440/2020|EPI_ISL_426525|2020-0	....	....	....	....	....	....
A	7877	hCoV-19/USA/AZ-TG269824/2020|EPI_ISL_426526|2020-0	....	....	....	....	....	....
A	7878	hCoV-19/USA/AZ-TG269848/2020|EPI_ISL_426527|2020-0	....	....	....	....	....	....
A	7879	hCoV-19/USA/AZ-TG269856/2020|EPI_ISL_426528|2020-0	....	....	....	....	....	....
A	7880	hCoV-19/USA/AZ-TG269863/2020|EPI_ISL_426529|2020-0	....	....	....	....	....	....
A	7881	hCoV-19/USA/AZ-TG269875/2020|EPI_ISL_426530|2020-0	....	....	....	....	....	....
A	7882	hCoV-19/USA/AZ-TG269883/2020|EPI_ISL_426531|2020-0	....	....	....	....	....	....
A	7883	hCoV-19/USA/AZ-TG270152/2020|EPI_ISL_426532|2020-0	....	....	....	....	....	....
A	7884	hCoV-19/USA/AZ-TG270200/2020|EPI_ISL_426533|2020-0	....	....	....	....	....	....
A	7885	hCoV-19/USA/AZ-TG271435/2020|EPI_ISL_426534|2020-0	....	....	....	....	....	....
A	7886	hCoV-19/USA/AZ-TG271578/2020|EPI_ISL_426535|2020-0	....	....	....	....	....	....
A	7887	hCoV-19/USA/AZ-TG271591/2020|EPI_ISL_426536|2020-0	....	....	....	....	....	....
A	7888	hCoV-19/USA/AZ-TG271854/2020|EPI_ISL_426537|2020-0	....	....	....	....	....	....
A	7889	hCoV-19/USA/AZ-TG271856/2020|EPI_ISL_426538|2020-0	....	....	....	....	....	....
A	7890	hCoV-19/USA/AZ-TG271864/2020|EPI_ISL_426540|2020-0	....	....	....	....	....	....
A	7891	hCoV-19/USA/AZ-TG271870/2020|EPI_ISL_426541|2020-0	....	....	....	....	....	....
A	7892	hCoV-19/USA/AZ-TG271878/2020|EPI_ISL_426542|2020-0	....	....	....	....	....	....
A	7893	hCoV-19/USA/AZ-TG271880/2020|EPI_ISL_426543|2020-0	....	....	....	....	....	....
A	7894	hCoV-19/USA/AZ-TG271882/2020|EPI_ISL_426544|2020-0	....	....	....	....	....	....
A	7895	hCoV-19/USA/AZ-TG271884/2020|EPI_ISL_426545|2020-0	....	....	....	....	....	....
A	7896	hCoV-19/USA/AZ-TG271886/2020|EPI_ISL_426546|2020-0	....	....	....	....	....	....
A	7897	hCoV-19/USA/AZ-TG271888/2020|EPI_ISL_426547|2020-0	....	....	....	....	....	....
A	7898	hCoV-19/USA/AZ-TG271890/2020|EPI_ISL_426548|2020-0	....	....	....	....	....	....

A	7899 hCoV-19/USA/AZ-TG271892/2020|EPI_ISL_426549|2020-0	····················	····················	····················	····················	····················	····················		
A	7900 hCoV-19/USA/AZ-TG271894/2020|EPI_ISL_426550|2020-0	····················	····················	····················	····················	····················	····················		
A	7901 hCoV-19/USA/AZ-TG271896/2020|EPI_ISL_426551|2020-0	····················	····················	····················	····················	····················	····················		
A	7902 hCoV-19/USA/AZ-TG271898/2020|EPI_ISL_426552|2020-0	····················	····················	····················	····················	····················	····················		
A	7903 hCoV-19/USA/AZ-TG271900/2020|EPI_ISL_426553|2020-0	····················	····················	····················	····················	····················	····················		
A	7904 hCoV-19/USA/AZ-TG271902/2020|EPI_ISL_426554|2020-0	····················	····················	····················	····················	····················	····················		
A	7905 hCoV-19/USA/AZ-TG271922/2020|EPI_ISL_426555|2020-0	····················	····················	····················	····················	····················	····················		
A	7906 hCoV-19/USA/AZ-TG272213/2020|EPI_ISL_426556|2020-0	····················	····················	····················	····················	····················	····················		
A	7907 hCoV-19/USA/AZ-TG272215/2020|EPI_ISL_426557|2020-0	····················	····················	····················	····················	····················	····················		
A	7908 hCoV-19/USA/AZ-TG273280/2020|EPI_ISL_426558|2020-0	····················	····················	····················	····················	····················	····················		
A	7909 hCoV-19/USA/AZ-TG273282/2020|EPI_ISL_426559|2020-0	····················	····················	····················	····················	····················	····················		
A	7910 hCoV-19/USA/AZ-TG273286/2020|EPI_ISL_426560|2020-0	····················	····················	····················	····················	····················	····················		
A	7911 hCoV-19/USA/AZ-TG273294/2020|EPI_ISL_426561|2020-0	····················	····················	····················	····················	····················	····················		
A	7912 hCoV-19/USA/AZ-TG273300/2020|EPI_ISL_426562|2020-0	····················	····················	····················	····················	····················	····················		
A	7913 hCoV-19/USA/AZ-TG273302/2020|EPI_ISL_426563|2020-0	····················	····················	····················	····················	····················	····················		
A	7914 hCoV-19/USA/AZ-TG273304/2020|EPI_ISL_426564|2020-0	····················	····················	····················	····················	····················	····················		
A	7915 hCoV-19/USA/AZ-TG273308/2020|EPI_ISL_426565|2020-0	····················	····················	····················	····················	····················	····················		
A	7916 hCoV-19/USA/AZ-TG273310/2020|EPI_ISL_426566|2020-0	····················	····················	····················	····················	····················	····················		
A	7917 hCoV-19/USA/AZ-TG273314/2020|EPI_ISL_426567|2020-0	····················	····················	····················	····················	····················	····················		
A	7918 hCoV-19/USA/AZ-TG273316/2020|EPI_ISL_426568|2020-0	····················	····················	····················	····················	····················	····················		
A	7919 hCoV-19/USA/AZ-TG273318/2020|EPI_ISL_426569|2020-0	····················	····················	····················	····················	····················	····················		
A	7920 hCoV-19/Brazil/DF-0001/2020|EPI_ISL_426580|2020-03	····················	····················	····················	····················	····················	····················		
A	7921 hCoV-19/Czech Republic/IAB20-006-16/2020|EPI_ISL_4	····················	····················	····················	····················	····················	····················		
A	7922 hCoV-19/Australia/NSW87/2020|EPI_ISL_427800|2020-0	····················	····················	····················	····················	····················	····················		
A	7923 hCoV-19/Australia/NSW173/2020|EPI_ISL_427801|2020-	····················	····················	····················	····················	····················	····················		
A	7924 hCoV-19/Australia/NSW141/2020|EPI_ISL_427802|2020-	····················	····················	····················	····················	····················	····················		
A	7925 hCoV-19/Australia/NSW186/2020|EPI_ISL_427803|2020-	····················	····················	····················	····················	····················	····················		
A	7926 hCoV-19/Australia/NSW108/2020|EPI_ISL_427804|2020-	····················	····················	····················	····················	····················	····················		
A	7927 hCoV-19/Germany/BY-ChVir-1020/2020|EPI_ISL_450200|	····················	····················	····················	····················	····················	····················		
A	7928 hCoV-19/Germany/BY-ChVir-1247/2020|EPI_ISL_450201|	····················	····················	····················	····················	····················	····················		
A	7929 hCoV-19/Germany/BY-ChVir-1248/2020|EPI_ISL_450202|	····················	····················	····················	····················	····················	····················		
A	7930 hCoV-19/Germany/BY-ChVir-1289/2020|EPI_ISL_450203|	····················	····················	····················	····················	····················	····················		
A	7931 hCoV-19/Germany/BY-ChVir-1356/2020|EPI_ISL_450204|	····················	····················	····················	····················	····················	····················		
A	7932 hCoV-19/Germany/BY-ChVir-1433/2020|EPI_ISL_450205|	····················	····················	····················	····················	····················	····················		
A	7933 hCoV-19/Germany/BY-ChVir-1459/2020|EPI_ISL_450206|	····················	····················	····················	····················	····················	····················		
A	7934 hCoV-19/Germany/BY-ChVir-1482/2020|EPI_ISL_450207|	····················	····················	····················	····················	····················	····················		
A	7935 hCoV-19/Germany/BY-ChVir-1483/2020|EPI_ISL_450208|	····················	····················	····················	····················	····················	····················		
A	7936 hCoV-19/Germany/BY-ChVir-1017/2020|EPI_ISL_450209|	····················	····················	····················	····················	····················	····················		
A	7937 hCoV-19/France/IDF-0515/2020|EPI_ISL_408430|2020-0	····················	····················	····················	····················	····················	····················		
A	7938 hCoV-19/France/IDF-0626/2020|EPI_ISL_408431|2020-0	····················	····················	····················	····················	····················	····················		
A	7939 hCoV-19/Chongqing/YC01/2020|EPI_ISL_408478|2020-01	····················	····················	····················	····················	····················	····················		
A	7940 hCoV-19/Chongqing/ZX01/2020|EPI_ISL_408479|2020-01	····················	····················	····················	····················	····················	····················		
A	7941 hCoV-19/Yunnan/IVDC-YN-003/2020|EPI_ISL_408480|202	····················	····················	····················	····················	····················	····················		
A	7942 hCoV-19/Chongqing/IVDC-CQ-001/2020|EPI_ISL_408481|	····················	····················	····················	····················	····················	····················		
A	7943 hCoV-19/Shandong/IVDC-SD-001/2020|EPI_ISL_408482|2	····················	····················	····················	····················	····················	····················		
A	7944 hCoV-19/Sichuan/IVDC-SC-001/2020|EPI_ISL_408484|20	····················	····················	····················	····················	····················	····················		
A	7945 hCoV-19/Beijing/IVDC-BJ-005/2020|EPI_ISL_408485|20	····················	····················	····················	····················	····················	····················		
A	7946 hCoV-19/Jiangxi/IVDC-JX-002/2020|EPI_ISL_408486|20	····················	····················	····················	····················	····················	····················		
A	7947 hCoV-19/Jiangsu/IVDC-JS-001/2020|EPI_ISL_408488|20	····················	····················	····················	····················	····················	····················		
A	7948 hCoV-19/Taiwan/NTU01/2020|EPI_ISL_408489|2020-01-3	····················	····················	····················	····················	····················	····················		
A	7949 hCoV-19/Singapore/11/2020|EPI_ISL_410719|2020-02-0	····················	····················	····················	····················	····················	····················		
A	7950 hCoV-19/France/IDF-0372-isl/2020|EPI_ISL_410720|20	····················	····················	····················	····················	····················	····················		
A	7951 hCoV-19/pangolin/Guangdong/1/2019|EPI_ISL_410721|2	····················	····················	····················	····················	·TCTT·T·A··T········	···A··T··G·····A··A·	1	1
A	7952 hCoV-19/Hong Kong/VM20002907/2020|EPI_ISL_414517|2	····················	····················	····················	····················	····················	····················		
A	7953 hCoV-19/Hong Kong/VM20002868/2020|EPI_ISL_414519|2	····················	····················	····················	····················	····················	····················		
A	7954 hCoV-19/Germany/BY-BIM-Pat2/2020|EPI_ISL_414520|20	····················	····················	····················	····················	····················	····················		
A	7955 hCoV-19/Germany/BY-BIM-Pat3/2020|EPI_ISL_414521|20	····················	····················	····················	····················	····················	····················		
A	7956 hCoV-19/England/200990002/2020|EPI_ISL_414522|2020	····················	····················	····················	····················	····················	····················		
A	7957 hCoV-19/England/200990660/2020|EPI_ISL_414523|2020	····················	····················	····················	····················	····················	····················		
A	7958 hCoV-19/England/200991076/2020|EPI_ISL_414524|2020	···T················	····················	····················	····················	····················	····················		
A	7959 hCoV-19/England/201040081/2020|EPI_ISL_414525|2020	····················	····················	····················	····················	····················	····················		
A	7960 hCoV-19/England/201040141/2020|EPI_ISL_414526|2020	····················	····················	····················	····················	····················	····················		
A	7961 hCoV-19/Hong Kong/VM20002493-2/2020|EPI_ISL_414527	····················	····················	····················	····················	····················	····················		
A	7962 hCoV-19/Hong Kong/VM20002507-2/2020|EPI_ISL_414528	····················	····················	····················	····················	····················	····················		
A	7963 hCoV-19/Netherlands/NoordBrabant_20/2020|EPI_ISL_4	····················	····················	····················	····················	····················	····················		
A	7964 hCoV-19/Netherlands/NoordBrabant_21/2020|EPI_ISL_4	····················	····················	····················	····················	····················	····················		
A	7965 hCoV-19/Netherlands/NoordBrabant_22/2020|EPI_ISL_4	····················	····················	····················	····················	····················	····················		
A	7966 hCoV-19/Netherlands/NoordBrabant_23/2020|EPI_ISL_4	····················	····················	····················	····················	····················	····················		
A	7967 hCoV-19/Netherlands/NoordBrabant_25/2020|EPI_ISL_4	····················	····················	····················	····················	····················	····················		
A	7968 hCoV-19/Netherlands/NoordBrabant_26/2020|EPI_ISL_4	····················	····················	····················	····················	····················	····················		
A	7969 hCoV-19/Netherlands/NoordBrabant_27/2020|EPI_ISL_4	····················	····················	····················	····················	····················	····················		
A	7970 hCoV-19/USA/WA-UW63/2020|EPI_ISL_415591|2020-03-10	····················	····················	····················	····················	····················	····················		
A	7971 hCoV-19/USA/WA-UW64/2020|EPI_ISL_415592|2020-03-09	····················	····················	····················	····················	····················	····················		
A	7972 hCoV-19/USA/WA-UW66/2020|EPI_ISL_415594|2020-03-10	····················	····················	····················	····················	····················	····················		
A	7973 hCoV-19/USA/WA-UW67/2020|EPI_ISL_415595|2020-03-09	····················	····················	····················	····················	····················	····················		
A	7974 hCoV-19/USA/WA-UW68/2020|EPI_ISL_415596|2020-03-09	····················	····················	····················	····················	····················	····················		
A	7975 hCoV-19/USA/WA-UW69/2020|EPI_ISL_415597|2020-03-10	····················	····················	····················	····················	····················	····················		
A	7976 hCoV-19/USA/WA-UW70/2020|EPI_ISL_415598|2020-03-10	····················	····················	····················	····················	····················	····················		

A	7977	hCoV-19/USA/WA-UW71/2020|EPI_ISL_415599|2020-03-09	....	....	....	....	....	....	
A	7978	hCoV-19/Singapore/23/2020|EPI_ISL_420100|2020-03-0	....	....	....	....	....	....	
A	7979	hCoV-19/Singapore/24/2020|EPI_ISL_420101|2020-03-0	....	....	....	....	....	....	
A	7980	hCoV-19/Singapore/25/2020|EPI_ISL_420102|2020-03-0	....	....	....	....	....	....	
A	7981	hCoV-19/Singapore/26/2020|EPI_ISL_420103|2020-03-0	....	....	....	....	....	....	
A	7982	hCoV-19/Singapore/27/2020|EPI_ISL_420104|2020-03-0	....	....	....	....	....	....	
A	7983	hCoV-19/Singapore/28/2020|EPI_ISL_420105|2020-03-0	....	....	....	....	....	....	
A	7984	hCoV-19/Singapore/29/2020|EPI_ISL_420106|2020-03-0	....	....	....	....	....	....	
A	7985	hCoV-19/Singapore/30/2020|EPI_ISL_420107|2020-03-0	....	....	....	....	....	....	
A	7986	hCoV-19/Singapore/31/2020|EPI_ISL_420108|2020-03-1	....	....	....	....	....	....	
A	7987	hCoV-19/Singapore/32/2020|EPI_ISL_420109|2020-03-1	....	....	....	....	....	....	
A	7988	hCoV-19/Singapore/33/2020|EPI_ISL_420110|2020-03-1	....	....	....	....	....	....	
A	7989	hCoV-19/Singapore/34/2020|EPI_ISL_420111|2020-03-1	....	....	....	....	....	....	
A	7990	hCoV-19/Spain/VC-FISABIO-27/2020|EPI_ISL_420112|20	....	....	....	....	....	....	
A	7991	hCoV-19/Spain/VC-FISABIO-28/2020|EPI_ISL_420113|20	....	....	....	....	....	....	
A	7992	hCoV-19/Spain/VC-FISABIO-29/2020|EPI_ISL_420114|20	....	....	....	....	....	....	
A	7993	hCoV-19/Spain/VC-FISABIO-30/2020|EPI_ISL_420115|20	....	....	....	....	....	....	
A	7994	hCoV-19/Spain/VC-FISABIO-31/2020|EPI_ISL_420116|20	....	....	....	....	....	....	
A	7995	hCoV-19/Spain/VC-FISABIO-32/2020|EPI_ISL_420117|20	....	....	....	....	....	....	
A	7996	hCoV-19/Spain/VC-FISABIO-33/2020|EPI_ISL_420118|20	....	....	....	....	....	....	
A	7997	hCoV-19/Spain/VC-FISABIO-34/2020|EPI_ISL_420119|20	....	....	....	....	....	....	
A	7998	hCoV-19/Spain/VC-FISABIO-35/2020|EPI_ISL_420120|20	....	....	....	....	....	....	
A	7999	hCoV-19/Spain/VC-FISABIO-36/2020|EPI_ISL_420121|20	....	....	....	....	....	....	
A	8000	hCoV-19/Spain/VC-FISABIO-37/2020|EPI_ISL_420122|20	....	....	....	....	....	....	
A	8001	hCoV-19/Spain/VC-FISABIO-38/2020|EPI_ISL_420123|20	....	....	....	....	....	....	
A	8002	hCoV-19/Spain/VC-FISABIO-39/2020|EPI_ISL_420124|20	....	....	....	....	....	....	
A	8003	hCoV-19/Spain/VC-FISABIO-41/2020|EPI_ISL_420125|20	....	....	....	....	....	....	
A	8004	hCoV-19/Spain/VC-FISABIO-42/2020|EPI_ISL_420126|20	....	....	....	....	....	....	
A	8005	hCoV-19/Spain/VC-FISABIO-43/2020|EPI_ISL_420127|20	....	....	....	....	....	....	
A	8006	hCoV-19/Spain/VC-FISABIO-44/2020|EPI_ISL_420128|20	....	....	....	....	....	....	
A	8007	hCoV-19/Spain/VC-FISABIO-45/2020|EPI_ISL_420129|20	....	....	....	....	....	....	
A	8008	hCoV-19/Spain/VC-FISABIO-49/2020|EPI_ISL_420130|20	....	....	....	....	....	....	
A	8009	hCoV-19/Spain/VC-FISABIO-50/2020|EPI_ISL_420131|20	....	....	....	....	....	....	
A	8010	hCoV-19/Spain/VC-FISABIO-51/2020|EPI_ISL_420132|20	....	....	....	....	....	....	
A	8011	hCoV-19/Norway/1455/2020|EPI_ISL_420135|2020-02-28	....	....	....	....	....	....	
A	8012	hCoV-19/USA/NY-PV08449/2020|EPI_ISL_421400|2020-03	....	....	....	....	....	....	
A	8013	hCoV-19/USA/NY-PV08450/2020|EPI_ISL_421401|2020-03	....	....	....	....	....	....	
A	8014	hCoV-19/USA/NY-PV08452/2020|EPI_ISL_421402|2020-03	....	....	....	....	....	....	
A	8015	hCoV-19/USA/NY-PV08453/2020|EPI_ISL_421403|2020-03	....	....	....	....	....	....	
A	8016	hCoV-19/USA/NY-PV08455/2020|EPI_ISL_421404|2020-03	....	....	....	....	....	....	
A	8017	hCoV-19/USA/NY-PV08456/2020|EPI_ISL_421405|2020-03	....	....	....	....	....	....	
A	8018	hCoV-19/USA/NY-PV08459/2020|EPI_ISL_421406|2020-03	....	....	....	....	....	....	
A	8019	hCoV-19/USA/NY-PV08461/2020|EPI_ISL_421407|2020-03	....	....	....	....	....	....	
A	8020	hCoV-19/USA/NY-PV08462/2020|EPI_ISL_421408|2020-03	....	....	....	....	....	....	
A	8021	hCoV-19/USA/NY-PV08463/2020|EPI_ISL_421409|2020-03	....	....	....	....	....	....	
A	8022	hCoV-19/USA/NY-PV08464/2020|EPI_ISL_421410|2020-03	....	....	....	....	....	....	
A	8023	hCoV-19/USA/NY-PV08466/2020|EPI_ISL_421411|2020-03	....	....	....	....	....	....	
A	8024	hCoV-19/USA/NY-PV08468/2020|EPI_ISL_421412|2020-03	....	......A.............	....	....	....	....	
A	8025	hCoV-19/USA/NY-PV08469/2020|EPI_ISL_421413|2020-03	....	....	....	....	....	....	
A	8026	hCoV-19/USA/NY-PV08471/2020|EPI_ISL_421414|2020-03	....	....	....	....	....	....	
A	8027	hCoV-19/USA/NY-PV08472/2020|EPI_ISL_421415|2020-03	....	....	....	....	....	....	
A	8028	hCoV-19/USA/NY-PV08473/2020|EPI_ISL_421416|2020-03	....	....	....	....	....	....	
A	8029	hCoV-19/USA/NY-PV08474/2020|EPI_ISL_421417|2020-03	....	....	....	....	....	....	
A	8030	hCoV-19/USA/NY-PV08477/2020|EPI_ISL_421419|2020-03	....	....	....	....	....	....	
A	8031	hCoV-19/USA/NY-PV08478/2020|EPI_ISL_421420|2020-03	....	...................A	....	....	....	....	1
A	8032	hCoV-19/USA/NY-PV08481/2020|EPI_ISL_421421|2020-03	....	....	....	....	....	....	
A	8033	hCoV-19/USA/NY-PV08485/2020|EPI_ISL_421422|2020-03	....	....	....	....	....	....	
A	8034	hCoV-19/USA/NY-PV08486/2020|EPI_ISL_421423|2020-03	....	....	....	....	....	....	
A	8035	hCoV-19/USA/NY-PV08487/2020|EPI_ISL_421424|2020-03	....	....	....	....	....	....	
A	8036	hCoV-19/USA/NY-PV08489/2020|EPI_ISL_421425|2020-03	....	....	....	....	....	....	
A	8037	hCoV-19/USA/NY-PV08490/2020|EPI_ISL_421426|2020-03	....	....	....	....	....	....	
A	8038	hCoV-19/USA/NY-PV08491/2020|EPI_ISL_421427|2020-03	....	....	....	....	....	....	
A	8039	hCoV-19/USA/NY-PV08492/2020|EPI_ISL_421428|2020-03	....	....	....	....	....	....	
A	8040	hCoV-19/USA/NY-PV08493/2020|EPI_ISL_421429|2020-03	....	....	....	....	....	....	
A	8041	hCoV-19/USA/NY-PV08494/2020|EPI_ISL_421430|2020-03	....	....	....	....	....	....	
A	8042	hCoV-19/USA/NY-PV08495/2020|EPI_ISL_421431|2020-03	....	....	....	....	....	....	
A	8043	hCoV-19/USA/NY-PV08496/2020|EPI_ISL_421432|2020-03	....	....	....	....	....	....	
A	8044	hCoV-19/USA/NY-PV08498/2020|EPI_ISL_421433|2020-03	....	....	....	....	....	....	
A	8045	hCoV-19/USA/NY-PV08500/2020|EPI_ISL_421434|2020-03	....	....	....	....	....	....	
A	8046	hCoV-19/USA/NY-PV09000/2020|EPI_ISL_421435|2020-03	....	....	....	....	....	....	
A	8047	hCoV-19/Portugal/PT0043/2020|EPI_ISL_421446|2020-0	....	....	....	....	....	....	
A	8048	hCoV-19/Portugal/PT0044/2020|EPI_ISL_421447|2020-0	....	....	....	....	....	....	
A	8049	hCoV-19/Portugal/PT0045/2020|EPI_ISL_421448|2020-0	....	....	....	....	....	....	
A	8050	hCoV-19/Portugal/PT0046/2020|EPI_ISL_421449|2020-0	....	....	....	....	....	....	
A	8051	hCoV-19/Portugal/PT0047/2020|EPI_ISL_421450|2020-0	....	....	....	....	....	....	
A	8052	hCoV-19/Portugal/PT0048/2020|EPI_ISL_421451|2020-0	....	....	....	....	....	....	
A	8053	hCoV-19/Portugal/PT0049/2020|EPI_ISL_421452|2020-0	....	....	....	....	....	....	
A	8054	hCoV-19/Portugal/PT0050/2020|EPI_ISL_421453|2020-0	....	....	....	....	....	....	

A	8055	hCoV-19/Portugal/PT0051/2020|EPI_ISL_421454|2020-0	....	....	....	....	....	....
A	8056	hCoV-19/Portugal/PT0052/2020|EPI_ISL_421455|2020-0	....	....	....	....	....	....
A	8057	hCoV-19/Portugal/PT0053/2020|EPI_ISL_421456|2020-0	....	....	....	....	....	....
A	8058	hCoV-19/Portugal/PT0054/2020|EPI_ISL_421457|2020-0	....	....	....	....	....	....
A	8059	hCoV-19/Portugal/PT0055/2020|EPI_ISL_421458|2020-0	....	....	....	....	....	....
A	8060	hCoV-19/Portugal/PT0056/2020|EPI_ISL_421459|2020-0	....	....	....	....	....	....
A	8061	hCoV-19/Portugal/PT0057/2020|EPI_ISL_421460|2020-0	....	.A..................	....	....	....	....
A	8062	hCoV-19/Portugal/PT0058/2020|EPI_ISL_421461|2020-0	....	....	....	....	....	....
A	8063	hCoV-19/Portugal/PT0059/2020|EPI_ISL_421462|2020-0	....	....	....	....	....	....
A	8064	hCoV-19/Portugal/PT0060/2020|EPI_ISL_421463|2020-0	....	....	....	....	....	....
A	8065	hCoV-19/Portugal/PT0061/2020|EPI_ISL_421464|2020-0	....	....	....	....	....	....
A	8066	hCoV-19/Portugal/PT0062/2020|EPI_ISL_421465|2020-0	....	....	....	....	....	....
A	8067	hCoV-19/Portugal/PT0063/2020|EPI_ISL_421466|2020-0	....	....	....	....	....	....
A	8068	hCoV-19/Netherlands/NA_165/2020|EPI_ISL_422700|202	....	....	....	....	....	....
A	8069	hCoV-19/Netherlands/NA_166/2020|EPI_ISL_422701|202	....	....	....	....	....	....
A	8070	hCoV-19/Netherlands/NA_167/2020|EPI_ISL_422702|202	....	....	....	....	....	....
A	8071	hCoV-19/Netherlands/NA_168/2020|EPI_ISL_422703|202	....	....	....	....	....	....
A	8072	hCoV-19/Netherlands/NA_169/2020|EPI_ISL_422704|202	....	....	....	....	...T................	....
A	8073	hCoV-19/Netherlands/NA_170/2020|EPI_ISL_422705|202	....	....	....	....	...T................	....
A	8074	hCoV-19/Netherlands/NA_171/2020|EPI_ISL_422706|202	....	....	....	....	....	....
A	8075	hCoV-19/Netherlands/NA_172/2020|EPI_ISL_422707|202	....	....	....	....	....	....
A	8076	hCoV-19/Netherlands/NA_173/2020|EPI_ISL_422708|202	....	....	....	....	....	....
A	8077	hCoV-19/Netherlands/NA_174/2020|EPI_ISL_422709|202	....	....	....	....	....	....
A	8078	hCoV-19/Netherlands/NA_176/2020|EPI_ISL_422711|202	....	....	....	....	....	....
A	8079	hCoV-19/Netherlands/NA_177/2020|EPI_ISL_422712|202	....	....	....	....	....	....
A	8080	hCoV-19/Netherlands/NA_179/2020|EPI_ISL_422714|202	....	....	....	....	....	....
A	8081	hCoV-19/Netherlands/NA_180/2020|EPI_ISL_422715|202	....	....	....	....	....	....
A	8082	hCoV-19/Netherlands/NA_181/2020|EPI_ISL_422716|202	....	....	....	....	....	....
A	8083	hCoV-19/Netherlands/NA_182/2020|EPI_ISL_422717|202	....	....	....	....	....	....
A	8084	hCoV-19/Netherlands/NA_183/2020|EPI_ISL_422718|202	....	....	....	....	....	....
A	8085	hCoV-19/Netherlands/NA_184/2020|EPI_ISL_422719|202	....	....	....	....	....	....
A	8086	hCoV-19/Netherlands/NA_185/2020|EPI_ISL_422720|202	....	....	....	....	....	....
A	8087	hCoV-19/Netherlands/NA_186/2020|EPI_ISL_422721|202	....	....	....	....	....	....
A	8088	hCoV-19/Netherlands/NA_187/2020|EPI_ISL_422722|202	....	....	....	....	....	....
A	8089	hCoV-19/Netherlands/NA_189/2020|EPI_ISL_422724|202	....	....	....	....	....	....
A	8090	hCoV-19/Netherlands/NA_190/2020|EPI_ISL_422725|202	....	....	....	....	....	....
A	8091	hCoV-19/Netherlands/NA_191/2020|EPI_ISL_422726|202	....	....	....	....	....	....
A	8092	hCoV-19/Netherlands/NA_192/2020|EPI_ISL_422727|202	....	....	....	....	....	....
A	8093	hCoV-19/Netherlands/NA_193/2020|EPI_ISL_422728|202	....	....	....	....	....	....
A	8094	hCoV-19/Netherlands/NA_194/2020|EPI_ISL_422729|202	....	....	....	....	....	....
A	8095	hCoV-19/Netherlands/NA_195/2020|EPI_ISL_422730|202	....	....	....	....	....	....
A	8096	hCoV-19/Netherlands/NA_197/2020|EPI_ISL_422732|202	....	....	....	....	....	....
A	8097	hCoV-19/Netherlands/NA_198/2020|EPI_ISL_422733|202	....	....	....	....	....	....
A	8098	hCoV-19/Netherlands/NA_199/2020|EPI_ISL_422734|202	....	....	....	....	....	....
A	8099	hCoV-19/Netherlands/NA_200/2020|EPI_ISL_422735|202	....	....	....	....	....	....
A	8100	hCoV-19/Netherlands/NA_201/2020|EPI_ISL_422736|202	....	....	....	....	....	....
A	8101	hCoV-19/Netherlands/NA_202/2020|EPI_ISL_422737|202	....	....	....	....	....	....
A	8102	hCoV-19/Netherlands/NA_203/2020|EPI_ISL_422738|202	....	....	....	....	....	....
A	8103	hCoV-19/Netherlands/NA_206/2020|EPI_ISL_422741|202	....	....	....	....	....	....
A	8104	hCoV-19/Netherlands/NA_207/2020|EPI_ISL_422742|202	....	....	....	....	....	....
A	8105	hCoV-19/Netherlands/NA_209/2020|EPI_ISL_422744|202	....	....	....	....	....	....
A	8106	hCoV-19/Netherlands/NA_210/2020|EPI_ISL_422745|202	....	....	....	....	....	....
A	8107	hCoV-19/Netherlands/NA_211/2020|EPI_ISL_422746|202	....	....	....	....	....	....
A	8108	hCoV-19/Netherlands/NA_212/2020|EPI_ISL_422747|202	....	....	....	....	....	....
A	8109	hCoV-19/Netherlands/NA_213/2020|EPI_ISL_422748|202	....	....	....	....	....	....
A	8110	hCoV-19/Netherlands/NA_214/2020|EPI_ISL_422749|202	....	....	....	....	....	....
A	8111	hCoV-19/Netherlands/NA_215/2020|EPI_ISL_422750|202	....	....	....	....	....	....
A	8112	hCoV-19/Netherlands/NA_216/2020|EPI_ISL_422751|202	....	....	....	....	....	....
A	8113	hCoV-19/Netherlands/NA_217/2020|EPI_ISL_422752|202	....	....	....	....	....	....
A	8114	hCoV-19/Netherlands/NA_218/2020|EPI_ISL_422753|202	....	.A..................	....	....	....	....
A	8115	hCoV-19/Netherlands/NA_219/2020|EPI_ISL_422754|202	....	....	....	....	....	....
A	8116	hCoV-19/Netherlands/NA_220/2020|EPI_ISL_422755|202	....	....	....	....	....	....
A	8117	hCoV-19/Netherlands/NA_221/2020|EPI_ISL_422756|202	....	....	....	....	....	....
A	8118	hCoV-19/Netherlands/NA_223/2020|EPI_ISL_422758|202	....	....	....	....	....	....
A	8119	hCoV-19/Netherlands/NA_224/2020|EPI_ISL_422759|202	....	....	....	....	....	....
A	8120	hCoV-19/Netherlands/NA_225/2020|EPI_ISL_422760|202	....	....	....	....	....	....
A	8121	hCoV-19/Netherlands/NA_226/2020|EPI_ISL_422761|202	....	....	....	....	....	....
A	8122	hCoV-19/Netherlands/NA_227/2020|EPI_ISL_422762|202	....	....	....	....	....	....
A	8123	hCoV-19/Netherlands/NA_229/2020|EPI_ISL_422764|202	....	....	....	....	....	....
A	8124	hCoV-19/Netherlands/NA_230/2020|EPI_ISL_422765|202	....	....	....	....	....	....
A	8125	hCoV-19/Netherlands/NA_231/2020|EPI_ISL_422766|202	....	....	....	....	....	....
A	8126	hCoV-19/Netherlands/NA_233/2020|EPI_ISL_422767|202	....	....	....	....	....	....
A	8127	hCoV-19/Netherlands/NA_234/2020|EPI_ISL_422768|202	....	....	....	....	....	....
A	8128	hCoV-19/Netherlands/NA_235/2020|EPI_ISL_422769|202	....	....	....	....	....	....
A	8129	hCoV-19/Netherlands/NA_236/2020|EPI_ISL_422770|202	....	....	....	....	....	....
A	8130	hCoV-19/Netherlands/NA_237/2020|EPI_ISL_422771|202	....	....	....	....	....	....
A	8131	hCoV-19/Netherlands/NA_238/2020|EPI_ISL_422772|202	....	....	....	....	....	....
A	8132	hCoV-19/Netherlands/NA_240/2020|EPI_ISL_422774|202	....	....	....	....	....	....

A	8133	hCoV-19/Netherlands/NA_242/2020|EPI_ISL_422776|202	····················	····················	····················	····················	····················	····················
A	8134	hCoV-19/Netherlands/NA_243/2020|EPI_ISL_422777|202	····················	····················	····················	····················	····················	····················
A	8135	hCoV-19/Netherlands/NA_244/2020|EPI_ISL_422778|202	····················	····················	····················	····················	····················	····················
A	8136	hCoV-19/Netherlands/NA_250/2020|EPI_ISL_422784|202	····················	····················	····················	····················	····················	····················
A	8137	hCoV-19/Netherlands/NA_251/2020|EPI_ISL_422785|202	····················	····················	····················	····················	····················	····················
A	8138	hCoV-19/Netherlands/NA_253/2020|EPI_ISL_422787|202	····················	····················	····················	····················	····················	····················
A	8139	hCoV-19/Netherlands/NA_254/2020|EPI_ISL_422788|202	····················	····················	····················	····················	····················	····················
A	8140	hCoV-19/Netherlands/NA_256/2020|EPI_ISL_422790|202	····················	····················	····················	····················	····················	····················
A	8141	hCoV-19/Netherlands/NA_257/2020|EPI_ISL_422791|202	····················	····················	····················	····················	····················	····················
A	8142	hCoV-19/Netherlands/NA_258/2020|EPI_ISL_422792|202	····················	····················	····················	····················	····················	····················
A	8143	hCoV-19/Netherlands/NA_259/2020|EPI_ISL_422793|202	····················	····················	····················	····················	····················	····················
A	8144	hCoV-19/Netherlands/NA_260/2020|EPI_ISL_422794|202	····················	····················	····················	····················	····················	····················
A	8145	hCoV-19/Netherlands/NA_261/2020|EPI_ISL_422795|202	····················	····················	····················	····················	····················	····················
A	8146	hCoV-19/Netherlands/NA_262/2020|EPI_ISL_422796|202	····················	·A··················	····················	····················	····················	····················
A	8147	hCoV-19/Netherlands/NA_36/2020|EPI_ISL_422797|2020	····················	····················	····················	····················	····················	····················
A	8148	hCoV-19/Latvia/05/2020|EPI_ISL_426285|2020-03-30	····················	····················	····················	····················	····················	····················
A	8149	hCoV-19/Latvia/06/2020|EPI_ISL_426286|2020-03-30	····················	····················	····················	····················	····················	····················
A	8150	hCoV-19/Latvia/07/2020|EPI_ISL_426287|2020-03-30	····················	····················	····················	····················	····················	····················
A	8151	hCoV-19/Latvia/08/2020|EPI_ISL_426288|2020-03-30	····················	····················	····················	····················	····················	····················
A	8152	hCoV-19/Latvia/09/2020|EPI_ISL_426289|2020-03-30	····················	····················	····················	····················	····················	····················
A	8153	hCoV-19/USA/NY-WCMP10E06P/2020|EPI_ISL_427570|2020	····················	····················	····················	····················	····················	····················
A	8154	hCoV-19/USA/NY-WCMP10E07P/2020|EPI_ISL_427571|2020	····················	····················	····················	····················	····················	····················
A	8155	hCoV-19/USA/NY-WCMP10F04P/2020|EPI_ISL_427572|2020	····················	····················	····················	····················	····················	····················
A	8156	hCoV-19/USA/NY-WCMP10F06P/2020|EPI_ISL_427573|2020	····················	····················	····················	····················	····················	····················
A	8157	hCoV-19/USA/NY-WCMP10F09P/2020|EPI_ISL_427574|2020	····················	····················	····················	····················	····················	····················
A	8158	hCoV-19/USA/NY-WCMP10F10P/2020|EPI_ISL_427575|2020	····················	····················	····················	····················	····················	····················
A	8159	hCoV-19/USA/NY-WCMP10G05P/2020|EPI_ISL_427576|2020	····················	····················	····················	····················	····················	····················
A	8160	hCoV-19/USA/NY-WCMP10G06P/2020|EPI_ISL_427577|2020	····················	····················	····················	····················	····················	····················
A	8161	hCoV-19/USA/NY-WCMP10G10P/2020|EPI_ISL_427578|2020	····················	····················	····················	····················	····················	····················
A	8162	hCoV-19/USA/NY-WCMP10G11P/2020|EPI_ISL_427579|2020	····················	····················	····················	····················	····················	····················
A	8163	hCoV-19/USA/NY-WCMP10H01P/2020|EPI_ISL_427580|2020	····················	····················	····················	····················	····················	····················
A	8164	hCoV-19/USA/NY-WCMP10H04P/2020|EPI_ISL_427581|2020	····················	····················	····················	····················	····················	····················
A	8165	hCoV-19/USA/NY-WCMP10H08P/2020|EPI_ISL_427582|2020	····················	····················	····················	····················	····················	····················
A	8166	hCoV-19/USA/NY-WCMP11A03P/2020|EPI_ISL_427583|2020	····················	····················	····················	····················	····················	····················
A	8167	hCoV-19/USA/NY-WCMP11A06P/2020|EPI_ISL_427584|2020	····················	····················	····················	····················	····················	····················
A	8168	hCoV-19/USA/NY-WCMP11B03P/2020|EPI_ISL_427585|2020	····················	····················	····················	····················	····················	····················
A	8169	hCoV-19/USA/NY-WCMP11B04P/2020|EPI_ISL_427586|2020	····················	····················	····················	····················	····················	····················
A	8170	hCoV-19/USA/NY-WCMP11B07P/2020|EPI_ISL_427587|2020	····················	····················	····················	····················	····················	····················
A	8171	hCoV-19/USA/NY-WCMP11C03P/2020|EPI_ISL_427588|2020	····················	····················	····················	····················	····················	····················
A	8172	hCoV-19/USA/NY-WCMP11C04P/2020|EPI_ISL_427589|2020	····················	····················	····················	····················	····················	····················
A	8173	hCoV-19/USA/NY-WCMP11C05P/2020|EPI_ISL_427590|2020	····················	····················	····················	····················	····················	····················
A	8174	hCoV-19/USA/NY-WCMP11D01N/2020|EPI_ISL_427591|2020	····················	····················	····················	····················	····················	····················
A	8175	hCoV-19/USA/NY-WCMP11E04P/2020|EPI_ISL_427592|2020	····················	····················	····················	····················	····················	····················
A	8176	hCoV-19/USA/NY-WCMP11E05P/2020|EPI_ISL_427593|2020	····················	····················	····················	····················	····················	····················
A	8177	hCoV-19/USA/NY-WCMP11E09P/2020|EPI_ISL_427594|2020	····················	····················	····················	····················	····················	····················
A	8178	hCoV-19/USA/NY-WCMP11F04P/2020|EPI_ISL_427595|2020	····················	····················	····················	····················	····················	····················
A	8179	hCoV-19/USA/NY-WCMP11F06P/2020|EPI_ISL_427596|2020	····················	····················	····················	····················	····················	····················
A	8180	hCoV-19/USA/NY-WCMP11F07P/2020|EPI_ISL_427597|2020	····················	····················	····················	····················	····················	····················
A	8181	hCoV-19/USA/NY-WCMP11H06P/2020|EPI_ISL_427598|2020	····················	····················	····················	····················	····················	····················
A	8182	hCoV-19/USA/NY-WCMP12B04P/2020|EPI_ISL_427599|2020	····················	····················	····················	····················	····················	····················
A	8183	hCoV-19/Australia/NSW82/2020|EPI_ISL_427805|2020-0	····················	····················	····················	····················	····················	····················
A	8184	hCoV-19/Australia/NSW196/2020|EPI_ISL_427806|2020-	····················	····················	····················	····················	····················	····················
A	8185	hCoV-19/Australia/NSW207/2020|EPI_ISL_427807|2020-	····················	····················	····················	····················	····················	····················
A	8186	hCoV-19/Australia/NSW127/2020|EPI_ISL_427808|2020-	····················	····················	····················	····················	····················	····················
A	8187	hCoV-19/South Korea/KCDC2015/2020|EPI_ISL_427809|2	····················	····················	····················	····················	····················	····················
A	8188	hCoV-19/South Korea/KCDC2016/2020|EPI_ISL_427810|2	····················	····················	····················	····················	····················	····················
A	8189	hCoV-19/South Korea/KCDC2017/2020|EPI_ISL_427811|2	····················	····················	····················	····················	····················	····················
A	8190	hCoV-19/South Korea/KCDC2018/2020|EPI_ISL_427812|2	····················	····················	····················	····················	····················	····················
A	8191	hCoV-19/South Korea/KCDC2019/2020|EPI_ISL_427813|2	····················	····················	····················	····················	····················	····················
A	8192	hCoV-19/Russia/StPetersburg-RII4706S/2020|EPI_ISL_	····················	····················	····················	····················	····················	····················
A	8193	hCoV-19/USA/WA1/2020|EPI_ISL_404895|2020-01-19	····················	····················	····················	····················	····················	····················
A	8194	hCoV-19/Singapore/7/2020|EPI_ISL_410713|2020-01-27	····················	······A·············	····················	····················	····················	····················
A	8195	hCoV-19/Singapore/8/2020|EPI_ISL_410714|2020-02-03	····················	····················	····················	····················	····················	····················
A	8196	hCoV-19/Singapore/9/2020|EPI_ISL_410715|2020-02-04	····················	····················	····················	····················	····················	····················
A	8197	hCoV-19/Singapore/10/2020|EPI_ISL_410716|2020-02-0	····················	····················	····················	····················	····················	····················
A	8198	hCoV-19/Australia/QLD03/2020|EPI_ISL_410717|2020-0	····················	····················	····················	····················	····················	····················
A	8199	hCoV-19/Australia/QLD04/2020|EPI_ISL_410718|2020-0	····················	····················	····················	····················	····················	····················
A	8200	hCoV-19/Netherlands/NoordBrabant_48/2020|EPI_ISL_4	····················	····················	····················	····················	····················	····················
A	8201	hCoV-19/Netherlands/NoordBrabant_49/2020|EPI_ISL_4	····················	····················	····················	····················	····················	····················
A	8202	hCoV-19/Netherlands/NoordBrabant_51/2020|EPI_ISL_4	····················	····················	····················	····················	····················	····················
A	8203	hCoV-19/Netherlands/NoordBrabant_52/2020|EPI_ISL_4	····················	····················	····················	····················	····················	····················
A	8204	hCoV-19/Netherlands/NoordBrabant_53/2020|EPI_ISL_4	····················	····················	····················	····················	····················	····················
A	8205	hCoV-19/Netherlands/NoordBrabant_54/2020|EPI_ISL_4	····················	····················	····················	····················	····················	····················
A	8206	hCoV-19/Netherlands/NoordBrabant_55/2020|EPI_ISL_4	····················	····················	····················	····················	····················	····················
A	8207	hCoV-19/Netherlands/NoordBrabant_56/2020|EPI_ISL_4	····················	····················	····················	····················	····················	····················
A	8208	hCoV-19/Netherlands/NoordBrabant_57/2020|EPI_ISL_4	····················	····················	····················	····················	····················	····················
A	8209	hCoV-19/Netherlands/NoordBrabant_58/2020|EPI_ISL_4	····················	····················	····················	····················	····················	····················
A	8210	hCoV-19/Netherlands/NoordBrabant_59/2020|EPI_ISL_4	····················	····················	····················	····················	····················	····················

A	8211 hCoV-19/Netherlands/NoordBrabant_61/2020|EPI_ISL_4	····················	····················	····················	····················	····················	····················		
A	8212 hCoV-19/Netherlands/NoordBrabant_62/2020|EPI_ISL_4	····················	····················	····················	····················	····················	····················		
A	8213 hCoV-19/Netherlands/NoordBrabant_63/2020|EPI_ISL_4	····················	····················	····················	····················	····················	····················		
A	8214 hCoV-19/Netherlands/NoordBrabant_64/2020|EPI_ISL_4	····················	····················	····················	····················	····················	····················		
A	8215 hCoV-19/Netherlands/NoordBrabant_65/2020|EPI_ISL_4	····················	····················	····················	····················	····················	····················		
A	8216 hCoV-19/Netherlands/NoordBrabant_66/2020|EPI_ISL_4	····················	····················	····················	····················	····················	····················		
A	8217 hCoV-19/Netherlands/NoordBrabant_67/2020|EPI_ISL_4	····················	····················	····················	····················	····················	····················		
A	8218 hCoV-19/Netherlands/NoordBrabant_68/2020|EPI_ISL_4	····················	····················	····················	····················	····················	····················		
A	8219 hCoV-19/Netherlands/NoordHolland_3/2020|EPI_ISL_41	····················	····················	····················	····················	····················	····················		
A	8220 hCoV-19/Netherlands/Utrecht_17/2020|EPI_ISL_415526	····················	····················	····················	····················	····················	····················		
A	8221 hCoV-19/Netherlands/Utrecht_18/2020|EPI_ISL_415527	····················	····················	····················	····················	····················	····················		
A	8222 hCoV-19/Netherlands/ZuidHolland_25/2020|EPI_ISL_41	····················	····················	····················	····················	····················	····················		
A	8223 hCoV-19/Netherlands/ZuidHolland_26/2020|EPI_ISL_41	····················	····················	····················	····················	····················	····················		
A	8224 hCoV-19/Netherlands/ZuidHolland_27/2020|EPI_ISL_41	····················	····················	····················	····················	····················	····················		
A	8225 hCoV-19/Netherlands/ZuidHolland_28/2020|EPI_ISL_41	····················	····················	····················	····················	····················	····················		
A	8226 hCoV-19/Netherlands/ZuidHolland_29/2020|EPI_ISL_41	····················	····················	····················	····················	····················	····················		
A	8227 hCoV-19/Netherlands/ZuidHolland_30/2020|EPI_ISL_41	····················	····················	····················	····················	····················	····················		
A	8228 hCoV-19/Netherlands/ZuidHolland_31/2020|EPI_ISL_41	····················	····················	····················	····················	····················	····················		
A	8229 hCoV-19/USA/UT-01/2020|EPI_ISL_415539|2020-03-13	····················	····················	····················	····················	····················	····················		
A	8230 hCoV-19/USA/UT-03/2020|EPI_ISL_415541|2020-03-13	····················	····················	····················	····················	····················	····················		
A	8231 hCoV-19/USA/UT-04/2020|EPI_ISL_415542|2020-03-13	····················	····················	····················	····················	····················	····················		
A	8232 hCoV-19/USA/UT-05/2020|EPI_ISL_415543|2020-03-13	····················	····················	····················	····················	····················	····················		
A	8233 hCoV-19/USA/UT-06/2020|EPI_ISL_415544|2020-03-13	····················	····················	····················	····················	····················	····················		
A	8234 hCoV-19/Canada/BC_13297/2020|EPI_ISL_415578|2020-0	····················	····················	····················	····················	····················	····················		
A	8235 hCoV-19/Canada/BC_17397/2020|EPI_ISL_415580|2020-0	····················	····················	····················	····················	····················	····················		
A	8236 hCoV-19/Canada/BC_02421/2020|EPI_ISL_415581|2020-0	····················	····················	····················	····················	····················	····················		
A	8237 hCoV-19/Canada/BC_35720/2020|EPI_ISL_415582|2020-0	····················	····················	····················	····················	····················	····················		
A	8238 hCoV-19/Canada/BC_40860/2020|EPI_ISL_415583|2020-0	····················	····················	····················	····················	····················	····················		
A	8239 hCoV-19/Canada/BC_41851/2020|EPI_ISL_415584|2020-0	····················	····················	····················	····················	····················	····················		
A	8242 hCoV-19/Canada/BC_83109/2020|EPI_ISL_415588|2020-0	····················	····················	····················	····················	·············N···N	NN·N················	1	1
A	8244 hCoV-19/Malaysia/MKAK-CL-2020-5047/2020|EPI_ISL_41	····················	····················	····················	····················	····················	····················		
A	8245 hCoV-19/Malaysia/MKAK-CL-2020-5096/2020|EPI_ISL_41	····················	····················	····················	····················	····················	····················		
A	8246 hCoV-19/Malaysia/MKAK-CL-2020-6430/2020|EPI_ISL_41	····················	····················	····················	····················	····················	····················		
A	8247 hCoV-19/USA/VA-DCLS-0033/2020|EPI_ISL_420019|2020-	····················	····················	····················	····················	····················	····················		
A	8248 hCoV-19/USA/VA-DCLS-0036/2020|EPI_ISL_420020|2020-	····················	····················	····················	····················	····················	····················		
A	8249 hCoV-19/USA/VA-DCLS-0039/2020|EPI_ISL_420021|2020-	····················	····················	····················	····················	····················	····················		
A	8250 hCoV-19/USA/VA-DCLS-0040/2020|EPI_ISL_420022|2020-	····················	····················	····················	····················	····················	····················		
A	8251 hCoV-19/USA/VA-DCLS-0042/2020|EPI_ISL_420023|2020-	····················	····················	····················	····················	····················	····················		
A	8252 hCoV-19/USA/VA-DCLS-0043/2020|EPI_ISL_420024|2020-	····················	····················	····················	····················	····················	····················		
A	8253 hCoV-19/USA/VA-DCLS-0044/2020|EPI_ISL_420025|2020-	····················	····················	····················	····················	····················	····················		
A	8254 hCoV-19/USA/VA-DCLS-0045/2020|EPI_ISL_420026|2020-	····················	····················	····················	····················	····················	····················		
A	8255 hCoV-19/USA/VA-DCLS-0046/2020|EPI_ISL_420027|2020-	····················	····················	····················	····················	····················	····················		
A	8256 hCoV-19/USA/VA-DCLS-0018/2020|EPI_ISL_420028|2020-	····················	····················	····················	····················	····················	····················		
A	8257 hCoV-19/USA/VA-DCLS-0019/2020|EPI_ISL_420029|2020-	····················	····················	····················	····················	····················	····················		
A	8258 hCoV-19/DRC/214/2020|EPI_ISL_420030|2020-03-21	····················	····················	····················	····················	····················	····················		
A	8259 hCoV-19/DRC/215/2020|EPI_ISL_420031|2020-03-21	····················	····················	····················	····················	····················	····················		
A	8260 hCoV-19/DRC/236/2020|EPI_ISL_420032|2020-03-22	····················	····················	····················	····················	····················	····················		
A	8261 hCoV-19/DRC/241/2020|EPI_ISL_420033|2020-03-22	····················	····················	····················	····················	····················	····················		
A	8262 hCoV-19/DRC/243/2020|EPI_ISL_420034|2020-03-22	····················	····················	····················	····················	····················	····················		
A	8263 hCoV-19/DRC/248/2020|EPI_ISL_420035|2020-03-22	····················	····················	····················	····················	····················	····················		
A	8264 hCoV-19/Australia/VIC256/2020|EPI_ISL_420036|2020-	····················	····················	····················	····················	····················	····················		
A	8265 hCoV-19/USA/NY-PV08121/2020|EPI_ISL_421350|2020-03	····················	····················	····················	····················	····················	····················		
A	8266 hCoV-19/USA/NY-PV08122/2020|EPI_ISL_421351|2020-03	····················	····················	····················	····················	····················	····················		
A	8267 hCoV-19/USA/NY-PV08123/2020|EPI_ISL_421352|2020-03	····················	····················	····················	····················	····················	····················		
A	8268 hCoV-19/USA/NY-PV08124/2020|EPI_ISL_421353|2020-03	····················	····················	····················	····················	····················	····················		
A	8269 hCoV-19/USA/NY-PV08125/2020|EPI_ISL_421354|2020-03	····················	····················	····················	····················	····················	····················		
A	8270 hCoV-19/USA/NY-PV08127/2020|EPI_ISL_421355|2020-03	····················	····················	····················	····················	····················	····················		
A	8271 hCoV-19/USA/NY-PV08135/2020|EPI_ISL_421357|2020-03	····················	·············A······	····················	····················	····················	····················		
A	8272 hCoV-19/USA/NY-PV08136/2020|EPI_ISL_421358|2020-03	····················	·············A······	····················	····················	····················	····················		
A	8273 hCoV-19/USA/NY-PV08137/2020|EPI_ISL_421359|2020-03	····················	·············A······	····················	····················	····················	····················		
A	8274 hCoV-19/USA/NY-PV08138/2020|EPI_ISL_421360|2020-03	····················	····················	····················	····················	····················	····················		
A	8275 hCoV-19/USA/NY-PV08143/2020|EPI_ISL_421363|2020-03	····················	····················	····················	····················	····················	····················		
A	8276 hCoV-19/USA/NY-PV08146/2020|EPI_ISL_421364|2020-03	····················	····················	····················	····················	····················	····················		
A	8277 hCoV-19/USA/NY-PV08148/2020|EPI_ISL_421365|2020-03	····················	····················	····················	····················	····················	····················		
A	8278 hCoV-19/USA/NY-PV08149/2020|EPI_ISL_421366|2020-03	····················	····················	····················	····················	····················	····················		
A	8279 hCoV-19/USA/NY-PV08150/2020|EPI_ISL_421367|2020-03	····················	····················	····················	····················	····················	····················		
A	8280 hCoV-19/Netherlands/NA_136/2020|EPI_ISL_422680|202	····················	····················	····················	····················	····················	····················		
A	8281 hCoV-19/Netherlands/NA_137/2020|EPI_ISL_422681|202	····················	····················	····················	····················	····················	····················		
A	8282 hCoV-19/Netherlands/NA_138/2020|EPI_ISL_422682|202	····················	····················	····················	····················	····················	····················		
A	8283 hCoV-19/Netherlands/NA_144/2020|EPI_ISL_422684|202	····················	····················	····················	····················	····················	····················		
A	8284 hCoV-19/Netherlands/NA_145/2020|EPI_ISL_422685|202	····················	·············A······	····················	····················	····················	····················		
A	8285 hCoV-19/Netherlands/NA_150/2020|EPI_ISL_422686|202	····················	····················	····················	····················	····················	····················		
A	8286 hCoV-19/Netherlands/NA_152/2020|EPI_ISL_422687|202	····················	····················	····················	····················	····················	····················		
A	8287 hCoV-19/Netherlands/NA_153/2020|EPI_ISL_422688|202	····················	····················	····················	····················	····················	····················		
A	8288 hCoV-19/Netherlands/NA_154/2020|EPI_ISL_422689|202	····················	····················	····················	····················	····················	····················		
A	8289 hCoV-19/Netherlands/NA_155/2020|EPI_ISL_422690|202	····················	····················	····················	····················	····················	····················		
A	8290 hCoV-19/Netherlands/NA_156/2020|EPI_ISL_422691|202	····················	····················	····················	····················	····················	····················		
A	8291 hCoV-19/Netherlands/NA_157/2020|EPI_ISL_422692|202	····················	····················	····················	····················	····················	····················		

A 8292 hCoV-19/Netherlands/NA_158/2020|EPI_ISL_422693|202 ....
A 8293 hCoV-19/Netherlands/NA_159/2020|EPI_ISL_422694|202 ....
A 8294 hCoV-19/Netherlands/NA_160/2020|EPI_ISL_422695|202 ....
A 8295 hCoV-19/Netherlands/NA_161/2020|EPI_ISL_422696|202 ....
A 8296 hCoV-19/Netherlands/NA_162/2020|EPI_ISL_422697|202 ....
A 8297 hCoV-19/Netherlands/NA_163/2020|EPI_ISL_422698|202 ....
A 8298 hCoV-19/USA/NY-Wadsworth-11735-01/2020|EPI_ISL_426 ....
A 8299 hCoV-19/USA/NY-Wadsworth-11717-01/2020|EPI_ISL_426 ....
A 8300 hCoV-19/USA/NY-Wadsworth-11207-01/2020|EPI_ISL_426 ....
A 8301 hCoV-19/USA/NY-Wadsworth-12883-02/2020|EPI_ISL_426 ....
A 8302 hCoV-19/USA/NY-Wadsworth-13468-02/2020|EPI_ISL_426 ....
A 8303 hCoV-19/USA/NY-Wadsworth-27073-01/2020|EPI_ISL_426 ....
A 8304 hCoV-19/USA/NY-Wadsworth-16080-01/2020|EPI_ISL_426 ....
A 8305 hCoV-19/USA/NY-Wadsworth-27804-01/2020|EPI_ISL_426 ....
A 8306 hCoV-19/USA/NY-Wadsworth-31488-01/2020|EPI_ISL_426 ....
A 8307 hCoV-19/USA/NY-Wadsworth-32851-01/2020|EPI_ISL_426 ....
A 8308 hCoV-19/USA/NY-NYUMC207/2020|EPI_ISL_427502|2020-0 ....
A 8309 hCoV-19/USA/NY-NYUMC208/2020|EPI_ISL_427503|2020-0 ....
A 8310 hCoV-19/USA/NY-NYUMC209/2020|EPI_ISL_427504|2020-0 ....
A 8311 hCoV-19/USA/NY-NYUMC210/2020|EPI_ISL_427505|2020-0 ....
A 8312 hCoV-19/USA/NY-NYUMC212/2020|EPI_ISL_427507|2020-0 ....
A 8313 hCoV-19/USA/NY-NYUMC213/2020|EPI_ISL_427508|2020-0 ....
A 8314 hCoV-19/USA/NY-NYUMC214/2020|EPI_ISL_427509|2020-0 ....
A 8315 hCoV-19/USA/NY-NYUMC215/2020|EPI_ISL_427510|2020-0 ....
A 8316 hCoV-19/USA/NY-NYUMC216/2020|EPI_ISL_427511|2020-0 ....
A 8317 hCoV-19/USA/NY-NYUMC217/2020|EPI_ISL_427512|2020-0 ....
A 8318 hCoV-19/USA/NY-NYUMC218/2020|EPI_ISL_427513|2020-0 ....
A 8319 hCoV-19/USA/NY-NYUMC219/2020|EPI_ISL_427514|2020-0 ....
A 8320 hCoV-19/USA/NY-NYUMC220/2020|EPI_ISL_427515|2020-0 ....
A 8321 hCoV-19/USA/NY-NYUMC221/2020|EPI_ISL_427516|2020-0 ....
A 8322 hCoV-19/USA/NY-NYUMC223/2020|EPI_ISL_427518|2020-0 ....
A 8323 hCoV-19/USA/NY-NYUMC224/2020|EPI_ISL_427519|2020-0 ....
A 8324 hCoV-19/USA/NY-NYUMC225/2020|EPI_ISL_427520|2020-0 ....
A 8325 hCoV-19/USA/NY-NYUMC226/2020|EPI_ISL_427521|2020-0 ....
A 8326 hCoV-19/USA/NY-NYUMC176/2020|EPI_ISL_427524|2020-0 ....
A 8327 hCoV-19/USA/NY-WCMP2C03P/2020|EPI_ISL_427526|2020- ....
A 8328 hCoV-19/USA/NY-WCMP2D02P/2020|EPI_ISL_427527|2020- ....
A 8329 hCoV-19/USA/NY-WCMP2D04P/2020|EPI_ISL_427528|2020- ....
A 8330 hCoV-19/USA/NY-WCMP2H03P/2020|EPI_ISL_427529|2020- ....
A 8331 hCoV-19/USA/NY-WCMP3E02P/2020|EPI_ISL_427530|2020- ....
A 8332 hCoV-19/USA/NY-WCMP4A02N/2020|EPI_ISL_427531|2020- ....
A 8333 hCoV-19/USA/NY-WCMP4B06P/2020|EPI_ISL_427532|2020- ....
A 8334 hCoV-19/USA/NY-WCMP4C01P/2020|EPI_ISL_427533|2020- ....
A 8335 hCoV-19/USA/NY-WCMP4C02N/2020|EPI_ISL_427534|2020- ....
A 8336 hCoV-19/USA/NY-WCMP4D01P/2020|EPI_ISL_427535|2020- ....
A 8337 hCoV-19/USA/NY-WCMP4D04P/2020|EPI_ISL_427536|2020- ....
A 8338 hCoV-19/USA/NY-WCMP4E05P/2020|EPI_ISL_427537|2020- ....
A 8339 hCoV-19/USA/NY-WCMP4G04P/2020|EPI_ISL_427538|2020- ....
A 8340 hCoV-19/USA/NY-WCMP4H03P/2020|EPI_ISL_427539|2020- ....
A 8341 hCoV-19/USA/NY-WCMP5F01P/2020|EPI_ISL_427540|2020- ....
A 8342 hCoV-19/USA/NY-WCMP5G03P/2020|EPI_ISL_427541|2020- ....
A 8343 hCoV-19/USA/NY-WCMP5H02P/2020|EPI_ISL_427542|2020- ....
A 8344 hCoV-19/USA/NY-WCMP5H03P/2020|EPI_ISL_427543|2020- ....
A 8345 hCoV-19/USA/NY-WCMP9A05P/2020|EPI_ISL_427544|2020- ....
A 8346 hCoV-19/USA/NY-WCMP9A08P/2020|EPI_ISL_427545|2020- ....
A 8347 hCoV-19/USA/NY-WCMP9B05P/2020|EPI_ISL_427546|2020- ....
A 8348 hCoV-19/USA/NY-WCMP9B06P/2020|EPI_ISL_427547|2020- ....
A 8349 hCoV-19/USA/NY-WCMP9B08P/2020|EPI_ISL_427548|2020- ....
A 8350 hCoV-19/USA/NY-WCMP9B09P/2020|EPI_ISL_427549|2020- ....
A 8351 hCoV-19/USA/NY-WCMP9D03P/2020|EPI_ISL_427550|2020- ....
A 8352 hCoV-19/USA/NY-WCMP9E04P/2020|EPI_ISL_427551|2020- ....
A 8353 hCoV-19/USA/NY-WCMP9G04P/2020|EPI_ISL_427552|2020- ....
A 8354 hCoV-19/USA/NY-WCMP10A02P/2020|EPI_ISL_427553|2020 ....
A 8355 hCoV-19/USA/NY-WCMP10A05P/2020|EPI_ISL_427554|2020 ....
A 8356 hCoV-19/USA/NY-WCMP10A06P/2020|EPI_ISL_427555|2020 ....
A 8357 hCoV-19/USA/NY-WCMP10A12P/2020|EPI_ISL_427556|2020 ....
A 8358 hCoV-19/USA/NY-WCMP10B03P/2020|EPI_ISL_427557|2020 ....
A 8359 hCoV-19/USA/NY-WCMP10B07N/2020|EPI_ISL_427558|2020 ....
A 8360 hCoV-19/USA/NY-WCMP10B08P/2020|EPI_ISL_427559|2020 ....
A 8361 hCoV-19/USA/NY-WCMP10B11P/2020|EPI_ISL_427560|2020 ....
A 8362 hCoV-19/USA/NY-WCMP10B12P/2020|EPI_ISL_427561|2020 ....
A 8363 hCoV-19/USA/NY-WCMP10C03P/2020|EPI_ISL_427562|2020 ....
A 8364 hCoV-19/USA/NY-WCMP10C04P/2020|EPI_ISL_427563|2020 ....
A 8365 hCoV-19/USA/NY-WCMP10C09P/2020|EPI_ISL_427564|2020 ....
A 8366 hCoV-19/USA/NY-WCMP10C11P/2020|EPI_ISL_427565|2020 ....
A 8367 hCoV-19/USA/NY-WCMP10D06P/2020|EPI_ISL_427566|2020 ....
A 8368 hCoV-19/USA/NY-WCMP10D09P/2020|EPI_ISL_427567|2020 ....
A 8369 hCoV-19/USA/NY-WCMP10D11P/2020|EPI_ISL_427568|2020 ....

A 8370 hCoV-19/USA/NY-WCMP10E02P/2020|EPI_ISL_427569|2020 ···················· ···················· ····················· ···················· ···················· ····················
A 8371 hCoV-19/Singapore/43/2020|EPI_ISL_428822|2020-02-1 ···················· ···················· ····················· ···················· ···················· ····················
A 8372 hCoV-19/Singapore/44/2020|EPI_ISL_428823|2020-03-0 ···················· ···················· ····················· ···················· ···················· ····················
A 8373 hCoV-19/Singapore/45/2020|EPI_ISL_428824|2020-03-1 ···················· ···················· ····················· ···················· ···················· ····················
A 8374 hCoV-19/Singapore/46/2020|EPI_ISL_428825|2020-03-1 ···················· ···················· ····················· ···················· ···················· ····················
A 8375 hCoV-19/Singapore/47/2020|EPI_ISL_428826|2020-03-1 ···················· ···················· ····················· ···················· ···················· ····················
A 8376 hCoV-19/Singapore/48/2020|EPI_ISL_428827|2020-03-1 ···················· ···················· ····················· ···················· ···················· ····················
A 8377 hCoV-19/Singapore/49/2020|EPI_ISL_428828|2020-03-0 ···················· ···················· ····················· ···················· ···················· ····················
A 8378 hCoV-19/Singapore/50/2020|EPI_ISL_428829|2020-03-0 ···················· ···················· ····················· ···················· ···················· ····················
A 8379 hCoV-19/Singapore/51/2020|EPI_ISL_428830|2020-02-2 ···················· ···················· ····················· ···················· ···················· ····················
A 8380 hCoV-19/Singapore/52/2020|EPI_ISL_428831|2020-03-1 ···················· ···················· ····················· ···················· ···················· ····················
A 8381 hCoV-19/Singapore/53/2020|EPI_ISL_428832|2020-04-0 ···················· ···················· ····················· ···················· ···················· ····················
A 8382 hCoV-19/Singapore/54/2020|EPI_ISL_428833|2020-04-0 ···················· ···················· ····················· ···················· ···················· ····················
A 8383 hCoV-19/Singapore/55/2020|EPI_ISL_428834|2020-04-0 ···················· ···················· ····················· ···················· ···················· ····················
A 8384 hCoV-19/Singapore/56/2020|EPI_ISL_428835|2020-04-0 ···················· ···················· ····················· ···················· ···················· ····················
A 8385 hCoV-19/Singapore/57/2020|EPI_ISL_428836|2020-04-0 ···················· ···················· ····················· ···················· ···················· ····················
A 8386 hCoV-19/Singapore/58/2020|EPI_ISL_428837|2020-04-0 ···················· ···················· ····················· ···················· ···················· ····················
A 8387 hCoV-19/Singapore/59/2020|EPI_ISL_428838|2020-04-0 ···················· ···················· ····················· ···················· ···················· ····················
A 8388 hCoV-19/Singapore/60/2020|EPI_ISL_428839|2020-04-0 ···················· ···················· ····················· ···················· ···················· ····················
A 8389 hCoV-19/Singapore/61/2020|EPI_ISL_428840|2020-04-0 ···················· ···················· ····················· ···················· ···················· ····················
A 8390 hCoV-19/Singapore/62/2020|EPI_ISL_428841|2020-04-0 ···················· ···················· ····················· ···················· ···················· ····················
A 8391 hCoV-19/Singapore/63/2020|EPI_ISL_428842|2020-04-0 ···················· ···················· ····················· ···················· ···················· ····················
A 8392 hCoV-19/Singapore/64/2020|EPI_ISL_428843|2020-04-0 ···················· ···················· ····················· ···················· ···················· ····················
A 8393 hCoV-19/Singapore/65/2020|EPI_ISL_428844|2020-04-0 ···················· ···················· ····················· ···················· ···················· ····················
A 8394 hCoV-19/Singapore/66/2020|EPI_ISL_428845|2020-04-0 ···················· ···················· ····················· ···················· ···················· ····················
A 8395 hCoV-19/Singapore/67/2020|EPI_ISL_428846|2020-04-1 ···················· ···················· ····················· ···················· ···················· ····················
A 8396 hCoV-19/Singapore/68/2020|EPI_ISL_428847|2020-04-1 ···················· ···················· ····················· ···················· ···················· ····················
A 8397 hCoV-19/Singapore/69/2020|EPI_ISL_428848|2020-04-1 ···················· ···················· ····················· ···················· ···················· ····················
A 8398 hCoV-19/Singapore/70/2020|EPI_ISL_428849|2020-04-1 ···················· ···················· ····················· ···················· ···················· ····················
A 8399 hCoV-19/Singapore/71/2020|EPI_ISL_428850|2020-04-1 ···················· ···················· ····················· ···················· ···················· ····················
A 8400 hCoV-19/Russia/Moscow-25I/2020|EPI_ISL_428851|2020 ···················· ···················· ····················· ···················· ···················· ····················
A 8401 hCoV-19/Russia/Moscow-35I/2020|EPI_ISL_428852|2020 ···················· ···················· ····················· ···················· ···················· ····················
A 8402 hCoV-19/Italy/FVG-ICGEB-S18/2020|EPI_ISL_428854|20 ···················· ···················· ····················· ···················· ···················· ····················
A 8403 hCoV-19/Gambia/GC19-015/2020|EPI_ISL_428855|2020-0 ···················· ···················· ····················· ···················· ···················· ····················
A 8404 hCoV-19/Gambia/GC19-026/2020|EPI_ISL_428856|2020-0 ···················· ···················· ····················· ···················· ···················· ····················
A 8405 hCoV-19/Gambia/GC19-029/2020|EPI_ISL_428857|2020-0 ···················· ···················· ····················· ···················· ···················· ····················
A 8406 hCoV-19/Russia/Moscow-62505/2020|EPI_ISL_428860|20 ···················· ···················· ····················· ···················· ···················· ····················
A 8407 hCoV-19/Russia/Moscow-62501/2020|EPI_ISL_428861|20 ···················· ···················· ····················· ···················· ···················· ····················
A 8408 hCoV-19/Russia/Moscow-62506/2020|EPI_ISL_428862|20 ···················· ···················· ····················· ···················· ···················· ····················
A 8409 hCoV-19/Russia/Moscow-62508/2020|EPI_ISL_428863|20 ···················· ···················· ····················· ···················· ···················· ····················
A 8410 hCoV-19/Russia/Moscow-62502/2020|EPI_ISL_428864|20 ···················· ···················· ····················· ···················· ···················· ····················
A 8411 hCoV-19/Russia/Lipetsk-62704/2020|EPI_ISL_428865|2 ···················· ···················· ····················· ···················· ···················· ····················
A 8412 hCoV-19/Russia/Lipetsk-62707/2020|EPI_ISL_428866|2 ···················· ···················· ····················· ···················· ···················· ····················
A 8413 hCoV-19/Russia/Krasnodar-63401/2020|EPI_ISL_428867 ···················· ···················· ····················· ···················· ···················· ····················
A 8414 hCoV-19/Russia/StPetersburg-64304/2020|EPI_ISL_428 ···················· ···················· ····················· ···················· ···················· ····················
A 8415 hCoV-19/Russia/Moscow_64405/2020|EPI_ISL_428869|20 ···················· ···················· ····················· ···················· ···················· ····················
A 8416 hCoV-19/Russia/Moscow-64601/2020|EPI_ISL_428870|20 ···················· ···················· ····················· ···················· ···················· ····················
A 8417 hCoV-19/Russia/Krasnoyarsk-66501/2020|EPI_ISL_4288 ···················· ···················· ····················· ···················· ···················· ····················
A 8418 hCoV-19/Russia/Moscow-71605/2020|EPI_ISL_428872|20 ···················· ···················· ····················· ···················· ···················· ····················
A 8419 hCoV-19/Russia/Sverdlovsk-71904/2020|EPI_ISL_42887 ···················· ···················· ····················· ···················· ···················· ····················
A 8420 hCoV-19/Russia/Yakutia-73709/2020|EPI_ISL_428874|2 ···················· ···················· ····················· ···················· ···················· ····················
A 8421 hCoV-19/Russia/Moscow-67117/2020|EPI_ISL_428875|20 ···················· ···················· ····················· ···················· ···················· ····················
A 8422 hCoV-19/Russia/Moscow-71604/2020|EPI_ISL_428876|20 ···················· ···················· ····················· ···················· ···················· ····················
A 8423 hCoV-19/Russia/Moscow-71602/2020|EPI_ISL_428877|20 ···················· ···················· ····················· ···················· ···················· ····················
A 8424 hCoV-19/Russia/Omsk-89001/2020|EPI_ISL_428878|2020 ···················· ···················· ····················· ···················· ···················· ····················
A 8425 hCoV-19/Russia/Sverdlovsk-71905/2020|EPI_ISL_42887 ···················· ···················· ····················· ···················· ···················· ····················
A 8426 hCoV-19/Russia/Khabarovsk-71403/2020|EPI_ISL_42888 ···················· ···················· ····················· ···················· ···················· ····················
A 8427 hCoV-19/Russia/Moscow-67142/2020|EPI_ISL_428881|20 ···················· ···················· ····················· ···················· ···················· ····················
A 8428 hCoV-19/Russia/Moscow-67609/2020|EPI_ISL_428882|20 ···················· ···················· ····················· ···················· ···················· ····················
A 8429 hCoV-19/Russia/StPetersburg-73603/2020|EPI_ISL_428 ···················· ···················· ····················· ···················· ···················· ····················
A 8430 hCoV-19/Russia/Moscow-77627/2020|EPI_ISL_428884|20 ···················· ···················· ····················· ···················· ···················· ····················
A 8431 hCoV-19/Russia/Samara-73406/2020|EPI_ISL_428885|20 ···················· ···················· ····················· ···················· ···················· ····················
A 8432 hCoV-19/Russia/Samara-73403/2020|EPI_ISL_428886|20 ···················· ···················· ····················· ···················· ···················· ····················
A 8433 hCoV-19/Russia/Moscow-77610/2020|EPI_ISL_428887|20 ···················· ···················· ····················· ···················· ···················· ····················
A 8434 hCoV-19/Russia/Yakutia-73703/2020|EPI_ISL_428888|2 ···················· ···················· ····················· ···················· ···················· ····················
A 8435 hCoV-19/Russia/Moscow-77620/2020|EPI_ISL_428889|20 ···················· ···················· ····················· ···················· ···················· ····················
A 8436 hCoV-19/Russia/Sverdlovsk-71903/2020|EPI_ISL_42889 ···················· ···················· ····················· ···················· ···················· ····················
A 8437 hCoV-19/Russia/Yakutia-83301/2020|EPI_ISL_428891|2 ···················· ···················· ····················· ···················· ···················· ····················
A 8438 hCoV-19/Russia/Krasnodar-80902/2020|EPI_ISL_428892 ···················· ···················· ····················· ···················· ···················· ····················
A 8439 hCoV-19/Russia/Kabardino-Balkaria-80501/2020|EPI_I ···················· ···················· ····················· ···················· ···················· ····················
A 8440 hCoV-19/Russia/Buryatia-84506/2020|EPI_ISL_428894| ···················· ···················· ····················· ···················· ···················· ····················
A 8441 hCoV-19/Russia/Perm-82707/2020|EPI_ISL_428895|2020 ···················· ···················· ····················· ···················· ···················· ····················
A 8442 hCoV-19/Russia/Vector_84801/2020|EPI_ISL_428896|20 ···················· ···················· ····················· ···················· ···················· ····················
A 8443 hCoV-19/Russia/StPetersburg-82005/2020|EPI_ISL_428 ···················· ············A······· ····················· ···················· ···················· ····················
A 8444 hCoV-19/Russia/Kabardino-Balkaria-80503/2020|EPI_I ···················· ···················· ····················· ···················· ···················· ····················
A 8445 hCoV-19/Russia/Buryatia-86204/2020|EPI_ISL_428899| ···················· ···················· ····················· ···················· ···················· ····················
A 8446 hCoV-19/USA/KY-9A06/2020|EPI_ISL_468063|2020-03-18 ···················· ···················· ····················· ···················· ···················· ····················
A 8447 hCoV-19/USA/KY-9A10/2020|EPI_ISL_468064|2020-03-18 ···················· ···················· ····················· ···················· ···················· ····················

A	8448 hCoV-19/USA/KY-9E09/2020|EPI_ISL_468065|2020-03-18	····················	····················	····················	····················	····················	····················	
A	8449 hCoV-19/England/01/2020|EPI_ISL_407071|2020-01-29	····················	····················	····················	····················	····················	····················	
A	8450 hCoV-19/England/02/2020|EPI_ISL_407073|2020-01-29	····················	····················	····················	····················	····················	····················	
A	8451 hCoV-19/Finland/1/2020|EPI_ISL_407079|2020-01-29	····················	····················	····················	····················	····················	····················	
A	8452 hCoV-19/USA/WA1-A12/2020|EPI_ISL_407214|2020-01-25	····················	····················	····················	····················	····················	····················	
A	8453 hCoV-19/USA/WA1-F6/2020|EPI_ISL_407215|2020-01-25	····················	····················	····················	····················	····················	····················	
A	8454 hCoV-19/Netherlands/NoordBrabant_42/2020|EPI_ISL_4	····················	····················	····················	····················	····················	····················	
A	8455 hCoV-19/Netherlands/NoordBrabant_44/2020|EPI_ISL_4	····················	····················	····················	····················	····················	····················	
A	8456 hCoV-19/Netherlands/NoordBrabant_45/2020|EPI_ISL_4	····················	····················	····················	····················	····················	····················	
A	8457 hCoV-19/Netherlands/NoordBrabant_46/2020|EPI_ISL_4	····················	····················	····················	····················	····················	····················	
A	8458 hCoV-19/Netherlands/NoordBrabant_47/2020|EPI_ISL_4	····················	····················	····················	····················	····················	····················	
A	8459 hCoV-19/Malaysia/MKAK-CL-2020-5045/2020|EPI_ISL_41	····················	····················	····················	····················	····················	····················	
A	8460 hCoV-19/USA/NY-NYUMC2/2020|EPI_ISL_416830|2020-03-	····················	····················	····················	····················	····················	····················	
A	8461 hCoV-19/USA/NY-NYUMC3/2020|EPI_ISL_416831|2020-03-	····················	····················	····················	····················	····················	····················	
A	8462 hCoV-19/USA/NY-NYUMC4/2020|EPI_ISL_416832|2020-03-	····················	····················	····················	····················	····················	····················	
A	8463 hCoV-19/Israel/ISR_JP0320/2020|EPI_ISL_419211|2020	····················	····················	····················	····················	····················	····················	
A	8464 hCoV-19/Hong Kong/HKPU39-3001/2020|EPI_ISL_419214|	····················	····················	····················	····················	····················	····················	
A	8465 hCoV-19/Hong Kong/HKPU40-2801/2020|EPI_ISL_419215|	····················	····················	····················	····················	····················	····················	
A	8466 hCoV-19/Hong Kong/HKPU41-0802/2020|EPI_ISL_419216|	····················	····················	····················	····················	····················	····················	
A	8467 hCoV-19/Hong Kong/HKPU42-0302/2020|EPI_ISL_419217|	····················	····················	····················	····················	····················	····················	
A	8468 hCoV-19/Hong Kong/HKPU44-0102/2020|EPI_ISL_419219|	····················	····················	····················	····················	····················	····················	
A	8469 hCoV-19/Hong Kong/HKPU45-0502/2020|EPI_ISL_419221|	····················	····················	····················	····················	····················	····················	
A	8470 hCoV-19/Hong Kong/HKPU48-0202/2020|EPI_ISL_419222|	····················	····················	····················	····················	····················	····················	
A	8471 hCoV-19/Hong Kong/HKPU52-3101/2020|EPI_ISL_419224|	····················	····················	····················	····················	····················	····················	
A	8472 hCoV-19/Hong Kong/HKPU53-0802/2020|EPI_ISL_419225|	····················	····················	····················	····················	····················	····················	
A	8473 hCoV-19/Hong Kong/HKPU54-0302/2020|EPI_ISL_419226|	····················	····················	····················	····················	····················	··················C·	1
A	8474 hCoV-19/Hong Kong/HKPU57-0702/2020|EPI_ISL_419227|	····················	····················	····················	····················	····················	····················	
A	8475 hCoV-19/Hong Kong/HKPU60-0802/2020|EPI_ISL_419228|	····················	····················	····················	····················	····················	····················	
A	8476 hCoV-19/Hong Kong/HKPU61-0202/2020|EPI_ISL_419229|	····················	····················	····················	····················	····················	····················	
A	8477 hCoV-19/Spain/AN-ISCIII-201617/2020|EPI_ISL_419230	····················	····················	····················	····················	····················	····················	
A	8478 hCoV-19/Hong Kong/HKPU63-1402/2020|EPI_ISL_419231|	····················	····················	····················	····················	····················	····················	
A	8479 hCoV-19/Hong Kong/HKPU64-1202/2020|EPI_ISL_419232|	····················	····················	····················	····················	····················	····················	
A	8480 hCoV-19/Spain/CN-ISCIII-201495/2020|EPI_ISL_419233	····················	····················	····················	····················	····················	····················	
A	8481 hCoV-19/Spain/RI-ISCIII-201575/2020|EPI_ISL_419234	····················	····················	····················	····················	····················	····················	
A	8482 hCoV-19/Spain/MD-ISCIII-201706/2020|EPI_ISL_419235	····················	····················	····················	····················	····················	····················	
A	8483 hCoV-19/Spain/MD-ISCIII-201709/2020|EPI_ISL_419236	····················	····················	····················	····················	····················	····················	
A	8484 hCoV-19/Spain/MD-ISCIII-201738/2020|EPI_ISL_419237	····················	····················	····················	····················	····················	····················	
A	8485 hCoV-19/Spain/PV-ISCIII-201493/2020|EPI_ISL_419238	····················	····················	····················	····················	····················	····················	
A	8486 hCoV-19/Spain/PV-ISCIII-201607/2020|EPI_ISL_419240	····················	····················	····················	····················	····················	····················	
A	8487 hCoV-19/Hong Kong/HKPU74-1302/2020|EPI_ISL_419245|	····················	····················	····················	····················	····················	····················	
A	8488 hCoV-19/Hong Kong/HKPU83-1702/2020|EPI_ISL_419247|	····················	····················	····················	····················	····················	····················	
A	8489 hCoV-19/USA/VA-DCLS-00010/2020|EPI_ISL_419262|2020	····················	····················	····················	····················	····················	····················	
A	8490 hCoV-19/USA/VA-DCLS-00011/2020|EPI_ISL_419263|2020	····················	····················	····················	····················	····················	····················	
A	8491 hCoV-19/Belgium/UGent-12/2020|EPI_ISL_419264|2020-	····················	····················	····················	····················	····················	····················	
A	8492 hCoV-19/Belgium/UGent-6/2020|EPI_ISL_419265|2020-0	····················	····················	····················	····················	····················	····················	
A	8493 hCoV-19/Australia/VIC324/2020|EPI_ISL_420015|2020-	····················	····················	····················	····················	····················	····················	
A	8494 hCoV-19/Australia/VIC325/2020|EPI_ISL_420016|2020-	····················	····················	····················	····················	····················	····················	
A	8495 hCoV-19/Australia/VIC326/2020|EPI_ISL_420017|2020-	····················	····················	····················	····················	····················	····················	
A	8496 hCoV-19/USA/VA-DCLS-0031/2020|EPI_ISL_420018|2020-	····················	····················	····················	····················	····················	····················	
A	8497 hCoV-19/Algeria/G0860_2262/2020|EPI_ISL_420037|202	····················	····················	····················	····················	····················	····················	
A	8498 hCoV-19/France/BFC-2709/2020|EPI_ISL_420038|2020-0	····················	····················	····················	····················	····················	····················	
A	8499 hCoV-19/France/GES-2720/2020|EPI_ISL_420039|2020-0	····················	····················	····················	····················	····················	····················	
A	8500 hCoV-19/France/GES-2722/2020|EPI_ISL_420040|2020-0	····················	····················	····················	····················	····················	····················	
A	8501 hCoV-19/France/HDF-2748/2020|EPI_ISL_420041|2020-0	····················	····················	····················	····················	····················	····················	
A	8502 hCoV-19/France/IDF-2768/2020|EPI_ISL_420042|2020-0	····················	····················	····················	····················	····················	····················	
A	8503 hCoV-19/France/IDF-2792/2020|EPI_ISL_420043|2020-0	····················	····················	····················	····················	····················	····················	
A	8504 hCoV-19/France/HDF-2797/2020|EPI_ISL_420044|2020-0	····················	····················	····················	····················	····················	····················	
A	8505 hCoV-19/France/GES-2843/2020|EPI_ISL_420045|2020-0	····················	····················	····················	····················	····················	····················	
A	8506 hCoV-19/France/IDF-2848/2020|EPI_ISL_420046|2020-0	····················	····················	····················	····················	····················	····················	
A	8507 hCoV-19/France/IDF-2849/2020|EPI_ISL_420047|2020-0	····················	····················	····················	····················	····················	····················	
A	8508 hCoV-19/France/IDF-2936/2020|EPI_ISL_420048|2020-0	····················	····················	····················	····················	····················	····················	
A	8509 hCoV-19/France/HDF-2946/2020|EPI_ISL_420049|2020-0	····················	····················	····················	····················	····················	····················	
A	8510 hCoV-19/France/HDF-2948/2020|EPI_ISL_420050|2020-0	····················	····················	····················	····················	····················	····················	
A	8511 hCoV-19/France/IDF-2989/2020|EPI_ISL_420051|2020-0	····················	····················	····················	····················	····················	····················	
A	8512 hCoV-19/France/IDF-3029/2020|EPI_ISL_420052|2020-0	····················	····················	····················	····················	····················	····················	
A	8513 hCoV-19/France/HDF-3036/2020|EPI_ISL_420053|2020-0	····················	····················	····················	····················	····················	····················	
A	8514 hCoV-19/France/GES-3067/2020|EPI_ISL_420055|2020-0	····················	····················	····················	····················	····················	····················	
A	8515 hCoV-19/France/HDF-3138/2020|EPI_ISL_420056|2020-0	····················	····················	····················	····················	····················	····················	
A	8516 hCoV-19/France/HDF-3141/2020|EPI_ISL_420057|2020-0	····················	····················	··A·················	····················	····················	····················	
A	8517 hCoV-19/France/IDF-3163/2020|EPI_ISL_420058|2020-0	····················	····················	····················	····················	····················	····················	
A	8518 hCoV-19/France/IDF-3165/2020|EPI_ISL_420059|2020-0	····················	····················	····················	····················	····················	····················	
A	8519 hCoV-19/France/IDF-3170/2020|EPI_ISL_420060|2020-0	····················	····················	····················	····················	····················	····················	
A	8520 hCoV-19/France/IDF-3212/2020|EPI_ISL_420061|2020-0	····················	····················	····················	····················	····················	····················	
A	8521 hCoV-19/France/IDF-3230/2020|EPI_ISL_420063|2020-0	····················	····················	····················	····················	····················	····················	
A	8522 hCoV-19/France/IDF-3235/2020|EPI_ISL_420064|2020-0	····················	····················	····················	····················	····················	····················	
A	8523 hCoV-19/Estonia/ChVir1982/2020|EPI_ISL_420065|2020	····················	····················	····················	····················	····················	····················	
A	8524 hCoV-19/Estonia/ChVir1983/2020|EPI_ISL_420066|2020	····················	····················	····················	····················	····················	····················	
A	8525 hCoV-19/Estonia/ChVir1985/2020|EPI_ISL_420067|2020	····················	····················	····················	····················	····················	····················	

A	8526 hCoV-19/Senegal/306/2020|EPI_ISL_420069|2020-03-17	....	....	....	....	....	....	
A	8527 hCoV-19/Senegal/315/2020|EPI_ISL_420070|2020-03-17	....	....	....	....	....	....	
A	8528 hCoV-19/Senegal/370/2020|EPI_ISL_420072|2020-03-18	....	....	....	....	....	....	
A	8529 hCoV-19/Senegal/382/2020|EPI_ISL_420073|2020-03-19	....	....	....	....	....	....	
A	8530 hCoV-19/Senegal/600/2020|EPI_ISL_420074|2020-03-20	....	....	....	....	....	....	
A	8531 hCoV-19/Senegal/611/2020|EPI_ISL_420076|2020-03-20	....	....	....	....	....	....	
A	8532 hCoV-19/Senegal/618/2020|EPI_ISL_420077|2020-03-20	....	....	....	....	....	....	
A	8533 hCoV-19/Senegal/620/2020|EPI_ISL_420078|2020-03-20	....	....	....	....	....	....	
A	8534 hCoV-19/Russia/StPetersburg-RII3992/2020|EPI_ISL_4	....	....	....	....	....	....	
A	8535 hCoV-19/Russia/StPetersburg-RII3997/2020|EPI_ISL_4	....	....	....	....	....	....	
A	8536 hCoV-19/Taiwan/125/2020|EPI_ISL_420082|2020-03-19	....	....	....	....	....	....	
A	8537 hCoV-19/Taiwan/128/2020|EPI_ISL_420083|2020-03-18	....	....	....	....	....	....	
A	8538 hCoV-19/Taiwan/170/2020|EPI_ISL_420084|2020-03-21	....	....	....	....	....	....	
A	8539 hCoV-19/Taiwan/107/2020|EPI_ISL_420085|2020-03-20	....	....	....	....	....	....	
A	8540 hCoV-19/Singapore/22/2020|EPI_ISL_420099|2020-03-0	....	....	....	....	....	....	
A	8541 hCoV-19/England/SHEF-C04C4/2020|EPI_ISL_420200|202	....	....	....	....	....	....	
A	8542 hCoV-19/England/SHEF-C0488/2020|EPI_ISL_420201|202	....	....	....	....	....	....	
A	8543 hCoV-19/England/SHEF-C04B5/2020|EPI_ISL_420202|202	....	....	....	....	....	....	
A	8544 hCoV-19/England/SHEF-C045B/2020|EPI_ISL_420203|202	....	....	....	....	....	....	
A	8545 hCoV-19/England/SHEF-C0215/2020|EPI_ISL_420204|202	....	....	....	....	....	....	
A	8546 hCoV-19/England/SHEF-C034F/2020|EPI_ISL_420205|202	....	...................A	....	....	....	....	1
A	8549 hCoV-19/England/SHEF-C02AC/2020|EPI_ISL_420208|202	....	......A.............	....	....	....	....	
A	8550 hCoV-19/England/SHEF-C027F/2020|EPI_ISL_420209|202	....	....	....	....	....	....	
A	8551 hCoV-19/England/SHEF-C041F/2020|EPI_ISL_420210|202	....	....	....	....	....	....	
A	8552 hCoV-19/England/SHEF-C0224/2020|EPI_ISL_420211|202	....	....	....	....	....	....	
A	8553 hCoV-19/England/SHEF-C03D6/2020|EPI_ISL_420212|202	....	....	....	....	....	....	
A	8554 hCoV-19/England/SHEF-C04D3/2020|EPI_ISL_420213|202	....	....	....	....	....	....	
A	8556 hCoV-19/England/SHEF-C046A/2020|EPI_ISL_420215|202	....	....	....	....	....	....	
A	8557 hCoV-19/England/SHEF-C0312/2020|EPI_ISL_420216|202	....	....	....	....	....	....	
A	8558 hCoV-19/England/SHEF-C043D/2020|EPI_ISL_420217|202	....	....	....	....	....	....	
A	8560 hCoV-19/England/SHEF-C037C/2020|EPI_ISL_420219|202	....	....	....	....	....	....	
A	8561 hCoV-19/England/SHEF-C035E/2020|EPI_ISL_420220|202	....	....	....	....	....	....	
A	8562 hCoV-19/England/SHEF-C03E5/2020|EPI_ISL_420221|202	....	....	....	....	....	....	
A	8563 hCoV-19/England/SHEF-C02F7/2020|EPI_ISL_420222|202	....	....	....	....	....	....	
A	8564 hCoV-19/England/SHEF-C0716/2020|EPI_ISL_420223|202	....	....	....	....	....	....	
A	8565 hCoV-19/England/SHEF-C0594/2020|EPI_ISL_420224|202	....	....	....	....	....	....	
A	8566 hCoV-19/England/SHEF-C052B/2020|EPI_ISL_420225|202	....	....	....	....	....	....	
A	8567 hCoV-19/England/SHEF-C0154/2020|EPI_ISL_420226|202	....	....	....	....	....	....	
A	8568 hCoV-19/England/SHEF-C01FA/2020|EPI_ISL_420227|202	....	....	....	....	....	....	
A	8569 hCoV-19/England/SHEF-C0127/2020|EPI_ISL_420228|202	....	....	....	....	....	....	
A	8570 hCoV-19/England/SHEF-C01CD/2020|EPI_ISL_420229|202	....	....	....	....	....	....	
A	8571 hCoV-19/England/SHEF-C0145/2020|EPI_ISL_420230|202	....	....	....	....	....	....	
A	8572 hCoV-19/England/SHEF-C0691/2020|EPI_ISL_420231|202	....	....	....	....	....	....	
A	8573 hCoV-19/England/SHEF-C0682/2020|EPI_ISL_420232|202	....	....	....	....	....	....	
A	8574 hCoV-19/England/SHEF-C050D/2020|EPI_ISL_420233|202	....	....	....	....	....	....	
A	8575 hCoV-19/England/SHEF-C0567/2020|EPI_ISL_420234|202	....	....	....	....	....	....	
A	8576 hCoV-19/England/SHEF-C01BE/2020|EPI_ISL_420253|202	....	....	....	....	....	....	
A	8577 hCoV-19/England/SHEF-C01EB/2020|EPI_ISL_420254|202	....	....	....	....	....	....	
A	8578 hCoV-19/England/SHEF-C0163/2020|EPI_ISL_420255|202	....	....	....	....	....	....	
A	8579 hCoV-19/England/SHEF-C01DC/2020|EPI_ISL_420256|202	....	....	....	....	....	....	
A	8580 hCoV-19/USA/WY-WYPHL004/2020|EPI_ISL_421346|2020-0	....	....	....	....	....	....	
A	8581 hCoV-19/USA/NY-PV08109/2020|EPI_ISL_421348|2020-03	....	....	....	....	....	....	
A	8582 hCoV-19/USA/NY-PV08120/2020|EPI_ISL_421349|2020-03	....	....	....	....	....	....	
A	8583 hCoV-19/USA/NY-PV08401/2020|EPI_ISL_421369|2020-03	....	....	....	....	....	....	
A	8584 hCoV-19/USA/NY-PV08402/2020|EPI_ISL_421370|2020-03	....	....	....	....	....	....	
A	8585 hCoV-19/USA/NY-PV08403/2020|EPI_ISL_421371|2020-03	....	....	....	....	....	....	
A	8586 hCoV-19/USA/NY-PV08412/2020|EPI_ISL_421375|2020-03	....	....	....	....	....	....	
A	8587 hCoV-19/USA/NY-PV08413/2020|EPI_ISL_421376|2020-03	....	....	....	....	....	....	
A	8588 hCoV-19/USA/NY-PV08415/2020|EPI_ISL_421378|2020-03	....	....	....	....	....	....	
A	8589 hCoV-19/USA/NY-PV08416/2020|EPI_ISL_421379|2020-03	....	....	....	....	....	....	
A	8590 hCoV-19/USA/NY-PV08425/2020|EPI_ISL_421383|2020-03	....	....	....	....	....	....	
A	8591 hCoV-19/USA/NY-PV08426/2020|EPI_ISL_421384|2020-03	....	....	....	....	....	....	
A	8592 hCoV-19/USA/NY-PV08427/2020|EPI_ISL_421385|2020-03	....	....	....	....	....	....	
A	8593 hCoV-19/USA/NY-PV08428/2020|EPI_ISL_421386|2020-03	....	....	....	....	....	....	
A	8594 hCoV-19/USA/NY-PV08429/2020|EPI_ISL_421387|2020-03	....	....	....	....	....	....	
A	8595 hCoV-19/USA/NY-PV08432/2020|EPI_ISL_421388|2020-03	....	....	....	....	....	....	
A	8596 hCoV-19/USA/NY-PV08434/2020|EPI_ISL_421389|2020-03	....	....	....	....	....	....	
A	8597 hCoV-19/USA/NY-PV08435/2020|EPI_ISL_421390|2020-03	....	....	....	....	....	....	
A	8598 hCoV-19/USA/NY-PV08436/2020|EPI_ISL_421391|2020-03	....	....	....	....	....	....	
A	8599 hCoV-19/USA/NY-PV08443/2020|EPI_ISL_421394|2020-03	....	......A.............	....	....	....	....	
A	8600 hCoV-19/USA/NY-PV08444/2020|EPI_ISL_421395|2020-03	....	....	....	....	....	....	
A	8601 hCoV-19/USA/NY-PV08445/2020|EPI_ISL_421396|2020-03	....	....	....	....	....	....	
A	8602 hCoV-19/USA/NY-PV08446/2020|EPI_ISL_421397|2020-03	....	....	....	....	....	....	
A	8603 hCoV-19/USA/NY-PV08447/2020|EPI_ISL_421398|2020-03	....	....	....	....	....	....	
A	8604 hCoV-19/USA/NY-PV08448/2020|EPI_ISL_421399|2020-03	....	....	....	....	....	....	
A	8605 hCoV-19/France/HDF-2306/2020|EPI_ISL_421500|2020-0	....	....	....	....	....	....	
A	8606 hCoV-19/France/IDF-2359/2020|EPI_ISL_421501|2020-0	....	....	....	....	....	....	
A	8607 hCoV-19/France/IDF-2412/2020|EPI_ISL_421502|2020-0	....	....	....	....	....	....	

A	8608 hCoV-19/France/IDF-2414/2020|EPI_ISL_421503|2020-0	····················	····················	····················	····················	····················	····················
A	8609 hCoV-19/France/IDF-2536/2020|EPI_ISL_421504|2020-0	····················	····················	····················	····················	····················	····················
A	8610 hCoV-19/France/IDF-2548/2020|EPI_ISL_421505|2020-0	····················	····················	····················	····················	····················	····················
A	8611 hCoV-19/France/IDF-3236/2020|EPI_ISL_421506|2020-0	····················	····················	····················	····················	····················	····················
A	8612 hCoV-19/France/IDF-3274/2020|EPI_ISL_421507|2020-0	····················	····················	····················	····················	····················	····················
A	8613 hCoV-19/France/IDF-3276/2020|EPI_ISL_421508|2020-0	····················	····················	····················	····················	····················	····················
A	8614 hCoV-19/France/HDF-3290/2020|EPI_ISL_421509|2020-0	····················	····················	····················	····················	····················	····················
A	8615 hCoV-19/France/HDF-3293/2020|EPI_ISL_421510|2020-0	····················	····················	····················	····················	····················	····················
A	8616 hCoV-19/France/HDF-3295/2020|EPI_ISL_421511|2020-0	····················	····················	····················	····················	····················	····················
A	8617 hCoV-19/France/IDF-3324/2020|EPI_ISL_421512|2020-0	····················	····················	····················	····················	····················	····················
A	8618 hCoV-19/France/IDF-3345/2020|EPI_ISL_421513|2020-0	····················	····················	····················	····················	····················	····················
A	8619 hCoV-19/France/CVL-3365/2020|EPI_ISL_421514|2020-0	····················	····················	····················	····················	····················	····················
A	8620 hCoV-19/Spain/VC-FISABIO-40/2020|EPI_ISL_421515|20	····················	····················	····················	····················	····················	····················
A	8621 hCoV-19/Spain/VC-FISABIO-52/2020|EPI_ISL_421519|20	····················	····················	····················	····················	····················	····················
A	8622 hCoV-19/USA/WY-WYPHL006/2020|EPI_ISL_421543|2020-0	····················	····················	····················	····················	····················	····················
A	8623 hCoV-19/USA/WY-WYPHL007/2020|EPI_ISL_421544|2020-0	····················	····················	····················	····················	····················	····················
A	8624 hCoV-19/USA/WY-WYPHL008/2020|EPI_ISL_421545|2020-0	····················	····················	····················	····················	····················	····················
A	8625 hCoV-19/USA/WY-WYPHL011/2020|EPI_ISL_421548|2020-0	····················	····················	····················	····················	····················	····················
A	8626 hCoV-19/USA/WY-WYPHL012/2020|EPI_ISL_421549|2020-0	····················	····················	····················	····················	····················	····················
A	8627 hCoV-19/USA/WY-WYPHL014/2020|EPI_ISL_421550|2020-0	····················	····················	····················	····················	····················	····················
A	8628 hCoV-19/USA/WY-WYPHL015/2020|EPI_ISL_421551|2020-0	····················	····················	····················	····················	····················	····················
A	8629 hCoV-19/USA/WY-WYPHL018/2020|EPI_ISL_421552|2020-0	····················	····················	····················	····················	····················	····················
A	8630 hCoV-19/USA/UT-035/2020|EPI_ISL_421560|2020-03-18	····················	····················	····················	····················	····················	····················
A	8631 hCoV-19/USA/UT-055/2020|EPI_ISL_421561|2020-03-17	····················	····················	····················	····················	····················	····················
A	8632 hCoV-19/USA/UT-0139/2020|EPI_ISL_421562|2020-03-19	····················	····················	····················	····················	····················	····················
A	8633 hCoV-19/USA/UT-0364/2020|EPI_ISL_421563|2020-03-29	····················	····················	····················	····················	····················	····················
A	8634 hCoV-19/USA/NY-NYUMC81/2020|EPI_ISL_421582|2020-03	····················	····················	····················	····················	····················	····················
A	8635 hCoV-19/USA/NJ-NYUMC83/2020|EPI_ISL_421584|2020-03	····················	····················	····················	····················	····················	····················
A	8636 hCoV-19/USA/NJ-NYUMC84/2020|EPI_ISL_421585|2020-03	····················	····················	····················	····················	····················	····················
A	8637 hCoV-19/USA/NY-NYUMC85/2020|EPI_ISL_421586|2020-03	····················	····················	····················	····················	····················	····················
A	8638 hCoV-19/USA/NY-NYUMC86/2020|EPI_ISL_421587|2020-03	····················	····················	····················	····················	····················	····················
A	8639 hCoV-19/Netherlands/NA_133/2020|EPI_ISL_422677|202	····················	····················	····················	····················	····················	····················
A	8640 hCoV-19/Netherlands/NA_134/2020|EPI_ISL_422678|202	····················	····················	····················	····················	····················	····················
A	8641 hCoV-19/Netherlands/NA_135/2020|EPI_ISL_422679|202	····················	····················	····················	····················	····················	····················
A	8642 hCoV-19/Netherlands/NA_164/2020|EPI_ISL_422699|202	····················	····················	····················	····················	····················	····················
A	8643 hCoV-19/Netherlands/NA_73/2020|EPI_ISL_422833|2020	····················	····················	····················	····················	····················	····················
A	8644 hCoV-19/Netherlands/NA_74/2020|EPI_ISL_422834|2020	····················	····················	····················	····················	····················	····················
A	8645 hCoV-19/Netherlands/NA_75/2020|EPI_ISL_422835|2020	····················	····················	····················	····················	····················	····················
A	8646 hCoV-19/Netherlands/NA_77/2020|EPI_ISL_422837|2020	····················	····················	····················	····················	····················	····················
A	8647 hCoV-19/Netherlands/NA_78/2020|EPI_ISL_422838|2020	····················	····················	····················	····················	····················	····················
A	8648 hCoV-19/Netherlands/NA_79/2020|EPI_ISL_422839|2020	····················	····················	····················	············A·······	····················	····················
A	8649 hCoV-19/Netherlands/NA_80/2020|EPI_ISL_422840|2020	····················	····················	····················	····················	····················	····················
A	8650 hCoV-19/Netherlands/NA_82/2020|EPI_ISL_422842|2020	····················	····················	····················	····················	····················	····················
A	8651 hCoV-19/Netherlands/NA_83/2020|EPI_ISL_422843|2020	····················	····················	····················	····················	····················	····················
A	8652 hCoV-19/Netherlands/NA_85/2020|EPI_ISL_422845|2020	····················	····················	····················	····················	····················	····················
A	8653 hCoV-19/Netherlands/NA_87/2020|EPI_ISL_422847|2020	·T··················	····················	····················	····················	····················	····················
A	8654 hCoV-19/Netherlands/NA_88/2020|EPI_ISL_422848|2020	····················	····················	····················	····················	····················	····················
A	8655 hCoV-19/Netherlands/NA_89/2020|EPI_ISL_422849|2020	····················	····················	····················	····················	····················	····················
A	8656 hCoV-19/Netherlands/NA_90/2020|EPI_ISL_422850|2020	····················	····················	····················	····················	····················	····················
A	8657 hCoV-19/Netherlands/NA_91/2020|EPI_ISL_422851|2020	····················	····················	····················	····················	····················	····················
A	8658 hCoV-19/Netherlands/NA_92/2020|EPI_ISL_422852|2020	····················	····················	····················	····················	····················	····················
A	8659 hCoV-19/Netherlands/NA_93/2020|EPI_ISL_422853|2020	····················	····················	····················	····················	····················	····················
A	8660 hCoV-19/Netherlands/NA_94/2020|EPI_ISL_422854|2020	····················	····················	····················	····················	····················	····················
A	8661 hCoV-19/Netherlands/NA_95/2020|EPI_ISL_422855|2020	····················	····················	····················	····················	····················	····················
A	8662 hCoV-19/Netherlands/NA_96/2020|EPI_ISL_422856|2020	·T··················	····················	····················	····················	····················	····················
A	8663 hCoV-19/Netherlands/NA_97/2020|EPI_ISL_422857|2020	····················	····················	····················	····················	····················	····················
A	8664 hCoV-19/Netherlands/NA_99/2020|EPI_ISL_422858|2020	····················	····················	····················	····················	····················	····················
A	8665 hCoV-19/Netherlands/NoordBrabant_100/2020|EPI_ISL_	····················	····················	····················	····················	····················	····················
A	8666 hCoV-19/Netherlands/NoordBrabant_101/2020|EPI_ISL_	····················	····················	····················	····················	····················	····················
A	8667 hCoV-19/Netherlands/NoordBrabant_102/2020|EPI_ISL_	····················	····················	····················	····················	····················	····················
A	8668 hCoV-19/Netherlands/NoordBrabant_103/2020|EPI_ISL_	····················	····················	····················	····················	····················	····················
A	8669 hCoV-19/Netherlands/NoordBrabant_104/2020|EPI_ISL_	····················	····················	····················	····················	····················	····················
A	8670 hCoV-19/Netherlands/NoordBrabant_105/2020|EPI_ISL_	····················	····················	····················	····················	····················	····················
A	8671 hCoV-19/Netherlands/NoordBrabant_69/2020|EPI_ISL_4	····················	····················	····················	····················	····················	····················
A	8672 hCoV-19/Netherlands/NoordBrabant_70/2020|EPI_ISL_4	····················	····················	····················	····················	····················	····················
A	8673 hCoV-19/Netherlands/NoordBrabant_71/2020|EPI_ISL_4	····················	····················	····················	····················	····················	····················
A	8674 hCoV-19/Netherlands/NoordBrabant_72/2020|EPI_ISL_4	····················	····················	····················	····················	····················	····················
A	8675 hCoV-19/Netherlands/NoordBrabant_73/2020|EPI_ISL_4	····················	····················	····················	····················	····················	····················
A	8676 hCoV-19/Netherlands/NoordBrabant_74/2020|EPI_ISL_4	····················	····················	····················	····················	····················	····················
A	8677 hCoV-19/Netherlands/NoordBrabant_75/2020|EPI_ISL_4	····················	····················	····················	····················	····················	····················
A	8678 hCoV-19/Netherlands/NoordBrabant_76/2020|EPI_ISL_4	····················	····················	····················	····················	····················	····················
A	8679 hCoV-19/Netherlands/NoordBrabant_77/2020|EPI_ISL_4	····················	····················	····················	····················	····················	····················
A	8680 hCoV-19/Netherlands/NoordBrabant_79/2020|EPI_ISL_4	····················	····················	····················	····················	····················	····················
A	8681 hCoV-19/Netherlands/NoordBrabant_80/2020|EPI_ISL_4	····················	····················	····················	····················	····················	····················
A	8682 hCoV-19/Netherlands/NoordBrabant_81/2020|EPI_ISL_4	····················	····················	····················	····················	····················	····················
A	8683 hCoV-19/Netherlands/NoordBrabant_82/2020|EPI_ISL_4	····················	····················	····················	····················	····················	····················
A	8684 hCoV-19/Netherlands/NoordBrabant_83/2020|EPI_ISL_4	····················	····················	····················	····················	····················	····················
A	8685 hCoV-19/Netherlands/NoordBrabant_85/2020|EPI_ISL_4	····················	····················	····················	····················	····················	····················

A	8686	hCoV-19/Netherlands/NoordBrabant_86/2020|EPI_ISL_4	···················· ···················· ····················· ···················· ···················· ····················
A	8687	hCoV-19/Netherlands/NoordBrabant_87/2020|EPI_ISL_4	···················· ···················· ····················· ···················· ···················· ····················
A	8688	hCoV-19/Netherlands/NoordBrabant_88/2020|EPI_ISL_4	···················· ···················· ····················· ···················· ···················· ····················
A	8689	hCoV-19/Netherlands/NoordBrabant_89/2020|EPI_ISL_4	···················· ···················· ····················· ···················· ···················· ····················
A	8690	hCoV-19/Netherlands/NoordBrabant_90/2020|EPI_ISL_4	···················· ···················· ····················· ···················· ···················· ····················
A	8691	hCoV-19/Netherlands/NoordBrabant_91/2020|EPI_ISL_4	···················· ···················· ····················· ···················· ···················· ····················
A	8692	hCoV-19/Netherlands/NoordBrabant_92/2020|EPI_ISL_4	···················· ···················· ····················· ···················· ···················· ····················
A	8693	hCoV-19/Netherlands/NoordBrabant_93/2020|EPI_ISL_4	···················· ···················· ····················· ···················· ···················· ····················
A	8694	hCoV-19/Netherlands/NoordBrabant_94/2020|EPI_ISL_4	···················· ···················· ····················· ···················· ···················· ····················
A	8695	hCoV-19/Netherlands/NoordBrabant_95/2020|EPI_ISL_4	···················· ···················· ····················· ···················· ···················· ····················
A	8696	hCoV-19/Netherlands/NoordBrabant_96/2020|EPI_ISL_4	···················· ···················· ····················· ···················· ···················· ····················
A	8697	hCoV-19/Netherlands/NoordBrabant_97/2020|EPI_ISL_4	···················· ···················· ····················· ···················· ···················· ····················
A	8698	hCoV-19/Netherlands/NoordBrabant_98/2020|EPI_ISL_4	···················· ···················· ····················· ···················· ···················· ····················
A	8699	hCoV-19/Netherlands/NoordBrabant_99/2020|EPI_ISL_4	···················· ···················· ····················· ···················· ···················· ····················
A	8700	hCoV-19/Netherlands/NoordHolland_31/2020|EPI_ISL_4	···················· ···················· ····················· ···················· ···················· ····················
A	8701	hCoV-19/Netherlands/NoordHolland_4/2020|EPI_ISL_42	···················· ···················· ····················· ···················· ···················· ····················
A	8702	hCoV-19/USA/NY-NYUMC287/2020|EPI_ISL_428801|2020-0	···················· ···················· ····················· ···················· ···················· ····················
A	8703	hCoV-19/USA/NY-NYUMC290/2020|EPI_ISL_428804|2020-0	···················· ···················· ····················· ···················· ···················· ····················
A	8704	hCoV-19/USA/NY-NYUMC291/2020|EPI_ISL_428805|2020-0	···················· ···················· ····················· ···················· ···················· ····················
A	8705	hCoV-19/env/Wuhan/IVDC-HBF13-20/2020|EPI_ISL_40851	···················· ···················· ····················· ···················· ···················· ····················
A	8706	hCoV-19/env/Wuhan/IVDC-HBF13-21/2020|EPI_ISL_40851	···················· ···················· ····················· ···················· ···················· ····················
A	8707	hCoV-19/Canada/ON-PHL-2445/2020|EPI_ISL_413014|202	···················· ·A·················· ····················· ···················· ···················· ····················
A	8708	hCoV-19/Canada/ON_VIDO-01/2020|EPI_ISL_413015|2020	···················· ·A·················· ··T·················· ···················· ···················· ····················
A	8709	hCoV-19/Brazil/SP-02/2020|EPI_ISL_413016|2020-02-2	···················· ···················· ····················· ···················· ···················· ····················
A	8710	hCoV-19/South Korea/KUMC01/2020|EPI_ISL_413017|202	···················· ···················· ····················· ···················· ···················· ····················
A	8711	hCoV-19/South Korea/KUMC02/2020|EPI_ISL_413018|202	···················· ···················· ····················· ···················· ···················· ····················
A	8712	hCoV-19/Switzerland/ZH-UZH-1000477102/2020|EPI_ISL	···················· ···················· ····················· ···················· ···················· ····················
A	8713	hCoV-19/Switzerland/ZH-UZH-1000477377/2020|EPI_ISL	···················· ···················· ····················· ···················· ···················· ····················
A	8714	hCoV-19/Switzerland/ZH-UZH-1000477757/2020|EPI_ISL	···················· ···················· ····················· ···················· ···················· ····················
A	8715	hCoV-19/Switzerland/ZH-UZH-1000477796/2020|EPI_ISL	···················· ···················· ····················· ···················· ···················· ····················
A	8716	hCoV-19/Switzerland/ZH-UZH-1000477797/2020|EPI_ISL	···················· ···················· ····················· ···················· ···················· ····················
A	8717	hCoV-19/Switzerland/ZH-UZH-1000477806/2020|EPI_ISL	···················· ···················· ····················· ···················· ···················· ····················
A	8718	hCoV-19/USA/WA3-UW1/2020|EPI_ISL_413025|2020-02-27	···················· ···················· ····················· ···················· ···················· ····················
A	8719	hCoV-19/USA/WA-UW15/2020|EPI_ISL_414363|2020-03-04	···················· ···················· ····················· ···················· ···················· ····················
A	8720	hCoV-19/USA/WA-UW17/2020|EPI_ISL_414365|2020-03-06	···················· ···················· ····················· ···················· ···················· ····················
A	8721	hCoV-19/Singapore/12/2020|EPI_ISL_414378|2020-02-1	···················· ···················· ····················· ···················· ···················· ····················
A	8722	hCoV-19/Singapore/13/2020|EPI_ISL_414379|2020-02-1	···················· ···················· ····················· ···················· ···················· ····················
A	8723	hCoV-19/Singapore/14/2020|EPI_ISL_414380|2020-02-1	···················· ···················· ····················· ···················· ···················· ····················
A	8724	hCoV-19/USA/WA-UW61/2020|EPI_ISL_415626|2020-03-10	···················· ···················· ····················· ···················· ···················· ····················
A	8725	hCoV-19/USA/WA-UW62/2020|EPI_ISL_415627|2020-03-09	···················· ···················· ····················· ···················· ···················· ····················
A	8726	hCoV-19/Scotland/EDB004/2020|EPI_ISL_415629|2020-0	···················· ···················· ····················· ···················· ···················· ····················
A	8727	hCoV-19/Scotland/CVR07/2020|EPI_ISL_415630|2020-03	···················· ···················· ····················· ···················· ···················· ····················
A	8728	hCoV-19/Scotland/CVR10/2020|EPI_ISL_415631|2020-03	···················· ···················· ····················· ···················· ···················· ····················
A	8729	hCoV-19/Scotland/EDB003/2020|EPI_ISL_415640|2020-0	···················· ···················· ····················· ···················· ···················· ····················
A	8730	hCoV-19/Georgia/Tb-54/2020|EPI_ISL_415641|2020-02-	···················· ···················· ····················· ···················· ···················· ····················
A	8731	hCoV-19/Georgia/Tb-477/2020|EPI_ISL_415642|2020-03	···················· ···················· ····················· ···················· ···················· ····················
A	8732	hCoV-19/Georgia/Tb-468/2020|EPI_ISL_415643|2020-03	···················· ···················· ····················· ···················· ···················· ····················
A	8733	hCoV-19/Georgia/Tb-82/2020|EPI_ISL_415644|2020-02-	···················· ···················· ····················· ···················· ···················· ····················
A	8734	hCoV-19/Denmark/SSI-104/2020|EPI_ISL_415648|2020-0	···················· ···················· ····················· ···················· ···················· ····················
A	8735	hCoV-19/France/HDF-2039/2020|EPI_ISL_415649|2020-0	···················· ···················· ····················· ···················· ···················· ····················
A	8736	hCoV-19/France/IDF-2075/2020|EPI_ISL_415650|2020-0	···················· ···················· ····················· ···················· ···················· ····················
A	8737	hCoV-19/France/BFC-2094/2020|EPI_ISL_415651|2020-0	···················· ···················· ····················· ···················· ···················· ····················
A	8738	hCoV-19/France/BFC-2147/2020|EPI_ISL_415652|2020-0	···················· ···················· ····················· ···················· ···················· ····················
A	8739	hCoV-19/France/HDF-2174/2020|EPI_ISL_415654|2020-0	···················· ···················· ····················· ···················· ···················· ····················
A	8740	hCoV-19/Wales/PHW27/2020|EPI_ISL_415655|2020-03-12	···················· ···················· ····················· ···················· ···················· ····················
A	8741	hCoV-19/Wales/PHW33/2020|EPI_ISL_415656|2020-03-12	···················· ···················· ····················· ···················· ···················· ····················
A	8742	hCoV-19/Wales/PHW37/2020|EPI_ISL_415657|2020-03-12	···················· ···A················ ····················· ···················· ···················· ····················
A	8743	hCoV-19/Chile/RM-MSHS-op2d1/2020|EPI_ISL_415658|20	···················· ···················· ····················· ···················· ···················· ····················
A	8744	hCoV-19/Wales/PHW28/2020|EPI_ISL_415659|2020-03-12	···················· ···················· ····················· ···················· ···················· ····················
A	8745	hCoV-19/Chile/RM-MSHS-op3d1/2020|EPI_ISL_415660|20	···················· ···················· ····················· ···················· ···················· ····················
A	8746	hCoV-19/Chile/RM-MSHS-op4d1/2020|EPI_ISL_415661|20	···················· ···················· ····················· ···················· ···················· ····················
A	8747	hCoV-19/Spain/CL-ISCIII-201437/2020|EPI_ISL_416994	···················· ···················· ····················· ···················· ···················· ····················
A	8749	hCoV-19/Hong Kong/HKPU89-2502/2020|EPI_ISL_419250|	···················· ···················· ····················· ···················· ···················· ····················
A	8750	hCoV-19/Hong Kong/HKPU91-2002/2020|EPI_ISL_419252|	···················· ···················· ····················· ···················· ···················· ····················
A	8751	hCoV-19/Italy/LAZ-INMI-6/2020|EPI_ISL_419254|2020-	···················· ···················· ····················· ···················· ···················· ····················
A	8752	hCoV-19/Italy/LAZ-INMI-7/2020|EPI_ISL_419255|2020-	···················· ···················· ····················· ···················· ···················· ····················
A	8753	hCoV-19/USA/VA-DCLS-0005/2020|EPI_ISL_419256|2020-	···················· ···················· ····················· ···················· ···················· ····················
A	8754	hCoV-19/USA/VA-DCLS-0006/2020|EPI_ISL_419257|2020-	···················· ···················· ····················· ···················· ···················· ····················
A	8755	hCoV-19/USA/VA-DCLS-0007/2020|EPI_ISL_419258|2020-	···················· ···················· ····················· ···················· ···················· ····················
A	8756	hCoV-19/Belgium/UGent-11/2020|EPI_ISL_419259|2020-	···················· ···················· ····················· ···················· ···················· ····················
A	8757	hCoV-19/USA/VA-DCLS-0008/2020|EPI_ISL_419260|2020-	···················· ···················· ····················· ···················· ···················· ····················
A	8758	hCoV-19/USA/VA-DCLS-0009/2020|EPI_ISL_419261|2020-	···················· ···················· ····················· ···················· ···················· ····················
A	8759	hCoV-19/Belgium/UGent-4/2020|EPI_ISL_419266|2020-0	···················· ···················· ····················· ···················· ···················· ····················
A	8760	hCoV-19/England/SHEF-C053A/2020|EPI_ISL_420235|202	···················· ···················· ····················· ···················· ···················· ····················
A	8761	hCoV-19/England/SHEF-C0664/2020|EPI_ISL_420236|202	···················· ···················· ····················· ···················· ···················· ····················
A	8762	hCoV-19/England/SHEF-C0628/2020|EPI_ISL_420237|202	···················· ···················· ····················· ···················· ···················· ····················
A	8763	hCoV-19/England/SHEF-C051C/2020|EPI_ISL_420238|202	···················· ···················· ····················· ···················· ···················· ····················
A	8764	hCoV-19/England/SHEF-C0770/2020|EPI_ISL_420239|202	···················· ···················· ····················· ···················· ···················· ····················

A 8765 hCoV-19/England/SHEF-C0084/2020|EPI_ISL_420240|202 ....
A 8766 hCoV-19/England/SHEF-C0637/2020|EPI_ISL_420241|202 ....
A 8767 hCoV-19/England/SHEF-C0734/2020|EPI_ISL_420242|202 ....
A 8768 hCoV-19/England/SHEF-C05FE/2020|EPI_ISL_420243|202 ....
A 8769 hCoV-19/England/SHEF-C05B2/2020|EPI_ISL_420244|202 ....
A 8770 hCoV-19/England/SHEF-C06CE/2020|EPI_ISL_420245|202 ....
A 8771 hCoV-19/England/SHEF-C05D0/2020|EPI_ISL_420246|202 ....
A 8772 hCoV-19/England/SHEF-C0585/2020|EPI_ISL_420247|202 ....A...... ....
A 8773 hCoV-19/England/SHEF-C06BF/2020|EPI_ISL_420248|202 ....
A 8774 hCoV-19/England/SHEF-C0549/2020|EPI_ISL_420249|202 ....
A 8775 hCoV-19/England/SHEF-C06DD/2020|EPI_ISL_420250|202 ....
A 8776 hCoV-19/England/SHEF-C0673/2020|EPI_ISL_420251|202 ....
A 8777 hCoV-19/England/SHEF-C01AF/2020|EPI_ISL_420252|202 ....
A 8778 hCoV-19/South Africa/KRISP-06/2020|EPI_ISL_421573| ....
A 8779 hCoV-19/South Africa/KRISP-07/2020|EPI_ISL_421574| ....
A 8780 hCoV-19/South Africa/KRISP-011/2020|EPI_ISL_421575 ....
A 8781 hCoV-19/South Africa/KRISP-012/2020|EPI_ISL_421576 ....
A 8782 hCoV-19/USA/NY-NYUMC76/2020|EPI_ISL_421577|2020-03 ....
A 8783 hCoV-19/USA/NY-NYUMC77/2020|EPI_ISL_421578|2020-03 ....
A 8784 hCoV-19/USA/NY-NYUMC78/2020|EPI_ISL_421579|2020-03 ....
A 8785 hCoV-19/USA/NY-NYUMC79/2020|EPI_ISL_421580|2020-03 ....
A 8786 hCoV-19/USA/NY-NYUMC80/2020|EPI_ISL_421581|2020-03 ....
A 8787 hCoV-19/USA/NY-NYUMC82/2020|EPI_ISL_421583|2020-03 ....
A 8788 hCoV-19/Netherlands/NA_40/2020|EPI_ISL_422801|2020 ....
A 8789 hCoV-19/Netherlands/NA_41/2020|EPI_ISL_422802|2020 ....
A 8790 hCoV-19/Netherlands/NA_42/2020|EPI_ISL_422803|2020 ....
A 8791 hCoV-19/Netherlands/NA_43/2020|EPI_ISL_422804|2020 ....
A 8792 hCoV-19/Netherlands/NA_44/2020|EPI_ISL_422805|2020 ....
A 8793 hCoV-19/Netherlands/NA_46/2020|EPI_ISL_422807|2020 ....
A 8794 hCoV-19/Netherlands/NA_47/2020|EPI_ISL_422808|2020 ....
A 8795 hCoV-19/Netherlands/NA_48/2020|EPI_ISL_422809|2020 ....
A 8796 hCoV-19/Netherlands/NA_49/2020|EPI_ISL_422810|2020 ....
A 8797 hCoV-19/Netherlands/NA_50/2020|EPI_ISL_422811|2020 ....
A 8798 hCoV-19/Netherlands/NA_51/2020|EPI_ISL_422812|2020 ....
A 8799 hCoV-19/Netherlands/NA_52/2020|EPI_ISL_422813|2020 ....
A 8800 hCoV-19/Netherlands/NA_54/2020|EPI_ISL_422815|2020 ....
A 8801 hCoV-19/Netherlands/NA_55/2020|EPI_ISL_422816|2020 ....
A 8802 hCoV-19/Netherlands/NA_56/2020|EPI_ISL_422817|2020 ....
A 8803 hCoV-19/Netherlands/NA_57/2020|EPI_ISL_422818|2020 ....
A 8804 hCoV-19/Netherlands/NA_58/2020|EPI_ISL_422819|2020 ....
A 8805 hCoV-19/Netherlands/NA_59/2020|EPI_ISL_422820|2020 ....
A 8806 hCoV-19/Netherlands/NA_60/2020|EPI_ISL_422821|2020 ....
A 8807 hCoV-19/Netherlands/NA_61/2020|EPI_ISL_422822|2020 ....
A 8808 hCoV-19/Netherlands/NA_62/2020|EPI_ISL_422823|2020 ....
A 8809 hCoV-19/Netherlands/NA_64/2020|EPI_ISL_422825|2020 ....
A 8810 hCoV-19/Netherlands/NA_67/2020|EPI_ISL_422827|2020 ....
A 8811 hCoV-19/Netherlands/Utrecht_20/2020|EPI_ISL_422897 ....
A 8812 hCoV-19/Netherlands/Utrecht_21/2020|EPI_ISL_422898 ....
A 8813 hCoV-19/Belgium/UGent-1/2020|EPI_ISL_425048|2020-0 ....
A 8814 hCoV-19/Belgium/UGent-2/2020|EPI_ISL_425050|2020-0 ....
A 8815 hCoV-19/Belgium/UGent-8/2020|EPI_ISL_425052|2020-0 ....
A 8816 hCoV-19/Belgium/UGent-9/2020|EPI_ISL_425053|2020-0 ....
A 8817 hCoV-19/Belgium/UGent-10/2020|EPI_ISL_425054|2020- ....
A 8818 hCoV-19/Belgium/UGent-13/2020|EPI_ISL_425055|2020- ....
A 8819 hCoV-19/Belgium/UGent-15/2020|EPI_ISL_425056|2020- ....
A 8820 hCoV-19/Belgium/UGent-17/2020|EPI_ISL_425057|2020- ....
A 8821 hCoV-19/Belgium/UGent-18/2020|EPI_ISL_425058|2020- ....
A 8822 hCoV-19/Belgium/UGent-19/2020|EPI_ISL_425059|2020- ....
A 8823 hCoV-19/Belgium/UGent-20/2020|EPI_ISL_425060|2020- ....
A 8824 hCoV-19/Belgium/UGent-21/2020|EPI_ISL_425061|2020- ....
A 8825 hCoV-19/Belgium/UGent-22/2020|EPI_ISL_425062|2020- ....
A 8826 hCoV-19/Belgium/UGent-24/2020|EPI_ISL_425063|2020- ....
A 8827 hCoV-19/Belgium/UGent-25/2020|EPI_ISL_425064|2020- ....
A 8828 hCoV-19/USA/NY-Wadsworth-26088-01/2020|EPI_ISL_426 ....
A 8829 hCoV-19/USA/NY-Wadsworth-31302-01/2020|EPI_ISL_426 ....
A 8830 hCoV-19/USA/NY-Wadsworth-26856-01/2020|EPI_ISL_426 ....
A 8831 hCoV-19/USA/NY-Wadsworth-31364-01/2020|EPI_ISL_426 ....
A 8832 hCoV-19/USA/NY-Wadsworth-27162-01/2020|EPI_ISL_426 ....
A 8833 hCoV-19/USA/NY-Wadsworth-31930-01/2020|EPI_ISL_426 ....
A 8834 hCoV-19/USA/NY-Wadsworth-16004-01/2020|EPI_ISL_426 ....
A 8835 hCoV-19/USA/NY-Wadsworth-30164-01/2020|EPI_ISL_426 ....
A 8836 hCoV-19/USA/NY-Wadsworth-30246-01/2020|EPI_ISL_426 ....
A 8837 hCoV-19/USA/NY-Wadsworth-32104-01/2020|EPI_ISL_426 ....
A 8838 hCoV-19/USA/NY-Wadsworth-15616-01/2020|EPI_ISL_426 ....
A 8839 hCoV-19/USA/NY-Wadsworth-11736-02/2020|EPI_ISL_426 ....
A 8840 hCoV-19/USA/NY-Wadsworth-30878-01/2020|EPI_ISL_426 ....
A 8841 hCoV-19/USA/NY-Wadsworth-22503-01/2020|EPI_ISL_426 ....
A 8842 hCoV-19/USA/NY-Wadsworth-29275-01/2020|EPI_ISL_426 ....

A 8843 hCoV-19/USA/NY-Wadsworth-22874-01/2020|EPI_ISL_426 ....
A 8844 hCoV-19/USA/NY-Wadsworth-16833-01/2020|EPI_ISL_426 ....
A 8845 hCoV-19/USA/NY-Wadsworth-16480-01/2020|EPI_ISL_426 ....
A 8846 hCoV-19/USA/NY-Wadsworth-26551-01/2020|EPI_ISL_426 ....
A 8847 hCoV-19/USA/NY-Wadsworth-16166-01/2020|EPI_ISL_426 ....
A 8848 hCoV-19/USA/NY-Wadsworth-25860-01/2020|EPI_ISL_426 ....
A 8849 hCoV-19/USA/NY-Wadsworth-31617-01/2020|EPI_ISL_426 ....
A 8850 hCoV-19/USA/NY-Wadsworth-14726-02/2020|EPI_ISL_426 ....
A 8851 hCoV-19/USA/NY-Wadsworth-16766-01/2020|EPI_ISL_426 ....
A 8852 hCoV-19/USA/NY-Wadsworth-11190-01/2020|EPI_ISL_426 ....
A 8853 hCoV-19/USA/NY-Wadsworth-11191-01/2020|EPI_ISL_426 ....
A 8854 hCoV-19/USA/NY-Wadsworth-11245-01/2020|EPI_ISL_426 ....
A 8855 hCoV-19/USA/NY-Wadsworth-11249-01/2020|EPI_ISL_426 ....
A 8856 hCoV-19/USA/NY-Wadsworth-11330-01/2020|EPI_ISL_426 ....
A 8857 hCoV-19/Czech Republic/Seq3/2020|EPI_ISL_426356|20 ....
A 8858 hCoV-19/Czech Republic/Seq4/2020|EPI_ISL_426357|20 ....
A 8859 hCoV-19/Czech Republic/Seq5/2020|EPI_ISL_426358|20 ....A....
A 8860 hCoV-19/Czech Republic/Seq6/2020|EPI_ISL_426359|20 ....
A 8861 hCoV-19/Czech Republic/Seq7/2020|EPI_ISL_426360|20 ....
A 8862 hCoV-19/Mexico/CMX-INCMNSZ-01/2020|EPI_ISL_426361| ....
A 8863 hCoV-19/Mexico/CMX-INCMNSZ-02/2020|EPI_ISL_426362| ....
A 8864 hCoV-19/Mexico/CMX-INCMNSZ-03/2020|EPI_ISL_426363| ....
A 8865 hCoV-19/Mexico/CMX-INCMNSZ-04/2020|EPI_ISL_426364| ....
A 8866 hCoV-19/Mexico/CMX-INCMNSZ-05/2020|EPI_ISL_426365| ....
A 8867 hCoV-19/Slovenia/MB0329/2020|EPI_ISL_426379|2020-0 ....
A 8868 hCoV-19/USA/NY-WCMP12D09P/2020|EPI_ISL_427605|2020 ....
A 8869 hCoV-19/USA/NY-WCMP12E01P/2020|EPI_ISL_427606|2020 ....
A 8870 hCoV-19/USA/NY-WCMP12E06P/2020|EPI_ISL_427607|2020 ....
A 8871 hCoV-19/USA/NY-WCMP12E09P/2020|EPI_ISL_427608|2020 ....
A 8872 hCoV-19/USA/NY-WCMP12F01P/2020|EPI_ISL_427609|2020 ....
A 8873 hCoV-19/USA/NY-WCMP12F02P/2020|EPI_ISL_427610|2020 ....
A 8874 hCoV-19/USA/NY-WCMP12F03P/2020|EPI_ISL_427611|2020 ....
A 8875 hCoV-19/USA/NY-WCMP12F07P/2020|EPI_ISL_427612|2020 ....
A 8876 hCoV-19/USA/NY-WCMP12G01P/2020|EPI_ISL_427613|2020 ....
A 8877 hCoV-19/USA/NY-WCMP12G02P/2020|EPI_ISL_427614|2020 ....
A 8878 hCoV-19/USA/NY-WCMP12G03P/2020|EPI_ISL_427615|2020 ....T........ ....
A 8879 hCoV-19/USA/NY-WCMP12G06P/2020|EPI_ISL_427616|2020 ....
A 8880 hCoV-19/USA/NY-WCMP12G08P/2020|EPI_ISL_427617|2020 ....
A 8881 hCoV-19/USA/NY-WCMP12H01P/2020|EPI_ISL_427618|2020 ....
A 8882 hCoV-19/USA/AK-PHL092/2020|EPI_ISL_427619|2020-04- ....
A 8883 hCoV-19/USA/AK-PHL101/2020|EPI_ISL_427620|2020-04- ....
A 8884 hCoV-19/USA/AK-PHL115/2020|EPI_ISL_427621|2020-03- ....
A 8885 hCoV-19/USA/AK-PHL118/2020|EPI_ISL_427622|2020-03- ....
A 8886 hCoV-19/USA/NY-NYUMC227/2020|EPI_ISL_427627|2020-0 ....
A 8887 hCoV-19/USA/NY-NYUMC228/2020|EPI_ISL_427628|2020-0 ....
A 8888 hCoV-19/USA/NY-NYUMC229/2020|EPI_ISL_427629|2020-0 ....
A 8889 hCoV-19/USA/NY-NYUMC230/2020|EPI_ISL_427630|2020-0 ....
A 8890 hCoV-19/USA/NY-NYUMC231/2020|EPI_ISL_427631|2020-0 ....
A 8891 hCoV-19/USA/NY-NYUMC232/2020|EPI_ISL_427632|2020-0 ....
A 8892 hCoV-19/USA/NY-NYUMC233/2020|EPI_ISL_427633|2020-0 ....
A 8893 hCoV-19/USA/NY-NYUMC235/2020|EPI_ISL_427634|2020-0 ....
A 8894 hCoV-19/USA/NY-NYUMC236/2020|EPI_ISL_427635|2020-0 ....
A 8895 hCoV-19/USA/NY-NYUMC237/2020|EPI_ISL_427636|2020-0 ....
A 8896 hCoV-19/USA/NY-NYUMC238/2020|EPI_ISL_427637|2020-0 ....
A 8897 hCoV-19/USA/NY-NYUMC239/2020|EPI_ISL_427638|2020-0 ....
A 8898 hCoV-19/USA/NY-NYUMC240/2020|EPI_ISL_427639|2020-0 ....
A 8899 hCoV-19/USA/NY-NYUMC241/2020|EPI_ISL_427640|2020-0 ....
A 8900 hCoV-19/USA/NY-NYUMC242/2020|EPI_ISL_427641|2020-0 ....
A 8901 hCoV-19/Australia/NSW219/2020|EPI_ISL_427643|2020- ....
A 8902 hCoV-19/Australia/NSW217/2020|EPI_ISL_427644|2020- ....
A 8903 hCoV-19/Australia/NSW65/2020|EPI_ISL_427645|2020-0 ....
A 8904 hCoV-19/Australia/NSW66/2020|EPI_ISL_427646|2020-0 ....
A 8905 hCoV-19/Australia/NSW155/2020|EPI_ISL_427647|2020- ....
A 8906 hCoV-19/Australia/NSW152/2020|EPI_ISL_427648|2020- ....
A 8907 hCoV-19/Australia/NSW22/2020|EPI_ISL_427649|2020-0 ....
A 8908 hCoV-19/Australia/NSW23/2020|EPI_ISL_427650|2020-0 ....
A 8909 hCoV-19/Australia/NSW29/2020|EPI_ISL_427651|2020-0 ....
A 8910 hCoV-19/Australia/NSW154/2020|EPI_ISL_427652|2020- ....
A 8911 hCoV-19/Australia/NSW153/2020|EPI_ISL_427653|2020- ....
A 8912 hCoV-19/Australia/NSW33/2020|EPI_ISL_427654|2020-0 ....
A 8913 hCoV-19/Australia/NSW34/2020|EPI_ISL_427655|2020-0 ....
A 8914 hCoV-19/Australia/NSW102/2020|EPI_ISL_427656|2020- ....
A 8915 hCoV-19/Australia/NSW103/2020|EPI_ISL_427657|2020- ....
A 8916 hCoV-19/Australia/NSW46/2020|EPI_ISL_427658|2020-0 ....
A 8917 hCoV-19/Australia/NSW49/2020|EPI_ISL_427659|2020-0 ....
A 8918 hCoV-19/Australia/NSW178/2020|EPI_ISL_427670|2020- ....
A 8919 hCoV-19/Australia/NSW212/2020|EPI_ISL_427671|2020- ....
A 8920 hCoV-19/Australia/NSW95/2020|EPI_ISL_427672|2020-0 ....

A	8921	hCoV-19/Australia/NSW135/2020|EPI_ISL_427673|2020-	....	....	....	....	....	....
A	8922	hCoV-19/Australia/NSW157/2020|EPI_ISL_427674|2020-	....	....	....	....	....	....
A	8923	hCoV-19/Australia/NSW92/2020|EPI_ISL_427675|2020-0	....	....	....	....	....	....
A	8924	hCoV-19/Australia/NSW170/2020|EPI_ISL_427676|2020-	....	....	....	....	....	....
A	8925	hCoV-19/Australia/NSW104/2020|EPI_ISL_427677|2020-	....	....	....	....	....	....
A	8926	hCoV-19/Australia/NSW96/2020|EPI_ISL_427678|2020-0	....	....	....	....	....	....
A	8927	hCoV-19/Australia/NSW120/2020|EPI_ISL_427679|2020-	....	....	....	....	....	....
A	8928	hCoV-19/Australia/NSW90/2020|EPI_ISL_427680|2020-0	....	....	....	....	....	....
A	8929	hCoV-19/Australia/NSW93/2020|EPI_ISL_427681|2020-0	....	....	....	....	....	....
A	8930	hCoV-19/Australia/NSW146/2020|EPI_ISL_427682|2020-	....	....	....	....	....	....
A	8931	hCoV-19/Australia/NSW148/2020|EPI_ISL_427683|2020-	....	....	....	....	....	....
A	8932	hCoV-19/Australia/NSW200/2020|EPI_ISL_427684|2020-	....	....	....	....	....	....
A	8933	hCoV-19/Australia/NSW179/2020|EPI_ISL_427685|2020-	....	....	....	....	....	....
A	8934	hCoV-19/Australia/NSW105/2020|EPI_ISL_427686|2020-	....	....	....	....	....	....
A	8935	hCoV-19/Australia/NSW175/2020|EPI_ISL_427687|2020-	....	....	....	....	....	....
A	8936	hCoV-19/Australia/NSW121/2020|EPI_ISL_427688|2020-	....	....	....	....	....	....
A	8937	hCoV-19/Australia/NSW128/2020|EPI_ISL_427689|2020-	....	....	....	....	....	....
A	8938	hCoV-19/Australia/NSW80/2020|EPI_ISL_427690|2020-0	....	....	....	....	....	....
A	8939	hCoV-19/Australia/NSW187/2020|EPI_ISL_427691|2020-	....	....	....	....	....	....
A	8940	hCoV-19/Australia/NSW136/2020|EPI_ISL_427692|2020-	....	....	....	....	....	....
A	8941	hCoV-19/Australia/NSW118/2020|EPI_ISL_427693|2020-	....	....	....	....	....	....
A	8942	hCoV-19/Australia/NSW188/2020|EPI_ISL_427694|2020-	....	....	....	....	....	....
A	8943	hCoV-19/Australia/NSW206/2020|EPI_ISL_427695|2020-	....	....	....	....	....	....
A	8944	hCoV-19/Australia/NSW115/2020|EPI_ISL_427696|2020-	....	....	....	....	....	....
A	8945	hCoV-19/Australia/NSW167/2020|EPI_ISL_427697|2020-	....	....	....	....	....	....
A	8946	hCoV-19/Australia/NSW116/2020|EPI_ISL_427698|2020-	....	....	....	....	....	....
A	8947	hCoV-19/Australia/NSW211/2020|EPI_ISL_427699|2020-	....	....	....	....	....	....
A	8948	hCoV-19/USA/WI-UW-260/2020|EPI_ISL_428936|2020-04-	....	....	....	....	....	....
A	8949	hCoV-19/Luxembourg/LNS3259987/2020|EPI_ISL_428939|	....	....	....	....	....	....
A	8950	hCoV-19/Luxembourg/LNS7841004/2020|EPI_ISL_428940|	....	....	....	....	....	....
A	8951	hCoV-19/Luxembourg/LNS6969569/2020|EPI_ISL_428943|	....	....	....	....	....	....
A	8952	hCoV-19/Luxembourg/LNS1387711/2020|EPI_ISL_428944|	....	....	....	....	....	....
A	8953	hCoV-19/Luxembourg/LNS3702558/2020|EPI_ISL_428945|	....	....	....	....	....	....
A	8954	hCoV-19/Luxembourg/LNS0078676/2020|EPI_ISL_428949|	....	....	....	....	....	....
A	8955	hCoV-19/Luxembourg/LNS5971805/2020|EPI_ISL_428954|	....	....	....	....	....	....
A	8956	hCoV-19/Luxembourg/LNS6854244/2020|EPI_ISL_428956|	....	....	....	....	....	....
A	8957	hCoV-19/Luxembourg/LNS4295690/2020|EPI_ISL_428959|	....	....	....	....	....	....
A	8958	hCoV-19/Luxembourg/LNS6326231/2020|EPI_ISL_428960|	....	....	....	....	....	....
A	8959	hCoV-19/Luxembourg/LNS9322962/2020|EPI_ISL_428961|	....	....	....	....	....	....
A	8960	hCoV-19/Luxembourg/LNS6274573/2020|EPI_ISL_428962|	....	....	....	....	....	....
A	8961	hCoV-19/South Korea/KCDC03/2020|EPI_ISL_407193|202	....	....	....	....	....	....
A	8962	hCoV-19/USA/WA-UW16/2020|EPI_ISL_414364|2020-03-05	....	....	....	....	....	....
A	8963	hCoV-19/USA/WA-UW18/2020|EPI_ISL_414366|2020-03-05	....	....	....	....	....	....
A	8964	hCoV-19/USA/WA-UW19/2020|EPI_ISL_414367|2020-03-05	....	....	....	....	....	....
A	8965	hCoV-19/USA/WA-UW20/2020|EPI_ISL_414368|2020-03-05	....	....	....	....	....	....
A	8966	hCoV-19/USA/WA-UW21/2020|EPI_ISL_414369|2020-03-05	....	....	....	....	....	....
A	8967	hCoV-19/USA/WA-UW72/2020|EPI_ISL_415600|2020-03-09	....	....	....	....	....	....
A	8968	hCoV-19/USA/WA-UW73/2020|EPI_ISL_415601|2020-03-10	....	....	....	....	....	....
A	8969	hCoV-19/USA/WA-UW74/2020|EPI_ISL_415602|2020-03-10	....	....	....	....	....	....
A	8970	hCoV-19/USA/WA-UW75/2020|EPI_ISL_415603|2020-03-10	....	....	....	....	....	....
A	8971	hCoV-19/USA/WA-UW76/2020|EPI_ISL_415604|2020-03-10	....	....	....	....	....	....
A	8972	hCoV-19/USA/WA-UW40/2020|EPI_ISL_415605|2020-03-05	....	....	....	....	....	....
A	8973	hCoV-19/USA/WA-UW41/2020|EPI_ISL_415606|2020-03-08	....	....	....	....	....	....
A	8974	hCoV-19/USA/WA-UW42/2020|EPI_ISL_415607|2020-03-09	....	....	....	....	....	....
A	8975	hCoV-19/USA/WA-UW43/2020|EPI_ISL_415608|2020-03-08	....	....	....	....	....	....
A	8976	hCoV-19/USA/WA-UW44/2020|EPI_ISL_415609|2020-03-08	....	....	....	....	....	....
A	8977	hCoV-19/USA/WA-UW45/2020|EPI_ISL_415610|2020-03-08	....	....	....	....	....	....
A	8978	hCoV-19/USA/WA-UW46/2020|EPI_ISL_415611|2020-03-09	....	....	....	....	....	....
A	8979	hCoV-19/USA/WA-UW47/2020|EPI_ISL_415612|2020-03-09	....	....	....	....	....	....
A	8980	hCoV-19/USA/WA-UW48/2020|EPI_ISL_415613|2020-03-09	....	....	....	....	....	....
A	8981	hCoV-19/USA/WA-UW49/2020|EPI_ISL_415614|2020-03-09	....	....	....	....	....	....
A	8982	hCoV-19/USA/WA-UW50/2020|EPI_ISL_415615|2020-03-08	....	....	....	....	....	....
A	8983	hCoV-19/USA/WA-UW51/2020|EPI_ISL_415616|2020-03-08	....	....	....	....	....	....
A	8984	hCoV-19/USA/WA-UW52/2020|EPI_ISL_415617|2020-03-09	....	....	....	....	....	....
A	8985	hCoV-19/USA/WA-UW54/2020|EPI_ISL_415619|2020-03-09	....	....	....	....	....	....
A	8986	hCoV-19/USA/WA-UW55/2020|EPI_ISL_415620|2020-03-09	....	....	....	....	....	....
A	8987	hCoV-19/USA/WA-UW56/2020|EPI_ISL_415621|2020-03-09	....	....	....	....	....	....
A	8988	hCoV-19/USA/WA-UW57/2020|EPI_ISL_415622|2020-03-09	....	....	....	....	....	....
A	8989	hCoV-19/USA/WA-UW59/2020|EPI_ISL_415624|2020-03-09	....	....	....	....	....	....
A	8990	hCoV-19/USA/WA-UW60/2020|EPI_ISL_415625|2020-03-09	....	....	....	....	....	....
A	8991	hCoV-19/Switzerland/GR-HUG-2988/2020|EPI_ISL_41569	....	....	....	....	....	....
A	8992	hCoV-19/Switzerland/GR-HUG-3043/2020|EPI_ISL_41569	....	....	....	....	....	....
A	8993	hCoV-19/Malaysia/MKAK-CL-2020-7554/2020|EPI_ISL_41	....	....	....	....	....	....
A	8994	hCoV-19/France/ARA-10552/2020|EPI_ISL_419168|2020-	....	....	....	....	....	....
A	8995	hCoV-19/France/ARA-11939/2020|EPI_ISL_419169|2020-	....	....	....	....	....	....
A	8996	hCoV-19/France/ARA-11943/2020|EPI_ISL_419170|2020-	....	....	....	....	....	....
A	8997	hCoV-19/France/ARA-11949/2020|EPI_ISL_419171|2020-	....	....	....	....	....	....
A	8998	hCoV-19/France/ARA-11950/2020|EPI_ISL_419172|2020-	....	....	....	....	....	....

A	8999 hCoV-19/France/ARA-11952/2020|EPI_ISL_419173|2020-	····················	····················	····················	····················	····················	····················	
A	9000 hCoV-19/France/ARA-11980/2020|EPI_ISL_419174|2020-	····················	····················	····················	····················	····················	····················	
A	9001 hCoV-19/France/ARA-11995/2020|EPI_ISL_419175|2020-	····················	····················	····················	····················	····················	····················	
A	9002 hCoV-19/France/ARA-11997/2020|EPI_ISL_419176|2020-	····················	····················	····················	····················	····················	····················	
A	9003 hCoV-19/France/ARA-12125/2020|EPI_ISL_419177|2020-	····················	····················	····················	····················	····················	····················	
A	9004 hCoV-19/France/ARA-12217/2020|EPI_ISL_419178|2020-	····················	····················	····················	····················	····················	····················	
A	9005 hCoV-19/France/ARA-12222/2020|EPI_ISL_419179|2020-	····················	····················	····················	····················	····················	····················	
A	9006 hCoV-19/France/ARA-12238/2020|EPI_ISL_419180|2020-	····················	····················	····················	····················	····················	····················	
A	9007 hCoV-19/France/ARA-12249/2020|EPI_ISL_419181|2020-	····················	····················	····················	····················	····················	····················	
A	9008 hCoV-19/France/ARA-12250/2020|EPI_ISL_419182|2020-	····················	····················	····················	····················	····················	····················	
A	9009 hCoV-19/France/ARA-12253/2020|EPI_ISL_419183|2020-	····················	····················	····················	····················	····················	····················	
A	9010 hCoV-19/France/ARA-12260/2020|EPI_ISL_419184|2020-	····················	····················	····················	····················	····················	····················	
A	9011 hCoV-19/France/ARA-12264/2020|EPI_ISL_419185|2020-	····················	····················	····················	····················	····················	····················	
A	9012 hCoV-19/France/ARA-12265/2020|EPI_ISL_419186|2020-	····················	····················	····················	····················	····················	····················	
A	9013 hCoV-19/France/ARA-12269/2020|EPI_ISL_419187|2020-	····················	····················	····················	····················	····················	····················	
A	9014 hCoV-19/France/ARA-12270/2020|EPI_ISL_419188|2020-	····················	····················	····················	····················	····················	····················	
A	9015 hCoV-19/Norway/1694/2020|EPI_ISL_420136|2020-03-04	····················	····················	····················	····················	····················	····················	
A	9016 hCoV-19/Norway/1772/2020|EPI_ISL_420137|2020-03-08	····················	····················	····················	····················	····················	····················	
A	9017 hCoV-19/Norway/1811/2020|EPI_ISL_420139|2020-03-05	····················	····················	····················	····················	····················	····················	
A	9018 hCoV-19/Georgia/Tb-1352/2020|EPI_ISL_420140|2020-0	····················	····················	····················	····················	····················	····················	
A	9019 hCoV-19/Norway/1867/2020|EPI_ISL_420141|2020-03-10	····················	····················	····················	····················	····················	····················	
A	9020 hCoV-19/Georgia/Tb-1523/2020|EPI_ISL_420142|2020-0	····················	····················	····················	····················	····················	····················	
A	9021 hCoV-19/Norway/1917/2020|EPI_ISL_420143|2020-03-10	····················	····················	····················	····················	····················	····················	
A	9022 hCoV-19/Georgia/Tb-1679/2020|EPI_ISL_420144|2020-0	····················	····················	····················	····················	····················	····················	
A	9023 hCoV-19/Norway/1951/2020|EPI_ISL_420145|2020-03-09	····················	····················	····················	····················	····················	····················	
A	9024 hCoV-19/Norway/1955/2020|EPI_ISL_420146|2020-03-11	····················	····················	····················	····················	····················	····················	
A	9025 hCoV-19/Norway/1989/2020|EPI_ISL_420147|2020-03-10	····················	····················	····················	····················	····················	····················	
A	9026 hCoV-19/Norway/2084/2020|EPI_ISL_420150|2020-03-09	····················	····················	····················	····················	····················	····················	
A	9027 hCoV-19/Norway/2087/2020|EPI_ISL_420151|2020-03-17	····················	····················	····················	····················	····················	····················	
A	9028 hCoV-19/Norway/2088/2020|EPI_ISL_420152|2020-03-17	····················	·A··················	····················	····················	····················	····················	
A	9031 hCoV-19/England/SHEF-C07AD/2020|EPI_ISL_420155|202	····················	····················	····················	····················	····················	····················	
A	9032 hCoV-19/England/SHEF-C07DA/2020|EPI_ISL_420156|202	····················	····················	····················	····················	····················	····················	
A	9033 hCoV-19/England/SHEF-C079E/2020|EPI_ISL_420157|202	····················	····················	····················	····················	····················	····················	
A	9034 hCoV-19/England/SHEF-C07CB/2020|EPI_ISL_420158|202	····················	····················	····················	····················	····················	····················	
A	9035 hCoV-19/England/SHEF-C07BC/2020|EPI_ISL_420159|202	····················	····················	····················	····················	····················	····················	
A	9036 hCoV-19/England/SHEF-C07E9/2020|EPI_ISL_420160|202	····················	····················	····················	····················	····················	····················	
A	9037 hCoV-19/England/SHEF-C08E6/2020|EPI_ISL_420161|202	····················	····················	····················	····················	····················	····················	
A	9038 hCoV-19/England/SHEF-C0831/2020|EPI_ISL_420162|202	····················	····················	····················	····················	····················	····················	
A	9039 hCoV-19/England/SHEF-C086E/2020|EPI_ISL_420163|202	····················	····················	····················	····················	····················	····················	
A	9040 hCoV-19/England/SHEF-C08AA/2020|EPI_ISL_420164|202	····················	····················	····················	····················	····················	····················	
A	9041 hCoV-19/England/SHEF-C08C8/2020|EPI_ISL_420165|202	····················	····················	····················	····················	····················	····················	
A	9042 hCoV-19/England/SHEF-C0813/2020|EPI_ISL_420166|202	····················	····················	····················	····················	····················	····················	
A	9043 hCoV-19/England/SHEF-C0804/2020|EPI_ISL_420167|202	····················	····················	····················	····················	····················	····················	
A	9044 hCoV-19/England/SHEF-C087D/2020|EPI_ISL_420168|202	····················	····················	····················	····················	····················	····················	
A	9045 hCoV-19/England/SHEF-C0822/2020|EPI_ISL_420169|202	····················	···················A	····················	····················	····················	····················	1
A	9046 hCoV-19/England/SHEF-C0901/2020|EPI_ISL_420170|202	····················	····················	····················	····················	····················	····················	
A	9047 hCoV-19/England/SHEF-C08B9/2020|EPI_ISL_420171|202	····················	····················	····················	····················	····················	····················	
A	9048 hCoV-19/England/SHEF-C089B/2020|EPI_ISL_420172|202	····················	····················	····················	····················	····················	····················	
A	9049 hCoV-19/England/SHEF-C08F5/2020|EPI_ISL_420173|202	····················	····················	····················	····················	····················	····················	
A	9050 hCoV-19/England/SHEF-C088C/2020|EPI_ISL_420174|202	····················	····················	····················	····················	····················	····················	
A	9051 hCoV-19/England/SHEF-C07F8/2020|EPI_ISL_420175|202	····················	····················	····················	····················	····················	····················	
A	9053 hCoV-19/England/SHEF-C0840/2020|EPI_ISL_420177|202	····················	····················	····················	····················	····················	····················	
A	9054 hCoV-19/England/SHEF-C04A6/2020|EPI_ISL_420178|202	····················	····················	····················	····················	····················	····················	
A	9055 hCoV-19/England/SHEF-C042E/2020|EPI_ISL_420179|202	····················	····················	····················	····················	····················	····················	
A	9056 hCoV-19/England/SHEF-C02CA/2020|EPI_ISL_420180|202	····················	····················	····················	····················	····················	····················	
A	9057 hCoV-19/England/SHEF-C0321/2020|EPI_ISL_420181|202	····················	····················	····················	····················	····················	····················	
A	9058 hCoV-19/England/SHEF-C029D/2020|EPI_ISL_420182|202	····················	····················	····················	····················	····················	····················	
A	9060 hCoV-19/England/SHEF-C03C7/2020|EPI_ISL_420184|202	····················	····················	····················	····················	····················	····················	
A	9061 hCoV-19/England/SHEF-C038B/2020|EPI_ISL_420185|202	····················	····················	····················	····················	····················	····················	
A	9062 hCoV-19/England/SHEF-C0479/2020|EPI_ISL_420186|202	····················	····················	····················	····················	····················	····················	
A	9064 hCoV-19/England/SHEF-C0400/2020|EPI_ISL_420188|202	····················	····················	····················	····················	····················	····················	
A	9065 hCoV-19/England/SHEF-C0497/2020|EPI_ISL_420189|202	····················	····················	····················	····················	····················	····················	
A	9066 hCoV-19/England/SHEF-C03F4/2020|EPI_ISL_420190|202	····················	····················	····················	····················	····················	····················	
A	9067 hCoV-19/England/SHEF-C0260/2020|EPI_ISL_420191|202	····················	····················	····················	····················	··············N·····	···N················	
A	9068 hCoV-19/England/SHEF-C02BB/2020|EPI_ISL_420192|202	····················	····················	····················	····················	····················	····················	
A	9069 hCoV-19/England/SHEF-C044C/2020|EPI_ISL_420193|202	····················	····················	····················	····················	····················	····················	
A	9070 hCoV-19/England/SHEF-C028E/2020|EPI_ISL_420194|202	····················	····················	····················	····················	····················	····················	
A	9071 hCoV-19/England/SHEF-C039A/2020|EPI_ISL_420195|202	····················	····················	····················	····················	····················	····················	
A	9072 hCoV-19/England/SHEF-C0251/2020|EPI_ISL_420196|202	····················	····················	····················	····················	····················	····················	
A	9073 hCoV-19/England/SHEF-C03B8/2020|EPI_ISL_420197|202	····················	····················	····················	····················	····················	····················	
A	9074 hCoV-19/England/SHEF-C0206/2020|EPI_ISL_420198|202	····················	····················	····················	····················	····················	····················	
A	9075 hCoV-19/England/SHEF-C0233/2020|EPI_ISL_420199|202	····················	····················	····················	····················	····················	····················	
A	9076 hCoV-19/Portugal/PT0064/2020|EPI_ISL_421467|2020-0	····················	····················	····················	····················	····················	····················	
A	9077 hCoV-19/Portugal/PT0065/2020|EPI_ISL_421468|2020-0	····················	····················	····················	····················	····················	····················	
A	9078 hCoV-19/Portugal/PT0066/2020|EPI_ISL_421469|2020-0	····················	····················	····················	····················	····················	····················	
A	9079 hCoV-19/Portugal/PT0067/2020|EPI_ISL_421470|2020-0	····················	····················	····················	····················	····················	····················	
A	9080 hCoV-19/Portugal/PT0068/2020|EPI_ISL_421471|2020-0	····················	····················	····················	····················	····················	····················	
A	9081 hCoV-19/Portugal/PT0069/2020|EPI_ISL_421472|2020-0	····················	····················	····················	····················	····················	····················	

```
A    9082 hCoV-19/Portugal/PT0070/2020|EPI_ISL_421473|2020-0   ····················  ····················  ····················  ····················  ····················  ····················
A    9083 hCoV-19/Portugal/PT0071/2020|EPI_ISL_421474|2020-0   ····················  ····················  ····················  ····················  ····················  ····················
A    9084 hCoV-19/Portugal/PT0072/2020|EPI_ISL_421475|2020-0   ····················  ····················  ····················  ····················  ····················  ····················
A    9085 hCoV-19/Portugal/PT0073/2020|EPI_ISL_421476|2020-0   ····················  ····················  ····················  ····················  ····················  ····················
A    9086 hCoV-19/Portugal/PT0074/2020|EPI_ISL_421477|2020-0   ····················  ····················  ····················  ····················  ····················  ····················
A    9087 hCoV-19/Portugal/PT0075/2020|EPI_ISL_421478|2020-0   ····················  ····················  ····················  ····················  ····················  ····················
A    9088 hCoV-19/Portugal/PT0076/2020|EPI_ISL_421479|2020-0   ····················  ····················  ····················  ····················  ····················  ····················
A    9089 hCoV-19/Portugal/PT0077/2020|EPI_ISL_421480|2020-0   ····················  ····················  ····················  ····················  ····················  ····················
A    9090 hCoV-19/Portugal/PT0078/2020|EPI_ISL_421481|2020-0   ····················  ····················  ····················  ····················  ····················  ····················
A    9091 hCoV-19/Portugal/PT0079/2020|EPI_ISL_421482|2020-0   ····················  ····················  ····················  ····················  ····················  ····················
A    9092 hCoV-19/Portugal/PT0080/2020|EPI_ISL_421483|2020-0   ····················  ····················  ····················  ····················  ····················  ····················
A    9093 hCoV-19/Portugal/PT0081/2020|EPI_ISL_421484|2020-0   ····················  ····················  ····················  ····················  ····················  ····················
A    9094 hCoV-19/Portugal/PT0082/2020|EPI_ISL_421485|2020-0   ····················  ····················  ····················  ····················  ····················  ····················
A    9095 hCoV-19/Portugal/PT0083/2020|EPI_ISL_421486|2020-0   ····················  ····················  ····················  ····················  ····················  ····················
A    9096 hCoV-19/Portugal/PT0084/2020|EPI_ISL_421487|2020-0   ····················  ····················  ····················  ····················  ····················  ····················
A    9097 hCoV-19/Portugal/PT0085/2020|EPI_ISL_421488|2020-0   ····················  ····················  ····················  ····················  ····················  ····················
A    9098 hCoV-19/Portugal/PT0086/2020|EPI_ISL_421489|2020-0   ····················  ····················  ····················  ····················  ····················  ····················
A    9099 hCoV-19/Portugal/PT0087/2020|EPI_ISL_421490|2020-0   ····················  ····················  ····················  ····················  ····················  ····················
A    9100 hCoV-19/Portugal/PT0088/2020|EPI_ISL_421491|2020-0   ····················  ····················  ····················  ····················  ····················  ····················
A    9101 hCoV-19/Portugal/PT0089/2020|EPI_ISL_421492|2020-0   ····················  ····················  ····················  ····················  ····················  ····················
A    9102 hCoV-19/Portugal/PT0091/2020|EPI_ISL_421494|2020-0   ····················  ····················  ····················  ····················  ····················  ····················
A    9103 hCoV-19/Portugal/PT0092/2020|EPI_ISL_421495|2020-0   ····················  ····················  ····················  ····················  ····················  ····················
A    9104 hCoV-19/Portugal/PT0093/2020|EPI_ISL_421496|2020-0   ····················  ····················  ····················  ····················  ····················  ····················
A    9105 hCoV-19/Portugal/PT0094/2020|EPI_ISL_421497|2020-0   ····················  ····················  ····················  ····················  ····················  ····················
A    9106 hCoV-19/Portugal/PT0095/2020|EPI_ISL_421498|2020-0   ····················  ····················  ····················  ····················  ····················  ····················
A    9107 hCoV-19/Portugal/PT0096/2020|EPI_ISL_421499|2020-0   ····················  ····················  ····················  ····················  ····················  ····················
A    9108 hCoV-19/Netherlands/NA_37/2020|EPI_ISL_422798|2020   ····················  ····················  ····················  ····················  ····················  ····················
A    9109 hCoV-19/Netherlands/NA_38/2020|EPI_ISL_422799|2020   ····················  ····················  ····················  ····················  ····················  ····················
A    9110 hCoV-19/USA/NY-WCMP12C01P/2020|EPI_ISL_427600|2020   ····················  ····················  ····················  ····················  ····················  ····················
A    9111 hCoV-19/USA/NY-WCMP12C04P/2020|EPI_ISL_427601|2020   ····················  ····················  ····················  ····················  ····················  ····················
A    9112 hCoV-19/USA/NY-WCMP12C09P/2020|EPI_ISL_427602|2020   ····················  ····················  ····················  ····················  ····················  ····················
A    9113 hCoV-19/USA/NY-WCMP12D02P/2020|EPI_ISL_427603|2020   ····················  ····················  ····················  ····················  ····················  ····················
A    9114 hCoV-19/USA/NY-WCMP12D08P/2020|EPI_ISL_427604|2020   ····················  ····················  ····················  ····················  ····················  ····················
A    9115 hCoV-19/Australia/NSW51/2020|EPI_ISL_427660|2020-0   ····················  ····················  ····················  ····················  ····················  ····················
A    9116 hCoV-19/Australia/NSW111/2020|EPI_ISL_427661|2020-   ····················  ····················  ····················  ····················  ····················  ····················
A    9117 hCoV-19/Australia/NSW59/2020|EPI_ISL_427662|2020-0   ····················  ····················  ····················  ····················  ····················  ····················
A    9118 hCoV-19/Australia/NSW60/2020|EPI_ISL_427663|2020-0   ····················  ····················  ····················  ····················  ····················  ····················
A    9119 hCoV-19/Australia/NSW63/2020|EPI_ISL_427664|2020-0   ····················  ····················  ····················  ····················  ····················  ····················
A    9120 hCoV-19/Australia/NSW218/2020|EPI_ISL_427665|2020-   ····················  ····················  ····················  ····················  ····················  ····················
A    9121 hCoV-19/Australia/NSW112/2020|EPI_ISL_427666|2020-   ····················  ····················  ····················  ····················  ····················  ····················
A    9122 hCoV-19/Australia/NSW177/2020|EPI_ISL_427667|2020-   ····················  ····················  ····················  ····················  ····················  ····················
A    9123 hCoV-19/Australia/NSW174/2020|EPI_ISL_427668|2020-   ····················  ····················  ····················  ····················  ····················  ····················
A    9124 hCoV-19/Australia/NSW119/2020|EPI_ISL_427669|2020-   ····················  ····················  ····················  ····················  ····················  ····················
A    9125 hCoV-19/Russia/Moscow-80112/2020|EPI_ISL_428900|20   ····················  ····················  ····················  ····················  ····················  ····················
A    9126 hCoV-19/Crimea/SRC-80603/2020|EPI_ISL_428901|2020-   ····················  ····················  ····················  ····················  ····················  ····················
A    9127 hCoV-19/Russia/Orenburg-80305/2020|EPI_ISL_428902|   ····················  ····················  ····················  ····················  ····················  ····················
A    9128 hCoV-19/Russia/Primorskiy-79001/2020|EPI_ISL_42890   ····················  ····················  ····················  ····················  ····················  ····················
A    9129 hCoV-19/Russia/Vector_77628/2020|EPI_ISL_428904|20   ····················  ····················  ····················  ····················  ····················  ····················
A    9130 hCoV-19/Russia/Moscow-80402/2020|EPI_ISL_428905|20   ····················  ····················  ····················  ····················  ····················  ····················
A    9131 hCoV-19/Russia/Buryatia-84504/2020|EPI_ISL_428906|   ····················  ····················  ····················  ····················  ····················  ····················
A    9132 hCoV-19/Russia/Sakha-89702/2020|EPI_ISL_428907|202   ····················  ····················  ····················  ····················  ····················  ····················
A    9133 hCoV-19/Russia/Pskov-83602/2020|EPI_ISL_428908|202   ····················  ····················  ····················  ····················  ····················  ····················
A    9134 hCoV-19/Russia/Chechenya-83801/2020|EPI_ISL_428909   ····················  ····················  ····················  ····················  ····················  ····················
A    9135 hCoV-19/Russia/Khabarovsk-88201/2020|EPI_ISL_42891   ····················  ····················  ····················  ····················  ····················  ····················
A    9136 hCoV-19/Russia/Buryatia-88903/2020|EPI_ISL_428911|   ····················  ····················  ····················  ····················  ····················  ····················
A    9137 hCoV-19/Russia/Sakhalin-84401/2020|EPI_ISL_428912|   ····················  ····················  ····················  ····················  ····················  ····················
A    9138 hCoV-19/Russia/Buryatia-84508/2020|EPI_ISL_428913|   ····················  ····················  ····················  ····················  ····················  ····················
A    9139 hCoV-19/Russia/Buryatia-87104/2020|EPI_ISL_428914|   ····················  ····················  ····················  ····················  ····················  ····················
A    9140 hCoV-19/Russia/Sverdlovsk-82904/2020|EPI_ISL_42891   ····················  ····················  ····················  ····················  ····················  ····················
A    9141 hCoV-19/Russia/Chechenya-83803/2020|EPI_ISL_428916   ····················  ····················  ····················  ····················  ····················  ····················
A    9142 hCoV-19/Russia/Tatarstan-82208/2020|EPI_ISL_428917   ····················  ····················  ····················  ····················  ····················  ····················
A    9143 hCoV-19/Russia/Novosibirsk-84101/2020|EPI_ISL_4289   ····················  ····················  ····················  ····················  ····················  ····················
A    9144 hCoV-19/Russia/Buryatia-87106/2020|EPI_ISL_428919|   ····················  ····················  ····················  ····················  ····················  ····················
A    9145 hCoV-19/Russia/Buryatia-87105/2020|EPI_ISL_428920|   ····················  ····················  ····················  ····················  ····················  ····················
A    9146 hCoV-19/Russia/Tver-72601/2020|EPI_ISL_428921|2020   ····················  ····················  ····················  ····················  ····················  ····················
A    9147 hCoV-19/Russia/Sverdlovsk-86704/2020|EPI_ISL_42892   ····················  ····················  ····················  ····················  ····················  ····················
A    9148 hCoV-19/Russia/Sverdlovsk-84302/2020|EPI_ISL_42892   ····················  ····················  ····················  ····················  ····················  ····················
A    9149 hCoV-19/Russia/Chelyabinsk-85311/2020|EPI_ISL_4289   ····················  ····················  ····················  ····················  ···T················  ····················
A    9150 hCoV-19/Poland/PL_P2/2020|EPI_ISL_428925|2020-03-2   ····················  ····················  ····················  ····················  ····················  ····················
A    9151 hCoV-19/Poland/PL_P3/2020|EPI_ISL_428926|2020-03-2   ····················  ····················  ····················  ····················  ····················  ····················
A    9152 hCoV-19/Poland/PL_P4/2020|EPI_ISL_428927|2020-03-2   ····················  ····················  ····················  ····················  ····················  ····················
A    9153 hCoV-19/Poland/PL_P5/2020|EPI_ISL_428928|2020-03-3   ····················  ····················  ····················  ····················  ····················  ····················
A    9154 hCoV-19/Poland/PL_P6/2020|EPI_ISL_428929|2020-03-2   ····················  ····················  ····················  ····················  ····················  ····················
A    9155 hCoV-19/Poland/PL_P7/2020|EPI_ISL_428930|2020-03-2   ····················  ····················  ····················  ····················  ····················  ····················
A    9156 hCoV-19/Poland/PL_P8/2020|EPI_ISL_428931|2020-03-2   ····················  ····················  ····················  ····················  ····················  ····················
A    9157 hCoV-19/Poland/PL_P9/2020|EPI_ISL_428932|2020-03-2   ····················  ····················  ····················  ····················  ····················  ····················
A    9158 hCoV-19/USA/WI-UW-259/2020|EPI_ISL_428935|2020-04-   ····················  ····················  ····················  ····················  ····················  ····················
A    9159 hCoV-19/USA/CA-CZB0104/2020|EPI_ISL_428990|2020-03   ····················  ····················  ····················  ····················  ····················  ····················
```

A	9160	hCoV-19/USA/CA-CZB071/2020|EPI_ISL_428991|2020-03-	····················	····················	·····················	····················	····················	····················
A	9161	hCoV-19/USA/CA-CZB0138/2020|EPI_ISL_428992|2020-04	····················	····················	·····················	····················	····················	····················
A	9162	hCoV-19/USA/CA-CZB0157/2020|EPI_ISL_428993|2020-04	····················	····················	·····················	····················	····················	····················
A	9163	hCoV-19/USA/CA-CZB0703/2020|EPI_ISL_428994|2020-03	····················	····················	·····················	····················	····················	····················
A	9164	hCoV-19/USA/CA-CZB0133/2020|EPI_ISL_428995|2020-04	····················	····················	·····················	····················	····················	····················
A	9165	hCoV-19/USA/CA-CZB077/2020|EPI_ISL_428996|2020-03-	····················	····················	·····················	····················	····················	····················
A	9166	hCoV-19/USA/CA-CZB015b/2020|EPI_ISL_428997|2020-03	····················	····················	·····················	····················	····················	····················
A	9167	hCoV-19/USA/CA-CZB084/2020|EPI_ISL_428998|2020-03-	····················	····················	·····················	····················	····················	····················
A	9168	hCoV-19/USA/CA-CZB069/2020|EPI_ISL_428999|2020-03-	····················	····················	·····················	····················	····················	····················
B	1	hCoV-19/USA/WA-S259/2020|EPI_ISL_430113|2020-03-04	····················	····················	·····················	····················	····················	····················
B	2	hCoV-19/USA/WA-S260/2020|EPI_ISL_430114|2020-03-12	····················	····················	·····················	····················	····················	····················
B	3	hCoV-19/USA/WA-S261/2020|EPI_ISL_430115|2020-03-30	····················	····················	·····················	····················	····················	····················
B	4	hCoV-19/USA/WA-S262/2020|EPI_ISL_430116|2020-03-16	····················	····················	·····················	····················	····················	····················
B	5	hCoV-19/USA/WA-S263/2020|EPI_ISL_430117|2020-03-05	····················	····················	·····················	····················	····················	····················
B	6	hCoV-19/USA/WA-S264/2020|EPI_ISL_430118|2020-03-03	····················	····················	·····················	····················	····················	····················
B	7	hCoV-19/USA/WA-S265/2020|EPI_ISL_430119|2020-03-05	····················	····················	·····················	····················	····················	····················
B	8	hCoV-19/USA/WA-S267/2020|EPI_ISL_430121|2020-03-31	····················	····················	·····················	····················	····················	····················
B	9	hCoV-19/USA/WA-S268/2020|EPI_ISL_430122|2020-03-05	····················	····················	·····················	····················	····················	····················
B	10	hCoV-19/USA/WA-S269/2020|EPI_ISL_430123|2020-03-10	····················	····················	·····················	····················	····················	····················
B	11	hCoV-19/USA/WA-S270/2020|EPI_ISL_430124|2020-03-06	····················	····················	·····················	····················	····················	····················
B	12	hCoV-19/USA/WA-S271/2020|EPI_ISL_430125|2020-03-31	····················	····················	·····················	····················	····················	····················
B	13	hCoV-19/USA/WA-S272/2020|EPI_ISL_430126|2020-02-26	····················	····················	·····················	····················	····················	····················
B	14	hCoV-19/USA/WA-S273/2020|EPI_ISL_430127|2020-03-12	····················	····················	·····················	····················	····················	····················
B	15	hCoV-19/USA/WA-S274/2020|EPI_ISL_430128|2020-03-08	····················	····················	·····················	····················	····················	····················
B	16	hCoV-19/USA/WA-S275/2020|EPI_ISL_430129|2020-03-06	····················	····················	·····················	····················	····················	····················
B	17	hCoV-19/USA/WA-S276/2020|EPI_ISL_430130|2020-04-01	····················	····················	·····················	····················	····················	····················
B	18	hCoV-19/USA/WA-S277/2020|EPI_ISL_430131|2020-02-22	····················	····················	·····················	····················	····················	····················
B	19	hCoV-19/USA/WA-S278/2020|EPI_ISL_430132|2020-03-17	····················	····················	·····················	····················	····················	····················
B	20	hCoV-19/USA/WA-S279/2020|EPI_ISL_430133|2020-03-04	····················	····················	·····················	····················	····················	····················
B	21	hCoV-19/USA/WA-S280/2020|EPI_ISL_430134|2020-03-09	····················	····················	·····················	····················	····················	····················
B	22	hCoV-19/USA/WA-S281/2020|EPI_ISL_430135|2020-02-29	····················	····················	·····················	····················	····················	····················
B	23	hCoV-19/USA/WA-S282/2020|EPI_ISL_430136|2020-03-10	····················	····················	·····················	····················	····················	····················
B	24	hCoV-19/USA/WA-S283/2020|EPI_ISL_430137|2020-03-21	····················	····················	·····················	····················	····················	····················
B	25	hCoV-19/USA/WA-S286/2020|EPI_ISL_430140|2020-03-15	····················	····················	·····················	····················	····················	····················
B	26	hCoV-19/USA/WA-S287/2020|EPI_ISL_430141|2020-03-06	····················	····················	·····················	····················	····················	····················
B	27	hCoV-19/USA/WA-S288/2020|EPI_ISL_430142|2020-03-06	····················	····················	·····················	····················	····················	····················
B	28	hCoV-19/USA/WA-S289/2020|EPI_ISL_430143|2020-03-23	····················	····················	·····················	····················	····················	····················
B	29	hCoV-19/USA/WA-S292/2020|EPI_ISL_430146|2020-03-05	····················	····················	·····················	····················	····················	····················
B	30	hCoV-19/USA/WA-S293/2020|EPI_ISL_430147|2020-04-01	····················	····················	·····················	····················	····················	····················
B	31	hCoV-19/USA/WA-S294/2020|EPI_ISL_430148|2020-03-23	····················	····················	·····················	····················	····················	····················
B	32	hCoV-19/USA/WA-S295/2020|EPI_ISL_430149|2020-03-31	····················	····················	·····················	····················	····················	····················
B	33	hCoV-19/USA/WA-S296/2020|EPI_ISL_430150|2020-03-13	····················	····················	·····················	A···················	····················	····················
B	34	hCoV-19/USA/WA-S297/2020|EPI_ISL_430151|2020-03-13	····················	····················	·····················	····················	····················	····················
B	35	hCoV-19/USA/WA-S298/2020|EPI_ISL_430152|2020-03-05	····················	····················	·····················	····················	····················	····················
B	36	hCoV-19/USA/WA-S299/2020|EPI_ISL_430153|2020-03-31	····················	····················	·····················	····················	····················	····················
B	37	hCoV-19/USA/WA-S300/2020|EPI_ISL_430154|2020-03-25	····················	····················	·····················	····················	····················	····················
B	38	hCoV-19/USA/WA-S301/2020|EPI_ISL_430155|2020-03-14	····················	····················	·····················	····················	····················	····················
B	40	hCoV-19/USA/WA-S123/2020|EPI_ISL_430160|2020-02-29	····················	····················	·····················	····················	····················	····················
B	41	hCoV-19/USA/WA-S124/2020|EPI_ISL_430161|2020-03-13	····················	····················	·····················	····················	····················	····················
B	42	hCoV-19/USA/WA-S125/2020|EPI_ISL_430162|2020-03-13	····················	····················	·····················	····················	····················	····················
B	43	hCoV-19/USA/WA-S126/2020|EPI_ISL_430163|2020-03-13	····················	····················	·····················	····················	····················	····················
B	44	hCoV-19/USA/WA-S127/2020|EPI_ISL_430164|2020-03-13	····················	····················	·····················	····················	····················	····················
B	45	hCoV-19/USA/WA-S128/2020|EPI_ISL_430165|2020-03-13	····················	····················	·····················	····················	····················	····················
B	46	hCoV-19/USA/WA-S129/2020|EPI_ISL_430166|2020-03-13	····················	····················	·····················	····················	····················	····················
B	47	hCoV-19/USA/WA-S130/2020|EPI_ISL_430167|2020-03-13	····················	····················	·····················	····················	····················	····················
B	48	hCoV-19/USA/WA-S131/2020|EPI_ISL_430168|2020-03-13	····················	····················	·····················	····················	····················	····················
B	49	hCoV-19/USA/WA-S132/2020|EPI_ISL_430169|2020-03-13	····················	····················	·····················	····················	····················	····················
B	50	hCoV-19/USA/WA-S133/2020|EPI_ISL_430170|2020-03-13	····················	····················	·····················	····················	····················	····················
B	51	hCoV-19/USA/WA-S134/2020|EPI_ISL_430171|2020-03-13	····················	····················	·····················	····················	····················	····················
B	52	hCoV-19/USA/WA-S135/2020|EPI_ISL_430172|2020-03-13	····················	····················	·····················	····················	····················	····················
B	53	hCoV-19/USA/WA-S136/2020|EPI_ISL_430173|2020-03-13	····················	····················	·····················	····················	····················	····················
B	54	hCoV-19/USA/WA-S137/2020|EPI_ISL_430174|2020-03-13	····················	····················	·····················	····················	····················	····················
B	55	hCoV-19/USA/WA-S138/2020|EPI_ISL_430175|2020-03-12	····················	····················	·····················	····················	····················	····················
B	56	hCoV-19/USA/WA-S139/2020|EPI_ISL_430176|2020-03-12	····················	····················	·····················	····················	····················	····················
B	57	hCoV-19/USA/WA-S140/2020|EPI_ISL_430177|2020-03-12	····················	····················	·····················	····················	····················	····················
B	58	hCoV-19/USA/WA-S141/2020|EPI_ISL_430178|2020-03-13	····················	····················	·····················	····················	····················	····················
B	59	hCoV-19/England/NOTT-10FC84/2020|EPI_ISL_432706|20	····················	····················	·····················	····················	····················	····················
B	60	hCoV-19/England/SHEF-C18E5/2020|EPI_ISL_432732|202	····················	····················	·····················	····················	····················	····················
B	61	hCoV-19/England/SHEF-C1B64/2020|EPI_ISL_432734|202	····················	····················	·····················	····················	····················	····················
B	62	hCoV-19/England/SHEF-C2222/2020|EPI_ISL_432742|202	····················	····················	·····················	····················	····················	····················
B	63	hCoV-19/England/SHEF-C2310/2020|EPI_ISL_432744|202	····················	····················	·····················	····················	····················	····················
B	64	hCoV-19/England/SHEF-C2626/2020|EPI_ISL_432746|202	····················	····················	·····················	····················	····················	····················
B	65	hCoV-19/England/SHEF-C2CF7/2020|EPI_ISL_432753|202	····················	····················	·····················	····················	····················	····················
B	66	hCoV-19/England/SHEF-C3115/2020|EPI_ISL_432762|202	····················	····················	·····················	····················	····················	····················
B	67	hCoV-19/England/SHEF-C3212/2020|EPI_ISL_432766|202	····················	····················	·····················	····················	····················	····················
B	68	hCoV-19/England/SHEF-C32C7/2020|EPI_ISL_432768|202	····················	····················	·····················	····················	····················	····················
B	69	hCoV-19/England/SHEF-C384D/2020|EPI_ISL_432777|202	····················	····················	·····················	····················	····················	····················
B	70	hCoV-19/England/SHEF-C3CC9/2020|EPI_ISL_432780|202	····················	····················	·····················	····················	····················	····················

B	71	hCoV-19/England/SHEF-C426C/2020|EPI_ISL_432786|202	....	....	....	....	....	....
B	72	hCoV-19/England/SHEF-C4493/2020|EPI_ISL_432788|202	....	....	....	....	....	....
B	73	hCoV-19/England/SHEF-C45BE/2020|EPI_ISL_432790|202	....	....	....	....	....	....
B	74	hCoV-19/England/SHEF-C51E6/2020|EPI_ISL_432799|202	....	....	....	....	....	....
B	75	hCoV-19/Spain/VC-FISABIO-187/2020|EPI_ISL_436282|2	....	....	....	....	....	....
B	76	hCoV-19/Spain/VC-FISABIO-188/2020|EPI_ISL_436283|2	....	....	....	....	....	....
B	77	hCoV-19/Spain/VC-FISABIO-189/2020|EPI_ISL_436284|2	....	....	....	....	....	....
B	78	hCoV-19/Spain/VC-FISABIO-190/2020|EPI_ISL_436285|2	....	....	....	....	....	....
B	79	hCoV-19/Spain/VC-FISABIO-192/2020|EPI_ISL_436287|2	....	....	....	....	....	....
B	80	hCoV-19/Spain/VC-FISABIO-193/2020|EPI_ISL_436288|2	....	....	....	....	....	....
B	81	hCoV-19/Spain/VC-FISABIO-195/2020|EPI_ISL_436290|2	....	....	....	....	....	....
B	82	hCoV-19/Spain/VC-FISABIO-196/2020|EPI_ISL_436291|2	....	....	....	....	....	....
B	83	hCoV-19/Spain/VC-FISABIO-197/2020|EPI_ISL_436292|2	....	....	....	....	....	....
B	84	hCoV-19/Spain/VC-FISABIO-200/2020|EPI_ISL_436295|2	....	....	....	....	....	....
B	86	hCoV-19/England/CAMB-7BF30/2020|EPI_ISL_442101|202	....	....	....	....	....	....
B	87	hCoV-19/England/CAMB-7C89E/2020|EPI_ISL_442102|202	....	....	....	....	....	....
B	88	hCoV-19/England/CAMB-7C782/2020|EPI_ISL_442103|202	....	....	....	....	....	....
B	89	hCoV-19/England/CAMB-7BDBE/2020|EPI_ISL_442104|202	....	....	....	....	....	....
B	90	hCoV-19/England/CAMB-7BBC3/2020|EPI_ISL_442105|202	....	....	....	....	....	....
B	91	hCoV-19/England/CAMB-7D666/2020|EPI_ISL_442106|202	....	....	....	....	....	....
B	92	hCoV-19/England/CAMB-7BD18/2020|EPI_ISL_442107|202	....	....	....	....	....	....
B	93	hCoV-19/England/CAMB-7D138/2020|EPI_ISL_442108|202	....	....	....	....	....	....
B	94	hCoV-19/England/CAMB-7BE42/2020|EPI_ISL_442109|202	....	....	....	....	....	....
B	95	hCoV-19/England/CAMB-7D03B/2020|EPI_ISL_442111|202	....	....	....	....	....	....
B	96	hCoV-19/England/CAMB-7BBE1/2020|EPI_ISL_442112|202	....	....	....	....	....	....
B	97	hCoV-19/England/CAMB-7D5C3/2020|EPI_ISL_442113|202	....	....	....	....	....	....
B	98	hCoV-19/England/CAMB-7C9B9/2020|EPI_ISL_442114|202	....	....	....	....	....	....
B	99	hCoV-19/England/CAMB-7C9F5/2020|EPI_ISL_442116|202	....	....	....	....	....	....
B	100	hCoV-19/England/CAMB-7B8DB/2020|EPI_ISL_442117|202	....	....	....	....	....	....
B	101	hCoV-19/England/CAMB-7C96E/2020|EPI_ISL_442120|202	....	....	....	....	....	....
B	102	hCoV-19/England/CAMB-7CB77/2020|EPI_ISL_442121|202	....	....	....	....	....	....
B	103	hCoV-19/England/CAMB-7BD54/2020|EPI_ISL_442122|202	....	....	....	....	....	....
B	104	hCoV-19/England/CAMB-7D262/2020|EPI_ISL_442123|202	....	....	....	....	....	....
B	105	hCoV-19/England/CAMB-7C8DA/2020|EPI_ISL_442124|202	....	....	....	....	....	....
B	106	hCoV-19/England/CAMB-7E094/2020|EPI_ISL_442125|202	....	.A..................	....	....	....	....
B	107	hCoV-19/England/CAMB-7BE33/2020|EPI_ISL_442126|202	....	....	....	....	....	....
B	108	hCoV-19/England/CAMB-7D60C/2020|EPI_ISL_442129|202	....	....	....	....	....	....
B	109	hCoV-19/England/CAMB-7BB5A/2020|EPI_ISL_442130|202	....	....	....	....	....	....
B	110	hCoV-19/England/CAMB-7E0A3/2020|EPI_ISL_442133|202	....	....	....	....	....	....
B	111	hCoV-19/England/CAMB-7BDEB/2020|EPI_ISL_442134|202	....	....	....	....	....	....
B	112	hCoV-19/England/CAMB-7C9D7/2020|EPI_ISL_442136|202	....	....	....	....	....	....
B	113	hCoV-19/England/CAMB-7BCDF/2020|EPI_ISL_442139|202	....	....	....	....	....	....
B	114	hCoV-19/England/CAMB-7CAD4/2020|EPI_ISL_442140|202	....	....	....	....	....	....
B	115	hCoV-19/England/CAMB-7D174/2020|EPI_ISL_442142|202	....	....	....	....	....	....
B	116	hCoV-19/England/CAMB-7CBFF/2020|EPI_ISL_442143|202	....	....	....	....	....	....
B	117	hCoV-19/England/CAMB-7BCFD/2020|EPI_ISL_442144|202	....	....	....	....	....	....
B	118	hCoV-19/England/CAMB-7BC84/2020|EPI_ISL_442146|202	....	....	....	....	....	....
B	119	hCoV-19/England/CAMB-7CA2F/2020|EPI_ISL_442147|202	....	....	....	....	....	....
B	120	hCoV-19/England/CAMB-7D244/2020|EPI_ISL_442149|202	....	....	....	....	....	....
B	121	hCoV-19/England/CAMB-7CC74/2020|EPI_ISL_442150|202	....	....	....	....	....	....
B	122	hCoV-19/England/CAMB-7BED9/2020|EPI_ISL_442154|202	....	....	....	....	....	....
B	123	hCoV-19/England/CAMB-7CC92/2020|EPI_ISL_442157|202	....	....	....	....	....	....
B	124	hCoV-19/England/CAMB-830AB/2020|EPI_ISL_443402|202	....	....	....	....	....	....
B	125	hCoV-19/England/CAMB-843DE/2020|EPI_ISL_443403|202	....	....	....	....	....	....
B	126	hCoV-19/England/CAMB-827F2/2020|EPI_ISL_443404|202	....	....	....	....	....	....
B	127	hCoV-19/England/CAMB-8459C/2020|EPI_ISL_443406|202	....	....	....	....	....	....
B	128	hCoV-19/England/CAMB-1AA2D4/2020|EPI_ISL_443408|20	....	....	....	....	....	....
B	129	hCoV-19/England/CAMB-838B2/2020|EPI_ISL_443409|202	....	....	....	....	....	....
B	130	hCoV-19/England/CAMB-1AA3FF/2020|EPI_ISL_443410|20	....	....	....	....	....	....
B	131	hCoV-19/England/CAMB-1AA395/2020|EPI_ISL_443411|20	....	....	....	....	....	....
B	132	hCoV-19/England/CAMB-1AA2C5/2020|EPI_ISL_443414|20	....	....	....	....	....	....
B	133	hCoV-19/England/CAMB-833DF/2020|EPI_ISL_443415|202	....	....	....	....	....	....
B	134	hCoV-19/England/CAMB-833A2/2020|EPI_ISL_443416|202	....	....	....	....	....	....
B	135	hCoV-19/England/CAMB-828D1/2020|EPI_ISL_443417|202	....	....	....	....	....	....
B	136	hCoV-19/England/CAMB-845D8/2020|EPI_ISL_443419|202	....	....	....	....	....	....
B	137	hCoV-19/England/CAMB-1AA3D1/2020|EPI_ISL_443421|20	....	....	....	....	....	....
B	138	hCoV-19/England/CAMB-82F72/2020|EPI_ISL_443425|202	....	....	....	....	....	....
B	139	hCoV-19/England/CAMB-82552/2020|EPI_ISL_443426|202	....	....	....	....	....	....
B	140	hCoV-19/England/CAMB-82D3C/2020|EPI_ISL_443427|202	....	....	....	....	....	....
B	141	hCoV-19/England/CAMB-8225B/2020|EPI_ISL_443428|202	....	....	....	....	....	....
B	142	hCoV-19/England/CAMB-831E4/2020|EPI_ISL_443429|202	....	....	....	....	....	....
B	143	hCoV-19/England/CAMB-84444/2020|EPI_ISL_443434|202	....	....	....	....	....	....
B	144	hCoV-19/England/CAMB-83788/2020|EPI_ISL_443436|202	....	....	....	....	....	....
B	145	hCoV-19/England/CAMB-83005/2020|EPI_ISL_443437|202	....	....	....	....	....	....
B	146	hCoV-19/England/CAMB-83366/2020|EPI_ISL_443440|202	....	....	....	....	....	....
B	147	hCoV-19/England/CAMB-1AA4ED/2020|EPI_ISL_443441|20	....	....	....	....	....	....
B	148	hCoV-19/England/CAMB-83560/2020|EPI_ISL_443444|202	....	....	....	....	....	....
B	149	hCoV-19/England/CAMB-843CF/2020|EPI_ISL_443445|202	....	....	....	....	....	....

B	150 hCoV-19/England/CAMB-83797/2020|EPI_ISL_443446|202	....	....	....	....	....	....
B	151 hCoV-19/England/CAMB-834DC/2020|EPI_ISL_443447|202	....	....	....	....	....	....
B	152 hCoV-19/England/CAMB-1AA3E0/2020|EPI_ISL_443448|20	....	....	....	....	....	....
B	153 hCoV-19/England/CAMB-82482/2020|EPI_ISL_443450|202	....	....	....	....	....	....
B	154 hCoV-19/England/CAMB-8224C/2020|EPI_ISL_443452|202	....	......R.............	....	....	....	....
B	155 hCoV-19/England/CAMB-83876/2020|EPI_ISL_443453|202	....	....	....	....	....	....
B	156 hCoV-19/England/CAMB-8422C/2020|EPI_ISL_443454|202	....	....	....	....	....	....
B	157 hCoV-19/England/CAMB-83515/2020|EPI_ISL_443455|202	....	....	....	....	....	....
B	158 hCoV-19/England/CAMB-826C8/2020|EPI_ISL_443456|202	....	....	....	....	....	....
B	159 hCoV-19/England/CAMB-1AA562/2020|EPI_ISL_443457|20	....	....	....	....	....	....
B	160 hCoV-19/England/CAMB-82491/2020|EPI_ISL_443458|202	....	....	....	....	....	....
B	161 hCoV-19/England/CAMB-82507/2020|EPI_ISL_443459|202	....	....	....	....	....	....
B	162 hCoV-19/England/CAMB-842D1/2020|EPI_ISL_443460|202	....	....	....	....	....	....
B	163 hCoV-19/England/CAMB-8233A/2020|EPI_ISL_443461|202	....	....	....	....	....	....
B	164 hCoV-19/England/CAMB-8358E/2020|EPI_ISL_443463|202	....	....	....	....	....	....
B	165 hCoV-19/England/CAMB-827A7/2020|EPI_ISL_443464|202	....	....	....	....	....	....
B	166 hCoV-19/England/CAMB-823EF/2020|EPI_ISL_443465|202	....	....	....	....	....	....
B	167 hCoV-19/England/CAMB-1AA474/2020|EPI_ISL_443466|20	....	.A..................	....	....	....	....
B	168 hCoV-19/England/CAMB-82D1E/2020|EPI_ISL_443467|202	....	....	....	....	....	....
B	169 hCoV-19/England/CAMB-84462/2020|EPI_ISL_443469|202	....	....	....	....	....	....
B	170 hCoV-19/England/CAMB-837D3/2020|EPI_ISL_443470|202	....	....	....	....	....	....
B	171 hCoV-19/England/CAMB-824EC/2020|EPI_ISL_443472|202	....	....	....	....	....	....
B	172 hCoV-19/England/CAMB-833FD/2020|EPI_ISL_443473|202	....	....	....	....	....	....
B	173 hCoV-19/England/CAMB-8374C/2020|EPI_ISL_443474|202	....	....	....	....	....	....
B	174 hCoV-19/England/CAMB-842A4/2020|EPI_ISL_443476|202	....	....	....	....	....	....
B	175 hCoV-19/England/CAMB-8376A/2020|EPI_ISL_443477|202	....	....	....	....	....	....
B	176 hCoV-19/England/CAMB-833B1/2020|EPI_ISL_443478|202	....	....	....	....	....	....
B	177 hCoV-19/England/CAMB-822E2/2020|EPI_ISL_443481|202	....	....	....	....	....	....
B	178 hCoV-19/England/CAMB-82FDC/2020|EPI_ISL_443482|202	....	....	....	....	....	....
B	179 hCoV-19/England/CAMB-82D0F/2020|EPI_ISL_443483|202	....	....	....	....	....	....
B	180 hCoV-19/England/CAMB-8223D/2020|EPI_ISL_443487|202	....	....	....	....	....	....
B	181 hCoV-19/England/CAMB-83472/2020|EPI_ISL_443488|202	....	....	....	....	....	....
B	182 hCoV-19/USA/NY-NYUMC593/2020|EPI_ISL_444710|2020-0	....	....	....	....	....	....
B	183 hCoV-19/USA/NY-NYUMC594/2020|EPI_ISL_444711|2020-0	....	....	....	....	....	....
B	184 hCoV-19/USA/NY-NYUMC595/2020|EPI_ISL_444712|2020-0	....	....	....	....	....	....
B	185 hCoV-19/USA/NY-NYUMC596/2020|EPI_ISL_444713|2020-0	....	....	....	....	....	....
B	186 hCoV-19/USA/NY-NYUMC597/2020|EPI_ISL_444714|2020-0	....	....	....	....	....	....
B	187 hCoV-19/USA/NY-NYUMC598/2020|EPI_ISL_444715|2020-0	....	....	....	....	....	....
B	188 hCoV-19/USA/NY-NYUMC599/2020|EPI_ISL_444716|2020-0	....	....	....	....	....	....
B	189 hCoV-19/USA/NY-NYUMC600/2020|EPI_ISL_444717|2020-0	....	....	....	....	....	....
B	190 hCoV-19/USA/NY-NYUMC601/2020|EPI_ISL_444718|2020-0	....	....	....	....	....	....
B	191 hCoV-19/USA/NY-NYUMC602/2020|EPI_ISL_444719|2020-0	....	....	....	....	....	....
B	192 hCoV-19/USA/NY-NYUMC603/2020|EPI_ISL_444720|2020-0	....	....	....	....	....	....
B	193 hCoV-19/USA/NY-NYUMC605/2020|EPI_ISL_444722|2020-0	....	....	....	....	....	....
B	194 hCoV-19/USA/NY-NYUMC606/2020|EPI_ISL_444723|2020-0	....	....	....	....	....	....
B	195 hCoV-19/USA/NY-NYUMC607/2020|EPI_ISL_444724|2020-0	....	....	....	....	....	....
B	196 hCoV-19/USA/NY-NYUMC612/2020|EPI_ISL_444729|2020-0	....	....	....	....	....	....
B	197 hCoV-19/USA/NY-NYUMC613/2020|EPI_ISL_444730|2020-0	....	....	....	....	....	....
B	198 hCoV-19/USA/NY-NYUMC614/2020|EPI_ISL_444731|2020-0	....	....	....	....	....	....
B	199 hCoV-19/USA/NY-NYUMC615/2020|EPI_ISL_444732|2020-0	....	....	....	....	....	....
B	200 hCoV-19/USA/NY-NYUMC616/2020|EPI_ISL_444733|2020-0	....	....	....	....	....	....
B	201 hCoV-19/USA/NY-NYUMC617/2020|EPI_ISL_444734|2020-0	....	....	....	....	....	....
B	202 hCoV-19/USA/NY-NYUMC618/2020|EPI_ISL_444735|2020-0	....	....	....	....	....	....
B	203 hCoV-19/USA/NY-NYUMC619/2020|EPI_ISL_444736|2020-0	....	....	....	....	....	....
B	204 hCoV-19/USA/NY-NYUMC620/2020|EPI_ISL_444737|2020-0	....	....A...............	....	....	....	....
B	205 hCoV-19/USA/NY-NYUMC621/2020|EPI_ISL_444738|2020-0	....	....	....	....	....	....
B	206 hCoV-19/USA/NY-NYUMC622/2020|EPI_ISL_444739|2020-0	....	....	....	....	....	....
B	207 hCoV-19/USA/NY-NYUMC623/2020|EPI_ISL_444740|2020-0	....	....	....	....	....	....
B	208 hCoV-19/USA/NY-NYUMC624/2020|EPI_ISL_444741|2020-0	....	....	....	....	....	....
B	209 hCoV-19/USA/NJ-NYUMC625/2020|EPI_ISL_444742|2020-0	....	....	....	....	....	....
B	210 hCoV-19/USA/NY-NYUMC626/2020|EPI_ISL_444743|2020-0	....	....	....	....	....	....
B	211 hCoV-19/USA/NJ-NYUMC627/2020|EPI_ISL_444744|2020-0	....	....	....	....	....	....
B	212 hCoV-19/USA/NY-NYUMC628/2020|EPI_ISL_444745|2020-0	....	....	....	....	....	....
B	213 hCoV-19/USA/NY-NYUMC629/2020|EPI_ISL_444746|2020-0	....	....	....	....	....	....
B	214 hCoV-19/USA/NY-NYUMC630/2020|EPI_ISL_444747|2020-0	....	....	....	....	....	....
B	215 hCoV-19/USA/NY-NYUMC631/2020|EPI_ISL_444748|2020-0	....	....	....	....	....	....
B	216 hCoV-19/USA/NY-NYUMC632/2020|EPI_ISL_444749|2020-0	....	....	....	....	....	....
B	217 hCoV-19/USA/NY-NYUMC633/2020|EPI_ISL_444750|2020-0	....	....	....	....	....	....
B	218 hCoV-19/USA/NY-NYUMC634/2020|EPI_ISL_444751|2020-0	....	....	....	....	....	....
B	219 hCoV-19/USA/NY-NYUMC635/2020|EPI_ISL_444752|2020-0	....	....	....	....	....	....
B	220 hCoV-19/USA/NY-NYUMC636/2020|EPI_ISL_444753|2020-0	....	....	....	....	....	....
B	221 hCoV-19/USA/NY-NYUMC637/2020|EPI_ISL_444754|2020-0	....	....	....	....	....	....
B	222 hCoV-19/USA/NJ-NYUMC638/2020|EPI_ISL_444755|2020-0	....	....	....	....	....	....
B	223 hCoV-19/USA/NY-NYUMC639/2020|EPI_ISL_444756|2020-0	....	....	....	....	....	....
B	224 hCoV-19/USA/NY-NYUMC640/2020|EPI_ISL_444757|2020-0	....	....	....	....	....	....
B	225 hCoV-19/USA/NY-NYUMC641/2020|EPI_ISL_444758|2020-0	....	....	....	....	....	....
B	226 hCoV-19/USA/NY-NYUMC642/2020|EPI_ISL_444759|2020-0	....	....	....	....	....	....
B	227 hCoV-19/USA/NY-NYUMC643/2020|EPI_ISL_444760|2020-0	....	....	....	....	....	....

B	228 hCoV-19/USA/NY-NYUMC644/2020|EPI_ISL_444761|2020-0	····················	····················	····················	····················	····················	····················
B	229 hCoV-19/USA/NY-NYUMC645/2020|EPI_ISL_444762|2020-0	····················	····················	····················	····················	····················	····················
B	230 hCoV-19/USA/NY-NYUMC646/2020|EPI_ISL_444763|2020-0	····················	····················	····················	····················	····················	····················
B	231 hCoV-19/USA/NY-NYUMC647/2020|EPI_ISL_444764|2020-0	····················	····················	····················	····················	····················	····················
B	232 hCoV-19/USA/NY-NYUMC648/2020|EPI_ISL_444765|2020-0	····················	····················	····················	····················	····················	····················
B	233 hCoV-19/USA/NY-NYUMC649/2020|EPI_ISL_444766|2020-0	····················	····················	····················	····················	····················	····················
B	234 hCoV-19/USA/NY-NYUMC650/2020|EPI_ISL_444767|2020-0	····················	····················	····················	····················	····················	····················
B	235 hCoV-19/USA/NY-NYUMC651/2020|EPI_ISL_444768|2020-0	····················	····················	····················	····················	····················	····················
B	236 hCoV-19/USA/NY-NYUMC652/2020|EPI_ISL_444769|2020-0	····················	····················	····················	····················	····················	····················
B	237 hCoV-19/USA/NY-NYUMC653/2020|EPI_ISL_444770|2020-0	····················	····················	····················	····················	····················	····················
B	238 hCoV-19/USA/NY-NYUMC654/2020|EPI_ISL_444771|2020-0	····················	····················	····················	····················	····················	····················
B	239 hCoV-19/USA/NY-NYUMC655/2020|EPI_ISL_444772|2020-0	····················	····················	····················	····················	····················	····················
B	240 hCoV-19/USA/NY-NYUMC656/2020|EPI_ISL_444773|2020-0	····················	····················	····················	····················	····················	····················
B	241 hCoV-19/USA/NY-NYUMC657/2020|EPI_ISL_444774|2020-0	····················	····················	····················	····················	····················	····················
B	242 hCoV-19/USA/NY-NYUMC658/2020|EPI_ISL_444775|2020-0	····················	····················	····················	····················	····················	····················
B	243 hCoV-19/USA/NY-NYUMC659/2020|EPI_ISL_444776|2020-0	····················	····················	····················	····················	····················	····················
B	244 hCoV-19/USA/NY-NYUMC660/2020|EPI_ISL_444777|2020-0	····················	····················	····················	····················	····················	····················
B	245 hCoV-19/USA/NJ-NYUMC663/2020|EPI_ISL_444780|2020-0	····················	····················	····················	····················	····················	····················
B	246 hCoV-19/USA/NY-NYUMC664/2020|EPI_ISL_444781|2020-0	····················	····················	····················	····················	····················	····················
B	247 hCoV-19/USA/NY-NYUMC665/2020|EPI_ISL_444782|2020-0	····················	····················	····················	····················	····················	····················
B	248 hCoV-19/USA/NY-NYUMC667/2020|EPI_ISL_444784|2020-0	····················	····················	····················	····················	····················	····················
B	249 hCoV-19/USA/NY-NYUMC669/2020|EPI_ISL_444786|2020-0	····················	····················	····················	····················	····················	····················
B	250 hCoV-19/USA/NY-NYUMC670/2020|EPI_ISL_444787|2020-0	····················	····················	····················	····················	····················	····················
B	251 hCoV-19/USA/NY-NYUMC671/2020|EPI_ISL_444788|2020-0	····················	····················	····················	····················	····················	····················
B	252 hCoV-19/USA/NY-NYUMC672/2020|EPI_ISL_444789|2020-0	····················	····················	····················	····················	····················	····················
B	253 hCoV-19/USA/NY-NYUMC673/2020|EPI_ISL_444790|2020-0	····················	····················	····················	····················	····················	····················
B	254 hCoV-19/USA/NY-NYUMC674/2020|EPI_ISL_444791|2020-0	····················	····················	····················	····················	····················	····················
B	255 hCoV-19/Australia/QLDID939/2020|EPI_ISL_444793|202	····················	····················	····················	····················	····················	····················
B	256 hCoV-19/Australia/QLDID940/2020|EPI_ISL_444794|202	····················	····················	····················	····················	····················	····················
B	257 hCoV-19/USA/WA-S199/2020|EPI_ISL_430237|2020-03-20	····················	····················	····················	····················	····················	····················
B	258 hCoV-19/England/SHEF-BFE8E/2020|EPI_ISL_432712|202	····················	····················	····················	····················	····················	····················
B	259 hCoV-19/England/SHEF-BFEF7/2020|EPI_ISL_432713|202	····················	····················	····················	····················	····················	····················
B	260 hCoV-19/England/SHEF-C0C35/2020|EPI_ISL_432716|202	····················	····················	····················	····················	····················	····················
B	261 hCoV-19/England/SHEF-C0FB4/2020|EPI_ISL_432720|202	····················	····················	····················	····················	····················	····················
B	262 hCoV-19/England/SHEF-C11BD/2020|EPI_ISL_432722|202	····················	····················	····················	····················	····················	····················
B	263 hCoV-19/England/SHEF-C151B/2020|EPI_ISL_432724|202	····················	····················	····················	····················	····················	····················
B	264 hCoV-19/England/SHEF-C1645/2020|EPI_ISL_432727|202	····················	····················	····················	····················	····················	····················
B	266 hCoV-19/Spain/VC-FISABIO-106/2020|EPI_ISL_436201|2	····················	····················	····················	····················	····················	····················
B	268 hCoV-19/Spain/VC-FISABIO-108/2020|EPI_ISL_436203|2	····················	····················	····················	····················	····················	····················
B	269 hCoV-19/Spain/VC-FISABIO-110/2020|EPI_ISL_436205|2	····················	····················	····················	····················	····················	····················
B	273 hCoV-19/Spain/VC-FISABIO-115/2020|EPI_ISL_436210|2	····················	····················	····················	····················	····················	····················
B	274 hCoV-19/Spain/VC-FISABIO-116/2020|EPI_ISL_436211|2	····················	····················	····················	····················	····················	····················
B	275 hCoV-19/Spain/VC-FISABIO-117/2020|EPI_ISL_436212|2	····················	····················	····················	····················	····················	····················
B	276 hCoV-19/Spain/VC-FISABIO-118/2020|EPI_ISL_436213|2	····················	····················	····················	····················	····················	····················
B	277 hCoV-19/Spain/VC-FISABIO-119/2020|EPI_ISL_436214|2	····················	····················	····················	····················	····················	····················
B	279 hCoV-19/Spain/VC-FISABIO-124/2020|EPI_ISL_436219|2	····················	····················	····················	····················	····················	····················
B	280 hCoV-19/Spain/VC-FISABIO-125/2020|EPI_ISL_436220|2	····················	····················	····················	····················	····················	····················
B	281 hCoV-19/Spain/VC-FISABIO-128/2020|EPI_ISL_436223|2	····················	····················	····················	····················	····················	····················
B	282 hCoV-19/Spain/VC-FISABIO-129/2020|EPI_ISL_436224|2	····················	····················	····················	····················	····················	····················
B	283 hCoV-19/Spain/VC-FISABIO-130/2020|EPI_ISL_436225|2	····················	····················	····················	····················	····················	····················
B	284 hCoV-19/Spain/VC-FISABIO-132/2020|EPI_ISL_436227|2	····················	····················	····················	····················	····················	····················
B	286 hCoV-19/Spain/VC-FISABIO-135/2020|EPI_ISL_436230|2	····················	····················	····················	····················	····················	····················
B	287 hCoV-19/Spain/VC-FISABIO-136/2020|EPI_ISL_436231|2	····················	····················	····················	····················	····················	····················
B	291 hCoV-19/Spain/VC-FISABIO-141/2020|EPI_ISL_436236|2	····················	····················	····················	····················	····················	····················
B	293 hCoV-19/Spain/VC-FISABIO-143/2020|EPI_ISL_436238|2	····················	····················	····················	····················	····················	····················
B	295 hCoV-19/Spain/VC-FISABIO-145/2020|EPI_ISL_436240|2	····················	····················	····················	····················	····················	····················
B	296 hCoV-19/Spain/VC-FISABIO-146/2020|EPI_ISL_436241|2	····················	····················	····················	····················	····················	····················
B	297 hCoV-19/Spain/VC-FISABIO-147/2020|EPI_ISL_436242|2	····················	····················	····················	····················	····················	····················
B	299 hCoV-19/Spain/VC-FISABIO-150/2020|EPI_ISL_436245|2	····················	····················	····················	····················	····················	····················
B	300 hCoV-19/Spain/VC-FISABIO-151/2020|EPI_ISL_436246|2	····················	····················	····················	····················	····················	····················
B	301 hCoV-19/Spain/VC-FISABIO-152/2020|EPI_ISL_436247|2	····················	····················	····················	····················	····················	····················
B	302 hCoV-19/Spain/VC-FISABIO-153/2020|EPI_ISL_436248|2	····················	····················	····················	····················	····················	····················
B	303 hCoV-19/Spain/VC-FISABIO-154/2020|EPI_ISL_436249|2	····················	····················	····················	····················	····················	····················
B	304 hCoV-19/Spain/VC-FISABIO-155/2020|EPI_ISL_436250|2	····················	····················	····················	····················	····················	····················
B	305 hCoV-19/Spain/VC-FISABIO-156/2020|EPI_ISL_436251|2	····················	····················	····················	····················	····················	····················
B	306 hCoV-19/Spain/VC-FISABIO-157/2020|EPI_ISL_436252|2	····················	····················	····················	····················	····················	····················
B	307 hCoV-19/Spain/VC-FISABIO-159/2020|EPI_ISL_436254|2	····················	····················	····················	····················	····················	····················
B	308 hCoV-19/Spain/VC-FISABIO-160/2020|EPI_ISL_436255|2	····················	····················	····················	····················	····················	····················
B	309 hCoV-19/Spain/VC-FISABIO-163/2020|EPI_ISL_436258|2	····················	····················	····················	····················	····················	····················
B	311 hCoV-19/Spain/VC-FISABIO-167/2020|EPI_ISL_436262|2	····················	····················	····················	····················	····················	····················
B	314 hCoV-19/Spain/VC-FISABIO-170/2020|EPI_ISL_436265|2	····················	····················	····················	····················	····················	····················
B	317 hCoV-19/Spain/VC-FISABIO-173/2020|EPI_ISL_436268|2	····················	····················	····················	····················	····················	····················
B	318 hCoV-19/Spain/VC-FISABIO-174/2020|EPI_ISL_436269|2	····················	····················	····················	····················	····················	····················
B	319 hCoV-19/Spain/VC-FISABIO-176/2020|EPI_ISL_436271|2	····················	····················	····················	····················	····················	····················
B	320 hCoV-19/Spain/VC-FISABIO-177/2020|EPI_ISL_436272|2	····················	····················	····················	····················	····················	····················
B	321 hCoV-19/Iran/HGRC-2-2162/2020|EPI_ISL_437512|2020-	····················	····················	····················	····················	····················	····················
B	322 hCoV-19/USA/AK-PHL034/2020|EPI_ISL_437513|2020-03-	····················	····················	····················	····················	····················	····················
B	323 hCoV-19/USA/AK-PHL041/2020|EPI_ISL_437514|2020-03-	····················	·········A··········	····················	····················	····················	····················

B	324 hCoV-19/USA/AK-PHL070/2020|EPI_ISL_437515|2020-03-	....	....	....	....	....	....	
B	325 hCoV-19/USA/AK-PHL122/2020|EPI_ISL_437516|2020-03-	....	....	....	....	....	....	
B	326 hCoV-19/USA/AK-PHL178/2020|EPI_ISL_437517|2020-04-	....	....	....	....	....	....	
B	327 hCoV-19/USA/AK-PHL283/2020|EPI_ISL_437518|2020-04-	....	....	....	....	....	....	
B	328 hCoV-19/Czech Republic/NRL_2615/2020|EPI_ISL_43751	....	....	....	....	....	....	
B	329 hCoV-19/USA/OR-OHSU-0012/2020|EPI_ISL_437521|2020-	....	....	....	....	....	....	
B	330 hCoV-19/USA/OR-OHSU-0023/2020|EPI_ISL_437523|2020-	....	....	....	....	....	....	
B	331 hCoV-19/USA/OR-OHSU-0027/2020|EPI_ISL_437524|2020-	....	....	....	....	....	....	
B	332 hCoV-19/USA/OR-OHSU-0043/2020|EPI_ISL_437525|2020-	....	....	....	....	....	....	
B	333 hCoV-19/USA/OR-OHSU-0048/2020|EPI_ISL_437528|2020-	....	....	....	....	....	....	
B	334 hCoV-19/USA/OR-OHSU-0005/2020|EPI_ISL_437529|2020-	....	....	....	....	....	....	
B	335 hCoV-19/USA/OR-OHSU-0051/2020|EPI_ISL_437531|2020-	....	....	....	....	....	....	
B	336 hCoV-19/USA/OR-OHSU-0052/2020|EPI_ISL_437532|2020-	....	....	....	....	....	....	
B	337 hCoV-19/USA/OR-OHSU-0008/2020|EPI_ISL_437535|2020-	....	....	....	....	....	....	
B	338 hCoV-19/India/WB-S12/2020|EPI_ISL_437539|2020-04-0	....	....	....	....	....	....	
B	339 hCoV-19/USA/LA-SR0215/2020|EPI_ISL_437541|2020-04-	....	....	....	....	....	....	
B	340 hCoV-19/USA/LA-SR0214/2020|EPI_ISL_437542|2020-04-	....	....	....	....	....	....	
B	341 hCoV-19/USA/LA-SR0209/2020|EPI_ISL_437546|2020-03-	....	....	....	....	....	....	
B	342 hCoV-19/USA/LA-SR0207/2020|EPI_ISL_437547|2020-03-	....	....	....	....	....	....	
B	343 hCoV-19/USA/CA-SR0263/2020|EPI_ISL_437549|2020-04-	....	....	....	....	....	....	
B	344 hCoV-19/USA/CA-SR0262/2020|EPI_ISL_437550|2020-04-	....	....	....	....	....	....	
B	345 hCoV-19/USA/CA-SR0261/2020|EPI_ISL_437551|2020-04-	....	....	....	....	....	....	
B	346 hCoV-19/USA/CA-SR0260/2020|EPI_ISL_437552|2020-03-	....	....	....	....	....	....	
B	347 hCoV-19/USA/CA-SR0259/2020|EPI_ISL_437553|2020-03-	....	....	....	....	....	....	
B	348 hCoV-19/USA/CA-SR0258/2020|EPI_ISL_437554|2020-03-	....	....	....	....	....	....	
B	349 hCoV-19/USA/CA-SR0257/2020|EPI_ISL_437555|2020-03-	....	....	....	....	....	....	
B	350 hCoV-19/USA/CA-SR0256/2020|EPI_ISL_437556|2020-03-	....	....	....	....	....	....	
B	351 hCoV-19/USA/CA-SR0252/2020|EPI_ISL_437559|2020-03-	....	....	....	....	....	....	
B	352 hCoV-19/USA/CA-SR0250/2020|EPI_ISL_437560|2020-03-	....	....	....	....	....	....	
B	353 hCoV-19/USA/CA-SR0249/2020|EPI_ISL_437561|2020-03-	....	....	....	....	....	....	
B	354 hCoV-19/USA/CA-SR0248/2020|EPI_ISL_437562|2020-03-	....	....	....	....	....	....	
B	355 hCoV-19/USA/CA-SR0247/2020|EPI_ISL_437563|2020-03-	....	....	....	....	....	....	
B	356 hCoV-19/USA/CA-SR0245/2020|EPI_ISL_437564|2020-03-	....	....	....	....	....	....	
B	357 hCoV-19/USA/CA-SR0240/2020|EPI_ISL_437566|2020-03-	....	....	....	....	....	....	
B	358 hCoV-19/USA/CA-SR0237/2020|EPI_ISL_437567|2020-04-	....	....	....	....	....	....	
B	359 hCoV-19/USA/CA-SR0236/2020|EPI_ISL_437568|2020-04-	....	.A..................	....	....	....	....	
B	360 hCoV-19/USA/CA-SR0235/2020|EPI_ISL_437569|2020-04-	....	....	....	....	....	....	
B	361 hCoV-19/USA/CA-SR0234/2020|EPI_ISL_437570|2020-04-	....	....	....	....	....	....	
B	362 hCoV-19/USA/CA-SR0233/2020|EPI_ISL_437571|2020-04-	....	.A..................	....	....	....	....	
B	363 hCoV-19/USA/CA-SR0232/2020|EPI_ISL_437572|2020-04-	....	.A..................	....	....	....	....	
B	364 hCoV-19/USA/CA-SR0228/2020|EPI_ISL_437574|2020-04-	....	....	....	....	....	....	
B	365 hCoV-19/USA/CA-SR0225/2020|EPI_ISL_437575|2020-04-	....	.A..................	....	....	....	....	
B	366 hCoV-19/USA/CA-SR0224/2020|EPI_ISL_437576|2020-04-	....	....	....	....	....	....	
B	367 hCoV-19/USA/CA-SR0218/2020|EPI_ISL_437580|2020-04-	....	.A..................	....	....	....	....	
B	368 hCoV-19/USA/CA-SR0204/2020|EPI_ISL_437582|2020-03-	....	....	....	....	....	....	
B	369 hCoV-19/USA/CA-SR0201/2020|EPI_ISL_437583|2020-03-	....	....	....	....	....	....	
B	370 hCoV-19/USA/CA-SR0199/2020|EPI_ISL_437584|2020-03-	....	....	....	....	....	....	
B	371 hCoV-19/USA/CA-SR0198/2020|EPI_ISL_437585|2020-03-	....	....	....	....	....	....	
B	372 hCoV-19/USA/CA-SR0197/2020|EPI_ISL_437586|2020-03-	....	....	....	....	....	....	
B	373 hCoV-19/USA/CA-SR0195/2020|EPI_ISL_437587|2020-03-	....	....	....	....	....	....	
B	374 hCoV-19/USA/CA-SR0193/2020|EPI_ISL_437588|2020-03-	....	....	....	....	....	....	
B	375 hCoV-19/USA/CA-SR0192/2020|EPI_ISL_437589|2020-03-	....	....	....	....	....	....	
B	376 hCoV-19/USA/CA-SR0190/2020|EPI_ISL_437590|2020-03-	....	....	....	....	....	....	
B	377 hCoV-19/USA/CA-SR0189/2020|EPI_ISL_437591|2020-03-	....	....	....	....	....	....	
B	378 hCoV-19/USA/CA-SR0188/2020|EPI_ISL_437592|2020-03-	....	....	....	....	....	....	
B	379 hCoV-19/USA/CA-SR0184/2020|EPI_ISL_437594|2020-03-	....	....	....	....	....	....	
B	380 hCoV-19/USA/CA-SR0182/2020|EPI_ISL_437595|2020-03-	....	....	....	....	....	....	
B	381 hCoV-19/USA/CA-SR0181/2020|EPI_ISL_437596|2020-03-	....	....	....	....	....	....	
B	382 hCoV-19/USA/CA-SR0179/2020|EPI_ISL_437598|2020-03-	....	....	....	....	....	....	
B	383 hCoV-19/USA/CA-SR0178/2020|EPI_ISL_437599|2020-03-	....	....	....	....	....	....	
B	384 hCoV-19/Scotland/CVR1427/2020|EPI_ISL_438806|2020-	....	...................A	....	....	....	....	1
B	385 hCoV-19/Scotland/CVR1448/2020|EPI_ISL_438815|2020-	....	....	....	....	....	....	
B	386 hCoV-19/Scotland/CVR1484/2020|EPI_ISL_438824|2020-	....	....	....	....	....	....	
B	387 hCoV-19/Scotland/CVR1496/2020|EPI_ISL_438827|2020-	....	.A..................	....	....	....	....	
B	388 hCoV-19/Scotland/CVR1499/2020|EPI_ISL_438829|2020-	....	....	....	....	....	....	
B	389 hCoV-19/Scotland/CVR1504/2020|EPI_ISL_438830|2020-	....	...................A	....	....	....	....	1
B	390 hCoV-19/Scotland/CVR1506/2020|EPI_ISL_438831|2020-	....	...................A	....	....	....	....	1
B	391 hCoV-19/Scotland/CVR1533/2020|EPI_ISL_438836|2020-	....	...................A	....	....	....	....	1
B	392 hCoV-19/Scotland/CVR1537/2020|EPI_ISL_438837|2020-	....	....	....	....	....	....	
B	393 hCoV-19/Scotland/CVR1557/2020|EPI_ISL_438839|2020-	....	....	....	....	....	....	
B	394 hCoV-19/Scotland/CVR1560/2020|EPI_ISL_438840|2020-	....	....	....	....	....	....	
B	395 hCoV-19/Scotland/CVR1573/2020|EPI_ISL_438841|2020-	....	....	....	....	....	....	
B	396 hCoV-19/Scotland/CVR1579/2020|EPI_ISL_438842|2020-	....	....	....	....	....	....	
B	397 hCoV-19/Scotland/CVR1615/2020|EPI_ISL_438844|2020-	....	....	....	....	....	....	
B	399 hCoV-19/Scotland/CVR1816/2020|EPI_ISL_438849|2020-	....	....	....	....	....	....	
B	400 hCoV-19/Scotland/CVR1843/2020|EPI_ISL_438850|2020-	....	....	....	....	....	....	
B	401 hCoV-19/Scotland/CVR1951/2020|EPI_ISL_438860|2020-	....	.A..................	....	....	....	....	
B	402 hCoV-19/Scotland/CVR1997/2020|EPI_ISL_438868|2020-	....	.A..................	....	....	....	....	

```
B   403 hCoV-19/Scotland/CVR2012/2020|EPI_ISL_438870|2020-   ....................  ....................  ....................  ....................  ....................  ....................
B   404 hCoV-19/Scotland/CVR2124/2020|EPI_ISL_438875|2020-   ....................  ....................  ....................  ....................  ....................  ....................
B   405 hCoV-19/Scotland/CVR2153/2020|EPI_ISL_438877|2020-   ....................  ....................  ....................  ....................  ....................  ....................
B   406 hCoV-19/Scotland/CVR2179/2020|EPI_ISL_438878|2020-   ....................  ....................  ....................  ....................  ....................  ....................
B   407 hCoV-19/Scotland/CVR2215/2020|EPI_ISL_438879|2020-   ....................  ....................  ....................  ....................  ....................  ....................
B   408 hCoV-19/Scotland/CVR226/2020|EPI_ISL_438883|2020-0   ....................  ....................  ....................  ....................  ....................  ....................
B   409 hCoV-19/Scotland/CVR2260/2020|EPI_ISL_438884|2020-   ....................  ....................  ....................  ....................  ....................  ....................
B   410 hCoV-19/Scotland/CVR234/2020|EPI_ISL_438889|2020-0   ....................  ....................  ....................  ....................  ....................  ....................
B   411 hCoV-19/Scotland/CVR2347/2020|EPI_ISL_438890|2020-   ....................  ....................  ....................  ....................  ....................  ....................
B   412 hCoV-19/Scotland/CVR2349/2020|EPI_ISL_438891|2020-   ....................  ....................  ....................  ....................  ....................  ....................
B   413 hCoV-19/Scotland/CVR2357/2020|EPI_ISL_438892|2020-   ....................  ....................  ....................  ....................  ....................  ....................
B   414 hCoV-19/Scotland/CVR2367/2020|EPI_ISL_438893|2020-   ....................  ...................A  ....................  ....................  ....................  ....................   1
B   415 hCoV-19/Scotland/CVR2372/2020|EPI_ISL_438894|2020-   ....................  ....................  ....................  ....................  ....................  ....................
B   416 hCoV-19/Scotland/CVR2378/2020|EPI_ISL_438895|2020-   ....................  ....................  ....................  ....................  ....................  ....................
B   417 hCoV-19/Scotland/CVR238/2020|EPI_ISL_438896|2020-0   ....................  ....................  ....................  ....................  ....................  ....................
B   418 hCoV-19/England/SHEF-C9935/2020|EPI_ISL_442043|202   ....................  ....................  ....................  ....................  ....................  ....................
B   419 hCoV-19/Iran/KHGRC-2-2162/2020|EPI_ISL_442044|2020   ....................  ....................  ....................  ....................  ....................  ....................
B   420 hCoV-19/England/CAMB-7D077/2020|EPI_ISL_442045|202   ....................  ....................  ....................  ....................  ....................  ....................
B   421 hCoV-19/England/CAMB-7BC66/2020|EPI_ISL_442046|202   ....................  ....................  ....................  ....................  ....................  ....................
B   422 hCoV-19/England/CAMB-7CBD1/2020|EPI_ISL_442047|202   ....................  ....................  ....................  ....................  ....................  ....................
B   423 hCoV-19/England/CAMB-7C7A0/2020|EPI_ISL_442048|202   ....................  ....................  ....................  ....................  ....................  ....................
B   424 hCoV-19/England/CAMB-7CA89/2020|EPI_ISL_442049|202   ....................  ....................  ....................  ....................  ....................  ....................
B   425 hCoV-19/England/CAMB-7C931/2020|EPI_ISL_442050|202   ....................  ....................  ....................  ....................  ....................  ....................
B   426 hCoV-19/England/CAMB-7D1DE/2020|EPI_ISL_442051|202   ....................  ....................  ....................  ....................  ....................  ....................
B   427 hCoV-19/England/CAMB-7BE24/2020|EPI_ISL_442053|202   ....................  ....................  ....................  ....................  ....................  ....................
B   428 hCoV-19/England/CAMB-7C8AD/2020|EPI_ISL_442054|202   ....................  ....................  ....................  ....................  ....................  ....................
B   429 hCoV-19/England/CAMB-7C7CE/2020|EPI_ISL_442055|202   ....................  ....................  ....................  ....................  ....................  ....................
B   430 hCoV-19/England/CAMB-7E0FE/2020|EPI_ISL_442056|202   ....................  ....................  ....................  ....................  ....................  ....................
B   431 hCoV-19/England/CAMB-7CC56/2020|EPI_ISL_442057|202   ....................  ....................  ....................  ....................  ....................  ....................
B   432 hCoV-19/England/CAMB-7D1B0/2020|EPI_ISL_442058|202   ....................  ....................  ....................  ....................  ....................  ....................
B   433 hCoV-19/England/CAMB-7D059/2020|EPI_ISL_442059|202   ....................  ....................  ....................  ....................  ....................  ....................
B   434 hCoV-19/England/CAMB-7B9C9/2020|EPI_ISL_442060|202   ....................  ....................  ....................  ....................  ....................  ....................
B   435 hCoV-19/England/CAMB-7C870/2020|EPI_ISL_442061|202   ....................  ....................  ....................  ....................  ....................  ....................
B   436 hCoV-19/England/CAMB-7BD81/2020|EPI_ISL_442062|202   ....................  ....................  ....................  ....................  ....................  ....................
B   437 hCoV-19/England/CAMB-7CAA7/2020|EPI_ISL_442064|202   ....................  ....................  ....................  ....................  ....................  ....................
B   438 hCoV-19/England/CAMB-7B871/2020|EPI_ISL_442066|202   ....................  ....................  ....................  ....................  ....................  ....................
B   439 hCoV-19/England/CAMB-7D271/2020|EPI_ISL_442067|202   ....................  ....................  ....................  ....................  ....................  ....................
B   440 hCoV-19/England/CAMB-7C99B/2020|EPI_ISL_442068|202   ....................  ....................  ....................  ....................  ....................  ....................
B   441 hCoV-19/England/CAMB-7E01C/2020|EPI_ISL_442070|202   ....................  ....................  ....................  ....................  ....................  ....................
B   442 hCoV-19/England/CAMB-7BC39/2020|EPI_ISL_442071|202   ....................  ....................  ....................  ....................  ....................  ....................
B   443 hCoV-19/England/CAMB-7D29F/2020|EPI_ISL_442073|202   ....................  ....................  ....................  ....................  ....................  ....................
B   444 hCoV-19/England/CAMB-7C9E6/2020|EPI_ISL_442074|202   ....................  ....................  ....................  ....................  ....................  ....................
B   445 hCoV-19/England/CAMB-7BBB4/2020|EPI_ISL_442075|202   ....................  ....................  ....................  ....................  ....................  ....................
B   446 hCoV-19/England/CAMB-7C8CB/2020|EPI_ISL_442076|202   ....................  ....................  ....................  ....................  ....................  ....................
B   447 hCoV-19/England/CAMB-7C728/2020|EPI_ISL_442077|202   ....................  ....................  ....................  ....................  ....................  ....................
B   448 hCoV-19/England/CAMB-7CDF9/2020|EPI_ISL_442078|202   ....................  ....................  ....................  ....................  ....................  ....................
B   449 hCoV-19/England/CAMB-7CB1D/2020|EPI_ISL_442079|202   ....................  ....................  ....................  ....................  ....................  ....................
B   450 hCoV-19/England/CAMB-7BF7C/2020|EPI_ISL_442080|202   ....................  ....................  ....................  ....................  ....................  ....................
B   451 hCoV-19/England/CAMB-7DE40/2020|EPI_ISL_442081|202   ....................  ....................  ....................  ....................  ....................  ....................
B   452 hCoV-19/England/CAMB-7CB3B/2020|EPI_ISL_442082|202   ....................  ....................  ....................  ....................  ....................  ....................
B   453 hCoV-19/England/CAMB-7DE6E/2020|EPI_ISL_442083|202   ....................  ....................  ....................  ....................  ....................  ....................
B   454 hCoV-19/England/CAMB-7DD43/2020|EPI_ISL_442084|202   ....................  ....................  ....................  ....................  ....................  ....................
B   455 hCoV-19/England/CAMB-7CA10/2020|EPI_ISL_442085|202   ....................  ....................  ....................  ....................  ....................  ....................
B   456 hCoV-19/England/CAMB-7BE06/2020|EPI_ISL_442086|202   ....................  ....................  ....................  ....................  ....................  ....................
B   457 hCoV-19/England/CAMB-7CAF2/2020|EPI_ISL_442088|202   ....................  ....................  ....................  ....................  ....................  ....................
B   458 hCoV-19/England/CAMB-7BC0C/2020|EPI_ISL_442089|202   ....................  ....................  ....................  ....................  ....................  ....................
B   459 hCoV-19/England/CAMB-7D226/2020|EPI_ISL_442090|202   ....................  ....................  ....................  ....................  ....................  ....................
B   460 hCoV-19/England/CAMB-7CAC5/2020|EPI_ISL_442091|202   ....................  ....................  ....................  ....................  ....................  ....................
B   461 hCoV-19/England/CAMB-7DE04/2020|EPI_ISL_442092|202   ....................  ....................  ....................  ....................  ....................  ....................
B   462 hCoV-19/England/CAMB-7E049/2020|EPI_ISL_442093|202   ....................  ....................  ....................  ....................  ....................  ....................
B   463 hCoV-19/England/CAMB-7BC93/2020|EPI_ISL_442094|202   ....................  ....................  ....................  ....................  ....................  ....................
B   464 hCoV-19/England/CAMB-7D01D/2020|EPI_ISL_442095|202   ....................  ....................  ....................  ....................  ....................  ...............A....
B   465 hCoV-19/England/CAMB-7BCEE/2020|EPI_ISL_442097|202   ....................  ....................  ....................  ....................  ....................  ....................
B   466 hCoV-19/England/CAMB-7C7EC/2020|EPI_ISL_442098|202   ....................  ....................  ....................  ....................  ....................  ....................
B   467 hCoV-19/England/CAMB-7CA7A/2020|EPI_ISL_442220|202   ....................  ....................  ....................  ....................  ....................  ....................
B   468 hCoV-19/England/CAMB-7DBEF/2020|EPI_ISL_442221|202   ....................  ....................  ....................  ....................  ....................  ....................
B   469 hCoV-19/England/CAMB-7C49A/2020|EPI_ISL_442222|202   ....................  ....................  ....................  ....................  ....................  ....................
B   470 hCoV-19/England/CAMB-7DAA6/2020|EPI_ISL_442223|202   ....................  ....................  ....................  ....................  ....................  ....................
B   471 hCoV-19/England/CAMB-83CC5/2020|EPI_ISL_443371|202   ....................  ....................  ....................  ....................  ....................  ....................
B   472 hCoV-19/England/CAMB-83CE3/2020|EPI_ISL_443372|202   ....................  ....................  ....................  ....................  ....................  ....................
B   473 hCoV-19/England/CAMB-83C10/2020|EPI_ISL_443375|202   ....................  ....................  ....................  ....................  ....................  ....................
B   474 hCoV-19/England/CAMB-83991/2020|EPI_ISL_443378|202   ....................  ....................  ....................  ....................  ....................  ....................
B   475 hCoV-19/England/CAMB-83937/2020|EPI_ISL_443383|202   ....................  ....................  ....................  ....................  ....................  ....................
B   476 hCoV-19/England/CAMB-8366D/2020|EPI_ISL_443384|202   ....................  ....................  ....................  ....................  ....................  ....................
B   477 hCoV-19/England/CAMB-8258F/2020|EPI_ISL_443385|202   ....................  ....................  ....................  ....................  ....................  ....................
B   478 hCoV-19/England/CAMB-830E7/2020|EPI_ISL_443386|202   ....................  ....................  ....................  ....................  ....................  ....................
B   479 hCoV-19/England/CAMB-82DB4/2020|EPI_ISL_443387|202   ....................  ....................  ....................  ....................  ....................  ....................
B   480 hCoV-19/England/CAMB-82D96/2020|EPI_ISL_443388|202   ....................  ....................  ....................  ....................  ....................  ....................
```

B	481	hCoV-19/England/CAMB-82E48/2020|EPI_ISL_443389|202	....	....	....	....	....	....
B	482	hCoV-19/England/CAMB-832E1/2020|EPI_ISL_443390|202	....	....	....	....	....	....
B	483	hCoV-19/England/CAMB-83867/2020|EPI_ISL_443391|202	....	....	....	....	....	....
B	484	hCoV-19/England/CAMB-82622/2020|EPI_ISL_443392|202	....	....	....	....	....	....
B	485	hCoV-19/England/CAMB-83603/2020|EPI_ISL_443393|202	....	....	....	....	....	....
B	486	hCoV-19/England/CAMB-835CA/2020|EPI_ISL_443394|202	....	....	....	....	....	....
B	487	hCoV-19/England/CAMB-834CD/2020|EPI_ISL_443395|202	....	....	....	....	....	....
B	488	hCoV-19/England/CAMB-832C3/2020|EPI_ISL_443396|202	....	....	....	....	....	....
B	489	hCoV-19/England/CAMB-8320F/2020|EPI_ISL_443398|202	....	....	....	....	....	....
B	490	hCoV-19/England/CAMB-82604/2020|EPI_ISL_443399|202	....	....	....	....	....	....
B	491	hCoV-19/England/CAMB-82CF3/2020|EPI_ISL_443552|202	....	....	....	....	....	....
B	492	hCoV-19/England/CAMB-1AA2A7/2020|EPI_ISL_443554|20	....	....	....	....	....	....
B	493	hCoV-19/USA/NY-NYUMC582/2020|EPI_ISL_444699|2020-0	....	....	....	....	....	....
B	494	hCoV-19/USA/NY-NYUMC583/2020|EPI_ISL_444700|2020-0	....	....	....	....	....	....
B	495	hCoV-19/USA/NY-NYUMC584/2020|EPI_ISL_444701|2020-0	....	....	....	....	....	....
B	496	hCoV-19/USA/NY-NYUMC585/2020|EPI_ISL_444702|2020-0	....	....	....	....	....	....
B	497	hCoV-19/USA/NY-NYUMC586/2020|EPI_ISL_444703|2020-0	....	....	....	....	....	....
B	498	hCoV-19/USA/NY-NYUMC587/2020|EPI_ISL_444704|2020-0	....	....	....	....	....	....
B	499	hCoV-19/USA/NY-NYUMC588/2020|EPI_ISL_444705|2020-0	....	....	....	....	....	....
B	500	hCoV-19/USA/NY-NYUMC589/2020|EPI_ISL_444706|2020-0	....	....	....	....	....	....
B	501	hCoV-19/USA/NY-NYUMC590/2020|EPI_ISL_444707|2020-0	....	....	....	....	....	....
B	502	hCoV-19/USA/NY-NYUMC591/2020|EPI_ISL_444708|2020-0	....	....	....	....	....	....
B	503	hCoV-19/USA/NY-NYUMC604/2020|EPI_ISL_444721|2020-0	....	....	....	....	....	....
B	504	hCoV-19/USA/NY-NYUMC608/2020|EPI_ISL_444725|2020-0	....	....	....	....	....	....
B	505	hCoV-19/USA/NY-NYUMC609/2020|EPI_ISL_444726|2020-0	....	....	....	....	....	....
B	506	hCoV-19/USA/NY-NYUMC610/2020|EPI_ISL_444727|2020-0	....	....	....	....	....	....
B	507	hCoV-19/USA/NY-NYUMC611/2020|EPI_ISL_444728|2020-0	....	....	....	....	....	....
B	508	hCoV-19/Denmark/ALAB-SSI-731/2020|EPI_ISL_444880|2	....	....	....	....	....	....
B	509	hCoV-19/Denmark/ALAB-SSI-735/2020|EPI_ISL_444881|2	....	....	....	....	....	....
B	510	hCoV-19/Denmark/ALAB-SSI-741/2020|EPI_ISL_444883|2	....	....	....	....	....	....
B	511	hCoV-19/Denmark/ALAB-SSI-742/2020|EPI_ISL_444884|2	....	....	....	....	....	....
B	512	hCoV-19/Denmark/ALAB-SSI-743/2020|EPI_ISL_444885|2	....	....	....	....	....	....
B	513	hCoV-19/USA/WA-S174/2020|EPI_ISL_430212|2020-03-19	....	....	....	....	....	....
B	514	hCoV-19/USA/WA-S175/2020|EPI_ISL_430213|2020-03-19	....	....	....	....	....	....
B	515	hCoV-19/USA/WA-S176/2020|EPI_ISL_430214|2020-03-19	....	....	....	....	....	....
B	516	hCoV-19/USA/WA-S177/2020|EPI_ISL_430215|2020-03-19	....	....	....	....	....	....
B	517	hCoV-19/USA/WA-S178/2020|EPI_ISL_430216|2020-03-20	....	....	....	....	....	....
B	518	hCoV-19/USA/WA-S179/2020|EPI_ISL_430217|2020-03-20	....	....	....	....	....	....
B	519	hCoV-19/USA/WA-S181/2020|EPI_ISL_430219|2020-03-19	....	....	....	....	....	....
B	520	hCoV-19/USA/WA-S182/2020|EPI_ISL_430220|2020-03-20	....	....	....	....	....	....
B	521	hCoV-19/USA/WA-S183/2020|EPI_ISL_430221|2020-03-19	....	....	....	....	....	....
B	522	hCoV-19/USA/WA-S184/2020|EPI_ISL_430222|2020-03-19	....	....	....	....	....	....
B	523	hCoV-19/USA/WA-S185/2020|EPI_ISL_430223|2020-03-19	....	....	....	....	....	....
B	524	hCoV-19/USA/WA-S186/2020|EPI_ISL_430224|2020-03-20	....	....	....	....	....	....
B	525	hCoV-19/USA/WA-S187/2020|EPI_ISL_430225|2020-03-20	....	....	....	....	....	....
B	526	hCoV-19/USA/WA-S188/2020|EPI_ISL_430226|2020-03-20	....	....	....	....	....	....
B	527	hCoV-19/USA/WA-S189/2020|EPI_ISL_430227|2020-03-20	....	....	....	....	....	....
B	528	hCoV-19/USA/WA-S190/2020|EPI_ISL_430228|2020-03-23	....	....	....	....	....	....
B	529	hCoV-19/USA/WA-S191/2020|EPI_ISL_430229|2020-03-20	....	....	....	....	....	....
B	530	hCoV-19/USA/WA-S192/2020|EPI_ISL_430230|2020-03-20	....	....	....	....	....	....
B	531	hCoV-19/USA/WA-S193/2020|EPI_ISL_430231|2020-03-19	....	....	....	....	....	....
B	532	hCoV-19/USA/WA-S194/2020|EPI_ISL_430232|2020-03-20	....	....	....	....	....	....
B	533	hCoV-19/USA/WA-S195/2020|EPI_ISL_430233|2020-03-20	....	....	....	....	....	....
B	534	hCoV-19/USA/WA-S196/2020|EPI_ISL_430234|2020-03-19	....	....	....	....	....	....
B	535	hCoV-19/USA/WA-S197/2020|EPI_ISL_430235|2020-03-20	....	....	....	....	....	....
B	536	hCoV-19/USA/WA-S198/2020|EPI_ISL_430236|2020-03-20	....	....	....	....	....	....
B	537	hCoV-19/USA/WA-S200/2020|EPI_ISL_430238|2020-03-20	....	....	....	....	....	....
B	538	hCoV-19/USA/WA-S201/2020|EPI_ISL_430239|2020-03-20	....	....	....	....	....	....
B	539	hCoV-19/USA/WA-S202/2020|EPI_ISL_430240|2020-03-20	....	....	....	....	....	....
B	540	hCoV-19/USA/WA-S203/2020|EPI_ISL_430241|2020-03-20	....	....	....	....	....	....
B	541	hCoV-19/USA/WA-S204/2020|EPI_ISL_430242|2020-03-22	....	....	....	....	....	....
B	542	hCoV-19/USA/WA-S205/2020|EPI_ISL_430243|2020-03-20	....	....	....	....	....	....
B	543	hCoV-19/USA/WA-S206/2020|EPI_ISL_430244|2020-03-20	....	....	....	....	....	....
B	544	hCoV-19/USA/WA-S207/2020|EPI_ISL_430245|2020-03-20	....	....	....	....	....	....
B	545	hCoV-19/USA/WA-S208/2020|EPI_ISL_430246|2020-03-23	....	....	....	....	....	....
B	546	hCoV-19/USA/WA-S209/2020|EPI_ISL_430247|2020-03-23	....	....	....	....	....	....
B	547	hCoV-19/USA/WA-S210/2020|EPI_ISL_430248|2020-03-19	....	....	....	....	....	....
B	548	hCoV-19/USA/WA-S211/2020|EPI_ISL_430249|2020-03-20	....	....	....	....	....	....
B	549	hCoV-19/USA/WA-S212/2020|EPI_ISL_430250|2020-03-19	....	....	....	....	....	....
B	550	hCoV-19/USA/WA-S214/2020|EPI_ISL_430252|2020-03-20	....	....	....	....	....	....
B	551	hCoV-19/USA/WA-S215/2020|EPI_ISL_430253|2020-03-20	....	....	....	....	....	....
B	552	hCoV-19/USA/WA-S216/2020|EPI_ISL_430254|2020-03-20	....	....	....	....	....	....
B	553	hCoV-19/USA/WA-S217/2020|EPI_ISL_430255|2020-03-20	....	....	....	....	....	....
B	554	hCoV-19/USA/WA-S218/2020|EPI_ISL_430256|2020-03-23	....	....	....	....	....	....
B	555	hCoV-19/USA/WA-S219/2020|EPI_ISL_430257|2020-03-20	....	....	....	....	....	....
B	556	hCoV-19/USA/WA-S220/2020|EPI_ISL_430258|2020-03-20	....	....	....	....	....	....
B	557	hCoV-19/USA/WA-S221/2020|EPI_ISL_430259|2020-03-19	....	....	....	....	....	....
B	558	hCoV-19/USA/WA-S223/2020|EPI_ISL_430261|2020-03-20	....	....	....	....	....	....

B 559 hCoV-19/USA/WA-S224/2020|EPI_ISL_430262|2020-03-19 ···················· ···················· ···················· ···················· ···················· ····················
B 560 hCoV-19/USA/WA-S225/2020|EPI_ISL_430263|2020-03-20 ···················· ···················· ···················· ···················· ···················· ····················
B 561 hCoV-19/USA/WA-S226/2020|EPI_ISL_430264|2020-03-21 ···················· ···················· ···················· ···················· ···················· ····················
B 562 hCoV-19/USA/WA-S227/2020|EPI_ISL_430265|2020-03-20 ···················· ···················· ···················· ···················· ···················· ····················
B 563 hCoV-19/USA/WA-S228/2020|EPI_ISL_430266|2020-03-20 ···················· ···················· ···················· ···················· ···················· ····················
B 564 hCoV-19/USA/WA-S229/2020|EPI_ISL_430267|2020-03-19 ···················· ···················· ···················· ···················· ···················· ····················
B 565 hCoV-19/USA/WA-S230/2020|EPI_ISL_430268|2020-03-19 ···················· ···················· ···················· ···················· ···················· ····················
B 566 hCoV-19/USA/WA-S231/2020|EPI_ISL_430269|2020-03-20 ···················· ···················· ···················· ···················· ···················· ····················
B 567 hCoV-19/USA/WA-S232/2020|EPI_ISL_430270|2020-03-08 ···················· ···················· ···················· ···················· ···················· ····················
B 568 hCoV-19/USA/WA-S233/2020|EPI_ISL_430271|2020-03-03 ···················· ···················· ···················· ···················· ···················· ····················
B 569 hCoV-19/USA/WA-S234/2020|EPI_ISL_430272|2020-02-28 ···················· ···················· ···················· ···················· ···················· ····················
B 570 hCoV-19/USA/WA-S235/2020|EPI_ISL_430273|2020-02-29 ···················· ···················· ···················· ···················· ···················· ····················
B 571 hCoV-19/USA/WA-S236/2020|EPI_ISL_430274|2020-03-01 ···················· ···················· ···················· ···················· ···················· ····················
B 572 hCoV-19/USA/WA-S237/2020|EPI_ISL_430275|2020-03-06 ···················· ···················· ···················· ···················· ···················· ····················
B 573 hCoV-19/USA/WA-S238/2020|EPI_ISL_430276|2020-03-02 ···················· ···················· ···················· ···················· ···················· ····················
B 574 hCoV-19/USA/WA-S239/2020|EPI_ISL_430277|2020-03-07 ···················· ···················· ···················· ···················· ···················· ····················
B 575 hCoV-19/USA/WA-S240/2020|EPI_ISL_430278|2020-03-06 ···················· ···················· ···················· ···················· ···················· ····················
B 576 hCoV-19/USA/WA-S241/2020|EPI_ISL_430279|2020-03-04 ···················· ···················· ···················· ···················· ···················· ····················
B 578 hCoV-19/USA/WA-S243/2020|EPI_ISL_430281|2020-02-29 ···················· ···················· ···················· ···················· ···················· ····················
B 579 hCoV-19/USA/WA-S244/2020|EPI_ISL_430282|2020-03-11 ···················· ···················· ···················· ···················· ···················· ····················
B 580 hCoV-19/USA/WA-S245/2020|EPI_ISL_430283|2020-03-02 ···················· ···················· ···················· ···················· ···················· ····················
B 581 hCoV-19/USA/WA-S246/2020|EPI_ISL_430284|2020-03-10 ···················· ···················· ···················· ···················· ···················· ····················
B 582 hCoV-19/USA/WA-S247/2020|EPI_ISL_430285|2020-03-01 ···················· ···················· ···················· ···················· ···················· ····················
B 583 hCoV-19/USA/WA-S248/2020|EPI_ISL_430286|2020-03-10 ···················· ···················· ···················· ···················· ···················· ····················
B 584 hCoV-19/USA/WA-S249/2020|EPI_ISL_430287|2020-03-01 ···················· ···················· ···················· ···················· ···················· ····················
B 585 hCoV-19/USA/WA-S250/2020|EPI_ISL_430288|2020-03-03 ···················· ···················· ···················· ···················· ···················· ····················
B 586 hCoV-19/USA/WA-S251/2020|EPI_ISL_430289|2020-03-11 ···················· ···················· ···················· ···················· ···················· ····················
B 587 hCoV-19/USA/WA-S252/2020|EPI_ISL_430290|2020-03-02 ···················· ···················· ···················· ···················· ···················· ····················
B 588 hCoV-19/USA/WA-S253/2020|EPI_ISL_430291|2020-03-07 ···················· ···················· ···················· ···················· ···················· ····················
B 589 hCoV-19/USA/WA-S254/2020|EPI_ISL_430292|2020-02-24 ···················· ···················· ···················· ···················· ···················· ····················
B 590 hCoV-19/USA/WA-S255/2020|EPI_ISL_430293|2020-03-08 ···················· ···················· ···················· ···················· ···················· ····················
B 591 hCoV-19/USA/WA-S256/2020|EPI_ISL_430294|2020-03-02 ···················· ···················· ···················· ···················· ···················· ····················
B 592 hCoV-19/USA/WA-S257/2020|EPI_ISL_430295|2020-02-25 ···················· ···················· ···················· ···················· ···················· ····················
B 593 hCoV-19/South Africa/R02827/2020|EPI_ISL_430297|20 ···················· ···················· ···················· ···················· ···················· ····················
B 594 hCoV-19/England/SHEF-C5D79/2020|EPI_ISL_432812|202 ···················· ···················· ···················· ···················· ···················· ····················
B 595 hCoV-19/England/SHEF-C5DD3/2020|EPI_ISL_432813|202 ···················· ·A·················· ···················· ···················· ···················· ····················
B 596 hCoV-19/Scotland/EDB909/2020|EPI_ISL_432874|2020-0 ···················· ···················· ···················· ···················· ··············N····· ····················
B 597 hCoV-19/Scotland/EDB910/2020|EPI_ISL_432875|2020-0 ···················· ···················· ···················· ···················· ···················· ····················
B 599 hCoV-19/Scotland/EDB914/2020|EPI_ISL_432877|2020-0 ···················· ···················· ···················· ···················· ···················· ····················
B 601 hCoV-19/Scotland/EDB921/2020|EPI_ISL_432880|2020-0 ···················· ···················· ···················· ···················· ···················· ····················
B 602 hCoV-19/Scotland/EDB922/2020|EPI_ISL_432881|2020-0 ···················· ···················· ···················· ···················· ···················· ····················
B 603 hCoV-19/Scotland/EDB924/2020|EPI_ISL_432883|2020-0 ···················· ···················· ···················· ···················· ···················· ····················
B 604 hCoV-19/Scotland/EDB926/2020|EPI_ISL_432885|2020-0 ···················· ·A·················· ···················· ···················· ···················· ····················
B 605 hCoV-19/Scotland/EDB927/2020|EPI_ISL_432886|2020-0 ···················· ···················· ···················· ···················· ···················· ····················
B 608 hCoV-19/Scotland/EDB945/2020|EPI_ISL_432890|2020-0 ···················· ···················· ···················· ···················· ···················· ····················
B 609 hCoV-19/Scotland/EDB964/2020|EPI_ISL_432891|2020-0 ···················· ·A·················· ···················· ···················· ··N················· ····················
B 610 hCoV-19/Scotland/EDB971/2020|EPI_ISL_432892|2020-0 ···················· ·A·················· ···················· ···················· ···················· ····················
B 611 hCoV-19/Scotland/EDB972/2020|EPI_ISL_432893|2020-0 ···················· ···················· ···················· ···················· ···················· ····················
B 612 hCoV-19/Scotland/EDB973/2020|EPI_ISL_432894|2020-0 ···················· ···················· ···················· ···················· ···················· ····················
B 613 hCoV-19/Scotland/EDB986/2020|EPI_ISL_432895|2020-0 ···················· ···················· ···················· ···················· ···················· ····················
B 615 hCoV-19/Scotland/EDB997/2020|EPI_ISL_432898|2020-0 ···················· ·A·················· ···················· ···················· ···················· ····················
B 616 hCoV-19/Scotland/EDB999/2020|EPI_ISL_432899|2020-0 ···················· ···················· ···················· ···················· ···················· ····················
B 617 hCoV-19/India/JK-NCDC-02326/2020|EPI_ISL_435090|20 ···················· ···················· ···················· ···················· ···················· ····················
B 618 hCoV-19/England/CAMB-7DFF2/2020|EPI_ISL_442200|202 ···················· ···················· ···················· ···················· ···················· ····················
B 619 hCoV-19/England/CAMB-7E0B2/2020|EPI_ISL_442201|202 ···················· ···················· ···················· ···················· ···················· ····················
B 620 hCoV-19/England/CAMB-7DE8C/2020|EPI_ISL_442202|202 ···················· ···················· ···················· ···················· ···················· ····················
B 621 hCoV-19/England/CAMB-7DFB6/2020|EPI_ISL_442203|202 ···················· ···················· ···················· ···················· ···················· ····················
B 622 hCoV-19/England/CAMB-7D62A/2020|EPI_ISL_442204|202 ···················· ···················· ···················· ···················· ···················· ····················
B 623 hCoV-19/England/CAMB-7D068/2020|EPI_ISL_442206|202 ···················· ···················· ···················· ···················· ···················· ····················
B 624 hCoV-19/England/CAMB-7BC1B/2020|EPI_ISL_442207|202 ···················· ···················· ···················· ···················· ···················· ····················
B 625 hCoV-19/England/CAMB-7CED8/2020|EPI_ISL_442208|202 ···················· ···················· ···················· ···················· ···················· ····················
B 626 hCoV-19/England/CAMB-7B6D1/2020|EPI_ISL_442210|202 ···················· ···················· ···················· ···················· ···················· ····················
B 627 hCoV-19/England/CAMB-7C755/2020|EPI_ISL_442211|202 ···················· ···················· ···················· ···················· ···················· ····················
B 628 hCoV-19/England/CAMB-7CB4A/2020|EPI_ISL_442213|202 ···················· ···················· ···················· ···················· ···················· ····················
B 629 hCoV-19/England/CAMB-7C737/2020|EPI_ISL_442214|202 ···················· ···················· ···················· ···················· ···················· ····················
B 630 hCoV-19/England/CAMB-7CD08/2020|EPI_ISL_442215|202 ···················· ···················· ···················· ···················· ···················· ····················
B 631 hCoV-19/England/CAMB-7D7BE/2020|EPI_ISL_442216|202 ···················· ···················· ···················· ···················· ···················· ····················
B 632 hCoV-19/England/CAMB-7D1CF/2020|EPI_ISL_442217|202 ···················· ···················· ···················· ···················· ···················· ····················
B 633 hCoV-19/England/CAMB-7CF7B/2020|EPI_ISL_442224|202 ···················· ···················· ···················· ···················· ···················· ····················
B 634 hCoV-19/England/CAMB-7DEAA/2020|EPI_ISL_442225|202 ···················· ···················· ···················· ···················· ···················· ····················
B 635 hCoV-19/England/CAMB-7DC0A/2020|EPI_ISL_442226|202 ···················· ···················· ···················· ···················· ···················· ····················
B 636 hCoV-19/England/CAMB-7CFB7/2020|EPI_ISL_442229|202 ···················· ···················· ···················· ···················· ···················· ····················
B 637 hCoV-19/England/CAMB-7CEC9/2020|EPI_ISL_442230|202 ···················· ···················· ···················· ···················· ···················· ····················
B 638 hCoV-19/England/CAMB-7D0C2/2020|EPI_ISL_442231|202 ···················· ···················· ···················· ···················· ···················· ····················
B 639 hCoV-19/England/CAMB-7CFC6/2020|EPI_ISL_442232|202 ···················· ···················· ···················· ···················· ···················· ····················
B 640 hCoV-19/England/CAMB-7D11A/2020|EPI_ISL_442234|202 ···················· ···················· ···················· ···················· ···················· ····················
B 641 hCoV-19/England/CAMB-7BAF3/2020|EPI_ISL_442235|202 ···················· A··················· ···················· ···················· ···················· ····················
B 642 hCoV-19/England/CAMB-7DF01/2020|EPI_ISL_442236|202 ···················· ···················· ···················· ···················· ···················· ····················

B	643	hCoV-19/England/CAMB-7C843/2020|EPI_ISL_442237|202	....	....	....	....	....	....
B	644	hCoV-19/England/CAMB-7D3E7/2020|EPI_ISL_442240|202	....	....	....	....	....	....
B	645	hCoV-19/England/CAMB-7D9B8/2020|EPI_ISL_442241|202	....	....	....	....	....	....
B	646	hCoV-19/England/CAMB-7D578/2020|EPI_ISL_442242|202	....	....	....	....	....	....
B	647	hCoV-19/England/CAMB-7C54C/2020|EPI_ISL_442244|202	....	....	....	....	....	....
B	648	hCoV-19/England/CAMB-7C658/2020|EPI_ISL_442246|202	....	....	....	....	....	....
B	649	hCoV-19/England/CAMB-7C825/2020|EPI_ISL_442247|202	....	....	....	....	....	....
B	650	hCoV-19/England/CAMB-7C4F4/2020|EPI_ISL_442248|202	....	....	....	....	....	....
B	651	hCoV-19/England/CAMB-7D7DC/2020|EPI_ISL_442250|202	....	....	....	....	....	....
B	652	hCoV-19/England/CAMB-7CDCC/2020|EPI_ISL_442251|202	....	....	....	....	....	....
B	653	hCoV-19/England/CAMB-7D38D/2020|EPI_ISL_442252|202	....	....	....	....	....	....
B	654	hCoV-19/England/CAMB-7D39C/2020|EPI_ISL_442253|202	....	....	....	....	....	....
B	655	hCoV-19/England/CAMB-7CE50/2020|EPI_ISL_442254|202	....	....	....	....	....	....
B	656	hCoV-19/England/CAMB-7CD80/2020|EPI_ISL_442255|202	....	....	....	....	....	....
B	657	hCoV-19/England/CAMB-7CF4E/2020|EPI_ISL_442256|202	....	....	....	....	....	....
B	658	hCoV-19/England/CAMB-7CDBD/2020|EPI_ISL_442257|202	....	....	....	....	....	....
B	659	hCoV-19/England/CAMB-7DCA0/2020|EPI_ISL_442258|202	....	....	....	....	....	....
B	660	hCoV-19/England/CAMB-7B6FF/2020|EPI_ISL_442259|202	....	....	....	....	....	....
B	661	hCoV-19/England/CAMB-7D350/2020|EPI_ISL_442260|202	....	....	....	....	....	....
B	662	hCoV-19/England/CAMB-7D9D6/2020|EPI_ISL_442261|202	....	....	....	....	....	....
B	663	hCoV-19/England/CAMB-7B729/2020|EPI_ISL_442262|202	....	....	....	....	....	....
B	664	hCoV-19/England/CAMB-7D2F9/2020|EPI_ISL_442263|202	....	....	....	....	....	....
B	665	hCoV-19/England/CAMB-7C60D/2020|EPI_ISL_442265|202	....	....	....	....	....	....
B	666	hCoV-19/England/CAMB-7D4F3/2020|EPI_ISL_442266|202	....	....A...............	....	....	....	....
B	667	hCoV-19/England/CAMB-7BB2D/2020|EPI_ISL_442268|202	....	....	....	....	....	....
B	668	hCoV-19/England/CAMB-7CFE4/2020|EPI_ISL_442269|202	....	....	....	....	....	....
B	669	hCoV-19/England/CAMB-7CF8A/2020|EPI_ISL_442270|202	....	....	....	....	....	....
B	670	hCoV-19/England/CAMB-7CF99/2020|EPI_ISL_442272|202	....	....	....	....	....	....
B	671	hCoV-19/England/CAMB-7DCEC/2020|EPI_ISL_442273|202	....	....	....	....	....	....
B	672	hCoV-19/England/CAMB-7D2DB/2020|EPI_ISL_442274|202	....	....	....	....	....	....
B	673	hCoV-19/England/CAMB-7BAA8/2020|EPI_ISL_442275|202	....	....	....	....	....	....
B	674	hCoV-19/England/CAMB-7D420/2020|EPI_ISL_442276|202	....	....	....	....	....	....
B	675	hCoV-19/England/CAMB-7C430/2020|EPI_ISL_442277|202	....	....	....	....	....	....
B	676	hCoV-19/England/CAMB-7C47C/2020|EPI_ISL_442278|202	....	....	....	....	....	....
B	677	hCoV-19/England/CAMB-8307E/2020|EPI_ISL_443522|202	....	....	....	....	....	....
B	678	hCoV-19/England/CAMB-836A9/2020|EPI_ISL_443523|202	....	....	....	....	....	....
B	679	hCoV-19/England/CAMB-826F5/2020|EPI_ISL_443524|202	....	....	....	....	....	....
B	680	hCoV-19/England/CAMB-8458D/2020|EPI_ISL_443525|202	....	....	....	....	....	....
B	681	hCoV-19/England/CAMB-82394/2020|EPI_ISL_443526|202	....	....	....	....	....	....
B	682	hCoV-19/England/CAMB-83418/2020|EPI_ISL_443527|202	....	....	....	....	....	....
B	683	hCoV-19/England/CAMB-8280E/2020|EPI_ISL_443528|202	....	....	....	....	....	....
B	684	hCoV-19/England/CAMB-1AA40B/2020|EPI_ISL_443529|20	....	....	....	....	....	....
B	685	hCoV-19/England/CAMB-8291A/2020|EPI_ISL_443530|202	....	....	....	....	....	....
B	686	hCoV-19/England/CAMB-82640/2020|EPI_ISL_443531|202	....	....	....	....	....	....
B	687	hCoV-19/England/CAMB-83612/2020|EPI_ISL_443532|202	....	....	....	....	....	....
B	688	hCoV-19/England/CAMB-82437/2020|EPI_ISL_443533|202	....	....	....	....	....	....
B	689	hCoV-19/England/CAMB-844CC/2020|EPI_ISL_443534|202	....	....	....	....	....	....
B	690	hCoV-19/England/CAMB-82974/2020|EPI_ISL_443535|202	....	....	....	....	....	....
B	691	hCoV-19/England/CAMB-82DD2/2020|EPI_ISL_443536|202	....	....	....	....	....	....
B	692	hCoV-19/England/CAMB-843A1/2020|EPI_ISL_443537|202	....	....	....	....	....	....
B	693	hCoV-19/England/CAMB-1AA3A4/2020|EPI_ISL_443538|20	....	....	....	....	....	....
B	694	hCoV-19/England/CAMB-822B5/2020|EPI_ISL_443540|202	....	....	....	....	....	....
B	695	hCoV-19/England/CAMB-82FEB/2020|EPI_ISL_443545|202	....	....	....	....	....	....
B	696	hCoV-19/England/CAMB-1AA32C/2020|EPI_ISL_443546|20	....	....	....	....	....	....
B	697	hCoV-19/England/CAMB-83111/2020|EPI_ISL_443548|202	....	....	....	....	....	....
B	698	hCoV-19/England/CAMB-838A3/2020|EPI_ISL_443549|202	....	....	....	....	....	....
B	699	hCoV-19/England/CAMB-833C0/2020|EPI_ISL_443557|202	....	....	....	....	....	....
B	700	hCoV-19/England/CAMB-825AD/2020|EPI_ISL_443558|202	....	....	....	....	....	....
B	701	hCoV-19/England/CAMB-823A3/2020|EPI_ISL_443559|202	....	....	....	....	....	....
B	702	hCoV-19/England/CAMB-82DC3/2020|EPI_ISL_443560|202	....	....	....	....	....	....
B	703	hCoV-19/England/CAMB-8313F/2020|EPI_ISL_443561|202	....	....	....	....	....	....
B	704	hCoV-19/England/CAMB-82570/2020|EPI_ISL_443562|202	....	....	....	....	....	....
B	705	hCoV-19/England/CAMB-8424A/2020|EPI_ISL_443564|202	....	....	....	....	....	....
B	706	hCoV-19/England/CAMB-843FC/2020|EPI_ISL_443566|202	....	....	....	....	....	....
B	707	hCoV-19/England/CAMB-82DF0/2020|EPI_ISL_443567|202	....	....	....	....	....	....
B	708	hCoV-19/England/CAMB-8382B/2020|EPI_ISL_443568|202	....	....	....	....	....	....
B	709	hCoV-19/England/CAMB-84523/2020|EPI_ISL_443569|202	....	....	....	....	....	....
B	710	hCoV-19/England/CAMB-83436/2020|EPI_ISL_443571|202	....	....	....	....	....	....
B	711	hCoV-19/England/CAMB-837B5/2020|EPI_ISL_443572|202	....	....	....	....	....	....
B	712	hCoV-19/England/CAMB-843B0/2020|EPI_ISL_443574|202	....	....	....	....	....	....
B	713	hCoV-19/England/CAMB-8318A/2020|EPI_ISL_443575|202	....	....	....	....	....	....
B	714	hCoV-19/England/CAMB-82859/2020|EPI_ISL_443576|202	....	....	....	....	....	....
B	715	hCoV-19/England/CAMB-82956/2020|EPI_ISL_443577|202	....	....	....	....	....	....
B	716	hCoV-19/England/CAMB-82D87/2020|EPI_ISL_443578|202	....	....	....	....	....	....
B	717	hCoV-19/England/CAMB-826E6/2020|EPI_ISL_443579|202	....	....	....	....	....	....
B	718	hCoV-19/England/CAMB-827D4/2020|EPI_ISL_443581|202	....	....	....	....	....	....
B	719	hCoV-19/England/CAMB-8317B/2020|EPI_ISL_443582|202	....	....	....	....	....	....
B	720	hCoV-19/England/CAMB-8266E/2020|EPI_ISL_443583|202	....	....	....	....	....	....

B	721 hCoV-19/England/CAMB-823C1/2020|EPI_ISL_443585|202	····················	····················	····················	····················	····················	····················	
B	722 hCoV-19/England/CAMB-82701/2020|EPI_ISL_443586|202	····················	····················	····················	····················	····················	····················	
B	723 hCoV-19/England/CAMB-82297/2020|EPI_ISL_443587|202	····················	····················	····················	····················	····················	····················	
B	724 hCoV-19/England/CAMB-8276B/2020|EPI_ISL_443588|202	····················	····················	····················	····················	····················	····················	
B	725 hCoV-19/England/CAMB-844BD/2020|EPI_ISL_443589|202	····················	····················	····················	····················	····················	····················	
B	726 hCoV-19/England/CAMB-824CE/2020|EPI_ISL_443590|202	····················	···················A	····················	····················	····················	····················	1
B	727 hCoV-19/England/CAMB-82543/2020|EPI_ISL_443591|202	····················	····················	····················	····················	····················	····················	
B	728 hCoV-19/England/CAMB-82446/2020|EPI_ISL_443592|202	····················	····················	····················	····················	····················	····················	
B	729 hCoV-19/England/CAMB-83A34/2020|EPI_ISL_443593|202	····················	····················	····················	····················	····················	····················	
B	730 hCoV-19/England/CAMB-837C4/2020|EPI_ISL_443594|202	····················	····················	····················	····················	····················	····················	
B	731 hCoV-19/England/CAMB-83348/2020|EPI_ISL_443595|202	····················	····················	····················	····················	····················	····················	
B	732 hCoV-19/England/CAMB-83885/2020|EPI_ISL_443598|202	····················	····················	····················	····················	····················	····················	
B	733 hCoV-19/England/CAMB-838C1/2020|EPI_ISL_443599|202	····················	····················	····················	····················	····················	····················	
B	734 hCoV-19/Denmark/ALAB-SSI-1387/2020|EPI_ISL_444853|	····················	····················	····················	····················	····················	····················	
B	735 hCoV-19/Denmark/ALAB-SSI-1388/2020|EPI_ISL_444854|	····················	····················	····················	····················	····················	····················	
B	736 hCoV-19/Denmark/ALAB-SSI-1390/2020|EPI_ISL_444855|	····················	····················	····················	····················	····················	····················	
B	737 hCoV-19/Denmark/ALAB-SSI-1391/2020|EPI_ISL_444856|	····················	····················	····················	····················	····················	····················	
B	738 hCoV-19/Denmark/ALAB-SSI-1393/2020|EPI_ISL_444857|	····················	····················	·············N······	····················	····················	····················	
B	739 hCoV-19/Denmark/ALAB-SSI-681/2020|EPI_ISL_444859|2	····················	····················	····················	····················	····················	····················	
B	740 hCoV-19/Denmark/ALAB-SSI-682/2020|EPI_ISL_444860|2	····················	····················	····················	····················	····················	····················	
B	741 hCoV-19/Denmark/ALAB-SSI-684/2020|EPI_ISL_444861|2	····················	····················	····················	····················	····················	····················	
B	742 hCoV-19/Denmark/ALAB-SSI-687/2020|EPI_ISL_444862|2	····················	····················	····················	····················	····················	····················	
B	743 hCoV-19/Denmark/ALAB-SSI-694/2020|EPI_ISL_444863|2	····················	····················	····················	····················	····················	····················	
B	744 hCoV-19/Denmark/ALAB-SSI-700/2020|EPI_ISL_444864|2	····················	····················	····················	····················	····················	····················	
B	745 hCoV-19/Denmark/ALAB-SSI-705/2020|EPI_ISL_444866|2	····················	····················	····················	····················	····················	····················	
B	746 hCoV-19/Denmark/ALAB-SSI-707/2020|EPI_ISL_444867|2	····················	····················	····················	····················	····················	····················	
B	747 hCoV-19/Denmark/ALAB-SSI-711/2020|EPI_ISL_444869|2	····················	····················	····················	····················	····················	····················	
B	748 hCoV-19/Denmark/ALAB-SSI-713/2020|EPI_ISL_444870|2	····················	····················	····················	····················	····················	····················	
B	749 hCoV-19/Denmark/ALAB-SSI-718/2020|EPI_ISL_444872|2	····················	····················	····················	····················	····················	····················	
B	750 hCoV-19/Denmark/ALAB-SSI-719/2020|EPI_ISL_444873|2	····················	····················	····················	····················	····················	····················	
B	751 hCoV-19/Denmark/ALAB-SSI-723/2020|EPI_ISL_444874|2	····················	····················	····················	····················	····················	····················	
B	752 hCoV-19/Denmark/ALAB-SSI-724/2020|EPI_ISL_444875|2	····················	····················	····················	····················	····················	····················	
B	753 hCoV-19/Denmark/ALAB-SSI-726/2020|EPI_ISL_444877|2	····················	····················	····················	····················	····················	····················	
B	754 hCoV-19/Denmark/ALAB-SSI-728/2020|EPI_ISL_444879|2	····················	····················	····················	····················	····················	····················	
B	755 hCoV-19/Denmark/ALAB-SSI-747/2020|EPI_ISL_444887|2	····················	····················	····················	····················	····················	····················	
B	756 hCoV-19/Denmark/ALAB-SSI-751/2020|EPI_ISL_444888|2	····················	····················	····················	····················	····················	····················	
B	757 hCoV-19/Denmark/ALAB-SSI-756/2020|EPI_ISL_444890|2	····················	····················	····················	····················	····················	····················	
B	758 hCoV-19/Denmark/ALAB-SSI-757/2020|EPI_ISL_444891|2	····················	····················	····················	····················	····················	····················	
B	759 hCoV-19/Denmark/ALAB-SSI-761/2020|EPI_ISL_444892|2	····················	····················	····················	····················	····················	····················	
B	760 hCoV-19/Denmark/ALAB-SSI-762/2020|EPI_ISL_444893|2	····················	····················	····················	····················	····················	····················	
B	761 hCoV-19/Denmark/ALAB-SSI-765/2020|EPI_ISL_444894|2	····················	····················	····················	····················	····················	····················	
B	762 hCoV-19/Denmark/ALAB-SSI-767/2020|EPI_ISL_444895|2	····················	····················	····················	····················	····················	····················	
B	763 hCoV-19/Denmark/ALAB-SSI-769/2020|EPI_ISL_444896|2	····················	····················	····················	····················	····················	····················	
B	764 hCoV-19/Denmark/ALAB-SSI-770/2020|EPI_ISL_444897|2	····················	····················	····················	····················	····················	····················	
B	765 hCoV-19/Denmark/ALAB-SSI-773/2020|EPI_ISL_444898|2	····················	····················	····················	····················	····················	····················	
B	766 hCoV-19/Denmark/ALAB-SSI-774/2020|EPI_ISL_444899|2	····················	····················	····················	····················	····················	····················	
B	767 hCoV-19/USA/MI-MDHHS-SC20273/2020|EPI_ISL_447071|2	····················	····················	····················	····················	····················	····················	
B	768 hCoV-19/USA/MI-MDHHS-SC20279/2020|EPI_ISL_447077|2	····················	····················	····················	····················	····················	····················	
B	769 hCoV-19/USA/WA-S166/2020|EPI_ISL_430204|2020-03-18	····················	····················	····················	····················	····················	····················	
B	770 hCoV-19/USA/WA-S167/2020|EPI_ISL_430205|2020-03-18	····················	····················	····················	····················	····················	····················	
B	771 hCoV-19/USA/WA-S168/2020|EPI_ISL_430206|2020-03-18	····················	····················	····················	····················	····················	····················	
B	772 hCoV-19/USA/WA-S169/2020|EPI_ISL_430207|2020-03-18	····················	····················	····················	····················	····················	····················	
B	773 hCoV-19/USA/WA-S170/2020|EPI_ISL_430208|2020-03-17	····················	····················	····················	····················	····················	····················	
B	774 hCoV-19/USA/WA-S171/2020|EPI_ISL_430209|2020-03-19	····················	····················	····················	····················	····················	····················	
B	775 hCoV-19/USA/WA-S172/2020|EPI_ISL_430210|2020-03-19	····················	····················	····················	····················	····················	····················	
B	776 hCoV-19/USA/WA-S173/2020|EPI_ISL_430211|2020-03-19	····················	····················	····················	····················	····················	····················	
B	777 hCoV-19/England/SHEF-C5359/2020|EPI_ISL_432802|202	····················	····················	····················	····················	····················	····················	
B	778 hCoV-19/England/SHEF-C56AB/2020|EPI_ISL_432804|202	····················	····················	····················	····················	····················	····················	
B	779 hCoV-19/England/SHEF-C6789/2020|EPI_ISL_432825|202	····················	····················	····················	····················	····················	····················	
B	780 hCoV-19/England/SHEF-C6C11/2020|EPI_ISL_432828|202	····················	····················	····················	····················	····················	····················	
B	781 hCoV-19/England/SHEF-C6EA2/2020|EPI_ISL_432831|202	····················	····················	····················	····················	····················	····················	
B	782 hCoV-19/England/SHEF-C715D/2020|EPI_ISL_432836|202	····················	····················	····················	····················	····················	····················	
B	783 hCoV-19/England/SHEF-C7296/2020|EPI_ISL_432837|202	····················	····················	····················	····················	····················	····················	
B	784 hCoV-19/England/SHEF-C7937/2020|EPI_ISL_432850|202	····················	····················	····················	····················	····················	····················	
B	785 hCoV-19/England/SHEF-C81D4/2020|EPI_ISL_432859|202	····················	····················	····················	····················	····················	····················	
B	786 hCoV-19/England/SHEF-C8338/2020|EPI_ISL_432861|202	····················	···A················	····················	····················	····················	····················	
B	787 hCoV-19/England/SHEF-C83ED/2020|EPI_ISL_432864|202	····················	····················	····················	····················	····················	····················	
B	788 hCoV-19/England/SHEF-C8532/2020|EPI_ISL_432867|202	····················	····················	····················	····················	····················	····················	
B	789 hCoV-19/Scotland/EDB894/2020|EPI_ISL_432868|2020-0	····················	·A··················	····················	····················	····················	····················	
B	790 hCoV-19/Scotland/EDB895/2020|EPI_ISL_432869|2020-0	····················	····················	····················	····················	····················	····················	
B	791 hCoV-19/Scotland/EDB896/2020|EPI_ISL_432870|2020-0	····················	····················	····················	····················	····················	····················	
B	792 hCoV-19/Scotland/EDB897/2020|EPI_ISL_432871|2020-0	····················	····················	····················	····················	····················	····················	
B	793 hCoV-19/Scotland/EDB899/2020|EPI_ISL_432872|2020-0	····················	····················	····················	····················	····················	····················	
B	794 hCoV-19/Scotland/EDB901/2020|EPI_ISL_432873|2020-0	····················	·A··················	····················	····················	····················	····················	
B	795 hCoV-19/USA/TX-HMH0416/2020|EPI_ISL_435000|2020-04	····················	····················	····················	····················	····················	····················	
B	796 hCoV-19/USA/TX-HMH0417/2020|EPI_ISL_435001|2020-04	····················	····················	····················	····················	····················	····················	
B	797 hCoV-19/USA/TX-HMH0418/2020|EPI_ISL_435002|2020-04	····················	····················	····················	····················	····················	····················	
B	798 hCoV-19/USA/TX-HMH0420/2020|EPI_ISL_435003|2020-04	····················	····················	····················	····················	····················	····················	

B	799 hCoV-19/USA/TX-HMH0422/2020|EPI_ISL_435005|2020-04	····················	····················	····················	····················	····················	····················
B	800 hCoV-19/USA/TX-HMH0423/2020|EPI_ISL_435006|2020-04	····················	····················	····················	····················	····················	····················
B	801 hCoV-19/USA/TX-HMH0424/2020|EPI_ISL_435007|2020-04	····················	····················	····················	····················	····················	····················
B	802 hCoV-19/USA/TX-HMH0425/2020|EPI_ISL_435008|2020-04	····················	····················	····················	····················	····················	····················
B	803 hCoV-19/USA/TX-HMH0426/2020|EPI_ISL_435009|2020-04	····················	····················	····················	····················	····················	····················
B	804 hCoV-19/USA/TX-HMH0427/2020|EPI_ISL_435010|2020-04	····················	····················	····················	····················	····················	····················
B	805 hCoV-19/USA/TX-HMH0428/2020|EPI_ISL_435011|2020-04	····················	····················	····················	····················	····················	····················
B	806 hCoV-19/USA/TX-HMH0429/2020|EPI_ISL_435012|2020-04	····················	····················	····················	····················	····················	····················
B	807 hCoV-19/USA/TX-HMH0430/2020|EPI_ISL_435013|2020-04	····················	····················	····················	····················	····················	····················
B	808 hCoV-19/USA/TX-HMH0431/2020|EPI_ISL_435014|2020-04	····················	····················	····················	····················	····················	····················
B	809 hCoV-19/USA/TX-HMH0432/2020|EPI_ISL_435015|2020-04	····················	····················	····················	····················	····················	····················
B	810 hCoV-19/USA/TX-HMH0433/2020|EPI_ISL_435016|2020-04	····················	····················	····················	····················	····················	····················
B	811 hCoV-19/USA/TX-HMH0434/2020|EPI_ISL_435017|2020-04	····················	····················	····················	····················	····················	····················
B	812 hCoV-19/USA/TX-HMH0435/2020|EPI_ISL_435018|2020-04	····················	····················	····················	····················	····················	····················
B	813 hCoV-19/USA/TX-HMH0436/2020|EPI_ISL_435019|2020-04	····················	····················	····················	····················	····················	····················
B	814 hCoV-19/USA/TX-HMH0437/2020|EPI_ISL_435020|2020-04	····················	····················	····················	····················	····················	····················
B	815 hCoV-19/USA/TX-HMH0438/2020|EPI_ISL_435021|2020-04	····················	····················	····················	····················	····················	····················
B	816 hCoV-19/USA/TX-HMH0439/2020|EPI_ISL_435022|2020-04	····················	····················	····················	····················	····················	····················
B	817 hCoV-19/USA/TX-HMH0440/2020|EPI_ISL_435023|2020-04	····················	····················	····················	····················	····················	····················
B	818 hCoV-19/USA/TX-HMH0441/2020|EPI_ISL_435024|2020-04	····················	····················	····················	····················	····················	····················
B	819 hCoV-19/USA/TX-HMH0449/2020|EPI_ISL_435028|2020-04	····················	····················	····················	····················	····················	····················
B	820 hCoV-19/USA/TX-HMH0450/2020|EPI_ISL_435029|2020-04	····················	····················	····················	····················	····················	····················
B	821 hCoV-19/India/GJ-GBRC3/2020|EPI_ISL_435050|2020-04	····················	····················	····················	····················	····················	····················
B	822 hCoV-19/India/GJ-GBRC4/2020|EPI_ISL_435051|2020-04	····················	····················	····················	····················	····················	····················
B	823 hCoV-19/India/GJ-GBRC5/2020|EPI_ISL_435052|2020-04	····················	····················	····················	····················	····················	····················
B	824 hCoV-19/India/GJ-GBRC6/2020|EPI_ISL_435053|2020-04	····················	····················	····················	····················	····················	····················
B	825 hCoV-19/India/GJ-GBRC7/2020|EPI_ISL_435054|2020-04	····················	····················	····················	····················	····················	····················
B	826 hCoV-19/India/GJ-GBRC8/2020|EPI_ISL_435055|2020-04	····················	·A··················	····················	····················	····················	··A·················
B	827 hCoV-19/India/GJ-GBRC9/2020|EPI_ISL_435056|2020-04	····················	····················	····················	····················	····················	····················
B	828 hCoV-19/India/UP-NCDC-01257/2020|EPI_ISL_435060|20	····················	····················	····················	····················	····················	····················
B	829 hCoV-19/India/DL-NCDC-01537/2020|EPI_ISL_435061|20	····················	····················	····················	····················	····················	····················
B	830 hCoV-19/India/PB-NCDC-01538/2020|EPI_ISL_435062|20	····················	·N··················	····················	····················	····················	····················
B	831 hCoV-19/India/DL-NCDC-01326/2020|EPI_ISL_435063|20	····················	····················	····················	····················	····················	····················
B	832 hCoV-19/India/DL-NCDC-01705/2020|EPI_ISL_435064|20	····················	····················	····················	····················	····················	····················
B	833 hCoV-19/India/DL-NCDC-01475/2020|EPI_ISL_435065|20	····················	·A··················	····················	····················	····················	··A·················
B	834 hCoV-19/India/DL-NCDC-01711/2020|EPI_ISL_435066|20	····················	·A··················	····················	····················	····················	··A·················
B	835 hCoV-19/India/DL-NCDC-01712/2020|EPI_ISL_435067|20	····················	·A··················	····················	····················	····················	··A·················
B	836 hCoV-19/India/DL-NCDC-01757/2020|EPI_ISL_435068|20	····················	·A··················	····················	····················	····················	··A·················
B	837 hCoV-19/India/DL-NCDC-01744/2020|EPI_ISL_435069|20	····················	·A··················	····················	····················	····················	··A·················
B	838 hCoV-19/India/DL-NCDC-01710/2020|EPI_ISL_435070|20	····················	····················	····················	····················	····················	····················
B	839 hCoV-19/India/DL-NCDC-02105/2020|EPI_ISL_435071|20	····················	····················	····················	····················	····················	····················
B	840 hCoV-19/India/DL-NCDC-02155/2020|EPI_ISL_435072|20	····················	····················	····················	····················	··········N·········	····················
B	841 hCoV-19/India/WB-NCDC-02250/2020|EPI_ISL_435074|20	····················	····················	····················	····················	····················	····················
B	842 hCoV-19/India/TN-NCDC-02311/2020|EPI_ISL_435078|20	····················	····················	····················	····················	·············N······	····················
B	843 hCoV-19/India/TN-NCDC-02334/2020|EPI_ISL_435080|20	····················	····················	····················	····················	····················	····················
B	844 hCoV-19/India/WB-NCDC-02248/2020|EPI_ISL_435081|20	····················	····················	····················	····················	····················	····················
B	845 hCoV-19/India/UP-NCDC-02322/2020|EPI_ISL_435082|20	····················	····················	····················	····················	····················	····················
B	846 hCoV-19/India/TN-NCDC-02242/2020|EPI_ISL_435083|20	····················	····················	····················	····················	····················	····················
B	847 hCoV-19/India/TN-NCDC-02309/2020|EPI_ISL_435084|20	····················	····················	····················	····················	····················	····················
B	848 hCoV-19/India/MH-NCDC-02310/2020|EPI_ISL_435085|20	····················	····················	····················	····················	····················	····················
B	849 hCoV-19/India/MH-NCDC-02315/2020|EPI_ISL_435086|20	····················	····················	····················	····················	····················	····················
B	850 hCoV-19/India/TN-NCDC-02333/2020|EPI_ISL_435087|20	····················	····················	····················	····················	····················	····················
B	851 hCoV-19/India/OR-NCDC-02336/2020|EPI_ISL_435088|20	····················	····················	····················	····················	····················	····················
B	852 hCoV-19/India/TN-NCDC-02245/2020|EPI_ISL_435091|20	····················	····················	····················	····················	····················	····················
B	853 hCoV-19/India/TN-NCDC-02244/2020|EPI_ISL_435092|20	····················	····················	····················	····················	·······N···W········	····················
B	854 hCoV-19/India/TN-NCDC-02312/2020|EPI_ISL_435093|20	····················	····················	····················	····················	··········N·········	····················
B	855 hCoV-19/India/TN-NCDC-02327/2020|EPI_ISL_435094|20	····················	····················	····················	····················	····················	····················
B	856 hCoV-19/India/TN-NCDC-02328/2020|EPI_ISL_435095|20	····················	····················	····················	····················	····················	····················
B	857 hCoV-19/India/TN-NCDC-02332/2020|EPI_ISL_435096|20	····················	····················	····················	····················	····················	····················
B	858 hCoV-19/India/WB-NCDC-02252/2020|EPI_ISL_435097|20	····················	····················	····················	····················	····················	····················
B	859 hCoV-19/India/UN-NCDC-02240/2020|EPI_ISL_435098|20	····················	····················	····················	····················	····················	····················
B	860 hCoV-19/India/UP-NCDC-02320/2020|EPI_ISL_435099|20	····················	····················	····················	····················	··········N·········	····················
B	861 hCoV-19/Spain/VC-FISABIO-222/2020|EPI_ISL_436317|2	····················	····················	····················	····················	····················	····················
B	862 hCoV-19/Spain/VC-FISABIO-223/2020|EPI_ISL_436318|2	····················	····················	····················	····················	····················	····················
B	864 hCoV-19/Spain/VC-FISABIO-231/2020|EPI_ISL_436326|2	····················	····················	····················	····················	····················	····················
B	865 hCoV-19/Spain/VC-FISABIO-232/2020|EPI_ISL_436327|2	····················	····················	····················	····················	····················	····················
B	866 hCoV-19/Spain/VC-FISABIO-233/2020|EPI_ISL_436328|2	····················	····················	····················	····················	····················	····················
B	867 hCoV-19/Spain/VC-FISABIO-235/2020|EPI_ISL_436330|2	····················	····················	····················	····················	····················	····················
B	868 hCoV-19/Spain/VC-FISABIO-240/2020|EPI_ISL_436335|2	····················	····················	····················	····················	····················	····················
B	870 hCoV-19/Spain/VC-FISABIO-242/2020|EPI_ISL_436337|2	····················	····················	····················	····················	····················	····················
B	872 hCoV-19/Spain/VC-FISABIO-247/2020|EPI_ISL_436342|2	····················	····················	····················	····················	····················	····················
B	873 hCoV-19/Spain/VC-FISABIO-250/2020|EPI_ISL_436345|2	····················	····················	····················	····················	····················	····················
B	874 hCoV-19/Spain/VC-FISABIO-251/2020|EPI_ISL_436346|2	····················	····················	····················	····················	····················	····················
B	877 hCoV-19/Spain/VC-FISABIO-261/2020|EPI_ISL_436356|2	····················	····················	····················	····················	····················	····················
B	878 hCoV-19/Spain/VC-FISABIO-285/2020|EPI_ISL_436380|2	····················	····················	····················	····················	····················	····················
B	879 hCoV-19/Spain/VC-FISABIO-287/2020|EPI_ISL_436382|2	····················	····················	····················	····················	····················	····················
B	880 hCoV-19/Spain/VC-FISABIO-288/2020|EPI_ISL_436383|2	····················	····················	····················	····················	····················	····················
B	881 hCoV-19/Spain/VC-FISABIO-289/2020|EPI_ISL_436384|2	····················	····················	····················	····················	····················	····················

B	882 hCoV-19/Spain/VC-FISABIO-291/2020|EPI_ISL_436386|2	....	....	....	....	....	....	
B	883 hCoV-19/Spain/VC-FISABIO-292/2020|EPI_ISL_436387|2	....	....	....	....	....	....	
B	884 hCoV-19/Spain/VC-FISABIO-293/2020|EPI_ISL_436388|2	....	....	....	....	....	....	
B	885 hCoV-19/Spain/VC-FISABIO-294/2020|EPI_ISL_436389|2	....	....	....	....	....	....	
B	886 hCoV-19/Spain/VC-FISABIO-295/2020|EPI_ISL_436390|2	....	....	....	....	....	....	
B	887 hCoV-19/Spain/VC-FISABIO-296/2020|EPI_ISL_436391|2	....	....	....	....	....	....	
B	888 hCoV-19/Spain/VC-FISABIO-302/2020|EPI_ISL_436397|2	....	....	....	....	....	....	
B	889 hCoV-19/Denmark/ALAB-SSI-136/2020|EPI_ISL_437648|2	....	....	....	....	....	....	
B	890 hCoV-19/Denmark/ALAB-SSI-137/2020|EPI_ISL_437649|2	....	....	....	....	....	....	
B	891 hCoV-19/Denmark/ALAB-SSI-138/2020|EPI_ISL_437650|2	....	....	....	....	....	....	
B	892 hCoV-19/Denmark/ALAB-SSI-1406/2020|EPI_ISL_437651|	....	....	....	....	....	....	
B	893 hCoV-19/Denmark/ALAB-SSI-1421/2020|EPI_ISL_437653|	....	....	....	....	....	....	
B	894 hCoV-19/Denmark/ALAB-SSI-1427/2020|EPI_ISL_437654|	....	....	....	....	....	....	
B	895 hCoV-19/Denmark/ALAB-SSI-1434/2020|EPI_ISL_437655|	....	....	....	....	....	....	
B	896 hCoV-19/Denmark/ALAB-SSI-1498/2020|EPI_ISL_437656|	....	....	....	....	....	....	
B	897 hCoV-19/Denmark/ALAB-SSI-242/2020|EPI_ISL_437657|2	....	....	....	....	....	....	
B	898 hCoV-19/Denmark/ALAB-SSI-243/2020|EPI_ISL_437658|2	....	....	....	....	....	....	
B	899 hCoV-19/Denmark/ALAB-SSI-244/2020|EPI_ISL_437659|2	....	....	....	....	....	....	
B	900 hCoV-19/Denmark/ALAB-SSI-245/2020|EPI_ISL_437660|2	....	....	....	....	....	....	
B	901 hCoV-19/Denmark/ALAB-SSI-246/2020|EPI_ISL_437661|2	....	....	....	....	....	....	
B	902 hCoV-19/Denmark/ALAB-SSI-248/2020|EPI_ISL_437663|2	....	....	....	....	....	....	
B	903 hCoV-19/Denmark/ALAB-SSI-251/2020|EPI_ISL_437664|2	....	....	....	....	....	....	
B	904 hCoV-19/Denmark/ALAB-SSI-745/2020|EPI_ISL_437667|2	....	....	....	....	....	....	
B	905 hCoV-19/Denmark/ALAB-SSI-748/2020|EPI_ISL_437668|2	....	....	....	....	....	....	
B	906 hCoV-19/Denmark/ALAB-SSI-783/2020|EPI_ISL_437669|2	....	....	....	....	....	....	
B	907 hCoV-19/Denmark/ALAB-SSI-786/2020|EPI_ISL_437670|2	....	....	....	....	....	....	
B	908 hCoV-19/Denmark/ALAB-SSI-800/2020|EPI_ISL_437672|2	....	....	....	....	....	....	
B	909 hCoV-19/Denmark/ALAB-SSI-817/2020|EPI_ISL_437674|2	....	....	....	....	....	....	
B	910 hCoV-19/Denmark/ALAB-SSI-825/2020|EPI_ISL_437676|2	....	....	....	....	....	....	
B	911 hCoV-19/Denmark/ALAB-SSI-834/2020|EPI_ISL_437677|2	....	....	....	....	....	....	
B	912 hCoV-19/Denmark/ALAB-SSI-845/2020|EPI_ISL_437678|2	....	....	....	....	....	....	
B	913 hCoV-19/Denmark/ALAB-SSI-864/2020|EPI_ISL_437679|2	....	....	....	....	....	....	
B	914 hCoV-19/Denmark/ALAB-SSI-991/2020|EPI_ISL_437683|2	....	....	....	....	....	....	
B	915 hCoV-19/Ireland/un-NVRL-20W266/2020|EPI_ISL_437684	....	....	....	....	....	....	
B	916 hCoV-19/Ireland/un-NVRL-20W267/2020|EPI_ISL_437685	....	....	....	....	....	....	
B	917 hCoV-19/Ireland/un-NVRL-20W268/2020|EPI_ISL_437686	....	....	....	....	....	....	
B	918 hCoV-19/Ireland/un-NVRL-20W269/2020|EPI_ISL_437687	....	....	....	....	....	....	
B	919 hCoV-19/Ireland/un-NVRL-20W270/2020|EPI_ISL_437688	....	....	....	....	....	....	
B	920 hCoV-19/France/IDF-CIBU200107/2020|EPI_ISL_437689|	....	....	....	....	....	....	
B	921 hCoV-19/Scotland/CVR2789/2020|EPI_ISL_438979|2020-	....	...................A	....	....	....	....	1
B	922 hCoV-19/Scotland/CVR2802/2020|EPI_ISL_438980|2020-	....	....	....	....	....	....	
B	923 hCoV-19/Scotland/CVR2803/2020|EPI_ISL_438981|2020-	....	....	....	....	....	....	
B	924 hCoV-19/Scotland/CVR2805/2020|EPI_ISL_438982|2020-	....	....	....	....	....	....	
B	925 hCoV-19/Scotland/CVR2810/2020|EPI_ISL_438983|2020-	....	....	....	....	....	....	
B	926 hCoV-19/Scotland/CVR2812/2020|EPI_ISL_438984|2020-	....	....	....	....	....	....	
B	927 hCoV-19/Scotland/CVR2814/2020|EPI_ISL_438985|2020-	....	....	....	....	....	....	
B	928 hCoV-19/Scotland/CVR2819/2020|EPI_ISL_438986|2020-	....	....	....	....	....	....	
B	929 hCoV-19/Scotland/CVR2906/2020|EPI_ISL_438995|2020-	....	....	....	....	....	....	
B	930 hCoV-19/Scotland/CVR2921/2020|EPI_ISL_438998|2020-	....	....	....	....	....	....	
B	931 hCoV-19/Scotland/CVR2937/2020|EPI_ISL_438999|2020-	....	....	...........T.T.......	....	....	....	
B	932 hCoV-19/England/CAMB-834AF/2020|EPI_ISL_443500|202	....	....	....	....	....	....	
B	933 hCoV-19/England/CAMB-82561/2020|EPI_ISL_443501|202	....	....	....	....	....	....	
B	934 hCoV-19/England/CAMB-1AA4B0/2020|EPI_ISL_443502|20	....	....	....	....	....	....	
B	935 hCoV-19/England/CAMB-8226A/2020|EPI_ISL_443503|202	....	....	....	....	....	....	
B	936 hCoV-19/England/CAMB-8267D/2020|EPI_ISL_443504|202	....	....	....	....	....	....	
B	937 hCoV-19/England/CAMB-84514/2020|EPI_ISL_443505|202	....	....	....	....	....	....	
B	938 hCoV-19/England/CAMB-826D7/2020|EPI_ISL_443507|202	....	....	....	....	....	....	
B	939 hCoV-19/England/CAMB-831F3/2020|EPI_ISL_443509|202	....	....	....	....	....	....	
B	940 hCoV-19/England/CAMB-84453/2020|EPI_ISL_443510|202	....	....	....	....	....	....	
B	941 hCoV-19/England/CAMB-84541/2020|EPI_ISL_443511|202	....	....	....	....	....	....	
B	942 hCoV-19/England/CAMB-83409/2020|EPI_ISL_443512|202	....	....	....	....	....	....	
B	943 hCoV-19/England/CAMB-1AA4DE/2020|EPI_ISL_443514|20	....	....	....	....	....	....	
B	944 hCoV-19/England/CAMB-1AA3C2/2020|EPI_ISL_443515|20	....	....	....	....	....	....	
B	945 hCoV-19/England/CAMB-1AA359/2020|EPI_ISL_443517|20	....	....	....	....	....	....	
B	946 hCoV-19/England/CAMB-845E7/2020|EPI_ISL_443519|202	....	....	....	....	....	....	
B	947 hCoV-19/Denmark/ALAB-SSI-1235/2020|EPI_ISL_444817|	....	....	....	....	....	....	
B	948 hCoV-19/Denmark/ALAB-SSI-1238/2020|EPI_ISL_444818|	....	....	....	....	....	....	
B	949 hCoV-19/Denmark/ALAB-SSI-1239/2020|EPI_ISL_444819|	....	....	....	....	....	....	
B	950 hCoV-19/Denmark/ALAB-SSI-1241/2020|EPI_ISL_444820|	....	....	....	....	....	....	
B	951 hCoV-19/Denmark/ALAB-SSI-1243/2020|EPI_ISL_444822|	....	....	....	....	....	....	
B	952 hCoV-19/Denmark/ALAB-SSI-1244/2020|EPI_ISL_444823|	....	....	....	....	....	....	
B	953 hCoV-19/Denmark/ALAB-SSI-1245/2020|EPI_ISL_444824|	....	....	....	....	....	....	
B	954 hCoV-19/Denmark/ALAB-SSI-1246/2020|EPI_ISL_444825|	....	....	....	....	....	....	
B	955 hCoV-19/Denmark/ALAB-SSI-1248/2020|EPI_ISL_444826|	....	....	....	....	....	....	
B	956 hCoV-19/Denmark/ALAB-SSI-1249/2020|EPI_ISL_444827|	....	....	....	....	....	....	
B	957 hCoV-19/Denmark/ALAB-SSI-1250/2020|EPI_ISL_444828|	....	....	....	....	....	....	
B	958 hCoV-19/Denmark/ALAB-SSI-1252/2020|EPI_ISL_444829|	....	....	....	....	....	....	
B	959 hCoV-19/Denmark/ALAB-SSI-1257/2020|EPI_ISL_444831|	....	....	....	....	....	....	

B	960	hCoV-19/Denmark/ALAB-SSI-1260/2020|EPI_ISL_444832|	····················	····················	····················	····················	····················	····················
B	961	hCoV-19/Denmark/ALAB-SSI-1261/2020|EPI_ISL_444833|	····················	····················	····················	····················	····················	····················
B	962	hCoV-19/Denmark/ALAB-SSI-1262/2020|EPI_ISL_444834|	····················	····················	····················	····················	····················	····················
B	963	hCoV-19/Denmark/ALAB-SSI-1263/2020|EPI_ISL_444835|	····················	····················	····················	····················	····················	····················
B	964	hCoV-19/Denmark/ALAB-SSI-1264/2020|EPI_ISL_444836|	····················	····················	····················	····················	····················	····················
B	965	hCoV-19/Denmark/ALAB-SSI-1265/2020|EPI_ISL_444837|	····················	····················	····················	····················	····················	····················
B	966	hCoV-19/Denmark/ALAB-SSI-1268/2020|EPI_ISL_444838|	····················	····················	····················	····················	····················	····················
B	967	hCoV-19/Denmark/ALAB-SSI-1269/2020|EPI_ISL_444839|	····················	····················	····················	····················	····················	····················
B	968	hCoV-19/Denmark/ALAB-SSI-1270/2020|EPI_ISL_444840|	····················	····················	····················	····················	····················	····················
B	969	hCoV-19/Denmark/ALAB-SSI-1272/2020|EPI_ISL_444841|	····················	····················	····················	····················	····················	····················
B	970	hCoV-19/Denmark/ALAB-SSI-1282/2020|EPI_ISL_444844|	····················	····················	····················	····················	····················	····················
B	971	hCoV-19/Denmark/ALAB-SSI-1288/2020|EPI_ISL_444846|	····················	····················	····················	····················	····················	····················
B	972	hCoV-19/Denmark/ALAB-SSI-1292/2020|EPI_ISL_444848|	····················	····················	····················	····················	····················	····················
B	973	hCoV-19/Denmark/ALAB-SSI-1378/2020|EPI_ISL_444849|	····················	····················	····················	····················	····················	····················
B	974	hCoV-19/Denmark/ALAB-SSI-1382/2020|EPI_ISL_444850|	····················	····················	····················	····················	····················	····················
B	975	hCoV-19/Denmark/ALAB-SSI-1386/2020|EPI_ISL_444852|	····················	····················	····················	····················	····················	····················
B	977	hCoV-19/India/GJ-GBRC56a/2020|EPI_ISL_447030|2020-	····················	··········A·········	····················	····················	····················	····················
B	978	hCoV-19/India/GJ-GBRC56b/2020|EPI_ISL_447031|2020-	····················	····················	····················	····················	····················	····················
B	979	hCoV-19/India/GJ-GBRC57/2020|EPI_ISL_447032|2020-0	····················	····················	····················	····················	····················	····················
B	980	hCoV-19/India/GJ-GBRC58/2020|EPI_ISL_447033|2020-0	····················	·A··················	····················	····················	····················	··A·················
B	981	hCoV-19/India/GJ-GBRC59/2020|EPI_ISL_447034|2020-0	····················	·A··················	····················	····················	····················	··A·················
B	982	hCoV-19/India/GJ-GBRC60/2020|EPI_ISL_447035|2020-0	····················	·A··················	····················	····················	····················	··A·················
B	983	hCoV-19/India/GJ-GBRC61/2020|EPI_ISL_447036|2020-0	····················	····················	····················	····················	····················	····················
B	984	hCoV-19/India/GJ-GBRC62/2020|EPI_ISL_447037|2020-0	····················	····················	····················	····················	····················	····················
B	985	hCoV-19/India/GJ-GBRC63/2020|EPI_ISL_447038|2020-0	····················	····················	····················	····················	····················	····················
B	986	hCoV-19/India/GJ-GBRC64/2020|EPI_ISL_447039|2020-0	····················	·A··················	····················	····················	····················	··A·················
B	987	hCoV-19/India/GJ-GBRC65a/2020|EPI_ISL_447040|2020-	····················	·A··················	····················	····················	····················	··A·················
B	988	hCoV-19/India/GJ-GBRC65b/2020|EPI_ISL_447041|2020-	····················	····················	····················	····················	····················	··A·················
B	989	hCoV-19/India/GJ-GBRC66/2020|EPI_ISL_447042|2020-0	····················	····················	····················	····················	····················	····················
B	990	hCoV-19/India/GJ-GBRC67a/2020|EPI_ISL_447043|2020-	····················	·A··················	····················	····················	····················	··A·················
B	991	hCoV-19/India/GJ-GBRC67b/2020|EPI_ISL_447044|2020-	····················	·A··················	····················	····················	····················	··A·················
B	992	hCoV-19/India/GJ-GBRC68/2020|EPI_ISL_447045|2020-0	····················	·A··················	····················	····················	····················	··A·················
B	993	hCoV-19/India/GJ-GBRC69/2020|EPI_ISL_447046|2020-0	····················	·A··················	····················	····················	····················	··A·················
B	994	hCoV-19/India/GJ-GBRC70/2020|EPI_ISL_447047|2020-0	····················	····················	····················	····················	····················	····················
B	995	hCoV-19/India/GJ-GBRC71/2020|EPI_ISL_447048|2020-0	····················	·A··················	····················	····················	····················	··A·················
B	996	hCoV-19/India/GJ-GBRC72a/2020|EPI_ISL_447049|2020-	····················	·A··················	····················	····················	····················	··A·················
B	997	hCoV-19/India/GJ-GBRC72b/2020|EPI_ISL_447050|2020-	····················	·A··················	····················	····················	····················	··A·················
B	998	hCoV-19/India/GJ-GBRC73/2020|EPI_ISL_447051|2020-0	····················	·A··················	····················	····················	····················	··A·················
B	999	hCoV-19/India/GJ-GBRC74/2020|EPI_ISL_447052|2020-0	····················	····················	····················	····················	····················	····················
B	1000	hCoV-19/India/GJ-GBRC75/2020|EPI_ISL_447053|2020-0	····················	····················	····················	····················	····················	····················
B	1001	hCoV-19/Romania/279068/2020|EPI_ISL_447054|2020-04	····················	····················	····················	····················	····················	····················
B	1002	hCoV-19/Georgia/Tb-6598/2020|EPI_ISL_447055|2020-0	····················	····················	····················	····················	····················	····················
B	1003	hCoV-19/Georgia/Tb-6572/2020|EPI_ISL_447056|2020-0	····················	····················	····················	····················	····················	····················
B	1004	hCoV-19/USA/MI-MDHHS-SC20192/2020|EPI_ISL_447057|2	····················	····················	····················	····················	····················	····················
B	1005	hCoV-19/USA/MI-MDHHS-SC20260/2020|EPI_ISL_447059|2	····················	····················	····················	····················	····················	····················
B	1006	hCoV-19/USA/MI-MDHHS-SC20264/2020|EPI_ISL_447063|2	····················	····················	····················	····················	····················	····················
B	1007	hCoV-19/USA/MI-MDHHS-SC20266/2020|EPI_ISL_447064|2	····················	····················	····················	····················	····················	····················
B	1008	hCoV-19/USA/MI-MDHHS-SC20270/2020|EPI_ISL_447068|2	····················	····················	····················	····················	····················	····················
B	1009	hCoV-19/USA/MI-MDHHS-SC20271/2020|EPI_ISL_447069|2	····················	····················	····················	····················	····················	····················
B	1010	hCoV-19/USA/MI-MDHHS-SC20274/2020|EPI_ISL_447072|2	····················	····················	····················	····················	····················	····················
B	1011	hCoV-19/USA/MI-MDHHS-SC20283/2020|EPI_ISL_447081|2	····················	····················	····················	····················	····················	····················
B	1012	hCoV-19/USA/MI-MDHHS-SC20284/2020|EPI_ISL_447082|2	····················	····················	····················	····················	····················	····················
B	1013	hCoV-19/USA/MI-MDHHS-SC20285/2020|EPI_ISL_447083|2	····················	····················	····················	····················	····················	····················
B	1014	hCoV-19/USA/MI-MDHHS-SC20291/2020|EPI_ISL_447088|2	····················	····················	····················	····················	····················	····················
B	1015	hCoV-19/USA/MI-MDHHS-SC20292/2020|EPI_ISL_447089|2	····················	····················	····················	····················	····················	····················
B	1016	hCoV-19/USA/MI-MDHHS-SC20293/2020|EPI_ISL_447090|2	····················	····················	····················	····················	····················	····················
B	1017	hCoV-19/USA/MI-MDHHS-SC20294/2020|EPI_ISL_447091|2	····················	····················	····················	····················	····················	····················
B	1018	hCoV-19/USA/MI-MDHHS-SC20295/2020|EPI_ISL_447092|2	····················	····················	····················	····················	····················	····················
B	1019	hCoV-19/USA/MI-MDHHS-SC20296/2020|EPI_ISL_447093|2	····················	····················	····················	····················	····················	····················
B	1020	hCoV-19/USA/MI-MDHHS-SC20297/2020|EPI_ISL_447094|2	····················	····················	····················	····················	····················	····················
B	1021	hCoV-19/USA/MI-MDHHS-SC20298/2020|EPI_ISL_447095|2	····················	····················	····················	····················	····················	····················
B	1022	hCoV-19/USA/MI-MDHHS-SC20299/2020|EPI_ISL_447096|2	····················	····················	····················	····················	····················	····················
B	1023	hCoV-19/USA/MI-MDHHS-SC20301/2020|EPI_ISL_447098|2	····················	····················	····················	····················	····················	····················
B	1024	hCoV-19/USA/MI-MDHHS-SC20302/2020|EPI_ISL_447099|2	····················	····················	····················	····················	····················	····················
B	1025	hCoV-19/USA/WA-S142/2020|EPI_ISL_430179|2020-03-13	····················	····················	····················	····················	····················	····················
B	1026	hCoV-19/USA/WA-S143/2020|EPI_ISL_430180|2020-03-15	····················	····················	····················	····················	····················	····················
B	1027	hCoV-19/USA/WA-S144/2020|EPI_ISL_430181|2020-03-15	····················	····················	····················	····················	····················	····················
B	1028	hCoV-19/USA/WA-S145/2020|EPI_ISL_430182|2020-03-15	····················	····················	····················	····················	····················	····················
B	1029	hCoV-19/USA/WA-S146/2020|EPI_ISL_430183|2020-03-14	····················	····················	····················	····················	····················	····················
B	1030	hCoV-19/USA/WA-S147/2020|EPI_ISL_430184|2020-03-14	····················	····················	····················	····················	····················	····················
B	1031	hCoV-19/USA/WA-S148/2020|EPI_ISL_430185|2020-03-13	····················	····················	····················	····················	····················	····················
B	1032	hCoV-19/USA/WA-S149/2020|EPI_ISL_430186|2020-03-14	····················	····················	····················	····················	····················	····················
B	1033	hCoV-19/USA/WA-S150/2020|EPI_ISL_430187|2020-03-14	····················	····················	····················	····················	····················	····················
B	1034	hCoV-19/USA/WA-S151/2020|EPI_ISL_430188|2020-03-16	····················	····················	····················	····················	····················	····················
B	1035	hCoV-19/USA/WA-S152/2020|EPI_ISL_430189|2020-03-16	····················	····················	····················	····················	····················	····················
B	1036	hCoV-19/USA/WA-S153/2020|EPI_ISL_430190|2020-03-16	····················	····················	····················	····················	····················	····················
B	1037	hCoV-19/USA/WA-S154/2020|EPI_ISL_430191|2020-03-16	····················	····················	····················	····················	····················	····················
B	1038	hCoV-19/USA/WA-S155/2020|EPI_ISL_430192|2020-03-16	····················	····················	····················	····················	····················	····················

B	1039 hCoV-19/USA/WA-S156/2020|EPI_ISL_430193|2020-03-16	····················	····················	····················	····················	····················	····················	
B	1040 hCoV-19/USA/WA-S158/2020|EPI_ISL_430196|2020-03-16	····················	····················	····················	····················	····················	····················	
B	1041 hCoV-19/USA/WA-S159/2020|EPI_ISL_430197|2020-03-16	····················	····················	····················	····················	····················	····················	
B	1042 hCoV-19/USA/WA-S160/2020|EPI_ISL_430198|2020-03-17	····················	····················	····················	····················	····················	····················	
B	1043 hCoV-19/USA/WA-S161/2020|EPI_ISL_430199|2020-03-17	····················	····················	····················	····················	····················	····················	
B	1044 hCoV-19/England/SHEF-CCC93/2020|EPI_ISL_432533|202	····················	····················	····················	····················	····················	····················	
B	1045 hCoV-19/England/SHEF-CD05A/2020|EPI_ISL_432539|202	····················	·A··················	····················	····················	····················	····················	
B	1046 hCoV-19/England/SHEF-CD2AF/2020|EPI_ISL_432541|202	····················	····················	····················	····················	····················	····················	
B	1047 hCoV-19/England/SHEF-CD315/2020|EPI_ISL_432542|202	····················	····················	····················	····················	····················	····················	
B	1048 hCoV-19/England/SHEF-CD52E/2020|EPI_ISL_432547|202	····················	····················	····················	····················	····················	····················	
B	1049 hCoV-19/England/SHEF-CD737/2020|EPI_ISL_432548|202	····················	····················	····················	····················	····················	····················	
B	1050 hCoV-19/England/SHEF-CD89E/2020|EPI_ISL_432549|202	····················	····················	····················	····················	····················	····················	
B	1051 hCoV-19/England/SHEF-CD97D/2020|EPI_ISL_432551|202	····················	····················	····················	····················	····················	····················	
B	1052 hCoV-19/England/CAMB-7ACC1/2020|EPI_ISL_433864|202	····················	····················	····················	····················	····················	····················	
B	1053 hCoV-19/England/CAMB-7ACD0/2020|EPI_ISL_433865|202	····················	····················	····················	····················	······T·············	····················	
B	1054 hCoV-19/England/CAMB-7ACFE/2020|EPI_ISL_433866|202	····················	···················A	····················	····················	····················	····················	1
B	1055 hCoV-19/England/CAMB-7AD28/2020|EPI_ISL_433868|202	····················	····················	····················	····················	····················	····················	
B	1056 hCoV-19/England/CAMB-7AD46/2020|EPI_ISL_433870|202	····················	····················	····················	····················	····················	····················	
B	1057 hCoV-19/England/CAMB-7AD55/2020|EPI_ISL_433871|202	····················	····················	····················	····················	····················	····················	
B	1058 hCoV-19/England/CAMB-7AD64/2020|EPI_ISL_433872|202	····················	····················	····················	····················	····················	····················	
B	1059 hCoV-19/England/CAMB-7AD82/2020|EPI_ISL_433873|202	····················	····················	····················	····················	····················	····················	
B	1060 hCoV-19/England/CAMB-7ADA0/2020|EPI_ISL_433875|202	····················	····················	····················	····················	····················	····················	
B	1061 hCoV-19/England/CAMB-7ADBF/2020|EPI_ISL_433876|202	····················	····················	····················	····················	····················	····················	
B	1062 hCoV-19/England/CAMB-7ADDD/2020|EPI_ISL_433877|202	····················	····················	····················	····················	····················	····················	
B	1063 hCoV-19/England/CAMB-7ADEC/2020|EPI_ISL_433878|202	····················	····················	····················	····················	····················	····················	
B	1064 hCoV-19/England/CAMB-7AE07/2020|EPI_ISL_433880|202	····················	····················	····················	····················	····················	····················	
B	1065 hCoV-19/England/CAMB-7AE34/2020|EPI_ISL_433882|202	····················	····················	····················	····················	····················	····················	
B	1066 hCoV-19/England/CAMB-7AE43/2020|EPI_ISL_433883|202	····················	····················	····················	····················	····················	····················	
B	1067 hCoV-19/England/CAMB-7AE52/2020|EPI_ISL_433884|202	····················	····················	····················	····················	····················	····················	
B	1068 hCoV-19/England/CAMB-7AE61/2020|EPI_ISL_433885|202	····················	····················	····················	····················	····················	····················	
B	1069 hCoV-19/USA/TX-HMH0451/2020|EPI_ISL_435030|2020-04	····················	····················	····················	····················	····················	····················	
B	1070 hCoV-19/USA/TX-HMH0452/2020|EPI_ISL_435031|2020-04	····················	····················	····················	····················	····················	····················	
B	1071 hCoV-19/DRC/1516/2020|EPI_ISL_435032|2020-04-09	····················	····················	····················	····················	····················	····················	
B	1072 hCoV-19/DRC/1324/2020|EPI_ISL_435033|2020-04-07	····················	····················	····················	····················	····················	····················	
B	1073 hCoV-19/USA/LA-EVTL01/2020|EPI_ISL_435034|2020-04-	····················	····················	····················	····················	····················	····················	
B	1074 hCoV-19/USA/LA-EVTL02/2020|EPI_ISL_435035|2020-04-	····················	····················	····················	····················	····················	····················	
B	1075 hCoV-19/USA/LA-EVTL03/2020|EPI_ISL_435036|2020-04-	····················	····················	····················	····················	····················	····················	
B	1076 hCoV-19/USA/LA-EVTL04/2020|EPI_ISL_435037|2020-04-	····················	····················	····················	····················	····················	····················	
B	1077 hCoV-19/USA/LA-EVTL05/2020|EPI_ISL_435038|2020-04-	····················	····················	····················	····················	····················	····················	
B	1078 hCoV-19/USA/LA-EVTL06/2020|EPI_ISL_435039|2020-04-	····················	····················	····················	····················	····················	····················	
B	1079 hCoV-19/USA/LA-EVTL07/2020|EPI_ISL_435040|2020-04-	····················	····················	····················	····················	····················	····················	
B	1080 hCoV-19/USA/LA-EVTL08/2020|EPI_ISL_435041|2020-04-	····················	····················	····················	····················	····················	····················	
B	1081 hCoV-19/USA/LA-EVTL09/2020|EPI_ISL_435042|2020-04-	····················	····················	····················	····················	····················	····················	
B	1082 hCoV-19/USA/LA-EVTL010/2020|EPI_ISL_435043|2020-04	····················	····················	····················	····················	····················	····················	
B	1083 hCoV-19/USA/LA-EVTL011/2020|EPI_ISL_435044|2020-04	····················	····················	····················	····················	····················	····················	
B	1084 hCoV-19/Kazakhstan/NCB-1/2020|EPI_ISL_435045|2020-	····················	····················	····················	····················	····················	····················	
B	1085 hCoV-19/Kazakhstan/NCB-2/2020|EPI_ISL_435046|2020-	····················	····················	····················	····················	····················	····················	
B	1086 hCoV-19/Kazakhstan/NCB-3/2020|EPI_ISL_435047|2020-	····················	····················	····················	····················	····················	····················	
B	1087 hCoV-19/Kazakhstan/NCB-5/2020|EPI_ISL_435048|2020-	····················	····················	····················	····················	····················	····················	
B	1088 hCoV-19/India/GJ-GBRC2/2020|EPI_ISL_435049|2020-04	····················	····················	····················	····················	····················	····················	
B	1089 hCoV-19/Spain/VC-FISABIO-206/2020|EPI_ISL_436301|2	····················	····················	····················	····················	····················	····················	
B	1091 hCoV-19/Spain/VC-FISABIO-209/2020|EPI_ISL_436304|2	····················	····················	····················	····················	····················	····················	
B	1092 hCoV-19/Spain/VC-FISABIO-212/2020|EPI_ISL_436307|2	····················	····················	····················	····················	····················	····················	
B	1094 hCoV-19/Spain/VC-FISABIO-216/2020|EPI_ISL_436311|2	····················	····················	····················	····················	····················	····················	
B	1095 hCoV-19/Spain/VC-FISABIO-219/2020|EPI_ISL_436314|2	····················	····················	····················	····················	····················	····················	
B	1096 hCoV-19/Spain/VC-FISABIO-221/2020|EPI_ISL_436316|2	····················	····················	····················	····················	····················	····················	
B	1097 hCoV-19/Spain/VC-FISABIO-266/2020|EPI_ISL_436361|2	····················	····················	····················	····················	····················	····················	
B	1102 hCoV-19/Spain/VC-FISABIO-274/2020|EPI_ISL_436369|2	····················	····················	····················	····················	····················	NNN·················	1
B	1103 hCoV-19/Spain/VC-FISABIO-276/2020|EPI_ISL_436371|2	····················	····················	····················	····················	··N·················	····················	
B	1107 hCoV-19/Spain/VC-FISABIO-282/2020|EPI_ISL_436377|2	····················	····················	····················	····················	····················	····················	
B	1109 hCoV-19/Spain/VC-FISABIO-284/2020|EPI_ISL_436379|2	····················	····················	····················	····················	····················	····················	
B	1110 hCoV-19/USA/CA-SR0177/2020|EPI_ISL_437600|2020-03-	····················	····················	····················	····················	····················	····················	
B	1111 hCoV-19/Japan/Donner1/2020|EPI_ISL_437601|2020-04-	····················	····················	····················	····················	····················	····················	
B	1112 hCoV-19/Thailand/SI206917-NT/2020|EPI_ISL_437602|2	····················	····················	····················	····················	····················	····················	
B	1113 hCoV-19/Thailand/SI206587-NT/2020|EPI_ISL_437603|2	····················	····················	····················	····················	····················	····················	
B	1114 hCoV-19/Thailand/SI206377-NST/2020|EPI_ISL_437604|	····················	····················	····················	····················	····················	····················	
B	1115 hCoV-19/Thailand/SI205929-NT/2020|EPI_ISL_437605|2	····················	····················	····················	····················	····················	····················	
B	1116 hCoV-19/Thailand/SI205529-NT/2020|EPI_ISL_437606|2	····················	····················	····················	····················	····················	····················	
B	1117 hCoV-19/Thailand/SI205457-NST/2020|EPI_ISL_437607|	····················	····················	····················	····················	····················	····················	
B	1118 hCoV-19/Thailand/SI204713-NT/2020|EPI_ISL_437608|2	····················	····················	····················	····················	····················	····················	
B	1119 hCoV-19/Thailand/SI204514-NT/2020|EPI_ISL_437609|2	····················	····················	····················	····················	····················	····················	
B	1120 hCoV-19/Thailand/SI204512-NT/2020|EPI_ISL_437610|2	····················	····················	····················	····················	····················	····················	
B	1121 hCoV-19/Thailand/SI204487-NT/2020|EPI_ISL_437611|2	····················	····················	····················	····················	····················	····················	
B	1122 hCoV-19/Thailand/SI203285-NT/2020|EPI_ISL_437612|2	····················	····················	····················	····················	····················	····················	
B	1123 hCoV-19/Thailand/SI202872-NT/2020|EPI_ISL_437613|2	····················	····················	····················	····················	····················	····················	
B	1124 hCoV-19/Thailand/SI202769-NT/2020|EPI_ISL_437614|2	····················	····················	····················	····················	····················	····················	
B	1125 hCoV-19/Thailand/SI202620-NT/2020|EPI_ISL_437615|2	····················	····················	····················	····················	····················	····················	
B	1126 hCoV-19/Thailand/SI202205-NT/2020|EPI_ISL_437616|2	····················	····················	····················	····················	····················	····················	

B	1127 hCoV-19/Thailand/SI201712-NT/2020|EPI_ISL_437617|2	····················	····················	····················	····················	····················	····················	
B	1128 hCoV-19/Thailand/SI200615-NT/2020|EPI_ISL_437618|2	····················	····················	····················	····················	····················	····················	
B	1129 hCoV-19/Thailand/SI200478-NST/2020|EPI_ISL_437619|	····················	····················	····················	····················	····················	····················	
B	1130 hCoV-19/Thailand/SI200468-NT/2020|EPI_ISL_437620|2	····················	····················	····················	····················	····················	····················	
B	1131 hCoV-19/Thailand/SI200433-NT/2020|EPI_ISL_437621|2	····················	····················	····················	····················	····················	····················	
B	1132 hCoV-19/Thailand/SI200383-NT/2020|EPI_ISL_437622|2	····················	····················	····················	····················	····················	····················	
B	1133 hCoV-19/Thailand/SI200040-NT/2020|EPI_ISL_437623|2	····················	····················	····················	····················	····················	····················	
B	1134 hCoV-19/Thailand/SI200893-NT/2020|EPI_ISL_437624|2	····················	····················	····················	····················	····················	····················	
B	1135 hCoV-19/Poland/Wro-02/2020|EPI_ISL_437625|2020-03-	····················	····················	····················	····················	····················	····················	
B	1136 hCoV-19/India/TG-GMC-KP1125/2020|EPI_ISL_437626|20	····················	····················	····················	····················	····················	····················	
B	1137 hCoV-19/Denmark/ALAB-SSI-1008/2020|EPI_ISL_437628|	····················	····················	····················	····················	····················	····················	
B	1138 hCoV-19/Denmark/ALAB-SSI-1033/2020|EPI_ISL_437629|	····················	····················	····················	····················	····················	····················	
B	1139 hCoV-19/Denmark/ALAB-SSI-1046/2020|EPI_ISL_437630|	····················	····················	····················	····················	····················	····················	
B	1140 hCoV-19/Denmark/ALAB-SSI-1065/2020|EPI_ISL_437631|	····················	····················	····················	····················	····················	····················	
B	1141 hCoV-19/Denmark/ALAB-SSI-1086/2020|EPI_ISL_437632|	····················	····················	····················	····················	····················	····················	
B	1142 hCoV-19/Denmark/ALAB-SSI-1096/2020|EPI_ISL_437633|	····················	····················	····················	····················	····················	····················	
B	1143 hCoV-19/Denmark/ALAB-SSI-1100/2020|EPI_ISL_437634|	····················	····················	····················	····················	····················	····················	
B	1144 hCoV-19/Denmark/ALAB-SSI-1102/2020|EPI_ISL_437635|	····················	····················	····················	····················	····················	····················	
B	1145 hCoV-19/Denmark/ALAB-SSI-1192/2020|EPI_ISL_437636|	····················	····················	····················	····················	····················	····················	
B	1146 hCoV-19/Denmark/ALAB-SSI-1230/2020|EPI_ISL_437637|	····················	····················	····················	····················	····················	····················	
B	1147 hCoV-19/Denmark/ALAB-SSI-1280/2020|EPI_ISL_437639|	····················	····················	····················	····················	····················	····················	
B	1148 hCoV-19/Denmark/ALAB-SSI-1299/2020|EPI_ISL_437640|	····················	····················	····················	····················	····················	····················	
B	1149 hCoV-19/Denmark/ALAB-SSI-133/2020|EPI_ISL_437642|2	····················	····················	····················	····················	····················	····················	
B	1150 hCoV-19/Denmark/ALAB-SSI-1334/2020|EPI_ISL_437643|	····················	····················	····················	····················	····················	····················	
B	1151 hCoV-19/Denmark/ALAB-SSI-1337/2020|EPI_ISL_437644|	····················	····················	····················	····················	····················	····················	
B	1152 hCoV-19/Denmark/ALAB-SSI-1348/2020|EPI_ISL_437645|	····················	····················	····················	····················	····················	····················	
B	1153 hCoV-19/Denmark/ALAB-SSI-1351/2020|EPI_ISL_437646|	····················	····················	····················	····················	····················	····················	
B	1154 hCoV-19/Denmark/ALAB-SSI-135/2020|EPI_ISL_437647|2	····················	····················	····················	····················	····················	····················	
B	1155 hCoV-19/France/HDF-CIBU200132/2020|EPI_ISL_437690|	····················	····················	····················	····················	····················	····················	
B	1156 hCoV-19/Saudi Arabia/KAUST-Makkah124/2020|EPI_ISL_	····················	····················	····················	····················	····················	····················	
B	1157 hCoV-19/Saudi Arabia/KAUST-Makkah126/2020|EPI_ISL_	····················	····················	····················	····················	····················	····················	
B	1158 hCoV-19/Saudi Arabia/KAUST-Makkah138/2020|EPI_ISL_	····················	····················	····················	····················	····················	····················	
B	1159 hCoV-19/Saudi Arabia/KAUST-Makkah139/2020|EPI_ISL_	····················	····················	····················	····················	····················	····················	
B	1160 hCoV-19/Saudi Arabia/KAUST-Makkah142/2020|EPI_ISL_	····················	····················	····················	····················	····················	····················	
B	1161 hCoV-19/Saudi Arabia/KAUST-Makkah156/2020|EPI_ISL_	····················	····················	····················	····················	····················	····················	
B	1162 hCoV-19/Saudi Arabia/KAUST-Makkah163/2020|EPI_ISL_	····················	····················	····················	····················	····················	····················	
B	1163 hCoV-19/Saudi Arabia/KAUST-Makkah166/2020|EPI_ISL_	····················	····················	····················	····················	····················	····················	
B	1164 hCoV-19/Saudi Arabia/KAUST-Makkah173/2020|EPI_ISL_	····················	····················	····················	····················	····················	····················	
B	1165 hCoV-19/Scotland/CVR2403/2020|EPI_ISL_438900|2020-	····················	····················	····················	····················	····················	····················	
B	1166 hCoV-19/Scotland/CVR2404/2020|EPI_ISL_438901|2020-	····················	····················	····················	····················	····················	····················	
B	1167 hCoV-19/Scotland/CVR2411/2020|EPI_ISL_438902|2020-	····················	···················A	····················	····················	····················	····················	1
B	1168 hCoV-19/Scotland/CVR2413/2020|EPI_ISL_438903|2020-	····················	····················	····················	····················	····················	····················	
B	1169 hCoV-19/Scotland/CVR2437/2020|EPI_ISL_438905|2020-	····················	····················	····················	····················	····················	····················	
B	1170 hCoV-19/Scotland/CVR2446/2020|EPI_ISL_438907|2020-	····················	····················	····················	····················	····················	····················	
B	1171 hCoV-19/Scotland/CVR2447/2020|EPI_ISL_438908|2020-	····················	····················	····················	····················	····················	····················	
B	1172 hCoV-19/Scotland/CVR2450/2020|EPI_ISL_438910|2020-	····················	···················A	····················	····················	····················	····················	1
B	1173 hCoV-19/Scotland/CVR2468/2020|EPI_ISL_438912|2020-	····················	····················	····················	····················	····················	····················	
B	1174 hCoV-19/Scotland/CVR247/2020|EPI_ISL_438913|2020-0	····················	····················	····················	····················	····················	····················	
B	1175 hCoV-19/Scotland/CVR249/2020|EPI_ISL_438914|2020-0	····················	····················	····················	····················	····················	····················	
B	1176 hCoV-19/Scotland/CVR2492/2020|EPI_ISL_438915|2020-	····················	····················	····················	····················	····················	····················	
B	1177 hCoV-19/Scotland/CVR2496/2020|EPI_ISL_438916|2020-	····················	····················	····················	····················	····················	····················	
B	1179 hCoV-19/Scotland/CVR2512/2020|EPI_ISL_438920|2020-	····················	····················	····················	····················	····················	····················	
B	1180 hCoV-19/Scotland/CVR2515/2020|EPI_ISL_438921|2020-	····················	···················A	····················	····················	····················	····················	1
B	1181 hCoV-19/Scotland/CVR2521/2020|EPI_ISL_438923|2020-	····················	····················	····················	····················	····················	····················	
B	1182 hCoV-19/Scotland/CVR2536/2020|EPI_ISL_438925|2020-	····················	····················	····················	····················	····················	····················	
B	1183 hCoV-19/Scotland/CVR255/2020|EPI_ISL_438926|2020-0	····················	····················	····················	····················	····················	····················	
B	1185 hCoV-19/Scotland/CVR2576/2020|EPI_ISL_438929|2020-	····················	···················A	····················	····················	····················	····················	1
B	1186 hCoV-19/Scotland/CVR258/2020|EPI_ISL_438930|2020-0	····················	····················	····················	····················	····················	····················	
B	1187 hCoV-19/Scotland/CVR2611/2020|EPI_ISL_438931|2020-	····················	····················	····················	····················	····················	····················	
B	1188 hCoV-19/Scotland/CVR263/2020|EPI_ISL_438933|2020-0	····················	····················	····················	····················	····················	····················	
B	1189 hCoV-19/Scotland/CVR2738/2020|EPI_ISL_438937|2020-	····················	···················A	····················	····················	····················	····················	1
B	1190 hCoV-19/Scotland/CVR2743/2020|EPI_ISL_438941|2020-	····················	····················	····················	····················	····················	····················	
B	1191 hCoV-19/Scotland/CVR2746/2020|EPI_ISL_438944|2020-	····················	····················	····················	····················	····················	····················	
B	1192 hCoV-19/Japan/Donner2/2020|EPI_ISL_438947|2020-04-	····················	····················	····················	····················	····················	····················	
B	1195 hCoV-19/Japan/Donner6/2020|EPI_ISL_438951|2020-04-	····················	····················	····················	····················	····················	····················	
B	1197 hCoV-19/Japan/Donner8/2020|EPI_ISL_438953|2020-04-	····················	····················	····················	····················	····················	····················	
B	1200 hCoV-19/Japan/Donner12/2020|EPI_ISL_438956|2020-04	····················	····················	····················	····················	····················	····················	
B	1203 hCoV-19/Japan/Donner17/2020|EPI_ISL_438961|2020-04	····················	····················	····················	····················	····················	····················	
B	1204 hCoV-19/Japan/Donner21/2020|EPI_ISL_438965|2020-04	····················	····················	····················	····················	····················	····················	
B	1205 hCoV-19/Japan/Donner22/2020|EPI_ISL_438966|2020-04	····················	····················	····················	····················	····················	····················	
B	1206 hCoV-19/Japan/Donner23/2020|EPI_ISL_438967|2020-04	····················	····················	····················	····················	····················	····················	
B	1208 hCoV-19/Japan/Donner26/2020|EPI_ISL_438970|2020-03	····················	····················	····················	····················	····················	····················	
B	1209 hCoV-19/Japan/Donner27/2020|EPI_ISL_438971|2020-04	····················	····················	····················	····················	····················	····················	
B	1210 hCoV-19/Japan/Donner28/2020|EPI_ISL_438972|2020-03	····················	····················	····················	····················	····················	····················	
B	1211 hCoV-19/Scotland/CVR2749/2020|EPI_ISL_438973|2020-	····················	····················	····················	····················	····················	····················	
B	1212 hCoV-19/Scotland/CVR2750/2020|EPI_ISL_438974|2020-	····················	····················	····················	····················	····················	····················	
B	1213 hCoV-19/Scotland/CVR2751/2020|EPI_ISL_438975|2020-	····················	····················	····················	····················	····················	····················	
B	1214 hCoV-19/Scotland/CVR2757/2020|EPI_ISL_438976|2020-	····················	····················	····················	····················	····················	····················	

```
B   1215 hCoV-19/Scotland/CVR2764/2020|EPI_ISL_438977|2020-   ····················  ····················  ····················  ····················  ····················  ····················
B   1216 hCoV-19/Scotland/CVR2770/2020|EPI_ISL_438978|2020-   ····················  ····················  ····················  ····················  ····················  ····················
B   1217 hCoV-19/England/CAMB-7E085/2020|EPI_ISL_442158|202   ····················  ····················  ····················  ····················  ····················  ····················
B   1218 hCoV-19/England/CAMB-7BB96/2020|EPI_ISL_442160|202   ····················  ····················  ····················  ····················  ····················  ····················
B   1219 hCoV-19/England/CAMB-7DE22/2020|EPI_ISL_442161|202   ····················  ·A··················  ····················  ····················  ····················  ····················
B   1220 hCoV-19/England/CAMB-7BDFA/2020|EPI_ISL_442163|202   ····················  ····················  ····················  ····················  ····················  ····················
B   1221 hCoV-19/England/CAMB-7CE23/2020|EPI_ISL_442164|202   ····················  ····················  ····················  ····················  ····················  ····················
B   1222 hCoV-19/England/CAMB-7BA5D/2020|EPI_ISL_442165|202   ····················  ····················  ····················  ····················  ····················  ····················
B   1223 hCoV-19/England/CAMB-7DFD4/2020|EPI_ISL_442167|202   ····················  ·A··················  ····················  ····················  ····················  ····················
B   1224 hCoV-19/England/CAMB-7CFF3/2020|EPI_ISL_442168|202   ····················  ····················  ····················  ····················  ····················  ····················
B   1225 hCoV-19/England/CAMB-7E0D0/2020|EPI_ISL_442169|202   ····················  ····················  ····················  ····················  ····················  ····················
B   1226 hCoV-19/England/CAMB-7B853/2020|EPI_ISL_442170|202   ····················  ····················  ····················  ····················  ····················  ····················
B   1227 hCoV-19/England/CAMB-7CC38/2020|EPI_ISL_442171|202   ····················  ····················  ····················  ····················  ····················  ····················
B   1228 hCoV-19/England/CAMB-7DDAD/2020|EPI_ISL_442172|202   ····················  ····················  ····················  ····················  ····················  ····················
B   1229 hCoV-19/England/CAMB-7B8F9/2020|EPI_ISL_442174|202   ····················  ····················  ····················  ····················  ····················  ····················
```
[truncated: 1,187,966 more chars]
